# Reading the Complex Skipper Butterfly Fauna of One Tropical Place

**DOI:** 10.1371/journal.pone.0019874

**Published:** 2011-08-16

**Authors:** Daniel H. Janzen, Winnie Hallwachs, John M. Burns, Mehrdad Hajibabaei, Claudia Bertrand, Paul D. N. Hebert

**Affiliations:** 1 Department of Biology, University of Pennsylvania, Philadelphia, Pennsylvania, United States of America; 2 Department of Entomology, National Museum of Natural History, Smithsonian Institution, Washington, D.C., United States of America; 3 Department of Integrative Biology, Biodiversity Institute of Ontario, University of Guelph, Guelph, Canada; American Museum of Natural History, United States of America

## Abstract

**Background:**

An intense, 30-year, ongoing biodiversity inventory of Lepidoptera, together with their food plants and parasitoids, is centered on the rearing of wild-caught caterpillars in the 120,000 terrestrial hectares of dry, rain, and cloud forest of Area de Conservacion Guanacaste (ACG) in northwestern Costa Rica. Since 2003, DNA barcoding of all species has aided their identification and discovery. We summarize the process and results for a large set of the species of two speciose subfamilies of ACG skipper butterflies (Hesperiidae) and emphasize the effectiveness of barcoding these species (which are often difficult and time-consuming to identify).

**Methodology/Principal Findings:**

Adults are DNA barcoded by the Biodiversity Institute of Ontario, Guelph, Canada; and they are identified by correlating the resulting COI barcode information with more traditional information such as food plant, facies, genitalia, microlocation within ACG, caterpillar traits, etc. This process has found about 303 morphologically defined species of eudamine and pyrgine Hesperiidae breeding in ACG (about 25% of the ACG butterfly fauna) and another 44 units indicated by distinct barcodes (n = 9,094), which may be additional species and therefore may represent as much as a 13% increase. All but the members of one complex can be identified by their DNA barcodes.

**Conclusions/Significance:**

Addition of DNA barcoding to the methodology greatly improved the inventory, both through faster (hence cheaper) accurate identification of the species that are distinguishable without barcoding, as well as those that require it, and through the revelation of species “hidden” within what have long been viewed as single species. Barcoding increased the recognition of species-level specialization. It would be no more appropriate to ignore barcode data in a species inventory than it would be to ignore adult genitalia variation or caterpillar ecology.

## Introduction

There is a living newspaper called “The Dicot-eating Skipper Butterflies” that covers Hesperiidae in the subfamilies Eudaminae and Pyrginae inhabiting Area de Conservacion Guanacaste (ACG) in northwestern Costa Rica. Field ecologists DHJ, WH, and a team of 1–33 parataxonomists [1] have been reading this daily since 1978, through the spectacles of a field inventory of ACG caterpillars, their parasitoids, and their food plants, e.g., http://janzen.sas.edu and [2]–[15]. In 1981, JMB, an evolutionary taxonomist focused on problems involving species and genera, began to read the same newspaper in response to the classical plea of the field ecologists for identification of the adult skippers being reared. His reading intensified as the years passed and the sample size exploded. In 2003, biodiversity geneticists PDNH, MH, and CB began to analyze distinctive 650-letter words of mitochondrial DNA that they extracted from thousands of pieces of this newspaper passed to them. Here we offer a collage of observations and conclusions-in-progress from our many and various readings through 2009. This account is the application of ecological and taxonomic literacy to a taxonomically circumscribed fraction of the species, specimens, and natural history of a complex tropical place. Costa Rica has long been heavily studied for its Lepidoptera biodiversity, e.g., [16]–[19], but its northwestern corner was generally ignored before 1978, owing largely to its long distance from the national seats of economic and political power in the coffee-growing San Jose area, a climate very different from most of that of ACG.

The biologist studying in complex tropical habitats is constantly plagued with how to discriminate and identify the innumerable specimens of similar insect species that can be encountered in one place with even a single survey method (e.g., Malaise trapping, light trapping, sweep sampling, foliage gleaning, caterpillar rearing, screening of vertebrate gut contents). For many decades a standard solution has been to designate look-alike specimens as “operational taxonomic units” (OTUs) in the hope that they approximate species, and then to get on with whatever ecological or biodiversity analysis is the goal, e.g. [20]–[24]. Field biologists almost never have the luxury of a taxonomic specialist standing at their side. Even when they are so fortunate, the taxonomist is usually a species-level specialist on only one slice of the taxonomic spectrum and is hampered by the lack of a museum reference collection, a library, a laboratory, and enough time to puzzle out what might be a species complex as opposed to a single species. And this taxonomic impediment is exacerbated by poorly known groups and by such intraspecific phenomena as polymorphism, sexual dimorphism, disparate developmental stages, ecophenotypic variation, etc. For getting at a host of applied and basic biology questions, this Gordian knot begs to be gnawed through (and see [25]).

Here we describe how the addition of DNA barcoding—species identification through information from a standardized 650 base pair section of mitochondrial DNA [26]–[32] – to “traditional” taxonomic practice has stimulated and facilitated the biodiversity inventory of about 303 morphologically defined species, and an additional 44 possible species as signaled by distinct clusters of barcodes, in two sister subfamilies of tropical skipper butterflies in one place. But given our extensive ecological and life history information on these species (food plants, caterpillar morphology/colors, microdistribution - see http://janzen.sas.upenn.edu, and [7], [33]), our large (and ever growing) samples of them, and their prior, extensive, morphologically-based, taxonomic history (Table S1), we emphasize the animals themselves. In this study we treat the DNA barcoding laboratory at the Biodiversity Institute of Ontario at the University of Guelph as if it were a pocket gadget yielding hardly more than an iterative DNA comparison with a growing DNA reference library. And we see what happens. We deal here only with data from adult specimens reared from wild-caught caterpillars, and only those that yielded DNA barcodes greater than 550 bp in length.

The ACG inventory of caterpillars and their presence in trophic webs is being done for various reasons: 1) to satisfy simple academic curiosity, 2) to know what is where, and when, in order to assist fine-tuning and prioritization of ACG conservation and restoration management decisions, 3) to establish an unparalleled database for both the scientific community and the inquisitive public at large, and 4) to serve as a vehicle for learning and developing protocols for more complex information gathering, management, and delivery by ACG staff – a.k.a. parataxonomists [1], [7], [33] – not previously trained in these skills.

This study is a test of whether DNA barcoding “works” only in the sense that daily reading a newspaper or web site is a test of whether literacy “works”. This study is also part of an on-going examination of the additional biodiversity that appears when we DNA barcode a huge and complex biota of tropical butterflies [11]. Here we focus on the Eudaminae and Pyrginae, two species-rich subfamilies of the family Hesperiidae, because we have invested 30 years in finding and rearing many thousands of their caterpillars and taxonomically processing their adults. However, this exploratory philosophy is not meant to be restricted to these two subfamilies in any way, e.g. [8], [34].

### The place as a biophysical unit

**Figure 1:**
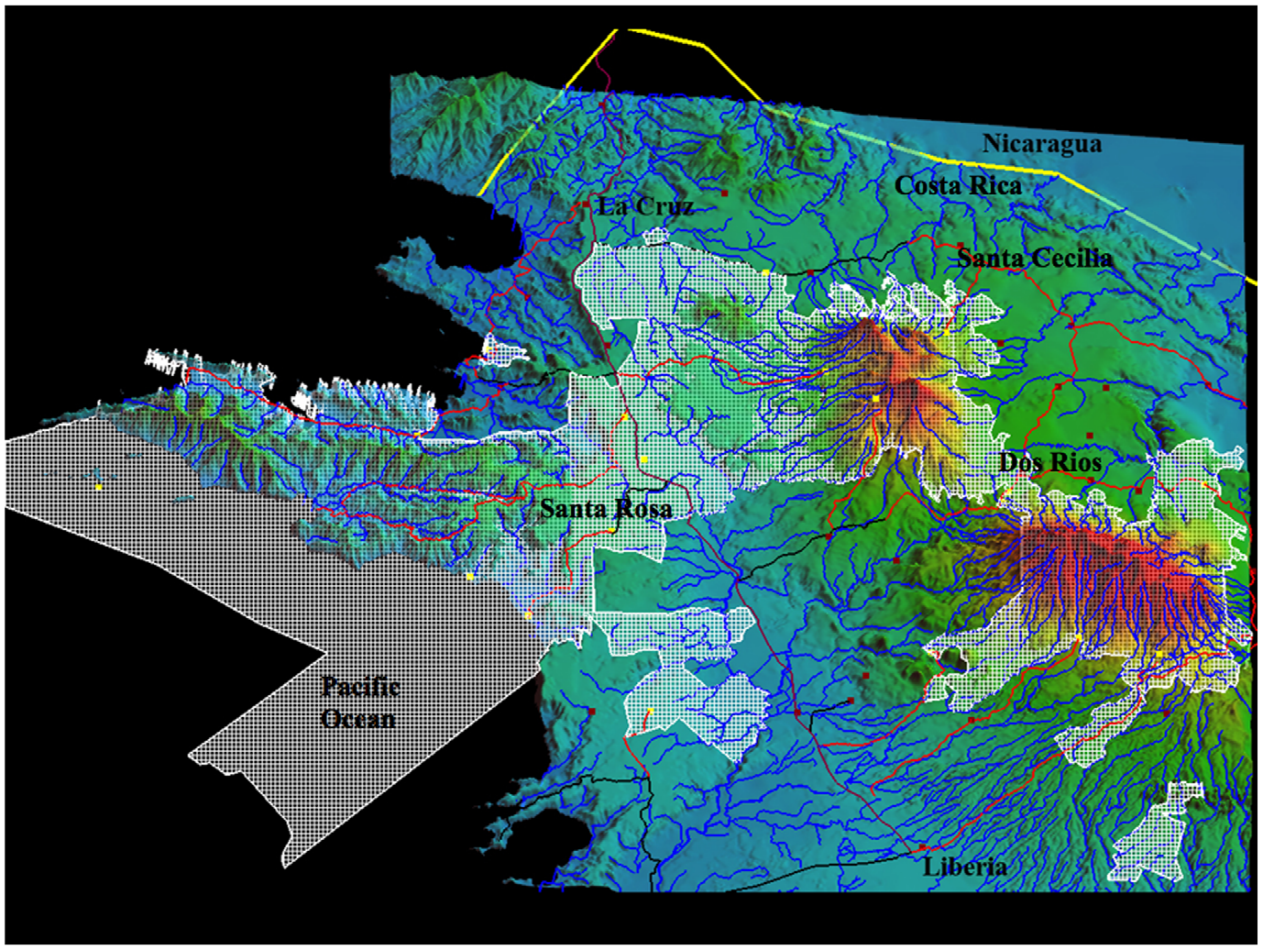
A contour map of Area de Conservacion Guanacaste (ACG: white grid). These 163,000 ha extend from 6 and 18 km out into the Pacific Ocean, eastward over three volcanos (1400–2000 m), and then down to 70 m elevation in the Caribbean lowlands. Red is the highest elevation, blue is the lowest elevation, and green to yellow is intermediate elevation. Blue lines are watercourses (largely seasonal on the Pacific side), while red or black lines are roads.

**Figure 2:**
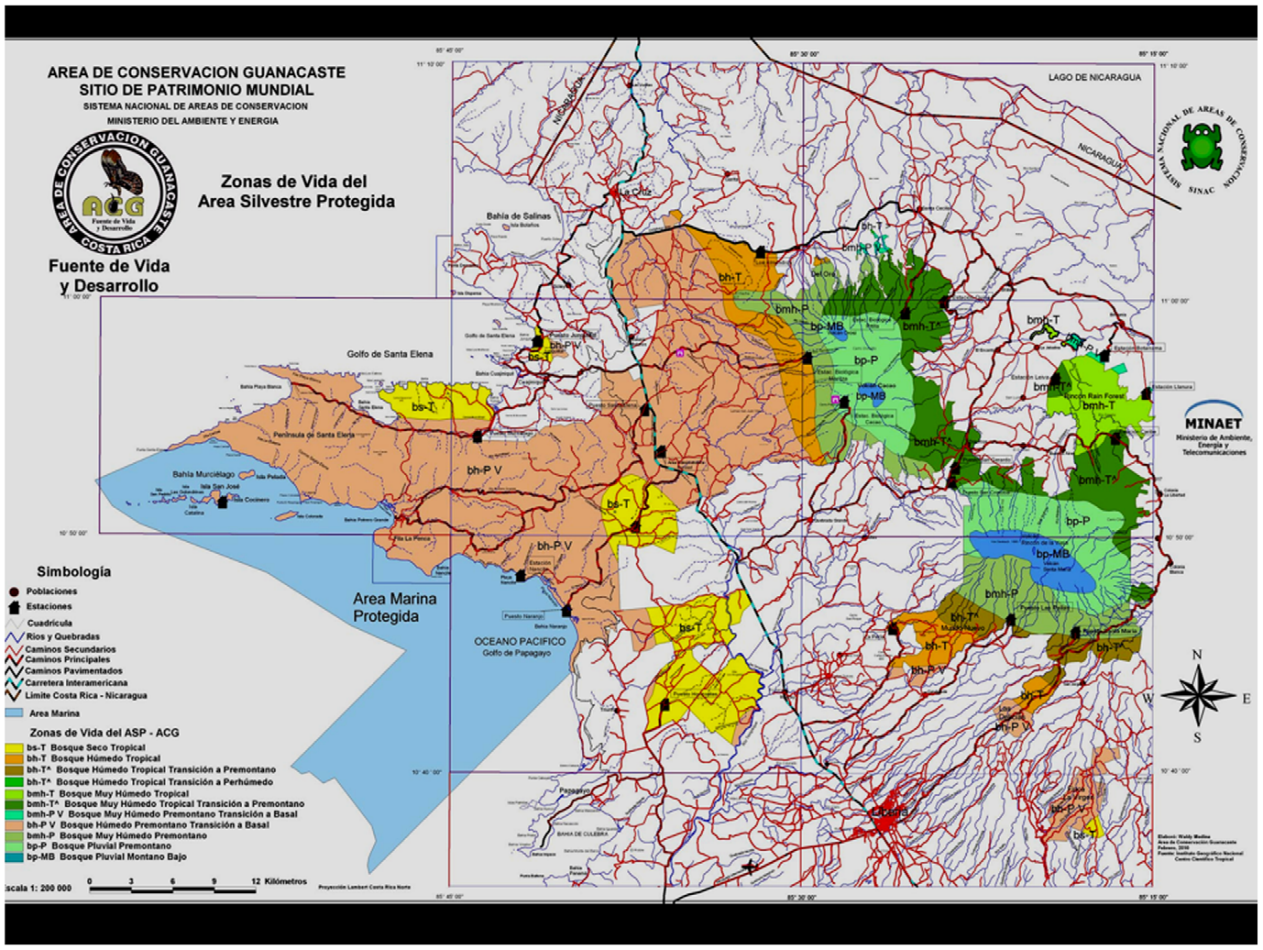
A Holdridge Life Zone map of Area de Conservacion Guanacaste (ACG). These 163,000 ha (colored area) extend from 6 and 18 km out in the Pacific Ocean, eastward over three volcanos (1400–2000 m), and then down to 70 m elevation in the Caribbean lowlands. The blue on the left (east) is Pacific Ocean, the yellow-brown-orange is dry forest, the greens are categories of rain forest, and the dark blue on the right is (shrinking) cloud forest.

Area de Conservacion Guanacaste (ACG) is a single decentralized unit of the Ministerio del Ambiente, Energia y Telecomunicaciones (MINAET; Ministry of Environment, Energy and Telecommunications) covering about 2% of Costa Rica in the northwest corner, slightly south of the southern border of Nicaragua (Figure 1–2). Comprising 1,200 km^2^ of terrestrial habitat (centered at 10.8 Latitude, −85.6 Longitude), it is a transect from Pacific coast mangroves across lowland dry forest (dry season deciduous), up the slopes of three volcanos to cloud forest (1400–2000 m), and down into Caribbean lowland (90 m) rain forest. It is only 85 km from east to west, yet contains portions of as many as eight Holdridge Life Zones (Figure 2) within mosaics as small as 15 km of linear direction and 50 km^2^. Nearly all of the ACG lowlands have been subjected to four centuries of light to intense cultivation, logging, burning, hunting, ranching, and other forms of habitat destruction followed by explicit protection and restoration beginning in 1971 and intensifying from 1985 onward [35]–[37]. The outcome is a mosaic of all imaginable ages and kinds of secondary succession intermingled with tiny to medium-sized fragments of approximations of intact forest (more intact in upper elevations than lower), as well as severe blurring and elimination of interdigitated boundaries between habitats and ecosystems [38]. All of the ACG region has also now experienced at least two decades of notable drying and increasing weather unpredictability, rendering it yet more difficult to know if the marked population changes are being generated by climate changes, successional changes, insularization of the ACG ecological island in the agroscape, species-by-species biological serendipity, and/or interactions among all of these.

Because ACG dry forest, rain forest, and cloud forest intergrade and interdigitate over a relatively short distance, individuals of a volant species that “occupies” one of these ecosystems can, and probably often do, contact at least the margins of the other two. Hence, for the purposes of the inventory, we consider ACG to be “one place,” while fully recognizing that the individuals and populations of its species are sensitive to its ecosystem, elevation, and seasonal heterogeneity, and are spatially and seasonally organized accordingly. In other words, all ACG organisms are “sympatric” at one scale, but variously parapatric to allopatric at other scales. Despite the tightly packed mosaic of habitats, disturbance types, and ecosystems within ACG - an area the size of a major national capital city and its suburbs - it has been historically commonplace for biological collectors to focus on its dry forest, or its rain forest, or (rarely) its cloud forest, thereby creating the illusion of three places, three biological systems. To the collector, one ACG species may be seen as characteristic of dry forest and another as characteristic of rain forest. However, there are many tens of square kilometers in ACG where the dry forest blurs rapidly into the adjacent rain forest both because the rainfall and seasonality gradient is very steep (less than a kilometer wide) and variable between years, and because of anthropogenic habitat modification. Creating a pasture in an ACG Caribbean rain forest turns that piece of rain forest partly into a microclimatic near mimic of Pacific dry forest. Equally, restoring a dry forest pasture to forest re-creates somewhat rain forest-like conditions absent from the site for centuries. Cutting a Pacific riverside “rain forest” along its river that originates in the volcano cloud forest converts it into dry forest. The outcome is that it is common for intense sampling to find members of the “Pacific dry forest biota” shoulder-to-shoulder with members of the “Caribbean rain forest biota” in the same square kilometer of the ecotone. This is especially true of the north-facing slopes (300–1000 m elevation) of Volcan Orosi, where the Caribbean rain forest to the east joins the Pacific dry forest to the west, and also in the low elevation (500–700 m) pass between Volcan Cacao and the Volcan Rincon de la Vieja massif (Figure 1). Apparently perfect ecosystem parapatry, so simplistically mapped (Figure 2), is actually blurry and complex parapatry within ACG, and within the normal flight distances of any volant species, and cannot be easily invoked to explain how seemingly very closely related species, e.g. [34] remain genetically distinct.

The ACG ecological island in the agroscape contains an estimated 325,000 species “bigger than microbes” [39], or about 65% of those occurring in Costa Rica, whose biota, in turn, is nearly as large as that of North America north of Mexico (and about 4% of the world's). Judging from our 30-year intense inventory of both caterpillars and free-flying adults, the size of other higher taxa, and the discovery of many cryptic species through DNA barcoding, e.g. [8], [13]–[15], ACG probably has a Lepidoptera fauna of about 12,500 species. Of these, about 1,100 species are butterflies of the families Riodinidae, Lycaenidae, Papilionidae, Nymphalidae, and Pieridae. About 450 species and presumed species of skipper butterflies have been found as caterpillars, and the likely total for Hesperiidae is about 550 species. In this examination of the hesperiid subfamilies Eudaminae and Pyrginae of ACG (formerly classified as Pyrginae and Pyrrhopyginae, see [40]), we treat about 303–347 species reared from wild-caught caterpillars, and the caterpillars of all but two of these species eat leaves of dicotyledonous plants. Caterpillars of the excluded species, which comprise hesperiid subfamilies Hesperiinae and Heteropterinae, eat monocotyledonous plants. There are about three times as many species of dicot-eating skippers in ACG as there are in all of North America north of Mexico [41].

By “species” we mean an array of individuals of what appears to be common descent occurring in ACG – an array of individuals that displays an array of traits - be they morphological (including DNA barcodes) and/or ecological - that are not shared with other ACG species. We presume that each of our species is a “single” entity in community interactions. Gene exchange among these ACG species is hypothesized to be restricted to occasional hybridization events, if at all. We do not formally describe what we think is probably a real biological species (i.e., what is effectively the “candidate species” of [25]) as a new species based on its DNA barcode alone. Rather, we do this when mutually supporting morphological, ecological, and barcode characters are evident, e.g. [8], [27], [34]. We do, however, initially view the species described by others, often more than 100 years ago (Table S1), as formal taxonomic species-level biological units until demonstrated otherwise.

When we suspect that a previously described species may actually consist of two or more, the interim naming convention now used throughout the inventory is to append DHJ01, DHJ02, etc. to the traditional specific epithet (e.g., *Astraptes* janeiraDHJ01, *Astraptes* janeiraDHJ02) and italicize only the generic epithet to emphasize that the species epithet is an interim name.

### The inventory of ACG caterpillars, their parasitoids, and their food plants

The inventory of Lepidoptera caterpillars of all taxa except leaf miners [11] began in dry forest in 1978 (the then Parque Nacional Santa Rosa, today Sector Santa Rosa of ACG). It gradually spread throughout ACG as the conservation area expanded [33], [36], [42], largely owing to the realization that the “dry forest” ecosystem was an integral part of the biology of all of ACG [42]. From 1–33 Costa Rican parataxonomists [1], [7], [11], [33] haphazardly, intensively, and structuredly search the vegetation of all plant species in all habitats and ecosystems for any species of free-living caterpillars. They rear them, record their rearing data individually, and individually voucher the specimens [11]. In 1978–2009, this process produced about 450,000 rearing pedigrees and about 125,000 pinned/spread adult Lepidoptera voucher specimens of at least 5,000 species. There are 97,700 rearing records of Hesperiidae. As noted above, they come from about 450 species, of which about 303–347 belong to the two dicot-eating, species-rich subfamilies Eudaminae and Pyrginae (about 65,200 rearing records). This activity has generated 19,164 pinned and vouchered museum specimens of these two subfamilies deposited in the National Museum of Natural History (USNM) at the Smithsonian Institution, Washington, DC (and ∼7,000 duplicates in INBio in Santo Domingo de Heredia, near San Jose, Costa Rica, and in the McGuire Center for Lepidoptera and Biodiversity at the University of Florida, Gainesville, Florida).

**Figure 3:**
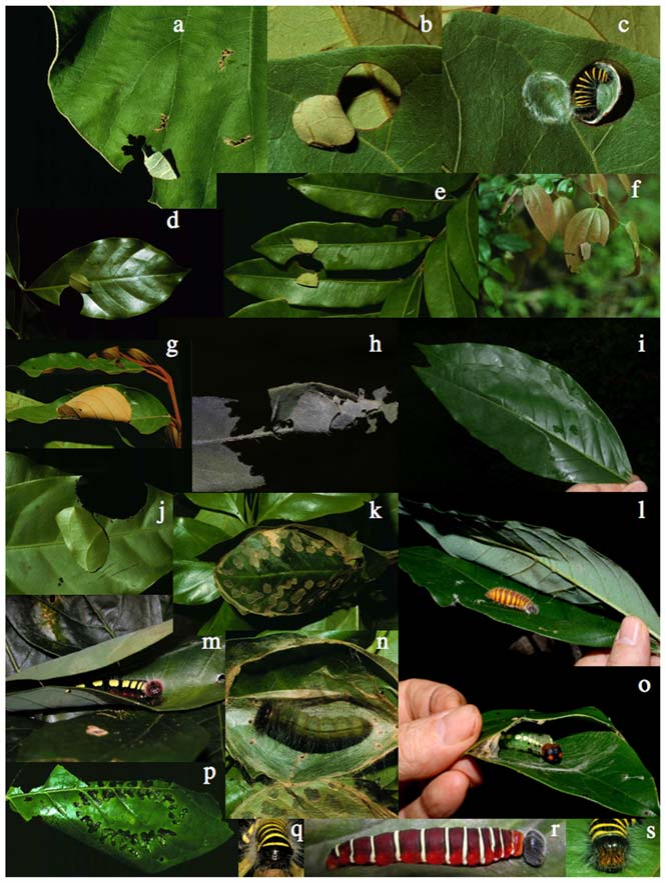
Some representative caterpillar shelters of ACG Eudaminae and Pyrginae (Hesperiidae). a) *Bungalotis astylos* first instar shelter, 94-SRNP-9715, b) *Melanopyge* Burns01 first instar shelter, 02-SRNP-14905, c) *Melanopyge* Burns01 first instar revealed by opening the shelter, 02-SRNP-14905, d) *Myscelus belti* first instar shelter, 02-SRNP-14661, e) *Bungalotis diophorus* two first instar shelters, 00-SRNP-11373, f) *Entheus* Burns02 second instar shelter, 01-SRNP-9788, g) *Pyrrhopyge zenodorus* penultimate instar shelter, 94-SRNP-707, h) *Urbanus* doryssusDHJ01 second instar shelter, 08-SRNP-30519, i) *Astraptes* INGCUP last instar shelter constructed of two leaves, 08-SRNP-35599, j) *Venada nevada* third instar shelter, 97-SRNP-1622, k) *Myscelus belti* last instar shelter, 99-SRNP-266, l) *Astraptes* INGCUP last instar exposed by opening the shelter in I) above, 08-SRNP-35599, m) *Astraptes* LONCHO last instar in its lightly rolled shelter, 03-SRNP-4343, n) *Myscelus belti* last instar exposed by opening the shelter in k) above, 99-SRNP-266, o) *Ridens panche* last instar facing off at the invading hand that has just opened one end of its shelter, 08-SRNP-35705, see [12], p) *Atarnes sallei* last instar shelter, 84-SRNP-1652, q) *Melanopyge* Burns01 last instar rear view, 05-SRNP-3132, r) *Astraptes apastus* penultimate instar exposed, 03-SRNP-21865, s) *Melanopyge* Burns01 face-on view, 05-SRNP-3132.

The inventory has been especially thorough for these two subfamilies because their species 1) feed only on dicot foliage (with but two exceptions to date: *Urbanus teleus* and *Cyclosemia subcaerulea*), which is generally easier to search and identify than is monocot foliage, 2) construct relatively conspicuous and semi-permanent leaf-and-silk shelters, e.g. [43] in the foliage (Figure 3), 3) are very food plant species-specific, 4) are particularly tolerant of primitive rearing conditions, 5) have been of enough interest to collectors and taxonomists, for about two and one-half centuries, that many taxonomic puzzles have been worked out, 6) have a focused and experienced hesperiid taxonomist (JMB) working closely with the inventory, and 7) are generally species-level identifiable as caterpillars from the combination of morphology (including colors) and food plant selection. At this date only five species that are known to occur in ACG have not been reared, though there must be at least another 50 species, as inferred from their ecological and geographic ranges elsewhere in the Neotropics.

In 2003, we began to DNA barcode a selection of the reared skippers already deposited in the USNM, Smithsonian Institution, and thereafter, to barcode all newly reared ones that were destined for deposition in USNM. This barcoding was done for the express purpose of determining to what degree their barcodes correlated with their prior identification on traditional taxonomic grounds and their food plant and ecology. Barcodes not only distinguished known species but also indicated possible cryptic species and, on occasion, the close relationship of supposedly unrelated species, e.g. [8]–[10], [27]–[28], [44]–[45].

So far, we have successfully barcoded 9,094 (out of 9,700 attempted) voucher specimens of the reared species of ACG eudamine and pyrgine skippers in USNM (Table S1, S2, Figure S1). This is a work in progress with respect to finding “all” of the hesperiid species in ACG, and to determining where they are, what they are, what their caterpillars eat, etc. We wish to increase their visibility to all of society and, in doing so, to increase their chances of surviving through explicit conservation. This is also a work in progress with respect to analysis of the specimens and collateral data obtained. The story is tangled, diffuse around its margins, and decidedly incomplete. This is a dive into a large and complex fauna, a process distinct from the current trend of examining only those portions of a system that can be cleanly teased out as a single line of investigation or as the test of a single hypothesis.

## Results

Raw inventory results (Table S1 and NJ tree in Figure S1) tell much of the story for these eudamine and pyrgine skippers, and may be divided into three categories (A, B, C in Table S1).

(A) There are 9 species represented in the inventory by just one specimen. They are distinguishable from all others by facies and genitalia, by caterpillar/food plant (in the 7 species that have been reared), and by DNA barcodes. We do not consider them further in this report because their sample size is too small.

(B) There are 240 species that were morphologically (and ecologically) identified prior to being DNA barcoded and whose barcodes, in each case, form a single tight cluster in the NJ tree (Figure S1). Each of these unambiguous clusters of barcodes are 1–12% different from any other cluster (see scale bar at the top of page 2 of Figure S1) and are identifiable by their distinctness in combination with morphology (and ecology) rather than by a particular percent difference. We have no reason to consider each of them as anything other than single species in ACG (especially in light of our usually large sample sizes). To date, additional specimens of these species keep falling where expected in the NJ tree, and so we consider the tree to be fully reliable (and equivalent to adult morphology and ecology) in the identification of ACG specimens. Likewise, the combination of caterpillar morphology (i.e., form and color pattern) plus food plant can also be used to identify these 240 species in ACG (see caterpillar images at http://janzen.sas.upenn.edu), except for the 4-membered cluster of long-tailed Fabaceae-eating *Urbanus* mentioned in the next paragraph.

**Figure 4:**
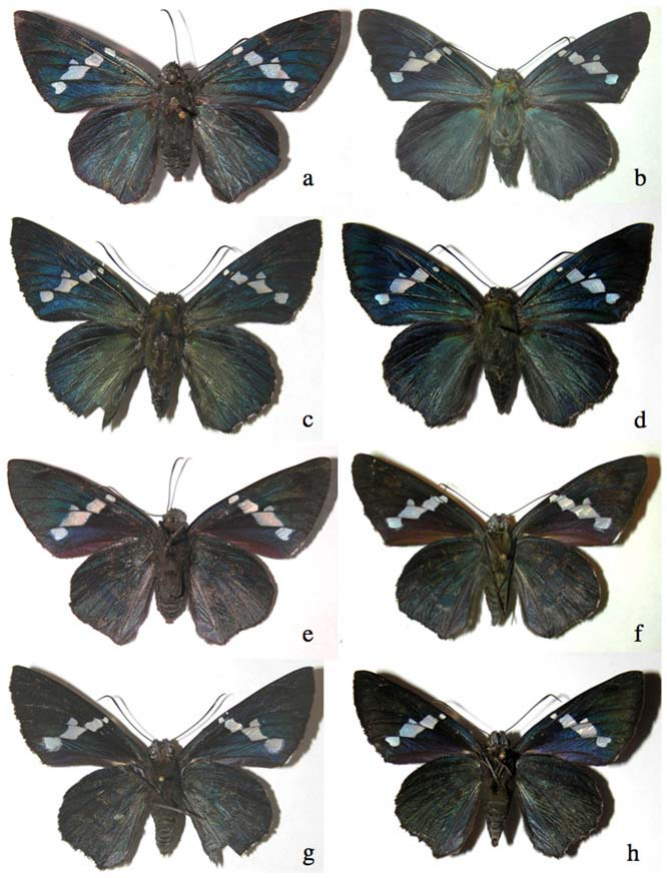
An example of ACG skippers that are indistinguishable by adult facies. Upperside, a) *Narcosius helen* female 00-SRNP-3919, b) *Narcosius samson* female 99-SRNP-5933, c) *Narcosius nazaraeus* female 09-SRNP-20099, d) *Narcosius colossus* female 05-SRNP-2080; underside, e) *Narcosius helen* female 00-SRNP-3919, f) *Narcosius samson* female 99-SRNP-5933, g) *Narcosius nazaraeus* female 09-SRNP-20099, h) *Narcosius colossus* female 05-SRNP-2080. Despite the identical facies of these four species, their genitalia, caterpillars (Fig. 5), and/or DNA barcodes (Fig. S1) allow easy and accurate identification.

**Figure 5:**
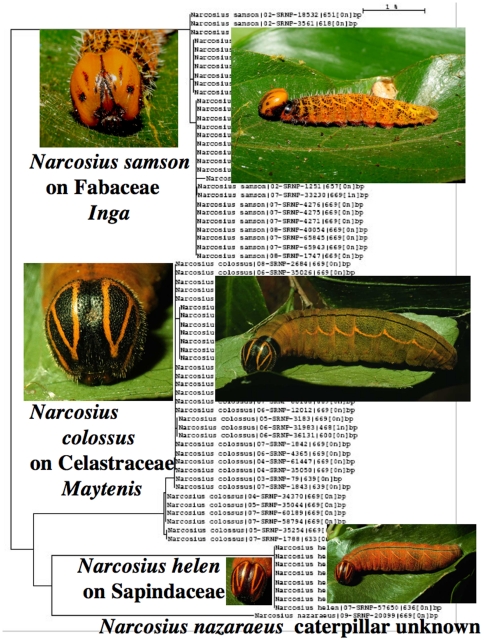
An example of indistinguishable adult ACG skippers (Figure 4) that are distinguishable by caterpillar facies, barcodes (Figure S1), or food plants. The distinctive last instar caterpillars of the three *Narcosius* that have been reared in ACG are here placed on their NJ tree (1% scale bar in upper right). *Narcosius samson* feeds on nine species of ACG rainforest *Inga* (Fabaceae) but does not extend to the population of the common dry forest *Inga vera*. *Narcosius colossus* feeds on *Maytenis* and *Gymnosporia* (Celastraceae) in ACG cloud forest, dry forest and rain forest. *Narcosius helen* feeds on five species of vines in the Sapindaceae in ACG dry forest and rain forest. The food plant of *Narcosius nazareus* is unknown, but all four species of caterpillars can be found in the same hectare of rain forest.

Many of these 240 species cannot be reliably identified by adult facies alone, despite intense scrutiny for characters that correlate with the groupings. Consider the four species of ACG *Narcosius* (Figure 4). Their caterpillars may all be found in the same square kilometer. They are superficially indistinguishable as adults but readily separable by any of the following features: genitalia morphology, caterpillar facies (Figure 5), food plant choice, or DNA barcodes (Figure 5, Figure S1). The single specimen of *Narcosius nazaraeus*, known in ACG only from an adult reared from a wild-caught pupa, would likely never have been noticed were it not for its distinctive barcode (Figure S1). Although four species of long-tailed brown (with green dorsal overscaling) *Urbanus* (*Urbanus proteus*, *Urbanus evona*, *Urbanus esmeraldus*, *Urbanus esta*), which resemble *Urbanus belli* (see comparisons in Figure 9 below), cannot be easily distinguished by their adult or immature facies or food plant, they can be distinguished by their barcodes (Figure S1) or genitalia.

**Figure 6:**
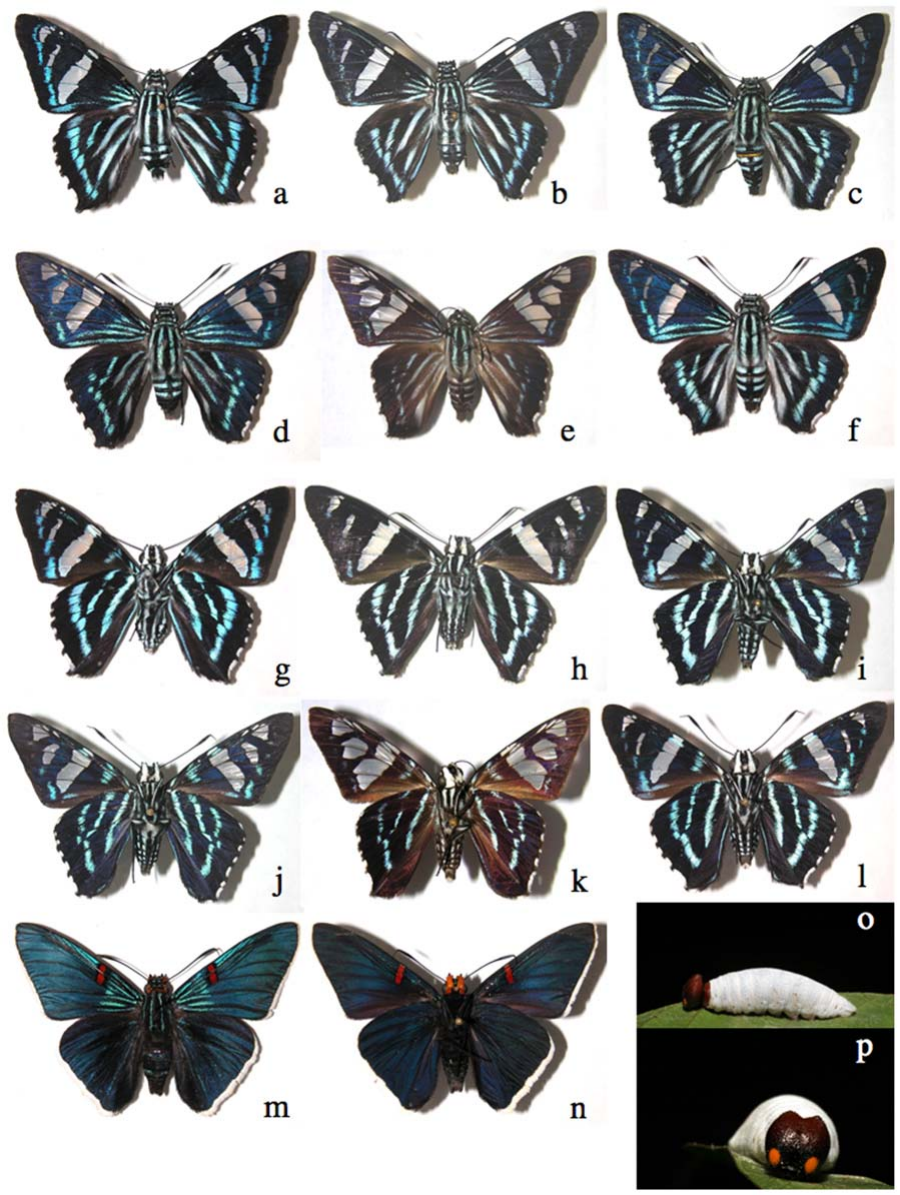
An example of congeneric similar ACG skippers that are partly distinguishable by their barcodes (see text and Figure S1). All males except the first specimen, upperside, a) *Phocides nigrescens* 02-SRNP-24513, b) *Phocides* Burns01 06-SRNP-42438, c) *Phocides* pigmalionDHJ02 06-SRNP-34234, d) *Phocides belus* 01-SRNP-18749, d) *Phocides* Warren01 00-SRNP-15186, f) *Phocides* pigmalionDHJ01 02-SRNP-14336; underside, g) *Phocides nigrescens* 02-SRNP-24513, h) *Phocides* Burns01 06-SRNP-42438, i) *Phocides* pigmalionDHJ02 06-SRNP-34234, j) *Phocides belus* 01-SRNP-18749, k) *Phocides* Warren01 00-SRNP-15186, l) *Phocides* pigmalionDHJ01 02-SRNP-14336, m) *Phocides lilea* upperside, n) Phocides lilea underside; o) and p) 5th (last) instar caterpillar of *Phocides* Burns01, which is essentially identical in appearance to the last instar caterpillars of all the other species in this figure.

A more complex example is the 7-species array of *Phocides* (Figure 6). All but two (*Phocides* pigmalionDHJ01 and *Phocides* pigmalionDHJ02) can be easily distinguished by their facies, but three of them (*Phocides* Warren01, *Phocides* pigmalionDHJ01, and *Phocides belus*) cannot be distinguished reliably by DNA barcode. However, these three barcode-sharing species are ecologically distinguishable: *Phocides* Warren01 is a coastal mangrove specialist, whereas *Phocides* pigmalionDHJ01 and *Phocides* pigmalionDHJ02 are in ACG dry forest and rain forest, respectively.

**Figure 7 pone-0019874-g007:**
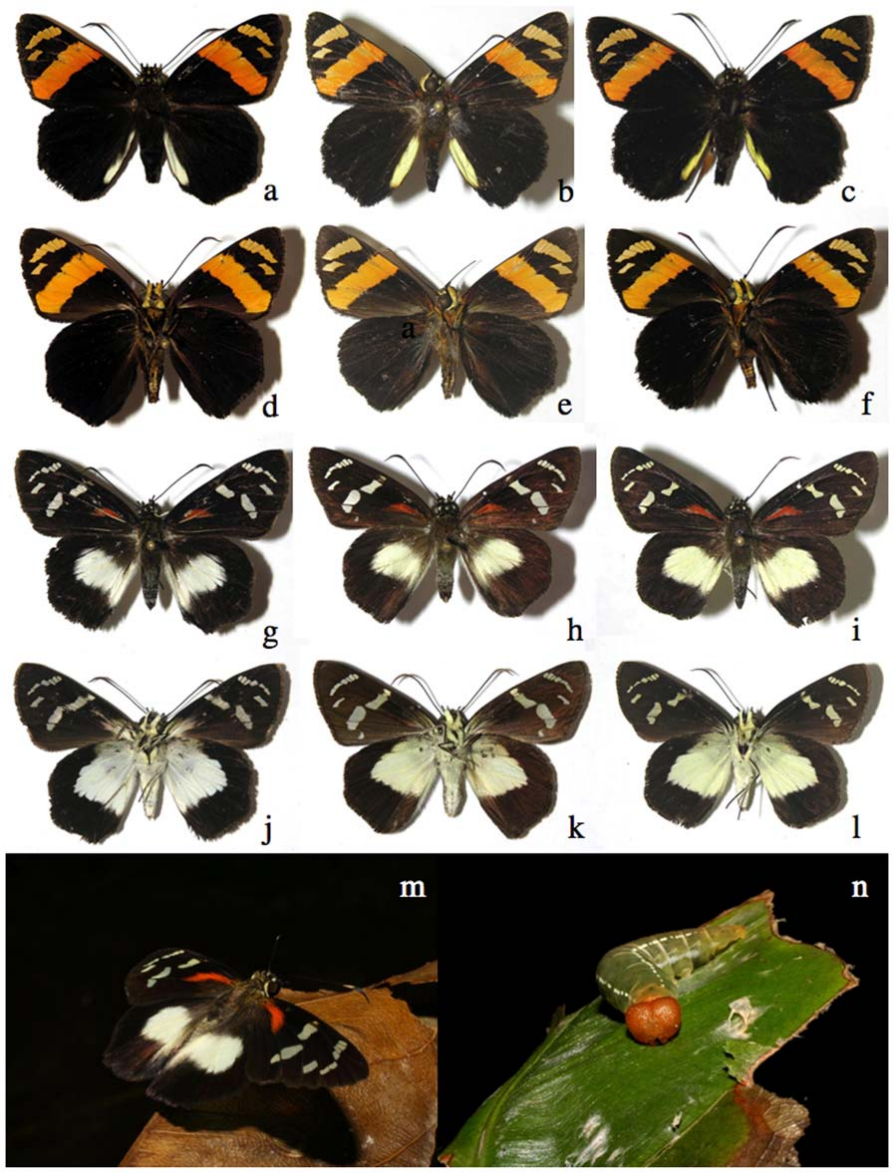
An example of similar ACG skippers that are readily distinguishable by their barcodes (Figure S1). Male upperside, a), and underside, d), *Entheus* Burns01 feeding on *Alfaroa* and *Lecythis* (Juglandaceae); same position, b) and e), *Entheus* Burns02 feeding on *Matudaea trinervia* (Hamamelidaceae) as in Figure 3f; same position, c) and f), *Entheus* Burns03 feeding on *Myrcia splendens* (Myrtaceae); female upperside, g), and underside, j), *Entheus* Burns01; same position, h) and k), *Entheus* Burns02; same position, i) and l), *Entheus* Burns03; m) adult female *Entheus* Burns01 in usual display position, n) 5th (last) instar caterpillar of *Entheus* Burns01 in defense position.

At first, the three species of *Entheus* (Figure 7) look nearly identical (except, for example, the males differ in the shade of their yellow-white sex patch in the anal fold of the hind wing), but their barcodes and food plants distinguish them; they cannot be distinguished by genitalia. Some other sets of confusingly similar species reared in ACG that are separable within-group by genitalia or barcodes are *Venada* (4 species), *Aguna* (6 of 8 species), *Telemiades fides* and *Telemiades* Burns01, *Celaenorrhinus* (3 of 6 species), females of *Cephise nuspesez* and *Cephise* Burns01, *Dyscophellus porcius* and *Dyscophellus* Burns01, *Epargyreus* (8 of 10 species), *Nascus* Burns01 and *Nascus* Burns02, *Nisoniades* Burns02 and *Nisoniades* Burns03, *Ouleus negrus* and *Ouleus* Burns01, *Polyctor cleta* and *Polyctor polyctor*, *Polythrix auginus*, *Polythrix asine* and *Polythrix mexicanus*, *Staphylus* and *Bolla* (13 species), and *Achalarus albociliatus*, *Achalarus toxeus*, and *Thessia jalapus*. Of course, the degree of confusability through visual inspection of facies within a set of similar species depends on the viewing conditions, condition of the specimens, number of specimens, and knowledge possessed by the viewer. As viewing moves from prolonged scrutiny by experts under museum conditions to hasty inspection by novices in the field, confusability of species within these sets greatly increases.

Among the 240 species with distinctive clumps of sequences (plus the 9 singletons) are two - *Polyctor cleta* and *Polyctor polyctor* - whose barcodes differ reliably, but by less than 1%, e.g. [34]. This is not bothersome for our analysis; we rely more on the distinctness of barcode clusters and their correlation with morphological and ecological traits than on any particular percent difference of one cluster from another, since barcodes are like any other taxonomic tool - they need to be considered along with other evidence. These parapatric species differ slightly in facies and sharply in both genitalic form and ecology, with *Polyctor cleta* occurring in dry forest and *Polyctor polyctor* in immediately adjacent rain forest [34]. (Though they are presumably each other's nearest living relatives, we do not mean to imply that they evolutionarily split into separate species within ACG; and see [46].) The existence of species pairs like this shows how important it is to check thoroughly for possible cryptic species even when the divergence between barcode clusters is slight. This is essentially the same as probing a species whose food plants seem unusually diverse, or whose genitalic variation looks bimodal, to see if that species comprises more than one.

**Figure 8 pone-0019874-g008:**
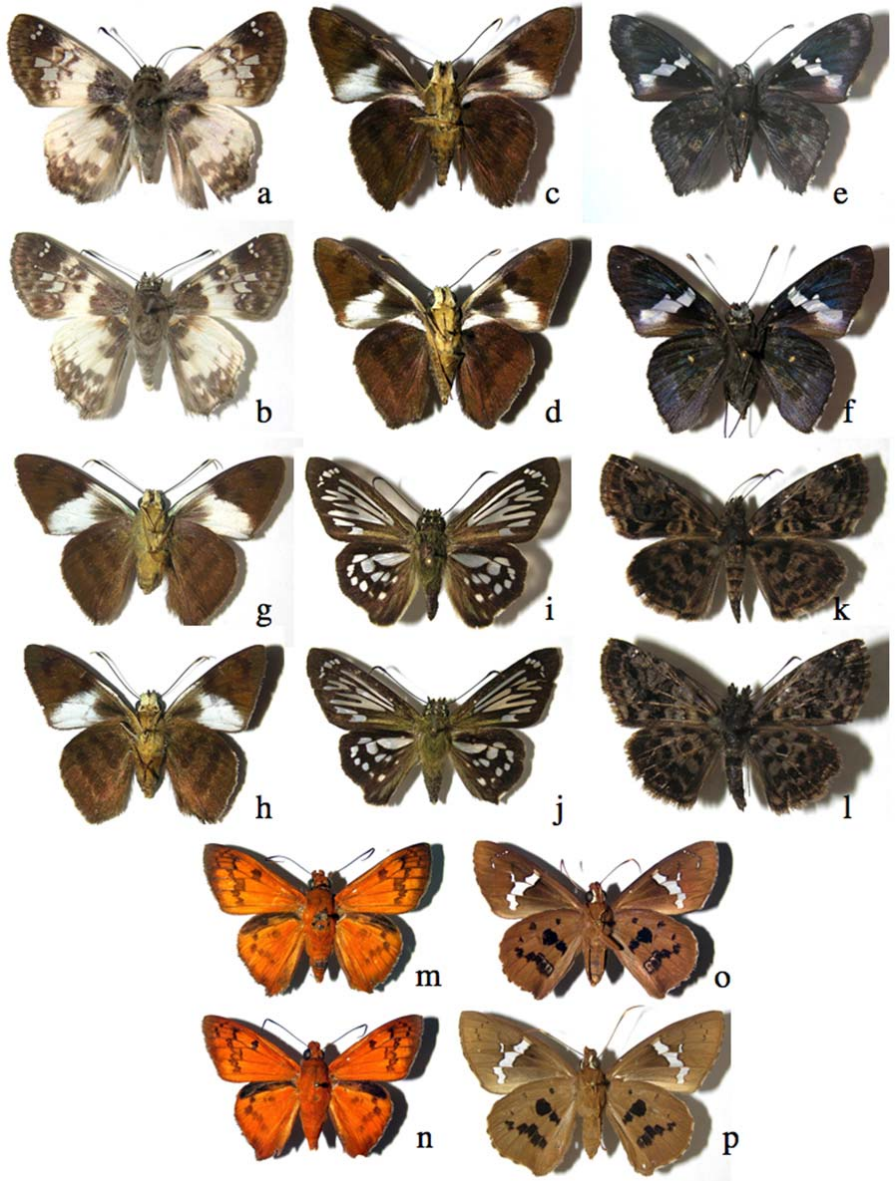
Examples of close pairs of barcode clusters (Figure S1) in which contain or may be two species. a) *Polyctor cleta*, 05-SRNP-61212, b) *Polyctor polyctor* 09-SRNP-20479 (barcodes differ by one base pair, genitalia differ in major ways, see [34]); c) underside A*straptes* hopfferiDHJ01, 05-SRNP-24692, d) underside *Astraptes* hopfferiDHJ02, 05-SRNP-19980 (no genitalia differences); e) underside *Astraptes* janeiraDHJ01, 06-SRNP-6959, f) underside *Astraptes* janeiraDHJ02, 05-SRNP-32361 (two species, genitalia differ); g) underside *Astraptes creteus* cranaDHJ01, 03-SRNP-4333, h) underside *Astraptes creteus* cranaDHJ02, 05-SRNP-35359 (genitalia similar); i) *Phanus* marshalliiDHJ01, 03-SRNP-16237, j) *Phanus* marshalliiDHJ02, 03-SRNP-16236 (likely two species, no genitalia differences); k) *Gorgythion begga* pyralinaDHJ01, 04-SRNP-50125, l) *Gorgythion begga* pyralinaDHJ02, 04-SRNP-56552 (no genitalia differences); m) upperside, male *Bungalotis* quadratumDHJ01, 01-SRNP-628, n) upperside, male *Bungalotis* quadratumDHJ02, 02-SRNP-20465, o) underside female *Bungalotis* quadratumDHJ01, 02-SRNP-14083, p) underside female *Bungalotis* quadratumDHJ02, 07-SRNP-66034 (no genitalia differences).

Four heavily probed barcode splits in *Dyscophellus phraxanor* (see discussion of Figure 10 under (C ii) below, and Figure S1) provide an outstanding example of variation that is not correlated with barcode variation, genitalia differences, or ecology. The females are dimorphic, with distinctive pattern morphs occurring equally on all food plants and within each of the four barcode clusters.

(C) There are 34 morphologically identified species, each of which, when barcoded, that split into two or more slightly to strongly different barcode clusters in the NJ tree (colored clusters in Table S1, Figure S1). Faced with these results one could analyze the 34 cases further with a) closer morphological examination, b) multigene (nuclear) probing, c) search for pseudogenes among the barcodes, d) search for correlated ecological traits (including food plants and microgeography), or any combination of these protocols. We have begun applying various of these protocols to the species in question, e.g. [8], [27], [34]. In some cases our results indicate one or more cryptic species corresponding to barcode splits, in some cases the jury is still out, and in others, there is no support for anything other than barcode polymorphism within a morphologically and ecologically defined species. For example:

- There are 11 cases (Table S1, dark green) where one (or more) of the subdivisions within a split cluster contains only 1–4 specimens, as opposed to a substantial number of specimens in the other(s) (e.g., *Aethilla* lavochreaDHJ01, *Astraptes* BYTTNER, *Astraptes* janeiraDHJ01, *Cephise* nuspesezDHJ03, *Eracon* cliniasDHJ02, *Phanus* vitreusDHJ03, *Ridens* pancheDHJ01, etc.). It is tempting to dismiss these cases as “variation”, rare morphs, analysis errors, “short” or incomplete barcodes, etc., and sometimes they are. However, there have been many cases in the ACG inventory where the oddly barcoding single specimen turns out to be the first example of a cryptic species that is later revealed by larger sample sizes or morpho/ecologic/microgeographic correlations. Two such newly discovered but as yet undeveloped cases are *Ridens* pancheDHJ01 and *Ridens* pancheDHJ02, and *Astraptes* janeiraDHJ01 and *Astraptes* janeiraDHJ02 (Figure 8). In both sets, the barcode differences prompted a second look that disclosed subtle facies and genitalia differences. Parenthetically, this also happened when four species of the hesperiine skipper genus *Perichares*, three of which were previously undescribed, were revealed by their barcodes within “one common species” [8]. The two specimens of *Polythrix* asineDHJ04 , with their highly divergent barcodes, would probably not have been discovered had very large samples of *Polythrix* asineDHJ01 and *Polythrix* asineDHJ02 (Table S1, Figure S1) not been barcoded. Frequently in the ACG Lepidoptera inventory as a whole, many tens of specimens of one member of a sibling pair have been found and reared before the first specimen of the second has surfaced. Each case of barcode splits must be thoroughly examined and judged on the basis of all available evidence. At present it appears unlikely that the single-specimen barcode segregates *Quadrus* cerialisDHJ01 and *Quadrus* cerialisDHJ02 reflect anything other than intraspecific variation within *Quadrus cerialis*.
- There are 22 cases where DNA barcoding has revealed a 2-, 3-, 4-, or even 11-way split in a long-known morphologically defined “species” that the inventory has documented with a substantial number of specimens (Table S1, yellow splits, blue being the morphologically defined legacy species; Figure 6–10). In each case, the field biology of the legacy species and the splits are being explored (DHJ and WH and the team of ACG parataxonomists), the facies, genitalia, and other morphological features are being examined (JMB), and the genetics are being probed (MH and PDNH). In the case of *Telemiades* chrysorrhoeaDHJ01 and *Telemiades* chrysorrhoeaDHJ02, the former has been found to be the real COI mitochondrial barcode and the latter a pseudogene captured only from females (and not from all of them). In these females, it appears that the pseudogene has outcompeted the real barcode for binding to the amplification primer, giving a misleading result. In numerous cases, barcoding has already led to the unpublished discovery of morphological, ecological, and small scale geographic differences analogous to those in published cases of closely related species among ACG hesperiids: *Astraptes janeira*, *Ridens panche*, *Spioniades abbreviata* and *Spioniades artemides*, *Autochton bipunctata* and *Autochton* Burns01. On the other hand, in at least two cases – *Astraptes anaphus annetta*, *Astraptes hopfferi* – we can find no other traits to suggest that the split is anything besides barcode polymorphism.

**Figure 9 pone-0019874-g009:**
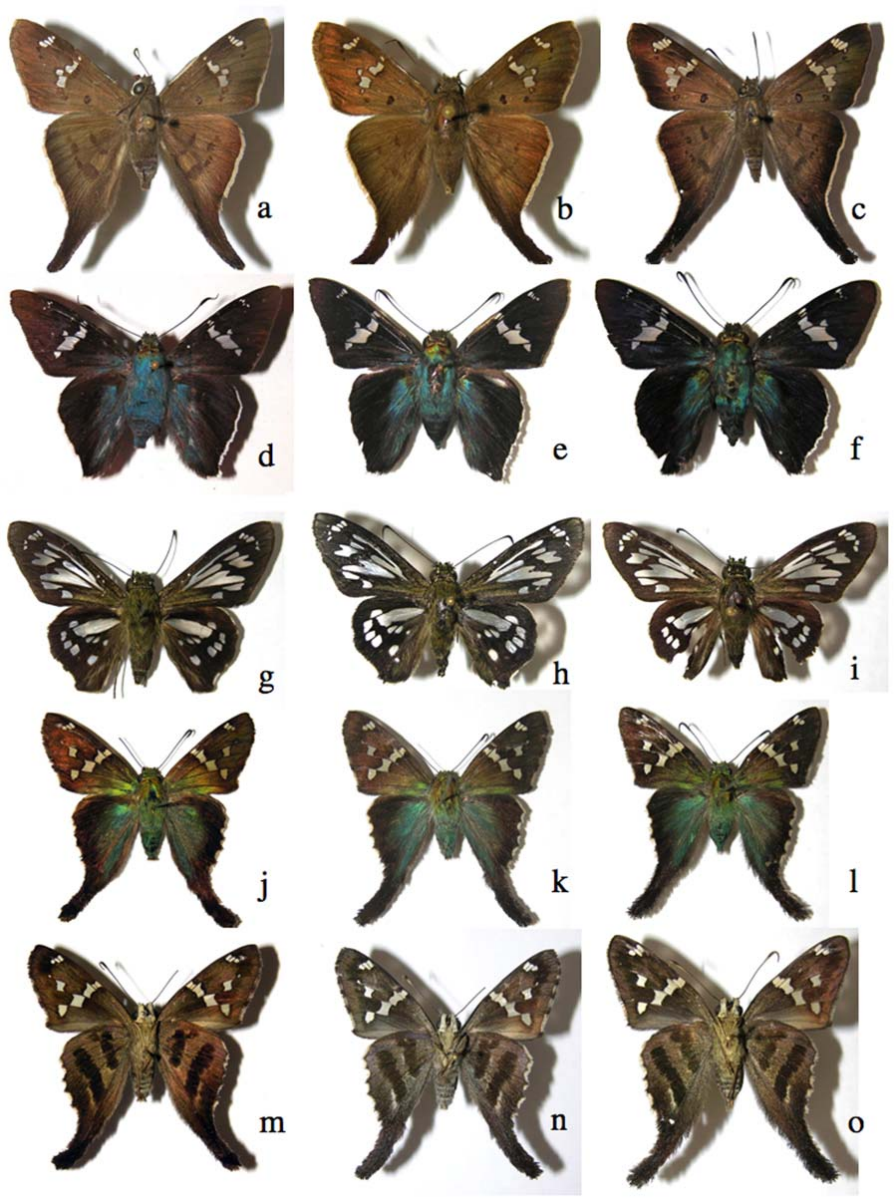
Examples of close triplets of barcode clusters in which there are or may be two or three species (Figure S1). a) *Polythrix* asineDHJ01, 00-SRNP-6494, b) *Polythrix* asineDHJ02, 00-SRNP-6495, c) *Polythrix* asineDHJ04, 04-SRNP-48896 (no genitalia differences between DHJ01 and DHJ02, strong genitalia differences with DHJ04); d) *Ridens* mephitisDHJ02, 03-SRNP-3595, e) *Ridens* mephitisDHJ03, 06-SRNP-36844, f) *Ridens* mephitisDHJ04, 06-SRNP-59545 (no genitalia differences, clear microgeographic differences among all three); g) *Phanus* vitreusDHJ01, 00-SRNP-2048, h) *Phanus* vitreusDHJ02, 07-SRNP-4607, i) *Phanus* vitreusDHJ03, 98-SRNP-4474 (no genitalia differences between DHJ01 and DHJ03, small genitalia differences between them and DHJ02); uppersides j) *Urbanus* belliDHJ01, 02-SRNP-2246, k) *Urbanus* belliDHJ02, 06-SRNP-2658, l) *Urbanus* belliDHJ03, 06-SRNP-43129 (no genitalia differences, but nuclear gene differences between DHJ01 and DHJ02, and DHJ03); m), n), and o) are undersides of same *Urbanus* specimens as in uppersides immediately above.

**Figure 10:**
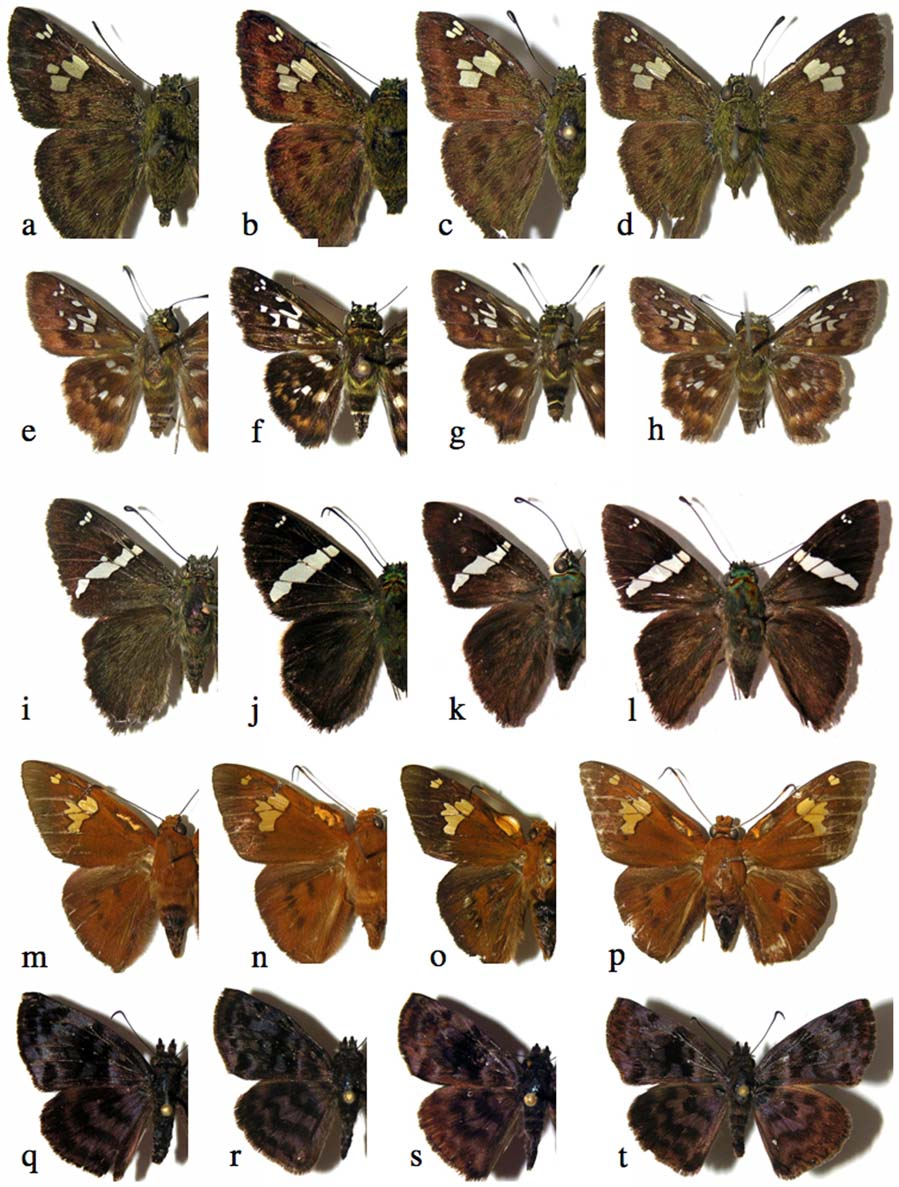
Examples of close quadruplets of barcode clusters (Fig. S1) in which there are or may be two to four species. a) *Telemiades* antiopeDHJ01, 07-SRNP-4183, b) *Telemiades* antiopeDHJ02, 02-SRNP-1003, c) *Telemiades* antiopeDHJ03, 08-SRNP-53, d) *Telemiades* antiopeDHJ04, 08-SRNP-66178 (no genitalia, food plant or microgeographic differences); e) *Udranomia* kikkawaiDHJ01, 02-SRNP-4127, f) *Udranomia* kikkawaiDHJ02, 05-SRNP-31092, g) *Udranomia* kikkawaiDHJ03, 02-SRNP-11244, h) *Udranomia* kikkawaiDHJ04, 02-SRNP-4194 (no genitalia differences, strong microgeographic differences among all four); i) *Autochton* Burns01DHJ02, 08-SRNP-1014, j) *Autochton* Burns01DHJ03, 05-SRNP-5444, k) *Autochton* Burns01DHJ04, 01-SRNP-2651, l) *Autochton* Burns01DHJ05, 00-SRNP-11187 (no genitalia differences, mild microgeographic differences, weak food plant differences); m) *Dyscophellus* phraxanorDHJ01, 99-SRNP-13329, n) *Dyscophellus* phraxanorDHJ02, 98-SRNP-6941, o) *Dyscophellus* phraxanorDHJ03, 07-SRNP-65189, p) *Dyscophellus* phraxanor, 08-SRNP-70094 (no differences among all four); q) *Ebrietas* anacreonDHJ01, 05-SRNP-24244, r) *Ebrietas* anacreonDHJ02, 06-SRNP-30796, s) *Ebrietas* anacreonDHJ03, 93-SRNP-5844, t) *Ebrietas* anacreonDHJ04, 07-SRNP-66037 (mild microgeographic differences, no genitalia differences). This is an upperside view of all specimens.

In all 11 and 22 cases, the barcode split of the species that goes under the legacy name does not lead to confusion with other morphologically defined (or ecologically or barcode defined) species, but, instead, simply raises the possibility that several species are hiding under one morphologically-based name. These splits await a species-level treatment as in *Cephise* [47]; *Drephalys* [48]; Pyrrhopygini [6]; *Venada* [49]; *Neoxeniades*, *Cobalus*, *Polyctor* [34]; *Perichares* [8]; *Thracides* [45]; *Porphyrogenes* [9]; *Neoxeniades* [10].

## Discussion

So far, the inventory of ACG wild-caught caterpillars of the Eudaminae and Pyrginae has found 303 morphologically defined species (not considering the additional information provided by DNA barcoding). The great majority already has names, most of them old (Table S1). Many species that do not are being described as new, using traditional taxonomic characters, e.g. [8], [9], [47]–[49]. In all probability, the three new species of *Perichares* would still be masquerading as “a single, common, polyphagous (palm- and grass-eating) species” had it not been barcoded and the resulting distinct barcode clusters then found to have ecological and behavioral as well as subtle morphological correlates [8]. If all of the splits found to date by DNA barcoding represent cryptic species, except for the conspicuous case of the pseudogene split in *Telemiades chrysorrhoea*, then the number of species inventoried increases from 303 to 347, a 13% increase. The experience to date with DNA barcoding of ACG butterflies and moths in other species-rich families and subfamilies with “large and showy” species suggests that this percent increase applies to all of them [11]. As expected, preliminary barcoding of species in relatively poorly studied taxa of small moths (e.g., Tortricidae, Elachistidae, Pyraloidea, Coleophoridae, Opostegidae, small Geometridae, small Noctuidae, etc.) shows a much greater percent increase in the number of species.

In all the 303–347 ACG species of reared Eudaminae and Pyrginae, the only ones with confusable full-length barcodes are three species of *Phocides*: *Phocides* Warren01, *Phocides belus*, and the dry forest *Phocides* pigmalionDHJ01. Serendipitously, all three can be distinguished from all others and from each other by facies alone (Figure 6). However there are pairs of species whose individuals can be confused if their barcodes are less than full length. A barcode of less than 650 base pairs may not separate the rain forest species *Polyctor polyctor* from the dry forest species *Polyctor cleta* (Figure 8) because they differ by just one base pair; full-length barcodes will [34]. Again, specimens of these two extremely similar species can be identified by both minor differences in facies and major differences in genitalia [34]. This extreme rarity of barcode failure in species discrimination is representative of all ACG family and subfamily-groups of large showy Lepidoptera [11].

If many of the similar species on either side of a shallow barcode split have a recent origin, then barcoding large samples might catalyze detailed studies of the ecology of sibling species earlier in their evolutionary histories than is generally the case. However, we are not suggesting that the large complex biota of ACG evolved *in situ*, or is even in any site-specific way evolving *in situ*. Rather, we believe that the ACG biota - though now on an ecological island in the agroscape - largely comprises somewhat to far more widespread continental species that have “ecologically fit” [50] themselves into the complex mosaic of ACG topography, weather, climate, and biodiversity.

Shallow to deep splits within a barcode cluster of an ACG “classically morphologically defined species”, suggesting the existence of one or more cryptic species, raise questions as to which (if any) of the barcode subclusters is conspecific with the holotype (or its equivalent). This is exacerbated by the fact that many thousands of species were described from one to a few specimens from widely separated neotropical countries in the 1800's or before (Table S1). Over time, it has often been assumed that look-alikes from diverse neotropical countries represent a single species [8] with slight geographic variation. Since this kind of “minor” morphological variation can signal the presence of several parapatric to sympatric similar species in an area as small as ACG, we are no longer confident that many of the broadly distributed neotropical “species” described long ago really are single biological entities [8], [45].

In light of our barcoding experience in ACG, we suspect that many of these widely distributed species will turn out to comprise multi-species complexes, usually with narrow habitat or ecosystem distributions but sometimes a large geographic range. For example, it will not surprise us to find that many of the 11 ACG species going under the 1775 name *Astraptes fulgerator* [27] are widely distributed in Mesoamerica at least, with others more restricted. And there may well be still other local cryptic species occurring only to the north or south of ACG. Unless the holotype for a name comes not only from Costa Rica but also from a locality that is ecologically equivalent to where an ACG barcode segregate occurs, it is problematic whether to apply the name to that segregate or to describe all of the segregates as new. The study of the biogeography of DNA barcode segregates within “established” morphologically defined Lepidoptera species in the tropics is in its infancy. These results raise the very real possibility that even when there is no split within ACG, there may be one (or more) elsewhere, that, when well understood, will elicit an “Of course those are separate species” response.

Inconvenient as this is for many kinds of legacy data, for modern studies accustomed to one-off identifications in the field, and for conservation and legislative efforts, it is a truth that cannot be ignored. On an ACG-specific basis it has sometimes made it impossible to interpret ecological data (such as parasitoid records) from before 2003, because not all voucher specimens were retained and thus cannot be barcoded to know to which barcode split the record belongs. For example, the inventory has applied the well-known name *Urbanus belli* to the low-elevation morphologically defined Asteraceae-feeding *Urbanus* that have no voucher (either because adults were discarded or because they were never obtained, thanks to caterpillars that succumbed to disease or parasitoids). But all three barcode clusters (*Urbanus* belliDHJ01, *Urbanus* belliDHJ02 and *Urbanus* belliDHJ03) (Figure 9) often occur in the same location feeding on the same Asteraceae; all three have strongly overlapping microgeographic distributions, each with different density peaks in ACG. This implies that when one thinks that one is “done” with the national inventory for this or that “well-known” species, it may not be so. As Costa Rica's national inventory conducted by INBio now expands to include DNA barcoding as a tool, its species-level biodiversity richness and geographic complexity is likely to substantially increase.

The seemingly subtle morphological differences that often correlate with each side of an ACG barcode split show that no matter how good our viewing technology, we still see the world from the viewpoint of a large diurnal vision-oriented mammal. This in turn leads us to view morphological differences that we can easily observe as somehow more important in the biology of species discreteness and “older” evolutionarily. While there may be large-scale truth to this, for any specific case the generality may not apply. The phenotypically distinctive *Phocides lilea* posed among its ACG congenerics in Figure 6 may be no older in its evolutionary separation from other *Phocides* than are the several similarly blue-white-black striped species, whose striking facies is conserved because it is that of a species-rich neotropical mimicry complex to which they belong. The hugely different-appearing male and female *Entheus* (Figure 7) and male and female *Bungalotis quadratum* (Figure 8) are of equal age within their respective species.

A preliminary view of intraspecific barcode diversity, in those ACG species that are truly a single taxon, comes from analyzing an approximately 10-specimen sample. (As a general rule, a smaller sample sent to Guelph (Table S1) reflects a lack of specimens.) Samples greater than 20 indicate that we had good cause to suspect two or more cryptic species, as yet unresolved taxonomically. The occasional very large samples within a species (Table S1 and Figure S1) are partly a reflection of the caterpillar inventory documenting 1) that what appear to be caterpillars of the same species (pre-2003), but eating different species of plants and in different parts of ACG, may not be conspecific, and 2) that it takes multiple rearings to get their parasitoids, whose presence in a wild-caught caterpillar is unknown until they are reared out [11]. One can find cryptic species within a morphologically defined species by pursuing variation in morphology and ecology with barcodes, or by pursuing barcode variation with targeted examination of variation in ecology and morphology [8]. Both routes require increasing the number of individuals barcoded, as well as the numbers of caterpillars found and reared. Although, at first glance, it would appear that rearing more adults may be unnecessary, they are generally needed for their morphological traits, at least until the taxonomic puzzle is resolved (at which time barcoding larval blood, turds, or other remains may be substituted for the laborious process of rearing).

Most cases of very large barcode samples per species stem mainly from four kinds of explorations. First, there are many seemingly single highly sympatric species that showed clean (though often shallow) splits early on, and continued to do so with ever increasing samples in the post-2003 inventory, yet no ecological or morphological correlate has been found (e.g., *Gorgythion begga pyralina*, *Telemiades antiope*, *Bungalotis quadratum*, *Autochton* Burns01, *Urbanus belli*, *Astraptes anaphus anetta*, *Udranomia kikkawai*, *Dyscophellus phraxanor*, etc. Figure 9–10).

Second, there is large barcode accumulation while “fishing” within a common species for what is known to be a hidden species that cannot be reliably captured without knowing its barcode (e.g., rare and sympatric females of *Telemiades* Burns01 within an ocean of *Telemiades fides*, rare *Dyscophellus porcius* hidden within common *Dyscophellus* Burns01, rare and semi-sympatric females of *Cephise* Burns01 hidden among numerous *Cephise nuspesez* females (males of these two species are readily distinguishable by facies).

Here, and in other similar situations, the Biodiversity Institute of Ontario at the University of Guelph has been used by the inventory as if it were a personal pocket barcorder to capture a needed taxonomic trait not visible in the field or museum. For example, another kind of search was conducted by barcoding apparent conspecifics from the three major ACG ecosystems - dry forest, cloud forest, and rain forest. For many species of ACG Lepidoptera, barcoding and close morphological examination have found that what appears to be a single species occurring in two of these ecosystems is actually a pair of broadly parapatric or semi-sympatric similar species whose barcodes may differ by few to many base pairs. Attempting to confirm or deny the presence of such pairs of cryptic species for any given morphologically defined species swells the barcode sample size (and see [34]).

Third, there have been a number of cases where the analysis of a single morphologically defined species produces a clean set of equal or near-equal barcodes except that one individual is off on a short but distinct side branch in the NJ tree (Figure S1). Is this seemingly deviant barcode a laboratory error, a pseudogene, a rare polymorph, or a single individual of another species? We often increased the sample size to attempt to find more of them. The specimen may or may not have what seem to be ecological correlates or slightly different morphology. Given that the goal of the ACG inventory is to get “all of them”, the detection of each rare singleton has provoked efforts to rear and barcode yet more specimens, while deliberately broadening the ecological and morphological net in the process (e.g., the four species found inside of what was initially viewed as one common and widespread species of *Perichares* [8]).

Fourth, there are a few cases where a morphologically defined species uses host plants that are in very different families (e.g., *Jonaspyge aesculapus* feeding on *Weinmannia wercklei* (Cunoniaceae), *Hampea appendiculata* (Malvaceae), and Lauraceae; *Astraptes enotrus* feeding on Dichapetalaceae and many species and genera of the very different Fabaceae). In the most spectacular case, barcoding of 1,130 specimens was extremely productive and necessary in teasing out the 11 species in the *Astraptes fulgerator* complex feeding on 11 plant families and 50+ species in all ACG habitats and ecosystems pooled [27] – a range of food plants and locations far greater than that of any other ACG butterfly.

Adding barcoding to an ongoing inventory [11], [44] increases both the species-level yield of the inventory and its costs. One large cost increase is finding and rearing more caterpillars of suspected cryptic species. A second reflects the need to retain larger numbers of vouchers for re-inspection once barcode results have been obtained, and the increased desire to save specimens for later (and retroactive) study in pursuit and understanding of cryptic species once their presence is confirmed. The latter has potential space and curatorial consequences for the museums that are housing voucher specimens, yet at the same time adds value to the specimens the museum already has.

Morphology-based taxonomy and specimen identification is a game of comparison and matching. The use of DNA barcoding to identify species and discover candidate species is as well, and similar caveats apply. The difference is that the items being compared/matched - letter strings - are less subjective, much easier to convey as code, and more repeatable to others distant in time or space. We all use morphological and ecological traits for identification and discovery of species because we find that this or that “key” character correlates with others, and because we feel that the suite of correlated information indicates the presence of a group of conspecifics. After DNA barcoding large numbers of individuals of large numbers of species in the same place, we have found that the same applies to barcode traits. Hundreds of species in one place can be separated perfectly by their DNA barcodes, and therefore are revealed by them. It is counterproductive to ignore the signal offered by a barcode split among the specimens of what is currently regarded as a single morphological- or ecological-based species.

The ACG eudamine and pyrgine Hesperiidae are now well positioned for phylogeographic exploration throughout Costa Rica, as well as Mexico and all of Central America, and, in that connection, the Neotropics as a whole. Morphological, ecological, and barcode characters will surely reveal some biological continuity and much discontinuity. We can no longer count on a few museum specimens of each “species” to reflect the biodiversity and distributions of these skippers, even in Central America. A mere listing of the legacy species names for a given country tells us far less than we thought it did just a decade ago. The complex skipper fauna needs a longer and deeper reading.

## Supporting Information

Figure S1 **NJ tree (BOLD TaxonID Tree) for all ACG barcoded Eudaminae and Pyrginae (Hesperiidae) skipper butterflies.** This Neighbor Joining (NJ)tree is a standard tool for identifying an unknown specimen, or revealing a potential undiscovered species, by comparing the barcode with the other available barcodes [26]–[32]. Similar barcodes cluster together, and their percent similarity is indicated by the length of the horizontal bar connecting it to others. However, we caution that this is not a phylogenetic tree. While a brief inspection shows that it contains substantial phylogenetic signal, in that members of a morphology-based genus usually appear in adjacent clusters of barcodes, higher levels of clustering of barcode clusters may only partly reflect what is currently considered to be the phylogenetic history of these taxa as based on morphology and other traits.
(PDF)

Table S1 **Summary statistics for all ACG reared Eudaminae and Pyrginae (Hesperiidae) skipper butterflies.** These were reared from wild-caught caterpillars 1978 to 2009, inclusive. The year of original description of the morphologically-characterized species is included so as to emphasize that these butterflies have long been subject to taxonomic examination in Costa Rica and elsewhere; they are not a neglected taxon, as compared with smaller and less attractive animals, and barcode revelation of cryptic species is a significant contribution to understanding their biodiversity. Blue records are those that split into distinct groups of barcodes (see NJ tree in Figure S1). Yellow records are those that we feel are, or are likely to be, representing cryptic, previously unnoticed species. Green records are those whose barcode cluster contains too few specimens to feel certain that it is significant, yet needs to be flagged for further sampling.
(PDF)

Table S2 **Accession codes for all specimens that are considered in Table S1.**
(PDF)
