# Supplementary material for: Reading the Complex Skipper Butterfly Fauna of One Tropical Place
Source: PLoS One. 2011 Aug 16;6(8):e19874. doi: 10.1371/journal.pone.0019874 (PMC3156701; doi:10.1371/journal.pone.0019874)
Supplement: Figure S1 — NJ tree (BOLD TaxonID Tree) for all ACG barcoded Eudaminae and Pyrginae (Hesperiidae) skipper butterflies. This Neighbor Joining (NJ)tree is a standard tool for identifying an unknown specimen, or revealing a potential undiscovered species, by comparing the barcode with the other available barcodes [26]–[32]. Similar barcodes cluster together, and their percent similarity is indicated by the length of the horizontal bar connecting it to others. However, we caution that this is not a phylogenetic tree. While a brief inspection shows that it contains substantial phylogenetic signal, in that members of a morphology-based genus usually appear in adjacent clusters of barcodes, higher levels of clustering of barcode clusters may only partly reflect what is currently considered to be the phylogenetic history of these taxa as based on morphology and other traits. (PDF) [file pone.0019874.s001.pdf]

BOLD TaxonID Tree

Project	:	BOLD PROCESSDS[NO CODE]
Date	:	4-April-2011
Data Type	:	Nucleotide
Distance Model	:	Kimura 2 Parameter
Codon Positions	:	1st, 2nd, 3rd
Labels	:	SampleID, Sequence Length,
Filters	:	Length > 300
Colorization	:	[blue]=Stop Codons [red]=Contamination or misidentification
Attachment	:	Photographs & Spreadsheet

Sequence Count	:	9397
Species count	:	356
Genus count	:	102
Family count	:	1
Unidentified	:	0

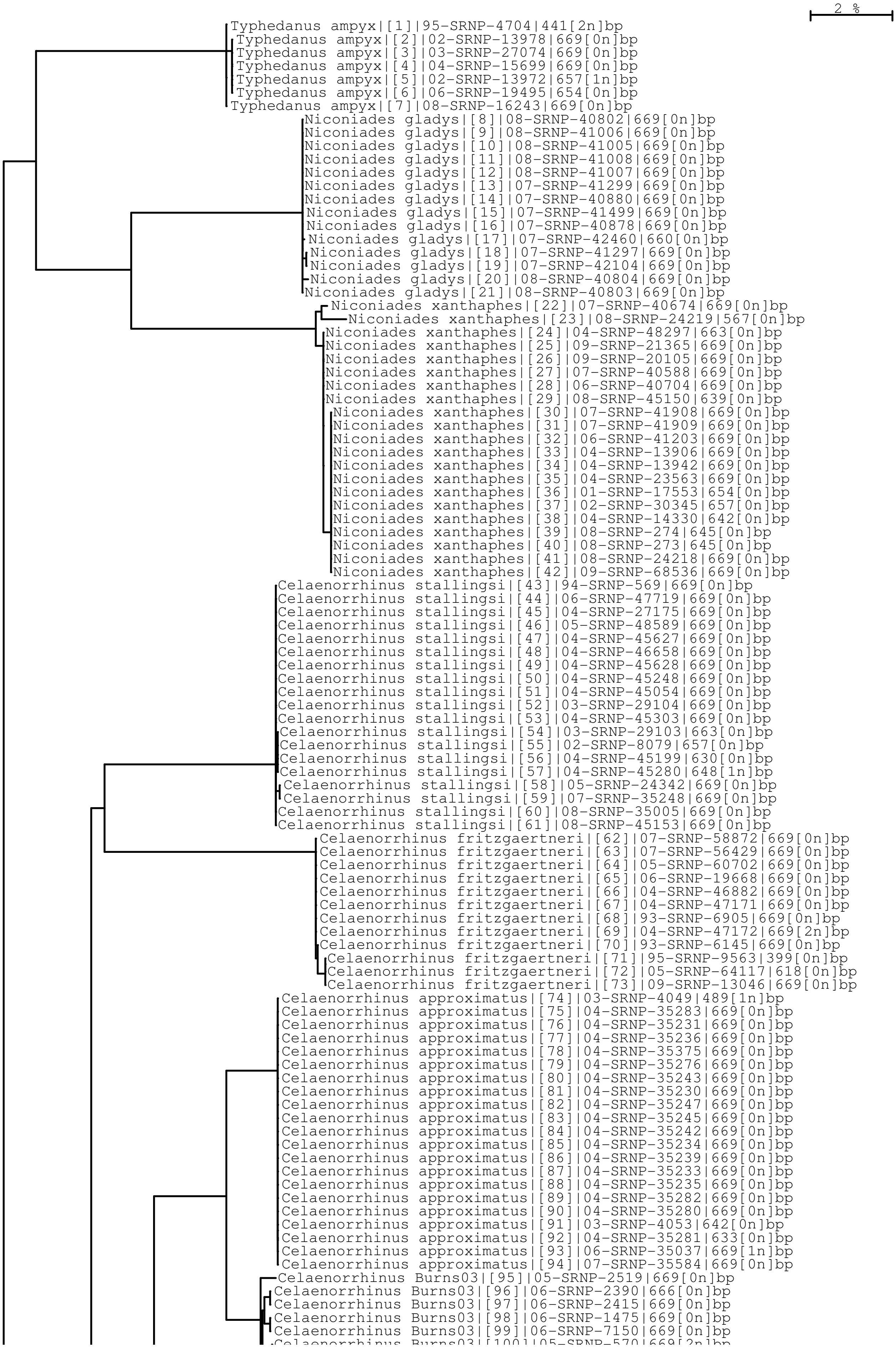
2 %
Typhedanus ampyx|[1]|95-SRNP-4704|441[2n]bp
Typhedanus ampyx|[2]|02-SRNP-13978|669[0n]bp
Typhedanus ampyx|[3]|03-SRNP-27074|669[0n]bp
Typhedanus ampyx|[4]|04-SRNP-15699|669[0n]bp
Typhedanus ampyx|[5]|02-SRNP-13972|657[1n]bp
Typhedanus ampyx|[6]|06-SRNP-19495|654[0n]bp
Typhedanus ampyx|[7]|08-SRNP-16243|669[0n]bp
Niconiades gladys|[8]|08-SRNP-40802|669[0n]bp
Niconiades gladys|[9]|08-SRNP-41006|669[0n]bp
Niconiades gladys|[10]|08-SRNP-41005|669[0n]bp
Niconiades gladys|[11]|08-SRNP-41008|669[0n]bp
Niconiades gladys|[12]|08-SRNP-41007|669[0n]bp
Niconiades gladys|[13]|07-SRNP-41299|669[0n]bp
Niconiades gladys|[14]|07-SRNP-40880|669[0n]bp
Niconiades gladys|[15]|07-SRNP-41499|669[0n]bp
Niconiades gladys|[16]|07-SRNP-40878|669[0n]bp
Niconiades gladys|[17]|07-SRNP-42460|660[0n]bp
Niconiades gladys|[18]|07-SRNP-41297|669[0n]bp
Niconiades gladys|[19]|07-SRNP-42104|669[0n]bp
Niconiades gladys|[20]|08-SRNP-40804|669[0n]bp
Niconiades gladys|[21]|08-SRNP-40803|669[0n]bp
Niconiades xanthaphes|[22]|07-SRNP-40674|669[0n]bp
Niconiades xanthaphes|[23]|08-SRNP-24219|567[0n]bp
Niconiades xanthaphes|[24]|04-SRNP-48297|663[0n]bp
Niconiades xanthaphes|[25]|09-SRNP-21365|669[0n]bp
Niconiades xanthaphes|[26]|09-SRNP-20105|669[0n]bp
Niconiades xanthaphes|[27]|07-SRNP-40588|669[0n]bp
Niconiades xanthaphes|[28]|06-SRNP-40704|669[0n]bp
Niconiades xanthaphes|[29]|08-SRNP-45150|639[0n]bp
Niconiades xanthaphes|[30]|07-SRNP-41908|669[0n]bp
Niconiades xanthaphes|[31]|07-SRNP-41909|669[0n]bp
Niconiades xanthaphes|[32]|06-SRNP-41203|669[0n]bp
Niconiades xanthaphes|[33]|04-SRNP-13906|669[0n]bp
Niconiades xanthaphes|[34]|04-SRNP-13942|669[0n]bp
Niconiades xanthaphes|[35]|04-SRNP-23563|669[0n]bp
Niconiades xanthaphes|[36]|01-SRNP-17553|654[0n]bp
Niconiades xanthaphes|[37]|02-SRNP-30345|657[0n]bp
Niconiades xanthaphes|[38]|04-SRNP-14330|642[0n]bp
Niconiades xanthaphes|[39]|08-SRNP-274|645[0n]bp
Niconiades xanthaphes|[40]|08-SRNP-273|645[0n]bp
Niconiades xanthaphes|[41]|08-SRNP-24218|669[0n]bp
Niconiades xanthaphes|[42]|09-SRNP-68536|669[0n]bp
Celaenorrhinus stallingsi|[43]|94-SRNP-569|669[0n]bp
Celaenorrhinus stallingsi|[44]|06-SRNP-47719|669[0n]bp
Celaenorrhinus stallingsi|[45]|04-SRNP-27175|669[0n]bp
Celaenorrhinus stallingsi|[46]|05-SRNP-48589|669[0n]bp
Celaenorrhinus stallingsi|[47]|04-SRNP-45627|669[0n]bp
Celaenorrhinus stallingsi|[48]|04-SRNP-46658|669[0n]bp
Celaenorrhinus stallingsi|[49]|04-SRNP-45628|669[0n]bp
Celaenorrhinus stallingsi|[50]|04-SRNP-45248|669[0n]bp
Celaenorrhinus stallingsi|[51]|04-SRNP-45054|669[0n]bp
Celaenorrhinus stallingsi|[52]|03-SRNP-29104|669[0n]bp
Celaenorrhinus stallingsi|[53]|04-SRNP-45303|669[0n]bp
Celaenorrhinus stallingsi|[54]|03-SRNP-29103|663[0n]bp
Celaenorrhinus stallingsi|[55]|02-SRNP-8079|657[0n]bp
Celaenorrhinus stallingsi|[56]|04-SRNP-45199|630[0n]bp
Celaenorrhinus stallingsi|[57]|04-SRNP-45280|648[1n]bp
Celaenorrhinus stallingsi|[58]|05-SRNP-24342|669[0n]bp
Celaenorrhinus stallingsi|[59]|07-SRNP-35248|669[0n]bp
Celaenorrhinus stallingsi|[60]|08-SRNP-35005|669[0n]bp
Celaenorrhinus stallingsi|[61]|08-SRNP-45153|669[0n]bp
Celaenorrhinus fritzgaertneri|[62]|07-SRNP-58872|669[0n]bp
Celaenorrhinus fritzgaertneri|[63]|07-SRNP-56429|669[0n]bp
Celaenorrhinus fritzgaertneri|[64]|05-SRNP-60702|669[0n]bp
Celaenorrhinus fritzgaertneri|[65]|06-SRNP-19668|669[0n]bp
Celaenorrhinus fritzgaertneri|[66]|04-SRNP-46882|669[0n]bp
Celaenorrhinus fritzgaertneri|[67]|04-SRNP-47171|669[0n]bp
Celaenorrhinus fritzgaertneri|[68]|93-SRNP-6905|669[0n]bp
Celaenorrhinus fritzgaertneri|[69]|04-SRNP-47172|669[2n]bp
Celaenorrhinus fritzgaertneri|[70]|93-SRNP-6145|669[0n]bp
Celaenorrhinus fritzgaertneri|[71]|95-SRNP-9563|399[0n]bp
Celaenorrhinus fritzgaertneri|[72]|05-SRNP-64117|618[0n]bp
Celaenorrhinus fritzgaertneri|[73]|09-SRNP-13046|669[0n]bp
Celaenorrhinus approximatus|[74]|03-SRNP-4049|489[1n]bp
Celaenorrhinus approximatus|[75]|04-SRNP-35283|669[0n]bp
Celaenorrhinus approximatus|[76]|04-SRNP-35231|669[0n]bp
Celaenorrhinus approximatus|[77]|04-SRNP-35236|669[0n]bp
Celaenorrhinus approximatus|[78]|04-SRNP-35375|669[0n]bp
Celaenorrhinus approximatus|[79]|04-SRNP-35276|669[0n]bp
Celaenorrhinus approximatus|[80]|04-SRNP-35243|669[0n]bp
Celaenorrhinus approximatus|[81]|04-SRNP-35230|669[0n]bp
Celaenorrhinus approximatus|[82]|04-SRNP-35247|669[0n]bp
Celaenorrhinus approximatus|[83]|04-SRNP-35245|669[0n]bp
Celaenorrhinus approximatus|[84]|04-SRNP-35242|669[0n]bp
Celaenorrhinus approximatus|[85]|04-SRNP-35234|669[0n]bp
Celaenorrhinus approximatus|[86]|04-SRNP-35239|669[0n]bp
Celaenorrhinus approximatus|[87]|04-SRNP-35233|669[0n]bp
Celaenorrhinus approximatus|[88]|04-SRNP-35235|669[0n]bp
Celaenorrhinus approximatus|[89]|04-SRNP-35282|669[0n]bp
Celaenorrhinus approximatus|[90]|04-SRNP-35280|669[0n]bp
Celaenorrhinus approximatus|[91]|03-SRNP-4053|642[0n]bp
Celaenorrhinus approximatus|[92]|04-SRNP-35281|633[0n]bp
Celaenorrhinus approximatus|[93]|06-SRNP-35037|669[1n]bp
Celaenorrhinus approximatus|[94]|07-SRNP-35584|669[0n]bp
Celaenorrhinus Burns03|[95]|05-SRNP-2519|669[0n]bp
Celaenorrhinus Burns03|[96]|06-SRNP-2390|666[0n]bp
Celaenorrhinus Burns03|[97]|06-SRNP-2415|669[0n]bp
Celaenorrhinus Burns03|[98]|06-SRNP-1475|669[0n]bp
Celaenorrhinus Burns03|[99]|06-SRNP-7150|669[0n]bp

Celaenorrhinus Burns03|[97]|[illegible]
Celaenorrhinus Burns03|[98]|06-SRNP-1475|669[0n]bp
Celaenorrhinus Burns03|[99]|06-SRNP-7150|669[0n]bp
Celaenorrhinus Burns03|[100]|05-SRNP-570|669[2n]bp
Celaenorrhinus Burns03|[101]|05-SRNP-567|669[0n]bp
Celaenorrhinus Burns03|[102]|05-SRNP-751|669[1n]bp
Celaenorrhinus Burns03|[103]|07-SRNP-1399|669[0n]bp
Celaenorrhinus Burns03|[104]|05-SRNP-6010|669[0n]bp
Celaenorrhinus Burns03|[105]|05-SRNP-5661|669[0n]bp
Celaenorrhinus Burns03|[106]|04-SRNP-26450|669[0n]bp
Celaenorrhinus Burns03|[107]|05-SRNP-1346|669[0n]bp
Celaenorrhinus Burns03|[108]|05-SRNP-569|669[0n]bp
Celaenorrhinus Burns03|[109]|04-SRNP-26452|669[0n]bp
Celaenorrhinus Burns03|[110]|05-SRNP-568|669[0n]bp
Celaenorrhinus Burns03|[111]|05-SRNP-6011|645[0n]bp
Celaenorrhinus Burns03|[112]|03-SRNP-5449|645[0n]bp
Celaenorrhinus Burns03|[113]|06-SRNP-679|633[0n]bp
Celaenorrhinus Burns03|[114]|06-SRNP-1485|669[0n]bp
Celaenorrhinus Burns03|[115]|05-SRNP-2378|567[0n]bp
Celaenorrhinus Burns03|[116]|05-SRNP-752|606[0n]bp
Celaenorrhinus Burns03|[117]|05-SRNP-6009|618[0n]bp
Celaenorrhinus Burns03|[118]|05-SRNP-5819|618[0n]bp
Celaenorrhinus Burns03|[119]|07-SRNP-609|663[0n]bp
Celaenorrhinus Burns03|[120]|07-SRNP-1398|669[0n]bp
Celaenorrhinus Burns03|[121]|07-SRNP-1659|669[0n]bp
Celaenorrhinus Burns03|[122]|07-SRNP-1478|669[0n]bp
Celaenorrhinus eligius|[123]|07-SRNP-21870|669[0n]bp
Celaenorrhinus eligius|[124]|05-SRNP-34575|669[0n]bp
Celaenorrhinus eligius|[125]|07-SRNP-23595|669[0n]bp
Celaenorrhinus eligius|[126]|07-SRNP-770|669[0n]bp
Celaenorrhinus eligius|[127]|07-SRNP-580|669[0n]bp
Celaenorrhinus eligius|[128]|06-SRNP-59174|669[0n]bp
Celaenorrhinus eligius|[129]|06-SRNP-1045|669[0n]bp
Celaenorrhinus eligius|[130]|06-SRNP-1044|669[0n]bp
Celaenorrhinus eligius|[131]|05-SRNP-31037|669[0n]bp
Celaenorrhinus eligius|[132]|97-SRNP-11551|669[0n]bp
Celaenorrhinus eligius|[133]|98-SRNP-6383|669[0n]bp
Celaenorrhinus eligius|[134]|04-SRNP-32649|669[0n]bp
Celaenorrhinus eligius|[135]|04-SRNP-45100|627[0n]bp
Celaenorrhinus eligius|[136]|07-SRNP-60093|660[0n]bp
Celaenorrhinus eligius|[137]|08-SRNP-65084|654[1n]bp
Celaenorrhinus eligius|[138]|07-SRNP-24063|636[0n]bp
Celaenorrhinus eligius|[139]|08-SRNP-703|669[0n]bp
Celaenorrhinus eligius|[140]|06-SRNP-2608|669[0n]bp
Celaenorrhinus eligius|[141]|07-SRNP-24053|669[0n]bp
Celaenorrhinus eligius|[142]|06-SRNP-9991|669[0n]bp
Celaenorrhinus eligius|[143]|07-SRNP-55146|669[0n]bp
Celaenorrhinus eligius|[144]|05-SRNP-20010|669[0n]bp
Celaenorrhinus eligius|[145]|06-SRNP-45187|669[0n]bp
Celaenorrhinus eligius|[146]|06-SRNP-45188|669[0n]bp
Celaenorrhinus eligius|[147]|06-SRNP-586|669[0n]bp
Celaenorrhinus eligius|[148]|05-SRNP-48036|669[0n]bp
Celaenorrhinus eligius|[149]|04-SRNP-21218|669[0n]bp
Celaenorrhinus eligius|[150]|06-SRNP-1795|633[0n]bp
Celaenorrhinus eligius|[151]|02-SRNP-4737|657[0n]bp
Celaenorrhinus eligius|[152]|04-SRNP-30412|645[0n]bp
Celaenorrhinus eligius|[153]|04-SRNP-45098|648[0n]bp
Celaenorrhinus eligius|[154]|06-SRNP-59205|618[0n]bp
Celaenorrhinus eligius|[155]|06-SRNP-59204|624[0n]bp
Celaenorrhinus eligius|[156]|04-SRNP-412|615[0n]bp
Celaenorrhinus eligius|[157]|95-SRNP-9367|396[0n]bp
Celaenorrhinus eligius|[158]|06-SRNP-47652|600[0n]bp
Celaenorrhinus eligius|[159]|08-SRNP-55519|639[0n]bp
Celaenorrhinus eligius|[160]|08-SRNP-65085|669[0n]bp
Celaenorrhinus eligius|[161]|08-SRNP-1523|669[0n]bp
Celaenorrhinus eligius|[162]|08-SRNP-702|669[0n]bp
Celaenorrhinus eligius|[163]|08-SRNP-1062|669[0n]bp
Celaenorrhinus eligius|[164]|08-SRNP-24026|669[0n]bp
Celaenorrhinus Burns01|[165]|07-SRNP-322|669[0n]bp
Celaenorrhinus Burns01|[166]|06-SRNP-34867|669[0n]bp
Celaenorrhinus Burns01|[167]|04-SRNP-31134|573[0n]bp
Celaenorrhinus Burns01|[168]|07-SRNP-2116|669[0n]bp
Celaenorrhinus Burns01|[169]|06-SRNP-7072|669[0n]bp
Celaenorrhinus Burns01|[170]|06-SRNP-43466|669[0n]bp
Celaenorrhinus Burns01|[171]|05-SRNP-30928|669[0n]bp
Celaenorrhinus Burns01|[172]|04-SRNP-26417|669[0n]bp
Celaenorrhinus Burns01|[173]|04-SRNP-26244|669[0n]bp
Celaenorrhinus Burns01|[174]|05-SRNP-1753|669[0n]bp
Celaenorrhinus Burns01|[175]|04-SRNP-1521|669[0n]bp
Celaenorrhinus Burns01|[176]|04-SRNP-26070|669[1n]bp
Celaenorrhinus Burns01|[177]|05-SRNP-31094|669[1n]bp
Celaenorrhinus Burns01|[178]|06-SRNP-31079|666[0n]bp
Celaenorrhinus Burns01|[179]|00-SRNP-20678|624[0n]bp
Celaenorrhinus Burns01|[180]|07-SRNP-1746|645[0n]bp
Celaenorrhinus Burns01|[181]|07-SRNP-3335|660[0n]bp
Celaenorrhinus Burns01|[182]|07-SRNP-66148|669[0n]bp
Celaenorrhinus Burns01|[183]|08-SRNP-1552|669[0n]bp
Celaenorrhinus Burns01|[184]|08-SRNP-31104|669[0n]bp
Celaenorrhinus Burns01|[185]|08-SRNP-4324|669[0n]bp
Celaenorrhinus Burns01|[186]|08-SRNP-1270|669[0n]bp
Celaenorrhinus Burns01|[187]|08-SRNP-32133|669[2n]bp
Celaenorrhinus Burns01|[188]|09-SRNP-56693|669[1n]bp
Celaenorrhinus Burns01|[189]|05-SRNP-33241|669[0n]bp
Celaenorrhinus Burns01|[190]|05-SRNP-6400|669[0n]bp
Celaenorrhinus Burns01|[191]|06-SRNP-43399|669[0n]bp
Celaenorrhinus Burns01|[192]|05-SRNP-5759|669[0n]bp
Celaenorrhinus Burns01|[193]|05-SRNP-1850|669[0n]bp
Celaenorrhinus Burns01|[194]|05-SRNP-1852|669[0n]bp
Celaenorrhinus Burns01|[195]|04-SRNP-1512|669[0n]bp
Celaenorrhinus Burns01|[196]|04-SRNP-1743|669[0n]bp
Celaenorrhinus Burns01|[197]|04-SRNP-40721|669[0n]bp
Celaenorrhinus Burns01|[198]|04-SRNP-33215|669[0n]bp
Celaenorrhinus Burns01|[199]|04-SRNP-30415|669[0n]bp
Celaenorrhinus Burns01|[200]|04-SRNP-30592|669[0n]bp

Celaenorrhinus Burns01|[197]|[illegible]|669[0n]bp
Celaenorrhinus Burns01|[198]|04-SRNP-33215|669[0n]bp
Celaenorrhinus Burns01|[199]|04-SRNP-30415|669[0n]bp
Celaenorrhinus Burns01|[200]|04-SRNP-30592|669[0n]bp
Celaenorrhinus Burns01|[201]|05-SRNP-976|669[4n]bp
Celaenorrhinus Burns01|[202]|05-SRNP-30880|669[1n]bp
Celaenorrhinus Burns01|[203]|07-SRNP-3289|660[1n]bp
Celaenorrhinus Burns01|[204]|02-SRNP-27941|660[0n]bp
Celaenorrhinus Burns01|[205]|07-SRNP-2663|645[0n]bp
Celaenorrhinus Burns01|[206]|07-SRNP-2535|645[0n]bp
Celaenorrhinus Burns01|[207]|05-SRNP-5758|642[1n]bp
Celaenorrhinus Burns01|[208]|07-SRNP-3397|660[0n]bp
Celaenorrhinus Burns01|[209]|07-SRNP-3148|660[0n]bp
Celaenorrhinus Burns01|[210]|07-SRNP-2970|666[0n]bp
Celaenorrhinus Burns01|[211]|07-SRNP-3214|660[1n]bp
Celaenorrhinus Burns01|[212]|07-SRNP-30343|669[0n]bp
Celaenorrhinus Burns01|[213]|08-SRNP-2417|669[0n]bp
Celaenorrhinus Burns01|[214]|08-SRNP-1521|669[0n]bp
Celaenorrhinus Burns01|[215]|08-SRNP-32287|669[0n]bp
Celaenorrhinus Burns01|[216]|08-SRNP-72595|669[0n]bp
Celaenorrhinus Burns01|[217]|08-SRNP-72651|669[0n]bp
Celaenorrhinus Burns01|[218]|09-SRNP-65032|669[0n]bp
Celaenorrhinus Burns01|[219]|09-SRNP-57269|669[0n]bp
Celaenorrhinus Burns01|[220]|09-SRNP-69994|669[0n]bp
Celaenorrhinus Burns01|[221]|09-SRNP-68526|669[0n]bp
Polythrix mexicanus|[222]|02-SRNP-13621|669[0n]bp
Polythrix mexicanus|[223]|02-SRNP-13008|669[0n]bp
Polythrix mexicanus|[224]|92-SRNP-5352|669[0n]bp
Polythrix mexicanus|[225]|93-SRNP-4855|609[0n]bp
Polythrix mexicanus|[226]|93-SRNP-3545|618[0n]bp
Polythrix mexicanus|[227]|92-SRNP-5324|669[0n]bp
Polythrix mexicanus|[228]|92-SRNP-5274|669[0n]bp
Polythrix mexicanus|[229]|93-SRNP-4630|669[0n]bp
Polythrix mexicanus|[230]|94-SRNP-5238|669[0n]bp
Polythrix mexicanus|[231]|93-SRNP-4636|669[0n]bp
Polythrix mexicanus|[232]|01-SRNP-12021|615[0n]bp
Polythrix mexicanus|[233]|92-SRNP-4357|663[0n]bp
Polythrix mexicanus|[234]|01-SRNP-12023|576[0n]bp
Polythrix mexicanus|[235]|92-SRNP-5323|579[2n]bp
Polythrix mexicanus|[236]|92-SRNP-4305|609[0n]bp
Polythrix mexicanus|[237]|97-SRNP-3333|669[0n]bp
Polythrix mexicanus|[238]|02-SRNP-32034|669[0n]bp
Polythrix mexicanus|[239]|02-SRNP-10227|669[0n]bp
Polythrix mexicanus|[240]|02-SRNP-13108|669[0n]bp
Polythrix mexicanus|[241]|02-SRNP-13603|669[0n]bp
Polythrix mexicanus|[242]|02-SRNP-13105|669[0n]bp
Polythrix mexicanus|[243]|02-SRNP-13009|669[0n]bp
Polythrix mexicanus|[244]|02-SRNP-13010|669[0n]bp
Polythrix mexicanus|[245]|02-SRNP-13536|669[0n]bp
Polythrix mexicanus|[246]|02-SRNP-32036|669[0n]bp
Polythrix mexicanus|[247]|02-SRNP-13220|669[0n]bp
Polythrix mexicanus|[248]|07-SRNP-12114|669[0n]bp
Polythrix mexicanus|[249]|07-SRNP-14671|669[0n]bp
Polythrix asineDHJ04|[250]|04-SRNP-48896|669[0n]bp
Polythrix asineDHJ04|[251]|06-SRNP-43437|669[0n]bp
Polythrix asineDHJ02|[252]|09-SRNP-156|306[0n]bp
Polythrix asineDHJ01|[253]|00-SRNP-6383|669[0n]bp
Polythrix asineDHJ01|[254]|00-SRNP-6384|606[0n]bp
Polythrix asineDHJ01|[255]|98-SRNP-4268|603[0n]bp
Polythrix asineDHJ01|[256]|05-SRNP-24590|624[0n]bp
Polythrix asineDHJ01|[257]|00-SRNP-6488|615[0n]bp
Polythrix asineDHJ01|[258]|05-SRNP-59384|669[1n]bp
Polythrix asineDHJ01|[259]|99-SRNP-18818|615[0n]bp
Polythrix asineDHJ01|[260]|00-SRNP-7383|669[1n]bp
Polythrix asineDHJ01|[261]|07-SRNP-12113|669[0n]bp
Polythrix asineDHJ01|[262]|06-SRNP-46863|669[0n]bp
Polythrix asineDHJ01|[263]|06-SRNP-46862|669[0n]bp
Polythrix asineDHJ01|[264]|00-SRNP-6220|669[0n]bp
Polythrix asineDHJ01|[265]|97-SRNP-3760|669[0n]bp
Polythrix asineDHJ01|[266]|99-SRNP-6116|669[0n]bp
Polythrix asineDHJ01|[267]|00-SRNP-6415|669[0n]bp
Polythrix asineDHJ01|[268]|00-SRNP-6824|669[0n]bp
Polythrix asineDHJ01|[269]|00-SRNP-6382|669[0n]bp
Polythrix asineDHJ01|[270]|03-SRNP-764|669[0n]bp
Polythrix asineDHJ01|[271]|02-SRNP-10287|669[0n]bp
Polythrix asineDHJ01|[272]|02-SRNP-10302|669[0n]bp
Polythrix asineDHJ01|[273]|02-SRNP-10070|669[0n]bp
Polythrix asineDHJ01|[274]|02-SRNP-17485|669[0n]bp
Polythrix asineDHJ01|[275]|02-SRNP-32959|669[0n]bp
Polythrix asineDHJ01|[276]|01-SRNP-12146|669[0n]bp
Polythrix asineDHJ01|[277]|95-SRNP-9068|669[0n]bp
Polythrix asineDHJ01|[278]|06-SRNP-12613|669[0n]bp
Polythrix asineDHJ01|[279]|04-SRNP-13416|669[0n]bp
Polythrix asineDHJ01|[280]|04-SRNP-16090|669[0n]bp
Polythrix asineDHJ01|[281]|04-SRNP-14315|669[0n]bp
Polythrix asineDHJ01|[282]|98-SRNP-4421|669[0n]bp
Polythrix asineDHJ01|[283]|04-SRNP-21571|669[0n]bp
Polythrix asineDHJ01|[284]|04-SRNP-21230|669[0n]bp
Polythrix asineDHJ01|[285]|04-SRNP-21464|669[0n]bp
Polythrix asineDHJ01|[286]|04-SRNP-21224|669[0n]bp
Polythrix asineDHJ01|[287]|04-SRNP-21232|669[0n]bp
Polythrix asineDHJ01|[288]|00-SRNP-6108|642[0n]bp
Polythrix asineDHJ01|[289]|95-SRNP-818|621[1n]bp
Polythrix asineDHJ01|[290]|92-SRNP-4290|627[0n]bp
Polythrix asineDHJ01|[291]|03-SRNP-765|624[0n]bp
Polythrix asineDHJ01|[292]|06-SRNP-55628|603[0n]bp
Polythrix asineDHJ01|[293]|96-SRNP-177|627[2n]bp
Polythrix asineDHJ01|[294]|00-SRNP-6015|636[0n]bp
Polythrix asineDHJ01|[295]|95-SRNP-817|585[0n]bp
Polythrix asineDHJ01|[296]|98-SRNP-4202|582[0n]bp
Polythrix asineDHJ01|[297]|97-SRNP-3720|600[0n]bp
Polythrix asineDHJ01|[298]|93-SRNP-3748|600[0n]bp
Polythrix asineDHJ01|[299]|93-SRNP-4295|615[0n]bp
Polythrix asineDHJ01|[300]|97-SRNP-3979|606[0n]bp

Polythrix asineDHJ01|[297]|[illegible]
Polythrix asineDHJ01|[298]|93-SRNP-3748|600[0n]bp
Polythrix asineDHJ01|[299]|93-SRNP-4295|615[0n]bp
Polythrix asineDHJ01|[300]|97-SRNP-3979|606[0n]bp
Polythrix asineDHJ01|[301]|96-SRNP-10668|582[0n]bp
Polythrix asineDHJ01|[302]|97-SRNP-4088|582[0n]bp
Polythrix asineDHJ01|[303]|00-SRNP-6491|609[0n]bp
Polythrix asineDHJ01|[304]|91-SRNP-1631|606[0n]bp
Polythrix asineDHJ01|[305]|91-SRNP-784|606[0n]bp
Polythrix asineDHJ01|[306]|00-SRNP-6954|606[0n]bp
Polythrix asineDHJ01|[307]|99-SRNP-2390|606[0n]bp
Polythrix asineDHJ01|[308]|93-SRNP-124|606[0n]bp
Polythrix asineDHJ01|[309]|94-SRNP-567|606[0n]bp
Polythrix asineDHJ01|[310]|93-SRNP-4312|606[0n]bp
Polythrix asineDHJ01|[311]|93-SRNP-4696|606[0n]bp
Polythrix asineDHJ01|[312]|93-SRNP-3899|606[0n]bp
Polythrix asineDHJ01|[313]|93-SRNP-8053|606[0n]bp
Polythrix asineDHJ01|[314]|93-SRNP-4515|606[0n]bp
Polythrix asineDHJ01|[315]|07-SRNP-56773|642[0n]bp
Polythrix asineDHJ01|[316]|08-SRNP-21481|669[0n]bp
Polythrix asineDHJ01|[317]|07-SRNP-12456|669[0n]bp
Polythrix asineDHJ01|[318]|07-SRNP-12455|669[0n]bp
Polythrix asineDHJ01|[319]|02-SRNP-19815|669[0n]bp
Polythrix asineDHJ01|[320]|92-SRNP-4286|606[0n]bp
Polythrix asineDHJ01|[321]|05-SRNP-34180|522[1n]bp
Polythrix asineDHJ01|[322]|04-SRNP-14104|555[0n]bp
Polythrix asineDHJ01|[323]|06-SRNP-12714|669[0n]bp
Polythrix asineDHJ01|[324]|96-SRNP-8726|669[0n]bp
Polythrix asineDHJ01|[325]|00-SRNP-6183|642[0n]bp
Polythrix asineDHJ01|[326]|00-SRNP-6737|642[0n]bp
Polythrix asineDHJ01|[327]|01-SRNP-12057|618[1n]bp
Polythrix asineDHJ01|[328]|01-SRNP-12044|633[0n]bp
Polythrix asineDHJ01|[329]|05-SRNP-5142|636[1n]bp
Polythrix asineDHJ01|[330]|96-SRNP-10264|609[0n]bp
Polythrix asineDHJ01|[331]|01-SRNP-12145|513[1n]bp
Polythrix asineDHJ01|[332]|96-SRNP-8830|606[0n]bp
Polythrix asineDHJ01|[333]|93-SRNP-4232|606[0n]bp
Polythrix asineDHJ01|[334]|08-SRNP-2029|669[0n]bp
Polythrix asineDHJ01|[335]|94-SRNP-7830|669[0n]bp
Polythrix asineDHJ01|[336]|95-SRNP-9644|669[0n]bp
Polythrix asineDHJ01|[337]|93-SRNP-4695|669[0n]bp
Polythrix asineDHJ01|[338]|93-SRNP-3926|669[0n]bp
Polythrix asineDHJ01|[339]|00-SRNP-6613|669[0n]bp
Polythrix asineDHJ01|[340]|00-SRNP-6394|669[0n]bp
Polythrix asineDHJ01|[341]|01-SRNP-12359|669[0n]bp
Polythrix asineDHJ01|[342]|02-SRNP-10304|669[0n]bp
Polythrix asineDHJ01|[343]|03-SRNP-29556|669[0n]bp
Polythrix asineDHJ01|[344]|03-SRNP-777|669[0n]bp
Polythrix asineDHJ01|[345]|00-SRNP-6487|669[0n]bp
Polythrix asineDHJ01|[346]|00-SRNP-6367|669[0n]bp
Polythrix asineDHJ01|[347]|00-SRNP-6497|669[0n]bp
Polythrix asineDHJ01|[348]|00-SRNP-6494|669[0n]bp
Polythrix asineDHJ01|[349]|00-SRNP-6395|669[0n]bp
Polythrix asineDHJ01|[350]|01-SRNP-18701|669[0n]bp
Polythrix asineDHJ01|[351]|00-SRNP-6700|669[0n]bp
Polythrix asineDHJ01|[352]|07-SRNP-20846|669[0n]bp
Polythrix asineDHJ01|[353]|07-SRNP-57195|669[0n]bp
Polythrix asineDHJ01|[354]|08-SRNP-56049|669[0n]bp
Polythrix asineDHJ01|[355]|08-SRNP-24005|669[0n]bp
Polythrix asineDHJ02|[356]|00-SRNP-6701|600[0n]bp
Polythrix asineDHJ02|[357]|01-SRNP-17366|669[0n]bp
Polythrix asineDHJ02|[358]|00-SRNP-6215|669[0n]bp
Polythrix asineDHJ02|[359]|02-SRNP-13219|669[0n]bp
Polythrix asineDHJ02|[360]|02-SRNP-10303|669[0n]bp
Polythrix asineDHJ02|[361]|98-SRNP-4205|621[0n]bp
Polythrix asineDHJ02|[362]|93-SRNP-3543|618[0n]bp
Polythrix asineDHJ02|[363]|94-SRNP-5275|606[0n]bp
Polythrix asineDHJ02|[364]|92-SRNP-204|606[0n]bp
Polythrix asineDHJ02|[365]|96-SRNP-957|606[0n]bp
Polythrix asineDHJ02|[366]|08-SRNP-2064|669[0n]bp
Polythrix asineDHJ02|[367]|00-SRNP-6572|669[0n]bp
Polythrix asineDHJ02|[368]|00-SRNP-6495|669[0n]bp
Polythrix asineDHJ02|[369]|07-SRNP-55549|669[0n]bp
Polythrix asineDHJ02|[370]|99-SRNP-6082|669[0n]bp
Polythrix asineDHJ02|[371]|99-SRNP-6074|669[0n]bp
Polythrix asineDHJ02|[372]|00-SRNP-6949|669[0n]bp
Polythrix asineDHJ02|[373]|00-SRNP-7137|669[0n]bp
Polythrix asineDHJ02|[374]|03-SRNP-18566|669[0n]bp
Polythrix asineDHJ02|[375]|03-SRNP-14435|669[0n]bp
Polythrix asineDHJ02|[376]|02-SRNP-32223|669[0n]bp
Polythrix asineDHJ02|[377]|00-SRNP-3211|669[0n]bp
Polythrix asineDHJ02|[378]|94-SRNP-710|669[0n]bp
Polythrix asineDHJ02|[379]|02-SRNP-10034|591[0n]bp
Polythrix asineDHJ02|[380]|97-SRNP-3363|582[0n]bp
Polythrix asineDHJ02|[381]|00-SRNP-6388|669[0n]bp
Polythrix asineDHJ02|[382]|00-SRNP-6950|606[0n]bp
Polythrix asineDHJ02|[383]|05-SRNP-61346|633[0n]bp
Polythrix asineDHJ02|[384]|94-SRNP-711|585[0n]bp
Polythrix asineDHJ02|[385]|05-SRNP-59292|633[2n]bp
Polythrix asineDHJ02|[386]|93-SRNP-3930|612[0n]bp
Polythrix asineDHJ02|[387]|93-SRNP-3398|606[0n]bp
Polythrix asineDHJ02|[388]|92-SRNP-5429|609[0n]bp
Polythrix asineDHJ02|[389]|91-SRNP-167|609[0n]bp
Polythrix asineDHJ02|[390]|92-SRNP-4524|606[0n]bp
Polythrix asineDHJ02|[391]|94-SRNP-699|618[1n]bp
Polythrix asineDHJ02|[392]|08-SRNP-56062|669[0n]bp
Polythrix asineDHJ02|[393]|08-SRNP-14903|669[0n]bp
Polythrix asineDHJ02|[394]|02-SRNP-10068|669[0n]bp
Polythrix asineDHJ02|[395]|01-SRNP-12056|669[0n]bp
Polythrix asineDHJ02|[396]|04-SRNP-21235|669[0n]bp
Polythrix asineDHJ02|[397]|93-SRNP-4231|606[0n]bp
Polythrix asineDHJ02|[398]|07-SRNP-55640|642[0n]bp
Polythrix asineDHJ02|[399]|93-SRNP-4239|606[0n]bp
Polythrix asineDHJ02|[400]|99-SRNP-6119|669[0n]bp

Polythrix asineDHJ02|[397]|9[illegible]-SRNP-[illegible]|[illegible][0n]bp
Polythrix asineDHJ02|[398]|07-SRNP-55640|642[0n]bp
Polythrix asineDHJ02|[399]|93-SRNP-4239|606[0n]bp
Polythrix asineDHJ02|[400]|99-SRNP-6119|669[0n]bp
Polythrix asineDHJ02|[401]|00-SRNP-7038|669[0n]bp
Polythrix asineDHJ02|[402]|93-SRNP-61|567[0n]bp
Polythrix asineDHJ02|[403]|92-SRNP-5030|609[0n]bp
Polythrix asineDHJ02|[404]|93-SRNP-3635|621[1n]bp
Polythrix asineDHJ02|[405]|97-SRNP-3311|606[0n]bp
Polythrix asineDHJ02|[406]|08-SRNP-21270|669[0n]bp
Polythrix asineDHJ02|[407]|90-SRNP-2362|606[0n]bp
Polythrix asineDHJ02|[408]|03-SRNP-151|606[0n]bp
Polythrix asineDHJ02|[409]|01-SRNP-17146|606[0n]bp
Polythrix asineDHJ02|[410]|00-SRNP-7129|669[0n]bp
Polythrix asineDHJ02|[411]|00-SRNP-2525|633[0n]bp
Polythrix asineDHJ02|[412]|97-SRNP-5334|594[0n]bp
Polythrix asineDHJ02|[413]|91-SRNP-2264|606[0n]bp
Polythrix asineDHJ02|[414]|96-SRNP-10685|606[0n]bp
Polythrix asineDHJ02|[415]|07-SRNP-56772|669[0n]bp
Polythrix asineDHJ02|[416]|00-SRNP-20213|669[1n]bp
Polythrix asineDHJ02|[417]|03-SRNP-949|669[0n]bp
Polythrix asineDHJ02|[418]|01-SRNP-18761|669[0n]bp
Polythrix asineDHJ02|[419]|03-SRNP-25832|669[0n]bp
Polythrix asineDHJ02|[420]|99-SRNP-6164|558[1n]bp
Polythrix asineDHJ02|[421]|01-SRNP-12169|615[0n]bp
Polythrix asineDHJ02|[422]|00-SRNP-7100|579[0n]bp
Polythrix asineDHJ02|[423]|00-SRNP-2164|579[0n]bp
Polythrix asineDHJ02|[424]|97-SRNP-10070|618[0n]bp
Polythrix asineDHJ02|[425]|07-SRNP-55609|669[0n]bp
Polythrix asineDHJ02|[426]|06-SRNP-47913|669[0n]bp
Polythrix asineDHJ02|[427]|00-SRNP-2609|669[0n]bp
Polythrix asineDHJ02|[428]|00-SRNP-17652|669[0n]bp
Polythrix asineDHJ02|[429]|00-SRNP-6951|669[0n]bp
Polythrix asineDHJ02|[430]|00-SRNP-6013|669[0n]bp
Polythrix asineDHJ02|[431]|00-SRNP-6014|669[0n]bp
Polythrix asineDHJ02|[432]|03-SRNP-678|669[0n]bp
Polythrix asineDHJ02|[433]|03-SRNP-14568|669[0n]bp
Polythrix asineDHJ02|[434]|03-SRNP-155|669[0n]bp
Polythrix asineDHJ02|[435]|02-SRNP-32221|669[0n]bp
Polythrix asineDHJ02|[436]|02-SRNP-5741|669[0n]bp
Polythrix asineDHJ02|[437]|01-SRNP-16459|669[0n]bp
Polythrix asineDHJ02|[438]|02-SRNP-13312|669[0n]bp
Polythrix asineDHJ02|[439]|06-SRNP-21342|669[0n]bp
Polythrix asineDHJ02|[440]|06-SRNP-12614|669[0n]bp
Polythrix asineDHJ02|[441]|05-SRNP-64404|669[0n]bp
Polythrix asineDHJ02|[442]|04-SRNP-14469|669[0n]bp
Polythrix asineDHJ02|[443]|04-SRNP-45139|669[0n]bp
Polythrix asineDHJ02|[444]|04-SRNP-2256|669[0n]bp
Polythrix asineDHJ02|[445]|00-SRNP-6483|612[0n]bp
Polythrix asineDHJ02|[446]|05-SRNP-21368|669[4n]bp
Polythrix asineDHJ02|[447]|93-SRNP-4178|627[2n]bp
Polythrix asineDHJ02|[448]|92-SRNP-5058|561[0n]bp
Polythrix asineDHJ02|[449]|05-SRNP-4485|669[6n]bp
Polythrix asineDHJ02|[450]|93-SRNP-4516|606[0n]bp
Polythrix asineDHJ02|[451]|93-SRNP-4921|642[0n]bp
Polythrix asineDHJ02|[452]|06-SRNP-46210|627[0n]bp
Polythrix asineDHJ02|[453]|94-SRNP-6175|618[0n]bp
Polythrix asineDHJ02|[454]|01-SRNP-12150|645[0n]bp
Polythrix asineDHJ02|[455]|94-SRNP-7825|603[0n]bp
Polythrix asineDHJ02|[456]|90-SRNP-1709|588[1n]bp
Polythrix asineDHJ02|[457]|92-SRNP-4667|600[0n]bp
Polythrix asineDHJ02|[458]|92-SRNP-4160|615[1n]bp
Polythrix asineDHJ02|[459]|99-SRNP-6176|615[1n]bp
Polythrix asineDHJ02|[460]|03-SRNP-1811|630[0n]bp
Polythrix asineDHJ02|[461]|93-SRNP-4316|606[0n]bp
Polythrix asineDHJ02|[462]|97-SRNP-5248|606[0n]bp
Polythrix asineDHJ02|[463]|98-SRNP-4204|591[0n]bp
Polythrix asineDHJ02|[464]|91-SRNP-2273|615[0n]bp
Polythrix asineDHJ02|[465]|92-SRNP-4981|609[0n]bp
Polythrix asineDHJ02|[466]|93-SRNP-22|381[0n]bp
Polythrix asineDHJ02|[467]|98-SRNP-4007|609[0n]bp
Polythrix asineDHJ02|[468]|07-SRNP-12116|642[0n]bp
Polythrix asineDHJ02|[469]|08-SRNP-21704|669[0n]bp
Polythrix asineDHJ02|[470]|08-SRNP-4981|669[0n]bp
Polythrix asineDHJ02|[471]|09-SRNP-14247|669[0n]bp
Polythrix caunus|[472]|06-SRNP-48047|669[1n]bp
Polythrix caunus|[473]|05-SRNP-41335|669[3n]bp
Polythrix caunus|[474]|07-SRNP-45093|669[0n]bp
Polythrix caunus|[475]|07-SRNP-45094|669[0n]bp
Polythrix caunus|[476]|06-SRNP-46961|669[0n]bp
Polythrix caunus|[477]|97-SRNP-411|669[0n]bp
Polythrix caunus|[478]|00-SRNP-3976|669[0n]bp
Polythrix caunus|[479]|01-SRNP-9329|669[0n]bp
Polythrix caunus|[480]|01-SRNP-11831|669[0n]bp
Polythrix caunus|[481]|01-SRNP-9037|669[0n]bp
Polythrix caunus|[482]|02-SRNP-6762|669[0n]bp
Polythrix caunus|[483]|04-SRNP-45147|669[0n]bp
Polythrix caunus|[484]|05-SRNP-61208|669[0n]bp
Polythrix caunus|[485]|04-SRNP-49813|669[0n]bp
Polythrix caunus|[486]|04-SRNP-47482|669[0n]bp
Polythrix caunus|[487]|04-SRNP-13678|669[0n]bp
Polythrix caunus|[488]|04-SRNP-56145|669[0n]bp
Polythrix caunus|[489]|04-SRNP-49793|669[0n]bp
Polythrix caunus|[490]|04-SRNP-48326|669[0n]bp
Polythrix caunus|[491]|06-SRNP-47938|669[1n]bp
Polythrix caunus|[492]|05-SRNP-40491|666[3n]bp
Polythrix caunus|[493]|03-SRNP-1670|642[0n]bp
Polythrix caunus|[494]|97-SRNP-5432|606[0n]bp
Polythrix caunus|[495]|00-SRNP-18463|606[0n]bp
Polythrix caunus|[496]|01-SRNP-9142|606[0n]bp
Polythrix caunus|[497]|01-SRNP-9036|606[0n]bp
Polythrix caunus|[498]|03-SRNP-1437|624[0n]bp
Polythrix caunus|[499]|02-SRNP-4214|606[0n]bp
Polythrix caunus|[500]|96-SRNP-230|582[0n]bp

[illegible]
Polythrix caunus|[498]|03-SRNP-1437|624[0n]bp
Polythrix caunus|[499]|02-SRNP-4214|606[0n]bp
Polythrix caunus|[500]|96-SRNP-230|582[0n]bp
Polythrix caunus|[501]|07-SRNP-46448|627[1n]bp
Polythrix caunus|[502]|07-SRNP-47110|657[2n]bp
Polythrix caunus|[503]|08-SRNP-31055|669[0n]bp
Polythrix caunus|[504]|08-SRNP-2598|669[0n]bp
Polythrix caunus|[505]|08-SRNP-2600|669[0n]bp
Polythrix caunus|[506]|08-SRNP-2433|669[0n]bp
Polythrix caunus|[507]|08-SRNP-2599|669[0n]bp
Chrysoplectrum Burns02|[508]|03-SRNP-20727|657[0n]bp
Polythrix octomaculata|[509]|01-SRNP-18777|639[0n]bp
Polythrix octomaculata|[510]|04-SRNP-16187|669[0n]bp
Polythrix octomaculata|[511]|06-SRNP-55510|669[0n]bp
Polythrix octomaculata|[512]|06-SRNP-58092|669[0n]bp
Polythrix octomaculata|[513]|08-SRNP-2053|669[0n]bp
Polythrix octomaculata|[514]|01-SRNP-12143|570[0n]bp
Polythrix octomaculata|[515]|06-SRNP-55601|669[0n]bp
Polythrix octomaculata|[516]|04-SRNP-16188|669[0n]bp
Polythrix octomaculata|[517]|06-SRNP-55352|669[2n]bp
Polythrix octomaculata|[518]|06-SRNP-55424|651[0n]bp
Polythrix octomaculata|[519]|06-SRNP-55112|489[0n]bp
Polythrix octomaculata|[520]|09-SRNP-55230|660[0n]bp
Polythrix auginus|[521]|08-SRNP-5805|597[0n]bp
Polythrix auginus|[522]|99-SRNP-2513|606[0n]bp
Polythrix auginus|[523]|01-SRNP-4476|606[0n]bp
Polythrix auginus|[524]|02-SRNP-5395|588[0n]bp
Polythrix auginus|[525]|05-SRNP-40942|609[0n]bp
Polythrix auginus|[526]|05-SRNP-40943|609[0n]bp
Polythrix auginus|[527]|07-SRNP-23809|606[0n]bp
Polythrix auginus|[528]|07-SRNP-23808|609[0n]bp
Polythrix auginus|[529]|02-SRNP-7476|594[5n]bp
Polythrix auginus|[530]|00-SRNP-21098|384[0n]bp
Polythrix auginus|[531]|08-SRNP-2432|393[0n]bp
Polythrix auginus|[532]|08-SRNP-32652|408[0n]bp
Polythrix auginus|[533]|09-SRNP-71706|669[0n]bp
Chrysoplectrum pervivax|[534]|08-SRNP-55673|669[0n]bp
Chrysoplectrum pervivax|[535]|07-SRNP-55670|669[0n]bp
Chrysoplectrum pervivax|[536]|06-SRNP-55724|669[0n]bp
Chrysoplectrum pervivax|[537]|06-SRNP-55725|669[0n]bp
Chrysoplectrum pervivax|[538]|05-SRNP-55019|669[0n]bp
Chrysoplectrum pervivax|[539]|05-SRNP-55012|669[0n]bp
Chrysoplectrum pervivax|[540]|03-SRNP-599|657[0n]bp
Chrysoplectrum pervivax|[541]|03-SRNP-27771|645[0n]bp
Chrysoplectrum pervivax|[542]|05-SRNP-12099|636[0n]bp
Chrysoplectrum pervivax|[543]|06-SRNP-55633|570[0n]bp
Chrysoplectrum pervivax|[544]|03-SRNP-285|651[0n]bp
Chrysoplectrum pervivax|[545]|03-SRNP-27214|663[0n]bp
Chrysoplectrum pervivax|[546]|06-SRNP-55678|666[0n]bp
Chrysoplectrum pervivax|[547]|08-SRNP-55510|669[0n]bp
Chrysoplectrum pervivax|[548]|08-SRNP-55708|669[0n]bp
Chrysoplectrum pervivax|[549]|08-SRNP-55674|669[0n]bp
Chrysoplectrum Burns01|[550]|05-SRNP-20359|669[0n]bp
Chrysoplectrum Burns01|[551]|03-SRNP-21246|657[0n]bp
Chrysoplectrum Burns01|[552]|08-SRNP-41220|669[0n]bp
Chrysoplectrum Burns01|[553]|08-SRNP-71264|669[0n]bp
Chrysoplectrum Burns01|[554]|08-SRNP-71263|669[0n]bp
Chrysoplectrum Burns01|[555]|07-SRNP-65253|645[0n]bp
Chrysoplectrum Burns01|[556]|03-SRNP-5985|624[0n]bp
Chrysoplectrum Burns01|[557]|07-SRNP-65263|666[1n]bp
Chrysoplectrum Burns01|[558]|04-SRNP-33618|669[0n]bp
Chrysoplectrum Burns01|[559]|04-SRNP-41884|669[0n]bp
Chrysoplectrum Burns01|[560]|04-SRNP-60797|669[0n]bp
Chrysoplectrum Burns01|[561]|04-SRNP-60793|669[0n]bp
Chrysoplectrum Burns01|[562]|04-SRNP-60305|669[0n]bp
Chrysoplectrum Burns01|[563]|03-SRNP-10713|657[0n]bp
Chrysoplectrum Burns01|[564]|03-SRNP-30510|654[0n]bp
Chrysoplectrum Burns01|[565]|07-SRNP-41409|654[0n]bp
Chrysoplectrum Burns01|[566]|07-SRNP-21008|669[0n]bp
Chrysoplectrum Burns01|[567]|07-SRNP-65264|669[0n]bp
Chrysoplectrum Burns01|[568]|07-SRNP-65771|669[0n]bp
Chrysoplectrum Burns01|[569]|07-SRNP-65755|669[0n]bp
Chrysoplectrum Burns01|[570]|07-SRNP-65394|669[0n]bp
Chrysoplectrum Burns01|[571]|08-SRNP-71635|669[0n]bp
Chrysoplectrum Burns01|[572]|08-SRNP-71122|669[0n]bp
Chrysoplectrum Burns01|[573]|08-SRNP-71266|669[0n]bp
Chrysoplectrum Burns01|[574]|08-SRNP-71123|669[0n]bp
Chrysoplectrum Burns01|[575]|09-SRNP-69180|669[0n]bp
Chrysoplectrum Burns01|[576]|09-SRNP-22283|669[0n]bp
Chrysoplectrum Burns01|[577]|09-SRNP-41395|669[0n]bp
Paches loxus|[578]|02-SRNP-5725|633[0n]bp
Paches loxus|[579]|02-SRNP-5985|657[0n]bp
Paches loxus|[580]|03-SRNP-30995|639[0n]bp
Paches loxus|[581]|06-SRNP-55156|669[0n]bp
Paches loxus|[582]|02-SRNP-5727|669[0n]bp
Paches loxus|[583]|02-SRNP-5726|669[0n]bp
Paches loxus|[584]|03-SRNP-30840|669[0n]bp
Paches loxus|[585]|03-SRNP-30960|669[0n]bp
Paches loxus|[586]|02-SRNP-5742|669[0n]bp
Paches loxus|[587]|02-SRNP-5517|669[0n]bp
Paches loxus|[588]|02-SRNP-5724|669[0n]bp
Paches loxus|[589]|07-SRNP-60833|669[0n]bp
Paches loxus|[590]|07-SRNP-60825|639[0n]bp
Paches loxus|[591]|04-SRNP-15261|669[0n]bp
Paches loxus|[592]|02-SRNP-14194|669[0n]bp
Paches loxus|[593]|02-SRNP-5743|669[0n]bp
Paches loxus|[594]|07-SRNP-60964|669[0n]bp
Phanus marshalliiDHJ01|[595]|03-SRNP-10532|669[0n]bp
Phanus marshalliiDHJ01|[596]|03-SRNP-5739|609[0n]bp
Phanus marshalliiDHJ01|[597]|03-SRNP-5850|609[0n]bp
Phanus marshalliiDHJ01|[598]|03-SRNP-16237|660[0n]bp
Phanus marshalliiDHJ01|[599]|03-SRNP-5857|669[0n]bp
Phanus marshalliiDHJ02|[600]|08-SRNP-2311|669[0n]bp

Phanus marshalliiDHJ01|[597]|03-SRNP-[illegible]|669[0n]bp
Phanus marshalliiDHJ01|[598]|03-SRNP-16237|660[0n]bp
Phanus marshalliiDHJ01|[599]|03-SRNP-5857|669[0n]bp
Phanus marshalliiDHJ02|[600]|08-SRNP-2311|669[0n]bp
Phanus marshalliiDHJ02|[601]|08-SRNP-21884|669[0n]bp
Phanus marshalliiDHJ02|[602]|07-SRNP-21440|669[0n]bp
Phanus marshalliiDHJ02|[603]|02-SRNP-14543|669[0n]bp
Phanus marshalliiDHJ02|[604]|02-SRNP-2186|669[0n]bp
Phanus marshalliiDHJ02|[605]|03-SRNP-16236|642[1n]bp
Phanus marshalliiDHJ02|[606]|03-SRNP-16234|669[0n]bp
Phanus marshalliiDHJ02|[607]|05-SRNP-2028|669[0n]bp
Phanus marshalliiDHJ02|[608]|02-SRNP-2185|639[0n]bp
Phanus marshalliiDHJ02|[609]|01-SRNP-14963|660[0n]bp
Phanus marshalliiDHJ02|[610]|08-SRNP-21663|615[0n]bp
Phanus obscurior|[611]|02-SRNP-1685|669[0n]bp
Phanus obscurior|[612]|08-SRNP-1691|669[0n]bp
Phanus obscurior|[613]|08-SRNP-1692|669[1n]bp
Phanus obscurior|[614]|03-SRNP-6083|669[0n]bp
Phanus obscurior|[615]|04-SRNP-60040|669[0n]bp
Phanus obscurior|[616]|06-SRNP-22131|669[0n]bp
Phanus obscurior|[617]|06-SRNP-22126|669[0n]bp
Phanus obscurior|[618]|06-SRNP-22146|669[0n]bp
Phanus obscurior|[619]|02-SRNP-3622|669[0n]bp
Phanus obscurior|[620]|02-SRNP-3270|669[0n]bp
Phanus obscurior|[621]|02-SRNP-17173|669[0n]bp
Phanus obscurior|[622]|03-SRNP-5866|669[0n]bp
Phanus obscurior|[623]|05-SRNP-1856|669[0n]bp
Phanus obscurior|[624]|03-SRNP-5851|624[1n]bp
Phanus obscurior|[625]|06-SRNP-22124|630[0n]bp
Phanus obscurior|[626]|06-SRNP-22129|642[0n]bp
Phanus obscurior|[627]|08-SRNP-1581|669[0n]bp
Phanus obscurior|[628]|08-SRNP-1580|669[0n]bp
Phanus obscurior|[629]|08-SRNP-4668|669[0n]bp
Phanus obscurior|[630]|08-SRNP-1112|669[0n]bp
Phanus obscurior|[631]|00-SRNP-870|645[0n]bp
Phanus obscurior|[632]|02-SRNP-2466|645[0n]bp
Phanus obscurior|[633]|06-SRNP-22134|669[1n]bp
Phanus obscurior|[634]|07-SRNP-2619|663[0n]bp
Phanus obscurior|[635]|06-SRNP-22127|666[0n]bp
Phanus obscurior|[636]|06-SRNP-3813|669[0n]bp
Phanus obscurior|[637]|06-SRNP-3624|669[0n]bp
Phanus obscurior|[638]|04-SRNP-60913|669[0n]bp
Phanus obscurior|[639]|06-SRNP-34130|660[0n]bp
Phanus obscurior|[640]|02-SRNP-2470|669[0n]bp
Phanus obscurior|[641]|08-SRNP-4659|669[0n]bp
Phanus vitreusDHJ02|[642]|05-SRNP-30876|669[0n]bp
Phanus vitreusDHJ02|[643]|07-SRNP-3510|669[0n]bp
Phanus vitreusDHJ02|[644]|07-SRNP-4681|669[0n]bp
Phanus vitreusDHJ02|[645]|07-SRNP-4607|627[0n]bp
Phanus vitreusDHJ02|[646]|07-SRNP-66113|669[0n]bp
Phanus vitreusDHJ02|[647]|07-SRNP-4605|669[0n]bp
Phanus vitreusDHJ02|[648]|07-SRNP-4110|669[0n]bp
Phanus vitreusDHJ02|[649]|06-SRNP-30361|669[0n]bp
Phanus vitreusDHJ02|[650]|05-SRNP-4944|669[0n]bp
Phanus vitreusDHJ02|[651]|08-SRNP-13|669[1n]bp
Phanus vitreusDHJ02|[652]|07-SRNP-42826|666[0n]bp
Phanus vitreusDHJ02|[653]|07-SRNP-4604|621[1n]bp
Phanus vitreusDHJ02|[654]|08-SRNP-40045|648[1n]bp
Phanus vitreusDHJ02|[655]|07-SRNP-24196|633[0n]bp
Phanus vitreusDHJ02|[656]|07-SRNP-24814|648[0n]bp
Phanus vitreusDHJ02|[657]|07-SRNP-4517|654[1n]bp
Phanus vitreusDHJ02|[658]|08-SRNP-41100|669[0n]bp
Phanus vitreusDHJ02|[659]|08-SRNP-40547|669[0n]bp
Phanus vitreusDHJ03|[660]|98-SRNP-4474|669[0n]bp
Phanus vitreusDHJ01|[661]|03-SRNP-3212|669[0n]bp
Phanus vitreusDHJ01|[662]|98-SRNP-4291|609[0n]bp
Phanus vitreusDHJ01|[663]|98-SRNP-4284|612[0n]bp
Phanus vitreusDHJ01|[664]|98-SRNP-4554|669[0n]bp
Phanus vitreusDHJ01|[665]|00-SRNP-2518|669[0n]bp
Phanus vitreusDHJ01|[666]|08-SRNP-5259|639[0n]bp
Phanus vitreusDHJ01|[667]|97-SRNP-647|639[0n]bp
Phanus vitreusDHJ01|[668]|98-SRNP-4552|669[0n]bp
Phanus vitreusDHJ01|[669]|98-SRNP-4289|669[0n]bp
Phanus vitreusDHJ01|[670]|98-SRNP-4556|669[1n]bp
Phanus vitreusDHJ01|[671]|98-SRNP-4466|606[0n]bp
Phanus vitreusDHJ01|[672]|97-SRNP-554|606[0n]bp
Phanus vitreusDHJ01|[673]|98-SRNP-4507|618[0n]bp
Phanus vitreusDHJ01|[674]|08-SRNP-893|669[0n]bp
Phanus vitreusDHJ01|[675]|08-SRNP-716|669[0n]bp
Phanus vitreusDHJ01|[676]|09-SRNP-55618|669[0n]bp
Phanus vitreusDHJ01|[677]|97-SRNP-648|606[0n]bp
Phanus vitreusDHJ01|[678]|02-SRNP-1762|645[0n]bp
Phanus vitreusDHJ01|[679]|00-SRNP-2561|642[0n]bp
Phanus vitreusDHJ01|[680]|97-SRNP-641|630[2n]bp
Phanus vitreusDHJ01|[681]|98-SRNP-4287|630[2n]bp
Phanus vitreusDHJ01|[682]|98-SRNP-4510|624[1n]bp
Phanus vitreusDHJ01|[683]|00-SRNP-2520|669[0n]bp
Phanus vitreusDHJ01|[684]|98-SRNP-4286|669[0n]bp
Phanus vitreusDHJ01|[685]|98-SRNP-4518|669[0n]bp
Phanus vitreusDHJ01|[686]|00-SRNP-2560|669[0n]bp
Phanus vitreusDHJ01|[687]|03-SRNP-3081|669[0n]bp
Phanus vitreusDHJ01|[688]|01-SRNP-9201|669[0n]bp
Phanus vitreusDHJ01|[689]|00-SRNP-2048|669[0n]bp
Phanus vitreusDHJ01|[690]|00-SRNP-2046|669[0n]bp
Phanus vitreusDHJ01|[691]|00-SRNP-2556|669[0n]bp
Phanus vitreusDHJ01|[692]|97-SRNP-640|669[0n]bp
Phanus vitreusDHJ01|[693]|00-SRNP-2517|669[0n]bp
Phanus vitreusDHJ01|[694]|98-SRNP-4553|669[0n]bp
Phanus vitreusDHJ01|[695]|06-SRNP-45146|669[0n]bp
Phanus vitreusDHJ01|[696]|98-SRNP-4288|657[0n]bp
Phanus vitreusDHJ01|[697]|98-SRNP-4467|657[0n]bp
Phanus vitreusDHJ01|[698]|98-SRNP-4282|618[1n]bp
Phanus vitreusDHJ01|[699]|97-SRNP-559|609[0n]bp
Phanus vitreusDHJ01|[700]|97-SRNP-562|606[0n]bp

[illegible]
Phanus vitreusDHJ01|[698]|98-SRNP-4282|618[1n]bp
Phanus vitreusDHJ01|[699]|97-SRNP-559|609[0n]bp
Phanus vitreusDHJ01|[700]|97-SRNP-562|606[0n]bp
Phanus vitreusDHJ01|[701]|97-SRNP-643|606[0n]bp
Phanus vitreusDHJ01|[702]|00-SRNP-2562|606[0n]bp
Phanus vitreusDHJ01|[703]|97-SRNP-526|606[0n]bp
Phanus vitreusDHJ01|[704]|97-SRNP-548|606[0n]bp
Phanus vitreusDHJ01|[705]|98-SRNP-4512.01|606[0n]bp
Phanus vitreusDHJ01|[706]|97-SRNP-642|606[0n]bp
Phanus vitreusDHJ01|[707]|98-SRNP-4473|606[0n]bp
Phanus vitreusDHJ01|[708]|97-SRNP-645|618[1n]bp
Phanus vitreusDHJ01|[709]|97-SRNP-646|633[0n]bp
Phanus vitreusDHJ01|[710]|97-SRNP-555|606[0n]bp
Phanus vitreusDHJ01|[711]|00-SRNP-2550|669[0n]bp
Phanus vitreusDHJ01|[712]|08-SRNP-713|669[0n]bp
Phanus vitreusDHJ01|[713]|08-SRNP-938|669[0n]bp
Phanus vitreusDHJ01|[714]|08-SRNP-717|669[0n]bp
Phanus vitreusDHJ01|[715]|08-SRNP-939|669[0n]bp
Phanus vitreusDHJ01|[716]|08-SRNP-824|669[0n]bp
Phanus vitreusDHJ01|[717]|08-SRNP-715|669[0n]bp
Phanus vitreusDHJ01|[718]|08-SRNP-825|669[0n]bp
Phanus vitreusDHJ01|[719]|08-SRNP-5260|669[0n]bp
Phanus vitreusDHJ01|[720]|09-SRNP-1728|669[0n]bp
Phanus vitreusDHJ01|[721]|00-SRNP-2047|669[0n]bp
Phanus vitreusDHJ01|[722]|09-SRNP-944|669[0n]bp
Hyalothyrus neleus|[723]|99-SRNP-2782|480[0n]bp
Hyalothyrus neleus|[724]|03-SRNP-15448|669[0n]bp
Hyalothyrus neleus|[725]|03-SRNP-10401|669[0n]bp
Hyalothyrus neleus|[726]|02-SRNP-5338|669[0n]bp
Hyalothyrus neleus|[727]|02-SRNP-15446|669[0n]bp
Hyalothyrus neleus|[728]|02-SRNP-4193|669[0n]bp
Hyalothyrus neleus|[729]|01-SRNP-9634|669[0n]bp
Hyalothyrus neleus|[730]|00-SRNP-2796|669[0n]bp
Hyalothyrus neleus|[731]|01-SRNP-24019|669[0n]bp
Hyalothyrus neleus|[732]|01-SRNP-11940|669[0n]bp
Hyalothyrus neleus|[733]|01-SRNP-9762|669[0n]bp
Hyalothyrus neleus|[734]|01-SRNP-9010|669[0n]bp
Hyalothyrus neleus|[735]|04-SRNP-14956|669[0n]bp
Hyalothyrus neleus|[736]|04-SRNP-23854|669[0n]bp
Hyalothyrus neleus|[737]|02-SRNP-5623|651[0n]bp
Hyalothyrus neleus|[738]|02-SRNP-2903|651[0n]bp
Hyalothyrus neleus|[739]|07-SRNP-1289|669[0n]bp
Hyalothyrus neleus|[740]|05-SRNP-23937|669[0n]bp
Hyalothyrus neleus|[741]|05-SRNP-24060|669[0n]bp
Hyalothyrus neleus|[742]|05-SRNP-33739|669[0n]bp
Hyalothyrus neleus|[743]|07-SRNP-1874|669[0n]bp
Hyalothyrus neleus|[744]|08-SRNP-20639|669[0n]bp
Entheus Burns01|[745]|05-SRNP-31825|669[0n]bp
Entheus Burns01|[746]|05-SRNP-31804|669[0n]bp
Entheus Burns01|[747]|03-SRNP-3912|669[0n]bp
Entheus Burns01|[748]|05-SRNP-31679|687[0n]bp
Entheus Burns01|[749]|05-SRNP-31813|687[0n]bp
Entheus Burns01|[750]|05-SRNP-31809|687[0n]bp
Entheus Burns01|[751]|05-SRNP-31684|687[0n]bp
Entheus Burns01|[752]|05-SRNP-31469|669[0n]bp
Entheus Burns01|[753]|05-SRNP-31803|669[0n]bp
Entheus Burns01|[754]|05-SRNP-31805|669[0n]bp
Entheus Burns01|[755]|05-SRNP-31467|669[0n]bp
Entheus Burns01|[756]|05-SRNP-31811|669[0n]bp
Entheus Burns01|[757]|05-SRNP-31807|669[0n]bp
Entheus Burns01|[758]|05-SRNP-31470|669[0n]bp
Entheus Burns01|[759]|05-SRNP-31681|669[0n]bp
Entheus Burns01|[760]|05-SRNP-31680|669[0n]bp
Entheus Burns01|[761]|05-SRNP-31685|669[0n]bp
Entheus Burns01|[762]|04-SRNP-35514|669[0n]bp
Entheus Burns01|[763]|04-SRNP-56807|669[0n]bp
Entheus Burns01|[764]|05-SRNP-30012|669[0n]bp
Entheus Burns01|[765]|05-SRNP-30006|669[0n]bp
Entheus Burns01|[766]|05-SRNP-30261|669[0n]bp
Entheus Burns01|[767]|04-SRNP-32629|669[0n]bp
Entheus Burns01|[768]|04-SRNP-32406|669[0n]bp
Entheus Burns01|[769]|04-SRNP-32627|669[0n]bp
Entheus Burns01|[770]|04-SRNP-22955|669[0n]bp
Entheus Burns01|[771]|04-SRNP-32897|669[0n]bp
Entheus Burns01|[772]|04-SRNP-32435|669[0n]bp
Entheus Burns01|[773]|04-SRNP-32100|669[0n]bp
Entheus Burns01|[774]|04-SRNP-32971|669[0n]bp
Entheus Burns01|[775]|04-SRNP-32102|669[0n]bp
Entheus Burns01|[776]|04-SRNP-32620|669[0n]bp
Entheus Burns01|[777]|04-SRNP-32626|669[0n]bp
Entheus Burns01|[778]|04-SRNP-32624|669[0n]bp
Entheus Burns01|[779]|04-SRNP-32619|669[0n]bp
Entheus Burns01|[780]|03-SRNP-4063|669[0n]bp
Entheus Burns01|[781]|03-SRNP-4085|669[0n]bp
Entheus Burns01|[782]|03-SRNP-4083|669[0n]bp
Entheus Burns01|[783]|03-SRNP-4089|669[0n]bp
Entheus Burns01|[784]|03-SRNP-4031|669[0n]bp
Entheus Burns01|[785]|03-SRNP-3913|669[0n]bp
Entheus Burns01|[786]|03-SRNP-4079|669[0n]bp
Entheus Burns01|[787]|03-SRNP-4150|669[0n]bp
Entheus Burns01|[788]|03-SRNP-3910|669[0n]bp
Entheus Burns01|[789]|03-SRNP-4088|669[0n]bp
Entheus Burns01|[790]|03-SRNP-3911|669[0n]bp
Entheus Burns01|[791]|03-SRNP-4062|669[0n]bp
Entheus Burns01|[792]|05-SRNP-31988|669[0n]bp
Entheus Burns01|[793]|04-SRNP-32128|669[0n]bp
Entheus Burns01|[794]|02-SRNP-9578|657[0n]bp
Entheus Burns01|[795]|02-SRNP-8498|603[0n]bp
Entheus Burns01|[796]|02-SRNP-8540|603[0n]bp
Entheus Burns01|[797]|02-SRNP-9309|651[0n]bp
Entheus Burns01|[798]|08-SRNP-35619|669[0n]bp
Entheus Burns01|[799]|08-SRNP-35620|669[0n]bp
[illegible]

Entheus Burns01|[797]|02-SRNP-9309|651[0n]bp
Entheus Burns01|[798]|08-SRNP-35619|669[0n]bp
Entheus Burns01|[799]|08-SRNP-35620|669[0n]bp
Entheus Burns03|[800]|07-SRNP-65862|621[0n]bp
Entheus Burns03|[801]|03-SRNP-6132|654[0n]bp
Entheus Burns03|[802]|06-SRNP-44097|621[2n]bp
Entheus Burns03|[803]|07-SRNP-65775|648[0n]bp
Entheus Burns03|[804]|03-SRNP-21789|630[0n]bp
Entheus Burns03|[805]|02-SRNP-19102|630[0n]bp
Entheus Burns03|[806]|05-SRNP-195|669[0n]bp
Entheus Burns03|[807]|04-SRNP-56418|669[0n]bp
Entheus Burns03|[808]|05-SRNP-30875|615[0n]bp
Entheus Burns03|[809]|05-SRNP-54|687[0n]bp
Entheus Burns03|[810]|05-SRNP-1251|687[0n]bp
Entheus Burns03|[811]|07-SRNP-65331|669[0n]bp
Entheus Burns03|[812]|07-SRNP-65174|669[0n]bp
Entheus Burns03|[813]|07-SRNP-2018|669[0n]bp
Entheus Burns03|[814]|07-SRNP-2055|669[0n]bp
Entheus Burns03|[815]|07-SRNP-65148|669[0n]bp
Entheus Burns03|[816]|07-SRNP-1374|669[0n]bp
Entheus Burns03|[817]|07-SRNP-65011|669[0n]bp
Entheus Burns03|[818]|06-SRNP-44104|669[0n]bp
Entheus Burns03|[819]|06-SRNP-31151|669[0n]bp
Entheus Burns03|[820]|05-SRNP-1370|669[0n]bp
Entheus Burns03|[821]|04-SRNP-55949|669[0n]bp
Entheus Burns03|[822]|05-SRNP-196|669[0n]bp
Entheus Burns03|[823]|04-SRNP-55940|669[0n]bp
Entheus Burns03|[824]|04-SRNP-61137|669[0n]bp
Entheus Burns03|[825]|04-SRNP-61243|669[0n]bp
Entheus Burns03|[826]|04-SRNP-2113|669[0n]bp
Entheus Burns03|[827]|04-SRNP-1610|669[0n]bp
Entheus Burns03|[828]|04-SRNP-1609|669[0n]bp
Entheus Burns03|[829]|04-SRNP-60791|666[0n]bp
Entheus Burns03|[830]|06-SRNP-31150|669[1n]bp
Entheus Burns03|[831]|03-SRNP-21250|639[0n]bp
Entheus Burns03|[832]|03-SRNP-6130|663[0n]bp
Entheus Burns03|[833]|07-SRNP-65230|660[0n]bp
Entheus Burns03|[834]|07-SRNP-65191|669[0n]bp
Entheus Burns03|[835]|08-SRNP-65008|669[0n]bp
Entheus Burns03|[836]|08-SRNP-65118|669[0n]bp
Entheus Burns03|[837]|08-SRNP-65323|669[0n]bp
Entheus Burns03|[838]|08-SRNP-66040|669[0n]bp
Entheus Burns03|[839]|09-SRNP-56806|669[0n]bp
Entheus Burns02|[840]|08-SRNP-72638|642[2n]bp
Entheus Burns02|[841]|00-SRNP-2001|639[0n]bp
Entheus Burns02|[842]|99-SRNP-15392|639[0n]bp
Entheus Burns02|[843]|05-SRNP-31934|669[0n]bp
Entheus Burns02|[844]|08-SRNP-31023|669[0n]bp
Entheus Burns02|[845]|08-SRNP-71195|669[0n]bp
Entheus Burns02|[846]|08-SRNP-33091|669[0n]bp
Entheus Burns02|[847]|08-SRNP-71849|669[0n]bp
Entheus Burns02|[848]|08-SRNP-72316|669[0n]bp
Entheus Burns02|[849]|08-SRNP-71739|669[0n]bp
Entheus Burns02|[850]|08-SRNP-70953|669[0n]bp
Entheus Burns02|[851]|08-SRNP-70952|669[0n]bp
Entheus Burns02|[852]|08-SRNP-71814|669[0n]bp
Entheus Burns02|[853]|08-SRNP-71740|669[0n]bp
Entheus Burns02|[854]|08-SRNP-71134|669[0n]bp
Entheus Burns02|[855]|06-SRNP-20135|669[0n]bp
Entheus Burns02|[856]|06-SRNP-34837|669[0n]bp
Entheus Burns02|[857]|05-SRNP-21189|669[0n]bp
Entheus Burns02|[858]|05-SRNP-32113|669[0n]bp
Entheus Burns02|[859]|05-SRNP-34263|591[0n]bp
Entheus Burns02|[860]|06-SRNP-32638|609[0n]bp
Entheus Burns02|[861]|05-SRNP-34780|609[0n]bp
Entheus Burns02|[862]|02-SRNP-14694|642[0n]bp
Entheus Burns02|[863]|09-SRNP-30020|615[0n]bp
Entheus Burns02|[864]|09-SRNP-70294|669[0n]bp
Entheus Burns02|[865]|09-SRNP-71529|669[0n]bp
Entheus Burns02|[866]|09-SRNP-70531|669[0n]bp
Antigonus nearchus|[867]|00-SRNP-12980|627[0n]bp
Antigonus nearchus|[868]|00-SRNP-10364|645[1n]bp
Antigonus nearchus|[869]|06-SRNP-32799|669[0n]bp
Antigonus nearchus|[870]|01-SRNP-5567|657[0n]bp
Antigonus nearchus|[871]|96-SRNP-11137|606[0n]bp
Antigonus nearchus|[872]|06-SRNP-67851|657[0n]bp
Timochreon satyrus|[873]|98-SRNP-4800|576[0n]bp
Timochreon satyrus|[874]|07-SRNP-55646|669[0n]bp
Timochreon satyrus|[875]|07-SRNP-58884|669[0n]bp
Timochreon satyrus|[876]|07-SRNP-55299|669[0n]bp
Timochreon satyrus|[877]|06-SRNP-59556|669[0n]bp
Timochreon satyrus|[878]|06-SRNP-57946|669[0n]bp
Timochreon satyrus|[879]|02-SRNP-5343|669[0n]bp
Timochreon satyrus|[880]|00-SRNP-3407|669[0n]bp
Timochreon satyrus|[881]|01-SRNP-11952|669[0n]bp
Timochreon satyrus|[882]|05-SRNP-24369|669[0n]bp
Timochreon satyrus|[883]|04-SRNP-16184|669[0n]bp
Timochreon satyrus|[884]|04-SRNP-26992|669[0n]bp
Timochreon satyrus|[885]|04-SRNP-21555|669[0n]bp
Timochreon satyrus|[886]|97-SRNP-4864|660[0n]bp
Timochreon satyrus|[887]|02-SRNP-4030|654[0n]bp
Timochreon satyrus|[888]|02-SRNP-15405|630[1n]bp
Timochreon satyrus|[889]|97-SRNP-4132|606[0n]bp
Timochreon satyrus|[890]|01-SRNP-11309|642[0n]bp
Timochreon satyrus|[891]|02-SRNP-14811|621[0n]bp
Timochreon satyrus|[892]|02-SRNP-33938|606[0n]bp
Timochreon satyrus|[893]|07-SRNP-55645|669[0n]bp
Timochreon satyrus|[894]|07-SRNP-55655|669[0n]bp
Timochreon satyrus|[895]|09-SRNP-73053|669[0n]bp
Heliopetes lavianaDHJ02|[896]|06-SRNP-19429|669[0n]bp
Heliopetes lavianaDHJ02|[897]|06-SRNP-19419|669[0n]bp
Heliopetes lavianaDHJ02|[898]|06-SRNP-19431|669[0n]bp
Heliopetes lavianaDHJ02|[899]|06-SRNP-18783|669[0n]bp
[illegible]

Heliopetes lavianaDHJ02|[897]|06-SRNP-19419|669[0n]bp
Heliopetes lavianaDHJ02|[898]|06-SRNP-19431|669[0n]bp
Heliopetes lavianaDHJ02|[899]|06-SRNP-18783|669[0n]bp
Heliopetes lavianaDHJ02|[900]|06-SRNP-19423|669[0n]bp
Heliopetes lavianaDHJ02|[901]|06-SRNP-19396|669[0n]bp
Heliopetes lavianaDHJ02|[902]|06-SRNP-19523|669[0n]bp
Heliopetes lavianaDHJ02|[903]|06-SRNP-19420|669[0n]bp
Heliopetes lavianaDHJ02|[904]|06-SRNP-19397|669[0n]bp
Heliopetes lavianaDHJ02|[905]|06-SRNP-19416|669[0n]bp
Heliopetes lavianaDHJ02|[906]|06-SRNP-18781|669[0n]bp
Heliopetes lavianaDHJ02|[907]|04-SRNP-45705|669[0n]bp
Heliopetes lavianaDHJ02|[908]|04-SRNP-45704|660[0n]bp
Heliopetes lavianaDHJ02|[909]|06-SRNP-19448|669[0n]bp
Heliopetes lavianaDHJ02|[910]|06-SRNP-19402|669[0n]bp
Heliopetes lavianaDHJ02|[911]|06-SRNP-19622|669[0n]bp
Heliopetes lavianaDHJ02|[912]|06-SRNP-18780|669[0n]bp
Heliopetes lavianaDHJ02|[913]|06-SRNP-19414|669[0n]bp
Heliopetes lavianaDHJ02|[914]|06-SRNP-19401|669[0n]bp
Heliopetes alana|[915]|04-SRNP-48169|669[0n]bp
Heliopetes alana|[916]|05-SRNP-55030|669[0n]bp
Heliopetes alana|[917]|05-SRNP-5611|669[0n]bp
Heliopetes alana|[918]|06-SRNP-6703|669[0n]bp
Heliopetes arsalte|[919]|09-SRNP-2102|669[0n]bp
Heliopetes arsalte|[920]|09-SRNP-44424|669[0n]bp
Heliopetes arsalte|[921]|05-SRNP-31020|669[0n]bp
Heliopetes arsalte|[922]|05-SRNP-34906|630[0n]bp
Heliopetes arsalte|[923]|06-SRNP-2053|633[0n]bp
Heliopetes arsalte|[924]|04-SRNP-42488|669[0n]bp
Heliopetes arsalte|[925]|05-SRNP-3992|669[0n]bp
Heliopetes arsalte|[926]|06-SRNP-19606|669[0n]bp
Heliopetes arsalte|[927]|07-SRNP-55805|669[0n]bp
Heliopetes arsalte|[928]|09-SRNP-71844|669[0n]bp
Heliopetes arsalte|[929]|09-SRNP-2103|669[0n]bp
Heliopetes arsalte|[930]|09-SRNP-72759|669[0n]bp
Pyrgus adepta|[931]|05-SRNP-61486|669[0n]bp
Pyrgus adepta|[932]|05-SRNP-3108|669[0n]bp
Pyrgus adepta|[933]|05-SRNP-4774|669[0n]bp
Pyrgus adepta|[934]|05-SRNP-3111|669[0n]bp
Pyrgus adepta|[935]|05-SRNP-6162|630[0n]bp
Pyrgus adepta|[936]|06-SRNP-3986|669[0n]bp
Pyrgus adepta|[937]|06-SRNP-22079|669[0n]bp
Pyrgus adepta|[938]|07-SRNP-57963|669[0n]bp
Pyrgus adepta|[939]|08-SRNP-40797|669[0n]bp
Pyrgus adepta|[940]|09-SRNP-35755|669[0n]bp
Pyrgus oileusDHJ01|[941]|05-SRNP-47404|669[0n]bp
Pyrgus oileusDHJ02|[942]|09-SRNP-72609|669[0n]bp
Pyrgus oileusDHJ02|[943]|09-SRNP-57316|669[0n]bp
Pyrgus oileusDHJ02|[944]|04-SRNP-4880|669[0n]bp
Pyrgus oileusDHJ02|[945]|97-SRNP-6018|372[0n]bp
Pyrgus oileusDHJ02|[946]|97-SRNP-6017|588[4n]bp
Pyrgus oileusDHJ02|[947]|97-SRNP-6323|660[0n]bp
Pyrgus oileusDHJ02|[948]|07-SRNP-30612|669[0n]bp
Pyrgus oileusDHJ02|[949]|07-SRNP-41537|669[0n]bp
Pyrgus oileusDHJ02|[950]|07-SRNP-33658|669[0n]bp
Pyrgus oileusDHJ02|[951]|09-SRNP-41756|669[0n]bp
Pyrgus oileusDHJ03|[952]|05-SRNP-41121|669[0n]bp
Pyrgus oileusDHJ03|[953]|05-SRNP-41120|684[0n]bp
Pyrgus oileusDHJ03|[954]|97-SRNP-468|669[0n]bp
Pyrgus oileusDHJ03|[955]|05-SRNP-5995|624[0n]bp
Pyrgus oileusDHJ03|[956]|93-SRNP-6385|666[1n]bp
Pyrgus oileusDHJ03|[957]|06-SRNP-59892|669[0n]bp
Pyrgus oileusDHJ03|[958]|05-SRNP-42130|669[0n]bp
Pyrgus oileusDHJ03|[959]|05-SRNP-3112|669[0n]bp
Pyrgus oileusDHJ03|[960]|04-SRNP-4881|669[0n]bp
Pyrgus oileusDHJ03|[961]|05-SRNP-56295|684[0n]bp
Pyrgus oileusDHJ03|[962]|07-SRNP-58554|660[0n]bp
Pyrgus oileusDHJ03|[963]|08-SRNP-71705|669[0n]bp
Pyrgus oileusDHJ03|[964]|09-SRNP-44136|669[0n]bp
Pyrgus oileusDHJ03|[965]|09-SRNP-73409|669[0n]bp
Pyrgus oileusDHJ03|[966]|09-SRNP-73394|669[0n]bp
Zopyrion sandace|[967]|93-SRNP-7566|669[0n]bp
Zopyrion sandace|[968]|97-SRNP-9768|669[0n]bp
Antigonus erosus|[969]|04-SRNP-14882|669[0n]bp
Antigonus erosus|[970]|04-SRNP-15034|669[0n]bp
Antigonus erosus|[971]|07-SRNP-57033|669[0n]bp
Antigonus erosus|[972]|04-SRNP-14311|669[0n]bp
Antigonus erosus|[973]|04-SRNP-47715|669[1n]bp
Antigonus erosus|[974]|04-SRNP-48661|669[0n]bp
Antigonus erosus|[975]|05-SRNP-34080|573[0n]bp
Antigonus erosus|[976]|05-SRNP-2158|669[0n]bp
Antigonus erosus|[977]|05-SRNP-45294|669[0n]bp
Antigonus erosus|[978]|05-SRNP-2157|669[0n]bp
Antigonus erosus|[979]|05-SRNP-55042|669[0n]bp
Antigonus erosus|[980]|07-SRNP-40136|669[0n]bp
Antigonus erosus|[981]|06-SRNP-6733|669[0n]bp
Antigonus erosus|[982]|06-SRNP-44597|669[0n]bp
Antigonus erosus|[983]|04-SRNP-15182|669[0n]bp
Antigonus erosus|[984]|04-SRNP-48127|669[0n]bp
Antigonus erosus|[985]|04-SRNP-47713|669[0n]bp
Antigonus erosus|[986]|04-SRNP-48470|669[0n]bp
Antigonus erosus|[987]|04-SRNP-4027|669[0n]bp
Antigonus erosus|[988]|04-SRNP-15037|669[0n]bp
Antigonus erosus|[989]|04-SRNP-14884|669[0n]bp
Antigonus erosus|[990]|04-SRNP-14704|669[0n]bp
Antigonus erosus|[991]|04-SRNP-48663|669[0n]bp
Antigonus erosus|[992]|04-SRNP-46846|669[0n]bp
Antigonus erosus|[993]|03-SRNP-916|642[0n]bp
Antigonus erosus|[994]|04-SRNP-14889|669[1n]bp
Antigonus erosus|[995]|06-SRNP-5959|669[0n]bp
Antigonus erosus|[996]|03-SRNP-5120|657[0n]bp
Antigonus erosus|[997]|05-SRNP-5668|645[0n]bp
Antigonus erosus|[998]|07-SRNP-2954|669[0n]bp
Antigonus erosus|[999]|07-SRNP-42448|669[0n]bp
Antigonus erosus|[1000]|07-SRNP-[illegible]|669[0n]bp

Antigonus erosus|[997]|05-SRNP-5668|645[0n]bp
Antigonus erosus|[998]|07-SRNP-2954|669[0n]bp
Antigonus erosus|[999]|07-SRNP-42448|669[0n]bp
Antigonus erosus|[1000]|07-SRNP-3283|669[0n]bp
Antigonus erosus|[1001]|07-SRNP-23787|669[0n]bp
Antigonus erosus|[1002]|08-SRNP-31067|669[0n]bp
Antigonus erosus|[1003]|09-SRNP-43129|669[0n]bp
Antigonus erosus|[1004]|09-SRNP-67761|669[0n]bp
Carrhenes calidius|[1005]|01-SRNP-11410|549[0n]bp
Carrhenes calidius|[1006]|05-SRNP-1277|642[2n]bp
Carrhenes calidius|[1007]|08-SRNP-21848|669[0n]bp
Carrhenes calidius|[1008]|08-SRNP-21846|669[0n]bp
Carrhenes calidius|[1009]|08-SRNP-6834|669[0n]bp
Carrhenes calidius|[1010]|06-SRNP-42410|669[0n]bp
Carrhenes calidius|[1011]|06-SRNP-42411|669[0n]bp
Carrhenes calidius|[1012]|05-SRNP-41755|645[2n]bp
Carrhenes calidius|[1013]|06-SRNP-23205|669[0n]bp
Carrhenes calidius|[1014]|06-SRNP-23245|669[0n]bp
Carrhenes calidius|[1015]|97-SRNP-5991|627[0n]bp
Carrhenes calidius|[1016]|05-SRNP-41905|669[6n]bp
Carrhenes calidius|[1017]|05-SRNP-41664|654[1n]bp
Carrhenes calidius|[1018]|08-SRNP-4738|669[0n]bp
Carrhenes calidius|[1019]|08-SRNP-4735|669[0n]bp
Carrhenes calidius|[1020]|08-SRNP-4737|669[0n]bp
Carrhenes calidius|[1021]|08-SRNP-4736|669[0n]bp
Carrhenes calidius|[1022]|08-SRNP-21817|669[0n]bp
Carrhenes calidius|[1023]|08-SRNP-20689|669[0n]bp
Carrhenes calidius|[1024]|08-SRNP-21489|669[0n]bp
Carrhenes calidius|[1025]|08-SRNP-20418|669[0n]bp
Carrhenes calidius|[1026]|06-SRNP-46362|669[0n]bp
Carrhenes calidius|[1027]|06-SRNP-23272|669[0n]bp
Carrhenes calidius|[1028]|06-SRNP-23273|669[0n]bp
Carrhenes calidius|[1029]|06-SRNP-23275|669[0n]bp
Carrhenes calidius|[1030]|06-SRNP-42412|669[0n]bp
Carrhenes calidius|[1031]|06-SRNP-42408|669[0n]bp
Carrhenes calidius|[1032]|04-SRNP-42196|669[0n]bp
Carrhenes calidius|[1033]|04-SRNP-49279|669[0n]bp
Carrhenes calidius|[1034]|03-SRNP-37789|669[0n]bp
Carrhenes calidius|[1035]|04-SRNP-40341|669[0n]bp
Carrhenes calidius|[1036]|05-SRNP-4276|654[2n]bp
Carrhenes calidius|[1037]|05-SRNP-25254|576[0n]bp
Carrhenes calidius|[1038]|06-SRNP-22483|633[0n]bp
Carrhenes calidius|[1039]|09-SRNP-21630|669[1n]bp
Carrhenes fuscescens|[1040]|92-SRNP-3412|663[0n]bp
Carrhenes fuscescens|[1041]|95-SRNP-6803|669[0n]bp
Carrhenes fuscescens|[1042]|95-SRNP-6809|669[0n]bp
Carrhenes fuscescens|[1043]|96-SRNP-7811|669[0n]bp
Carrhenes fuscescens|[1044]|95-SRNP-6818|669[0n]bp
Carrhenes fuscescens|[1045]|95-SRNP-6813|669[0n]bp
Carrhenes fuscescens|[1046]|95-SRNP-6806|669[0n]bp
Carrhenes fuscescens|[1047]|95-SRNP-6801|669[0n]bp
Carrhenes fuscescens|[1048]|95-SRNP-6819|630[0n]bp
Carrhenes fuscescens|[1049]|95-SRNP-6804|627[0n]bp
Carrhenes fuscescens|[1050]|92-SRNP-3012|570[2n]bp
Carrhenes fuscescens|[1051]|92-SRNP-3014|609[0n]bp
Carrhenes fuscescens|[1052]|93-SRNP-1137|669[0n]bp
Carrhenes fuscescens|[1053]|95-SRNP-6820|669[0n]bp
Carrhenes fuscescens|[1054]|92-SRNP-3004|669[0n]bp
Carrhenes fuscescens|[1055]|92-SRNP-3393|669[0n]bp
Anisochoria polysticta|[1056]|03-SRNP-26746|660[0n]bp
Anisochoria polysticta|[1057]|98-SRNP-12227|639[0n]bp
Anisochoria polysticta|[1058]|04-SRNP-15485|669[0n]bp
Anisochoria polysticta|[1059]|04-SRNP-15751|669[0n]bp
Anisochoria polysticta|[1060]|04-SRNP-15486|669[0n]bp
Anisochoria polysticta|[1061]|07-SRNP-55564|669[0n]bp
Xenophanes tryxus|[1062]|05-SRNP-55815|669[1n]bp
Xenophanes tryxus|[1063]|04-SRNP-23772|633[0n]bp
Xenophanes tryxus|[1064]|08-SRNP-709|669[0n]bp
Xenophanes tryxus|[1065]|08-SRNP-40407|669[0n]bp
Xenophanes tryxus|[1066]|08-SRNP-40406|669[0n]bp
Xenophanes tryxus|[1067]|07-SRNP-42914|669[0n]bp
Xenophanes tryxus|[1068]|07-SRNP-21825|669[0n]bp
Xenophanes tryxus|[1069]|07-SRNP-42042|669[0n]bp
Xenophanes tryxus|[1070]|07-SRNP-2177|669[0n]bp
Xenophanes tryxus|[1071]|07-SRNP-2117|669[0n]bp
Xenophanes tryxus|[1072]|07-SRNP-32155|669[0n]bp
Xenophanes tryxus|[1073]|05-SRNP-55198|669[0n]bp
Xenophanes tryxus|[1074]|04-SRNP-15683|669[0n]bp
Xenophanes tryxus|[1075]|04-SRNP-41977|669[0n]bp
Xenophanes tryxus|[1076]|04-SRNP-15686|669[0n]bp
Xenophanes tryxus|[1077]|04-SRNP-23770|669[0n]bp
Xenophanes tryxus|[1078]|04-SRNP-23767|669[0n]bp
Xenophanes tryxus|[1079]|04-SRNP-23769|669[0n]bp
Xenophanes tryxus|[1080]|04-SRNP-24073|669[1n]bp
Xenophanes tryxus|[1081]|05-SRNP-30987|669[2n]bp
Xenophanes tryxus|[1082]|08-SRNP-40408|594[3n]bp
Xenophanes tryxus|[1083]|92-SRNP-4663|399[0n]bp
Xenophanes tryxus|[1084]|93-SRNP-5818|399[0n]bp
Xenophanes tryxus|[1085]|08-SRNP-40109|630[0n]bp
Xenophanes tryxus|[1086]|08-SRNP-40108|669[0n]bp
Xenophanes tryxus|[1087]|08-SRNP-652|669[0n]bp
Xenophanes tryxus|[1088]|08-SRNP-653|669[0n]bp
Xenophanes tryxus|[1089]|08-SRNP-650|669[0n]bp
Xenophanes tryxus|[1090]|08-SRNP-710|669[0n]bp
Xenophanes tryxus|[1091]|08-SRNP-1428|669[0n]bp
Xenophanes tryxus|[1092]|09-SRNP-75047|669[0n]bp
Xenophanes tryxus|[1093]|09-SRNP-42129|669[0n]bp
Carrhenes meridensis|[1094]|97-SRNP-1522|624[0n]bp
Carrhenes canescensDHJ02|[1095]|04-SRNP-47072|669[0n]bp
Carrhenes canescensDHJ02|[1096]|05-SRNP-65241|576[0n]bp
Carrhenes canescensDHJ02|[1097]|04-SRNP-2161|669[0n]bp
Carrhenes canescensDHJ02|[1098]|08-SRNP-1435|669[0n]bp
Carrhenes canescensDHJ02|[1099]|07-SRNP-2297|669[0n]bp
Carrhenes canescensDHJ02|[1100]|07-SRNP-57525|669[0n]bp

Carrhenes canescensDHJ02|[1097]|04-SRNP-2161|669[0n]bp
Carrhenes canescensDHJ02|[1098]|08-SRNP-1435|669[0n]bp
Carrhenes canescensDHJ02|[1099]|07-SRNP-2297|669[0n]bp
Carrhenes canescensDHJ02|[1100]|07-SRNP-57525|669[0n]bp
Carrhenes canescensDHJ02|[1101]|05-SRNP-55226|669[0n]bp
Carrhenes canescensDHJ02|[1102]|04-SRNP-40196|639[0n]bp
Carrhenes canescensDHJ02|[1103]|94-SRNP-576|618[0n]bp
Carrhenes canescensDHJ02|[1104]|97-SRNP-796|669[0n]bp
Carrhenes canescensDHJ02|[1105]|98-SRNP-6223|669[0n]bp
Carrhenes canescensDHJ02|[1106]|98-SRNP-2076|669[0n]bp
Carrhenes canescensDHJ02|[1107]|98-SRNP-2077|669[0n]bp
Carrhenes canescensDHJ02|[1108]|09-SRNP-55887|669[0n]bp
Carrhenes canescensDHJ02|[1109]|08-SRNP-1302|669[0n]bp
Carrhenes canescensDHJ02|[1110]|04-SRNP-35287|669[0n]bp
Carrhenes canescensDHJ02|[1111]|98-SRNP-2086|627[0n]bp
Carrhenes canescensDHJ02|[1112]|06-SRNP-3394|666[0n]bp
Carrhenes canescensDHJ02|[1113]|94-SRNP-572|399[0n]bp
Carrhenes canescensDHJ02|[1114]|94-SRNP-598|606[0n]bp
Carrhenes canescensDHJ02|[1115]|00-SRNP-11710|669[0n]bp
Carrhenes canescensDHJ02|[1116]|02-SRNP-32202|669[0n]bp
Carrhenes canescensDHJ02|[1117]|98-SRNP-2075|669[0n]bp
Carrhenes canescensDHJ02|[1118]|98-SRNP-6658|669[0n]bp
Carrhenes canescensDHJ02|[1119]|99-SRNP-4815|669[0n]bp
Carrhenes canescensDHJ01|[1120]|08-SRNP-1544|669[0n]bp
Carrhenes canescensDHJ01|[1121]|01-SRNP-2081|669[0n]bp
Carrhenes canescensDHJ01|[1122]|96-SRNP-11205|669[0n]bp
Carrhenes canescensDHJ01|[1123]|99-SRNP-2264|669[0n]bp
Carrhenes canescensDHJ01|[1124]|99-SRNP-2257|669[0n]bp
Carrhenes canescensDHJ01|[1125]|02-SRNP-18792|669[0n]bp
Carrhenes canescensDHJ01|[1126]|98-SRNP-4264|669[0n]bp
Carrhenes canescensDHJ01|[1127]|04-SRNP-45774|669[0n]bp
Carrhenes canescensDHJ01|[1128]|01-SRNP-1279|669[0n]bp
Carrhenes canescensDHJ01|[1129]|98-SRNP-6283|669[0n]bp
Carrhenes canescensDHJ01|[1130]|97-SRNP-6032|669[0n]bp
Carrhenes canescensDHJ01|[1131]|98-SRNP-6194|669[0n]bp
Carrhenes canescensDHJ01|[1132]|98-SRNP-6497|669[0n]bp
Carrhenes canescensDHJ01|[1133]|98-SRNP-6704|669[0n]bp
Carrhenes canescensDHJ01|[1134]|08-SRNP-45047|669[0n]bp
Carrhenes canescensDHJ01|[1135]|07-SRNP-1460|669[0n]bp
Carrhenes canescensDHJ01|[1136]|06-SRNP-59186|669[0n]bp
Carrhenes canescensDHJ01|[1137]|06-SRNP-3104|669[0n]bp
Carrhenes canescensDHJ01|[1138]|06-SRNP-2357|669[0n]bp
Carrhenes canescensDHJ01|[1139]|06-SRNP-3298|669[0n]bp
Carrhenes canescensDHJ01|[1140]|04-SRNP-35258|669[0n]bp
Carrhenes canescensDHJ01|[1141]|00-SRNP-1813|669[0n]bp
Carrhenes canescensDHJ01|[1142]|04-SRNP-48517|669[0n]bp
Carrhenes canescensDHJ01|[1143]|05-SRNP-65401|522[0n]bp
Carrhenes canescensDHJ01|[1144]|98-SRNP-6890|654[0n]bp
Carrhenes canescensDHJ01|[1145]|97-SRNP-6031|669[0n]bp
Atarnes sallei|[1146]|06-SRNP-45182|669[0n]bp
Atarnes sallei|[1147]|06-SRNP-1582|669[0n]bp
Atarnes sallei|[1148]|07-SRNP-35256|669[0n]bp
Atarnes sallei|[1149]|08-SRNP-20794|669[0n]bp
Atarnes sallei|[1150]|06-SRNP-3263|669[0n]bp
Atarnes sallei|[1151]|05-SRNP-2058|687[0n]bp
Atarnes sallei|[1152]|08-SRNP-36067|669[0n]bp
Atarnes sallei|[1153]|08-SRNP-56142|669[0n]bp
Atarnes sallei|[1154]|07-SRNP-58936|669[0n]bp
Atarnes sallei|[1155]|07-SRNP-59529|669[0n]bp
Atarnes sallei|[1156]|06-SRNP-18572|669[0n]bp
Atarnes sallei|[1157]|06-SRNP-59199|669[0n]bp
Atarnes sallei|[1158]|06-SRNP-3340|669[0n]bp
Atarnes sallei|[1159]|06-SRNP-35204|618[5n]bp
Atarnes sallei|[1160]|05-SRNP-60344|630[0n]bp
Atarnes sallei|[1161]|05-SRNP-61255|627[0n]bp
Atarnes sallei|[1162]|05-SRNP-60346|627[1n]bp
Atarnes sallei|[1163]|06-SRNP-55198|591[0n]bp
Atarnes sallei|[1164]|95-SRNP-435|576[1n]bp
Atarnes sallei|[1165]|89-SRNP-92|357[0n]bp
Atarnes sallei|[1166]|06-SRNP-21052|363[0n]bp
Atarnes sallei|[1167]|06-SRNP-21070|603[2n]bp
Atarnes sallei|[1168]|06-SRNP-57921|612[0n]bp
Atarnes sallei|[1169]|07-SRNP-56900|621[0n]bp
Atarnes sallei|[1170]|07-SRNP-65059|621[0n]bp
Atarnes sallei|[1171]|07-SRNP-65058|669[0n]bp
Atarnes sallei|[1172]|08-SRNP-56256|669[0n]bp
Potamanaxas unifasciata|[1173]|07-SRNP-2633|633[0n]bp
Potamanaxas unifasciata|[1174]|98-SRNP-2034|669[0n]bp
Potamanaxas unifasciata|[1175]|04-SRNP-805|669[0n]bp
Potamanaxas unifasciata|[1176]|05-SRNP-58611|669[0n]bp
Potamanaxas unifasciata|[1177]|05-SRNP-35001|669[0n]bp
Potamanaxas unifasciata|[1178]|94-SRNP-6414|669[0n]bp
Potamanaxas unifasciata|[1179]|00-SRNP-12802|669[0n]bp
Potamanaxas unifasciata|[1180]|00-SRNP-12804|669[0n]bp
Potamanaxas unifasciata|[1181]|04-SRNP-363|669[0n]bp
Potamanaxas unifasciata|[1182]|04-SRNP-365|669[0n]bp
Potamanaxas unifasciata|[1183]|00-SRNP-12805|669[0n]bp
Potamanaxas unifasciata|[1184]|04-SRNP-496|669[1n]bp
Potamanaxas unifasciata|[1185]|98-SRNP-2036|669[0n]bp
Potamanaxas unifasciata|[1186]|04-SRNP-364|669[0n]bp
Potamanaxas unifasciata|[1187]|95-SRNP-433|630[0n]bp
Potamanaxas unifasciata|[1188]|95-SRNP-437|576[1n]bp
Potamanaxas unifasciata|[1189]|95-SRNP-441|603[0n]bp
Potamanaxas unifasciata|[1190]|95-SRNP-436|603[0n]bp
Potamanaxas unifasciata|[1191]|95-SRNP-438|558[0n]bp
Potamanaxas unifasciata|[1192]|03-SRNP-4147|564[0n]bp
Potamanaxas unifasciata|[1193]|03-SRNP-5425|570[0n]bp
Potamanaxas unifasciata|[1194]|00-SRNP-12803|633[0n]bp
Potamanaxas unifasciata|[1195]|06-SRNP-21553|633[0n]bp
Potamanaxas unifasciata|[1196]|04-SRNP-2128|669[0n]bp
Potamanaxas unifasciata|[1197]|05-SRNP-35251|669[0n]bp
Potamanaxas unifasciata|[1198]|08-SRNP-56559|669[0n]bp
Milanion marciana|[1199]|07-SRNP-41972|606[0n]bp
[illegible]

Potamanaxas unifasciata|[1197]|[illegible]|669[0n]bp
Potamanaxas unifasciata|[1198]|08-SRNP-56559|669[0n]bp
Milanion marciana|[1199]|07-SRNP-41972|606[0n]bp
Milanion marciana|[1200]|07-SRNP-1128|669[0n]bp
Milanion marciana|[1201]|04-SRNP-41660|669[0n]bp
Milanion marciana|[1202]|04-SRNP-42477|669[0n]bp
Milanion marciana|[1203]|05-SRNP-188|669[0n]bp
Milanion marciana|[1204]|01-SRNP-5343|630[0n]bp
Milanion marciana|[1205]|01-SRNP-5185|642[0n]bp
Milanion marciana|[1206]|04-SRNP-41204|366[0n]bp
Milanion marciana|[1207]|04-SRNP-42614|669[0n]bp
Milanion marciana|[1208]|05-SRNP-41228|669[0n]bp
Milanion marciana|[1209]|07-SRNP-42347|669[0n]bp
Milanion marciana|[1210]|09-SRNP-42042|669[0n]bp
Pythonides amaryllis|[1211]|06-SRNP-7522|669[0n]bp
Pythonides amaryllis|[1212]|07-SRNP-2293|669[0n]bp
Pythonides amaryllis|[1213]|07-SRNP-45416|669[0n]bp
Pythonides amaryllis|[1214]|06-SRNP-65338|669[0n]bp
Pythonides amaryllis|[1215]|07-SRNP-2936|669[0n]bp
Pythonides amaryllis|[1216]|07-SRNP-65576|669[0n]bp
Pythonides amaryllis|[1217]|08-SRNP-70591|669[0n]bp
Pythonides amaryllis|[1218]|07-SRNP-296|669[0n]bp
Pythonides amaryllis|[1219]|02-SRNP-547|630[0n]bp
Pythonides amaryllis|[1220]|07-SRNP-45759|669[0n]bp
Pythonides amaryllis|[1221]|07-SRNP-45350|669[0n]bp
Pythonides amaryllis|[1222]|07-SRNP-41120|669[0n]bp
Pythonides amaryllis|[1223]|07-SRNP-2294|669[0n]bp
Pythonides amaryllis|[1224]|07-SRNP-949|669[0n]bp
Pythonides amaryllis|[1225]|07-SRNP-30555|669[0n]bp
Pythonides amaryllis|[1226]|07-SRNP-30884|669[0n]bp
Pythonides amaryllis|[1227]|06-SRNP-65311|669[0n]bp
Pythonides amaryllis|[1228]|06-SRNP-6930|669[0n]bp
Pythonides amaryllis|[1229]|06-SRNP-9220|669[0n]bp
Pythonides amaryllis|[1230]|06-SRNP-65571|645[0n]bp
Pythonides amaryllis|[1231]|06-SRNP-7674|645[0n]bp
Pythonides amaryllis|[1232]|02-SRNP-6320|645[0n]bp
Pythonides amaryllis|[1233]|07-SRNP-45758|666[0n]bp
Pythonides amaryllis|[1234]|06-SRNP-9644|648[0n]bp
Pythonides amaryllis|[1235]|07-SRNP-45250|657[0n]bp
Pythonides amaryllis|[1236]|07-SRNP-65279|657[0n]bp
Pythonides amaryllis|[1237]|08-SRNP-70034|621[0n]bp
Pythonides amaryllis|[1238]|08-SRNP-71826|669[0n]bp
Pythonides pteras|[1239]|05-SRNP-31884|669[0n]bp
Pythonides pteras|[1240]|05-SRNP-31882|669[0n]bp
Pythonides pteras|[1241]|05-SRNP-32629|669[0n]bp
Pythonides pteras|[1242]|06-SRNP-32649|669[0n]bp
Pythonides pteras|[1243]|06-SRNP-32650|669[0n]bp
Pythonides pteras|[1244]|07-SRNP-32651|669[0n]bp
Morvina fissimacula pelarge|[1245]|07-SRNP-41267|645[0n]bp
Morvina fissimacula pelarge|[1246]|06-SRNP-31970|669[0n]bp
Morvina fissimacula pelarge|[1247]|06-SRNP-31945|669[0n]bp
Morvina fissimacula pelarge|[1248]|06-SRNP-32336|666[0n]bp
Morvina fissimacula pelarge|[1249]|02-SRNP-6631|657[0n]bp
Morvina fissimacula pelarge|[1250]|02-SRNP-6922|657[0n]bp
Morvina fissimacula pelarge|[1251]|06-SRNP-33952|660[0n]bp
Morvina fissimacula pelarge|[1252]|06-SRNP-34246|654[2n]bp
Morvina fissimacula pelarge|[1253]|06-SRNP-34680|669[0n]bp
Morvina fissimacula pelarge|[1254]|06-SRNP-65215|669[0n]bp
Morvina fissimacula pelarge|[1255]|04-SRNP-2511|669[0n]bp
Morvina fissimacula pelarge|[1256]|08-SRNP-65933|669[0n]bp
Ouleus cyrna|[1257]|03-SRNP-22100|669[1n]bp
Ouleus cyrna|[1258]|00-SRNP-23507|564[1n]bp
Ouleus cyrna|[1259]|03-SRNP-22212|633[0n]bp
Ouleus cyrna|[1260]|06-SRNP-35224|666[0n]bp
Zera hosta|[1261]|02-SRNP-24501|642[0n]bp
Zera hosta|[1262]|02-SRNP-24500|639[0n]bp
Zera hosta|[1263]|03-SRNP-3007|669[0n]bp
Zera hosta|[1264]|03-SRNP-3008|669[0n]bp
Zera hosta|[1265]|02-SRNP-24498|669[0n]bp
Zera Burns01DHJ03|[1266]|07-SRNP-41866|669[0n]bp
Zera Burns01DHJ03|[1267]|06-SRNP-6823|624[0n]bp
Zera Burns01DHJ03|[1268]|08-SRNP-4248|669[1n]bp
Zera Burns01DHJ02|[1269]|06-SRNP-22961|669[0n]bp
Zera Burns01DHJ02|[1270]|06-SRNP-9109|669[0n]bp
Zera Burns01DHJ02|[1271]|06-SRNP-9107|669[0n]bp
Zera Burns01DHJ02|[1272]|06-SRNP-9105|669[0n]bp
Zera Burns01DHJ02|[1273]|02-SRNP-24499|669[0n]bp
Zera Burns01DHJ02|[1274]|02-SRNP-24503|669[0n]bp
Zera Burns01DHJ02|[1275]|02-SRNP-4228|669[0n]bp
Zera Burns01DHJ02|[1276]|05-SRNP-23870|669[0n]bp
Zera Burns01DHJ02|[1277]|05-SRNP-55739|669[0n]bp
Zera Burns01DHJ02|[1278]|06-SRNP-9106|663[0n]bp
Zera Burns01DHJ02|[1279]|07-SRNP-32559|645[0n]bp
Zera Burns01DHJ02|[1280]|07-SRNP-3659|669[0n]bp
Zera Burns01DHJ02|[1281]|08-SRNP-56931|669[0n]bp
Zera Burns01DHJ02|[1282]|08-SRNP-56930|669[0n]bp
Gindanes brontinus|[1283]|08-SRNP-57236|669[0n]bp
Gindanes brontinus|[1284]|06-SRNP-58321|669[0n]bp
Gindanes brontinus|[1285]|05-SRNP-63509|669[0n]bp
Gindanes brontinus|[1286]|05-SRNP-65501|669[0n]bp
Gindanes brontinus|[1287]|04-SRNP-14564|669[0n]bp
Gindanes brontinus|[1288]|04-SRNP-14565|669[0n]bp
Gindanes brontinus|[1289]|94-SRNP-15.1|399[0n]bp
Gindanes brontinus|[1290]|94-SRNP-10|627[0n]bp
Gindanes brontinus|[1291]|04-SRNP-14563|630[0n]bp
Gindanes brontinus|[1292]|05-SRNP-64401|630[0n]bp
Gindanes brontinus|[1293]|05-SRNP-65494|630[0n]bp
Gindanes brontinus|[1294]|08-SRNP-57426|669[0n]bp
Pythonides proxenus|[1295]|07-SRNP-42356|666[0n]bp
Pythonides proxenus|[1296]|06-SRNP-6782|669[0n]bp
Pythonides proxenus|[1297]|06-SRNP-23081|669[0n]bp
Pythonides proxenus|[1298]|04-SRNP-41615|669[0n]bp
Pythonides proxenus|[1299]|04-SRNP-26794|669[1n]bp
Pythonides proxenus|[1300]|[illegible]

Pythonides proxenus|[1297]|06-SRNP-[illegible]|669[0n]bp
Pythonides proxenus|[1298]|04-SRNP-41615|669[0n]bp
Pythonides proxenus|[1299]|04-SRNP-26794|669[1n]bp
Pythonides proxenus|[1300]|02-SRNP-20155|645[0n]bp
Pythonides proxenus|[1301]|02-SRNP-20156|630[0n]bp
Pythonides proxenus|[1302]|07-SRNP-21605|669[0n]bp
Pythonides proxenus|[1303]|07-SRNP-21699|669[0n]bp
Pythonides proxenus|[1304]|08-SRNP-40390|585[0n]bp
Pythonides proxenus|[1305]|09-SRNP-22673|669[0n]bp
Pythonides proxenus|[1306]|04-SRNP-26795|687[0n]bp
Pythonides proxenus|[1307]|06-SRNP-44676|669[0n]bp
Pythonides proxenus|[1308]|07-SRNP-3103|669[0n]bp
Pythonides proxenus|[1309]|06-SRNP-36323|669[0n]bp
Pythonides proxenus|[1310]|06-SRNP-44677|669[0n]bp
Pythonides proxenus|[1311]|06-SRNP-43506|669[0n]bp
Pythonides proxenus|[1312]|06-SRNP-22943|669[0n]bp
Pythonides proxenus|[1313]|04-SRNP-60627|669[0n]bp
Pythonides proxenus|[1314]|04-SRNP-45116|669[0n]bp
Pythonides proxenus|[1315]|08-SRNP-1431|669[0n]bp
Pythonides proxenus|[1316]|09-SRNP-20219|669[0n]bp
Pythonides proxenus|[1317]|09-SRNP-20218|669[0n]bp
Pythonides proxenus|[1318]|09-SRNP-20220|669[0n]bp
Quadrus francesius|[1319]|04-SRNP-3839|669[0n]bp
Quadrus francesius|[1320]|04-SRNP-4110|669[0n]bp
Quadrus francesius|[1321]|06-SRNP-7067|669[0n]bp
Quadrus francesius|[1322]|06-SRNP-7066|669[0n]bp
Quadrus francesius|[1323]|08-SRNP-4233|669[0n]bp
Quadrus francesius|[1324]|08-SRNP-4232|669[0n]bp
Quadrus francesius|[1325]|05-SRNP-5074|669[0n]bp
Quadrus francesius|[1326]|05-SRNP-31504|669[0n]bp
Quadrus francesius|[1327]|04-SRNP-2313|669[0n]bp
Quadrus francesius|[1328]|07-SRNP-21126|645[0n]bp
Quadrus francesius|[1329]|05-SRNP-21430|669[0n]bp
Quadrus francesius|[1330]|05-SRNP-60|669[0n]bp
Quadrus francesius|[1331]|05-SRNP-62|669[0n]bp
Quadrus francesius|[1332]|04-SRNP-4111|669[0n]bp
Quadrus francesius|[1333]|05-SRNP-30292|669[0n]bp
Quadrus francesius|[1334]|04-SRNP-3262|669[0n]bp
Quadrus francesius|[1335]|03-SRNP-5537|663[0n]bp
Quadrus francesius|[1336]|03-SRNP-5745|633[0n]bp
Quadrus francesius|[1337]|08-SRNP-30995|621[0n]bp
Quadrus francesius|[1338]|09-SRNP-80528|669[0n]bp
Quadrus contubernalis|[1339]|01-SRNP-2577|630[0n]bp
Quadrus contubernalis|[1340]|07-SRNP-2968|669[0n]bp
Quadrus contubernalis|[1341]|08-SRNP-794|669[0n]bp
Quadrus contubernalis|[1342]|04-SRNP-3684|669[0n]bp
Quadrus contubernalis|[1343]|04-SRNP-3683|669[0n]bp
Quadrus contubernalis|[1344]|04-SRNP-60962|669[0n]bp
Quadrus contubernalis|[1345]|04-SRNP-4515|669[0n]bp
Quadrus contubernalis|[1346]|04-SRNP-4514|669[0n]bp
Quadrus contubernalis|[1347]|04-SRNP-3680|669[0n]bp
Quadrus contubernalis|[1348]|02-SRNP-19658|657[0n]bp
Quadrus contubernalis|[1349]|04-SRNP-60961|627[0n]bp
Quadrus contubernalis|[1350]|06-SRNP-2063|627[0n]bp
Quadrus contubernalis|[1351]|06-SRNP-2062|669[0n]bp
Quadrus contubernalis|[1352]|06-SRNP-2192|669[0n]bp
Quadrus contubernalis|[1353]|06-SRNP-3969|669[0n]bp
Quadrus contubernalis|[1354]|06-SRNP-7060|669[0n]bp
Quadrus contubernalis|[1355]|06-SRNP-7061|669[0n]bp
Quadrus contubernalis|[1356]|07-SRNP-1429|669[0n]bp
Quadrus contubernalis|[1357]|07-SRNP-2969|669[0n]bp
Quadrus contubernalis|[1358]|08-SRNP-793|669[0n]bp
Ouleus dilla baruDHJ01|[1359]|02-SRNP-29|669[0n]bp
Ouleus dilla baruDHJ01|[1360]|05-SRNP-34972|669[0n]bp
Ouleus dilla baruDHJ01|[1361]|06-SRNP-44270|669[0n]bp
Ouleus dilla baruDHJ01|[1362]|08-SRNP-40639|669[0n]bp
Ouleus dilla baruDHJ02|[1363]|05-SRNP-314|669[1n]bp
Ouleus dilla baruDHJ02|[1364]|04-SRNP-40712|669[0n]bp
Ouleus dilla baruDHJ02|[1365]|04-SRNP-4906|669[0n]bp
Ouleus dilla baruDHJ02|[1366]|04-SRNP-42883|669[0n]bp
Ouleus dilla baruDHJ02|[1367]|04-SRNP-3168|669[0n]bp
Ouleus dilla baruDHJ02|[1368]|03-SRNP-5456|669[0n]bp
Ouleus dilla baruDHJ02|[1369]|01-SRNP-25074|669[0n]bp
Ouleus dilla baruDHJ02|[1370]|02-SRNP-21428|666[0n]bp
Ouleus dilla baruDHJ02|[1371]|02-SRNP-18307|666[0n]bp
Ouleus dilla baruDHJ02|[1372]|03-SRNP-5228|630[1n]bp
Ouleus dilla baruDHJ02|[1373]|02-SRNP-1148|576[0n]bp
Ouleus dilla baruDHJ02|[1374]|05-SRNP-41777|669[1n]bp
Ouleus dilla baruDHJ02|[1375]|05-SRNP-30721|669[0n]bp
Ouleus dilla baruDHJ02|[1376]|05-SRNP-7449|669[0n]bp
Ouleus dilla baruDHJ02|[1377]|06-SRNP-31341|669[0n]bp
Ouleus dilla baruDHJ02|[1378]|07-SRNP-42528|669[0n]bp
Ouleus dilla baruDHJ02|[1379]|08-SRNP-72306|669[0n]bp
Ouleus dilla baruDHJ02|[1380]|08-SRNP-72304|669[0n]bp
Ouleus dilla baruDHJ02|[1381]|09-SRNP-80444|669[0n]bp
Ouleus Burns01|[1382]|04-SRNP-4356|669[0n]bp
Ouleus Burns01|[1383]|03-SRNP-5719|627[2n]bp
Ouleus Burns01|[1384]|06-SRNP-45127|669[1n]bp
Ouleus Burns01|[1385]|06-SRNP-8586|669[0n]bp
Ouleus Burns01|[1386]|06-SRNP-8585|669[0n]bp
Ouleus negrus|[1387]|04-SRNP-491|669[0n]bp
Ouleus negrus|[1388]|98-SRNP-6834|516[0n]bp
Ouleus negrus|[1389]|02-SRNP-2323|669[0n]bp
Ouleus negrus|[1390]|02-SRNP-5597|576[0n]bp
Ouleus negrus|[1391]|00-SRNP-162|606[0n]bp
Ouleus negrus|[1392]|03-SRNP-34272|645[3n]bp
Ouleus negrus|[1393]|03-SRNP-6700|660[0n]bp
Ouleus negrus|[1394]|00-SRNP-14610|669[1n]bp
Ouleus negrus|[1395]|07-SRNP-2215|669[0n]bp
Ouleus negrus|[1396]|07-SRNP-2470|669[0n]bp
Quadrus cerialisDHJ01|[1397]|08-SRNP-65628|669[0n]bp
Quadrus cerialisDHJ02|[1398]|08-SRNP-70040|669[4n]bp
Quadrus cerialisDHJ03|[1399]|04-SRNP-55879|669[2n]bp
Quadrus cerialisDHJ03|[1400]|04-SRNP-[illegible]

Quadrus cerialisDHJ01|[1397]|08-SRNP-65628|669[0n]bp
Quadrus cerialisDHJ02|[1398]|08-SRNP-70040|669[4n]bp
Quadrus cerialisDHJ03|[1399]|04-SRNP-55879|669[2n]bp
Quadrus cerialisDHJ03|[1400]|04-SRNP-55680|669[0n]bp
Quadrus cerialisDHJ03|[1401]|07-SRNP-65780|669[0n]bp
Quadrus cerialisDHJ03|[1402]|05-SRNP-32312|669[0n]bp
Quadrus cerialisDHJ03|[1403]|05-SRNP-32317|669[0n]bp
Quadrus cerialisDHJ03|[1404]|04-SRNP-49439|669[0n]bp
Quadrus cerialisDHJ03|[1405]|04-SRNP-49987|669[0n]bp
Quadrus cerialisDHJ03|[1406]|04-SRNP-15284|669[0n]bp
Quadrus cerialisDHJ03|[1407]|04-SRNP-49389|669[0n]bp
Quadrus cerialisDHJ03|[1408]|04-SRNP-23614|669[1n]bp
Quadrus cerialisDHJ03|[1409]|07-SRNP-66046|660[0n]bp
Quadrus cerialisDHJ03|[1410]|05-SRNP-31921|627[1n]bp
Quadrus cerialisDHJ03|[1411]|03-SRNP-3074|642[0n]bp
Quadrus cerialisDHJ03|[1412]|08-SRNP-40247|648[1n]bp
Quadrus cerialisDHJ03|[1413]|03-SRNP-3037|657[0n]bp
Quadrus cerialisDHJ03|[1414]|08-SRNP-40381|618[0n]bp
Quadrus cerialisDHJ03|[1415]|08-SRNP-45059|669[0n]bp
Quadrus cerialisDHJ03|[1416]|08-SRNP-1036|669[0n]bp
Quadrus cerialisDHJ03|[1417]|08-SRNP-70469|669[0n]bp
Quadrus cerialisDHJ03|[1418]|08-SRNP-65398|669[0n]bp
Quadrus cerialisDHJ03|[1419]|04-SRNP-49986|669[1n]bp
Quadrus cerialisDHJ03|[1420]|06-SRNP-36670|669[0n]bp
Quadrus cerialisDHJ03|[1421]|06-SRNP-3782|669[0n]bp
Quadrus cerialisDHJ03|[1422]|04-SRNP-14171|669[0n]bp
Quadrus cerialisDHJ03|[1423]|04-SRNP-23615|669[0n]bp
Quadrus cerialisDHJ03|[1424]|04-SRNP-15266|669[0n]bp
Quadrus cerialisDHJ03|[1425]|04-SRNP-56788|669[0n]bp
Quadrus cerialisDHJ03|[1426]|08-SRNP-65058|618[0n]bp
Quadrus cerialisDHJ03|[1427]|08-SRNP-2098|669[0n]bp
Quadrus cerialisDHJ03|[1428]|08-SRNP-71737|669[0n]bp
Quadrus cerialisDHJ03|[1429]|08-SRNP-70012|669[0n]bp
Quadrus cerialisDHJ03|[1430]|08-SRNP-1186|669[0n]bp
Quadrus cerialisDHJ03|[1431]|08-SRNP-72329|669[0n]bp
Quadrus cerialisDHJ03|[1432]|09-SRNP-66024|669[4n]bp
Quadrus cerialisDHJ03|[1433]|09-SRNP-75200|669[2n]bp
Quadrus cerialisDHJ03|[1434]|08-SRNP-45063|669[1n]bp
Quadrus cerialisDHJ03|[1435]|07-SRNP-66047|669[1n]bp
Quadrus cerialisDHJ03|[1436]|05-SRNP-32596|669[0n]bp
Quadrus cerialisDHJ03|[1437]|07-SRNP-42399|660[0n]bp
Quadrus cerialisDHJ03|[1438]|08-SRNP-70496|669[0n]bp
Quadrus cerialisDHJ03|[1439]|08-SRNP-65610|669[2n]bp
Quadrus cerialisDHJ03|[1440]|08-SRNP-72231|669[1n]bp
Quadrus cerialisDHJ03|[1441]|08-SRNP-72232|669[1n]bp
Quadrus cerialisDHJ03|[1442]|09-SRNP-80394|669[1n]bp
Quadrus lugubris|[1443]|06-SRNP-55695|630[0n]bp
Quadrus lugubris|[1444]|06-SRNP-59481|621[1n]bp
Quadrus lugubris|[1445]|06-SRNP-18855|603[1n]bp
Quadrus lugubris|[1446]|06-SRNP-20192|633[1n]bp
Quadrus lugubris|[1447]|03-SRNP-3179|642[0n]bp
Quadrus lugubris|[1448]|06-SRNP-46983|645[0n]bp
Quadrus lugubris|[1449]|06-SRNP-46868|630[0n]bp
Quadrus lugubris|[1450]|06-SRNP-59203|669[0n]bp
Quadrus lugubris|[1451]|06-SRNP-18688|669[0n]bp
Quadrus lugubris|[1452]|08-SRNP-57856|669[0n]bp
Ouleus salvinaDHJ02|[1453]|06-SRNP-59480|669[0n]bp
Ouleus salvinaDHJ02|[1454]|09-SRNP-44396|666[0n]bp
Ouleus salvinaDHJ02|[1455]|09-SRNP-44315|669[0n]bp
Ouleus salvinaDHJ02|[1456]|09-SRNP-68013|669[0n]bp
Ouleus salvinaDHJ02|[1457]|07-SRNP-41960|669[0n]bp
Ouleus salvinaDHJ02|[1458]|09-SRNP-67763|669[0n]bp
Ouleus salvinaDHJ02|[1459]|06-SRNP-42461|669[0n]bp
Ouleus salvinaDHJ02|[1460]|06-SRNP-55683|669[0n]bp
Ouleus salvinaDHJ02|[1461]|04-SRNP-41470|669[0n]bp
Ouleus salvinaDHJ02|[1462]|02-SRNP-14934|570[0n]bp
Ouleus salvinaDHJ02|[1463]|02-SRNP-3943|567[0n]bp
Ouleus salvinaDHJ02|[1464]|95-SRNP-117|591[1n]bp
Ouleus salvinaDHJ02|[1465]|04-SRNP-41611|627[0n]bp
Ouleus salvinaDHJ02|[1466]|05-SRNP-55605|669[0n]bp
Ouleus salvinaDHJ02|[1467]|05-SRNP-55640|669[0n]bp
Ouleus salvinaDHJ02|[1468]|00-SRNP-20778|669[0n]bp
Ouleus salvinaDHJ02|[1469]|06-SRNP-55688|669[0n]bp
Ouleus salvinaDHJ02|[1470]|07-SRNP-312|669[0n]bp
Ouleus salvinaDHJ02|[1471]|07-SRNP-55498|669[0n]bp
Ouleus salvinaDHJ02|[1472]|07-SRNP-55500|669[0n]bp
Ouleus salvinaDHJ02|[1473]|07-SRNP-55682|669[0n]bp
Ouleus salvinaDHJ02|[1474]|07-SRNP-55748|669[0n]bp
Ouleus salvinaDHJ02|[1475]|07-SRNP-55742|669[0n]bp
Ouleus salvinaDHJ02|[1476]|07-SRNP-55810|669[0n]bp
Ouleus salvinaDHJ02|[1477]|07-SRNP-55828|669[0n]bp
Ouleus salvinaDHJ02|[1478]|07-SRNP-55829|669[0n]bp
Ouleus salvinaDHJ02|[1479]|09-SRNP-75766|669[0n]bp
Ouleus salvinaDHJ01|[1480]|06-SRNP-59753|669[0n]bp
Ouleus salvinaDHJ01|[1481]|07-SRNP-56446|669[0n]bp
Ouleus salvinaDHJ01|[1482]|07-SRNP-55582|669[0n]bp
Ouleus salvinaDHJ01|[1483]|07-SRNP-55584|669[0n]bp
Ouleus salvinaDHJ01|[1484]|07-SRNP-55693|660[0n]bp
Ouleus salvinaDHJ01|[1485]|09-SRNP-65468|660[0n]bp
Ouleus salvinaDHJ01|[1486]|07-SRNP-42350|669[0n]bp
Ouleus salvinaDHJ01|[1487]|08-SRNP-70345|669[0n]bp
Ouleus salvinaDHJ01|[1488]|08-SRNP-70346|669[0n]bp
Ouleus salvinaDHJ01|[1489]|09-SRNP-70105|669[0n]bp
Ouleus salvinaDHJ01|[1490]|09-SRNP-68071|669[0n]bp
Ouleus salvinaDHJ01|[1491]|06-SRNP-31160|633[0n]bp
Ouleus salvinaDHJ01|[1492]|06-SRNP-55656|669[0n]bp
Ouleus salvinaDHJ01|[1493]|06-SRNP-55943|669[0n]bp
Ouleus salvinaDHJ01|[1494]|06-SRNP-55655|669[0n]bp
Ouleus salvinaDHJ01|[1495]|09-SRNP-68307|669[0n]bp
Ouleus salvinaDHJ01|[1496]|08-SRNP-55837|669[0n]bp
Ouleus salvinaDHJ01|[1497]|08-SRNP-55820|669[0n]bp
Ouleus salvinaDHJ01|[1498]|05-SRNP-21647|669[0n]bp
Ouleus salvinaDHJ01|[1499]|05-SRNP-55686|669[0n]bp
Ouleus salvinaDHJ01|[1500]|[illegible]|669[0n]bp

Ouleus salvinaDHJ01|[1497]|08-SRNP-55820|669[0n]bp
Ouleus salvinaDHJ01|[1498]|05-SRNP-21647|669[0n]bp
Ouleus salvinaDHJ01|[1499]|05-SRNP-55686|669[0n]bp
Ouleus salvinaDHJ01|[1500]|09-SRNP-67934|669[0n]bp
Ouleus salvinaDHJ01|[1501]|07-SRNP-55569|642[0n]bp
Ouleus salvinaDHJ01|[1502]|02-SRNP-14802|660[0n]bp
Ouleus salvinaDHJ01|[1503]|09-SRNP-75801|633[0n]bp
Ouleus salvinaDHJ01|[1504]|07-SRNP-41975|669[0n]bp
Ouleus salvinaDHJ01|[1505]|06-SRNP-6666|669[0n]bp
Ouleus salvinaDHJ01|[1506]|06-SRNP-42460|669[0n]bp
Ouleus salvinaDHJ01|[1507]|05-SRNP-55394|669[0n]bp
Ouleus salvinaDHJ01|[1508]|05-SRNP-55393|669[0n]bp
Ouleus salvinaDHJ01|[1509]|05-SRNP-55399|669[0n]bp
Ouleus salvinaDHJ01|[1510]|04-SRNP-41462|669[0n]bp
Ouleus salvinaDHJ01|[1511]|04-SRNP-41847|669[0n]bp
Ouleus salvinaDHJ01|[1512]|04-SRNP-31499|630[0n]bp
Ouleus salvinaDHJ01|[1513]|02-SRNP-14703|576[0n]bp
Ouleus salvinaDHJ01|[1514]|02-SRNP-14705|576[0n]bp
Ouleus salvinaDHJ01|[1515]|02-SRNP-4232|591[0n]bp
Ouleus salvinaDHJ01|[1516]|02-SRNP-14805|600[0n]bp
Ouleus salvinaDHJ01|[1517]|03-SRNP-34140|594[0n]bp
Ouleus salvinaDHJ01|[1518]|06-SRNP-31161|627[0n]bp
Ouleus salvinaDHJ01|[1519]|07-SRNP-21111|645[0n]bp
Ouleus salvinaDHJ01|[1520]|07-SRNP-21258|645[0n]bp
Ouleus salvinaDHJ01|[1521]|07-SRNP-65146|669[0n]bp
Ouleus salvinaDHJ01|[1522]|07-SRNP-65555|669[0n]bp
Ouleus salvinaDHJ01|[1523]|08-SRNP-65516|669[0n]bp
Ouleus salvinaDHJ01|[1524]|08-SRNP-2541|669[0n]bp
Ouleus salvinaDHJ01|[1525]|09-SRNP-20454|669[0n]bp
Ouleus salvinaDHJ01|[1526]|09-SRNP-80738|669[0n]bp
Eracon lachesis|[1527]|05-SRNP-32007|669[0n]bp
Eracon lachesis|[1528]|05-SRNP-47074|669[0n]bp
Eracon lachesis|[1529]|05-SRNP-33238|645[0n]bp
Eracon lachesis|[1530]|08-SRNP-40919|669[0n]bp
Eracon lachesis|[1531]|07-SRNP-32478|636[0n]bp
Eracon lachesis|[1532]|04-SRNP-56232|669[0n]bp
Eracon lachesis|[1533]|06-SRNP-65850|669[0n]bp
Eracon lachesis|[1534]|07-SRNP-45762|615[0n]bp
Eracon lachesis|[1535]|09-SRNP-55623|669[0n]bp
Eracon lachesis|[1536]|09-SRNP-31097|669[0n]bp
Eracon lachesis|[1537]|08-SRNP-40965|669[0n]bp
Eracon lachesis|[1538]|08-SRNP-31173|669[0n]bp
Eracon lachesis|[1539]|08-SRNP-40962|669[0n]bp
Eracon lachesis|[1540]|07-SRNP-45763|669[0n]bp
Eracon lachesis|[1541]|07-SRNP-32129|669[0n]bp
Eracon lachesis|[1542]|06-SRNP-31728|669[0n]bp
Eracon lachesis|[1543]|05-SRNP-31900|669[0n]bp
Eracon lachesis|[1544]|05-SRNP-31902|669[0n]bp
Eracon lachesis|[1545]|05-SRNP-47073|669[0n]bp
Eracon lachesis|[1546]|05-SRNP-47071|669[0n]bp
Eracon lachesis|[1547]|05-SRNP-47487|669[0n]bp
Eracon lachesis|[1548]|05-SRNP-3998|669[0n]bp
Eracon lachesis|[1549]|05-SRNP-4680|669[0n]bp
Eracon lachesis|[1550]|05-SRNP-3997|669[0n]bp
Eracon lachesis|[1551]|04-SRNP-55974|669[0n]bp
Eracon lachesis|[1552]|04-SRNP-31099|669[0n]bp
Eracon lachesis|[1553]|05-SRNP-70315|669[1n]bp
Eracon lachesis|[1554]|01-SRNP-1331|576[0n]bp
Eracon lachesis|[1555]|07-SRNP-46421|618[1n]bp
Eracon lachesis|[1556]|09-SRNP-57098|669[0n]bp
Eracon lachesis|[1557]|09-SRNP-58020|669[0n]bp
Eracon lachesis|[1558]|09-SRNP-41521|669[0n]bp
Tosta niger|[1559]|00-SRNP-9034|570[0n]bp
Tosta niger|[1560]|00-SRNP-9020|570[0n]bp
Tosta niger|[1561]|04-SRNP-35560|669[0n]bp
Tosta niger|[1562]|08-SRNP-36395|669[0n]bp
Tosta niger|[1563]|08-SRNP-37594|669[0n]bp
Tosta platypterus|[1564]|01-SRNP-25099|528[0n]bp
Tosta platypterus|[1565]|04-SRNP-60213|630[0n]bp
Tosta platypterus|[1566]|04-SRNP-4462|669[0n]bp
Tosta platypterus|[1567]|06-SRNP-6600|669[0n]bp
Tosta platypterus|[1568]|06-SRNP-2124|669[0n]bp
Tosta platypterus|[1569]|04-SRNP-4497|669[0n]bp
Tosta platypterus|[1570]|04-SRNP-42429|669[0n]bp
Tosta platypterus|[1571]|04-SRNP-43095|669[0n]bp
Tosta platypterus|[1572]|04-SRNP-815|669[0n]bp
Tosta platypterus|[1573]|01-SRNP-3610|591[0n]bp
Tosta platypterus|[1574]|07-SRNP-315|639[0n]bp
Tosta platypterus|[1575]|07-SRNP-41224|669[0n]bp
Tosta platypterus|[1576]|07-SRNP-2877|669[0n]bp
Tosta platypterus|[1577]|08-SRNP-584|669[0n]bp
Tosta platypterus|[1578]|09-SRNP-67348|669[0n]bp
Tosta platypterus|[1579]|04-SRNP-55109|669[0n]bp
Tosta platypterus|[1580]|04-SRNP-55252|669[0n]bp
Tosta platypterus|[1581]|07-SRNP-41435|666[0n]bp
Tosta platypterus|[1582]|07-SRNP-3108|666[0n]bp
Tosta platypterus|[1583]|07-SRNP-45523|669[1n]bp
Tosta platypterus|[1584]|07-SRNP-46134|669[0n]bp
Tosta platypterus|[1585]|07-SRNP-45525|669[0n]bp
Tosta platypterus|[1586]|08-SRNP-70842|669[0n]bp
Tosta platypterus|[1587]|09-SRNP-72364|669[0n]bp
Tosta platypterus|[1588]|09-SRNP-72095|669[0n]bp
Iliana Burns01|[1589]|07-SRNP-32919|669[0n]bp
Iliana Burns01|[1590]|07-SRNP-32830|645[0n]bp
Tosta gorgus|[1591]|05-SRNP-64260|669[0n]bp
Tosta gorgus|[1592]|05-SRNP-25010|669[0n]bp
Tosta gorgus|[1593]|05-SRNP-25011|669[0n]bp
Tosta gorgus|[1594]|04-SRNP-14209|669[0n]bp
Tosta gorgus|[1595]|04-SRNP-16178|669[0n]bp
Tosta gorgus|[1596]|00-SRNP-2772|597[0n]bp
Tosta gorgus|[1597]|00-SRNP-2404|594[0n]bp
Tosta gorgus|[1598]|08-SRNP-22908|639[0n]bp
Cyclosemia Burns01|[1599]|02-SRNP-21532|576[1n]bp
Cyclosemia Burns01|[1600]|02-SRNP-21374|630[0n]bp

Tosta gorgus|[1597]|0[illegible]-SRNP-2404|594[0n]bp
Tosta gorgus|[1598]|08-SRNP-22908|639[0n]bp
Cyclosemia Burns01|[1599]|02-SRNP-21532|576[1n]bp
Cyclosemia Burns01|[1600]|02-SRNP-21274|630[0n]bp
Cyclosemia Burns01|[1601]|08-SRNP-70951|669[0n]bp
Cyclosemia Burns01|[1602]|08-SRNP-40739|669[0n]bp
Cyclosemia Burns01|[1603]|07-SRNP-41474|669[0n]bp
Cyclosemia Burns01|[1604]|07-SRNP-32839|669[0n]bp
Cyclosemia Burns01|[1605]|07-SRNP-41339|669[0n]bp
Cyclosemia Burns01|[1606]|06-SRNP-41379|669[0n]bp
Cyclosemia Burns01|[1607]|05-SRNP-33219|669[0n]bp
Cyclosemia Burns01|[1608]|05-SRNP-32358|669[0n]bp
Cyclosemia Burns01|[1609]|05-SRNP-41022|669[0n]bp
Cyclosemia Burns01|[1610]|04-SRNP-56318|669[0n]bp
Cyclosemia Burns01|[1611]|05-SRNP-3974|669[0n]bp
Cyclosemia Burns01|[1612]|05-SRNP-41706|669[0n]bp
Cyclosemia Burns01|[1613]|04-SRNP-42740|669[0n]bp
Cyclosemia Burns01|[1614]|07-SRNP-21340|669[0n]bp
Cyclosemia Burns01|[1615]|07-SRNP-21976|669[0n]bp
Cyclosemia Burns01|[1616]|06-SRNP-30843|657[0n]bp
Cyclosemia Burns01|[1617]|07-SRNP-65003|645[0n]bp
Cyclosemia Burns01|[1618]|04-SRNP-42705|627[0n]bp
Cyclosemia Burns01|[1619]|04-SRNP-42378|627[0n]bp
Cyclosemia Burns01|[1620]|04-SRNP-41976|627[0n]bp
Cyclosemia Burns01|[1621]|04-SRNP-42926|627[0n]bp
Cyclosemia Burns01|[1622]|04-SRNP-55128|627[0n]bp
Cyclosemia Burns01|[1623]|04-SRNP-30480|627[0n]bp
Cyclosemia Burns01|[1624]|04-SRNP-43086|609[0n]bp
Cyclosemia Burns01|[1625]|04-SRNP-42679|591[0n]bp
Cyclosemia Burns01|[1626]|07-SRNP-3363|639[0n]bp
Cyclosemia Burns01|[1627]|08-SRNP-70207|669[0n]bp
Cyclosemia Burns01|[1628]|08-SRNP-70211|669[0n]bp
Cyclosemia Burns01|[1629]|08-SRNP-40741|669[0n]bp
Cyclosemia Burns01|[1630]|08-SRNP-72009|669[0n]bp
Cyclosemia Burns01|[1631]|09-SRNP-70459|669[0n]bp
Cyclosemia Burns01|[1632]|09-SRNP-67109|669[0n]bp
Cyclosemia Burns01|[1633]|09-SRNP-73946|669[0n]bp
Cyclosemia anastomosis|[1634]|04-SRNP-48002|603[1n]bp
Cyclosemia anastomosis|[1635]|01-SRNP-464|645[0n]bp
Cyclosemia anastomosis|[1636]|01-SRNP-2931|663[0n]bp
Cyclosemia anastomosis|[1637]|05-SRNP-41199|669[0n]bp
Cyclosemia anastomosis|[1638]|08-SRNP-70103|669[0n]bp
Cyclosemia anastomosis|[1639]|08-SRNP-71654|669[0n]bp
Cyclosemia anastomosis|[1640]|07-SRNP-42171|669[0n]bp
Cyclosemia anastomosis|[1641]|07-SRNP-30971|669[0n]bp
Cyclosemia anastomosis|[1642]|05-SRNP-43097|669[0n]bp
Cyclosemia anastomosis|[1643]|05-SRNP-34289|669[0n]bp
Cyclosemia anastomosis|[1644]|05-SRNP-41292|669[0n]bp
Cyclosemia anastomosis|[1645]|05-SRNP-41296|669[0n]bp
Cyclosemia anastomosis|[1646]|05-SRNP-45117|669[0n]bp
Cyclosemia anastomosis|[1647]|04-SRNP-40770|669[0n]bp
Cyclosemia anastomosis|[1648]|05-SRNP-34539|660[0n]bp
Cyclosemia anastomosis|[1649]|07-SRNP-45516|666[0n]bp
Cyclosemia anastomosis|[1650]|07-SRNP-45515|633[0n]bp
Cyclosemia anastomosis|[1651]|07-SRNP-45573|660[0n]bp
Cyclosemia anastomosis|[1652]|06-SRNP-43745|657[0n]bp
Cyclosemia anastomosis|[1653]|06-SRNP-42938|609[0n]bp
Cyclosemia anastomosis|[1654]|06-SRNP-33946|609[0n]bp
Cyclosemia anastomosis|[1655]|07-SRNP-2044|654[0n]bp
Cyclosemia anastomosis|[1656]|07-SRNP-45517|645[0n]bp
Cyclosemia anastomosis|[1657]|07-SRNP-46695|645[0n]bp
Cyclosemia anastomosis|[1658]|08-SRNP-72776|648[0n]bp
Cyclosemia anastomosis|[1659]|05-SRNP-43883|669[0n]bp
Cyclosemia anastomosis|[1660]|09-SRNP-33098|669[0n]bp
Cyclosemia subcaerulea|[1661]|06-SRNP-42573|660[0n]bp
Cyclosemia subcaerulea|[1662]|02-SRNP-6718|657[0n]bp
Cyclosemia subcaerulea|[1663]|02-SRNP-7780|660[0n]bp
Cyclosemia subcaerulea|[1664]|06-SRNP-42489|630[1n]bp
Cyclosemia subcaerulea|[1665]|07-SRNP-65063|660[0n]bp
Cyclosemia subcaerulea|[1666]|09-SRNP-69386|669[0n]bp
Pellicia Janzen01|[1667]|05-SRNP-34546|591[0n]bp
Pellicia Janzen01|[1668]|08-SRNP-2348|666[0n]bp
Pellicia dimidiata|[1669]|06-SRNP-30813|669[0n]bp
Pellicia dimidiata|[1670]|93-SRNP-6528|537[0n]bp
Pellicia dimidiata|[1671]|06-SRNP-4815|669[0n]bp
Pellicia dimidiata|[1672]|06-SRNP-3027|669[0n]bp
Pellicia dimidiata|[1673]|06-SRNP-1886|669[0n]bp
Pellicia dimidiata|[1674]|06-SRNP-2646|669[0n]bp
Pellicia dimidiata|[1675]|06-SRNP-2668|669[0n]bp
Pellicia dimidiata|[1676]|05-SRNP-42242|669[0n]bp
Pellicia dimidiata|[1677]|04-SRNP-46357|669[0n]bp
Pellicia dimidiata|[1678]|02-SRNP-4585|669[0n]bp
Pellicia dimidiata|[1679]|93-SRNP-8674|627[0n]bp
Pellicia dimidiata|[1680]|07-SRNP-45157|663[0n]bp
Pellicia dimidiata|[1681]|07-SRNP-1514|666[0n]bp
Pellicia dimidiata|[1682]|07-SRNP-2217|669[0n]bp
Pellicia dimidiata|[1683]|08-SRNP-2140|669[0n]bp
Pellicia arina|[1684]|04-SRNP-13705|669[0n]bp
Pellicia arina|[1685]|03-SRNP-12773.1|669[1n]bp
Pellicia arina|[1686]|00-SRNP-4592|669[0n]bp
Pellicia arina|[1687]|02-SRNP-32880|669[0n]bp
Pellicia arina|[1688]|04-SRNP-15702|669[0n]bp
Pellicia arina|[1689]|94-SRNP-139|576[0n]bp
Pellicia arina|[1690]|05-SRNP-65245|633[0n]bp
Pellicia arina|[1691]|05-SRNP-61204|627[0n]bp
Pellicia arina|[1692]|05-SRNP-62646|630[0n]bp
Pellicia arina|[1693]|06-SRNP-57936|669[0n]bp
Pellicia arina|[1694]|06-SRNP-19742|669[0n]bp
Pellicia arina|[1695]|07-SRNP-14674|669[0n]bp
Mictris crispus caerula|[1696]|03-SRNP-18922|492[0n]bp
Mictris crispus caerula|[1697]|05-SRNP-4397|669[0n]bp
Mictris crispus caerula|[1698]|05-SRNP-4032|627[0n]bp
Mictris crispus caerula|[1699]|05-SRNP-3008|669[0n]bp
Mictris crispus caerula|[1700]|05-SRNP-34281|588[0n]bp

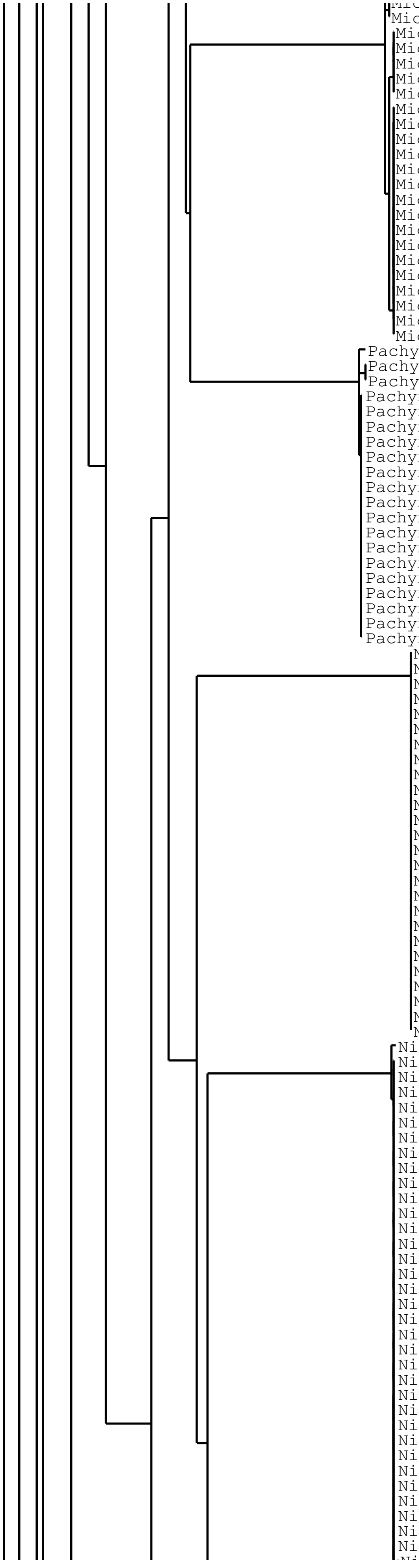

Mictris crispus caerula|[1697]|05-SRNP-4397|669[0n]bp
Mictris crispus caerula|[1698]|05-SRNP-4032|627[0n]bp
Mictris crispus caerula|[1699]|05-SRNP-3008|669[0n]bp
Mictris crispus caerula|[1700]|05-SRNP-34281|588[0n]bp
Mictris crispus caerula|[1701]|07-SRNP-3356|660[0n]bp
Mictris crispus caerula|[1702]|08-SRNP-40396|669[0n]bp
Mictris crispus caerula|[1703]|09-SRNP-71308|669[0n]bp
Mictris crispus caerula|[1704]|07-SRNP-33227|669[0n]bp
Mictris crispus caerula|[1705]|07-SRNP-32363|669[0n]bp
Mictris crispus caerula|[1706]|07-SRNP-31656|669[0n]bp
Mictris crispus caerula|[1707]|07-SRNP-31094|669[0n]bp
Mictris crispus caerula|[1708]|05-SRNP-34280|669[0n]bp
Mictris crispus caerula|[1709]|05-SRNP-2161|669[0n]bp
Mictris crispus caerula|[1710]|04-SRNP-4244|669[0n]bp
Mictris crispus caerula|[1711]|03-SRNP-6065|663[0n]bp
Mictris crispus caerula|[1712]|03-SRNP-9772|663[0n]bp
Mictris crispus caerula|[1713]|05-SRNP-34282|615[1n]bp
Mictris crispus caerula|[1714]|05-SRNP-34208|588[0n]bp
Mictris crispus caerula|[1715]|08-SRNP-195|645[0n]bp
Mictris crispus caerula|[1716]|08-SRNP-196|669[0n]bp
Mictris crispus caerula|[1717]|08-SRNP-31176|669[0n]bp
Mictris crispus caerula|[1718]|08-SRNP-2184|669[0n]bp
Mictris crispus caerula|[1719]|09-SRNP-71399|669[0n]bp
Pachyneuria licisca|[1720]|05-SRNP-58420|669[0n]bp
Pachyneuria licisca|[1721]|03-SRNP-30838|669[0n]bp
Pachyneuria licisca|[1722]|07-SRNP-57201|669[0n]bp
Pachyneuria licisca|[1723]|03-SRNP-27023|618[0n]bp
Pachyneuria licisca|[1724]|03-SRNP-27271|585[0n]bp
Pachyneuria licisca|[1725]|03-SRNP-27670|669[0n]bp
Pachyneuria licisca|[1726]|03-SRNP-30847|669[0n]bp
Pachyneuria licisca|[1727]|03-SRNP-27200|669[0n]bp
Pachyneuria licisca|[1728]|03-SRNP-27033|669[0n]bp
Pachyneuria licisca|[1729]|03-SRNP-27671|669[0n]bp
Pachyneuria licisca|[1730]|03-SRNP-27754|669[0n]bp
Pachyneuria licisca|[1731]|03-SRNP-27025|612[0n]bp
Pachyneuria licisca|[1732]|97-SRNP-5856|558[0n]bp
Pachyneuria licisca|[1733]|02-SRNP-5984|567[0n]bp
Pachyneuria licisca|[1734]|06-SRNP-58286|657[0n]bp
Pachyneuria licisca|[1735]|06-SRNP-58263|669[0n]bp
Pachyneuria licisca|[1736]|06-SRNP-67766|669[0n]bp
Pachyneuria licisca|[1737]|09-SRNP-57271|669[0n]bp
Pachyneuria licisca|[1738]|09-SRNP-57272|669[0n]bp
Pachyneuria licisca|[1739]|09-SRNP-57273|669[0n]bp
Nisoniades castolus|[1740]|05-SRNP-6591|588[2n]bp
Nisoniades castolus|[1741]|06-SRNP-32805|594[0n]bp
Nisoniades castolus|[1742]|05-SRNP-45293|687[1n]bp
Nisoniades castolus|[1743]|05-SRNP-31414|687[0n]bp
Nisoniades castolus|[1744]|05-SRNP-1751|687[0n]bp
Nisoniades castolus|[1745]|07-SRNP-41530|669[0n]bp
Nisoniades castolus|[1746]|05-SRNP-31711|669[0n]bp
Nisoniades castolus|[1747]|04-SRNP-41670|669[0n]bp
Nisoniades castolus|[1748]|04-SRNP-34843|669[0n]bp
Nisoniades castolus|[1749]|04-SRNP-56347|669[0n]bp
Nisoniades castolus|[1750]|04-SRNP-60852|669[0n]bp
Nisoniades castolus|[1751]|05-SRNP-2312|687[1n]bp
Nisoniades castolus|[1752]|04-SRNP-55872|669[1n]bp
Nisoniades castolus|[1753]|05-SRNP-41450|687[2n]bp
Nisoniades castolus|[1754]|05-SRNP-41083|636[1n]bp
Nisoniades castolus|[1755]|06-SRNP-32630|642[1n]bp
Nisoniades castolus|[1756]|07-SRNP-65198|648[0n]bp
Nisoniades castolus|[1757]|07-SRNP-65620|669[0n]bp
Nisoniades castolus|[1758]|08-SRNP-2616|669[0n]bp
Nisoniades castolus|[1759]|08-SRNP-70988|669[0n]bp
Nisoniades castolus|[1760]|08-SRNP-70583|669[0n]bp
Nisoniades castolus|[1761]|08-SRNP-65475|669[0n]bp
Nisoniades castolus|[1762]|08-SRNP-65509|669[0n]bp
Nisoniades castolus|[1763]|08-SRNP-70402|669[0n]bp
Nisoniades castolus|[1764]|08-SRNP-2617|669[0n]bp
Nisoniades castolus|[1765]|09-SRNP-71609|669[0n]bp
Nisoniades godma|[1766]|07-SRNP-21783|669[0n]bp
Nisoniades godma|[1767]|08-SRNP-2515|669[0n]bp
Nisoniades godma|[1768]|09-SRNP-67771|669[0n]bp
Nisoniades godma|[1769]|08-SRNP-35368|669[0n]bp
Nisoniades godma|[1770]|08-SRNP-20591|669[0n]bp
Nisoniades godma|[1771]|08-SRNP-70907|669[0n]bp
Nisoniades godma|[1772]|08-SRNP-35367|669[0n]bp
Nisoniades godma|[1773]|07-SRNP-41906|669[0n]bp
Nisoniades godma|[1774]|07-SRNP-22159|669[0n]bp
Nisoniades godma|[1775]|07-SRNP-2603|669[0n]bp
Nisoniades godma|[1776]|07-SRNP-21785|669[0n]bp
Nisoniades godma|[1777]|07-SRNP-2675|669[0n]bp
Nisoniades godma|[1778]|07-SRNP-3043|669[0n]bp
Nisoniades godma|[1779]|06-SRNP-60085|669[0n]bp
Nisoniades godma|[1780]|06-SRNP-60084|669[0n]bp
Nisoniades godma|[1781]|98-SRNP-4340|669[0n]bp
Nisoniades godma|[1782]|07-SRNP-35261|669[0n]bp
Nisoniades godma|[1783]|98-SRNP-4343|669[0n]bp
Nisoniades godma|[1784]|98-SRNP-4337|669[0n]bp
Nisoniades godma|[1785]|05-SRNP-30669|669[0n]bp
Nisoniades godma|[1786]|98-SRNP-4617|669[0n]bp
Nisoniades godma|[1787]|07-SRNP-65813|669[0n]bp
Nisoniades godma|[1788]|07-SRNP-65579|669[0n]bp
Nisoniades godma|[1789]|07-SRNP-55229|669[0n]bp
Nisoniades godma|[1790]|07-SRNP-55227|669[0n]bp
Nisoniades godma|[1791]|02-SRNP-5568|669[0n]bp
Nisoniades godma|[1792]|02-SRNP-5569|669[0n]bp
Nisoniades godma|[1793]|02-SRNP-6253|669[0n]bp
Nisoniades godma|[1794]|02-SRNP-7146|669[0n]bp
Nisoniades godma|[1795]|02-SRNP-5582|669[0n]bp
Nisoniades godma|[1796]|02-SRNP-5571|669[0n]bp
Nisoniades godma|[1797]|04-SRNP-47998|669[0n]bp
Nisoniades godma|[1798]|04-SRNP-47987|669[0n]bp
Nisoniades godma|[1799]|04-SRNP-23805|669[0n]bp
Nisoniades godma|[1800]|07-SRNP-55730|663[0n]bp

Nisoniades godma|[1797]|04-SRNP-47998|669[0n]bp
Nisoniades godma|[1798]|04-SRNP-47987|669[0n]bp
Nisoniades godma|[1799]|04-SRNP-23805|669[0n]bp
Nisoniades godma|[1800]|07-SRNP-55730|663[0n]bp
Nisoniades godma|[1801]|07-SRNP-21248|660[0n]bp
Nisoniades godma|[1802]|05-SRNP-34138|630[0n]bp
Nisoniades godma|[1803]|02-SRNP-5578|669[1n]bp
Nisoniades godma|[1804]|03-SRNP-1412|657[0n]bp
Nisoniades godma|[1805]|02-SRNP-18975|603[1n]bp
Nisoniades godma|[1806]|06-SRNP-31175|627[0n]bp
Nisoniades godma|[1807]|05-SRNP-59705|633[0n]bp
Nisoniades godma|[1808]|08-SRNP-72371|408[0n]bp
Nisoniades godma|[1809]|09-SRNP-67700|669[0n]bp
Nisoniades godma|[1810]|09-SRNP-75599|669[0n]bp
Nisoniades godma|[1811]|09-SRNP-75202|669[0n]bp
Nisoniades Burns02|[1812]|05-SRNP-24032|669[0n]bp
Nisoniades Burns02|[1813]|01-SRNP-14613|669[0n]bp
Nisoniades tortaDHJ01|[1814]|08-SRNP-2752|669[0n]bp
Nisoniades tortaDHJ02|[1815]|08-SRNP-5186|669[0n]bp
Nisoniades tortaDHJ02|[1816]|06-SRNP-659|669[0n]bp
Nisoniades tortaDHJ02|[1817]|09-SRNP-3730|669[0n]bp
Nisoniades rubescensDHJ01|[1818]|01-SRNP-16913|669[0n]bp
Nisoniades rubescensDHJ02|[1819]|99-SRNP-2497|609[0n]bp
Nisoniades rubescensDHJ02|[1820]|99-SRNP-2290|669[0n]bp
Nisoniades rubescensDHJ02|[1821]|01-SRNP-9028|669[0n]bp
Nisoniades rubescensDHJ02|[1822]|06-SRNP-4756|669[0n]bp
Nisoniades rubescensDHJ02|[1823]|05-SRNP-41322|669[0n]bp
Nisoniades rubescensDHJ02|[1824]|04-SRNP-22441|669[0n]bp
Nisoniades rubescensDHJ02|[1825]|04-SRNP-48521|669[0n]bp
Nisoniades rubescensDHJ02|[1826]|04-SRNP-22824|669[0n]bp
Nisoniades rubescensDHJ02|[1827]|05-SRNP-61949|630[0n]bp
Nisoniades rubescensDHJ02|[1828]|08-SRNP-65094|645[0n]bp
Nisoniades rubescensDHJ02|[1829]|09-SRNP-75982|669[0n]bp
Polyctor enops|[1830]|06-SRNP-23424|525[6n]bp
Polyctor enops|[1831]|07-SRNP-20052|669[0n]bp
Polyctor enops|[1832]|06-SRNP-23355|669[0n]bp
Polyctor enops|[1833]|06-SRNP-23356|669[0n]bp
Polyctor enops|[1834]|06-SRNP-23354|669[0n]bp
Polyctor enops|[1835]|05-SRNP-24534|669[0n]bp
Polyctor enops|[1836]|05-SRNP-24533|669[0n]bp
Polyctor enops|[1837]|05-SRNP-24532|633[0n]bp
Polyctor enops|[1838]|07-SRNP-23555|669[0n]bp
Polyctor enops|[1839]|07-SRNP-23554|669[0n]bp
Polyctor enops|[1840]|07-SRNP-23640|669[0n]bp
Polyctor enops|[1841]|07-SRNP-23639|669[0n]bp
Polyctor enops|[1842]|07-SRNP-22844|669[0n]bp
Polyctor enops|[1843]|07-SRNP-23549|669[0n]bp
Polyctor enops|[1844]|07-SRNP-22845|669[0n]bp
Polyctor enops|[1845]|07-SRNP-23636|669[0n]bp
Polyctor enops|[1846]|07-SRNP-23542|669[0n]bp
Polyctor enops|[1847]|07-SRNP-23544|669[0n]bp
Polyctor enops|[1848]|07-SRNP-23548|669[0n]bp
Polyctor enops|[1849]|07-SRNP-23551|669[0n]bp
Polyctor enops|[1850]|07-SRNP-23553|669[0n]bp
Polyctor enops|[1851]|07-SRNP-23552|666[0n]bp
Polyctor enops|[1852]|07-SRNP-23550|657[0n]bp
Polyctor enops|[1853]|02-SRNP-30383|657[0n]bp
Polyctor enops|[1854]|02-SRNP-30386|657[0n]bp
Polyctor enops|[1855]|07-SRNP-23545|642[0n]bp
Polyctor enops|[1856]|09-SRNP-23331|669[0n]bp
Polyctor polyctor|[1857]|09-SRNP-20156|333[0n]bp
Polyctor polyctor|[1858]|03-SRNP-21493|519[0n]bp
Polyctor polyctor|[1859]|03-SRNP-29770|522[0n]bp
Polyctor cleta|[1860]|04-SRNP-16175|669[0n]bp
Polyctor cleta|[1861]|04-SRNP-15360|669[0n]bp
Polyctor cleta|[1862]|04-SRNP-15455|669[0n]bp
Polyctor cleta|[1863]|04-SRNP-16111|669[0n]bp
Polyctor cleta|[1864]|95-SRNP-9502|642[0n]bp
Polyctor cleta|[1865]|03-SRNP-37549|642[0n]bp
Polyctor cleta|[1866]|03-SRNP-30883|642[0n]bp
Polyctor cleta|[1867]|98-SRNP-4183|525[0n]bp
Polyctor cleta|[1868]|00-SRNP-20253|627[0n]bp
Polyctor cleta|[1869]|00-SRNP-2150|627[0n]bp
Polyctor cleta|[1870]|03-SRNP-30880|627[0n]bp
Polyctor cleta|[1871]|03-SRNP-30882|627[0n]bp
Polyctor cleta|[1872]|02-SRNP-32285|627[0n]bp
Polyctor cleta|[1873]|02-SRNP-31414|627[0n]bp
Polyctor cleta|[1874]|03-SRNP-37550|627[0n]bp
Polyctor cleta|[1875]|93-SRNP-5853|627[2n]bp
Polyctor cleta|[1876]|95-SRNP-9500|627[0n]bp
Polyctor cleta|[1877]|05-SRNP-61212|627[0n]bp
Polyctor polyctor|[1878]|02-SRNP-27779|630[0n]bp
Polyctor cleta|[1879]|92-SRNP-4986|606[0n]bp
Polyctor polyctor|[1880]|05-SRNP-41712|669[0n]bp
Polyctor polyctor|[1881]|07-SRNP-21961|669[0n]bp
Polyctor polyctor|[1882]|03-SRNP-29891|615[0n]bp
Polyctor polyctor|[1883]|00-SRNP-14180|618[0n]bp
Polyctor polyctor|[1884]|03-SRNP-20449|627[0n]bp
Polyctor polyctor|[1885]|02-SRNP-7128|627[0n]bp
Polyctor polyctor|[1886]|80-SRNP-364|585[1n]bp
Polyctor polyctor|[1887]|05-SRNP-42248|669[1n]bp
Polyctor polyctor|[1888]|03-SRNP-21456|591[2n]bp
Polyctor polyctor|[1889]|05-SRNP-6247|666[1n]bp
Polyctor polyctor|[1890]|05-SRNP-1141|627[2n]bp
Polyctor polyctor|[1891]|05-SRNP-33391|633[0n]bp
Polyctor polyctor|[1892]|03-SRNP-9639|630[0n]bp
Polyctor polyctor|[1893]|03-SRNP-20028|636[0n]bp
Polyctor polyctor|[1894]|05-SRNP-41517|687[0n]bp
Polyctor polyctor|[1895]|08-SRNP-4909|669[0n]bp
Polyctor polyctor|[1896]|08-SRNP-4993|669[0n]bp
Polyctor polyctor|[1897]|08-SRNP-4991|669[0n]bp
Polyctor polyctor|[1898]|08-SRNP-5183|669[0n]bp
Polyctor polyctor|[1899]|08-SRNP-5182|669[0n]bp
Polyctor polyctor|[1900]|08-SRNP-5181|669[0n]bp

Polyctor polyctor|[1897]|08-SRNP-[illegible]|669[0n]bp
Polyctor polyctor|[1898]|08-SRNP-5183|669[0n]bp
Polyctor polyctor|[1899]|08-SRNP-5182|669[0n]bp
Polyctor polyctor|[1900]|08-SRNP-5181|669[0n]bp
Polyctor polyctor|[1901]|08-SRNP-5273|669[0n]bp
Polyctor polyctor|[1902]|08-SRNP-4906|669[0n]bp
Polyctor polyctor|[1903]|08-SRNP-4903|669[0n]bp
Polyctor polyctor|[1904]|08-SRNP-4905|669[0n]bp
Polyctor polyctor|[1905]|08-SRNP-4902|669[0n]bp
Polyctor polyctor|[1906]|08-SRNP-4904|669[0n]bp
Polyctor polyctor|[1907]|08-SRNP-4996|669[0n]bp
Polyctor polyctor|[1908]|08-SRNP-20490|669[0n]bp
Polyctor polyctor|[1909]|07-SRNP-2990|669[0n]bp
Polyctor polyctor|[1910]|07-SRNP-21952|669[0n]bp
Polyctor polyctor|[1911]|07-SRNP-21283|669[0n]bp
Polyctor polyctor|[1912]|06-SRNP-31806|669[0n]bp
Polyctor polyctor|[1913]|06-SRNP-31800|669[0n]bp
Polyctor polyctor|[1914]|05-SRNP-41516|669[0n]bp
Polyctor polyctor|[1915]|05-SRNP-42247|669[0n]bp
Polyctor polyctor|[1916]|04-SRNP-21386|669[0n]bp
Polyctor polyctor|[1917]|04-SRNP-61064|669[0n]bp
Polyctor polyctor|[1918]|04-SRNP-21385|669[0n]bp
Polyctor polyctor|[1919]|04-SRNP-23706|669[0n]bp
Polyctor polyctor|[1920]|04-SRNP-61026|669[0n]bp
Polyctor polyctor|[1921]|04-SRNP-60859|669[0n]bp
Polyctor polyctor|[1922]|04-SRNP-60861|669[0n]bp
Polyctor polyctor|[1923]|04-SRNP-14595|669[0n]bp
Polyctor polyctor|[1924]|04-SRNP-42603|669[0n]bp
Polyctor polyctor|[1925]|04-SRNP-61027|669[0n]bp
Polyctor polyctor|[1926]|04-SRNP-14594|669[0n]bp
Polyctor polyctor|[1927]|03-SRNP-9643|591[0n]bp
Polyctor polyctor|[1928]|03-SRNP-9706|591[0n]bp
Polyctor polyctor|[1929]|02-SRNP-7127|642[0n]bp
Polyctor polyctor|[1930]|03-SRNP-9701|642[0n]bp
Polyctor polyctor|[1931]|03-SRNP-9641|642[0n]bp
Polyctor polyctor|[1932]|95-SRNP-9527|642[0n]bp
Polyctor polyctor|[1933]|02-SRNP-31973|642[0n]bp
Polyctor polyctor|[1934]|07-SRNP-24016|654[0n]bp
Polyctor polyctor|[1935]|07-SRNP-20959|657[0n]bp
Polyctor polyctor|[1936]|08-SRNP-355|648[0n]bp
Polyctor polyctor|[1937]|08-SRNP-356|651[0n]bp
Polyctor polyctor|[1938]|08-SRNP-358|660[0n]bp
Polyctor polyctor|[1939]|02-SRNP-7132|645[0n]bp
Polyctor polyctor|[1940]|04-SRNP-61128|648[0n]bp
Polyctor polyctor|[1941]|04-SRNP-56096|645[0n]bp
Polyctor polyctor|[1942]|05-SRNP-34721|633[0n]bp
Polyctor polyctor|[1943]|08-SRNP-350|645[0n]bp
Polyctor polyctor|[1944]|08-SRNP-499|645[0n]bp
Polyctor polyctor|[1945]|08-SRNP-501|645[0n]bp
Polyctor polyctor|[1946]|08-SRNP-500|645[0n]bp
Polyctor polyctor|[1947]|08-SRNP-359|645[0n]bp
Polyctor polyctor|[1948]|08-SRNP-22736|645[0n]bp
Polyctor polyctor|[1949]|09-SRNP-20479|669[0n]bp
Polyctor polyctor|[1950]|09-SRNP-20478|669[0n]bp
Polyctor polyctor|[1951]|09-SRNP-20485|669[0n]bp
Polyctor polyctor|[1952]|09-SRNP-56143|669[0n]bp
Polyctor polyctor|[1953]|09-SRNP-20724|669[0n]bp
Polyctor polyctor|[1954]|08-SRNP-22527|669[0n]bp
Polyctor polyctor|[1955]|07-SRNP-20902|654[0n]bp
Polyctor polyctor|[1956]|08-SRNP-360|651[0n]bp
Polyctor polyctor|[1957]|08-SRNP-353|648[1n]bp
Polyctor polyctor|[1958]|09-SRNP-20298|420[0n]bp
Polyctor polyctor|[1959]|09-SRNP-23649|636[0n]bp
Polyctor cleta|[1960]|09-SRNP-12336|669[0n]bp
Noctuana lactifera|[1961]|02-SRNP-24244|642[0n]bp
Noctuana lactifera|[1962]|02-SRNP-23370|630[0n]bp
Noctuana stator|[1963]|04-SRNP-2409|627[0n]bp
Noctuana stator|[1964]|04-SRNP-24455|669[0n]bp
Noctuana stator|[1965]|04-SRNP-24257|669[0n]bp
Noctuana stator|[1966]|06-SRNP-60240|669[0n]bp
Noctuana stator|[1967]|01-SRNP-2693|669[1n]bp
Noctuana stator|[1968]|07-SRNP-2653|669[0n]bp
Noctuana stator|[1969]|07-SRNP-2654|669[0n]bp
Noctuana stator|[1970]|07-SRNP-31904|669[0n]bp
Noctuana stator|[1971]|07-SRNP-736|669[0n]bp
Noctuana stator|[1972]|07-SRNP-735|669[0n]bp
Noctuana stator|[1973]|06-SRNP-7267|669[0n]bp
Noctuana stator|[1974]|06-SRNP-6258|669[0n]bp
Noctuana stator|[1975]|06-SRNP-43409|669[0n]bp
Noctuana stator|[1976]|05-SRNP-4577|669[0n]bp
Noctuana stator|[1977]|05-SRNP-3987|669[0n]bp
Noctuana stator|[1978]|05-SRNP-32434|669[0n]bp
Noctuana stator|[1979]|01-SRNP-2492|669[0n]bp
Noctuana stator|[1980]|01-SRNP-2692|669[0n]bp
Noctuana stator|[1981]|01-SRNP-2691|669[0n]bp
Noctuana stator|[1982]|02-SRNP-6661|669[0n]bp
Noctuana stator|[1983]|02-SRNP-7532|669[0n]bp
Noctuana stator|[1984]|02-SRNP-17990|669[0n]bp
Noctuana stator|[1985]|04-SRNP-3780|669[0n]bp
Noctuana stator|[1986]|04-SRNP-2095|669[0n]bp
Noctuana stator|[1987]|05-SRNP-5563|669[1n]bp
Noctuana stator|[1988]|01-SRNP-2493|669[0n]bp
Noctuana stator|[1989]|05-SRNP-453|669[0n]bp
Noctuana stator|[1990]|02-SRNP-17989|645[0n]bp
Noctuana stator|[1991]|01-SRNP-2491|624[0n]bp
Noctuana stator|[1992]|04-SRNP-41356|624[0n]bp
Noctuana stator|[1993]|07-SRNP-24112|591[3n]bp
Noctuana stator|[1994]|08-SRNP-4026|669[0n]bp
Noctuana stator|[1995]|08-SRNP-4027|669[0n]bp
Noctuana stator|[1996]|08-SRNP-4533|669[0n]bp
Staphylus evemerus|[1997]|00-SRNP-10647|558[0n]bp
Staphylus evemerus|[1998]|99-SRNP-740|633[0n]bp
Staphylus evemerus|[1999]|00-SRNP-10651|669[0n]bp
[illegible]

Staphylus evemerus|[1997]|00-SRNP-10647|558[0n]bp
Staphylus evemerus|[1998]|99-SRNP-740|633[0n]bp
Staphylus evemerus|[1999]|00-SRNP-10651|669[0n]bp
Staphylus evemerus|[2000]|00-SRNP-10765|669[0n]bp
Staphylus evemerus|[2001]|00-SRNP-10414|669[0n]bp
Staphylus evemerus|[2002]|00-SRNP-10763|669[0n]bp
Staphylus evemerus|[2003]|00-SRNP-10413|669[0n]bp
Staphylus evemerus|[2004]|03-SRNP-23252|669[0n]bp
Staphylus evemerus|[2005]|05-SRNP-35826|669[0n]bp
Staphylus evemerus|[2006]|04-SRNP-35967|669[0n]bp
Staphylus evemerus|[2007]|00-SRNP-10644|594[0n]bp
Staphylus evemerus|[2008]|00-SRNP-10648|594[0n]bp
Staphylus evemerus|[2009]|00-SRNP-10645|342[0n]bp
Staphylus evemerus|[2010]|00-SRNP-10654|324[0n]bp
Staphylus evemerus|[2011]|02-SRNP-23336|669[0n]bp
Staphylus evemerus|[2012]|02-SRNP-23337|669[0n]bp
Staphylus evemerus|[2013]|06-SRNP-35702|669[0n]bp
Staphylus evemerus|[2014]|07-SRNP-36178|669[0n]bp
Bolla zorillaDHJ11|[2015]|05-SRNP-5923|669[0n]bp
Bolla zorillaDHJ11|[2016]|03-SRNP-6884|669[0n]bp
Bolla zorillaDHJ11|[2017]|06-SRNP-7810|669[0n]bp
Bolla zorillaDHJ11|[2018]|06-SRNP-7811|669[0n]bp
Bolla zorillaDHJ09|[2019]|05-SRNP-7192|669[1n]bp
Bolla zorillaDHJ09|[2020]|05-SRNP-5451|669[0n]bp
Bolla zorillaDHJ09|[2021]|05-SRNP-7193|669[0n]bp
Bolla zorillaDHJ09|[2022]|05-SRNP-7191|669[0n]bp
Bolla zorillaDHJ09|[2023]|06-SRNP-1939|669[0n]bp
Bolla zorillaDHJ09|[2024]|06-SRNP-9445|669[0n]bp
Bolla zorillaDHJ13|[2025]|98-SRNP-3082|669[0n]bp
Bolla zorillaDHJ13|[2026]|04-SRNP-3961|669[0n]bp
Bolla zorillaDHJ13|[2027]|98-SRNP-3081|651[2n]bp
Bolla zorillaDHJ13|[2028]|06-SRNP-7350|669[0n]bp
Bolla zorillaDHJ02|[2029]|04-SRNP-3517|669[0n]bp
Bolla zorillaDHJ02|[2030]|05-SRNP-546|651[0n]bp
Bolla zorillaDHJ02|[2031]|05-SRNP-7189|669[0n]bp
Bolla zorillaDHJ02|[2032]|05-SRNP-7190|669[0n]bp
Bolla zorillaDHJ02|[2033]|02-SRNP-3822|669[0n]bp
Bolla zorillaDHJ02|[2034]|04-SRNP-3614|669[0n]bp
Bolla zorillaDHJ02|[2035]|06-SRNP-9042|669[0n]bp
Bolla zorillaDHJ02|[2036]|07-SRNP-1168|669[0n]bp
Bolla zorillaDHJ02|[2037]|07-SRNP-611|669[0n]bp
Bolla zorillaDHJ02|[2038]|05-SRNP-5922|669[0n]bp
Bolla zorillaDHJ02|[2039]|05-SRNP-7218|669[0n]bp
Bolla zorillaDHJ02|[2040]|06-SRNP-2051|669[0n]bp
Bolla zorillaDHJ02|[2041]|06-SRNP-4375|669[0n]bp
Bolla zorillaDHJ02|[2042]|06-SRNP-3931|669[0n]bp
Bolla zorillaDHJ02|[2043]|06-SRNP-6672|669[0n]bp
Bolla zorillaDHJ02|[2044]|06-SRNP-6230|669[0n]bp
Bolla zorillaDHJ02|[2045]|06-SRNP-6674|669[0n]bp
Bolla zorillaDHJ02|[2046]|06-SRNP-7405|669[0n]bp
Bolla zorillaDHJ02|[2047]|07-SRNP-2467|669[0n]bp
Arteurotia tractipennis|[2048]|95-SRNP-11460|528[0n]bp
Arteurotia tractipennis|[2049]|06-SRNP-1612|669[0n]bp
Arteurotia tractipennis|[2050]|95-SRNP-11459|639[1n]bp
Arteurotia tractipennis|[2051]|04-SRNP-32635|630[0n]bp
Arteurotia tractipennis|[2052]|04-SRNP-32574|630[0n]bp
Arteurotia tractipennis|[2053]|96-SRNP-1080|606[3n]bp
Arteurotia tractipennis|[2054]|00-SRNP-2356|615[3n]bp
Arteurotia tractipennis|[2055]|00-SRNP-2402|618[0n]bp
Arteurotia tractipennis|[2056]|00-SRNP-2258|681[0n]bp
Arteurotia tractipennis|[2057]|96-SRNP-1001|612[5n]bp
Arteurotia tractipennis|[2058]|05-SRNP-30659|594[1n]bp
Arteurotia tractipennis|[2059]|06-SRNP-57518|648[0n]bp
Bolla evippe|[2060]|09-SRNP-57008|669[0n]bp
Staphylus Janzen03|[2061]|04-SRNP-47281|669[0n]bp
Staphylus Janzen08|[2062]|05-SRNP-7212|669[7n]bp
Staphylus Janzen08|[2063]|06-SRNP-1211|669[0n]bp
Staphylus Janzen08|[2064]|06-SRNP-9509|669[0n]bp
Staphylus ascalaphus|[2065]|99-SRNP-3147|669[0n]bp
Staphylus ascalaphus|[2066]|00-SRNP-20227|669[0n]bp
Staphylus ascalaphus|[2067]|08-SRNP-55731|669[2n]bp
Staphylus ascalaphus|[2068]|05-SRNP-55703|687[0n]bp
Staphylus ascalaphus|[2069]|00-SRNP-6524|669[1n]bp
Staphylus ascalaphus|[2070]|05-SRNP-22540|669[0n]bp
Staphylus ascalaphus|[2071]|05-SRNP-55693|669[0n]bp
Staphylus ascalaphus|[2072]|06-SRNP-7407|669[0n]bp
Staphylus ascalaphus|[2073]|07-SRNP-55673|669[0n]bp
Staphylus ascalaphus|[2074]|06-SRNP-19545|669[0n]bp
Staphylus ascalaphus|[2075]|08-SRNP-55942|669[0n]bp
Staphylus ascalaphus|[2076]|08-SRNP-55778|669[0n]bp
Staphylus ascalaphus|[2077]|08-SRNP-55888|669[0n]bp
Staphylus ascalaphus|[2078]|05-SRNP-55700|669[1n]bp
Staphylus ascalaphus|[2079]|07-SRNP-55652|663[0n]bp
Staphylus ascalaphus|[2080]|05-SRNP-55699|669[0n]bp
Staphylus ascalaphus|[2081]|05-SRNP-55697|669[0n]bp
Staphylus ascalaphus|[2082]|06-SRNP-9159|669[0n]bp
Staphylus ascalaphus|[2083]|06-SRNP-8678|669[0n]bp
Staphylus ascalaphus|[2084]|07-SRNP-55651|669[0n]bp
Staphylus ascalaphus|[2085]|07-SRNP-55830|669[0n]bp
Staphylus ascalaphus|[2086]|08-SRNP-55975|669[0n]bp
Staphylus Janzen10|[2087]|05-SRNP-55789|669[0n]bp
Staphylus vulgata|[2088]|04-SRNP-45556|669[0n]bp
Staphylus vulgata|[2089]|03-SRNP-28632|669[0n]bp
Staphylus vulgata|[2090]|04-SRNP-20675|669[0n]bp
Staphylus vulgata|[2091]|04-SRNP-20668|669[0n]bp
Staphylus vulgata|[2092]|04-SRNP-22975|669[0n]bp
Staphylus vulgata|[2093]|05-SRNP-45426|669[0n]bp
Staphylus vulgata|[2094]|02-SRNP-15565|669[0n]bp
Staphylus vulgata|[2095]|02-SRNP-5534|669[0n]bp
Staphylus vulgata|[2096]|04-SRNP-20840|669[0n]bp
Staphylus vulgata|[2097]|02-SRNP-5685|669[0n]bp
Staphylus vulgata|[2098]|02-SRNP-5693|669[0n]bp
Staphylus vulgata|[2099]|02-SRNP-5530|669[0n]bp
Staphylus vulgata|[2100]|02-SRNP-5689|669[0n]bp

Staphylus vulgata|[2097]|02-SRNP-5685|669[0n]bp
Staphylus vulgata|[2098]|02-SRNP-5693|669[0n]bp
Staphylus vulgata|[2099]|02-SRNP-5530|669[0n]bp
Staphylus vulgata|[2100]|02-SRNP-5689|669[0n]bp
Staphylus vulgata|[2101]|02-SRNP-5535|669[0n]bp
Staphylus vulgata|[2102]|06-SRNP-22894|669[0n]bp
Staphylus vulgata|[2103]|07-SRNP-55674|669[0n]bp
Staphylus vulgata|[2104]|04-SRNP-22011|669[0n]bp
Staphylus vulgata|[2105]|07-SRNP-55812|669[1n]bp
Staphylus vulgata|[2106]|06-SRNP-58302|669[0n]bp
Staphylus vulgata|[2107]|06-SRNP-22880|669[0n]bp
Staphylus vulgata|[2108]|04-SRNP-45022|669[0n]bp
Staphylus vulgata|[2109]|03-SRNP-29100|669[0n]bp
Staphylus vulgata|[2110]|02-SRNP-5684|669[0n]bp
Staphylus vulgata|[2111]|02-SRNP-5692|669[0n]bp
Staphylus vulgata|[2112]|04-SRNP-45563|669[0n]bp
Staphylus vulgata|[2113]|04-SRNP-45512|669[0n]bp
Staphylus vulgata|[2114]|04-SRNP-45548|669[0n]bp
Staphylus vulgata|[2115]|04-SRNP-45554|669[0n]bp
Staphylus vulgata|[2116]|05-SRNP-55314|669[0n]bp
Staphylus vulgata|[2117]|05-SRNP-55698|669[0n]bp
Staphylus vulgata|[2118]|05-SRNP-55849|669[0n]bp
Staphylus vulgata|[2119]|05-SRNP-57366|669[0n]bp
Staphylus vulgata|[2120]|05-SRNP-55705|669[0n]bp
Staphylus vulgata|[2121]|05-SRNP-59885|669[0n]bp
Staphylus vulgata|[2122]|05-SRNP-46990|669[0n]bp
Staphylus vulgata|[2123]|05-SRNP-59424|669[0n]bp
Staphylus vulgata|[2124]|04-SRNP-45189|669[0n]bp
Staphylus vulgata|[2125]|04-SRNP-23419|669[0n]bp
Staphylus vulgata|[2126]|04-SRNP-48166|669[0n]bp
Staphylus vulgata|[2127]|04-SRNP-22233|627[0n]bp
Staphylus vulgata|[2128]|04-SRNP-45224|627[0n]bp
Staphylus vulgata|[2129]|04-SRNP-45552|618[0n]bp
Staphylus vulgata|[2130]|98-SRNP-5965|591[0n]bp
Staphylus vulgata|[2131]|02-SRNP-5690|324[0n]bp
Staphylus vulgata|[2132]|98-SRNP-4751|594[1n]bp
Staphylus vulgata|[2133]|06-SRNP-46873|648[1n]bp
Staphylus vulgata|[2134]|06-SRNP-58287|624[0n]bp
Staphylus vulgata|[2135]|07-SRNP-55628|669[0n]bp
Staphylus vulgata|[2136]|07-SRNP-55813|669[0n]bp
Staphylus vulgata|[2137]|07-SRNP-55675|669[0n]bp
Staphylus vulgata|[2138]|07-SRNP-55822|669[0n]bp
Staphylus vulgata|[2139]|07-SRNP-23532|669[0n]bp
Staphylus vulgata|[2140]|07-SRNP-65029|669[0n]bp
Staphylus vulgata|[2141]|08-SRNP-55775|669[0n]bp
Staphylus vulgata|[2142]|03-SRNP-1698|669[0n]bp
Staphylus vulgata|[2143]|04-SRNP-22010|669[0n]bp
Staphylus vulgata|[2144]|04-SRNP-45540|669[0n]bp
Staphylus vulgata|[2145]|05-SRNP-24781|669[0n]bp
Staphylus vulgata|[2146]|04-SRNP-20669|669[0n]bp
Staphylus vulgata|[2147]|05-SRNP-20931|636[0n]bp
Staphylus vulgata|[2148]|02-SRNP-5681|669[2n]bp
Staphylus vulgata|[2149]|03-SRNP-1969|669[0n]bp
Staphylus vulgata|[2150]|02-SRNP-15215|669[0n]bp
Staphylus vulgata|[2151]|02-SRNP-5917|669[0n]bp
Staphylus vulgata|[2152]|04-SRNP-20841|669[0n]bp
Staphylus vulgata|[2153]|03-SRNP-28724|669[0n]bp
Staphylus vulgata|[2154]|02-SRNP-14335|669[0n]bp
Staphylus vulgata|[2155]|06-SRNP-57164|669[0n]bp
Staphylus vulgata|[2156]|06-SRNP-22103|669[0n]bp
Staphylus vulgata|[2157]|06-SRNP-22892|669[0n]bp
Staphylus vulgata|[2158]|07-SRNP-55155|669[0n]bp
Staphylus vulgata|[2159]|08-SRNP-65738|669[0n]bp
Staphylus vulgata|[2160]|08-SRNP-24000|669[0n]bp
Staphylus azteca|[2161]|00-SRNP-6527|669[5n]bp
Staphylus azteca|[2162]|05-SRNP-55694|669[2n]bp
Staphylus azteca|[2163]|00-SRNP-6505|609[4n]bp
Staphylus azteca|[2164]|02-SRNP-17510|669[1n]bp
Staphylus azteca|[2165]|02-SRNP-5531|669[1n]bp
Staphylus azteca|[2166]|98-SRNP-5974|669[0n]bp
Staphylus azteca|[2167]|00-SRNP-6532|669[0n]bp
Staphylus azteca|[2168]|02-SRNP-5688|669[0n]bp
Staphylus azteca|[2169]|00-SRNP-20190|669[0n]bp
Staphylus azteca|[2170]|03-SRNP-2688|669[0n]bp
Staphylus azteca|[2171]|03-SRNP-2728|669[0n]bp
Staphylus azteca|[2172]|04-SRNP-20603|669[0n]bp
Staphylus azteca|[2173]|02-SRNP-28722|669[0n]bp
Staphylus azteca|[2174]|02-SRNP-5769|669[0n]bp
Staphylus azteca|[2175]|05-SRNP-55695|669[0n]bp
Staphylus azteca|[2176]|05-SRNP-22538|669[0n]bp
Staphylus azteca|[2177]|04-SRNP-22976|669[0n]bp
Staphylus azteca|[2178]|04-SRNP-23046|669[0n]bp
Staphylus azteca|[2179]|98-SRNP-5973|594[0n]bp
Staphylus azteca|[2180]|00-SRNP-6536|576[0n]bp
Staphylus azteca|[2181]|08-SRNP-55660|669[0n]bp
Staphylus azteca|[2182]|08-SRNP-55779|669[0n]bp
Staphylus azteca|[2183]|08-SRNP-55777|669[0n]bp
Staphylus azteca|[2184]|00-SRNP-20191|669[1n]bp
Staphylus azteca|[2185]|08-SRNP-55729|669[0n]bp
Staphylus azteca|[2186]|07-SRNP-55831|669[0n]bp
Staphylus azteca|[2187]|06-SRNP-18820|669[0n]bp
Staphylus azteca|[2188]|02-SRNP-5529|669[0n]bp
Staphylus azteca|[2189]|00-SRNP-6529|669[0n]bp
Staphylus azteca|[2190]|03-SRNP-2686|669[0n]bp
Staphylus azteca|[2191]|00-SRNP-6598|669[0n]bp
Staphylus azteca|[2192]|00-SRNP-7041|669[0n]bp
Staphylus azteca|[2193]|04-SRNP-22251|669[0n]bp
Staphylus azteca|[2194]|04-SRNP-22252|669[0n]bp
Staphylus azteca|[2195]|05-SRNP-55771|669[0n]bp
Staphylus azteca|[2196]|05-SRNP-55417|669[0n]bp
Staphylus azteca|[2197]|03-SRNP-2646|669[0n]bp
Staphylus azteca|[2198]|02-SRNP-27769|669[0n]bp
Staphylus azteca|[2199]|02-SRNP-27768|669[0n]bp
Staphylus azteca|[2200]|00-SRNP-7043|669[2n]bp

Staphylus azteca|[2197]|03-SRNP-2046|669[0n]bp
Staphylus azteca|[2198]|02-SRNP-27769|669[0n]bp
Staphylus azteca|[2199]|02-SRNP-27768|669[0n]bp
Staphylus azteca|[2200]|00-SRNP-7043|669[2n]bp
Staphylus azteca|[2201]|03-SRNP-19829|669[0n]bp
Staphylus azteca|[2202]|02-SRNP-27766|669[0n]bp
Staphylus azteca|[2203]|98-SRNP-13484|669[0n]bp
Staphylus azteca|[2204]|00-SRNP-6518|654[0n]bp
Staphylus azteca|[2205]|02-SRNP-27389|627[1n]bp
Staphylus azteca|[2206]|00-SRNP-6585|603[0n]bp
Staphylus azteca|[2207]|00-SRNP-6599|606[0n]bp
Staphylus azteca|[2208]|07-SRNP-55820|663[0n]bp
Staphylus azteca|[2209]|00-SRNP-20193|669[1n]bp
Staphylus azteca|[2210]|02-SRNP-27392|669[2n]bp
Staphylus azteca|[2211]|08-SRNP-55943|669[0n]bp
Staphylus azteca|[2212]|98-SRNP-5691|669[0n]bp
Staphylus azteca|[2213]|02-SRNP-29445|669[0n]bp
Staphylus azteca|[2214]|02-SRNP-27393|669[0n]bp
Staphylus azteca|[2215]|00-SRNP-6587|669[0n]bp
Staphylus azteca|[2216]|00-SRNP-6533|603[0n]bp
Staphylus azteca|[2217]|07-SRNP-55875|669[0n]bp
Staphylus azteca|[2218]|08-SRNP-55945|669[0n]bp
Staphylus azteca|[2219]|09-SRNP-68493|669[0n]bp
Staphylus caribbea|[2220]|07-SRNP-65990|669[7n]bp
Staphylus caribbea|[2221]|05-SRNP-6745|567[3n]bp
Staphylus caribbea|[2222]|08-SRNP-6754|453[0n]bp
Staphylus caribbea|[2223]|07-SRNP-22962|669[4n]bp
Staphylus caribbea|[2224]|04-SRNP-42021|627[0n]bp
Staphylus caribbea|[2225]|07-SRNP-3050|669[1n]bp
Staphylus caribbea|[2226]|06-SRNP-2133|669[2n]bp
Staphylus caribbea|[2227]|07-SRNP-3052|621[4n]bp
Staphylus caribbea|[2228]|06-SRNP-316|573[0n]bp
Staphylus caribbea|[2229]|09-SRNP-75511|669[0n]bp
Staphylus caribbea|[2230]|09-SRNP-42728|669[0n]bp
Staphylus caribbea|[2231]|09-SRNP-68492|669[0n]bp
Staphylus caribbea|[2232]|06-SRNP-6883|552[0n]bp
Staphylus caribbea|[2233]|05-SRNP-7211|669[0n]bp
Staphylus caribbea|[2234]|05-SRNP-7214|669[0n]bp
Staphylus caribbea|[2235]|05-SRNP-6743|669[0n]bp
Staphylus caribbea|[2236]|05-SRNP-6797|669[0n]bp
Staphylus caribbea|[2237]|05-SRNP-7213|669[0n]bp
Staphylus caribbea|[2238]|05-SRNP-6906|669[0n]bp
Staphylus caribbea|[2239]|05-SRNP-7752|669[0n]bp
Staphylus caribbea|[2240]|04-SRNP-42026|669[0n]bp
Staphylus caribbea|[2241]|04-SRNP-42022|669[0n]bp
Staphylus caribbea|[2242]|05-SRNP-7750|627[0n]bp
Staphylus caribbea|[2243]|06-SRNP-5510|645[0n]bp
Staphylus caribbea|[2244]|06-SRNP-9444|669[0n]bp
Staphylus caribbea|[2245]|06-SRNP-8603|669[0n]bp
Staphylus caribbea|[2246]|06-SRNP-8454|669[0n]bp
Staphylus caribbea|[2247]|06-SRNP-9154|669[0n]bp
Staphylus caribbea|[2248]|06-SRNP-9158|669[0n]bp
Staphylus caribbea|[2249]|06-SRNP-6882|669[0n]bp
Staphylus caribbea|[2250]|07-SRNP-3051|669[0n]bp
Staphylus caribbea|[2251]|08-SRNP-6933|669[0n]bp
Staphylus caribbea|[2252]|09-SRNP-44334|669[0n]bp
Staphylus caribbea|[2253]|09-SRNP-44524|669[0n]bp
Staphylus caribbea|[2254]|09-SRNP-75315|669[0n]bp
Staphylus caribbea|[2255]|09-SRNP-68494|669[0n]bp
Staphylus caribbea|[2256]|09-SRNP-44999|669[0n]bp
Spioniades artemides|[2257]|07-SRNP-65617|660[0n]bp
Spioniades abbreviata|[2258]|05-SRNP-40403|669[1n]bp
Spioniades abbreviata|[2259]|05-SRNP-6908|669[1n]bp
Spioniades abbreviata|[2260]|03-SRNP-20661|663[0n]bp
Spioniades abbreviata|[2261]|03-SRNP-20234|663[0n]bp
Spioniades abbreviata|[2262]|04-SRNP-42534|669[0n]bp
Spioniades abbreviata|[2263]|08-SRNP-70324|669[0n]bp
Spioniades abbreviata|[2264]|09-SRNP-72126|669[0n]bp
Grais stigmaticus|[2265]|95-SRNP-11369|576[0n]bp
Doberes anticus|[2266]|00-SRNP-9187|669[0n]bp
Doberes anticus|[2267]|00-SRNP-9201|627[0n]bp
Doberes anticus|[2268]|00-SRNP-9246|663[1n]bp
Achlyodes pallida|[2269]|06-SRNP-55557|669[1n]bp
Achlyodes pallida|[2270]|04-SRNP-35303|669[0n]bp
Achlyodes pallida|[2271]|04-SRNP-46891|669[0n]bp
Achlyodes pallida|[2272]|04-SRNP-35345|669[0n]bp
Achlyodes pallida|[2273]|04-SRNP-35699|669[0n]bp
Achlyodes pallida|[2274]|06-SRNP-59832|669[0n]bp
Achlyodes pallida|[2275]|05-SRNP-35395|669[0n]bp
Achlyodes pallida|[2276]|07-SRNP-1848|669[0n]bp
Achlyodes pallida|[2277]|05-SRNP-2176|669[0n]bp
Achlyodes pallida|[2278]|05-SRNP-59488|669[0n]bp
Achlyodes pallida|[2279]|06-SRNP-60158|669[0n]bp
Achlyodes pallida|[2280]|06-SRNP-58206|669[0n]bp
Achlyodes pallida|[2281]|04-SRNP-61445|669[0n]bp
Achlyodes pallida|[2282]|04-SRNP-35305|669[0n]bp
Achlyodes pallida|[2283]|04-SRNP-35977|669[0n]bp
Achlyodes pallida|[2284]|04-SRNP-35301|669[0n]bp
Achlyodes pallida|[2285]|04-SRNP-35304|669[0n]bp
Achlyodes pallida|[2286]|04-SRNP-45082|669[0n]bp
Achlyodes pallida|[2287]|06-SRNP-55370|486[0n]bp
Achlyodes pallida|[2288]|03-SRNP-3397|528[0n]bp
Achlyodes pallida|[2289]|06-SRNP-60271|636[0n]bp
Achlyodes pallida|[2290]|02-SRNP-23406|576[0n]bp
Achlyodes pallida|[2291]|04-SRNP-35390|576[0n]bp
Achlyodes pallida|[2292]|07-SRNP-35141|435[0n]bp
Achlyodes pallida|[2293]|07-SRNP-35829|633[0n]bp
Achlyodes pallida|[2294]|07-SRNP-36029|627[0n]bp
Achlyodes pallida|[2295]|09-SRNP-57230|669[0n]bp
Eantis thraso|[2296]|07-SRNP-1978|669[0n]bp
Eantis thraso|[2297]|06-SRNP-22421|669[0n]bp
Eantis thraso|[2298]|96-SRNP-1072|573[0n]bp
Eantis thraso|[2299]|96-SRNP-262|576[0n]bp
Eantis thraso|[2300]|04-SRNP-40038|669[0n]bp

Eantis thraso|[2297]|06-SRNP-[illegible]|669[0n]bp
Eantis thraso|[2298]|96-SRNP-1072|573[0n]bp
Eantis thraso|[2299]|96-SRNP-262|576[0n]bp
Eantis thraso|[2300]|04-SRNP-49038|669[0n]bp
Eantis thraso|[2301]|04-SRNP-41151|669[0n]bp
Eantis thraso|[2302]|04-SRNP-50037|669[0n]bp
Eantis thraso|[2303]|04-SRNP-45216|669[0n]bp
Eantis thraso|[2304]|04-SRNP-48345|669[0n]bp
Eantis thraso|[2305]|04-SRNP-15358|669[0n]bp
Eantis thraso|[2306]|04-SRNP-4114|669[0n]bp
Eantis thraso|[2307]|94-SRNP-279|669[0n]bp
Eantis thraso|[2308]|07-SRNP-21152|669[0n]bp
Eantis thraso|[2309]|07-SRNP-1975|669[0n]bp
Eantis thraso|[2310]|04-SRNP-45084|669[0n]bp
Eantis thraso|[2311]|08-SRNP-6018|663[2n]bp
Achlyodes busirus|[2312]|03-SRNP-5736|540[0n]bp
Achlyodes busirus|[2313]|07-SRNP-1804|669[0n]bp
Achlyodes busirus|[2314]|07-SRNP-56770|669[0n]bp
Achlyodes busirus|[2315]|07-SRNP-55259|669[0n]bp
Achlyodes busirus|[2316]|07-SRNP-2136|669[0n]bp
Achlyodes busirus|[2317]|07-SRNP-1984|669[0n]bp
Achlyodes busirus|[2318]|07-SRNP-55258|669[0n]bp
Achlyodes busirus|[2319]|07-SRNP-55531|669[0n]bp
Achlyodes busirus|[2320]|06-SRNP-44397|669[0n]bp
Achlyodes busirus|[2321]|06-SRNP-44330|669[0n]bp
Achlyodes busirus|[2322]|06-SRNP-9750|669[0n]bp
Achlyodes busirus|[2323]|06-SRNP-59206|669[0n]bp
Achlyodes busirus|[2324]|06-SRNP-44403|669[0n]bp
Achlyodes busirus|[2325]|04-SRNP-47915|669[0n]bp
Achlyodes busirus|[2326]|04-SRNP-48202|669[0n]bp
Achlyodes busirus|[2327]|04-SRNP-48915|669[0n]bp
Achlyodes busirus|[2328]|04-SRNP-48497|669[0n]bp
Achlyodes busirus|[2329]|04-SRNP-32916|669[0n]bp
Achlyodes busirus|[2330]|04-SRNP-48413|669[0n]bp
Achlyodes busirus|[2331]|06-SRNP-44329|618[1n]bp
Achlyodes busirus|[2332]|06-SRNP-44395|624[1n]bp
Achlyodes busirus|[2333]|03-SRNP-5928|642[0n]bp
Achlyodes busirus|[2334]|06-SRNP-58729|609[0n]bp
Achlyodes busirus|[2335]|06-SRNP-44631|621[0n]bp
Achlyodes busirus|[2336]|06-SRNP-59810|630[2n]bp
Achlyodes busirus|[2337]|07-SRNP-1973|654[1n]bp
Achlyodes busirus|[2338]|07-SRNP-57186|645[0n]bp
Achlyodes busirus|[2339]|07-SRNP-45267|669[0n]bp
Achlyodes busirus|[2340]|07-SRNP-45958|669[0n]bp
Achlyodes busirus|[2341]|08-SRNP-2576|669[0n]bp
Achlyodes busirus|[2342]|08-SRNP-70349|669[0n]bp
Achlyodes busirus|[2343]|08-SRNP-70359|669[0n]bp
Achlyodes busirus|[2344]|08-SRNP-2332|669[0n]bp
Achlyodes busirus|[2345]|08-SRNP-2138|669[0n]bp
Achlyodes busirus|[2346]|08-SRNP-30931|669[0n]bp
Achlyodes busirus|[2347]|08-SRNP-20760|669[0n]bp
Achlyodes busirus|[2348]|08-SRNP-2135|669[0n]bp
Achlyodes busirus|[2349]|08-SRNP-4869|669[0n]bp
Achlyodes busirus|[2350]|08-SRNP-5054|669[0n]bp
Achlyodes busirus|[2351]|09-SRNP-69109|669[0n]bp
Aethilla echina|[2352]|05-SRNP-20|669[0n]bp
Aethilla lavochreaDHJ01|[2353]|08-SRNP-23841|669[0n]bp
Aethilla lavochreaDHJ02|[2354]|07-SRNP-24077|669[0n]bp
Aethilla lavochreaDHJ02|[2355]|08-SRNP-55465|669[0n]bp
Aethilla lavochreaDHJ02|[2356]|08-SRNP-22923|669[0n]bp
Aethilla lavochreaDHJ02|[2357]|09-SRNP-4333|669[0n]bp
Aethilla lavochreaDHJ02|[2358]|02-SRNP-14545|669[1n]bp
Aethilla lavochreaDHJ02|[2359]|00-SRNP-2103|669[0n]bp
Aethilla lavochreaDHJ02|[2360]|02-SRNP-4022|669[0n]bp
Aethilla lavochreaDHJ02|[2361]|00-SRNP-4557|669[0n]bp
Aethilla lavochreaDHJ02|[2362]|00-SRNP-2105|669[0n]bp
Aethilla lavochreaDHJ02|[2363]|02-SRNP-4467|669[0n]bp
Aethilla lavochreaDHJ02|[2364]|00-SRNP-4261|669[0n]bp
Aethilla lavochreaDHJ02|[2365]|00-SRNP-4439|669[0n]bp
Aethilla lavochreaDHJ02|[2366]|00-SRNP-4556|669[0n]bp
Aethilla lavochreaDHJ02|[2367]|00-SRNP-4260|669[0n]bp
Aethilla lavochreaDHJ02|[2368]|04-SRNP-49719|627[0n]bp
Aethilla lavochreaDHJ02|[2369]|08-SRNP-23663|669[0n]bp
Aethilla lavochreaDHJ02|[2370]|08-SRNP-23664|669[0n]bp
Aethilla lavochreaDHJ02|[2371]|09-SRNP-4334|669[1n]bp
Ephyriades eugramma|[2372]|03-SRNP-3195|489[0n]bp
Ephyriades eugramma|[2373]|03-SRNP-3270|663[0n]bp
Ephyriades eugramma|[2374]|03-SRNP-22990|663[0n]bp
Ephyriades eugramma|[2375]|03-SRNP-22535|663[0n]bp
Ephyriades eugramma|[2376]|07-SRNP-35417|648[0n]bp
Ephyriades eugramma|[2377]|08-SRNP-31277|669[0n]bp
Ephyriades eugramma|[2378]|08-SRNP-35337|669[0n]bp
Ephyriades eugramma|[2379]|09-SRNP-35830|669[0n]bp
Ephyriades eugramma|[2380]|09-SRNP-35828|669[0n]bp
Cycloglypha thrasibulus|[2381]|06-SRNP-55047|669[0n]bp
Cycloglypha thrasibulus|[2382]|06-SRNP-1733|669[0n]bp
Cycloglypha thrasibulus|[2383]|05-SRNP-2186|669[0n]bp
Cycloglypha thrasibulus|[2384]|05-SRNP-56292|669[0n]bp
Cycloglypha thrasibulus|[2385]|05-SRNP-2205|669[0n]bp
Cycloglypha thrasibulus|[2386]|06-SRNP-40014|669[0n]bp
Cycloglypha thrasibulus|[2387]|07-SRNP-55252|669[0n]bp
Cycloglypha thrasibulus|[2388]|07-SRNP-55550|669[0n]bp
Cycloglypha thrasibulus|[2389]|07-SRNP-55357|669[0n]bp
Cycloglypha thrasibulus|[2390]|06-SRNP-18966|669[0n]bp
Cycloglypha thrasibulus|[2391]|06-SRNP-18825|669[0n]bp
Cycloglypha thrasibulus|[2392]|06-SRNP-19741|669[0n]bp
Cycloglypha thrasibulus|[2393]|07-SRNP-59528|669[0n]bp
Cycloglypha thrasibulus|[2394]|08-SRNP-56046|669[0n]bp
Cycloglypha thrasibulus|[2395]|01-SRNP-18101|669[0n]bp
Cycloglypha thrasibulus|[2396]|05-SRNP-40775|669[0n]bp
Cycloglypha thrasibulus|[2397]|02-SRNP-31147|669[0n]bp
Cycloglypha thrasibulus|[2398]|02-SRNP-7413|669[0n]bp
Cycloglypha thrasibulus|[2399]|02-SRNP-4616|669[0n]bp
Cycloglypha thrasibulus|[2400]|[illegible]|669[0n]bp

Cycloglypha thrasibulus|[2397]|02-SRNP-31147|669[0n]bp
Cycloglypha thrasibulus|[2398]|02-SRNP-7413|669[0n]bp
Cycloglypha thrasibulus|[2399]|02-SRNP-4616|669[0n]bp
Cycloglypha thrasibulus|[2400]|02-SRNP-3233|669[0n]bp
Cycloglypha thrasibulus|[2401]|04-SRNP-3850|669[0n]bp
Cycloglypha thrasibulus|[2402]|04-SRNP-14593|669[0n]bp
Cycloglypha thrasibulus|[2403]|04-SRNP-55588|669[0n]bp
Cycloglypha thrasibulus|[2404]|05-SRNP-55022|669[0n]bp
Cycloglypha thrasibulus|[2405]|04-SRNP-15456|669[0n]bp
Cycloglypha thrasibulus|[2406]|02-SRNP-32143|669[0n]bp
Cycloglypha thrasibulus|[2407]|99-SRNP-18551|540[0n]bp
Cycloglypha thrasibulus|[2408]|02-SRNP-31149|642[0n]bp
Cycloglypha thrasibulus|[2409]|01-SRNP-4284|636[0n]bp
Cycloglypha thrasibulus|[2410]|04-SRNP-45636|534[0n]bp
Cycloglypha thrasibulus|[2411]|07-SRNP-23056|618[0n]bp
Cycloglypha thrasibulus|[2412]|04-SRNP-15458|618[0n]bp
Cycloglypha thrasibulus|[2413]|06-SRNP-19660|669[0n]bp
Cycloglypha thrasibulus|[2414]|08-SRNP-56190|669[0n]bp
Cycloglypha thrasibulus|[2415]|08-SRNP-56183|669[0n]bp
Cycloglypha thrasibulus|[2416]|08-SRNP-21510|669[0n]bp
Cycloglypha thrasibulus|[2417]|08-SRNP-56623|669[0n]bp
Cycloglypha thrasibulus|[2418]|08-SRNP-57718|669[0n]bp
Cycloglypha thrasibulus|[2419]|08-SRNP-36360|669[0n]bp
Ebrietas evanidus|[2420]|05-SRNP-24244|669[0n]bp
Ebrietas evanidus|[2421]|06-SRNP-3928|609[0n]bp
Ebrietas evanidus|[2422]|08-SRNP-4776|669[0n]bp
Chiomara mithrax|[2423]|08-SRNP-71174|669[0n]bp
Camptopleura theramenes|[2424]|05-SRNP-23809|669[0n]bp
Camptopleura theramenes|[2425]|06-SRNP-46916|645[0n]bp
Camptopleura auxoDHJ01|[2426]|05-SRNP-43216|630[0n]bp
Camptopleura auxoDHJ01|[2427]|82-SRNP-324|624[0n]bp
Camptopleura auxoDHJ01|[2428]|06-SRNP-65116|609[0n]bp
Camptopleura auxoDHJ01|[2429]|07-SRNP-42068|669[0n]bp
Camptopleura auxoDHJ01|[2430]|07-SRNP-42282|669[0n]bp
Camptopleura auxoDHJ01|[2431]|08-SRNP-71022|669[0n]bp
Camptopleura auxoDHJ02|[2432]|03-SRNP-31276|669[0n]bp
Camptopleura auxoDHJ02|[2433]|07-SRNP-41965|669[0n]bp
Camptopleura auxoDHJ02|[2434]|07-SRNP-24033|669[0n]bp
Camptopleura auxoDHJ02|[2435]|07-SRNP-42067|669[0n]bp
Camptopleura auxoDHJ02|[2436]|07-SRNP-42191|669[0n]bp
Camptopleura auxoDHJ02|[2437]|06-SRNP-42908|669[0n]bp
Camptopleura auxoDHJ02|[2438]|04-SRNP-40680|645[1n]bp
Camptopleura auxoDHJ02|[2439]|05-SRNP-43763|576[0n]bp
Camptopleura auxoDHJ02|[2440]|07-SRNP-40646|642[0n]bp
Camptopleura auxoDHJ02|[2441]|08-SRNP-65495|669[1n]bp
Camptopleura auxoDHJ02|[2442]|08-SRNP-65880|669[0n]bp
Mylon salvia|[2443]|06-SRNP-1769|669[0n]bp
Mylon salvia|[2444]|02-SRNP-16859|630[4n]bp
Mylon salvia|[2445]|02-SRNP-27057|612[5n]bp
Mylon salvia|[2446]|02-SRNP-14878|669[0n]bp
Mylon salvia|[2447]|05-SRNP-32566|624[0n]bp
Mylon salvia|[2448]|02-SRNP-14879|537[0n]bp
Mylon salvia|[2449]|02-SRNP-14882|669[0n]bp
Mylon salvia|[2450]|02-SRNP-14815|669[0n]bp
Mylon salvia|[2451]|06-SRNP-22322|669[0n]bp
Mylon salvia|[2452]|03-SRNP-3274|645[0n]bp
Mylon salvia|[2453]|02-SRNP-14880|669[0n]bp
Mylon salvia|[2454]|02-SRNP-14883|669[0n]bp
Mylon salvia|[2455]|02-SRNP-14884|645[0n]bp
Mylon maimon|[2456]|06-SRNP-65117|669[0n]bp
Mylon maimon|[2457]|04-SRNP-15734|669[0n]bp
Mylon maimon|[2458]|04-SRNP-15726|669[0n]bp
Mylon maimon|[2459]|06-SRNP-33362|609[0n]bp
Mylon maimon|[2460]|06-SRNP-55541|669[0n]bp
Mylon maimon|[2461]|05-SRNP-33196|669[0n]bp
Mylon maimon|[2462]|05-SRNP-45185|669[0n]bp
Mylon maimon|[2463]|04-SRNP-15661|669[0n]bp
Mylon maimon|[2464]|04-SRNP-15653|669[0n]bp
Mylon maimon|[2465]|02-SRNP-33927|657[0n]bp
Mylon maimon|[2466]|04-SRNP-25458|645[3n]bp
Mylon maimon|[2467]|05-SRNP-34629|624[0n]bp
Mylon maimon|[2468]|96-SRNP-9924|576[0n]bp
Mylon maimon|[2469]|05-SRNP-66553|477[0n]bp
Mylon maimon|[2470]|07-SRNP-60850|669[0n]bp
Mylon lassia|[2471]|04-SRNP-47729|669[0n]bp
Mylon lassia|[2472]|04-SRNP-49068|669[0n]bp
Mylon lassia|[2473]|05-SRNP-46313|669[0n]bp
Mylon lassia|[2474]|07-SRNP-45249|669[0n]bp
Mylon lassia|[2475]|09-SRNP-22793|669[0n]bp
Mylon lassia|[2476]|06-SRNP-32968|669[0n]bp
Mylon lassia|[2477]|04-SRNP-47995|669[1n]bp
Mylon lassia|[2478]|05-SRNP-45388|669[1n]bp
Mylon lassia|[2479]|06-SRNP-33008|669[1n]bp
Mylon lassia|[2480]|05-SRNP-32431|669[0n]bp
Mylon lassia|[2481]|05-SRNP-35383|669[0n]bp
Mylon lassia|[2482]|05-SRNP-45964|669[0n]bp
Mylon lassia|[2483]|04-SRNP-48829|669[0n]bp
Mylon lassia|[2484]|04-SRNP-49039|669[0n]bp
Mylon lassia|[2485]|06-SRNP-31605|669[0n]bp
Mylon lassia|[2486]|02-SRNP-28954|654[0n]bp
Mylon lassia|[2487]|02-SRNP-28773|624[0n]bp
Mylon lassia|[2488]|04-SRNP-32066|630[0n]bp
Mylon lassia|[2489]|04-SRNP-35482|591[0n]bp
Mylon lassia|[2490]|07-SRNP-33635|645[0n]bp
Mylon lassia|[2491]|09-SRNP-36601|669[0n]bp
Mylon lassia|[2492]|09-SRNP-22448|669[0n]bp
Timochares trifasciata|[2493]|05-SRNP-13161|429[0n]bp
Timochares trifasciata|[2494]|05-SRNP-12097|609[0n]bp
Timochares trifasciata|[2495]|03-SRNP-12286|588[0n]bp
Timochares trifasciata|[2496]|05-SRNP-12096|687[0n]bp
Timochares trifasciata|[2497]|05-SRNP-13158|669[1n]bp
Timochares trifasciata|[2498]|05-SRNP-13159|669[1n]bp
Timochares trifasciata|[2499]|03-SRNP-13719|669[0n]bp
Timochares trifasciata|[2500]|05-SRNP-[illegible]

Timochares trifasciata|[2497]|05-SRNP-13158|669[1n]bp
Timochares trifasciata|[2498]|05-SRNP-13159|669[1n]bp
Timochares trifasciata|[2499]|03-SRNP-13719|669[0n]bp
Timochares trifasciata|[2500]|05-SRNP-40802|669[2n]bp
Timochares trifasciata|[2501]|05-SRNP-13162|687[0n]bp
Timochares trifasciata|[2502]|04-SRNP-14040|669[0n]bp
Timochares trifasciata|[2503]|04-SRNP-14041|669[0n]bp
Timochares trifasciata|[2504]|03-SRNP-960|669[0n]bp
Timochares trifasciata|[2505]|03-SRNP-14113|669[0n]bp
Timochares trifasciata|[2506]|05-SRNP-19552|630[0n]bp
Timochares trifasciata|[2507]|08-SRNP-72653|669[0n]bp
Helias cama|[2508]|01-SRNP-9588|510[2n]bp
Helias cama|[2509]|05-SRNP-30554|669[0n]bp
Helias cama|[2510]|05-SRNP-6813|669[0n]bp
Helias cama|[2511]|06-SRNP-41760|669[0n]bp
Helias cama|[2512]|07-SRNP-20080|669[0n]bp
Helias cama|[2513]|08-SRNP-70554|669[0n]bp
Helias cama|[2514]|02-SRNP-15049|573[0n]bp
Helias cama|[2515]|04-SRNP-31168|669[0n]bp
Helias cama|[2516]|05-SRNP-30170|669[0n]bp
Helias cama|[2517]|04-SRNP-30824|669[0n]bp
Helias cama|[2518]|05-SRNP-31048|669[0n]bp
Helias cama|[2519]|05-SRNP-31049|669[0n]bp
Helias cama|[2520]|05-SRNP-31193|669[0n]bp
Helias cama|[2521]|05-SRNP-3359|669[0n]bp
Helias cama|[2522]|05-SRNP-3199|669[0n]bp
Helias cama|[2523]|05-SRNP-2671|669[0n]bp
Helias cama|[2524]|06-SRNP-453|669[0n]bp
Helias cama|[2525]|06-SRNP-2367|669[0n]bp
Helias cama|[2526]|07-SRNP-30806|669[0n]bp
Helias cama|[2527]|06-SRNP-22077|669[0n]bp
Helias cama|[2528]|07-SRNP-20157|669[0n]bp
Helias cama|[2529]|07-SRNP-65129|669[0n]bp
Helias cama|[2530]|08-SRNP-2560|669[0n]bp
Helias cama|[2531]|08-SRNP-2431|669[0n]bp
Helias cama|[2532]|08-SRNP-72018|669[0n]bp
Helias cama|[2533]|09-SRNP-30202|669[0n]bp
Helias cama|[2534]|09-SRNP-30198|669[0n]bp
Ebrietas osyris|[2535]|05-SRNP-20008|669[0n]bp
Ebrietas osyris|[2536]|05-SRNP-34654|669[0n]bp
Ebrietas osyris|[2537]|08-SRNP-70289|669[0n]bp
Ebrietas osyris|[2538]|08-SRNP-72341|669[0n]bp
Cycloglypha enega|[2539]|08-SRNP-32246|669[0n]bp
Ebrietas anacreonDHJ04|[2540]|07-SRNP-61246|669[0n]bp
Ebrietas anacreonDHJ04|[2541]|07-SRNP-66037|669[0n]bp
Ebrietas anacreonDHJ02|[2542]|07-SRNP-30890|669[0n]bp
Ebrietas anacreonDHJ02|[2543]|01-SRNP-17938|669[1n]bp
Ebrietas anacreonDHJ02|[2544]|93-SRNP-5851|669[0n]bp
Ebrietas anacreonDHJ02|[2545]|06-SRNP-30797|669[0n]bp
Ebrietas anacreonDHJ02|[2546]|04-SRNP-31276|630[0n]bp
Ebrietas anacreonDHJ02|[2547]|04-SRNP-30931|633[0n]bp
Ebrietas anacreonDHJ02|[2548]|06-SRNP-59644|669[0n]bp
Ebrietas anacreonDHJ02|[2549]|07-SRNP-31391|669[1n]bp
Ebrietas anacreonDHJ02|[2550]|06-SRNP-30796|669[0n]bp
Ebrietas anacreonDHJ02|[2551]|06-SRNP-31143|669[0n]bp
Ebrietas anacreonDHJ02|[2552]|06-SRNP-23020|669[0n]bp
Ebrietas anacreonDHJ02|[2553]|07-SRNP-59135|669[0n]bp
Ebrietas anacreonDHJ02|[2554]|08-SRNP-23566|669[0n]bp
Ebrietas anacreonDHJ02|[2555]|09-SRNP-30594|669[0n]bp
Ebrietas anacreonDHJ03|[2556]|93-SRNP-5844|621[2n]bp
Ebrietas anacreonDHJ03|[2557]|01-SRNP-18080|669[2n]bp
Ebrietas anacreonDHJ03|[2558]|94-SRNP-282|669[1n]bp
Ebrietas anacreonDHJ03|[2559]|05-SRNP-33161|669[0n]bp
Ebrietas anacreonDHJ03|[2560]|06-SRNP-30257|669[0n]bp
Ebrietas anacreonDHJ03|[2561]|05-SRNP-24345|669[0n]bp
Ebrietas anacreonDHJ03|[2562]|04-SRNP-32065|669[0n]bp
Ebrietas anacreonDHJ03|[2563]|01-SRNP-18079|669[0n]bp
Ebrietas anacreonDHJ03|[2564]|93-SRNP-5879|669[0n]bp
Ebrietas anacreonDHJ03|[2565]|02-SRNP-32566|621[0n]bp
Ebrietas anacreonDHJ03|[2566]|93-SRNP-5847|585[0n]bp
Ebrietas anacreonDHJ03|[2567]|93-SRNP-5836|534[0n]bp
Ebrietas anacreonDHJ03|[2568]|93-SRNP-5928|633[0n]bp
Ebrietas anacreonDHJ03|[2569]|02-SRNP-28985|633[0n]bp
Ebrietas anacreonDHJ03|[2570]|93-SRNP-5876|597[0n]bp
Ebrietas anacreonDHJ03|[2571]|93-SRNP-5846|609[0n]bp
Ebrietas anacreonDHJ03|[2572]|93-SRNP-6182|609[0n]bp
Ebrietas anacreonDHJ03|[2573]|93-SRNP-5839|600[3n]bp
Ebrietas anacreonDHJ03|[2574]|93-SRNP-5629|591[1n]bp
Ebrietas anacreonDHJ03|[2575]|93-SRNP-5840|582[0n]bp
Ebrietas anacreonDHJ03|[2576]|05-SRNP-31047|627[0n]bp
Ebrietas anacreonDHJ03|[2577]|05-SRNP-66020|627[0n]bp
Ebrietas anacreonDHJ03|[2578]|06-SRNP-30135|669[0n]bp
Ebrietas anacreonDHJ03|[2579]|06-SRNP-30138|669[0n]bp
Ebrietas anacreonDHJ03|[2580]|06-SRNP-30137|669[0n]bp
Ebrietas anacreonDHJ03|[2581]|06-SRNP-55045|669[0n]bp
Ebrietas anacreonDHJ03|[2582]|06-SRNP-30798|669[0n]bp
Ebrietas anacreonDHJ03|[2583]|06-SRNP-32332|669[0n]bp
Ebrietas anacreonDHJ03|[2584]|07-SRNP-55471|669[0n]bp
Ebrietas anacreonDHJ03|[2585]|08-SRNP-23847|669[0n]bp
Ebrietas anacreonDHJ03|[2586]|09-SRNP-71739|669[0n]bp
Ebrietas anacreonDHJ03|[2587]|09-SRNP-57295|669[0n]bp
Ebrietas anacreonDHJ03|[2588]|09-SRNP-12023|669[0n]bp
Potamanaxas Burns01|[2589]|91-SRNP-132|354[1n]bp
Potamanaxas Burns01|[2590]|07-SRNP-31875|648[0n]bp
Potamanaxas Burns02|[2591]|07-SRNP-65900|669[0n]bp
Potamanaxas Burns02|[2592]|07-SRNP-65901|669[0n]bp
Potamanaxas Burns02|[2593]|09-SRNP-71252|669[0n]bp
Mylon pelopidas|[2594]|00-SRNP-6099|669[0n]bp
Mylon pelopidas|[2595]|00-SRNP-6369|669[0n]bp
Mylon pelopidas|[2596]|00-SRNP-6128|669[0n]bp
Mylon pelopidas|[2597]|06-SRNP-12608|669[0n]bp
Mylon pelopidas|[2598]|01-SRNP-17181|663[0n]bp
Mylon pelopidas|[2599]|03-SRNP-649|657[0n]bp
Mylon pelopidas|[2600]|00-SRNP-6171|633[0n]bp

Mylon pelopidas|[2597]|06-SRNP-[illegible]|669[0n]bp
Mylon pelopidas|[2598]|01-SRNP-17181|663[0n]bp
Mylon pelopidas|[2599]|03-SRNP-649|657[0n]bp
Mylon pelopidas|[2600]|99-SRNP-6171|633[0n]bp
Mylon pelopidas|[2601]|97-SRNP-2965|645[0n]bp
Mylon pelopidas|[2602]|97-SRNP-3873|645[0n]bp
Mylon pelopidas|[2603]|02-SRNP-10065|648[1n]bp
Mylon pelopidas|[2604]|03-SRNP-12052|642[0n]bp
Mylon pelopidas|[2605]|93-SRNP-4445|633[1n]bp
Mylon pelopidas|[2606]|05-SRNP-55075|624[0n]bp
Mylon pelopidas|[2607]|97-SRNP-54|573[0n]bp
Mylon pelopidas|[2608]|94-SRNP-9689|600[0n]bp
Mylon pelopidas|[2609]|95-SRNP-6458|612[0n]bp
Mylon pelopidas|[2610]|07-SRNP-55508|633[0n]bp
Mylon pelopidas|[2611]|07-SRNP-55502|669[0n]bp
Mylon pelopidas|[2612]|07-SRNP-55540|669[0n]bp
Mylon pelopidas|[2613]|07-SRNP-55977|669[0n]bp
Mylon pelopidas|[2614]|07-SRNP-55365|669[0n]bp
Mylon pelopidas|[2615]|08-SRNP-55556|669[0n]bp
Potamanaxas Burns03|[2616]|07-SRNP-32749|669[0n]bp
Potamanaxas Burns03|[2617]|07-SRNP-32750|666[1n]bp
Potamanaxas Burns03|[2618]|07-SRNP-32792|669[0n]bp
Sostrata pusilla|[2619]|05-SRNP-7425|669[1n]bp
Sostrata pusilla|[2620]|07-SRNP-30539|669[0n]bp
Sostrata pusilla|[2621]|06-SRNP-4305|669[0n]bp
Sostrata pusilla|[2622]|06-SRNP-2403|669[0n]bp
Sostrata pusilla|[2623]|05-SRNP-1719|669[0n]bp
Sostrata pusilla|[2624]|05-SRNP-1859|669[0n]bp
Sostrata pusilla|[2625]|05-SRNP-41675|669[0n]bp
Sostrata pusilla|[2626]|04-SRNP-3394|669[0n]bp
Sostrata pusilla|[2627]|02-SRNP-18306|657[0n]bp
Sostrata pusilla|[2628]|05-SRNP-42029|645[0n]bp
Sostrata pusilla|[2629]|07-SRNP-1449|633[0n]bp
Sostrata pusilla|[2630]|07-SRNP-2998|669[0n]bp
Sostrata pusilla|[2631]|08-SRNP-30865|669[0n]bp
Sostrata bifasciata nordica|[2632]|03-SRNP-1099|648[0n]bp
Sostrata bifasciata nordica|[2633]|05-SRNP-2227|669[0n]bp
Sostrata bifasciata nordica|[2634]|08-SRNP-844|669[0n]bp
Sostrata bifasciata nordica|[2635]|07-SRNP-1567|669[0n]bp
Sostrata bifasciata nordica|[2636]|07-SRNP-1932|669[0n]bp
Sostrata bifasciata nordica|[2637]|07-SRNP-58638|669[0n]bp
Sostrata bifasciata nordica|[2638]|06-SRNP-3708|627[0n]bp
Sostrata bifasciata nordica|[2639]|06-SRNP-3696|666[0n]bp
Sostrata bifasciata nordica|[2640]|05-SRNP-213|669[0n]bp
Sostrata bifasciata nordica|[2641]|05-SRNP-214|669[0n]bp
Sostrata bifasciata nordica|[2642]|04-SRNP-22231|669[0n]bp
Sostrata bifasciata nordica|[2643]|04-SRNP-61492|669[0n]bp
Sostrata bifasciata nordica|[2644]|04-SRNP-61382|669[0n]bp
Sostrata bifasciata nordica|[2645]|04-SRNP-3640|669[0n]bp
Sostrata bifasciata nordica|[2646]|04-SRNP-1343|669[0n]bp
Sostrata bifasciata nordica|[2647]|04-SRNP-22116|669[0n]bp
Sostrata bifasciata nordica|[2648]|04-SRNP-2136|669[0n]bp
Sostrata bifasciata nordica|[2649]|05-SRNP-211|669[0n]bp
Sostrata bifasciata nordica|[2650]|04-SRNP-50041|669[0n]bp
Sostrata bifasciata nordica|[2651]|05-SRNP-212|669[0n]bp
Sostrata bifasciata nordica|[2652]|05-SRNP-296|669[1n]bp
Sostrata bifasciata nordica|[2653]|05-SRNP-2711|546[0n]bp
Sostrata bifasciata nordica|[2654]|04-SRNP-47237|657[0n]bp
Sostrata bifasciata nordica|[2655]|03-SRNP-5074|657[0n]bp
Sostrata bifasciata nordica|[2656]|08-SRNP-24554|612[0n]bp
Sostrata bifasciata nordica|[2657]|04-SRNP-23519|669[0n]bp
Sostrata bifasciata nordica|[2658]|04-SRNP-24494|669[0n]bp
Sostrata bifasciata nordica|[2659]|04-SRNP-22118|669[0n]bp
Sostrata bifasciata nordica|[2660]|05-SRNP-47046|669[0n]bp
Sostrata bifasciata nordica|[2661]|05-SRNP-4845|669[0n]bp
Sostrata bifasciata nordica|[2662]|05-SRNP-32215|669[0n]bp
Sostrata bifasciata nordica|[2663]|05-SRNP-46868|669[0n]bp
Sostrata bifasciata nordica|[2664]|05-SRNP-3026|669[0n]bp
Sostrata bifasciata nordica|[2665]|05-SRNP-45186|669[0n]bp
Sostrata bifasciata nordica|[2666]|05-SRNP-2678|669[0n]bp
Sostrata bifasciata nordica|[2667]|05-SRNP-31549|669[0n]bp
Sostrata bifasciata nordica|[2668]|05-SRNP-22997|669[0n]bp
Sostrata bifasciata nordica|[2669]|05-SRNP-40774|669[0n]bp
Sostrata bifasciata nordica|[2670]|05-SRNP-707|669[0n]bp
Sostrata bifasciata nordica|[2671]|05-SRNP-2270|669[0n]bp
Sostrata bifasciata nordica|[2672]|05-SRNP-295|669[0n]bp
Sostrata bifasciata nordica|[2673]|05-SRNP-6575|669[0n]bp
Sostrata bifasciata nordica|[2674]|05-SRNP-33197|669[0n]bp
Sostrata bifasciata nordica|[2675]|06-SRNP-1499|669[0n]bp
Sostrata bifasciata nordica|[2676]|06-SRNP-55120|669[0n]bp
Sostrata bifasciata nordica|[2677]|06-SRNP-3033|669[0n]bp
Sostrata bifasciata nordica|[2678]|06-SRNP-7519|669[0n]bp
Sostrata bifasciata nordica|[2679]|06-SRNP-23019|669[0n]bp
Sostrata bifasciata nordica|[2680]|07-SRNP-637|669[0n]bp
Sostrata bifasciata nordica|[2681]|07-SRNP-36575|669[0n]bp
Sostrata bifasciata nordica|[2682]|07-SRNP-58637|669[0n]bp
Sostrata bifasciata nordica|[2683]|07-SRNP-36790|669[0n]bp
Sostrata bifasciata nordica|[2684]|08-SRNP-2265|669[0n]bp
Sostrata bifasciata nordica|[2685]|08-SRNP-1977|669[0n]bp
Sostrata bifasciata nordica|[2686]|07-SRNP-60822|669[0n]bp
Sostrata bifasciata nordica|[2687]|08-SRNP-35027|669[0n]bp
Sostrata bifasciata nordica|[2688]|08-SRNP-36391|669[0n]bp
Sostrata bifasciata nordica|[2689]|09-SRNP-30271|669[0n]bp
Sostrata bifasciata nordica|[2690]|09-SRNP-35749|669[0n]bp
Sostrata bifasciata nordica|[2691]|09-SRNP-35141|669[0n]bp
Gesta gesta|[2692]|05-SRNP-62098|627[0n]bp
Gesta gesta|[2693]|96-SRNP-10372|576[0n]bp
Gesta gesta|[2694]|06-SRNP-18550|669[0n]bp
Gesta gesta|[2695]|05-SRNP-61955|669[1n]bp
Gesta gesta|[2696]|93-SRNP-5391|579[1n]bp
Gesta gesta|[2697]|05-SRNP-63396|627[0n]bp
Gesta gesta|[2698]|06-SRNP-18548|669[0n]bp
Gesta gesta|[2699]|06-SRNP-19618|648[1n]bp
Gesta gesta|[2700]|06-SRNP-18549|651[0n]bp

Gesta gesta|[2697]|05-SRNP-65396|627[0n]bp
Gesta gesta|[2698]|06-SRNP-18548|669[0n]bp
Gesta gesta|[2699]|06-SRNP-19618|648[1n]bp
Gesta gesta|[2700]|06-SRNP-18549|651[0n]bp
Gesta gesta|[2701]|04-SRNP-15977|669[0n]bp
Gesta gesta|[2702]|05-SRNP-63399|669[0n]bp
Gesta gesta|[2703]|07-SRNP-61370|669[0n]bp
Gesta gesta|[2704]|07-SRNP-61369|669[0n]bp
Erynnis tristis|[2705]|07-SRNP-55295|540[3n]bp
Erynnis tristis|[2706]|09-SRNP-55044|651[0n]bp
Erynnis tristis|[2707]|07-SRNP-55321|645[0n]bp
Erynnis tristis|[2708]|02-SRNP-10325|573[1n]bp
Erynnis tristis|[2709]|09-SRNP-55140|666[0n]bp
Erynnis tristis|[2710]|06-SRNP-57856|669[0n]bp
Erynnis tristis|[2711]|06-SRNP-57862|669[0n]bp
Erynnis tristis|[2712]|06-SRNP-57467|669[0n]bp
Erynnis tristis|[2713]|06-SRNP-57463|669[0n]bp
Erynnis tristis|[2714]|06-SRNP-57863|669[0n]bp
Erynnis tristis|[2715]|06-SRNP-57940|669[0n]bp
Erynnis tristis|[2716]|01-SRNP-15342|669[0n]bp
Erynnis tristis|[2717]|05-SRNP-64103|669[0n]bp
Erynnis tristis|[2718]|05-SRNP-64104|669[0n]bp
Erynnis tristis|[2719]|04-SRNP-12369|669[0n]bp
Erynnis tristis|[2720]|01-SRNP-16043|603[0n]bp
Erynnis tristis|[2721]|05-SRNP-13296|594[0n]bp
Erynnis tristis|[2722]|06-SRNP-57870|627[0n]bp
Erynnis tristis|[2723]|02-SRNP-10318|642[1n]bp
Erynnis tristis|[2724]|08-SRNP-58579|633[0n]bp
Erynnis tristis|[2725]|06-SRNP-57465|648[0n]bp
Erynnis tristis|[2726]|05-SRNP-64102|645[0n]bp
Erynnis tristis|[2727]|02-SRNP-10323|615[2n]bp
Erynnis tristis|[2728]|02-SRNP-10320|615[0n]bp
Erynnis tristis|[2729]|08-SRNP-58610|408[0n]bp
Erynnis tristis|[2730]|08-SRNP-58615|669[0n]bp
Anastrus sempiternus|[2731]|01-SRNP-17323|594[0n]bp
Anastrus sempiternus|[2732]|08-SRNP-2645|669[0n]bp
Anastrus sempiternus|[2733]|07-SRNP-4363|669[0n]bp
Anastrus sempiternus|[2734]|07-SRNP-4306|669[0n]bp
Anastrus sempiternus|[2735]|07-SRNP-32152|669[0n]bp
Anastrus sempiternus|[2736]|07-SRNP-12147|669[0n]bp
Anastrus sempiternus|[2737]|07-SRNP-55616|669[0n]bp
Anastrus sempiternus|[2738]|06-SRNP-60163|669[0n]bp
Anastrus sempiternus|[2739]|06-SRNP-60206|669[0n]bp
Anastrus sempiternus|[2740]|06-SRNP-57897|669[0n]bp
Anastrus sempiternus|[2741]|06-SRNP-57895|669[0n]bp
Anastrus sempiternus|[2742]|06-SRNP-57849|669[0n]bp
Anastrus sempiternus|[2743]|06-SRNP-23197|669[0n]bp
Anastrus sempiternus|[2744]|05-SRNP-64291|669[0n]bp
Anastrus sempiternus|[2745]|05-SRNP-64292|669[0n]bp
Anastrus sempiternus|[2746]|05-SRNP-1770|669[0n]bp
Anastrus sempiternus|[2747]|04-SRNP-4595|669[0n]bp
Anastrus sempiternus|[2748]|08-SRNP-66006|645[0n]bp
Anastrus sempiternus|[2749]|08-SRNP-58583|657[0n]bp
Anastrus sempiternus|[2750]|07-SRNP-4302|669[0n]bp
Anastrus sempiternus|[2751]|01-SRNP-17736|570[0n]bp
Anastrus sempiternus|[2752]|09-SRNP-57315|669[0n]bp
Clito aberrans|[2753]|06-SRNP-12726|669[0n]bp
Clito aberrans|[2754]|07-SRNP-55359|669[0n]bp
Clito aberrans|[2755]|05-SRNP-46256|657[1n]bp
Clito aberrans|[2756]|08-SRNP-55841|669[0n]bp
Clito aberrans|[2757]|99-SRNP-18483|528[0n]bp
Clito aberrans|[2758]|94-SRNP-3152|576[0n]bp
Clito aberrans|[2759]|05-SRNP-46254|669[0n]bp
Clito aberrans|[2760]|05-SRNP-46249|669[0n]bp
Clito aberrans|[2761]|06-SRNP-15483|669[0n]bp
Clito aberrans|[2762]|07-SRNP-3664|669[0n]bp
Clito aberrans|[2763]|07-SRNP-3662|669[0n]bp
Clito aberrans|[2764]|07-SRNP-3660|669[0n]bp
Clito aberrans|[2765]|08-SRNP-4314|669[0n]bp
Clito aberrans|[2766]|08-SRNP-4317|669[0n]bp
Clito aberrans|[2767]|08-SRNP-4315|669[0n]bp
Clito aberrans|[2768]|08-SRNP-4313|669[0n]bp
Clito Burns01|[2769]|05-SRNP-34516|669[0n]bp
Clito Burns01|[2770]|01-SRNP-9229|663[0n]bp
Clito Burns01|[2771]|01-SRNP-9304|657[0n]bp
Clito Burns01|[2772]|04-SRNP-21031|645[0n]bp
Clito Burns01|[2773]|08-SRNP-30288|660[0n]bp
Clito Burns01|[2774]|08-SRNP-70430|669[0n]bp
Anastrus neaeris|[2775]|05-SRNP-6078|630[0n]bp
Anastrus neaeris|[2776]|07-SRNP-65050|669[1n]bp
Anastrus neaeris|[2777]|05-SRNP-5537|669[0n]bp
Anastrus neaeris|[2778]|04-SRNP-60781|669[0n]bp
Anastrus neaeris|[2779]|04-SRNP-60867|669[0n]bp
Anastrus neaeris|[2780]|04-SRNP-60780|669[0n]bp
Anastrus neaeris|[2781]|02-SRNP-18593|657[0n]bp
Anastrus neaeris|[2782]|07-SRNP-65052|669[0n]bp
Anastrus neaeris|[2783]|04-SRNP-60339|669[0n]bp
Anastrus neaeris|[2784]|06-SRNP-1371|393[0n]bp
Anastrus neaeris|[2785]|06-SRNP-1373|393[0n]bp
Anastrus neaeris|[2786]|06-SRNP-1372|396[0n]bp
Anastrus neaeris|[2787]|07-SRNP-65051|633[0n]bp
Anastrus neaeris|[2788]|07-SRNP-35858|669[0n]bp
Anastrus neaeris|[2789]|08-SRNP-65477|669[0n]bp
Anastrus neaeris|[2790]|08-SRNP-65478|669[0n]bp
Anastrus neaeris|[2791]|08-SRNP-40484|669[0n]bp
Anastrus neaeris|[2792]|08-SRNP-65476|669[0n]bp
Anastrus neaeris|[2793]|08-SRNP-2106|669[0n]bp
Anastrus neaeris|[2794]|02-SRNP-16276|624[0n]bp
Anastrus neaeris|[2795]|04-SRNP-56411|588[0n]bp
Anastrus neaeris|[2796]|04-SRNP-34562|669[0n]bp
Anastrus neaeris|[2797]|08-SRNP-36419|669[0n]bp
Anastrus neaeris|[2798]|09-SRNP-67369|669[0n]bp
Eracon cliniasDHJ02|[2799]|05-SRNP-1688|669[2n]bp
Eracon cliniasDHJ02|[2800]|09-SRNP-40707|669[0n]bp

Anastrus neaeris|[2797]|08-SRNP-56419|669[0n]bp
Anastrus neaeris|[2798]|09-SRNP-67369|669[0n]bp
Eracon cliniasDHJ02|[2799]|05-SRNP-1688|669[2n]bp
Eracon cliniasDHJ02|[2800]|09-SRNP-40707|669[0n]bp
Eracon cliniasDHJ01|[2801]|05-SRNP-43583|669[0n]bp
Eracon cliniasDHJ01|[2802]|05-SRNP-22364|669[5n]bp
Eracon cliniasDHJ01|[2803]|07-SRNP-42619|669[0n]bp
Eracon cliniasDHJ01|[2804]|07-SRNP-42093|669[0n]bp
Eracon cliniasDHJ01|[2805]|07-SRNP-2980|669[0n]bp
Eracon cliniasDHJ01|[2806]|06-SRNP-44151|669[0n]bp
Eracon cliniasDHJ01|[2807]|06-SRNP-40016|669[0n]bp
Eracon cliniasDHJ01|[2808]|05-SRNP-7368|669[0n]bp
Eracon cliniasDHJ01|[2809]|05-SRNP-22120|669[0n]bp
Eracon cliniasDHJ01|[2810]|05-SRNP-22121|669[0n]bp
Eracon cliniasDHJ01|[2811]|05-SRNP-22467|669[1n]bp
Eracon cliniasDHJ01|[2812]|07-SRNP-42094|663[0n]bp
Eracon cliniasDHJ01|[2813]|03-SRNP-37802|663[0n]bp
Eracon cliniasDHJ01|[2814]|01-SRNP-5247|663[0n]bp
Eracon cliniasDHJ01|[2815]|05-SRNP-1713|576[0n]bp
Eracon cliniasDHJ01|[2816]|05-SRNP-22396|648[1n]bp
Eracon cliniasDHJ01|[2817]|05-SRNP-3449|561[0n]bp
Eracon cliniasDHJ01|[2818]|05-SRNP-1714|567[0n]bp
Eracon cliniasDHJ01|[2819]|01-SRNP-5289|645[0n]bp
Eracon cliniasDHJ01|[2820]|05-SRNP-3823|621[0n]bp
Eracon cliniasDHJ01|[2821]|05-SRNP-4520|642[2n]bp
Eracon cliniasDHJ01|[2822]|07-SRNP-42620|648[0n]bp
Eracon cliniasDHJ01|[2823]|08-SRNP-65365|669[0n]bp
Eracon cliniasDHJ01|[2824]|08-SRNP-21838|669[0n]bp
Eracon cliniasDHJ01|[2825]|08-SRNP-41449|669[0n]bp
Eracon cliniasDHJ01|[2826]|08-SRNP-41450|669[0n]bp
Eracon cliniasDHJ01|[2827]|09-SRNP-1734|669[0n]bp
Chiomara georgina|[2828]|07-SRNP-55431|669[0n]bp
Chiomara georgina|[2829]|03-SRNP-12148|630[0n]bp
Chiomara georgina|[2830]|07-SRNP-20138|669[0n]bp
Chiomara georgina|[2831]|08-SRNP-55568|669[0n]bp
Chiomara georgina|[2832]|06-SRNP-12610|669[0n]bp
Chiomara georgina|[2833]|04-SRNP-14129|669[0n]bp
Chiomara georgina|[2834]|04-SRNP-14128|669[0n]bp
Chiomara georgina|[2835]|04-SRNP-14583|669[0n]bp
Chiomara georgina|[2836]|03-SRNP-12149|624[1n]bp
Chiomara georgina|[2837]|05-SRNP-12171|627[0n]bp
Chiomara georgina|[2838]|06-SRNP-22681|642[0n]bp
Chiomara georgina|[2839]|06-SRNP-22680|669[0n]bp
Chiomara georgina|[2840]|07-SRNP-12184|669[0n]bp
Chiomara georgina|[2841]|07-SRNP-55287|669[0n]bp
Chiomara georgina|[2842]|07-SRNP-20170|669[0n]bp
Chiomara georgina|[2843]|07-SRNP-57560|669[0n]bp
Chiomara georgina|[2844]|09-SRNP-72879|669[0n]bp
Gorgythion begga pyralinaDHJ01|[2845]|01-SRNP-15890|669[0n]bp
Gorgythion begga pyralinaDHJ01|[2846]|02-SRNP-16048|516[0n]bp
Gorgythion begga pyralinaDHJ01|[2847]|02-SRNP-32020|516[0n]bp
Gorgythion begga pyralinaDHJ01|[2848]|05-SRNP-31852|669[0n]bp
Gorgythion begga pyralinaDHJ01|[2849]|07-SRNP-20872|528[0n]bp
Gorgythion begga pyralinaDHJ01|[2850]|05-SRNP-358|669[1n]bp
Gorgythion begga pyralinaDHJ01|[2851]|05-SRNP-40713|669[0n]bp
Gorgythion begga pyralinaDHJ01|[2852]|05-SRNP-1709|669[0n]bp
Gorgythion begga pyralinaDHJ01|[2853]|05-SRNP-40714|669[0n]bp
Gorgythion begga pyralinaDHJ01|[2854]|05-SRNP-1707|669[0n]bp
Gorgythion begga pyralinaDHJ01|[2855]|05-SRNP-1706|669[0n]bp
Gorgythion begga pyralinaDHJ01|[2856]|05-SRNP-1710|669[0n]bp
Gorgythion begga pyralinaDHJ01|[2857]|05-SRNP-64097|669[0n]bp
Gorgythion begga pyralinaDHJ01|[2858]|06-SRNP-531|669[0n]bp
Gorgythion begga pyralinaDHJ01|[2859]|06-SRNP-532|669[0n]bp
Gorgythion begga pyralinaDHJ01|[2860]|06-SRNP-41261|669[0n]bp
Gorgythion begga pyralinaDHJ01|[2861]|06-SRNP-31441|669[0n]bp
Gorgythion begga pyralinaDHJ01|[2862]|06-SRNP-2368|669[0n]bp
Gorgythion begga pyralinaDHJ01|[2863]|06-SRNP-20632|669[0n]bp
Gorgythion begga pyralinaDHJ01|[2864]|06-SRNP-41943|669[0n]bp
Gorgythion begga pyralinaDHJ01|[2865]|97-SRNP-5411|669[0n]bp
Gorgythion begga pyralinaDHJ01|[2866]|00-SRNP-2146|669[0n]bp
Gorgythion begga pyralinaDHJ01|[2867]|00-SRNP-2408|669[0n]bp
Gorgythion begga pyralinaDHJ01|[2868]|99-SRNP-2221|669[0n]bp
Gorgythion begga pyralinaDHJ01|[2869]|99-SRNP-15519|669[0n]bp
Gorgythion begga pyralinaDHJ01|[2870]|00-SRNP-11026|669[0n]bp
Gorgythion begga pyralinaDHJ01|[2871]|99-SRNP-15470|669[0n]bp
Gorgythion begga pyralinaDHJ01|[2872]|00-SRNP-3210|669[0n]bp
Gorgythion begga pyralinaDHJ01|[2873]|99-SRNP-4380|669[0n]bp
Gorgythion begga pyralinaDHJ01|[2874]|98-SRNP-4012|669[0n]bp
Gorgythion begga pyralinaDHJ01|[2875]|04-SRNP-50125|669[0n]bp
Gorgythion begga pyralinaDHJ01|[2876]|04-SRNP-14948|669[0n]bp
Gorgythion begga pyralinaDHJ01|[2877]|06-SRNP-1869|669[0n]bp
Gorgythion begga pyralinaDHJ01|[2878]|06-SRNP-1799|669[0n]bp
Gorgythion begga pyralinaDHJ01|[2879]|98-SRNP-4195|669[0n]bp
Gorgythion begga pyralinaDHJ01|[2880]|04-SRNP-45528|669[0n]bp
Gorgythion begga pyralinaDHJ01|[2881]|04-SRNP-21113|669[0n]bp
Gorgythion begga pyralinaDHJ01|[2882]|04-SRNP-45237|669[0n]bp
Gorgythion begga pyralinaDHJ01|[2883]|07-SRNP-55339|669[0n]bp
Gorgythion begga pyralinaDHJ01|[2884]|08-SRNP-70487|669[0n]bp
Gorgythion begga pyralinaDHJ01|[2885]|08-SRNP-45022|669[0n]bp
Gorgythion begga pyralinaDHJ01|[2886]|08-SRNP-20764|669[0n]bp
Gorgythion begga pyralinaDHJ01|[2887]|08-SRNP-30986|669[0n]bp
Gorgythion begga pyralinaDHJ01|[2888]|08-SRNP-30987|669[0n]bp
Gorgythion begga pyralinaDHJ01|[2889]|05-SRNP-24241|669[0n]bp
Gorgythion begga pyralinaDHJ01|[2890]|05-SRNP-24247|669[0n]bp
Gorgythion begga pyralinaDHJ01|[2891]|02-SRNP-32047|669[0n]bp
Gorgythion begga pyralinaDHJ01|[2892]|02-SRNP-2287|669[0n]bp
Gorgythion begga pyralinaDHJ01|[2893]|02-SRNP-32909|669[0n]bp
Gorgythion begga pyralinaDHJ01|[2894]|02-SRNP-5882|669[0n]bp
Gorgythion begga pyralinaDHJ01|[2895]|02-SRNP-13764|669[0n]bp
Gorgythion begga pyralinaDHJ01|[2896]|02-SRNP-5878|669[0n]bp
Gorgythion begga pyralinaDHJ01|[2897]|02-SRNP-14308|669[0n]bp
Gorgythion begga pyralinaDHJ01|[2898]|01-SRNP-4872|669[0n]bp
Gorgythion begga pyralinaDHJ01|[2899]|02-SRNP-13763|669[0n]bp
Gorgythion begga pyralinaDHJ01|[2900]|04-SRNP-15314|669[0n]bp

Gorgythion begga pyralinaDHJ01|[2897]|02-SRNP-14508|669[0n]bp
Gorgythion begga pyralinaDHJ01|[2898]|01-SRNP-4872|669[0n]bp
Gorgythion begga pyralinaDHJ01|[2899]|02-SRNP-13763|669[0n]bp
Gorgythion begga pyralinaDHJ01|[2900]|04-SRNP-15314|669[0n]bp
Gorgythion begga pyralinaDHJ01|[2901]|02-SRNP-2285|474[2n]bp
Gorgythion begga pyralinaDHJ01|[2902]|06-SRNP-30280|669[0n]bp
Gorgythion begga pyralinaDHJ01|[2903]|05-SRNP-206|663[0n]bp
Gorgythion begga pyralinaDHJ01|[2904]|06-SRNP-753|645[0n]bp
Gorgythion begga pyralinaDHJ01|[2905]|05-SRNP-23371|630[0n]bp
Gorgythion begga pyralinaDHJ01|[2906]|06-SRNP-1800|627[0n]bp
Gorgythion begga pyralinaDHJ01|[2907]|04-SRNP-27297|627[0n]bp
Gorgythion begga pyralinaDHJ01|[2908]|96-SRNP-886|606[0n]bp
Gorgythion begga pyralinaDHJ01|[2909]|06-SRNP-30794|642[0n]bp
Gorgythion begga pyralinaDHJ01|[2910]|99-SRNP-322|624[0n]bp
Gorgythion begga pyralinaDHJ01|[2911]|99-SRNP-298|618[1n]bp
Gorgythion begga pyralinaDHJ01|[2912]|96-SRNP-12038|618[1n]bp
Gorgythion begga pyralinaDHJ01|[2913]|99-SRNP-2514|615[1n]bp
Gorgythion begga pyralinaDHJ01|[2914]|06-SRNP-263|609[0n]bp
Gorgythion begga pyralinaDHJ01|[2915]|95-SRNP-6465|615[0n]bp
Gorgythion begga pyralinaDHJ01|[2916]|03-SRNP-28548|576[0n]bp
Gorgythion begga pyralinaDHJ01|[2917]|02-SRNP-17248|576[1n]bp
Gorgythion begga pyralinaDHJ01|[2918]|92-SRNP-3165|378[0n]bp
Gorgythion begga pyralinaDHJ01|[2919]|07-SRNP-20992|540[1n]bp
Gorgythion begga pyralinaDHJ01|[2920]|07-SRNP-20948|633[0n]bp
Gorgythion begga pyralinaDHJ01|[2921]|08-SRNP-40557|669[0n]bp
Gorgythion begga pyralinaDHJ01|[2922]|08-SRNP-70553|321[0n]bp
Gorgythion begga pyralinaDHJ02|[2923]|03-SRNP-1239|516[0n]bp
Gorgythion begga pyralinaDHJ02|[2924]|03-SRNP-20279|516[0n]bp
Gorgythion begga pyralinaDHJ02|[2925]|03-SRNP-1176|516[0n]bp
Gorgythion begga pyralinaDHJ02|[2926]|05-SRNP-33821|429[2n]bp
Gorgythion begga pyralinaDHJ02|[2927]|05-SRNP-33754|669[4n]bp
Gorgythion begga pyralinaDHJ02|[2928]|05-SRNP-33763|669[0n]bp
Gorgythion begga pyralinaDHJ02|[2929]|01-SRNP-17454|669[0n]bp
Gorgythion begga pyralinaDHJ02|[2930]|06-SRNP-40222|666[0n]bp
Gorgythion begga pyralinaDHJ02|[2931]|07-SRNP-55213|666[2n]bp
Gorgythion begga pyralinaDHJ02|[2932]|01-SRNP-1269|669[1n]bp
Gorgythion begga pyralinaDHJ02|[2933]|08-SRNP-20679|669[0n]bp
Gorgythion begga pyralinaDHJ02|[2934]|08-SRNP-24442|669[0n]bp
Gorgythion begga pyralinaDHJ02|[2935]|07-SRNP-60837|669[0n]bp
Gorgythion begga pyralinaDHJ02|[2936]|08-SRNP-2157|669[0n]bp
Gorgythion begga pyralinaDHJ02|[2937]|06-SRNP-19738|669[0n]bp
Gorgythion begga pyralinaDHJ02|[2938]|07-SRNP-55196|669[0n]bp
Gorgythion begga pyralinaDHJ02|[2939]|99-SRNP-2400|669[0n]bp
Gorgythion begga pyralinaDHJ02|[2940]|98-SRNP-4745|669[0n]bp
Gorgythion begga pyralinaDHJ02|[2941]|00-SRNP-2145|669[0n]bp
Gorgythion begga pyralinaDHJ02|[2942]|98-SRNP-13474|669[0n]bp
Gorgythion begga pyralinaDHJ02|[2943]|06-SRNP-42907|669[0n]bp
Gorgythion begga pyralinaDHJ02|[2944]|06-SRNP-22800|669[0n]bp
Gorgythion begga pyralinaDHJ02|[2945]|04-SRNP-42567|669[0n]bp
Gorgythion begga pyralinaDHJ02|[2946]|04-SRNP-41839|669[0n]bp
Gorgythion begga pyralinaDHJ02|[2947]|06-SRNP-57189|669[0n]bp
Gorgythion begga pyralinaDHJ02|[2948]|06-SRNP-57188|669[0n]bp
Gorgythion begga pyralinaDHJ02|[2949]|97-SRNP-127|669[0n]bp
Gorgythion begga pyralinaDHJ02|[2950]|02-SRNP-14120|669[0n]bp
Gorgythion begga pyralinaDHJ02|[2951]|06-SRNP-30444|669[0n]bp
Gorgythion begga pyralinaDHJ02|[2952]|06-SRNP-30443|669[0n]bp
Gorgythion begga pyralinaDHJ02|[2953]|05-SRNP-65607|669[0n]bp
Gorgythion begga pyralinaDHJ02|[2954]|05-SRNP-66197|669[0n]bp
Gorgythion begga pyralinaDHJ02|[2955]|05-SRNP-30599|669[0n]bp
Gorgythion begga pyralinaDHJ02|[2956]|05-SRNP-2471|669[0n]bp
Gorgythion begga pyralinaDHJ02|[2957]|06-SRNP-22074|669[0n]bp
Gorgythion begga pyralinaDHJ02|[2958]|06-SRNP-65353|669[0n]bp
Gorgythion begga pyralinaDHJ02|[2959]|07-SRNP-42322|669[0n]bp
Gorgythion begga pyralinaDHJ02|[2960]|07-SRNP-42321|669[0n]bp
Gorgythion begga pyralinaDHJ02|[2961]|06-SRNP-30986|669[0n]bp
Gorgythion begga pyralinaDHJ02|[2962]|06-SRNP-31171|669[0n]bp
Gorgythion begga pyralinaDHJ02|[2963]|05-SRNP-33765|669[0n]bp
Gorgythion begga pyralinaDHJ02|[2964]|05-SRNP-33759|669[0n]bp
Gorgythion begga pyralinaDHJ02|[2965]|02-SRNP-5740|669[0n]bp
Gorgythion begga pyralinaDHJ02|[2966]|02-SRNP-5307|669[0n]bp
Gorgythion begga pyralinaDHJ02|[2967]|02-SRNP-5972|669[0n]bp
Gorgythion begga pyralinaDHJ02|[2968]|02-SRNP-4537|669[0n]bp
Gorgythion begga pyralinaDHJ02|[2969]|02-SRNP-5739|669[0n]bp
Gorgythion begga pyralinaDHJ02|[2970]|02-SRNP-33710|669[0n]bp
Gorgythion begga pyralinaDHJ02|[2971]|01-SRNP-9221|669[0n]bp
Gorgythion begga pyralinaDHJ02|[2972]|02-SRNP-2286|669[0n]bp
Gorgythion begga pyralinaDHJ02|[2973]|01-SRNP-1270|669[0n]bp
Gorgythion begga pyralinaDHJ02|[2974]|01-SRNP-17453|669[0n]bp
Gorgythion begga pyralinaDHJ02|[2975]|01-SRNP-15739|669[0n]bp
Gorgythion begga pyralinaDHJ02|[2976]|02-SRNP-27923|669[2n]bp
Gorgythion begga pyralinaDHJ02|[2977]|04-SRNP-41616|669[0n]bp
Gorgythion begga pyralinaDHJ02|[2978]|03-SRNP-21505|642[0n]bp
Gorgythion begga pyralinaDHJ02|[2979]|06-SRNP-23305|669[0n]bp
Gorgythion begga pyralinaDHJ02|[2980]|08-SRNP-65479|669[0n]bp
Gorgythion begga pyralinaDHJ02|[2981]|08-SRNP-4702|666[0n]bp
Gorgythion begga pyralinaDHJ02|[2982]|05-SRNP-66163|630[0n]bp
Gorgythion begga pyralinaDHJ02|[2983]|05-SRNP-40934|636[0n]bp
Gorgythion begga pyralinaDHJ02|[2984]|02-SRNP-15032|627[0n]bp
Gorgythion begga pyralinaDHJ02|[2985]|07-SRNP-31672|642[0n]bp
Gorgythion begga pyralinaDHJ02|[2986]|07-SRNP-65124|639[0n]bp
Gorgythion begga pyralinaDHJ02|[2987]|05-SRNP-33764|630[0n]bp
Gorgythion begga pyralinaDHJ02|[2988]|03-SRNP-28550|609[2n]bp
Gorgythion begga pyralinaDHJ02|[2989]|98-SRNP-4629|594[0n]bp
Gorgythion begga pyralinaDHJ02|[2990]|99-SRNP-2220|630[1n]bp
Gorgythion begga pyralinaDHJ02|[2991]|98-SRNP-4280|630[1n]bp
Gorgythion begga pyralinaDHJ02|[2992]|99-SRNP-2219|630[1n]bp
Gorgythion begga pyralinaDHJ02|[2993]|02-SRNP-14184|630[1n]bp
Gorgythion begga pyralinaDHJ02|[2994]|05-SRNP-41201|588[0n]bp
Gorgythion begga pyralinaDHJ02|[2995]|95-SRNP-7044|606[0n]bp
Gorgythion begga pyralinaDHJ02|[2996]|06-SRNP-40632|606[0n]bp
Gorgythion begga pyralinaDHJ02|[2997]|95-SRNP-6166|624[0n]bp
Gorgythion begga pyralinaDHJ02|[2998]|06-SRNP-30946|633[0n]bp
Gorgythion begga pyralinaDHJ02|[2999]|05-SRNP-40817|633[0n]bp
Gorgythion begga pyralinaDHJ02|[3000]|06-SRNP-9680|639[0n]bp

Gorgythion begga pyralinaDHJ02|[2997]|95-SRNP-6166|624[0n]bp
Gorgythion begga pyralinaDHJ02|[2998]|06-SRNP-30946|633[0n]bp
Gorgythion begga pyralinaDHJ02|[2999]|05-SRNP-40817|633[0n]bp
Gorgythion begga pyralinaDHJ02|[3000]|96-SRNP-9689|639[0n]bp
Gorgythion begga pyralinaDHJ02|[3001]|97-SRNP-5666|624[0n]bp
Gorgythion begga pyralinaDHJ02|[3002]|99-SRNP-3525|624[0n]bp
Gorgythion begga pyralinaDHJ02|[3003]|03-SRNP-21608|561[0n]bp
Gorgythion begga pyralinaDHJ02|[3004]|02-SRNP-5088|576[0n]bp
Gorgythion begga pyralinaDHJ02|[3005]|98-SRNP-4756|630[1n]bp
Gorgythion begga pyralinaDHJ02|[3006]|04-SRNP-43161|573[0n]bp
Gorgythion begga pyralinaDHJ02|[3007]|97-SRNP-380|603[0n]bp
Gorgythion begga pyralinaDHJ02|[3008]|05-SRNP-32342|525[0n]bp
Gorgythion begga pyralinaDHJ02|[3009]|97-SRNP-11537|609[0n]bp
Gorgythion begga pyralinaDHJ02|[3010]|00-SRNP-2655|636[0n]bp
Gorgythion begga pyralinaDHJ02|[3011]|08-SRNP-40397|633[0n]bp
Gorgythion begga pyralinaDHJ02|[3012]|08-SRNP-40417|594[0n]bp
Gorgythion begga pyralinaDHJ02|[3013]|93-SRNP-7034|558[0n]bp
Gorgythion begga pyralinaDHJ02|[3014]|03-SRNP-20828|645[0n]bp
Gorgythion begga pyralinaDHJ02|[3015]|05-SRNP-30971|687[0n]bp
Gorgythion begga pyralinaDHJ02|[3016]|07-SRNP-23849|669[0n]bp
Gorgythion begga pyralinaDHJ02|[3017]|06-SRNP-59585|669[0n]bp
Gorgythion begga pyralinaDHJ02|[3018]|06-SRNP-59797|669[0n]bp
Gorgythion begga pyralinaDHJ02|[3019]|06-SRNP-59614|669[0n]bp
Gorgythion begga pyralinaDHJ02|[3020]|06-SRNP-43424|669[0n]bp
Gorgythion begga pyralinaDHJ02|[3021]|06-SRNP-22111|669[0n]bp
Gorgythion begga pyralinaDHJ02|[3022]|04-SRNP-30434|669[0n]bp
Gorgythion begga pyralinaDHJ02|[3023]|04-SRNP-40895|669[0n]bp
Gorgythion begga pyralinaDHJ02|[3024]|04-SRNP-35213|669[0n]bp
Gorgythion begga pyralinaDHJ02|[3025]|04-SRNP-15268|669[0n]bp
Gorgythion begga pyralinaDHJ02|[3026]|04-SRNP-33308|669[0n]bp
Gorgythion begga pyralinaDHJ02|[3027]|00-SRNP-6011|669[0n]bp
Gorgythion begga pyralinaDHJ02|[3028]|00-SRNP-2364|669[0n]bp
Gorgythion begga pyralinaDHJ02|[3029]|97-SRNP-10376|669[0n]bp
Gorgythion begga pyralinaDHJ02|[3030]|00-SRNP-2182|669[0n]bp
Gorgythion begga pyralinaDHJ02|[3031]|00-SRNP-2038|669[0n]bp
Gorgythion begga pyralinaDHJ02|[3032]|06-SRNP-41729|669[0n]bp
Gorgythion begga pyralinaDHJ02|[3033]|06-SRNP-30448|669[0n]bp
Gorgythion begga pyralinaDHJ02|[3034]|06-SRNP-30751|669[0n]bp
Gorgythion begga pyralinaDHJ02|[3035]|06-SRNP-30445|669[0n]bp
Gorgythion begga pyralinaDHJ02|[3036]|06-SRNP-30942|669[0n]bp
Gorgythion begga pyralinaDHJ02|[3037]|05-SRNP-33753|669[0n]bp
Gorgythion begga pyralinaDHJ02|[3038]|05-SRNP-33752|669[0n]bp
Gorgythion begga pyralinaDHJ02|[3039]|05-SRNP-33272|669[0n]bp
Gorgythion begga pyralinaDHJ02|[3040]|05-SRNP-30970|669[0n]bp
Gorgythion begga pyralinaDHJ02|[3041]|05-SRNP-31860|669[0n]bp
Gorgythion begga pyralinaDHJ02|[3042]|02-SRNP-21092|669[0n]bp
Gorgythion begga pyralinaDHJ02|[3043]|02-SRNP-33944|669[0n]bp
Gorgythion begga pyralinaDHJ02|[3044]|02-SRNP-19850|669[0n]bp
Gorgythion begga pyralinaDHJ02|[3045]|02-SRNP-5107|669[0n]bp
Gorgythion begga pyralinaDHJ02|[3046]|02-SRNP-27777|669[0n]bp
Gorgythion begga pyralinaDHJ02|[3047]|07-SRNP-55244|666[0n]bp
Gorgythion begga pyralinaDHJ02|[3048]|07-SRNP-55198|663[0n]bp
Gorgythion begga pyralinaDHJ02|[3049]|05-SRNP-31861|630[3n]bp
Gorgythion begga pyralinaDHJ02|[3050]|04-SRNP-43162|660[0n]bp
Gorgythion begga pyralinaDHJ02|[3051]|05-SRNP-30226|660[0n]bp
Gorgythion begga pyralinaDHJ02|[3052]|06-SRNP-57350|657[0n]bp
Gorgythion begga pyralinaDHJ02|[3053]|05-SRNP-30688|618[0n]bp
Gorgythion begga pyralinaDHJ02|[3054]|02-SRNP-14229|648[0n]bp
Gorgythion begga pyralinaDHJ02|[3055]|02-SRNP-13839|642[0n]bp
Gorgythion begga pyralinaDHJ02|[3056]|04-SRNP-56552|642[0n]bp
Gorgythion begga pyralinaDHJ02|[3057]|07-SRNP-41296|645[0n]bp
Gorgythion begga pyralinaDHJ02|[3058]|08-SRNP-40399|660[0n]bp
Gorgythion begga pyralinaDHJ02|[3059]|08-SRNP-70432|669[0n]bp
Gorgythion begga pyralinaDHJ02|[3060]|08-SRNP-1816|669[0n]bp
Gorgythion begga pyralinaDHJ02|[3061]|08-SRNP-30607|669[0n]bp
Gorgythion begga pyralinaDHJ02|[3062]|08-SRNP-20433|669[0n]bp
Gorgythion begga pyralinaDHJ02|[3063]|08-SRNP-30605|669[0n]bp
Gorgythion begga pyralinaDHJ02|[3064]|08-SRNP-30606|669[0n]bp
Gorgythion begga pyralinaDHJ02|[3065]|08-SRNP-22577|669[0n]bp
Gorgythion begga pyralinaDHJ02|[3066]|08-SRNP-24676|669[0n]bp
Gorgythion begga pyralinaDHJ02|[3067]|09-SRNP-72858|669[0n]bp
Gorgythion begga pyralinaDHJ02|[3068]|06-SRNP-60373|669[0n]bp
Gorgythion begga pyralinaDHJ02|[3069]|04-SRNP-42979|669[0n]bp
Gorgythion begga pyralinaDHJ02|[3070]|05-SRNP-66115|669[0n]bp
Gorgythion begga pyralinaDHJ02|[3071]|05-SRNP-41039|669[0n]bp
Gorgythion begga pyralinaDHJ02|[3072]|03-SRNP-21087|645[0n]bp
Gorgythion begga pyralinaDHJ02|[3073]|06-SRNP-30446|666[0n]bp
Gorgythion begga pyralinaDHJ02|[3074]|04-SRNP-60168|666[0n]bp
Gorgythion begga pyralinaDHJ02|[3075]|05-SRNP-20969|669[0n]bp
Gorgythion begga pyralinaDHJ02|[3076]|08-SRNP-65113|669[0n]bp
Gorgythion begga pyralinaDHJ02|[3077]|09-SRNP-66079|669[0n]bp
Telemiades fides|[3078]|07-SRNP-16611|321[0n]bp
Telemiades fides|[3079]|05-SRNP-55775|669[0n]bp
Telemiades fides|[3080]|08-SRNP-65257|669[0n]bp
Telemiades fides|[3081]|02-SRNP-14055|669[0n]bp
Telemiades fides|[3082]|07-SRNP-23507|669[0n]bp
Telemiades fides|[3083]|05-SRNP-13191|636[1n]bp
Telemiades fides|[3084]|07-SRNP-65513|642[0n]bp
Telemiades fides|[3085]|07-SRNP-61381|642[0n]bp
Telemiades fides|[3086]|07-SRNP-60109|642[0n]bp
Telemiades fides|[3087]|07-SRNP-21983|642[0n]bp
Telemiades fides|[3088]|07-SRNP-65137|642[0n]bp
Telemiades fides|[3089]|07-SRNP-31688|642[0n]bp
Telemiades fides|[3090]|07-SRNP-31687|642[0n]bp
Telemiades fides|[3091]|07-SRNP-21628|642[0n]bp
Telemiades fides|[3092]|07-SRNP-20486|642[0n]bp
Telemiades fides|[3093]|07-SRNP-21257|642[0n]bp
Telemiades fides|[3094]|06-SRNP-60265|642[0n]bp
Telemiades fides|[3095]|02-SRNP-3677|669[1n]bp
Telemiades fides|[3096]|07-SRNP-5104|636[0n]bp
Telemiades fides|[3097]|07-SRNP-31870|648[0n]bp
Telemiades fides|[3098]|07-SRNP-65687|645[0n]bp
Telemiades fides|[3099]|03-SRNP-1234|645[0n]bp
Telemiades fides|[3100]|02-SRNP-4033|654[0n]bp

Telemiades fides|[3097]|07-SRNP-31870|648[0n]bp
Telemiades fides|[3098]|07-SRNP-65687|645[0n]bp
Telemiades fides|[3099]|03-SRNP-1234|645[0n]bp
Telemiades fides|[3100]|02-SRNP-4033|654[0n]bp
Telemiades fides|[3101]|07-SRNP-3685|630[0n]bp
Telemiades fides|[3102]|02-SRNP-17931|627[2n]bp
Telemiades fides|[3103]|07-SRNP-31873|627[0n]bp
Telemiades fides|[3104]|08-SRNP-45092|669[1n]bp
Telemiades fides|[3105]|08-SRNP-24174|627[0n]bp
Telemiades fides|[3106]|05-SRNP-55645|669[0n]bp
Telemiades fides|[3107]|07-SRNP-3734|669[0n]bp
Telemiades fides|[3108]|07-SRNP-3520|642[0n]bp
Telemiades fides|[3109]|05-SRNP-55619|669[0n]bp
Telemiades fides|[3110]|07-SRNP-32190|654[0n]bp
Telemiades fides|[3111]|02-SRNP-10030|666[1n]bp
Telemiades fides|[3112]|07-SRNP-31486|552[0n]bp
Telemiades fides|[3113]|05-SRNP-13188|687[0n]bp
Telemiades fides|[3114]|07-SRNP-55335|669[0n]bp
Telemiades fides|[3115]|07-SRNP-55240|669[0n]bp
Telemiades fides|[3116]|07-SRNP-55110|669[0n]bp
Telemiades fides|[3117]|07-SRNP-1307|669[0n]bp
Telemiades fides|[3118]|07-SRNP-55464|669[0n]bp
Telemiades fides|[3119]|07-SRNP-55163|669[0n]bp
Telemiades fides|[3120]|07-SRNP-1762|669[0n]bp
Telemiades fides|[3121]|07-SRNP-21361|669[0n]bp
Telemiades fides|[3122]|07-SRNP-21318|669[0n]bp
Telemiades fides|[3123]|07-SRNP-21194|669[0n]bp
Telemiades fides|[3124]|07-SRNP-20999|669[0n]bp
Telemiades fides|[3125]|07-SRNP-21302|669[0n]bp
Telemiades fides|[3126]|07-SRNP-21275|669[0n]bp
Telemiades fides|[3127]|07-SRNP-21243|669[0n]bp
Telemiades fides|[3128]|07-SRNP-21453|669[0n]bp
Telemiades fides|[3129]|07-SRNP-65185|669[0n]bp
Telemiades fides|[3130]|07-SRNP-20827|669[0n]bp
Telemiades fides|[3131]|07-SRNP-31658|669[0n]bp
Telemiades fides|[3132]|07-SRNP-30815|669[0n]bp
Telemiades fides|[3133]|07-SRNP-57095|669[0n]bp
Telemiades fides|[3134]|07-SRNP-2224|669[0n]bp
Telemiades fides|[3135]|07-SRNP-56685|669[0n]bp
Telemiades fides|[3136]|07-SRNP-31298|669[0n]bp
Telemiades fides|[3137]|07-SRNP-3731|669[0n]bp
Telemiades fides|[3138]|07-SRNP-3500|669[0n]bp
Telemiades fides|[3139]|07-SRNP-3567|669[0n]bp
Telemiades fides|[3140]|07-SRNP-3838|669[0n]bp
Telemiades fides|[3141]|07-SRNP-65761|669[0n]bp
Telemiades fides|[3142]|07-SRNP-4407|669[0n]bp
Telemiades fides|[3143]|07-SRNP-4674|669[0n]bp
Telemiades fides|[3144]|07-SRNP-59719|669[0n]bp
Telemiades fides|[3145]|07-SRNP-65688|669[0n]bp
Telemiades fides|[3146]|07-SRNP-65514|669[0n]bp
Telemiades fides|[3147]|07-SRNP-65511|669[0n]bp
Telemiades fides|[3148]|07-SRNP-65572|669[0n]bp
Telemiades fides|[3149]|07-SRNP-59610|669[0n]bp
Telemiades fides|[3150]|07-SRNP-20495|669[0n]bp
Telemiades fides|[3151]|07-SRNP-3892|669[0n]bp
Telemiades fides|[3152]|07-SRNP-4701|669[0n]bp
Telemiades fides|[3153]|07-SRNP-4102|669[0n]bp
Telemiades fides|[3154]|07-SRNP-33424|669[0n]bp
Telemiades fides|[3155]|07-SRNP-3891|669[0n]bp
Telemiades fides|[3156]|07-SRNP-3839|669[0n]bp
Telemiades fides|[3157]|07-SRNP-3983|669[0n]bp
Telemiades fides|[3158]|07-SRNP-4100|669[0n]bp
Telemiades fides|[3159]|07-SRNP-65718|669[0n]bp
Telemiades fides|[3160]|07-SRNP-5003|669[0n]bp
Telemiades fides|[3161]|07-SRNP-5103|669[0n]bp
Telemiades fides|[3162]|07-SRNP-5105|669[0n]bp
Telemiades fides|[3163]|07-SRNP-5174|669[0n]bp
Telemiades fides|[3164]|08-SRNP-551|669[0n]bp
Telemiades fides|[3165]|07-SRNP-5111|669[0n]bp
Telemiades fides|[3166]|07-SRNP-5168|669[0n]bp
Telemiades fides|[3167]|07-SRNP-3984|669[0n]bp
Telemiades fides|[3168]|07-SRNP-42715|669[0n]bp
Telemiades fides|[3169]|07-SRNP-4405|669[0n]bp
Telemiades fides|[3170]|07-SRNP-3835|669[0n]bp
Telemiades fides|[3171]|07-SRNP-4291|669[0n]bp
Telemiades fides|[3172]|07-SRNP-5162|669[0n]bp
Telemiades fides|[3173]|07-SRNP-4770|669[0n]bp
Telemiades fides|[3174]|08-SRNP-129|669[0n]bp
Telemiades fides|[3175]|07-SRNP-5139|669[0n]bp
Telemiades fides|[3176]|07-SRNP-46433|669[0n]bp
Telemiades fides|[3177]|07-SRNP-4902|669[0n]bp
Telemiades fides|[3178]|08-SRNP-1791|669[0n]bp
Telemiades fides|[3179]|08-SRNP-20925|669[0n]bp
Telemiades fides|[3180]|08-SRNP-20965|669[0n]bp
Telemiades fides|[3181]|08-SRNP-21076|669[0n]bp
Telemiades fides|[3182]|08-SRNP-21078|669[0n]bp
Telemiades fides|[3183]|08-SRNP-20956|669[0n]bp
Telemiades fides|[3184]|08-SRNP-21028|669[0n]bp
Telemiades fides|[3185]|08-SRNP-21036|669[0n]bp
Telemiades fides|[3186]|08-SRNP-21077|669[0n]bp
Telemiades fides|[3187]|08-SRNP-21084|669[0n]bp
Telemiades fides|[3188]|08-SRNP-20967|669[0n]bp
Telemiades fides|[3189]|08-SRNP-21070|669[0n]bp
Telemiades fides|[3190]|08-SRNP-20494|669[0n]bp
Telemiades fides|[3191]|08-SRNP-65208|669[0n]bp
Telemiades fides|[3192]|08-SRNP-20966|669[0n]bp
Telemiades fides|[3193]|08-SRNP-21083|669[0n]bp
Telemiades fides|[3194]|08-SRNP-971|669[0n]bp
Telemiades fides|[3195]|08-SRNP-2340|669[0n]bp
Telemiades fides|[3196]|08-SRNP-65455|669[0n]bp
Telemiades fides|[3197]|08-SRNP-45069|669[0n]bp
Telemiades fides|[3198]|07-SRNP-24562|669[0n]bp
Telemiades fides|[3199]|08-SRNP-45067|669[0n]bp
Telemiades fides|[3200]|[illegible]

Telemiades fides|[3197]|08-SRNP-45069|669[0n]bp
Telemiades fides|[3198]|07-SRNP-24562|669[0n]bp
Telemiades fides|[3199]|08-SRNP-45067|669[0n]bp
Telemiades fides|[3200]|08-SRNP-55478|669[0n]bp
Telemiades fides|[3201]|08-SRNP-45144|669[0n]bp
Telemiades fides|[3202]|08-SRNP-2342|669[0n]bp
Telemiades fides|[3203]|07-SRNP-61016|669[0n]bp
Telemiades fides|[3204]|08-SRNP-1790|669[0n]bp
Telemiades fides|[3205]|08-SRNP-65277|669[0n]bp
Telemiades fides|[3206]|08-SRNP-45161|669[0n]bp
Telemiades fides|[3207]|07-SRNP-61357|669[0n]bp
Telemiades fides|[3208]|08-SRNP-21071|669[0n]bp
Telemiades fides|[3209]|08-SRNP-45065|669[0n]bp
Telemiades fides|[3210]|08-SRNP-65278|669[0n]bp
Telemiades fides|[3211]|08-SRNP-65292|669[0n]bp
Telemiades fides|[3212]|08-SRNP-65279|669[0n]bp
Telemiades fides|[3213]|08-SRNP-45093|669[0n]bp
Telemiades fides|[3214]|08-SRNP-45143|669[0n]bp
Telemiades fides|[3215]|07-SRNP-61184|669[0n]bp
Telemiades fides|[3216]|08-SRNP-21378|669[0n]bp
Telemiades fides|[3217]|08-SRNP-2123|669[0n]bp
Telemiades fides|[3218]|08-SRNP-40973|669[0n]bp
Telemiades fides|[3219]|08-SRNP-55982|669[0n]bp
Telemiades fides|[3220]|08-SRNP-2424|669[0n]bp
Telemiades fides|[3221]|08-SRNP-1346|669[0n]bp
Telemiades fides|[3222]|07-SRNP-61358|669[0n]bp
Telemiades fides|[3223]|08-SRNP-2357|669[0n]bp
Telemiades fides|[3224]|07-SRNP-60943|669[0n]bp
Telemiades fides|[3225]|08-SRNP-55069|669[0n]bp
Telemiades fides|[3226]|08-SRNP-55417|669[0n]bp
Telemiades fides|[3227]|08-SRNP-65209|669[0n]bp
Telemiades fides|[3228]|08-SRNP-65076|669[0n]bp
Telemiades fides|[3229]|08-SRNP-2338|669[0n]bp
Telemiades fides|[3230]|08-SRNP-2339|669[0n]bp
Telemiades fides|[3231]|08-SRNP-65207|669[0n]bp
Telemiades fides|[3232]|08-SRNP-700|669[0n]bp
Telemiades fides|[3233]|08-SRNP-65456|669[0n]bp
Telemiades fides|[3234]|08-SRNP-55442|669[0n]bp
Telemiades fides|[3235]|08-SRNP-1136|669[0n]bp
Telemiades fides|[3236]|08-SRNP-65115|669[0n]bp
Telemiades fides|[3237]|08-SRNP-55513|669[0n]bp
Telemiades fides|[3238]|02-SRNP-31483|669[0n]bp
Telemiades fides|[3239]|02-SRNP-33670|669[0n]bp
Telemiades fides|[3240]|02-SRNP-34173|669[0n]bp
Telemiades fides|[3241]|02-SRNP-5432|669[0n]bp
Telemiades fides|[3242]|02-SRNP-5434|669[0n]bp
Telemiades fides|[3243]|02-SRNP-31828|669[0n]bp
Telemiades fides|[3244]|02-SRNP-5700|669[0n]bp
Telemiades fides|[3245]|02-SRNP-27163|669[0n]bp
Telemiades fides|[3246]|02-SRNP-14146|669[0n]bp
Telemiades fides|[3247]|02-SRNP-5436|669[0n]bp
Telemiades fides|[3248]|02-SRNP-19106|669[0n]bp
Telemiades fides|[3249]|02-SRNP-18972|669[0n]bp
Telemiades fides|[3250]|02-SRNP-2328|669[0n]bp
Telemiades fides|[3251]|02-SRNP-2456|669[0n]bp
Telemiades fides|[3252]|03-SRNP-5752|669[0n]bp
Telemiades fides|[3253]|03-SRNP-5014|669[0n]bp
Telemiades fides|[3254]|03-SRNP-31075|669[0n]bp
Telemiades fides|[3255]|02-SRNP-20490|669[0n]bp
Telemiades fides|[3256]|02-SRNP-18846|669[1n]bp
Telemiades fides|[3257]|07-SRNP-4623|663[0n]bp
Telemiades fides|[3258]|07-SRNP-4756|663[0n]bp
Telemiades fides|[3259]|07-SRNP-429|669[2n]bp
Telemiades fides|[3260]|07-SRNP-20828|618[0n]bp
Telemiades fides|[3261]|08-SRNP-20412|621[0n]bp
Telemiades fides|[3262]|05-SRNP-45213|669[0n]bp
Telemiades fides|[3263]|07-SRNP-3837|657[0n]bp
Telemiades fides|[3264]|07-SRNP-33828|621[0n]bp
Telemiades fides|[3265]|07-SRNP-3730|615[0n]bp
Telemiades fides|[3266]|05-SRNP-55621|669[0n]bp
Telemiades fides|[3267]|96-SRNP-992|594[0n]bp
Telemiades fides|[3268]|00-SRNP-20860|606[0n]bp
Telemiades fides|[3269]|92-SRNP-6139|582[0n]bp
Telemiades fides|[3270]|02-SRNP-31421|651[0n]bp
Telemiades fides|[3271]|92-SRNP-5681|603[0n]bp
Telemiades fides|[3272]|03-SRNP-12110|618[0n]bp
Telemiades fides|[3273]|06-SRNP-12339|633[0n]bp
Telemiades fides|[3274]|07-SRNP-1494|594[0n]bp
Telemiades fides|[3275]|07-SRNP-4234|606[0n]bp
Telemiades fides|[3276]|07-SRNP-60566|606[0n]bp
Telemiades fides|[3277]|07-SRNP-30817|528[5n]bp
Telemiades fides|[3278]|08-SRNP-20211|633[0n]bp
Telemiades fides|[3279]|07-SRNP-42397|660[0n]bp
Telemiades fides|[3280]|07-SRNP-3474|660[0n]bp
Telemiades fides|[3281]|07-SRNP-4408|657[0n]bp
Telemiades fides|[3282]|08-SRNP-65408|621[0n]bp
Telemiades fides|[3283]|07-SRNP-4099|621[0n]bp
Telemiades fides|[3284]|07-SRNP-3733|645[0n]bp
Telemiades fides|[3285]|07-SRNP-5157|669[0n]bp
Telemiades fides|[3286]|07-SRNP-4492|669[0n]bp
Telemiades fides|[3287]|07-SRNP-24713|621[0n]bp
Telemiades fides|[3288]|08-SRNP-65206|669[0n]bp
Telemiades fides|[3289]|08-SRNP-55064|669[0n]bp
Telemiades fides|[3290]|08-SRNP-55444|669[0n]bp
Telemiades fides|[3291]|07-SRNP-60770|669[0n]bp
Telemiades fides|[3292]|08-SRNP-65185|669[0n]bp
Telemiades fides|[3293]|08-SRNP-2343|669[0n]bp
Telemiades fides|[3294]|07-SRNP-15871|669[0n]bp
Telemiades fides|[3295]|07-SRNP-16610|669[0n]bp
Telemiades fides|[3296]|08-SRNP-20930|669[0n]bp
Telemiades fides|[3297]|07-SRNP-31883|669[0n]bp
Telemiades fides|[3298]|07-SRNP-15746|669[0n]bp
Telemiades fides|[3299]|07-SRNP-16467|669[0n]bp
[illegible]

Telemiades fides|[3297]|07-SRNP-31883|669[0n]bp
Telemiades fides|[3298]|07-SRNP-15746|669[0n]bp
Telemiades fides|[3299]|07-SRNP-16467|669[0n]bp
Telemiades fides|[3300]|08-SRNP-5844|669[0n]bp
Telemiades fides|[3301]|08-SRNP-5513|669[0n]bp
Telemiades fides|[3302]|07-SRNP-30816|669[0n]bp
Telemiades fides|[3303]|07-SRNP-16643|669[0n]bp
Telemiades fides|[3304]|07-SRNP-16048|669[0n]bp
Telemiades fides|[3305]|07-SRNP-16680|669[0n]bp
Telemiades fides|[3306]|08-SRNP-66070|669[0n]bp
Telemiades fides|[3307]|08-SRNP-23199|669[0n]bp
Telemiades fides|[3308]|07-SRNP-20485|669[0n]bp
Telemiades fides|[3309]|07-SRNP-20483|669[0n]bp
Telemiades fides|[3310]|06-SRNP-23268|669[0n]bp
Telemiades fides|[3311]|06-SRNP-67814|669[0n]bp
Telemiades fides|[3312]|07-SRNP-55749|669[0n]bp
Telemiades fides|[3313]|06-SRNP-22405|669[0n]bp
Telemiades fides|[3314]|02-SRNP-14139|669[0n]bp
Telemiades fides|[3315]|02-SRNP-5368|669[0n]bp
Telemiades fides|[3316]|02-SRNP-10004|669[0n]bp
Telemiades fides|[3317]|02-SRNP-10052|669[0n]bp
Telemiades fides|[3318]|02-SRNP-13773|669[0n]bp
Telemiades fides|[3319]|01-SRNP-9333|669[0n]bp
Telemiades fides|[3320]|05-SRNP-55777|669[0n]bp
Telemiades fides|[3321]|04-SRNP-36226|669[0n]bp
Telemiades fides|[3322]|05-SRNP-2772|669[0n]bp
Telemiades fides|[3323]|05-SRNP-1858|669[0n]bp
Telemiades fides|[3324]|05-SRNP-32225|669[0n]bp
Telemiades fides|[3325]|05-SRNP-13190|669[0n]bp
Telemiades fides|[3326]|05-SRNP-55784|669[0n]bp
Telemiades fides|[3327]|05-SRNP-55783|669[0n]bp
Telemiades fides|[3328]|05-SRNP-1265|669[0n]bp
Telemiades fides|[3329]|05-SRNP-55774|669[0n]bp
Telemiades fides|[3330]|06-SRNP-12677|669[0n]bp
Telemiades fides|[3331]|06-SRNP-12708|669[0n]bp
Telemiades fides|[3332]|06-SRNP-60264|669[0n]bp
Telemiades fides|[3333]|06-SRNP-60266|669[0n]bp
Telemiades fides|[3334]|02-SRNP-1254|669[0n]bp
Telemiades fides|[3335]|02-SRNP-2194|669[0n]bp
Telemiades fides|[3336]|09-SRNP-65293|669[0n]bp
Telemiades fides|[3337]|09-SRNP-65344|669[0n]bp
Telemiades fides|[3338]|09-SRNP-44485|669[0n]bp
Telemiades fides|[3339]|09-SRNP-66013|669[0n]bp
Telemiades Burns02|[3340]|05-SRNP-40200|669[0n]bp
Telemiades Burns02|[3341]|05-SRNP-2164|669[0n]bp
Telemiades Burns02|[3342]|05-SRNP-33036|669[0n]bp
Telemiades Burns02|[3343]|05-SRNP-43775|669[0n]bp
Telemiades Burns02|[3344]|07-SRNP-3595|669[0n]bp
Telemiades gallius|[3345]|00-SRNP-4456|639[0n]bp
Telemiades gallius|[3346]|99-SRNP-13875|576[0n]bp
Telemiades chrysorrhoeaDHJ01|[3347]|03-SRNP-3888|540[0n]bp
Telemiades chrysorrhoeaDHJ01|[3348]|02-SRNP-23372|570[0n]bp
Telemiades chrysorrhoeaDHJ01|[3349]|02-SRNP-23231|669[3n]bp
Telemiades chrysorrhoeaDHJ01|[3350]|04-SRNP-35467|510[1n]bp
Telemiades chrysorrhoeaDHJ01|[3351]|03-SRNP-4126|621[0n]bp
Telemiades chrysorrhoeaDHJ01|[3352]|03-SRNP-4445|621[0n]bp
Telemiades chrysorrhoeaDHJ01|[3353]|03-SRNP-3666|621[0n]bp
Telemiades chrysorrhoeaDHJ01|[3354]|03-SRNP-4745|621[0n]bp
Telemiades chrysorrhoeaDHJ01|[3355]|03-SRNP-4261|621[0n]bp
Telemiades chrysorrhoeaDHJ01|[3356]|02-SRNP-9407|621[0n]bp
Telemiades chrysorrhoeaDHJ01|[3357]|02-SRNP-9730|621[0n]bp
Telemiades chrysorrhoeaDHJ01|[3358]|02-SRNP-9405|621[0n]bp
Telemiades chrysorrhoeaDHJ01|[3359]|02-SRNP-9230|621[0n]bp
Telemiades chrysorrhoeaDHJ01|[3360]|07-SRNP-36106|642[0n]bp
Telemiades chrysorrhoeaDHJ01|[3361]|00-SRNP-9621|669[1n]bp
Telemiades chrysorrhoeaDHJ01|[3362]|00-SRNP-9804|669[0n]bp
Telemiades chrysorrhoeaDHJ01|[3363]|01-SRNP-6022|669[0n]bp
Telemiades chrysorrhoeaDHJ01|[3364]|02-SRNP-8012|669[0n]bp
Telemiades chrysorrhoeaDHJ01|[3365]|01-SRNP-6829|669[0n]bp
Telemiades chrysorrhoeaDHJ01|[3366]|03-SRNP-4023|669[0n]bp
Telemiades chrysorrhoeaDHJ01|[3367]|03-SRNP-4298|669[0n]bp
Telemiades chrysorrhoeaDHJ01|[3368]|03-SRNP-4631|669[0n]bp
Telemiades chrysorrhoeaDHJ01|[3369]|01-SRNP-6010|669[0n]bp
Telemiades chrysorrhoeaDHJ01|[3370]|03-SRNP-4020|585[1n]bp
Telemiades chrysorrhoeaDHJ01|[3371]|03-SRNP-4299|669[0n]bp
Telemiades chrysorrhoeaDHJ01|[3372]|02-SRNP-23178|600[0n]bp
Telemiades chrysorrhoeaDHJ01|[3373]|02-SRNP-23351|600[1n]bp
Telemiades chrysorrhoeaDHJ01|[3374]|02-SRNP-23358|621[0n]bp
Telemiades chrysorrhoeaDHJ01|[3375]|02-SRNP-23070|585[0n]bp
Telemiades chrysorrhoeaDHJ01|[3376]|03-SRNP-4448|585[0n]bp
Telemiades chrysorrhoeaDHJ01|[3377]|02-SRNP-9520|585[0n]bp
Telemiades chrysorrhoeaDHJ01|[3378]|03-SRNP-4025|585[0n]bp
Telemiades chrysorrhoeaDHJ01|[3379]|02-SRNP-9560|585[0n]bp
Telemiades chrysorrhoeaDHJ01|[3380]|02-SRNP-9227|585[0n]bp
Telemiades chrysorrhoeaDHJ01|[3381]|02-SRNP-8011|354[0n]bp
Telemiades chrysorrhoeaDHJ01|[3382]|03-SRNP-4743|357[0n]bp
Telemiades chrysorrhoeaDHJ01|[3383]|01-SRNP-6170|609[0n]bp
Telemiades chrysorrhoeaDHJ01|[3384]|04-SRNP-35573|669[0n]bp
Telemiades chrysorrhoeaDHJ01|[3385]|08-SRNP-35603|669[0n]bp
Telemiades chrysorrhoeaDHJ01|[3386]|08-SRNP-35580|669[0n]bp
Telemiades chrysorrhoeaDHJ01|[3387]|08-SRNP-35605|669[0n]bp
Telemiades chrysorrhoeaDHJ02|[3388]|03-SRNP-4636|612[5n]bp
Telemiades chrysorrhoeaDHJ02|[3389]|03-SRNP-4892|612[8n]bp
Telemiades chrysorrhoeaDHJ02|[3390]|08-SRNP-35601|669[1n]bp
Telemiades chrysorrhoeaDHJ02|[3391]|02-SRNP-8074|588[8n]bp
Telemiades chrysorrhoeaDHJ02|[3392]|03-SRNP-4837|612[6n]bp
Telemiades chrysorrhoeaDHJ02|[3393]|02-SRNP-23202|675[6n]bp
Telemiades chrysorrhoeaDHJ02|[3394]|08-SRNP-35613|669[0n]bp
Telemiades chrysorrhoeaDHJ02|[3395]|08-SRNP-35384|669[0n]bp
Telemiades chrysorrhoeaDHJ02|[3396]|08-SRNP-35968|669[0n]bp
Telemiades chrysorrhoeaDHJ02|[3397]|08-SRNP-35614|669[0n]bp
Telemiades chrysorrhoeaDHJ02|[3398]|08-SRNP-35579|669[0n]bp
Telemiades chrysorrhoeaDHJ02|[3399]|08-SRNP-35966|669[0n]bp
[illegible]

Telemiades chrysorrhoeaDHJ02|[3397]|08-SRNP-35614|669[0n]bp
Telemiades chrysorrhoeaDHJ02|[3398]|08-SRNP-35579|669[0n]bp
Telemiades chrysorrhoeaDHJ02|[3399]|08-SRNP-35966|669[0n]bp
Telemiades chrysorrhoeaDHJ02|[3400]|03-SRNP-4632|612[1n]bp
Telemiades chrysorrhoeaDHJ02|[3401]|03-SRNP-4447|576[5n]bp
Telemiades chrysorrhoeaDHJ02|[3402]|02-SRNP-9408|609[1n]bp
Telemiades chrysorrhoeaDHJ02|[3403]|02-SRNP-9930|615[1n]bp
Telemiades chrysorrhoeaDHJ02|[3404]|03-SRNP-4026|633[3n]bp
Telemiades chrysorrhoeaDHJ02|[3405]|03-SRNP-4297|585[5n]bp
Telemiades chrysorrhoeaDHJ02|[3406]|03-SRNP-4742|675[2n]bp
Telemiades chrysorrhoeaDHJ02|[3407]|03-SRNP-3440|630[2n]bp
Telemiades chrysorrhoeaDHJ02|[3408]|03-SRNP-3441|645[1n]bp
Telemiades chrysorrhoeaDHJ02|[3409]|02-SRNP-23096|645[1n]bp
Telemiades chrysorrhoeaDHJ02|[3410]|02-SRNP-23179|639[1n]bp
Telemiades chrysorrhoeaDHJ02|[3411]|00-SRNP-9561|675[0n]bp
Telemiades chrysorrhoeaDHJ02|[3412]|01-SRNP-6918|675[2n]bp
Telemiades chrysorrhoeaDHJ02|[3413]|02-SRNP-9664|675[1n]bp
Telemiades chrysorrhoeaDHJ02|[3414]|01-SRNP-6864|675[3n]bp
Telemiades chrysorrhoeaDHJ02|[3415]|02-SRNP-9445|588[1n]bp
Telemiades chrysorrhoeaDHJ02|[3416]|02-SRNP-9929|588[0n]bp
Telemiades chrysorrhoeaDHJ02|[3417]|02-SRNP-23273|588[0n]bp
Telemiades chrysorrhoeaDHJ02|[3418]|02-SRNP-23201|618[0n]bp
Telemiades chrysorrhoeaDHJ02|[3419]|08-SRNP-37068|642[5n]bp
Telemiades megallus|[3420]|08-SRNP-66068|669[0n]bp
Telemiades megallus|[3421]|08-SRNP-40055|654[0n]bp
Telemiades megallus|[3422]|07-SRNP-42824|645[0n]bp
Telemiades megallus|[3423]|03-SRNP-31164|492[0n]bp
Telemiades megallus|[3424]|07-SRNP-3719|669[0n]bp
Telemiades megallus|[3425]|08-SRNP-40056|651[0n]bp
Telemiades megallus|[3426]|07-SRNP-42778|642[0n]bp
Telemiades megallus|[3427]|07-SRNP-42752|642[0n]bp
Telemiades megallus|[3428]|07-SRNP-33307|642[0n]bp
Telemiades megallus|[3429]|07-SRNP-24437|621[0n]bp
Telemiades megallus|[3430]|07-SRNP-43135|645[2n]bp
Telemiades megallus|[3431]|07-SRNP-43082|669[0n]bp
Telemiades megallus|[3432]|09-SRNP-69855|669[0n]bp
Telemiades megallus|[3433]|07-SRNP-42971|669[0n]bp
Telemiades megallus|[3434]|07-SRNP-42910|669[0n]bp
Telemiades megallus|[3435]|07-SRNP-3809|669[0n]bp
Telemiades megallus|[3436]|07-SRNP-42969|669[0n]bp
Telemiades megallus|[3437]|07-SRNP-33308|669[0n]bp
Telemiades megallus|[3438]|07-SRNP-41026|669[0n]bp
Telemiades megallus|[3439]|07-SRNP-30422|669[0n]bp
Telemiades megallus|[3440]|05-SRNP-24526|669[0n]bp
Telemiades megallus|[3441]|04-SRNP-111|639[0n]bp
Telemiades megallus|[3442]|03-SRNP-30710|492[0n]bp
Telemiades megallus|[3443]|07-SRNP-42440|660[0n]bp
Telemiades megallus|[3444]|00-SRNP-21554|624[0n]bp
Telemiades megallus|[3445]|07-SRNP-4414|621[0n]bp
Telemiades megallus|[3446]|07-SRNP-42987|648[0n]bp
Telemiades megallus|[3447]|07-SRNP-5171|630[0n]bp
Telemiades megallus|[3448]|07-SRNP-42879|663[0n]bp
Telemiades megallus|[3449]|09-SRNP-69856|633[0n]bp
Telemiades oiclus|[3450]|03-SRNP-7387|669[0n]bp
Telemiades oiclus|[3451]|05-SRNP-5462|687[0n]bp
Telemiades oiclus|[3452]|03-SRNP-12536.1|669[0n]bp
Telemiades oiclus|[3453]|04-SRNP-30865|669[0n]bp
Telemiades oiclus|[3454]|04-SRNP-55048|669[0n]bp
Telemiades oiclus|[3455]|04-SRNP-56957|669[0n]bp
Telemiades oiclus|[3456]|04-SRNP-56954|669[0n]bp
Telemiades oiclus|[3457]|03-SRNP-7308|663[0n]bp
Telemiades oiclus|[3458]|01-SRNP-3727|663[0n]bp
Telemiades oiclus|[3459]|03-SRNP-12588.1|576[3n]bp
Telemiades oiclus|[3460]|03-SRNP-20865|636[0n]bp
Telemiades oiclus|[3461]|00-SRNP-22202|648[1n]bp
Telemiades oiclus|[3462]|01-SRNP-2947|669[0n]bp
Telemiades oiclus|[3463]|07-SRNP-2201|663[0n]bp
Telemiades oiclus|[3464]|01-SRNP-25089|669[0n]bp
Telemiades oiclus|[3465]|05-SRNP-42621|669[0n]bp
Telemiades oiclus|[3466]|07-SRNP-2119|645[0n]bp
Telemiades oiclus|[3467]|07-SRNP-1786|669[0n]bp
Telemiades oiclus|[3468]|07-SRNP-2582|669[0n]bp
Telemiades oiclus|[3469]|07-SRNP-33526|669[0n]bp
Telemiades nicomedes|[3470]|07-SRNP-42342|669[0n]bp
Telemiades nicomedes|[3471]|05-SRNP-33522|630[0n]bp
Telemiades nicomedes|[3472]|06-SRNP-42935|669[0n]bp
Telemiades nicomedes|[3473]|05-SRNP-33746|687[0n]bp
Telemiades nicomedes|[3474]|03-SRNP-21802|663[0n]bp
Telemiades nicomedes|[3475]|03-SRNP-27835|384[0n]bp
Telemiades nicomedes|[3476]|04-SRNP-2174|585[0n]bp
Telemiades nicomedes|[3477]|05-SRNP-43154|669[0n]bp
Telemiades nicomedes|[3478]|06-SRNP-31788|669[0n]bp
Telemiades nicomedes|[3479]|06-SRNP-31705|669[0n]bp
Telemiades nicomedes|[3480]|06-SRNP-43594|669[0n]bp
Telemiades nicomedes|[3481]|06-SRNP-65735|669[0n]bp
Telemiades nicomedes|[3482]|07-SRNP-42490|669[0n]bp
Telemiades nicomedes|[3483]|08-SRNP-65358|669[0n]bp
Telemiades nicomedes|[3484]|08-SRNP-65381|669[0n]bp
Telemiades nicomedes|[3485]|08-SRNP-40801|669[0n]bp
Telemiades nicomedes|[3486]|08-SRNP-22689|669[0n]bp
Telemiades nicomedes|[3487]|08-SRNP-22691|669[0n]bp
Telemiades nicomedes|[3488]|08-SRNP-2225|669[0n]bp
Telemiades nicomedes|[3489]|05-SRNP-33923|627[0n]bp
Telemiades nicomedes|[3490]|06-SRNP-44852|669[0n]bp
Telemiades nicomedes|[3491]|05-SRNP-34684|669[0n]bp
Telemiades nicomedes|[3492]|03-SRNP-38197|645[0n]bp
Telemiades nicomedes|[3493]|08-SRNP-40117|642[0n]bp
Telemiades nicomedes|[3494]|08-SRNP-66200|669[0n]bp
Telemiades nicomedes|[3495]|08-SRNP-72830|669[0n]bp
Telemiades Burns01|[3496]|98-SRNP-6688|480[0n]bp
Telemiades Burns01|[3497]|02-SRNP-19058|489[1n]bp
Telemiades Burns01|[3498]|07-SRNP-4410|645[0n]bp
Telemiades Burns01|[3499]|07-SRNP-3801|645[0n]bp
Telemiades Burns01|[3500]|07-SRNP-[illegible]

Telemiades Burns01|[3497]|02-SRNP-19058|489[1n]bp
Telemiades Burns01|[3498]|07-SRNP-4410|645[0n]bp
Telemiades Burns01|[3499]|07-SRNP-3801|645[0n]bp
Telemiades Burns01|[3500]|07-SRNP-3542|669[0n]bp
Telemiades Burns01|[3501]|07-SRNP-3804|669[0n]bp
Telemiades Burns01|[3502]|07-SRNP-3803|669[0n]bp
Telemiades Burns01|[3503]|07-SRNP-2223|669[0n]bp
Telemiades Burns01|[3504]|07-SRNP-1335|669[0n]bp
Telemiades Burns01|[3505]|02-SRNP-19057|669[0n]bp
Telemiades Burns01|[3506]|03-SRNP-13003.1|669[0n]bp
Telemiades Burns01|[3507]|07-SRNP-3807|621[0n]bp
Telemiades Burns01|[3508]|07-SRNP-4083|669[0n]bp
Telemiades Burns01|[3509]|07-SRNP-4699|669[0n]bp
Telemiades Burns01|[3510]|08-SRNP-79|669[0n]bp
Telemiades Burns01|[3511]|08-SRNP-80|669[0n]bp
Telemiades Burns01|[3512]|08-SRNP-941|669[0n]bp
Telemiades Burns01|[3513]|08-SRNP-41014|669[0n]bp
Telemiades Burns01|[3514]|08-SRNP-2336|669[0n]bp
Telemiades Burns01|[3515]|02-SRNP-18967|669[0n]bp
Telemiades Burns01|[3516]|07-SRNP-3519|669[0n]bp
Telemiades Burns01|[3517]|07-SRNP-4034|669[0n]bp
Telemiades Burns01|[3518]|08-SRNP-1107|669[0n]bp
Telemiades Burns08|[3519]|08-SRNP-65335|669[0n]bp
Telemiades Burns08|[3520]|09-SRNP-65738|669[0n]bp
Telemiades Burns08|[3521]|09-SRNP-65754|669[0n]bp
Telemiades antiopeDHJ04|[3522]|03-SRNP-12790.1|576[0n]bp
Telemiades antiopeDHJ04|[3523]|08-SRNP-65458|621[0n]bp
Telemiades antiopeDHJ04|[3524]|07-SRNP-5113|606[0n]bp
Telemiades antiopeDHJ04|[3525]|08-SRNP-40546|669[0n]bp
Telemiades antiopeDHJ04|[3526]|08-SRNP-20658|669[0n]bp
Telemiades antiopeDHJ04|[3527]|08-SRNP-2356|669[0n]bp
Telemiades antiopeDHJ04|[3528]|07-SRNP-5149|669[0n]bp
Telemiades antiopeDHJ04|[3529]|08-SRNP-213|669[0n]bp
Telemiades antiopeDHJ04|[3530]|07-SRNP-4662|669[0n]bp
Telemiades antiopeDHJ04|[3531]|07-SRNP-42683|669[0n]bp
Telemiades antiopeDHJ04|[3532]|08-SRNP-25|669[0n]bp
Telemiades antiopeDHJ04|[3533]|07-SRNP-65997|669[0n]bp
Telemiades antiopeDHJ04|[3534]|07-SRNP-4830|669[0n]bp
Telemiades antiopeDHJ04|[3535]|08-SRNP-90|669[0n]bp
Telemiades antiopeDHJ04|[3536]|08-SRNP-40014|669[0n]bp
Telemiades antiopeDHJ04|[3537]|08-SRNP-78|669[0n]bp
Telemiades antiopeDHJ04|[3538]|07-SRNP-4413|669[0n]bp
Telemiades antiopeDHJ04|[3539]|07-SRNP-4181|669[0n]bp
Telemiades antiopeDHJ04|[3540]|07-SRNP-66030|669[0n]bp
Telemiades antiopeDHJ04|[3541]|02-SRNP-5610|669[0n]bp
Telemiades antiopeDHJ04|[3542]|07-SRNP-4622|666[0n]bp
Telemiades antiopeDHJ04|[3543]|08-SRNP-77|624[1n]bp
Telemiades antiopeDHJ04|[3544]|07-SRNP-3260|657[0n]bp
Telemiades antiopeDHJ04|[3545]|07-SRNP-5121|648[1n]bp
Telemiades antiopeDHJ04|[3546]|02-SRNP-31972|594[0n]bp
Telemiades antiopeDHJ04|[3547]|08-SRNP-20471|642[0n]bp
Telemiades antiopeDHJ04|[3548]|08-SRNP-65366|669[0n]bp
Telemiades antiopeDHJ04|[3549]|08-SRNP-20470|669[0n]bp
Telemiades antiopeDHJ04|[3550]|08-SRNP-85|669[0n]bp
Telemiades antiopeDHJ04|[3551]|08-SRNP-22138|669[0n]bp
Telemiades antiopeDHJ04|[3552]|08-SRNP-66178|669[0n]bp
Telemiades antiopeDHJ04|[3553]|08-SRNP-66078|669[0n]bp
Telemiades antiopeDHJ04|[3554]|08-SRNP-32462|669[0n]bp
Telemiades antiopeDHJ02|[3555]|08-SRNP-32857|633[0n]bp
Telemiades antiopeDHJ02|[3556]|02-SRNP-19299|582[0n]bp
Telemiades antiopeDHJ02|[3557]|07-SRNP-32031|669[0n]bp
Telemiades antiopeDHJ02|[3558]|07-SRNP-4321|669[0n]bp
Telemiades antiopeDHJ02|[3559]|08-SRNP-31060|669[0n]bp
Telemiades antiopeDHJ02|[3560]|08-SRNP-66086|669[0n]bp
Telemiades antiopeDHJ02|[3561]|08-SRNP-66041|669[0n]bp
Telemiades antiopeDHJ02|[3562]|01-SRNP-25374|621[0n]bp
Telemiades antiopeDHJ02|[3563]|07-SRNP-4412|669[0n]bp
Telemiades antiopeDHJ02|[3564]|07-SRNP-4814|669[0n]bp
Telemiades antiopeDHJ02|[3565]|07-SRNP-4822|669[0n]bp
Telemiades antiopeDHJ02|[3566]|07-SRNP-3864|669[0n]bp
Telemiades antiopeDHJ02|[3567]|06-SRNP-9304|669[0n]bp
Telemiades antiopeDHJ02|[3568]|00-SRNP-14600|669[0n]bp
Telemiades antiopeDHJ02|[3569]|07-SRNP-42387|660[0n]bp
Telemiades antiopeDHJ02|[3570]|07-SRNP-45352|660[0n]bp
Telemiades antiopeDHJ02|[3571]|07-SRNP-4821|663[0n]bp
Telemiades antiopeDHJ02|[3572]|07-SRNP-42887|669[0n]bp
Telemiades antiopeDHJ02|[3573]|07-SRNP-3822|669[0n]bp
Telemiades antiopeDHJ02|[3574]|08-SRNP-2038|669[0n]bp
Telemiades antiopeDHJ02|[3575]|08-SRNP-40378|669[0n]bp
Telemiades antiopeDHJ02|[3576]|08-SRNP-31062|669[0n]bp
Telemiades antiopeDHJ02|[3577]|08-SRNP-1018|669[0n]bp
Telemiades antiopeDHJ02|[3578]|08-SRNP-5514|669[0n]bp
Telemiades antiopeDHJ02|[3579]|08-SRNP-72542|669[0n]bp
Telemiades antiopeDHJ02|[3580]|09-SRNP-40086|576[0n]bp
Telemiades antiopeDHJ02|[3581]|07-SRNP-1844|645[0n]bp
Telemiades antiopeDHJ02|[3582]|02-SRNP-1005|621[0n]bp
Telemiades antiopeDHJ02|[3583]|02-SRNP-1008|621[0n]bp
Telemiades antiopeDHJ02|[3584]|02-SRNP-1004|621[0n]bp
Telemiades antiopeDHJ02|[3585]|02-SRNP-1451|621[0n]bp
Telemiades antiopeDHJ02|[3586]|08-SRNP-41239|669[0n]bp
Telemiades antiopeDHJ02|[3587]|08-SRNP-1347|669[0n]bp
Telemiades antiopeDHJ02|[3588]|08-SRNP-40203|669[0n]bp
Telemiades antiopeDHJ02|[3589]|08-SRNP-30634|669[0n]bp
Telemiades antiopeDHJ02|[3590]|08-SRNP-40136|669[0n]bp
Telemiades antiopeDHJ02|[3591]|08-SRNP-520|669[0n]bp
Telemiades antiopeDHJ02|[3592]|08-SRNP-2122|669[0n]bp
Telemiades antiopeDHJ02|[3593]|08-SRNP-75|669[0n]bp
Telemiades antiopeDHJ02|[3594]|07-SRNP-4808|669[0n]bp
Telemiades antiopeDHJ02|[3595]|07-SRNP-4177|669[0n]bp
Telemiades antiopeDHJ02|[3596]|07-SRNP-4810|669[0n]bp
Telemiades antiopeDHJ02|[3597]|07-SRNP-4465|669[0n]bp
Telemiades antiopeDHJ02|[3598]|07-SRNP-5159|669[0n]bp
Telemiades antiopeDHJ02|[3599]|07-SRNP-4154|669[0n]bp
[illegible]

Telemiades antiopeDHJ02|[3597]|07-SRNP-4465|669[0n]bp
Telemiades antiopeDHJ02|[3598]|07-SRNP-5159|669[0n]bp
Telemiades antiopeDHJ02|[3599]|07-SRNP-4154|669[0n]bp
Telemiades antiopeDHJ02|[3600]|02-SRNP-1007|669[0n]bp
Telemiades antiopeDHJ02|[3601]|02-SRNP-19206|669[0n]bp
Telemiades antiopeDHJ02|[3602]|02-SRNP-4634|669[0n]bp
Telemiades antiopeDHJ02|[3603]|07-SRNP-30338|669[0n]bp
Telemiades antiopeDHJ02|[3604]|07-SRNP-3292|666[0n]bp
Telemiades antiopeDHJ02|[3605]|07-SRNP-42386|660[0n]bp
Telemiades antiopeDHJ02|[3606]|07-SRNP-42388|657[0n]bp
Telemiades antiopeDHJ02|[3607]|02-SRNP-1638|540[0n]bp
Telemiades antiopeDHJ02|[3608]|02-SRNP-1003|642[0n]bp
Telemiades antiopeDHJ02|[3609]|02-SRNP-19728|651[0n]bp
Telemiades antiopeDHJ02|[3610]|09-SRNP-65787|606[0n]bp
Telemiades antiopeDHJ02|[3611]|09-SRNP-69681|669[0n]bp
Telemiades antiopeDHJ03|[3612]|03-SRNP-5635|639[1n]bp
Telemiades antiopeDHJ03|[3613]|07-SRNP-3532|654[0n]bp
Telemiades antiopeDHJ03|[3614]|07-SRNP-5153|669[0n]bp
Telemiades antiopeDHJ03|[3615]|08-SRNP-56|669[0n]bp
Telemiades antiopeDHJ03|[3616]|07-SRNP-4621|669[0n]bp
Telemiades antiopeDHJ03|[3617]|08-SRNP-88|669[0n]bp
Telemiades antiopeDHJ03|[3618]|07-SRNP-65881|669[0n]bp
Telemiades antiopeDHJ03|[3619]|07-SRNP-4322|669[0n]bp
Telemiades antiopeDHJ03|[3620]|07-SRNP-65754|669[0n]bp
Telemiades antiopeDHJ03|[3621]|07-SRNP-2288|669[0n]bp
Telemiades antiopeDHJ03|[3622]|05-SRNP-31699|669[0n]bp
Telemiades antiopeDHJ03|[3623]|07-SRNP-4816|663[0n]bp
Telemiades antiopeDHJ03|[3624]|07-SRNP-3834|660[0n]bp
Telemiades antiopeDHJ03|[3625]|07-SRNP-3258|660[0n]bp
Telemiades antiopeDHJ03|[3626]|08-SRNP-53|651[1n]bp
Telemiades antiopeDHJ03|[3627]|08-SRNP-94|645[0n]bp
Telemiades antiopeDHJ03|[3628]|08-SRNP-212|669[0n]bp
Telemiades antiopeDHJ03|[3629]|08-SRNP-65345|669[0n]bp
Telemiades antiopeDHJ03|[3630]|08-SRNP-2235|669[0n]bp
Telemiades antiopeDHJ03|[3631]|08-SRNP-65187|669[0n]bp
Telemiades antiopeDHJ03|[3632]|08-SRNP-2052|669[0n]bp
Telemiades antiopeDHJ03|[3633]|08-SRNP-1022|669[0n]bp
Telemiades antiopeDHJ03|[3634]|08-SRNP-5335|669[0n]bp
Telemiades antiopeDHJ03|[3635]|09-SRNP-80154|669[0n]bp
Telemiades antiopeDHJ01|[3636]|03-SRNP-5756|639[0n]bp
Telemiades antiopeDHJ01|[3637]|03-SRNP-5262|630[0n]bp
Telemiades antiopeDHJ01|[3638]|07-SRNP-4829|621[0n]bp
Telemiades antiopeDHJ01|[3639]|07-SRNP-42569|621[0n]bp
Telemiades antiopeDHJ01|[3640]|07-SRNP-33538|621[0n]bp
Telemiades antiopeDHJ01|[3641]|02-SRNP-882|621[0n]bp
Telemiades antiopeDHJ01|[3642]|02-SRNP-19458|648[0n]bp
Telemiades antiopeDHJ01|[3643]|07-SRNP-4743|669[0n]bp
Telemiades antiopeDHJ01|[3644]|07-SRNP-5126|669[0n]bp
Telemiades antiopeDHJ01|[3645]|07-SRNP-4700|669[0n]bp
Telemiades antiopeDHJ01|[3646]|07-SRNP-4317|669[0n]bp
Telemiades antiopeDHJ01|[3647]|07-SRNP-4480|669[0n]bp
Telemiades antiopeDHJ01|[3648]|07-SRNP-4666|669[0n]bp
Telemiades antiopeDHJ01|[3649]|07-SRNP-41772|669[0n]bp
Telemiades antiopeDHJ01|[3650]|07-SRNP-41859|669[0n]bp
Telemiades antiopeDHJ01|[3651]|07-SRNP-32623|669[0n]bp
Telemiades antiopeDHJ01|[3652]|05-SRNP-2943|669[0n]bp
Telemiades antiopeDHJ01|[3653]|05-SRNP-31617|669[0n]bp
Telemiades antiopeDHJ01|[3654]|05-SRNP-31616|669[0n]bp
Telemiades antiopeDHJ01|[3655]|06-SRNP-22168|669[0n]bp
Telemiades antiopeDHJ01|[3656]|02-SRNP-19799|669[0n]bp
Telemiades antiopeDHJ01|[3657]|02-SRNP-31830|669[0n]bp
Telemiades antiopeDHJ01|[3658]|97-SRNP-6271|669[0n]bp
Telemiades antiopeDHJ01|[3659]|98-SRNP-4297|669[0n]bp
Telemiades antiopeDHJ01|[3660]|01-SRNP-9439|669[0n]bp
Telemiades antiopeDHJ01|[3661]|98-SRNP-4296|669[0n]bp
Telemiades antiopeDHJ01|[3662]|07-SRNP-65162|669[0n]bp
Telemiades antiopeDHJ01|[3663]|07-SRNP-4438|666[0n]bp
Telemiades antiopeDHJ01|[3664]|08-SRNP-74|660[2n]bp
Telemiades antiopeDHJ01|[3665]|07-SRNP-3358|666[0n]bp
Telemiades antiopeDHJ01|[3666]|07-SRNP-5012|660[0n]bp
Telemiades antiopeDHJ01|[3667]|07-SRNP-3831|660[0n]bp
Telemiades antiopeDHJ01|[3668]|07-SRNP-3359|660[0n]bp
Telemiades antiopeDHJ01|[3669]|02-SRNP-31831|603[0n]bp
Telemiades antiopeDHJ01|[3670]|02-SRNP-19598|579[0n]bp
Telemiades antiopeDHJ01|[3671]|02-SRNP-19126|630[0n]bp
Telemiades antiopeDHJ01|[3672]|02-SRNP-6041|585[0n]bp
Telemiades antiopeDHJ01|[3673]|01-SRNP-23390|594[0n]bp
Telemiades antiopeDHJ01|[3674]|07-SRNP-4015|384[0n]bp
Telemiades antiopeDHJ01|[3675]|07-SRNP-4768|651[1n]bp
Telemiades antiopeDHJ01|[3676]|07-SRNP-5014|633[0n]bp
Telemiades antiopeDHJ01|[3677]|07-SRNP-4178|669[0n]bp
Telemiades antiopeDHJ01|[3678]|07-SRNP-4705|669[0n]bp
Telemiades antiopeDHJ01|[3679]|07-SRNP-4179|669[0n]bp
Telemiades antiopeDHJ01|[3680]|08-SRNP-72|669[0n]bp
Telemiades antiopeDHJ01|[3681]|08-SRNP-84|669[0n]bp
Telemiades antiopeDHJ01|[3682]|07-SRNP-4472|669[0n]bp
Telemiades antiopeDHJ01|[3683]|07-SRNP-4747|669[0n]bp
Telemiades antiopeDHJ01|[3684]|07-SRNP-5047|669[0n]bp
Telemiades antiopeDHJ01|[3685]|07-SRNP-4146|669[0n]bp
Telemiades antiopeDHJ01|[3686]|07-SRNP-4186|669[0n]bp
Telemiades antiopeDHJ01|[3687]|07-SRNP-4665|669[0n]bp
Telemiades antiopeDHJ01|[3688]|07-SRNP-65996|669[0n]bp
Telemiades antiopeDHJ01|[3689]|07-SRNP-4466|669[0n]bp
Telemiades antiopeDHJ01|[3690]|07-SRNP-42746|669[0n]bp
Telemiades antiopeDHJ01|[3691]|07-SRNP-4202|669[0n]bp
Telemiades antiopeDHJ01|[3692]|08-SRNP-65077|669[0n]bp
Telemiades antiopeDHJ01|[3693]|08-SRNP-2032|669[0n]bp
Telemiades antiopeDHJ01|[3694]|08-SRNP-20474|669[0n]bp
Telemiades antiopeDHJ01|[3695]|08-SRNP-241|669[0n]bp
Telemiades antiopeDHJ01|[3696]|08-SRNP-21085|669[0n]bp
Telemiades antiopeDHJ01|[3697]|07-SRNP-23802|669[0n]bp
Telemiades antiopeDHJ01|[3698]|07-SRNP-4183|669[0n]bp
Telemiades antiopeDHJ01|[3699]|07-SRNP-3832|669[0n]bp
[illegible]

Telemiades antiopeDHJ01|[3697]|07-SRNP-23802|669[0n]bp
Telemiades antiopeDHJ01|[3698]|07-SRNP-4183|669[0n]bp
Telemiades antiopeDHJ01|[3699]|07-SRNP-3832|669[0n]bp
Telemiades antiopeDHJ01|[3700]|07-SRNP-42570|669[0n]bp
Telemiades antiopeDHJ01|[3701]|08-SRNP-593|669[0n]bp
Telemiades antiopeDHJ01|[3702]|08-SRNP-31223|669[0n]bp
Telemiades antiopeDHJ01|[3703]|07-SRNP-4831|669[0n]bp
Telemiades antiopeDHJ01|[3704]|08-SRNP-1861|669[0n]bp
Telemiades antiopeDHJ01|[3705]|08-SRNP-65346|669[0n]bp
Telemiades antiopeDHJ01|[3706]|08-SRNP-2236|669[0n]bp
Telemiades antiopeDHJ01|[3707]|08-SRNP-40725|669[0n]bp
Telemiades antiopeDHJ01|[3708]|08-SRNP-1849|669[0n]bp
Telemiades antiopeDHJ01|[3709]|08-SRNP-66184|669[0n]bp
Telemiades antiopeDHJ01|[3710]|08-SRNP-22019|669[0n]bp
Telemiades antiopeDHJ01|[3711]|09-SRNP-66051|669[0n]bp
Telemiades antiopeDHJ01|[3712]|09-SRNP-44901|669[0n]bp
Telemiades Burns03|[3713]|06-SRNP-43497|669[0n]bp
Cogia eluina|[3714]|04-SRNP-50096|489[0n]bp
Cogia eluina|[3715]|04-SRNP-50103|333[2n]bp
Cogia eluina|[3716]|05-SRNP-64296|645[0n]bp
Cogia eluina|[3717]|05-SRNP-64303|600[0n]bp
Cogia eluina|[3718]|01-SRNP-16128|645[0n]bp
Cogia eluina|[3719]|02-SRNP-32045|645[3n]bp
Cogia eluina|[3720]|04-SRNP-15978|627[0n]bp
Cogia eluina|[3721]|02-SRNP-17121|645[0n]bp
Cogia eluina|[3722]|91-SRNP-1362|576[0n]bp
Cogia eluina|[3723]|04-SRNP-16107|669[0n]bp
Cogia eluina|[3724]|06-SRNP-21562|669[0n]bp
Cogia eluina|[3725]|05-SRNP-56836|669[0n]bp
Cogia eluina|[3726]|05-SRNP-56837|669[0n]bp
Cogia eluina|[3727]|04-SRNP-16191|669[0n]bp
Cogia eluina|[3728]|04-SRNP-16194|669[0n]bp
Cogia eluina|[3729]|04-SRNP-14135|669[0n]bp
Cogia eluina|[3730]|04-SRNP-14051|669[0n]bp
Cogia eluina|[3731]|04-SRNP-13940|669[0n]bp
Cogia eluina|[3732]|05-SRNP-12004|666[0n]bp
Cogia eluina|[3733]|07-SRNP-22100|669[0n]bp
Telemiades avitus|[3734]|05-SRNP-55667|633[0n]bp
Telemiades avitus|[3735]|07-SRNP-41266|669[0n]bp
Telemiades avitus|[3736]|08-SRNP-65922|669[0n]bp
Heliopetes lavianaDHJ01|[3737]|07-SRNP-55300|486[5n]bp
Heliopetes lavianaDHJ01|[3738]|09-SRNP-57620|669[0n]bp
Polygonus savignyDHJ01|[3739]|01-SRNP-14590|660[0n]bp
Polygonus savignyDHJ01|[3740]|06-SRNP-2530|669[0n]bp
Polygonus savignyDHJ01|[3741]|02-SRNP-16605|669[0n]bp
Polygonus savignyDHJ01|[3742]|03-SRNP-15205|669[0n]bp
Polygonus savignyDHJ01|[3743]|91-SRNP-954|669[0n]bp
Polygonus savignyDHJ01|[3744]|03-SRNP-16118|669[0n]bp
Polygonus savignyDHJ01|[3745]|04-SRNP-46430|669[0n]bp
Polygonus savignyDHJ01|[3746]|04-SRNP-46324|669[0n]bp
Polygonus savignyDHJ01|[3747]|06-SRNP-45458|669[0n]bp
Polygonus savignyDHJ01|[3748]|02-SRNP-1520|669[0n]bp
Polygonus savignyDHJ01|[3749]|01-SRNP-14675|669[0n]bp
Polygonus savignyDHJ01|[3750]|02-SRNP-17731|669[0n]bp
Polygonus savignyDHJ01|[3751]|01-SRNP-14738|669[0n]bp
Polygonus savignyDHJ02|[3752]|06-SRNP-16949|594[0n]bp
Polygonus savignyDHJ02|[3753]|06-SRNP-56369|666[0n]bp
Polygonus savignyDHJ02|[3754]|06-SRNP-56370|669[0n]bp
Polygonus savignyDHJ02|[3755]|01-SRNP-14737|669[0n]bp
Polygonus savignyDHJ02|[3756]|01-SRNP-14672|669[0n]bp
Polygonus savignyDHJ02|[3757]|01-SRNP-14673|669[0n]bp
Polygonus savignyDHJ02|[3758]|01-SRNP-14671|669[0n]bp
Polygonus savignyDHJ02|[3759]|01-SRNP-14670|669[0n]bp
Polygonus savignyDHJ02|[3760]|01-SRNP-14669|669[0n]bp
Polygonus savignyDHJ02|[3761]|01-SRNP-14668|669[0n]bp
Polygonus savignyDHJ02|[3762]|01-SRNP-14674|669[0n]bp
Polygonus savignyDHJ02|[3763]|01-SRNP-14676|669[0n]bp
Polygonus savignyDHJ02|[3764]|91-SRNP-431|606[0n]bp
Polygonus savignyDHJ02|[3765]|08-SRNP-1689|669[0n]bp
Polygonus savignyDHJ02|[3766]|08-SRNP-1690|669[0n]bp
Polygonus leo|[3767]|05-SRNP-1988|669[0n]bp
Polygonus leo|[3768]|05-SRNP-45857|627[0n]bp
Polygonus leo|[3769]|04-SRNP-46889|669[0n]bp
Polygonus leo|[3770]|05-SRNP-1445|669[0n]bp
Polygonus leo|[3771]|05-SRNP-1699|669[0n]bp
Polygonus leo|[3772]|05-SRNP-63797|633[0n]bp
Polygonus leo|[3773]|05-SRNP-1444|669[0n]bp
Polygonus leo|[3774]|05-SRNP-1448|669[0n]bp
Polygonus leo|[3775]|05-SRNP-1987|669[0n]bp
Polygonus leo|[3776]|08-SRNP-55973|669[0n]bp
Polygonus leo|[3777]|06-SRNP-15696|669[0n]bp
Polygonus leo|[3778]|06-SRNP-15549|669[0n]bp
Polygonus leo|[3779]|06-SRNP-15550|669[1n]bp
Polygonus leo|[3780]|06-SRNP-15695|600[1n]bp
Polygonus leo|[3781]|08-SRNP-55955|669[0n]bp
Polygonus leo|[3782]|04-SRNP-46785|669[0n]bp
Polygonus leo|[3783]|09-SRNP-14024|669[0n]bp
Astraptes INGCUP|[3784]|97-SRNP-6203|645[0n]bp
Astraptes INGCUP|[3785]|00-SRNP-20677|645[0n]bp
Astraptes INGCUP|[3786]|07-SRNP-4771|603[1n]bp
Astraptes INGCUP|[3787]|08-SRNP-1416|645[0n]bp
Astraptes INGCUP|[3788]|08-SRNP-1414|645[0n]bp
Astraptes INGCUP|[3789]|08-SRNP-82|645[0n]bp
Astraptes INGCUP|[3790]|08-SRNP-130|645[0n]bp
Astraptes INGCUP|[3791]|95-SRNP-8692|651[0n]bp
Astraptes INGCUP|[3792]|01-SRNP-5312|651[0n]bp
Astraptes INGCUP|[3793]|02-SRNP-29904|651[0n]bp
Astraptes INGCUP|[3794]|01-SRNP-508|651[0n]bp
Astraptes INGCUP|[3795]|99-SRNP-4982|651[0n]bp
Astraptes INGCUP|[3796]|96-SRNP-9864|651[0n]bp
Astraptes INGCUP|[3797]|98-SRNP-6438|651[0n]bp
Astraptes INGCUP|[3798]|97-SRNP-6595|651[0n]bp
Astraptes INGCUP|[3799]|99-SRNP-4173|651[0n]bp
[illegible]

Astraptes INGCUP|[3797]|98-SRNP-6438|651[0n]bp
Astraptes INGCUP|[3798]|97-SRNP-6595|651[0n]bp
Astraptes INGCUP|[3799]|99-SRNP-4173|651[0n]bp
Astraptes INGCUP|[3800]|02-SRNP-24431|651[0n]bp
Astraptes INGCUP|[3801]|97-SRNP-6205|651[0n]bp
Astraptes INGCUP|[3802]|02-SRNP-9735|651[0n]bp
Astraptes INGCUP|[3803]|02-SRNP-20351|651[0n]bp
Astraptes INGCUP|[3804]|02-SRNP-19906|651[0n]bp
Astraptes INGCUP|[3805]|02-SRNP-20353|651[0n]bp
Astraptes INGCUP|[3806]|02-SRNP-9734|651[0n]bp
Astraptes INGCUP|[3807]|99-SRNP-5123|651[0n]bp
Astraptes INGCUP|[3808]|99-SRNP-4985|651[0n]bp
Astraptes INGCUP|[3809]|97-SRNP-6231|651[0n]bp
Astraptes INGCUP|[3810]|99-SRNP-5917|651[0n]bp
Astraptes INGCUP|[3811]|95-SRNP-4448|651[0n]bp
Astraptes INGCUP|[3812]|97-SRNP-6024|651[0n]bp
Astraptes INGCUP|[3813]|01-SRNP-1966|651[0n]bp
Astraptes INGCUP|[3814]|01-SRNP-1389|651[0n]bp
Astraptes INGCUP|[3815]|01-SRNP-710|651[0n]bp
Astraptes INGCUP|[3816]|02-SRNP-19350|651[0n]bp
Astraptes INGCUP|[3817]|02-SRNP-21515|651[0n]bp
Astraptes INGCUP|[3818]|97-SRNP-7123|651[0n]bp
Astraptes INGCUP|[3819]|01-SRNP-947|651[0n]bp
Astraptes INGCUP|[3820]|97-SRNP-7160|651[0n]bp
Astraptes INGCUP|[3821]|93-SRNP-2396|651[0n]bp
Astraptes INGCUP|[3822]|00-SRNP-15880|651[0n]bp
Astraptes INGCUP|[3823]|93-SRNP-2622|651[0n]bp
Astraptes INGCUP|[3824]|93-SRNP-2796|651[0n]bp
Astraptes INGCUP|[3825]|99-SRNP-10568|651[0n]bp
Astraptes INGCUP|[3826]|02-SRNP-32205|651[0n]bp
Astraptes INGCUP|[3827]|92-SRNP-3026|651[0n]bp
Astraptes INGCUP|[3828]|91-SRNP-2219|651[0n]bp
Astraptes INGCUP|[3829]|00-SRNP-2547|651[0n]bp
Astraptes INGCUP|[3830]|02-SRNP-32206|651[0n]bp
Astraptes INGCUP|[3831]|02-SRNP-19576|651[0n]bp
Astraptes INGCUP|[3832]|02-SRNP-20092|651[0n]bp
Astraptes INGCUP|[3833]|96-SRNP-934|651[0n]bp
Astraptes INGCUP|[3834]|02-SRNP-19721|651[0n]bp
Astraptes INGCUP|[3835]|98-SRNP-6516|651[0n]bp
Astraptes INGCUP|[3836]|02-SRNP-29906|651[0n]bp
Astraptes INGCUP|[3837]|98-SRNP-6370|651[0n]bp
Astraptes INGCUP|[3838]|94-SRNP-4843|651[0n]bp
Astraptes INGCUP|[3839]|02-SRNP-32204|651[0n]bp
Astraptes INGCUP|[3840]|92-SRNP-3121|651[0n]bp
Astraptes INGCUP|[3841]|99-SRNP-10560|651[0n]bp
Astraptes INGCUP|[3842]|94-SRNP-3011|651[0n]bp
Astraptes INGCUP|[3843]|00-SRNP-15081|651[0n]bp
Astraptes INGCUP|[3844]|94-SRNP-4958|651[0n]bp
Astraptes INGCUP|[3845]|96-SRNP-6841.1|651[0n]bp
Astraptes INGCUP|[3846]|08-SRNP-5592|669[0n]bp
Astraptes INGCUP|[3847]|08-SRNP-1415|669[0n]bp
Astraptes INGCUP|[3848]|07-SRNP-42970|669[0n]bp
Astraptes INGCUP|[3849]|08-SRNP-21067|669[0n]bp
Astraptes INGCUP|[3850]|08-SRNP-65396|669[0n]bp
Astraptes INGCUP|[3851]|08-SRNP-70409|669[0n]bp
Astraptes INGCUP|[3852]|08-SRNP-2460|669[0n]bp
Astraptes INGCUP|[3853]|08-SRNP-1900|669[0n]bp
Astraptes INGCUP|[3854]|07-SRNP-61013|669[0n]bp
Astraptes INGCUP|[3855]|08-SRNP-1792|669[0n]bp
Astraptes INGCUP|[3856]|08-SRNP-40658|669[0n]bp
Astraptes INGCUP|[3857]|08-SRNP-81|669[0n]bp
Astraptes INGCUP|[3858]|08-SRNP-280|669[0n]bp
Astraptes INGCUP|[3859]|08-SRNP-1417|669[0n]bp
Astraptes INGCUP|[3860]|07-SRNP-5172|669[0n]bp
Astraptes INGCUP|[3861]|08-SRNP-1901|669[0n]bp
Astraptes INGCUP|[3862]|07-SRNP-42840|669[0n]bp
Astraptes INGCUP|[3863]|07-SRNP-5170|669[0n]bp
Astraptes INGCUP|[3864]|08-SRNP-59|669[0n]bp
Astraptes INGCUP|[3865]|07-SRNP-45415|669[0n]bp
Astraptes INGCUP|[3866]|07-SRNP-58527|669[0n]bp
Astraptes INGCUP|[3867]|07-SRNP-3511|669[0n]bp
Astraptes INGCUP|[3868]|07-SRNP-45414|669[0n]bp
Astraptes INGCUP|[3869]|07-SRNP-58050|669[0n]bp
Astraptes INGCUP|[3870]|07-SRNP-3512|669[0n]bp
Astraptes INGCUP|[3871]|07-SRNP-21910|669[0n]bp
Astraptes INGCUP|[3872]|07-SRNP-1753|669[0n]bp
Astraptes INGCUP|[3873]|07-SRNP-1790|669[0n]bp
Astraptes INGCUP|[3874]|07-SRNP-40141|669[0n]bp
Astraptes INGCUP|[3875]|07-SRNP-579|669[0n]bp
Astraptes INGCUP|[3876]|07-SRNP-1286|669[0n]bp
Astraptes INGCUP|[3877]|07-SRNP-1285|669[0n]bp
Astraptes INGCUP|[3878]|07-SRNP-380|669[0n]bp
Astraptes INGCUP|[3879]|07-SRNP-381|669[0n]bp
Astraptes INGCUP|[3880]|06-SRNP-9898|669[0n]bp
Astraptes INGCUP|[3881]|06-SRNP-9899|669[0n]bp
Astraptes INGCUP|[3882]|06-SRNP-46318|669[0n]bp
Astraptes INGCUP|[3883]|06-SRNP-34320|669[0n]bp
Astraptes INGCUP|[3884]|06-SRNP-8910|669[0n]bp
Astraptes INGCUP|[3885]|06-SRNP-34321|669[0n]bp
Astraptes INGCUP|[3886]|06-SRNP-46151|669[0n]bp
Astraptes INGCUP|[3887]|06-SRNP-35256|669[0n]bp
Astraptes INGCUP|[3888]|06-SRNP-8752|669[0n]bp
Astraptes INGCUP|[3889]|04-SRNP-46483|669[0n]bp
Astraptes INGCUP|[3890]|04-SRNP-40688|669[0n]bp
Astraptes INGCUP|[3891]|04-SRNP-46477|669[0n]bp
Astraptes INGCUP|[3892]|04-SRNP-60805|669[0n]bp
Astraptes INGCUP|[3893]|03-SRNP-5634|669[0n]bp
Astraptes INGCUP|[3894]|05-SRNP-48590|669[0n]bp
Astraptes INGCUP|[3895]|05-SRNP-58120|669[0n]bp
Astraptes INGCUP|[3896]|05-SRNP-40683|669[0n]bp
Astraptes INGCUP|[3897]|05-SRNP-2221|669[0n]bp
Astraptes INGCUP|[3898]|06-SRNP-55334|669[0n]bp
Astraptes INGCUP|[3899]|07-SRNP-4895|621[0n]bp
[illegible]

Astraptes INGCUP|[3897]|05-SRNP-2221|669[0n]bp
Astraptes INGCUP|[3898]|06-SRNP-55334|669[0n]bp
Astraptes INGCUP|[3899]|07-SRNP-4895|621[0n]bp
Astraptes INGCUP|[3900]|07-SRNP-4323|657[1n]bp
Astraptes INGCUP|[3901]|07-SRNP-4552|654[0n]bp
Astraptes INGCUP|[3902]|07-SRNP-4225|654[0n]bp
Astraptes INGCUP|[3903]|07-SRNP-42991|654[0n]bp
Astraptes INGCUP|[3904]|07-SRNP-4229|648[0n]bp
Astraptes INGCUP|[3905]|07-SRNP-46516|654[1n]bp
Astraptes INGCUP|[3906]|07-SRNP-5013|627[0n]bp
Astraptes INGCUP|[3907]|07-SRNP-4884|633[0n]bp
Astraptes INGCUP|[3908]|06-SRNP-31876|633[0n]bp
Astraptes INGCUP|[3909]|99-SRNP-4980|642[0n]bp
Astraptes INGCUP|[3910]|97-SRNP-6144|639[0n]bp
Astraptes INGCUP|[3911]|02-SRNP-20352|651[0n]bp
Astraptes INGCUP|[3912]|02-SRNP-19284|651[0n]bp
Astraptes INGCUP|[3913]|07-SRNP-4462|630[0n]bp
Astraptes INGCUP|[3914]|07-SRNP-4324|657[1n]bp
Astraptes INGCUP|[3915]|07-SRNP-4806|636[0n]bp
Astraptes INGCUP|[3916]|07-SRNP-2374|666[0n]bp
Astraptes INGCUP|[3917]|07-SRNP-3068|666[0n]bp
Astraptes INGCUP|[3918]|04-SRNP-40472|663[0n]bp
Astraptes INGCUP|[3919]|06-SRNP-46319|666[0n]bp
Astraptes INGCUP|[3920]|03-SRNP-5870|666[0n]bp
Astraptes INGCUP|[3921]|04-SRNP-47856|666[0n]bp
Astraptes INGCUP|[3922]|99-SRNP-13677|651[0n]bp
Astraptes INGCUP|[3923]|07-SRNP-31660|669[0n]bp
Astraptes INGCUP|[3924]|06-SRNP-46276|666[0n]bp
Astraptes INGCUP|[3925]|04-SRNP-47345|666[0n]bp
Astraptes INGCUP|[3926]|04-SRNP-2251|666[0n]bp
Astraptes INGCUP|[3927]|05-SRNP-1361|666[0n]bp
Astraptes INGCUP|[3928]|07-SRNP-58736|669[0n]bp
Astraptes INGCUP|[3929]|04-SRNP-47196|666[0n]bp
Astraptes INGCUP|[3930]|06-SRNP-55405|669[2n]bp
Astraptes INGCUP|[3931]|07-SRNP-1054|669[0n]bp
Astraptes INGCUP|[3932]|07-SRNP-1595|666[0n]bp
Astraptes INGCUP|[3933]|04-SRNP-61428|666[0n]bp
Astraptes INGCUP|[3934]|05-SRNP-19576|666[0n]bp
Astraptes INGCUP|[3935]|05-SRNP-58121|666[0n]bp
Astraptes INGCUP|[3936]|07-SRNP-4606|660[2n]bp
Astraptes INGCUP|[3937]|97-SRNP-5640|651[0n]bp
Astraptes INGCUP|[3938]|07-SRNP-4488|633[0n]bp
Astraptes INGCUP|[3939]|07-SRNP-4489|621[0n]bp
Astraptes INGCUP|[3940]|07-SRNP-4608|654[1n]bp
Astraptes INGCUP|[3941]|04-SRNP-47346|666[0n]bp
Astraptes INGCUP|[3942]|08-SRNP-1418|654[1n]bp
Astraptes INGCUP|[3943]|04-SRNP-40582|624[0n]bp
Astraptes INGCUP|[3944]|07-SRNP-4277|633[0n]bp
Astraptes INGCUP|[3945]|04-SRNP-35086|630[1n]bp
Astraptes INGCUP|[3946]|07-SRNP-4833|642[0n]bp
Astraptes INGCUP|[3947]|03-SRNP-15979|669[1n]bp
Astraptes HIHAMP|[3948]|04-SRNP-35067|648[0n]bp
Astraptes HIHAMP|[3949]|04-SRNP-35081|615[2n]bp
Astraptes HIHAMP|[3950]|06-SRNP-36085|618[0n]bp
Astraptes HIHAMP|[3951]|07-SRNP-35469|669[1n]bp
Astraptes HIHAMP|[3952]|06-SRNP-36608|615[0n]bp
Astraptes HIHAMP|[3953]|99-SRNP-1215|651[0n]bp
Astraptes HIHAMP|[3954]|04-SRNP-56052|669[0n]bp
Astraptes HIHAMP|[3955]|03-SRNP-22543|669[0n]bp
Astraptes HIHAMP|[3956]|07-SRNP-45714|669[0n]bp
Astraptes HIHAMP|[3957]|05-SRNP-35903|666[0n]bp
Astraptes HIHAMP|[3958]|07-SRNP-45437|669[0n]bp
Astraptes HIHAMP|[3959]|07-SRNP-36293|669[0n]bp
Astraptes HIHAMP|[3960]|07-SRNP-35467|669[0n]bp
Astraptes HIHAMP|[3961]|07-SRNP-35468|669[0n]bp
Astraptes HIHAMP|[3962]|06-SRNP-36705|669[0n]bp
Astraptes HIHAMP|[3963]|06-SRNP-36706|669[0n]bp
Astraptes HIHAMP|[3964]|06-SRNP-35831|669[0n]bp
Astraptes HIHAMP|[3965]|06-SRNP-36787|669[0n]bp
Astraptes HIHAMP|[3966]|06-SRNP-36083|669[0n]bp
Astraptes HIHAMP|[3967]|06-SRNP-35716|669[0n]bp
Astraptes HIHAMP|[3968]|03-SRNP-3079|669[0n]bp
Astraptes HIHAMP|[3969]|05-SRNP-35763|669[0n]bp
Astraptes HIHAMP|[3970]|05-SRNP-35765|669[0n]bp
Astraptes HIHAMP|[3971]|06-SRNP-35832|663[0n]bp
Astraptes HIHAMP|[3972]|97-SRNP-1804|651[0n]bp
Astraptes HIHAMP|[3973]|01-SRNP-7374|651[0n]bp
Astraptes HIHAMP|[3974]|02-SRNP-23035|651[0n]bp
Astraptes HIHAMP|[3975]|99-SRNP-1220|651[0n]bp
Astraptes HIHAMP|[3976]|00-SRNP-22183|651[0n]bp
Astraptes HIHAMP|[3977]|01-SRNP-6199|651[0n]bp
Astraptes HIHAMP|[3978]|99-SRNP-1098|651[0n]bp
Astraptes HIHAMP|[3979]|95-SRNP-666|651[0n]bp
Astraptes HIHAMP|[3980]|00-SRNP-10424|651[0n]bp
Astraptes HIHAMP|[3981]|97-SRNP-1588|651[0n]bp
Astraptes HIHAMP|[3982]|97-SRNP-1613|651[0n]bp
Astraptes INGCUP|[3983]|05-SRNP-34301|642[0n]bp
Astraptes HIHAMP|[3984]|01-SRNP-7327|642[0n]bp
Astraptes HIHAMP|[3985]|97-SRNP-1641|645[0n]bp
Astraptes HIHAMP|[3986]|07-SRNP-45715|633[0n]bp
Astraptes HIHAMP|[3987]|07-SRNP-36295|654[2n]bp
Astraptes HIHAMP|[3988]|08-SRNP-35798|669[0n]bp
Astraptes HIHAMP|[3989]|08-SRNP-6196|669[0n]bp
Astraptes fulgerator group|[3990]|09-SRNP-72096|669[0n]bp
Astraptes INGCUP|[3991]|06-SRNP-23021|648[0n]bp
Astraptes INGCUP|[3992]|07-SRNP-4664|648[1n]bp
Astraptes INGCUP|[3993]|07-SRNP-55016|669[1n]bp
Astraptes MYST|[3994]|90-SRNP-1632|585[0n]bp
Astraptes MYST|[3995]|02-SRNP-33369|651[0n]bp
Astraptes MYST|[3996]|02-SRNP-33451|651[0n]bp
Astraptes MYST|[3997]|03-SRNP-21723|669[0n]bp
Astraptes MYST|[3998]|03-SRNP-20884|669[0n]bp
Astraptes MYST|[3999]|03-SRNP-31167|669[0n]bp
[illegible]

Astraptes MYST|[3997]|03-SRNP-21723|669[0n]bp
Astraptes MYST|[3998]|03-SRNP-20884|669[0n]bp
Astraptes MYST|[3999]|03-SRNP-31167|669[0n]bp
Astraptes MYST|[4000]|06-SRNP-41913|669[0n]bp
Astraptes YESENN|[4001]|07-SRNP-45998|612[0n]bp
Astraptes INGCUP|[4002]|03-SRNP-10415|669[0n]bp
Astraptes INGCUP|[4003]|06-SRNP-3381|669[0n]bp
Astraptes INGCUP|[4004]|07-SRNP-4663|618[0n]bp
Astraptes INGCUP|[4005]|02-SRNP-33256|651[0n]bp
Astraptes INGCUP|[4006]|07-SRNP-1756|561[4n]bp
Astraptes INGCUP|[4007]|07-SRNP-4469|615[5n]bp
Astraptes INGCUP|[4008]|07-SRNP-33557|618[3n]bp
Astraptes FABOV|[4009]|94-SRNP-678|651[0n]bp
Astraptes FABOV|[4010]|06-SRNP-47519|618[0n]bp
Astraptes FABOV|[4011]|98-SRNP-79|651[0n]bp
Astraptes FABOV|[4012]|03-SRNP-38625|624[0n]bp
Astraptes FABOV|[4013]|05-SRNP-34585|669[1n]bp
Astraptes FABOV|[4014]|03-SRNP-38030|666[0n]bp
Astraptes FABOV|[4015]|04-SRNP-47477|666[0n]bp
Astraptes FABOV|[4016]|03-SRNP-18628|666[0n]bp
Astraptes FABOV|[4017]|03-SRNP-21672|666[0n]bp
Astraptes FABOV|[4018]|03-SRNP-1100|666[0n]bp
Astraptes FABOV|[4019]|04-SRNP-47263|666[0n]bp
Astraptes FABOV|[4020]|05-SRNP-20134|666[0n]bp
Astraptes FABOV|[4021]|04-SRNP-27294|666[0n]bp
Astraptes FABOV|[4022]|04-SRNP-47478|666[0n]bp
Astraptes FABOV|[4023]|04-SRNP-15696|666[0n]bp
Astraptes FABOV|[4024]|07-SRNP-40452|666[0n]bp
Astraptes FABOV|[4025]|05-SRNP-30622|666[0n]bp
Astraptes FABOV|[4026]|08-SRNP-45170|669[0n]bp
Astraptes FABOV|[4027]|08-SRNP-6000|669[0n]bp
Astraptes FABOV|[4028]|07-SRNP-21410|669[0n]bp
Astraptes FABOV|[4029]|07-SRNP-58607|669[0n]bp
Astraptes FABOV|[4030]|07-SRNP-40453|669[0n]bp
Astraptes FABOV|[4031]|07-SRNP-22021|669[0n]bp
Astraptes FABOV|[4032]|04-SRNP-22024|669[0n]bp
Astraptes FABOV|[4033]|05-SRNP-25512|669[0n]bp
Astraptes FABOV|[4034]|03-SRNP-15973|669[0n]bp
Astraptes FABOV|[4035]|03-SRNP-19356|669[0n]bp
Astraptes FABOV|[4036]|04-SRNP-50141|669[0n]bp
Astraptes FABOV|[4037]|04-SRNP-56195|669[0n]bp
Astraptes FABOV|[4038]|04-SRNP-26414|669[0n]bp
Astraptes FABOV|[4039]|94-SRNP-5469|651[0n]bp
Astraptes FABOV|[4040]|80-SRNP-216|651[0n]bp
Astraptes FABOV|[4041]|97-SRNP-5023|651[0n]bp
Astraptes FABOV|[4042]|97-SRNP-5143|651[0n]bp
Astraptes FABOV|[4043]|02-SRNP-13079|651[0n]bp
Astraptes FABOV|[4044]|95-SRNP-6867|651[0n]bp
Astraptes FABOV|[4045]|92-SRNP-6023|651[0n]bp
Astraptes FABOV|[4046]|93-SRNP-6312|651[0n]bp
Astraptes FABOV|[4047]|95-SRNP-6871|651[0n]bp
Astraptes FABOV|[4048]|97-SRNP-5060|651[0n]bp
Astraptes FABOV|[4049]|93-SRNP-7060|651[0n]bp
Astraptes FABOV|[4050]|02-SRNP-31579|651[0n]bp
Astraptes FABOV|[4051]|95-SRNP-9320|651[0n]bp
Astraptes FABOV|[4052]|02-SRNP-31569|651[0n]bp
Astraptes FABOV|[4053]|02-SRNP-13082|651[0n]bp
Astraptes FABOV|[4054]|96-SRNP-171|651[0n]bp
Astraptes FABOV|[4055]|95-SRNP-6103|651[0n]bp
Astraptes FABOV|[4056]|95-SRNP-511|651[0n]bp
Astraptes FABOV|[4057]|99-SRNP-8551|651[0n]bp
Astraptes FABOV|[4058]|02-SRNP-28909|651[0n]bp
Astraptes FABOV|[4059]|97-SRNP-5809|642[0n]bp
Astraptes FABOV|[4060]|97-SRNP-5926|645[0n]bp
Astraptes FABOV|[4061]|92-SRNP-4645|648[0n]bp
Astraptes FABOV|[4062]|95-SRNP-6866|648[0n]bp
Astraptes FABOV|[4063]|94-SRNP-745|630[0n]bp
Astraptes FABOV|[4064]|04-SRNP-15697|633[0n]bp
Astraptes FABOV|[4065]|05-SRNP-45420|669[0n]bp
Astraptes FABOV|[4066]|80-SRNP-160|627[0n]bp
Astraptes FABOV|[4067]|97-SRNP-5927|651[0n]bp
Astraptes FABOV|[4068]|04-SRNP-27295|669[0n]bp
Astraptes FABOV|[4069]|07-SRNP-56300|669[0n]bp
Astraptes FABOV|[4070]|05-SRNP-20856|669[0n]bp
Astraptes FABOV|[4071]|95-SRNP-8306|651[0n]bp
Astraptes FABOV|[4072]|97-SRNP-5969|642[0n]bp
Astraptes FABOV|[4073]|05-SRNP-21651|669[0n]bp
Astraptes FABOV|[4074]|05-SRNP-60050|669[0n]bp
Astraptes FABOV|[4075]|08-SRNP-58528|669[0n]bp
Astraptes enta|[4076]|03-SRNP-11423|666[0n]bp
Astraptes enta|[4077]|07-SRNP-42567|669[0n]bp
Astraptes enta|[4078]|09-SRNP-75531|669[0n]bp
Astraptes BYTTNER|[4079]|95-SRNP-8045|651[0n]bp
Astraptes BYTTNER|[4080]|95-SRNP-8046|651[0n]bp
Astraptes BYTTNER|[4081]|95-SRNP-8044|645[0n]bp
Astraptes LOHAMP|[4082]|99-SRNP-17057|375[0n]bp
Astraptes LOHAMP|[4083]|06-SRNP-36233|588[1n]bp
Astraptes LOHAMP|[4084]|06-SRNP-36395|612[2n]bp
Astraptes LOHAMP|[4085]|06-SRNP-31669|633[2n]bp
Astraptes LOHAMP|[4086]|06-SRNP-36238|600[0n]bp
Astraptes LOHAMP|[4087]|03-SRNP-4251|666[1n]bp
Astraptes LOHAMP|[4088]|06-SRNP-36296|615[0n]bp
Astraptes LOHAMP|[4089]|07-SRNP-2042|618[0n]bp
Astraptes LOHAMP|[4090]|04-SRNP-35879|624[0n]bp
Astraptes LOHAMP|[4091]|06-SRNP-36344|612[0n]bp
Astraptes LOHAMP|[4092]|06-SRNP-36346|612[0n]bp
Astraptes LOHAMP|[4093]|06-SRNP-36338|621[0n]bp
Astraptes LOHAMP|[4094]|06-SRNP-7043|657[0n]bp
Astraptes LOHAMP|[4095]|06-SRNP-7363|657[0n]bp
Astraptes LOHAMP|[4096]|06-SRNP-8179|633[0n]bp
Astraptes LOHAMP|[4097]|04-SRNP-35147|633[0n]bp
Astraptes LOHAMP|[4098]|03-SRNP-3139|633[0n]bp
Astraptes LOHAMP|[4099]|04-SRNP-61342|633[0n]bp
[illegible]

Astraptes LOHAMP|[4097]|04-SRNP-35147|633[0n]bp
Astraptes LOHAMP|[4098]|03-SRNP-3139|633[0n]bp
Astraptes LOHAMP|[4099]|04-SRNP-61342|633[0n]bp
Astraptes LOHAMP|[4100]|04-SRNP-56771|633[0n]bp
Astraptes LOHAMP|[4101]|03-SRNP-15889|633[0n]bp
Astraptes LOHAMP|[4102]|06-SRNP-36052|651[0n]bp
Astraptes LOHAMP|[4103]|06-SRNP-6884|651[0n]bp
Astraptes LOHAMP|[4104]|08-SRNP-35977|669[0n]bp
Astraptes LOHAMP|[4105]|08-SRNP-35969|669[0n]bp
Astraptes LOHAMP|[4106]|08-SRNP-35978|669[0n]bp
Astraptes LOHAMP|[4107]|08-SRNP-72414|669[0n]bp
Astraptes LOHAMP|[4108]|08-SRNP-35033|669[0n]bp
Astraptes LOHAMP|[4109]|08-SRNP-21411|669[0n]bp
Astraptes LOHAMP|[4110]|08-SRNP-35306|669[0n]bp
Astraptes LOHAMP|[4111]|07-SRNP-36287|669[0n]bp
Astraptes LOHAMP|[4112]|07-SRNP-3020|669[0n]bp
Astraptes LOHAMP|[4113]|07-SRNP-42071|669[0n]bp
Astraptes LOHAMP|[4114]|07-SRNP-36173|669[0n]bp
Astraptes LOHAMP|[4115]|07-SRNP-35819|669[0n]bp
Astraptes LOHAMP|[4116]|07-SRNP-35848|669[0n]bp
Astraptes LOHAMP|[4117]|07-SRNP-42123|669[0n]bp
Astraptes LOHAMP|[4118]|07-SRNP-35826|669[0n]bp
Astraptes LOHAMP|[4119]|07-SRNP-35754|669[0n]bp
Astraptes LOHAMP|[4120]|07-SRNP-35755|669[0n]bp
Astraptes LOHAMP|[4121]|07-SRNP-35709|669[0n]bp
Astraptes LOHAMP|[4122]|07-SRNP-697|669[0n]bp
Astraptes LOHAMP|[4123]|07-SRNP-1983|669[0n]bp
Astraptes LOHAMP|[4124]|07-SRNP-35414|669[0n]bp
Astraptes LOHAMP|[4125]|06-SRNP-65614|669[0n]bp
Astraptes LOHAMP|[4126]|07-SRNP-35525|669[0n]bp
Astraptes LOHAMP|[4127]|07-SRNP-1211|669[0n]bp
Astraptes LOHAMP|[4128]|07-SRNP-1467|669[0n]bp
Astraptes LOHAMP|[4129]|06-SRNP-36758|669[0n]bp
Astraptes LOHAMP|[4130]|06-SRNP-36094|669[0n]bp
Astraptes LOHAMP|[4131]|06-SRNP-36941|669[0n]bp
Astraptes LOHAMP|[4132]|06-SRNP-36618|669[0n]bp
Astraptes LOHAMP|[4133]|06-SRNP-36087|669[0n]bp
Astraptes LOHAMP|[4134]|06-SRNP-36707|669[0n]bp
Astraptes LOHAMP|[4135]|06-SRNP-47853|669[0n]bp
Astraptes LOHAMP|[4136]|06-SRNP-36541|669[0n]bp
Astraptes LOHAMP|[4137]|06-SRNP-36616|669[0n]bp
Astraptes LOHAMP|[4138]|06-SRNP-36544|669[0n]bp
Astraptes LOHAMP|[4139]|06-SRNP-36237|669[0n]bp
Astraptes LOHAMP|[4140]|07-SRNP-35045|669[0n]bp
Astraptes LOHAMP|[4141]|07-SRNP-35040|669[0n]bp
Astraptes LOHAMP|[4142]|06-SRNP-35627|669[0n]bp
Astraptes LOHAMP|[4143]|06-SRNP-35607|669[0n]bp
Astraptes LOHAMP|[4144]|06-SRNP-35688|669[0n]bp
Astraptes LOHAMP|[4145]|06-SRNP-35628|669[0n]bp
Astraptes LOHAMP|[4146]|06-SRNP-35619|669[0n]bp
Astraptes LOHAMP|[4147]|06-SRNP-35710|669[0n]bp
Astraptes LOHAMP|[4148]|06-SRNP-9530|669[0n]bp
Astraptes LOHAMP|[4149]|06-SRNP-6234|669[0n]bp
Astraptes LOHAMP|[4150]|06-SRNP-21646|669[0n]bp
Astraptes LOHAMP|[4151]|06-SRNP-9420|669[0n]bp
Astraptes LOHAMP|[4152]|06-SRNP-23242|669[0n]bp
Astraptes LOHAMP|[4153]|06-SRNP-6037|669[0n]bp
Astraptes LOHAMP|[4154]|06-SRNP-6038|669[0n]bp
Astraptes LOHAMP|[4155]|06-SRNP-47681|669[0n]bp
Astraptes LOHAMP|[4156]|06-SRNP-7042|669[0n]bp
Astraptes LOHAMP|[4157]|06-SRNP-5587|669[0n]bp
Astraptes LOHAMP|[4158]|06-SRNP-6933|669[0n]bp
Astraptes LOHAMP|[4159]|06-SRNP-8178|669[0n]bp
Astraptes LOHAMP|[4160]|06-SRNP-9171|669[0n]bp
Astraptes LOHAMP|[4161]|06-SRNP-8076|669[0n]bp
Astraptes LOHAMP|[4162]|06-SRNP-35562|669[0n]bp
Astraptes LOHAMP|[4163]|06-SRNP-4848|669[0n]bp
Astraptes LOHAMP|[4164]|04-SRNP-35126|669[0n]bp
Astraptes LOHAMP|[4165]|04-SRNP-35127|669[0n]bp
Astraptes LOHAMP|[4166]|04-SRNP-35162|669[0n]bp
Astraptes LOHAMP|[4167]|04-SRNP-35149|669[0n]bp
Astraptes LOHAMP|[4168]|04-SRNP-46671|669[0n]bp
Astraptes LOHAMP|[4169]|04-SRNP-50042|669[0n]bp
Astraptes LOHAMP|[4170]|04-SRNP-49891|669[0n]bp
Astraptes LOHAMP|[4171]|04-SRNP-60810|669[0n]bp
Astraptes LOHAMP|[4172]|04-SRNP-55659|669[0n]bp
Astraptes LOHAMP|[4173]|04-SRNP-56335|669[0n]bp
Astraptes LOHAMP|[4174]|04-SRNP-61002|669[0n]bp
Astraptes LOHAMP|[4175]|04-SRNP-60550|669[0n]bp
Astraptes LOHAMP|[4176]|04-SRNP-61012|669[0n]bp
Astraptes LOHAMP|[4177]|04-SRNP-60933|669[0n]bp
Astraptes LOHAMP|[4178]|04-SRNP-36186|669[0n]bp
Astraptes LOHAMP|[4179]|04-SRNP-36170|669[0n]bp
Astraptes LOHAMP|[4180]|03-SRNP-22299|669[0n]bp
Astraptes LOHAMP|[4181]|03-SRNP-3138|669[0n]bp
Astraptes LOHAMP|[4182]|03-SRNP-3509|669[0n]bp
Astraptes LOHAMP|[4183]|03-SRNP-3100|669[0n]bp
Astraptes LOHAMP|[4184]|03-SRNP-4933|669[0n]bp
Astraptes LOHAMP|[4185]|03-SRNP-4776|669[0n]bp
Astraptes LOHAMP|[4186]|03-SRNP-4281|669[0n]bp
Astraptes LOHAMP|[4187]|03-SRNP-4252|669[0n]bp
Astraptes LOHAMP|[4188]|03-SRNP-4410|669[0n]bp
Astraptes LOHAMP|[4189]|03-SRNP-6959|669[0n]bp
Astraptes LOHAMP|[4190]|03-SRNP-7550|669[0n]bp
Astraptes LOHAMP|[4191]|04-SRNP-40265|669[0n]bp
Astraptes LOHAMP|[4192]|03-SRNP-4699|669[0n]bp
Astraptes LOHAMP|[4193]|03-SRNP-3193|669[0n]bp
Astraptes LOHAMP|[4194]|03-SRNP-3258|669[0n]bp
Astraptes LOHAMP|[4195]|03-SRNP-5544|669[0n]bp
Astraptes LOHAMP|[4196]|03-SRNP-7135|669[0n]bp
Astraptes LOHAMP|[4197]|03-SRNP-3112|669[0n]bp
Astraptes LOHAMP|[4198]|03-SRNP-3343|669[0n]bp
Astraptes LOHAMP|[4199]|03-SRNP-10879|669[0n]bp
[illegible]

Astraptes LOHAMP|[4197]|03-SRNP-3112|669[0n]bp
Astraptes LOHAMP|[4198]|03-SRNP-3343|669[0n]bp
Astraptes LOHAMP|[4199]|03-SRNP-10879|669[0n]bp
Astraptes LOHAMP|[4200]|03-SRNP-6958|669[0n]bp
Astraptes LOHAMP|[4201]|06-SRNP-35048|669[0n]bp
Astraptes LOHAMP|[4202]|06-SRNP-35127|669[0n]bp
Astraptes LOHAMP|[4203]|04-SRNP-36231|669[0n]bp
Astraptes LOHAMP|[4204]|05-SRNP-41118|669[0n]bp
Astraptes LOHAMP|[4205]|04-SRNP-35980|669[0n]bp
Astraptes LOHAMP|[4206]|05-SRNP-350|669[0n]bp
Astraptes LOHAMP|[4207]|05-SRNP-35593|669[0n]bp
Astraptes LOHAMP|[4208]|04-SRNP-56334|669[0n]bp
Astraptes LOHAMP|[4209]|05-SRNP-231|669[0n]bp
Astraptes LOHAMP|[4210]|07-SRNP-35931|666[0n]bp
Astraptes LOHAMP|[4211]|07-SRNP-40830|666[0n]bp
Astraptes LOHAMP|[4212]|06-SRNP-36607|666[0n]bp
Astraptes LOHAMP|[4213]|07-SRNP-35002|666[0n]bp
Astraptes LOHAMP|[4214]|06-SRNP-42492|666[0n]bp
Astraptes LOHAMP|[4215]|06-SRNP-42491|666[0n]bp
Astraptes LOHAMP|[4216]|04-SRNP-35218|666[0n]bp
Astraptes LOHAMP|[4217]|04-SRNP-35559|666[0n]bp
Astraptes LOHAMP|[4218]|03-SRNP-23253|666[0n]bp
Astraptes LOHAMP|[4219]|03-SRNP-3354|666[0n]bp
Astraptes LOHAMP|[4220]|03-SRNP-4998|666[0n]bp
Astraptes LOHAMP|[4221]|03-SRNP-4369|666[0n]bp
Astraptes LOHAMP|[4222]|03-SRNP-1331|666[0n]bp
Astraptes LOHAMP|[4223]|04-SRNP-35877|666[0n]bp
Astraptes LOHAMP|[4224]|04-SRNP-35875|666[0n]bp
Astraptes LOHAMP|[4225]|04-SRNP-35694|666[0n]bp
Astraptes LOHAMP|[4226]|04-SRNP-3509|666[0n]bp
Astraptes LOHAMP|[4227]|04-SRNP-35873|666[0n]bp
Astraptes LOHAMP|[4228]|04-SRNP-3852|666[0n]bp
Astraptes LOHAMP|[4229]|04-SRNP-35321|666[0n]bp
Astraptes LOHAMP|[4230]|04-SRNP-35170|666[0n]bp
Astraptes LOHAMP|[4231]|04-SRNP-46672|666[0n]bp
Astraptes LOHAMP|[4232]|04-SRNP-35683|666[0n]bp
Astraptes LOHAMP|[4233]|04-SRNP-35449|666[0n]bp
Astraptes LOHAMP|[4234]|04-SRNP-35500|666[0n]bp
Astraptes LOHAMP|[4235]|04-SRNP-35346|666[0n]bp
Astraptes LOHAMP|[4236]|04-SRNP-35269|666[0n]bp
Astraptes LOHAMP|[4237]|04-SRNP-35395|666[0n]bp
Astraptes LOHAMP|[4238]|04-SRNP-35343|666[0n]bp
Astraptes LOHAMP|[4239]|04-SRNP-35436|666[0n]bp
Astraptes LOHAMP|[4240]|03-SRNP-23619|666[0n]bp
Astraptes LOHAMP|[4241]|03-SRNP-4294|666[0n]bp
Astraptes LOHAMP|[4242]|03-SRNP-6225|666[0n]bp
Astraptes LOHAMP|[4243]|03-SRNP-13031.1|666[0n]bp
Astraptes LOHAMP|[4244]|03-SRNP-31151|666[0n]bp
Astraptes LOHAMP|[4245]|03-SRNP-23573|666[0n]bp
Astraptes LOHAMP|[4246]|03-SRNP-22087|666[0n]bp
Astraptes LOHAMP|[4247]|06-SRNP-35022|666[0n]bp
Astraptes LOHAMP|[4248]|04-SRNP-36163|666[0n]bp
Astraptes LOHAMP|[4249]|05-SRNP-1602|666[0n]bp
Astraptes LOHAMP|[4250]|04-SRNP-36230|666[0n]bp
Astraptes LOHAMP|[4251]|05-SRNP-35006|666[0n]bp
Astraptes LOHAMP|[4252]|04-SRNP-36185|666[0n]bp
Astraptes LOHAMP|[4253]|07-SRNP-35003|666[0n]bp
Astraptes LOHAMP|[4254]|06-SRNP-7285|663[0n]bp
Astraptes LOHAMP|[4255]|07-SRNP-35593|663[0n]bp
Astraptes LOHAMP|[4256]|06-SRNP-36339|627[1n]bp
Astraptes LOHAMP|[4257]|07-SRNP-1982|654[0n]bp
Astraptes LOHAMP|[4258]|04-SRNP-4381|654[0n]bp
Astraptes LOHAMP|[4259]|00-SRNP-9957|651[0n]bp
Astraptes LOHAMP|[4260]|01-SRNP-7375|651[0n]bp
Astraptes LOHAMP|[4261]|02-SRNP-23196|651[0n]bp
Astraptes LOHAMP|[4262]|02-SRNP-8010|651[0n]bp
Astraptes LOHAMP|[4263]|01-SRNP-21272|651[0n]bp
Astraptes LOHAMP|[4264]|02-SRNP-19946|651[0n]bp
Astraptes LOHAMP|[4265]|02-SRNP-9906|651[0n]bp
Astraptes LOHAMP|[4266]|02-SRNP-19945|651[0n]bp
Astraptes LOHAMP|[4267]|02-SRNP-9775|651[0n]bp
Astraptes LOHAMP|[4268]|02-SRNP-7893|651[0n]bp
Astraptes LOHAMP|[4269]|02-SRNP-9769|651[0n]bp
Astraptes LOHAMP|[4270]|02-SRNP-19727|651[0n]bp
Astraptes LOHAMP|[4271]|02-SRNP-7856|651[0n]bp
Astraptes LOHAMP|[4272]|02-SRNP-23086|651[0n]bp
Astraptes LOHAMP|[4273]|02-SRNP-23926|651[0n]bp
Astraptes LOHAMP|[4274]|02-SRNP-19182|651[0n]bp
Astraptes LOHAMP|[4275]|01-SRNP-6254|651[0n]bp
Astraptes LOHAMP|[4276]|98-SRNP-2129|651[0n]bp
Astraptes LOHAMP|[4277]|97-SRNP-804|651[0n]bp
Astraptes LOHAMP|[4278]|98-SRNP-2071|651[0n]bp
Astraptes LOHAMP|[4279]|01-SRNP-21069|651[0n]bp
Astraptes LOHAMP|[4280]|98-SRNP-2132|651[0n]bp
Astraptes LOHAMP|[4281]|98-SRNP-6256|651[0n]bp
Astraptes LOHAMP|[4282]|98-SRNP-6331|651[0n]bp
Astraptes LOHAMP|[4283]|98-SRNP-2607|651[0n]bp
Astraptes LOHAMP|[4284]|94-SRNP-10101|651[0n]bp
Astraptes LOHAMP|[4285]|99-SRNP-17142|651[0n]bp
Astraptes LOHAMP|[4286]|99-SRNP-17117|651[0n]bp
Astraptes LOHAMP|[4287]|97-SRNP-795|651[0n]bp
Astraptes LOHAMP|[4288]|99-SRNP-17030|651[0n]bp
Astraptes LOHAMP|[4289]|99-SRNP-17039|651[0n]bp
Astraptes LOHAMP|[4290]|00-SRNP-11876|651[0n]bp
Astraptes LOHAMP|[4291]|99-SRNP-1893|651[0n]bp
Astraptes LOHAMP|[4292]|00-SRNP-11725|651[0n]bp
Astraptes LOHAMP|[4293]|99-SRNP-17192|651[0n]bp
Astraptes LOHAMP|[4294]|96-SRNP-7037|651[0n]bp
Astraptes LOHAMP|[4295]|95-SRNP-244|651[0n]bp
Astraptes LOHAMP|[4296]|02-SRNP-9525|651[0n]bp
Astraptes LOHAMP|[4297]|05-SRNP-2170|648[0n]bp
Astraptes LOHAMP|[4298]|07-SRNP-45889|648[0n]bp
Astraptes LOHAMP|[4299]|99-SRNP-1403|642[0n]bp
[illegible]

Astraptes LOHAMP|[4297]|05-SRNP-2170|648[0n]bp
Astraptes LOHAMP|[4298]|07-SRNP-45889|648[0n]bp
Astraptes LOHAMP|[4299]|99-SRNP-1403|642[0n]bp
Astraptes LOHAMP|[4300]|97-SRNP-1898|639[0n]bp
Astraptes LOHAMP|[4301]|97-SRNP-6178|639[0n]bp
Astraptes LOHAMP|[4302]|97-SRNP-6757|648[0n]bp
Astraptes LOHAMP|[4303]|95-SRNP-365|648[0n]bp
Astraptes LOHAMP|[4304]|95-SRNP-867|648[0n]bp
Astraptes LOHAMP|[4305]|06-SRNP-36701|624[0n]bp
Astraptes LOHAMP|[4306]|06-SRNP-36231|624[0n]bp
Astraptes LOHAMP|[4307]|04-SRNP-36169|666[0n]bp
Astraptes LOHAMP|[4308]|05-SRNP-40961|669[0n]bp
Astraptes LOHAMP|[4309]|06-SRNP-7520|669[0n]bp
Astraptes LOHAMP|[4310]|06-SRNP-35608|669[0n]bp
Astraptes LOHAMP|[4311]|06-SRNP-36679|669[0n]bp
Astraptes LOHAMP|[4312]|07-SRNP-30949|669[0n]bp
Astraptes LOHAMP|[4313]|07-SRNP-2787|669[0n]bp
Astraptes LOHAMP|[4314]|07-SRNP-2858|669[0n]bp
Astraptes LOHAMP|[4315]|08-SRNP-71001|669[0n]bp
Astraptes LOHAMP|[4316]|08-SRNP-35886|669[0n]bp
Astraptes LOHAMP|[4317]|08-SRNP-5784|669[0n]bp
Astraptes LOHAMP|[4318]|09-SRNP-69238|669[0n]bp
Astraptes LOHAMP|[4319]|09-SRNP-72074|669[0n]bp
Astraptes LOHAMP|[4320]|09-SRNP-35515|669[0n]bp
Astraptes LOHAMP|[4321]|09-SRNP-41801|669[0n]bp
Astraptes YESENN|[4322]|98-SRNP-2713|600[4n]bp
Astraptes YESENN|[4323]|03-SRNP-9129|666[0n]bp
Astraptes YESENN|[4324]|05-SRNP-1745|666[0n]bp
Astraptes YESENN|[4325]|03-SRNP-22479|666[0n]bp
Astraptes YESENN|[4326]|06-SRNP-31612|669[0n]bp
Astraptes YESENN|[4327]|07-SRNP-1716|669[0n]bp
Astraptes YESENN|[4328]|07-SRNP-1714|669[0n]bp
Astraptes YESENN|[4329]|08-SRNP-1634|669[0n]bp
Astraptes YESENN|[4330]|08-SRNP-1633|669[0n]bp
Astraptes YESENN|[4331]|95-SRNP-512|651[1n]bp
Astraptes YESENN|[4332]|02-SRNP-9495|651[1n]bp
Astraptes YESENN|[4333]|95-SRNP-840|651[1n]bp
Astraptes YESENN|[4334]|07-SRNP-23723|618[0n]bp
Astraptes YESENN|[4335]|05-SRNP-2389|669[0n]bp
Astraptes YESENN|[4336]|98-SRNP-14616|651[0n]bp
Astraptes YESENN|[4337]|02-SRNP-21442|651[0n]bp
Astraptes YESENN|[4338]|03-SRNP-5145|666[0n]bp
Astraptes YESENN|[4339]|06-SRNP-34242|669[0n]bp
Astraptes YESENN|[4340]|04-SRNP-48269|669[0n]bp
Astraptes YESENN|[4341]|03-SRNP-3194|669[1n]bp
Astraptes YESENN|[4342]|02-SRNP-9939|618[0n]bp
Astraptes YESENN|[4343]|98-SRNP-7982|609[0n]bp
Astraptes YESENN|[4344]|06-SRNP-45979|657[0n]bp
Astraptes YESENN|[4345]|95-SRNP-4551|420[0n]bp
Astraptes YESENN|[4346]|99-SRNP-302|651[0n]bp
Astraptes YESENN|[4347]|04-SRNP-46518|642[1n]bp
Astraptes YESENN|[4348]|04-SRNP-46751|642[1n]bp
Astraptes YESENN|[4349]|95-SRNP-858|591[0n]bp
Astraptes YESENN|[4350]|03-SRNP-15877|669[0n]bp
Astraptes YESENN|[4351]|08-SRNP-36525|669[0n]bp
Astraptes YESENN|[4352]|08-SRNP-36993|669[0n]bp
Astraptes YESENN|[4353]|08-SRNP-2398|669[0n]bp
Astraptes YESENN|[4354]|08-SRNP-2397|669[0n]bp
Astraptes YESENN|[4355]|07-SRNP-23843|669[0n]bp
Astraptes YESENN|[4356]|08-SRNP-65542|669[0n]bp
Astraptes YESENN|[4357]|07-SRNP-45395|669[0n]bp
Astraptes YESENN|[4358]|07-SRNP-65880|669[0n]bp
Astraptes YESENN|[4359]|07-SRNP-45394|669[0n]bp
Astraptes YESENN|[4360]|07-SRNP-45298|669[0n]bp
Astraptes YESENN|[4361]|07-SRNP-21700|669[0n]bp
Astraptes YESENN|[4362]|07-SRNP-36004|669[0n]bp
Astraptes YESENN|[4363]|06-SRNP-65728|669[0n]bp
Astraptes YESENN|[4364]|07-SRNP-1339|669[0n]bp
Astraptes INGCUP|[4365]|06-SRNP-7673|669[0n]bp
Astraptes YESENN|[4366]|06-SRNP-7197|669[0n]bp
Astraptes YESENN|[4367]|06-SRNP-45902|669[0n]bp
Astraptes YESENN|[4368]|06-SRNP-7523|669[0n]bp
Astraptes YESENN|[4369]|03-SRNP-12813.1|669[0n]bp
Astraptes YESENN|[4370]|03-SRNP-20867|669[0n]bp
Astraptes YESENN|[4371]|03-SRNP-20900|669[0n]bp
Astraptes YESENN|[4372]|03-SRNP-31190|669[0n]bp
Astraptes YESENN|[4373]|03-SRNP-9131|669[0n]bp
Astraptes YESENN|[4374]|03-SRNP-13054.1|669[0n]bp
Astraptes YESENN|[4375]|03-SRNP-19233|669[0n]bp
Astraptes YESENN|[4376]|03-SRNP-21719|669[0n]bp
Astraptes YESENN|[4377]|03-SRNP-20039|669[0n]bp
Astraptes YESENN|[4378]|03-SRNP-12396.1|669[0n]bp
Astraptes YESENN|[4379]|03-SRNP-5830|669[0n]bp
Astraptes YESENN|[4380]|03-SRNP-18629|669[0n]bp
Astraptes YESENN|[4381]|03-SRNP-19981|669[0n]bp
Astraptes YESENN|[4382]|03-SRNP-12811.1|669[0n]bp
Astraptes YESENN|[4383]|06-SRNP-2596|669[0n]bp
Astraptes YESENN|[4384]|03-SRNP-19923|669[0n]bp
Astraptes YESENN|[4385]|06-SRNP-35002|669[0n]bp
Astraptes YESENN|[4386]|05-SRNP-343|669[0n]bp
Astraptes YESENN|[4387]|05-SRNP-46248|669[0n]bp
Astraptes YESENN|[4388]|05-SRNP-46247|669[0n]bp
Astraptes YESENN|[4389]|05-SRNP-23710|669[1n]bp
Astraptes YESENN|[4390]|07-SRNP-1582|666[0n]bp
Astraptes YESENN|[4391]|04-SRNP-46657|666[0n]bp
Astraptes YESENN|[4392]|04-SRNP-46761|666[0n]bp
Astraptes YESENN|[4393]|03-SRNP-29977|666[0n]bp
Astraptes YESENN|[4394]|03-SRNP-19925|666[0n]bp
Astraptes YESENN|[4395]|04-SRNP-61087|666[0n]bp
Astraptes YESENN|[4396]|04-SRNP-42351|666[0n]bp
Astraptes YESENN|[4397]|04-SRNP-21144|666[0n]bp
Astraptes YESENN|[4398]|03-SRNP-17617|666[0n]bp
Astraptes YESENN|[4399]|03-SRNP-19500|666[0n]bp
[illegible]

Astraptes YESENN|[4397]|04-SRNP-21144|666[0n]bp
Astraptes YESENN|[4398]|03-SRNP-17617|666[0n]bp
Astraptes YESENN|[4399]|03-SRNP-19500|666[0n]bp
Astraptes YESENN|[4400]|03-SRNP-11292|666[0n]bp
Astraptes YESENN|[4401]|03-SRNP-11291|666[0n]bp
Astraptes YESENN|[4402]|03-SRNP-13084.1|666[0n]bp
Astraptes YESENN|[4403]|03-SRNP-31311|666[0n]bp
Astraptes YESENN|[4404]|04-SRNP-56956|669[0n]bp
Astraptes YESENN|[4405]|04-SRNP-56955|669[0n]bp
Astraptes YESENN|[4406]|03-SRNP-31192|666[0n]bp
Astraptes YESENN|[4407]|06-SRNP-46048|669[0n]bp
Astraptes YESENN|[4408]|03-SRNP-8219|666[0n]bp
Astraptes YESENN|[4409]|03-SRNP-21599|666[0n]bp
Astraptes YESENN|[4410]|05-SRNP-2166|669[0n]bp
Astraptes YESENN|[4411]|06-SRNP-35000|669[0n]bp
Astraptes YESENN|[4412]|06-SRNP-2689|666[0n]bp
Astraptes YESENN|[4413]|07-SRNP-3219|669[0n]bp
Astraptes YESENN|[4414]|06-SRNP-35003|666[0n]bp
Astraptes YESENN|[4415]|01-SRNP-9652|651[0n]bp
Astraptes YESENN|[4416]|07-SRNP-31395|669[0n]bp
Astraptes YESENN|[4417]|06-SRNP-7000|660[0n]bp
Astraptes YESENN|[4418]|00-SRNP-11880|651[0n]bp
Astraptes YESENN|[4419]|99-SRNP-3015|651[0n]bp
Astraptes YESENN|[4420]|02-SRNP-30212|651[0n]bp
Astraptes YESENN|[4421]|02-SRNP-30211|651[0n]bp
Astraptes YESENN|[4422]|00-SRNP-11188|651[0n]bp
Astraptes YESENN|[4423]|94-SRNP-9330|651[0n]bp
Astraptes YESENN|[4424]|02-SRNP-19308|651[0n]bp
Astraptes YESENN|[4425]|98-SRNP-6561|651[0n]bp
Astraptes YESENN|[4426]|02-SRNP-31858|651[0n]bp
Astraptes YESENN|[4427]|02-SRNP-29805|651[0n]bp
Astraptes YESENN|[4428]|02-SRNP-30062|651[0n]bp
Astraptes YESENN|[4429]|02-SRNP-33820|651[0n]bp
Astraptes YESENN|[4430]|02-SRNP-30059|651[0n]bp
Astraptes YESENN|[4431]|02-SRNP-20190|651[0n]bp
Astraptes YESENN|[4432]|02-SRNP-21384|651[0n]bp
Astraptes YESENN|[4433]|99-SRNP-4820|651[0n]bp
Astraptes YESENN|[4434]|99-SRNP-203|651[0n]bp
Astraptes YESENN|[4435]|99-SRNP-11|651[0n]bp
Astraptes YESENN|[4436]|99-SRNP-386|651[0n]bp
Astraptes YESENN|[4437]|99-SRNP-12|651[0n]bp
Astraptes YESENN|[4438]|99-SRNP-2446|651[0n]bp
Astraptes YESENN|[4439]|01-SRNP-6896|651[0n]bp
Astraptes YESENN|[4440]|01-SRNP-1047|651[0n]bp
Astraptes YESENN|[4441]|00-SRNP-22164|651[0n]bp
Astraptes YESENN|[4442]|02-SRNP-9496|651[0n]bp
Astraptes YESENN|[4443]|98-SRNP-14632|651[4n]bp
Astraptes YESENN|[4444]|03-SRNP-20899|645[0n]bp
Astraptes YESENN|[4445]|02-SRNP-23365|642[0n]bp
Astraptes YESENN|[4446]|98-SRNP-7874|639[0n]bp
Astraptes YESENN|[4447]|99-SRNP-301|636[0n]bp
Astraptes YESENN|[4448]|98-SRNP-4773|636[0n]bp
Astraptes YESENN|[4449]|06-SRNP-41253|633[0n]bp
Astraptes YESENN|[4450]|06-SRNP-35006|633[0n]bp
Astraptes YESENN|[4451]|06-SRNP-45767|630[1n]bp
Astraptes YESENN|[4452]|07-SRNP-42668|552[0n]bp
Astraptes YESENN|[4453]|09-SRNP-57375|669[0n]bp
Astraptes YESENN|[4454]|03-SRNP-19491|666[1n]bp
Astraptes YESENN|[4455]|03-SRNP-1327|669[0n]bp
Astraptes YESENN|[4456]|06-SRNP-45771|657[0n]bp
Astraptes YESENN|[4457]|06-SRNP-3660|657[0n]bp
Astraptes YESENN|[4458]|03-SRNP-12405.1|669[0n]bp
Astraptes YESENN|[4459]|07-SRNP-23600|573[1n]bp
Astraptes YESENN|[4460]|08-SRNP-1537|645[0n]bp
Astraptes YESENN|[4461]|06-SRNP-5657|627[1n]bp
Astraptes YESENN|[4462]|03-SRNP-7325|669[0n]bp
Astraptes YESENN|[4463]|03-SRNP-19236|669[0n]bp
Astraptes YESENN|[4464]|03-SRNP-16333|669[0n]bp
Astraptes YESENN|[4465]|03-SRNP-16844|669[0n]bp
Astraptes YESENN|[4466]|03-SRNP-30303|669[0n]bp
Astraptes YESENN|[4467]|06-SRNP-43726|669[0n]bp
Astraptes YESENN|[4468]|03-SRNP-12638.1|669[0n]bp
Astraptes YESENN|[4469]|03-SRNP-37033|669[0n]bp
Astraptes YESENN|[4470]|03-SRNP-19241|669[0n]bp
Astraptes YESENN|[4471]|03-SRNP-20422|669[0n]bp
Astraptes YESENN|[4472]|03-SRNP-15876|669[0n]bp
Astraptes YESENN|[4473]|03-SRNP-4202|669[0n]bp
Astraptes YESENN|[4474]|06-SRNP-35033|669[0n]bp
Astraptes YESENN|[4475]|04-SRNP-27148|669[0n]bp
Astraptes YESENN|[4476]|05-SRNP-41425|669[0n]bp
Astraptes YESENN|[4477]|05-SRNP-23696|666[0n]bp
Astraptes YESENN|[4478]|05-SRNP-23780|666[0n]bp
Astraptes YESENN|[4479]|06-SRNP-2688|666[0n]bp
Astraptes YESENN|[4480]|95-SRNP-4464|651[0n]bp
Astraptes YESENN|[4481]|02-SRNP-33384|651[0n]bp
Astraptes YESENN|[4482]|02-SRNP-31970|651[0n]bp
Astraptes YESENN|[4483]|00-SRNP-20862|651[0n]bp
Astraptes YESENN|[4484]|01-SRNP-9728|651[0n]bp
Astraptes YESENN|[4485]|97-SRNP-6181|651[0n]bp
Astraptes YESENN|[4486]|99-SRNP-4773|651[0n]bp
Astraptes YESENN|[4487]|98-SRNP-15072|651[0n]bp
Astraptes YESENN|[4488]|00-SRNP-10415|651[0n]bp
Astraptes YESENN|[4489]|02-SRNP-17335|642[0n]bp
Astraptes YESENN|[4490]|03-SRNP-19235|633[0n]bp
Astraptes YESENN|[4491]|04-SRNP-30116|660[0n]bp
Astraptes YESENN|[4492]|03-SRNP-21722|666[0n]bp
Astraptes YESENN|[4493]|03-SRNP-21302|666[0n]bp
Astraptes YESENN|[4494]|04-SRNP-47991|666[0n]bp
Astraptes YESENN|[4495]|04-SRNP-56475|666[0n]bp
Astraptes YESENN|[4496]|03-SRNP-11346|666[0n]bp
Astraptes YESENN|[4497]|03-SRNP-2947|666[0n]bp
Astraptes YESENN|[4498]|03-SRNP-31288|666[0n]bp
Astraptes YESENN|[4499]|04-SRNP-45272|666[0n]bp
[illegible]

Astraptes YESENN|[4497]|03-SRNP-2947|666[0n]bp
Astraptes YESENN|[4498]|03-SRNP-31288|666[0n]bp
Astraptes YESENN|[4499]|04-SRNP-45272|666[0n]bp
Astraptes YESENN|[4500]|06-SRNP-22582|666[0n]bp
Astraptes YESENN|[4501]|06-SRNP-7387|669[0n]bp
Astraptes YESENN|[4502]|06-SRNP-23309|669[0n]bp
Astraptes YESENN|[4503]|06-SRNP-23308|669[0n]bp
Astraptes YESENN|[4504]|06-SRNP-46392|669[0n]bp
Astraptes YESENN|[4505]|09-SRNP-21124|669[0n]bp
Astraptes YESENN|[4506]|09-SRNP-80729|669[0n]bp
Astraptes YESENN|[4507]|03-SRNP-1328|669[2n]bp
Astraptes YESENN|[4508]|99-SRNP-17158|651[1n]bp
Astraptes YESENN|[4509]|04-SRNP-35012|666[0n]bp
Astraptes YESENN|[4510]|02-SRNP-29803|651[0n]bp
Astraptes YESENN|[4511]|02-SRNP-18849|651[0n]bp
Astraptes YESENN|[4512]|02-SRNP-19921|651[0n]bp
Astraptes YESENN|[4513]|06-SRNP-33146|669[0n]bp
Astraptes YESENN|[4514]|07-SRNP-1583|645[0n]bp
Astraptes YESENN|[4515]|06-SRNP-46808|669[0n]bp
Astraptes YESENN|[4516]|06-SRNP-46810|669[0n]bp
Astraptes YESENN|[4517]|06-SRNP-21578|669[0n]bp
Astraptes YESENN|[4518]|06-SRNP-21607|669[0n]bp
Astraptes YESENN|[4519]|06-SRNP-22150|669[0n]bp
Astraptes YESENN|[4520]|06-SRNP-46491|669[0n]bp
Astraptes YESENN|[4521]|06-SRNP-43633|669[0n]bp
Astraptes YESENN|[4522]|06-SRNP-45450|669[0n]bp
Astraptes YESENN|[4523]|06-SRNP-2751|669[0n]bp
Astraptes YESENN|[4524]|04-SRNP-46943|669[0n]bp
Astraptes YESENN|[4525]|03-SRNP-12768.1|669[0n]bp
Astraptes YESENN|[4526]|03-SRNP-13044.1|669[0n]bp
Astraptes YESENN|[4527]|03-SRNP-31188|669[0n]bp
Astraptes YESENN|[4528]|03-SRNP-12300.1|666[0n]bp
Astraptes YESENN|[4529]|03-SRNP-28876|666[0n]bp
Astraptes YESENN|[4530]|03-SRNP-15893|666[0n]bp
Astraptes YESENN|[4531]|03-SRNP-5207|666[0n]bp
Astraptes YESENN|[4532]|03-SRNP-28260|666[1n]bp
Astraptes YESENN|[4533]|03-SRNP-19922|666[0n]bp
Astraptes YESENN|[4534]|03-SRNP-31295|666[0n]bp
Astraptes YESENN|[4535]|03-SRNP-13045.1|666[0n]bp
Astraptes YESENN|[4536]|03-SRNP-37165|666[0n]bp
Astraptes YESENN|[4537]|03-SRNP-18626|669[0n]bp
Astraptes YESENN|[4538]|98-SRNP-6537|651[0n]bp
Astraptes YESENN|[4539]|04-SRNP-46760|666[0n]bp
Astraptes YESENN|[4540]|05-SRNP-23548|666[0n]bp
Astraptes YESENN|[4541]|03-SRNP-28257|669[0n]bp
Astraptes YESENN|[4542]|03-SRNP-28256|666[0n]bp
Astraptes YESENN|[4543]|05-SRNP-65076|666[0n]bp
Astraptes YESENN|[4544]|06-SRNP-43453|657[0n]bp
Astraptes YESENN|[4545]|89-SRNP-112|651[0n]bp
Astraptes YESENN|[4546]|99-SRNP-155|651[0n]bp
Astraptes YESENN|[4547]|99-SRNP-4383|651[0n]bp
Astraptes YESENN|[4548]|00-SRNP-11805|651[0n]bp
Astraptes YESENN|[4549]|02-SRNP-19802|651[0n]bp
Astraptes YESENN|[4550]|02-SRNP-19922|651[0n]bp
Astraptes YESENN|[4551]|98-SRNP-7848|651[0n]bp
Astraptes YESENN|[4552]|01-SRNP-6800|651[0n]bp
Astraptes YESENN|[4553]|99-SRNP-4436|651[0n]bp
Astraptes YESENN|[4554]|98-SRNP-15015|651[0n]bp
Astraptes YESENN|[4555]|97-SRNP-6252|651[0n]bp
Astraptes YESENN|[4556]|98-SRNP-2712|651[0n]bp
Astraptes YESENN|[4557]|00-SRNP-22003|651[0n]bp
Astraptes YESENN|[4558]|98-SRNP-6332|651[0n]bp
Astraptes YESENN|[4559]|99-SRNP-525|651[0n]bp
Astraptes YESENN|[4560]|95-SRNP-4402|648[0n]bp
Astraptes YESENN|[4561]|99-SRNP-524|642[0n]bp
Astraptes YESENN|[4562]|99-SRNP-4418|639[0n]bp
Astraptes YESENN|[4563]|99-SRNP-2444|639[0n]bp
Astraptes YESENN|[4564]|06-SRNP-7361|639[0n]bp
Astraptes YESENN|[4565]|06-SRNP-47922|618[0n]bp
Astraptes YESENN|[4566]|07-SRNP-1266|666[0n]bp
Astraptes YESENN|[4567]|07-SRNP-42069|669[0n]bp
Astraptes YESENN|[4568]|07-SRNP-65846|669[0n]bp
Astraptes YESENN|[4569]|08-SRNP-2539|669[0n]bp
Astraptes YESENN|[4570]|09-SRNP-2732|669[0n]bp
Astraptes YESENN|[4571]|06-SRNP-7524|669[0n]bp
Astraptes YESENN|[4572]|06-SRNP-7624|669[0n]bp
Astraptes YESENN|[4573]|06-SRNP-46103|669[0n]bp
Astraptes YESENN|[4574]|04-SRNP-47834|666[0n]bp
Astraptes YESENN|[4575]|03-SRNP-19933|666[0n]bp
Astraptes YESENN|[4576]|06-SRNP-5658|663[0n]bp
Astraptes YESENN|[4577]|93-SRNP-3364|651[0n]bp
Astraptes YESENN|[4578]|03-SRNP-23561|666[0n]bp
Astraptes YESENN|[4579]|99-SRNP-402|651[0n]bp
Astraptes YESENN|[4580]|06-SRNP-5995|642[0n]bp
Astraptes YESENN|[4581]|06-SRNP-22572|669[0n]bp
Astraptes YESENN|[4582]|09-SRNP-20756|669[0n]bp
Astraptes LONCHO|[4583]|04-SRNP-35659|642[0n]bp
Astraptes LONCHO|[4584]|97-SRNP-721|651[0n]bp
Astraptes LONCHO|[4585]|04-SRNP-35510|570[1n]bp
Astraptes LONCHO|[4586]|02-SRNP-8471|558[1n]bp
Astraptes LONCHO|[4587]|02-SRNP-9547|612[0n]bp
Astraptes LONCHO|[4588]|02-SRNP-8068|621[0n]bp
Astraptes LONCHO|[4589]|02-SRNP-8361|558[1n]bp
Astraptes LONCHO|[4590]|02-SRNP-8165|558[1n]bp
Astraptes LONCHO|[4591]|97-SRNP-840|651[0n]bp
Astraptes LONCHO|[4592]|04-SRNP-35270|540[1n]bp
Astraptes LONCHO|[4593]|04-SRNP-35660|666[0n]bp
Astraptes LONCHO|[4594]|03-SRNP-22396|660[0n]bp
Astraptes LONCHO|[4595]|06-SRNP-35047|660[0n]bp
Astraptes LONCHO|[4596]|00-SRNP-10977|651[0n]bp
Astraptes LONCHO|[4597]|02-SRNP-9135|651[0n]bp
Astraptes LONCHO|[4598]|98-SRNP-2703|651[0n]bp
Astraptes LONCHO|[4599]|00-SRNP-10502|651[0n]bp
[illegible]

Astraptes LONCHO|[4597]|02-SRNP-9135|651[0n]bp
Astraptes LONCHO|[4598]|98-SRNP-2703|651[0n]bp
Astraptes LONCHO|[4599]|00-SRNP-10502|651[0n]bp
Astraptes LONCHO|[4600]|99-SRNP-17068|651[0n]bp
Astraptes LONCHO|[4601]|01-SRNP-21505|651[0n]bp
Astraptes LONCHO|[4602]|99-SRNP-17184|651[0n]bp
Astraptes LONCHO|[4603]|02-SRNP-9976|651[0n]bp
Astraptes LONCHO|[4604]|00-SRNP-10501|651[0n]bp
Astraptes LONCHO|[4605]|00-SRNP-10500|651[0n]bp
Astraptes LONCHO|[4606]|89-SRNP-736|651[0n]bp
Astraptes LONCHO|[4607]|97-SRNP-1147|651[0n]bp
Astraptes LONCHO|[4608]|97-SRNP-842|651[0n]bp
Astraptes LONCHO|[4609]|02-SRNP-9448|651[0n]bp
Astraptes LONCHO|[4610]|97-SRNP-1856|651[0n]bp
Astraptes LONCHO|[4611]|02-SRNP-8281|651[0n]bp
Astraptes LONCHO|[4612]|97-SRNP-724|651[0n]bp
Astraptes LONCHO|[4613]|02-SRNP-8140|651[0n]bp
Astraptes LONCHO|[4614]|97-SRNP-1127|651[0n]bp
Astraptes LONCHO|[4615]|97-SRNP-755|651[0n]bp
Astraptes LONCHO|[4616]|02-SRNP-8439|651[0n]bp
Astraptes LONCHO|[4617]|00-SRNP-9539|651[0n]bp
Astraptes LONCHO|[4618]|02-SRNP-8360|651[0n]bp
Astraptes LONCHO|[4619]|97-SRNP-722|651[0n]bp
Astraptes LONCHO|[4620]|00-SRNP-9040|651[0n]bp
Astraptes LONCHO|[4621]|02-SRNP-8249|651[0n]bp
Astraptes LONCHO|[4622]|02-SRNP-8343|651[0n]bp
Astraptes LONCHO|[4623]|01-SRNP-21520|651[0n]bp
Astraptes LONCHO|[4624]|97-SRNP-1050|651[0n]bp
Astraptes LONCHO|[4625]|02-SRNP-8438|651[0n]bp
Astraptes LONCHO|[4626]|97-SRNP-723|651[0n]bp
Astraptes LONCHO|[4627]|97-SRNP-720|651[0n]bp
Astraptes LONCHO|[4628]|02-SRNP-24219|633[0n]bp
Astraptes LONCHO|[4629]|99-SRNP-17162|639[0n]bp
Astraptes LONCHO|[4630]|07-SRNP-35892|669[0n]bp
Astraptes LONCHO|[4631]|07-SRNP-36055|669[0n]bp
Astraptes LONCHO|[4632]|07-SRNP-45518|669[0n]bp
Astraptes LONCHO|[4633]|07-SRNP-36122|669[0n]bp
Astraptes LONCHO|[4634]|08-SRNP-36242|669[0n]bp
Astraptes LONCHO|[4635]|08-SRNP-35999|669[0n]bp
Astraptes LONCHO|[4636]|04-SRNP-56583|669[0n]bp
Astraptes LONCHO|[4637]|06-SRNP-35016|669[0n]bp
Astraptes LONCHO|[4638]|09-SRNP-36882|669[0n]bp
Astraptes SENNOV|[4639]|01-SRNP-2604|378[1n]bp
Astraptes SENNOV|[4640]|91-SRNP-2454|558[6n]bp
Astraptes SENNOV|[4641]|95-SRNP-4409|651[0n]bp
Astraptes SENNOV|[4642]|05-SRNP-45060|669[0n]bp
Astraptes SENNOV|[4643]|93-SRNP-3363|651[0n]bp
Astraptes SENNOV|[4644]|92-SRNP-3975.1|651[0n]bp
Astraptes SENNOV|[4645]|95-SRNP-6137|651[0n]bp
Astraptes SENNOV|[4646]|93-SRNP-3377|651[0n]bp
Astraptes SENNOV|[4647]|90-SRNP-1711|636[0n]bp
Astraptes SENNOV|[4648]|92-SRNP-3975|651[0n]bp
Astraptes SENNOV|[4649]|96-SRNP-10407|651[0n]bp
Astraptes SENNOV|[4650]|99-SRNP-10683.1|651[0n]bp
Astraptes SENNOV|[4651]|96-SRNP-10408|651[0n]bp
Astraptes SENNOV|[4652]|94-SRNP-8089|651[0n]bp
Astraptes SENNOV|[4653]|96-SRNP-10410|651[0n]bp
Astraptes SENNOV|[4654]|96-SRNP-10409|651[0n]bp
Astraptes SENNOV|[4655]|04-SRNP-30803|666[0n]bp
Astraptes SENNOV|[4656]|91-SRNP-1741|651[0n]bp
Astraptes SENNOV|[4657]|06-SRNP-19450|669[0n]bp
Astraptes SENNOV|[4658]|99-SRNP-4144|651[0n]bp
Astraptes SENNOV|[4659]|06-SRNP-31423|645[1n]bp
Astraptes SENNOV|[4660]|93-SRNP-954|651[0n]bp
Astraptes SENNOV|[4661]|93-SRNP-955|651[0n]bp
Astraptes SENNOV|[4662]|95-SRNP-8555|651[1n]bp
Astraptes SENNOV|[4663]|07-SRNP-45042|669[0n]bp
Astraptes SENNOV|[4664]|07-SRNP-45041|669[0n]bp
Astraptes SENNOV|[4665]|07-SRNP-45040|669[0n]bp
Astraptes SENNOV|[4666]|07-SRNP-45043|669[0n]bp
Astraptes SENNOV|[4667]|04-SRNP-46788|669[0n]bp
Astraptes SENNOV|[4668]|06-SRNP-47119|669[0n]bp
Astraptes SENNOV|[4669]|05-SRNP-60388|669[0n]bp
Astraptes SENNOV|[4670]|04-SRNP-46986|669[0n]bp
Astraptes SENNOV|[4671]|05-SRNP-47264|669[0n]bp
Astraptes SENNOV|[4672]|05-SRNP-59407|669[0n]bp
Astraptes SENNOV|[4673]|05-SRNP-46328|669[0n]bp
Astraptes SENNOV|[4674]|05-SRNP-46845|669[0n]bp
Astraptes SENNOV|[4675]|05-SRNP-46843|669[0n]bp
Astraptes SENNOV|[4676]|05-SRNP-59544|669[0n]bp
Astraptes SENNOV|[4677]|05-SRNP-57580|669[0n]bp
Astraptes SENNOV|[4678]|06-SRNP-20364|669[0n]bp
Astraptes SENNOV|[4679]|04-SRNP-48059|666[0n]bp
Astraptes SENNOV|[4680]|05-SRNP-46846|666[0n]bp
Astraptes SENNOV|[4681]|95-SRNP-4807|651[0n]bp
Astraptes SENNOV|[4682]|99-SRNP-411|651[0n]bp
Astraptes SENNOV|[4683]|98-SRNP-2714|651[0n]bp
Astraptes SENNOV|[4684]|99-SRNP-413|651[0n]bp
Astraptes SENNOV|[4685]|99-SRNP-409|651[0n]bp
Astraptes SENNOV|[4686]|99-SRNP-412|651[0n]bp
Astraptes SENNOV|[4687]|95-SRNP-6435|651[0n]bp
Astraptes SENNOV|[4688]|96-SRNP-10154|651[0n]bp
Astraptes SENNOV|[4689]|93-SRNP-3603.2|651[0n]bp
Astraptes SENNOV|[4690]|89-SRNP-23|651[0n]bp
Astraptes SENNOV|[4691]|93-SRNP-5558.1|651[0n]bp
Astraptes SENNOV|[4692]|93-SRNP-3701|651[0n]bp
Astraptes SENNOV|[4693]|95-SRNP-8643|651[0n]bp
Astraptes SENNOV|[4694]|95-SRNP-9333|651[0n]bp
Astraptes SENNOV|[4695]|96-SRNP-8770|651[0n]bp
Astraptes SENNOV|[4696]|96-SRNP-8797|651[0n]bp
Astraptes SENNOV|[4697]|99-SRNP-207|651[0n]bp
Astraptes SENNOV|[4698]|95-SRNP-6869|651[0n]bp
Astraptes SENNOV|[4699]|99-SRNP-403|651[0n]bp
[illegible]

Astraptes SENNOV|[4697]|99-SRNP-207|651[0n]bp
Astraptes SENNOV|[4698]|95-SRNP-6869|651[0n]bp
Astraptes SENNOV|[4699]|99-SRNP-403|651[0n]bp
Astraptes SENNOV|[4700]|93-SRNP-2206|651[0n]bp
Astraptes SENNOV|[4701]|93-SRNP-2201|651[0n]bp
Astraptes SENNOV|[4702]|02-SRNP-13088|651[0n]bp
Astraptes SENNOV|[4703]|95-SRNP-4533|651[0n]bp
Astraptes SENNOV|[4704]|93-SRNP-1025|651[0n]bp
Astraptes SENNOV|[4705]|93-SRNP-2208|651[0n]bp
Astraptes SENNOV|[4706]|96-SRNP-8773|645[0n]bp
Astraptes SENNOV|[4707]|99-SRNP-399|645[0n]bp
Astraptes SENNOV|[4708]|92-SRNP-69|645[0n]bp
Astraptes SENNOV|[4709]|92-SRNP-70|636[0n]bp
Astraptes SENNOV|[4710]|96-SRNP-812|639[0n]bp
Astraptes SENNOV|[4711]|99-SRNP-410|639[0n]bp
Astraptes SENNOV|[4712]|95-SRNP-6476|609[0n]bp
Astraptes SENNOV|[4713]|91-SRNP-3005|609[0n]bp
Astraptes SENNOV|[4714]|96-SRNP-8769|645[0n]bp
Astraptes SENNOV|[4715]|04-SRNP-47619|633[0n]bp
Astraptes SENNOV|[4716]|04-SRNP-47520|666[0n]bp
Astraptes SENNOV|[4717]|04-SRNP-47264|666[0n]bp
Astraptes SENNOV|[4718]|06-SRNP-46624|666[0n]bp
Astraptes SENNOV|[4719]|07-SRNP-23823|669[0n]bp
Astraptes SENNOV|[4720]|99-SRNP-17188|651[1n]bp
Astraptes SENNOV|[4721]|03-SRNP-22238|666[0n]bp
Astraptes SENNOV|[4722]|03-SRNP-22239|666[0n]bp
Astraptes SENNOV|[4723]|06-SRNP-2651|666[0n]bp
Astraptes SENNOV|[4724]|06-SRNP-36042|669[0n]bp
Astraptes SENNOV|[4725]|03-SRNP-3011|669[2n]bp
Astraptes SENNOV|[4726]|05-SRNP-55272|666[0n]bp
Astraptes SENNOV|[4727]|05-SRNP-45346|669[1n]bp
Astraptes SENNOV|[4728]|95-SRNP-10951|642[0n]bp
Astraptes SENNOV|[4729]|06-SRNP-47118|669[0n]bp
Astraptes SENNOV|[4730]|07-SRNP-58784|669[0n]bp
Astraptes SENNOV|[4731]|92-SRNP-5660|645[1n]bp
Astraptes SENNOV|[4732]|06-SRNP-31380|645[1n]bp
Astraptes SENNOV|[4733]|06-SRNP-46575|669[0n]bp
Astraptes SENNOV|[4734]|06-SRNP-46050|669[0n]bp
Astraptes SENNOV|[4735]|04-SRNP-49527|669[0n]bp
Astraptes SENNOV|[4736]|04-SRNP-45246|669[0n]bp
Astraptes SENNOV|[4737]|03-SRNP-22051|669[0n]bp
Astraptes SENNOV|[4738]|05-SRNP-7221|669[0n]bp
Astraptes SENNOV|[4739]|05-SRNP-46983|669[0n]bp
Astraptes SENNOV|[4740]|04-SRNP-47268|666[0n]bp
Astraptes SENNOV|[4741]|03-SRNP-14687|666[0n]bp
Astraptes SENNOV|[4742]|06-SRNP-46931|669[0n]bp
Astraptes SENNOV|[4743]|06-SRNP-460|666[1n]bp
Astraptes SENNOV|[4744]|91-SRNP-2715|651[0n]bp
Astraptes SENNOV|[4745]|93-SRNP-3656|651[0n]bp
Astraptes SENNOV|[4746]|93-SRNP-3655|651[0n]bp
Astraptes SENNOV|[4747]|96-SRNP-8802|651[0n]bp
Astraptes SENNOV|[4748]|99-SRNP-2445|651[0n]bp
Astraptes SENNOV|[4749]|99-SRNP-401|651[0n]bp
Astraptes SENNOV|[4750]|96-SRNP-6857|651[0n]bp
Astraptes SENNOV|[4751]|95-SRNP-6898|651[0n]bp
Astraptes SENNOV|[4752]|92-SRNP-4398|651[0n]bp
Astraptes SENNOV|[4753]|95-SRNP-6899|648[0n]bp
Astraptes SENNOV|[4754]|04-SRNP-47266|633[0n]bp
Astraptes SENNOV|[4755]|98-SRNP-3453|651[0n]bp
Astraptes SENNOV|[4756]|91-SRNP-3045|438[0n]bp
Astraptes SENNOV|[4757]|93-SRNP-3603.1|651[0n]bp
Astraptes SENNOV|[4758]|06-SRNP-59595|645[0n]bp
Astraptes SENNOV|[4759]|06-SRNP-47181|669[0n]bp
Astraptes SENNOV|[4760]|07-SRNP-30435|669[0n]bp
Astraptes SENNOV|[4761]|08-SRNP-55549|645[0n]bp
Astraptes INGCUPnumt|[4762]|02-SRNP-13284|651[7n]bp
Astraptes SENNOVnumt|[4763]|06-SRNP-23360|645[1n]bp
Astraptes YESENNnumt|[4764]|06-SRNP-46388|666[3n]bp
Astraptes YESENNnumt|[4765]|06-SRNP-33139|639[1n]bp
Astraptes SENNOVnumt|[4766]|04-SRNP-47236|663[0n]bp
Astraptes SENNOVnumt|[4767]|03-SRNP-3010|669[1n]bp
Astraptes YESENNnumt|[4768]|00-SRNP-11386|651[0n]bp
Astraptes SENNOVnumt|[4769]|94-SRNP-6452|651[0n]bp
Astraptes SENNOVnumt|[4770]|93-SRNP-2680|651[0n]bp
Astraptes YESENNnumt|[4771]|05-SRNP-1089|669[0n]bp
Astraptes YESENNnumt|[4772]|06-SRNP-46389|669[0n]bp
Astraptes YESENNnumt|[4773]|07-SRNP-24037|654[0n]bp
Astraptes CELT|[4774]|02-SRNP-31493|651[0n]bp
Astraptes CELT|[4775]|02-SRNP-31136|651[0n]bp
Astraptes CELT|[4776]|06-SRNP-48008|594[0n]bp
Astraptes CELT|[4777]|06-SRNP-47989|609[0n]bp
Astraptes CELT|[4778]|07-SRNP-45471|669[1n]bp
Astraptes CELT|[4779]|07-SRNP-1959|669[1n]bp
Astraptes CELT|[4780]|02-SRNP-29799|651[0n]bp
Astraptes CELT|[4781]|06-SRNP-3010|666[0n]bp
Astraptes CELT|[4782]|05-SRNP-20730|666[0n]bp
Astraptes CELT|[4783]|05-SRNP-1765|669[0n]bp
Astraptes CELT|[4784]|06-SRNP-3231|669[0n]bp
Astraptes CELT|[4785]|05-SRNP-20729|669[0n]bp
Astraptes CELT|[4786]|06-SRNP-46989|669[0n]bp
Astraptes CELT|[4787]|07-SRNP-23567|669[0n]bp
Astraptes CELT|[4788]|07-SRNP-23826|669[0n]bp
Astraptes CELT|[4789]|97-SRNP-9604|645[0n]bp
Astraptes CELT|[4790]|04-SRNP-48372|666[1n]bp
Astraptes CELT|[4791]|06-SRNP-45462|669[3n]bp
Astraptes CELT|[4792]|06-SRNP-4757|657[0n]bp
Astraptes CELT|[4793]|05-SRNP-754|669[1n]bp
Astraptes CELT|[4794]|04-SRNP-46734|663[0n]bp
Astraptes CELT|[4795]|02-SRNP-31741|651[0n]bp
Astraptes CELT|[4796]|02-SRNP-29082|651[0n]bp
Astraptes CELT|[4797]|04-SRNP-46737|633[0n]bp
Astraptes CELT|[4798]|04-SRNP-46063|633[0n]bp
Astraptes CELT|[4799]|07-SRNP-1969|654[0n]bp
[illegible]

Astraptes CELT|[4797]|04-SRNP-46737|633[0n]bp
Astraptes CELT|[4798]|04-SRNP-46063|633[0n]bp
Astraptes CELT|[4799]|07-SRNP-1969|654[0n]bp
Astraptes CELT|[4800]|94-SRNP-9389|651[0n]bp
Astraptes CELT|[4801]|99-SRNP-5312|651[0n]bp
Astraptes CELT|[4802]|02-SRNP-33197|651[0n]bp
Astraptes CELT|[4803]|01-SRNP-1413|651[0n]bp
Astraptes CELT|[4804]|02-SRNP-31745|651[0n]bp
Astraptes CELT|[4805]|02-SRNP-33016|651[0n]bp
Astraptes CELT|[4806]|02-SRNP-29083|651[0n]bp
Astraptes CELT|[4807]|02-SRNP-31738|651[0n]bp
Astraptes CELT|[4808]|02-SRNP-29080|651[0n]bp
Astraptes CELT|[4809]|02-SRNP-29798|651[0n]bp
Astraptes CELT|[4810]|96-SRNP-227|651[0n]bp
Astraptes CELT|[4811]|02-SRNP-30241|651[0n]bp
Astraptes CELT|[4812]|02-SRNP-33002|651[0n]bp
Astraptes CELT|[4813]|02-SRNP-29054|651[0n]bp
Astraptes CELT|[4814]|02-SRNP-31739|651[0n]bp
Astraptes CELT|[4805]|02-SRNP-33016|651[0n]bp
Astraptes CELT|[4802]|02-SRNP-33197|651[0n]bp
Astraptes CELT|[4815]|06-SRNP-3011|666[0n]bp
Astraptes CELT|[4816]|04-SRNP-46459|666[0n]bp
Astraptes CELT|[4817]|05-SRNP-20279|666[0n]bp
Astraptes CELT|[4818]|05-SRNP-20731|666[0n]bp
Astraptes CELT|[4819]|05-SRNP-6757|666[0n]bp
Astraptes CELT|[4820]|05-SRNP-1608|666[0n]bp
Astraptes CELT|[4821]|05-SRNP-45088|666[0n]bp
Astraptes CELT|[4822]|02-SRNP-31377|642[0n]bp
Astraptes CELT|[4823]|05-SRNP-24514|669[0n]bp
Astraptes CELT|[4824]|04-SRNP-46431|669[0n]bp
Astraptes CELT|[4825]|06-SRNP-4630|669[0n]bp
Astraptes CELT|[4826]|06-SRNP-4631|669[0n]bp
Astraptes CELT|[4827]|06-SRNP-9528|669[0n]bp
Astraptes CELT|[4828]|06-SRNP-47745|669[0n]bp
Astraptes CELT|[4829]|07-SRNP-1958|669[0n]bp
Astraptes CELT|[4830]|07-SRNP-1967|669[0n]bp
Astraptes CELT|[4831]|07-SRNP-45467|669[0n]bp
Astraptes CELT|[4832]|07-SRNP-3042|669[0n]bp
Astraptes CELT|[4833]|07-SRNP-45470|669[0n]bp
Astraptes CELT|[4834]|07-SRNP-45603|669[0n]bp
Astraptes CELT|[4835]|07-SRNP-45602|669[0n]bp
Astraptes CELT|[4836]|07-SRNP-45608|669[0n]bp
Astraptes CELT|[4837]|07-SRNP-45607|669[0n]bp
Astraptes CELT|[4838]|07-SRNP-45600|669[0n]bp
Astraptes CELT|[4839]|07-SRNP-22224|669[0n]bp
Astraptes CELT|[4840]|08-SRNP-4065|669[0n]bp
Astraptes CELT|[4841]|09-SRNP-65963|669[0n]bp
Astraptes TRIGO|[4842]|06-SRNP-55033|669[3n]bp
Astraptes TRIGO|[4843]|07-SRNP-45479|669[0n]bp
Astraptes TRIGO|[4844]|07-SRNP-45480|669[0n]bp
Astraptes TRIGO|[4845]|06-SRNP-59720|669[0n]bp
Astraptes TRIGO|[4846]|07-SRNP-45478|669[0n]bp
Astraptes TRIGO|[4847]|07-SRNP-45482|669[0n]bp
Astraptes TRIGO|[4848]|02-SRNP-31761|651[0n]bp
Astraptes TRIGO|[4849]|91-SRNP-2346|627[0n]bp
Astraptes TRIGO|[4850]|06-SRNP-59719|615[0n]bp
Astraptes TRIGO|[4851]|97-SRNP-5364|651[0n]bp
Astraptes TRIGO|[4852]|97-SRNP-5500|642[0n]bp
Astraptes TRIGO|[4853]|07-SRNP-55072|627[0n]bp
Astraptes TRIGO|[4854]|02-SRNP-30290|651[0n]bp
Astraptes TRIGO|[4855]|02-SRNP-30291|651[0n]bp
Astraptes TRIGO|[4856]|92-SRNP-4386|651[0n]bp
Astraptes TRIGO|[4857]|02-SRNP-31535|651[0n]bp
Astraptes TRIGO|[4858]|03-SRNP-17651|666[0n]bp
Astraptes TRIGO|[4859]|05-SRNP-45112|666[0n]bp
Astraptes TRIGO|[4860]|05-SRNP-45125|666[0n]bp
Astraptes TRIGO|[4861]|05-SRNP-45076|666[0n]bp
Astraptes TRIGO|[4862]|05-SRNP-45073|666[0n]bp
Astraptes TRIGO|[4863]|01-SRNP-9825|651[0n]bp
Astraptes TRIGO|[4864]|02-SRNP-29103|651[0n]bp
Astraptes TRIGO|[4865]|02-SRNP-31684|651[0n]bp
Astraptes TRIGO|[4866]|02-SRNP-33258|651[0n]bp
Astraptes TRIGO|[4867]|02-SRNP-31538|651[0n]bp
Astraptes TRIGO|[4868]|02-SRNP-31536|651[0n]bp
Astraptes TRIGO|[4869]|02-SRNP-33259|651[0n]bp
Astraptes TRIGO|[4870]|02-SRNP-31537|651[0n]bp
Astraptes TRIGO|[4871]|02-SRNP-31762|651[0n]bp
Astraptes TRIGO|[4872]|02-SRNP-31756|651[0n]bp
Astraptes TRIGO|[4873]|02-SRNP-33261|651[0n]bp
Astraptes TRIGO|[4874]|98-SRNP-5868|651[0n]bp
Astraptes TRIGO|[4875]|02-SRNP-31748|651[0n]bp
Astraptes TRIGO|[4876]|02-SRNP-33260|651[0n]bp
Astraptes TRIGO|[4877]|02-SRNP-31539|651[0n]bp
Astraptes TRIGO|[4878]|02-SRNP-31755|651[0n]bp
Astraptes TRIGO|[4879]|97-SRNP-5215|651[0n]bp
Astraptes TRIGO|[4880]|97-SRNP-5499|651[0n]bp
Astraptes TRIGO|[4881]|02-SRNP-31620|651[0n]bp
Astraptes TRIGO|[4882]|92-SRNP-5726|651[0n]bp
Astraptes TRIGO|[4883]|02-SRNP-31621|651[0n]bp
Astraptes TRIGO|[4884]|91-SRNP-2576|651[0n]bp
Astraptes TRIGO|[4885]|02-SRNP-31784|651[0n]bp
Astraptes TRIGO|[4886]|02-SRNP-31782|651[0n]bp
Astraptes TRIGO|[4887]|91-SRNP-2694|651[0n]bp
Astraptes TRIGO|[4888]|02-SRNP-31817|651[0n]bp
Astraptes TRIGO|[4889]|02-SRNP-33453|651[0n]bp
Astraptes TRIGO|[4890]|97-SRNP-4485|651[0n]bp
Astraptes TRIGO|[4891]|91-SRNP-2631|651[0n]bp
Astraptes TRIGO|[4892]|92-SRNP-4379|651[0n]bp
Astraptes TRIGO|[4893]|97-SRNP-5939|651[0n]bp
Astraptes TRIGO|[4894]|93-SRNP-7152|651[0n]bp
Astraptes TRIGO|[4895]|93-SRNP-8663|651[0n]bp
Astraptes TRIGO|[4896]|97-SRNP-5453|651[0n]bp
Astraptes TRIGO|[4897]|02-SRNP-31820|651[0n]bp
[illegible]

Astraptes TRIGO|[4895]|93-SRNP-8663|651[0n]bp
Astraptes TRIGO|[4896]|97-SRNP-5453|651[0n]bp
Astraptes TRIGO|[4897]|02-SRNP-31820|651[0n]bp
Astraptes TRIGO|[4898]|02-SRNP-31786|651[0n]bp
Astraptes TRIGO|[4899]|92-SRNP-4372|651[0n]bp
Astraptes TRIGO|[4900]|97-SRNP-5173|651[0n]bp
Astraptes TRIGO|[4901]|87-SRNP-1357|642[0n]bp
Astraptes TRIGO|[4902]|91-SRNP-2522|642[0n]bp
Astraptes TRIGO|[4903]|01-SRNP-9111|645[0n]bp
Astraptes TRIGO|[4904]|96-SRNP-9543|651[0n]bp
Astraptes TRIGO|[4904]|96-SRNP-9543|651[0n]bp
Astraptes TRIGO|[4905]|06-SRNP-55035|558[0n]bp
Astraptes TRIGO|[4906]|05-SRNP-45110|669[0n]bp
Astraptes TRIGO|[4907]|05-SRNP-45111|669[0n]bp
Astraptes TRIGO|[4908]|05-SRNP-65453|669[0n]bp
Astraptes TRIGO|[4909]|06-SRNP-58075|669[0n]bp
Astraptes TRIGO|[4910]|06-SRNP-58074|669[0n]bp
Astraptes TRIGO|[4911]|06-SRNP-58073|669[0n]bp
Astraptes TRIGO|[4912]|06-SRNP-59785|669[0n]bp
Astraptes TRIGO|[4913]|07-SRNP-45559|669[0n]bp
Astraptes TRIGO|[4914]|08-SRNP-2317|645[0n]bp
Urbanus evona|[4915]|93-SRNP-3406|567[1n]bp
Urbanus evona|[4916]|90-SRNP-1654.1|600[1n]bp
Urbanus evona|[4917]|04-SRNP-45778|669[1n]bp
Urbanus evona|[4918]|05-SRNP-66411|669[0n]bp
Urbanus evona|[4919]|05-SRNP-59072|669[0n]bp
Urbanus evona|[4920]|05-SRNP-57365|669[0n]bp
Urbanus evona|[4921]|05-SRNP-57364|669[0n]bp
Urbanus evona|[4922]|05-SRNP-58937|669[0n]bp
Urbanus evona|[4923]|04-SRNP-15286|669[0n]bp
Urbanus evona|[4924]|04-SRNP-14470|669[0n]bp
Urbanus evona|[4925]|02-SRNP-32829|669[0n]bp
Urbanus evona|[4926]|02-SRNP-32830|669[0n]bp
Urbanus evona|[4927]|01-SRNP-14067|669[0n]bp
Urbanus evona|[4928]|04-SRNP-45787|669[0n]bp
Urbanus evona|[4929]|97-SRNP-4685|615[0n]bp
Urbanus evona|[4930]|02-SRNP-32828|669[0n]bp
Urbanus evona|[4931]|06-SRNP-57688|669[0n]bp
Urbanus evona|[4932]|07-SRNP-57988|669[0n]bp
Urbanus esta|[4933]|93-SRNP-5934|522[0n]bp
Urbanus esta|[4934]|97-SRNP-2009|519[0n]bp
Urbanus esta|[4935]|97-SRNP-2006|522[0n]bp
Urbanus esta|[4936]|04-SRNP-56961|669[0n]bp
Urbanus esta|[4937]|07-SRNP-45289|669[0n]bp
Urbanus esta|[4938]|06-SRNP-40068|585[0n]bp
Urbanus esta|[4939]|06-SRNP-1725|669[0n]bp
Urbanus esta|[4940]|93-SRNP-1169|384[0n]bp
Urbanus esta|[4941]|01-SRNP-3184|639[0n]bp
Urbanus esta|[4942]|06-SRNP-19547|609[0n]bp
Urbanus esta|[4943]|00-SRNP-1522|669[0n]bp
Urbanus esta|[4944]|06-SRNP-31215|669[0n]bp
Urbanus esta|[4945]|05-SRNP-416|669[1n]bp
Urbanus esta|[4946]|07-SRNP-65020|663[0n]bp
Urbanus esta|[4947]|06-SRNP-57925|663[0n]bp
Urbanus esta|[4948]|06-SRNP-2685|666[0n]bp
Urbanus esta|[4949]|01-SRNP-17208|669[1n]bp
Urbanus esta|[4950]|05-SRNP-65398|669[0n]bp
Urbanus esta|[4951]|07-SRNP-20182|669[0n]bp
Urbanus esta|[4952]|06-SRNP-31006|669[0n]bp
Urbanus esta|[4953]|05-SRNP-33820|669[0n]bp
Urbanus esta|[4954]|05-SRNP-58910|669[0n]bp
Urbanus esta|[4955]|06-SRNP-31058|669[0n]bp
Urbanus esta|[4956]|05-SRNP-59305|669[0n]bp
Urbanus esta|[4957]|01-SRNP-473|669[0n]bp
Urbanus esta|[4958]|02-SRNP-1933|669[0n]bp
Urbanus esta|[4959]|02-SRNP-1930|669[0n]bp
Urbanus esta|[4960]|00-SRNP-1520|669[0n]bp
Urbanus esta|[4961]|00-SRNP-1519|669[0n]bp
Urbanus esta|[4962]|01-SRNP-17209|669[0n]bp
Urbanus esta|[4963]|04-SRNP-15166|669[0n]bp
Urbanus esta|[4964]|04-SRNP-15168|669[0n]bp
Urbanus esta|[4965]|02-SRNP-1932|669[0n]bp
Urbanus esta|[4966]|02-SRNP-32029|642[0n]bp
Urbanus esta|[4967]|06-SRNP-19546|606[0n]bp
Urbanus esta|[4968]|07-SRNP-31014|669[0n]bp
Urbanus esta|[4969]|06-SRNP-30965|669[0n]bp
Urbanus esta|[4970]|06-SRNP-31059|669[0n]bp
Urbanus esta|[4971]|06-SRNP-1984|669[0n]bp
Urbanus esta|[4972]|06-SRNP-1745|669[0n]bp
Urbanus esta|[4973]|06-SRNP-30964|669[0n]bp
Urbanus esta|[4974]|06-SRNP-30990|669[0n]bp
Urbanus esta|[4975]|06-SRNP-30961|669[0n]bp
Urbanus esta|[4976]|06-SRNP-31004|669[0n]bp
Urbanus esta|[4977]|06-SRNP-1743|669[0n]bp
Urbanus esta|[4978]|06-SRNP-1985|669[0n]bp
Urbanus esta|[4979]|05-SRNP-59539|669[0n]bp
Urbanus esta|[4980]|05-SRNP-59065|669[0n]bp
Urbanus esta|[4981]|06-SRNP-1798|669[0n]bp
Urbanus esta|[4982]|06-SRNP-1747|669[0n]bp
Urbanus esta|[4983]|06-SRNP-1746|669[0n]bp
Urbanus esta|[4984]|06-SRNP-1982|669[0n]bp
Urbanus esta|[4985]|06-SRNP-2690|669[0n]bp
Urbanus esta|[4986]|06-SRNP-4071|669[0n]bp
Urbanus esta|[4987]|07-SRNP-56430|669[0n]bp
Urbanus esta|[4988]|07-SRNP-1190|669[0n]bp
Urbanus esta|[4989]|07-SRNP-65022|669[0n]bp
Urbanus esta|[4990]|07-SRNP-65019|669[0n]bp
Urbanus esta|[4991]|06-SRNP-1532|669[0n]bp
Urbanus esta|[4992]|06-SRNP-30959|669[0n]bp
Urbanus esta|[4993]|06-SRNP-30040|669[0n]bp
Urbanus esta|[4994]|06-SRNP-1983|669[0n]bp
Urbanus esta|[4995]|05-SRNP-417|669[0n]bp
Urbanus esta|[4996]|05-SRNP-41969|669[0n]bp
[illegible]

Urbanus esta|[4994]|06-SRNP-1983|669[illegible]
Urbanus esta|[4995]|05-SRNP-417|669[0n]bp
Urbanus esta|[4996]|05-SRNP-41969|669[0n]bp
Urbanus esta|[4997]|02-SRNP-2902|669[0n]bp
Urbanus esta|[4998]|01-SRNP-1157|669[0n]bp
Urbanus esta|[4999]|00-SRNP-20821|669[0n]bp
Urbanus esta|[5000]|02-SRNP-1896|669[0n]bp
Urbanus esta|[5001]|02-SRNP-1436|669[0n]bp
Urbanus esta|[5002]|02-SRNP-2373|669[0n]bp
Urbanus esta|[5003]|02-SRNP-1435|669[0n]bp
Urbanus esta|[5004]|02-SRNP-546|669[0n]bp
Urbanus esta|[5005]|01-SRNP-16946|669[0n]bp
Urbanus esta|[5006]|02-SRNP-32024|669[0n]bp
Urbanus esta|[5007]|04-SRNP-55662|669[0n]bp
Urbanus esta|[5008]|05-SRNP-30144|669[0n]bp
Urbanus esta|[5009]|04-SRNP-30928|669[0n]bp
Urbanus esta|[5010]|02-SRNP-2320|612[0n]bp
Urbanus esta|[5011]|02-SRNP-402|669[0n]bp
Urbanus esta|[5012]|02-SRNP-401|669[0n]bp
Urbanus esta|[5013]|06-SRNP-20646|669[5n]bp
Urbanus esta|[5014]|07-SRNP-58873|669[0n]bp
Urbanus esmeraldus|[5015]|97-SRNP-9206|384[0n]bp
Urbanus esmeraldus|[5016]|97-SRNP-9537|384[0n]bp
Urbanus esmeraldus|[5017]|97-SRNP-9643|384[0n]bp
Urbanus esmeraldus|[5018]|97-SRNP-9544|588[0n]bp
Urbanus esmeraldus|[5019]|92-SRNP-4090|567[0n]bp
Urbanus esmeraldus|[5020]|93-SRNP-6647.1|567[0n]bp
Urbanus esmeraldus|[5021]|02-SRNP-28832|669[0n]bp
Urbanus esmeraldus|[5022]|02-SRNP-28843|669[0n]bp
Urbanus esmeraldus|[5023]|99-SRNP-3218|669[0n]bp
Urbanus esmeraldus|[5024]|02-SRNP-28844|669[0n]bp
Urbanus esmeraldus|[5025]|06-SRNP-59207|603[0n]bp
Urbanus esmeraldus|[5026]|97-SRNP-9775|588[0n]bp
Urbanus esmeraldus|[5027]|06-SRNP-56793|618[2n]bp
Urbanus esmeraldus|[5028]|97-SRNP-9209|621[0n]bp
Urbanus esmeraldus|[5029]|97-SRNP-9213|609[0n]bp
Urbanus esmeraldus|[5030]|97-SRNP-9207|609[0n]bp
Urbanus esmeraldus|[5031]|97-SRNP-9532|609[0n]bp
Urbanus esmeraldus|[5032]|02-SRNP-15771|669[0n]bp
Urbanus esmeraldus|[5033]|01-SRNP-17432|372[0n]bp
Urbanus esmeraldus|[5034]|01-SRNP-17161|627[0n]bp
Urbanus esmeraldus|[5035]|07-SRNP-20116|669[0n]bp
Urbanus esmeraldus|[5036]|05-SRNP-56429|669[0n]bp
Urbanus esmeraldus|[5037]|05-SRNP-56430|627[0n]bp
Urbanus esmeraldus|[5038]|03-SRNP-1944|663[0n]bp
Urbanus esmeraldus|[5039]|02-SRNP-17015|660[0n]bp
Urbanus esmeraldus|[5040]|03-SRNP-20895|660[0n]bp
Urbanus esmeraldus|[5041]|01-SRNP-17567|666[0n]bp
Urbanus esmeraldus|[5042]|03-SRNP-17407|666[0n]bp
Urbanus esmeraldus|[5043]|05-SRNP-32493|666[0n]bp
Urbanus esmeraldus|[5044]|06-SRNP-31303|666[0n]bp
Urbanus esmeraldus|[5045]|06-SRNP-31301|666[0n]bp
Urbanus esmeraldus|[5046]|06-SRNP-31302|666[0n]bp
Urbanus esmeraldus|[5047]|06-SRNP-30507|669[1n]bp
Urbanus esmeraldus|[5048]|01-SRNP-17046|669[0n]bp
Urbanus esmeraldus|[5049]|01-SRNP-17434|669[0n]bp
Urbanus esmeraldus|[5050]|01-SRNP-17160|669[0n]bp
Urbanus esmeraldus|[5051]|01-SRNP-17141|669[0n]bp
Urbanus esmeraldus|[5052]|01-SRNP-17081|669[0n]bp
Urbanus esmeraldus|[5053]|99-SRNP-3219|669[0n]bp
Urbanus esmeraldus|[5054]|01-SRNP-17561|669[0n]bp
Urbanus esmeraldus|[5055]|02-SRNP-32156|669[0n]bp
Urbanus esmeraldus|[5056]|99-SRNP-7525|669[0n]bp
Urbanus esmeraldus|[5057]|03-SRNP-30344|669[0n]bp
Urbanus esmeraldus|[5058]|04-SRNP-22082|669[0n]bp
Urbanus esmeraldus|[5059]|04-SRNP-22250|669[0n]bp
Urbanus esmeraldus|[5060]|03-SRNP-17392|669[0n]bp
Urbanus esmeraldus|[5061]|03-SRNP-17393|669[0n]bp
Urbanus esmeraldus|[5062]|03-SRNP-17394|669[0n]bp
Urbanus esmeraldus|[5063]|06-SRNP-33147|669[0n]bp
Urbanus esmeraldus|[5064]|06-SRNP-45697|669[0n]bp
Urbanus esmeraldus|[5065]|05-SRNP-34511|669[0n]bp
Urbanus esmeraldus|[5066]|05-SRNP-32807|669[0n]bp
Urbanus esmeraldus|[5067]|05-SRNP-32806|669[0n]bp
Urbanus esmeraldus|[5068]|05-SRNP-34529|669[0n]bp
Urbanus esmeraldus|[5069]|05-SRNP-32491|669[0n]bp
Urbanus esmeraldus|[5070]|05-SRNP-59480|669[0n]bp
Urbanus esmeraldus|[5071]|05-SRNP-46986|669[0n]bp
Urbanus esmeraldus|[5072]|05-SRNP-45421|669[0n]bp
Urbanus esmeraldus|[5073]|05-SRNP-46937|669[0n]bp
Urbanus esmeraldus|[5074]|05-SRNP-59048|669[0n]bp
Urbanus esmeraldus|[5075]|04-SRNP-26751|669[0n]bp
Urbanus esmeraldus|[5076]|01-SRNP-17442|669[0n]bp
Urbanus esmeraldus|[5077]|02-SRNP-29483|669[0n]bp
Urbanus esmeraldus|[5078]|97-SRNP-4935|669[0n]bp
Urbanus esmeraldus|[5079]|04-SRNP-15171|669[0n]bp
Urbanus esmeraldus|[5080]|04-SRNP-16070|669[0n]bp
Urbanus esmeraldus|[5081]|04-SRNP-13825|669[0n]bp
Urbanus esmeraldus|[5082]|04-SRNP-14316|669[0n]bp
Urbanus esmeraldus|[5083]|04-SRNP-48164|669[0n]bp
Urbanus esmeraldus|[5084]|04-SRNP-13677|669[0n]bp
Urbanus esmeraldus|[5085]|04-SRNP-13824|669[0n]bp
Urbanus esmeraldus|[5086]|01-SRNP-17077|669[0n]bp
Urbanus esmeraldus|[5087]|01-SRNP-17185|669[0n]bp
Urbanus esmeraldus|[5088]|01-SRNP-17421|669[0n]bp
Urbanus esmeraldus|[5089]|01-SRNP-17080.01|669[0n]bp
Urbanus esmeraldus|[5090]|01-SRNP-17163|669[0n]bp
Urbanus esmeraldus|[5091]|01-SRNP-17433|669[0n]bp
Urbanus esmeraldus|[5092]|01-SRNP-11002|669[0n]bp
Urbanus esmeraldus|[5093]|02-SRNP-29162|669[0n]bp
Urbanus esmeraldus|[5094]|01-SRNP-17443|669[0n]bp
Urbanus esmeraldus|[5095]|02-SRNP-5542|669[0n]bp
Urbanus esmeraldus|[5096]|01-SRNP-17426|669[0n]bp
[illegible]

Urbanus esmeraldus|[5094]|01-SRNP-17443|669[0n]bp
Urbanus esmeraldus|[5095]|02-SRNP-5542|669[0n]bp
Urbanus esmeraldus|[5096]|01-SRNP-17426|669[0n]bp
Urbanus esmeraldus|[5097]|01-SRNP-17562|669[0n]bp
Urbanus esmeraldus|[5098]|02-SRNP-13383|669[0n]bp
Urbanus esmeraldus|[5099]|04-SRNP-45661|669[0n]bp
Urbanus esmeraldus|[5100]|04-SRNP-45660|669[0n]bp
Urbanus esmeraldus|[5101]|04-SRNP-45662|669[0n]bp
Urbanus esmeraldus|[5102]|05-SRNP-33816|666[0n]bp
Urbanus esmeraldus|[5103]|03-SRNP-16910|669[0n]bp
Urbanus esmeraldus|[5104]|01-SRNP-17439|669[0n]bp
Urbanus esmeraldus|[5105]|97-SRNP-4639|609[0n]bp
Urbanus esmeraldus|[5106]|97-SRNP-4567|609[0n]bp
Urbanus esmeraldus|[5107]|97-SRNP-4640|609[0n]bp
Urbanus esmeraldus|[5108]|99-SRNP-8598|645[0n]bp
Urbanus esmeraldus|[5109]|04-SRNP-45064|657[0n]bp
Urbanus esmeraldus|[5110]|01-SRNP-17131|660[0n]bp
Urbanus esmeraldus|[5111]|97-SRNP-4936|618[0n]bp
Urbanus esmeraldus|[5112]|97-SRNP-9658|609[0n]bp
Urbanus esmeraldus|[5113]|01-SRNP-17047|615[0n]bp
Urbanus esmeraldus|[5114]|97-SRNP-9211|615[0n]bp
Urbanus esmeraldus|[5115]|04-SRNP-21981|609[0n]bp
Urbanus esmeraldus|[5116]|04-SRNP-45065|609[0n]bp
Urbanus esmeraldus|[5117]|03-SRNP-19022|609[0n]bp
Urbanus esmeraldus|[5118]|03-SRNP-17465|609[0n]bp
Urbanus esmeraldus|[5119]|97-SRNP-5646|609[0n]bp
Urbanus esmeraldus|[5120]|97-SRNP-9554|609[0n]bp
Urbanus esmeraldus|[5121]|96-SRNP-11210|609[0n]bp
Urbanus esmeraldus|[5122]|97-SRNP-9552|609[0n]bp
Urbanus esmeraldus|[5123]|97-SRNP-9531|609[0n]bp
Urbanus esmeraldus|[5124]|97-SRNP-9530|609[0n]bp
Urbanus esmeraldus|[5125]|97-SRNP-9208|609[0n]bp
Urbanus esmeraldus|[5126]|97-SRNP-9647|609[0n]bp
Urbanus esmeraldus|[5127]|97-SRNP-9558|609[0n]bp
Urbanus esmeraldus|[5128]|97-SRNP-9645|609[0n]bp
Urbanus esmeraldus|[5129]|97-SRNP-9843|609[0n]bp
Urbanus esmeraldus|[5130]|97-SRNP-9646|609[0n]bp
Urbanus esmeraldus|[5131]|97-SRNP-5807|606[0n]bp
Urbanus esmeraldus|[5132]|05-SRNP-32029|636[0n]bp
Urbanus esmeraldus|[5133]|03-SRNP-29671|633[0n]bp
Urbanus esmeraldus|[5134]|02-SRNP-5961|609[0n]bp
Urbanus esmeraldus|[5135]|01-SRNP-17079|609[0n]bp
Urbanus esmeraldus|[5136]|01-SRNP-17158|609[0n]bp
Urbanus esmeraldus|[5137]|01-SRNP-17566|609[0n]bp
Urbanus esmeraldus|[5138]|95-SRNP-9803|609[0n]bp
Urbanus esmeraldus|[5139]|95-SRNP-9805|609[0n]bp
Urbanus esmeraldus|[5140]|95-SRNP-9968|609[0n]bp
Urbanus esmeraldus|[5141]|95-SRNP-8976|609[0n]bp
Urbanus esmeraldus|[5142]|01-SRNP-17083|609[0n]bp
Urbanus esmeraldus|[5143]|03-SRNP-16636|669[0n]bp
Urbanus esmeraldus|[5144]|06-SRNP-34518|669[0n]bp
Urbanus esmeraldus|[5145]|06-SRNP-46734|669[0n]bp
Urbanus esmeraldus|[5146]|07-SRNP-56341|669[0n]bp
Urbanus esmeraldus|[5147]|07-SRNP-56359|669[0n]bp
Urbanus esmeraldus|[5148]|07-SRNP-56490|669[0n]bp
Urbanus esmeraldus|[5149]|07-SRNP-20117|669[0n]bp
Urbanus esmeraldus|[5150]|07-SRNP-58471|669[0n]bp
Urbanus esmeraldus|[5151]|07-SRNP-57861|669[0n]bp
Urbanus esmeraldus|[5152]|07-SRNP-57862|669[0n]bp
Urbanus esmeraldus|[5153]|08-SRNP-21596|669[0n]bp
Urbanus esmeraldus|[5154]|08-SRNP-23543|669[0n]bp
Urbanus esmeraldus|[5155]|08-SRNP-58525|669[0n]bp
Urbanus proteus|[5156]|97-SRNP-2922|609[0n]bp
Urbanus proteus|[5157]|02-SRNP-17912|669[0n]bp
Urbanus proteus|[5158]|02-SRNP-17915|669[0n]bp
Urbanus proteus|[5159]|02-SRNP-15156|669[0n]bp
Urbanus proteus|[5160]|02-SRNP-28591|669[0n]bp
Urbanus proteus|[5161]|03-SRNP-16230|669[0n]bp
Urbanus proteus|[5162]|02-SRNP-17914|669[0n]bp
Urbanus proteus|[5163]|97-SRNP-4503|645[0n]bp
Urbanus proteus|[5164]|94-SRNP-744|609[0n]bp
Urbanus proteus|[5165]|07-SRNP-55548|669[0n]bp
Urbanus proteus|[5166]|08-SRNP-71742|669[0n]bp
Urbanus proteus|[5167]|03-SRNP-16609|633[0n]bp
Urbanus proteus|[5168]|04-SRNP-12473|669[1n]bp
Urbanus proteus|[5169]|04-SRNP-12477|669[0n]bp
Urbanus proteus|[5170]|02-SRNP-1925|669[0n]bp
Urbanus proteus|[5171]|04-SRNP-12475|669[0n]bp
Urbanus proteus|[5172]|01-SRNP-4528|624[2n]bp
Urbanus proteus|[5173]|06-SRNP-4935|669[0n]bp
Urbanus proteus|[5174]|06-SRNP-20639|669[0n]bp
Urbanus proteus|[5175]|03-SRNP-16767|645[0n]bp
Urbanus proteus|[5176]|06-SRNP-31010|669[0n]bp
Urbanus proteus|[5177]|05-SRNP-31866|669[0n]bp
Urbanus proteus|[5178]|04-SRNP-45320|669[0n]bp
Urbanus proteus|[5179]|04-SRNP-45321|669[0n]bp
Urbanus proteus|[5180]|06-SRNP-33300|666[0n]bp
Urbanus proteus|[5181]|02-SRNP-15123|669[0n]bp
Urbanus proteus|[5182]|03-SRNP-16621|669[0n]bp
Urbanus proteus|[5183]|03-SRNP-16637|669[0n]bp
Urbanus proteus|[5184]|03-SRNP-12324|669[0n]bp
Urbanus proteus|[5185]|03-SRNP-16601|669[0n]bp
Urbanus proteus|[5186]|03-SRNP-16778|669[0n]bp
Urbanus proteus|[5187]|02-SRNP-29399|669[0n]bp
Urbanus proteus|[5188]|03-SRNP-16782|669[0n]bp
Urbanus proteus|[5189]|03-SRNP-16605|669[0n]bp
Urbanus proteus|[5190]|98-SRNP-4627|669[0n]bp
Urbanus proteus|[5191]|02-SRNP-29473|669[0n]bp
Urbanus proteus|[5192]|02-SRNP-17900|669[0n]bp
Urbanus proteus|[5193]|02-SRNP-17897|669[0n]bp
Urbanus proteus|[5194]|02-SRNP-17894|669[0n]bp
Urbanus proteus|[5195]|01-SRNP-12172|669[0n]bp
Urbanus proteus|[5196]|02-SRNP-28088|669[0n]bp
[illegible]

Urbanus proteus|[5194]|02-SRNP-17894|669[0n]bp
Urbanus proteus|[5195]|01-SRNP-12172|669[0n]bp
Urbanus proteus|[5196]|02-SRNP-28088|669[0n]bp
Urbanus proteus|[5197]|02-SRNP-28087|669[0n]bp
Urbanus proteus|[5198]|97-SRNP-2035|627[0n]bp
Urbanus proteus|[5199]|95-SRNP-4168|615[0n]bp
Urbanus proteus|[5200]|02-SRNP-15336|468[0n]bp
Urbanus proteus|[5201]|97-SRNP-3765|624[0n]bp
Urbanus proteus|[5202]|97-SRNP-450|585[0n]bp
Urbanus proteus|[5203]|97-SRNP-4059|564[0n]bp
Urbanus proteus|[5204]|07-SRNP-65362|669[0n]bp
Urbanus proteus|[5205]|07-SRNP-65363|669[0n]bp
Urbanus proteus|[5206]|07-SRNP-65361|669[0n]bp
Urbanus proteus|[5207]|04-SRNP-22094|669[0n]bp
Urbanus proteus|[5208]|06-SRNP-20989|669[0n]bp
Urbanus proteus|[5209]|06-SRNP-20638|669[0n]bp
Urbanus proteus|[5210]|05-SRNP-42674|669[0n]bp
Urbanus proteus|[5211]|05-SRNP-42675|669[0n]bp
Urbanus proteus|[5212]|02-SRNP-28144|669[0n]bp
Urbanus proteus|[5213]|09-SRNP-44384|669[0n]bp
Urbanus proteus|[5214]|95-SRNP-4171|639[0n]bp
Urbanus proteus|[5215]|08-SRNP-65051|651[0n]bp
Urbanus proteus|[5216]|07-SRNP-20070|645[0n]bp
Urbanus proteus|[5217]|02-SRNP-1919|645[0n]bp
Urbanus proteus|[5218]|03-SRNP-16226|645[0n]bp
Urbanus proteus|[5219]|04-SRNP-45331|645[0n]bp
Urbanus proteus|[5220]|04-SRNP-22097|645[0n]bp
Urbanus proteus|[5221]|03-SRNP-16604|645[0n]bp
Urbanus proteus|[5222]|03-SRNP-16643|645[0n]bp
Urbanus proteus|[5223]|03-SRNP-2587|645[0n]bp
Urbanus proteus|[5224]|03-SRNP-2943|645[0n]bp
Urbanus proteus|[5225]|06-SRNP-20992|645[0n]bp
Urbanus proteus|[5226]|03-SRNP-16622|645[0n]bp
Urbanus proteus|[5227]|97-SRNP-4440|621[0n]bp
Urbanus proteus|[5228]|03-SRNP-16638|621[0n]bp
Urbanus proteus|[5229]|97-SRNP-5092|609[0n]bp
Urbanus proteus|[5230]|97-SRNP-2067|609[0n]bp
Urbanus proteus|[5231]|95-SRNP-4170|609[0n]bp
Urbanus proteus|[5232]|97-SRNP-3766|609[0n]bp
Urbanus proteus|[5233]|02-SRNP-28072|609[0n]bp
Urbanus proteus|[5234]|01-SRNP-452|609[0n]bp
Urbanus proteus|[5235]|01-SRNP-978|609[0n]bp
Urbanus proteus|[5236]|95-SRNP-6395|609[0n]bp
Urbanus proteus|[5237]|95-SRNP-9306|609[0n]bp
Urbanus proteus|[5238]|97-SRNP-3010|609[0n]bp
Urbanus proteus|[5239]|95-SRNP-9354|609[0n]bp
Urbanus proteus|[5240]|94-SRNP-3026|588[0n]bp
Urbanus proteus|[5241]|97-SRNP-2037|606[0n]bp
Urbanus proteus|[5242]|04-SRNP-45179|669[0n]bp
Urbanus proteus|[5243]|02-SRNP-28093|663[0n]bp
Urbanus proteus|[5244]|03-SRNP-16771|669[0n]bp
Urbanus proteus|[5245]|02-SRNP-15128|669[0n]bp
Urbanus proteus|[5246]|95-SRNP-4169|615[0n]bp
Urbanus proteus|[5247]|97-SRNP-5093|582[0n]bp
Urbanus proteus|[5248]|97-SRNP-4803|609[0n]bp
Urbanus proteus|[5249]|07-SRNP-65369|669[0n]bp
Urbanus proteus|[5250]|03-SRNP-16611|657[0n]bp
Urbanus proteus|[5251]|03-SRNP-16895|657[0n]bp
Urbanus proteus|[5252]|02-SRNP-28761|657[0n]bp
Urbanus proteus|[5253]|02-SRNP-28758|669[0n]bp
Urbanus proteus|[5254]|01-SRNP-443|609[0n]bp
Urbanus proteus|[5255]|04-SRNP-22098|654[0n]bp
Urbanus proteus|[5256]|04-SRNP-22007|654[0n]bp
Urbanus proteus|[5257]|04-SRNP-47421|669[0n]bp
Urbanus proteus|[5258]|04-SRNP-33891|669[0n]bp
Urbanus proteus|[5259]|05-SRNP-32458|669[0n]bp
Urbanus proteus|[5260]|05-SRNP-32459|669[0n]bp
Urbanus proteus|[5261]|04-SRNP-22087|669[0n]bp
Urbanus proteus|[5262]|03-SRNP-16631|669[0n]bp
Urbanus proteus|[5263]|03-SRNP-17441|669[0n]bp
Urbanus proteus|[5264]|07-SRNP-65360|669[0n]bp
Urbanus proteus|[5265]|08-SRNP-55989|669[0n]bp
Urbanus proteus|[5266]|08-SRNP-55990|669[0n]bp
Urbanus proteus|[5267]|02-SRNP-28070|663[0n]bp
Urbanus proteus|[5268]|04-SRNP-33890|669[0n]bp
Urbanus proteus|[5269]|06-SRNP-32779|609[0n]bp
Urbanus proteus|[5270]|97-SRNP-4502|618[0n]bp
Urbanus proteus|[5271]|04-SRNP-21999|669[0n]bp
Urbanus proteus|[5272]|04-SRNP-21804|654[1n]bp
Urbanus proteus|[5273]|07-SRNP-22160|666[0n]bp
Urbanus proteus|[5274]|02-SRNP-28876|633[0n]bp
Urbanus proteus|[5275]|05-SRNP-21817|669[0n]bp
Urbanus proteus|[5276]|02-SRNP-17496|633[0n]bp
Urbanus proteus|[5277]|03-SRNP-16618|612[0n]bp
Urbanus proteus|[5278]|94-SRNP-2742|615[0n]bp
Urbanus proteus|[5279]|94-SRNP-3012|603[1n]bp
Urbanus proteus|[5280]|01-SRNP-1156|666[0n]bp
Urbanus proteus|[5281]|03-SRNP-6987|657[0n]bp
Urbanus proteus|[5282]|01-SRNP-5597|669[0n]bp
Urbanus proteus|[5283]|03-SRNP-2029|669[0n]bp
Urbanus proteus|[5284]|02-SRNP-28921|669[0n]bp
Urbanus proteus|[5285]|06-SRNP-20652|669[0n]bp
Urbanus proteus|[5286]|08-SRNP-65133|669[0n]bp
Urbanus proteus|[5287]|02-SRNP-28879|669[1n]bp
Urbanus proteus|[5288]|04-SRNP-45319|669[1n]bp
Urbanus proteus|[5289]|03-SRNP-2585|651[1n]bp
Urbanus proteus|[5290]|04-SRNP-45322|669[0n]bp
Urbanus proteus|[5291]|04-SRNP-22092|669[0n]bp
Urbanus proteus|[5292]|04-SRNP-32060|669[0n]bp
Urbanus proteus|[5293]|03-SRNP-20447|669[0n]bp
Urbanus proteus|[5294]|06-SRNP-6068|669[0n]bp
Urbanus proteus|[5295]|06-SRNP-6067|669[0n]bp
Urbanus proteus|[5296]|02-SRNP-17493|669[0n]bp
[illegible]

Urbanus proteus|[5294]|06-SRNP-6068|669[0n]bp
Urbanus proteus|[5295]|06-SRNP-6067|669[0n]bp
Urbanus proteus|[5296]|02-SRNP-17493|669[0n]bp
Urbanus proteus|[5297]|02-SRNP-29476|669[0n]bp
Urbanus proteus|[5298]|03-SRNP-16629|669[0n]bp
Urbanus proteus|[5299]|02-SRNP-28091|669[0n]bp
Urbanus proteus|[5300]|02-SRNP-17905|669[0n]bp
Urbanus proteus|[5301]|02-SRNP-17917|669[0n]bp
Urbanus proteus|[5302]|02-SRNP-17500|669[0n]bp
Urbanus proteus|[5303]|04-SRNP-22439|669[0n]bp
Urbanus proteus|[5304]|04-SRNP-22142|669[0n]bp
Urbanus proteus|[5305]|04-SRNP-21276|669[0n]bp
Urbanus proteus|[5306]|04-SRNP-45255|669[0n]bp
Urbanus proteus|[5307]|04-SRNP-21421|669[0n]bp
Urbanus proteus|[5308]|03-SRNP-16772|645[0n]bp
Urbanus proteus|[5309]|02-SRNP-28089|669[0n]bp
Urbanus proteus|[5310]|05-SRNP-42099|669[0n]bp
Urbanus proteus|[5311]|04-SRNP-45328|669[1n]bp
Urbanus proteus|[5312]|04-SRNP-21992|669[0n]bp
Urbanus proteus|[5313]|04-SRNP-22095|669[0n]bp
Urbanus proteus|[5314]|06-SRNP-20650|669[0n]bp
Urbanus proteus|[5315]|02-SRNP-28763|669[0n]bp
Urbanus proteus|[5316]|04-SRNP-21997|669[0n]bp
Urbanus proteus|[5317]|04-SRNP-21991|645[0n]bp
Urbanus proteus|[5318]|04-SRNP-21993|669[0n]bp
Urbanus proteus|[5319]|03-SRNP-17430|666[0n]bp
Urbanus proteus|[5320]|05-SRNP-22290|666[0n]bp
Urbanus proteus|[5321]|07-SRNP-56187|666[0n]bp
Urbanus proteus|[5322]|06-SRNP-22060|666[1n]bp
Urbanus proteus|[5323]|06-SRNP-20672|669[0n]bp
Urbanus proteus|[5324]|06-SRNP-32781|612[1n]bp
Urbanus proteus|[5325]|02-SRNP-32259|627[0n]bp
Urbanus proteus|[5326]|02-SRNP-1921|669[0n]bp
Urbanus proteus|[5327]|03-SRNP-2512|639[0n]bp
Urbanus proteus|[5328]|03-SRNP-16896|636[0n]bp
Urbanus proteus|[5329]|03-SRNP-16625|669[0n]bp
Urbanus proteus|[5330]|03-SRNP-16606|669[0n]bp
Urbanus proteus|[5331]|02-SRNP-28094|669[0n]bp
Urbanus proteus|[5332]|07-SRNP-32675|531[0n]bp
Urbanus proteus|[5333]|08-SRNP-71996|669[0n]bp
Urbanus proteus|[5334]|09-SRNP-70674|669[1n]bp
Urbanus proteus|[5335]|02-SRNP-17909|669[0n]bp
Urbanus proteus|[5336]|02-SRNP-17892|669[0n]bp
Urbanus proteus|[5337]|06-SRNP-32816|669[0n]bp
Urbanus proteus|[5338]|02-SRNP-15161|669[0n]bp
Urbanus proteus|[5339]|06-SRNP-32782|669[0n]bp
Urbanus proteus|[5340]|06-SRNP-32807|669[0n]bp
Urbanus proteus|[5341]|06-SRNP-21095|669[0n]bp
Urbanus proteus|[5342]|06-SRNP-20993|669[0n]bp
Urbanus proteus|[5343]|06-SRNP-21060|669[0n]bp
Urbanus proteus|[5344]|06-SRNP-21281|669[0n]bp
Urbanus proteus|[5345]|06-SRNP-20649|669[0n]bp
Urbanus proteus|[5346]|06-SRNP-21046|669[0n]bp
Urbanus proteus|[5347]|05-SRNP-55628|669[0n]bp
Urbanus proteus|[5348]|06-SRNP-30958|669[0n]bp
Urbanus proteus|[5349]|05-SRNP-31865|669[0n]bp
Urbanus proteus|[5350]|05-SRNP-41526|669[0n]bp
Urbanus proteus|[5351]|05-SRNP-33815|669[0n]bp
Urbanus proteus|[5352]|05-SRNP-46456|669[0n]bp
Urbanus proteus|[5353]|02-SRNP-17495|669[0n]bp
Urbanus proteus|[5354]|01-SRNP-12344|669[0n]bp
Urbanus proteus|[5355]|02-SRNP-29166|669[0n]bp
Urbanus proteus|[5356]|02-SRNP-17910|669[0n]bp
Urbanus proteus|[5357]|02-SRNP-17902|669[0n]bp
Urbanus proteus|[5358]|02-SRNP-28882|669[0n]bp
Urbanus proteus|[5359]|02-SRNP-17499|669[0n]bp
Urbanus proteus|[5360]|02-SRNP-17904|669[0n]bp
Urbanus proteus|[5361]|01-SRNP-17048|669[0n]bp
Urbanus proteus|[5362]|02-SRNP-17891|669[0n]bp
Urbanus proteus|[5363]|01-SRNP-444|669[0n]bp
Urbanus proteus|[5364]|01-SRNP-5650|669[0n]bp
Urbanus proteus|[5365]|01-SRNP-448|669[0n]bp
Urbanus proteus|[5366]|01-SRNP-12349|669[0n]bp
Urbanus proteus|[5367]|01-SRNP-12371|669[0n]bp
Urbanus proteus|[5368]|01-SRNP-449|669[0n]bp
Urbanus proteus|[5369]|01-SRNP-12318|669[0n]bp
Urbanus proteus|[5370]|01-SRNP-12370|669[0n]bp
Urbanus proteus|[5371]|01-SRNP-12346|669[0n]bp
Urbanus proteus|[5372]|01-SRNP-12345|669[0n]bp
Urbanus proteus|[5373]|04-SRNP-45076|669[0n]bp
Urbanus proteus|[5374]|03-SRNP-7405|669[0n]bp
Urbanus proteus|[5375]|04-SRNP-45299|669[0n]bp
Urbanus proteus|[5376]|04-SRNP-45323|669[0n]bp
Urbanus proteus|[5377]|02-SRNP-31168|669[0n]bp
Urbanus proteus|[5378]|02-SRNP-28914|669[0n]bp
Urbanus proteus|[5379]|02-SRNP-16019|669[0n]bp
Urbanus proteus|[5380]|02-SRNP-28092|669[0n]bp
Urbanus proteus|[5381]|01-SRNP-446|669[0n]bp
Urbanus proteus|[5382]|01-SRNP-917|669[0n]bp
Urbanus proteus|[5383]|02-SRNP-17899|669[0n]bp
Urbanus proteus|[5384]|02-SRNP-17918|669[0n]bp
Urbanus proteus|[5385]|01-SRNP-447|669[0n]bp
Urbanus proteus|[5386]|02-SRNP-17896|669[0n]bp
Urbanus proteus|[5387]|01-SRNP-453|669[0n]bp
Urbanus proteus|[5388]|01-SRNP-4731|669[0n]bp
Urbanus proteus|[5389]|02-SRNP-15124|669[0n]bp
Urbanus proteus|[5390]|01-SRNP-12352|669[0n]bp
Urbanus proteus|[5391]|06-SRNP-33586|669[0n]bp
Urbanus proteus|[5392]|06-SRNP-33301|669[0n]bp
Urbanus proteus|[5393]|06-SRNP-32780|669[0n]bp
Urbanus proteus|[5394]|06-SRNP-32342|669[0n]bp
Urbanus proteus|[5395]|06-SRNP-21280|669[0n]bp
Urbanus proteus|[5396]|06-SRNP-20994|669[0n]bp
[illegible]

Urbanus proteus|[5394]|06-SRNP-32342|669[0n]bp
Urbanus proteus|[5395]|06-SRNP-21280|669[0n]bp
Urbanus proteus|[5396]|06-SRNP-20994|669[0n]bp
Urbanus proteus|[5397]|06-SRNP-31653|669[0n]bp
Urbanus proteus|[5398]|06-SRNP-20991|669[0n]bp
Urbanus proteus|[5399]|05-SRNP-24255|669[0n]bp
Urbanus proteus|[5400]|06-SRNP-45207|669[0n]bp
Urbanus proteus|[5401]|05-SRNP-32492|669[0n]bp
Urbanus proteus|[5402]|05-SRNP-32595|669[0n]bp
Urbanus proteus|[5403]|05-SRNP-42295|669[0n]bp
Urbanus proteus|[5404]|05-SRNP-31008|669[0n]bp
Urbanus proteus|[5405]|02-SRNP-15154|669[0n]bp
Urbanus proteus|[5406]|04-SRNP-12474|669[0n]bp
Urbanus proteus|[5407]|02-SRNP-17907|669[0n]bp
Urbanus proteus|[5408]|02-SRNP-29478|669[0n]bp
Urbanus proteus|[5409]|04-SRNP-22006|669[0n]bp
Urbanus proteus|[5410]|03-SRNP-16908|669[0n]bp
Urbanus proteus|[5411]|03-SRNP-16781|669[0n]bp
Urbanus proteus|[5412]|03-SRNP-16617|669[0n]bp
Urbanus proteus|[5413]|03-SRNP-2586|669[0n]bp
Urbanus proteus|[5414]|03-SRNP-16902|669[0n]bp
Urbanus proteus|[5415]|03-SRNP-8110|669[0n]bp
Urbanus proteus|[5416]|03-SRNP-2945|669[0n]bp
Urbanus proteus|[5417]|03-SRNP-16897|669[0n]bp
Urbanus proteus|[5418]|03-SRNP-2584|669[0n]bp
Urbanus proteus|[5419]|04-SRNP-22103|669[0n]bp
Urbanus proteus|[5420]|03-SRNP-2108|669[0n]bp
Urbanus proteus|[5421]|04-SRNP-22000|669[0n]bp
Urbanus proteus|[5422]|04-SRNP-21996|669[0n]bp
Urbanus proteus|[5423]|04-SRNP-22004|669[0n]bp
Urbanus proteus|[5424]|04-SRNP-45366|669[0n]bp
Urbanus proteus|[5425]|04-SRNP-32097|669[0n]bp
Urbanus proteus|[5426]|04-SRNP-22008|669[0n]bp
Urbanus proteus|[5427]|03-SRNP-16893|669[0n]bp
Urbanus proteus|[5428]|03-SRNP-16899|669[0n]bp
Urbanus proteus|[5429]|03-SRNP-16635|669[0n]bp
Urbanus proteus|[5430]|03-SRNP-16774|669[0n]bp
Urbanus proteus|[5431]|03-SRNP-2535|669[0n]bp
Urbanus proteus|[5432]|03-SRNP-16624|669[0n]bp
Urbanus proteus|[5433]|02-SRNP-15147|669[0n]bp
Urbanus proteus|[5434]|03-SRNP-2027|669[0n]bp
Urbanus proteus|[5435]|02-SRNP-28073|669[0n]bp
Urbanus proteus|[5436]|02-SRNP-7152|669[0n]bp
Urbanus proteus|[5437]|02-SRNP-17906|669[0n]bp
Urbanus proteus|[5438]|01-SRNP-976|669[0n]bp
Urbanus proteus|[5439]|02-SRNP-17901|669[0n]bp
Urbanus proteus|[5440]|02-SRNP-28917|669[0n]bp
Urbanus proteus|[5441]|02-SRNP-28874|669[0n]bp
Urbanus proteus|[5442]|02-SRNP-17911|669[0n]bp
Urbanus proteus|[5443]|03-SRNP-2940|669[0n]bp
Urbanus proteus|[5444]|03-SRNP-16894|669[0n]bp
Urbanus proteus|[5445]|03-SRNP-19131|669[0n]bp
Urbanus proteus|[5446]|03-SRNP-2367|669[0n]bp
Urbanus proteus|[5447]|03-SRNP-2588|669[0n]bp
Urbanus proteus|[5448]|03-SRNP-2028|669[0n]bp
Urbanus proteus|[5449]|04-SRNP-22090|669[0n]bp
Urbanus proteus|[5450]|04-SRNP-22089|669[0n]bp
Urbanus proteus|[5451]|03-SRNP-2093|669[0n]bp
Urbanus proteus|[5452]|04-SRNP-22002|669[0n]bp
Urbanus proteus|[5453]|03-SRNP-20714|669[0n]bp
Urbanus proteus|[5454]|03-SRNP-16616|669[0n]bp
Urbanus proteus|[5455]|04-SRNP-45332|669[0n]bp
Urbanus proteus|[5456]|03-SRNP-18903|669[0n]bp
Urbanus proteus|[5457]|02-SRNP-28096|669[0n]bp
Urbanus proteus|[5458]|02-SRNP-28071|669[0n]bp
Urbanus proteus|[5459]|02-SRNP-17898|669[0n]bp
Urbanus proteus|[5460]|02-SRNP-15366|669[0n]bp
Urbanus proteus|[5461]|02-SRNP-28090|669[0n]bp
Urbanus proteus|[5462]|02-SRNP-1917|669[0n]bp
Urbanus proteus|[5463]|03-SRNP-16594|669[0n]bp
Urbanus proteus|[5464]|03-SRNP-17445|669[0n]bp
Urbanus proteus|[5465]|03-SRNP-16769|669[0n]bp
Urbanus proteus|[5466]|03-SRNP-16898|669[0n]bp
Urbanus proteus|[5467]|03-SRNP-2030|669[0n]bp
Urbanus proteus|[5468]|03-SRNP-2112|669[0n]bp
Urbanus proteus|[5469]|03-SRNP-2583|669[0n]bp
Urbanus proteus|[5470]|04-SRNP-21995|669[0n]bp
Urbanus proteus|[5471]|03-SRNP-2537|669[0n]bp
Urbanus proteus|[5472]|03-SRNP-16770|669[0n]bp
Urbanus proteus|[5473]|03-SRNP-15468|669[0n]bp
Urbanus proteus|[5474]|03-SRNP-2944|669[0n]bp
Urbanus proteus|[5475]|03-SRNP-2536|669[0n]bp
Urbanus proteus|[5476]|03-SRNP-2582|669[0n]bp
Urbanus proteus|[5477]|03-SRNP-16634|669[0n]bp
Urbanus proteus|[5478]|03-SRNP-17406|669[0n]bp
Urbanus proteus|[5479]|09-SRNP-70673|669[0n]bp
Urbanus proteus|[5480]|09-SRNP-70675|669[0n]bp
Urbanus proteus|[5481]|09-SRNP-44562|669[0n]bp
Urbanus proteus|[5482]|09-SRNP-70361|669[0n]bp
Urbanus proteus|[5483]|09-SRNP-70360|669[0n]bp
Urbanus proteus|[5484]|09-SRNP-44561|669[0n]bp
Urbanus proteus|[5485]|09-SRNP-44385|669[0n]bp
Urbanus proteus|[5486]|09-SRNP-44317|669[0n]bp
Urbanus proteus|[5487]|08-SRNP-72574|669[0n]bp
Urbanus proteus|[5488]|08-SRNP-72588|669[0n]bp
Urbanus proteus|[5489]|08-SRNP-71676|669[0n]bp
Urbanus proteus|[5490]|08-SRNP-71995|669[0n]bp
Urbanus proteus|[5491]|08-SRNP-45057|669[0n]bp
Urbanus proteus|[5492]|08-SRNP-45169|669[0n]bp
Urbanus proteus|[5493]|08-SRNP-71277|669[0n]bp
Urbanus proteus|[5494]|08-SRNP-1259|669[0n]bp
Urbanus proteus|[5495]|08-SRNP-65631|669[0n]bp
Urbanus proteus|[5496]|08-SRNP-65632|669[0n]bp

Urbanus proteus|[5494]|08-SRNP-1259|669[0n]bp
Urbanus proteus|[5495]|08-SRNP-65631|669[0n]bp
Urbanus proteus|[5496]|08-SRNP-65632|669[0n]bp
Urbanus proteus|[5497]|08-SRNP-65633|669[0n]bp
Urbanus proteus|[5498]|08-SRNP-65634|669[0n]bp
Urbanus proteus|[5499]|07-SRNP-65419|669[0n]bp
Urbanus proteus|[5500]|08-SRNP-65052|669[0n]bp
Urbanus proteus|[5501]|07-SRNP-57891|669[0n]bp
Urbanus proteus|[5502]|07-SRNP-65273|669[0n]bp
Urbanus proteus|[5503]|07-SRNP-1369|669[0n]bp
Urbanus proteus|[5504]|07-SRNP-56212|669[0n]bp
Urbanus proteus|[5505]|07-SRNP-20079|669[0n]bp
Urbanus proteus|[5506]|07-SRNP-20455|669[0n]bp
Urbanus proteus|[5507]|02-SRNP-5760|669[0n]bp
Urbanus proteus|[5508]|02-SRNP-29402|669[0n]bp
Urbanus proteus|[5509]|07-SRNP-55788|669[0n]bp
Urbanus proteus|[5510]|07-SRNP-56167|669[0n]bp
Urbanus proteus|[5511]|07-SRNP-32038|669[0n]bp
Urbanus proteus|[5512]|07-SRNP-20045|669[0n]bp
Urbanus proteus|[5513]|02-SRNP-15127|669[0n]bp
Urbanus proteus|[5514]|02-SRNP-28881|669[0n]bp
Urbanus proteus|[5515]|02-SRNP-28760|669[0n]bp
Urbanus proteus|[5516]|97-SRNP-4472|669[0n]bp
Urbanus proteus|[5517]|02-SRNP-17913|669[0n]bp
Urbanus proteus|[5518]|02-SRNP-2958|669[0n]bp
Urbanus proteus|[5519]|01-SRNP-1274|669[0n]bp
Urbanus proteus|[5520]|02-SRNP-14208|669[0n]bp
Urbanus proteus|[5521]|02-SRNP-15153|669[0n]bp
Urbanus proteus|[5522]|01-SRNP-450|669[0n]bp
Urbanus proteus|[5523]|02-SRNP-15121|669[0n]bp
Urbanus proteus|[5524]|02-SRNP-15567|669[0n]bp
Urbanus proteus|[5525]|02-SRNP-15155|669[0n]bp
Urbanus proteus|[5526]|02-SRNP-2957|669[0n]bp
Urbanus proteus|[5527]|02-SRNP-29165|669[0n]bp
Urbanus proteus|[5528]|97-SRNP-9660|669[0n]bp
Urbanus proteus|[5529]|01-SRNP-12350|669[0n]bp
Urbanus proteus|[5530]|01-SRNP-445|669[0n]bp
Urbanus proteus|[5531]|02-SRNP-17908|669[0n]bp
Urbanus proteus|[5532]|01-SRNP-977|669[0n]bp
Urbanus proteus|[5533]|01-SRNP-451|669[0n]bp
Urbanus proteus|[5534]|01-SRNP-981|669[0n]bp
Urbanus proteus|[5535]|02-SRNP-28762|669[0n]bp
Urbanus proteus|[5536]|02-SRNP-28920|669[0n]bp
Urbanus proteus|[5537]|03-SRNP-16599|669[0n]bp
Urbanus proteus|[5538]|01-SRNP-1273|669[0n]bp
Urbanus proteus|[5539]|04-SRNP-45304|669[0n]bp
Urbanus proteus|[5540]|04-SRNP-45579|669[0n]bp
Urbanus proteus|[5541]|03-SRNP-17056|669[0n]bp
Urbanus proteus|[5542]|03-SRNP-20455|669[0n]bp
Urbanus proteus|[5543]|04-SRNP-45408|669[0n]bp
Urbanus proteus|[5544]|03-SRNP-16784|669[0n]bp
Urbanus proteus|[5545]|04-SRNP-45364|669[0n]bp
Urbanus proteus|[5546]|04-SRNP-1039|669[0n]bp
Urbanus proteus|[5547]|04-SRNP-45324|669[0n]bp
Urbanus proteus|[5548]|04-SRNP-45365|669[0n]bp
Urbanus proteus|[5549]|04-SRNP-22001|669[0n]bp
Urbanus proteus|[5550]|04-SRNP-22021|669[0n]bp
Urbanus proteus|[5551]|03-SRNP-16641|669[0n]bp
Urbanus proteus|[5552]|03-SRNP-16603|669[0n]bp
Urbanus proteus|[5553]|03-SRNP-16627|669[0n]bp
Urbanus proteus|[5554]|03-SRNP-16620|669[0n]bp
Urbanus proteus|[5555]|03-SRNP-16891|669[0n]bp
Urbanus proteus|[5556]|03-SRNP-16640|669[0n]bp
Urbanus proteus|[5557]|07-SRNP-21374|669[0n]bp
Urbanus proteus|[5558]|07-SRNP-22165|669[0n]bp
Urbanus proteus|[5559]|07-SRNP-1159|669[0n]bp
Urbanus proteus|[5560]|07-SRNP-3075|669[0n]bp
Urbanus proteus|[5561]|07-SRNP-21822|669[0n]bp
Urbanus proteus|[5562]|07-SRNP-45406|669[0n]bp
Urbanus proteus|[5563]|07-SRNP-56883|669[0n]bp
Urbanus proteus|[5564]|07-SRNP-57878|669[0n]bp
Urbanus proteus|[5565]|07-SRNP-65274|669[0n]bp
Urbanus proteus|[5566]|07-SRNP-65275|669[0n]bp
Urbanus proteus|[5567]|08-SRNP-65195|669[0n]bp
Urbanus proteus|[5568]|08-SRNP-55985|669[0n]bp
Urbanus proteus|[5569]|08-SRNP-55979|669[0n]bp
Urbanus proteus|[5570]|08-SRNP-65630|669[0n]bp
Urbanus proteus|[5571]|09-SRNP-57115|669[0n]bp
Urbanus proteus|[5572]|09-SRNP-75601|669[0n]bp
Urbanus belliDHJ02|[5573]|04-SRNP-45491|669[0n]bp
Urbanus belliDHJ02|[5574]|03-SRNP-12624.1|669[0n]bp
Urbanus belliDHJ02|[5575]|03-SRNP-12634.1|669[0n]bp
Urbanus belliDHJ02|[5576]|06-SRNP-47816|669[0n]bp
Urbanus belliDHJ02|[5577]|07-SRNP-45071|666[0n]bp
Urbanus belliDHJ02|[5578]|06-SRNP-30895|669[0n]bp
Urbanus belliDHJ02|[5579]|95-SRNP-10572|669[0n]bp
Urbanus belliDHJ02|[5580]|01-SRNP-2952|669[0n]bp
Urbanus belliDHJ02|[5581]|01-SRNP-4475|669[0n]bp
Urbanus belliDHJ02|[5582]|01-SRNP-487|669[0n]bp
Urbanus belliDHJ02|[5583]|06-SRNP-3621|669[0n]bp
Urbanus belliDHJ02|[5584]|07-SRNP-40856|669[0n]bp
Urbanus belliDHJ02|[5585]|06-SRNP-47814|630[0n]bp
Urbanus belliDHJ02|[5586]|06-SRNP-30896|669[0n]bp
Urbanus belliDHJ02|[5587]|06-SRNP-46174|669[0n]bp
Urbanus belliDHJ02|[5588]|06-SRNP-46171|645[0n]bp
Urbanus belliDHJ02|[5589]|06-SRNP-57638|609[0n]bp
Urbanus belliDHJ02|[5590]|02-SRNP-1417|669[0n]bp
Urbanus belliDHJ02|[5591]|04-SRNP-45586|669[0n]bp
Urbanus belliDHJ02|[5592]|95-SRNP-6826|669[0n]bp
Urbanus belliDHJ02|[5593]|02-SRNP-954|669[0n]bp
Urbanus belliDHJ02|[5594]|04-SRNP-14877|669[0n]bp
Urbanus belliDHJ02|[5595]|04-SRNP-49377|669[0n]bp
Urbanus belliDHJ02|[5596]|04-SRNP-15474|669[0n]bp

Urbanus belliDHJ02|[5594]|04-SRNP-14877|669[0n]bp
Urbanus belliDHJ02|[5595]|04-SRNP-49377|669[0n]bp
Urbanus belliDHJ02|[5596]|04-SRNP-15474|669[0n]bp
Urbanus belliDHJ02|[5597]|04-SRNP-15145|669[0n]bp
Urbanus belliDHJ02|[5598]|04-SRNP-48357|669[0n]bp
Urbanus belliDHJ02|[5599]|04-SRNP-47954|669[0n]bp
Urbanus belliDHJ02|[5600]|04-SRNP-14569|669[0n]bp
Urbanus belliDHJ02|[5601]|02-SRNP-32172|669[0n]bp
Urbanus belliDHJ02|[5602]|02-SRNP-392|669[0n]bp
Urbanus belliDHJ02|[5603]|02-SRNP-166|669[0n]bp
Urbanus belliDHJ02|[5604]|04-SRNP-20968|669[0n]bp
Urbanus belliDHJ02|[5605]|04-SRNP-20876|669[0n]bp
Urbanus belliDHJ02|[5606]|05-SRNP-59447|669[1n]bp
Urbanus belliDHJ02|[5607]|06-SRNP-47801|609[0n]bp
Urbanus belliDHJ02|[5608]|02-SRNP-1026|669[0n]bp
Urbanus belliDHJ02|[5609]|03-SRNP-12592|669[0n]bp
Urbanus belliDHJ02|[5610]|06-SRNP-46184|666[0n]bp
Urbanus belliDHJ02|[5611]|07-SRNP-55283|669[0n]bp
Urbanus belliDHJ02|[5612]|04-SRNP-45494|669[0n]bp
Urbanus belliDHJ02|[5613]|05-SRNP-1832|669[0n]bp
Urbanus belliDHJ02|[5614]|05-SRNP-47043|669[0n]bp
Urbanus belliDHJ02|[5615]|05-SRNP-47231|669[0n]bp
Urbanus belliDHJ02|[5616]|06-SRNP-2662|669[0n]bp
Urbanus belliDHJ02|[5617]|05-SRNP-19630|669[0n]bp
Urbanus belliDHJ02|[5618]|05-SRNP-59448|669[0n]bp
Urbanus belliDHJ02|[5619]|05-SRNP-59444|669[0n]bp
Urbanus belliDHJ02|[5620]|04-SRNP-45492|669[0n]bp
Urbanus belliDHJ02|[5621]|03-SRNP-29774|669[0n]bp
Urbanus belliDHJ02|[5622]|06-SRNP-2658|669[0n]bp
Urbanus belliDHJ02|[5623]|06-SRNP-46172|669[0n]bp
Urbanus belliDHJ02|[5624]|06-SRNP-46486|669[0n]bp
Urbanus belliDHJ02|[5625]|06-SRNP-46572|669[0n]bp
Urbanus belliDHJ02|[5626]|06-SRNP-46179|669[0n]bp
Urbanus belliDHJ02|[5627]|06-SRNP-46315|669[0n]bp
Urbanus belliDHJ02|[5628]|06-SRNP-6511|669[0n]bp
Urbanus belliDHJ02|[5629]|06-SRNP-46639|669[0n]bp
Urbanus belliDHJ02|[5630]|06-SRNP-46460|669[0n]bp
Urbanus belliDHJ02|[5631]|06-SRNP-47817|669[0n]bp
Urbanus belliDHJ02|[5632]|07-SRNP-40676|669[0n]bp
Urbanus belliDHJ02|[5633]|07-SRNP-30613|669[0n]bp
Urbanus belliDHJ02|[5634]|07-SRNP-56829|669[0n]bp
Urbanus belliDHJ02|[5635]|07-SRNP-56547|669[0n]bp
Urbanus belliDHJ02|[5636]|07-SRNP-30615|669[0n]bp
Urbanus belliDHJ02|[5637]|07-SRNP-20318|669[0n]bp
Urbanus belliDHJ02|[5638]|06-SRNP-18276|669[0n]bp
Urbanus belliDHJ02|[5639]|07-SRNP-57868|669[0n]bp
Urbanus belliDHJ02|[5640]|08-SRNP-31826|669[0n]bp
Urbanus viterboana|[5641]|03-SRNP-23514|669[0n]bp
Urbanus viterboana|[5642]|01-SRNP-21478|669[0n]bp
Urbanus viterboana|[5643]|01-SRNP-21220|669[0n]bp
Urbanus viterboana|[5644]|99-SRNP-363|669[0n]bp
Urbanus viterboana|[5645]|03-SRNP-23516|669[0n]bp
Urbanus viterboana|[5646]|06-SRNP-35186|669[0n]bp
Urbanus viterboana|[5647]|05-SRNP-35999|660[0n]bp
Urbanus viterboana|[5648]|01-SRNP-21475|669[0n]bp
Urbanus viterboana|[5649]|01-SRNP-21228|669[0n]bp
Urbanus viterboana|[5650]|01-SRNP-21233|627[0n]bp
Urbanus viterboana|[5651]|01-SRNP-21223|669[0n]bp
Urbanus viterboana|[5652]|99-SRNP-366|669[0n]bp
Urbanus viterboana|[5653]|05-SRNP-35553|669[0n]bp
Urbanus viterboana|[5654]|06-SRNP-35190|669[0n]bp
Urbanus viterboana|[5655]|07-SRNP-35463|669[0n]bp
Urbanus viterboana|[5656]|07-SRNP-35538|669[0n]bp
Urbanus viterboana|[5657]|08-SRNP-35218|669[0n]bp
Urbanus belliDHJ03|[5658]|03-SRNP-12654.1|669[0n]bp
Urbanus belliDHJ03|[5659]|06-SRNP-31264|324[0n]bp
Urbanus belliDHJ03|[5660]|07-SRNP-1121|645[0n]bp
Urbanus belliDHJ03|[5661]|07-SRNP-1122|645[0n]bp
Urbanus belliDHJ03|[5662]|06-SRNP-65065|660[0n]bp
Urbanus belliDHJ03|[5663]|07-SRNP-33182|660[0n]bp
Urbanus belliDHJ03|[5664]|08-SRNP-65993|669[0n]bp
Urbanus belliDHJ03|[5665]|08-SRNP-66028|669[0n]bp
Urbanus belliDHJ03|[5666]|07-SRNP-33183|669[0n]bp
Urbanus belliDHJ03|[5667]|07-SRNP-33184|669[0n]bp
Urbanus belliDHJ03|[5668]|07-SRNP-287|669[0n]bp
Urbanus belliDHJ03|[5669]|07-SRNP-40761|669[0n]bp
Urbanus belliDHJ03|[5670]|06-SRNP-46887|669[0n]bp
Urbanus belliDHJ03|[5671]|06-SRNP-7473|669[0n]bp
Urbanus belliDHJ03|[5672]|06-SRNP-46891|669[0n]bp
Urbanus belliDHJ03|[5673]|05-SRNP-7209|669[0n]bp
Urbanus belliDHJ03|[5674]|05-SRNP-40545|669[0n]bp
Urbanus belliDHJ03|[5675]|05-SRNP-41753|669[0n]bp
Urbanus belliDHJ03|[5676]|03-SRNP-12629.1|669[0n]bp
Urbanus belliDHJ03|[5677]|03-SRNP-10927|669[0n]bp
Urbanus belliDHJ03|[5678]|03-SRNP-12109.1|669[0n]bp
Urbanus belliDHJ03|[5679]|03-SRNP-10327|669[0n]bp
Urbanus belliDHJ03|[5680]|03-SRNP-12655.1|669[0n]bp
Urbanus belliDHJ03|[5681]|99-SRNP-5541|669[0n]bp
Urbanus belliDHJ03|[5682]|01-SRNP-23227|669[0n]bp
Urbanus belliDHJ03|[5683]|04-SRNP-42794|669[0n]bp
Urbanus belliDHJ03|[5684]|01-SRNP-22184|669[0n]bp
Urbanus belliDHJ03|[5685]|01-SRNP-23228|669[0n]bp
Urbanus belliDHJ03|[5686]|01-SRNP-2104|669[0n]bp
Urbanus belliDHJ03|[5687]|02-SRNP-7163|669[0n]bp
Urbanus belliDHJ03|[5688]|05-SRNP-42394|639[0n]bp
Urbanus belliDHJ03|[5689]|06-SRNP-43129|384[0n]bp
Urbanus belliDHJ03|[5690]|09-SRNP-57089|669[0n]bp
Urbanus belliDHJ01|[5691]|04-SRNP-49713|669[0n]bp
Urbanus belliDHJ01|[5692]|04-SRNP-49711|669[0n]bp
Urbanus belliDHJ01|[5693]|05-SRNP-49623|381[0n]bp
Urbanus belliDHJ01|[5694]|01-SRNP-22185|669[0n]bp
Urbanus belliDHJ01|[5695]|05-SRNP-43429|669[0n]bp
Urbanus belliDHJ01|[5696]|06-SRNP-31091|669[0n]bp

Urbanus belliDHJ01|[5694]|01-SRNP-22185|669[0n]bp
Urbanus belliDHJ01|[5695]|05-SRNP-43429|669[0n]bp
Urbanus belliDHJ01|[5696]|06-SRNP-31091|669[0n]bp
Urbanus belliDHJ01|[5697]|06-SRNP-46459|630[0n]bp
Urbanus belliDHJ01|[5698]|06-SRNP-19690|618[1n]bp
Urbanus belliDHJ01|[5699]|07-SRNP-1053|633[0n]bp
Urbanus belliDHJ01|[5700]|06-SRNP-47304|645[0n]bp
Urbanus belliDHJ01|[5701]|07-SRNP-23883|633[1n]bp
Urbanus belliDHJ01|[5702]|06-SRNP-46173|627[0n]bp
Urbanus belliDHJ01|[5703]|06-SRNP-48039|609[0n]bp
Urbanus belliDHJ01|[5704]|98-SRNP-4170|669[0n]bp
Urbanus belliDHJ01|[5705]|05-SRNP-47230|669[0n]bp
Urbanus belliDHJ01|[5706]|05-SRNP-42614|669[2n]bp
Urbanus belliDHJ01|[5707]|07-SRNP-45068|660[0n]bp
Urbanus belliDHJ01|[5708]|06-SRNP-47295|666[0n]bp
Urbanus belliDHJ01|[5709]|06-SRNP-31265|666[0n]bp
Urbanus belliDHJ01|[5710]|05-SRNP-60977|666[0n]bp
Urbanus belliDHJ01|[5711]|05-SRNP-42873|666[0n]bp
Urbanus belliDHJ01|[5712]|06-SRNP-40907|666[0n]bp
Urbanus belliDHJ01|[5713]|07-SRNP-20462|660[0n]bp
Urbanus belliDHJ01|[5714]|96-SRNP-11569|654[0n]bp
Urbanus belliDHJ01|[5715]|07-SRNP-41187|654[1n]bp
Urbanus belliDHJ01|[5716]|08-SRNP-1439|669[0n]bp
Urbanus belliDHJ01|[5717]|08-SRNP-65153|669[0n]bp
Urbanus belliDHJ01|[5718]|07-SRNP-65582|669[0n]bp
Urbanus belliDHJ01|[5719]|07-SRNP-33232|669[0n]bp
Urbanus belliDHJ01|[5720]|07-SRNP-1119|669[0n]bp
Urbanus belliDHJ01|[5721]|07-SRNP-42421|669[0n]bp
Urbanus belliDHJ01|[5722]|07-SRNP-42423|669[0n]bp
Urbanus belliDHJ01|[5723]|07-SRNP-31148|669[0n]bp
Urbanus belliDHJ01|[5724]|07-SRNP-31150|669[0n]bp
Urbanus belliDHJ01|[5725]|07-SRNP-20874|669[0n]bp
Urbanus belliDHJ01|[5726]|07-SRNP-31149|669[0n]bp
Urbanus belliDHJ01|[5727]|07-SRNP-20875|669[0n]bp
Urbanus belliDHJ01|[5728]|07-SRNP-30807|669[0n]bp
Urbanus belliDHJ01|[5729]|07-SRNP-30614|669[0n]bp
Urbanus belliDHJ01|[5730]|07-SRNP-45090|669[0n]bp
Urbanus belliDHJ01|[5731]|07-SRNP-45088|669[0n]bp
Urbanus belliDHJ01|[5732]|07-SRNP-40762|669[0n]bp
Urbanus belliDHJ01|[5733]|07-SRNP-30617|669[0n]bp
Urbanus belliDHJ01|[5734]|06-SRNP-60091|669[0n]bp
Urbanus belliDHJ01|[5735]|06-SRNP-47955|669[0n]bp
Urbanus belliDHJ01|[5736]|06-SRNP-59197|669[0n]bp
Urbanus belliDHJ01|[5737]|06-SRNP-46570|669[0n]bp
Urbanus belliDHJ01|[5738]|06-SRNP-46180|669[0n]bp
Urbanus belliDHJ01|[5739]|06-SRNP-46176|669[0n]bp
Urbanus belliDHJ01|[5740]|06-SRNP-47233|669[0n]bp
Urbanus belliDHJ01|[5741]|06-SRNP-7474|669[0n]bp
Urbanus belliDHJ01|[5742]|06-SRNP-6164|669[0n]bp
Urbanus belliDHJ01|[5743]|06-SRNP-46703|669[0n]bp
Urbanus belliDHJ01|[5744]|06-SRNP-7378|669[0n]bp
Urbanus belliDHJ01|[5745]|06-SRNP-46183|669[0n]bp
Urbanus belliDHJ01|[5746]|06-SRNP-46487|669[0n]bp
Urbanus belliDHJ01|[5747]|06-SRNP-46333|669[0n]bp
Urbanus belliDHJ01|[5748]|06-SRNP-43281|669[0n]bp
Urbanus belliDHJ01|[5749]|06-SRNP-32923|669[0n]bp
Urbanus belliDHJ01|[5750]|06-SRNP-46181|669[0n]bp
Urbanus belliDHJ01|[5751]|06-SRNP-5512|669[0n]bp
Urbanus belliDHJ01|[5752]|06-SRNP-30699|669[0n]bp
Urbanus belliDHJ01|[5753]|06-SRNP-3004|669[0n]bp
Urbanus belliDHJ01|[5754]|06-SRNP-2656|669[0n]bp
Urbanus belliDHJ01|[5755]|05-SRNP-43001|669[0n]bp
Urbanus belliDHJ01|[5756]|06-SRNP-40981|669[0n]bp
Urbanus belliDHJ01|[5757]|06-SRNP-2074|669[0n]bp
Urbanus belliDHJ01|[5758]|06-SRNP-2199|669[0n]bp
Urbanus belliDHJ01|[5759]|06-SRNP-30737|669[0n]bp
Urbanus belliDHJ01|[5760]|05-SRNP-40888|669[0n]bp
Urbanus belliDHJ01|[5761]|05-SRNP-45196|669[0n]bp
Urbanus belliDHJ01|[5762]|05-SRNP-42399|669[0n]bp
Urbanus belliDHJ01|[5763]|05-SRNP-40898|669[0n]bp
Urbanus belliDHJ01|[5764]|05-SRNP-47058|669[0n]bp
Urbanus belliDHJ01|[5765]|05-SRNP-42700|669[0n]bp
Urbanus belliDHJ01|[5766]|05-SRNP-47229|669[0n]bp
Urbanus belliDHJ01|[5767]|05-SRNP-20694|669[0n]bp
Urbanus belliDHJ01|[5768]|05-SRNP-47232|669[0n]bp
Urbanus belliDHJ01|[5769]|04-SRNP-4302|669[0n]bp
Urbanus belliDHJ01|[5770]|04-SRNP-21913|669[0n]bp
Urbanus belliDHJ01|[5771]|03-SRNP-12633.1|669[0n]bp
Urbanus belliDHJ01|[5772]|03-SRNP-12657.1|669[0n]bp
Urbanus belliDHJ01|[5773]|03-SRNP-12627.1|669[0n]bp
Urbanus belliDHJ01|[5774]|03-SRNP-12947.1|669[0n]bp
Urbanus belliDHJ01|[5775]|03-SRNP-11566|669[0n]bp
Urbanus belliDHJ01|[5776]|03-SRNP-12345.1|669[0n]bp
Urbanus belliDHJ01|[5777]|03-SRNP-10529|669[0n]bp
Urbanus belliDHJ01|[5778]|03-SRNP-12298.1|669[0n]bp
Urbanus belliDHJ01|[5779]|03-SRNP-11211|669[0n]bp
Urbanus belliDHJ01|[5780]|04-SRNP-21912|669[0n]bp
Urbanus belliDHJ01|[5781]|01-SRNP-23117|669[0n]bp
Urbanus belliDHJ01|[5782]|02-SRNP-7107|669[0n]bp
Urbanus belliDHJ01|[5783]|01-SRNP-1028|669[0n]bp
Urbanus belliDHJ01|[5784]|01-SRNP-22505|669[0n]bp
Urbanus belliDHJ01|[5785]|00-SRNP-22025|669[0n]bp
Urbanus belliDHJ01|[5786]|99-SRNP-2440|669[0n]bp
Urbanus belliDHJ01|[5787]|02-SRNP-590|669[0n]bp
Urbanus belliDHJ01|[5788]|02-SRNP-7734|669[0n]bp
Urbanus belliDHJ01|[5789]|01-SRNP-5700|669[0n]bp
Urbanus belliDHJ01|[5790]|00-SRNP-1534|669[0n]bp
Urbanus belliDHJ01|[5791]|00-SRNP-2200|669[0n]bp
Urbanus belliDHJ01|[5792]|00-SRNP-22026|669[0n]bp
Urbanus belliDHJ01|[5793]|02-SRNP-12992|669[0n]bp
Urbanus belliDHJ01|[5794]|04-SRNP-49712|669[0n]bp
Urbanus belliDHJ01|[5795]|04-SRNP-49960|669[0n]bp
Urbanus belliDHJ01|[5796]|04-SRNP-49714|669[0n]bp

Urbanus belliDHJ01|[5794]|04-SRNP-49712|669[0n]bp
Urbanus belliDHJ01|[5795]|04-SRNP-49960|669[0n]bp
Urbanus belliDHJ01|[5796]|04-SRNP-49714|669[0n]bp
Urbanus belliDHJ01|[5797]|04-SRNP-49963|669[0n]bp
Urbanus belliDHJ01|[5798]|04-SRNP-41942|669[0n]bp
Urbanus belliDHJ01|[5799]|04-SRNP-15157|669[0n]bp
Urbanus belliDHJ01|[5800]|04-SRNP-15160|669[0n]bp
Urbanus belliDHJ01|[5801]|04-SRNP-15153|669[0n]bp
Urbanus belliDHJ01|[5802]|04-SRNP-15679|669[0n]bp
Urbanus belliDHJ01|[5803]|04-SRNP-15149|669[0n]bp
Urbanus belliDHJ01|[5804]|04-SRNP-15189|669[0n]bp
Urbanus belliDHJ01|[5805]|04-SRNP-15161|669[0n]bp
Urbanus belliDHJ01|[5806]|04-SRNP-15155|669[0n]bp
Urbanus belliDHJ01|[5807]|04-SRNP-15147|669[0n]bp
Urbanus belliDHJ01|[5808]|04-SRNP-15146|669[0n]bp
Urbanus belliDHJ01|[5809]|04-SRNP-15154|669[0n]bp
Urbanus belliDHJ01|[5810]|04-SRNP-15137|669[0n]bp
Urbanus belliDHJ01|[5811]|04-SRNP-15162|669[0n]bp
Urbanus belliDHJ01|[5812]|04-SRNP-15150|669[0n]bp
Urbanus belliDHJ01|[5813]|04-SRNP-15674|669[0n]bp
Urbanus belliDHJ01|[5814]|04-SRNP-15680|669[0n]bp
Urbanus belliDHJ01|[5815]|04-SRNP-15677|669[0n]bp
Urbanus belliDHJ01|[5816]|04-SRNP-48641|669[0n]bp
Urbanus belliDHJ01|[5817]|04-SRNP-48028|669[0n]bp
Urbanus belliDHJ01|[5818]|04-SRNP-48033|669[0n]bp
Urbanus belliDHJ01|[5819]|04-SRNP-47563|669[0n]bp
Urbanus belliDHJ01|[5820]|04-SRNP-14307|669[0n]bp
Urbanus belliDHJ01|[5821]|04-SRNP-14390|669[0n]bp
Urbanus belliDHJ01|[5822]|04-SRNP-14389|669[0n]bp
Urbanus belliDHJ01|[5823]|04-SRNP-41681|669[0n]bp
Urbanus belliDHJ01|[5824]|04-SRNP-41680|669[0n]bp
Urbanus belliDHJ01|[5825]|04-SRNP-48639|669[0n]bp
Urbanus belliDHJ01|[5826]|04-SRNP-47956|669[0n]bp
Urbanus belliDHJ01|[5827]|04-SRNP-47824|669[0n]bp
Urbanus belliDHJ01|[5828]|04-SRNP-14108|669[0n]bp
Urbanus belliDHJ01|[5829]|01-SRNP-5310|669[0n]bp
Urbanus belliDHJ01|[5830]|01-SRNP-5311|669[0n]bp
Urbanus belliDHJ01|[5831]|01-SRNP-3745|669[0n]bp
Urbanus belliDHJ01|[5832]|01-SRNP-3404|669[0n]bp
Urbanus belliDHJ01|[5833]|01-SRNP-23060|669[0n]bp
Urbanus belliDHJ01|[5834]|02-SRNP-13670|669[0n]bp
Urbanus belliDHJ01|[5835]|02-SRNP-2246|669[0n]bp
Urbanus belliDHJ01|[5836]|02-SRNP-28991|669[0n]bp
Urbanus belliDHJ01|[5837]|02-SRNP-32169|669[0n]bp
Urbanus belliDHJ01|[5838]|02-SRNP-11533|669[0n]bp
Urbanus belliDHJ01|[5839]|02-SRNP-13111|669[0n]bp
Urbanus belliDHJ01|[5840]|02-SRNP-1028|669[0n]bp
Urbanus belliDHJ01|[5841]|02-SRNP-13671|669[0n]bp
Urbanus belliDHJ01|[5842]|02-SRNP-163|669[0n]bp
Urbanus belliDHJ01|[5843]|04-SRNP-33067|669[0n]bp
Urbanus belliDHJ01|[5844]|04-SRNP-21152|669[0n]bp
Urbanus belliDHJ01|[5845]|04-SRNP-31053|669[0n]bp
Urbanus belliDHJ01|[5846]|04-SRNP-22913|669[0n]bp
Urbanus belliDHJ01|[5847]|04-SRNP-45034|669[0n]bp
Urbanus belliDHJ01|[5848]|04-SRNP-20680|669[0n]bp
Urbanus belliDHJ01|[5849]|04-SRNP-20872|669[0n]bp
Urbanus belliDHJ01|[5850]|04-SRNP-20925|669[0n]bp
Urbanus belliDHJ01|[5851]|06-SRNP-59672|669[0n]bp
Urbanus belliDHJ01|[5852]|04-SRNP-47963|669[0n]bp
Urbanus belliDHJ01|[5853]|04-SRNP-47882|669[0n]bp
Urbanus belliDHJ01|[5854]|04-SRNP-47558|669[0n]bp
Urbanus belliDHJ01|[5855]|04-SRNP-14305|669[2n]bp
Urbanus belliDHJ01|[5856]|06-SRNP-40146|630[0n]bp
Urbanus belliDHJ01|[5857]|07-SRNP-20167|645[0n]bp
Urbanus belliDHJ01|[5858]|01-SRNP-3403|669[0n]bp
Urbanus belliDHJ01|[5859]|02-SRNP-21477|669[0n]bp
Urbanus belliDHJ01|[5860]|03-SRNP-12581.1|669[0n]bp
Urbanus belliDHJ01|[5861]|03-SRNP-12948.1|669[0n]bp
Urbanus belliDHJ01|[5862]|07-SRNP-41928|669[0n]bp
Urbanus belliDHJ01|[5863]|08-SRNP-4727|669[0n]bp
Urbanus belliDHJ01|[5864]|08-SRNP-65935|669[0n]bp
Urbanus belliDHJ01|[5865]|08-SRNP-5848|669[0n]bp
Urbanus belliDHJ01|[5866]|08-SRNP-23800|669[0n]bp
Urbanus belliDHJ01|[5867]|08-SRNP-24278|669[0n]bp
Urbanus belliDHJ01|[5868]|09-SRNP-69873|669[0n]bp
Astraptes tucuti|[5869]|98-SRNP-15995|657[0n]bp
Astraptes tucuti|[5870]|01-SRNP-21502|642[0n]bp
Astraptes tucuti|[5871]|01-SRNP-21504|630[0n]bp
Astraptes tucuti|[5872]|04-SRNP-3376|669[0n]bp
Astraptes tucuti|[5873]|05-SRNP-4479|645[2n]bp
Astraptes tucuti|[5874]|05-SRNP-5094|651[1n]bp
Astraptes tucuti|[5875]|07-SRNP-42005|645[0n]bp
Astraptes tucuti|[5876]|04-SRNP-35322|666[0n]bp
Astraptes tucuti|[5877]|04-SRNP-35430|666[0n]bp
Astraptes tucuti|[5878]|04-SRNP-35323|666[0n]bp
Astraptes tucuti|[5879]|02-SRNP-3446|639[0n]bp
Astraptes tucuti|[5880]|07-SRNP-1173|669[0n]bp
Astraptes tucuti|[5881]|06-SRNP-41042|669[0n]bp
Astraptes tucuti|[5882]|06-SRNP-2459|669[0n]bp
Astraptes tucuti|[5883]|05-SRNP-4645|669[0n]bp
Astraptes tucuti|[5884]|04-SRNP-2718|669[0n]bp
Astraptes tucuti|[5885]|04-SRNP-60520|669[0n]bp
Astraptes tucuti|[5886]|04-SRNP-60491|669[0n]bp
Astraptes tucuti|[5887]|07-SRNP-2625|639[0n]bp
Astraptes tucuti|[5888]|07-SRNP-1968|609[0n]bp
Astraptes tucuti|[5889]|07-SRNP-35969|669[0n]bp
Astraptes tucuti|[5890]|07-SRNP-2349|669[0n]bp
Astraptes tucuti|[5891]|09-SRNP-56674|669[0n]bp
Urbanus pronta|[5892]|04-SRNP-46061|669[0n]bp
Urbanus pronta|[5893]|04-SRNP-46058|669[0n]bp
Urbanus pronta|[5894]|04-SRNP-46056|669[0n]bp
Urbanus pronta|[5895]|04-SRNP-46057|669[0n]bp
Urbanus pronta|[5896]|08-SRNP-36361|669[0n]bp

Urbanus pronta|[5894]|04-SRNP-46056|669[0n]bp
Urbanus pronta|[5895]|04-SRNP-46057|669[0n]bp
Urbanus pronta|[5896]|08-SRNP-36361|669[0n]bp
Urbanus pronta|[5897]|06-SRNP-9393|669[0n]bp
Urbanus pronta|[5898]|06-SRNP-45648|669[0n]bp
Urbanus pronta|[5899]|06-SRNP-45649|669[0n]bp
Urbanus pronta|[5900]|06-SRNP-3187|669[0n]bp
Urbanus pronta|[5901]|01-SRNP-3205|669[0n]bp
Urbanus pronta|[5902]|01-SRNP-22501|669[0n]bp
Urbanus pronta|[5903]|02-SRNP-17474|669[0n]bp
Urbanus pronta|[5904]|02-SRNP-18620|669[0n]bp
Urbanus pronta|[5905]|02-SRNP-27305|669[0n]bp
Urbanus pronta|[5906]|02-SRNP-27308|669[0n]bp
Urbanus pronta|[5907]|02-SRNP-1605|669[0n]bp
Urbanus pronta|[5908]|02-SRNP-17477|669[3n]bp
Urbanus pronta|[5909]|02-SRNP-17476|606[0n]bp
Urbanus pronta|[5910]|06-SRNP-5958|507[0n]bp
Urbanus pronta|[5911]|06-SRNP-23251|654[1n]bp
Urbanus pronta|[5912]|09-SRNP-58058|669[0n]bp
Urbanus pronta|[5913]|09-SRNP-58053|669[0n]bp
Astraptes apastus|[5914]|03-SRNP-20746|663[0n]bp
Astraptes apastus|[5915]|07-SRNP-40564|669[0n]bp
Astraptes brevicauda|[5916]|03-SRNP-11830|669[0n]bp
Astraptes brevicauda|[5917]|08-SRNP-1126|669[0n]bp
Astraptes brevicauda|[5918]|05-SRNP-40306|669[0n]bp
Astraptes brevicauda|[5919]|01-SRNP-3278|630[0n]bp
Astraptes brevicauda|[5920]|01-SRNP-5574|618[2n]bp
Astraptes brevicauda|[5921]|05-SRNP-41750|669[2n]bp
Astraptes brevicauda|[5922]|04-SRNP-23822|669[0n]bp
Astraptes brevicauda|[5923]|04-SRNP-41627|669[0n]bp
Astraptes brevicauda|[5924]|04-SRNP-42195|669[0n]bp
Astraptes brevicauda|[5925]|04-SRNP-4716|669[0n]bp
Astraptes brevicauda|[5926]|03-SRNP-11273|669[0n]bp
Astraptes brevicauda|[5927]|03-SRNP-6444|669[0n]bp
Astraptes brevicauda|[5928]|03-SRNP-6247|669[0n]bp
Astraptes brevicauda|[5929]|05-SRNP-390|669[0n]bp
Astraptes brevicauda|[5930]|05-SRNP-41809|669[0n]bp
Astraptes brevicauda|[5931]|06-SRNP-31309|669[0n]bp
Astraptes brevicauda|[5932]|06-SRNP-3274|669[0n]bp
Astraptes brevicauda|[5933]|07-SRNP-1174|669[0n]bp
Astraptes brevicauda|[5934]|05-SRNP-7334|669[0n]bp
Astraptes brevicauda|[5935]|07-SRNP-66036|669[0n]bp
Astraptes brevicauda|[5936]|08-SRNP-1013|669[0n]bp
Astraptes brevicauda|[5937]|08-SRNP-5071|669[0n]bp
Astraptes anaphus annettaDHJ01|[5938]|04-SRNP-686|669[0n]bp
Astraptes anaphus annettaDHJ01|[5939]|04-SRNP-1067|669[0n]bp
Astraptes anaphus annettaDHJ01|[5940]|01-SRNP-16954|669[0n]bp
Astraptes anaphus annettaDHJ01|[5941]|02-SRNP-28707|669[0n]bp
Astraptes anaphus annettaDHJ01|[5942]|07-SRNP-33566|669[0n]bp
Astraptes anaphus annettaDHJ03|[5943]|97-SRNP-417|618[0n]bp
Astraptes anaphus annettaDHJ03|[5944]|97-SRNP-451|609[0n]bp
Astraptes anaphus annettaDHJ03|[5945]|02-SRNP-28432|660[0n]bp
Astraptes anaphus annettaDHJ03|[5946]|00-SRNP-20224|657[0n]bp
Astraptes anaphus annettaDHJ03|[5947]|97-SRNP-492|624[0n]bp
Astraptes anaphus annettaDHJ03|[5948]|93-SRNP-4847|609[0n]bp
Astraptes anaphus annettaDHJ03|[5949]|03-SRNP-26790|669[0n]bp
Astraptes anaphus annettaDHJ03|[5950]|97-SRNP-457|609[0n]bp
Astraptes anaphus annettaDHJ03|[5951]|93-SRNP-7914|615[0n]bp
Astraptes anaphus annettaDHJ03|[5952]|97-SRNP-462|669[0n]bp
Astraptes anaphus annettaDHJ03|[5953]|02-SRNP-5751|669[0n]bp
Astraptes anaphus annettaDHJ03|[5954]|02-SRNP-5101|669[0n]bp
Astraptes anaphus annettaDHJ03|[5955]|02-SRNP-5100|669[0n]bp
Astraptes anaphus annettaDHJ03|[5956]|02-SRNP-29489|633[0n]bp
Astraptes anaphus annettaDHJ03|[5957]|94-SRNP-7681|618[0n]bp
Astraptes anaphus annettaDHJ03|[5958]|06-SRNP-46316|669[0n]bp
Astraptes anaphus annettaDHJ03|[5959]|07-SRNP-20031|669[0n]bp
Astraptes anaphus annettaDHJ03|[5960]|07-SRNP-20299|669[0n]bp
Astraptes anaphus annettaDHJ03|[5961]|07-SRNP-20217|669[0n]bp
Astraptes anaphus annettaDHJ03|[5962]|07-SRNP-23872|669[0n]bp
Astraptes anaphus annettaDHJ03|[5963]|94-SRNP-7840|645[0n]bp
Astraptes anaphus annettaDHJ03|[5964]|00-SRNP-20296|669[0n]bp
Astraptes anaphus annettaDHJ03|[5965]|06-SRNP-3246|633[0n]bp
Astraptes anaphus annettaDHJ03|[5966]|00-SRNP-20174|642[0n]bp
Astraptes anaphus annettaDHJ03|[5967]|94-SRNP-7838|669[0n]bp
Astraptes anaphus annettaDHJ03|[5968]|93-SRNP-6735|609[0n]bp
Astraptes anaphus annettaDHJ03|[5969]|92-SRNP-4964|609[0n]bp
Astraptes anaphus annettaDHJ03|[5970]|97-SRNP-10083|609[0n]bp
Astraptes anaphus annettaDHJ03|[5971]|92-SRNP-4961|609[0n]bp
Astraptes anaphus annettaDHJ03|[5972]|07-SRNP-20148|669[0n]bp
Astraptes anaphus annettaDHJ03|[5973]|02-SRNP-28615|660[0n]bp
Astraptes anaphus annettaDHJ03|[5974]|05-SRNP-2000|687[0n]bp
Astraptes anaphus annettaDHJ03|[5975]|00-SRNP-19298|669[0n]bp
Astraptes anaphus annettaDHJ03|[5976]|00-SRNP-20267|669[0n]bp
Astraptes anaphus annettaDHJ03|[5977]|97-SRNP-11538|669[0n]bp
Astraptes anaphus annettaDHJ03|[5978]|07-SRNP-20264|669[0n]bp
Astraptes anaphus annettaDHJ03|[5979]|93-SRNP-8375|669[0n]bp
Astraptes anaphus annettaDHJ03|[5980]|00-SRNP-17721|669[0n]bp
Astraptes anaphus annettaDHJ03|[5981]|02-SRNP-28828|669[0n]bp
Astraptes anaphus annettaDHJ03|[5982]|00-SRNP-6012|669[0n]bp
Astraptes anaphus annettaDHJ03|[5983]|01-SRNP-17042|669[0n]bp
Astraptes anaphus annettaDHJ03|[5984]|97-SRNP-9242|669[0n]bp
Astraptes anaphus annettaDHJ03|[5985]|00-SRNP-20270|669[0n]bp
Astraptes anaphus annettaDHJ03|[5986]|02-SRNP-30063|669[0n]bp
Astraptes anaphus annettaDHJ03|[5987]|02-SRNP-28709|669[0n]bp
Astraptes anaphus annettaDHJ03|[5988]|02-SRNP-28395|669[0n]bp
Astraptes anaphus annettaDHJ03|[5989]|97-SRNP-2276|669[0n]bp
Astraptes anaphus annettaDHJ03|[5990]|03-SRNP-34428|669[0n]bp
Astraptes anaphus annettaDHJ03|[5991]|03-SRNP-7277|669[0n]bp
Astraptes anaphus annettaDHJ03|[5992]|06-SRNP-41210|669[0n]bp
Astraptes anaphus annettaDHJ03|[5993]|06-SRNP-41208|669[0n]bp
Astraptes anaphus annettaDHJ03|[5994]|04-SRNP-13408|669[0n]bp
Astraptes anaphus annettaDHJ03|[5995]|93-SRNP-4846|615[0n]bp
Astraptes anaphus annettaDHJ03|[5996]|93-SRNP-7913|612[0n]bp

Astraptes anaphus annettaDHJ03|[5994]|04-SRNP-13408|669[0n]bp
Astraptes anaphus annettaDHJ03|[5995]|93-SRNP-4846|615[0n]bp
Astraptes anaphus annettaDHJ03|[5996]|93-SRNP-7913|612[0n]bp
Astraptes anaphus annettaDHJ03|[5997]|07-SRNP-20180|669[0n]bp
Astraptes anaphus annettaDHJ03|[5998]|07-SRNP-42788|654[0n]bp
Astraptes anaphus annettaDHJ02|[5999]|92-SRNP-4965|645[1n]bp
Astraptes anaphus annettaDHJ02|[6000]|97-SRNP-421|657[0n]bp
Astraptes anaphus annettaDHJ02|[6001]|00-SRNP-19297|657[0n]bp
Astraptes anaphus annettaDHJ02|[6002]|97-SRNP-480|660[0n]bp
Astraptes anaphus annettaDHJ02|[6003]|92-SRNP-4960|621[0n]bp
Astraptes anaphus annettaDHJ02|[6004]|06-SRNP-30279|603[0n]bp
Astraptes anaphus annettaDHJ02|[6005]|97-SRNP-3128|669[0n]bp
Astraptes anaphus annettaDHJ02|[6006]|97-SRNP-3009|615[0n]bp
Astraptes anaphus annettaDHJ02|[6007]|93-SRNP-8376|666[0n]bp
Astraptes anaphus annettaDHJ02|[6008]|06-SRNP-41207|666[0n]bp
Astraptes anaphus annettaDHJ02|[6009]|02-SRNP-28509|669[0n]bp
Astraptes anaphus annettaDHJ02|[6010]|93-SRNP-6180|669[0n]bp
Astraptes anaphus annettaDHJ02|[6011]|97-SRNP-2560|669[0n]bp
Astraptes anaphus annettaDHJ02|[6012]|96-SRNP-4565|669[0n]bp
Astraptes anaphus annettaDHJ02|[6013]|93-SRNP-6733|669[0n]bp
Astraptes anaphus annettaDHJ02|[6014]|02-SRNP-32066|669[0n]bp
Astraptes anaphus annettaDHJ02|[6015]|97-SRNP-2566|669[0n]bp
Astraptes anaphus annettaDHJ02|[6016]|02-SRNP-28069|669[0n]bp
Astraptes anaphus annettaDHJ02|[6017]|02-SRNP-2245|669[0n]bp
Astraptes anaphus annettaDHJ02|[6018]|06-SRNP-41209|669[0n]bp
Astraptes anaphus annettaDHJ02|[6019]|06-SRNP-41039|669[0n]bp
Astraptes anaphus annettaDHJ02|[6020]|04-SRNP-61482|669[0n]bp
Astraptes anaphus annettaDHJ02|[6021]|94-SRNP-6194|609[0n]bp
Astraptes anaphus annettaDHJ02|[6022]|96-SRNP-4564|609[0n]bp
Astraptes anaphus annettaDHJ02|[6023]|93-SRNP-6178|609[0n]bp
Astraptes anaphus annettaDHJ02|[6024]|94-SRNP-7265|609[0n]bp
Astraptes anaphus annettaDHJ02|[6025]|93-SRNP-4708|609[0n]bp
Astraptes anaphus annettaDHJ02|[6026]|93-SRNP-8379|609[0n]bp
Astraptes anaphus annettaDHJ02|[6027]|93-SRNP-6179|609[0n]bp
Astraptes anaphus annettaDHJ02|[6028]|92-SRNP-4963|609[0n]bp
Astraptes anaphus annettaDHJ02|[6029]|02-SRNP-5753|669[0n]bp
Astraptes anaphus annettaDHJ02|[6030]|93-SRNP-7917|597[0n]bp
Astraptes anaphus annettaDHJ02|[6031]|93-SRNP-5032|615[0n]bp
Astraptes anaphus annettaDHJ02|[6032]|94-SRNP-6195|621[0n]bp
Astraptes anaphus annettaDHJ02|[6033]|97-SRNP-2556|612[0n]bp
Astraptes anaphus annettaDHJ02|[6034]|93-SRNP-8378|612[0n]bp
Astraptes anaphus annettaDHJ02|[6035]|97-SRNP-2559|618[0n]bp
Astraptes anaphus annettaDHJ02|[6036]|97-SRNP-418|618[0n]bp
Astraptes anaphus annettaDHJ02|[6037]|06-SRNP-21977|669[0n]bp
Astraptes anaphus annettaDHJ02|[6038]|07-SRNP-20150|669[0n]bp
Astraptes anaphus annettaDHJ02|[6039]|07-SRNP-20439|669[0n]bp
Astraptes anaphus annettaDHJ02|[6040]|07-SRNP-20089|669[0n]bp
Astraptes anaphus annettaDHJ02|[6041]|02-SRNP-28508|669[0n]bp
Astraptes anaphus annettaDHJ02|[6042]|97-SRNP-477|669[0n]bp
Astraptes anaphus annettaDHJ02|[6043]|97-SRNP-420|669[0n]bp
Astraptes anaphus annettaDHJ02|[6044]|00-SRNP-20266|669[0n]bp
Astraptes anaphus annettaDHJ02|[6045]|09-SRNP-44302|669[0n]bp
Astraptes anaphus annettaDHJ02|[6046]|09-SRNP-20617|669[0n]bp
Astraptes hopfferiDHJ02|[6047]|00-SRNP-6038|591[0n]bp
Astraptes hopfferiDHJ02|[6048]|05-SRNP-60351|633[0n]bp
Astraptes hopfferiDHJ02|[6049]|05-SRNP-60641|633[0n]bp
Astraptes hopfferiDHJ02|[6050]|05-SRNP-24819|630[0n]bp
Astraptes hopfferiDHJ02|[6051]|05-SRNP-24755|630[0n]bp
Astraptes hopfferiDHJ02|[6052]|05-SRNP-24197|630[0n]bp
Astraptes hopfferiDHJ02|[6053]|05-SRNP-19978|633[0n]bp
Astraptes hopfferiDHJ02|[6054]|05-SRNP-66135|630[0n]bp
Astraptes hopfferiDHJ02|[6055]|05-SRNP-24396|630[0n]bp
Astraptes hopfferiDHJ02|[6056]|05-SRNP-60568|630[0n]bp
Astraptes hopfferiDHJ02|[6057]|05-SRNP-61464|633[0n]bp
Astraptes hopfferiDHJ02|[6058]|05-SRNP-24340|567[0n]bp
Astraptes hopfferiDHJ02|[6059]|07-SRNP-57872|669[0n]bp
Astraptes hopfferiDHJ02|[6060]|00-SRNP-6035|624[0n]bp
Astraptes hopfferiDHJ02|[6061]|06-SRNP-58062|657[0n]bp
Astraptes hopfferiDHJ02|[6062]|07-SRNP-21123|669[0n]bp
Astraptes hopfferiDHJ02|[6063]|07-SRNP-20481|669[0n]bp
Astraptes hopfferiDHJ02|[6064]|07-SRNP-20482|669[0n]bp
Astraptes hopfferiDHJ02|[6065]|02-SRNP-5424|669[0n]bp
Astraptes hopfferiDHJ02|[6066]|99-SRNP-2552|669[0n]bp
Astraptes hopfferiDHJ02|[6067]|98-SRNP-4521|669[0n]bp
Astraptes hopfferiDHJ02|[6068]|01-SRNP-16888|669[0n]bp
Astraptes hopfferiDHJ02|[6069]|01-SRNP-16907|669[0n]bp
Astraptes hopfferiDHJ02|[6070]|95-SRNP-10163|669[0n]bp
Astraptes hopfferiDHJ02|[6071]|05-SRNP-19981|669[0n]bp
Astraptes hopfferiDHJ02|[6072]|05-SRNP-19980|669[0n]bp
Astraptes hopfferiDHJ02|[6073]|04-SRNP-15947|669[0n]bp
Astraptes hopfferiDHJ02|[6074]|05-SRNP-21448|633[0n]bp
Astraptes hopfferiDHJ02|[6075]|96-SRNP-9303|462[1n]bp
Astraptes hopfferiDHJ02|[6076]|05-SRNP-24589|633[0n]bp
Astraptes hopfferiDHJ02|[6077]|05-SRNP-24568|633[0n]bp
Astraptes hopfferiDHJ02|[6078]|98-SRNP-4411|618[0n]bp
Astraptes hopfferiDHJ02|[6079]|07-SRNP-55079|609[0n]bp
Astraptes hopfferiDHJ02|[6080]|07-SRNP-20899|627[0n]bp
Astraptes hopfferiDHJ02|[6081]|07-SRNP-24342|669[1n]bp
Astraptes hopfferiDHJ02|[6082]|07-SRNP-15266|669[0n]bp
Astraptes hopfferiDHJ02|[6083]|07-SRNP-15267|669[0n]bp
Astraptes hopfferiDHJ02|[6084]|07-SRNP-15268|669[0n]bp
Astraptes hopfferiDHJ02|[6085]|08-SRNP-16500|669[0n]bp
Astraptes hopfferiDHJ02|[6086]|08-SRNP-16501|669[0n]bp
Astraptes hopfferiDHJ02|[6087]|08-SRNP-16509|669[0n]bp
Astraptes hopfferiDHJ02|[6088]|08-SRNP-65151|669[0n]bp
Astraptes hopfferiDHJ02|[6089]|07-SRNP-24343|669[0n]bp
Astraptes hopfferiDHJ02|[6090]|08-SRNP-16506|669[0n]bp
Astraptes hopfferiDHJ02|[6091]|08-SRNP-14782|669[0n]bp
Astraptes hopfferiDHJ02|[6092]|08-SRNP-16510|669[0n]bp
Astraptes hopfferiDHJ02|[6093]|08-SRNP-16504|669[0n]bp
Astraptes hopfferiDHJ02|[6094]|00-SRNP-18470|669[0n]bp
Astraptes hopfferiDHJ02|[6095]|03-SRNP-15756|669[0n]bp
Astraptes hopfferiDHJ02|[6096]|02-SRNP-13859|669[0n]bp

Astraptes hopfferiDHJ02|[6094]|00-SRNP-18470|669[0n]bp
Astraptes hopfferiDHJ02|[6095]|03-SRNP-15756|669[0n]bp
Astraptes hopfferiDHJ02|[6096]|02-SRNP-13859|669[0n]bp
Astraptes hopfferiDHJ02|[6097]|01-SRNP-16887|669[0n]bp
Astraptes hopfferiDHJ02|[6098]|02-SRNP-13861|669[0n]bp
Astraptes hopfferiDHJ02|[6099]|02-SRNP-33228|669[0n]bp
Astraptes hopfferiDHJ02|[6100]|02-SRNP-33292|669[0n]bp
Astraptes hopfferiDHJ02|[6101]|01-SRNP-16885|669[0n]bp
Astraptes hopfferiDHJ02|[6102]|07-SRNP-55080|669[0n]bp
Astraptes hopfferiDHJ02|[6103]|07-SRNP-57649|669[0n]bp
Astraptes hopfferiDHJ02|[6104]|07-SRNP-57556|669[0n]bp
Astraptes hopfferiDHJ02|[6105]|08-SRNP-13809|669[0n]bp
Astraptes hopfferiDHJ02|[6106]|08-SRNP-13808|669[0n]bp
Astraptes hopfferiDHJ02|[6107]|08-SRNP-16352|669[0n]bp
Astraptes hopfferiDHJ02|[6108]|08-SRNP-16353|669[0n]bp
Astraptes hopfferiDHJ01|[6109]|05-SRNP-60355|633[0n]bp
Astraptes hopfferiDHJ01|[6110]|05-SRNP-24692|630[0n]bp
Astraptes hopfferiDHJ01|[6111]|05-SRNP-24395|630[0n]bp
Astraptes hopfferiDHJ01|[6112]|05-SRNP-21444|669[0n]bp
Astraptes hopfferiDHJ01|[6113]|98-SRNP-4412|669[0n]bp
Astraptes hopfferiDHJ01|[6114]|98-SRNP-4402|645[0n]bp
Astraptes hopfferiDHJ01|[6115]|98-SRNP-4409|669[0n]bp
Astraptes hopfferiDHJ01|[6116]|98-SRNP-4399|669[0n]bp
Astraptes hopfferiDHJ01|[6117]|98-SRNP-4408|669[0n]bp
Astraptes hopfferiDHJ01|[6118]|08-SRNP-24553|669[0n]bp
Astraptes chiriquensis|[6119]|05-SRNP-32306|669[0n]bp
Astraptes chiriquensis|[6120]|04-SRNP-33647|669[0n]bp
Astraptes chiriquensis|[6121]|04-SRNP-2338|669[0n]bp
Astraptes chiriquensis|[6122]|06-SRNP-31610|633[1n]bp
Astraptes chiriquensis|[6123]|07-SRNP-41188|669[0n]bp
Astraptes chiriquensis|[6124]|07-SRNP-40863|669[0n]bp
Astraptes chiriquensis|[6125]|07-SRNP-23622|663[0n]bp
Astraptes alardus|[6126]|05-SRNP-66269|669[3n]bp
Astraptes alardus|[6127]|05-SRNP-57547|651[0n]bp
Astraptes alardus|[6128]|07-SRNP-56409|669[0n]bp
Astraptes alardus|[6129]|07-SRNP-56408|669[0n]bp
Astraptes alardus|[6130]|07-SRNP-35878|669[0n]bp
Astraptes alardus|[6131]|07-SRNP-56961|669[0n]bp
Astraptes alardus|[6132]|07-SRNP-56397|669[0n]bp
Astraptes alardus|[6133]|06-SRNP-58880|669[0n]bp
Astraptes alardus|[6134]|04-SRNP-60018|669[0n]bp
Astraptes alardus|[6135]|04-SRNP-35460|669[0n]bp
Astraptes alardus|[6136]|04-SRNP-45519|669[0n]bp
Astraptes alardus|[6137]|04-SRNP-35461|669[0n]bp
Astraptes alardus|[6138]|04-SRNP-24624|669[0n]bp
Astraptes alardus|[6139]|07-SRNP-35859|645[0n]bp
Astraptes alardus|[6140]|07-SRNP-56299|645[0n]bp
Astraptes alardus|[6141]|05-SRNP-57546|669[4n]bp
Astraptes alardus|[6142]|05-SRNP-66293|669[5n]bp
Astraptes alardus|[6143]|04-SRNP-34471|627[0n]bp
Astraptes alardus|[6144]|03-SRNP-3693|624[0n]bp
Astraptes alardus|[6145]|07-SRNP-56473|648[0n]bp
Astraptes alardus|[6146]|06-SRNP-35909|612[0n]bp
Astraptes alardus|[6147]|06-SRNP-35908|609[0n]bp
Astraptes alardus|[6148]|03-SRNP-3691|540[0n]bp
Astraptes alardus|[6149]|05-SRNP-20286|573[0n]bp
Astraptes alardus|[6150]|07-SRNP-35953|633[0n]bp
Astraptes alardus|[6151]|07-SRNP-56765|669[0n]bp
Astraptes alardus|[6152]|07-SRNP-65143|582[0n]bp
Astraptes alardus|[6153]|07-SRNP-35914|621[0n]bp
Astraptes alardus|[6154]|08-SRNP-45019|669[0n]bp
Astraptes alardus|[6155]|08-SRNP-36614|669[0n]bp
Astraptes creteus cranaDHJ01|[6156]|02-SRNP-23100|669[6n]bp
Astraptes creteus cranaDHJ01|[6157]|97-SRNP-1464|588[0n]bp
Astraptes creteus cranaDHJ01|[6158]|02-SRNP-23417|585[0n]bp
Astraptes creteus cranaDHJ01|[6159]|99-SRNP-13962|669[0n]bp
Astraptes creteus cranaDHJ01|[6160]|02-SRNP-23933|669[0n]bp
Astraptes creteus cranaDHJ01|[6161]|99-SRNP-13961|669[0n]bp
Astraptes creteus cranaDHJ01|[6162]|03-SRNP-4189|669[0n]bp
Astraptes creteus cranaDHJ01|[6163]|03-SRNP-4188|669[0n]bp
Astraptes creteus cranaDHJ01|[6164]|03-SRNP-4333|669[0n]bp
Astraptes creteus cranaDHJ01|[6165]|05-SRNP-35360|669[0n]bp
Astraptes creteus cranaDHJ01|[6166]|97-SRNP-1467|609[0n]bp
Astraptes creteus cranaDHJ01|[6167]|97-SRNP-1465|384[0n]bp
Astraptes creteus cranaDHJ01|[6168]|98-SRNP-3078|669[0n]bp
Astraptes creteus cranaDHJ01|[6169]|97-SRNP-1253|669[0n]bp
Astraptes creteus cranaDHJ01|[6170]|99-SRNP-13963|669[0n]bp
Astraptes creteus cranaDHJ02|[6171]|98-SRNP-2979|642[0n]bp
Astraptes creteus cranaDHJ02|[6172]|02-SRNP-23727|630[0n]bp
Astraptes creteus cranaDHJ02|[6173]|02-SRNP-24378|630[0n]bp
Astraptes creteus cranaDHJ02|[6174]|02-SRNP-9497|669[0n]bp
Astraptes creteus cranaDHJ02|[6175]|98-SRNP-3853|633[0n]bp
Astraptes creteus cranaDHJ02|[6176]|00-SRNP-9497|504[0n]bp
Astraptes creteus cranaDHJ02|[6177]|00-SRNP-9363|669[0n]bp
Astraptes creteus cranaDHJ02|[6178]|03-SRNP-4452|669[0n]bp
Astraptes creteus cranaDHJ02|[6179]|03-SRNP-23876|669[0n]bp
Astraptes creteus cranaDHJ02|[6180]|03-SRNP-13379|669[0n]bp
Astraptes creteus cranaDHJ02|[6181]|03-SRNP-23795|669[0n]bp
Astraptes creteus cranaDHJ02|[6182]|03-SRNP-3042|669[0n]bp
Astraptes creteus cranaDHJ02|[6183]|03-SRNP-23880|669[0n]bp
Astraptes creteus cranaDHJ02|[6184]|05-SRNP-35359|669[0n]bp
Astraptes creteus cranaDHJ02|[6185]|05-SRNP-35230|669[0n]bp
Astraptes creteus cranaDHJ02|[6186]|05-SRNP-35582|669[0n]bp
Astraptes creteus cranaDHJ02|[6187]|05-SRNP-35228|669[0n]bp
Astraptes creteus cranaDHJ02|[6188]|05-SRNP-35253|669[0n]bp
Astraptes creteus cranaDHJ02|[6189]|04-SRNP-35324|669[0n]bp
Astraptes creteus cranaDHJ02|[6190]|04-SRNP-35590|669[0n]bp
Astraptes creteus cranaDHJ02|[6191]|04-SRNP-35599|669[0n]bp
Astraptes creteus cranaDHJ02|[6192]|00-SRNP-9654|585[0n]bp
Astraptes creteus cranaDHJ02|[6193]|03-SRNP-4174|669[1n]bp
Astraptes creteus cranaDHJ02|[6194]|00-SRNP-9951|669[0n]bp
Astraptes creteus cranaDHJ02|[6195]|00-SRNP-9783|669[0n]bp
Astraptes creteus cranaDHJ02|[6196]|07-SRNP-57106|645[0n]bp

Astraptes creteus cranaDHJ02|[6194]|00-SRNP-9951|669[0n]bp
Astraptes creteus cranaDHJ02|[6195]|00-SRNP-9783|669[0n]bp
Astraptes creteus cranaDHJ02|[6196]|07-SRNP-57106|645[0n]bp
Astraptes creteus cranaDHJ02|[6197]|02-SRNP-8259|645[0n]bp
Astraptes creteus cranaDHJ02|[6198]|98-SRNP-2019|645[0n]bp
Astraptes creteus cranaDHJ02|[6199]|02-SRNP-9883|585[0n]bp
Astraptes creteus cranaDHJ02|[6200]|97-SRNP-1362|630[0n]bp
Astraptes creteus cranaDHJ02|[6201]|99-SRNP-291|642[1n]bp
Astraptes creteus cranaDHJ02|[6202]|00-SRNP-10716|624[0n]bp
Astraptes creteus cranaDHJ02|[6203]|02-SRNP-8156|585[0n]bp
Astraptes creteus cranaDHJ02|[6204]|02-SRNP-23538|585[0n]bp
Astraptes creteus cranaDHJ02|[6205]|97-SRNP-1617|615[0n]bp
Astraptes creteus cranaDHJ02|[6206]|98-SRNP-11988|609[0n]bp
Astraptes creteus cranaDHJ02|[6207]|98-SRNP-2628|609[0n]bp
Astraptes creteus cranaDHJ02|[6208]|98-SRNP-2871|609[0n]bp
Astraptes creteus cranaDHJ02|[6209]|98-SRNP-2709|609[0n]bp
Astraptes creteus cranaDHJ02|[6210]|00-SRNP-9398|609[0n]bp
Astraptes creteus cranaDHJ02|[6211]|01-SRNP-6265|606[0n]bp
Astraptes creteus cranaDHJ02|[6212]|01-SRNP-6809|606[0n]bp
Astraptes creteus cranaDHJ02|[6213]|95-SRNP-537|363[2n]bp
Astraptes creteus cranaDHJ02|[6214]|94-SRNP-5247|609[0n]bp
Astraptes creteus cranaDHJ02|[6215]|98-SRNP-2873|588[0n]bp
Astraptes creteus cranaDHJ02|[6216]|07-SRNP-57105|639[0n]bp
Astraptes creteus cranaDHJ02|[6217]|02-SRNP-24377|669[0n]bp
Astraptes creteus cranaDHJ02|[6218]|01-SRNP-6805|669[0n]bp
Astraptes creteus cranaDHJ02|[6219]|01-SRNP-7968|669[0n]bp
Astraptes creteus cranaDHJ02|[6220]|02-SRNP-8216|669[0n]bp
Astraptes creteus cranaDHJ02|[6221]|01-SRNP-7967|669[0n]bp
Astraptes creteus cranaDHJ02|[6222]|02-SRNP-23585|669[0n]bp
Astraptes creteus cranaDHJ02|[6223]|02-SRNP-24509|669[0n]bp
Astraptes creteus cranaDHJ02|[6224]|02-SRNP-23630|669[0n]bp
Astraptes creteus cranaDHJ02|[6225]|01-SRNP-6182|669[0n]bp
Astraptes creteus cranaDHJ02|[6226]|01-SRNP-6806|669[0n]bp
Astraptes creteus cranaDHJ02|[6227]|02-SRNP-23118|669[0n]bp
Astraptes creteus cranaDHJ02|[6228]|02-SRNP-23539|669[0n]bp
Astraptes creteus cranaDHJ02|[6229]|98-SRNP-2980|669[0n]bp
Astraptes creteus cranaDHJ02|[6230]|97-SRNP-1252|669[0n]bp
Astraptes creteus cranaDHJ02|[6231]|97-SRNP-11002|669[0n]bp
Astraptes creteus cranaDHJ02|[6232]|02-SRNP-8258|669[0n]bp
Astraptes creteus cranaDHJ02|[6233]|00-SRNP-9624|669[0n]bp
Astraptes creteus cranaDHJ02|[6234]|98-SRNP-2805|669[0n]bp
Astraptes creteus cranaDHJ02|[6235]|00-SRNP-9502|669[0n]bp
Astraptes creteus cranaDHJ02|[6236]|98-SRNP-2055|669[0n]bp
Astraptes creteus cranaDHJ02|[6237]|98-SRNP-2806|669[0n]bp
Astraptes creteus cranaDHJ02|[6238]|98-SRNP-3912|669[0n]bp
Astraptes creteus cranaDHJ02|[6239]|97-SRNP-1835|669[0n]bp
Astraptes creteus cranaDHJ02|[6240]|06-SRNP-35585|669[0n]bp
Astraptes creteus cranaDHJ02|[6241]|06-SRNP-46102|669[0n]bp
Astraptes creteus cranaDHJ02|[6242]|07-SRNP-36022|669[0n]bp
Astraptes creteus cranaDHJ02|[6243]|07-SRNP-57188|669[0n]bp
Astraptes creteus cranaDHJ02|[6244]|07-SRNP-36021|669[0n]bp
Astraptes creteus cranaDHJ02|[6245]|07-SRNP-36883|669[0n]bp
Astraptes creteus cranaDHJ02|[6246]|08-SRNP-35767|669[0n]bp
Astraptes creteus cranaDHJ02|[6247]|08-SRNP-35790|669[0n]bp
Astraptes creteus cranaDHJ02|[6248]|08-SRNP-35544|669[0n]bp
Astraptes creteus cranaDHJ02|[6249]|09-SRNP-56363|669[0n]bp
Astraptes creteus cranaDHJ02|[6250]|09-SRNP-56364|669[0n]bp
Astraptes creteus cranaDHJ02|[6251]|02-SRNP-9064|669[1n]bp
Astraptes creteus cranaDHJ02|[6252]|04-SRNP-36066|669[0n]bp
Astraptes creteus cranaDHJ02|[6253]|09-SRNP-36873|669[0n]bp
Astraptes talus|[6254]|03-SRNP-1776|540[1n]bp
Astraptes talus|[6255]|02-SRNP-28715|570[0n]bp
Astraptes talus|[6256]|05-SRNP-22282|657[0n]bp
Astraptes talus|[6257]|07-SRNP-42996|669[0n]bp
Astraptes talus|[6258]|06-SRNP-21979|669[0n]bp
Astraptes talus|[6259]|06-SRNP-56333|669[0n]bp
Astraptes talus|[6260]|05-SRNP-22277|669[0n]bp
Astraptes talus|[6261]|05-SRNP-22036|669[0n]bp
Astraptes talus|[6262]|05-SRNP-22279|669[0n]bp
Astraptes talus|[6263]|05-SRNP-22031|669[0n]bp
Astraptes talus|[6264]|05-SRNP-22257|669[0n]bp
Astraptes talus|[6265]|05-SRNP-22033|669[0n]bp
Astraptes talus|[6266]|07-SRNP-1322|663[0n]bp
Astraptes talus|[6267]|05-SRNP-25199|453[1n]bp
Astraptes talus|[6268]|05-SRNP-25202|642[0n]bp
Astraptes talus|[6269]|07-SRNP-43000|669[0n]bp
Astraptes talus|[6270]|08-SRNP-40181|669[0n]bp
Astraptes talus|[6271]|09-SRNP-41979|669[0n]bp
Astraptes talus|[6272]|09-SRNP-41975|669[0n]bp
Astraptes talus|[6273]|09-SRNP-41973|669[0n]bp
Astraptes talus|[6274]|09-SRNP-41980|669[0n]bp
Astraptes talus|[6275]|09-SRNP-41977|669[0n]bp
Astraptes talus|[6276]|09-SRNP-41974|669[0n]bp
Astraptes talus|[6277]|09-SRNP-41983|669[0n]bp
Astraptes talus|[6278]|09-SRNP-41978|669[0n]bp
Astraptes talus|[6279]|09-SRNP-41982|669[0n]bp
Autochton longipennis|[6280]|01-SRNP-2960|390[0n]bp
Achalarus albociliatus|[6281]|05-SRNP-56408|636[0n]bp
Achalarus albociliatus|[6282]|96-SRNP-1083|570[0n]bp
Achalarus albociliatus|[6283]|00-SRNP-6810|582[3n]bp
Achalarus albociliatus|[6284]|02-SRNP-14451|669[0n]bp
Achalarus albociliatus|[6285]|02-SRNP-14113|669[0n]bp
Achalarus albociliatus|[6286]|02-SRNP-14234|669[0n]bp
Achalarus albociliatus|[6287]|00-SRNP-6827|669[0n]bp
Achalarus albociliatus|[6288]|02-SRNP-4786|669[1n]bp
Achalarus albociliatus|[6289]|07-SRNP-12984|669[0n]bp
Achalarus toxeus|[6290]|96-SRNP-8690|576[0n]bp
Achalarus toxeus|[6291]|93-SRNP-7539|396[0n]bp
Achalarus toxeus|[6292]|02-SRNP-10179|669[0n]bp
Achalarus toxeus|[6293]|02-SRNP-10180|669[0n]bp
Achalarus toxeus|[6294]|03-SRNP-237|669[0n]bp
Thessia jalapus|[6295]|08-SRNP-22900|669[0n]bp
Thessia jalapus|[6296]|97-SRNP-4702|639[0n]bp

Achalarus toxeus|[6294]|03-SRNP-237|669[0n]bp
Thessia jalapus|[6295]|08-SRNP-22900|669[0n]bp
Thessia jalapus|[6296]|97-SRNP-4702|639[0n]bp
Thessia jalapus|[6297]|08-SRNP-22673|669[0n]bp
Thessia jalapus|[6298]|08-SRNP-30977|669[0n]bp
Thessia jalapus|[6299]|08-SRNP-30974|669[0n]bp
Thessia jalapus|[6300]|02-SRNP-13559|669[0n]bp
Thessia jalapus|[6301]|08-SRNP-30979|669[0n]bp
Thessia jalapus|[6302]|09-SRNP-30937|408[0n]bp
Thessia jalapus|[6303]|09-SRNP-20372|669[0n]bp
Thessia jalapus|[6304]|08-SRNP-23350|669[0n]bp
Thessia jalapus|[6305]|09-SRNP-20138|669[0n]bp
Thessia jalapus|[6306]|08-SRNP-24707|669[0n]bp
Thessia jalapus|[6307]|04-SRNP-22865|669[0n]bp
Thessia jalapus|[6308]|08-SRNP-55016|669[0n]bp
Thessia jalapus|[6309]|08-SRNP-30973|669[0n]bp
Thessia jalapus|[6310]|07-SRNP-55386|669[0n]bp
Thessia jalapus|[6311]|02-SRNP-13729|669[0n]bp
Thessia jalapus|[6312]|02-SRNP-13560|669[0n]bp
Thessia jalapus|[6313]|02-SRNP-4830|669[0n]bp
Thessia jalapus|[6314]|02-SRNP-14049|669[0n]bp
Thessia jalapus|[6315]|04-SRNP-14601|669[0n]bp
Thessia jalapus|[6316]|07-SRNP-21140|663[0n]bp
Thessia jalapus|[6317]|97-SRNP-4570|573[0n]bp
Thessia jalapus|[6318]|08-SRNP-21898|669[0n]bp
Thessia jalapus|[6319]|09-SRNP-20140|669[0n]bp
Astraptes phalaecus|[6320]|00-SRNP-17853|630[0n]bp
Astraptes phalaecus|[6321]|09-SRNP-33034|669[0n]bp
Astraptes phalaecus|[6322]|09-SRNP-33035|669[0n]bp
Urbanus albimargo|[6323]|99-SRNP-6189|642[0n]bp
Urbanus albimargo|[6324]|05-SRNP-55418|687[0n]bp
Urbanus albimargo|[6325]|97-SRNP-4266|396[0n]bp
Urbanus albimargo|[6326]|06-SRNP-2798|633[0n]bp
Urbanus albimargo|[6327]|08-SRNP-4797|669[0n]bp
Urbanus albimargo|[6328]|08-SRNP-4799|669[0n]bp
Urbanus albimargo|[6329]|08-SRNP-4798|669[0n]bp
Urbanus doryssusDHJ01|[6330]|09-SRNP-65298|669[0n]bp
Urbanus doryssusDHJ01|[6331]|08-SRNP-66077|669[0n]bp
Urbanus doryssusDHJ01|[6332]|08-SRNP-65793|669[0n]bp
Urbanus doryssusDHJ01|[6333]|08-SRNP-32448|669[0n]bp
Urbanus doryssusDHJ01|[6334]|08-SRNP-65792|669[0n]bp
Urbanus doryssusDHJ01|[6335]|08-SRNP-30716|669[0n]bp
Urbanus doryssusDHJ01|[6336]|08-SRNP-65388|669[0n]bp
Urbanus doryssusDHJ01|[6337]|08-SRNP-41168|669[0n]bp
Urbanus doryssusDHJ01|[6338]|08-SRNP-30697|669[0n]bp
Urbanus doryssusDHJ01|[6339]|08-SRNP-65154|669[0n]bp
Urbanus doryssusDHJ01|[6340]|07-SRNP-33176|669[0n]bp
Urbanus doryssusDHJ01|[6341]|07-SRNP-65317|669[0n]bp
Urbanus doryssusDHJ01|[6342]|07-SRNP-32796|669[0n]bp
Urbanus doryssusDHJ01|[6343]|07-SRNP-65529|669[0n]bp
Urbanus doryssusDHJ01|[6344]|07-SRNP-41431|669[0n]bp
Urbanus doryssusDHJ01|[6345]|07-SRNP-32630|669[0n]bp
Urbanus doryssusDHJ01|[6346]|07-SRNP-32039|669[0n]bp
Urbanus doryssusDHJ01|[6347]|07-SRNP-30997|669[0n]bp
Urbanus doryssusDHJ01|[6348]|07-SRNP-21334|669[0n]bp
Urbanus doryssusDHJ01|[6349]|02-SRNP-6305|669[0n]bp
Urbanus doryssusDHJ01|[6350]|06-SRNP-31223|669[0n]bp
Urbanus doryssusDHJ01|[6351]|07-SRNP-32446|642[0n]bp
Urbanus doryssusDHJ01|[6352]|04-SRNP-42344|618[0n]bp
Urbanus doryssusDHJ01|[6353]|07-SRNP-41459|660[0n]bp
Urbanus doryssusDHJ01|[6354]|05-SRNP-70279|396[0n]bp
Urbanus doryssusDHJ01|[6355]|09-SRNP-30058|645[0n]bp
Urbanus doryssusDHJ01|[6356]|09-SRNP-33053|639[0n]bp
Urbanus doryssusDHJ01|[6357]|09-SRNP-31697|669[0n]bp
Urbanus doryssusDHJ01|[6358]|09-SRNP-31399|669[0n]bp
Urbanus doryssusDHJ01|[6359]|09-SRNP-80602|669[0n]bp
Urbanus doryssusDHJ01|[6360]|09-SRNP-32813|630[0n]bp
Urbanus doryssusDHJ03|[6361]|04-SRNP-46471|669[0n]bp
Urbanus doryssusDHJ03|[6362]|04-SRNP-46473|669[0n]bp
Urbanus doryssusDHJ02|[6363]|05-SRNP-2102|669[0n]bp
Urbanus doryssusDHJ02|[6364]|05-SRNP-65996|669[0n]bp
Urbanus doryssusDHJ02|[6365]|06-SRNP-1298|669[2n]bp
Urbanus doryssusDHJ02|[6366]|07-SRNP-59558|669[0n]bp
Urbanus doryssusDHJ02|[6367]|04-SRNP-46474|627[0n]bp
Urbanus doryssusDHJ02|[6368]|06-SRNP-3568|669[0n]bp
Urbanus doryssusDHJ02|[6369]|09-SRNP-44478|669[0n]bp
Urbanus doryssusDHJ02|[6370]|09-SRNP-44475|669[0n]bp
Urbanus doryssusDHJ02|[6371]|96-SRNP-11042|618[0n]bp
Urbanus doryssusDHJ02|[6372]|05-SRNP-2322|669[0n]bp
Urbanus doryssusDHJ02|[6373]|09-SRNP-65637|636[0n]bp
Urbanus doryssusDHJ02|[6374]|05-SRNP-20746|669[0n]bp
Urbanus doryssusDHJ02|[6375]|08-SRNP-1784|669[0n]bp
Urbanus doryssusDHJ02|[6376]|08-SRNP-1779|669[0n]bp
Urbanus doryssusDHJ02|[6377]|09-SRNP-20147|669[0n]bp
Urbanus doryssusDHJ02|[6378]|09-SRNP-65199|669[0n]bp
Urbanus doryssusDHJ02|[6379]|08-SRNP-1324|669[0n]bp
Urbanus doryssusDHJ02|[6380]|08-SRNP-1771|669[0n]bp
Urbanus doryssusDHJ02|[6381]|08-SRNP-1774|669[0n]bp
Urbanus doryssusDHJ02|[6382]|08-SRNP-2350|669[0n]bp
Urbanus doryssusDHJ02|[6383]|07-SRNP-1310|669[0n]bp
Urbanus doryssusDHJ02|[6384]|07-SRNP-1447|669[0n]bp
Urbanus doryssusDHJ02|[6385]|06-SRNP-3275|669[0n]bp
Urbanus doryssusDHJ02|[6386]|06-SRNP-3569|669[0n]bp
Urbanus doryssusDHJ02|[6387]|06-SRNP-20390|669[0n]bp
Urbanus doryssusDHJ02|[6388]|05-SRNP-2104|669[0n]bp
Urbanus doryssusDHJ02|[6389]|05-SRNP-2309|669[0n]bp
Urbanus doryssusDHJ02|[6390]|05-SRNP-2103|669[0n]bp
Urbanus doryssusDHJ02|[6391]|05-SRNP-2105|669[0n]bp
Urbanus doryssusDHJ02|[6392]|05-SRNP-46461|669[0n]bp
Urbanus doryssusDHJ02|[6393]|05-SRNP-2108|669[0n]bp
Urbanus doryssusDHJ02|[6394]|05-SRNP-2339|669[0n]bp
Urbanus doryssusDHJ02|[6395]|05-SRNP-46459|669[0n]bp
Urbanus doryssusDHJ02|[6396]|05-SRNP-2106|669[0n]bp

Urbanus doryssusDHJ02|[6394]|05-SRNP-2339|669[0n]bp
Urbanus doryssusDHJ02|[6395]|05-SRNP-46459|669[0n]bp
Urbanus doryssusDHJ02|[6396]|05-SRNP-2106|669[0n]bp
Urbanus doryssusDHJ02|[6397]|08-SRNP-2185|666[0n]bp
Urbanus doryssusDHJ02|[6398]|06-SRNP-3214|669[0n]bp
Urbanus doryssusDHJ02|[6399]|08-SRNP-1781|669[0n]bp
Urbanus doryssusDHJ02|[6400]|07-SRNP-1739|612[0n]bp
Urbanus doryssusDHJ02|[6401]|06-SRNP-1796|633[0n]bp
Urbanus doryssusDHJ02|[6402]|93-SRNP-4324|564[0n]bp
Urbanus doryssusDHJ02|[6403]|09-SRNP-65198|606[0n]bp
Urbanus doryssusDHJ02|[6404]|09-SRNP-65161|618[0n]bp
Urbanus doryssusDHJ02|[6405]|09-SRNP-68103|669[0n]bp
Urbanus doryssusDHJ02|[6406]|05-SRNP-58857|669[0n]bp
Urbanus doryssusDHJ02|[6407]|04-SRNP-22318|669[0n]bp
Urbanus doryssusDHJ02|[6408]|04-SRNP-22317|669[0n]bp
Urbanus doryssusDHJ02|[6409]|04-SRNP-48319|669[0n]bp
Urbanus doryssusDHJ02|[6410]|06-SRNP-3376|669[0n]bp
Urbanus doryssusDHJ02|[6411]|08-SRNP-2186|669[0n]bp
Urbanus doryssusDHJ02|[6412]|09-SRNP-23060|669[0n]bp
Urbanus teleus|[6413]|01-SRNP-16521|669[0n]bp
Urbanus teleus|[6414]|97-SRNP-3309|513[23n]bp
Urbanus teleus|[6415]|00-SRNP-4055|669[0n]bp
Urbanus teleus|[6416]|00-SRNP-4054|669[0n]bp
Urbanus teleus|[6417]|04-SRNP-30475|669[0n]bp
Urbanus teleus|[6418]|07-SRNP-55852|669[0n]bp
Urbanus teleus|[6419]|07-SRNP-55853|669[0n]bp
Urbanus teleus|[6420]|07-SRNP-58338|669[0n]bp
Autochton zarex|[6421]|04-SRNP-42624|669[0n]bp
Autochton zarex|[6422]|05-SRNP-57363|669[0n]bp
Autochton zarex|[6423]|05-SRNP-43733|669[0n]bp
Autochton zarex|[6424]|07-SRNP-58889|642[0n]bp
Autochton zarex|[6425]|05-SRNP-59312|669[0n]bp
Autochton zarex|[6426]|06-SRNP-23079|669[0n]bp
Autochton zarex|[6427]|03-SRNP-8036|639[3n]bp
Autochton zarex|[6428]|95-SRNP-7760|411[0n]bp
Autochton zarex|[6429]|97-SRNP-5452|618[0n]bp
Autochton zarex|[6430]|97-SRNP-4767|552[2n]bp
Autochton zarex|[6431]|06-SRNP-22476|645[0n]bp
Autochton zarex|[6432]|08-SRNP-70876|669[0n]bp
Urbanus dorantes|[6433]|05-SRNP-34161|627[0n]bp
Urbanus dorantes|[6434]|05-SRNP-6828|627[0n]bp
Urbanus dorantes|[6435]|07-SRNP-65123|636[0n]bp
Urbanus dorantes|[6436]|02-SRNP-1897|570[0n]bp
Urbanus dorantes|[6437]|02-SRNP-15449|645[0n]bp
Urbanus dorantes|[6438]|06-SRNP-1986|669[0n]bp
Urbanus dorantes|[6439]|06-SRNP-30970|669[0n]bp
Urbanus dorantes|[6440]|05-SRNP-59442|669[0n]bp
Urbanus dorantes|[6441]|04-SRNP-16100|669[0n]bp
Urbanus dorantes|[6442]|05-SRNP-42411|573[0n]bp
Urbanus dorantes|[6443]|06-SRNP-3492|666[0n]bp
Urbanus dorantes|[6444]|07-SRNP-32150|669[0n]bp
Urbanus dorantes|[6445]|07-SRNP-56431|669[0n]bp
Urbanus dorantes|[6446]|07-SRNP-45404|669[0n]bp
Urbanus dorantes|[6447]|07-SRNP-45405|669[0n]bp
Urbanus dorantes|[6448]|08-SRNP-72130|669[0n]bp
Astraptes egregiusDHJ01|[6449]|06-SRNP-41788|669[0n]bp
Astraptes egregiusDHJ01|[6450]|05-SRNP-40504|669[0n]bp
Astraptes egregiusDHJ01|[6451]|04-SRNP-56511|669[0n]bp
Astraptes egregiusDHJ01|[6452]|07-SRNP-21401|648[0n]bp
Astraptes egregiusDHJ02|[6453]|02-SRNP-15436|624[0n]bp
Astraptes egregiusDHJ02|[6454]|03-SRNP-21843|576[0n]bp
Astraptes egregiusDHJ02|[6455]|96-SRNP-11827|570[0n]bp
Astraptes egregiusDHJ02|[6456]|05-SRNP-59306|669[0n]bp
Astraptes egregiusDHJ02|[6457]|06-SRNP-40597|669[0n]bp
Astraptes egregiusDHJ02|[6458]|07-SRNP-40219|669[0n]bp
Astraptes egregiusDHJ02|[6459]|08-SRNP-36561|669[0n]bp
Urbanus simplicius|[6460]|04-SRNP-45191|666[0n]bp
Urbanus simplicius|[6461]|04-SRNP-30838|669[0n]bp
Urbanus simplicius|[6462]|02-SRNP-5635|645[0n]bp
Urbanus simplicius|[6463]|07-SRNP-20188|669[0n]bp
Urbanus simplicius|[6464]|07-SRNP-20175|669[0n]bp
Urbanus simplicius|[6465]|07-SRNP-20181|669[0n]bp
Urbanus simplicius|[6466]|07-SRNP-20078|669[0n]bp
Urbanus simplicius|[6467]|06-SRNP-20658|669[0n]bp
Urbanus simplicius|[6468]|06-SRNP-58893|669[0n]bp
Urbanus simplicius|[6469]|04-SRNP-45789|669[0n]bp
Urbanus simplicius|[6470]|02-SRNP-4745|639[0n]bp
Urbanus simplicius|[6471]|07-SRNP-20177|669[0n]bp
Urbanus simplicius|[6472]|08-SRNP-58533|669[0n]bp
Urbanus simplicius|[6473]|08-SRNP-24796|669[0n]bp
Astraptes aulus|[6474]|97-SRNP-10301|639[0n]bp
Astraptes aulus|[6475]|06-SRNP-55030|663[5n]bp
Astraptes aulus|[6476]|94-SRNP-7852|399[0n]bp
Astraptes aulus|[6477]|05-SRNP-12046|669[0n]bp
Astraptes aulus|[6478]|05-SRNP-64313|669[0n]bp
Astraptes aulus|[6479]|06-SRNP-60359|669[0n]bp
Astraptes janeiraDHJ01|[6480]|06-SRNP-6959|669[0n]bp
Astraptes janeiraDHJ01|[6481]|09-SRNP-65806|669[0n]bp
Astraptes janeiraDHJ02|[6482]|08-SRNP-65223|669[1n]bp
Astraptes janeiraDHJ02|[6483]|08-SRNP-31029|669[0n]bp
Astraptes janeiraDHJ02|[6484]|08-SRNP-40223|669[0n]bp
Astraptes janeiraDHJ02|[6485]|08-SRNP-65370|669[0n]bp
Astraptes janeiraDHJ02|[6486]|04-SRNP-1932|669[0n]bp
Astraptes janeiraDHJ02|[6487]|03-SRNP-21497|657[0n]bp
Astraptes janeiraDHJ02|[6488]|05-SRNP-43094|630[0n]bp
Astraptes janeiraDHJ02|[6489]|05-SRNP-21164|669[0n]bp
Astraptes janeiraDHJ02|[6490]|05-SRNP-21166|669[0n]bp
Astraptes janeiraDHJ02|[6491]|99-SRNP-2448|630[0n]bp
Astraptes janeiraDHJ02|[6492]|02-SRNP-5607|585[0n]bp
Astraptes janeiraDHJ02|[6493]|05-SRNP-32361|624[0n]bp
Astraptes janeiraDHJ02|[6494]|06-SRNP-2015|669[0n]bp
Astraptes janeiraDHJ02|[6495]|09-SRNP-67362|669[0n]bp
Astraptes enotrus|[6496]|03-SRNP-3278|630[2n]bp

Astraptes janeiraDHJ02|[6494]|06-SRNP-2015|669[0n]bp
Astraptes janeiraDHJ02|[6495]|09-SRNP-67362|669[0n]bp
Astraptes enotrus|[6496]|03-SRNP-3278|630[2n]bp
Astraptes enotrus|[6497]|08-SRNP-4337|321[0n]bp
Astraptes enotrus|[6498]|08-SRNP-66239|669[3n]bp
Astraptes enotrus|[6499]|08-SRNP-2132|669[3n]bp
Astraptes enotrus|[6500]|02-SRNP-29258|657[3n]bp
Astraptes enotrus|[6501]|08-SRNP-4382|642[0n]bp
Astraptes enotrus|[6502]|07-SRNP-45300|642[0n]bp
Astraptes enotrus|[6503]|07-SRNP-57189|642[0n]bp
Astraptes enotrus|[6504]|07-SRNP-2586|669[0n]bp
Astraptes enotrus|[6505]|03-SRNP-8361|663[0n]bp
Astraptes enotrus|[6506]|07-SRNP-45178|639[0n]bp
Astraptes enotrus|[6507]|03-SRNP-1513|570[0n]bp
Astraptes enotrus|[6508]|04-SRNP-32395|669[1n]bp
Astraptes enotrus|[6509]|05-SRNP-31823|669[0n]bp
Astraptes enotrus|[6510]|04-SRNP-30579|669[0n]bp
Astraptes enotrus|[6511]|04-SRNP-33020|669[0n]bp
Astraptes enotrus|[6512]|04-SRNP-30578|669[0n]bp
Astraptes enotrus|[6513]|04-SRNP-31311|669[0n]bp
Astraptes enotrus|[6514]|04-SRNP-32345|669[0n]bp
Astraptes enotrus|[6515]|04-SRNP-22616|669[0n]bp
Astraptes enotrus|[6516]|04-SRNP-1431|669[0n]bp
Astraptes enotrus|[6517]|04-SRNP-4633|669[1n]bp
Astraptes enotrus|[6518]|01-SRNP-9736|669[0n]bp
Astraptes enotrus|[6519]|06-SRNP-23089|615[1n]bp
Astraptes enotrus|[6520]|04-SRNP-47730|669[0n]bp
Astraptes enotrus|[6521]|03-SRNP-20571|669[0n]bp
Astraptes enotrus|[6522]|03-SRNP-19578|669[0n]bp
Astraptes enotrus|[6523]|08-SRNP-2124|630[3n]bp
Astraptes enotrus|[6524]|06-SRNP-36179|666[0n]bp
Astraptes enotrus|[6525]|01-SRNP-1057|669[1n]bp
Astraptes enotrus|[6526]|03-SRNP-10882|669[1n]bp
Astraptes enotrus|[6527]|04-SRNP-4407|669[1n]bp
Astraptes enotrus|[6528]|04-SRNP-4531|669[1n]bp
Astraptes enotrus|[6529]|04-SRNP-15289|669[1n]bp
Astraptes enotrus|[6530]|04-SRNP-23231|627[0n]bp
Astraptes enotrus|[6531]|03-SRNP-5824|645[0n]bp
Astraptes enotrus|[6532]|06-SRNP-40208|657[0n]bp
Astraptes enotrus|[6533]|07-SRNP-31387|660[0n]bp
Astraptes enotrus|[6534]|95-SRNP-7803|621[0n]bp
Astraptes enotrus|[6535]|99-SRNP-4986|615[0n]bp
Astraptes enotrus|[6536]|00-SRNP-1877|651[1n]bp
Astraptes enotrus|[6537]|98-SRNP-4808|636[0n]bp
Astraptes enotrus|[6538]|02-SRNP-18995|630[1n]bp
Astraptes enotrus|[6539]|07-SRNP-35000|609[0n]bp
Astraptes enotrus|[6540]|03-SRNP-20573|669[1n]bp
Astraptes enotrus|[6541]|06-SRNP-23433|660[0n]bp
Astraptes enotrus|[6542]|03-SRNP-5834|669[0n]bp
Astraptes enotrus|[6543]|08-SRNP-1658|660[0n]bp
Astraptes enotrus|[6544]|08-SRNP-65001|669[0n]bp
Astraptes enotrus|[6545]|04-SRNP-23876|669[0n]bp
Astraptes enotrus|[6546]|04-SRNP-48434|669[0n]bp
Astraptes enotrus|[6547]|04-SRNP-48802|669[0n]bp
Astraptes enotrus|[6548]|04-SRNP-4532|669[0n]bp
Astraptes enotrus|[6549]|03-SRNP-37547|669[0n]bp
Astraptes enotrus|[6550]|02-SRNP-30318|669[0n]bp
Astraptes enotrus|[6551]|03-SRNP-23940|669[0n]bp
Astraptes enotrus|[6552]|07-SRNP-30922|669[0n]bp
Astraptes enotrus|[6553]|07-SRNP-895|669[0n]bp
Astraptes enotrus|[6554]|06-SRNP-23363|669[0n]bp
Astraptes enotrus|[6555]|07-SRNP-32164|669[0n]bp
Astraptes enotrus|[6556]|07-SRNP-31091|669[0n]bp
Astraptes enotrus|[6557]|07-SRNP-40850|669[0n]bp
Astraptes enotrus|[6558]|07-SRNP-2585|669[0n]bp
Astraptes enotrus|[6559]|07-SRNP-23362|669[0n]bp
Astraptes enotrus|[6560]|03-SRNP-4337|669[0n]bp
Astraptes enotrus|[6561]|00-SRNP-3195|669[0n]bp
Astraptes enotrus|[6562]|03-SRNP-20168|669[0n]bp
Astraptes enotrus|[6563]|03-SRNP-5007|669[0n]bp
Astraptes enotrus|[6564]|03-SRNP-38023|669[0n]bp
Astraptes enotrus|[6565]|01-SRNP-1374|669[0n]bp
Astraptes enotrus|[6566]|01-SRNP-578|669[0n]bp
Astraptes enotrus|[6567]|04-SRNP-4535|669[0n]bp
Astraptes enotrus|[6568]|04-SRNP-15007|669[0n]bp
Astraptes enotrus|[6569]|04-SRNP-61500|669[0n]bp
Astraptes enotrus|[6570]|04-SRNP-55371|669[0n]bp
Astraptes enotrus|[6571]|02-SRNP-29261|669[0n]bp
Astraptes enotrus|[6572]|02-SRNP-4701|669[0n]bp
Astraptes enotrus|[6573]|02-SRNP-29260|669[0n]bp
Astraptes enotrus|[6574]|02-SRNP-18992|669[0n]bp
Astraptes enotrus|[6575]|04-SRNP-14410|669[0n]bp
Astraptes enotrus|[6576]|02-SRNP-19031|669[0n]bp
Astraptes enotrus|[6577]|02-SRNP-4310|669[0n]bp
Astraptes enotrus|[6578]|00-SRNP-12241|669[0n]bp
Astraptes enotrus|[6579]|02-SRNP-29259|669[0n]bp
Astraptes enotrus|[6580]|03-SRNP-21678|669[0n]bp
Astraptes enotrus|[6581]|03-SRNP-27838|669[0n]bp
Astraptes enotrus|[6582]|01-SRNP-569|669[0n]bp
Astraptes enotrus|[6583]|04-SRNP-16060|669[0n]bp
Astraptes enotrus|[6584]|03-SRNP-7340|669[0n]bp
Astraptes enotrus|[6585]|03-SRNP-1596|669[0n]bp
Astraptes enotrus|[6586]|07-SRNP-60787|669[0n]bp
Astraptes enotrus|[6587]|08-SRNP-65002|669[0n]bp
Astraptes enotrus|[6588]|08-SRNP-30981|669[0n]bp
Astraptes enotrus|[6589]|08-SRNP-40360|669[0n]bp
Astraptes enotrus|[6590]|08-SRNP-2125|669[0n]bp
Astraptes enotrus|[6591]|08-SRNP-2321|669[0n]bp
Astraptes enotrus|[6592]|08-SRNP-65460|669[0n]bp
Astraptes enotrus|[6593]|08-SRNP-65411|669[0n]bp
Astraptes enotrus|[6594]|08-SRNP-20582|669[0n]bp
Astraptes enotrus|[6595]|08-SRNP-20893|669[0n]bp
Astraptes enotrus|[6596]|08-SRNP-1656|669[0n]bp

Astraptes enotrus|[6594]|08-SRNP-20582|669[0n]bp
Astraptes enotrus|[6595]|08-SRNP-20893|669[0n]bp
Astraptes enotrus|[6596]|08-SRNP-1656|669[0n]bp
Astraptes enotrus|[6597]|08-SRNP-40359|669[0n]bp
Astraptes enotrus|[6598]|08-SRNP-1657|669[0n]bp
Astraptes enotrus|[6599]|08-SRNP-56038|669[0n]bp
Astraptes enotrus|[6600]|05-SRNP-30647|669[0n]bp
Astraptes enotrus|[6601]|05-SRNP-30648|669[0n]bp
Astraptes enotrus|[6602]|05-SRNP-791|669[0n]bp
Astraptes enotrus|[6603]|05-SRNP-30598|669[0n]bp
Astraptes enotrus|[6604]|09-SRNP-32555|669[0n]bp
Astraptes enotrus|[6605]|09-SRNP-20260|669[0n]bp
Astraptes enotrus|[6606]|06-SRNP-2122|669[0n]bp
Astraptes enotrus|[6607]|06-SRNP-32266|669[0n]bp
Astraptes enotrus|[6608]|06-SRNP-35215|669[0n]bp
Astraptes enotrus|[6609]|06-SRNP-22782|669[0n]bp
Astraptes enotrus|[6610]|09-SRNP-22799|669[0n]bp
Astraptes enotrus|[6611]|09-SRNP-20286|669[0n]bp
Chioides catillus|[6612]|07-SRNP-57955|669[0n]bp
Chioides catillus|[6613]|07-SRNP-20324|666[0n]bp
Chioides catillus|[6614]|06-SRNP-21086|666[0n]bp
Chioides catillus|[6615]|07-SRNP-938|669[0n]bp
Chioides catillus|[6616]|07-SRNP-58548|669[0n]bp
Chioides catillus|[6617]|07-SRNP-32811|669[0n]bp
Chioides catillus|[6618]|07-SRNP-939|669[0n]bp
Chioides catillus|[6619]|07-SRNP-20124|669[0n]bp
Chioides catillus|[6620]|07-SRNP-22429|669[0n]bp
Chioides catillus|[6621]|07-SRNP-20115|669[0n]bp
Chioides catillus|[6622]|07-SRNP-20187|669[0n]bp
Chioides catillus|[6623]|07-SRNP-20128|669[0n]bp
Chioides catillus|[6624]|07-SRNP-45146|669[0n]bp
Chioides catillus|[6625]|05-SRNP-45682|669[0n]bp
Chioides catillus|[6626]|04-SRNP-34914|669[0n]bp
Chioides catillus|[6627]|04-SRNP-56106|669[0n]bp
Chioides catillus|[6628]|04-SRNP-23839|669[0n]bp
Chioides catillus|[6629]|04-SRNP-34756|669[0n]bp
Chioides catillus|[6630]|04-SRNP-23173|669[0n]bp
Chioides catillus|[6631]|04-SRNP-24064|669[0n]bp
Chioides catillus|[6632]|03-SRNP-1704|654[0n]bp
Chioides catillus|[6633]|04-SRNP-23511|669[4n]bp
Chioides catillus|[6634]|03-SRNP-1498|642[0n]bp
Chioides catillus|[6635]|05-SRNP-31764|576[1n]bp
Chioides catillus|[6636]|07-SRNP-58581|645[0n]bp
Chioides zilpa|[6637]|02-SRNP-10079|645[0n]bp
Chioides zilpa|[6638]|07-SRNP-45681|669[0n]bp
Chioides zilpa|[6639]|04-SRNP-45690|669[0n]bp
Chioides zilpa|[6640]|04-SRNP-45597|669[0n]bp
Chioides zilpa|[6641]|04-SRNP-45616|669[0n]bp
Chioides zilpa|[6642]|04-SRNP-45596|669[0n]bp
Chioides zilpa|[6643]|04-SRNP-45594|669[0n]bp
Chioides zilpa|[6644]|04-SRNP-45618|669[0n]bp
Chioides zilpa|[6645]|04-SRNP-45595|669[0n]bp
Chioides zilpa|[6646]|04-SRNP-45749|669[0n]bp
Chioides zilpa|[6647]|02-SRNP-10078|654[0n]bp
Chioides zilpa|[6648]|07-SRNP-45680|660[0n]bp
Chioides zilpa|[6649]|07-SRNP-45864|669[0n]bp
Chioides zilpa|[6650]|09-SRNP-57613|669[0n]bp
Epargyreus Burns04|[6651]|02-SRNP-29831|639[0n]bp
Epargyreus Burns04|[6652]|02-SRNP-29832|639[0n]bp
Epargyreus Burns04|[6653]|01-SRNP-14765|639[0n]bp
Epargyreus Burns04|[6654]|03-SRNP-30086|669[0n]bp
Epargyreus Burns04|[6655]|03-SRNP-29910|669[0n]bp
Epargyreus Burns04|[6656]|03-SRNP-29907|669[0n]bp
Epargyreus Burns04|[6657]|01-SRNP-17907|669[0n]bp
Epargyreus Burns05|[6658]|02-SRNP-4752|669[0n]bp
Epargyreus Burns04|[6659]|03-SRNP-29911|669[0n]bp
Epargyreus Burns04|[6660]|07-SRNP-45120|669[0n]bp
Epargyreus Burns04|[6661]|02-SRNP-16314|630[1n]bp
Epargyreus Burns04|[6662]|04-SRNP-24319|669[0n]bp
Epargyreus Burns04|[6663]|96-SRNP-9218|405[0n]bp
Epargyreus Burns04|[6664]|01-SRNP-16938|504[0n]bp
Epargyreus Burns04|[6665]|07-SRNP-20185|669[0n]bp
Epargyreus Burns04|[6666]|07-SRNP-20050|645[0n]bp
Epargyreus Burns04|[6667]|07-SRNP-20051|630[0n]bp
Epargyreus Burns07|[6668]|07-SRNP-21582|612[0n]bp
Epargyreus Burns07|[6669]|07-SRNP-21586|627[0n]bp
Epargyreus Burns07|[6670]|04-SRNP-55660|669[0n]bp
Epargyreus Burns07|[6671]|05-SRNP-30037|645[0n]bp
Epargyreus Burns07|[6672]|05-SRNP-21988|648[1n]bp
Epargyreus Burns07|[6673]|04-SRNP-15059|669[0n]bp
Epargyreus Burns07|[6674]|04-SRNP-1184|669[0n]bp
Epargyreus Burns07|[6675]|02-SRNP-16668|639[0n]bp
Epargyreus Burns07|[6676]|02-SRNP-16077|657[0n]bp
Epargyreus Burns07|[6677]|06-SRNP-32932|609[0n]bp
Epargyreus Burns07|[6678]|07-SRNP-21670|669[0n]bp
Epargyreus Burns07|[6679]|08-SRNP-2670|669[0n]bp
Epargyreus Burns07|[6680]|08-SRNP-31981|669[0n]bp
Epargyreus Burns05|[6681]|06-SRNP-46832|669[0n]bp
Epargyreus Burns05|[6682]|06-SRNP-46827|669[0n]bp
Epargyreus Burns05|[6683]|02-SRNP-29177|669[0n]bp
Epargyreus Burns05|[6684]|02-SRNP-29170|669[0n]bp
Epargyreus Burns05|[6685]|02-SRNP-29171|669[0n]bp
Epargyreus Burns05|[6686]|02-SRNP-4753|669[0n]bp
Epargyreus Burns05|[6687]|02-SRNP-5328|657[0n]bp
Epargyreus Burns05|[6688]|02-SRNP-17323|651[0n]bp
Epargyreus Burns05|[6689]|02-SRNP-4118|480[0n]bp
Epargyreus Burns05|[6690]|02-SRNP-15163|501[1n]bp
Epargyreus Burns05|[6691]|07-SRNP-56896|633[0n]bp
Epargyreus Burns05|[6692]|07-SRNP-46910|669[0n]bp
Epargyreus Burns05|[6693]|05-SRNP-65949|645[0n]bp
Epargyreus Burns05|[6694]|02-SRNP-28000|639[0n]bp
Epargyreus Burns05|[6695]|02-SRNP-4513|480[0n]bp
Epargyreus Burns05|[6696]|04-SRNP-14225|669[0n]bp

Epargyreus Burns05|[6694]|02-SRNP-28000|639[0n]bp
Epargyreus Burns05|[6695]|02-SRNP-4513|480[0n]bp
Epargyreus Burns05|[6696]|04-SRNP-14225|669[0n]bp
Epargyreus Burns05|[6697]|02-SRNP-29175|669[0n]bp
Epargyreus Burns05|[6698]|08-SRNP-15158|669[0n]bp
Epargyreus Burns06|[6699]|97-SRNP-1659|561[0n]bp
Epargyreus Burns06|[6700]|02-SRNP-8179|618[0n]bp
Epargyreus Burns06|[6701]|03-SRNP-20073|669[0n]bp
Epargyreus Burns06|[6702]|07-SRNP-1276|669[0n]bp
Epargyreus Burns06|[6703]|07-SRNP-427|669[0n]bp
Epargyreus Burns06|[6704]|08-SRNP-1110|669[0n]bp
Epargyreus Burns11|[6705]|01-SRNP-16427|621[0n]bp
Epargyreus Burns11|[6706]|07-SRNP-20344|669[0n]bp
Epargyreus Burns11|[6707]|07-SRNP-20063|669[0n]bp
Epargyreus Burns11|[6708]|04-SRNP-14219|609[0n]bp
Epargyreus Burns11|[6709]|07-SRNP-20341|621[0n]bp
Epargyreus Burns11|[6710]|07-SRNP-23521|621[0n]bp
Epargyreus Burns11|[6711]|05-SRNP-45151|669[0n]bp
Epargyreus Burns11|[6712]|07-SRNP-20334|669[0n]bp
Epargyreus Burns11|[6713]|05-SRNP-45152|669[1n]bp
Epargyreus Burns11|[6714]|07-SRNP-20333|603[0n]bp
Epargyreus Burns11|[6715]|06-SRNP-67531|606[0n]bp
Epargyreus Burns11|[6716]|04-SRNP-21554|606[0n]bp
Epargyreus Burns11|[6717]|01-SRNP-16541|384[0n]bp
Epargyreus Burns11|[6718]|06-SRNP-58925|591[0n]bp
Epargyreus Burns11|[6719]|07-SRNP-20296|606[0n]bp
Epargyreus Burns11|[6720]|07-SRNP-20364|606[0n]bp
Epargyreus Burns11|[6721]|07-SRNP-20329|669[0n]bp
Epargyreus Burns11|[6722]|09-SRNP-55106|669[0n]bp
Epargyreus Burns12|[6723]|08-SRNP-24800|627[0n]bp
Epargyreus Burns12|[6724]|07-SRNP-46132|660[2n]bp
Epargyreus Burns12|[6725]|07-SRNP-21329|495[0n]bp
Epargyreus Burns12|[6726]|07-SRNP-65538|669[0n]bp
Epargyreus Burns12|[6727]|08-SRNP-24801|669[0n]bp
Epargyreus Burns03|[6728]|08-SRNP-45027|669[0n]bp
Epargyreus Burns03|[6729]|07-SRNP-57869|645[0n]bp
Epargyreus Burns03|[6730]|94-SRNP-8731|540[0n]bp
Epargyreus Burns03|[6731]|04-SRNP-45025|669[0n]bp
Epargyreus Burns03|[6732]|04-SRNP-47759|630[0n]bp
Epargyreus Burns03|[6733]|07-SRNP-57031|669[0n]bp
Epargyreus Burns03|[6734]|05-SRNP-45698|669[0n]bp
Epargyreus Burns03|[6735]|06-SRNP-47189|669[0n]bp
Epargyreus Burns03|[6736]|04-SRNP-46436|669[0n]bp
Epargyreus Burns03|[6737]|04-SRNP-46435|669[0n]bp
Epargyreus Burns03|[6738]|04-SRNP-46800|669[0n]bp
Epargyreus Burns03|[6739]|04-SRNP-46437|669[0n]bp
Epargyreus Burns03|[6740]|04-SRNP-46819|669[0n]bp
Epargyreus Burns03|[6741]|04-SRNP-48332|669[0n]bp
Epargyreus Burns03|[6742]|04-SRNP-47032|669[0n]bp
Epargyreus Burns03|[6743]|04-SRNP-47031|669[0n]bp
Epargyreus Burns03|[6744]|04-SRNP-46799|669[0n]bp
Epargyreus Burns03|[6745]|04-SRNP-46820|669[0n]bp
Epargyreus Burns03|[6746]|94-SRNP-8741|384[0n]bp
Epargyreus Burns03|[6747]|96-SRNP-10052|384[0n]bp
Epargyreus Burns03|[6748]|04-SRNP-48150|669[0n]bp
Epargyreus Burns03|[6749]|08-SRNP-58522|669[2n]bp
Epargyreus Burns02|[6750]|01-SRNP-3183|639[0n]bp
Epargyreus Burns02|[6751]|02-SRNP-29202|639[0n]bp
Epargyreus Burns02|[6752]|05-SRNP-30057|645[0n]bp
Epargyreus Burns02|[6753]|04-SRNP-27300|669[0n]bp
Epargyreus Burns02|[6754]|07-SRNP-61270|669[0n]bp
Epargyreus Burns02|[6755]|08-SRNP-4758|669[0n]bp
Epargyreus Burns02|[6756]|09-SRNP-71932|669[0n]bp
Epargyreus Burns02|[6757]|01-SRNP-18804|639[0n]bp
Epargyreus Burns02|[6758]|01-SRNP-18806|651[0n]bp
Epargyreus Burns02|[6759]|02-SRNP-15408|639[1n]bp
Epargyreus Burns02|[6760]|08-SRNP-72029|669[0n]bp
Epargyreus Burns02|[6761]|04-SRNP-15015|669[0n]bp
Epargyreus Burns02|[6762]|07-SRNP-57672|645[0n]bp
Epargyreus Burns02|[6763]|04-SRNP-15177|669[0n]bp
Epargyreus Burns02|[6764]|05-SRNP-33218|645[0n]bp
Epargyreus Burns02|[6765]|07-SRNP-42506|642[0n]bp
Epargyreus Burns02|[6766]|02-SRNP-524|639[0n]bp
Epargyreus Burns02|[6767]|02-SRNP-525|642[0n]bp
Epargyreus Burns02|[6768]|04-SRNP-24265|669[0n]bp
Epargyreus Burns02|[6769]|92-SRNP-2367|615[1n]bp
Epargyreus Burns02|[6770]|05-SRNP-2701|639[0n]bp
Epargyreus Burns02|[6771]|04-SRNP-13417|669[0n]bp
Epargyreus Burns02|[6772]|04-SRNP-24405|669[0n]bp
Epargyreus Burns02|[6773]|02-SRNP-29200|657[0n]bp
Epargyreus Burns02|[6774]|02-SRNP-32945|663[0n]bp
Epargyreus Burns02|[6775]|04-SRNP-14174|669[0n]bp
Epargyreus Burns02|[6776]|04-SRNP-15077|669[0n]bp
Epargyreus Burns02|[6777]|04-SRNP-15090|669[0n]bp
Epargyreus Burns02|[6778]|04-SRNP-15384|669[0n]bp
Epargyreus Burns02|[6779]|04-SRNP-4655|669[0n]bp
Epargyreus Burns02|[6780]|04-SRNP-15720|669[0n]bp
Epargyreus Burns02|[6781]|06-SRNP-43439|669[0n]bp
Epargyreus Burns02|[6782]|06-SRNP-43442|669[0n]bp
Epargyreus Burns02|[6783]|06-SRNP-43440|669[0n]bp
Epargyreus Burns02|[6784]|05-SRNP-32694|669[0n]bp
Epargyreus Burns02|[6785]|05-SRNP-1416|669[0n]bp
Epargyreus Burns02|[6786]|04-SRNP-27299|669[0n]bp
Epargyreus Burns02|[6787]|05-SRNP-92|669[0n]bp
Epargyreus Burns02|[6788]|08-SRNP-4814|669[0n]bp
Epargyreus Burns02|[6789]|08-SRNP-4777|669[0n]bp
Epargyreus Burns02|[6790]|09-SRNP-72516|669[0n]bp
Epargyreus Burns02|[6791]|09-SRNP-73319|669[0n]bp
Epargyreus Burns02|[6792]|09-SRNP-76379|669[0n]bp
Epargyreus Burns02|[6793]|09-SRNP-75608|669[0n]bp
Codatractus imalena|[6794]|00-SRNP-10752|594[8n]bp
Codatractus imalena|[6795]|97-SRNP-1645|606[6n]bp
Codatractus imalena|[6796]|02-SRNP-24526|315[1n]bp

Codatractus imalena|[6794]|00-SRNP-10752|594[8n]bp
Codatractus imalena|[6795]|97-SRNP-1645|606[6n]bp
Codatractus imalena|[6796]|02-SRNP-24526|315[1n]bp
Codatractus imalena|[6797]|01-SRNP-7390|321[1n]bp
Codatractus imalena|[6798]|00-SRNP-10734|525[2n]bp
Codatractus imalena|[6799]|04-SRNP-35578|627[1n]bp
Codatractus imalena|[6800]|01-SRNP-7384|576[0n]bp
Codatractus imalena|[6801]|98-SRNP-3451|600[0n]bp
Codatractus imalena|[6802]|99-SRNP-5496|606[0n]bp
Codatractus melon|[6803]|92-SRNP-3686|618[1n]bp
Codatractus imalena|[6804]|00-SRNP-9613|603[0n]bp
Codatractus imalena|[6805]|00-SRNP-10005|621[2n]bp
Codatractus imalena|[6806]|03-SRNP-4322|561[1n]bp
Codatractus imalena|[6807]|00-SRNP-10020|606[0n]bp
Codatractus imalena|[6808]|01-SRNP-21206|537[0n]bp
Codatractus imalena|[6809]|00-SRNP-9747|588[0n]bp
Codatractus imalena|[6810]|01-SRNP-21186|609[1n]bp
Codatractus imalena|[6811]|01-SRNP-7119|606[0n]bp
Codatractus imalena|[6812]|01-SRNP-7392|588[0n]bp
Codatractus imalena|[6813]|04-SRNP-46325|669[0n]bp
Codatractus imalena|[6814]|04-SRNP-46730|669[0n]bp
Codatractus imalena|[6815]|04-SRNP-35580|669[0n]bp
Codatractus imalena|[6816]|04-SRNP-35579|669[0n]bp
Codatractus imalena|[6817]|04-SRNP-46729|669[0n]bp
Codatractus imalena|[6818]|04-SRNP-47215|669[0n]bp
Codatractus imalena|[6819]|08-SRNP-35884|669[0n]bp
Codatractus imalena|[6820]|01-SRNP-21138|621[1n]bp
Codatractus melon|[6821]|93-SRNP-5542|669[0n]bp
Codatractus melon|[6822]|93-SRNP-5545|669[0n]bp
Codatractus melon|[6823]|92-SRNP-3678|609[0n]bp
Codatractus melon|[6824]|90-SRNP-1878|603[0n]bp
Codatractus melon|[6825]|92-SRNP-3626|495[1n]bp
Codatractus melon|[6826]|93-SRNP-3061|609[0n]bp
Codatractus melon|[6827]|93-SRNP-5544|669[0n]bp
Codatractus alcaeus|[6828]|01-SRNP-16125|654[0n]bp
Codatractus alcaeus|[6829]|99-SRNP-2489|669[0n]bp
Codatractus carlos|[6830]|06-SRNP-2517|669[0n]bp
Codatractus imalena|[6831]|00-SRNP-10506|669[0n]bp
Codatractus alcaeus|[6832]|04-SRNP-45206|669[0n]bp
Codatractus imalena|[6833]|09-SRNP-12121|669[0n]bp
Codatractus imalena|[6834]|09-SRNP-12120|669[0n]bp
Ridens mephitisDHJ02|[6835]|04-SRNP-35805|669[0n]bp
Ridens mephitisDHJ02|[6836]|04-SRNP-36233|669[0n]bp
Ridens mephitisDHJ02|[6837]|02-SRNP-8370|669[0n]bp
Ridens mephitisDHJ02|[6838]|03-SRNP-4203|669[0n]bp
Ridens mephitisDHJ02|[6839]|00-SRNP-9345|669[1n]bp
Ridens mephitisDHJ02|[6840]|03-SRNP-3199|480[0n]bp
Ridens mephitisDHJ02|[6841]|02-SRNP-23638|642[0n]bp
Ridens mephitisDHJ02|[6842]|99-SRNP-1768|615[1n]bp
Ridens mephitisDHJ02|[6843]|05-SRNP-36028|669[5n]bp
Ridens mephitisDHJ02|[6844]|05-SRNP-36029|669[2n]bp
Ridens mephitisDHJ04|[6845]|06-SRNP-60255|669[0n]bp
Ridens mephitisDHJ04|[6846]|06-SRNP-60252|669[0n]bp
Ridens mephitisDHJ04|[6847]|93-SRNP-3068|612[0n]bp
Ridens mephitisDHJ04|[6848]|92-SRNP-5754|618[0n]bp
Ridens mephitisDHJ04|[6849]|06-SRNP-59545|606[1n]bp
Ridens mephitisDHJ04|[6850]|08-SRNP-57638|645[0n]bp
Ridens mephitisDHJ04|[6851]|08-SRNP-57623|669[0n]bp
Ridens mephitisDHJ04|[6852]|08-SRNP-57620|669[0n]bp
Ridens mephitisDHJ04|[6853]|08-SRNP-57621|669[0n]bp
Ridens mephitisDHJ03|[6854]|07-SRNP-56610|633[0n]bp
Ridens mephitisDHJ03|[6855]|99-SRNP-10376|633[1n]bp
Ridens mephitisDHJ03|[6856]|08-SRNP-57249|669[0n]bp
Ridens mephitisDHJ03|[6857]|06-SRNP-57708|669[0n]bp
Ridens mephitisDHJ03|[6858]|06-SRNP-60058|669[0n]bp
Ridens mephitisDHJ03|[6859]|06-SRNP-59544|669[0n]bp
Ridens mephitisDHJ03|[6860]|06-SRNP-46964|669[0n]bp
Ridens mephitisDHJ03|[6861]|99-SRNP-11670|669[0n]bp
Ridens mephitisDHJ03|[6862]|02-SRNP-32153|669[0n]bp
Ridens mephitisDHJ03|[6863]|02-SRNP-32151|669[0n]bp
Ridens mephitisDHJ03|[6864]|98-SRNP-2715|669[0n]bp
Ridens mephitisDHJ03|[6865]|99-SRNP-11297|669[0n]bp
Ridens mephitisDHJ03|[6866]|99-SRNP-10355|669[0n]bp
Ridens mephitisDHJ03|[6867]|06-SRNP-59546|621[1n]bp
Ridens mephitisDHJ03|[6868]|08-SRNP-56874|333[0n]bp
Ridens mephitisDHJ03|[6869]|05-SRNP-65555|627[0n]bp
Ridens mephitisDHJ03|[6870]|06-SRNP-36844|594[0n]bp
Ridens mephitisDHJ03|[6871]|07-SRNP-57507|642[0n]bp
Ridens mephitisDHJ03|[6872]|07-SRNP-57088|642[0n]bp
Ridens mephitisDHJ03|[6873]|07-SRNP-57579|639[0n]bp
Ridens mephitisDHJ03|[6874]|08-SRNP-57406|669[0n]bp
Ridens mephitisDHJ03|[6875]|06-SRNP-56397|669[0n]bp
Ridens mephitisDHJ03|[6876]|97-SRNP-1073.01|669[0n]bp
Ridens mephitisDHJ03|[6877]|99-SRNP-11678|669[0n]bp
Ridens mephitisDHJ03|[6878]|99-SRNP-11679|669[0n]bp
Ridens mephitisDHJ03|[6879]|06-SRNP-58702|582[0n]bp
Ridens mephitisDHJ03|[6880]|06-SRNP-60253|603[0n]bp
Ridens mephitisDHJ03|[6881]|06-SRNP-59547|609[0n]bp
Ridens mephitisDHJ03|[6882]|06-SRNP-60254|615[1n]bp
Ridens mephitisDHJ03|[6883]|08-SRNP-57289|660[0n]bp
Ridens mephitisDHJ03|[6884]|08-SRNP-57560|669[0n]bp
Ridens mephitisDHJ03|[6885]|08-SRNP-57622|669[0n]bp
Ridens pancheDHJ01|[6886]|06-SRNP-31875|669[0n]bp
Ridens pancheDHJ01|[6887]|06-SRNP-31873|669[0n]bp
Ridens pancheDHJ01|[6888]|06-SRNP-32333|642[0n]bp
Ridens pancheDHJ01|[6889]|00-SRNP-9482|615[0n]bp
Ridens pancheDHJ02|[6890]|07-SRNP-35883|642[0n]bp
Ridens pancheDHJ02|[6891]|01-SRNP-7463|642[0n]bp
Ridens pancheDHJ02|[6892]|07-SRNP-35884|651[1n]bp
Ridens pancheDHJ02|[6893]|02-SRNP-9387|630[0n]bp
Ridens pancheDHJ02|[6894]|01-SRNP-6746|648[0n]bp
Ridens pancheDHJ02|[6895]|02-SRNP-9385|645[0n]bp
Ridens pancheDHJ02|[6896]|03-SRNP-4091|645[0n]bp

Ridens pancheDHJ02|[6894]|01-SRNP-6746|648[0n]bp
Ridens pancheDHJ02|[6895]|02-SRNP-9385|645[0n]bp
Ridens pancheDHJ02|[6896]|03-SRNP-4091|645[0n]bp
Ridens pancheDHJ02|[6897]|02-SRNP-8372|645[0n]bp
Ridens pancheDHJ02|[6898]|01-SRNP-6929|669[0n]bp
Ridens pancheDHJ02|[6899]|02-SRNP-8538|669[0n]bp
Ridens pancheDHJ02|[6900]|06-SRNP-3632|669[0n]bp
Ridens pancheDHJ02|[6901]|06-SRNP-35129|669[0n]bp
Ridens pancheDHJ02|[6902]|05-SRNP-35185|669[0n]bp
Ridens pancheDHJ02|[6903]|05-SRNP-35180|669[0n]bp
Ridens pancheDHJ02|[6904]|04-SRNP-35425|669[0n]bp
Ridens pancheDHJ02|[6905]|05-SRNP-31719|669[0n]bp
Ridens pancheDHJ02|[6906]|04-SRNP-35446|669[0n]bp
Ridens pancheDHJ02|[6907]|04-SRNP-35428|669[0n]bp
Ridens pancheDHJ02|[6908]|03-SRNP-6073|666[0n]bp
Ridens pancheDHJ02|[6909]|06-SRNP-3638|618[0n]bp
Ridens pancheDHJ02|[6910]|06-SRNP-3482|648[0n]bp
Ridens pancheDHJ02|[6911]|03-SRNP-3775|591[3n]bp
Ridens pancheDHJ02|[6912]|01-SRNP-6839|624[0n]bp
Ridens pancheDHJ02|[6913]|02-SRNP-9450|654[0n]bp
Ridens pancheDHJ02|[6914]|02-SRNP-8537|618[0n]bp
Ridens pancheDHJ02|[6915]|03-SRNP-3740|609[0n]bp
Ridens pancheDHJ02|[6916]|03-SRNP-4014|612[0n]bp
Ridens pancheDHJ02|[6917]|01-SRNP-9377|666[0n]bp
Ridens pancheDHJ02|[6918]|01-SRNP-6552|666[0n]bp
Ridens pancheDHJ02|[6919]|00-SRNP-10808|669[0n]bp
Ridens pancheDHJ02|[6920]|00-SRNP-10799|669[0n]bp
Ridens pancheDHJ02|[6921]|03-SRNP-3772|669[0n]bp
Ridens pancheDHJ02|[6922]|03-SRNP-3769|669[0n]bp
Ridens pancheDHJ02|[6923]|98-SRNP-2005|669[0n]bp
Ridens pancheDHJ02|[6924]|03-SRNP-4067|669[0n]bp
Ridens pancheDHJ02|[6925]|03-SRNP-3596|669[0n]bp
Ridens pancheDHJ02|[6926]|03-SRNP-4312|669[0n]bp
Ridens pancheDHJ02|[6927]|03-SRNP-3808|669[0n]bp
Ridens pancheDHJ02|[6928]|02-SRNP-8369|669[0n]bp
Ridens pancheDHJ02|[6929]|02-SRNP-8368|669[0n]bp
Ridens pancheDHJ02|[6930]|03-SRNP-3774|669[0n]bp
Ridens pancheDHJ02|[6931]|03-SRNP-3634|669[0n]bp
Ridens pancheDHJ02|[6932]|99-SRNP-453|669[0n]bp
Ridens pancheDHJ02|[6933]|06-SRNP-3634|669[0n]bp
Ridens pancheDHJ02|[6934]|07-SRNP-35521|669[0n]bp
Ridens pancheDHJ02|[6935]|08-SRNP-35179|669[0n]bp
Ridens pancheDHJ02|[6936]|08-SRNP-35569|669[0n]bp
Ridens pancheDHJ02|[6937]|03-SRNP-23955|669[0n]bp
Ridens pancheDHJ02|[6938]|03-SRNP-3637|669[0n]bp
Ridens pancheDHJ02|[6939]|08-SRNP-35487|669[0n]bp
Ridens pancheDHJ02|[6940]|08-SRNP-35331|639[0n]bp
Ridens Burns01|[6941]|06-SRNP-31900|642[0n]bp
Ridens Burns01|[6942]|05-SRNP-30993|636[0n]bp
Ridens Burns01|[6943]|05-SRNP-30994|633[1n]bp
Ridens Burns01|[6944]|05-SRNP-30995|633[1n]bp
Ridens Burns01|[6945]|02-SRNP-28461|630[0n]bp
Ridens Burns01|[6946]|05-SRNP-31102|687[0n]bp
Ridens Burns01|[6947]|02-SRNP-28462|576[0n]bp
Ridens Burns01|[6948]|05-SRNP-31099|669[0n]bp
Ridens Burns01|[6949]|06-SRNP-31896|669[0n]bp
Ridens Burns01|[6950]|06-SRNP-7320|669[0n]bp
Ridens Burns01|[6951]|06-SRNP-31898|669[0n]bp
Ridens Burns01|[6952]|06-SRNP-31899|669[0n]bp
Ridens Burns01|[6953]|06-SRNP-31901|669[0n]bp
Ridens biolleyi|[6954]|09-SRNP-35558|663[0n]bp
Ridens biolleyi|[6955]|02-SRNP-8021|624[0n]bp
Ridens biolleyi|[6956]|01-SRNP-6396|591[0n]bp
Ridens biolleyi|[6957]|98-SRNP-2547|594[0n]bp
Ridens biolleyi|[6958]|00-SRNP-9333|594[0n]bp
Ridens biolleyi|[6959]|00-SRNP-9370|594[0n]bp
Ridens biolleyi|[6960]|00-SRNP-9238|591[0n]bp
Ridens biolleyi|[6961]|00-SRNP-9259|378[0n]bp
Ridens biolleyi|[6962]|02-SRNP-8016|396[0n]bp
Ridens biolleyi|[6963]|00-SRNP-9229|384[0n]bp
Ridens biolleyi|[6964]|01-SRNP-6395|384[0n]bp
Ridens biolleyi|[6965]|99-SRNP-17191|558[0n]bp
Ridens biolleyi|[6966]|08-SRNP-31106|669[0n]bp
Ridens biolleyi|[6967]|09-SRNP-36067|669[0n]bp
Ridens biolleyi|[6968]|09-SRNP-36267|669[0n]bp
Ridens cachinnans|[6969]|08-SRNP-37306|669[0n]bp
Ridens cachinnans|[6970]|02-SRNP-23213|612[0n]bp
Ridens cachinnans|[6971]|02-SRNP-23212|612[0n]bp
Ridens cachinnans|[6972]|08-SRNP-35997|576[0n]bp
Ridens cachinnans|[6973]|08-SRNP-35556|576[0n]bp
Ridens cachinnans|[6974]|09-SRNP-36708|669[0n]bp
Venada nevada|[6975]|05-SRNP-35056|666[0n]bp
Venada nevada|[6976]|07-SRNP-35995|645[0n]bp
Venada nevada|[6977]|06-SRNP-36696|645[0n]bp
Venada nevada|[6978]|03-SRNP-3106|645[0n]bp
Venada nevada|[6979]|03-SRNP-4419|645[0n]bp
Venada nevada|[6980]|03-SRNP-4070|645[0n]bp
Venada nevada|[6981]|03-SRNP-3148|645[0n]bp
Venada nevada|[6982]|03-SRNP-3972|645[1n]bp
Venada nevada|[6983]|00-SRNP-9986|642[0n]bp
Venada nevada|[6984]|05-SRNP-227|669[0n]bp
Venada nevada|[6985]|05-SRNP-30877|669[0n]bp
Venada nevada|[6986]|05-SRNP-226|669[0n]bp
Venada nevada|[6987]|05-SRNP-577|669[0n]bp
Venada nevada|[6988]|05-SRNP-35565|669[0n]bp
Venada nevada|[6989]|05-SRNP-35356|669[0n]bp
Venada nevada|[6990]|04-SRNP-35614|669[0n]bp
Venada nevada|[6991]|04-SRNP-35681|669[0n]bp
Venada nevada|[6992]|04-SRNP-56616|669[0n]bp
Venada nevada|[6993]|04-SRNP-56617|669[0n]bp
Venada nevada|[6994]|04-SRNP-61462|648[0n]bp
Venada nevada|[6995]|06-SRNP-36333|654[0n]bp
Venada nevada|[6996]|06-SRNP-36619|648[0n]bp

Venada nevada|[6994]|04-SRNP-61462|648[0n]bp
Venada nevada|[6995]|06-SRNP-36333|654[0n]bp
Venada nevada|[6996]|06-SRNP-36619|648[0n]bp
Venada nevada|[6997]|98-SRNP-2758|630[0n]bp
Venada nevada|[6998]|03-SRNP-3990|492[1n]bp
Venada nevada|[6999]|03-SRNP-4768|492[0n]bp
Venada nevada|[7000]|03-SRNP-3346|636[1n]bp
Venada nevada|[7001]|05-SRNP-35244|651[0n]bp
Venada nevada|[7002]|05-SRNP-30878|669[0n]bp
Venada nevada|[7003]|05-SRNP-30663|669[0n]bp
Venada nevada|[7004]|05-SRNP-30665|669[0n]bp
Venada nevada|[7005]|07-SRNP-36157|669[0n]bp
Venada nevada|[7006]|07-SRNP-36499|669[0n]bp
Venada nevada|[7007]|07-SRNP-35918|669[0n]bp
Venada nevada|[7008]|07-SRNP-35840|669[0n]bp
Venada nevada|[7009]|07-SRNP-35841|669[0n]bp
Venada nevada|[7010]|08-SRNP-35509|669[0n]bp
Venada nevada|[7011]|08-SRNP-35511|669[0n]bp
Venada nevada|[7012]|08-SRNP-35510|669[0n]bp
Venada nevada|[7013]|08-SRNP-35612|642[0n]bp
Venada daneva|[7014]|06-SRNP-4962|669[0n]bp
Venada daneva|[7015]|03-SRNP-5810|645[0n]bp
Venada daneva|[7016]|02-SRNP-20143|645[0n]bp
Venada daneva|[7017]|03-SRNP-6535|657[0n]bp
Venada daneva|[7018]|99-SRNP-18830|594[0n]bp
Venada daneva|[7019]|03-SRNP-5862|492[0n]bp
Venada daneva|[7020]|97-SRNP-11560|396[0n]bp
Venada daneva|[7021]|03-SRNP-5863|576[0n]bp
Venada daneva|[7022]|03-SRNP-5651|576[0n]bp
Venada daneva|[7023]|04-SRNP-32605|669[0n]bp
Venada daneva|[7024]|04-SRNP-2360|669[0n]bp
Venada daneva|[7025]|04-SRNP-2359|669[0n]bp
Venada daneva|[7026]|04-SRNP-2191|669[0n]bp
Venada daneva|[7027]|04-SRNP-2688|669[0n]bp
Venada daneva|[7028]|05-SRNP-2577|669[0n]bp
Venada daneva|[7029]|07-SRNP-2501|669[0n]bp
Venada daneva|[7030]|07-SRNP-2502|669[0n]bp
Venada daneva|[7031]|07-SRNP-2371|669[0n]bp
Venada daneva|[7032]|08-SRNP-2594|669[0n]bp
Venada daneva|[7033]|08-SRNP-2680|669[0n]bp
Venada daneva|[7034]|08-SRNP-2705|669[0n]bp
Venada daneva|[7035]|08-SRNP-31224|669[0n]bp
Venada daneva|[7036]|09-SRNP-32294|669[0n]bp
Venada naranja|[7037]|00-SRNP-9226|669[0n]bp
Venada naranja|[7038]|01-SRNP-6992|669[0n]bp
Venada naranja|[7039]|02-SRNP-8086|669[0n]bp
Venada naranja|[7040]|03-SRNP-22199|669[0n]bp
Venada naranja|[7041]|00-SRNP-9327|669[0n]bp
Venada naranja|[7042]|00-SRNP-9252|669[0n]bp
Venada naranja|[7043]|00-SRNP-9183|669[0n]bp
Venada naranja|[7044]|00-SRNP-9324|669[0n]bp
Venada naranja|[7045]|99-SRNP-1069|669[0n]bp
Venada naranja|[7046]|00-SRNP-9050|669[0n]bp
Venada naranja|[7047]|06-SRNP-35940|669[0n]bp
Venada naranja|[7048]|03-SRNP-4740|657[0n]bp
Venada naranja|[7049]|02-SRNP-24573|636[0n]bp
Venada naranja|[7050]|03-SRNP-3901|636[0n]bp
Venada naranja|[7051]|04-SRNP-35682|642[0n]bp
Venada naranja|[7052]|00-SRNP-9329|669[0n]bp
Venada naranja|[7053]|04-SRNP-36174|669[0n]bp
Venada naranja|[7054]|05-SRNP-35059|669[0n]bp
Venada naranja|[7055]|09-SRNP-35765|669[0n]bp
Venada Janzen01|[7056]|09-SRNP-36371|669[0n]bp
Venada Janzen01|[7057]|09-SRNP-36370|669[0n]bp
Venada cacao|[7058]|02-SRNP-23324|669[0n]bp
Venada cacao|[7059]|02-SRNP-23004|657[0n]bp
Venada cacao|[7060]|02-SRNP-23362|669[0n]bp
Venada cacao|[7061]|02-SRNP-23364|663[0n]bp
Venada cacao|[7062]|01-SRNP-6880|633[0n]bp
Venada cacaoDHJ02|[7063]|09-SRNP-36883|669[0n]bp
Polythrix kanshul|[7064]|04-SRNP-60560|669[0n]bp
Polythrix kanshul|[7065]|05-SRNP-5452|633[0n]bp
Polythrix kanshul|[7066]|09-SRNP-65100|669[0n]bp
Polythrix kanshul|[7067]|05-SRNP-2332|666[1n]bp
Polythrix kanshul|[7068]|00-SRNP-11396|555[0n]bp
Polythrix kanshul|[7069]|00-SRNP-373|576[0n]bp
Polythrix kanshul|[7070]|04-SRNP-60051|669[0n]bp
Polythrix kanshul|[7071]|06-SRNP-8907|669[0n]bp
Polythrix kanshul|[7072]|06-SRNP-9915|669[0n]bp
Polythrix kanshul|[7073]|07-SRNP-738|669[0n]bp
Polythrix kanshul|[7074]|07-SRNP-65895|669[0n]bp
Polythrix kanshul|[7075]|09-SRNP-42534|669[0n]bp
Porphyrogenes sula|[7076]|06-SRNP-42916|666[0n]bp
Ocyba calathana|[7077]|98-SRNP-6146|612[0n]bp
Ocyba calathana|[7078]|06-SRNP-55791|666[0n]bp
Ocyba calathana|[7079]|06-SRNP-55767|666[0n]bp
Ocyba calathana|[7080]|01-SRNP-12165|594[0n]bp
Ocyba calathana|[7081]|00-SRNP-2597|600[0n]bp
Ocyba calathana|[7082]|06-SRNP-12192|633[1n]bp
Ocyba calathana|[7083]|05-SRNP-64295|669[0n]bp
Ocyba calathana|[7084]|07-SRNP-65680|669[0n]bp
Ocyba calathana|[7085]|08-SRNP-12162|321[0n]bp
Porphyrogenes peterwegei|[7086]|06-SRNP-41790|603[0n]bp
Porphyrogenes peterwegei|[7087]|06-SRNP-59687|594[0n]bp
Porphyrogenes peterwegei|[7088]|08-SRNP-65472|669[0n]bp
Porphyrogenes peterwegei|[7089]|07-SRNP-45285|642[0n]bp
Porphyrogenes peterwegei|[7090]|05-SRNP-2308|642[0n]bp
Porphyrogenes peterwegei|[7091]|05-SRNP-4096|642[0n]bp
Porphyrogenes peterwegei|[7092]|02-SRNP-14654|627[0n]bp
Porphyrogenes peterwegei|[7093]|07-SRNP-65894|666[0n]bp
Porphyrogenes peterwegei|[7094]|06-SRNP-7825|666[0n]bp
Porphyrogenes peterwegei|[7095]|05-SRNP-25294|666[0n]bp
Porphyrogenes peterwegei|[7096]|06-SRNP-31563|666[0n]bp

Porphyrogenes peterwegei|[7094]|06-SRNP-7825|666[0n]bp
Porphyrogenes peterwegei|[7095]|05-SRNP-25294|666[0n]bp
Porphyrogenes peterwegei|[7096]|06-SRNP-31563|666[0n]bp
Porphyrogenes peterwegei|[7097]|06-SRNP-32892|663[0n]bp
Porphyrogenes peterwegei|[7098]|03-SRNP-6133|657[0n]bp
Porphyrogenes peterwegei|[7099]|07-SRNP-23407|669[0n]bp
Porphyrogenes peterwegei|[7100]|06-SRNP-65685|669[0n]bp
Porphyrogenes peterwegei|[7101]|07-SRNP-31916|669[0n]bp
Porphyrogenes peterwegei|[7102]|07-SRNP-30450|669[0n]bp
Porphyrogenes peterwegei|[7103]|05-SRNP-1922|669[0n]bp
Porphyrogenes peterwegei|[7104]|05-SRNP-41224|669[0n]bp
Porphyrogenes peterwegei|[7105]|05-SRNP-2099|669[0n]bp
Porphyrogenes peterwegei|[7106]|05-SRNP-22389|669[0n]bp
Porphyrogenes peterwegei|[7107]|07-SRNP-40389|669[0n]bp
Porphyrogenes peterwegei|[7108]|06-SRNP-22070|669[0n]bp
Porphyrogenes peterwegei|[7109]|06-SRNP-22071|669[0n]bp
Porphyrogenes peterwegei|[7110]|06-SRNP-22084|669[0n]bp
Porphyrogenes peterwegei|[7111]|06-SRNP-33708|669[0n]bp
Porphyrogenes peterwegei|[7112]|06-SRNP-43467|669[0n]bp
Porphyrogenes peterwegei|[7113]|05-SRNP-1925|669[0n]bp
Porphyrogenes peterwegei|[7114]|06-SRNP-45298|669[0n]bp
Porphyrogenes peterwegei|[7115]|06-SRNP-45299|669[0n]bp
Porphyrogenes peterwegei|[7116]|06-SRNP-31354|669[0n]bp
Porphyrogenes peterwegei|[7117]|05-SRNP-43764|669[0n]bp
Porphyrogenes peterwegei|[7118]|04-SRNP-4234|669[0n]bp
Porphyrogenes peterwegei|[7119]|04-SRNP-24187|669[0n]bp
Porphyrogenes peterwegei|[7120]|04-SRNP-4400|669[0n]bp
Porphyrogenes peterwegei|[7121]|04-SRNP-45221|669[0n]bp
Porphyrogenes peterwegei|[7122]|05-SRNP-43653|669[0n]bp
Porphyrogenes peterwegei|[7123]|06-SRNP-22153|669[0n]bp
Porphyrogenes peterwegei|[7124]|03-SRNP-21521|576[1n]bp
Porphyrogenes peterwegei|[7125]|05-SRNP-45183|660[0n]bp
Porphyrogenes peterwegei|[7126]|07-SRNP-41033|660[0n]bp
Porphyrogenes peterwegei|[7127]|07-SRNP-2767|666[0n]bp
Porphyrogenes peterwegei|[7128]|04-SRNP-22615|669[0n]bp
Porphyrogenes peterwegei|[7129]|07-SRNP-42518|669[0n]bp
Porphyrogenes peterwegei|[7130]|08-SRNP-65165|669[0n]bp
Porphyrogenes peterwegei|[7131]|08-SRNP-55407|669[0n]bp
Porphyrogenes peterwegei|[7132]|08-SRNP-2369|669[0n]bp
Porphyrogenes peterwegei|[7133]|08-SRNP-65274|669[0n]bp
Porphyrogenes peterwegei|[7134]|08-SRNP-2549|669[0n]bp
Porphyrogenes peterwegei|[7135]|08-SRNP-2551|669[0n]bp
Porphyrogenes peterwegei|[7136]|08-SRNP-2281|669[0n]bp
Porphyrogenes peterwegei|[7137]|08-SRNP-2554|669[0n]bp
Porphyrogenes peterwegei|[7138]|08-SRNP-2550|669[0n]bp
Porphyrogenes peterwegei|[7139]|08-SRNP-65453|669[0n]bp
Porphyrogenes peterwegei|[7140]|08-SRNP-65190|669[0n]bp
Porphyrogenes peterwegei|[7141]|08-SRNP-5053|669[0n]bp
Porphyrogenes peterwegei|[7142]|08-SRNP-4015|669[0n]bp
Porphyrogenes peterwegei|[7143]|08-SRNP-65797|669[0n]bp
Porphyrogenes peterwegei|[7144]|08-SRNP-5051|669[0n]bp
Porphyrogenes peterwegei|[7145]|09-SRNP-70382|669[0n]bp
Porphyrogenes peterwegei|[7146]|09-SRNP-21376|669[0n]bp
Porphyrogenes peterwegei|[7147]|03-SRNP-20026|624[0n]bp
Porphyrogenes peterwegei|[7148]|03-SRNP-9403|627[0n]bp
Porphyrogenes peterwegei|[7149]|02-SRNP-6580|627[0n]bp
Porphyrogenes peterwegei|[7150]|05-SRNP-42535|666[0n]bp
Porphyrogenes peterwegei|[7151]|06-SRNP-32698|639[0n]bp
Porphyrogenes peterwegei|[7152]|06-SRNP-32697|630[0n]bp
Porphyrogenes peterwegei|[7153]|06-SRNP-22072|633[0n]bp
Porphyrogenes peterwegei|[7154]|03-SRNP-9405|627[0n]bp
Porphyrogenes peterwegei|[7155]|02-SRNP-21541|645[0n]bp
Porphyrogenes peterwegei|[7156]|06-SRNP-22118|669[0n]bp
Porphyrogenes peterwegei|[7157]|07-SRNP-904|669[0n]bp
Porphyrogenes peterwegei|[7158]|05-SRNP-46602|669[0n]bp
Porphyrogenes peterwegei|[7159]|09-SRNP-2624|669[0n]bp
Calliades zeutus|[7160]|05-SRNP-58434|687[0n]bp
Calliades zeutus|[7161]|06-SRNP-19561|669[0n]bp
Calliades zeutus|[7162]|07-SRNP-20366|669[0n]bp
Calliades zeutus|[7163]|06-SRNP-58046|669[0n]bp
Calliades zeutus|[7164]|04-SRNP-49938|669[0n]bp
Calliades zeutus|[7165]|04-SRNP-14586|669[0n]bp
Calliades zeutus|[7166]|04-SRNP-14067|627[0n]bp
Calliades zeutus|[7167]|04-SRNP-49935|627[0n]bp
Calliades zeutus|[7168]|04-SRNP-49393|627[0n]bp
Calliades zeutus|[7169]|05-SRNP-61209|669[1n]bp
Calliades zeutus|[7170]|06-SRNP-19539|669[0n]bp
Calliades zeutus|[7171]|05-SRNP-55236|669[2n]bp
Calliades zeutus|[7172]|02-SRNP-4294|639[0n]bp
Calliades zeutus|[7173]|02-SRNP-4104|576[0n]bp
Calliades zeutus|[7174]|07-SRNP-20861|636[0n]bp
Calliades zeutus|[7175]|07-SRNP-21845|648[0n]bp
Calliades zeutus|[7176]|07-SRNP-21844|669[0n]bp
Proteides mercurius|[7177]|95-SRNP-6422|594[1n]bp
Proteides mercurius|[7178]|05-SRNP-59505|669[1n]bp
Proteides mercurius|[7179]|95-SRNP-4261|597[0n]bp
Proteides mercurius|[7180]|95-SRNP-7319|615[0n]bp
Proteides mercurius|[7181]|02-SRNP-4516|669[1n]bp
Proteides mercurius|[7182]|95-SRNP-74|669[0n]bp
Proteides mercurius|[7183]|00-SRNP-2023|669[1n]bp
Proteides mercurius|[7184]|99-SRNP-6062|657[3n]bp
Proteides mercurius|[7185]|95-SRNP-6413|588[4n]bp
Proteides mercurius|[7186]|95-SRNP-7833|600[1n]bp
Proteides mercurius|[7187]|04-SRNP-2133|627[1n]bp
Proteides mercurius|[7188]|05-SRNP-19609|669[0n]bp
Proteides mercurius|[7189]|04-SRNP-14109|669[0n]bp
Proteides mercurius|[7190]|95-SRNP-6352|669[1n]bp
Proteides mercurius|[7191]|95-SRNP-4269|609[1n]bp
Proteides mercurius|[7192]|06-SRNP-55587|603[0n]bp
Proteides mercurius|[7193]|95-SRNP-6265|627[0n]bp
Proteides mercurius|[7194]|07-SRNP-40797|645[0n]bp
Spathilepia clonius|[7195]|95-SRNP-9249|312[0n]bp
Spathilepia clonius|[7196]|02-SRNP-5756|633[0n]bp

Proteides mercurius|[7194]|07-SRNP-40797|645[0n]bp
Spathilepia clonius|[7195]|95-SRNP-9249|312[0n]bp
Spathilepia clonius|[7196]|02-SRNP-5756|633[0n]bp
Spathilepia clonius|[7197]|07-SRNP-20091|669[0n]bp
Spathilepia clonius|[7198]|05-SRNP-20626|669[0n]bp
Spathilepia clonius|[7199]|07-SRNP-20254|669[0n]bp
Spathilepia clonius|[7200]|04-SRNP-47760|669[0n]bp
Spathilepia clonius|[7201]|04-SRNP-27278|657[0n]bp
Spathilepia clonius|[7202]|05-SRNP-46985|654[2n]bp
Spathilepia clonius|[7203]|07-SRNP-20183|669[1n]bp
Spathilepia clonius|[7204]|01-SRNP-15478|624[0n]bp
Spathilepia clonius|[7205]|07-SRNP-20074|669[0n]bp
Spathilepia clonius|[7206]|07-SRNP-20201|669[0n]bp
Spathilepia clonius|[7207]|07-SRNP-20075|642[0n]bp
Spathilepia clonius|[7208]|09-SRNP-76098|669[0n]bp
Narcosius samson|[7209]|05-SRNP-32509|633[0n]bp
Narcosius samson|[7210]|02-SRNP-3561|618[0n]bp
Narcosius samson|[7211]|02-SRNP-18532|651[0n]bp
Narcosius samson|[7212]|07-SRNP-45154|669[0n]bp
Narcosius samson|[7213]|05-SRNP-21384|669[0n]bp
Narcosius samson|[7214]|04-SRNP-23182|669[0n]bp
Narcosius samson|[7215]|04-SRNP-46044|669[0n]bp
Narcosius samson|[7216]|07-SRNP-2014|660[0n]bp
Narcosius samson|[7217]|07-SRNP-33701|669[0n]bp
Narcosius samson|[7218]|08-SRNP-30598|669[0n]bp
Narcosius samson|[7219]|05-SRNP-22628|627[0n]bp
Narcosius samson|[7220]|06-SRNP-6237|669[0n]bp
Narcosius samson|[7221]|02-SRNP-373|651[0n]bp
Narcosius samson|[7222]|07-SRNP-65943|669[0n]bp
Narcosius samson|[7223]|07-SRNP-65845|669[0n]bp
Narcosius samson|[7224]|08-SRNP-40054|669[0n]bp
Narcosius samson|[7225]|07-SRNP-4271|669[0n]bp
Narcosius samson|[7226]|07-SRNP-4275|669[0n]bp
Narcosius samson|[7227]|07-SRNP-4276|669[0n]bp
Narcosius samson|[7228]|08-SRNP-65210|669[0n]bp
Narcosius samson|[7229]|07-SRNP-41022|669[0n]bp
Narcosius samson|[7230]|07-SRNP-1456|669[0n]bp
Narcosius samson|[7231]|07-SRNP-1341|669[0n]bp
Narcosius samson|[7232]|06-SRNP-33348|669[0n]bp
Narcosius samson|[7233]|04-SRNP-60936|669[0n]bp
Narcosius samson|[7234]|07-SRNP-33230|669[1n]bp
Narcosius samson|[7235]|02-SRNP-1251|657[0n]bp
Narcosius samson|[7236]|07-SRNP-4272|633[0n]bp
Narcosius samson|[7237]|08-SRNP-1747|669[0n]bp
Narcosius colossus|[7238]|07-SRNP-1788|633[0n]bp
Narcosius colossus|[7239]|05-SRNP-3183|669[0n]bp
Narcosius colossus|[7240]|05-SRNP-35254|669[0n]bp
Narcosius colossus|[7241]|04-SRNP-34370|669[0n]bp
Narcosius colossus|[7242]|05-SRNP-35044|669[0n]bp
Narcosius colossus|[7243]|07-SRNP-60189|669[0n]bp
Narcosius colossus|[7244]|07-SRNP-58794|669[0n]bp
Narcosius colossus|[7245]|07-SRNP-1843|639[0n]bp
Narcosius colossus|[7246]|03-SRNP-79|639[0n]bp
Narcosius colossus|[7247]|06-SRNP-4365|669[0n]bp
Narcosius colossus|[7248]|06-SRNP-35026|669[0n]bp
Narcosius colossus|[7249]|06-SRNP-2399|669[0n]bp
Narcosius colossus|[7250]|06-SRNP-12012|669[0n]bp
Narcosius colossus|[7251]|04-SRNP-61447|669[0n]bp
Narcosius colossus|[7252]|04-SRNP-35290|669[0n]bp
Narcosius colossus|[7253]|04-SRNP-35050|669[0n]bp
Narcosius colossus|[7254]|04-SRNP-35268|669[0n]bp
Narcosius colossus|[7255]|04-SRNP-35266|669[0n]bp
Narcosius colossus|[7256]|05-SRNP-35355|384[0n]bp
Narcosius colossus|[7257]|06-SRNP-31983|468[1n]bp
Narcosius colossus|[7258]|02-SRNP-9121|651[0n]bp
Narcosius colossus|[7259]|06-SRNP-36131|600[0n]bp
Narcosius colossus|[7260]|07-SRNP-1842|669[0n]bp
Narcosius colossus|[7261]|07-SRNP-60168|669[0n]bp
Narcosius colossus|[7262]|08-SRNP-1953|669[0n]bp
Narcosius colossus|[7263]|08-SRNP-2685|669[0n]bp
Narcosius colossus|[7264]|08-SRNP-2684|669[0n]bp
Narcosius colossus|[7265]|07-SRNP-57343|627[0n]bp
Narcosius colossus|[7266]|07-SRNP-23367|609[0n]bp
Narcosius colossus|[7267]|04-SRNP-35101|669[0n]bp
Narcosius colossus|[7268]|05-SRNP-2080|669[0n]bp
Narcosius colossus|[7269]|07-SRNP-23315|669[0n]bp
Narcosius colossus|[7270]|08-SRNP-4789|669[0n]bp
Narcosius helen|[7271]|96-SRNP-7367|576[1n]bp
Narcosius helen|[7272]|06-SRNP-59517|669[0n]bp
Narcosius helen|[7273]|06-SRNP-22184|669[0n]bp
Narcosius helen|[7274]|06-SRNP-3211|669[0n]bp
Narcosius helen|[7275]|05-SRNP-65551|630[0n]bp
Narcosius helen|[7276]|06-SRNP-46972|657[0n]bp
Narcosius helen|[7277]|06-SRNP-60063|615[0n]bp
Narcosius helen|[7278]|07-SRNP-57650|636[0n]bp
Narcosius nazaraeus|[7279]|09-SRNP-20099|669[0n]bp
Aguna claxon|[7280]|05-SRNP-41660|669[0n]bp
Aguna claxon|[7281]|05-SRNP-41659|669[0n]bp
Aguna claxon|[7282]|05-SRNP-41925|669[0n]bp
Aguna claxon|[7283]|06-SRNP-42825|669[0n]bp
Aguna claxon|[7284]|07-SRNP-41625|669[0n]bp
Aguna claxon|[7285]|09-SRNP-44598|669[0n]bp
Aguna panama|[7286]|07-SRNP-55426|669[0n]bp
Aguna panama|[7287]|00-SRNP-6020|615[2n]bp
Aguna panama|[7288]|07-SRNP-61073|669[0n]bp
Aguna panama|[7289]|07-SRNP-56460|669[0n]bp
Aguna panama|[7290]|06-SRNP-58291|669[0n]bp
Aguna panama|[7291]|05-SRNP-57368|669[0n]bp
Aguna panama|[7292]|00-SRNP-6052|627[2n]bp
Aguna panama|[7293]|08-SRNP-55747|669[1n]bp
Aguna panama|[7294]|07-SRNP-61072|669[0n]bp
Aguna panama|[7295]|08-SRNP-55705|669[0n]bp
Aguna panama|[7296]|09-SRNP-57341|669[0n]bp

Aguna panama|[7294]|07-SRNP-61072|669[0n]bp
Aguna panama|[7295]|08-SRNP-55705|669[0n]bp
Aguna panama|[7296]|09-SRNP-57341|669[0n]bp
Aguna metophis|[7297]|05-SRNP-41056|669[0n]bp
Aguna asander|[7298]|05-SRNP-56838|669[0n]bp
Aguna asander|[7299]|06-SRNP-56534|669[0n]bp
Aguna asander|[7300]|07-SRNP-56019|669[0n]bp
Aguna asander|[7301]|07-SRNP-56139|669[0n]bp
Aguna asander|[7302]|05-SRNP-56840|669[0n]bp
Aguna asander|[7303]|05-SRNP-56839|669[0n]bp
Aguna asander|[7304]|06-SRNP-56535|660[2n]bp
Aguna asander|[7305]|07-SRNP-56140|660[0n]bp
Aguna asander|[7306]|07-SRNP-56354|669[1n]bp
Aguna asander|[7307]|07-SRNP-57812|624[0n]bp
Aguna asander|[7308]|07-SRNP-56518|669[0n]bp
Aguna asander|[7309]|07-SRNP-56519|669[0n]bp
Aguna asander|[7310]|07-SRNP-56833|669[0n]bp
Aguna asander|[7311]|98-SRNP-3363|639[0n]bp
Aguna asander|[7312]|06-SRNP-56500|669[0n]bp
Aguna asander|[7313]|06-SRNP-56093|669[0n]bp
Aguna asander|[7314]|97-SRNP-2360|576[0n]bp
Aguna asander|[7315]|07-SRNP-56789|648[0n]bp
Aguna asander|[7316]|06-SRNP-56319|624[0n]bp
Aguna asander|[7317]|07-SRNP-56524|642[0n]bp
Aguna asander|[7318]|07-SRNP-56433|669[0n]bp
Aguna asander|[7319]|07-SRNP-57226|642[0n]bp
Aguna asander|[7320]|07-SRNP-56791|642[0n]bp
Aguna arunce hypozonius|[7321]|05-SRNP-57391|669[0n]bp
Aguna arunce hypozonius|[7322]|05-SRNP-57392|669[0n]bp
Aguna arunce hypozonius|[7323]|05-SRNP-57393|669[0n]bp
Aguna arunce hypozonius|[7324]|07-SRNP-56141|666[0n]bp
Aguna arunce hypozonius|[7325]|07-SRNP-58253|645[0n]bp
Aguna Burns01|[7326]|04-SRNP-42390|669[0n]bp
Aguna Burns01|[7327]|03-SRNP-21307|657[0n]bp
Aguna Burns01|[7328]|03-SRNP-21306|621[0n]bp
Aguna Burns01|[7329]|03-SRNP-37032|639[0n]bp
Aguna Burns01|[7330]|05-SRNP-41612|669[0n]bp
Aguna Burns01|[7331]|05-SRNP-70024|669[0n]bp
Aguna Burns01|[7332]|03-SRNP-21373|669[0n]bp
Aguna Burns01|[7333]|03-SRNP-21386|669[0n]bp
Aguna Burns01|[7334]|08-SRNP-71779|669[0n]bp
Aguna Burns01|[7335]|08-SRNP-71112|669[0n]bp
Aguna Burns01|[7336]|08-SRNP-71115|669[0n]bp
Aguna Burns01|[7337]|08-SRNP-71111|669[0n]bp
Aguna coeloides|[7338]|07-SRNP-31814|669[0n]bp
Aguna coeloides|[7339]|02-SRNP-6676|645[0n]bp
Aguna coeloides|[7340]|03-SRNP-20032|645[0n]bp
Aguna coeloides|[7341]|03-SRNP-20033|612[0n]bp
Aguna coeloides|[7342]|02-SRNP-6344|627[0n]bp
Aguna coeloides|[7343]|08-SRNP-71144|669[0n]bp
Aguna coeloides|[7344]|08-SRNP-32489|669[0n]bp
Aguna Burns02|[7345]|03-SRNP-10346|525[1n]bp
Aguna Burns02|[7346]|05-SRNP-43291|669[0n]bp
Aguna Burns02|[7347]|05-SRNP-42263|669[0n]bp
Aguna Burns02|[7348]|05-SRNP-40266|669[0n]bp
Aguna Burns02|[7349]|03-SRNP-10765|669[0n]bp
Aguna Burns02|[7350]|03-SRNP-10241|639[0n]bp
Aguna Burns02|[7351]|03-SRNP-10064|657[0n]bp
Aguna Burns02|[7352]|03-SRNP-31756|645[0n]bp
Aguna Burns02|[7353]|06-SRNP-42285|618[0n]bp
Aguna Burns02|[7354]|06-SRNP-42575|669[0n]bp
Aguna Burns02|[7355]|06-SRNP-42313|669[0n]bp
Aguna Burns02|[7356]|06-SRNP-44328|669[0n]bp
Aguna Burns02|[7357]|06-SRNP-44327|669[0n]bp
Aguna Burns02|[7358]|09-SRNP-69431|669[0n]bp
Aguna Burns02|[7359]|09-SRNP-69465|669[0n]bp
Autochton bipunctatus|[7360]|08-SRNP-30643|669[0n]bp
Autochton Burns01DHJ02|[7361]|04-SRNP-45713|669[0n]bp
Autochton Burns01DHJ02|[7362]|08-SRNP-1103|669[0n]bp
Autochton Burns01DHJ02|[7363]|08-SRNP-1650|669[0n]bp
Autochton Burns01DHJ02|[7364]|08-SRNP-1753|669[0n]bp
Autochton Burns01DHJ02|[7365]|02-SRNP-2645|669[0n]bp
Autochton Burns01DHJ02|[7366]|03-SRNP-19145|669[0n]bp
Autochton Burns01DHJ02|[7367]|06-SRNP-59183|669[0n]bp
Autochton Burns01DHJ02|[7368]|02-SRNP-2824|669[0n]bp
Autochton Burns01DHJ02|[7369]|02-SRNP-2646|669[0n]bp
Autochton Burns01DHJ02|[7370]|02-SRNP-2647|669[0n]bp
Autochton Burns01DHJ02|[7371]|02-SRNP-2644|669[0n]bp
Autochton Burns01DHJ02|[7372]|02-SRNP-2825|669[0n]bp
Autochton Burns01DHJ02|[7373]|02-SRNP-2822|669[0n]bp
Autochton Burns01DHJ02|[7374]|02-SRNP-2821|669[0n]bp
Autochton Burns01DHJ02|[7375]|02-SRNP-6791|669[0n]bp
Autochton Burns01DHJ02|[7376]|02-SRNP-2643|669[0n]bp
Autochton Burns01DHJ02|[7377]|06-SRNP-2611|669[0n]bp
Autochton Burns01DHJ02|[7378]|06-SRNP-1336|669[0n]bp
Autochton Burns01DHJ02|[7379]|04-SRNP-45765|669[0n]bp
Autochton Burns01DHJ02|[7380]|00-SRNP-3690|669[0n]bp
Autochton Burns01DHJ02|[7381]|05-SRNP-46948|630[0n]bp
Autochton Burns01DHJ02|[7382]|01-SRNP-1372|612[0n]bp
Autochton Burns01DHJ02|[7383]|02-SRNP-2823|561[0n]bp
Autochton Burns01DHJ02|[7384]|05-SRNP-59071|636[0n]bp
Autochton Burns01DHJ02|[7385]|06-SRNP-9152|645[1n]bp
Autochton Burns01DHJ02|[7386]|06-SRNP-9303|609[0n]bp
Autochton Burns01DHJ02|[7387]|08-SRNP-1961|645[0n]bp
Autochton Burns01DHJ02|[7388]|08-SRNP-1113|669[0n]bp
Autochton Burns01DHJ02|[7389]|08-SRNP-1014|669[0n]bp
Autochton Burns01DHJ02|[7390]|08-SRNP-1534|669[0n]bp
Autochton Burns01DHJ02|[7391]|08-SRNP-1533|669[0n]bp
Autochton Burns01DHJ02|[7392]|08-SRNP-40551|669[0n]bp
Autochton Burns01DHJ02|[7393]|08-SRNP-749|669[0n]bp
Autochton Burns01DHJ02|[7394]|08-SRNP-718|669[0n]bp
Autochton Burns01DHJ02|[7395]|08-SRNP-1752|669[0n]bp
Autochton Burns01DHJ02|[7396]|08-SRNP-1710|669[0n]bp

Autochton Burns01DHJ02|[7394]|08-SRNP-718|669[0n]bp
Autochton Burns01DHJ02|[7395]|08-SRNP-1752|669[0n]bp
Autochton Burns01DHJ02|[7396]|08-SRNP-1710|669[0n]bp
Autochton Burns01DHJ02|[7397]|08-SRNP-1649|669[0n]bp
Autochton Burns01DHJ02|[7398]|08-SRNP-1532|669[0n]bp
Autochton Burns01DHJ03|[7399]|03-SRNP-21795|645[4n]bp
Autochton Burns01DHJ03|[7400]|05-SRNP-5444|651[7n]bp
Autochton Burns01DHJ04|[7401]|01-SRNP-2361|669[2n]bp
Autochton Burns01DHJ03|[7402]|03-SRNP-8841|660[3n]bp
Autochton Burns01DHJ03|[7403]|02-SRNP-7452|669[1n]bp
Autochton Burns01DHJ04|[7404]|05-SRNP-42409|648[6n]bp
Autochton Burns01DHJ03|[7405]|02-SRNP-7369|666[1n]bp
Autochton Burns01DHJ03|[7406]|02-SRNP-19075|657[2n]bp
Autochton Burns01DHJ03|[7407]|07-SRNP-1204|669[2n]bp
Autochton Burns01DHJ03|[7408]|00-SRNP-11807|669[2n]bp
Autochton Burns01DHJ03|[7409]|01-SRNP-2359|669[1n]bp
Autochton Burns01DHJ03|[7410]|02-SRNP-6764|663[1n]bp
Autochton Burns01DHJ03|[7411]|01-SRNP-2360|657[1n]bp
Autochton Burns01DHJ03|[7412]|01-SRNP-25357|669[0n]bp
Autochton Burns01DHJ03|[7413]|07-SRNP-524|669[1n]bp
Autochton Burns01DHJ03|[7414]|07-SRNP-466|669[1n]bp
Autochton Burns01DHJ03|[7415]|06-SRNP-34485|669[1n]bp
Autochton Burns01DHJ03|[7416]|03-SRNP-9633|660[1n]bp
Autochton Burns01DHJ03|[7417]|06-SRNP-3321|669[2n]bp
Autochton Burns01DHJ03|[7418]|06-SRNP-3415|666[3n]bp
Autochton Burns01DHJ03|[7419]|07-SRNP-40843|621[0n]bp
Autochton Burns01DHJ03|[7420]|03-SRNP-9631|669[1n]bp
Autochton Burns01DHJ03|[7421]|00-SRNP-1531|636[0n]bp
Autochton Burns01DHJ03|[7422]|03-SRNP-21535|645[1n]bp
Autochton Burns01DHJ03|[7423]|02-SRNP-15018|645[1n]bp
Autochton Burns01DHJ03|[7424]|06-SRNP-43554|657[3n]bp
Autochton Burns01DHJ03|[7425]|02-SRNP-15017|669[2n]bp
Autochton Burns01DHJ03|[7426]|03-SRNP-8817|633[3n]bp
Autochton Burns01DHJ03|[7427]|01-SRNP-3734|657[1n]bp
Autochton Burns01DHJ03|[7428]|03-SRNP-9632|645[2n]bp
Autochton Burns01DHJ04|[7429]|03-SRNP-6742|582[2n]bp
Autochton Burns01DHJ04|[7430]|02-SRNP-17340|636[0n]bp
Autochton Burns01DHJ04|[7431]|07-SRNP-76|609[1n]bp
Autochton Burns01DHJ03|[7432]|07-SRNP-834|669[0n]bp
Autochton Burns01DHJ03|[7433]|03-SRNP-21652|669[0n]bp
Autochton Burns01DHJ03|[7434]|03-SRNP-9634|669[0n]bp
Autochton Burns01DHJ03|[7435]|03-SRNP-6163|669[0n]bp
Autochton Burns01DHJ03|[7436]|03-SRNP-21660|669[0n]bp
Autochton Burns01DHJ03|[7437]|06-SRNP-43772|669[0n]bp
Autochton Burns01DHJ03|[7438]|07-SRNP-3126|621[0n]bp
Autochton Burns01DHJ03|[7439]|08-SRNP-1863|669[0n]bp
Autochton Burns01DHJ03|[7440]|08-SRNP-724|669[0n]bp
Autochton Burns01DHJ03|[7441]|01-SRNP-1271|669[2n]bp
Autochton Burns01DHJ03|[7442]|08-SRNP-723|669[5n]bp
Autochton Burns01DHJ04|[7443]|03-SRNP-5976|669[1n]bp
Autochton Burns01DHJ04|[7444]|06-SRNP-40471|669[3n]bp
Autochton Burns01DHJ04|[7445]|02-SRNP-5075|633[3n]bp
Autochton Burns01DHJ05|[7446]|07-SRNP-2033|648[0n]bp
Autochton Burns01DHJ04|[7447]|02-SRNP-17338|582[2n]bp
Autochton Burns01DHJ04|[7448]|08-SRNP-1535|639[5n]bp
Autochton Burns01DHJ04|[7449]|08-SRNP-1648|639[3n]bp
Autochton Burns01DHJ04|[7450]|05-SRNP-4301|687[5n]bp
Autochton Burns01DHJ04|[7451]|05-SRNP-32030|669[1n]bp
Autochton Burns01DHJ04|[7452]|01-SRNP-2650|669[0n]bp
Autochton Burns01DHJ04|[7453]|01-SRNP-2651|624[1n]bp
Autochton Burns01DHJ04|[7454]|01-SRNP-2652|621[3n]bp
Autochton Burns01DHJ04|[7455]|02-SRNP-17339|669[0n]bp
Autochton Burns01DHJ04|[7456]|04-SRNP-60519|669[4n]bp
Autochton Burns01DHJ04|[7457]|01-SRNP-1371|642[3n]bp
Autochton Burns01DHJ04|[7458]|05-SRNP-43435|669[4n]bp
Autochton Burns01DHJ04|[7459]|03-SRNP-8819|669[3n]bp
Autochton Burns01DHJ04|[7460]|03-SRNP-21425|669[3n]bp
Autochton Burns01DHJ04|[7461]|08-SRNP-41353|669[0n]bp
Autochton Burns01DHJ04|[7462]|06-SRNP-43553|669[1n]bp
Autochton Burns01DHJ04|[7463]|03-SRNP-9628|645[1n]bp
Autochton Burns01DHJ04|[7464]|07-SRNP-1157|657[0n]bp
Autochton Burns01DHJ04|[7465]|02-SRNP-30483|669[0n]bp
Autochton Burns01DHJ04|[7466]|02-SRNP-1953|669[0n]bp
Autochton Burns01DHJ04|[7467]|02-SRNP-3878|669[0n]bp
Autochton Burns01DHJ04|[7468]|02-SRNP-28933|669[0n]bp
Autochton Burns01DHJ04|[7469]|03-SRNP-9630|669[0n]bp
Autochton Burns01DHJ04|[7470]|00-SRNP-11621|669[0n]bp
Autochton Burns01DHJ04|[7471]|00-SRNP-12409|669[0n]bp
Autochton Burns01DHJ04|[7472]|01-SRNP-3899|669[0n]bp
Autochton Burns01DHJ04|[7473]|06-SRNP-65526|669[0n]bp
Autochton Burns01DHJ04|[7474]|01-SRNP-25365|669[0n]bp
Autochton Burns01DHJ04|[7475]|02-SRNP-19076|669[0n]bp
Autochton Burns01DHJ04|[7476]|04-SRNP-3794|669[0n]bp
Autochton Burns01DHJ04|[7477]|02-SRNP-2648|669[0n]bp
Autochton Burns01DHJ04|[7478]|01-SRNP-3735|645[0n]bp
Autochton Burns01DHJ04|[7479]|04-SRNP-60826|627[0n]bp
Autochton Burns01DHJ04|[7480]|06-SRNP-65122|666[0n]bp
Autochton Burns01DHJ04|[7481]|06-SRNP-34487|657[1n]bp
Autochton Burns01DHJ04|[7482]|05-SRNP-21742|648[6n]bp
Autochton Burns01DHJ04|[7483]|03-SRNP-21668|639[0n]bp
Autochton Burns01DHJ04|[7484]|03-SRNP-9627|639[1n]bp
Autochton Burns01DHJ04|[7485]|03-SRNP-9626|609[0n]bp
Autochton Burns01DHJ04|[7486]|02-SRNP-19077|645[0n]bp
Autochton Burns01DHJ04|[7487]|08-SRNP-5469|669[0n]bp
Autochton Burns01DHJ05|[7488]|00-SRNP-11187|645[3n]bp
Autochton Burns01DHJ05|[7489]|06-SRNP-2060|669[2n]bp
Autochton Burns01DHJ05|[7490]|01-SRNP-3733|669[1n]bp
Autochton Burns01DHJ05|[7491]|03-SRNP-9837|645[0n]bp
Autochton Burns01DHJ05|[7492]|03-SRNP-21655|669[0n]bp
Autochton Burns01DHJ05|[7493]|03-SRNP-31321|600[0n]bp
Autochton Burns01DHJ05|[7494]|06-SRNP-9700|669[0n]bp
Autochton Burns01DHJ05|[7495]|03-SRNP-13047.1|669[0n]bp
Autochton Burns01DHJ05|[7496]|03-SRNP-10151|645[0n]bp

Autochton Burns01DHJ05|[7494]|06-SRNP-9700|669[0n]bp
Autochton Burns01DHJ05|[7495]|03-SRNP-13047.1|669[0n]bp
Autochton Burns01DHJ05|[7496]|03-SRNP-10151|645[0n]bp
Autochton Burns01DHJ05|[7497]|04-SRNP-22708|669[0n]bp
Autochton Burns01DHJ05|[7498]|04-SRNP-22710|669[0n]bp
Autochton Burns01DHJ05|[7499]|02-SRNP-14078|669[0n]bp
Autochton Burns01DHJ05|[7500]|01-SRNP-3736|669[0n]bp
Autochton Burns01DHJ05|[7501]|01-SRNP-1893|669[0n]bp
Autochton Burns01DHJ05|[7502]|08-SRNP-1358|669[0n]bp
Autochton Burns01DHJ05|[7503]|08-SRNP-6305|669[0n]bp
Cabares potrillo|[7504]|09-SRNP-57001|669[0n]bp
Cabares potrillo|[7505]|06-SRNP-58154|669[0n]bp
Cabares potrillo|[7506]|95-SRNP-6015|351[0n]bp
Cabares potrillo|[7507]|09-SRNP-57000|669[0n]bp
Cabares potrillo|[7508]|09-SRNP-57002|669[0n]bp
Cabares potrillo|[7509]|09-SRNP-57090|669[0n]bp
Mysoria ambigua|[7510]|03-SRNP-1431|645[0n]bp
Mysoria ambigua|[7511]|08-SRNP-24392|669[0n]bp
Mysoria ambigua|[7512]|08-SRNP-55431|669[0n]bp
Mysoria ambigua|[7513]|05-SRNP-57606|669[0n]bp
Mysoria ambigua|[7514]|05-SRNP-55647|669[0n]bp
Mysoria ambigua|[7515]|04-SRNP-23872|669[0n]bp
Mysoria ambigua|[7516]|04-SRNP-14802|669[0n]bp
Mysoria ambigua|[7517]|04-SRNP-14797|669[0n]bp
Mysoria ambigua|[7518]|04-SRNP-14796|669[0n]bp
Mysoria ambigua|[7519]|04-SRNP-14795|669[0n]bp
Mysoria ambigua|[7520]|04-SRNP-14800|669[0n]bp
Mysoria ambigua|[7521]|04-SRNP-14801|669[0n]bp
Mysoria ambigua|[7522]|05-SRNP-55178|669[0n]bp
Mysoria ambigua|[7523]|04-SRNP-14558|669[0n]bp
Mysoria ambigua|[7524]|04-SRNP-14099|669[0n]bp
Mysoria ambigua|[7525]|05-SRNP-55193|669[0n]bp
Mysoria ambigua|[7526]|05-SRNP-55175|669[0n]bp
Mysoria ambigua|[7527]|08-SRNP-55530|669[0n]bp
Mysoria ambigua|[7528]|03-SRNP-1293|657[0n]bp
Mysoria ambigua|[7529]|04-SRNP-14584|627[0n]bp
Mysoria ambigua|[7530]|08-SRNP-24269|627[1n]bp
Jemadia Burns01|[7531]|01-SRNP-9029|567[1n]bp
Jemadia Burns01|[7532]|04-SRNP-30754|669[0n]bp
Jemadia Burns01|[7533]|03-SRNP-21823|669[0n]bp
Jemadia Burns01|[7534]|03-SRNP-21528|669[0n]bp
Jemadia Burns01|[7535]|04-SRNP-32358|669[0n]bp
Jemadia Burns01|[7536]|04-SRNP-34396|669[0n]bp
Jemadia Burns01|[7537]|05-SRNP-31969|669[0n]bp
Jemadia Burns01|[7538]|00-SRNP-4482|630[0n]bp
Jemadia Burns01|[7539]|04-SRNP-56811|669[0n]bp
Jemadia Burns01|[7540]|05-SRNP-31086|669[0n]bp
Jemadia Burns01|[7541]|96-SRNP-12846|618[0n]bp
Jemadia Burns01|[7542]|02-SRNP-13059|645[0n]bp
Jemadia Burns01|[7543]|09-SRNP-30034|642[0n]bp
Elbella scylla|[7544]|04-SRNP-13405|669[0n]bp
Elbella scylla|[7545]|07-SRNP-32122|669[0n]bp
Elbella scylla|[7546]|07-SRNP-65187|669[0n]bp
Elbella scylla|[7547]|08-SRNP-70068|669[0n]bp
Elbella scylla|[7548]|08-SRNP-70229|669[0n]bp
Elbella scylla|[7549]|05-SRNP-31078|669[0n]bp
Elbella scylla|[7550]|05-SRNP-12075|669[0n]bp
Elbella scylla|[7551]|05-SRNP-31568|669[0n]bp
Elbella scylla|[7552]|08-SRNP-72172|669[0n]bp
Elbella scylla|[7553]|08-SRNP-70307|669[0n]bp
Elbella scylla|[7554]|08-SRNP-55460|669[0n]bp
Elbella scylla|[7555]|08-SRNP-55401|669[0n]bp
Elbella scylla|[7556]|08-SRNP-55397|669[0n]bp
Elbella scylla|[7557]|08-SRNP-70314|669[0n]bp
Elbella scylla|[7558]|06-SRNP-67774|669[0n]bp
Elbella scylla|[7559]|05-SRNP-31077|669[0n]bp
Elbella scylla|[7560]|05-SRNP-55736|669[0n]bp
Elbella scylla|[7561]|05-SRNP-13170|669[0n]bp
Elbella scylla|[7562]|04-SRNP-13706|669[0n]bp
Elbella scylla|[7563]|04-SRNP-13406|669[0n]bp
Elbella scylla|[7564]|04-SRNP-13082|669[0n]bp
Elbella scylla|[7565]|04-SRNP-13081|669[0n]bp
Elbella scylla|[7566]|04-SRNP-21136|669[0n]bp
Elbella scylla|[7567]|07-SRNP-21861|666[0n]bp
Elbella scylla|[7568]|07-SRNP-57964|657[0n]bp
Elbella scylla|[7569]|03-SRNP-232|573[0n]bp
Elbella scylla|[7570]|07-SRNP-65934|648[0n]bp
Elbella scylla|[7571]|08-SRNP-70185|645[1n]bp
Elbella scylla|[7572]|03-SRNP-635|642[0n]bp
Elbella scylla|[7573]|08-SRNP-23853|612[0n]bp
Elbella patrobas|[7574]|07-SRNP-31402|669[0n]bp
Elbella patrobas|[7575]|06-SRNP-33024|669[0n]bp
Elbella patrobas|[7576]|03-SRNP-6393|639[0n]bp
Elbella patrobas|[7577]|07-SRNP-2792|669[1n]bp
Elbella patrobas|[7578]|07-SRNP-4287|669[0n]bp
Elbella patrobas|[7579]|08-SRNP-2588|669[0n]bp
Elbella patrobasDHJ05|[7580]|07-SRNP-3722|669[0n]bp
Elbella merops|[7581]|05-SRNP-32782|669[0n]bp
Elbella merops|[7582]|08-SRNP-70940|669[0n]bp
Elbella merops|[7583]|05-SRNP-41519|669[0n]bp
Elbella merops|[7584]|09-SRNP-30538|633[0n]bp
Elbella merops|[7585]|09-SRNP-71893|669[0n]bp
Jemadia pseudognetus|[7586]|01-SRNP-1773|528[1n]bp
Jemadia pseudognetus|[7587]|02-SRNP-3211|576[0n]bp
Jemadia pseudognetus|[7588]|08-SRNP-4381|669[0n]bp
Jemadia pseudognetus|[7589]|08-SRNP-639|669[0n]bp
Jemadia pseudognetus|[7590]|07-SRNP-65061|669[0n]bp
Jemadia pseudognetus|[7591]|07-SRNP-45220|669[0n]bp
Jemadia pseudognetus|[7592]|07-SRNP-2561|669[0n]bp
Jemadia pseudognetus|[7593]|07-SRNP-1947|669[0n]bp
Jemadia pseudognetus|[7594]|07-SRNP-2837|669[0n]bp
Jemadia pseudognetus|[7595]|07-SRNP-3517|669[0n]bp
Jemadia pseudognetus|[7596]|05-SRNP-7825|669[0n]bp

Jemadia pseudognetus|[7594]|07-SRNP-2837|669[0n]bp
Jemadia pseudognetus|[7595]|07-SRNP-3517|669[0n]bp
Jemadia pseudognetus|[7596]|05-SRNP-7825|669[0n]bp
Jemadia pseudognetus|[7597]|05-SRNP-4394|669[0n]bp
Jemadia pseudognetus|[7598]|05-SRNP-2922|669[0n]bp
Jemadia pseudognetus|[7599]|05-SRNP-3192|669[0n]bp
Jemadia pseudognetus|[7600]|05-SRNP-4069|669[0n]bp
Jemadia pseudognetus|[7601]|04-SRNP-4113|669[0n]bp
Jemadia pseudognetus|[7602]|06-SRNP-1088|669[1n]bp
Jemadia pseudognetus|[7603]|05-SRNP-4395|669[0n]bp
Jemadia pseudognetus|[7604]|02-SRNP-18659|627[0n]bp
Jemadia pseudognetus|[7605]|02-SRNP-18802|627[0n]bp
Jemadia pseudognetus|[7606]|04-SRNP-26478|561[1n]bp
Jemadia pseudognetus|[7607]|06-SRNP-67850|633[0n]bp
Jemadia pseudognetus|[7608]|07-SRNP-2602|645[0n]bp
Jemadia pseudognetus|[7609]|08-SRNP-289|654[0n]bp
Jemadia pseudognetus|[7610]|01-SRNP-2420|654[0n]bp
Jemadia pseudognetus|[7611]|02-SRNP-1552|576[0n]bp
Jemadia pseudognetus|[7612]|08-SRNP-65423|669[0n]bp
Parelbella macleannani|[7613]|06-SRNP-8158|669[0n]bp
Parelbella macleannani|[7614]|04-SRNP-24065|669[0n]bp
Parelbella macleannani|[7615]|04-SRNP-23689|669[0n]bp
Parelbella macleannani|[7616]|04-SRNP-21620|669[0n]bp
Parelbella macleannani|[7617]|04-SRNP-23317|669[0n]bp
Parelbella macleannani|[7618]|04-SRNP-23795|669[0n]bp
Parelbella macleannani|[7619]|04-SRNP-23300|669[0n]bp
Parelbella macleannani|[7620]|01-SRNP-163|534[3n]bp
Parelbella macleannani|[7621]|00-SRNP-571|597[0n]bp
Parelbella macleannani|[7622]|07-SRNP-19|660[0n]bp
Parelbella macleannani|[7623]|07-SRNP-425|669[0n]bp
Parelbella macleannani|[7624]|07-SRNP-20933|669[0n]bp
Parelbella macleannani|[7625]|06-SRNP-23440|669[0n]bp
Parelbella macleannani|[7626]|07-SRNP-2509|669[0n]bp
Parelbella macleannani|[7627]|07-SRNP-3362|669[0n]bp
Parelbella macleannani|[7628]|07-SRNP-2510|669[0n]bp
Parelbella macleannani|[7629]|07-SRNP-23463|669[0n]bp
Parelbella macleannani|[7630]|07-SRNP-22879|669[0n]bp
Parelbella macleannani|[7631]|09-SRNP-20914|669[0n]bp
Yanguna cosyra|[7632]|03-SRNP-5253|567[1n]bp
Yanguna cosyra|[7633]|03-SRNP-3068|663[0n]bp
Yanguna cosyra|[7634]|04-SRNP-35097|669[0n]bp
Yanguna cosyra|[7635]|04-SRNP-35058|669[0n]bp
Yanguna cosyra|[7636]|04-SRNP-35285|669[0n]bp
Yanguna cosyra|[7637]|04-SRNP-35120|669[0n]bp
Yanguna cosyra|[7638]|04-SRNP-35115|669[0n]bp
Yanguna cosyra|[7639]|04-SRNP-35110|669[0n]bp
Yanguna cosyra|[7640]|04-SRNP-35116|669[0n]bp
Yanguna cosyra|[7641]|04-SRNP-35122|669[0n]bp
Yanguna cosyra|[7642]|05-SRNP-35027|669[0n]bp
Yanguna cosyra|[7643]|05-SRNP-35042|669[0n]bp
Yanguna cosyra|[7644]|05-SRNP-35054|669[0n]bp
Yanguna cosyra|[7645]|06-SRNP-65100|669[0n]bp
Yanguna cosyra|[7646]|07-SRNP-41792|669[0n]bp
Yanguna cosyra|[7647]|07-SRNP-2192|669[0n]bp
Yanguna cosyra|[7648]|07-SRNP-2272|669[0n]bp
Yanguna cosyra|[7649]|07-SRNP-41479|669[0n]bp
Yanguna cosyra|[7650]|07-SRNP-41979|669[0n]bp
Yanguna cosyra|[7651]|07-SRNP-36506|669[0n]bp
Yanguna cosyra|[7652]|07-SRNP-35955|669[0n]bp
Yanguna cosyra|[7653]|08-SRNP-35914|669[0n]bp
Yanguna cosyra|[7654]|08-SRNP-31712|669[0n]bp
Jonaspyge aesculapus|[7655]|03-SRNP-3336|669[0n]bp
Jonaspyge aesculapus|[7656]|04-SRNP-35065|669[0n]bp
Jonaspyge aesculapus|[7657]|98-SRNP-2286|633[0n]bp
Jonaspyge aesculapus|[7658]|98-SRNP-2048.1|630[0n]bp
Jonaspyge aesculapus|[7659]|97-SRNP-514|639[0n]bp
Jonaspyge aesculapus|[7660]|04-SRNP-35064|576[0n]bp
Jonaspyge aesculapus|[7661]|04-SRNP-35130|669[0n]bp
Jonaspyge aesculapus|[7662]|05-SRNP-35055|669[0n]bp
Jonaspyge aesculapus|[7663]|02-SRNP-23111|657[0n]bp
Jonaspyge aesculapus|[7664]|04-SRNP-35066|630[0n]bp
Jonaspyge aesculapus|[7665]|97-SRNP-2105|621[0n]bp
Jonaspyge aesculapus|[7666]|00-SRNP-9360|630[0n]bp
Creonpyge creon|[7667]|06-SRNP-36175|669[0n]bp
Creonpyge creon|[7668]|02-SRNP-8028|669[0n]bp
Creonpyge creon|[7669]|01-SRNP-7147|669[0n]bp
Creonpyge creon|[7670]|01-SRNP-7399|669[0n]bp
Creonpyge creon|[7671]|02-SRNP-9441|669[0n]bp
Creonpyge creon|[7672]|03-SRNP-3109|669[0n]bp
Creonpyge creon|[7673]|98-SRNP-2317|633[0n]bp
Creonpyge creon|[7674]|03-SRNP-3108|657[0n]bp
Creonpyge creon|[7675]|02-SRNP-8026|660[0n]bp
Creonpyge creon|[7676]|06-SRNP-36176|669[0n]bp
Melanopyge erythrosticta|[7677]|03-SRNP-5457|645[0n]bp
Melanopyge erythrosticta|[7678]|00-SRNP-15831|663[0n]bp
Melanopyge erythrosticta|[7679]|04-SRNP-34289|669[0n]bp
Melanopyge erythrosticta|[7680]|05-SRNP-894|669[0n]bp
Melanopyge Burns01|[7681]|07-SRNP-992|669[0n]bp
Melanopyge Burns01|[7682]|09-SRNP-722|669[0n]bp
Melanopyge Burns01|[7683]|05-SRNP-34170|669[0n]bp
Melanopyge Burns01|[7684]|07-SRNP-2732|669[0n]bp
Melanopyge Burns01|[7685]|05-SRNP-3132|669[0n]bp
Melanopyge Burns01|[7686]|05-SRNP-309|669[0n]bp
Melanopyge Burns01|[7687]|04-SRNP-3561|669[0n]bp
Melanopyge Burns01|[7688]|04-SRNP-3562|669[0n]bp
Melanopyge Burns01|[7689]|04-SRNP-4008|669[0n]bp
Melanopyge Burns01|[7690]|04-SRNP-3996|669[0n]bp
Melanopyge Burns01|[7691]|04-SRNP-3563|669[0n]bp
Melanopyge Burns01|[7692]|07-SRNP-65400|642[0n]bp
Melanopyge Burns01|[7693]|02-SRNP-14905|642[0n]bp
Melanopyge Burns01|[7694]|05-SRNP-34206|630[0n]bp
Melanopyge Burns01|[7695]|08-SRNP-71628|639[0n]bp
Melanopyge Burns01|[7696]|09-SRNP-65504|657[0n]bp

Melanopyge Burns01|[7694]|05-SRNP-34206|630[0n]bp
Melanopyge Burns01|[7695]|08-SRNP-71628|639[0n]bp
Melanopyge Burns01|[7696]|09-SRNP-65504|657[0n]bp
Pyrrhopyge zenodorusDHJ01|[7697]|04-SRNP-41678|669[0n]bp
Pyrrhopyge zenodorusDHJ01|[7698]|00-SRNP-1645|669[0n]bp
Pyrrhopyge zenodorusDHJ01|[7699]|01-SRNP-9155|669[0n]bp
Pyrrhopyge zenodorusDHJ01|[7700]|06-SRNP-1697|669[0n]bp
Pyrrhopyge zenodorusDHJ01|[7701]|06-SRNP-42909|669[0n]bp
Pyrrhopyge zenodorusDHJ01|[7702]|06-SRNP-4369|669[0n]bp
Pyrrhopyge zenodorusDHJ03|[7703]|05-SRNP-59692|669[1n]bp
Pyrrhopyge zenodorusDHJ03|[7704]|06-SRNP-60372|669[0n]bp
Pyrrhopyge zenodorusDHJ03|[7705]|05-SRNP-66260|603[1n]bp
Pyrrhopyge zenodorusDHJ03|[7706]|05-SRNP-66171|573[1n]bp
Pyrrhopyge zenodorusDHJ03|[7707]|99-SRNP-5161|669[0n]bp
Pyrrhopyge zenodorusDHJ03|[7708]|07-SRNP-3197|669[0n]bp
Pyrrhopyge zenodorusDHJ03|[7709]|08-SRNP-70278|669[0n]bp
Pyrrhopyge zenodorusDHJ03|[7710]|08-SRNP-70280|669[0n]bp
Pyrrhopyge zenodorusDHJ02|[7711]|08-SRNP-72317|549[0n]bp
Pyrrhopyge zenodorusDHJ02|[7712]|00-SRNP-20566|609[0n]bp
Pyrrhopyge zenodorusDHJ02|[7713]|07-SRNP-65619|669[0n]bp
Pyrrhopyge zenodorusDHJ02|[7714]|07-SRNP-45223|669[0n]bp
Pyrrhopyge zenodorusDHJ02|[7715]|06-SRNP-9502|669[0n]bp
Pyrrhopyge zenodorusDHJ02|[7716]|06-SRNP-46215|669[0n]bp
Pyrrhopyge zenodorusDHJ02|[7717]|06-SRNP-6822|669[0n]bp
Pyrrhopyge zenodorusDHJ02|[7718]|06-SRNP-7823|669[0n]bp
Pyrrhopyge zenodorusDHJ02|[7719]|06-SRNP-20133|669[0n]bp
Pyrrhopyge zenodorusDHJ02|[7720]|99-SRNP-12050|669[0n]bp
Pyrrhopyge zenodorusDHJ02|[7721]|99-SRNP-5532|669[0n]bp
Pyrrhopyge zenodorusDHJ02|[7722]|02-SRNP-14950|669[0n]bp
Pyrrhopyge zenodorusDHJ02|[7723]|01-SRNP-1501|669[0n]bp
Pyrrhopyge zenodorusDHJ02|[7724]|00-SRNP-14177|669[0n]bp
Pyrrhopyge zenodorusDHJ02|[7725]|00-SRNP-2007|669[0n]bp
Pyrrhopyge zenodorusDHJ02|[7726]|00-SRNP-22185|669[0n]bp
Pyrrhopyge zenodorusDHJ02|[7727]|00-SRNP-14143|669[0n]bp
Pyrrhopyge zenodorusDHJ02|[7728]|00-SRNP-11309|669[0n]bp
Pyrrhopyge zenodorusDHJ02|[7729]|00-SRNP-2010|669[0n]bp
Pyrrhopyge zenodorusDHJ02|[7730]|02-SRNP-23595|669[0n]bp
Pyrrhopyge zenodorusDHJ02|[7731]|02-SRNP-14179|669[0n]bp
Pyrrhopyge zenodorusDHJ02|[7732]|01-SRNP-4744|669[0n]bp
Pyrrhopyge zenodorusDHJ02|[7733]|03-SRNP-1421|669[0n]bp
Pyrrhopyge zenodorusDHJ02|[7734]|05-SRNP-49652|669[0n]bp
Pyrrhopyge zenodorusDHJ02|[7735]|06-SRNP-116|669[0n]bp
Pyrrhopyge zenodorusDHJ02|[7736]|05-SRNP-59691|669[0n]bp
Pyrrhopyge zenodorusDHJ02|[7737]|04-SRNP-47942|669[0n]bp
Pyrrhopyge zenodorusDHJ02|[7738]|04-SRNP-47938|669[0n]bp
Pyrrhopyge zenodorusDHJ02|[7739]|04-SRNP-48199|669[0n]bp
Pyrrhopyge zenodorusDHJ02|[7740]|04-SRNP-47940|669[0n]bp
Pyrrhopyge zenodorusDHJ02|[7741]|02-SRNP-15035|666[0n]bp
Pyrrhopyge zenodorusDHJ02|[7742]|07-SRNP-45520|669[0n]bp
Pyrrhopyge zenodorusDHJ02|[7743]|07-SRNP-45519|669[0n]bp
Pyrrhopyge zenodorusDHJ02|[7744]|06-SRNP-65140|663[0n]bp
Pyrrhopyge zenodorusDHJ02|[7745]|03-SRNP-1418|624[0n]bp
Pyrrhopyge zenodorusDHJ02|[7746]|03-SRNP-5466|630[0n]bp
Pyrrhopyge zenodorusDHJ02|[7747]|00-SRNP-14681|618[0n]bp
Pyrrhopyge zenodorusDHJ02|[7748]|06-SRNP-44426|597[0n]bp
Pyrrhopyge zenodorusDHJ02|[7749]|08-SRNP-65047|606[0n]bp
Pyrrhopyge zenodorusDHJ02|[7750]|08-SRNP-70328|669[0n]bp
Pyrrhopyge zenodorusDHJ02|[7751]|08-SRNP-2416|669[0n]bp
Pyrrhopyge zenodorusDHJ02|[7752]|09-SRNP-69426|669[0n]bp
Pyrrhopyge zenodorusDHJ02|[7753]|09-SRNP-71854|669[0n]bp
Pyrrhopyge zenodorusDHJ02|[7754]|09-SRNP-71853|669[0n]bp
Pyrrhopyge zenodorusDHJ02|[7755]|09-SRNP-41493|669[0n]bp
Pyrrhopyge crida|[7756]|07-SRNP-65395|633[0n]bp
Pyrrhopyge crida|[7757]|07-SRNP-65251|660[0n]bp
Pyrrhopyge crida|[7758]|07-SRNP-65439|660[0n]bp
Pyrrhopyge crida|[7759]|07-SRNP-65440|669[0n]bp
Pyrrhopyge crida|[7760]|07-SRNP-65431|669[0n]bp
Pyrrhopyge crida|[7761]|07-SRNP-65427|669[0n]bp
Pyrrhopyge crida|[7762]|07-SRNP-65438|669[0n]bp
Pyrrhopyge crida|[7763]|07-SRNP-65517|669[0n]bp
Pyrrhopyge crida|[7764]|07-SRNP-65248|669[0n]bp
Pyrrhopyge crida|[7765]|07-SRNP-65186|669[0n]bp
Pyrrhopyge crida|[7766]|07-SRNP-21159|669[0n]bp
Pyrrhopyge crida|[7767]|07-SRNP-65432|669[0n]bp
Pyrrhopyge crida|[7768]|05-SRNP-23087|669[0n]bp
Pyrrhopyge crida|[7769]|05-SRNP-20774|669[0n]bp
Pyrrhopyge crida|[7770]|05-SRNP-20610|669[0n]bp
Pyrrhopyge crida|[7771]|05-SRNP-20173|669[0n]bp
Pyrrhopyge crida|[7772]|04-SRNP-32575|669[0n]bp
Pyrrhopyge crida|[7773]|04-SRNP-34740|669[0n]bp
Pyrrhopyge crida|[7774]|04-SRNP-34738|669[0n]bp
Pyrrhopyge crida|[7775]|04-SRNP-34984|669[0n]bp
Pyrrhopyge crida|[7776]|04-SRNP-33852|669[0n]bp
Pyrrhopyge crida|[7777]|04-SRNP-34744|669[0n]bp
Pyrrhopyge crida|[7778]|04-SRNP-34924|669[0n]bp
Pyrrhopyge crida|[7779]|05-SRNP-20692|669[0n]bp
Pyrrhopyge crida|[7780]|05-SRNP-20180|669[0n]bp
Pyrrhopyge crida|[7781]|05-SRNP-20171|669[0n]bp
Pyrrhopyge crida|[7782]|05-SRNP-20775|669[0n]bp
Pyrrhopyge crida|[7783]|04-SRNP-34275|669[0n]bp
Pyrrhopyge crida|[7784]|04-SRNP-32812|669[0n]bp
Pyrrhopyge crida|[7785]|04-SRNP-32576|669[0n]bp
Pyrrhopyge crida|[7786]|03-SRNP-21852|669[0n]bp
Pyrrhopyge crida|[7787]|03-SRNP-21862|669[0n]bp
Pyrrhopyge crida|[7788]|04-SRNP-30780|669[0n]bp
Pyrrhopyge crida|[7789]|03-SRNP-21855|669[0n]bp
Pyrrhopyge crida|[7790]|03-SRNP-21853|669[0n]bp
Pyrrhopyge crida|[7791]|05-SRNP-20952|651[0n]bp
Pyrrhopyge crida|[7792]|07-SRNP-65585|666[0n]bp
Pyrrhopyge crida|[7793]|07-SRNP-65426|666[0n]bp
Pyrrhopyge crida|[7794]|03-SRNP-1149|642[0n]bp
Pyrrhopyge crida|[7795]|01-SRNP-9130|624[0n]bp
Pyrrhopyge crida|[7796]|07-SRNP-65584|621[0n]bp

Pyrrhopyge crida|[7794]|03-SRNP-1149|642[0n]bp
Pyrrhopyge crida|[7795]|01-SRNP-9130|624[0n]bp
Pyrrhopyge crida|[7796]|07-SRNP-65584|621[0n]bp
Pyrrhopyge crida|[7797]|07-SRNP-66213|648[0n]bp
Pyrrhopyge crida|[7798]|03-SRNP-21536|564[2n]bp
Pyrrhopyge crida|[7799]|03-SRNP-21486|603[1n]bp
Pyrrhopyge crida|[7800]|05-SRNP-20964|585[0n]bp
Pyrrhopyge crida|[7801]|08-SRNP-65031|603[0n]bp
Pyrrhopyge crida|[7802]|07-SRNP-65757|669[0n]bp
Pyrrhopyge crida|[7803]|07-SRNP-65984|669[0n]bp
Pyrrhopyge crida|[7804]|09-SRNP-20790|669[0n]bp
Myscelus amystis|[7805]|96-SRNP-365|537[3n]bp
Myscelus amystis|[7806]|05-SRNP-20512|669[1n]bp
Myscelus amystis|[7807]|04-SRNP-26567|687[0n]bp
Myscelus amystis|[7808]|05-SRNP-20288|669[0n]bp
Myscelus amystis|[7809]|05-SRNP-20428|669[0n]bp
Myscelus amystis|[7810]|05-SRNP-20589|669[0n]bp
Myscelus amystis|[7811]|05-SRNP-20514|669[0n]bp
Myscelus amystis|[7812]|04-SRNP-24102|669[0n]bp
Myscelus amystis|[7813]|04-SRNP-21290|669[0n]bp
Myscelus amystis|[7814]|00-SRNP-6023|669[0n]bp
Myscelus amystis|[7815]|00-SRNP-20049|594[0n]bp
Myscelus amystis|[7816]|05-SRNP-20426|375[2n]bp
Myscelus amystis|[7817]|05-SRNP-20706|669[0n]bp
Myscelus amystis|[7818]|04-SRNP-26566|669[0n]bp
Myscelus amystis|[7819]|05-SRNP-20582|669[0n]bp
Passova gellias|[7820]|06-SRNP-47761|609[0n]bp
Passova gellias|[7821]|08-SRNP-5050|669[0n]bp
Passova gellias|[7822]|98-SRNP-2698|639[0n]bp
Passova gellias|[7823]|02-SRNP-33556|666[0n]bp
Passova gellias|[7824]|03-SRNP-4222|666[0n]bp
Passova gellias|[7825]|01-SRNP-3818|666[0n]bp
Passova gellias|[7826]|08-SRNP-5586|669[0n]bp
Passova gellias|[7827]|08-SRNP-5267|669[0n]bp
Passova gellias|[7828]|07-SRNP-23493|669[0n]bp
Passova gellias|[7829]|07-SRNP-65612|669[0n]bp
Passova gellias|[7830]|07-SRNP-2695|669[0n]bp
Passova gellias|[7831]|07-SRNP-65778|669[0n]bp
Passova gellias|[7832]|02-SRNP-33544|669[0n]bp
Passova gellias|[7833]|02-SRNP-18208|669[0n]bp
Passova gellias|[7834]|03-SRNP-25876|669[0n]bp
Passova gellias|[7835]|03-SRNP-34742|669[0n]bp
Passova gellias|[7836]|02-SRNP-18690|669[0n]bp
Passova gellias|[7837]|02-SRNP-18842|669[0n]bp
Passova gellias|[7838]|04-SRNP-35968|669[0n]bp
Passova gellias|[7839]|04-SRNP-33962|669[0n]bp
Passova gellias|[7840]|04-SRNP-46731|669[0n]bp
Passova gellias|[7841]|04-SRNP-2750|669[0n]bp
Passova gellias|[7842]|02-SRNP-18968|669[0n]bp
Passova gellias|[7843]|07-SRNP-501|660[0n]bp
Passova gellias|[7844]|02-SRNP-903|660[0n]bp
Passova gellias|[7845]|04-SRNP-4530|648[0n]bp
Passova gellias|[7846]|02-SRNP-18863|528[0n]bp
Passova gellias|[7847]|08-SRNP-348|582[0n]bp
Passova gellias|[7848]|09-SRNP-881|636[0n]bp
Passova gellias|[7849]|08-SRNP-5268|669[0n]bp
Passova gellias|[7850]|09-SRNP-57397|669[0n]bp
Myscelus assaricus michaeli|[7851]|04-SRNP-1946|669[0n]bp
Myscelus assaricus michaeli|[7852]|02-SRNP-33561|669[0n]bp
Myscelus assaricus michaeli|[7853]|02-SRNP-33559|669[0n]bp
Myscelus assaricus michaeli|[7854]|02-SRNP-33574|669[0n]bp
Myscelus assaricus michaeli|[7855]|04-SRNP-2100|669[0n]bp
Myscelus assaricus michaeli|[7856]|04-SRNP-2099|669[0n]bp
Myscelus assaricus michaeli|[7857]|03-SRNP-6544|657[0n]bp
Myscelus assaricus michaeli|[7858]|03-SRNP-6120|633[0n]bp
Myscelus assaricus michaeli|[7859]|02-SRNP-33572|660[0n]bp
Myscelus assaricus michaeli|[7860]|02-SRNP-33568|660[0n]bp
Myscelus assaricus michaeli|[7861]|03-SRNP-5954|669[0n]bp
Myscelus assaricus michaeli|[7862]|08-SRNP-1740|669[0n]bp
Myscelus assaricus michaeli|[7863]|08-SRNP-65643|669[0n]bp
Myscelus perissodora|[7864]|03-SRNP-6256|654[0n]bp
Myscelus perissodora|[7865]|99-SRNP-2587|657[0n]bp
Myscelus belti|[7866]|07-SRNP-66198|666[0n]bp
Myscelus belti|[7867]|07-SRNP-1252|669[0n]bp
Myscelus belti|[7868]|07-SRNP-3251|669[0n]bp
Myscelus belti|[7869]|08-SRNP-1651|669[0n]bp
Myscelus belti|[7870]|08-SRNP-72474|669[0n]bp
Myscelus belti|[7871]|07-SRNP-3252|669[0n]bp
Myscelus belti|[7872]|07-SRNP-36505|669[0n]bp
Myscelus belti|[7873]|09-SRNP-20103|669[0n]bp
Myscelus belti|[7874]|09-SRNP-20720|669[0n]bp
Myscelus belti|[7875]|09-SRNP-4463|669[0n]bp
Myscelus belti|[7876]|09-SRNP-4464|669[0n]bp
Myscelus belti|[7877]|07-SRNP-32809|669[0n]bp
Myscelus belti|[7878]|05-SRNP-2091|669[0n]bp
Myscelus belti|[7879]|04-SRNP-34960|669[0n]bp
Myscelus belti|[7880]|04-SRNP-3418|669[0n]bp
Myscelus belti|[7881]|04-SRNP-23919|669[0n]bp
Myscelus belti|[7882]|04-SRNP-23918|669[0n]bp
Myscelus belti|[7883]|04-SRNP-31804|669[0n]bp
Myscelus belti|[7884]|04-SRNP-33998|669[0n]bp
Myscelus belti|[7885]|04-SRNP-35333|669[0n]bp
Myscelus belti|[7886]|04-SRNP-35100|669[0n]bp
Myscelus belti|[7887]|04-SRNP-35393|669[0n]bp
Myscelus belti|[7888]|07-SRNP-65976|669[1n]bp
Myscelus belti|[7889]|07-SRNP-3391|660[0n]bp
Myscelus belti|[7890]|03-SRNP-6307|645[0n]bp
Myscelus belti|[7891]|07-SRNP-65974|654[0n]bp
Myscelus belti|[7892]|03-SRNP-3151|630[0n]bp
Myscelus belti|[7893]|07-SRNP-66043|642[0n]bp
Myscelus belti|[7894]|09-SRNP-69008|642[0n]bp
Myscelus belti|[7895]|09-SRNP-20102|669[0n]bp
Oxynetra hopfferi|[7896]|02-SRNP-23286|630[0n]bp

Myscelus belti|[7894]|09-SRNP-69008|642[0n]bp
Myscelus belti|[7895]|09-SRNP-20102|669[0n]bp
Oxynetra hopfferi|[7896]|02-SRNP-23286|630[0n]bp
Oxynetra hopfferi|[7897]|02-SRNP-23284|645[0n]bp
Oxynetra hopfferi|[7898]|01-SRNP-6995|669[0n]bp
Oxynetra hopfferi|[7899]|02-SRNP-23283|669[0n]bp
Oxynetra hopfferi|[7900]|02-SRNP-23540|669[0n]bp
Oxynetra hopfferi|[7901]|02-SRNP-24529|669[0n]bp
Oxynetra hopfferi|[7902]|02-SRNP-23285|669[0n]bp
Oxynetra hopfferi|[7903]|02-SRNP-23109|669[0n]bp
Oxynetra hopfferi|[7904]|02-SRNP-23110|669[0n]bp
Oxynetra hopfferi|[7905]|03-SRNP-3638|669[0n]bp
Bungalotis erythus|[7906]|08-SRNP-65506|669[0n]bp
Bungalotis erythus|[7907]|08-SRNP-70954|669[0n]bp
Bungalotis erythus|[7908]|08-SRNP-21006|669[0n]bp
Bungalotis erythus|[7909]|08-SRNP-20672|669[0n]bp
Bungalotis erythus|[7910]|07-SRNP-46538|669[0n]bp
Bungalotis erythus|[7911]|07-SRNP-65250|669[0n]bp
Bungalotis erythus|[7912]|07-SRNP-41269|669[0n]bp
Bungalotis erythus|[7913]|07-SRNP-21991|669[0n]bp
Bungalotis erythus|[7914]|07-SRNP-940|669[0n]bp
Bungalotis erythus|[7915]|05-SRNP-33392|669[0n]bp
Bungalotis erythus|[7916]|06-SRNP-20583|669[0n]bp
Bungalotis erythus|[7917]|05-SRNP-45216|669[0n]bp
Bungalotis erythus|[7918]|05-SRNP-47613|669[0n]bp
Bungalotis erythus|[7919]|04-SRNP-55857|669[0n]bp
Bungalotis erythus|[7920]|04-SRNP-22883|669[0n]bp
Bungalotis erythus|[7921]|04-SRNP-61431|669[0n]bp
Bungalotis erythus|[7922]|04-SRNP-3609|669[0n]bp
Bungalotis erythus|[7923]|05-SRNP-20556|669[0n]bp
Bungalotis erythus|[7924]|04-SRNP-22815|669[0n]bp
Bungalotis erythus|[7925]|04-SRNP-4963|669[0n]bp
Bungalotis erythus|[7926]|07-SRNP-20903|666[0n]bp
Bungalotis erythus|[7927]|01-SRNP-24401|654[0n]bp
Bungalotis erythus|[7928]|07-SRNP-941|645[0n]bp
Bungalotis erythus|[7929]|97-SRNP-6142|627[0n]bp
Bungalotis erythus|[7930]|08-SRNP-70955|621[0n]bp
Bungalotis erythus|[7931]|08-SRNP-65224|645[0n]bp
Bungalotis erythus|[7932]|07-SRNP-36879|645[0n]bp
Bungalotis erythus|[7933]|08-SRNP-200|645[0n]bp
Bungalotis erythus|[7934]|08-SRNP-65769|669[0n]bp
Bungalotis quadratumDHJ02|[7935]|05-SRNP-2515|669[2n]bp
Bungalotis quadratumDHJ02|[7936]|07-SRNP-66034|666[0n]bp
Bungalotis quadratumDHJ02|[7937]|05-SRNP-23625|666[0n]bp
Bungalotis quadratumDHJ02|[7938]|05-SRNP-3116|654[0n]bp
Bungalotis quadratumDHJ02|[7939]|07-SRNP-4096|651[0n]bp
Bungalotis quadratumDHJ02|[7940]|07-SRNP-66035|645[0n]bp
Bungalotis quadratumDHJ02|[7941]|06-SRNP-31687|666[0n]bp
Bungalotis quadratumDHJ02|[7942]|03-SRNP-1484|645[1n]bp
Bungalotis quadratumDHJ02|[7943]|02-SRNP-33238|594[0n]bp
Bungalotis quadratumDHJ02|[7944]|02-SRNP-14657|585[0n]bp
Bungalotis quadratumDHJ02|[7945]|02-SRNP-20465|585[0n]bp
Bungalotis quadratumDHJ02|[7946]|05-SRNP-21979|669[0n]bp
Bungalotis quadratumDHJ02|[7947]|06-SRNP-517|669[0n]bp
Bungalotis quadratumDHJ02|[7948]|08-SRNP-65212|669[0n]bp
Bungalotis quadratumDHJ02|[7949]|08-SRNP-65420|669[0n]bp
Bungalotis quadratumDHJ02|[7950]|05-SRNP-41328|669[0n]bp
Bungalotis quadratumDHJ02|[7951]|05-SRNP-3115|687[0n]bp
Bungalotis quadratumDHJ02|[7952]|04-SRNP-10345|669[0n]bp
Bungalotis quadratumDHJ02|[7953]|05-SRNP-21480|669[0n]bp
Bungalotis quadratumDHJ02|[7954]|05-SRNP-41367|669[0n]bp
Bungalotis quadratumDHJ02|[7955]|05-SRNP-40975|669[0n]bp
Bungalotis quadratumDHJ02|[7956]|06-SRNP-3854|669[0n]bp
Bungalotis quadratumDHJ02|[7957]|08-SRNP-65367|669[0n]bp
Bungalotis quadratumDHJ02|[7958]|08-SRNP-65296|669[0n]bp
Bungalotis quadratumDHJ02|[7959]|08-SRNP-2181|669[0n]bp
Bungalotis quadratumDHJ02|[7960]|05-SRNP-21366|666[2n]bp
Bungalotis quadratumDHJ02|[7961]|07-SRNP-42298|669[0n]bp
Bungalotis quadratumDHJ02|[7962]|07-SRNP-2736|669[0n]bp
Bungalotis quadratumDHJ02|[7963]|05-SRNP-1726|669[0n]bp
Bungalotis quadratumDHJ02|[7964]|05-SRNP-12052|669[0n]bp
Bungalotis quadratumDHJ02|[7965]|04-SRNP-21541|669[0n]bp
Bungalotis quadratumDHJ02|[7966]|05-SRNP-8122|669[0n]bp
Bungalotis quadratumDHJ02|[7967]|04-SRNP-20799|669[0n]bp
Bungalotis quadratumDHJ02|[7968]|05-SRNP-64394|633[0n]bp
Bungalotis quadratumDHJ02|[7969]|07-SRNP-2182|669[0n]bp
Bungalotis quadratumDHJ02|[7970]|07-SRNP-65520|645[0n]bp
Bungalotis quadratumDHJ02|[7971]|08-SRNP-65306|669[0n]bp
Bungalotis quadratumDHJ02|[7972]|08-SRNP-318|669[0n]bp
Bungalotis quadratumDHJ02|[7973]|08-SRNP-21949|669[0n]bp
Bungalotis quadratumDHJ02|[7974]|08-SRNP-21946|669[0n]bp
Bungalotis quadratumDHJ01|[7975]|07-SRNP-20456|666[0n]bp
Bungalotis quadratumDHJ01|[7976]|07-SRNP-65502|669[0n]bp
Bungalotis quadratumDHJ01|[7977]|08-SRNP-1902|669[0n]bp
Bungalotis quadratumDHJ01|[7978]|08-SRNP-55655|669[0n]bp
Bungalotis quadratumDHJ01|[7979]|08-SRNP-65339|669[0n]bp
Bungalotis quadratumDHJ01|[7980]|08-SRNP-20633|669[0n]bp
Bungalotis quadratumDHJ01|[7981]|08-SRNP-20846|669[0n]bp
Bungalotis quadratumDHJ01|[7982]|08-SRNP-65211|669[0n]bp
Bungalotis quadratumDHJ01|[7983]|07-SRNP-42880|669[0n]bp
Bungalotis quadratumDHJ01|[7984]|08-SRNP-65342|669[0n]bp
Bungalotis quadratumDHJ01|[7985]|08-SRNP-2564|669[0n]bp
Bungalotis quadratumDHJ01|[7986]|07-SRNP-1559|669[0n]bp
Bungalotis quadratumDHJ01|[7987]|07-SRNP-1560|669[0n]bp
Bungalotis quadratumDHJ01|[7988]|07-SRNP-1561|669[0n]bp
Bungalotis quadratumDHJ01|[7989]|06-SRNP-5401|669[0n]bp
Bungalotis quadratumDHJ01|[7990]|05-SRNP-2294|669[0n]bp
Bungalotis quadratumDHJ01|[7991]|05-SRNP-2447|669[0n]bp
Bungalotis quadratumDHJ01|[7992]|05-SRNP-21145|669[0n]bp
Bungalotis quadratumDHJ01|[7993]|05-SRNP-12053|669[0n]bp
Bungalotis quadratumDHJ01|[7994]|05-SRNP-2293|669[0n]bp
Bungalotis quadratumDHJ01|[7995]|05-SRNP-21739|669[0n]bp
Bungalotis quadratumDHJ01|[7996]|04-SRNP-32299|669[0n]bp

Bungalotis quadratumDHJ01|[7994]|05-SRNP-2293|669[0n]bp
Bungalotis quadratumDHJ01|[7995]|05-SRNP-21739|669[0n]bp
Bungalotis quadratumDHJ01|[7996]|04-SRNP-32299|669[0n]bp
Bungalotis quadratumDHJ01|[7997]|04-SRNP-20807|669[0n]bp
Bungalotis quadratumDHJ01|[7998]|04-SRNP-21611|669[0n]bp
Bungalotis quadratumDHJ01|[7999]|07-SRNP-1876|666[0n]bp
Bungalotis quadratumDHJ01|[8000]|03-SRNP-1652|642[0n]bp
Bungalotis quadratumDHJ01|[8001]|07-SRNP-21698|663[0n]bp
Bungalotis quadratumDHJ01|[8002]|05-SRNP-3021|552[0n]bp
Bungalotis quadratumDHJ01|[8003]|02-SRNP-1284|621[0n]bp
Bungalotis quadratumDHJ01|[8004]|02-SRNP-20466|621[0n]bp
Bungalotis quadratumDHJ01|[8005]|02-SRNP-31454|621[0n]bp
Bungalotis quadratumDHJ01|[8006]|03-SRNP-1253|621[0n]bp
Bungalotis quadratumDHJ01|[8007]|02-SRNP-14135|621[0n]bp
Bungalotis quadratumDHJ01|[8008]|02-SRNP-4554|594[1n]bp
Bungalotis quadratumDHJ01|[8009]|02-SRNP-2667|594[0n]bp
Bungalotis quadratumDHJ01|[8010]|02-SRNP-1283|585[0n]bp
Bungalotis quadratumDHJ01|[8011]|02-SRNP-33827|585[0n]bp
Bungalotis quadratumDHJ01|[8012]|02-SRNP-2075|585[0n]bp
Bungalotis quadratumDHJ01|[8013]|02-SRNP-4552|585[0n]bp
Bungalotis quadratumDHJ01|[8014]|07-SRNP-46004|513[1n]bp
Bungalotis quadratumDHJ01|[8015]|08-SRNP-65328|669[0n]bp
Bungalotis quadratumDHJ01|[8016]|08-SRNP-40971|669[0n]bp
Bungalotis quadratumDHJ01|[8017]|08-SRNP-65688|669[0n]bp
Bungalotis quadratumDHJ01|[8018]|09-SRNP-20206|669[0n]bp
Bungalotis quadratumDHJ01|[8019]|05-SRNP-12177|645[0n]bp
Bungalotis quadratumDHJ01|[8020]|05-SRNP-3054|669[3n]bp
Bungalotis quadratumDHJ01|[8021]|05-SRNP-30602|669[4n]bp
Bungalotis quadratumDHJ01|[8022]|05-SRNP-3055|645[1n]bp
Bungalotis quadratumDHJ01|[8023]|07-SRNP-2743|666[0n]bp
Bungalotis quadratumDHJ01|[8024]|02-SRNP-6982|621[0n]bp
Bungalotis quadratumDHJ01|[8025]|05-SRNP-3484|669[0n]bp
Bungalotis quadratumDHJ01|[8026]|07-SRNP-5038|648[0n]bp
Bungalotis quadratumDHJ01|[8027]|05-SRNP-3088|663[1n]bp
Bungalotis quadratumDHJ01|[8028]|08-SRNP-40281|669[0n]bp
Bungalotis quadratumDHJ01|[8029]|08-SRNP-40180|669[0n]bp
Bungalotis quadratumDHJ01|[8030]|08-SRNP-586|669[0n]bp
Bungalotis quadratumDHJ01|[8031]|08-SRNP-65341|669[0n]bp
Bungalotis quadratumDHJ01|[8032]|08-SRNP-65340|669[0n]bp
Bungalotis quadratumDHJ01|[8033]|07-SRNP-12438|669[0n]bp
Bungalotis quadratumDHJ01|[8034]|08-SRNP-65454|669[0n]bp
Bungalotis quadratumDHJ01|[8035]|08-SRNP-65515|669[0n]bp
Bungalotis quadratumDHJ01|[8036]|08-SRNP-65368|669[0n]bp
Bungalotis quadratumDHJ01|[8037]|08-SRNP-65459|669[0n]bp
Bungalotis quadratumDHJ01|[8038]|08-SRNP-65164|669[0n]bp
Bungalotis quadratumDHJ01|[8039]|08-SRNP-267|669[0n]bp
Bungalotis quadratumDHJ01|[8040]|07-SRNP-4896|669[0n]bp
Bungalotis quadratumDHJ01|[8041]|08-SRNP-65276|669[0n]bp
Bungalotis quadratumDHJ01|[8042]|08-SRNP-585|669[0n]bp
Bungalotis quadratumDHJ01|[8043]|08-SRNP-65305|669[0n]bp
Bungalotis quadratumDHJ01|[8044]|08-SRNP-21065|669[0n]bp
Bungalotis quadratumDHJ01|[8045]|08-SRNP-1750|669[0n]bp
Bungalotis quadratumDHJ01|[8046]|08-SRNP-45152|669[0n]bp
Bungalotis quadratumDHJ01|[8047]|08-SRNP-296|669[0n]bp
Bungalotis quadratumDHJ01|[8048]|08-SRNP-2182|669[0n]bp
Bungalotis quadratumDHJ01|[8049]|08-SRNP-65327|669[0n]bp
Bungalotis quadratumDHJ01|[8050]|08-SRNP-164|669[0n]bp
Bungalotis quadratumDHJ01|[8051]|07-SRNP-3504|669[0n]bp
Bungalotis quadratumDHJ01|[8052]|08-SRNP-40213|669[0n]bp
Bungalotis quadratumDHJ01|[8053]|07-SRNP-21331|669[0n]bp
Bungalotis quadratumDHJ01|[8054]|07-SRNP-3488|669[0n]bp
Bungalotis quadratumDHJ01|[8055]|07-SRNP-566|669[0n]bp
Bungalotis quadratumDHJ01|[8056]|07-SRNP-21349|669[0n]bp
Bungalotis quadratumDHJ01|[8057]|05-SRNP-22370|669[0n]bp
Bungalotis quadratumDHJ01|[8058]|06-SRNP-41272|669[0n]bp
Bungalotis quadratumDHJ01|[8059]|06-SRNP-20160|669[0n]bp
Bungalotis quadratumDHJ01|[8060]|06-SRNP-1694|669[0n]bp
Bungalotis quadratumDHJ01|[8061]|06-SRNP-2564|669[0n]bp
Bungalotis quadratumDHJ01|[8062]|06-SRNP-1945|669[0n]bp
Bungalotis quadratumDHJ01|[8063]|06-SRNP-1686|669[0n]bp
Bungalotis quadratumDHJ01|[8064]|06-SRNP-260|669[0n]bp
Bungalotis quadratumDHJ01|[8065]|05-SRNP-4567|669[0n]bp
Bungalotis quadratumDHJ01|[8066]|05-SRNP-999|669[0n]bp
Bungalotis quadratumDHJ01|[8067]|05-SRNP-3053|669[0n]bp
Bungalotis quadratumDHJ01|[8068]|04-SRNP-48823|669[0n]bp
Bungalotis quadratumDHJ01|[8069]|05-SRNP-55167|669[0n]bp
Bungalotis quadratumDHJ01|[8070]|07-SRNP-4148|636[0n]bp
Bungalotis quadratumDHJ01|[8071]|05-SRNP-20357|636[0n]bp
Bungalotis quadratumDHJ01|[8072]|07-SRNP-4097|630[0n]bp
Bungalotis quadratumDHJ01|[8073]|07-SRNP-1859|660[0n]bp
Bungalotis quadratumDHJ01|[8074]|02-SRNP-3261|621[1n]bp
Bungalotis quadratumDHJ01|[8075]|02-SRNP-14043|621[0n]bp
Bungalotis quadratumDHJ01|[8076]|02-SRNP-3643|621[0n]bp
Bungalotis quadratumDHJ01|[8077]|02-SRNP-2979|621[0n]bp
Bungalotis quadratumDHJ01|[8078]|02-SRNP-3867|621[0n]bp
Bungalotis quadratumDHJ01|[8079]|02-SRNP-18838|621[0n]bp
Bungalotis quadratumDHJ01|[8080]|02-SRNP-2994|621[0n]bp
Bungalotis quadratumDHJ01|[8081]|02-SRNP-14137|621[0n]bp
Bungalotis quadratumDHJ01|[8082]|02-SRNP-14083|621[0n]bp
Bungalotis quadratumDHJ01|[8083]|01-SRNP-628|594[0n]bp
Bungalotis quadratumDHJ01|[8084]|02-SRNP-5698|594[0n]bp
Bungalotis quadratumDHJ01|[8085]|02-SRNP-2310|585[0n]bp
Bungalotis quadratumDHJ01|[8086]|02-SRNP-14094|585[0n]bp
Bungalotis quadratumDHJ01|[8087]|02-SRNP-14599|585[0n]bp
Bungalotis quadratumDHJ01|[8088]|02-SRNP-5850|582[0n]bp
Bungalotis quadratumDHJ01|[8089]|08-SRNP-959|636[0n]bp
Bungalotis quadratumDHJ01|[8090]|08-SRNP-40212|645[0n]bp
Bungalotis quadratumDHJ01|[8091]|09-SRNP-20809|669[0n]bp
Bungalotis astylos|[8092]|04-SRNP-23054|627[0n]bp
Bungalotis astylos|[8093]|05-SRNP-3123|669[0n]bp
Bungalotis astylos|[8094]|07-SRNP-32268|669[0n]bp
Bungalotis astylos|[8095]|06-SRNP-55070|669[0n]bp
Bungalotis astylos|[8096]|04-SRNP-48647|669[0n]bp

Bungalotis astylos|[8094]|07-SRNP-32268|669[0n]bp
Bungalotis astylos|[8095]|06-SRNP-55070|669[0n]bp
Bungalotis astylos|[8096]|04-SRNP-48647|669[0n]bp
Bungalotis astylos|[8097]|07-SRNP-31525|669[0n]bp
Bungalotis astylos|[8098]|07-SRNP-40972|669[0n]bp
Bungalotis astylos|[8099]|07-SRNP-2056|669[0n]bp
Bungalotis astylos|[8100]|07-SRNP-42318|669[0n]bp
Bungalotis astylos|[8101]|07-SRNP-33568|669[0n]bp
Bungalotis astylos|[8102]|06-SRNP-57444|669[0n]bp
Bungalotis astylos|[8103]|06-SRNP-59176|669[0n]bp
Bungalotis astylos|[8104]|06-SRNP-43639|669[0n]bp
Bungalotis astylos|[8105]|06-SRNP-33017|669[0n]bp
Bungalotis astylos|[8106]|07-SRNP-57567|669[0n]bp
Bungalotis astylos|[8107]|07-SRNP-57566|669[0n]bp
Bungalotis astylos|[8108]|05-SRNP-3124|669[0n]bp
Bungalotis astylos|[8109]|06-SRNP-58700|585[1n]bp
Bungalotis astylos|[8110]|01-SRNP-1961|663[0n]bp
Bungalotis astylos|[8111]|07-SRNP-57565|660[0n]bp
Bungalotis astylos|[8112]|09-SRNP-31141|660[0n]bp
Bungalotis astylos|[8113]|05-SRNP-66563|669[3n]bp
Bungalotis astylos|[8114]|06-SRNP-59177|621[0n]bp
Bungalotis astylos|[8115]|96-SRNP-11379|639[0n]bp
Bungalotis astylos|[8116]|09-SRNP-1890|669[0n]bp
Bungalotis midas|[8117]|03-SRNP-12881.1|567[0n]bp
Bungalotis midas|[8118]|06-SRNP-43436|666[0n]bp
Bungalotis midas|[8119]|03-SRNP-9076|654[0n]bp
Bungalotis midas|[8120]|05-SRNP-4908|687[0n]bp
Bungalotis midas|[8121]|03-SRNP-15139|660[0n]bp
Bungalotis midas|[8122]|94-SRNP-308|399[0n]bp
Bungalotis midas|[8123]|03-SRNP-9194|576[0n]bp
Bungalotis midas|[8124]|03-SRNP-34185|663[0n]bp
Bungalotis midas|[8125]|05-SRNP-4469|669[0n]bp
Bungalotis midas|[8126]|06-SRNP-21109|669[0n]bp
Bungalotis midas|[8127]|07-SRNP-40390|669[0n]bp
Bungalotis midas|[8128]|09-SRNP-44040|669[0n]bp
Bungalotis midas|[8129]|09-SRNP-20274|669[0n]bp
Bungalotis diophorus|[8130]|06-SRNP-1016|669[2n]bp
Bungalotis diophorus|[8131]|05-SRNP-24718|669[0n]bp
Bungalotis diophorus|[8132]|07-SRNP-3318|669[0n]bp
Bungalotis diophorus|[8133]|07-SRNP-3315|669[0n]bp
Bungalotis diophorus|[8134]|08-SRNP-70990|663[0n]bp
Bungalotis diophorus|[8135]|04-SRNP-34119|669[0n]bp
Bungalotis diophorus|[8136]|04-SRNP-56177|669[0n]bp
Bungalotis diophorus|[8137]|08-SRNP-70423|669[0n]bp
Bungalotis diophorus|[8138]|08-SRNP-65707|669[1n]bp
Bungalotis diophorus|[8139]|03-SRNP-1854|654[0n]bp
Bungalotis diophorus|[8140]|03-SRNP-10878|645[0n]bp
Bungalotis diophorus|[8141]|04-SRNP-34165|669[0n]bp
Bungalotis diophorus|[8142]|04-SRNP-34027|669[0n]bp
Bungalotis diophorus|[8143]|04-SRNP-33359|669[0n]bp
Bungalotis diophorus|[8144]|04-SRNP-33198|669[0n]bp
Bungalotis diophorus|[8145]|05-SRNP-21655|669[0n]bp
Bungalotis diophorus|[8146]|05-SRNP-34150|669[0n]bp
Bungalotis diophorus|[8147]|07-SRNP-65195|669[0n]bp
Bungalotis diophorus|[8148]|07-SRNP-3311|669[0n]bp
Bungalotis diophorus|[8149]|07-SRNP-23801|669[0n]bp
Bungalotis diophorus|[8150]|07-SRNP-23522|669[0n]bp
Bungalotis diophorus|[8151]|08-SRNP-70422|669[0n]bp
Bungalotis diophorus|[8152]|08-SRNP-70986|669[0n]bp
Bungalotis diophorus|[8153]|08-SRNP-71152|669[0n]bp
Bungalotis diophorus|[8154]|09-SRNP-22402|669[0n]bp
Nascus paulliniae|[8155]|08-SRNP-5198|321[0n]bp
Nascus paulliniae|[8156]|08-SRNP-5133|321[0n]bp
Nascus paulliniae|[8157]|99-SRNP-15218|558[0n]bp
Nascus paulliniae|[8158]|04-SRNP-55249|669[0n]bp
Nascus paulliniae|[8159]|06-SRNP-7877|669[0n]bp
Nascus paulliniae|[8160]|05-SRNP-42441|669[0n]bp
Nascus paulliniae|[8161]|05-SRNP-5420|669[0n]bp
Nascus paulliniae|[8162]|05-SRNP-888|669[0n]bp
Nascus paulliniae|[8163]|04-SRNP-14706|669[0n]bp
Nascus paulliniae|[8164]|04-SRNP-34021|669[0n]bp
Nascus paulliniae|[8165]|04-SRNP-34682|669[0n]bp
Nascus paulliniae|[8166]|04-SRNP-14655|669[0n]bp
Nascus paulliniae|[8167]|04-SRNP-55654|669[0n]bp
Nascus paulliniae|[8168]|04-SRNP-55794|669[0n]bp
Nascus paulliniae|[8169]|04-SRNP-55367|669[0n]bp
Nascus paulliniae|[8170]|04-SRNP-55964|669[0n]bp
Nascus paulliniae|[8171]|02-SRNP-6701|669[0n]bp
Nascus paulliniae|[8172]|95-SRNP-9964|627[0n]bp
Nascus paulliniae|[8173]|05-SRNP-45154|627[0n]bp
Nascus paulliniae|[8174]|08-SRNP-481|633[0n]bp
Nascus paulliniae|[8175]|04-SRNP-14559|669[0n]bp
Nicephellus nicephorus|[8176]|02-SRNP-27688|495[3n]bp
Nicephellus nicephorus|[8177]|02-SRNP-27689|654[0n]bp
Salatis canalis|[8178]|02-SRNP-18982|600[1n]bp
Salatis canalis|[8179]|05-SRNP-3157|687[0n]bp
Salatis canalis|[8180]|08-SRNP-70373|669[0n]bp
Salatis canalis|[8181]|08-SRNP-70839|669[0n]bp
Salatis canalis|[8182]|08-SRNP-20448|669[0n]bp
Salatis canalis|[8183]|07-SRNP-65776|669[0n]bp
Salatis canalis|[8184]|08-SRNP-287|669[0n]bp
Salatis canalis|[8185]|06-SRNP-9549|669[0n]bp
Salatis canalis|[8186]|06-SRNP-44103|669[0n]bp
Salatis canalis|[8187]|05-SRNP-1100|669[0n]bp
Salatis canalis|[8188]|04-SRNP-60727|669[0n]bp
Salatis canalis|[8189]|04-SRNP-1838|669[0n]bp
Salatis canalis|[8190]|03-SRNP-1802|645[0n]bp
Salatis canalis|[8191]|03-SRNP-35001|645[0n]bp
Salatis canalis|[8192]|02-SRNP-18867|645[0n]bp
Salatis canalis|[8193]|02-SRNP-19694|645[1n]bp
Salatis canalis|[8194]|05-SRNP-1017|666[0n]bp
Salatis canalis|[8195]|02-SRNP-3899|645[0n]bp
Salatis canalis|[8196]|03-SRNP-5164|645[0n]bp

Salatis canalis|[8194]|05-SRNP-1017|666[0n]bp
Salatis canalis|[8195]|02-SRNP-3899|645[0n]bp
Salatis canalis|[8196]|03-SRNP-5164|645[0n]bp
Salatis canalis|[8197]|07-SRNP-2774|660[0n]bp
Salatis canalis|[8198]|08-SRNP-20447|645[0n]bp
Salatis canalis|[8199]|08-SRNP-71642|639[0n]bp
Salatis canalis|[8200]|08-SRNP-4733|669[0n]bp
Salatis canalis|[8201]|09-SRNP-22934|669[0n]bp
Cephise aelius|[8202]|06-SRNP-20598|582[1n]bp
Cephise aelius|[8203]|06-SRNP-2857|669[0n]bp
Cephise aelius|[8204]|06-SRNP-30466|669[0n]bp
Cephise aelius|[8205]|06-SRNP-12607|669[0n]bp
Cephise aelius|[8206]|06-SRNP-12587|669[0n]bp
Cephise aelius|[8207]|06-SRNP-12591|669[0n]bp
Cephise aelius|[8208]|05-SRNP-55123|669[0n]bp
Cephise aelius|[8209]|06-SRNP-3213|666[0n]bp
Cephise aelius|[8210]|03-SRNP-234|630[0n]bp
Cephise aelius|[8211]|03-SRNP-652|597[1n]bp
Cephise aelius|[8212]|02-SRNP-5365|651[0n]bp
Cephise aelius|[8213]|02-SRNP-5356|651[0n]bp
Cephise aelius|[8214]|07-SRNP-55696|669[0n]bp
Cephise aelius|[8215]|08-SRNP-295|669[0n]bp
Cephise aelius|[8216]|07-SRNP-61378|669[0n]bp
Cephise aelius|[8217]|08-SRNP-55427|669[0n]bp
Cephise aelius|[8218]|09-SRNP-22247|669[0n]bp
Cephise nuspesezDHJ01|[8219]|05-SRNP-30053|645[0n]bp
Cephise nuspesezDHJ01|[8220]|07-SRNP-30952|669[0n]bp
Cephise nuspesezDHJ01|[8221]|08-SRNP-20453|669[0n]bp
Cephise nuspesezDHJ01|[8222]|08-SRNP-20452|669[0n]bp
Cephise nuspesezDHJ01|[8223]|09-SRNP-68309|669[0n]bp
Cephise nuspesezDHJ03|[8224]|02-SRNP-663|651[0n]bp
Cephise nuspesezDHJ02|[8225]|06-SRNP-59594|618[1n]bp
Cephise nuspesezDHJ02|[8226]|05-SRNP-40841|669[0n]bp
Cephise nuspesezDHJ02|[8227]|07-SRNP-58078|669[0n]bp
Cephise nuspesezDHJ02|[8228]|08-SRNP-2349|669[0n]bp
Cephise nuspesezDHJ02|[8229]|04-SRNP-21120|585[0n]bp
Cephise nuspesezDHJ02|[8230]|04-SRNP-41085|624[0n]bp
Cephise nuspesezDHJ02|[8231]|04-SRNP-56573|624[0n]bp
Cephise nuspesezDHJ02|[8232]|04-SRNP-24552|624[0n]bp
Cephise nuspesezDHJ02|[8233]|04-SRNP-42550|624[0n]bp
Cephise nuspesezDHJ02|[8234]|04-SRNP-56917|624[0n]bp
Cephise nuspesezDHJ02|[8235]|04-SRNP-1731|624[0n]bp
Cephise nuspesezDHJ02|[8236]|04-SRNP-56576|624[0n]bp
Cephise nuspesezDHJ02|[8237]|04-SRNP-56570|624[0n]bp
Cephise nuspesezDHJ02|[8238]|04-SRNP-47334|624[0n]bp
Cephise nuspesezDHJ02|[8239]|04-SRNP-61251|624[3n]bp
Cephise nuspesezDHJ02|[8240]|04-SRNP-35344|624[0n]bp
Cephise nuspesezDHJ02|[8241]|04-SRNP-45451|669[0n]bp
Cephise nuspesezDHJ02|[8242]|05-SRNP-40050|669[0n]bp
Cephise nuspesezDHJ02|[8243]|06-SRNP-23457|669[0n]bp
Cephise nuspesezDHJ02|[8244]|07-SRNP-31118|645[0n]bp
Cephise nuspesezDHJ02|[8245]|04-SRNP-1075|645[0n]bp
Cephise nuspesezDHJ02|[8246]|04-SRNP-40599|645[0n]bp
Cephise nuspesezDHJ02|[8247]|03-SRNP-21962|645[0n]bp
Cephise nuspesezDHJ02|[8248]|03-SRNP-21961|645[0n]bp
Cephise nuspesezDHJ02|[8249]|03-SRNP-5407|645[0n]bp
Cephise nuspesezDHJ02|[8250]|03-SRNP-3473|645[0n]bp
Cephise nuspesezDHJ02|[8251]|09-SRNP-20005|669[0n]bp
Cephise nuspesezDHJ02|[8252]|09-SRNP-387|669[0n]bp
Cephise nuspesezDHJ02|[8253]|08-SRNP-23259|669[0n]bp
Cephise nuspesezDHJ02|[8254]|08-SRNP-23248|669[0n]bp
Cephise nuspesezDHJ02|[8255]|08-SRNP-40706|669[0n]bp
Cephise nuspesezDHJ02|[8256]|08-SRNP-35369|669[0n]bp
Cephise nuspesezDHJ02|[8257]|08-SRNP-20435|669[0n]bp
Cephise nuspesezDHJ02|[8258]|08-SRNP-945|669[0n]bp
Cephise nuspesezDHJ02|[8259]|08-SRNP-748|669[0n]bp
Cephise nuspesezDHJ02|[8260]|08-SRNP-40861|669[0n]bp
Cephise nuspesezDHJ02|[8261]|07-SRNP-58267|669[0n]bp
Cephise nuspesezDHJ02|[8262]|04-SRNP-61487|669[0n]bp
Cephise nuspesezDHJ02|[8263]|04-SRNP-48306|669[0n]bp
Cephise nuspesezDHJ02|[8264]|03-SRNP-5930|651[0n]bp
Cephise nuspesezDHJ02|[8265]|08-SRNP-655|642[0n]bp
Cephise nuspesezDHJ02|[8266]|02-SRNP-33859|657[0n]bp
Cephise nuspesezDHJ02|[8267]|03-SRNP-10602|666[0n]bp
Cephise nuspesezDHJ02|[8268]|05-SRNP-43495|663[0n]bp
Cephise nuspesezDHJ02|[8269]|07-SRNP-31639|648[1n]bp
Cephise nuspesezDHJ02|[8270]|00-SRNP-110|630[0n]bp
Cephise nuspesezDHJ02|[8271]|03-SRNP-5879|576[0n]bp
Cephise nuspesezDHJ02|[8272]|04-SRNP-61513|648[1n]bp
Cephise nuspesezDHJ02|[8273]|03-SRNP-5406|624[0n]bp
Cephise nuspesezDHJ02|[8274]|02-SRNP-5636|651[0n]bp
Cephise nuspesezDHJ02|[8275]|03-SRNP-5475|492[0n]bp
Cephise nuspesezDHJ02|[8276]|07-SRNP-66199|594[0n]bp
Cephise nuspesezDHJ02|[8277]|08-SRNP-531|645[0n]bp
Cephise nuspesezDHJ02|[8278]|09-SRNP-20293|669[0n]bp
Cephise nuspesezDHJ02|[8279]|09-SRNP-20294|669[0n]bp
Cephise Burns01|[8280]|03-SRNP-10046|639[0n]bp
Cephise Burns01|[8281]|06-SRNP-41883|669[0n]bp
Cephise Burns01|[8282]|08-SRNP-31038|669[0n]bp
Cephise Burns01|[8283]|09-SRNP-69829|669[0n]bp
Cephise Burns01|[8284]|03-SRNP-10720|657[0n]bp
Cephise Burns01|[8285]|03-SRNP-10166|657[0n]bp
Cephise Burns01|[8286]|04-SRNP-34910|669[0n]bp
Cephise Burns01|[8287]|04-SRNP-34943|669[0n]bp
Cephise Burns01|[8288]|08-SRNP-42404|633[0n]bp
Cephise Burns01|[8289]|03-SRNP-21880|597[0n]bp
Cephise Burns01|[8290]|03-SRNP-10462|513[1n]bp
Cephise Burns01|[8291]|08-SRNP-24687|669[0n]bp
Cephise Burns01|[8292]|00-SRNP-11741|630[0n]bp
Cephise Burns01|[8293]|05-SRNP-24566|645[0n]bp
Cephise Burns01|[8294]|04-SRNP-32954|669[0n]bp
Cephise Burns01|[8295]|04-SRNP-41887|669[0n]bp
Cephise Burns01|[8296]|06-SRNP-41095|669[0n]bp

Cephise Burns01|[8294]|04-SRNP-32954|669[0n]bp
Cephise Burns01|[8295]|04-SRNP-41887|669[0n]bp
Cephise Burns01|[8296]|06-SRNP-41095|669[0n]bp
Cephise Burns01|[8297]|06-SRNP-23379|669[0n]bp
Cephise Burns01|[8298]|05-SRNP-33964|669[0n]bp
Cephise Burns01|[8299]|09-SRNP-80522|669[0n]bp
Cephise Burns01|[8300]|07-SRNP-41982|645[0n]bp
Cephise Burns01|[8301]|03-SRNP-21881|645[0n]bp
Cephise Burns01|[8302]|05-SRNP-41674|603[0n]bp
Cephise Burns01|[8303]|05-SRNP-23773|645[0n]bp
Cephise Burns01|[8304]|06-SRNP-65516|642[0n]bp
Cephise Burns01|[8305]|08-SRNP-40693|669[0n]bp
Cephise Burns01|[8306]|09-SRNP-40285|669[0n]bp
Cephise Burns01|[8307]|09-SRNP-30023|669[0n]bp
Cephise Burns01|[8308]|09-SRNP-40454|669[0n]bp
Cephise Burns01|[8309]|09-SRNP-67702|669[0n]bp
Cephise Burns01|[8310]|09-SRNP-67936|669[0n]bp
Cephise Burns01|[8311]|08-SRNP-72778|669[0n]bp
Cephise Burns01|[8312]|08-SRNP-42162|669[0n]bp
Cephise Burns01|[8313]|09-SRNP-69828|669[0n]bp
Cephise Burns01|[8314]|07-SRNP-32126|669[0n]bp
Cephise Burns01|[8315]|07-SRNP-32127|669[0n]bp
Cephise Burns01|[8316]|07-SRNP-31871|669[0n]bp
Cephise Burns01|[8317]|06-SRNP-41266|669[0n]bp
Cephise Burns01|[8318]|04-SRNP-42479|669[0n]bp
Cephise Burns01|[8319]|04-SRNP-32936|669[0n]bp
Cephise Burns01|[8320]|04-SRNP-55099|669[0n]bp
Cephise Burns01|[8321]|04-SRNP-56368|669[0n]bp
Cephise Burns01|[8322]|04-SRNP-32391|669[0n]bp
Cephise Burns01|[8323]|04-SRNP-42353|669[0n]bp
Cephise Burns01|[8324]|06-SRNP-34228|630[0n]bp
Cephise Burns01|[8325]|07-SRNP-32173|633[0n]bp
Cephise Burns01|[8326]|01-SRNP-4401|624[0n]bp
Cephise Burns01|[8327]|08-SRNP-24688|603[0n]bp
Cephise Burns01|[8328]|09-SRNP-68422|615[0n]bp
Dyscophellus phraxanorDHJ01|[8329]|00-SRNP-14033|669[0n]bp
Dyscophellus phraxanorDHJ01|[8330]|07-SRNP-32221|669[0n]bp
Dyscophellus phraxanorDHJ01|[8331]|02-SRNP-3482|669[0n]bp
Dyscophellus phraxanorDHJ01|[8332]|01-SRNP-5699|669[0n]bp
Dyscophellus phraxanorDHJ01|[8333]|01-SRNP-22949|669[0n]bp
Dyscophellus phraxanorDHJ01|[8334]|02-SRNP-6877|669[0n]bp
Dyscophellus phraxanorDHJ01|[8335]|99-SRNP-13675|669[0n]bp
Dyscophellus phraxanorDHJ01|[8336]|99-SRNP-12738|669[0n]bp
Dyscophellus phraxanorDHJ01|[8337]|99-SRNP-12756|669[0n]bp
Dyscophellus phraxanorDHJ01|[8338]|99-SRNP-12686|669[0n]bp
Dyscophellus phraxanorDHJ01|[8339]|99-SRNP-12371|669[0n]bp
Dyscophellus phraxanorDHJ01|[8340]|99-SRNP-12368|669[0n]bp
Dyscophellus phraxanorDHJ01|[8341]|01-SRNP-25007|669[0n]bp
Dyscophellus phraxanorDHJ01|[8342]|01-SRNP-175|669[0n]bp
Dyscophellus phraxanorDHJ01|[8343]|00-SRNP-14611|669[0n]bp
Dyscophellus phraxanorDHJ01|[8344]|00-SRNP-14606|669[0n]bp
Dyscophellus phraxanorDHJ01|[8345]|00-SRNP-12745|669[0n]bp
Dyscophellus phraxanorDHJ01|[8346]|99-SRNP-5588|669[0n]bp
Dyscophellus phraxanorDHJ01|[8347]|01-SRNP-64|669[0n]bp
Dyscophellus phraxanorDHJ01|[8348]|99-SRNP-13192|669[0n]bp
Dyscophellus phraxanorDHJ01|[8349]|99-SRNP-13329|669[0n]bp
Dyscophellus phraxanorDHJ01|[8350]|98-SRNP-7943|669[0n]bp
Dyscophellus phraxanorDHJ01|[8351]|02-SRNP-2032|669[0n]bp
Dyscophellus phraxanorDHJ01|[8352]|02-SRNP-982|669[0n]bp
Dyscophellus phraxanorDHJ01|[8353]|02-SRNP-3712|669[0n]bp
Dyscophellus phraxanorDHJ01|[8354]|01-SRNP-3949|669[0n]bp
Dyscophellus phraxanorDHJ01|[8355]|99-SRNP-13337|669[0n]bp
Dyscophellus phraxanorDHJ01|[8356]|00-SRNP-4533|669[0n]bp
Dyscophellus phraxanorDHJ01|[8357]|01-SRNP-9094|669[0n]bp
Dyscophellus phraxanorDHJ01|[8358]|00-SRNP-14920|618[1n]bp
Dyscophellus phraxanorDHJ01|[8359]|99-SRNP-4770|660[0n]bp
Dyscophellus phraxanorDHJ01|[8360]|00-SRNP-22143|645[0n]bp
Dyscophellus phraxanorDHJ01|[8361]|98-SRNP-7950|654[1n]bp
Dyscophellus phraxanorDHJ01|[8362]|99-SRNP-12369|609[1n]bp
Dyscophellus phraxanorDHJ01|[8363]|99-SRNP-13331|609[0n]bp
Dyscophellus phraxanorDHJ01|[8364]|99-SRNP-12797|597[0n]bp
Dyscophellus phraxanorDHJ01|[8365]|07-SRNP-41526|627[0n]bp
Dyscophellus phraxanorDHJ01|[8366]|07-SRNP-41240|669[0n]bp
Dyscophellus phraxanorDHJ01|[8367]|07-SRNP-65772|669[0n]bp
Dyscophellus phraxanorDHJ01|[8368]|08-SRNP-65547|669[0n]bp
Dyscophellus phraxanorDHJ01|[8369]|09-SRNP-68521|669[0n]bp
Dyscophellus phraxanorDHJ01|[8370]|09-SRNP-68520|669[0n]bp
Dyscophellus phraxanorDHJ04|[8371]|99-SRNP-12766|669[0n]bp
Dyscophellus phraxanorDHJ04|[8372]|00-SRNP-4233|588[0n]bp
Dyscophellus phraxanorDHJ04|[8373]|07-SRNP-65144|669[1n]bp
Dyscophellus phraxanorDHJ04|[8374]|08-SRNP-70094|669[0n]bp
Dyscophellus phraxanorDHJ04|[8375]|99-SRNP-12825|591[0n]bp
Dyscophellus phraxanorDHJ04|[8376]|09-SRNP-68523|669[0n]bp
Dyscophellus phraxanorDHJ02|[8377]|08-SRNP-66081|669[1n]bp
Dyscophellus phraxanorDHJ02|[8378]|07-SRNP-65679|633[0n]bp
Dyscophellus phraxanorDHJ02|[8379]|00-SRNP-12285|669[0n]bp
Dyscophellus phraxanorDHJ02|[8380]|00-SRNP-14612|669[0n]bp
Dyscophellus phraxanorDHJ02|[8381]|99-SRNP-12737|645[0n]bp
Dyscophellus phraxanorDHJ02|[8382]|99-SRNP-12807|669[0n]bp
Dyscophellus phraxanorDHJ02|[8383]|02-SRNP-6962|669[0n]bp
Dyscophellus phraxanorDHJ02|[8384]|06-SRNP-65714|669[0n]bp
Dyscophellus phraxanorDHJ02|[8385]|09-SRNP-67283|669[0n]bp
Dyscophellus phraxanorDHJ03|[8386]|07-SRNP-65163|669[0n]bp
Dyscophellus phraxanorDHJ03|[8387]|01-SRNP-4859|594[0n]bp
Dyscophellus phraxanorDHJ03|[8388]|08-SRNP-7035|669[0n]bp
Dyscophellus phraxanorDHJ03|[8389]|01-SRNP-23461|669[0n]bp
Dyscophellus phraxanorDHJ03|[8390]|01-SRNP-22998|669[0n]bp
Dyscophellus phraxanorDHJ03|[8391]|01-SRNP-23558|669[0n]bp
Dyscophellus phraxanorDHJ03|[8392]|01-SRNP-22434|624[0n]bp
Dyscophellus phraxanorDHJ03|[8393]|97-SRNP-6236|588[0n]bp
Dyscophellus phraxanorDHJ03|[8394]|00-SRNP-14728|606[0n]bp
Dyscophellus phraxanorDHJ03|[8395]|07-SRNP-42162|669[0n]bp
Dyscophellus phraxanorDHJ03|[8396]|07-SRNP-65384|669[0n]bp

Dyscophellus phraxanorDHJ03|[8394]|00-SRNP-14728|606[0n]bp
Dyscophellus phraxanorDHJ03|[8395]|07-SRNP-42162|669[0n]bp
Dyscophellus phraxanorDHJ03|[8396]|07-SRNP-65384|669[0n]bp
Dyscophellus phraxanorDHJ03|[8397]|02-SRNP-2879|669[0n]bp
Dyscophellus phraxanorDHJ03|[8398]|07-SRNP-65189|669[0n]bp
Dyscophellus phraxanorDHJ03|[8399]|98-SRNP-15000|669[0n]bp
Dyscophellus phraxanorDHJ03|[8400]|99-SRNP-12565|669[0n]bp
Dyscophellus phraxanorDHJ03|[8401]|99-SRNP-5787|669[0n]bp
Dyscophellus phraxanorDHJ03|[8402]|04-SRNP-27330|669[0n]bp
Dyscophellus phraxanorDHJ03|[8403]|04-SRNP-27328|669[0n]bp
Dyscophellus phraxanorDHJ03|[8404]|04-SRNP-34002|669[0n]bp
Dyscophellus phraxanorDHJ03|[8405]|99-SRNP-13498|669[0n]bp
Dyscophellus phraxanorDHJ02|[8406]|99-SRNP-5485|654[0n]bp
Dyscophellus phraxanorDHJ02|[8407]|98-SRNP-6815|606[0n]bp
Dyscophellus phraxanorDHJ02|[8408]|99-SRNP-12566|633[0n]bp
Dyscophellus phraxanorDHJ02|[8409]|01-SRNP-22109|585[0n]bp
Dyscophellus phraxanorDHJ02|[8410]|97-SRNP-6367|609[0n]bp
Dyscophellus phraxanorDHJ02|[8411]|01-SRNP-3190|594[0n]bp
Dyscophellus phraxanorDHJ02|[8412]|99-SRNP-12255|606[0n]bp
Dyscophellus phraxanorDHJ02|[8413]|97-SRNP-6218|582[0n]bp
Dyscophellus phraxanorDHJ02|[8414]|99-SRNP-5786|585[0n]bp
Dyscophellus phraxanorDHJ03|[8415]|01-SRNP-3948|669[0n]bp
Dyscophellus phraxanorDHJ03|[8416]|99-SRNP-12568|669[0n]bp
Dyscophellus phraxanorDHJ03|[8417]|99-SRNP-13505|669[0n]bp
Dyscophellus phraxanorDHJ03|[8418]|99-SRNP-15163|669[0n]bp
Dyscophellus phraxanorDHJ03|[8419]|07-SRNP-45755|669[0n]bp
Dyscophellus phraxanorDHJ03|[8420]|08-SRNP-31603|669[0n]bp
Dyscophellus phraxanorDHJ03|[8421]|07-SRNP-45264|669[0n]bp
Dyscophellus phraxanorDHJ03|[8422]|99-SRNP-12512|669[0n]bp
Dyscophellus phraxanorDHJ03|[8423]|01-SRNP-23096|669[0n]bp
Dyscophellus phraxanorDHJ03|[8424]|99-SRNP-12186|669[0n]bp
Dyscophellus phraxanorDHJ03|[8425]|01-SRNP-22953|624[0n]bp
Dyscophellus phraxanorDHJ03|[8426]|02-SRNP-18265|627[0n]bp
Dyscophellus phraxanorDHJ03|[8427]|98-SRNP-14893|651[0n]bp
Dyscophellus phraxanorDHJ03|[8428]|05-SRNP-20557|645[0n]bp
Dyscophellus phraxanorDHJ03|[8429]|01-SRNP-9032|669[0n]bp
Dyscophellus phraxanorDHJ03|[8430]|08-SRNP-32859|669[0n]bp
Dyscophellus phraxanorDHJ02|[8431]|08-SRNP-6169|666[5n]bp
Dyscophellus phraxanorDHJ02|[8432]|00-SRNP-14734|669[0n]bp
Dyscophellus phraxanorDHJ02|[8433]|99-SRNP-13462|669[0n]bp
Dyscophellus phraxanorDHJ02|[8434]|01-SRNP-9165|669[0n]bp
Dyscophellus phraxanorDHJ02|[8435]|99-SRNP-5874|669[0n]bp
Dyscophellus phraxanorDHJ02|[8436]|02-SRNP-3484|669[0n]bp
Dyscophellus phraxanorDHJ02|[8437]|00-SRNP-11848|669[0n]bp
Dyscophellus phraxanorDHJ02|[8438]|00-SRNP-11388|669[0n]bp
Dyscophellus phraxanorDHJ02|[8439]|99-SRNP-5875|630[0n]bp
Dyscophellus phraxanorDHJ02|[8440]|00-SRNP-4333|669[0n]bp
Dyscophellus phraxanorDHJ02|[8441]|99-SRNP-12489|669[0n]bp
Dyscophellus phraxanorDHJ02|[8442]|00-SRNP-14680|669[0n]bp
Dyscophellus phraxanorDHJ02|[8443]|01-SRNP-3501|669[0n]bp
Dyscophellus phraxanorDHJ02|[8444]|07-SRNP-32987|669[0n]bp
Dyscophellus phraxanorDHJ02|[8445]|04-SRNP-34005|669[0n]bp
Dyscophellus phraxanorDHJ02|[8446]|04-SRNP-34935|669[0n]bp
Dyscophellus phraxanorDHJ02|[8447]|07-SRNP-32986|669[0n]bp
Dyscophellus phraxanorDHJ02|[8448]|98-SRNP-6943|669[0n]bp
Dyscophellus phraxanorDHJ02|[8449]|02-SRNP-3485|669[0n]bp
Dyscophellus phraxanorDHJ02|[8450]|02-SRNP-3481|669[0n]bp
Dyscophellus phraxanorDHJ02|[8451]|99-SRNP-13135|669[0n]bp
Dyscophellus phraxanorDHJ02|[8452]|00-SRNP-21015|669[0n]bp
Dyscophellus phraxanorDHJ02|[8453]|00-SRNP-4194|669[0n]bp
Dyscophellus phraxanorDHJ02|[8454]|01-SRNP-3303|669[0n]bp
Dyscophellus phraxanorDHJ02|[8455]|01-SRNP-1559|669[0n]bp
Dyscophellus phraxanorDHJ02|[8456]|06-SRNP-44849|669[1n]bp
Dyscophellus phraxanorDHJ02|[8457]|02-SRNP-6149|624[0n]bp
Dyscophellus phraxanorDHJ02|[8458]|04-SRNP-23209|669[0n]bp
Dyscophellus phraxanorDHJ02|[8459]|01-SRNP-3381|669[0n]bp
Dyscophellus phraxanorDHJ02|[8460]|01-SRNP-9118|669[0n]bp
Dyscophellus phraxanorDHJ02|[8461]|01-SRNP-3519|669[0n]bp
Dyscophellus phraxanorDHJ02|[8462]|97-SRNP-6945|630[0n]bp
Dyscophellus phraxanorDHJ02|[8463]|01-SRNP-25126|603[0n]bp
Dyscophellus phraxanorDHJ02|[8464]|01-SRNP-22999|669[0n]bp
Dyscophellus phraxanorDHJ02|[8465]|01-SRNP-3785|627[1n]bp
Dyscophellus phraxanorDHJ02|[8466]|02-SRNP-18329|645[0n]bp
Dyscophellus phraxanorDHJ02|[8467]|97-SRNP-6959|582[0n]bp
Dyscophellus phraxanorDHJ02|[8468]|00-SRNP-4255|564[0n]bp
Dyscophellus phraxanorDHJ02|[8469]|97-SRNP-6601|606[0n]bp
Dyscophellus phraxanorDHJ02|[8470]|99-SRNP-12220|606[0n]bp
Dyscophellus phraxanorDHJ02|[8471]|99-SRNP-12767|606[0n]bp
Dyscophellus phraxanorDHJ02|[8472]|99-SRNP-12263|657[0n]bp
Dyscophellus phraxanorDHJ02|[8473]|99-SRNP-13041|603[0n]bp
Dyscophellus phraxanorDHJ02|[8474]|99-SRNP-12798|606[0n]bp
Dyscophellus phraxanorDHJ02|[8475]|00-SRNP-11209|582[0n]bp
Dyscophellus phraxanorDHJ02|[8476]|99-SRNP-12200|633[1n]bp
Dyscophellus phraxanorDHJ02|[8477]|99-SRNP-13130|627[0n]bp
Dyscophellus phraxanorDHJ02|[8478]|01-SRNP-25001|588[0n]bp
Dyscophellus phraxanorDHJ02|[8479]|01-SRNP-22878|594[0n]bp
Dyscophellus phraxanorDHJ02|[8480]|98-SRNP-6998|609[0n]bp
Dyscophellus phraxanorDHJ02|[8481]|97-SRNP-6566|609[0n]bp
Dyscophellus phraxanorDHJ02|[8482]|97-SRNP-6747|606[0n]bp
Dyscophellus phraxanorDHJ02|[8483]|00-SRNP-4551|606[0n]bp
Dyscophellus phraxanorDHJ02|[8484]|02-SRNP-3885|624[0n]bp
Dyscophellus phraxanorDHJ02|[8485]|08-SRNP-65057|645[0n]bp
Dyscophellus phraxanorDHJ02|[8486]|08-SRNP-2144|645[0n]bp
Dyscophellus phraxanorDHJ02|[8487]|01-SRNP-3380|669[0n]bp
Dyscophellus phraxanorDHJ02|[8488]|01-SRNP-3900|669[0n]bp
Dyscophellus phraxanorDHJ02|[8489]|01-SRNP-3947|669[0n]bp
Dyscophellus phraxanorDHJ02|[8490]|07-SRNP-30937|669[0n]bp
Dyscophellus phraxanorDHJ02|[8491]|07-SRNP-45419|669[0n]bp
Dyscophellus phraxanorDHJ02|[8492]|07-SRNP-65225|669[0n]bp
Dyscophellus phraxanorDHJ02|[8493]|99-SRNP-13193|669[0n]bp
Dyscophellus phraxanorDHJ02|[8494]|99-SRNP-15209|669[0n]bp
Dyscophellus phraxanorDHJ02|[8495]|07-SRNP-65246|669[0n]bp
Dyscophellus phraxanorDHJ02|[8496]|07-SRNP-33141|669[0n]bp

Dyscophellus phraxanorDHJ02|[8494]|99-SRNP-15209|669[0n]bp
Dyscophellus phraxanorDHJ02|[8495]|07-SRNP-65246|669[0n]bp
Dyscophellus phraxanorDHJ02|[8496]|07-SRNP-33141|669[0n]bp
Dyscophellus phraxanorDHJ02|[8497]|07-SRNP-65247|669[0n]bp
Dyscophellus phraxanorDHJ02|[8498]|08-SRNP-4730|669[0n]bp
Dyscophellus phraxanorDHJ02|[8499]|08-SRNP-65758|669[0n]bp
Dyscophellus phraxanorDHJ02|[8500]|08-SRNP-65873|669[0n]bp
Dyscophellus phraxanorDHJ02|[8501]|99-SRNP-12518|669[0n]bp
Dyscophellus phraxanorDHJ02|[8502]|99-SRNP-12486|669[0n]bp
Dyscophellus phraxanorDHJ02|[8503]|98-SRNP-15081|669[0n]bp
Dyscophellus phraxanorDHJ02|[8504]|00-SRNP-12297|669[0n]bp
Dyscophellus phraxanorDHJ02|[8505]|01-SRNP-3326|669[0n]bp
Dyscophellus phraxanorDHJ02|[8506]|99-SRNP-12366|669[0n]bp
Dyscophellus phraxanorDHJ02|[8507]|99-SRNP-12485|669[0n]bp
Dyscophellus phraxanorDHJ02|[8508]|99-SRNP-12722|669[0n]bp
Dyscophellus phraxanorDHJ02|[8509]|99-SRNP-12705|669[0n]bp
Dyscophellus phraxanorDHJ02|[8510]|99-SRNP-12881|669[0n]bp
Dyscophellus phraxanorDHJ02|[8511]|99-SRNP-12370|669[0n]bp
Dyscophellus phraxanorDHJ02|[8512]|99-SRNP-12381|669[0n]bp
Dyscophellus phraxanorDHJ02|[8513]|99-SRNP-12380|669[0n]bp
Dyscophellus phraxanorDHJ02|[8514]|99-SRNP-12383|669[0n]bp
Dyscophellus phraxanorDHJ02|[8515]|08-SRNP-66201|669[0n]bp
Dyscophellus phraxanorDHJ02|[8516]|09-SRNP-56856|669[0n]bp
Dyscophellus phraxanorDHJ02|[8517]|09-SRNP-57092|669[0n]bp
Dyscophellus phraxanorDHJ02|[8518]|00-SRNP-22220|669[0n]bp
Dyscophellus phraxanorDHJ02|[8519]|99-SRNP-12282|669[0n]bp
Dyscophellus phraxanorDHJ02|[8520]|99-SRNP-12435|669[0n]bp
Dyscophellus phraxanorDHJ02|[8521]|99-SRNP-4100|669[0n]bp
Dyscophellus phraxanorDHJ02|[8522]|99-SRNP-4009|669[0n]bp
Dyscophellus phraxanorDHJ02|[8523]|01-SRNP-22948|669[0n]bp
Dyscophellus phraxanorDHJ02|[8524]|02-SRNP-18890|669[0n]bp
Dyscophellus phraxanorDHJ02|[8525]|99-SRNP-13335|669[0n]bp
Dyscophellus phraxanorDHJ02|[8526]|99-SRNP-13173|669[0n]bp
Dyscophellus phraxanorDHJ02|[8527]|01-SRNP-1865|669[0n]bp
Dyscophellus phraxanorDHJ02|[8528]|99-SRNP-4099|624[0n]bp
Dyscophellus phraxanorDHJ02|[8529]|00-SRNP-12748|669[0n]bp
Dyscophellus phraxanorDHJ02|[8530]|02-SRNP-3831|624[0n]bp
Dyscophellus phraxanorDHJ02|[8531]|99-SRNP-12291|630[0n]bp
Dyscophellus phraxanorDHJ02|[8532]|98-SRNP-15001|642[0n]bp
Dyscophellus phraxanorDHJ02|[8533]|99-SRNP-4098|657[0n]bp
Dyscophellus phraxanorDHJ02|[8534]|98-SRNP-6939|657[1n]bp
Dyscophellus phraxanorDHJ02|[8535]|97-SRNP-6580|606[0n]bp
Dyscophellus phraxanorDHJ02|[8536]|97-SRNP-6790|606[0n]bp
Dyscophellus phraxanorDHJ02|[8537]|99-SRNP-5790|654[3n]bp
Dyscophellus phraxanorDHJ02|[8538]|98-SRNP-6941|633[0n]bp
Dyscophellus phraxanorDHJ02|[8539]|97-SRNP-6220|609[0n]bp
Dyscophellus phraxanorDHJ02|[8540]|99-SRNP-12194|606[0n]bp
Dyscophellus phraxanorDHJ02|[8541]|00-SRNP-14791|579[0n]bp
Dyscophellus phraxanorDHJ02|[8542]|01-SRNP-497|669[0n]bp
Dyscophellus phraxanorDHJ02|[8543]|01-SRNP-9119|669[0n]bp
Dyscophellus phraxanorDHJ02|[8544]|00-SRNP-21016|669[0n]bp
Dyscophellus phraxanorDHJ02|[8545]|99-SRNP-12367|669[0n]bp
Dyscophellus phraxanorDHJ02|[8546]|99-SRNP-12688|669[0n]bp
Dyscophellus phraxanorDHJ02|[8547]|07-SRNP-45265|669[0n]bp
Dyscophellus phraxanorDHJ02|[8548]|07-SRNP-45263|669[0n]bp
Dyscophellus phraxanorDHJ02|[8549]|09-SRNP-72343|669[0n]bp
Dyscophellus phraxanorDHJ02|[8550]|97-SRNP-6512|627[1n]bp
Dyscophellus phraxanorDHJ02|[8551]|09-SRNP-65857|669[0n]bp
Dyscophellus phraxanorDHJ02|[8552]|09-SRNP-32343|669[0n]bp
Dyscophellus phraxanorDHJ02|[8553]|09-SRNP-67110|669[0n]bp
Dyscophellus phraxanorDHJ02|[8554]|09-SRNP-72763|669[0n]bp
Dyscophellus phraxanorDHJ02|[8555]|07-SRNP-45964|669[0n]bp
Dyscophellus phraxanorDHJ02|[8556]|07-SRNP-45952|669[0n]bp
Dyscophellus phraxanorDHJ02|[8557]|01-SRNP-2916|669[0n]bp
Dyscophellus phraxanorDHJ02|[8558]|02-SRNP-2635|669[0n]bp
Dyscophellus phraxanorDHJ02|[8559]|00-SRNP-1057|669[0n]bp
Dyscophellus phraxanorDHJ02|[8560]|01-SRNP-1058|669[0n]bp
Dyscophellus phraxanorDHJ02|[8561]|00-SRNP-12293|669[0n]bp
Dyscophellus phraxanorDHJ02|[8562]|99-SRNP-13496|669[0n]bp
Dyscophellus phraxanorDHJ02|[8563]|99-SRNP-13336|669[0n]bp
Dyscophellus phraxanorDHJ02|[8564]|99-SRNP-13063|669[0n]bp
Dyscophellus phraxanorDHJ02|[8565]|98-SRNP-15079|669[0n]bp
Dyscophellus phraxanorDHJ02|[8566]|99-SRNP-12290|669[0n]bp
Dyscophellus phraxanorDHJ02|[8567]|04-SRNP-34379|669[0n]bp
Dyscophellus phraxanorDHJ02|[8568]|02-SRNP-481|669[0n]bp
Dyscophellus phraxanorDHJ02|[8569]|01-SRNP-22947|669[0n]bp
Dyscophellus phraxanorDHJ02|[8570]|01-SRNP-23370|669[0n]bp
Dyscophellus phraxanorDHJ02|[8571]|01-SRNP-3488|669[0n]bp
Dyscophellus phraxanorDHJ02|[8572]|00-SRNP-4520|669[0n]bp
Dyscophellus phraxanorDHJ02|[8573]|99-SRNP-12028|657[0n]bp
Dyscophellus phraxanorDHJ02|[8574]|99-SRNP-13339|606[0n]bp
Dyscophellus phraxanorDHJ02|[8575]|00-SRNP-4195|609[1n]bp
Dyscophellus phraxanorDHJ02|[8576]|09-SRNP-68551|669[0n]bp
Dyscophellus Burns02|[8577]|05-SRNP-33035|669[0n]bp
Dyscophellus Burns02|[8578]|04-SRNP-61423|687[0n]bp
Dyscophellus Burns02|[8579]|00-SRNP-21086|669[0n]bp
Dyscophellus Burns02|[8580]|98-SRNP-6071|669[0n]bp
Dyscophellus Burns02|[8581]|08-SRNP-70869|669[0n]bp
Dyscophellus Burns02|[8582]|06-SRNP-4389|669[0n]bp
Dyscophellus Burns02|[8583]|02-SRNP-6754|669[0n]bp
Dyscophellus Burns02|[8584]|03-SRNP-12963.1|669[0n]bp
Dyscophellus Burns02|[8585]|04-SRNP-32586|630[0n]bp
Dyscophellus Burns02|[8586]|06-SRNP-812|315[0n]bp
Dyscophellus Burns02|[8587]|02-SRNP-7471|657[0n]bp
Dyscophellus Burns02|[8588]|02-SRNP-6761|657[0n]bp
Dyscophellus Burns02|[8589]|04-SRNP-41726|669[0n]bp
Dyscophellus Burns02|[8590]|04-SRNP-3811|669[0n]bp
Dyscophellus Burns02|[8591]|04-SRNP-41073|669[0n]bp
Dyscophellus Burns02|[8592]|05-SRNP-40223|669[0n]bp
Dyscophellus Burns02|[8593]|02-SRNP-7460|669[0n]bp
Dyscophellus Burns02|[8594]|01-SRNP-468|669[0n]bp
Dyscophellus Burns02|[8595]|03-SRNP-11903|669[0n]bp
Dyscophellus Burns02|[8596]|02-SRNP-7467|669[0n]bp

Dyscophellus Burns02|[8594]|01-SRNP-468|669[0n]bp
Dyscophellus Burns02|[8595]|03-SRNP-11903|669[0n]bp
Dyscophellus Burns02|[8596]|02-SRNP-7467|669[0n]bp
Dyscophellus Burns02|[8597]|01-SRNP-5675|669[0n]bp
Dyscophellus Burns02|[8598]|06-SRNP-40602|669[0n]bp
Dyscophellus Burns02|[8599]|08-SRNP-70835|669[0n]bp
Dyscophellus Burns02|[8600]|08-SRNP-65702|669[0n]bp
Dyscophellus porcius|[8601]|01-SRNP-5603|630[0n]bp
Dyscophellus porcius|[8602]|07-SRNP-42003|669[0n]bp
Dyscophellus Burns01|[8603]|08-SRNP-4491|669[0n]bp
Dyscophellus Burns01|[8604]|08-SRNP-914|669[1n]bp
Dyscophellus Burns01|[8605]|08-SRNP-32137|669[0n]bp
Dyscophellus Burns01|[8606]|08-SRNP-70995|669[0n]bp
Dyscophellus Burns01|[8607]|08-SRNP-65309|669[0n]bp
Dyscophellus Burns01|[8608]|08-SRNP-790|669[0n]bp
Dyscophellus Burns01|[8609]|08-SRNP-21088|669[0n]bp
Dyscophellus Burns01|[8610]|08-SRNP-21089|669[0n]bp
Dyscophellus Burns01|[8611]|07-SRNP-45367|669[0n]bp
Dyscophellus Burns01|[8612]|07-SRNP-33600|669[0n]bp
Dyscophellus Burns01|[8613]|07-SRNP-2605|669[0n]bp
Dyscophellus Burns01|[8614]|07-SRNP-45366|669[0n]bp
Dyscophellus Burns01|[8615]|07-SRNP-2606|669[0n]bp
Dyscophellus Burns01|[8616]|07-SRNP-1820|669[0n]bp
Dyscophellus Burns01|[8617]|07-SRNP-31532|669[0n]bp
Dyscophellus Burns01|[8618]|07-SRNP-1821|669[0n]bp
Dyscophellus Burns01|[8619]|06-SRNP-4434|669[0n]bp
Dyscophellus Burns01|[8620]|06-SRNP-45197|669[0n]bp
Dyscophellus Burns01|[8621]|06-SRNP-2566|669[0n]bp
Dyscophellus Burns01|[8622]|04-SRNP-1382|669[0n]bp
Dyscophellus Burns01|[8623]|04-SRNP-3019|669[0n]bp
Dyscophellus Burns01|[8624]|04-SRNP-1472|669[0n]bp
Dyscophellus Burns01|[8625]|04-SRNP-1145|669[0n]bp
Dyscophellus Burns01|[8626]|04-SRNP-41583|669[0n]bp
Dyscophellus Burns01|[8627]|05-SRNP-34746|669[2n]bp
Dyscophellus Burns01|[8628]|02-SRNP-17977|645[0n]bp
Dyscophellus Burns01|[8629]|03-SRNP-5975|645[0n]bp
Dyscophellus Burns01|[8630]|01-SRNP-1161|639[0n]bp
Dyscophellus Burns01|[8631]|01-SRNP-22233|639[0n]bp
Dyscophellus Burns01|[8632]|07-SRNP-20844|645[0n]bp
Dyscophellus Burns01|[8633]|08-SRNP-70860|645[0n]bp
Dyscophellus Burns01|[8634]|08-SRNP-21379|669[0n]bp
Dyscophellus Burns01|[8635]|08-SRNP-21611|669[0n]bp
Dyscophellus Burns01|[8636]|08-SRNP-915|669[0n]bp
Dyscophellus Burns01|[8637]|08-SRNP-1071|669[0n]bp
Dyscophellus Burns01|[8638]|08-SRNP-32136|669[0n]bp
Dyscophellus Burns01|[8639]|09-SRNP-57048|669[0n]bp
Dyscophellus Burns01|[8640]|07-SRNP-42747|669[0n]bp
Dyscophellus Burns01|[8641]|09-SRNP-67513|669[0n]bp
Dyscophellus Burns01|[8642]|06-SRNP-5185|627[0n]bp
Dyscophellus Burns01|[8643]|08-SRNP-2351|669[1n]bp
Dyscophellus Burns01|[8644]|08-SRNP-70059|669[0n]bp
Dyscophellus Burns01|[8645]|08-SRNP-1139|669[0n]bp
Dyscophellus Burns01|[8646]|08-SRNP-1140|669[0n]bp
Dyscophellus Burns01|[8647]|07-SRNP-46539|669[0n]bp
Dyscophellus Burns01|[8648]|07-SRNP-65613|669[0n]bp
Dyscophellus Burns01|[8649]|06-SRNP-5990|669[0n]bp
Dyscophellus Burns01|[8650]|06-SRNP-31676|669[0n]bp
Dyscophellus Burns01|[8651]|06-SRNP-2371|669[0n]bp
Dyscophellus Burns01|[8652]|07-SRNP-45716|660[0n]bp
Dyscophellus Burns01|[8653]|03-SRNP-1735|528[1n]bp
Dyscophellus Burns01|[8654]|06-SRNP-5186|624[0n]bp
Dyscophellus Burns01|[8655]|08-SRNP-1423|645[0n]bp
Dyscophellus Burns01|[8656]|08-SRNP-1424|645[0n]bp
Dyscophellus Burns01|[8657]|08-SRNP-70994|669[0n]bp
Dyscophellus Burns01|[8658]|07-SRNP-42565|669[0n]bp
Dyscophellus Burns01|[8659]|08-SRNP-40847|669[3n]bp
Dyscophellus Burns01|[8660]|07-SRNP-3025|669[0n]bp
Dyscophellus Burns01|[8661]|07-SRNP-833|669[0n]bp
Dyscophellus Burns01|[8662]|06-SRNP-4432|669[0n]bp
Dyscophellus Burns01|[8663]|06-SRNP-4193|669[0n]bp
Dyscophellus Burns01|[8664]|04-SRNP-34190|669[0n]bp
Dyscophellus Burns01|[8665]|07-SRNP-45717|660[0n]bp
Dyscophellus Burns01|[8666]|08-SRNP-1274|669[0n]bp
Dyscophellus Burns01|[8667]|08-SRNP-70502|669[0n]bp
Dyscophellus Burns01|[8668]|08-SRNP-35933|669[0n]bp
Dyscophellus Burns01|[8669]|09-SRNP-68136|669[0n]bp
Dyscophellus ramon|[8670]|01-SRNP-3569|669[0n]bp
Dyscophellus ramon|[8671]|09-SRNP-40055|669[0n]bp
Dyscophellus ramon|[8672]|03-SRNP-5396|654[1n]bp
Dyscophellus ramon|[8673]|01-SRNP-25139|669[0n]bp
Dyscophellus ramon|[8674]|01-SRNP-1770|669[0n]bp
Dyscophellus ramon|[8675]|01-SRNP-22279|669[0n]bp
Dyscophellus ramon|[8676]|01-SRNP-5763|669[0n]bp
Dyscophellus ramon|[8677]|00-SRNP-12919|669[0n]bp
Dyscophellus ramon|[8678]|07-SRNP-60549|669[0n]bp
Dyscophellus ramon|[8679]|03-SRNP-5318|630[0n]bp
Dyscophellus ramon|[8680]|01-SRNP-24190|630[1n]bp
Dyscophellus ramon|[8681]|08-SRNP-1584|669[1n]bp
Dyscophellus ramon|[8682]|08-SRNP-71274|666[0n]bp
Dyscophellus ramon|[8683]|03-SRNP-30935|669[0n]bp
Dyscophellus ramon|[8684]|02-SRNP-169|669[0n]bp
Dyscophellus ramon|[8685]|08-SRNP-24064|633[0n]bp
Dyscophellus ramon|[8686]|06-SRNP-370|669[0n]bp
Dyscophellus ramon|[8687]|08-SRNP-24061|636[0n]bp
Dyscophellus ramon|[8688]|08-SRNP-24063|669[0n]bp
Dyscophellus ramon|[8689]|03-SRNP-30164|669[0n]bp
Dyscophellus ramon|[8690]|00-SRNP-21755|669[0n]bp
Dyscophellus ramon|[8691]|02-SRNP-18736|669[0n]bp
Dyscophellus ramon|[8692]|02-SRNP-1636|669[0n]bp
Dyscophellus ramon|[8693]|02-SRNP-4089|669[0n]bp
Dyscophellus ramon|[8694]|07-SRNP-20011|669[0n]bp
Dyscophellus ramon|[8695]|05-SRNP-42006|669[0n]bp
Dyscophellus ramon|[8696]|07-SRNP-41237|669[0n]bp

Dyscophellus ramon|[8694]|07-SRNP-20011|669[0n]bp
Dyscophellus ramon|[8695]|05-SRNP-42006|669[0n]bp
Dyscophellus ramon|[8696]|07-SRNP-41237|669[0n]bp
Dyscophellus ramon|[8697]|07-SRNP-45229|669[0n]bp
Dyscophellus ramon|[8698]|08-SRNP-4118|669[0n]bp
Dyscophellus ramon|[8699]|02-SRNP-33929|669[0n]bp
Dyscophellus ramon|[8700]|01-SRNP-22278|669[0n]bp
Dyscophellus ramon|[8701]|02-SRNP-20433|669[0n]bp
Dyscophellus ramon|[8702]|00-SRNP-22195|669[0n]bp
Dyscophellus ramon|[8703]|02-SRNP-7461|669[0n]bp
Dyscophellus ramon|[8704]|02-SRNP-7470|669[0n]bp
Dyscophellus ramon|[8705]|01-SRNP-22392|669[0n]bp
Dyscophellus ramon|[8706]|02-SRNP-3214|669[0n]bp
Dyscophellus ramon|[8707]|01-SRNP-3012|669[0n]bp
Dyscophellus ramon|[8708]|01-SRNP-25138|669[0n]bp
Dyscophellus ramon|[8709]|02-SRNP-7463|669[0n]bp
Dyscophellus ramon|[8710]|02-SRNP-7117|669[0n]bp
Dyscophellus ramon|[8711]|00-SRNP-11389|669[0n]bp
Dyscophellus ramon|[8712]|02-SRNP-20419|669[0n]bp
Dyscophellus ramon|[8713]|02-SRNP-19579|669[0n]bp
Dyscophellus ramon|[8714]|01-SRNP-22456|669[0n]bp
Dyscophellus ramon|[8715]|06-SRNP-77|669[0n]bp
Dyscophellus ramon|[8716]|04-SRNP-60594|669[0n]bp
Dyscophellus ramon|[8717]|04-SRNP-3520|669[0n]bp
Dyscophellus ramon|[8718]|03-SRNP-5316|669[0n]bp
Dyscophellus ramon|[8719]|02-SRNP-1606|669[0n]bp
Dyscophellus ramon|[8720]|97-SRNP-6149|666[0n]bp
Dyscophellus ramon|[8721]|01-SRNP-11993|654[0n]bp
Dyscophellus ramon|[8722]|08-SRNP-4781|645[0n]bp
Dyscophellus ramon|[8723]|08-SRNP-23241|624[0n]bp
Dyscophellus ramon|[8724]|08-SRNP-58183|669[0n]bp
Dyscophellus ramon|[8725]|09-SRNP-67984|669[0n]bp
Phareas coeleste|[8726]|02-SRNP-35232|540[0n]bp
Phareas coeleste|[8727]|02-SRNP-35231|576[0n]bp
Phareas coeleste|[8728]|04-SRNP-56226|669[0n]bp
Phareas coeleste|[8729]|05-SRNP-30644|669[0n]bp
Phareas coeleste|[8730]|05-SRNP-30576|669[0n]bp
Phareas coeleste|[8731]|05-SRNP-30642|669[0n]bp
Phareas coeleste|[8732]|05-SRNP-30469|669[0n]bp
Phareas coeleste|[8733]|05-SRNP-30643|669[0n]bp
Phareas coeleste|[8734]|05-SRNP-30577|669[0n]bp
Phocides belus|[8735]|06-SRNP-55175|642[0n]bp
Phocides belus|[8736]|06-SRNP-55419|642[0n]bp
Phocides belus|[8737]|02-SRNP-10072|663[0n]bp
Phocides belus|[8738]|06-SRNP-55185|669[1n]bp
Phocides belus|[8739]|96-SRNP-51|612[0n]bp
Phocides belus|[8740]|01-SRNP-18748|669[0n]bp
Phocides belus|[8741]|96-SRNP-12439|669[0n]bp
Phocides belus|[8742]|96-SRNP-12538|669[0n]bp
Phocides belus|[8743]|96-SRNP-12436|669[0n]bp
Phocides belus|[8744]|07-SRNP-55324|669[0n]bp
Phocides belus|[8745]|07-SRNP-55469|669[0n]bp
Phocides belus|[8746]|06-SRNP-55343|669[0n]bp
Phocides belus|[8747]|01-SRNP-17031|669[0n]bp
Phocides belus|[8748]|07-SRNP-56444|669[0n]bp
Phocides belus|[8749]|96-SRNP-12539|630[0n]bp
Phocides Warren01|[8750]|00-SRNP-15104|639[0n]bp
Phocides Warren01|[8751]|00-SRNP-15731|639[0n]bp
Phocides Warren01|[8752]|01-SRNP-17035|645[0n]bp
Phocides Warren01|[8753]|00-SRNP-8936|645[0n]bp
Phocides Warren01|[8754]|00-SRNP-15124|594[0n]bp
Phocides Warren01|[8755]|00-SRNP-8940|669[0n]bp
Phocides Warren01|[8756]|00-SRNP-15109|669[0n]bp
Phocides Warren01|[8757]|00-SRNP-15096|669[0n]bp
Phocides Warren01|[8758]|00-SRNP-15097|669[0n]bp
Phocides Warren01|[8759]|00-SRNP-15177|669[0n]bp
Phocides Warren01|[8760]|00-SRNP-15103|669[0n]bp
Phocides Warren01|[8761]|01-SRNP-17848|669[0n]bp
Phocides Warren01|[8762]|00-SRNP-15187|669[0n]bp
Phocides Warren01|[8763]|00-SRNP-15115|669[0n]bp
Phocides Warren01|[8764]|01-SRNP-16953|669[0n]bp
Phocides Warren01|[8765]|00-SRNP-15106|669[0n]bp
Phocides Warren01|[8766]|00-SRNP-15221|669[0n]bp
Phocides Warren01|[8767]|00-SRNP-15114|669[0n]bp
Phocides Warren01|[8768]|00-SRNP-15894|669[0n]bp
Phocides Warren01|[8769]|00-SRNP-15110|669[0n]bp
Phocides Warren01|[8770]|01-SRNP-16950|669[0n]bp
Phocides Warren01|[8771]|00-SRNP-15186|669[0n]bp
Phocides pigmalionDHJ01|[8772]|95-SRNP-7824|570[0n]bp
Phocides pigmalionDHJ01|[8773]|00-SRNP-4542|567[0n]bp
Phocides Warren01|[8774]|00-SRNP-15238|576[0n]bp
Phocides pigmalionDHJ01|[8775]|02-SRNP-14336|576[0n]bp
Phocides pigmalionDHJ01|[8776]|02-SRNP-14447|669[0n]bp
Phocides pigmalionDHJ01|[8777]|00-SRNP-16167|669[0n]bp
Phocides pigmalionDHJ01|[8778]|96-SRNP-9016|669[0n]bp
Phocides pigmalionDHJ01|[8779]|00-SRNP-4322|669[0n]bp
Phocides pigmalionDHJ01|[8780]|96-SRNP-9017|669[0n]bp
Phocides pigmalionDHJ01|[8781]|96-SRNP-9015|669[0n]bp
Phocides pigmalionDHJ01|[8782]|04-SRNP-14106|669[0n]bp
Phocides pigmalionDHJ01|[8783]|05-SRNP-61368|669[0n]bp
Phocides pigmalionDHJ01|[8784]|07-SRNP-42344|669[0n]bp
Phocides pigmalionDHJ01|[8785]|07-SRNP-42389|669[0n]bp
Phocides pigmalionDHJ01|[8786]|07-SRNP-42343|669[0n]bp
Phocides pigmalionDHJ01|[8787]|07-SRNP-42390|669[0n]bp
Phocides pigmalionDHJ01|[8788]|08-SRNP-55462|669[0n]bp
Phocides pigmalionDHJ01|[8789]|08-SRNP-5779|669[0n]bp
Phocides belus|[8790]|05-SRNP-64113|633[0n]bp
Phocides belus|[8791]|06-SRNP-60076|609[0n]bp
Phocides pigmalionDHJ01|[8792]|05-SRNP-6300|627[0n]bp
Phocides pigmalionDHJ01|[8793]|00-SRNP-4541|639[0n]bp
Phocides pigmalionDHJ01|[8794]|99-SRNP-16188|639[0n]bp
Phocides pigmalionDHJ01|[8795]|06-SRNP-60042|606[0n]bp
Phocides Warren01|[8796]|00-SRNP-15726|642[0n]bp

Phocides pigmalionDHJ01|[8794]|99-SRNP-16188|639[0n]bp
Phocides pigmalionDHJ01|[8795]|06-SRNP-60042|606[0n]bp
Phocides Warren01|[8796]|00-SRNP-15726|642[0n]bp
Phocides belus|[8797]|06-SRNP-59670|597[0n]bp
Phocides belus|[8798]|05-SRNP-66416|669[1n]bp
Phocides belus|[8799]|05-SRNP-66414|669[0n]bp
Phocides belus|[8800]|06-SRNP-55154|669[1n]bp
Phocides belus|[8801]|96-SRNP-12435.1|669[0n]bp
Phocides belus|[8802]|99-SRNP-8611|669[0n]bp
Phocides belus|[8803]|06-SRNP-60276|615[1n]bp
Phocides belus|[8804]|01-SRNP-18749|648[0n]bp
Phocides belus|[8805]|94-SRNP-7489|645[0n]bp
Phocides belus|[8806]|01-SRNP-17030|600[0n]bp
Phocides belus|[8807]|05-SRNP-55231|669[0n]bp
Phocides belus|[8808]|07-SRNP-55795|669[0n]bp
Phocides belus|[8809]|07-SRNP-55340|669[0n]bp
Phocides belus|[8810]|07-SRNP-55373|669[0n]bp
Phocides belus|[8811]|00-SRNP-20234|573[0n]bp
Phocides belus|[8812]|00-SRNP-20231|669[0n]bp
Phocides belus|[8813]|96-SRNP-12432|669[0n]bp
Phocides belus|[8814]|07-SRNP-55377|669[0n]bp
Phocides belus|[8815]|96-SRNP-9221|669[0n]bp
Phocides belus|[8816]|97-SRNP-248|669[0n]bp
Phocides belus|[8817]|00-SRNP-3582|669[0n]bp
Phocides belus|[8818]|93-SRNP-6630|669[0n]bp
Phocides Warren01|[8819]|00-SRNP-15102|669[0n]bp
Phocides Warren01|[8820]|00-SRNP-15185|669[0n]bp
Phocides Warren01|[8821]|00-SRNP-15194|669[0n]bp
Phocides Warren01|[8822]|00-SRNP-15236|669[0n]bp
Phocides belus|[8823]|06-SRNP-55176|669[0n]bp
Phocides belus|[8824]|06-SRNP-55418|669[0n]bp
Phocides belus|[8825]|05-SRNP-55233|669[0n]bp
Phocides belus|[8826]|05-SRNP-55235|669[0n]bp
Phocides belus|[8827]|06-SRNP-55173|669[1n]bp
Phocides belus|[8828]|05-SRNP-66417|669[2n]bp
Phocides belus|[8829]|08-SRNP-55675|669[0n]bp
Phocides belus|[8830]|09-SRNP-55421|642[0n]bp
Phocides pigmalionDHJ02|[8831]|06-SRNP-34234|663[0n]bp
Phocides pigmalionDHJ02|[8832]|06-SRNP-44090|615[0n]bp
Phocides pigmalionDHJ02|[8833]|08-SRNP-41365|669[0n]bp
Phocides pigmalionDHJ02|[8834]|08-SRNP-41366|669[0n]bp
Phocides pigmalionDHJ02|[8835]|07-SRNP-42096|669[0n]bp
Phocides pigmalionDHJ02|[8836]|07-SRNP-42095|669[0n]bp
Phocides pigmalionDHJ02|[8837]|07-SRNP-943|669[0n]bp
Phocides pigmalionDHJ02|[8838]|07-SRNP-1304|669[0n]bp
Phocides pigmalionDHJ02|[8839]|07-SRNP-1303|669[0n]bp
Phocides pigmalionDHJ02|[8840]|06-SRNP-34235|669[0n]bp
Phocides pigmalionDHJ02|[8841]|06-SRNP-43725|669[0n]bp
Phocides pigmalionDHJ02|[8842]|06-SRNP-43751|669[0n]bp
Phocides pigmalionDHJ02|[8843]|06-SRNP-34232|669[0n]bp
Phocides pigmalionDHJ02|[8844]|06-SRNP-34233|669[0n]bp
Phocides pigmalionDHJ02|[8845]|06-SRNP-41789|669[0n]bp
Phocides pigmalionDHJ02|[8846]|00-SRNP-11147|669[0n]bp
Phocides pigmalionDHJ02|[8847]|00-SRNP-12628|669[0n]bp
Phocides pigmalionDHJ02|[8848]|08-SRNP-66135|669[0n]bp
Phocides pigmalionDHJ02|[8849]|09-SRNP-21125|669[0n]bp
Phocides Burns01|[8850]|05-SRNP-41960|669[0n]bp
Phocides Burns01|[8851]|05-SRNP-2576|669[0n]bp
Phocides Burns01|[8852]|04-SRNP-40653|669[0n]bp
Phocides Burns01|[8853]|06-SRNP-42438|630[0n]bp
Phocides Burns01|[8854]|06-SRNP-42881|669[0n]bp
Phocides lilea|[8855]|02-SRNP-4215|645[1n]bp
Phocides lilea|[8856]|05-SRNP-36020|642[0n]bp
Phocides lilea|[8857]|02-SRNP-4582|642[1n]bp
Phocides lilea|[8858]|07-SRNP-45148|669[0n]bp
Phocides lilea|[8859]|06-SRNP-55337|603[0n]bp
Phocides lilea|[8860]|08-SRNP-45148|669[0n]bp
Phocides lilea|[8861]|08-SRNP-55012|669[0n]bp
Phocides lilea|[8862]|06-SRNP-36875|669[0n]bp
Phocides lilea|[8863]|07-SRNP-1991|669[0n]bp
Phocides lilea|[8864]|05-SRNP-45014|669[0n]bp
Phocides lilea|[8865]|05-SRNP-45013|669[0n]bp
Phocides lilea|[8866]|05-SRNP-45012|669[0n]bp
Phocides lilea|[8867]|05-SRNP-55954|669[0n]bp
Phocides lilea|[8868]|05-SRNP-31874|669[0n]bp
Phocides lilea|[8869]|05-SRNP-42094|669[0n]bp
Phocides lilea|[8870]|04-SRNP-48827|669[0n]bp
Phocides lilea|[8871]|04-SRNP-22905|669[0n]bp
Phocides lilea|[8872]|04-SRNP-49744|669[0n]bp
Phocides lilea|[8873]|06-SRNP-55641|669[2n]bp
Phocides lilea|[8874]|05-SRNP-64067|669[0n]bp
Phocides lilea|[8875]|06-SRNP-12035|669[0n]bp
Phocides lilea|[8876]|05-SRNP-64081|669[0n]bp
Phocides lilea|[8877]|05-SRNP-64071|669[0n]bp
Phocides lilea|[8878]|06-SRNP-12616|669[0n]bp
Phocides lilea|[8879]|05-SRNP-64077|669[0n]bp
Phocides lilea|[8880]|07-SRNP-56871|669[0n]bp
Phocides lilea|[8881]|08-SRNP-1660|669[0n]bp
Phocides lilea|[8882]|08-SRNP-70870|669[0n]bp
Phocides lilea|[8883]|07-SRNP-43071|669[0n]bp
Phocides lilea|[8884]|08-SRNP-45036|669[0n]bp
Phocides lilea|[8885]|08-SRNP-72107|669[0n]bp
Phocides lilea|[8886]|08-SRNP-71270|669[0n]bp
Phocides lilea|[8887]|08-SRNP-71658|669[0n]bp
Phocides lilea|[8888]|08-SRNP-72658|669[0n]bp
Phocides lilea|[8889]|08-SRNP-37041|669[0n]bp
Phocides nigrescens|[8890]|02-SRNP-24513|642[0n]bp
Phocides nigrescens|[8891]|03-SRNP-1248|639[0n]bp
Phocides nigrescens|[8892]|09-SRNP-20813|669[1n]bp
Phocides nigrescens|[8893]|06-SRNP-6937|654[0n]bp
Phocides nigrescens|[8894]|06-SRNP-9777|669[0n]bp
Phocides nigrescens|[8895]|07-SRNP-60225|669[0n]bp
Phocides nigrescens|[8896]|08-SRNP-30726|669[0n]bp

Phocides nigrescens|[8894]|06-SRNP-9777|669[0n]bp
Phocides nigrescens|[8895]|07-SRNP-60225|669[0n]bp
Phocides nigrescens|[8896]|08-SRNP-30726|669[0n]bp
Phocides nigrescens|[8897]|08-SRNP-30757|669[0n]bp
Phocides nigrescens|[8898]|07-SRNP-1654|669[0n]bp
Phocides nigrescens|[8899]|07-SRNP-1584|669[0n]bp
Phocides nigrescens|[8900]|07-SRNP-1585|669[0n]bp
Phocides nigrescens|[8901]|07-SRNP-1599|669[0n]bp
Phocides nigrescens|[8902]|07-SRNP-3394|669[0n]bp
Phocides nigrescens|[8903]|07-SRNP-45870|669[0n]bp
Phocides nigrescens|[8904]|08-SRNP-1566|669[0n]bp
Phocides nigrescens|[8905]|08-SRNP-30756|669[0n]bp
Phocides nigrescens|[8906]|08-SRNP-2469|669[0n]bp
Phocides nigrescens|[8907]|08-SRNP-23739|669[0n]bp
Phocides nigrescens|[8908]|09-SRNP-20870|669[0n]bp
Phocides nigrescens|[8909]|09-SRNP-80647|669[0n]bp
Phocides nigrescens|[8910]|08-SRNP-33055|669[0n]bp
Phocides nigrescens|[8911]|08-SRNP-6886|669[0n]bp
Phocides nigrescens|[8912]|06-SRNP-4789|669[0n]bp
Phocides nigrescens|[8913]|07-SRNP-1656|669[0n]bp
Phocides nigrescens|[8914]|06-SRNP-4244|669[0n]bp
Phocides nigrescens|[8915]|06-SRNP-6938|669[0n]bp
Phocides nigrescens|[8916]|06-SRNP-7174|669[0n]bp
Phocides nigrescens|[8917]|06-SRNP-6010|669[0n]bp
Phocides nigrescens|[8918]|06-SRNP-6017|669[0n]bp
Phocides nigrescens|[8919]|06-SRNP-6172|669[0n]bp
Phocides nigrescens|[8920]|06-SRNP-6612|669[0n]bp
Phocides nigrescens|[8921]|06-SRNP-23407|669[0n]bp
Phocides nigrescens|[8922]|06-SRNP-4060|669[0n]bp
Phocides nigrescens|[8923]|05-SRNP-7497|669[0n]bp
Phocides nigrescens|[8924]|06-SRNP-2402|669[0n]bp
Phocides nigrescens|[8925]|05-SRNP-7669|669[0n]bp
Phocides nigrescens|[8926]|05-SRNP-31898|669[0n]bp
Phocides nigrescens|[8927]|05-SRNP-21431|669[0n]bp
Phocides nigrescens|[8928]|05-SRNP-3478|669[0n]bp
Phocides nigrescens|[8929]|05-SRNP-2553|669[0n]bp
Phocides nigrescens|[8930]|04-SRNP-35496|669[0n]bp
Phocides nigrescens|[8931]|04-SRNP-3410|669[0n]bp
Phocides nigrescens|[8932]|04-SRNP-23796|669[0n]bp
Phocides nigrescens|[8933]|04-SRNP-4780|669[0n]bp
Phocides nigrescens|[8934]|04-SRNP-35586|669[0n]bp
Phocides nigrescens|[8935]|06-SRNP-6936|648[0n]bp
Phocides nigrescens|[8936]|06-SRNP-9333|651[2n]bp
Phocides nigrescens|[8937]|09-SRNP-21126|669[0n]bp
Nascus phintias|[8938]|05-SRNP-2410|669[0n]bp
Nascus phintias|[8939]|07-SRNP-2575|669[0n]bp
Nascus phintias|[8940]|02-SRNP-3373|645[0n]bp
Nascus phintias|[8941]|05-SRNP-2034|687[0n]bp
Nascus phintias|[8942]|05-SRNP-43646|669[0n]bp
Nascus phintias|[8943]|07-SRNP-35296|669[0n]bp
Nascus phintias|[8944]|05-SRNP-47456|669[0n]bp
Nascus phintias|[8945]|05-SRNP-3879|669[0n]bp
Nascus phintias|[8946]|05-SRNP-40848|669[0n]bp
Nascus phintias|[8947]|05-SRNP-2532|669[0n]bp
Nascus phintias|[8948]|05-SRNP-2535|669[0n]bp
Nascus phintias|[8949]|04-SRNP-2156|669[0n]bp
Nascus phintias|[8950]|02-SRNP-3469|669[0n]bp
Nascus phintias|[8951]|07-SRNP-36387|633[0n]bp
Nascus phintias|[8952]|08-SRNP-31018|669[0n]bp
Nascus Burns01|[8953]|04-SRNP-60540|669[0n]bp
Nascus Burns01|[8954]|02-SRNP-135|591[0n]bp
Nascus Burns01|[8955]|00-SRNP-1575|528[0n]bp
Nascus Burns01|[8956]|04-SRNP-14201|630[0n]bp
Nascus Burns01|[8957]|08-SRNP-14631|669[0n]bp
Nascus Burns01|[8958]|05-SRNP-47006|669[0n]bp
Nascus Burns01|[8959]|05-SRNP-47054|669[0n]bp
Nascus Burns01|[8960]|05-SRNP-47007|669[0n]bp
Nascus Burns01|[8961]|98-SRNP-15119|636[2n]bp
Nascus Burns01|[8962]|07-SRNP-23590|621[0n]bp
Nascus Burns01|[8963]|04-SRNP-2064|609[1n]bp
Nascus Burns01|[8964]|96-SRNP-12541|624[0n]bp
Nascus Burns01|[8965]|98-SRNP-15121|624[0n]bp
Nascus Burns01|[8966]|06-SRNP-47760|594[0n]bp
Nascus Burns01|[8967]|08-SRNP-6958|660[0n]bp
Nascus Burns01|[8968]|08-SRNP-6946|660[0n]bp
Nascus Burns02|[8969]|07-SRNP-45185|669[0n]bp
Nascus Burns02|[8970]|02-SRNP-5385|630[0n]bp
Nascus Burns02|[8971]|96-SRNP-7368|669[0n]bp
Nascus Burns02|[8972]|02-SRNP-2085|594[0n]bp
Nascus Burns02|[8973]|02-SRNP-5744|600[0n]bp
Nascus Burns02|[8974]|04-SRNP-45037|669[0n]bp
Nascus Burns02|[8975]|04-SRNP-48523|669[0n]bp
Nascus Burns02|[8976]|04-SRNP-23315|669[0n]bp
Nascus Burns02|[8977]|04-SRNP-21643|669[0n]bp
Nascus Burns02|[8978]|04-SRNP-21927|669[0n]bp
Nascus Burns02|[8979]|04-SRNP-47932|669[0n]bp
Nascus Burns02|[8980]|04-SRNP-47541|669[0n]bp
Nascus Burns02|[8981]|04-SRNP-48314|669[0n]bp
Nascus Burns02|[8982]|04-SRNP-48044|669[0n]bp
Nascus Burns02|[8983]|04-SRNP-48045|669[0n]bp
Nascus Burns02|[8984]|04-SRNP-47542|669[0n]bp
Nascus Burns02|[8985]|04-SRNP-11980|669[0n]bp
Nascus Burns02|[8986]|06-SRNP-21092|669[0n]bp
Nascus Burns02|[8987]|06-SRNP-3613|669[0n]bp
Nascus Burns02|[8988]|06-SRNP-58211|669[0n]bp
Nascus Burns02|[8989]|06-SRNP-59675|669[0n]bp
Nascus Burns02|[8990]|06-SRNP-47830|669[0n]bp
Nascus Burns02|[8991]|06-SRNP-59883|669[0n]bp
Nascus Burns02|[8992]|06-SRNP-59658|669[0n]bp
Nascus Burns02|[8993]|07-SRNP-60000|669[0n]bp
Nascus Burns02|[8994]|08-SRNP-1817|669[0n]bp
Nascus Burns02|[8995]|07-SRNP-65044|669[0n]bp
Nascus Burns02|[8996]|08-SRNP-22926|669[0n]bp

Nascus Burns02|[8994]|08-SRNP-1817|669[0n]bp
Nascus Burns02|[8995]|07-SRNP-65044|669[0n]bp
Nascus Burns02|[8996]|08-SRNP-22926|669[0n]bp
Nascus Burns02|[8997]|09-SRNP-44171|669[0n]bp
Nascus broteas|[8998]|07-SRNP-58744|642[0n]bp
Nascus broteas|[8999]|07-SRNP-65708|642[0n]bp
Nascus broteas|[9000]|07-SRNP-45315|642[0n]bp
Nascus broteas|[9001]|07-SRNP-45363|642[0n]bp
Nascus broteas|[9002]|07-SRNP-65709|633[0n]bp
Nascus broteas|[9003]|07-SRNP-20402|654[0n]bp
Nascus broteas|[9004]|07-SRNP-65689|651[0n]bp
Nascus broteas|[9005]|05-SRNP-47047|687[0n]bp
Nascus broteas|[9006]|08-SRNP-66188|669[0n]bp
Nascus broteas|[9007]|09-SRNP-57232|669[0n]bp
Nascus broteas|[9008]|08-SRNP-66059|669[0n]bp
Nascus broteas|[9009]|08-SRNP-66039|669[0n]bp
Nascus broteas|[9010]|08-SRNP-22159|669[0n]bp
Nascus broteas|[9011]|08-SRNP-32159|669[0n]bp
Nascus broteas|[9012]|07-SRNP-58175|669[0n]bp
Nascus broteas|[9013]|07-SRNP-35984|669[0n]bp
Nascus broteas|[9014]|09-SRNP-20230|669[0n]bp
Nascus broteas|[9015]|08-SRNP-6161|669[0n]bp
Nascus broteas|[9016]|08-SRNP-66029|669[0n]bp
Nascus broteas|[9017]|08-SRNP-66038|669[0n]bp
Nascus broteas|[9018]|07-SRNP-32448|669[0n]bp
Nascus broteas|[9019]|07-SRNP-36061|669[0n]bp
Nascus broteas|[9020]|05-SRNP-2095|669[0n]bp
Nascus broteas|[9021]|05-SRNP-5955|669[0n]bp
Nascus broteas|[9022]|05-SRNP-2780|669[0n]bp
Nascus broteas|[9023]|05-SRNP-3276|669[0n]bp
Nascus broteas|[9024]|05-SRNP-887|669[0n]bp
Nascus broteas|[9025]|05-SRNP-47048|669[0n]bp
Nascus broteas|[9026]|04-SRNP-46661|669[0n]bp
Nascus broteas|[9027]|03-SRNP-37108|669[0n]bp
Nascus broteas|[9028]|01-SRNP-7365|663[0n]bp
Nascus broteas|[9029]|02-SRNP-9577|663[0n]bp
Nascus broteas|[9030]|05-SRNP-47061|669[2n]bp
Nascus broteas|[9031]|05-SRNP-30597|669[1n]bp
Nascus broteas|[9032]|06-SRNP-1723|669[0n]bp
Nascus broteas|[9033]|09-SRNP-80142|669[0n]bp
Nascus solon|[9034]|94-SRNP-4830|627[0n]bp
Nascus solon|[9035]|05-SRNP-55806|687[0n]bp
Nascus solon|[9036]|04-SRNP-50064|669[0n]bp
Nascus solon|[9037]|05-SRNP-46699|669[0n]bp
Nascus solon|[9038]|05-SRNP-705|669[0n]bp
Nascus solon|[9039]|05-SRNP-58399|669[0n]bp
Nascus solon|[9040]|05-SRNP-2536|669[0n]bp
Nascus solon|[9041]|05-SRNP-46952|669[0n]bp
Nascus solon|[9042]|05-SRNP-30650|669[0n]bp
Nascus solon|[9043]|04-SRNP-49682|669[0n]bp
Nascus solon|[9044]|04-SRNP-45029|669[0n]bp
Nascus solon|[9045]|04-SRNP-35293|669[0n]bp
Nascus solon|[9046]|04-SRNP-49716|669[0n]bp
Nascus solon|[9047]|04-SRNP-49717|669[0n]bp
Nascus solon|[9048]|04-SRNP-49681|669[0n]bp
Nascus solon|[9049]|04-SRNP-49777|669[0n]bp
Nascus solon|[9050]|04-SRNP-49768|669[0n]bp
Nascus solon|[9051]|04-SRNP-49335|669[0n]bp
Nascus solon|[9052]|04-SRNP-49680|669[0n]bp
Nascus solon|[9053]|02-SRNP-168|669[0n]bp
Nascus solon|[9054]|01-SRNP-597|663[0n]bp
Nascus solon|[9055]|04-SRNP-49336|627[0n]bp
Nascus solon|[9056]|04-SRNP-49775|363[0n]bp
Nascus solon|[9057]|06-SRNP-59999|642[1n]bp
Nascus solon|[9058]|05-SRNP-25375|669[0n]bp
Nascus solon|[9059]|05-SRNP-47450|669[0n]bp
Nascus solon|[9060]|07-SRNP-21820|669[0n]bp
Nascus solon|[9061]|07-SRNP-45627|669[0n]bp
Nascus solon|[9062]|08-SRNP-55518|669[0n]bp
Nascus solon|[9063]|08-SRNP-21263|669[0n]bp
Nascus solon|[9064]|07-SRNP-61336|669[0n]bp
Nascus solon|[9065]|08-SRNP-21932|669[0n]bp
Nascus solon|[9066]|09-SRNP-57395|669[0n]bp
Drephalys alcmon|[9067]|01-SRNP-9223|669[0n]bp
Drephalys alcmon|[9068]|99-SRNP-2617|633[0n]bp
Drephalys alcmon|[9069]|97-SRNP-4047|633[1n]bp
Drephalys alcmon|[9070]|99-SRNP-2188|645[0n]bp
Drephalys alcmon|[9071]|01-SRNP-9331|639[0n]bp
Drephalys alcmon|[9072]|97-SRNP-4150|657[0n]bp
Drephalys alcmon|[9073]|02-SRNP-29274|669[0n]bp
Drephalys alcmon|[9074]|02-SRNP-4202|669[0n]bp
Drephalys alcmon|[9075]|99-SRNP-2646|669[0n]bp
Drephalys alcmon|[9076]|98-SRNP-4184|669[0n]bp
Drephalys alcmon|[9077]|00-SRNP-2692|669[0n]bp
Drephalys Burns01|[9078]|05-SRNP-70289|567[0n]bp
Drephalys Burns01|[9079]|06-SRNP-5699|666[0n]bp
Drephalys Burns01|[9080]|07-SRNP-42336|669[0n]bp
Drephalys Burns01|[9081]|06-SRNP-42502|669[0n]bp
Drephalys Burns01|[9082]|06-SRNP-42807|669[0n]bp
Drephalys Burns01|[9083]|05-SRNP-31284|669[0n]bp
Drephalys Burns01|[9084]|05-SRNP-43718|669[0n]bp
Drephalys Burns01|[9085]|05-SRNP-34501|669[0n]bp
Drephalys Burns01|[9086]|05-SRNP-41556|669[0n]bp
Drephalys Burns01|[9087]|05-SRNP-41680|669[0n]bp
Drephalys Burns01|[9088]|05-SRNP-41447|669[0n]bp
Drephalys Burns01|[9089]|05-SRNP-41557|639[0n]bp
Drephalys Burns01|[9090]|03-SRNP-21317|645[0n]bp
Drephalys Burns01|[9091]|06-SRNP-30793|411[0n]bp
Drephalys Burns01|[9092]|06-SRNP-42501|654[1n]bp
Drephalys Burns01|[9093]|07-SRNP-41814|657[0n]bp
Drephalys Burns01|[9094]|08-SRNP-345|669[0n]bp
Drephalys Burns01|[9095]|08-SRNP-41486|669[0n]bp
Drephalys Burns01|[9096]|08-SRNP-71839|669[0n]bp

Drephalys Burns01|[9094]|08-SRNP-345|669[0n]bp
Drephalys Burns01|[9095]|08-SRNP-41486|669[0n]bp
Drephalys Burns01|[9096]|08-SRNP-71839|669[0n]bp
Drephalys kidonoi|[9097]|06-SRNP-60166|576[0n]bp
Drephalys kidonoi|[9098]|02-SRNP-12965|618[0n]bp
Drephalys kidonoi|[9099]|02-SRNP-12979|609[0n]bp
Drephalys kidonoi|[9100]|02-SRNP-13368|597[1n]bp
Drephalys kidonoi|[9101]|02-SRNP-10261|615[0n]bp
Drephalys kidonoi|[9102]|02-SRNP-13429|669[0n]bp
Drephalys kidonoi|[9103]|02-SRNP-12978|669[0n]bp
Drephalys kidonoi|[9104]|02-SRNP-10329|669[0n]bp
Drephalys kidonoi|[9105]|02-SRNP-10250|669[0n]bp
Drephalys kidonoi|[9106]|02-SRNP-13369|669[0n]bp
Drephalys kidonoi|[9107]|02-SRNP-10252|669[0n]bp
Drephalys kidonoi|[9108]|02-SRNP-13103|669[0n]bp
Drephalys kidonoi|[9109]|01-SRNP-12341|528[0n]bp
Drephalys kidonoi|[9110]|02-SRNP-13370|492[0n]bp
Drephalys kidonoi|[9111]|06-SRNP-60079|615[0n]bp
Drephalys kidonoi|[9112]|07-SRNP-55278|609[0n]bp
Drephalys kidonoi|[9113]|09-SRNP-55224|669[0n]bp
Drephalys kidonoi|[9114]|09-SRNP-55518|669[0n]bp
Cogia hiska|[9115]|03-SRNP-250|645[0n]bp
Cogia hiska|[9116]|03-SRNP-101|630[0n]bp
Cogia hiska|[9117]|06-SRNP-12193|669[0n]bp
Cogia hiska|[9118]|03-SRNP-18|669[0n]bp
Cogia hiska|[9119]|03-SRNP-14096|669[0n]bp
Cogia hiska|[9120]|03-SRNP-13714|669[0n]bp
Cogia hiska|[9121]|02-SRNP-12905|669[0n]bp
Cogia hiska|[9122]|02-SRNP-13343|669[0n]bp
Cogia hiska|[9123]|02-SRNP-13341|669[0n]bp
Cogia hiska|[9124]|02-SRNP-13338|669[0n]bp
Cogia hiska|[9125]|02-SRNP-13244|669[0n]bp
Cogia hiska|[9126]|02-SRNP-12906|669[0n]bp
Cogia hiska|[9127]|02-SRNP-13604|669[0n]bp
Cogia hiska|[9128]|02-SRNP-13580|669[0n]bp
Cogia hiska|[9129]|02-SRNP-13357|669[0n]bp
Cogia hiska|[9130]|02-SRNP-13348|669[0n]bp
Cogia hiska|[9131]|02-SRNP-13346|669[0n]bp
Typhedanus undulatus|[9132]|05-SRNP-5907|669[4n]bp
Typhedanus undulatus|[9133]|05-SRNP-7507|669[1n]bp
Typhedanus undulatus|[9134]|05-SRNP-5906|627[0n]bp
Typhedanus undulatus|[9135]|99-SRNP-12788|624[0n]bp
Typhedanus undulatus|[9136]|98-SRNP-6246|639[0n]bp
Typhedanus undulatus|[9137]|04-SRNP-24403|669[0n]bp
Typhedanus undulatus|[9138]|06-SRNP-21153|669[0n]bp
Typhedanus undulatus|[9139]|07-SRNP-41835|669[0n]bp
Typhedanus undulatus|[9140]|07-SRNP-3071|669[0n]bp
Typhedanus undulatus|[9141]|07-SRNP-23877|669[0n]bp
Cogia calchasDHJ01|[9142]|05-SRNP-12001|591[0n]bp
Cogia calchasDHJ01|[9143]|04-SRNP-49737|669[0n]bp
Cogia calchasDHJ01|[9144]|07-SRNP-55314|669[0n]bp
Cogia calchasDHJ01|[9145]|98-SRNP-6237|669[0n]bp
Cogia calchasDHJ01|[9146]|93-SRNP-6683|669[0n]bp
Cogia calchasDHJ01|[9147]|06-SRNP-13361|669[0n]bp
Cogia calchasDHJ01|[9148]|04-SRNP-48664|669[0n]bp
Cogia calchasDHJ01|[9149]|97-SRNP-379|624[0n]bp
Cogia calchasDHJ01|[9150]|07-SRNP-45272|645[0n]bp
Cogia calchasDHJ01|[9151]|03-SRNP-31305|669[0n]bp
Cogia calchasDHJ01|[9152]|98-SRNP-6173|669[0n]bp
Cogia calchasDHJ01|[9153]|98-SRNP-6178|669[0n]bp
Cogia calchasDHJ01|[9154]|06-SRNP-13417|669[0n]bp
Cogia calchasDHJ01|[9155]|05-SRNP-31856|669[0n]bp
Cogia calchasDHJ01|[9156]|03-SRNP-6772|663[0n]bp
Cogia calchasDHJ01|[9157]|97-SRNP-296|666[0n]bp
Cogia calchasDHJ01|[9158]|98-SRNP-6163|669[0n]bp
Cogia calchasDHJ01|[9159]|08-SRNP-2425|669[0n]bp
Cogia calchasDHJ01|[9160]|08-SRNP-2467|669[0n]bp
Cogia calchasDHJ01|[9161]|09-SRNP-80678|669[0n]bp
Cogia calchasDHJ02|[9162]|05-SRNP-32463|669[0n]bp
Cogia calchasDHJ02|[9163]|04-SRNP-55362|669[0n]bp
Cogia calchasDHJ02|[9164]|05-SRNP-32462|651[0n]bp
Cogia calchasDHJ02|[9165]|00-SRNP-21991|669[0n]bp
Cogia calchasDHJ02|[9166]|09-SRNP-73335|669[0n]bp
Cogia calchasDHJ02|[9167]|09-SRNP-73332|669[0n]bp
Cogia calchasDHJ02|[9168]|09-SRNP-80699|669[0n]bp
Udranomia orcinus|[9169]|04-SRNP-41230|648[0n]bp
Udranomia orcinus|[9170]|05-SRNP-2555|669[4n]bp
Udranomia orcinus|[9171]|04-SRNP-30933|669[0n]bp
Udranomia orcinus|[9172]|04-SRNP-31229|669[1n]bp
Udranomia orcinus|[9173]|03-SRNP-5582|525[1n]bp
Udranomia orcinus|[9174]|05-SRNP-1457|669[0n]bp
Udranomia orcinus|[9175]|07-SRNP-40320|669[0n]bp
Udranomia orcinus|[9176]|08-SRNP-1673|669[0n]bp
Udranomia orcinus|[9177]|05-SRNP-21131|687[0n]bp
Udranomia orcinus|[9178]|08-SRNP-22893|669[0n]bp
Udranomia orcinus|[9179]|08-SRNP-41217|669[0n]bp
Udranomia orcinus|[9180]|07-SRNP-20955|669[0n]bp
Udranomia orcinus|[9181]|08-SRNP-31332|669[0n]bp
Udranomia orcinus|[9182]|08-SRNP-40514|669[0n]bp
Udranomia orcinus|[9183]|08-SRNP-1672|669[0n]bp
Udranomia orcinus|[9184]|08-SRNP-20817|669[0n]bp
Udranomia orcinus|[9185]|08-SRNP-20850|669[0n]bp
Udranomia orcinus|[9186]|08-SRNP-818|669[0n]bp
Udranomia orcinus|[9187]|08-SRNP-30988|669[0n]bp
Udranomia orcinus|[9188]|07-SRNP-65382|669[0n]bp
Udranomia orcinus|[9189]|07-SRNP-40260|669[0n]bp
Udranomia orcinus|[9190]|07-SRNP-1287|669[0n]bp
Udranomia orcinus|[9191]|06-SRNP-23065|669[0n]bp
Udranomia orcinus|[9192]|04-SRNP-31004|669[0n]bp
Udranomia orcinus|[9193]|04-SRNP-42858|669[0n]bp
Udranomia orcinus|[9194]|07-SRNP-40862|666[0n]bp
Udranomia orcinus|[9195]|06-SRNP-41751|666[1n]bp
Udranomia orcinus|[9196]|05-SRNP-31979|669[3n]bp

Udranomia orcinus|[9194]|07-SRNP-40862|666[0n]bp
Udranomia orcinus|[9195]|06-SRNP-41751|666[1n]bp
Udranomia orcinus|[9196]|05-SRNP-31979|669[3n]bp
Udranomia orcinus|[9197]|03-SRNP-10433|519[1n]bp
Udranomia orcinus|[9198]|05-SRNP-8128|648[1n]bp
Udranomia orcinus|[9199]|05-SRNP-41043|567[2n]bp
Udranomia orcinus|[9200]|05-SRNP-8135|633[0n]bp
Udranomia orcinus|[9201]|07-SRNP-24023|645[0n]bp
Udranomia orcinus|[9202]|08-SRNP-70812|639[0n]bp
Udranomia orcinus|[9203]|08-SRNP-66180|669[0n]bp
Udranomia orcinus|[9204]|09-SRNP-67117|669[0n]bp
Udranomia orcinus|[9205]|09-SRNP-69354|669[0n]bp
Udranomia orcinus|[9206]|09-SRNP-80703|669[0n]bp
Udranomia eurus|[9207]|03-SRNP-11250|561[0n]bp
Udranomia kikkawaiDHJ03|[9208]|08-SRNP-21971|669[0n]bp
Udranomia kikkawaiDHJ03|[9209]|08-SRNP-21969|669[0n]bp
Udranomia kikkawaiDHJ03|[9210]|96-SRNP-1042|594[0n]bp
Udranomia kikkawaiDHJ03|[9211]|06-SRNP-55723|669[0n]bp
Udranomia kikkawaiDHJ03|[9212]|07-SRNP-55337|639[0n]bp
Udranomia kikkawaiDHJ03|[9213]|07-SRNP-55336|639[0n]bp
Udranomia kikkawaiDHJ03|[9214]|08-SRNP-21281|669[0n]bp
Udranomia kikkawaiDHJ03|[9215]|07-SRNP-56346|669[0n]bp
Udranomia kikkawaiDHJ03|[9216]|07-SRNP-56440|669[0n]bp
Udranomia kikkawaiDHJ03|[9217]|07-SRNP-56118|669[0n]bp
Udranomia kikkawaiDHJ03|[9218]|07-SRNP-55138|669[0n]bp
Udranomia kikkawaiDHJ03|[9219]|01-SRNP-9758|669[0n]bp
Udranomia kikkawaiDHJ03|[9220]|01-SRNP-24435|669[0n]bp
Udranomia kikkawaiDHJ03|[9221]|02-SRNP-11244|669[0n]bp
Udranomia kikkawaiDHJ03|[9222]|02-SRNP-11245|669[0n]bp
Udranomia kikkawaiDHJ03|[9223]|00-SRNP-2831|669[0n]bp
Udranomia kikkawaiDHJ03|[9224]|01-SRNP-17596|669[0n]bp
Udranomia kikkawaiDHJ03|[9225]|07-SRNP-55917|630[0n]bp
Udranomia kikkawaiDHJ03|[9226]|02-SRNP-32867|633[1n]bp
Udranomia kikkawaiDHJ03|[9227]|07-SRNP-56117|654[0n]bp
Udranomia kikkawaiDHJ03|[9228]|07-SRNP-20950|660[0n]bp
Udranomia kikkawaiDHJ03|[9229]|07-SRNP-23337|669[0n]bp
Udranomia kikkawaiDHJ03|[9230]|07-SRNP-23338|669[0n]bp
Udranomia kikkawaiDHJ03|[9231]|08-SRNP-56040|669[0n]bp
Udranomia kikkawaiDHJ03|[9232]|08-SRNP-20600|669[0n]bp
Udranomia kikkawaiDHJ03|[9233]|08-SRNP-20931|669[0n]bp
Udranomia kikkawaiDHJ03|[9234]|08-SRNP-21219|669[0n]bp
Udranomia kikkawaiDHJ03|[9235]|08-SRNP-21967|669[0n]bp
Udranomia kikkawaiDHJ03|[9236]|08-SRNP-21965|669[0n]bp
Udranomia kikkawaiDHJ03|[9237]|08-SRNP-23929|669[0n]bp
Udranomia kikkawaiDHJ03|[9238]|08-SRNP-16988|669[0n]bp
Udranomia kikkawaiDHJ01|[9239]|03-SRNP-12017|567[0n]bp
Udranomia kikkawaiDHJ01|[9240]|96-SRNP-807|594[0n]bp
Udranomia kikkawaiDHJ01|[9241]|96-SRNP-1053|594[0n]bp
Udranomia kikkawaiDHJ01|[9242]|96-SRNP-1007|576[0n]bp
Udranomia kikkawaiDHJ01|[9243]|00-SRNP-6094|669[0n]bp
Udranomia kikkawaiDHJ01|[9244]|01-SRNP-17593|630[1n]bp
Udranomia kikkawaiDHJ01|[9245]|02-SRNP-4194|630[0n]bp
Udranomia kikkawaiDHJ01|[9246]|02-SRNP-10083|627[0n]bp
Udranomia kikkawaiDHJ01|[9247]|07-SRNP-55350|642[0n]bp
Udranomia kikkawaiDHJ01|[9248]|08-SRNP-21920|633[0n]bp
Udranomia kikkawaiDHJ01|[9249]|07-SRNP-55803|669[0n]bp
Udranomia kikkawaiDHJ01|[9250]|07-SRNP-55804|669[0n]bp
Udranomia kikkawaiDHJ01|[9251]|07-SRNP-55142|669[0n]bp
Udranomia kikkawaiDHJ01|[9252]|06-SRNP-56437|669[0n]bp
Udranomia kikkawaiDHJ01|[9253]|01-SRNP-24380|669[0n]bp
Udranomia kikkawaiDHJ01|[9254]|03-SRNP-966|669[0n]bp
Udranomia kikkawaiDHJ01|[9255]|01-SRNP-17592|669[0n]bp
Udranomia kikkawaiDHJ01|[9256]|06-SRNP-13261|666[0n]bp
Udranomia kikkawaiDHJ01|[9257]|97-SRNP-4074|573[0n]bp
Udranomia kikkawaiDHJ01|[9258]|06-SRNP-13260|669[1n]bp
Udranomia kikkawaiDHJ01|[9259]|96-SRNP-930|324[2n]bp
Udranomia kikkawaiDHJ01|[9260]|07-SRNP-20867|600[0n]bp
Udranomia kikkawaiDHJ01|[9261]|07-SRNP-20972|669[0n]bp
Udranomia kikkawaiDHJ01|[9262]|07-SRNP-23327|669[0n]bp
Udranomia kikkawaiDHJ01|[9263]|08-SRNP-20601|669[0n]bp
Udranomia kikkawaiDHJ01|[9264]|08-SRNP-20596|669[0n]bp
Udranomia kikkawaiDHJ01|[9265]|08-SRNP-21165|669[0n]bp
Udranomia kikkawaiDHJ01|[9266]|08-SRNP-12275|669[0n]bp
Udranomia kikkawaiDHJ01|[9267]|08-SRNP-12197|645[0n]bp
Udranomia kikkawaiDHJ01|[9268]|07-SRNP-55686|645[0n]bp
Udranomia kikkawaiDHJ01|[9269]|07-SRNP-56119|645[0n]bp
Udranomia kikkawaiDHJ01|[9270]|07-SRNP-12122|645[0n]bp
Udranomia kikkawaiDHJ01|[9271]|02-SRNP-14029|627[0n]bp
Udranomia kikkawaiDHJ01|[9272]|08-SRNP-12195|621[0n]bp
Udranomia kikkawaiDHJ01|[9273]|97-SRNP-4069|624[0n]bp
Udranomia kikkawaiDHJ01|[9274]|08-SRNP-16999|669[0n]bp
Udranomia kikkawaiDHJ01|[9275]|08-SRNP-75002|669[0n]bp
Udranomia kikkawaiDHJ01|[9276]|08-SRNP-16997|669[0n]bp
Udranomia kikkawaiDHJ01|[9277]|08-SRNP-12158|669[0n]bp
Udranomia kikkawaiDHJ01|[9278]|08-SRNP-21923|669[0n]bp
Udranomia kikkawaiDHJ01|[9279]|08-SRNP-21924|669[0n]bp
Udranomia kikkawaiDHJ01|[9280]|08-SRNP-21968|669[0n]bp
Udranomia kikkawaiDHJ01|[9281]|08-SRNP-21926|669[0n]bp
Udranomia kikkawaiDHJ01|[9282]|07-SRNP-20946|669[0n]bp
Udranomia kikkawaiDHJ01|[9283]|08-SRNP-55525|669[0n]bp
Udranomia kikkawaiDHJ01|[9284]|08-SRNP-20481|669[0n]bp
Udranomia kikkawaiDHJ01|[9285]|08-SRNP-20636|669[0n]bp
Udranomia kikkawaiDHJ01|[9286]|08-SRNP-20882|669[0n]bp
Udranomia kikkawaiDHJ01|[9287]|08-SRNP-20635|669[0n]bp
Udranomia kikkawaiDHJ01|[9288]|08-SRNP-45146|669[0n]bp
Udranomia kikkawaiDHJ01|[9289]|07-SRNP-23341|669[0n]bp
Udranomia kikkawaiDHJ01|[9290]|07-SRNP-20956|669[0n]bp
Udranomia kikkawaiDHJ01|[9291]|07-SRNP-20953|669[0n]bp
Udranomia kikkawaiDHJ01|[9292]|07-SRNP-56274|669[0n]bp
Udranomia kikkawaiDHJ01|[9293]|07-SRNP-12359|669[0n]bp
Udranomia kikkawaiDHJ01|[9294]|07-SRNP-12102|669[0n]bp
Udranomia kikkawaiDHJ01|[9295]|07-SRNP-55349|669[0n]bp
Udranomia kikkawaiDHJ01|[9296]|07-SRNP-55351|669[0n]bp

Udranom i a k i kkawa i DHJ01|[9294]|07-SRNP-12102|669[0n]bp
Udranomia kikkawaiDHJ01|[9295]|07-SRNP-55349|669[0n]bp
Udranomia kikkawaiDHJ01|[9296]|07-SRNP-55351|669[0n]bp
Udranomia kikkawaiDHJ01|[9297]|07-SRNP-12180|669[0n]bp
Udranomia kikkawaiDHJ01|[9298]|07-SRNP-12127|669[0n]bp
Udranomia kikkawaiDHJ01|[9299]|07-SRNP-12034|669[0n]bp
Udranomia kikkawaiDHJ01|[9300]|07-SRNP-12098|669[0n]bp
Udranomia kikkawaiDHJ01|[9301]|07-SRNP-55263|669[0n]bp
Udranomia kikkawaiDHJ01|[9302]|06-SRNP-13329|669[0n]bp
Udranomia kikkawaiDHJ01|[9303]|06-SRNP-56079|669[0n]bp
Udranomia kikkawaiDHJ01|[9304]|96-SRNP-1155|669[0n]bp
Udranomia kikkawaiDHJ01|[9305]|96-SRNP-832|669[0n]bp
Udranomia kikkawaiDHJ01|[9306]|99-SRNP-2706|669[0n]bp
Udranomia kikkawaiDHJ01|[9307]|01-SRNP-24490|669[0n]bp
Udranomia kikkawaiDHJ01|[9308]|01-SRNP-24434|669[0n]bp
Udranomia kikkawaiDHJ01|[9309]|02-SRNP-5956|669[0n]bp
Udranomia kikkawaiDHJ01|[9310]|01-SRNP-17953|669[0n]bp
Udranomia kikkawaiDHJ01|[9311]|02-SRNP-4127|669[0n]bp
Udranomia kikkawaiDHJ01|[9312]|01-SRNP-17591|669[0n]bp
Udranomia kikkawaiDHJ01|[9313]|03-SRNP-12056|669[0n]bp
Udranomia kikkawaiDHJ01|[9314]|05-SRNP-45233|669[0n]bp
Udranomia kikkawaiDHJ01|[9315]|05-SRNP-45219|669[0n]bp
Udranomia kikkawaiDHJ01|[9316]|04-SRNP-45504|669[0n]bp
Udranomia kikkawaiDHJ01|[9317]|04-SRNP-16075|669[0n]bp
Udranomia kikkawaiDHJ01|[9318]|04-SRNP-45505|669[0n]bp
Udranomia kikkawaiDHJ01|[9319]|07-SRNP-20954|621[0n]bp
Udranomia kikkawaiDHJ01|[9320]|05-SRNP-12029|669[0n]bp
Udranomia kikkawaiDHJ01|[9321]|04-SRNP-45599|669[0n]bp
Udranomia kikkawaiDHJ01|[9322]|07-SRNP-12099|666[0n]bp
Udranomia kikkawaiDHJ01|[9323]|07-SRNP-12101|663[1n]bp
Udranomia kikkawaiDHJ01|[9324]|07-SRNP-56275|651[0n]bp
Udranomia kikkawaiDHJ01|[9325]|07-SRNP-55265|642[0n]bp
Udranomia kikkawaiDHJ01|[9326]|07-SRNP-20952|603[0n]bp
Udranomia kikkawaiDHJ01|[9327]|08-SRNP-75004|669[1n]bp
Udranomia kikkawaiDHJ01|[9328]|09-SRNP-12113|669[1n]bp
Udranomia kikkawaiDHJ01|[9329]|08-SRNP-16994|669[0n]bp
Udranomia kikkawaiDHJ01|[9330]|09-SRNP-20996|669[0n]bp
Udranomia kikkawaiDHJ02|[9331]|96-SRNP-1054|594[0n]bp
Udranomia kikkawaiDHJ02|[9332]|05-SRNP-41045|669[0n]bp
Udranomia kikkawaiDHJ02|[9333]|04-SRNP-30985|669[0n]bp
Udranomia kikkawaiDHJ02|[9334]|05-SRNP-21026|669[1n]bp
Udranomia kikkawaiDHJ02|[9335]|01-SRNP-22738|669[0n]bp
Udranomia kikkawaiDHJ02|[9336]|03-SRNP-10461|669[0n]bp
Udranomia kikkawaiDHJ02|[9337]|05-SRNP-40465|669[0n]bp
Udranomia kikkawaiDHJ02|[9338]|06-SRNP-3255|645[0n]bp
Udranomia kikkawaiDHJ02|[9339]|03-SRNP-10577|645[0n]bp
Udranomia kikkawaiDHJ02|[9340]|04-SRNP-40828|669[1n]bp
Udranomia kikkawaiDHJ02|[9341]|08-SRNP-31128|669[0n]bp
Udranomia kikkawaiDHJ02|[9342]|08-SRNP-21090|669[0n]bp
Udranomia kikkawaiDHJ02|[9343]|08-SRNP-70104|669[0n]bp
Udranomia kikkawaiDHJ02|[9344]|07-SRNP-40724|669[0n]bp
Udranomia kikkawaiDHJ02|[9345]|06-SRNP-23003|669[0n]bp
Udranomia kikkawaiDHJ02|[9346]|06-SRNP-41739|669[0n]bp
Udranomia kikkawaiDHJ02|[9347]|06-SRNP-41738|669[0n]bp
Udranomia kikkawaiDHJ02|[9348]|06-SRNP-41442|669[0n]bp
Udranomia kikkawaiDHJ02|[9349]|03-SRNP-10604|669[0n]bp
Udranomia kikkawaiDHJ02|[9350]|03-SRNP-10392|669[0n]bp
Udranomia kikkawaiDHJ02|[9351]|03-SRNP-10390|669[0n]bp
Udranomia kikkawaiDHJ02|[9352]|03-SRNP-11383|669[0n]bp
Udranomia kikkawaiDHJ02|[9353]|02-SRNP-21390|669[0n]bp
Udranomia kikkawaiDHJ02|[9354]|03-SRNP-10391|669[0n]bp
Udranomia kikkawaiDHJ02|[9355]|02-SRNP-2617|669[0n]bp
Udranomia kikkawaiDHJ02|[9356]|02-SRNP-6916|669[0n]bp
Udranomia kikkawaiDHJ02|[9357]|06-SRNP-3420|669[0n]bp
Udranomia kikkawaiDHJ02|[9358]|06-SRNP-3422|669[0n]bp
Udranomia kikkawaiDHJ02|[9359]|05-SRNP-31041|669[0n]bp
Udranomia kikkawaiDHJ02|[9360]|05-SRNP-21024|669[0n]bp
Udranomia kikkawaiDHJ02|[9361]|04-SRNP-31720|669[0n]bp
Udranomia kikkawaiDHJ02|[9362]|04-SRNP-41255|669[0n]bp
Udranomia kikkawaiDHJ02|[9363]|04-SRNP-2413|669[0n]bp
Udranomia kikkawaiDHJ02|[9364]|06-SRNP-41737|669[1n]bp
Udranomia kikkawaiDHJ02|[9365]|06-SRNP-41741|669[1n]bp
Udranomia kikkawaiDHJ02|[9366]|06-SRNP-3253|669[1n]bp
Udranomia kikkawaiDHJ02|[9367]|06-SRNP-32415|606[0n]bp
Udranomia kikkawaiDHJ02|[9368]|06-SRNP-34647|660[0n]bp
Udranomia kikkawaiDHJ02|[9369]|07-SRNP-40376|663[0n]bp
Udranomia kikkawaiDHJ02|[9370]|08-SRNP-40430|648[1n]bp
Udranomia kikkawaiDHJ02|[9371]|08-SRNP-65801|669[1n]bp
Udranomia kikkawaiDHJ02|[9372]|08-SRNP-41215|669[0n]bp
Udranomia kikkawaiDHJ02|[9373]|08-SRNP-32449|669[1n]bp
Udranomia kikkawaiDHJ02|[9374]|03-SRNP-10011|627[0n]bp
Udranomia kikkawaiDHJ02|[9375]|06-SRNP-41523|669[0n]bp
Udranomia kikkawaiDHJ02|[9376]|09-SRNP-69425|669[0n]bp
Udranomia kikkawaiDHJ02|[9377]|06-SRNP-3714|669[0n]bp
Udranomia kikkawaiDHJ02|[9378]|06-SRNP-3715|669[1n]bp
Udranomia kikkawaiDHJ02|[9379]|06-SRNP-32537|669[0n]bp
Udranomia kikkawaiDHJ02|[9380]|03-SRNP-10389|642[0n]bp
Udranomia kikkawaiDHJ02|[9381]|05-SRNP-31092|669[0n]bp
Udranomia kikkawaiDHJ02|[9382]|06-SRNP-31634|669[0n]bp
Udranomia kikkawaiDHJ02|[9383]|08-SRNP-40400|669[0n]bp
Udranomia kikkawaiDHJ02|[9384]|08-SRNP-71686|669[0n]bp
Udranomia kikkawaiDHJ02|[9385]|09-SRNP-69506|669[0n]bp
Udranomia kikkawaiDHJ02|[9386]|09-SRNP-69421|669[0n]bp
Udranomia kikkawaiDHJ02|[9387]|09-SRNP-69505|669[0n]bp
Udranomia kikkawaiDHJ02|[9388]|09-SRNP-41711|669[0n]bp
Udranomia kikkawaiDHJ02|[9389]|02-SRNP-19637|618[0n]bp
Udranomia kikkawaiDHJ02|[9390]|06-SRNP-41526|669[0n]bp
Udranomia kikkawaiDHJ02|[9391]|05-SRNP-31080|669[0n]bp
Udranomia kikkawaiDHJ02|[9392]|03-SRNP-5785|669[1n]bp
Udranomia kikkawaiDHJ02|[9393]|06-SRNP-41524|669[1n]bp
Udranomia kikkawaiDHJ02|[9394]|09-SRNP-42041|669[0n]bp
