# Supplementary material for: Reading the Complex Skipper Butterfly Fauna of One Tropical Place
Source: PLoS One. 2011 Aug 16;6(8):e19874. doi: 10.1371/journal.pone.0019874 (PMC3156701; doi:10.1371/journal.pone.0019874)
Supplement: Table S1 — Summary statistics for all ACG reared Eudaminae and Pyrginae (Hesperiidae) skipper butterflies. These were reared from wild-caught caterpillars 1978 to 2009, inclusive. The year of original description of the morphologically-characterized species is included so as to emphasize that these butterflies have long been subject to taxonomic examination in Costa Rica and elsewhere; they are not a neglected taxon, as compared with smaller and less attractive animals, and barcode revelation of cryptic species is a significant contribution to understanding their biodiversity. Blue records are those that split into distinct groups of barcodes (see NJ tree in Figure S1). Yellow records are those that we feel are, or are likely to be, representing cryptic, previously unnoticed species. Green records are those whose barcode cluster contains too few specimens to feel certain that it is significant, yet needs to be flagged for further sampling. (PDF) [file pone.0019874.s002.pdf]

Table S1. Summary statistics for all ACG successfully reared Eudaminae and Pyrginae (Hesperiidae) skipper butterflies 1978-2009 [1/]

species name in database http://janzen.sas.upenn.edu	YEAR DESCRIBED	category A=singleton B =normal C=BC split	# rearing records total for morphospecies	number of barcode groups in morphological unit	# in split	# spread in SI, INBio, & McGuire Center	# sampled & sent to BIO	# successful barcodes	why a sample of this size
Achalarus albociliatus	1877	B	30	1		23	10	9	confused with other species at a glance
Achalarus toxeus	1882	B	40	1		24	5	5	reared few after 2003
Achlyodes busirus	1779	B	1461	1		215	46	40	probing food plant ecology variation
Achlyodes pallida	1869	B	423	1		180	30	27	probing food plant ecology variation
Aethilla echina	1870	A	1	single record		1	1	1	wild-caught pupa, host not known in ACG
Aethilla lavochrea	1872	C	136	2		49	20	19	suspected would be two
Aethilla lavochreaDHJ01					1	1			unlikely to be another species, need more
Aethilla lavochreaDHJ02					18	18			unlikely to be another species, need more
Aguna arunce hypozonius	1880	B	15	1		5	5	5	reared few
Aguna asander	1867	B	442	1		89	24	23	probing seasonal ecology variation
Aguna Burns01		B	22	1		12	12	12	reared few, confused with other species at a glance
Aguna Burns02		B	43	1		19	18	15	reared few, confused with other species at a glance
Aguna claxon	1952	B	12	1		8	8	6	reared few
Aguna coeloides	1998	B	42	1		9	8	7	reared few
Aguna metophis	1824	B	2	1		2	1	1	reared few
Aguna panama	1998	B	60	1		12	12	11	reared few
Anastrus neaeris	1879	B	129	1		56	24	24	probing microgeographic ecology variation
Anastrus sempiternus	1872	B	94	1		50	22	22	probing food plant ecology variation
Anisochoria polysticta	1877	B	50	1		22	6	6	reared few
Antigonus erosus	1812	B	732	1		185	36	36	probing microgeographic ecology variation
Antigonus nearchus	1817	B	7	1		6	6	6	reared few
Arteurotia tractipennis	1872	B	76	1		31	12	12	reared few
Astraptes alardus	1790	B	858	1		185	31	30	probing microgeographic ecology variation
Astraptes anaphus annetta	1952	C	823	3		142	117	116	found early to be more than one barcode
Astraptes anaphus annettaDHJ01					5	5			no difference in facies or genitalia
Astraptes anaphus annettaDHJ02					49	49			no difference in facies or genitalia
Astraptes anaphus annettaDHJ03					56	56			no difference in facies or genitalia
Astraptes apastus	1777	B	8	1		2	2	2	reared few
Astraptes aulus	1881	B	9	1		8	6	6	reared few
Astraptes brevicauda	1886	B	72	1		28	22	22	probing food plant ecology variation
Astraptes chiriquensis	1876	B	7	1		7	7	7	reared few
Astraptes creteus crana	1952	C	193	2		108	103	98	found early to be more than one barcode
Astraptes creteus cranaDHJ01					15	15			
Astraptes creteus cranaDHJ02					85	85			
Astraptes egregius	1870	C	14	2		11	11	11	reared few
Astraptes egregiusDHJ01					4	4			no difference in facies or genitalia
Astraptes egregiusDHJ02					7	7			no difference in facies or genitalia
Astraptes enotrus	1781	B	686	1		260	120	116	probing extreme food plant ecology variation
Astraptes fulgerator group	1867	C	4757	11		1916	1223	1130	found early to be more than one barcode
Astraptes BYTTNER					6	4			perfect food plant match
Astraptes CELT					327	134			
Astraptes ENTA					4	3			perfect food plant match
Astraptes FABOV					107	72			confused with other species at a glance
Astraptes HIHAMP					81	46			
Astraptes INGCUP					745	278			probing food plant ecology variation
Astraptes INGCUPnumt					1	1			
Astraptes LOHAMP					1213	596			probing microgeographic ecology variation
Astraptes LONCHO					96	62			probing microgeographic ecology variation
Astraptes MYST					7	7			

species name in database http://janzen.sas.upenn.edu	YEAR DESCRIBED	category A=singleton B =normal C=BC split	# rearing records total for morphospecies	number of barcode groups in morphological unit	# in split	# spread in SI, INBio, & McGuire Center	# sampled & sent to BIO	# successful barcodes	why a sample of this size
Astraptes SENNOV					583	171			probing microgeographic ecology variation
Astraptes SENNOVnumt					6	6			
Astraptes TRIGO					362	158			probing food plant ecology variation
Astraptes YESENN					1202	365			probing microgeographic ecology variation
Astraptes YESENNnumt					6	6			
Astraptes hopfferi	1881	C	451	2		135	72	72	found early to be more than one barcode
Astraptes hopfferiDHJ01					10	10			no difference in facies or genitalia
Astraptes hopfferiDHJ02					62	62			no difference in facies or genitalia
Astraptes janeira	1902	C	28	2		21	18	16	probing food plant ecology variation
Astraptes janeiraDHJ01					2	2			definitely another species by facies and genitalia
Astraptes janeiraDHJ02					18	18			definitely another species by facies and genitalia
Astraptes phalaecus	1893	B	3	1		3	3	3	reared few
Astraptes talus	1771	B	189	1		91	28	26	probing food plant ecology variation
Astraptes tucuti	1927	B	135	1		79	24	23	probing food plant ecology variation
Atarnes sallei	1867	B	926	1		144	46	27	probing microgeographic ecology variation
Autochton Burns01		C	335	4		189	156	142	found early to be more than one barcode
Autochton Burns01DHJ02					61	61			
Autochton Burns01DHJ03					38	38			
Autochton Burns01DHJ04					49	49			
Autochton Burns01DHJ05					17	17			
Autochton bipunctatus	1790	A	1	single record	1	1		1	reared few
Autochton longipennis	1882	B	4	1		1	1	1	reared few
Autochton zarex	1818	B	19	1		13	12	12	probing food plant ecology variation
Bolla evippe	1896	A	1	1		1	1	1	reared few
Bolla zorilla	1886			4		36	35	33	found early to be more than one barcode
Bolla zorillaDHJ02		B	26	1		22	21	19	confused with other species at a glance
Bolla zorillaDHJ09		B	6	1		6	6	6	reared few
Bolla zorillaDHJ11		B	4	1		4	4	4	reared few
Bolla zorillaDHJ13		B	4	1		4	4	4	reared few
Bungalotis astylos	1780	B	287	1		61	25	25	probing microgeographic ecology variation
Bungalotis diophorus	1883	B	635	1		90	26	25	probing microgeographic ecology variation
Bungalotis erythus	1775	B	724	1		223	30	29	probing microgeographic ecology variation
Bungalotis midas	1775	B	38	1		13	13	13	probing food plant ecology variation
Bungalotis quadratum	1845	C	1019	2		315	161	157	found early to be more than one barcode
Bungalotis quadratumDHJ01					117	117			
Bungalotis quadratumDHJ02					40	40			
Cabares potrillo	1857	B	109	1		33	7	6	reared few
Calliades zeutus	1879	B	361	1		79	17	17	suspected would be two
Camptopleura theramenes	1877	B	2	1		2	2	2	reared few
Camptopleura auxo	1879	C	19	2		18	18	17	confused with other species at a glance
Camptopleura auxoDHJ01					6	6			no difference in facies or genitalia or food plants
Camptopleura auxoDHJ02					11	11			no difference in facies or genitalia or food plants
Carrhenes calidius	1895	B	210	1		107	36	35	confused with other species at a glance
Carrhenes canescens	1869	C	213	2		109	24	22	confused with other species at a glance
Carrhenes canescensDHJ01					10	10			
Carrhenes canescensDHJ02					11	11			
Carrhenes fuscescens	1891	B	56	1		27	2	2	process error, intended to send more
Carrhenes meridensis	1895	A	1	single record		1	1	1	reared few
Celaenorrhinus approximatus	1940	B	180	1		81	21	21	probing food plant ecology variation
Celaenorrhinus Burns01		B	714	1		270	57	57	confused with other species at a glance
Celaenorrhinus Burns03		B	30	1		29	28	28	confused with other species at a glance

species name in database http://janzen.sas.upenn.edu	YEAR DESCRIBED	category A=singleton B =normal C=BC split	# rearing records total for morphospecies	number of barcode groups in morphological unit	# in split	# spread in SI, INBio, & McGuire Center	# sampled & sent to BIO	# successful barcodes	why a sample of this size
Celaenorrhinus eligius		B	229	1		116	44	42	confused with other species at a glance
Celaenorrhinus fritzgaertneri	1880	B	107	1		53	13	12	
Celaenorrhinus stallingsi	1946	B	458	1		183	21	19	probing food plant ecology variation
Cephise aelius	1880	B	447	1		143	18	17	probing microgeographic ecology variation
Cephise Burns01		B	105	1		72	50	48	confused with other species at a glance
Cephise nuspesez	1996	C	1413	3		454	64	61	found early to be more than one barcode
Cephise nuspesezDHJ01					5	5			
Cephise nuspesezDHJ02					58	58			
Cephise nuspesezDHJ03					1	1			
Chioides catillus	1779	B	591	1		192	25	25	probing food plant ecology variation
Chioides zilpa	1872	B	40	1		15	14	14	
Chiomara georgina	1868	B	428	1		98	18	17	
Chiomara mithrax	1879	A	1	single record		1	1	1	reared few
Chrysoplectrum Burns01		B	412	1		132	30	28	confused with other species at a glance
Chrysoplectrum Burns02		B	5	1		1	1	1	reared few
Chrysoplectrum pervivax	1819	B	154	1		22	16	16	
Clito aberrans	1924	B	366	1		66	16	16	
Clito Burns01		B	101	1		9	8	6	reared few, undescribed species
Codatractus alcaeus	1867	B	4	1		4	3	3	reared few
Codatractus carlos	1952	B	1	1		1	1	1	reared few
Codatractus imalena	1872	B	131	1		72	37	29	confused with other species at a glance
Codatractus melon	1893	B	22	1		13	9	8	confused with other species at a glance
Cogia calchas	1869	C	673	2		87	28	27	found to be more than one barcpde
Cogia calchasDHJ01					20	20			
Cogia calchasDHJ02					7	7			
Cogia eluina	1894	B	491	1		95	20	20	probing microgeographic ecology variation
Cogia hiska	1953	B	87	1		24	17	17	
Creonpyge creon	1874	B	22	1		12	12	10	
Cycloglypha enega	1877	A	1	single record		1	1	1	reared few
Cycloglypha thrasibulus	1793	B	293	1		114	41	39	confused with other species at a glance
Cyclosemia anastomosis	1878	B	53	1		36	28	27	suspected would be two
Cyclosemia Burns01		B	127	1		72	35	35	confused with other species at a glance
Cyclosemia subcaerulea	1913	B	125	1		40	7	6	
Doberes anticus	1884	B	55	1		25	4	3	reared few
Drephalys alcmon	1779	B	147	1		30	12	11	
Drephalys Burns01		B	127	1		21	19	19	probing microgeographic ecology variation
Drephalys kidonoi	2000	B	427	1		98	18	18	probing microgeographic ecology variation
Dyscophellus Burns01		B	696	1		233	70	67	confused with other species at a glance
Dyscophellus Burns02		B	68	1		26	25	24	confused with other species at a glance
Dyscophellus phraxanor	1876	C	1169	4		370	248	163	found early to be more than one barcode
Dyscophellus phraxanorDHJ01					40	40			morphologically polymorphic
Dyscophellus phraxanorDHJ02					162	162			morphologically polymorphic
Dyscophellus phraxanorDHJ03					36	36			morphologically polymorphic
Dyscophellus phraxanorDHJ04					5	5			morphologically polymorphic
Dyscophellus porcius	1862	B	2	1		2	2	2	reared few
Dyscophellus ramon	1952	B	293	1		112	57	56	confused with other species at a glance
Eantis thraso	1807	B	387	1		90	16	16	confused with other species at a glance
Ebrietas anacreon	1876	C	156	4		80	61	52	found early to be more than one barcode
Ebrietas anacreonDHJ02					14	14			morphologically to variable to say anything
Ebrietas anacreonDHJ03					33	33			morphologically to variable to say anything
Ebrietas anacreonDHJ04					2	2			need more specimens

species name in database http://janzen.sas.upenn.edu	YEAR DESCRIBED	category A=singleton B =normal C=BC split	# rearing records total for morphospecies	number of barcode groups in morphological unit	# in split	# spread in SI, INBio, & McGuire Center	# sampled & sent to BIO	# successful barcodes	why a sample of this size
Ebrietas evanidus	1898	B	3	1		3	3	3	reared few, discovered by barcoding
Ebrietas osyris	1876	B	4	1		4	4	4	reared few
Elbella merops	1934	B	21	1		5	5	5	
Elbella miodesmiata	1925	B	2	1		1	1	1	
Elbella patrobas	1857	B	26	1		6	6	6	
Elbella patrobasDHJ05		B	14	1		1	1	0	reared few, all but one larva died
Elbella scylla	1855	B	425	1		134	31	30	probing microgeographic ecology variation
Entheus Burns01		B	385	1		178	57	55	probing microgeographic ecology variation
Entheus Burns02		B	153	1		68	28	28	confused with other species at a glance
Entheus Burns03		B	164	1		74	39	39	confused with other species at a glance
Epargyreus Burns02		B	1148	1		505	49	44	probing microgeographic ecology variation
Epargyreus Burns03		B	205	1		51	25	22	
Epargyreus Burns04		B	54	1		24	18	16	
Epargyreus Burns05		B	157	1		93	29	19	
Epargyreus Burns06		B	25	1		21	8	6	reared few
Epargyreus Burns07		B	146	1		55	16	13	
Epargyreus Burns09		B	1	1		1	1	0	reared few
Epargyreus Burns10		B	4	1		2	1	0	reared few
Epargyreus Burns11		B	327	1		110	41	17	
Epargyreus Burns12		B	75	1		7	7	6	reared few
Ephyriades eugramma	1888	B	25	1		18	9	9	reared few
Eracon clinias	1878	C	77	2		36	30	29	found early to be more than one barcode
Eracon cliniasDHJ01					28	28			no difference in facies or genitalia or food plants
Eracon cliniasDHJ02					2	2			no difference in facies or genitalia or food plants
Eracon lachesis	1918	B	560	1		158	33	32	probing variation in food plant ecology
Erynnis tristis	1852	B	249	1		85	27	26	
Gesta gesta	1863	B	363	1		37	13	13	
Gindanes brontinus	1895	B	74	1		14	12	12	
Gorgythion begga pyralina	1877	C	553	2		268	237	231	found early to be more than one barcode
Gorgythion begga pyralinaDHJ01					99	78			no difference in facies or genitalia or food plants
Gorgythion begga pyralinaDHJ02					175	153			no difference in facies or genitalia or food plants
Grais stigmaticus	1883	B	115	1		26	2	1	reared few
Helias cama	1953	B	60	1		43	27	27	confused with other species at a glance
Heliopetes alana	1868	B	6	1		6	5	5	reared few
Heliopetes arsalte	1758	B	26	1		21	12	12	
Heliopetes laviana	1868	B	90	1		47	22	19	suspected would be two
Heliopetes macaira	1867	B	16	1		10	2	2	specimens old and reared few
Heliopyrgus domicella	1849	B	40	1		20	0	0	overlooked species
Hyalothyrus neleus	1758	B	582	1		123	23	22	probing microgeographic ecology variation
Iliana Burns01		B	6	1		2	2	2	reared few
Jemadia Burns01		B	68	1		16	13	13	
Jemadia pseudognetus	1878	B	375	1		151	26	27	confused with other species at a glance
Jonaspyge aesculapus	1876	B	24	1		12	12	12	
Melanopyge Burns01		B	55	1		17	17	16	confused with other species at a glance
Melanopyge erythrosticta	1879	B	5	1		4	4	4	reared few
Mictris crispus caerula	1870	B	81	1		37	25	24	
Milanion marciana	1895	B	208	1		63	12	12	
Morvina fissimacula pelarge	1894	B	168	1		55	12	12	
Mylon lassia	1868	B	199	1		57	23	22	
Mylon maimon	1775	B	132	1		52	15	15	
Mylon pelopidas	1793	B	103	1		33	22	22	

species name in database http://janzen.sas.upenn.edu	YEAR DESCRIBED	category A=singleton B=normal C=BC split	# rearing records total for morphospecies	number of barcode groups in morphological unit	# in split	# spread in SI, INBio, & McGuire Center	# sampled & sent to BIO	# successful barcodes	why a sample of this size
Mylon salvia	1953	B	24	1		15	13	13	
Myscelus amystis	1867	B	179	1		64	20	15	
Myscelus assaricus michaeli	1975	B	18	1		13	13	13	
Myscelus belti	1879	B	626	1		182	30	30	confused with other species at a glance
Myscelus perissodora	1914	B	3	1		3	2	2	reared few
Mysoria ambigua	1908	B	1440	1		197	21	21	
Narcosius colossus	1869	B	453	1		211	36	33	confused with other species at a glance
Narcosius helen	1952	B	38	1		14	9	8	reared few
Narcosius nazaraeus	1986	A	1	single record		1	1	1	known only from single wild caught pupa
Narcosius samson	1952	B	395	1		153	30	29	confused with other species at a glance
Nascus broteas	1780	B	362	1		126	38	36	confused with other species at a glance
Nascus Burns01		B	67	1		38	22	16	confused with other species at a glance
Nascus Burns02		B	224	1		98	32	29	confused with other species at a glance
Nascus paulliniae	1842	B	604	1		170	21	21	
Nascus phintias	1913	B	52	1		24	16	15	probing food plant ecology variation
Nascus solon	1882	B	174	1		72	34	33	confused with other species at a glance
Nicephellus nicephorus	1876	B	36	1		8	2	2	reared few
Nisoniades ephora	1870	B	4	1		2	2		reared few, discovered by barcoding
Nisoniades castolus	1878	B	218	1		87	29	26	confused with other species at a glance
Nisoniades godma	1953	B	789	1		253	48	46	confused with other species at a glance
Nisoniades rubescens	1877		15	2		12	12	12	reared few
Nisoniades rubescensDHJ01			1	1	1	1			reared few, discovered by barcoding
Nisoniades rubescensDHJ02			14		11	11			reared few, discovered by barcoding
Nisoniades torta	1989	C	4	2		4	4	4	reared few
Nisoniades tortaDHJ02			5	1	3	3			reared few, discovered by barcoding
Nisoniades tortaDHJ01				1	1	1			reared few, discovered by barcoding
Noctuana lactifera	1872	B	37	1		21	2	2	reared few
Noctuana stator	1899	B	87	1		48	35	34	confused with other species at a glance
Ocyba calathana	1868	B	197	1		45	12	9	reared few
Ouleus Burns01		B	5	1		5	5	5	reared few
Ouleus cyrna	1895	B	6	1		6	5	4	reared few
Ouleus dilla baru	1973	C	70	2		43	23	23	confused with other species at a glance
Ouleus dilla baruDHJ01					4				
Ouleus dilla baruDHJ02					19				
Ouleus negrus	1980	B	10	1			10	10	reared few
Ouleus salvina	1953	C	145	2		109	76	74	confused with other species at a glance
Ouleus salvinaDHJ01					59	47			
Ouleus salvinaDHJ02					32	27			
Oxynetra hopfferi	1888	B	29	1		10	10	10	reared few, confused with another *Oxynetra*
Paches loxus	1852	B	183	1		31	17	17	
Pachyneuria licisca	1882	B	122	1		35	21	20	
Parelbella macleannani	1893	B	488	1		108	22	19	
Passova gellias	1893	B	532	1		143	32	31	suspected would be two
Pellicia arina	1953	B	258	1		56	14	12	
Pellicia dimidiata	1870	B	117	1		40	16	16	
Pellicia Janzen01		B	2	1		2	2	2	reared few
Phanus marshallii	1880	C	78	2		50	16	16	found to be more than one barcode
Phanus marshalliiDHJ01					5	5			cannot separate by facies or genitalia from DHJ02
Phanus marshalliiDHJ02					11	11			cannot separate by facies or genitalia from DHJ01
Phanus obscurior	1925	B	87	1		54	32	31	confused with other species at a glance
Phanus vitreus	1781	C	311	2		117	83	83	found early to be more than one barcode

species name in database http://janzen.sas.upenn.edu	YEAR DESCRIBED	category A=singleton B =normal C=BC split	# rearing records total for morphospecies	number of barcode groups in morphological unit	# in split	# spread in SI, INBio, & McGuire Center	# sampled & sent to BIO	# successful barcodes	why a sample of this size
Phanus vitreusDHJ01					62	62			cannot separate by facies or genitalia from DHJ03
Phanus vitreusDHJ02					20	19			distinct genitalia differences from DHJ01
Phanus vitreusDHJ03					1	1			cannot separate by facies or genitalia from DHJ01
Phareas coeleste	1852	B	28	1		10	9	9	reared few
Phocides belus	1893	B	138	1		55	47	46	confused with other species at a glance
Phocides Burns01		B	7	1		7	5	4	reared few
Phocides lilea	1867	B	241	1		75	36	35	probing microgeographic ecology variation
Phocides nigrescens	1938	B	564	1		240	50	48	confused with other species at a glance
Phocides pigmalion	1779	C	122	2		42	41	40	confused with other species at a glance
Phocides pigmalionDHJ01					69	23			
Phocides pigmalionDHJ02					52	19			
Phocides Warren01		B	130	1		47	29	29	confused with other species at a glance
Polyctor cleta	1953	B	163	1		93	31	20	confused with other species at a glance
Polyctor enops	1894	B	56	1		41	27	27	
Polyctor polyctor	1868	B	353	1		192	93	82	confused with other species at a glance
Polygonus leo	1790	B	596	1		125	20	17	
Polygonus savigny	1824	C	44	2		30	28	28	found to be more than one barcode
Polygonus savignyDHJ01					13	13			
Polygonus savignyDHJ02					15	15			
Polythrix asine	1867	C	931	3		248	230	222	found to be more than one barcode
Polythrix asineDHJ01					104	104			
Polythrix asineDHJ02					118	117			
Polythrix asineDHJ04					2	2			different genitalia
Polythrix auginus	1867	B	19	1		15	15	12	
Polythrix caunus	1869	B	179	1		66	38	36	confused with other species at a glance
Polythrix kanshul	1991	B	43	1		15	13	12	
Polythrix mexicanus	1969	B	40	1		39	28	28	
Polythrix octomaculata	1844	B	98	1		32	13	12	
Porphyrogenes peterwegei	2010	B	250	1		100	77	74	confused with other species at a glance
Porphyrogenes sula	1940	B	2	1		1	1	1	reared few
Potamanaxas Burns01		B	2	1		2	2	2	reared few
Potamanaxas Burns02		B	4	1		3	3	3	reared few
Potamanaxas Burns03		B	3	1		3	3	3	reared few
Potamanaxas unifasciata	1867	B	131	1		60	26	26	
Proteides mercurius	1787	B	357	1		41	23	18	
Pyrrhopyge crida	1871								
Pyrrhopyge zenodorus	1893								
Pythonides amaryllis	1876	B	303	1		90	29	28	
Pythonides proxenus	1895	B	151	1		73	25	25	
Pythonides pteras	1895	B	28	1		8	6	6	reared few
Quadrus cerialis	1782	C	1658	3		293	47	47	found to be more than one barcode
Quadrus cerialisDHJ01					1	1			no difference in facies or genitalia
Quadrus cerialisDHJ02					1	1			no difference in facies or genitalia
Quadrus cerialisDHJ03					44	43			
Quadrus contubernalis	1883	B	64	1		32	22	20	
Quadrus francesius	1969	B	109	1		48	21	20	
Quadrus lugubris	1869	B	138	1		68	22	10	
Ridens biolleyi	1900	B	210	1		17	16	15	
Ridens Burns01		B	59	1		26	13	13	
Ridens cachinnans	1901	B	9	1		3	6	6	reared few
Ridens mephitis	1876	C	143	3		55	54	51	found early to be more than one barcode

species name in database http://janzen.sas.upenn.edu	YEAR DESCRIBED	category A=singleton B =normal C=BC split	# rearing records total for morphospecies	number of barcode groups in morphological unit	# in split	# spread in SI, INBio, & McGuire Center	# sampled & sent to BIO	# successful barcodes	why a sample of this size
Ridens mephitisDHJ02					21	12			
Ridens mephitisDHJ03					79	33			
Ridens mephitisDHJ04					9	9			
Ridens panche	1927	C	301	2		124	56	55	found to be more than one barcode
Ridens pancheDHJ01					4	4			need more specimens, which is R. panche?
Ridens pancheDHJ02					52	52			need more specimens, which is R. panche?
Salatis canalis	1920	B	91	1		32	25	24	
Sostrata bifasciata nordica	1953	B	439	1		161	64	60	suspected would be two
Sostrata pusilla	1895	B	44	1		25	15	13	
Spathilepia clonius	1775	B	235	1		50	16	14	
Spioniades abbreviata	1888	B	23	1		14	7	7	reared few
Spioniades artemides	1782	B	1	1		1	1	1	reared few
Staphylus ascalaphus	1876	B	31	1		22	22	22	confused with other species at a glance
Staphylus azteca	1896	B	91	1					confused with other species at a glance
Staphylus caribbea	1940	B	46	1		38	38	37	confused with other species at a glance
Staphylus evemerus		B	65	1		42	18	18	
Staphylus Janzen03		B	10	1		1	1	1	reared few
Staphylus Janzen08		B	4	1		4	3	3	reared few
Staphylus Janzen10		A	1	single record		1	1	1	reared few
Staphylus vulgata	1879	B	404	1		123	74	74	confused with other species at a glance
Telemiades antiope	1882	C	705	4		316	201	191	found to be more than one
Telemiades antiopeDHJ01					77	77			
Telemiades antiopeDHJ02					57	57			
Telemiades antiopeDHJ03					24	24			
Telemiades antiopeDHJ04					33	33			
Telemiades avitus	1781	B	6	1		3	3	3	reared few
Telemiades Burns01		B	24	1		24	23	23	
Telemiades Burns02		B	10	1		5	5	5	reared few
Telemiades Burns03		B	1	1		1	1	1	reared few
Telemiades Burns08		B	3	1		3	3	3	reared few
Telemiades chrysorrhoea	1893	C	282	2		177	88	74	found early to be more than one barcode
Telemiades chrysorrhoeaDHJ01					41	41			pseudogene, entirely females
Telemiades chrysorrhoeaDHJ02					33	33			real barcode, males and females
Telemiades fides	1949	B	2025	1		467	264	262	searching for rare included other species
Telemiades gallius	1888	B	8	1		2	2	2	reared few
Telemiades megallus	1888	B	59	1		32	32	30	
Telemiades nicomedes	1879	B	49	1		28	27	26	
Telemiades oiclus	1889	B	127	1		51	21	20	
Thessia jalapus	1881	B	256	1		125	25	25	confused with other species at a glance
Timochares trifasciata	1868	B	492	1		70	17	15	
Timochreon satyrus	1867	B	146	1		51	24	23	
Tosta gorgus	1937	B	122	1		44	8	8	reared few
Tosta niger	1940	B	26	1		9	5	5	reared few
Tosta platypterus	1895	B	323	1		141	25	25	confused with other species at a glance
Typhedanus ampyx	1893	B	85	1		49	8	7	reared few
Typhedanus undulatus	1867	B	194	1		60	11	10	
Udranomia eurus	1919	A	1	single record		1	1	1	reared few
Udranomia kikkawai	1906	C	348	4		355	194	189	found early to be more than one barcode
Udranomia kikkawaiDHJ01					811	111			
Udranomia kikkawaiDHJ02					494	136			
Udranomia kikkawaiDHJ03					31	31			

species name in database http://janzen.sas.upenn.edu	YEAR DESCRIBED	category A=singleton B =normal C=BC split	# rearing records total for morphospecies	number of barcode groups in morphological unit	# in split	# spread in SI, INBio, & McGuire Center	# sampled & sent to BIO	# successful barcodes	why a sample of this size
Udranomia kikkawaiDHJ04					29	29			
Udranomia orcinus	1867	B	150	1		94	39	38	probing microgeographic ecology variation
Urbanus albimargo	1876	B	126	1		38	11	7	reared few
Urbanus belli	1935	C	534	3		319	287	279	found early to be more than one barcode
Urbanus belliDHJ01					189	178			
Urbanus belliDHJ02					77	68			
Urbanus belliDHJ03					34	33			
Urbanus dorantes	1790	B	209	1		79	16	16	
Urbanus doryssus	1831	C	330	3		203	86	83	found to be more than one
Urbanus doryssusDHJ01					78	32			
Urbanus doryssusDHJ02					228	90			
Urbanus doryssusDHJ03					2	2			
Urbanus esmeraldus	1877	B	386	1		172	148	140	confused with other species at a glance
Urbanus esta	1952	B	262	1		143	84	82	confused with other species at a glance
Urbanus evona	1952	B	38	1		19	18	18	confused with other species at a glance
Urbanus pronta	1952	B	302	1		85	24	22	confused with other species at a glance
Urbanus proteus	1758	B	946	1		505	421	417	confused with other species at a glance
Urbanus simplicius	1790	B	204	1		66	14	14	
Urbanus teleus	1821	B	32	1		17	9	8	reared few
Urbanus viterboana	1907	B	143	1		34	26	17	
Venada cacao	2005	B	27	2		8	8	8	reared few
Venada cacaoDHJ01					2	2			facies slightly different, undescribed species
Venada cacaoDHJ02					6	6			facies slightly different, undescribed species
Venada daneva	2005	B	209	1		88	27	23	confused with other species at a glance
Venada naranja	2005	B	63	1		26	24	19	confused with other species at a glance
Venada nevada	2005	B	529	1		288	43	39	probing microgeographic ecology variation
Xenophanes tryxus	1780	B	782	1		190	32	32	probing microgeographic ecology variation
Yanguna cosyra	1875	B	1048	1		280	23	23	
Zera hosta	1953	B	27	1		5	5	5	reared few
Zera Burns01		C	18	2		18	17	17	reared few
Zera Burns01DHJ02					15	14			genitalia different, undescribed species
Zera Burns01DHJ03					3	3			genitalia different, undescribed species
Zopyrion sandace			2			2	0	N/A	overlooked species
TOTALS			65298		9184	27047	9701	9095	
