# Supplementary material for: Reading the Complex Skipper Butterfly Fauna of One Tropical Place
Source: PLoS One. 2011 Aug 16;6(8):e19874. doi: 10.1371/journal.pone.0019874 (PMC3156701; doi:10.1371/journal.pone.0019874)
Supplement: Table S2 — Accession codes for all specimens that are considered in Table S1. (PDF) [file pone.0019874.s003.pdf]

Table S2. Accession codes for all specimens considered in Table S1.

Tree Order	Species	Subfamily	ACG Sampleid	BOLD Processid	Genbank Accession
1	Typhedanus ampyx	Pyrginae	95-SRNP-4704	MHAHH757-06	GU155675
2	Typhedanus ampyx	Pyrginae	02-SRNP-13978	MHAHH758-06	GU155676
3	Typhedanus ampyx	Pyrginae	03-SRNP-27074	MHAHH753-06	GU155674
4	Typhedanus ampyx	Pyrginae	04-SRNP-15699	MHAHE155-05	GU150132
5	Typhedanus ampyx	Pyrginae	02-SRNP-13972	CSCR269-04	DQ293621
6	Typhedanus ampyx	Pyrginae	06-SRNP-19495	MHAHI530-06	GU156375
7	Typhedanus ampyx	Pyrginae	08-SRNP-16243	MHMXY1095-09	GU666443
8	Niconiades gladys	Pyrginae	08-SRNP-40802	MHMXX986-09	JF778133
9	Niconiades gladys	Pyrginae	08-SRNP-41006	MHMXX985-09	JF778132
10	Niconiades gladys	Pyrginae	08-SRNP-41005	MHMXX984-09	JF778131
11	Niconiades gladys	Pyrginae	08-SRNP-41008	MHMXX983-09	JF778130
12	Niconiades gladys	Pyrginae	08-SRNP-41007	MHMXX982-09	JF778129
13	Niconiades gladys	Pyrginae	07-SRNP-41299	MHAHL396-07	JF762437
14	Niconiades gladys	Pyrginae	07-SRNP-40880	MHAHL393-07	JF762435
15	Niconiades gladys	Pyrginae	07-SRNP-41499	MHAHL392-07	JF762434
16	Niconiades gladys	Pyrginae	07-SRNP-40878	MHAHL391-07	JF762433
17	Niconiades gladys	Pyrginae	07-SRNP-42460	MHMXO956-08	JF762439
18	Niconiades gladys	Pyrginae	07-SRNP-41297	MHAHL395-07	JF762436
19	Niconiades gladys	Pyrginae	07-SRNP-42104	MHMXO957-08	JF762438
20	Niconiades gladys	Pyrginae	08-SRNP-40804	MHMXX987-09	JF778134
21	Niconiades gladys	Pyrginae	08-SRNP-40803	MHMXX988-09	JF778135
22	Niconiades xanthaphes	Pyrginae	07-SRNP-40674	MHMXK006-07	JF762444
23	Niconiades xanthaphes	Pyrginae	08-SRNP-24219	MHMYB136-09	HM893823
24	Niconiades xanthaphes	Pyrginae	04-SRNP-48297	MHAHE033-05	GU149763
25	Niconiades xanthaphes	Pyrginae	09-SRNP-21365	MHMYH152-10	HM887300
26	Niconiades xanthaphes	Pyrginae	09-SRNP-20105	MHMYB135-09	GU649692
27	Niconiades xanthaphes	Pyrginae	07-SRNP-40588	MHMXK005-07	JF762445
28	Niconiades xanthaphes	Pyrginae	06-SRNP-40704	MHAHG674-06	GU151494
29	Niconiades xanthaphes	Pyrginae	08-SRNP-45150	MHMXX980-09	JF778136
30	Niconiades xanthaphes	Pyrginae	07-SRNP-41908	MHAHL398-07	JF762441
31	Niconiades xanthaphes	Pyrginae	07-SRNP-41909	MHAHL397-07	JF762440
32	Niconiades xanthaphes	Pyrginae	06-SRNP-41203	MHAHG675-06	GU151495
33	Niconiades xanthaphes	Pyrginae	04-SRNP-13906	MHAHE031-05	GU149767
34	Niconiades xanthaphes	Pyrginae	04-SRNP-13942	MHAHE030-05	GU149765
35	Niconiades xanthaphes	Pyrginae	04-SRNP-23563	MHAHE029-05	GU149766
36	Niconiades xanthaphes	Pyrginae	01-SRNP-17553	CSCR158-04	DQ292760
37	Niconiades xanthaphes	Pyrginae	02-SRNP-30345	CSCR159-04	DQ292761
38	Niconiades xanthaphes	Pyrginae	04-SRNP-14330	MHAHE032-05	GU149764
39	Niconiades xanthaphes	Pyrginae	08-SRNP-274	MHMXT152-08	JF762443
40	Niconiades xanthaphes	Pyrginae	08-SRNP-273	MHMXT153-08	JF762442
41	Niconiades xanthaphes	Pyrginae	08-SRNP-24218	MHMYB137-09	GU649687
42	Niconiades xanthaphes	Pyrginae	09-SRNP-68536	MHMYI586-10	HQ963902
43	Celaenorrhinus stallingsi	Pyrginae	94-SRNP-569	MHMXI582-07	JF760536
44	Celaenorrhinus stallingsi	Pyrginae	06-SRNP-47719	MHAHJ878-07	JF752566
45	Celaenorrhinus stallingsi	Pyrginae	04-SRNP-27175	MHAHI320-06	GU155894
46	Celaenorrhinus stallingsi	Pyrginae	05-SRNP-48589	MHAHG193-06	GU151298
47	Celaenorrhinus stallingsi	Pyrginae	04-SRNP-45627	MHAHC155-05	DQ292083
48	Celaenorrhinus stallingsi	Pyrginae	04-SRNP-46658	MHAHC147-05	DQ292082
49	Celaenorrhinus stallingsi	Pyrginae	04-SRNP-45628	MHAHC139-05	DQ292081
50	Celaenorrhinus stallingsi	Pyrginae	04-SRNP-45248	MHAHC133-05	DQ292080
51	Celaenorrhinus stallingsi	Pyrginae	04-SRNP-45054	MHAHC125-05	DQ292079
52	Celaenorrhinus stallingsi	Pyrginae	03-SRNP-29104	MHAHC117-05	DQ292078
53	Celaenorrhinus stallingsi	Pyrginae	04-SRNP-45303	MHAHC101-05	DQ292077
54	Celaenorrhinus stallingsi	Pyrginae	03-SRNP-29103	CSCR492-04	DQ292074
55	Celaenorrhinus stallingsi	Pyrginae	02-SRNP-8079	CSCR065-04	DQ292073

Tree Order	Species	Subfamily	ACG Sampleid	BOLD Processid	Genbank Accession
56	Celaenorrhinus stallingsi	Pyrginae	04-SRNP-45199	MHAHC056-05	DQ292075
57	Celaenorrhinus stallingsi	Pyrginae	04-SRNP-45280	MHAHC072-05	DQ292076
58	Celaenorrhinus stallingsi	Pyrginae	05-SRNP-24342	MHAHK019-07	JF760535
59	Celaenorrhinus stallingsi	Pyrginae	07-SRNP-35248	MHMXK018-07	JF761898
60	Celaenorrhinus stallingsi	Pyrginae	08-SRNP-35005	MHMXX1040-09	JF777761
61	Celaenorrhinus stallingsi	Pyrginae	08-SRNP-45153	MHMXX1045-09	JF777762
62	Celaenorrhinus fritzgaertneri	Pyrginae	07-SRNP-58872	MHMXR892-08	JF761896
63	Celaenorrhinus fritzgaertneri	Pyrginae	07-SRNP-56429	MHMXK014-07	JF761897
64	Celaenorrhinus fritzgaertneri	Pyrginae	05-SRNP-60702	MHAHK030-07	JF760534
65	Celaenorrhinus fritzgaertneri	Pyrginae	06-SRNP-19668	MHAHK020-07	JF760532
66	Celaenorrhinus fritzgaertneri	Pyrginae	04-SRNP-46882	MHAHC163-05	DQ292072
67	Celaenorrhinus fritzgaertneri	Pyrginae	04-SRNP-47171	MHAHC123-05	DQ292070
68	Celaenorrhinus fritzgaertneri	Pyrginae	93-SRNP-6905	CSRII436-04	DQ292069
69	Celaenorrhinus fritzgaertneri	Pyrginae	04-SRNP-47172	MHAHC131-05	DQ292071
70	Celaenorrhinus fritzgaertneri	Pyrginae	93-SRNP-6145	CSRII435-04	DQ292068
71	Celaenorrhinus fritzgaertneri	Pyrginae	95-SRNP-9563	CSCR062-04	DQ292067
72	Celaenorrhinus fritzgaertneri	Pyrginae	05-SRNP-64117	MHAHK027-07	JF760533
73	Celaenorrhinus fritzgaertneri	Pyrginae	09-SRNP-13046	MHMYB150-09	GU649688
74	Celaenorrhinus approximatus	Pyrginae	03-SRNP-4049	CSCR490-04	DQ292027
75	Celaenorrhinus approximatus	Pyrginae	04-SRNP-35283	MHAHC107-05	DQ292031
76	Celaenorrhinus approximatus	Pyrginae	04-SRNP-35231	MHAHC099-05	DQ292029
77	Celaenorrhinus approximatus	Pyrginae	04-SRNP-35236	MHAHC180-05	DQ292044
78	Celaenorrhinus approximatus	Pyrginae	04-SRNP-35375	MHAHC148-05	DQ292039
79	Celaenorrhinus approximatus	Pyrginae	04-SRNP-35276	MHAHC140-05	DQ292037
80	Celaenorrhinus approximatus	Pyrginae	04-SRNP-35243	MHAHC132-05	DQ292036
81	Celaenorrhinus approximatus	Pyrginae	04-SRNP-35230	MHAHC124-05	DQ292035
82	Celaenorrhinus approximatus	Pyrginae	04-SRNP-35247	MHAHC116-05	DQ292034
83	Celaenorrhinus approximatus	Pyrginae	04-SRNP-35245	MHAHC108-05	DQ292032
84	Celaenorrhinus approximatus	Pyrginae	04-SRNP-35242	MHAHC100-05	DQ292030
85	Celaenorrhinus approximatus	Pyrginae	04-SRNP-35234	MHAHC173-05	DQ292043
86	Celaenorrhinus approximatus	Pyrginae	04-SRNP-35239	MHAHC165-05	DQ292042
87	Celaenorrhinus approximatus	Pyrginae	04-SRNP-35233	MHAHC157-05	DQ292041
88	Celaenorrhinus approximatus	Pyrginae	04-SRNP-35235	MHAHC149-05	DQ292040
89	Celaenorrhinus approximatus	Pyrginae	04-SRNP-35282	MHAHC141-05	DQ292038
90	Celaenorrhinus approximatus	Pyrginae	04-SRNP-35280	MHAHC109-05	DQ292033
91	Celaenorrhinus approximatus	Pyrginae	03-SRNP-4053	CSCR491-04	DQ292028
92	Celaenorrhinus approximatus	Pyrginae	04-SRNP-35281	MHAHC181-05	DQ292045
93	Celaenorrhinus approximatus	Pyrginae	06-SRNP-35037	MHAHG649-06	GU151268
94	Celaenorrhinus approximatus	Pyrginae	07-SRNP-35584	MHMXK017-07	JF761867
95	Celaenorrhinus Burns03	Pyrginae	05-SRNP-2519	MHAHI313-06	GU155887
96	Celaenorrhinus Burns03	Pyrginae	06-SRNP-2390	MHAHG643-06	GU151285
97	Celaenorrhinus Burns03	Pyrginae	06-SRNP-2415	MHAHG644-06	GU151284
98	Celaenorrhinus Burns03	Pyrginae	06-SRNP-1475	MHAHG645-06	GU151283
99	Celaenorrhinus Burns03	Pyrginae	06-SRNP-7150	MHAHJ495-07	JF752565
100	Celaenorrhinus Burns03	Pyrginae	05-SRNP-570	MHAHG093-06	GU151281
101	Celaenorrhinus Burns03	Pyrginae	05-SRNP-567	MHAHI316-06	GU155890
102	Celaenorrhinus Burns03	Pyrginae	05-SRNP-751	MHAHG081-06	GU151282
103	Celaenorrhinus Burns03	Pyrginae	07-SRNP-1399	MHMXK013-07	JF761885
104	Celaenorrhinus Burns03	Pyrginae	05-SRNP-6010	MHAHK028-07	JF760525
105	Celaenorrhinus Burns03	Pyrginae	05-SRNP-5661	MHAHK025-07	JF760524
106	Celaenorrhinus Burns03	Pyrginae	04-SRNP-26450	MHAHI318-06	GU155892
107	Celaenorrhinus Burns03	Pyrginae	05-SRNP-1346	MHAHI317-06	GU155891
108	Celaenorrhinus Burns03	Pyrginae	05-SRNP-569	MHAHI315-06	GU155889
109	Celaenorrhinus Burns03	Pyrginae	04-SRNP-26452	MHAHI314-06	GU155888
110	Celaenorrhinus Burns03	Pyrginae	05-SRNP-568	MHAHG094-06	GU151280
111	Celaenorrhinus Burns03	Pyrginae	05-SRNP-6011	MHAHK029-07	JF760526

Tree Order	Species	Subfamily	ACG Sampleid	BOLD Processid	Genbank Accession
112	Celaenorrhinus Burns03	Pyrginae	03-SRNP-5449	CSCR494-04	DQ292056
113	Celaenorrhinus Burns03	Pyrginae	06-SRNP-679	MHAHG191-06	GU151287
114	Celaenorrhinus Burns03	Pyrginae	06-SRNP-1485	MHAHG646-06	GU151286
115	Celaenorrhinus Burns03	Pyrginae	05-SRNP-2378	MHAHI311-06	GU155885
116	Celaenorrhinus Burns03	Pyrginae	05-SRNP-752	MHAHI312-06	GU155886
117	Celaenorrhinus Burns03	Pyrginae	05-SRNP-6009	MHAHK023-07	JF760523
118	Celaenorrhinus Burns03	Pyrginae	05-SRNP-5819	MHAHK031-07	JF760527
119	Celaenorrhinus Burns03	Pyrginae	07-SRNP-609	MHMXK023-07	JF761884
120	Celaenorrhinus Burns03	Pyrginae	07-SRNP-1398	MHMXK024-07	JF761883
121	Celaenorrhinus Burns03	Pyrginae	07-SRNP-1659	MHAHL557-07	JF761881
122	Celaenorrhinus Burns03	Pyrginae	07-SRNP-1478	MHAHL559-07	JF761882
123	Celaenorrhinus eligius	Pyrginae	07-SRNP-21870	MHAHL558-07	JF761886
124	Celaenorrhinus eligius	Pyrginae	05-SRNP-34575	MHAHG194-06	GU151296
125	Celaenorrhinus eligius	Pyrginae	07-SRNP-23595	MHMXR894-08	JF761890
126	Celaenorrhinus eligius	Pyrginae	07-SRNP-770	MHMXK019-07	JF761893
127	Celaenorrhinus eligius	Pyrginae	07-SRNP-580	MHMXK015-07	JF761895
128	Celaenorrhinus eligius	Pyrginae	06-SRNP-59174	MHAHK018-07	JF760530
129	Celaenorrhinus eligius	Pyrginae	06-SRNP-1045	MHAHG195-06	GU151297
130	Celaenorrhinus eligius	Pyrginae	06-SRNP-1044	MHAHG190-06	GU151294
131	Celaenorrhinus eligius	Pyrginae	05-SRNP-31037	MHAHG083-06	GU151288
132	Celaenorrhinus eligius	Pyrginae	97-SRNP-11551	MHAHC374-05	DQ292066
133	Celaenorrhinus eligius	Pyrginae	98-SRNP-6383	MHAHC373-05	DQ292065
134	Celaenorrhinus eligius	Pyrginae	04-SRNP-32649	MHAHC048-05	DQ292061
135	Celaenorrhinus eligius	Pyrginae	04-SRNP-45100	MHAHC064-05	DQ292062
136	Celaenorrhinus eligius	Pyrginae	07-SRNP-60093	MHMXO936-08	JF761891
137	Celaenorrhinus eligius	Pyrginae	08-SRNP-65084	MHMXT094-08	JF761889
138	Celaenorrhinus eligius	Pyrginae	07-SRNP-24063	MHMXT095-08	JF761888
139	Celaenorrhinus eligius	Pyrginae	08-SRNP-703	MHMXX1049-09	JF777759
140	Celaenorrhinus eligius	Pyrginae	06-SRNP-2608	MHAHG650-06	GU151291
141	Celaenorrhinus eligius	Pyrginae	07-SRNP-24053	MHMXT096-08	JF761887
142	Celaenorrhinus eligius	Pyrginae	06-SRNP-9991	MHMXK020-07	JF761892
143	Celaenorrhinus eligius	Pyrginae	07-SRNP-55146	MHMXK016-07	JF761894
144	Celaenorrhinus eligius	Pyrginae	05-SRNP-20010	MHAHI319-06	GU155893
145	Celaenorrhinus eligius	Pyrginae	06-SRNP-45187	MHAHG648-06	GU151293
146	Celaenorrhinus eligius	Pyrginae	06-SRNP-45188	MHAHG647-06	GU151292
147	Celaenorrhinus eligius	Pyrginae	06-SRNP-586	MHAHG192-06	GU151295
148	Celaenorrhinus eligius	Pyrginae	05-SRNP-48036	MHAHG092-06	GU151289
149	Celaenorrhinus eligius	Pyrginae	04-SRNP-21218	MHAHC172-05	DQ292064
150	Celaenorrhinus eligius	Pyrginae	06-SRNP-1795	MHAHG652-06	GU151290
151	Celaenorrhinus eligius	Pyrginae	02-SRNP-4737	CSCR061-04	DQ292057
152	Celaenorrhinus eligius	Pyrginae	04-SRNP-30412	CSRII179-04	DQ292060
153	Celaenorrhinus eligius	Pyrginae	04-SRNP-45098	MHAHC088-05	DQ292063
154	Celaenorrhinus eligius	Pyrginae	06-SRNP-59205	MHAHK017-07	JF760529
155	Celaenorrhinus eligius	Pyrginae	06-SRNP-59204	MHAHK016-07	JF760528
156	Celaenorrhinus eligius	Pyrginae	04-SRNP-412	CSRII178-04	DQ292059
157	Celaenorrhinus eligius	Pyrginae	95-SRNP-9367	CSCR493-04	DQ292058
158	Celaenorrhinus eligius	Pyrginae	06-SRNP-47652	MHAHK034-07	JF760531
159	Celaenorrhinus eligius	Pyrginae	08-SRNP-55519	MHMXX1041-09	JF777755
160	Celaenorrhinus eligius	Pyrginae	08-SRNP-65085	MHMXX1042-09	JF777756
161	Celaenorrhinus eligius	Pyrginae	08-SRNP-1523	MHMXX1043-09	JF777757
162	Celaenorrhinus eligius	Pyrginae	08-SRNP-702	MHMXX1047-09	JF777758
163	Celaenorrhinus eligius	Pyrginae	08-SRNP-1062	MHMXX1052-09	JF777760
164	Celaenorrhinus eligius	Pyrginae	08-SRNP-24026	MHMYB151-09	GU649683
165	Celaenorrhinus Burns01	Pyrginae	07-SRNP-322	MHMXK021-07	JF761880
166	Celaenorrhinus Burns01	Pyrginae	06-SRNP-34867	MHAHJ877-07	JF752564
167	Celaenorrhinus Burns01	Pyrginae	04-SRNP-31134	MHAHC080-05	DQ292048

Tree Order	Species	Subfamily	ACG Sampleid	BOLD Processid	Genbank Accession
168	Celaenorrhinus Burns01	Pyrginae	07-SRNP-2116	MHAHL556-07	JF761871
169	Celaenorrhinus Burns01	Pyrginae	06-SRNP-7072	MHAHJ496-07	JF752563
170	Celaenorrhinus Burns01	Pyrginae	06-SRNP-43466	MHAHI495-06	GU155883
171	Celaenorrhinus Burns01	Pyrginae	05-SRNP-30928	MHAHG091-06	GU151273
172	Celaenorrhinus Burns01	Pyrginae	04-SRNP-26417	MHAHG089-06	GU151278
173	Celaenorrhinus Burns01	Pyrginae	04-SRNP-26244	MHAHG088-06	GU151277
174	Celaenorrhinus Burns01	Pyrginae	05-SRNP-1753	MHAHG085-06	GU151271
175	Celaenorrhinus Burns01	Pyrginae	04-SRNP-1521	MHAHC187-05	DQ292055
176	Celaenorrhinus Burns01	Pyrginae	04-SRNP-26070	MHAHG090-06	GU151276
177	Celaenorrhinus Burns01	Pyrginae	05-SRNP-31094	MHAHG087-06	GU151274
178	Celaenorrhinus Burns01	Pyrginae	06-SRNP-31079	MHAHG642-06	GU151279
179	Celaenorrhinus Burns01	Pyrginae	00-SRNP-20678	CSCR059-04	DQ292046
180	Celaenorrhinus Burns01	Pyrginae	07-SRNP-1746	MHAHL555-07	JF761870
181	Celaenorrhinus Burns01	Pyrginae	07-SRNP-3335	MHMXO937-08	JF761878
182	Celaenorrhinus Burns01	Pyrginae	07-SRNP-66148	MHMXR893-08	JF761872
183	Celaenorrhinus Burns01	Pyrginae	08-SRNP-1552	MHMXX1048-09	JF777751
184	Celaenorrhinus Burns01	Pyrginae	08-SRNP-31104	MHMXX1050-09	JF777752
185	Celaenorrhinus Burns01	Pyrginae	08-SRNP-4324	MHMXX634-09	JF777754
186	Celaenorrhinus Burns01	Pyrginae	08-SRNP-1270	MHMXX1044-09	JF777749
187	Celaenorrhinus Burns01	Pyrginae	08-SRNP-32133	MHMXY1013-09	GU666518
188	Celaenorrhinus Burns01	Pyrginae	09-SRNP-56693	MHMYH187-10	HM887334
189	Celaenorrhinus Burns01	Pyrginae	05-SRNP-33241	MHAHK024-07	JF760521
190	Celaenorrhinus Burns01	Pyrginae	05-SRNP-6400	MHAHK026-07	JF760522
191	Celaenorrhinus Burns01	Pyrginae	06-SRNP-43399	MHAHI494-06	GU155884
192	Celaenorrhinus Burns01	Pyrginae	05-SRNP-5759	MHAHK021-07	JF760519
193	Celaenorrhinus Burns01	Pyrginae	05-SRNP-1850	MHAHG084-06	GU151272
194	Celaenorrhinus Burns01	Pyrginae	05-SRNP-1852	MHAHG082-06	GU151269
195	Celaenorrhinus Burns01	Pyrginae	04-SRNP-1512	MHAHC098-05	DQ292049
196	Celaenorrhinus Burns01	Pyrginae	04-SRNP-1743	MHAHC179-05	DQ292054
197	Celaenorrhinus Burns01	Pyrginae	04-SRNP-40721	MHAHC171-05	DQ292053
198	Celaenorrhinus Burns01	Pyrginae	04-SRNP-33215	MHAHC115-05	DQ292050
199	Celaenorrhinus Burns01	Pyrginae	04-SRNP-30415	MHAHC164-05	DQ292052
200	Celaenorrhinus Burns01	Pyrginae	04-SRNP-30592	MHAHC156-05	DQ292051
201	Celaenorrhinus Burns01	Pyrginae	05-SRNP-976	MHAHG080-06	GU151270
202	Celaenorrhinus Burns01	Pyrginae	05-SRNP-30880	MHAHG086-06	GU151275
203	Celaenorrhinus Burns01	Pyrginae	07-SRNP-3289	MHMXO940-08	JF761875
204	Celaenorrhinus Burns01	Pyrginae	02-SRNP-27941	CSCR060-04	DQ292047
205	Celaenorrhinus Burns01	Pyrginae	07-SRNP-2663	MHAHL553-07	JF761868
206	Celaenorrhinus Burns01	Pyrginae	07-SRNP-2535	MHAHL554-07	JF761869
207	Celaenorrhinus Burns01	Pyrginae	05-SRNP-5758	MHAHK022-07	JF760520
208	Celaenorrhinus Burns01	Pyrginae	07-SRNP-3397	MHMXO938-08	JF761877
209	Celaenorrhinus Burns01	Pyrginae	07-SRNP-3148	MHMXO939-08	JF761876
210	Celaenorrhinus Burns01	Pyrginae	07-SRNP-2970	MHMXO941-08	JF761874
211	Celaenorrhinus Burns01	Pyrginae	07-SRNP-3214	MHMXO942-08	JF761873
212	Celaenorrhinus Burns01	Pyrginae	07-SRNP-30343	MHMXK022-07	JF761879
213	Celaenorrhinus Burns01	Pyrginae	08-SRNP-2417	MHMXX1046-09	JF777750
214	Celaenorrhinus Burns01	Pyrginae	08-SRNP-1521	MHMXX1051-09	JF777753
215	Celaenorrhinus Burns01	Pyrginae	08-SRNP-32287	MHMXY1014-09	GU666511
216	Celaenorrhinus Burns01	Pyrginae	08-SRNP-72595	MHMXY1015-09	GU666512
217	Celaenorrhinus Burns01	Pyrginae	08-SRNP-72651	MHMXY1016-09	GU666513
218	Celaenorrhinus Burns01	Pyrginae	09-SRNP-65032	MHMYC457-09	GU649881
219	Celaenorrhinus Burns01	Pyrginae	09-SRNP-57269	MHMYE1492-09	GU653512
220	Celaenorrhinus Burns01	Pyrginae	09-SRNP-69994	MHMYH188-10	HM887335
221	Celaenorrhinus Burns01	Pyrginae	09-SRNP-68526	MHMYH189-10	HM887336
222	Polythrix mexicanus	Pyrginae	02-SRNP-13621	MHAHI408-06	GU156262
223	Polythrix mexicanus	Pyrginae	02-SRNP-13008	MHAHI409-06	GU156264

Tree Order	Species	Subfamily	ACG Sampleid	BOLD Processid	Genbank Accession
224	Polythrix mexicanus	Pyrginae	92-SRNP-5352	MHAHC474-05	DQ293081
225	Polythrix mexicanus	Pyrginae	93-SRNP-4855	MHAHI357-06	GU156260
226	Polythrix mexicanus	Pyrginae	93-SRNP-3545	MHAHC489-05	DQ293084
227	Polythrix mexicanus	Pyrginae	92-SRNP-5324	MHAHI367-06	GU156254
228	Polythrix mexicanus	Pyrginae	92-SRNP-5274	MHAHI365-06	GU156256
229	Polythrix mexicanus	Pyrginae	93-SRNP-4630	MHAHI362-06	GU156253
230	Polythrix mexicanus	Pyrginae	94-SRNP-5238	MHAHI358-06	GU156258
231	Polythrix mexicanus	Pyrginae	93-SRNP-4636	MHAHC475-05	DQ293082
232	Polythrix mexicanus	Pyrginae	01-SRNP-12021	CSRII149-04	DQ293079
233	Polythrix mexicanus	Pyrginae	92-SRNP-4357	MHAHI366-06	GU156257
234	Polythrix mexicanus	Pyrginae	01-SRNP-12023	CSRII150-04	DQ293080
235	Polythrix mexicanus	Pyrginae	92-SRNP-5323	MHAHC482-05	DQ293083
236	Polythrix mexicanus	Pyrginae	92-SRNP-4305	MHAHI368-06	GU156255
237	Polythrix mexicanus	Pyrginae	97-SRNP-3333	MHAHI379-06	GU156252
238	Polythrix mexicanus	Pyrginae	02-SRNP-32034	MHAHI387-06	GU156259
239	Polythrix mexicanus	Pyrginae	02-SRNP-10227	MHAHI389-06	GU156261
240	Polythrix mexicanus	Pyrginae	02-SRNP-13108	MHAHI391-06	GU156271
241	Polythrix mexicanus	Pyrginae	02-SRNP-13603	MHAHI394-06	GU156270
242	Polythrix mexicanus	Pyrginae	02-SRNP-13105	MHAHI395-06	GU156268
243	Polythrix mexicanus	Pyrginae	02-SRNP-13009	MHAHI400-06	GU156269
244	Polythrix mexicanus	Pyrginae	02-SRNP-13010	MHAHI401-06	GU156265
245	Polythrix mexicanus	Pyrginae	02-SRNP-13536	MHAHI402-06	GU156266
246	Polythrix mexicanus	Pyrginae	02-SRNP-32036	MHAHI403-06	GU156267
247	Polythrix mexicanus	Pyrginae	02-SRNP-13220	MHAHI410-06	GU156263
248	Polythrix mexicanus	Pyrginae	07-SRNP-12114	MHMXK271-07	JF762671
249	Polythrix mexicanus	Pyrginae	07-SRNP-14671	MHMXX712-09	JF778411
250	Polythrix asineDHJ04	Pyrginae	04-SRNP-48896	MHAHE165-05	GU149857
251	Polythrix asineDHJ04	Pyrginae	06-SRNP-43437	MHAHJ892-07	JF753081
252	Polythrix asineDHJ02	Pyrginae	09-SRNP-156	MHMYC524-09	
253	Polythrix asineDHJ01	Pyrginae	00-SRNP-6383	MHAHI441-06	GU156099
254	Polythrix asineDHJ01	Pyrginae	00-SRNP-6384	MHAHI433-06	GU156117
255	Polythrix asineDHJ01	Pyrginae	98-SRNP-4268	MHAHI356-06	GU156131
256	Polythrix asineDHJ01	Pyrginae	05-SRNP-24590	MHAHF742-06	GU150713
257	Polythrix asineDHJ01	Pyrginae	00-SRNP-6488	CSRII142-04	DQ293048
258	Polythrix asineDHJ01	Pyrginae	05-SRNP-59384	MHAHF294-06	GU150712
259	Polythrix asineDHJ01	Pyrginae	99-SRNP-18818	CSRII146-04	DQ293050
260	Polythrix asineDHJ01	Pyrginae	00-SRNP-7383	MHAHI1008-07	GU156125
261	Polythrix asineDHJ01	Pyrginae	07-SRNP-12113	MHMXK275-07	JF762656
262	Polythrix asineDHJ01	Pyrginae	06-SRNP-46863	MHAHJ677-07	JF753080
263	Polythrix asineDHJ01	Pyrginae	06-SRNP-46862	MHAHJ645-07	JF753079
264	Polythrix asineDHJ01	Pyrginae	00-SRNP-6220	MHAHI996-07	GU156116
265	Polythrix asineDHJ01	Pyrginae	97-SRNP-3760	MHAHI457-06	GU156110
266	Polythrix asineDHJ01	Pyrginae	99-SRNP-6116	MHAHI456-06	GU156109
267	Polythrix asineDHJ01	Pyrginae	00-SRNP-6415	MHAHI449-06	GU156140
268	Polythrix asineDHJ01	Pyrginae	00-SRNP-6824	MHAHI446-06	GU156119
269	Polythrix asineDHJ01	Pyrginae	00-SRNP-6382	MHAHI430-06	GU156121
270	Polythrix asineDHJ01	Pyrginae	03-SRNP-764	MHAHI423-06	GU156123
271	Polythrix asineDHJ01	Pyrginae	02-SRNP-10287	MHAHI415-06	GU156137
272	Polythrix asineDHJ01	Pyrginae	02-SRNP-10302	MHAHI414-06	GU156095
273	Polythrix asineDHJ01	Pyrginae	02-SRNP-10070	MHAHI406-06	GU156146
274	Polythrix asineDHJ01	Pyrginae	02-SRNP-17485	MHAHI390-06	GU156149
275	Polythrix asineDHJ01	Pyrginae	02-SRNP-32959	MHAHI386-06	GU156088
276	Polythrix asineDHJ01	Pyrginae	01-SRNP-12146	MHAHI382-06	GU156135
277	Polythrix asineDHJ01	Pyrginae	95-SRNP-9068	MHAHI372-06	GU156098
278	Polythrix asineDHJ01	Pyrginae	06-SRNP-12613	MHAHG659-06	GU151574
279	Polythrix asineDHJ01	Pyrginae	04-SRNP-13416	MHAHE170-05	GU149853

Tree Order	Species	Subfamily	ACG Sampleid	BOLD Processid	Genbank Accession
280	Polythrix asineDHJ01	Pyrginae	04-SRNP-16090	MHAHE167-05	GU149854
281	Polythrix asineDHJ01	Pyrginae	04-SRNP-14315	MHAHE166-05	GU149855
282	Polythrix asineDHJ01	Pyrginae	98-SRNP-4421	MHAHC486-05	DQ293071
283	Polythrix asineDHJ01	Pyrginae	04-SRNP-21571	MHAHC035-05	DQ293055
284	Polythrix asineDHJ01	Pyrginae	04-SRNP-21230	MHAHC020-05	DQ293053
285	Polythrix asineDHJ01	Pyrginae	04-SRNP-21464	MHAHC012-05	DQ293052
286	Polythrix asineDHJ01	Pyrginae	04-SRNP-21224	MHAHC004-05	DQ293051
287	Polythrix asineDHJ01	Pyrginae	04-SRNP-21232	MHAHC092-05	DQ293058
288	Polythrix asineDHJ01	Pyrginae	00-SRNP-6108	MHAHI995-07	GU156087
289	Polythrix asineDHJ01	Pyrginae	95-SRNP-818	MHAHI1031-07	GU156147
290	Polythrix asineDHJ01	Pyrginae	92-SRNP-4290	MHAHI361-06	GU156101
291	Polythrix asineDHJ01	Pyrginae	03-SRNP-765	MHAHI425-06	GU156118
292	Polythrix asineDHJ01	Pyrginae	06-SRNP-55628	MHAHG128-06	GU151575
293	Polythrix asineDHJ01	Pyrginae	96-SRNP-177	MHAHI1032-07	GU156124
294	Polythrix asineDHJ01	Pyrginae	00-SRNP-6015	MHAHI374-06	GU156097
295	Polythrix asineDHJ01	Pyrginae	95-SRNP-817	MHAHC481-05	DQ293068
296	Polythrix asineDHJ01	Pyrginae	98-SRNP-4202	MHAHI1052-07	GU156105
297	Polythrix asineDHJ01	Pyrginae	97-SRNP-3720	MHAHI381-06	GU156094
298	Polythrix asineDHJ01	Pyrginae	93-SRNP-3748	MHAHC473-05	DQ293063
299	Polythrix asineDHJ01	Pyrginae	93-SRNP-4295	MHAHI1038-07	GU156133
300	Polythrix asineDHJ01	Pyrginae	97-SRNP-3979	MHAHC470-05	DQ293060
301	Polythrix asineDHJ01	Pyrginae	96-SRNP-10668	MHAHC478-05	DQ293066
302	Polythrix asineDHJ01	Pyrginae	97-SRNP-4088	MHAHC469-05	DQ293059
303	Polythrix asineDHJ01	Pyrginae	00-SRNP-6491	MHAHI451-06	GU156112
304	Polythrix asineDHJ01	Pyrginae	91-SRNP-1631	MHAHI1020-07	GU156128
305	Polythrix asineDHJ01	Pyrginae	91-SRNP-784	MHAHI1022-07	GU156130
306	Polythrix asineDHJ01	Pyrginae	00-SRNP-6954	MHAHI1025-07	GU156134
307	Polythrix asineDHJ01	Pyrginae	99-SRNP-2390	MHAHI1026-07	GU156136
308	Polythrix asineDHJ01	Pyrginae	93-SRNP-124	MHAHI1029-07	GU156143
309	Polythrix asineDHJ01	Pyrginae	94-SRNP-567	MHAHI1030-07	GU156144
310	Polythrix asineDHJ01	Pyrginae	93-SRNP-4312	MHAHI1036-07	GU156141
311	Polythrix asineDHJ01	Pyrginae	93-SRNP-4696	MHAHI1039-07	GU156138
312	Polythrix asineDHJ01	Pyrginae	93-SRNP-3899	MHAHI1042-07	GU156142
313	Polythrix asineDHJ01	Pyrginae	93-SRNP-8053	MHAHI1047-07	GU156104
314	Polythrix asineDHJ01	Pyrginae	93-SRNP-4515	MHAHI1055-07	GU156107
315	Polythrix asineDHJ01	Pyrginae	07-SRNP-56773	MHAHL466-07	JF762654
316	Polythrix asineDHJ01	Pyrginae	08-SRNP-21481	MHMXW495-09	JF754090
317	Polythrix asineDHJ01	Pyrginae	07-SRNP-12456	MHMXX1176-09	JF778407
318	Polythrix asineDHJ01	Pyrginae	07-SRNP-12455	MHMXX1177-09	JF778408
319	Polythrix asineDHJ01	Pyrginae	02-SRNP-19815	MHAHI405-06	GU156145
320	Polythrix asineDHJ01	Pyrginae	92-SRNP-4286	MHAHI1001-07	GU156122
321	Polythrix asineDHJ01	Pyrginae	05-SRNP-34180	MHAHF745-06	GU150710
322	Polythrix asineDHJ01	Pyrginae	04-SRNP-14104	MHAHE171-05	GU149852
323	Polythrix asineDHJ01	Pyrginae	06-SRNP-12714	MHAHH562-06	GU155458
324	Polythrix asineDHJ01	Pyrginae	96-SRNP-8726	MHAHC472-05	DQ293062
325	Polythrix asineDHJ01	Pyrginae	00-SRNP-6183	MHAHI1024-07	GU156132
326	Polythrix asineDHJ01	Pyrginae	00-SRNP-6737	MHAHI999-07	GU156103
327	Polythrix asineDHJ01	Pyrginae	01-SRNP-12057	MHAHI994-07	GU156086
328	Polythrix asineDHJ01	Pyrginae	01-SRNP-12044	MHAHI998-07	GU156102
329	Polythrix asineDHJ01	Pyrginae	05-SRNP-5142	MHAHF293-06	GU150711
330	Polythrix asineDHJ01	Pyrginae	96-SRNP-10264	MHAHC488-05	DQ293072
331	Polythrix asineDHJ01	Pyrginae	01-SRNP-12145	MHAHI383-06	GU156092
332	Polythrix asineDHJ01	Pyrginae	96-SRNP-8830	MHAHI1053-07	GU156106
333	Polythrix asineDHJ01	Pyrginae	93-SRNP-4232	MHAHI1056-07	GU156108
334	Polythrix asineDHJ01	Pyrginae	08-SRNP-2029	MHMXW476-09	JF754088
335	Polythrix asineDHJ01	Pyrginae	94-SRNP-7830	MHAHI351-06	GU156127

Tree Order	Species	Subfamily	ACG Sampleid	BOLD Processid	Genbank Accession
336	Polythrix asineDHJ01	Pyrginae	95-SRNP-9644	MHAHI354-06	GU156129
337	Polythrix asineDHJ01	Pyrginae	93-SRNP-4695	MHAHI364-06	GU156100
338	Polythrix asineDHJ01	Pyrginae	93-SRNP-3926	MHAHI370-06	GU156089
339	Polythrix asineDHJ01	Pyrginae	00-SRNP-6613	MHAHI377-06	GU156096
340	Polythrix asineDHJ01	Pyrginae	00-SRNP-6394	MHAHI378-06	GU156090
341	Polythrix asineDHJ01	Pyrginae	01-SRNP-12359	MHAHI384-06	GU156093
342	Polythrix asineDHJ01	Pyrginae	02-SRNP-10304	MHAHI399-06	GU156148
343	Polythrix asineDHJ01	Pyrginae	03-SRNP-29556	MHAHI424-06	GU156139
344	Polythrix asineDHJ01	Pyrginae	03-SRNP-777	MHAHI429-06	GU156120
345	Polythrix asineDHJ01	Pyrginae	00-SRNP-6487	MHAHI431-06	GU156085
346	Polythrix asineDHJ01	Pyrginae	00-SRNP-6367	MHAHI439-06	GU156115
347	Polythrix asineDHJ01	Pyrginae	00-SRNP-6497	MHAHI440-06	GU156091
348	Polythrix asineDHJ01	Pyrginae	00-SRNP-6494	MHAHI444-06	GU156113
349	Polythrix asineDHJ01	Pyrginae	00-SRNP-6395	MHAHI445-06	GU156114
350	Polythrix asineDHJ01	Pyrginae	01-SRNP-18701	MHAHI1005-07	GU156111
351	Polythrix asineDHJ01	Pyrginae	00-SRNP-6700	MHAHI1009-07	GU156126
352	Polythrix asineDHJ01	Pyrginae	07-SRNP-20846	MHMXK269-07	JF762657
353	Polythrix asineDHJ01	Pyrginae	07-SRNP-57195	MHMXO822-08	JF762655
354	Polythrix asineDHJ01	Pyrginae	08-SRNP-56049	MHMXW491-09	JF754089
355	Polythrix asineDHJ01	Pyrginae	08-SRNP-24005	MHMYB134-09	GU649691
356	Polythrix asineDHJ02	Pyrginae	00-SRNP-6701	MHAHI437-06	GU156158
357	Polythrix asineDHJ02	Pyrginae	01-SRNP-17366	MHAHI413-06	GU156190
358	Polythrix asineDHJ02	Pyrginae	00-SRNP-6215	MHAHI434-06	GU156183
359	Polythrix asineDHJ02	Pyrginae	02-SRNP-13219	MHAHI388-06	GU156180
360	Polythrix asineDHJ02	Pyrginae	02-SRNP-10303	MHAHI404-06	GU156225
361	Polythrix asineDHJ02	Pyrginae	98-SRNP-4205	MHAHI353-06	GU156203
362	Polythrix asineDHJ02	Pyrginae	93-SRNP-3543	MHAHI1014-07	GU156199
363	Polythrix asineDHJ02	Pyrginae	94-SRNP-5275	MHAHI363-06	GU156157
364	Polythrix asineDHJ02	Pyrginae	92-SRNP-204	MHAHI1034-07	GU156216
365	Polythrix asineDHJ02	Pyrginae	96-SRNP-957	MHAHI1035-07	GU156218
366	Polythrix asineDHJ02	Pyrginae	08-SRNP-2064	MHMXW493-09	JF754092
367	Polythrix asineDHJ02	Pyrginae	00-SRNP-6572	MHAHI460-06	GU156170
368	Polythrix asineDHJ02	Pyrginae	00-SRNP-6495	MHAHI1000-07	GU156205
369	Polythrix asineDHJ02	Pyrginae	07-SRNP-55549	MHMXK276-07	JF762658
370	Polythrix asineDHJ02	Pyrginae	99-SRNP-6082	MHAHI459-06	GU156172
371	Polythrix asineDHJ02	Pyrginae	99-SRNP-6074	MHAHI453-06	GU156175
372	Polythrix asineDHJ02	Pyrginae	00-SRNP-6949	MHAHI452-06	GU156234
373	Polythrix asineDHJ02	Pyrginae	00-SRNP-7137	MHAHI447-06	GU156223
374	Polythrix asineDHJ02	Pyrginae	03-SRNP-18566	MHAHI427-06	GU156186
375	Polythrix asineDHJ02	Pyrginae	03-SRNP-14435	MHAHI420-06	GU156230
376	Polythrix asineDHJ02	Pyrginae	02-SRNP-32223	MHAHI385-06	GU156151
377	Polythrix asineDHJ02	Pyrginae	00-SRNP-3211	MHAHI376-06	GU156155
378	Polythrix asineDHJ02	Pyrginae	94-SRNP-710	MHAHI359-06	GU156159
379	Polythrix asineDHJ02	Pyrginae	02-SRNP-10034	MHAHI412-06	GU156221
380	Polythrix asineDHJ02	Pyrginae	97-SRNP-3363	MHAHI380-06	GU156153
381	Polythrix asineDHJ02	Pyrginae	00-SRNP-6388	MHAHI997-07	GU156152
382	Polythrix asineDHJ02	Pyrginae	00-SRNP-6950	MHAHI1003-07	GU156189
383	Polythrix asineDHJ02	Pyrginae	05-SRNP-61346	MHAHF740-06	GU150714
384	Polythrix asineDHJ02	Pyrginae	94-SRNP-711	MHAHC485-05	DQ293070
385	Polythrix asineDHJ02	Pyrginae	05-SRNP-59292	MHAHF296-06	GU150715
386	Polythrix asineDHJ02	Pyrginae	93-SRNP-3930	MHAHI1044-07	GU156222
387	Polythrix asineDHJ02	Pyrginae	93-SRNP-3398	MHAHI1016-07	GU156200
388	Polythrix asineDHJ02	Pyrginae	92-SRNP-5429	MHAHC483-05	DQ293069
389	Polythrix asineDHJ02	Pyrginae	91-SRNP-167	MHAHI1021-07	GU156204
390	Polythrix asineDHJ02	Pyrginae	92-SRNP-4524	MHAHI1048-07	GU156166
391	Polythrix asineDHJ02	Pyrginae	94-SRNP-699	MHAHI1050-07	GU156167

Tree Order	Species	Subfamily	ACG Sampleid	BOLD Processid	Genbank Accession
392	Polythrix asineDHJ02	Pyrginae	08-SRNP-56062	MHMXX627-09	JF778409
393	Polythrix asineDHJ02	Pyrginae	08-SRNP-14903	MHMXY950-09	GU666574
394	Polythrix asineDHJ02	Pyrginae	02-SRNP-10068	MHAHI398-06	GU156229
395	Polythrix asineDHJ02	Pyrginae	01-SRNP-12056	MHAHI1004-07	GU156226
396	Polythrix asineDHJ02	Pyrginae	04-SRNP-21235	MHAHC084-05	DQ293057
397	Polythrix asineDHJ02	Pyrginae	93-SRNP-4231	MHAHI1046-07	GU156227
398	Polythrix asineDHJ02	Pyrginae	07-SRNP-55640	MHMXK272-07	JF762661
399	Polythrix asineDHJ02	Pyrginae	93-SRNP-4239	MHAHI1040-07	GU156214
400	Polythrix asineDHJ02	Pyrginae	99-SRNP-6119	MHAHI455-06	GU156171
401	Polythrix asineDHJ02	Pyrginae	00-SRNP-7038	MHAHI442-06	GU156178
402	Polythrix asineDHJ02	Pyrginae	93-SRNP-61	MHAHI369-06	GU156209
403	Polythrix asineDHJ02	Pyrginae	92-SRNP-5030	MHAHI1041-07	GU156215
404	Polythrix asineDHJ02	Pyrginae	93-SRNP-3635	MHAHI1045-07	GU156224
405	Polythrix asineDHJ02	Pyrginae	97-SRNP-3311	MHAHI1051-07	GU156168
406	Polythrix asineDHJ02	Pyrginae	08-SRNP-21270	MHMXW492-09	JF754091
407	Polythrix asineDHJ02	Pyrginae	90-SRNP-2362	MHAHI1023-07	GU156210
408	Polythrix asineDHJ02	Pyrginae	03-SRNP-151	MHAHI421-06	GU156162
409	Polythrix asineDHJ02	Pyrginae	01-SRNP-17146	MHAHI407-06	GU156217
410	Polythrix asineDHJ02	Pyrginae	00-SRNP-7129	MHAHI435-06	GU156182
411	Polythrix asineDHJ02	Pyrginae	00-SRNP-2525	MHAHI1006-07	GU156188
412	Polythrix asineDHJ02	Pyrginae	97-SRNP-5334	MHAHI360-06	GU156160
413	Polythrix asineDHJ02	Pyrginae	91-SRNP-2264	MHAHI1002-07	GU156181
414	Polythrix asineDHJ02	Pyrginae	96-SRNP-10685	MHAHI1018-07	GU156196
415	Polythrix asineDHJ02	Pyrginae	07-SRNP-56772	MHMXK270-07	JF762662
416	Polythrix asineDHJ02	Pyrginae	00-SRNP-20213	MHAHI1007-07	GU156207
417	Polythrix asineDHJ02	Pyrginae	03-SRNP-949	MHAHI418-06	GU156208
418	Polythrix asineDHJ02	Pyrginae	01-SRNP-18761	MHAHI393-06	GU156233
419	Polythrix asineDHJ02	Pyrginae	03-SRNP-25832	MHAHI417-06	GU156197
420	Polythrix asineDHJ02	Pyrginae	99-SRNP-6164	MHAHI454-06	GU156176
421	Polythrix asineDHJ02	Pyrginae	01-SRNP-12169	CSRII143-04	DQ293049
422	Polythrix asineDHJ02	Pyrginae	00-SRNP-7100	MHAHI458-06	GU156173
423	Polythrix asineDHJ02	Pyrginae	00-SRNP-2164	MHAHI450-06	GU156174
424	Polythrix asineDHJ02	Pyrginae	97-SRNP-10070	MHAHC471-05	DQ293061
425	Polythrix asineDHJ02	Pyrginae	07-SRNP-55609	MHMXK273-07	JF762660
426	Polythrix asineDHJ02	Pyrginae	06-SRNP-47913	MHMXH839-07	JF761058
427	Polythrix asineDHJ02	Pyrginae	00-SRNP-2609	MHAHI448-06	GU156177
428	Polythrix asineDHJ02	Pyrginae	00-SRNP-17652	MHAHI443-06	GU156232
429	Polythrix asineDHJ02	Pyrginae	00-SRNP-6951	MHAHI438-06	GU156179
430	Polythrix asineDHJ02	Pyrginae	00-SRNP-6013	MHAHI436-06	GU156161
431	Polythrix asineDHJ02	Pyrginae	00-SRNP-6014	MHAHI432-06	GU156150
432	Polythrix asineDHJ02	Pyrginae	03-SRNP-678	MHAHI428-06	GU156185
433	Polythrix asineDHJ02	Pyrginae	03-SRNP-14568	MHAHI426-06	GU156184
434	Polythrix asineDHJ02	Pyrginae	03-SRNP-155	MHAHI422-06	GU156163
435	Polythrix asineDHJ02	Pyrginae	02-SRNP-32221	MHAHI416-06	GU156212
436	Polythrix asineDHJ02	Pyrginae	02-SRNP-5741	MHAHI411-06	GU156219
437	Polythrix asineDHJ02	Pyrginae	01-SRNP-16459	MHAHI397-06	GU156231
438	Polythrix asineDHJ02	Pyrginae	02-SRNP-13312	MHAHI396-06	GU156228
439	Polythrix asineDHJ02	Pyrginae	06-SRNP-21342	MHAHH561-06	GU155459
440	Polythrix asineDHJ02	Pyrginae	06-SRNP-12614	MHAHG720-06	GU151577
441	Polythrix asineDHJ02	Pyrginae	05-SRNP-64404	MHAHG660-06	GU151576
442	Polythrix asineDHJ02	Pyrginae	04-SRNP-14469	MHAHE169-05	GU149856
443	Polythrix asineDHJ02	Pyrginae	04-SRNP-45139	MHAHC028-05	DQ293054
444	Polythrix asineDHJ02	Pyrginae	04-SRNP-2256	MHAHC076-05	DQ293056
445	Polythrix asineDHJ02	Pyrginae	00-SRNP-6483	MHAHI375-06	GU156154
446	Polythrix asineDHJ02	Pyrginae	05-SRNP-21368	MHAHF297-06	GU150717
447	Polythrix asineDHJ02	Pyrginae	93-SRNP-4178	MHAHI1015-07	GU156198

Tree Order	Species	Subfamily	ACG Sampleid	BOLD Processid	Genbank Accession
448	Polythrix asineDHJ02	Pyrginae	92-SRNP-5058	MHAHI352-06	GU156202
449	Polythrix asineDHJ02	Pyrginae	05-SRNP-4485	MHAHF295-06	GU150716
450	Polythrix asineDHJ02	Pyrginae	93-SRNP-4516	MHAHC480-05	DQ293067
451	Polythrix asineDHJ02	Pyrginae	93-SRNP-4921	MHAHI371-06	GU156156
452	Polythrix asineDHJ02	Pyrginae	06-SRNP-46210	MHAHI130-06	GU156164
453	Polythrix asineDHJ02	Pyrginae	94-SRNP-6175	MHAHI1037-07	GU156195
454	Polythrix asineDHJ02	Pyrginae	01-SRNP-12150	MHAHI1010-07	GU156191
455	Polythrix asineDHJ02	Pyrginae	94-SRNP-7825	MHAHI373-06	GU156211
456	Polythrix asineDHJ02	Pyrginae	90-SRNP-1709	MHAHC477-05	DQ293065
457	Polythrix asineDHJ02	Pyrginae	92-SRNP-4667	MHAHC476-05	DQ293064
458	Polythrix asineDHJ02	Pyrginae	92-SRNP-4160	MHAHI1049-07	GU156165
459	Polythrix asineDHJ02	Pyrginae	99-SRNP-6176	MHAHI1011-07	GU156192
460	Polythrix asineDHJ02	Pyrginae	03-SRNP-1811	MHAHI419-06	GU156187
461	Polythrix asineDHJ02	Pyrginae	93-SRNP-4316	MHAHI1017-07	GU156201
462	Polythrix asineDHJ02	Pyrginae	97-SRNP-5248	MHAHI1013-07	GU156194
463	Polythrix asineDHJ02	Pyrginae	98-SRNP-4204	MHAHI1033-07	GU156213
464	Polythrix asineDHJ02	Pyrginae	91-SRNP-2273	MHAHI355-06	GU156206
465	Polythrix asineDHJ02	Pyrginae	92-SRNP-4981	MHAHI1019-07	GU156193
466	Polythrix asineDHJ02	Pyrginae	93-SRNP-22	MHAHI1043-07	GU156220
467	Polythrix asineDHJ02	Pyrginae	98-SRNP-4007	MHAHI1054-07	GU156169
468	Polythrix asineDHJ02	Pyrginae	07-SRNP-12116	MHMXK274-07	JF762659
469	Polythrix asineDHJ02	Pyrginae	08-SRNP-21704	MHMXW494-09	JF754093
470	Polythrix asineDHJ02	Pyrginae	08-SRNP-4981	MHMXX628-09	JF778410
471	Polythrix asineDHJ02	Pyrginae	09-SRNP-14247	MHMYE869-09	GU653732
472	Polythrix caunus	Pyrginae	06-SRNP-48047	MHMXH838-07	JF761059
473	Polythrix caunus	Pyrginae	05-SRNP-41335	MHAHF354-06	GU150721
474	Polythrix caunus	Pyrginae	07-SRNP-45093	MHMXK330-07	JF762667
475	Polythrix caunus	Pyrginae	07-SRNP-45094	MHMXK329-07	JF762668
476	Polythrix caunus	Pyrginae	06-SRNP-46961	MHAHJ644-07	JF753082
477	Polythrix caunus	Pyrginae	97-SRNP-411	MHAHI265-06	GU156242
478	Polythrix caunus	Pyrginae	00-SRNP-3976	MHAHI260-06	GU156240
479	Polythrix caunus	Pyrginae	01-SRNP-9329	MHAHI257-06	GU156251
480	Polythrix caunus	Pyrginae	01-SRNP-11831	MHAHI256-06	GU156250
481	Polythrix caunus	Pyrginae	01-SRNP-9037	MHAHI255-06	GU156249
482	Polythrix caunus	Pyrginae	02-SRNP-6762	MHAHI253-06	GU156244
483	Polythrix caunus	Pyrginae	04-SRNP-45147	MHAHI248-06	GU156245
484	Polythrix caunus	Pyrginae	05-SRNP-61208	MHAHF741-06	GU150723
485	Polythrix caunus	Pyrginae	04-SRNP-49813	MHAHE238-05	GU149862
486	Polythrix caunus	Pyrginae	04-SRNP-47482	MHAHE237-05	GU149863
487	Polythrix caunus	Pyrginae	04-SRNP-13678	MHAHE236-05	GU149858
488	Polythrix caunus	Pyrginae	04-SRNP-56145	MHAHE235-05	GU149859
489	Polythrix caunus	Pyrginae	04-SRNP-49793	MHAHE234-05	GU149860
490	Polythrix caunus	Pyrginae	04-SRNP-48326	MHAHE233-05	GU149861
491	Polythrix caunus	Pyrginae	06-SRNP-47938	MHMXH841-07	JF761060
492	Polythrix caunus	Pyrginae	05-SRNP-40491	MHAHF353-06	GU150722
493	Polythrix caunus	Pyrginae	03-SRNP-1670	CSCR396-04	DQ293075
494	Polythrix caunus	Pyrginae	97-SRNP-5432	MHAHI263-06	GU156248
495	Polythrix caunus	Pyrginae	00-SRNP-18463	MHAHI259-06	GU156239
496	Polythrix caunus	Pyrginae	01-SRNP-9142	MHAHI250-06	GU156246
497	Polythrix caunus	Pyrginae	01-SRNP-9036	MHAHI249-06	GU156243
498	Polythrix caunus	Pyrginae	03-SRNP-1437	CSCR395-04	DQ293074
499	Polythrix caunus	Pyrginae	02-SRNP-4214	MHAHI252-06	GU156247
500	Polythrix caunus	Pyrginae	96-SRNP-230	MHAHI264-06	GU156241
501	Polythrix caunus	Pyrginae	07-SRNP-46448	MHMXT183-08	JF762666
502	Polythrix caunus	Pyrginae	07-SRNP-47110	MHMXT184-08	JF762665
503	Polythrix caunus	Pyrginae	08-SRNP-31055	MHMXW485-09	JF754095

Tree Order	Species	Subfamily	ACG Sampleid	BOLD Processid	Genbank Accession
504	Polythrix caunus	Pyrginae	08-SRNP-2598	MHMXW486-09	JF754096
505	Polythrix caunus	Pyrginae	08-SRNP-2600	MHMXW487-09	JF754097
506	Polythrix caunus	Pyrginae	08-SRNP-2433	MHMXW488-09	JF754098
507	Polythrix caunus	Pyrginae	08-SRNP-2599	MHMXW490-09	JF754099
508	Chrysoplectrum Burns02	Pyrginae	03-SRNP-20727	CSCR347-04	DQ292147
509	Polythrix octomaculata	Pyrginae	01-SRNP-18777	CSRII153-04	DQ293086
510	Polythrix octomaculata	Pyrginae	04-SRNP-16187	MHAHE149-05	GU149866
511	Polythrix octomaculata	Pyrginae	06-SRNP-55510	MHAHG132-06	GU151578
512	Polythrix octomaculata	Pyrginae	06-SRNP-58092	MHAHJ718-07	JF753085
513	Polythrix octomaculata	Pyrginae	08-SRNP-2053	MHMXW475-09	JF754100
514	Polythrix octomaculata	Pyrginae	01-SRNP-12143	CSRII152-04	DQ293085
515	Polythrix octomaculata	Pyrginae	06-SRNP-55601	MHAHG185-06	GU151582
516	Polythrix octomaculata	Pyrginae	04-SRNP-16188	MHAHE150-05	GU149865
517	Polythrix octomaculata	Pyrginae	06-SRNP-55352	MHAHG186-06	GU151581
518	Polythrix octomaculata	Pyrginae	06-SRNP-55424	MHAHG187-06	GU151580
519	Polythrix octomaculata	Pyrginae	06-SRNP-55112	MHAHG133-06	GU151579
520	Polythrix octomaculata	Pyrginae	09-SRNP-55230	MHMYE888-09	GU653715
521	Polythrix auginus	Pyrginae	08-SRNP-5805	MHMXY951-09	HM390686
522	Polythrix auginus	Pyrginae	99-SRNP-2513	MHAHI262-06	GU156236
523	Polythrix auginus	Pyrginae	01-SRNP-4476	MHAHI247-06	GU156237
524	Polythrix auginus	Pyrginae	02-SRNP-5395	MHAHI251-06	GU156238
525	Polythrix auginus	Pyrginae	05-SRNP-40942	MHAHF358-06	GU150720
526	Polythrix auginus	Pyrginae	05-SRNP-40943	MHAHF357-06	GU150719
527	Polythrix auginus	Pyrginae	07-SRNP-23809	MHMXT185-08	JF762663
528	Polythrix auginus	Pyrginae	07-SRNP-23808	MHMXR920-08	JF762664
529	Polythrix auginus	Pyrginae	02-SRNP-7476	MHAHI261-06	GU156235
530	Polythrix auginus	Pyrginae	00-SRNP-21098	CSRII144-04	DQ293073
531	Polythrix auginus	Pyrginae	08-SRNP-2432	MHMXW489-09	JF754094
532	Polythrix auginus	Pyrginae	08-SRNP-32652	MHMXY952-09	
533	Polythrix auginus	Pyrginae	09-SRNP-71706	MHMYE1461-09	HQ992248
534	Chrysoplectrum pervivax	Pyrginae	08-SRNP-55673	MHMXW549-09	JF753821
535	Chrysoplectrum pervivax	Pyrginae	07-SRNP-55670	MHMXK057-07	JF761949
536	Chrysoplectrum pervivax	Pyrginae	06-SRNP-55724	MHAHH574-06	GU155331
537	Chrysoplectrum pervivax	Pyrginae	06-SRNP-55725	MHAHH573-06	GU155332
538	Chrysoplectrum pervivax	Pyrginae	05-SRNP-55019	MHAHC812-05	DQ292153
539	Chrysoplectrum pervivax	Pyrginae	05-SRNP-55012	MHAHC787-05	DQ292152
540	Chrysoplectrum pervivax	Pyrginae	03-SRNP-599	CSCR349-04	DQ292149
541	Chrysoplectrum pervivax	Pyrginae	03-SRNP-27771	CSCR500-04	DQ292151
542	Chrysoplectrum pervivax	Pyrginae	05-SRNP-12099	MHAHF251-06	GU150335
543	Chrysoplectrum pervivax	Pyrginae	06-SRNP-55633	MHAHG135-06	GU151304
544	Chrysoplectrum pervivax	Pyrginae	03-SRNP-285	CSCR348-04	DQ292148
545	Chrysoplectrum pervivax	Pyrginae	03-SRNP-27214	CSCR499-04	DQ292150
546	Chrysoplectrum pervivax	Pyrginae	06-SRNP-55678	MHAHH572-06	GU155333
547	Chrysoplectrum pervivax	Pyrginae	08-SRNP-55510	MHMXW550-09	JF753822
548	Chrysoplectrum pervivax	Pyrginae	08-SRNP-55708	MHMXW551-09	JF753823
549	Chrysoplectrum pervivax	Pyrginae	08-SRNP-55674	MHMXW553-09	JF753824
550	Chrysoplectrum Burns01	Pyrginae	05-SRNP-20359	MHAHC810-05	DQ292143
551	Chrysoplectrum Burns01	Pyrginae	03-SRNP-21246	CSCR496-04	DQ292139
552	Chrysoplectrum Burns01	Pyrginae	08-SRNP-41220	MHMXX487-09	GU666364
553	Chrysoplectrum Burns01	Pyrginae	08-SRNP-71264	MHMXY1097-09	GU666445
554	Chrysoplectrum Burns01	Pyrginae	08-SRNP-71263	MHMXY1113-09	GU666428
555	Chrysoplectrum Burns01	Pyrginae	07-SRNP-65253	MHAHL563-07	JF761941
556	Chrysoplectrum Burns01	Pyrginae	03-SRNP-5985	CSCR346-04	DQ292138
557	Chrysoplectrum Burns01	Pyrginae	07-SRNP-65263	MHMXP169-08	JF761945
558	Chrysoplectrum Burns01	Pyrginae	04-SRNP-33618	MHAHC814-05	DQ292146
559	Chrysoplectrum Burns01	Pyrginae	04-SRNP-41884	MHAHC813-05	DQ292145

Tree Order	Species	Subfamily	ACG Sampleid	BOLD Processid	Genbank Accession
560	Chrysoplectrum Burns01	Pyrginae	04-SRNP-60797	MHAHC811-05	DQ292144
561	Chrysoplectrum Burns01	Pyrginae	04-SRNP-60793	MHAHC809-05	DQ292142
562	Chrysoplectrum Burns01	Pyrginae	04-SRNP-60305	MHAHC808-05	DQ292141
563	Chrysoplectrum Burns01	Pyrginae	03-SRNP-10713	CSCR345-04	DQ292137
564	Chrysoplectrum Burns01	Pyrginae	03-SRNP-30510	CSCR498-04	DQ292140
565	Chrysoplectrum Burns01	Pyrginae	07-SRNP-41409	MHMXN287-07	JF761948
566	Chrysoplectrum Burns01	Pyrginae	07-SRNP-21008	MHMXN290-07	JF761947
567	Chrysoplectrum Burns01	Pyrginae	07-SRNP-65264	MHMXP168-08	JF761946
568	Chrysoplectrum Burns01	Pyrginae	07-SRNP-65771	MHMXR736-08	JF761944
569	Chrysoplectrum Burns01	Pyrginae	07-SRNP-65755	MHMXR737-08	JF761943
570	Chrysoplectrum Burns01	Pyrginae	07-SRNP-65394	MHMXR738-08	JF761942
571	Chrysoplectrum Burns01	Pyrginae	08-SRNP-71635	MHMXW552-09	JF753820
572	Chrysoplectrum Burns01	Pyrginae	08-SRNP-71122	MHMXX488-09	JF777767
573	Chrysoplectrum Burns01	Pyrginae	08-SRNP-71266	MHMXX489-09	JF777768
574	Chrysoplectrum Burns01	Pyrginae	08-SRNP-71123	MHMXY1099-09	GU666439
575	Chrysoplectrum Burns01	Pyrginae	09-SRNP-69180	MHMYE1523-09	HM391083
576	Chrysoplectrum Burns01	Pyrginae	09-SRNP-22283	MHMYG2406-10	HM885828
577	Chrysoplectrum Burns01	Pyrginae	09-SRNP-41395	MHMYG2408-10	HM885830
578	Paches loxus	Pyrginae	02-SRNP-5725	CSCR172-04	DQ292821
579	Paches loxus	Pyrginae	02-SRNP-5985	CSCR173-04	DQ292822
580	Paches loxus	Pyrginae	03-SRNP-30995	MHAHJ284-07	JF752951
581	Paches loxus	Pyrginae	06-SRNP-55156	MHAHG219-06	GU151512
582	Paches loxus	Pyrginae	02-SRNP-5727	MHAHJ282-07	JF752949
583	Paches loxus	Pyrginae	02-SRNP-5726	MHAHJ283-07	JF752950
584	Paches loxus	Pyrginae	03-SRNP-30840	MHAHJ285-07	JF752952
585	Paches loxus	Pyrginae	03-SRNP-30960	MHAHJ286-07	JF752953
586	Paches loxus	Pyrginae	02-SRNP-5742	MHAHJ288-07	JF752955
587	Paches loxus	Pyrginae	02-SRNP-5517	MHAHJ289-07	JF752956
588	Paches loxus	Pyrginae	02-SRNP-5724	MHAHJ290-07	JF752957
589	Paches loxus	Pyrginae	07-SRNP-60833	MHMXR775-08	JF762503
590	Paches loxus	Pyrginae	07-SRNP-60825	MHMXR776-08	JF762502
591	Paches loxus	Pyrginae	04-SRNP-15261	MHAHF075-06	GU150646
592	Paches loxus	Pyrginae	02-SRNP-14194	MHAHJ287-07	JF752954
593	Paches loxus	Pyrginae	02-SRNP-5743	MHAHJ291-07	JF752958
594	Paches loxus	Pyrginae	07-SRNP-60964	MHMXR777-08	JF762501
595	Phanus marshalliiDHJ01	Pyrginae	03-SRNP-10532	MHAHJ225-07	JF752983
596	Phanus marshalliiDHJ01	Pyrginae	03-SRNP-5739	MHAHJ222-07	JF752981
597	Phanus marshalliiDHJ01	Pyrginae	03-SRNP-5850	MHAHJ226-07	JF752984
598	Phanus marshalliiDHJ01	Pyrginae	03-SRNP-16237	MHAHJ224-07	JF752982
599	Phanus marshalliiDHJ01	Pyrginae	03-SRNP-5857	MHAHJ227-07	JF752985
600	Phanus marshalliiDHJ02	Pyrginae	08-SRNP-2311	MHMXW353-09	JF754044
601	Phanus marshalliiDHJ02	Pyrginae	08-SRNP-21884	MHMXW345-09	JF754043
602	Phanus marshalliiDHJ02	Pyrginae	07-SRNP-21440	MHAHL560-07	JF762578
603	Phanus marshalliiDHJ02	Pyrginae	02-SRNP-14543	MHAHJ221-07	JF752987
604	Phanus marshalliiDHJ02	Pyrginae	02-SRNP-2186	MHAHJ220-07	JF752986
605	Phanus marshalliiDHJ02	Pyrginae	03-SRNP-16236	MHAHJ223-07	JF752988
606	Phanus marshalliiDHJ02	Pyrginae	03-SRNP-16234	MHAHJ228-07	JF752989
607	Phanus marshalliiDHJ02	Pyrginae	05-SRNP-2028	MHAHF547-06	GU150678
608	Phanus marshalliiDHJ02	Pyrginae	02-SRNP-2185	CSCR185-04	DQ292962
609	Phanus marshalliiDHJ02	Pyrginae	01-SRNP-14963	CSCR184-04	DQ292961
610	Phanus marshalliiDHJ02	Pyrginae	08-SRNP-21663	MHMXW360-09	JF754046
611	Phanus obscurior	Pyrginae	02-SRNP-1685	MHAHJ219-07	JF752997
612	Phanus obscurior	Pyrginae	08-SRNP-1691	MHMXW351-09	JF754049
613	Phanus obscurior	Pyrginae	08-SRNP-1692	MHMXW357-09	JF754050
614	Phanus obscurior	Pyrginae	03-SRNP-6083	MHAHJ212-07	JF752990
615	Phanus obscurior	Pyrginae	04-SRNP-60040	MHAHC820-05	DQ292965

Tree Order	Species	Subfamily	ACG Sampleid	BOLD Processid	Genbank Accession
616	Phanus obscurior	Pyrginae	06-SRNP-22131	MHAHJ766-07	JF753000
617	Phanus obscurior	Pyrginae	06-SRNP-22126	MHAHJ596-07	JF752999
618	Phanus obscurior	Pyrginae	06-SRNP-22146	MHAHJ565-07	JF752998
619	Phanus obscurior	Pyrginae	02-SRNP-3622	MHAHJ218-07	JF752996
620	Phanus obscurior	Pyrginae	02-SRNP-3270	MHAHJ217-07	JF752995
621	Phanus obscurior	Pyrginae	02-SRNP-17173	MHAHJ216-07	JF752994
622	Phanus obscurior	Pyrginae	03-SRNP-5866	MHAHJ214-07	JF752992
623	Phanus obscurior	Pyrginae	05-SRNP-1856	MHAHF550-06	GU150679
624	Phanus obscurior	Pyrginae	03-SRNP-5851	MHAHJ213-07	JF752991
625	Phanus obscurior	Pyrginae	06-SRNP-22124	MHAHJ769-07	JF753003
626	Phanus obscurior	Pyrginae	06-SRNP-22129	MHAHJ873-07	JF753004
627	Phanus obscurior	Pyrginae	08-SRNP-1581	MHMXW347-09	JF754048
628	Phanus obscurior	Pyrginae	08-SRNP-1580	MHMXW358-09	JF754051
629	Phanus obscurior	Pyrginae	08-SRNP-4668	MHMXX572-09	JF778382
630	Phanus obscurior	Pyrginae	08-SRNP-1112	MHMXW341-09	JF754047
631	Phanus obscurior	Pyrginae	00-SRNP-870	CSCR186-04	DQ292963
632	Phanus obscurior	Pyrginae	02-SRNP-2466	CSCR187-04	DQ292964
633	Phanus obscurior	Pyrginae	06-SRNP-22134	MHAHJ767-07	JF753001
634	Phanus obscurior	Pyrginae	07-SRNP-2619	MHAHL561-07	JF762579
635	Phanus obscurior	Pyrginae	06-SRNP-22127	MHAHJ768-07	JF753002
636	Phanus obscurior	Pyrginae	06-SRNP-3813	MHAHI160-06	GU156076
637	Phanus obscurior	Pyrginae	06-SRNP-3624	MHAHH430-06	GU155451
638	Phanus obscurior	Pyrginae	04-SRNP-60913	MHAHC821-05	DQ292966
639	Phanus obscurior	Pyrginae	06-SRNP-34130	MHAHI514-06	GU156075
640	Phanus obscurior	Pyrginae	02-SRNP-2470	MHAHJ215-07	JF752993
641	Phanus obscurior	Pyrginae	08-SRNP-4659	MHMXX573-09	JF778383
642	Phanus vitreusDHJ02	Pyrginae	05-SRNP-30876	MHAHF549-06	GU150681
643	Phanus vitreusDHJ02	Pyrginae	07-SRNP-3510	MHMXP182-08	JF762592
644	Phanus vitreusDHJ02	Pyrginae	07-SRNP-4681	MHMXR638-08	JF762591
645	Phanus vitreusDHJ02	Pyrginae	07-SRNP-4607	MHMXR639-08	JF762590
646	Phanus vitreusDHJ02	Pyrginae	07-SRNP-66113	MHMXR644-08	JF762585
647	Phanus vitreusDHJ02	Pyrginae	07-SRNP-4605	MHMXR641-08	JF762588
648	Phanus vitreusDHJ02	Pyrginae	07-SRNP-4110	MHMXR640-08	JF762589
649	Phanus vitreusDHJ02	Pyrginae	06-SRNP-30361	MHAHG138-06	GU151550
650	Phanus vitreusDHJ02	Pyrginae	05-SRNP-4944	MHAHF548-06	GU150680
651	Phanus vitreusDHJ02	Pyrginae	08-SRNP-13	MHMXT059-08	JF762581
652	Phanus vitreusDHJ02	Pyrginae	07-SRNP-42826	MHMXR643-08	JF762586
653	Phanus vitreusDHJ02	Pyrginae	07-SRNP-4604	MHMXR642-08	JF762587
654	Phanus vitreusDHJ02	Pyrginae	08-SRNP-40045	MHMXT056-08	JF762584
655	Phanus vitreusDHJ02	Pyrginae	07-SRNP-24196	MHMXT057-08	JF762583
656	Phanus vitreusDHJ02	Pyrginae	07-SRNP-24814	MHMXT058-08	JF762582
657	Phanus vitreusDHJ02	Pyrginae	07-SRNP-4517	MHMXT060-08	JF762580
658	Phanus vitreusDHJ02	Pyrginae	08-SRNP-41100	MHMXW346-09	JF754061
659	Phanus vitreusDHJ02	Pyrginae	08-SRNP-40547	MHMXW348-09	JF754062
660	Phanus vitreusDHJ03	Pyrginae	98-SRNP-4474	MHAHJ171-07	JF753050
661	Phanus vitreusDHJ01	Pyrginae	03-SRNP-3212	MHAHJ186-07	JF753024
662	Phanus vitreusDHJ01	Pyrginae	98-SRNP-4291	MHAHJ194-07	JF753032
663	Phanus vitreusDHJ01	Pyrginae	98-SRNP-4284	MHAHJ211-07	JF753049
664	Phanus vitreusDHJ01	Pyrginae	98-SRNP-4554	MHAHJ202-07	JF753040
665	Phanus vitreusDHJ01	Pyrginae	00-SRNP-2518	MHAHJ187-07	JF753025
666	Phanus vitreusDHJ01	Pyrginae	08-SRNP-5259	MHMXX708-09	JF778384
667	Phanus vitreusDHJ01	Pyrginae	97-SRNP-647	CSCR188-04	DQ292967
668	Phanus vitreusDHJ01	Pyrginae	98-SRNP-4552	MHAHJ170-07	JF753009
669	Phanus vitreusDHJ01	Pyrginae	98-SRNP-4289	MHAHJ166-07	JF753005
670	Phanus vitreusDHJ01	Pyrginae	98-SRNP-4556	MHAHJ192-07	JF753030
671	Phanus vitreusDHJ01	Pyrginae	98-SRNP-4466	MHAHJ189-07	JF753027

Tree Order	Species	Subfamily	ACG Sampleid	BOLD Processid	Genbank Accession
672	Phanus vitreusDHJ01	Pyrginae	97-SRNP-554	MHAHJ179-07	JF753017
673	Phanus vitreusDHJ01	Pyrginae	98-SRNP-4507	MHAHJ208-07	JF753046
674	Phanus vitreusDHJ01	Pyrginae	08-SRNP-893	MHMXW343-09	JF754053
675	Phanus vitreusDHJ01	Pyrginae	08-SRNP-716	MHMXW355-09	JF754059
676	Phanus vitreusDHJ01	Pyrginae	09-SRNP-55618	MHMYE1479-09	GU653523
677	Phanus vitreusDHJ01	Pyrginae	97-SRNP-648	MHAHJ210-07	JF753048
678	Phanus vitreusDHJ01	Pyrginae	02-SRNP-1762	CSCR189-04	DQ292968
679	Phanus vitreusDHJ01	Pyrginae	00-SRNP-2561	MHAHJ207-07	JF753045
680	Phanus vitreusDHJ01	Pyrginae	97-SRNP-641	MHAHJ191-07	JF753029
681	Phanus vitreusDHJ01	Pyrginae	98-SRNP-4287	MHAHJ176-07	JF753014
682	Phanus vitreusDHJ01	Pyrginae	98-SRNP-4510	MHAHJ196-07	JF753034
683	Phanus vitreusDHJ01	Pyrginae	00-SRNP-2520	MHAHJ200-07	JF753038
684	Phanus vitreusDHJ01	Pyrginae	98-SRNP-4286	MHAHJ201-07	JF753039
685	Phanus vitreusDHJ01	Pyrginae	98-SRNP-4518	MHAHJ198-07	JF753036
686	Phanus vitreusDHJ01	Pyrginae	00-SRNP-2560	MHAHJ188-07	JF753026
687	Phanus vitreusDHJ01	Pyrginae	03-SRNP-3081	MHAHJ185-07	JF753023
688	Phanus vitreusDHJ01	Pyrginae	01-SRNP-9201	MHAHJ178-07	JF753016
689	Phanus vitreusDHJ01	Pyrginae	00-SRNP-2048	MHAHJ177-07	JF753015
690	Phanus vitreusDHJ01	Pyrginae	00-SRNP-2046	MHAHJ175-07	JF753013
691	Phanus vitreusDHJ01	Pyrginae	00-SRNP-2556	MHAHJ174-07	JF753012
692	Phanus vitreusDHJ01	Pyrginae	97-SRNP-640	MHAHJ173-07	JF753011
693	Phanus vitreusDHJ01	Pyrginae	00-SRNP-2517	MHAHJ168-07	JF753007
694	Phanus vitreusDHJ01	Pyrginae	98-SRNP-4553	MHAHJ167-07	JF753006
695	Phanus vitreusDHJ01	Pyrginae	06-SRNP-45146	MHAHG719-06	GU151549
696	Phanus vitreusDHJ01	Pyrginae	98-SRNP-4288	MHAHJ195-07	JF753033
697	Phanus vitreusDHJ01	Pyrginae	98-SRNP-4467	MHAHJ184-07	JF753022
698	Phanus vitreusDHJ01	Pyrginae	98-SRNP-4282	MHAHJ181-07	JF753019
699	Phanus vitreusDHJ01	Pyrginae	97-SRNP-559	MHAHJ199-07	JF753037
700	Phanus vitreusDHJ01	Pyrginae	97-SRNP-562	MHAHJ204-07	JF753042
701	Phanus vitreusDHJ01	Pyrginae	97-SRNP-643	MHAHJ203-07	JF753041
702	Phanus vitreusDHJ01	Pyrginae	00-SRNP-2562	MHAHJ197-07	JF753035
703	Phanus vitreusDHJ01	Pyrginae	97-SRNP-526	MHAHJ193-07	JF753031
704	Phanus vitreusDHJ01	Pyrginae	97-SRNP-548	MHAHJ190-07	JF753028
705	Phanus vitreusDHJ01	Pyrginae	98-SRNP-4512.01	MHAHJ183-07	JF753021
706	Phanus vitreusDHJ01	Pyrginae	97-SRNP-642	MHAHJ182-07	JF753020
707	Phanus vitreusDHJ01	Pyrginae	98-SRNP-4473	MHAHJ169-07	JF753008
708	Phanus vitreusDHJ01	Pyrginae	97-SRNP-645	MHAHJ180-07	JF753018
709	Phanus vitreusDHJ01	Pyrginae	97-SRNP-646	MHAHJ206-07	JF753044
710	Phanus vitreusDHJ01	Pyrginae	97-SRNP-555	MHAHJ209-07	JF753047
711	Phanus vitreusDHJ01	Pyrginae	00-SRNP-2550	MHAHJ205-07	JF753043
712	Phanus vitreusDHJ01	Pyrginae	08-SRNP-713	MHMXW342-09	JF754052
713	Phanus vitreusDHJ01	Pyrginae	08-SRNP-938	MHMXW344-09	JF754054
714	Phanus vitreusDHJ01	Pyrginae	08-SRNP-717	MHMXW349-09	JF754055
715	Phanus vitreusDHJ01	Pyrginae	08-SRNP-939	MHMXW350-09	JF754056
716	Phanus vitreusDHJ01	Pyrginae	08-SRNP-824	MHMXW352-09	JF754057
717	Phanus vitreusDHJ01	Pyrginae	08-SRNP-715	MHMXW354-09	JF754058
718	Phanus vitreusDHJ01	Pyrginae	08-SRNP-825	MHMXW356-09	JF754060
719	Phanus vitreusDHJ01	Pyrginae	08-SRNP-5260	MHMXY1033-09	GU666497
720	Phanus vitreusDHJ01	Pyrginae	09-SRNP-1728	MHMYE1480-09	GU653524
721	Phanus vitreusDHJ01	Pyrginae	00-SRNP-2047	MHAHJ172-07	JF753010
722	Phanus vitreusDHJ01	Pyrginae	09-SRNP-944	MHMYE1481-09	GU653521
723	Hyalothyrus neleus	Pyrginae	99-SRNP-2782	CSCR115-04	DQ292566
724	Hyalothyrus neleus	Pyrginae	03-SRNP-15448	MHAHJ165-07	JF752884
725	Hyalothyrus neleus	Pyrginae	03-SRNP-10401	MHAHJ164-07	JF752883
726	Hyalothyrus neleus	Pyrginae	02-SRNP-5338	MHAHJ163-07	JF752882
727	Hyalothyrus neleus	Pyrginae	02-SRNP-15446	MHAHJ162-07	JF752881

Tree Order	Species	Subfamily	ACG Sampleid	BOLD Processid	Genbank Accession
728	Hyalothyrus neleus	Pyrginae	02-SRNP-4193	MHAHJ161-07	JF752880
729	Hyalothyrus neleus	Pyrginae	01-SRNP-9634	MHAHJ160-07	JF752879
730	Hyalothyrus neleus	Pyrginae	00-SRNP-2796	MHAHJ159-07	JF752878
731	Hyalothyrus neleus	Pyrginae	01-SRNP-24019	MHAHJ158-07	JF752877
732	Hyalothyrus neleus	Pyrginae	01-SRNP-11940	MHAHJ157-07	JF752876
733	Hyalothyrus neleus	Pyrginae	01-SRNP-9762	MHAHJ156-07	JF752875
734	Hyalothyrus neleus	Pyrginae	01-SRNP-9010	MHAHJ155-07	JF752874
735	Hyalothyrus neleus	Pyrginae	04-SRNP-14956	MHAHC816-05	DQ292568
736	Hyalothyrus neleus	Pyrginae	04-SRNP-23854	MHAHC815-05	DQ292567
737	Hyalothyrus neleus	Pyrginae	02-SRNP-5623	CSCR468-04	DQ292565
738	Hyalothyrus neleus	Pyrginae	02-SRNP-2903	CSCR463-04	DQ292564
739	Hyalothyrus neleus	Pyrginae	07-SRNP-1289	MHMXK040-07	JF762294
740	Hyalothyrus neleus	Pyrginae	05-SRNP-23937	MHAHL097-07	JF762290
741	Hyalothyrus neleus	Pyrginae	05-SRNP-24060	MHAHL099-07	JF762291
742	Hyalothyrus neleus	Pyrginae	05-SRNP-33739	MHAHL100-07	JF762292
743	Hyalothyrus neleus	Pyrginae	07-SRNP-1874	MHAHL204-07	JF762293
744	Hyalothyrus neleus	Pyrginae	08-SRNP-20639	MHMXX904-09	JF778031
745	Entheus Burns01	Pyrginae	05-SRNP-31825	MHAHF398-06	GU150413
746	Entheus Burns01	Pyrginae	05-SRNP-31804	MHAHF406-06	GU150412
747	Entheus Burns01	Pyrginae	03-SRNP-3912	CSRII639-05	DQ292417
748	Entheus Burns01	Pyrginae	05-SRNP-31679	MHAHF413-06	GU150420
749	Entheus Burns01	Pyrginae	05-SRNP-31813	MHAHF410-06	GU150424
750	Entheus Burns01	Pyrginae	05-SRNP-31809	MHAHF403-06	GU150417
751	Entheus Burns01	Pyrginae	05-SRNP-31684	MHAHF401-06	GU150416
752	Entheus Burns01	Pyrginae	05-SRNP-31469	MHAHF418-06	GU150419
753	Entheus Burns01	Pyrginae	05-SRNP-31803	MHAHF412-06	GU150425
754	Entheus Burns01	Pyrginae	05-SRNP-31805	MHAHF409-06	GU150423
755	Entheus Burns01	Pyrginae	05-SRNP-31467	MHAHF408-06	GU150409
756	Entheus Burns01	Pyrginae	05-SRNP-31811	MHAHF407-06	GU150418
757	Entheus Burns01	Pyrginae	05-SRNP-31807	MHAHF405-06	GU150422
758	Entheus Burns01	Pyrginae	05-SRNP-31470	MHAHF404-06	GU150421
759	Entheus Burns01	Pyrginae	05-SRNP-31681	MHAHF400-06	GU150410
760	Entheus Burns01	Pyrginae	05-SRNP-31680	MHAHF399-06	GU150411
761	Entheus Burns01	Pyrginae	05-SRNP-31685	MHAHF397-06	GU150414
762	Entheus Burns01	Pyrginae	04-SRNP-35514	MHAHC807-05	DQ292438
763	Entheus Burns01	Pyrginae	04-SRNP-56807	MHAHC385-05	DQ292437
764	Entheus Burns01	Pyrginae	05-SRNP-30012	MHAHC383-05	DQ292436
765	Entheus Burns01	Pyrginae	05-SRNP-30006	MHAHC380-05	DQ292435
766	Entheus Burns01	Pyrginae	05-SRNP-30261	MHAHC378-05	DQ292434
767	Entheus Burns01	Pyrginae	04-SRNP-32629	MHAHC040-05	DQ292428
768	Entheus Burns01	Pyrginae	04-SRNP-32406	MHAHC032-05	DQ292426
769	Entheus Burns01	Pyrginae	04-SRNP-32627	MHAHC017-05	DQ292422
770	Entheus Burns01	Pyrginae	04-SRNP-22955	MHAHC001-05	DQ292420
771	Entheus Burns01	Pyrginae	04-SRNP-32897	MHAHC089-05	DQ292433
772	Entheus Burns01	Pyrginae	04-SRNP-32435	MHAHC081-05	DQ292432
773	Entheus Burns01	Pyrginae	04-SRNP-32100	MHAHC073-05	DQ292431
774	Entheus Burns01	Pyrginae	04-SRNP-32971	MHAHC057-05	DQ292430
775	Entheus Burns01	Pyrginae	04-SRNP-32102	MHAHC049-05	DQ292429
776	Entheus Burns01	Pyrginae	04-SRNP-32620	MHAHC033-05	DQ292427
777	Entheus Burns01	Pyrginae	04-SRNP-32626	MHAHC026-05	DQ292425
778	Entheus Burns01	Pyrginae	04-SRNP-32624	MHAHC018-05	DQ292423
779	Entheus Burns01	Pyrginae	04-SRNP-32619	MHAHC010-05	DQ292421
780	Entheus Burns01	Pyrginae	03-SRNP-4063	CSRII638-05	DQ292416
781	Entheus Burns01	Pyrginae	03-SRNP-4085	CSRII637-05	DQ292415
782	Entheus Burns01	Pyrginae	03-SRNP-4083	CSRII636-05	DQ292414
783	Entheus Burns01	Pyrginae	03-SRNP-4089	CSRII635-05	DQ292413

Tree Order	Species	Subfamily	ACG Sampleid	BOLD Processid	Genbank Accession
784	Entheus Burns01	Pyrginae	03-SRNP-4031	CSRII634-05	DQ292412
785	Entheus Burns01	Pyrginae	03-SRNP-3913	CSRII633-05	DQ292411
786	Entheus Burns01	Pyrginae	03-SRNP-4079	CSRII632-05	DQ292410
787	Entheus Burns01	Pyrginae	03-SRNP-4150	CSRII631-05	DQ292409
788	Entheus Burns01	Pyrginae	03-SRNP-3910	CSRII630-05	DQ292408
789	Entheus Burns01	Pyrginae	03-SRNP-4088	CSRII629-05	DQ292407
790	Entheus Burns01	Pyrginae	03-SRNP-3911	CSRII628-05	DQ292406
791	Entheus Burns01	Pyrginae	03-SRNP-4062	CSRII627-05	DQ292405
792	Entheus Burns01	Pyrginae	05-SRNP-31988	MHAHF402-06	GU150415
793	Entheus Burns01	Pyrginae	04-SRNP-32128	MHAHC025-05	DQ292424
794	Entheus Burns01	Pyrginae	02-SRNP-9578	CSCR105-04	DQ292404
795	Entheus Burns01	Pyrginae	02-SRNP-8498	CSRII677-05	DQ292419
796	Entheus Burns01	Pyrginae	02-SRNP-8540	CSRII640-05	DQ292418
797	Entheus Burns01	Pyrginae	02-SRNP-9309	CSCR473-04	DQ292403
798	Entheus Burns01	Pyrginae	08-SRNP-35619	MHMXX1036-09	JF777899
799	Entheus Burns01	Pyrginae	08-SRNP-35620	MHMXX1037-09	JF777900
800	Entheus Burns03	Pyrginae	07-SRNP-65862	MHMXR834-08	JF762188
801	Entheus Burns03	Pyrginae	03-SRNP-6132	CSCR363-04	DQ292390
802	Entheus Burns03	Pyrginae	06-SRNP-44097	MHAHK079-07	JF760674
803	Entheus Burns03	Pyrginae	07-SRNP-65775	MHMXR835-08	JF762187
804	Entheus Burns03	Pyrginae	03-SRNP-21789	CSRII129-04	DQ292393
805	Entheus Burns03	Pyrginae	02-SRNP-19102	CSCR103-04	DQ292389
806	Entheus Burns03	Pyrginae	05-SRNP-195	MHAHF414-06	GU150404
807	Entheus Burns03	Pyrginae	04-SRNP-56418	MHAHF415-06	GU150407
808	Entheus Burns03	Pyrginae	05-SRNP-30875	MHAHF420-06	GU150405
809	Entheus Burns03	Pyrginae	05-SRNP-54	MHAHF419-06	GU150403
810	Entheus Burns03	Pyrginae	05-SRNP-1251	MHAHF416-06	GU150406
811	Entheus Burns03	Pyrginae	07-SRNP-65331	MHMXR831-08	JF762191
812	Entheus Burns03	Pyrginae	07-SRNP-65174	MHMXP191-08	JF762192
813	Entheus Burns03	Pyrginae	07-SRNP-2018	MHAHL221-07	JF762185
814	Entheus Burns03	Pyrginae	07-SRNP-2055	MHAHL220-07	JF762184
815	Entheus Burns03	Pyrginae	07-SRNP-65148	MHAHL219-07	JF762183
816	Entheus Burns03	Pyrginae	07-SRNP-1374	MHAHL218-07	JF762182
817	Entheus Burns03	Pyrginae	07-SRNP-65011	MHAHL217-07	JF762181
818	Entheus Burns03	Pyrginae	06-SRNP-44104	MHAHK078-07	JF760673
819	Entheus Burns03	Pyrginae	06-SRNP-31151	MHAHH509-06	GU155401
820	Entheus Burns03	Pyrginae	05-SRNP-1370	MHAHF417-06	GU150408
821	Entheus Burns03	Pyrginae	04-SRNP-55949	MHAHC384-05	DQ292402
822	Entheus Burns03	Pyrginae	05-SRNP-196	MHAHC382-05	DQ292401
823	Entheus Burns03	Pyrginae	04-SRNP-55940	MHAHC379-05	DQ292400
824	Entheus Burns03	Pyrginae	04-SRNP-61137	MHAHC376-05	DQ292398
825	Entheus Burns03	Pyrginae	04-SRNP-61243	MHAHC375-05	DQ292397
826	Entheus Burns03	Pyrginae	04-SRNP-2113	MHAHC009-05	DQ292394
827	Entheus Burns03	Pyrginae	04-SRNP-1610	MHAHC065-05	DQ292396
828	Entheus Burns03	Pyrginae	04-SRNP-1609	MHAHC041-05	DQ292395
829	Entheus Burns03	Pyrginae	04-SRNP-60791	MHAHC377-05	DQ292399
830	Entheus Burns03	Pyrginae	06-SRNP-31150	MHAHH510-06	GU155400
831	Entheus Burns03	Pyrginae	03-SRNP-21250	CSRII128-04	DQ292392
832	Entheus Burns03	Pyrginae	03-SRNP-6130	CSCR509-04	DQ292391
833	Entheus Burns03	Pyrginae	07-SRNP-65230	MHMXR832-08	JF762190
834	Entheus Burns03	Pyrginae	07-SRNP-65191	MHMXR833-08	JF762189
835	Entheus Burns03	Pyrginae	08-SRNP-65008	MHMXT149-08	JF762186
836	Entheus Burns03	Pyrginae	08-SRNP-65118	MHMXX1034-09	JF777909
837	Entheus Burns03	Pyrginae	08-SRNP-65323	MHMXX1035-09	JF777910
838	Entheus Burns03	Pyrginae	08-SRNP-66040	MHMXY1087-09	GU666452
839	Entheus Burns03	Pyrginae	09-SRNP-56806	MHMYE1478-09	GU653526

Tree Order	Species	Subfamily	ACG Sampleid	BOLD Processid	Genbank Accession
840	Entheus Burns02	Pyrginae	08-SRNP-72638	MHMYC459-09	GU649874
841	Entheus Burns02	Pyrginae	00-SRNP-2001	CSCR102-04	DQ292388
842	Entheus Burns02	Pyrginae	99-SRNP-15392	CSCR101-04	DQ292387
843	Entheus Burns02	Pyrginae	05-SRNP-31934	MHAHF411-06	GU150401
844	Entheus Burns02	Pyrginae	08-SRNP-31023	MHMXX1038-09	JF777901
845	Entheus Burns02	Pyrginae	08-SRNP-71195	MHMXX577-09	JF777905
846	Entheus Burns02	Pyrginae	08-SRNP-33091	MHMYC460-09	GU649875
847	Entheus Burns02	Pyrginae	08-SRNP-71849	MHMXY1088-09	GU666453
848	Entheus Burns02	Pyrginae	08-SRNP-72316	MHMXY1086-09	GU666451
849	Entheus Burns02	Pyrginae	08-SRNP-71739	MHMXX718-09	JF777908
850	Entheus Burns02	Pyrginae	08-SRNP-70953	MHMXX579-09	JF777907
851	Entheus Burns02	Pyrginae	08-SRNP-70952	MHMXX578-09	JF777906
852	Entheus Burns02	Pyrginae	08-SRNP-71814	MHMXX576-09	JF777904
853	Entheus Burns02	Pyrginae	08-SRNP-71740	MHMXX575-09	JF777903
854	Entheus Burns02	Pyrginae	08-SRNP-71134	MHMXX574-09	JF777902
855	Entheus Burns02	Pyrginae	06-SRNP-20135	MHAHL098-07	JF762180
856	Entheus Burns02	Pyrginae	06-SRNP-34837	MHAHK714-07	JF760672
857	Entheus Burns02	Pyrginae	05-SRNP-21189	MHAHG736-06	GU151402
858	Entheus Burns02	Pyrginae	05-SRNP-32113	MHAHF716-06	GU150402
859	Entheus Burns02	Pyrginae	05-SRNP-34263	MHAHG148-06	GU151401
860	Entheus Burns02	Pyrginae	06-SRNP-32638	MHAHI570-06	GU155960
861	Entheus Burns02	Pyrginae	05-SRNP-34780	MHAHG147-06	GU151400
862	Entheus Burns02	Pyrginae	02-SRNP-14694	CSCR472-04	DQ292386
863	Entheus Burns02	Pyrginae	09-SRNP-30020	MHMYC458-09	GU649873
864	Entheus Burns02	Pyrginae	09-SRNP-70294	MHMYE886-09	GU653717
865	Entheus Burns02	Pyrginae	09-SRNP-71529	MHMYE1477-09	GU653525
866	Entheus Burns02	Pyrginae	09-SRNP-70531	MHMYG2420-10	HM885842
867	Antigonus nearchus	Pyrginae	00-SRNP-12980	CSCR014-04	DQ291816
868	Antigonus nearchus	Pyrginae	00-SRNP-10364	MHAHI215-06	GU155757
869	Antigonus nearchus	Pyrginae	06-SRNP-32799	MHAHI181-06	GU155758
870	Antigonus nearchus	Pyrginae	01-SRNP-5567	CSCR015-04	DQ291817
871	Antigonus nearchus	Pyrginae	96-SRNP-11137	MHAHI216-06	GU155756
872	Antigonus nearchus	Pyrginae	06-SRNP-67851	MHMXH855-07	JF760245
873	Timochreon satyrus	Pyrginae	98-SRNP-4800	MHAHI212-06	GU156361
874	Timochreon satyrus	Pyrginae	07-SRNP-55646	MHMXK012-07	JF763253
875	Timochreon satyrus	Pyrginae	07-SRNP-58884	MHMXR023-08	JF763252
876	Timochreon satyrus	Pyrginae	07-SRNP-55299	MHMXK009-07	JF763256
877	Timochreon satyrus	Pyrginae	06-SRNP-59556	MHMXH893-07	JF761223
878	Timochreon satyrus	Pyrginae	06-SRNP-57946	MHAHJ668-07	JF753193
879	Timochreon satyrus	Pyrginae	02-SRNP-5343	MHAHI208-06	GU156365
880	Timochreon satyrus	Pyrginae	00-SRNP-3407	MHAHI207-06	GU156363
881	Timochreon satyrus	Pyrginae	01-SRNP-11952	MHAHI206-06	GU156366
882	Timochreon satyrus	Pyrginae	05-SRNP-24369	MHAHF610-06	GU150937
883	Timochreon satyrus	Pyrginae	04-SRNP-16184	MHAHF123-06	GU150935
884	Timochreon satyrus	Pyrginae	04-SRNP-26992	MHAHF122-06	GU150936
885	Timochreon satyrus	Pyrginae	04-SRNP-21555	MHAHD878-05	GU161896
886	Timochreon satyrus	Pyrginae	97-SRNP-4864	MHAHI210-06	GU156367
887	Timochreon satyrus	Pyrginae	02-SRNP-4030	CSCR266-04	DQ293605
888	Timochreon satyrus	Pyrginae	02-SRNP-15405	CSCR267-04	DQ293606
889	Timochreon satyrus	Pyrginae	97-SRNP-4132	MHAHI211-06	GU156362
890	Timochreon satyrus	Pyrginae	01-SRNP-11309	MHAHI209-06	GU156364
891	Timochreon satyrus	Pyrginae	02-SRNP-14811	MHAHI213-06	GU156368
892	Timochreon satyrus	Pyrginae	02-SRNP-33938	MHAHI214-06	GU156360
893	Timochreon satyrus	Pyrginae	07-SRNP-55645	MHMXK010-07	JF763255
894	Timochreon satyrus	Pyrginae	07-SRNP-55655	MHMXK011-07	JF763254
895	Timochreon satyrus	Pyrginae	09-SRNP-73053	MHMYG2421-10	HM885844

Tree Order	Species	Subfamily	ACG Sampleid	BOLD Processid	Genbank Accession
896	Heliopetes lavianaDHJ02	Pyrginae	06-SRNP-19429	MHAHI522-06	GU155983
897	Heliopetes lavianaDHJ02	Pyrginae	06-SRNP-19419	MHAHK726-07	JF760794
898	Heliopetes lavianaDHJ02	Pyrginae	06-SRNP-19431	MHAHK725-07	JF760793
899	Heliopetes lavianaDHJ02	Pyrginae	06-SRNP-18783	MHAHK724-07	JF760792
900	Heliopetes lavianaDHJ02	Pyrginae	06-SRNP-19423	MHAHK723-07	JF760791
901	Heliopetes lavianaDHJ02	Pyrginae	06-SRNP-19396	MHAHK721-07	JF760789
902	Heliopetes lavianaDHJ02	Pyrginae	06-SRNP-19523	MHAHK159-07	JF760787
903	Heliopetes lavianaDHJ02	Pyrginae	06-SRNP-19420	MHAHI528-06	GU155986
904	Heliopetes lavianaDHJ02	Pyrginae	06-SRNP-19397	MHAHI526-06	GU155982
905	Heliopetes lavianaDHJ02	Pyrginae	06-SRNP-19416	MHAHK720-07	JF760788
906	Heliopetes lavianaDHJ02	Pyrginae	06-SRNP-18781	MHAHK727-07	JF760795
907	Heliopetes lavianaDHJ02	Pyrginae	04-SRNP-45705	MHAHD679-05	GU161540
908	Heliopetes lavianaDHJ02	Pyrginae	04-SRNP-45704	MHAHD680-05	GU161541
909	Heliopetes lavianaDHJ02	Pyrginae	06-SRNP-19448	MHAHI524-06	GU155985
910	Heliopetes lavianaDHJ02	Pyrginae	06-SRNP-19402	MHAHI525-06	GU155984
911	Heliopetes lavianaDHJ02	Pyrginae	06-SRNP-19622	MHAHK158-07	JF760786
912	Heliopetes lavianaDHJ02	Pyrginae	06-SRNP-18780	MHAHK722-07	JF760790
913	Heliopetes lavianaDHJ02	Pyrginae	06-SRNP-19414	MHAHK728-07	JF760796
914	Heliopetes lavianaDHJ02	Pyrginae	06-SRNP-19401	MHAHK729-07	JF760797
915	Heliopetes alana	Pyrginae	04-SRNP-48169	MHAHD676-05	GU161538
916	Heliopetes alana	Pyrginae	05-SRNP-55030	MHAHD678-05	GU161537
917	Heliopetes alana	Pyrginae	05-SRNP-5611	MHAHE566-06	GU149686
918	Heliopetes alana	Pyrginae	06-SRNP-6703	MHAHJ761-07	JF752847
919	Heliopetes arsalte	Pyrginae	09-SRNP-2102	MHMYE1536-09	HM391096
920	Heliopetes arsalte	Pyrginae	09-SRNP-44424	MHMYE871-09	GU653730
921	Heliopetes arsalte	Pyrginae	05-SRNP-31020	MHAHF537-06	GU150500
922	Heliopetes arsalte	Pyrginae	05-SRNP-34906	MHAHF708-06	GU150501
923	Heliopetes arsalte	Pyrginae	06-SRNP-2053	MHAHG803-06	GU151432
924	Heliopetes arsalte	Pyrginae	04-SRNP-42488	MHAHD672-05	GU161539
925	Heliopetes arsalte	Pyrginae	05-SRNP-3992	MHAHE565-06	GU149687
926	Heliopetes arsalte	Pyrginae	06-SRNP-19606	MHMXH896-07	JF760783
927	Heliopetes arsalte	Pyrginae	07-SRNP-55805	MHAHK701-07	JF760784
928	Heliopetes arsalte	Pyrginae	09-SRNP-71844	MHMYE1535-09	HM391095
929	Heliopetes arsalte	Pyrginae	09-SRNP-2103	MHMYE1537-09	HM391097
930	Heliopetes arsalte	Pyrginae	09-SRNP-72759	MHMYE1538-09	HM391098
931	Pyrgus adepta	Pyrginae	05-SRNP-61486	MHAHF705-06	GU150730
932	Pyrgus adepta	Pyrginae	05-SRNP-3108	MHAHF552-06	GU150729
933	Pyrgus adepta	Pyrginae	05-SRNP-4774	MHAHE569-06	GU149867
934	Pyrgus adepta	Pyrginae	05-SRNP-3111	MHAHF551-06	GU150728
935	Pyrgus adepta	Pyrginae	05-SRNP-6162	MHAHF706-06	GU150731
936	Pyrgus adepta	Pyrginae	06-SRNP-3986	MHAHI168-06	GU156285
937	Pyrgus adepta	Pyrginae	06-SRNP-22079	MHAHJ710-07	JF753095
938	Pyrgus adepta	Pyrginae	07-SRNP-57963	MHMXO973-08	JF762698
939	Pyrgus adepta	Pyrginae	08-SRNP-40797	MHMXX865-09	JF778418
940	Pyrgus adepta	Pyrginae	09-SRNP-35755	MHMYE1565-09	HM391125
941	Pyrgus oileusDHJ01	Pyrginae	05-SRNP-47404	MHAHE568-06	GU149868
942	Pyrgus oileusDHJ02	Pyrginae	09-SRNP-72609	MHMYE1534-09	HM391094
943	Pyrgus oileusDHJ02	Pyrginae	09-SRNP-57316	MHMYH158-10	HM887306
944	Pyrgus oileusDHJ02	Pyrginae	04-SRNP-4880	MHAHD683-05	GU161798
945	Pyrgus oileusDHJ02	Pyrginae	97-SRNP-6018	MHAHK479-07	JF761073
946	Pyrgus oileusDHJ02	Pyrginae	97-SRNP-6017	MHAHK480-07	JF761074
947	Pyrgus oileusDHJ02	Pyrginae	97-SRNP-6323	MHAHK482-07	JF761075
948	Pyrgus oileusDHJ02	Pyrginae	07-SRNP-30612	MHAHK702-07	JF761076
949	Pyrgus oileusDHJ02	Pyrginae	07-SRNP-41537	MHAHL203-07	JF762699
950	Pyrgus oileusDHJ02	Pyrginae	07-SRNP-33658	MHMXR001-08	JF762700
951	Pyrgus oileusDHJ02	Pyrginae	09-SRNP-41756	MHMYH159-10	HM887307

Tree Order	Species	Subfamily	ACG Sampleid	BOLD Processid	Genbank Accession
952	Pyrgus oileusDHJ03	Pyrginae	05-SRNP-41121	MHAHF553-06	GU150733
953	Pyrgus oileusDHJ03	Pyrginae	05-SRNP-41120	MHAHF555-06	GU150732
954	Pyrgus oileusDHJ03	Pyrginae	97-SRNP-468	MHAHK481-07	JF761078
955	Pyrgus oileusDHJ03	Pyrginae	05-SRNP-5995	MHAHF707-06	GU150735
956	Pyrgus oileusDHJ03	Pyrginae	93-SRNP-6385	MHAHK484-07	JF761079
957	Pyrgus oileusDHJ03	Pyrginae	06-SRNP-59892	MHMXH869-07	JF761077
958	Pyrgus oileusDHJ03	Pyrginae	05-SRNP-42130	MHAHE567-06	GU149870
959	Pyrgus oileusDHJ03	Pyrginae	05-SRNP-3112	MHAHE373-05	GU149869
960	Pyrgus oileusDHJ03	Pyrginae	04-SRNP-4881	MHAHD684-05	GU161797
961	Pyrgus oileusDHJ03	Pyrginae	05-SRNP-56295	MHAHF554-06	GU150734
962	Pyrgus oileusDHJ03	Pyrginae	07-SRNP-58554	MHMXO972-08	JF762701
963	Pyrgus oileusDHJ03	Pyrginae	08-SRNP-71705	MHMXX866-09	JF778419
964	Pyrgus oileusDHJ03	Pyrginae	09-SRNP-44136	MHMYE870-09	GU653733
965	Pyrgus oileusDHJ03	Pyrginae	09-SRNP-73409	MHMYH157-10	HM887305
966	Pyrgus oileusDHJ03	Pyrginae	09-SRNP-73394	MHMYH160-10	HM887308
967	Zopyrion sandace	Pyrginae	93-SRNP-7566	MHMYH1661-10	HQ992672
968	Zopyrion sandace	Pyrginae	97-SRNP-9768	MHMYH1662-10	HQ992673
969	Antigonus erosus	Pyrginae	04-SRNP-14882	MHAHE205-05	GU149382
970	Antigonus erosus	Pyrginae	04-SRNP-15034	MHAHE198-05	GU149378
971	Antigonus erosus	Pyrginae	07-SRNP-57033	MHAHL208-07	JF761410
972	Antigonus erosus	Pyrginae	04-SRNP-14311	MHAHE201-05	GU149376
973	Antigonus erosus	Pyrginae	04-SRNP-47715	MHAHE204-05	GU149388
974	Antigonus erosus	Pyrginae	04-SRNP-48661	MHAHE199-05	GU149381
975	Antigonus erosus	Pyrginae	05-SRNP-34080	MHAHG146-06	GU151066
976	Antigonus erosus	Pyrginae	05-SRNP-2158	MHMXN264-07	JF761416
977	Antigonus erosus	Pyrginae	05-SRNP-45294	MHMXN263-07	JF761417
978	Antigonus erosus	Pyrginae	05-SRNP-2157	MHMXN262-07	JF761418
979	Antigonus erosus	Pyrginae	05-SRNP-55042	MHMXN261-07	JF761419
980	Antigonus erosus	Pyrginae	07-SRNP-40136	MHAHK362-07	JF760244
981	Antigonus erosus	Pyrginae	06-SRNP-6733	MHAHJ753-07	JF752376
982	Antigonus erosus	Pyrginae	06-SRNP-44597	MHAHJ602-07	JF752375
983	Antigonus erosus	Pyrginae	04-SRNP-15182	MHAHE212-05	GU149380
984	Antigonus erosus	Pyrginae	04-SRNP-48127	MHAHE211-05	GU149384
985	Antigonus erosus	Pyrginae	04-SRNP-47713	MHAHE210-05	GU149386
986	Antigonus erosus	Pyrginae	04-SRNP-48470	MHAHE209-05	GU149387
987	Antigonus erosus	Pyrginae	04-SRNP-4027	MHAHE207-05	GU149383
988	Antigonus erosus	Pyrginae	04-SRNP-15037	MHAHE206-05	GU149379
989	Antigonus erosus	Pyrginae	04-SRNP-14884	MHAHE203-05	GU149375
990	Antigonus erosus	Pyrginae	04-SRNP-14704	MHAHE202-05	GU149377
991	Antigonus erosus	Pyrginae	04-SRNP-48663	MHAHE200-05	GU149373
992	Antigonus erosus	Pyrginae	04-SRNP-46846	MHAHE197-05	GU149374
993	Antigonus erosus	Pyrginae	03-SRNP-916	CSCR303-04	DQ291814
994	Antigonus erosus	Pyrginae	04-SRNP-14889	MHAHE208-05	GU149385
995	Antigonus erosus	Pyrginae	06-SRNP-5959	MHAHI178-06	GU155755
996	Antigonus erosus	Pyrginae	03-SRNP-5120	CSCR304-04	DQ291815
997	Antigonus erosus	Pyrginae	05-SRNP-5668	MHMXN265-07	JF761415
998	Antigonus erosus	Pyrginae	07-SRNP-2954	MHAHL209-07	JF761411
999	Antigonus erosus	Pyrginae	07-SRNP-42448	MHMXP220-08	JF761414
1000	Antigonus erosus	Pyrginae	07-SRNP-3283	MHMXP221-08	JF761413
1001	Antigonus erosus	Pyrginae	07-SRNP-23787	MHMXR024-08	JF761412
1002	Antigonus erosus	Pyrginae	08-SRNP-31067	MHMXX860-09	JF777591
1003	Antigonus erosus	Pyrginae	09-SRNP-43129	MHMYG2428-10	HM885851
1004	Antigonus erosus	Pyrginae	09-SRNP-67761	MHMYG2499-10	HM885927
1005	Carrhenes calidius	Pyrginae	01-SRNP-11410	CSCR051-04	DQ291965
1006	Carrhenes calidius	Pyrginae	05-SRNP-1277	MHAHF074-06	GU150306
1007	Carrhenes calidius	Pyrginae	08-SRNP-21848	MHMXX876-09	JF777722

Tree Order	Species	Subfamily	ACG Sampleid	BOLD Processid	Genbank Accession
1008	Carrhenes calidius	Pyrginae	08-SRNP-21846	MHMXX878-09	JF777724
1009	Carrhenes calidius	Pyrginae	08-SRNP-6834	MHMYC508-09	GU649827
1010	Carrhenes calidius	Pyrginae	06-SRNP-42410	MHAHI615-06	GU155854
1011	Carrhenes calidius	Pyrginae	06-SRNP-42411	MHAHI688-06	GU155857
1012	Carrhenes calidius	Pyrginae	05-SRNP-41755	MHAHF324-06	GU150311
1013	Carrhenes calidius	Pyrginae	06-SRNP-23205	MHAHJ491-07	JF752497
1014	Carrhenes calidius	Pyrginae	06-SRNP-23245	MHAHJ547-07	JF752500
1015	Carrhenes calidius	Pyrginae	97-SRNP-5991	CSCR050-04	DQ291964
1016	Carrhenes calidius	Pyrginae	05-SRNP-41905	MHAHF325-06	GU150309
1017	Carrhenes calidius	Pyrginae	05-SRNP-41664	MHAHF323-06	GU150310
1018	Carrhenes calidius	Pyrginae	08-SRNP-4738	MHMXX517-09	JF777718
1019	Carrhenes calidius	Pyrginae	08-SRNP-4735	MHMXX516-09	JF777717
1020	Carrhenes calidius	Pyrginae	08-SRNP-4737	MHMXX515-09	JF777716
1021	Carrhenes calidius	Pyrginae	08-SRNP-4736	MHMXX514-09	JF777715
1022	Carrhenes calidius	Pyrginae	08-SRNP-21817	MHMXX877-09	JF777723
1023	Carrhenes calidius	Pyrginae	08-SRNP-20689	MHMXX875-09	JF777721
1024	Carrhenes calidius	Pyrginae	08-SRNP-21489	MHMXX873-09	JF777720
1025	Carrhenes calidius	Pyrginae	08-SRNP-20418	MHMXX872-09	JF777719
1026	Carrhenes calidius	Pyrginae	06-SRNP-46362	MHAHJ728-07	JF752503
1027	Carrhenes calidius	Pyrginae	06-SRNP-23272	MHAHJ715-07	JF752502
1028	Carrhenes calidius	Pyrginae	06-SRNP-23273	MHAHJ493-07	JF752499
1029	Carrhenes calidius	Pyrginae	06-SRNP-23275	MHAHJ492-07	JF752498
1030	Carrhenes calidius	Pyrginae	06-SRNP-42412	MHAHI614-06	GU155855
1031	Carrhenes calidius	Pyrginae	06-SRNP-42408	MHAHI613-06	GU155856
1032	Carrhenes calidius	Pyrginae	04-SRNP-42196	MHAHE064-05	GU149471
1033	Carrhenes calidius	Pyrginae	04-SRNP-49279	MHAHE063-05	GU149473
1034	Carrhenes calidius	Pyrginae	03-SRNP-37789	MHAHE062-05	GU149472
1035	Carrhenes calidius	Pyrginae	04-SRNP-40341	MHAHC184-05	DQ291966
1036	Carrhenes calidius	Pyrginae	05-SRNP-4276	MHAHF326-06	GU150308
1037	Carrhenes calidius	Pyrginae	05-SRNP-25254	MHAHF711-06	GU150307
1038	Carrhenes calidius	Pyrginae	06-SRNP-22483	MHAHJ548-07	JF752501
1039	Carrhenes calidius	Pyrginae	09-SRNP-21630	MHMYG2380-10	HM885803
1040	Carrhenes fuscescens	Pyrginae	92-SRNP-3412	MHMYH1672-10	HQ992680
1041	Carrhenes fuscescens	Pyrginae	95-SRNP-6803	MHMYH1674-10	HQ992681
1042	Carrhenes fuscescens	Pyrginae	95-SRNP-6809	MHMYH1670-10	HQ992679
1043	Carrhenes fuscescens	Pyrginae	96-SRNP-7811	MHMYH1669-10	HQ992678
1044	Carrhenes fuscescens	Pyrginae	95-SRNP-6818	MHMYH1668-10	HQ992677
1045	Carrhenes fuscescens	Pyrginae	95-SRNP-6813	MHMYH1667-10	HQ992676
1046	Carrhenes fuscescens	Pyrginae	95-SRNP-6806	MHMYH1666-10	HQ992675
1047	Carrhenes fuscescens	Pyrginae	95-SRNP-6801	MHMYH1665-10	HQ992674
1048	Carrhenes fuscescens	Pyrginae	95-SRNP-6819	CSCR055-04	DQ291972
1049	Carrhenes fuscescens	Pyrginae	95-SRNP-6804	MHMYH1675-10	HQ992682
1050	Carrhenes fuscescens	Pyrginae	92-SRNP-3012	CSCR054-04	DQ291971
1051	Carrhenes fuscescens	Pyrginae	92-SRNP-3014	MHMYH1676-10	HQ992683
1052	Carrhenes fuscescens	Pyrginae	93-SRNP-1137	MHMYH1677-10	HQ992684
1053	Carrhenes fuscescens	Pyrginae	95-SRNP-6820	MHMYH1678-10	HQ992685
1054	Carrhenes fuscescens	Pyrginae	92-SRNP-3004	MHMYH1679-10	HQ992686
1055	Carrhenes fuscescens	Pyrginae	92-SRNP-3393	MHMYH1680-10	HQ992687
1056	Anisochoria polysticta	Pyrginae	03-SRNP-26746	CSCR302-04	DQ291810
1057	Anisochoria polysticta	Pyrginae	98-SRNP-12227	CSCR477-04	DQ291811
1058	Anisochoria polysticta	Pyrginae	04-SRNP-15485	MHAHD064-05	GU161224
1059	Anisochoria polysticta	Pyrginae	04-SRNP-15751	MHAHD065-05	GU161225
1060	Anisochoria polysticta	Pyrginae	04-SRNP-15486	MHAHD066-05	GU161223
1061	Anisochoria polysticta	Pyrginae	07-SRNP-55564	MHAHK363-07	JF760231
1062	Xenophanes tryxus	Pyrginae	05-SRNP-55815	MHAHF291-06	GU151038
1063	Xenophanes tryxus	Pyrginae	04-SRNP-23772	MHAHD690-05	GU162044

Tree Order	Species	Subfamily	ACG Sampleid	BOLD Processid	Genbank Accession
1064	Xenophanes tryxus	Pyrginae	08-SRNP-709	MHMXX884-09	JF778632
1065	Xenophanes tryxus	Pyrginae	08-SRNP-40407	MHMXT110-08	JF763508
1066	Xenophanes tryxus	Pyrginae	08-SRNP-40406	MHMXT109-08	JF763509
1067	Xenophanes tryxus	Pyrginae	07-SRNP-42914	MHMXR022-08	JF763511
1068	Xenophanes tryxus	Pyrginae	07-SRNP-21825	MHAHL197-07	JF763505
1069	Xenophanes tryxus	Pyrginae	07-SRNP-42042	MHAHL196-07	JF763504
1070	Xenophanes tryxus	Pyrginae	07-SRNP-2177	MHAHL195-07	JF763503
1071	Xenophanes tryxus	Pyrginae	07-SRNP-2117	MHAHL194-07	JF763502
1072	Xenophanes tryxus	Pyrginae	07-SRNP-32155	MHAHK367-07	JF761318
1073	Xenophanes tryxus	Pyrginae	05-SRNP-55198	MHAHD698-05	GU162046
1074	Xenophanes tryxus	Pyrginae	04-SRNP-15683	MHAHD697-05	GU162042
1075	Xenophanes tryxus	Pyrginae	04-SRNP-41977	MHAHD696-05	GU162048
1076	Xenophanes tryxus	Pyrginae	04-SRNP-15686	MHAHD695-05	GU162047
1077	Xenophanes tryxus	Pyrginae	04-SRNP-23770	MHAHD693-05	GU162041
1078	Xenophanes tryxus	Pyrginae	04-SRNP-23767	MHAHD692-05	GU162043
1079	Xenophanes tryxus	Pyrginae	04-SRNP-23769	MHAHD691-05	GU162045
1080	Xenophanes tryxus	Pyrginae	04-SRNP-24073	MHAHD694-05	GU162049
1081	Xenophanes tryxus	Pyrginae	05-SRNP-30987	MHAHF290-06	GU151039
1082	Xenophanes tryxus	Pyrginae	08-SRNP-40408	MHMXT108-08	JF763510
1083	Xenophanes tryxus	Pyrginae	92-SRNP-4663	CSCR293-04	DQ293930
1084	Xenophanes tryxus	Pyrginae	93-SRNP-5818	CSCR294-04	DQ293931
1085	Xenophanes tryxus	Pyrginae	08-SRNP-40109	MHMXT111-08	JF763507
1086	Xenophanes tryxus	Pyrginae	08-SRNP-40108	MHMXT112-08	JF763506
1087	Xenophanes tryxus	Pyrginae	08-SRNP-652	MHMXX879-09	JF778627
1088	Xenophanes tryxus	Pyrginae	08-SRNP-653	MHMXX880-09	JF778628
1089	Xenophanes tryxus	Pyrginae	08-SRNP-650	MHMXX881-09	JF778629
1090	Xenophanes tryxus	Pyrginae	08-SRNP-710	MHMXX882-09	JF778630
1091	Xenophanes tryxus	Pyrginae	08-SRNP-1428	MHMXX883-09	JF778631
1092	Xenophanes tryxus	Pyrginae	09-SRNP-75047	MHMYE1532-09	HM391092
1093	Xenophanes tryxus	Pyrginae	09-SRNP-42129	MHMYG2377-10	HM885800
1094	Carrhenes meridensis	Pyrginae	97-SRNP-1522	CSCR056-04	DQ291973
1095	Carrhenes canescensDHJ02	Pyrginae	04-SRNP-47072	MHAHC176-05	DQ291970
1096	Carrhenes canescensDHJ02	Pyrginae	05-SRNP-65241	MHAHF710-06	GU150313
1097	Carrhenes canescensDHJ02	Pyrginae	04-SRNP-2161	MHMYH1696-10	HQ963287
1098	Carrhenes canescensDHJ02	Pyrginae	08-SRNP-1435	MHMXX870-09	JF777727
1099	Carrhenes canescensDHJ02	Pyrginae	07-SRNP-2297	MHAHL193-07	JF761756
1100	Carrhenes canescensDHJ02	Pyrginae	07-SRNP-57525	MHAHL192-07	JF761755
1101	Carrhenes canescensDHJ02	Pyrginae	05-SRNP-55226	MHAHE066-05	GU149476
1102	Carrhenes canescensDHJ02	Pyrginae	04-SRNP-40196	CSRII111-04	DQ291968
1103	Carrhenes canescensDHJ02	Pyrginae	94-SRNP-576	MHMYH1690-10	HQ963281
1104	Carrhenes canescensDHJ02	Pyrginae	97-SRNP-796	MHMYH1692-10	HQ963283
1105	Carrhenes canescensDHJ02	Pyrginae	98-SRNP-6223	MHMYH1688-10	HQ992695
1106	Carrhenes canescensDHJ02	Pyrginae	98-SRNP-2076	MHMYH1687-10	HQ992694
1107	Carrhenes canescensDHJ02	Pyrginae	98-SRNP-2077	MHMYH1686-10	HQ992693
1108	Carrhenes canescensDHJ02	Pyrginae	09-SRNP-55887	MHMYE1539-09	HM391099
1109	Carrhenes canescensDHJ02	Pyrginae	08-SRNP-1302	MHMXX874-09	JF777728
1110	Carrhenes canescensDHJ02	Pyrginae	04-SRNP-35287	MHAHC168-05	DQ291969
1111	Carrhenes canescensDHJ02	Pyrginae	98-SRNP-2086	MHMYH1681-10	HQ992688
1112	Carrhenes canescensDHJ02	Pyrginae	06-SRNP-3394	MHAHH223-06	GU155212
1113	Carrhenes canescensDHJ02	Pyrginae	94-SRNP-572	CSCR053-04	DQ291967
1114	Carrhenes canescensDHJ02	Pyrginae	94-SRNP-598	MHMYH1689-10	JF751717
1115	Carrhenes canescensDHJ02	Pyrginae	00-SRNP-11710	MHMYH1694-10	HQ963285
1116	Carrhenes canescensDHJ02	Pyrginae	02-SRNP-32202	MHMYH1699-10	HQ963290
1117	Carrhenes canescensDHJ02	Pyrginae	98-SRNP-2075	MHMYH1700-10	HQ963291
1118	Carrhenes canescensDHJ02	Pyrginae	98-SRNP-6658	MHMYH1702-10	HQ963293
1119	Carrhenes canescensDHJ02	Pyrginae	99-SRNP-4815	MHMYH1704-10	HQ963295

Tree Order	Species	Subfamily	ACG Sampleid	BOLD Processid	Genbank Accession
1120	Carrhenes canescensDHJ01	Pyrginae	08-SRNP-1544	MHMXX871-09	JF777726
1121	Carrhenes canescensDHJ01	Pyrginae	01-SRNP-2081	MHMYH1707-10	HQ963298
1122	Carrhenes canescensDHJ01	Pyrginae	96-SRNP-11205	MHMYH1708-10	HQ963299
1123	Carrhenes canescensDHJ01	Pyrginae	99-SRNP-2264	MHMYH1705-10	HQ963296
1124	Carrhenes canescensDHJ01	Pyrginae	99-SRNP-2257	MHMYH1706-10	HQ963297
1125	Carrhenes canescensDHJ01	Pyrginae	02-SRNP-18792	MHMYH1698-10	HQ963289
1126	Carrhenes canescensDHJ01	Pyrginae	98-SRNP-4264	MHMYH1701-10	HQ963292
1127	Carrhenes canescensDHJ01	Pyrginae	04-SRNP-45774	MHMYH1695-10	HQ963286
1128	Carrhenes canescensDHJ01	Pyrginae	01-SRNP-1279	MHMYH1697-10	HQ963288
1129	Carrhenes canescensDHJ01	Pyrginae	98-SRNP-6283	MHMYH1685-10	HQ992692
1130	Carrhenes canescensDHJ01	Pyrginae	97-SRNP-6032	MHMYH1691-10	HQ963282
1131	Carrhenes canescensDHJ01	Pyrginae	98-SRNP-6194	MHMYH1684-10	HQ992691
1132	Carrhenes canescensDHJ01	Pyrginae	98-SRNP-6497	MHMYH1683-10	HQ992690
1133	Carrhenes canescensDHJ01	Pyrginae	98-SRNP-6704	MHMYH1682-10	HQ992689
1134	Carrhenes canescensDHJ01	Pyrginae	08-SRNP-45047	MHMXX869-09	JF777725
1135	Carrhenes canescensDHJ01	Pyrginae	07-SRNP-1460	MHAHK703-07	JF760461
1136	Carrhenes canescensDHJ01	Pyrginae	06-SRNP-59186	MHMXH876-07	JF760460
1137	Carrhenes canescensDHJ01	Pyrginae	06-SRNP-3104	MHAHH426-06	GU155213
1138	Carrhenes canescensDHJ01	Pyrginae	06-SRNP-2357	MHAHG825-06	GU151224
1139	Carrhenes canescensDHJ01	Pyrginae	06-SRNP-3298	MHAHG824-06	GU151223
1140	Carrhenes canescensDHJ01	Pyrginae	04-SRNP-35258	MHAHE067-05	GU149475
1141	Carrhenes canescensDHJ01	Pyrginae	00-SRNP-1813	MHMYH1693-10	HQ963284
1142	Carrhenes canescensDHJ01	Pyrginae	04-SRNP-48517	MHAHE065-05	GU149474
1143	Carrhenes canescensDHJ01	Pyrginae	05-SRNP-65401	MHAHF709-06	GU150312
1144	Carrhenes canescensDHJ01	Pyrginae	98-SRNP-6890	MHMYH1703-10	HQ963294
1145	Carrhenes canescensDHJ01	Pyrginae	97-SRNP-6031	MHMYH1709-10	HQ963300
1146	Atarnes sallei	Pyrginae	06-SRNP-45182	MHAHG802-06	GU151206
1147	Atarnes sallei	Pyrginae	06-SRNP-1582	MHAHG154-06	GU151204
1148	Atarnes sallei	Pyrginae	07-SRNP-35256	MHMXN421-07	JF761670
1149	Atarnes sallei	Pyrginae	08-SRNP-20794	MHMXX861-09	JF777640
1150	Atarnes sallei	Pyrginae	06-SRNP-3263	MHAHH475-06	GU155202
1151	Atarnes sallei	Pyrginae	05-SRNP-2058	MHAHF307-06	GU150256
1152	Atarnes sallei	Pyrginae	08-SRNP-36067	MHMXX530-09	GU666354
1153	Atarnes sallei	Pyrginae	08-SRNP-56142	MHMXX863-09	JF777641
1154	Atarnes sallei	Pyrginae	07-SRNP-58936	MHMXR917-08	JF761668
1155	Atarnes sallei	Pyrginae	07-SRNP-59529	MHMXR916-08	JF761669
1156	Atarnes sallei	Pyrginae	06-SRNP-18572	MHAHK713-07	JF760396
1157	Atarnes sallei	Pyrginae	06-SRNP-59199	MHMXH871-07	JF760397
1158	Atarnes sallei	Pyrginae	06-SRNP-3340	MHAHG801-06	GU151205
1159	Atarnes sallei	Pyrginae	06-SRNP-35204	MHAHH476-06	GU155201
1160	Atarnes sallei	Pyrginae	05-SRNP-60344	MHAHF685-06	GU150259
1161	Atarnes sallei	Pyrginae	05-SRNP-61255	MHAHF684-06	GU150258
1162	Atarnes sallei	Pyrginae	05-SRNP-60346	MHAHF683-06	GU150257
1163	Atarnes sallei	Pyrginae	06-SRNP-55198	MHAHG155-06	GU151203
1164	Atarnes sallei	Pyrginae	95-SRNP-435	CSCR042-04	DQ291893
1165	Atarnes sallei	Pyrginae	89-SRNP-92	CSCR041-04	DQ291892
1166	Atarnes sallei	Pyrginae	06-SRNP-21052	MHAHH479-06	GU155204
1167	Atarnes sallei	Pyrginae	06-SRNP-21070	MHAHH480-06	GU155203
1168	Atarnes sallei	Pyrginae	06-SRNP-57921	MHAHJ716-07	JF752468
1169	Atarnes sallei	Pyrginae	07-SRNP-56900	MHAHK712-07	JF760395
1170	Atarnes sallei	Pyrginae	07-SRNP-65059	MHMXN419-07	JF761672
1171	Atarnes sallei	Pyrginae	07-SRNP-65058	MHMXN420-07	JF761671
1172	Atarnes sallei	Pyrginae	08-SRNP-56256	MHMXX715-09	JF777639
1173	Potamanaxas unifasciata	Pyrginae	07-SRNP-2633	MHMXN354-07	JF762696
1174	Potamanaxas unifasciata	Pyrginae	98-SRNP-2034	CSRII740-05	DQ293110
1175	Potamanaxas unifasciata	Pyrginae	04-SRNP-805	CSRII626-05	DQ293108

Tree Order	Species	Subfamily	ACG Sampleid	BOLD Processid	Genbank Accession
1176	Potamanaxas unifasciata	Pyrginae	05-SRNP-58611	MHAHI644-06	GU156284
1177	Potamanaxas unifasciata	Pyrginae	05-SRNP-35001	MHAHI645-06	GU156283
1178	Potamanaxas unifasciata	Pyrginae	94-SRNP-6414	CSRII754-05	DQ293118
1179	Potamanaxas unifasciata	Pyrginae	00-SRNP-12802	CSRII744-05	DQ293114
1180	Potamanaxas unifasciata	Pyrginae	00-SRNP-12804	CSRII743-05	DQ293113
1181	Potamanaxas unifasciata	Pyrginae	04-SRNP-363	CSRII625-05	DQ293107
1182	Potamanaxas unifasciata	Pyrginae	04-SRNP-365	CSRII623-05	DQ293105
1183	Potamanaxas unifasciata	Pyrginae	00-SRNP-12805	CSRII741-05	DQ293111
1184	Potamanaxas unifasciata	Pyrginae	04-SRNP-496	CSRII624-05	DQ293106
1185	Potamanaxas unifasciata	Pyrginae	98-SRNP-2036	CSRII739-05	DQ293109
1186	Potamanaxas unifasciata	Pyrginae	04-SRNP-364	CSRII622-05	DQ293104
1187	Potamanaxas unifasciata	Pyrginae	95-SRNP-433	CSCR210-04	DQ293100
1188	Potamanaxas unifasciata	Pyrginae	95-SRNP-437	CSCR211-04	DQ293101
1189	Potamanaxas unifasciata	Pyrginae	95-SRNP-441	CSRII751-05	DQ293115
1190	Potamanaxas unifasciata	Pyrginae	95-SRNP-436	CSRII752-05	DQ293116
1191	Potamanaxas unifasciata	Pyrginae	95-SRNP-438	CSRII753-05	DQ293117
1192	Potamanaxas unifasciata	Pyrginae	03-SRNP-4147	CSCR398-04	DQ293102
1193	Potamanaxas unifasciata	Pyrginae	03-SRNP-5425	CSCR399-04	DQ293103
1194	Potamanaxas unifasciata	Pyrginae	00-SRNP-12803	CSRII742-05	DQ293112
1195	Potamanaxas unifasciata	Pyrginae	06-SRNP-21553	MHAHH473-06	GU155463
1196	Potamanaxas unifasciata	Pyrginae	04-SRNP-2128	MHAHC135-05	DQ293119
1197	Potamanaxas unifasciata	Pyrginae	05-SRNP-35251	MHAHI646-06	GU156282
1198	Potamanaxas unifasciata	Pyrginae	08-SRNP-56559	MHMXX524-09	GU666357
1199	Milanion marciana	Pyrginae	07-SRNP-41972	MHMXN353-07	JF762339
1200	Milanion marciana	Pyrginae	07-SRNP-1128	MHAHK711-07	JF760840
1201	Milanion marciana	Pyrginae	04-SRNP-41660	MHAHC151-05	DQ292619
1202	Milanion marciana	Pyrginae	04-SRNP-42477	MHAHD810-05	GU161593
1203	Milanion marciana	Pyrginae	05-SRNP-188	MHAHD812-05	GU161592
1204	Milanion marciana	Pyrginae	01-SRNP-5343	CSCR125-04	DQ292617
1205	Milanion marciana	Pyrginae	01-SRNP-5185	CSCR124-04	DQ292616
1206	Milanion marciana	Pyrginae	04-SRNP-41204	MHAHC143-05	DQ292618
1207	Milanion marciana	Pyrginae	04-SRNP-42614	MHAHD811-05	GU161591
1208	Milanion marciana	Pyrginae	05-SRNP-41228	MHAHF292-06	GU150532
1209	Milanion marciana	Pyrginae	07-SRNP-42347	MHMXO966-08	JF762340
1210	Milanion marciana	Pyrginae	09-SRNP-42042	MHMYH162-10	HM887309
1211	Pythonides amaryllis	Pyrginae	06-SRNP-7522	MHAHJ760-07	JF753107
1212	Pythonides amaryllis	Pyrginae	07-SRNP-2293	MHAHL251-07	JF762733
1213	Pythonides amaryllis	Pyrginae	07-SRNP-45416	MHMXO951-08	JF762741
1214	Pythonides amaryllis	Pyrginae	06-SRNP-65338	MHAHJ713-07	JF753105
1215	Pythonides amaryllis	Pyrginae	07-SRNP-2936	MHAHL252-07	JF762734
1216	Pythonides amaryllis	Pyrginae	07-SRNP-65576	MHMXR836-08	JF762737
1217	Pythonides amaryllis	Pyrginae	08-SRNP-70591	MHMXW426-09	JF754123
1218	Pythonides amaryllis	Pyrginae	07-SRNP-296	MHAHK351-07	JF761083
1219	Pythonides amaryllis	Pyrginae	02-SRNP-547	CSCR218-04	DQ293156
1220	Pythonides amaryllis	Pyrginae	07-SRNP-45759	MHMXR837-08	JF762736
1221	Pythonides amaryllis	Pyrginae	07-SRNP-45350	MHMXO953-08	JF762739
1222	Pythonides amaryllis	Pyrginae	07-SRNP-41120	MHAHL254-07	JF762735
1223	Pythonides amaryllis	Pyrginae	07-SRNP-2294	MHAHL250-07	JF762732
1224	Pythonides amaryllis	Pyrginae	07-SRNP-949	MHAHK352-07	JF761084
1225	Pythonides amaryllis	Pyrginae	07-SRNP-30555	MHMXH892-07	JF761081
1226	Pythonides amaryllis	Pyrginae	07-SRNP-30884	MHMXH891-07	JF761082
1227	Pythonides amaryllis	Pyrginae	06-SRNP-65311	MHAHJ712-07	JF753104
1228	Pythonides amaryllis	Pyrginae	06-SRNP-6930	MHAHJ467-07	JF753102
1229	Pythonides amaryllis	Pyrginae	06-SRNP-9220	MHAHJ466-07	JF753101
1230	Pythonides amaryllis	Pyrginae	06-SRNP-65571	MHAHJ871-07	JF753108
1231	Pythonides amaryllis	Pyrginae	06-SRNP-7674	MHAHJ759-07	JF753106

Tree Order	Species	Subfamily	ACG Sampleid	BOLD Processid	Genbank Accession
1232	Pythonides amaryllis	Pyrginae	02-SRNP-6320	CSCR219-04	DQ293157
1233	Pythonides amaryllis	Pyrginae	07-SRNP-45758	MHMXO952-08	JF762740
1234	Pythonides amaryllis	Pyrginae	06-SRNP-9644	MHAHJ711-07	JF753103
1235	Pythonides amaryllis	Pyrginae	07-SRNP-45250	MHMXO950-08	JF762742
1236	Pythonides amaryllis	Pyrginae	07-SRNP-65279	MHMXO954-08	JF762738
1237	Pythonides amaryllis	Pyrginae	08-SRNP-70034	MHMXW427-09	JF754124
1238	Pythonides amaryllis	Pyrginae	08-SRNP-71826	MHMXW526-09	JF754122
1239	Pythonides pteras	Pyrginae	05-SRNP-31884	MHAHF172-06	GU150740
1240	Pythonides pteras	Pyrginae	05-SRNP-31882	MHAHF173-06	GU150741
1241	Pythonides pteras	Pyrginae	05-SRNP-32629	MHAHF174-06	GU150742
1242	Pythonides pteras	Pyrginae	06-SRNP-32649	MHAHI199-06	GU156288
1243	Pythonides pteras	Pyrginae	06-SRNP-32650	MHAHI200-06	GU156289
1244	Pythonides pteras	Pyrginae	07-SRNP-32651	MHAHL259-07	JF762748
1245	Morvina fissimacula pelarge	Pyrginae	07-SRNP-41267	MHAHL240-07	JF762347
1246	Morvina fissimacula pelarge	Pyrginae	06-SRNP-31970	MHAHH469-06	GU155426
1247	Morvina fissimacula pelarge	Pyrginae	06-SRNP-31945	MHAHH468-06	GU155427
1248	Morvina fissimacula pelarge	Pyrginae	06-SRNP-32336	MHAHH470-06	GU155425
1249	Morvina fissimacula pelarge	Pyrginae	02-SRNP-6631	CSCR128-04	DQ292632
1250	Morvina fissimacula pelarge	Pyrginae	02-SRNP-6922	CSCR129-04	DQ292633
1251	Morvina fissimacula pelarge	Pyrginae	06-SRNP-33952	MHAHI534-06	GU155997
1252	Morvina fissimacula pelarge	Pyrginae	06-SRNP-34246	MHAHI535-06	GU155996
1253	Morvina fissimacula pelarge	Pyrginae	06-SRNP-34680	MHAHJ832-07	JF752922
1254	Morvina fissimacula pelarge	Pyrginae	06-SRNP-65215	MHAHJ883-07	JF752923
1255	Morvina fissimacula pelarge	Pyrginae	04-SRNP-2511	MHMXY1048-09	GU666483
1256	Morvina fissimacula pelarge	Pyrginae	08-SRNP-65933	MHMXY1049-09	GU666484
1257	Ouleus cyrna	Pyrginae	03-SRNP-22100	CSRII402-04	DQ292787
1258	Ouleus cyrna	Pyrginae	00-SRNP-23507	CSRII361-04	DQ292786
1259	Ouleus cyrna	Pyrginae	03-SRNP-22212	CSRII403-04	DQ292788
1260	Ouleus cyrna	Pyrginae	06-SRNP-35224	MHAHH463-06	GU155441
1261	Zera hosta	Pyrginae	02-SRNP-24501	CSCR296-04	DQ293943
1262	Zera hosta	Pyrginae	02-SRNP-24500	CSCR295-04	DQ293942
1263	Zera hosta	Pyrginae	03-SRNP-3007	MHAHH106-06	GU155726
1264	Zera hosta	Pyrginae	03-SRNP-3008	MHAHH107-06	GU155727
1265	Zera hosta	Pyrginae	02-SRNP-24498	MHAHH109-06	GU155725
1266	Zera Burns01DHJ03	Pyrginae	07-SRNP-41866	MHAHL169-07	JF763528
1267	Zera Burns01DHJ03	Pyrginae	06-SRNP-6823	MHAHJ489-07	JF753271
1268	Zera Burns01DHJ03	Pyrginae	08-SRNP-4248	MHMXW537-09	JF754434
1269	Zera Burns01DHJ02	Pyrginae	06-SRNP-22961	MHAHJ702-07	JF753270
1270	Zera Burns01DHJ02	Pyrginae	06-SRNP-9109	MHAHJ601-07	JF753269
1271	Zera Burns01DHJ02	Pyrginae	06-SRNP-9107	MHAHJ490-07	JF753267
1272	Zera Burns01DHJ02	Pyrginae	06-SRNP-9105	MHAHJ488-07	JF753266
1273	Zera Burns01DHJ02	Pyrginae	02-SRNP-24499	MHAHH111-06	GU155728
1274	Zera Burns01DHJ02	Pyrginae	02-SRNP-24503	MHAHH110-06	GU155729
1275	Zera Burns01DHJ02	Pyrginae	02-SRNP-4228	MHAHH108-06	GU155730
1276	Zera Burns01DHJ02	Pyrginae	05-SRNP-23870	MHAHF612-06	GU151041
1277	Zera Burns01DHJ02	Pyrginae	05-SRNP-55739	MHAHF117-06	GU151040
1278	Zera Burns01DHJ02	Pyrginae	06-SRNP-9106	MHAHJ546-07	JF753268
1279	Zera Burns01DHJ02	Pyrginae	07-SRNP-32559	MHMXK033-07	JF763527
1280	Zera Burns01DHJ02	Pyrginae	07-SRNP-3659	MHMXP173-08	JF763526
1281	Zera Burns01DHJ02	Pyrginae	08-SRNP-56931	MHMXW536-09	JF754433
1282	Zera Burns01DHJ02	Pyrginae	08-SRNP-56930	MHMXX529-09	JF778649
1283	Gindanes brontinus	Pyrginae	08-SRNP-57236	MHMXX518-09	JF777978
1284	Gindanes brontinus	Pyrginae	06-SRNP-58321	MHMXH875-07	JF760738
1285	Gindanes brontinus	Pyrginae	05-SRNP-63509	MHAHF609-06	GU150454
1286	Gindanes brontinus	Pyrginae	05-SRNP-65501	MHAHF607-06	GU150452
1287	Gindanes brontinus	Pyrginae	04-SRNP-14564	MHAHF115-06	GU150450

Tree Order	Species	Subfamily	ACG Sampleid	BOLD Processid	Genbank Accession
1288	Gindanes brontinus	Pyrginae	04-SRNP-14565	MHAHD681-05	GU161532
1289	Gindanes brontinus	Pyrginae	94-SRNP-15.1	CSCR111-04	DQ292511
1290	Gindanes brontinus	Pyrginae	94-SRNP-10	CSCR110-04	DQ292510
1291	Gindanes brontinus	Pyrginae	04-SRNP-14563	MHAHD682-05	GU161533
1292	Gindanes brontinus	Pyrginae	05-SRNP-64401	MHAHF608-06	GU150451
1293	Gindanes brontinus	Pyrginae	05-SRNP-65494	MHAHF606-06	GU150453
1294	Gindanes brontinus	Pyrginae	08-SRNP-57426	MHMXX519-09	JF777979
1295	Pythonides proxenus	Pyrginae	07-SRNP-42356	MHMXO948-08	JF762747
1296	Pythonides proxenus	Pyrginae	06-SRNP-6782	MHAHJ837-07	JF753111
1297	Pythonides proxenus	Pyrginae	06-SRNP-23081	MHAHJ541-07	JF753110
1298	Pythonides proxenus	Pyrginae	04-SRNP-41615	MHAHE086-05	GU149873
1299	Pythonides proxenus	Pyrginae	04-SRNP-26794	MHAHF171-06	GU150738
1300	Pythonides proxenus	Pyrginae	02-SRNP-20155	CSCR220-04	DQ293158
1301	Pythonides proxenus	Pyrginae	02-SRNP-20156	CSCR221-04	DQ293159
1302	Pythonides proxenus	Pyrginae	07-SRNP-21605	MHAHL258-07	JF762744
1303	Pythonides proxenus	Pyrginae	07-SRNP-21699	MHAHL257-07	JF762743
1304	Pythonides proxenus	Pyrginae	08-SRNP-40390	MHMXT199-08	JF762745
1305	Pythonides proxenus	Pyrginae	09-SRNP-22673	MHMYH155-10	HM887303
1306	Pythonides proxenus	Pyrginae	04-SRNP-26795	MHAHF170-06	GU150739
1307	Pythonides proxenus	Pyrginae	06-SRNP-44676	MHAHK355-07	JF761087
1308	Pythonides proxenus	Pyrginae	07-SRNP-3103	MHMXO947-08	JF762746
1309	Pythonides proxenus	Pyrginae	06-SRNP-36323	MHMXH889-07	JF761085
1310	Pythonides proxenus	Pyrginae	06-SRNP-44677	MHAHK354-07	JF761086
1311	Pythonides proxenus	Pyrginae	06-SRNP-43506	MHAHI549-06	GU156287
1312	Pythonides proxenus	Pyrginae	06-SRNP-22943	MHAHJ540-07	JF753109
1313	Pythonides proxenus	Pyrginae	04-SRNP-60627	MHAHE085-05	GU149871
1314	Pythonides proxenus	Pyrginae	04-SRNP-45116	MHAHE087-05	GU149872
1315	Pythonides proxenus	Pyrginae	08-SRNP-1431	MHMXW431-09	JF754125
1316	Pythonides proxenus	Pyrginae	09-SRNP-20219	MHMYH154-10	HM887302
1317	Pythonides proxenus	Pyrginae	09-SRNP-20218	MHMYH156-10	HM887304
1318	Pythonides proxenus	Pyrginae	09-SRNP-20220	MHMYG2504-10	HM885933
1319	Quadrus francesius	Pyrginae	04-SRNP-3839	MHAHE089-05	GU149883
1320	Quadrus francesius	Pyrginae	04-SRNP-4110	MHAHE090-05	GU149884
1321	Quadrus francesius	Pyrginae	06-SRNP-7067	MHAHJ754-07	JF753114
1322	Quadrus francesius	Pyrginae	06-SRNP-7066	MHAHJ836-07	JF753115
1323	Quadrus francesius	Pyrginae	08-SRNP-4233	MHMXW428-09	JF754141
1324	Quadrus francesius	Pyrginae	08-SRNP-4232	MHMXW429-09	JF754142
1325	Quadrus francesius	Pyrginae	05-SRNP-5074	MHAHF321-06	GU150744
1326	Quadrus francesius	Pyrginae	05-SRNP-31504	MHAHF186-06	GU150743
1327	Quadrus francesius	Pyrginae	04-SRNP-2313	MHAHC725-05	DQ293179
1328	Quadrus francesius	Pyrginae	07-SRNP-21126	MHMXK034-07	JF762762
1329	Quadrus francesius	Pyrginae	05-SRNP-21430	MHAHF184-06	GU150745
1330	Quadrus francesius	Pyrginae	05-SRNP-60	MHAHE092-05	GU149882
1331	Quadrus francesius	Pyrginae	05-SRNP-62	MHAHE091-05	GU149885
1332	Quadrus francesius	Pyrginae	04-SRNP-4111	MHAHE088-05	GU149881
1333	Quadrus francesius	Pyrginae	05-SRNP-30292	MHAHC731-05	DQ293180
1334	Quadrus francesius	Pyrginae	04-SRNP-3262	MHAHC724-05	DQ293178
1335	Quadrus francesius	Pyrginae	03-SRNP-5537	CSCR567-04	DQ293176
1336	Quadrus francesius	Pyrginae	03-SRNP-5745	CSCR568-04	DQ293177
1337	Quadrus francesius	Pyrginae	08-SRNP-30995	MHMXW430-09	JF754143
1338	Quadrus francesius	Pyrginae	09-SRNP-80528	MHMYG2008-10	JF751939
1339	Quadrus contubernalis	Pyrginae	01-SRNP-2577	CSCR222-04	DQ293169
1340	Quadrus contubernalis	Pyrginae	07-SRNP-2968	MHAHL256-07	JF762761
1341	Quadrus contubernalis	Pyrginae	08-SRNP-794	MHMXW432-09	JF754139
1342	Quadrus contubernalis	Pyrginae	04-SRNP-3684	MHAHE084-05	GU149879
1343	Quadrus contubernalis	Pyrginae	04-SRNP-3683	MHAHE083-05	GU149880

Tree Order	Species	Subfamily	ACG Sampleid	BOLD Processid	Genbank Accession
1344	Quadrus contubernalis	Pyrginae	04-SRNP-60962	MHAHC749-05	DQ293174
1345	Quadrus contubernalis	Pyrginae	04-SRNP-4515	MHAHC746-05	DQ293173
1346	Quadrus contubernalis	Pyrginae	04-SRNP-4514	MHAHC745-05	DQ293172
1347	Quadrus contubernalis	Pyrginae	04-SRNP-3680	MHAHC744-05	DQ293171
1348	Quadrus contubernalis	Pyrginae	02-SRNP-19658	CSCR223-04	DQ293170
1349	Quadrus contubernalis	Pyrginae	04-SRNP-60961	MHAHC750-05	DQ293175
1350	Quadrus contubernalis	Pyrginae	06-SRNP-2063	MHAHG793-06	GU151594
1351	Quadrus contubernalis	Pyrginae	06-SRNP-2062	MHAHG794-06	GU151593
1352	Quadrus contubernalis	Pyrginae	06-SRNP-2192	MHAHG795-06	GU151595
1353	Quadrus contubernalis	Pyrginae	06-SRNP-3969	MHAHH411-06	GU155487
1354	Quadrus contubernalis	Pyrginae	06-SRNP-7060	MHAHJ468-07	JF753112
1355	Quadrus contubernalis	Pyrginae	06-SRNP-7061	MHAHJ755-07	JF753113
1356	Quadrus contubernalis	Pyrginae	07-SRNP-1429	MHAHK353-07	JF761089
1357	Quadrus contubernalis	Pyrginae	07-SRNP-2969	MHAHL255-07	JF762760
1358	Quadrus contubernalis	Pyrginae	08-SRNP-793	MHMXW444-09	JF754140
1359	Ouleus dilla baruDHJ01	Pyrginae	02-SRNP-29	CSRII373-04	DQ292790
1360	Ouleus dilla baruDHJ01	Pyrginae	05-SRNP-34972	MHAHF802-06	GU150637
1361	Ouleus dilla baruDHJ01	Pyrginae	06-SRNP-44270	MHAHJ534-07	JF752945
1362	Ouleus dilla baruDHJ01	Pyrginae	08-SRNP-40639	MHMXX1121-09	JF778158
1363	Ouleus dilla baruDHJ02	Pyrginae	05-SRNP-314	MHAHF097-06	GU150634
1364	Ouleus dilla baruDHJ02	Pyrginae	04-SRNP-40712	MHAHD793-05	GU161713
1365	Ouleus dilla baruDHJ02	Pyrginae	04-SRNP-4906	MHAHD792-05	GU161712
1366	Ouleus dilla baruDHJ02	Pyrginae	04-SRNP-42883	MHAHD790-05	GU161710
1367	Ouleus dilla baruDHJ02	Pyrginae	04-SRNP-3168	MHAHD788-05	GU161711
1368	Ouleus dilla baruDHJ02	Pyrginae	03-SRNP-5456	CSRII392-04	DQ292795
1369	Ouleus dilla baruDHJ02	Pyrginae	01-SRNP-25074	CSRII371-04	DQ292789
1370	Ouleus dilla baruDHJ02	Pyrginae	02-SRNP-21428	CSRII386-04	DQ292793
1371	Ouleus dilla baruDHJ02	Pyrginae	02-SRNP-18307	CSRII385-04	DQ292792
1372	Ouleus dilla baruDHJ02	Pyrginae	03-SRNP-5228	CSRII391-04	DQ292794
1373	Ouleus dilla baruDHJ02	Pyrginae	02-SRNP-1148	CSRII374-04	DQ292791
1374	Ouleus dilla baruDHJ02	Pyrginae	05-SRNP-41777	MHAHF061-06	GU150633
1375	Ouleus dilla baruDHJ02	Pyrginae	05-SRNP-30721	MHAHF096-06	GU150635
1376	Ouleus dilla baruDHJ02	Pyrginae	05-SRNP-7449	MHAHF791-06	GU150636
1377	Ouleus dilla baruDHJ02	Pyrginae	06-SRNP-31341	MHAHG753-06	GU151509
1378	Ouleus dilla baruDHJ02	Pyrginae	07-SRNP-42528	MHMXR053-08	JF762483
1379	Ouleus dilla baruDHJ02	Pyrginae	08-SRNP-72306	MHMXX590-09	JF778159
1380	Ouleus dilla baruDHJ02	Pyrginae	08-SRNP-72304	MHMXX591-09	JF778160
1381	Ouleus dilla baruDHJ02	Pyrginae	09-SRNP-80444	MHMYG2486-10	HM885913
1382	Ouleus Burns01	Pyrginae	04-SRNP-4356	MHAHD787-05	GU161709
1383	Ouleus Burns01	Pyrginae	03-SRNP-5719	CSRII393-04	DQ292785
1384	Ouleus Burns01	Pyrginae	06-SRNP-45127	MHAHG748-06	GU151508
1385	Ouleus Burns01	Pyrginae	06-SRNP-8586	MHAHJ465-07	JF752943
1386	Ouleus Burns01	Pyrginae	06-SRNP-8585	MHAHJ536-07	JF752944
1387	Ouleus negrus	Pyrginae	04-SRNP-491	MHAHI243-06	GU156032
1388	Ouleus negrus	Pyrginae	98-SRNP-6834	CSRII348-04	DQ292796
1389	Ouleus negrus	Pyrginae	02-SRNP-2323	MHAHI244-06	GU156031
1390	Ouleus negrus	Pyrginae	02-SRNP-5597	CSRII378-04	DQ292797
1391	Ouleus negrus	Pyrginae	00-SRNP-162	MHAHI246-06	GU156033
1392	Ouleus negrus	Pyrginae	03-SRNP-34272	CSRII411-04	DQ292799
1393	Ouleus negrus	Pyrginae	03-SRNP-6700	CSRII394-04	DQ292798
1394	Ouleus negrus	Pyrginae	00-SRNP-14610	MHAHI245-06	GU156034
1395	Ouleus negrus	Pyrginae	07-SRNP-2215	MHAHL150-07	JF762484
1396	Ouleus negrus	Pyrginae	07-SRNP-2470	MHAHL163-07	JF762485
1397	Quadrus cerialisDHJ01	Pyrginae	08-SRNP-65628	MHMXW441-09	JF754126
1398	Quadrus cerialisDHJ02	Pyrginae	08-SRNP-70040	MHMXW434-09	JF754127
1399	Quadrus cerialisDHJ03	Pyrginae	04-SRNP-55879	MHAHE093-05	GU149875

Tree Order	Species	Subfamily	ACG Sampleid	BOLD Processid	Genbank Accession
1400	Quadrus cerialisDHJ03	Pyrginae	04-SRNP-55680	MHAHC730-05	DQ293165
1401	Quadrus cerialisDHJ03	Pyrginae	07-SRNP-65780	MHMXR772-08	JF762753
1402	Quadrus cerialisDHJ03	Pyrginae	05-SRNP-32312	MHMXN259-07	JF762755
1403	Quadrus cerialisDHJ03	Pyrginae	05-SRNP-32317	MHMXN257-07	JF762757
1404	Quadrus cerialisDHJ03	Pyrginae	04-SRNP-49439	MHAHE097-05	GU149878
1405	Quadrus cerialisDHJ03	Pyrginae	04-SRNP-49987	MHAHC734-05	DQ293168
1406	Quadrus cerialisDHJ03	Pyrginae	04-SRNP-15284	MHAHC732-05	DQ293166
1407	Quadrus cerialisDHJ03	Pyrginae	04-SRNP-49389	MHAHC729-05	DQ293164
1408	Quadrus cerialisDHJ03	Pyrginae	04-SRNP-23614	MHAHE096-05	GU149877
1409	Quadrus cerialisDHJ03	Pyrginae	07-SRNP-66046	MHMXR773-08	JF762752
1410	Quadrus cerialisDHJ03	Pyrginae	05-SRNP-31921	MHMXN256-07	JF762758
1411	Quadrus cerialisDHJ03	Pyrginae	03-SRNP-3074	CSCR405-04	DQ293161
1412	Quadrus cerialisDHJ03	Pyrginae	08-SRNP-40247	MHMXT197-08	JF762750
1413	Quadrus cerialisDHJ03	Pyrginae	03-SRNP-3037	CSCR404-04	DQ293160
1414	Quadrus cerialisDHJ03	Pyrginae	08-SRNP-40381	MHMXT198-08	JF762749
1415	Quadrus cerialisDHJ03	Pyrginae	08-SRNP-45059	MHMXW435-09	JF754128
1416	Quadrus cerialisDHJ03	Pyrginae	08-SRNP-1036	MHMXW439-09	JF754132
1417	Quadrus cerialisDHJ03	Pyrginae	08-SRNP-70469	MHMXW440-09	JF754133
1418	Quadrus cerialisDHJ03	Pyrginae	08-SRNP-65398	MHMXW442-09	JF754134
1419	Quadrus cerialisDHJ03	Pyrginae	04-SRNP-49986	MHAHC728-05	DQ293163
1420	Quadrus cerialisDHJ03	Pyrginae	06-SRNP-36670	MHMXH890-07	JF761088
1421	Quadrus cerialisDHJ03	Pyrginae	06-SRNP-3782	MHAHH410-06	GU155486
1422	Quadrus cerialisDHJ03	Pyrginae	04-SRNP-14171	MHAHE095-05	GU149876
1423	Quadrus cerialisDHJ03	Pyrginae	04-SRNP-23615	MHAHE094-05	GU149874
1424	Quadrus cerialisDHJ03	Pyrginae	04-SRNP-15266	MHAHC733-05	DQ293167
1425	Quadrus cerialisDHJ03	Pyrginae	04-SRNP-56788	MHAHC727-05	DQ293162
1426	Quadrus cerialisDHJ03	Pyrginae	08-SRNP-65058	MHMXT196-08	JF762751
1427	Quadrus cerialisDHJ03	Pyrginae	08-SRNP-2098	MHMXW437-09	JF754130
1428	Quadrus cerialisDHJ03	Pyrginae	08-SRNP-71737	MHMXW445-09	JF754136
1429	Quadrus cerialisDHJ03	Pyrginae	08-SRNP-70012	MHMXW446-09	JF754137
1430	Quadrus cerialisDHJ03	Pyrginae	08-SRNP-1186	MHMXW447-09	JF754138
1431	Quadrus cerialisDHJ03	Pyrginae	08-SRNP-72329	MHMXY1028-09	GU666500
1432	Quadrus cerialisDHJ03	Pyrginae	09-SRNP-66024	MHMYH130-10	HM887281
1433	Quadrus cerialisDHJ03	Pyrginae	09-SRNP-75200	MHMYH131-10	HM887282
1434	Quadrus cerialisDHJ03	Pyrginae	08-SRNP-45063	MHMXW436-09	JF754129
1435	Quadrus cerialisDHJ03	Pyrginae	07-SRNP-66047	MHMXR771-08	JF762754
1436	Quadrus cerialisDHJ03	Pyrginae	05-SRNP-32596	MHMXN258-07	JF762756
1437	Quadrus cerialisDHJ03	Pyrginae	07-SRNP-42399	MHMXO949-08	JF762759
1438	Quadrus cerialisDHJ03	Pyrginae	08-SRNP-70496	MHMXW438-09	JF754131
1439	Quadrus cerialisDHJ03	Pyrginae	08-SRNP-65610	MHMXW443-09	JF754135
1440	Quadrus cerialisDHJ03	Pyrginae	08-SRNP-72231	MHMXY1026-09	GU666506
1441	Quadrus cerialisDHJ03	Pyrginae	08-SRNP-72232	MHMXY1027-09	GU666499
1442	Quadrus cerialisDHJ03	Pyrginae	09-SRNP-80394	MHMYG1993-10	JF751938
1443	Quadrus lugubris	Pyrginae	06-SRNP-55695	MHAHH815-06	GU155488
1444	Quadrus lugubris	Pyrginae	06-SRNP-59481	MHMXH880-07	JF761090
1445	Quadrus lugubris	Pyrginae	06-SRNP-18855	MHAHI556-06	GU156290
1446	Quadrus lugubris	Pyrginae	06-SRNP-20192	MHAHF763-06	GU150746
1447	Quadrus lugubris	Pyrginae	03-SRNP-3179	CSCR407-04	DQ293181
1448	Quadrus lugubris	Pyrginae	06-SRNP-46983	MHAHJ860-07	JF753116
1449	Quadrus lugubris	Pyrginae	06-SRNP-46868	MHAHJ861-07	JF753117
1450	Quadrus lugubris	Pyrginae	06-SRNP-59203	MHMXH877-07	JF761091
1451	Quadrus lugubris	Pyrginae	06-SRNP-18688	MHAHK364-07	JF761092
1452	Quadrus lugubris	Pyrginae	08-SRNP-57856	MHMXY1052-09	HM390676
1453	Ouleus salvinaDHJ02	Pyrginae	06-SRNP-59480	MHMXH885-07	JF760945
1454	Ouleus salvinaDHJ02	Pyrginae	09-SRNP-44396	MHMYE872-09	GU653731
1455	Ouleus salvinaDHJ02	Pyrginae	09-SRNP-44315	MHMYE1566-09	HM391126

Tree Order	Species	Subfamily	ACG Sampleid	BOLD Processid	Genbank Accession
1456	Ouleus salvinaDHJ02	Pyrginae	09-SRNP-68013	MHMYG2492-10	HM885920
1457	Ouleus salvinaDHJ02	Pyrginae	07-SRNP-41960	MHAHL168-07	JF762493
1458	Ouleus salvinaDHJ02	Pyrginae	09-SRNP-67763	MHMYE1551-09	HM391111
1459	Ouleus salvinaDHJ02	Pyrginae	06-SRNP-42461	MHAHI195-06	GU156036
1460	Ouleus salvinaDHJ02	Pyrginae	06-SRNP-55683	MHAHH423-06	GU155445
1461	Ouleus salvinaDHJ02	Pyrginae	04-SRNP-41470	MHAHD791-05	GU161717
1462	Ouleus salvinaDHJ02	Pyrginae	02-SRNP-14934	CSRII543-04	DQ292809
1463	Ouleus salvinaDHJ02	Pyrginae	02-SRNP-3943	CSRII375-04	DQ292800
1464	Ouleus salvinaDHJ02	Pyrginae	95-SRNP-117	CSRII538-04	DQ292805
1465	Ouleus salvinaDHJ02	Pyrginae	04-SRNP-41611	MHAHD795-05	GU161718
1466	Ouleus salvinaDHJ02	Pyrginae	05-SRNP-55605	MHAHF031-06	GU150642
1467	Ouleus salvinaDHJ02	Pyrginae	05-SRNP-55640	MHAHF064-06	GU150643
1468	Ouleus salvinaDHJ02	Pyrginae	00-SRNP-20778	MHAHH006-06	GU155447
1469	Ouleus salvinaDHJ02	Pyrginae	06-SRNP-55688	MHAHH420-06	GU155446
1470	Ouleus salvinaDHJ02	Pyrginae	07-SRNP-312	MHMXH886-07	JF760944
1471	Ouleus salvinaDHJ02	Pyrginae	07-SRNP-55498	MHAHK311-07	JF760946
1472	Ouleus salvinaDHJ02	Pyrginae	07-SRNP-55500	MHAHK313-07	JF760947
1473	Ouleus salvinaDHJ02	Pyrginae	07-SRNP-55682	MHAHK316-07	JF760948
1474	Ouleus salvinaDHJ02	Pyrginae	07-SRNP-55748	MHAHK318-07	JF760949
1475	Ouleus salvinaDHJ02	Pyrginae	07-SRNP-55742	MHAHK323-07	JF760950
1476	Ouleus salvinaDHJ02	Pyrginae	07-SRNP-55810	MHAHK325-07	JF760951
1477	Ouleus salvinaDHJ02	Pyrginae	07-SRNP-55828	MHAHK326-07	JF760952
1478	Ouleus salvinaDHJ02	Pyrginae	07-SRNP-55829	MHAHK327-07	JF760953
1479	Ouleus salvinaDHJ02	Pyrginae	09-SRNP-75766	MHMYG2494-10	HM885922
1480	Ouleus salvinaDHJ01	Pyrginae	06-SRNP-59753	MHMXH866-07	JF760939
1481	Ouleus salvinaDHJ01	Pyrginae	07-SRNP-56446	MHMXK415-07	JF762492
1482	Ouleus salvinaDHJ01	Pyrginae	07-SRNP-55582	MHAHK312-07	JF760941
1483	Ouleus salvinaDHJ01	Pyrginae	07-SRNP-55584	MHAHK308-07	JF760940
1484	Ouleus salvinaDHJ01	Pyrginae	07-SRNP-55693	MHAHK317-07	JF760943
1485	Ouleus salvinaDHJ01	Pyrginae	09-SRNP-65468	MHMYE898-09	GU653704
1486	Ouleus salvinaDHJ01	Pyrginae	07-SRNP-42350	MHMXP213-08	JF762491
1487	Ouleus salvinaDHJ01	Pyrginae	08-SRNP-70345	MHMXX1122-09	JF778161
1488	Ouleus salvinaDHJ01	Pyrginae	08-SRNP-70346	MHMXX1123-09	JF778162
1489	Ouleus salvinaDHJ01	Pyrginae	09-SRNP-70105	MHMYE897-09	GU653707
1490	Ouleus salvinaDHJ01	Pyrginae	09-SRNP-68071	MHMYG2490-10	HM885917
1491	Ouleus salvinaDHJ01	Pyrginae	06-SRNP-31160	MHAHG785-06	GU151511
1492	Ouleus salvinaDHJ01	Pyrginae	06-SRNP-55656	MHAHH419-06	GU155443
1493	Ouleus salvinaDHJ01	Pyrginae	06-SRNP-55943	MHAHH421-06	GU155444
1494	Ouleus salvinaDHJ01	Pyrginae	06-SRNP-55655	MHAHH422-06	GU155442
1495	Ouleus salvinaDHJ01	Pyrginae	09-SRNP-68307	MHMYG2491-10	HM885919
1496	Ouleus salvinaDHJ01	Pyrginae	08-SRNP-55837	MHMXX1126-09	JF778165
1497	Ouleus salvinaDHJ01	Pyrginae	08-SRNP-55820	MHMXX1125-09	JF778164
1498	Ouleus salvinaDHJ01	Pyrginae	05-SRNP-21647	MHAHF078-06	GU150640
1499	Ouleus salvinaDHJ01	Pyrginae	05-SRNP-55686	MHAHF073-06	GU150638
1500	Ouleus salvinaDHJ01	Pyrginae	09-SRNP-67934	MHMYG2489-10	HM885916
1501	Ouleus salvinaDHJ01	Pyrginae	07-SRNP-55569	MHAHK314-07	JF760942
1502	Ouleus salvinaDHJ01	Pyrginae	02-SRNP-14802	CSRII541-04	DQ292807
1503	Ouleus salvinaDHJ01	Pyrginae	09-SRNP-75801	MHMYG2493-10	HM885921
1504	Ouleus salvinaDHJ01	Pyrginae	07-SRNP-41975	MHAHL167-07	JF762488
1505	Ouleus salvinaDHJ01	Pyrginae	06-SRNP-6666	MHAHJ461-07	JF752946
1506	Ouleus salvinaDHJ01	Pyrginae	06-SRNP-42460	MHAHI194-06	GU156035
1507	Ouleus salvinaDHJ01	Pyrginae	05-SRNP-55394	MHAHF080-06	GU150641
1508	Ouleus salvinaDHJ01	Pyrginae	05-SRNP-55393	MHAHF063-06	GU150639
1509	Ouleus salvinaDHJ01	Pyrginae	05-SRNP-55399	MHAHD794-05	GU161716
1510	Ouleus salvinaDHJ01	Pyrginae	04-SRNP-41462	MHAHD789-05	GU161715
1511	Ouleus salvinaDHJ01	Pyrginae	04-SRNP-41847	MHAHD263-05	GU161714

Tree Order	Species	Subfamily	ACG Sampleid	BOLD Processid	Genbank Accession
1512	Ouleus salvinaDHJ01	Pyrginae	04-SRNP-31499	CSRII422-04	DQ292803
1513	Ouleus salvinaDHJ01	Pyrginae	02-SRNP-14703	CSRII382-04	DQ292801
1514	Ouleus salvinaDHJ01	Pyrginae	02-SRNP-14705	CSRII383-04	DQ292802
1515	Ouleus salvinaDHJ01	Pyrginae	02-SRNP-4232	CSRII540-04	DQ292806
1516	Ouleus salvinaDHJ01	Pyrginae	02-SRNP-14805	CSRII542-04	DQ292808
1517	Ouleus salvinaDHJ01	Pyrginae	03-SRNP-34140	CSRII537-04	DQ292804
1518	Ouleus salvinaDHJ01	Pyrginae	06-SRNP-31161	MHAHG774-06	GU151510
1519	Ouleus salvinaDHJ01	Pyrginae	07-SRNP-21111	MHAHL149-07	JF762486
1520	Ouleus salvinaDHJ01	Pyrginae	07-SRNP-21258	MHAHL151-07	JF762487
1521	Ouleus salvinaDHJ01	Pyrginae	07-SRNP-65146	MHAHL175-07	JF762489
1522	Ouleus salvinaDHJ01	Pyrginae	07-SRNP-65555	MHMXR054-08	JF762490
1523	Ouleus salvinaDHJ01	Pyrginae	08-SRNP-65516	MHMXX1124-09	JF778163
1524	Ouleus salvinaDHJ01	Pyrginae	08-SRNP-2541	MHMXX1187-09	JF778166
1525	Ouleus salvinaDHJ01	Pyrginae	09-SRNP-20454	MHMYG2496-10	HM885924
1526	Ouleus salvinaDHJ01	Pyrginae	09-SRNP-80738	MHMYG2009-10	JF751936
1527	Eracon lachesis	Pyrginae	05-SRNP-32007	MHAHF267-06	GU150441
1528	Eracon lachesis	Pyrginae	05-SRNP-47074	MHAHE654-06	GU149655
1529	Eracon lachesis	Pyrginae	05-SRNP-33238	MHAHI647-06	GU155964
1530	Eracon lachesis	Pyrginae	08-SRNP-40919	MHMXX907-09	JF777922
1531	Eracon lachesis	Pyrginae	07-SRNP-32478	MHMXP124-08	JF762242
1532	Eracon lachesis	Pyrginae	04-SRNP-56232	MHAHE294-05	GU149664
1533	Eracon lachesis	Pyrginae	06-SRNP-65850	MHMXK035-07	JF762244
1534	Eracon lachesis	Pyrginae	07-SRNP-45762	MHMXP123-08	JF762243
1535	Eracon lachesis	Pyrginae	09-SRNP-55623	MHMYE1498-09	GU653506
1536	Eracon lachesis	Pyrginae	09-SRNP-31097	MHMYC546-09	GU649799
1537	Eracon lachesis	Pyrginae	08-SRNP-40965	MHMXX910-09	JF777925
1538	Eracon lachesis	Pyrginae	08-SRNP-31173	MHMXX909-09	JF777924
1539	Eracon lachesis	Pyrginae	08-SRNP-40962	MHMXX908-09	JF777923
1540	Eracon lachesis	Pyrginae	07-SRNP-45763	MHMXR896-08	JF762241
1541	Eracon lachesis	Pyrginae	07-SRNP-32129	MHAHL247-07	JF762239
1542	Eracon lachesis	Pyrginae	06-SRNP-31728	MHAHH462-06	GU155402
1543	Eracon lachesis	Pyrginae	05-SRNP-31900	MHAHF268-06	GU150440
1544	Eracon lachesis	Pyrginae	05-SRNP-31902	MHAHF266-06	GU150439
1545	Eracon lachesis	Pyrginae	05-SRNP-47073	MHAHE653-06	GU149656
1546	Eracon lachesis	Pyrginae	05-SRNP-47071	MHAHE652-06	GU149657
1547	Eracon lachesis	Pyrginae	05-SRNP-47487	MHAHE651-06	GU149658
1548	Eracon lachesis	Pyrginae	05-SRNP-3998	MHAHE650-06	GU149659
1549	Eracon lachesis	Pyrginae	05-SRNP-4680	MHAHE649-06	GU149660
1550	Eracon lachesis	Pyrginae	05-SRNP-3997	MHAHE648-06	GU149661
1551	Eracon lachesis	Pyrginae	04-SRNP-55974	MHAHE295-05	GU149662
1552	Eracon lachesis	Pyrginae	04-SRNP-31099	MHAHE293-05	GU149663
1553	Eracon lachesis	Pyrginae	05-SRNP-70315	MHAHG108-06	GU151404
1554	Eracon lachesis	Pyrginae	01-SRNP-1331	CSCR106-04	DQ292502
1555	Eracon lachesis	Pyrginae	07-SRNP-46421	MHMXT162-08	JF762240
1556	Eracon lachesis	Pyrginae	09-SRNP-57098	MHMYG2435-10	HM885859
1557	Eracon lachesis	Pyrginae	09-SRNP-58020	MHMYG2436-10	HM885860
1558	Eracon lachesis	Pyrginae	09-SRNP-41521	MHMYG2437-10	HM885861
1559	Tosta niger	Pyrginae	00-SRNP-9034	CSRII359-04	DQ293610
1560	Tosta niger	Pyrginae	00-SRNP-9020	CSRII358-04	DQ293609
1561	Tosta niger	Pyrginae	04-SRNP-35560	MHAHD873-05	GU161899
1562	Tosta niger	Pyrginae	08-SRNP-36395	MHMXX586-09	JF778554
1563	Tosta niger	Pyrginae	08-SRNP-37594	MHMYC527-09	GU649812
1564	Tosta platypterus	Pyrginae	01-SRNP-25099	CSRII372-04	DQ293612
1565	Tosta platypterus	Pyrginae	04-SRNP-60213	MHAHE323-05	GU150127
1566	Tosta platypterus	Pyrginae	04-SRNP-4462	MHAHE324-05	GU150130
1567	Tosta platypterus	Pyrginae	06-SRNP-6600	MHAHJ462-07	JF753194

Tree Order	Species	Subfamily	ACG Sampleid	BOLD Processid	Genbank Accession
1568	Tosta platypterus	Pyrginae	06-SRNP-2124	MHAHG743-06	GU151746
1569	Tosta platypterus	Pyrginae	04-SRNP-4497	MHAHE326-05	GU150128
1570	Tosta platypterus	Pyrginae	04-SRNP-42429	MHAHE325-05	GU150125
1571	Tosta platypterus	Pyrginae	04-SRNP-43095	MHAHE322-05	GU150129
1572	Tosta platypterus	Pyrginae	04-SRNP-815	MHAHE123-05	GU150126
1573	Tosta platypterus	Pyrginae	01-SRNP-3610	CSRII363-04	DQ293611
1574	Tosta platypterus	Pyrginae	07-SRNP-315	MHMXK060-07	JF763271
1575	Tosta platypterus	Pyrginae	07-SRNP-41224	MHAHL237-07	JF763265
1576	Tosta platypterus	Pyrginae	07-SRNP-2877	MHAHL238-07	JF763266
1577	Tosta platypterus	Pyrginae	08-SRNP-584	MHMXX1062-09	JF778556
1578	Tosta platypterus	Pyrginae	09-SRNP-67348	MHMYE1525-09	HM391085
1579	Tosta platypterus	Pyrginae	04-SRNP-55109	MHAHD809-05	GU161901
1580	Tosta platypterus	Pyrginae	04-SRNP-55252	MHAHD808-05	GU161900
1581	Tosta platypterus	Pyrginae	07-SRNP-41435	MHAHL236-07	JF763264
1582	Tosta platypterus	Pyrginae	07-SRNP-3108	MHMXP171-08	JF763270
1583	Tosta platypterus	Pyrginae	07-SRNP-45523	MHMXR766-08	JF763269
1584	Tosta platypterus	Pyrginae	07-SRNP-46134	MHMXR778-08	JF763268
1585	Tosta platypterus	Pyrginae	07-SRNP-45525	MHMXR779-08	JF763267
1586	Tosta platypterus	Pyrginae	08-SRNP-70842	MHMXX1061-09	JF778555
1587	Tosta platypterus	Pyrginae	09-SRNP-72364	MHMYE1526-09	HM391086
1588	Tosta platypterus	Pyrginae	09-SRNP-72095	MHMYE1527-09	HM391087
1589	Iliana Burns01	Pyrginae	07-SRNP-32919	MHAHL226-07	JF762295
1590	Iliana Burns01	Pyrginae	07-SRNP-32830	MHMXP122-08	JF762296
1591	Tosta gorgus	Pyrginae	05-SRNP-64260	MHAHG776-06	GU151745
1592	Tosta gorgus	Pyrginae	05-SRNP-25010	MHAHF651-06	GU150939
1593	Tosta gorgus	Pyrginae	05-SRNP-25011	MHAHF650-06	GU150938
1594	Tosta gorgus	Pyrginae	04-SRNP-14209	MHAHD797-05	GU161897
1595	Tosta gorgus	Pyrginae	04-SRNP-16178	MHAHD796-05	GU161898
1596	Tosta gorgus	Pyrginae	00-SRNP-2772	CSCR593-04	DQ293608
1597	Tosta gorgus	Pyrginae	00-SRNP-2404	CSCR592-04	DQ293607
1598	Tosta gorgus	Pyrginae	08-SRNP-22908	MHMXX601-09	JF778553
1599	Cyclosemia Burns01	Pyrginae	02-SRNP-21532	CSCR083-04	DQ292232
1600	Cyclosemia Burns01	Pyrginae	02-SRNP-21274	CSCR082-04	DQ292231
1601	Cyclosemia Burns01	Pyrginae	08-SRNP-70951	MHMXW528-09	JF753847
1602	Cyclosemia Burns01	Pyrginae	08-SRNP-40739	MHMXW529-09	JF753848
1603	Cyclosemia Burns01	Pyrginae	07-SRNP-41474	MHAHL244-07	JF762014
1604	Cyclosemia Burns01	Pyrginae	07-SRNP-32839	MHMXR895-08	JF762015
1605	Cyclosemia Burns01	Pyrginae	07-SRNP-41339	MHAHL243-07	JF762013
1606	Cyclosemia Burns01	Pyrginae	06-SRNP-41379	MHAHH428-06	GU155341
1607	Cyclosemia Burns01	Pyrginae	05-SRNP-33219	MHAHF817-06	GU150371
1608	Cyclosemia Burns01	Pyrginae	05-SRNP-32358	MHAHF782-06	GU150369
1609	Cyclosemia Burns01	Pyrginae	05-SRNP-41022	MHAHF183-06	GU150366
1610	Cyclosemia Burns01	Pyrginae	04-SRNP-56318	MHAHF181-06	GU150367
1611	Cyclosemia Burns01	Pyrginae	05-SRNP-3974	MHAHF178-06	GU150368
1612	Cyclosemia Burns01	Pyrginae	05-SRNP-41706	MHAHF176-06	GU150370
1613	Cyclosemia Burns01	Pyrginae	04-SRNP-42740	MHAHD896-05	GU161400
1614	Cyclosemia Burns01	Pyrginae	07-SRNP-21340	MHMXK432-07	JF762017
1615	Cyclosemia Burns01	Pyrginae	07-SRNP-21976	MHAHL242-07	JF762012
1616	Cyclosemia Burns01	Pyrginae	06-SRNP-30843	MHAHG786-06	GU151338
1617	Cyclosemia Burns01	Pyrginae	07-SRNP-65003	MHAHL241-07	JF762011
1618	Cyclosemia Burns01	Pyrginae	04-SRNP-42705	MHAHD895-05	GU161401
1619	Cyclosemia Burns01	Pyrginae	04-SRNP-42378	MHAHD894-05	GU161402
1620	Cyclosemia Burns01	Pyrginae	04-SRNP-41976	MHAHD893-05	GU161395
1621	Cyclosemia Burns01	Pyrginae	04-SRNP-42926	MHAHD890-05	GU161399
1622	Cyclosemia Burns01	Pyrginae	04-SRNP-55128	MHAHD889-05	GU161394
1623	Cyclosemia Burns01	Pyrginae	04-SRNP-30480	MHAHD888-05	GU161396

Tree Order	Species	Subfamily	ACG Sampleid	BOLD Processid	Genbank Accession
1624	Cyclosemia Burns01	Pyrginae	04-SRNP-43086	MHAHD891-05	GU161397
1625	Cyclosemia Burns01	Pyrginae	04-SRNP-42679	MHAHD892-05	GU161398
1626	Cyclosemia Burns01	Pyrginae	07-SRNP-3363	MHMXP188-08	JF762016
1627	Cyclosemia Burns01	Pyrginae	08-SRNP-70207	MHMXW532-09	JF753850
1628	Cyclosemia Burns01	Pyrginae	08-SRNP-70211	MHMXW531-09	JF753849
1629	Cyclosemia Burns01	Pyrginae	08-SRNP-40741	MHMXW533-09	JF753851
1630	Cyclosemia Burns01	Pyrginae	08-SRNP-72009	MHMXX585-09	JF777825
1631	Cyclosemia Burns01	Pyrginae	09-SRNP-70459	MHMYE1500-09	GU653504
1632	Cyclosemia Burns01	Pyrginae	09-SRNP-67109	MHMYE1501-09	GU653501
1633	Cyclosemia Burns01	Pyrginae	09-SRNP-73946	MHMYG2433-10	HM885857
1634	Cyclosemia anastomosis	Pyrginae	04-SRNP-48002	MHAHE081-05	GU149575
1635	Cyclosemia anastomosis	Pyrginae	01-SRNP-464	CSCR503-04	DQ292230
1636	Cyclosemia anastomosis	Pyrginae	01-SRNP-2931	CSCR502-04	DQ292229
1637	Cyclosemia anastomosis	Pyrginae	05-SRNP-41199	MHAHF182-06	GU150361
1638	Cyclosemia anastomosis	Pyrginae	08-SRNP-70103	MHMXW530-09	JF753846
1639	Cyclosemia anastomosis	Pyrginae	08-SRNP-71654	MHMXW527-09	JF753845
1640	Cyclosemia anastomosis	Pyrginae	07-SRNP-42171	MHMXP186-08	JF762007
1641	Cyclosemia anastomosis	Pyrginae	07-SRNP-30971	MHMXH861-07	JF760639
1642	Cyclosemia anastomosis	Pyrginae	05-SRNP-43097	MHAHF784-06	GU150360
1643	Cyclosemia anastomosis	Pyrginae	05-SRNP-34289	MHAHF767-06	GU150365
1644	Cyclosemia anastomosis	Pyrginae	05-SRNP-41292	MHAHF185-06	GU150363
1645	Cyclosemia anastomosis	Pyrginae	05-SRNP-41296	MHAHF177-06	GU150362
1646	Cyclosemia anastomosis	Pyrginae	05-SRNP-45117	MHAHF175-06	GU150359
1647	Cyclosemia anastomosis	Pyrginae	04-SRNP-40770	MHAHE082-05	GU149574
1648	Cyclosemia anastomosis	Pyrginae	05-SRNP-34539	MHAHF759-06	GU150364
1649	Cyclosemia anastomosis	Pyrginae	07-SRNP-45516	MHMXP187-08	JF762006
1650	Cyclosemia anastomosis	Pyrginae	07-SRNP-45515	MHMXP183-08	JF762010
1651	Cyclosemia anastomosis	Pyrginae	07-SRNP-45573	MHMXP184-08	JF762009
1652	Cyclosemia anastomosis	Pyrginae	06-SRNP-43745	MHAHJ487-07	JF752586
1653	Cyclosemia anastomosis	Pyrginae	06-SRNP-42938	MHAHI536-06	GU155900
1654	Cyclosemia anastomosis	Pyrginae	06-SRNP-33946	MHAHI568-06	GU155901
1655	Cyclosemia anastomosis	Pyrginae	07-SRNP-2044	MHAHL239-07	JF762004
1656	Cyclosemia anastomosis	Pyrginae	07-SRNP-45517	MHMXP185-08	JF762008
1657	Cyclosemia anastomosis	Pyrginae	07-SRNP-46695	MHMXT163-08	JF762005
1658	Cyclosemia anastomosis	Pyrginae	08-SRNP-72776	MHMXY1047-09	GU666490
1659	Cyclosemia anastomosis	Pyrginae	05-SRNP-43883	MHAHG787-06	GU151337
1660	Cyclosemia anastomosis	Pyrginae	09-SRNP-33098	MHMYG2434-10	HM885858
1661	Cyclosemia subcaerulea	Pyrginae	06-SRNP-42573	MHAHI573-06	GU155903
1662	Cyclosemia subcaerulea	Pyrginae	02-SRNP-6718	CSCR084-04	DQ292233
1663	Cyclosemia subcaerulea	Pyrginae	02-SRNP-7780	CSCR085-04	DQ292234
1664	Cyclosemia subcaerulea	Pyrginae	06-SRNP-42489	MHAHI569-06	GU155902
1665	Cyclosemia subcaerulea	Pyrginae	07-SRNP-65063	MHAHL245-07	JF762018
1666	Cyclosemia subcaerulea	Pyrginae	09-SRNP-69386	MHMYE1499-09	GU653503
1667	Pellicia Janzen01	Pyrginae	05-SRNP-34546	MHAHF721-06	GU150676
1668	Pellicia Janzen01	Pyrginae	08-SRNP-2348	MHMXS084-08	JF762546
1669	Pellicia dimidiata	Pyrginae	06-SRNP-30813	MHAHG241-06	GU151544
1670	Pellicia dimidiata	Pyrginae	93-SRNP-6528	CSCR179-04	DQ292840
1671	Pellicia dimidiata	Pyrginae	06-SRNP-4815	MHAHI186-06	GU156074
1672	Pellicia dimidiata	Pyrginae	06-SRNP-3027	MHAHG780-06	GU151540
1673	Pellicia dimidiata	Pyrginae	06-SRNP-1886	MHAHG779-06	GU151541
1674	Pellicia dimidiata	Pyrginae	06-SRNP-2646	MHAHG778-06	GU151542
1675	Pellicia dimidiata	Pyrginae	06-SRNP-2668	MHAHG777-06	GU151543
1676	Pellicia dimidiata	Pyrginae	05-SRNP-42242	MHAHF103-06	GU150675
1677	Pellicia dimidiata	Pyrginae	04-SRNP-46357	MHAHD806-05	GU161781
1678	Pellicia dimidiata	Pyrginae	02-SRNP-4585	MHAHG923-06	GU151545
1679	Pellicia dimidiata	Pyrginae	93-SRNP-8674	CSCR180-04	DQ292841

Tree Order	Species	Subfamily	ACG Sampleid	BOLD Processid	Genbank Accession
1680	Pellicia dimidiata	Pyrginae	07-SRNP-45157	MHAHK315-07	JF760999
1681	Pellicia dimidiata	Pyrginae	07-SRNP-1514	MHAHK324-07	JF761000
1682	Pellicia dimidiata	Pyrginae	07-SRNP-2217	MHAHL152-07	JF762545
1683	Pellicia dimidiata	Pyrginae	08-SRNP-2140	MHMXX1101-09	JF778310
1684	Pellicia arina	Pyrginae	04-SRNP-13705	MHAHD802-05	GU161779
1685	Pellicia arina	Pyrginae	03-SRNP-12773.1	MHAHI239-06	GU156072
1686	Pellicia arina	Pyrginae	00-SRNP-4592	MHAHI240-06	GU156073
1687	Pellicia arina	Pyrginae	02-SRNP-32880	MHAHI241-06	GU156071
1688	Pellicia arina	Pyrginae	04-SRNP-15702	MHAHD803-05	GU161780
1689	Pellicia arina	Pyrginae	94-SRNP-139	CSCR177-04	DQ292839
1690	Pellicia arina	Pyrginae	05-SRNP-65245	MHAHF652-06	GU150672
1691	Pellicia arina	Pyrginae	05-SRNP-61204	MHAHF678-06	GU150674
1692	Pellicia arina	Pyrginae	05-SRNP-62646	MHAHF679-06	GU150673
1693	Pellicia arina	Pyrginae	06-SRNP-57936	MHAHJ830-07	JF752978
1694	Pellicia arina	Pyrginae	06-SRNP-19742	MHAHK321-07	JF760998
1695	Pellicia arina	Pyrginae	07-SRNP-14674	MHMXX716-09	JF778309
1696	Mictris crispus caerula	Pyrginae	03-SRNP-18922	CSCR546-04	DQ292614
1697	Mictris crispus caerula	Pyrginae	05-SRNP-4397	MHAHF854-06	GU150529
1698	Mictris crispus caerula	Pyrginae	05-SRNP-4032	MHAHF856-06	GU150528
1699	Mictris crispus caerula	Pyrginae	05-SRNP-3008	MHAHF855-06	GU150530
1700	Mictris crispus caerula	Pyrginae	05-SRNP-34281	MHAHF713-06	GU150531
1701	Mictris crispus caerula	Pyrginae	07-SRNP-3356	MHMXP176-08	JF762337
1702	Mictris crispus caerula	Pyrginae	08-SRNP-40396	MHMXT164-08	JF762333
1703	Mictris crispus caerula	Pyrginae	09-SRNP-71308	MHMYE1496-09	GU653508
1704	Mictris crispus caerula	Pyrginae	07-SRNP-33227	MHMXR780-08	JF762336
1705	Mictris crispus caerula	Pyrginae	07-SRNP-32363	MHAHL230-07	JF762332
1706	Mictris crispus caerula	Pyrginae	07-SRNP-31656	MHMXK036-07	JF762338
1707	Mictris crispus caerula	Pyrginae	07-SRNP-31094	MHMXH859-07	JF760839
1708	Mictris crispus caerula	Pyrginae	05-SRNP-34280	MHAHG257-06	GU151456
1709	Mictris crispus caerula	Pyrginae	05-SRNP-2161	MHAHF857-06	GU150527
1710	Mictris crispus caerula	Pyrginae	04-SRNP-4244	MHAHD872-05	GU161590
1711	Mictris crispus caerula	Pyrginae	03-SRNP-6065	CSCR547-04	DQ292615
1712	Mictris crispus caerula	Pyrginae	03-SRNP-9772	CSCR385-04	DQ292613
1713	Mictris crispus caerula	Pyrginae	05-SRNP-34282	MHAHG256-06	GU151457
1714	Mictris crispus caerula	Pyrginae	05-SRNP-34208	MHAHF720-06	GU150526
1715	Mictris crispus caerula	Pyrginae	08-SRNP-195	MHMXT150-08	JF762335
1716	Mictris crispus caerula	Pyrginae	08-SRNP-196	MHMXT151-08	JF762334
1717	Mictris crispus caerula	Pyrginae	08-SRNP-31176	MHMXX905-09	JF778083
1718	Mictris crispus caerula	Pyrginae	08-SRNP-2184	MHMXX906-09	JF778084
1719	Mictris crispus caerula	Pyrginae	09-SRNP-71399	MHMYE1497-09	GU653505
1720	Pachyneuria licisca	Pyrginae	05-SRNP-58420	MHAHF098-06	GU150647
1721	Pachyneuria licisca	Pyrginae	03-SRNP-30838	MHAHI321-06	GU156038
1722	Pachyneuria licisca	Pyrginae	07-SRNP-57201	MHAHL166-07	JF762504
1723	Pachyneuria licisca	Pyrginae	03-SRNP-27023	MHAHI323-06	GU156040
1724	Pachyneuria licisca	Pyrginae	03-SRNP-27271	MHAHI330-06	GU156046
1725	Pachyneuria licisca	Pyrginae	03-SRNP-27670	MHAHI329-06	GU156045
1726	Pachyneuria licisca	Pyrginae	03-SRNP-30847	MHAHI328-06	GU156044
1727	Pachyneuria licisca	Pyrginae	03-SRNP-27200	MHAHI326-06	GU156042
1728	Pachyneuria licisca	Pyrginae	03-SRNP-27033	MHAHI325-06	GU156041
1729	Pachyneuria licisca	Pyrginae	03-SRNP-27671	MHAHI324-06	GU156037
1730	Pachyneuria licisca	Pyrginae	03-SRNP-27754	MHAHI322-06	GU156039
1731	Pachyneuria licisca	Pyrginae	03-SRNP-27025	MHAHI327-06	GU156043
1732	Pachyneuria licisca	Pyrginae	97-SRNP-5856	CSCR174-04	DQ292823
1733	Pachyneuria licisca	Pyrginae	02-SRNP-5984	CSCR559-04	DQ292824
1734	Pachyneuria licisca	Pyrginae	06-SRNP-58286	MHAHJ463-07	JF752959
1735	Pachyneuria licisca	Pyrginae	06-SRNP-58263	MHAHJ692-07	JF752960

Tree Order	Species	Subfamily	ACG Sampleid	BOLD Processid	Genbank Accession
1736	Pachyneuria licisca	Pyrginae	06-SRNP-67766	MHAHK320-07	JF760955
1737	Pachyneuria licisca	Pyrginae	09-SRNP-57271	MHMYE1530-09	HM391090
1738	Pachyneuria licisca	Pyrginae	09-SRNP-57272	MHMYE1531-09	HM391091
1739	Pachyneuria licisca	Pyrginae	09-SRNP-57273	MHMYG2476-10	HM885903
1740	Nisoniades castolus	Pyrginae	05-SRNP-6591	MHAHF800-06	GU150623
1741	Nisoniades castolus	Pyrginae	06-SRNP-32805	MHAHI091-06	GU156021
1742	Nisoniades castolus	Pyrginae	05-SRNP-45293	MHAHF068-06	GU150619
1743	Nisoniades castolus	Pyrginae	05-SRNP-31414	MHAHF079-06	GU150622
1744	Nisoniades castolus	Pyrginae	05-SRNP-1751	MHAHF067-06	GU150620
1745	Nisoniades castolus	Pyrginae	07-SRNP-41530	MHAHL155-07	JF762446
1746	Nisoniades castolus	Pyrginae	05-SRNP-31711	MHAHF028-06	GU150618
1747	Nisoniades castolus	Pyrginae	04-SRNP-41670	MHAHD786-05	GU161697
1748	Nisoniades castolus	Pyrginae	04-SRNP-34843	MHAHD785-05	GU161696
1749	Nisoniades castolus	Pyrginae	04-SRNP-56347	MHAHD783-05	GU161693
1750	Nisoniades castolus	Pyrginae	04-SRNP-60852	MHAHD782-05	GU161695
1751	Nisoniades castolus	Pyrginae	05-SRNP-2312	MHAHF070-06	GU150617
1752	Nisoniades castolus	Pyrginae	04-SRNP-55872	MHAHD784-05	GU161694
1753	Nisoniades castolus	Pyrginae	05-SRNP-41450	MHAHF076-06	GU150621
1754	Nisoniades castolus	Pyrginae	05-SRNP-41083	MHAHF072-06	GU150616
1755	Nisoniades castolus	Pyrginae	06-SRNP-32630	MHAHI190-06	GU156020
1756	Nisoniades castolus	Pyrginae	07-SRNP-65198	MHAHL174-07	JF762447
1757	Nisoniades castolus	Pyrginae	07-SRNP-65620	MHMXR049-08	JF762448
1758	Nisoniades castolus	Pyrginae	08-SRNP-2616	MHMXX1071-09	JF778137
1759	Nisoniades castolus	Pyrginae	08-SRNP-70988	MHMXX1072-09	JF778138
1760	Nisoniades castolus	Pyrginae	08-SRNP-70583	MHMXX1073-09	JF778139
1761	Nisoniades castolus	Pyrginae	08-SRNP-65475	MHMXX1074-09	JF778140
1762	Nisoniades castolus	Pyrginae	08-SRNP-65509	MHMXX1075-09	JF778141
1763	Nisoniades castolus	Pyrginae	08-SRNP-70402	MHMXX1076-09	JF778142
1764	Nisoniades castolus	Pyrginae	08-SRNP-2617	MHMXX1077-09	JF778143
1765	Nisoniades castolus	Pyrginae	09-SRNP-71609	MHMYE1550-09	HM391110
1766	Nisoniades godma	Pyrginae	07-SRNP-21783	MHAHL173-07	JF762454
1767	Nisoniades godma	Pyrginae	08-SRNP-2515	MHMXX1070-09	JF778148
1768	Nisoniades godma	Pyrginae	09-SRNP-67771	MHMYE1528-09	HM391088
1769	Nisoniades godma	Pyrginae	08-SRNP-35368	MHMXX1067-09	JF778146
1770	Nisoniades godma	Pyrginae	08-SRNP-20591	MHMXX1069-09	JF778147
1771	Nisoniades godma	Pyrginae	08-SRNP-70907	MHMXX1064-09	JF778144
1772	Nisoniades godma	Pyrginae	08-SRNP-35367	MHMXX1066-09	JF778145
1773	Nisoniades godma	Pyrginae	07-SRNP-41906	MHAHL170-07	JF762453
1774	Nisoniades godma	Pyrginae	07-SRNP-22159	MHAHL234-07	JF762455
1775	Nisoniades godma	Pyrginae	07-SRNP-2603	MHAHL160-07	JF762451
1776	Nisoniades godma	Pyrginae	07-SRNP-21785	MHAHL164-07	JF762452
1777	Nisoniades godma	Pyrginae	07-SRNP-2675	MHAHL157-07	JF762449
1778	Nisoniades godma	Pyrginae	07-SRNP-3043	MHAHL158-07	JF762450
1779	Nisoniades godma	Pyrginae	06-SRNP-60085	MHMXH863-07	JF760896
1780	Nisoniades godma	Pyrginae	06-SRNP-60084	MHMXH882-07	JF760898
1781	Nisoniades godma	Pyrginae	98-SRNP-4340	MHAHI339-06	GU156026
1782	Nisoniades godma	Pyrginae	07-SRNP-35261	MHMXH862-07	JF760897
1783	Nisoniades godma	Pyrginae	98-SRNP-4343	MHAHI337-06	GU156024
1784	Nisoniades godma	Pyrginae	98-SRNP-4337	MHAHI338-06	GU156025
1785	Nisoniades godma	Pyrginae	05-SRNP-30669	MHAHF071-06	GU150624
1786	Nisoniades godma	Pyrginae	98-SRNP-4617	MHAHI336-06	GU156023
1787	Nisoniades godma	Pyrginae	07-SRNP-65813	MHMXR048-08	JF762457
1788	Nisoniades godma	Pyrginae	07-SRNP-65579	MHMXR051-08	JF762456
1789	Nisoniades godma	Pyrginae	07-SRNP-55229	MHAHK297-07	JF760900
1790	Nisoniades godma	Pyrginae	07-SRNP-55227	MHAHK298-07	JF760901
1791	Nisoniades godma	Pyrginae	02-SRNP-5568	MHAHE137-05	GU149768

Tree Order	Species	Subfamily	ACG Sampleid	BOLD Processid	Genbank Accession
1792	Nisoniades godma	Pyrginae	02-SRNP-5569	MHAHE136-05	GU149770
1793	Nisoniades godma	Pyrginae	02-SRNP-6253	MHAHE135-05	GU149774
1794	Nisoniades godma	Pyrginae	02-SRNP-7146	MHAHE134-05	GU149772
1795	Nisoniades godma	Pyrginae	02-SRNP-5582	MHAHE133-05	GU149773
1796	Nisoniades godma	Pyrginae	02-SRNP-5571	MHAHE131-05	GU149769
1797	Nisoniades godma	Pyrginae	04-SRNP-47998	MHAHD801-05	GU161698
1798	Nisoniades godma	Pyrginae	04-SRNP-47987	MHAHD800-05	GU161699
1799	Nisoniades godma	Pyrginae	04-SRNP-23805	MHAHD799-05	GU161700
1800	Nisoniades godma	Pyrginae	07-SRNP-55730	MHAHK328-07	JF760902
1801	Nisoniades godma	Pyrginae	07-SRNP-21248	MHAHK289-07	JF760899
1802	Nisoniades godma	Pyrginae	05-SRNP-34138	MHAHF671-06	GU150626
1803	Nisoniades godma	Pyrginae	02-SRNP-5578	MHAHE132-05	GU149771
1804	Nisoniades godma	Pyrginae	03-SRNP-1412	CSCR562-04	DQ293125
1805	Nisoniades godma	Pyrginae	02-SRNP-18975	MHAHI238-06	GU156022
1806	Nisoniades godma	Pyrginae	06-SRNP-31175	MHAHG784-06	GU151498
1807	Nisoniades godma	Pyrginae	05-SRNP-59705	MHAHF077-06	GU150625
1808	Nisoniades godma	Pyrginae	08-SRNP-72371	MHMXX582-09	JF778149
1809	Nisoniades godma	Pyrginae	09-SRNP-67700	MHMYE1529-09	HM391089
1810	Nisoniades godma	Pyrginae	09-SRNP-75599	MHMYG2477-10	HM885904
1811	Nisoniades godma	Pyrginae	09-SRNP-75202	MHMYG2478-10	HM885905
1812	Nisoniades Burns02	Pyrginae	05-SRNP-24032	MHAHG033-06	GU151496
1813	Nisoniades Burns02	Pyrginae	01-SRNP-14613	MHAHI335-06	GU156019
1814	Nisoniades tortaDHJ01	Pyrginae	08-SRNP-2752	MHMXX1065-09	JF778150
1815	Nisoniades tortaDHJ02	Pyrginae	08-SRNP-5186	MHMXX583-09	JF778151
1816	Nisoniades tortaDHJ02	Pyrginae	06-SRNP-659	MHAHG231-06	GU151497
1817	Nisoniades tortaDHJ02	Pyrginae	09-SRNP-3730	MHMYG2479-10	HM885906
1818	Nisoniades rubescensDHJ01	Pyrginae	01-SRNP-16913	MHAHI334-06	GU156014
1819	Nisoniades rubescensDHJ02	Pyrginae	99-SRNP-2497	MHAHI332-06	GU156017
1820	Nisoniades rubescensDHJ02	Pyrginae	99-SRNP-2290	MHAHI333-06	GU156018
1821	Nisoniades rubescensDHJ02	Pyrginae	01-SRNP-9028	MHAHI331-06	GU156016
1822	Nisoniades rubescensDHJ02	Pyrginae	06-SRNP-4756	MHAHI179-06	GU156015
1823	Nisoniades rubescensDHJ02	Pyrginae	05-SRNP-41322	MHAHF099-06	GU150614
1824	Nisoniades rubescensDHJ02	Pyrginae	04-SRNP-22441	MHAHD807-05	GU161690
1825	Nisoniades rubescensDHJ02	Pyrginae	04-SRNP-48521	MHAHD805-05	GU161692
1826	Nisoniades rubescensDHJ02	Pyrginae	04-SRNP-22824	MHAHD804-05	GU161691
1827	Nisoniades rubescensDHJ02	Pyrginae	05-SRNP-61949	MHAHF686-06	GU150615
1828	Nisoniades rubescensDHJ02	Pyrginae	08-SRNP-65094	MHMXT165-08	JF762458
1829	Nisoniades rubescensDHJ02	Pyrginae	09-SRNP-75982	MHMYG2495-10	HM885923
1830	Polyctor enops	Pyrginae	06-SRNP-23424	MHAHK365-07	JF761042
1831	Polyctor enops	Pyrginae	07-SRNP-20052	MHAHK366-07	JF761043
1832	Polyctor enops	Pyrginae	06-SRNP-23355	MHAHJ551-07	JF753070
1833	Polyctor enops	Pyrginae	06-SRNP-23356	MHAHJ550-07	JF753069
1834	Polyctor enops	Pyrginae	06-SRNP-23354	MHAHJ549-07	JF753068
1835	Polyctor enops	Pyrginae	05-SRNP-24534	MHAHF636-06	GU150696
1836	Polyctor enops	Pyrginae	05-SRNP-24533	MHAHF635-06	GU150694
1837	Polyctor enops	Pyrginae	05-SRNP-24532	MHAHF637-06	GU150695
1838	Polyctor enops	Pyrginae	07-SRNP-23555	MHMXR021-08	JF762623
1839	Polyctor enops	Pyrginae	07-SRNP-23554	MHMXR020-08	JF762624
1840	Polyctor enops	Pyrginae	07-SRNP-23640	MHMXR019-08	JF762625
1841	Polyctor enops	Pyrginae	07-SRNP-23639	MHMXR018-08	JF762626
1842	Polyctor enops	Pyrginae	07-SRNP-22844	MHMXR017-08	JF762627
1843	Polyctor enops	Pyrginae	07-SRNP-23549	MHMXR016-08	JF762628
1844	Polyctor enops	Pyrginae	07-SRNP-22845	MHMXR015-08	JF762629
1845	Polyctor enops	Pyrginae	07-SRNP-23636	MHMXR014-08	JF762630
1846	Polyctor enops	Pyrginae	07-SRNP-23542	MHMXR013-08	JF762631
1847	Polyctor enops	Pyrginae	07-SRNP-23544	MHMXR012-08	JF762632

Tree Order	Species	Subfamily	ACG Sampleid	BOLD Processid	Genbank Accession
1848	Polyctor enops	Pyrginae	07-SRNP-23548	MHMXO985-08	JF762635
1849	Polyctor enops	Pyrginae	07-SRNP-23551	MHMXO984-08	JF762636
1850	Polyctor enops	Pyrginae	07-SRNP-23553	MHMXO983-08	JF762637
1851	Polyctor enops	Pyrginae	07-SRNP-23552	MHMXO986-08	JF762634
1852	Polyctor enops	Pyrginae	07-SRNP-23550	MHMXO987-08	JF762633
1853	Polyctor enops	Pyrginae	02-SRNP-30383	CSCR204-04	DQ293023
1854	Polyctor enops	Pyrginae	02-SRNP-30386	CSCR205-04	DQ293024
1855	Polyctor enops	Pyrginae	07-SRNP-23545	MHMXT190-08	JF762622
1856	Polyctor enops	Pyrginae	09-SRNP-23331	MHMYG2013-10	HM885415
1857	Polyctor polyctor	Pyrginae	09-SRNP-20156	MHMYB139-09	
1858	Polyctor polyctor	Pyrginae	03-SRNP-21493	CSRII277-04	DQ293036
1859	Polyctor polyctor	Pyrginae	03-SRNP-29770	CSRII279-04	DQ293037
1860	Polyctor cleta	Pyrginae	04-SRNP-16175	MHAHE054-05	GU149840
1861	Polyctor cleta	Pyrginae	04-SRNP-15360	MHAHC741-05	DQ293020
1862	Polyctor cleta	Pyrginae	04-SRNP-15455	MHAHC740-05	DQ293019
1863	Polyctor cleta	Pyrginae	04-SRNP-16111	MHAHC739-05	DQ293018
1864	Polyctor cleta	Pyrginae	95-SRNP-9502	CSRII264-04	DQ293008
1865	Polyctor cleta	Pyrginae	03-SRNP-37549	CSRII259-04	DQ293003
1866	Polyctor cleta	Pyrginae	03-SRNP-30883	CSRII258-04	DQ293002
1867	Polyctor cleta	Pyrginae	98-SRNP-4183	CSRII265-04	DQ293009
1868	Polyctor cleta	Pyrginae	00-SRNP-20253	CSRII266-04	DQ293010
1869	Polyctor cleta	Pyrginae	00-SRNP-2150	CSRII267-04	DQ293011
1870	Polyctor cleta	Pyrginae	03-SRNP-30880	CSRII256-04	DQ293000
1871	Polyctor cleta	Pyrginae	03-SRNP-30882	CSRII257-04	DQ293001
1872	Polyctor cleta	Pyrginae	02-SRNP-32285	CSRII269-04	DQ293013
1873	Polyctor cleta	Pyrginae	02-SRNP-31414	CSRII268-04	DQ293012
1874	Polyctor cleta	Pyrginae	03-SRNP-37550	CSRII260-04	DQ293004
1875	Polyctor cleta	Pyrginae	93-SRNP-5853	CSRII262-04	DQ293006
1876	Polyctor cleta	Pyrginae	95-SRNP-9500	CSRII263-04	DQ293007
1877	Polyctor cleta	Pyrginae	05-SRNP-61212	MHAHF701-06	GU150693
1878	Polyctor polyctor	Pyrginae	02-SRNP-27779	CSCR207-04	DQ293027
1879	Polyctor cleta	Pyrginae	92-SRNP-4986	CSRII261-04	DQ293005
1880	Polyctor polyctor	Pyrginae	05-SRNP-41712	MHAHF278-06	GU150701
1881	Polyctor polyctor	Pyrginae	07-SRNP-21961	MHAHL200-07	JF762640
1882	Polyctor polyctor	Pyrginae	03-SRNP-29891	CSRII280-04	DQ293038
1883	Polyctor polyctor	Pyrginae	00-SRNP-14180	CSRII283-04	DQ293041
1884	Polyctor polyctor	Pyrginae	03-SRNP-20449	CSRII255-04	DQ293028
1885	Polyctor polyctor	Pyrginae	02-SRNP-7128	CSRII285-04	DQ293043
1886	Polyctor polyctor	Pyrginae	80-SRNP-364	CSRII281-04	DQ293039
1887	Polyctor polyctor	Pyrginae	05-SRNP-42248	MHAHF276-06	GU150698
1888	Polyctor polyctor	Pyrginae	03-SRNP-21456	CSRII276-04	DQ293035
1889	Polyctor polyctor	Pyrginae	05-SRNP-6247	MHAHF702-06	GU150704
1890	Polyctor polyctor	Pyrginae	05-SRNP-1141	MHAHF277-06	GU150700
1891	Polyctor polyctor	Pyrginae	05-SRNP-33391	MHAHF700-06	GU150705
1892	Polyctor polyctor	Pyrginae	03-SRNP-9639	CSRII270-04	DQ293029
1893	Polyctor polyctor	Pyrginae	03-SRNP-20028	CSRII275-04	DQ293034
1894	Polyctor polyctor	Pyrginae	05-SRNP-41517	MHAHF274-06	GU150697
1895	Polyctor polyctor	Pyrginae	08-SRNP-4909	MHMXX512-09	JF778400
1896	Polyctor polyctor	Pyrginae	08-SRNP-4993	MHMXX511-09	JF778399
1897	Polyctor polyctor	Pyrginae	08-SRNP-4991	MHMXX510-09	JF778398
1898	Polyctor polyctor	Pyrginae	08-SRNP-5183	MHMXX509-09	JF778397
1899	Polyctor polyctor	Pyrginae	08-SRNP-5182	MHMXX508-09	JF778396
1900	Polyctor polyctor	Pyrginae	08-SRNP-5181	MHMXX507-09	JF778395
1901	Polyctor polyctor	Pyrginae	08-SRNP-5273	MHMXX506-09	JF778394
1902	Polyctor polyctor	Pyrginae	08-SRNP-4906	MHMXX505-09	JF778393
1903	Polyctor polyctor	Pyrginae	08-SRNP-4903	MHMXX504-09	JF778392

Tree Order	Species	Subfamily	ACG Sampleid	BOLD Processid	Genbank Accession
1904	Polyctor polyctor	Pyrginae	08-SRNP-4905	MHMXX503-09	JF778391
1905	Polyctor polyctor	Pyrginae	08-SRNP-4902	MHMXX502-09	JF778390
1906	Polyctor polyctor	Pyrginae	08-SRNP-4904	MHMXX501-09	JF778389
1907	Polyctor polyctor	Pyrginae	08-SRNP-4996	MHMXX500-09	GU666356
1908	Polyctor polyctor	Pyrginae	08-SRNP-20490	MHMXX885-09	JF778402
1909	Polyctor polyctor	Pyrginae	07-SRNP-2990	MHAHL202-07	JF762642
1910	Polyctor polyctor	Pyrginae	07-SRNP-21952	MHAHL201-07	JF762641
1911	Polyctor polyctor	Pyrginae	07-SRNP-21283	MHAHK705-07	JF761044
1912	Polyctor polyctor	Pyrginae	06-SRNP-31806	MHAHH793-06	GU155453
1913	Polyctor polyctor	Pyrginae	06-SRNP-31800	MHAHH792-06	GU155452
1914	Polyctor polyctor	Pyrginae	05-SRNP-41516	MHAHF279-06	GU150699
1915	Polyctor polyctor	Pyrginae	05-SRNP-42247	MHAHF275-06	GU150702
1916	Polyctor polyctor	Pyrginae	04-SRNP-21386	MHAHE061-05	GU149847
1917	Polyctor polyctor	Pyrginae	04-SRNP-61064	MHAHE060-05	GU149842
1918	Polyctor polyctor	Pyrginae	04-SRNP-21385	MHAHE059-05	GU149843
1919	Polyctor polyctor	Pyrginae	04-SRNP-23706	MHAHE058-05	GU149844
1920	Polyctor polyctor	Pyrginae	04-SRNP-61026	MHAHE056-05	GU149846
1921	Polyctor polyctor	Pyrginae	04-SRNP-60859	MHAHC743-05	DQ293022
1922	Polyctor polyctor	Pyrginae	04-SRNP-60861	MHAHC742-05	DQ293021
1923	Polyctor polyctor	Pyrginae	04-SRNP-14595	MHAHC738-05	DQ293017
1924	Polyctor polyctor	Pyrginae	04-SRNP-42603	MHAHC737-05	DQ293016
1925	Polyctor polyctor	Pyrginae	04-SRNP-61027	MHAHC736-05	DQ293015
1926	Polyctor polyctor	Pyrginae	04-SRNP-14594	MHAHC735-05	DQ293014
1927	Polyctor polyctor	Pyrginae	03-SRNP-9643	CSRII272-04	DQ293031
1928	Polyctor polyctor	Pyrginae	03-SRNP-9706	CSRII274-04	DQ293033
1929	Polyctor polyctor	Pyrginae	02-SRNP-7127	CSRII284-04	DQ293042
1930	Polyctor polyctor	Pyrginae	03-SRNP-9701	CSRII273-04	DQ293032
1931	Polyctor polyctor	Pyrginae	03-SRNP-9641	CSRII271-04	DQ293030
1932	Polyctor polyctor	Pyrginae	95-SRNP-9527	CSRII282-04	DQ293040
1933	Polyctor polyctor	Pyrginae	02-SRNP-31973	CSCR203-04	DQ293025
1934	Polyctor polyctor	Pyrginae	07-SRNP-24016	MHMXT099-08	JF762650
1935	Polyctor polyctor	Pyrginae	07-SRNP-20959	MHAHL199-07	JF762639
1936	Polyctor polyctor	Pyrginae	08-SRNP-355	MHMXT097-08	JF762652
1937	Polyctor polyctor	Pyrginae	08-SRNP-356	MHMXT100-08	JF762649
1938	Polyctor polyctor	Pyrginae	08-SRNP-358	MHMXT107-08	JF762653
1939	Polyctor polyctor	Pyrginae	02-SRNP-7132	CSCR206-04	DQ293026
1940	Polyctor polyctor	Pyrginae	04-SRNP-61128	MHAHE057-05	GU149845
1941	Polyctor polyctor	Pyrginae	04-SRNP-56096	MHAHE055-05	GU149841
1942	Polyctor polyctor	Pyrginae	05-SRNP-34721	MHAHF704-06	GU150703
1943	Polyctor polyctor	Pyrginae	08-SRNP-350	MHMXT101-08	JF762648
1944	Polyctor polyctor	Pyrginae	08-SRNP-499	MHMXT102-08	JF762647
1945	Polyctor polyctor	Pyrginae	08-SRNP-501	MHMXT103-08	JF762646
1946	Polyctor polyctor	Pyrginae	08-SRNP-500	MHMXT104-08	JF762645
1947	Polyctor polyctor	Pyrginae	08-SRNP-359	MHMXT106-08	JF762643
1948	Polyctor polyctor	Pyrginae	08-SRNP-22736	MHMXY1046-09	GU666489
1949	Polyctor polyctor	Pyrginae	09-SRNP-20479	MHMYG2374-10	HM885797
1950	Polyctor polyctor	Pyrginae	09-SRNP-20478	MHMYG2375-10	HM885798
1951	Polyctor polyctor	Pyrginae	09-SRNP-20485	MHMYG2376-10	HM885799
1952	Polyctor polyctor	Pyrginae	09-SRNP-56143	MHMYG2505-10	HM885934
1953	Polyctor polyctor	Pyrginae	09-SRNP-20724	MHMYH190-10	HM887337
1954	Polyctor polyctor	Pyrginae	08-SRNP-22527	MHMXX513-09	JF778401
1955	Polyctor polyctor	Pyrginae	07-SRNP-20902	MHAHL198-07	JF762638
1956	Polyctor polyctor	Pyrginae	08-SRNP-360	MHMXT098-08	JF762651
1957	Polyctor polyctor	Pyrginae	08-SRNP-353	MHMXT105-08	JF762644
1958	Polyctor polyctor	Pyrginae	09-SRNP-20298	MHMYB142-09	
1959	Polyctor polyctor	Pyrginae	09-SRNP-23649	MHMYG2014-10	HM885416

Tree Order	Species	Subfamily	ACG Sampleid	BOLD Processid	Genbank Accession
1960	Polyctor cleta	Pyrginae	09-SRNP-12336	MHMYG2040-10	HM885442
1961	Noctuana lactifera	Pyrginae	02-SRNP-24244	CSCR163-04	DQ292765
1962	Noctuana lactifera	Pyrginae	02-SRNP-23370	CSCR162-04	DQ292764
1963	Noctuana stator	Pyrginae	04-SRNP-2409	MHAHD047-05	GU161701
1964	Noctuana stator	Pyrginae	04-SRNP-24455	MHAHD050-05	GU161704
1965	Noctuana stator	Pyrginae	04-SRNP-24257	MHAHE932-06	GU149776
1966	Noctuana stator	Pyrginae	06-SRNP-60240	MHMXH857-07	JF760903
1967	Noctuana stator	Pyrginae	01-SRNP-2693	MHAHE143-05	GU149781
1968	Noctuana stator	Pyrginae	07-SRNP-2653	MHAHL207-07	JF762461
1969	Noctuana stator	Pyrginae	07-SRNP-2654	MHAHL206-07	JF762460
1970	Noctuana stator	Pyrginae	07-SRNP-31904	MHAHL205-07	JF762459
1971	Noctuana stator	Pyrginae	07-SRNP-736	MHMXK418-07	JF762463
1972	Noctuana stator	Pyrginae	07-SRNP-735	MHMXK417-07	JF762464
1973	Noctuana stator	Pyrginae	06-SRNP-7267	MHAHJ537-07	JF752942
1974	Noctuana stator	Pyrginae	06-SRNP-6258	MHAHJ486-07	JF752941
1975	Noctuana stator	Pyrginae	06-SRNP-43409	MHAHI583-06	GU156027
1976	Noctuana stator	Pyrginae	05-SRNP-4577	MHAHF102-06	GU150630
1977	Noctuana stator	Pyrginae	05-SRNP-3987	MHAHF100-06	GU150627
1978	Noctuana stator	Pyrginae	05-SRNP-32434	MHAHF093-06	GU150628
1979	Noctuana stator	Pyrginae	01-SRNP-2492	MHAHE145-05	GU149783
1980	Noctuana stator	Pyrginae	01-SRNP-2692	MHAHE144-05	GU149782
1981	Noctuana stator	Pyrginae	01-SRNP-2691	MHAHE142-05	GU149779
1982	Noctuana stator	Pyrginae	02-SRNP-6661	MHAHE140-05	GU149780
1983	Noctuana stator	Pyrginae	02-SRNP-7532	MHAHE139-05	GU149778
1984	Noctuana stator	Pyrginae	02-SRNP-17990	MHAHE138-05	GU149777
1985	Noctuana stator	Pyrginae	04-SRNP-3780	MHAHD051-05	GU161705
1986	Noctuana stator	Pyrginae	04-SRNP-2095	MHAHD048-05	GU161702
1987	Noctuana stator	Pyrginae	05-SRNP-5563	MHAHF799-06	GU150631
1988	Noctuana stator	Pyrginae	01-SRNP-2493	MHAHE141-05	GU149775
1989	Noctuana stator	Pyrginae	05-SRNP-453	MHAHF101-06	GU150629
1990	Noctuana stator	Pyrginae	02-SRNP-17989	CSCR161-04	DQ292763
1991	Noctuana stator	Pyrginae	01-SRNP-2491	CSCR160-04	DQ292762
1992	Noctuana stator	Pyrginae	04-SRNP-41356	MHAHD049-05	GU161703
1993	Noctuana stator	Pyrginae	07-SRNP-24112	MHMXT192-08	JF762462
1994	Noctuana stator	Pyrginae	08-SRNP-4026	MHMXX1094-09	JF778153
1995	Noctuana stator	Pyrginae	08-SRNP-4027	MHMXX1095-09	JF778154
1996	Noctuana stator	Pyrginae	08-SRNP-4533	MHMXX626-09	JF778152
1997	Staphylus evemerus	Pyrginae	00-SRNP-10647	MHAHG881-06	GU151651
1998	Staphylus evemerus	Pyrginae	99-SRNP-740	MHAHG884-06	GU151655
1999	Staphylus evemerus	Pyrginae	00-SRNP-10651	MHAHG880-06	GU151652
2000	Staphylus evemerus	Pyrginae	00-SRNP-10765	MHAHG879-06	GU151653
2001	Staphylus evemerus	Pyrginae	00-SRNP-10414	MHAHG878-06	GU151649
2002	Staphylus evemerus	Pyrginae	00-SRNP-10763	MHAHG876-06	GU151645
2003	Staphylus evemerus	Pyrginae	00-SRNP-10413	MHAHG875-06	GU151647
2004	Staphylus evemerus	Pyrginae	03-SRNP-23252	MHAHG873-06	GU151648
2005	Staphylus evemerus	Pyrginae	05-SRNP-35826	MHAHF094-06	GU150855
2006	Staphylus evemerus	Pyrginae	04-SRNP-35967	MHAHD122-05	GU161853
2007	Staphylus evemerus	Pyrginae	00-SRNP-10644	MHAHG877-06	GU151650
2008	Staphylus evemerus	Pyrginae	00-SRNP-10648	MHAHG874-06	GU151646
2009	Staphylus evemerus	Pyrginae	00-SRNP-10645	MHAHG882-06	GU151654
2010	Staphylus evemerus	Pyrginae	00-SRNP-10654	MHAHG883-06	GU151656
2011	Staphylus evemerus	Pyrginae	02-SRNP-23336	MHAHG897-06	GU151658
2012	Staphylus evemerus	Pyrginae	02-SRNP-23337	MHAHG898-06	GU151657
2013	Staphylus evemerus	Pyrginae	06-SRNP-35702	MHAHJ841-07	JF753167
2014	Staphylus evemerus	Pyrginae	07-SRNP-36178	MHMXP210-08	JF762940
2015	Bolla zorillaDHJ11	Pyrginae	05-SRNP-5923	MHAHF026-06	GU150883

Tree Order	Species	Subfamily	ACG Sampleid	BOLD Processid	Genbank Accession
2016	Bolla zorillaDHJ11	Pyrginae	03-SRNP-6884	MHAHG885-06	GU151686
2017	Bolla zorillaDHJ11	Pyrginae	06-SRNP-7810	MHAHJ435-07	JF752487
2018	Bolla zorillaDHJ11	Pyrginae	06-SRNP-7811	MHAHJ436-07	JF752488
2019	Bolla zorillaDHJ09	Pyrginae	05-SRNP-7192	MHAHF809-06	GU150886
2020	Bolla zorillaDHJ09	Pyrginae	05-SRNP-5451	MHAHF062-06	GU150884
2021	Bolla zorillaDHJ09	Pyrginae	05-SRNP-7193	MHAHF807-06	GU150887
2022	Bolla zorillaDHJ09	Pyrginae	05-SRNP-7191	MHAHF808-06	GU150885
2023	Bolla zorillaDHJ09	Pyrginae	06-SRNP-1939	MHAHG781-06	GU151685
2024	Bolla zorillaDHJ09	Pyrginae	06-SRNP-9445	MHAHJ726-07	JF752486
2025	Bolla zorillaDHJ13	Pyrginae	98-SRNP-3082	MHAHH029-06	GU155495
2026	Bolla zorillaDHJ13	Pyrginae	04-SRNP-3961	MHAHD134-05	GU161855
2027	Bolla zorillaDHJ13	Pyrginae	98-SRNP-3081	MHAHH030-06	GU155496
2028	Bolla zorillaDHJ13	Pyrginae	06-SRNP-7350	MHAHJ693-07	JF752489
2029	Bolla zorillaDHJ02	Pyrginae	04-SRNP-3517	MHAHD135-05	GU161856
2030	Bolla zorillaDHJ02	Pyrginae	05-SRNP-546	MHAHF025-06	GU150856
2031	Bolla zorillaDHJ02	Pyrginae	05-SRNP-7189	MHAHF812-06	GU150858
2032	Bolla zorillaDHJ02	Pyrginae	05-SRNP-7190	MHAHF813-06	GU150860
2033	Bolla zorillaDHJ02	Pyrginae	02-SRNP-3822	MHAHG886-06	GU151660
2034	Bolla zorillaDHJ02	Pyrginae	04-SRNP-3614	MHAHD128-05	GU161854
2035	Bolla zorillaDHJ02	Pyrginae	06-SRNP-9042	MHAHJ458-07	JF752484
2036	Bolla zorillaDHJ02	Pyrginae	07-SRNP-1168	MHAHK299-07	JF760453
2037	Bolla zorillaDHJ02	Pyrginae	07-SRNP-611	MHAHK304-07	JF760454
2038	Bolla zorillaDHJ02	Pyrginae	05-SRNP-5922	MHAHF798-06	GU150857
2039	Bolla zorillaDHJ02	Pyrginae	05-SRNP-7218	MHAHF805-06	GU150859
2040	Bolla zorillaDHJ02	Pyrginae	06-SRNP-2051	MHAHG782-06	GU151659
2041	Bolla zorillaDHJ02	Pyrginae	06-SRNP-4375	MHAHI184-06	GU156311
2042	Bolla zorillaDHJ02	Pyrginae	06-SRNP-3931	MHAHI185-06	GU156312
2043	Bolla zorillaDHJ02	Pyrginae	06-SRNP-6672	MHAHJ437-07	JF752481
2044	Bolla zorillaDHJ02	Pyrginae	06-SRNP-6230	MHAHJ455-07	JF752482
2045	Bolla zorillaDHJ02	Pyrginae	06-SRNP-6674	MHAHJ456-07	JF752483
2046	Bolla zorillaDHJ02	Pyrginae	06-SRNP-7405	MHAHJ697-07	JF752485
2047	Bolla zorillaDHJ02	Pyrginae	07-SRNP-2467	MHAHL159-07	JF761683
2048	Arteurotia tractipennis	Pyrginae	95-SRNP-11460	CSCR019-04	DQ291842
2049	Arteurotia tractipennis	Pyrginae	06-SRNP-1612	MHAHG796-06	GU151080
2050	Arteurotia tractipennis	Pyrginae	95-SRNP-11459	CSCR018-04	DQ291841
2051	Arteurotia tractipennis	Pyrginae	04-SRNP-32635	MHAHE070-05	GU149405
2052	Arteurotia tractipennis	Pyrginae	04-SRNP-32574	MHAHE069-05	GU149404
2053	Arteurotia tractipennis	Pyrginae	96-SRNP-1080	CSRII168-04	DQ291844
2054	Arteurotia tractipennis	Pyrginae	00-SRNP-2356	CSRII170-04	DQ291846
2055	Arteurotia tractipennis	Pyrginae	00-SRNP-2402	CSRII171-04	DQ291847
2056	Arteurotia tractipennis	Pyrginae	00-SRNP-2258	CSRII169-04	DQ291845
2057	Arteurotia tractipennis	Pyrginae	96-SRNP-1001	CSRII167-04	DQ291843
2058	Arteurotia tractipennis	Pyrginae	05-SRNP-30659	MHAHF121-06	GU150194
2059	Arteurotia tractipennis	Pyrginae	06-SRNP-57518	MHAHJ831-07	JF752382
2060	Bolla evippe	Pyrginae	09-SRNP-57008	MHMYG2488-10	HM885915
2061	Staphylus Janzen03	Pyrginae	04-SRNP-47281	MHAHD124-05	GU161857
2062	Staphylus Janzen08	Pyrginae	05-SRNP-7212	MHAHF777-06	GU150882
2063	Staphylus Janzen08	Pyrginae	06-SRNP-1211	MHAHG218-06	GU151684
2064	Staphylus Janzen08	Pyrginae	06-SRNP-9509	MHAHJ457-07	JF753168
2065	Staphylus ascalaphus	Pyrginae	99-SRNP-3147	MHAHG931-06	GU151661
2066	Staphylus ascalaphus	Pyrginae	00-SRNP-20227	MHAHH010-06	GU155497
2067	Staphylus ascalaphus	Pyrginae	08-SRNP-55731	MHMXX1081-09	JF778473
2068	Staphylus ascalaphus	Pyrginae	05-SRNP-55703	MHAHF030-06	GU150862
2069	Staphylus ascalaphus	Pyrginae	00-SRNP-6524	MHAHG930-06	GU151662
2070	Staphylus ascalaphus	Pyrginae	05-SRNP-22540	MHAHF018-06	GU150861
2071	Staphylus ascalaphus	Pyrginae	05-SRNP-55693	MHAHF023-06	GU150863

Tree Order	Species	Subfamily	ACG Sampleid	BOLD Processid	Genbank Accession
2072	Staphylus ascalaphus	Pyrginae	06-SRNP-7407	MHAHJ695-07	JF753159
2073	Staphylus ascalaphus	Pyrginae	07-SRNP-55673	MHAHK303-07	JF761176
2074	Staphylus ascalaphus	Pyrginae	06-SRNP-19545	MHAHK305-07	JF761177
2075	Staphylus ascalaphus	Pyrginae	08-SRNP-55942	MHMXX1084-09	JF778474
2076	Staphylus ascalaphus	Pyrginae	08-SRNP-55778	MHMXX1085-09	JF778475
2077	Staphylus ascalaphus	Pyrginae	08-SRNP-55888	MHMXX1086-09	JF778476
2078	Staphylus ascalaphus	Pyrginae	05-SRNP-55700	MHAHF029-06	GU150864
2079	Staphylus ascalaphus	Pyrginae	07-SRNP-55652	MHAHK301-07	JF761174
2080	Staphylus ascalaphus	Pyrginae	05-SRNP-55699	MHAHF046-06	GU150866
2081	Staphylus ascalaphus	Pyrginae	05-SRNP-55697	MHAHF047-06	GU150865
2082	Staphylus ascalaphus	Pyrginae	06-SRNP-9159	MHAHJ526-07	JF753157
2083	Staphylus ascalaphus	Pyrginae	06-SRNP-8678	MHAHJ528-07	JF753158
2084	Staphylus ascalaphus	Pyrginae	07-SRNP-55651	MHAHK300-07	JF761173
2085	Staphylus ascalaphus	Pyrginae	07-SRNP-55830	MHAHK302-07	JF761175
2086	Staphylus ascalaphus	Pyrginae	08-SRNP-55975	MHMXX1087-09	JF778477
2087	Staphylus Janzen10	Pyrginae	05-SRNP-55789	MHAHF024-06	GU150888
2088	Staphylus vulgata	Pyrginae	04-SRNP-45556	MHAHG891-06	GU151694
2089	Staphylus vulgata	Pyrginae	03-SRNP-28632	MHAHG902-06	GU151700
2090	Staphylus vulgata	Pyrginae	04-SRNP-20675	MHAHG913-06	GU151705
2091	Staphylus vulgata	Pyrginae	04-SRNP-20668	MHAHH003-06	GU155526
2092	Staphylus vulgata	Pyrginae	04-SRNP-22975	MHAHD119-05	GU161865
2093	Staphylus vulgata	Pyrginae	05-SRNP-45426	MHAHF020-06	GU150890
2094	Staphylus vulgata	Pyrginae	02-SRNP-15565	MHAHH026-06	GU155517
2095	Staphylus vulgata	Pyrginae	02-SRNP-5534	MHAHG912-06	GU151706
2096	Staphylus vulgata	Pyrginae	04-SRNP-20840	MHAHH007-06	GU155525
2097	Staphylus vulgata	Pyrginae	02-SRNP-5685	MHAHH012-06	GU155515
2098	Staphylus vulgata	Pyrginae	02-SRNP-5693	MHAHH019-06	GU155521
2099	Staphylus vulgata	Pyrginae	02-SRNP-5530	MHAHH024-06	GU155520
2100	Staphylus vulgata	Pyrginae	02-SRNP-5689	MHAHH025-06	GU155518
2101	Staphylus vulgata	Pyrginae	02-SRNP-5535	MHAHH027-06	GU155516
2102	Staphylus vulgata	Pyrginae	06-SRNP-22894	MHAHJ439-07	JF753169
2103	Staphylus vulgata	Pyrginae	07-SRNP-55674	MHAHK306-07	JF761187
2104	Staphylus vulgata	Pyrginae	04-SRNP-22011	MHAHG889-06	GU151692
2105	Staphylus vulgata	Pyrginae	07-SRNP-55812	MHAHK291-07	JF761184
2106	Staphylus vulgata	Pyrginae	06-SRNP-58302	MHAHJ696-07	JF753173
2107	Staphylus vulgata	Pyrginae	06-SRNP-22880	MHAHJ527-07	JF753171
2108	Staphylus vulgata	Pyrginae	04-SRNP-45022	MHAHH023-06	GU155522
2109	Staphylus vulgata	Pyrginae	03-SRNP-29100	MHAHH021-06	GU155519
2110	Staphylus vulgata	Pyrginae	02-SRNP-5684	MHAHG925-06	GU151707
2111	Staphylus vulgata	Pyrginae	02-SRNP-5692	MHAHG910-06	GU151687
2112	Staphylus vulgata	Pyrginae	04-SRNP-45563	MHAHG893-06	GU151689
2113	Staphylus vulgata	Pyrginae	04-SRNP-45512	MHAHG892-06	GU151691
2114	Staphylus vulgata	Pyrginae	04-SRNP-45548	MHAHG890-06	GU151695
2115	Staphylus vulgata	Pyrginae	04-SRNP-45554	MHAHG887-06	GU151688
2116	Staphylus vulgata	Pyrginae	05-SRNP-55314	MHAHF069-06	GU150897
2117	Staphylus vulgata	Pyrginae	05-SRNP-55698	MHAHF034-06	GU150891
2118	Staphylus vulgata	Pyrginae	05-SRNP-55849	MHAHF033-06	GU150895
2119	Staphylus vulgata	Pyrginae	05-SRNP-57366	MHAHF032-06	GU150896
2120	Staphylus vulgata	Pyrginae	05-SRNP-55705	MHAHF021-06	GU150889
2121	Staphylus vulgata	Pyrginae	05-SRNP-59885	MHAHF017-06	GU150894
2122	Staphylus vulgata	Pyrginae	05-SRNP-46990	MHAHF016-06	GU150892
2123	Staphylus vulgata	Pyrginae	05-SRNP-59424	MHAHF014-06	GU150893
2124	Staphylus vulgata	Pyrginae	04-SRNP-45189	MHAHD126-05	GU161863
2125	Staphylus vulgata	Pyrginae	04-SRNP-23419	MHAHD123-05	GU161864
2126	Staphylus vulgata	Pyrginae	04-SRNP-48166	MHAHD121-05	GU161867
2127	Staphylus vulgata	Pyrginae	04-SRNP-22233	MHAHG895-06	GU151699

Tree Order	Species	Subfamily	ACG Sampleid	BOLD Processid	Genbank Accession
2128	Staphylus vulgata	Pyrginae	04-SRNP-45224	MHAHD132-05	GU161866
2129	Staphylus vulgata	Pyrginae	04-SRNP-45552	MHAHG888-06	GU151693
2130	Staphylus vulgata	Pyrginae	98-SRNP-5965	MHAHG904-06	GU151696
2131	Staphylus vulgata	Pyrginae	02-SRNP-5690	MHAHG909-06	GU151690
2132	Staphylus vulgata	Pyrginae	98-SRNP-4751	MHAHG915-06	GU151703
2133	Staphylus vulgata	Pyrginae	06-SRNP-46873	MHAHJ725-07	JF753174
2134	Staphylus vulgata	Pyrginae	06-SRNP-58287	MHAHJ849-07	JF753175
2135	Staphylus vulgata	Pyrginae	07-SRNP-55628	MHAHK290-07	JF761183
2136	Staphylus vulgata	Pyrginae	07-SRNP-55813	MHAHK294-07	JF761185
2137	Staphylus vulgata	Pyrginae	07-SRNP-55675	MHAHK296-07	JF761186
2138	Staphylus vulgata	Pyrginae	07-SRNP-55822	MHAHK309-07	JF761188
2139	Staphylus vulgata	Pyrginae	07-SRNP-23532	MHMXR057-08	JF762942
2140	Staphylus vulgata	Pyrginae	07-SRNP-65029	MHMXR070-08	JF762941
2141	Staphylus vulgata	Pyrginae	08-SRNP-55775	MHMXX1118-09	JF778485
2142	Staphylus vulgata	Pyrginae	03-SRNP-1698	MHAHG905-06	GU151702
2143	Staphylus vulgata	Pyrginae	04-SRNP-22010	MHAHG896-06	GU151698
2144	Staphylus vulgata	Pyrginae	04-SRNP-45540	MHAHG894-06	GU151697
2145	Staphylus vulgata	Pyrginae	05-SRNP-24781	MHAHF769-06	GU150899
2146	Staphylus vulgata	Pyrginae	04-SRNP-20669	MHAHD131-05	GU161868
2147	Staphylus vulgata	Pyrginae	05-SRNP-20931	MHAHF066-06	GU150898
2148	Staphylus vulgata	Pyrginae	02-SRNP-5681	MHAHG906-06	GU151701
2149	Staphylus vulgata	Pyrginae	03-SRNP-1969	MHAHG914-06	GU151704
2150	Staphylus vulgata	Pyrginae	02-SRNP-15215	MHAHG932-06	GU151708
2151	Staphylus vulgata	Pyrginae	02-SRNP-5917	MHAHG933-06	GU151709
2152	Staphylus vulgata	Pyrginae	04-SRNP-20841	MHAHH001-06	GU155524
2153	Staphylus vulgata	Pyrginae	03-SRNP-28724	MHAHH028-06	GU155514
2154	Staphylus vulgata	Pyrginae	02-SRNP-14335	MHAHH033-06	GU155523
2155	Staphylus vulgata	Pyrginae	06-SRNP-57164	MHAHI187-06	GU156320
2156	Staphylus vulgata	Pyrginae	06-SRNP-22103	MHAHJ464-07	JF753170
2157	Staphylus vulgata	Pyrginae	06-SRNP-22892	MHAHJ531-07	JF753172
2158	Staphylus vulgata	Pyrginae	07-SRNP-55155	MHAHK153-07	JF761182
2159	Staphylus vulgata	Pyrginae	08-SRNP-65738	MHMXX1117-09	JF778484
2160	Staphylus vulgata	Pyrginae	08-SRNP-24000	MHMYB157-09	GU649680
2161	Staphylus azteca	Pyrginae	00-SRNP-6527	MHAHH016-06	GU155502
2162	Staphylus azteca	Pyrginae	05-SRNP-55694	MHAHF081-06	GU150881
2163	Staphylus azteca	Pyrginae	00-SRNP-6505	MHAHI237-06	GU156319
2164	Staphylus azteca	Pyrginae	02-SRNP-17510	MHAHH022-06	GU155506
2165	Staphylus azteca	Pyrginae	02-SRNP-5531	MHAHH020-06	GU155505
2166	Staphylus azteca	Pyrginae	98-SRNP-5974	MHAHH031-06	GU155508
2167	Staphylus azteca	Pyrginae	00-SRNP-6532	MHAHH014-06	GU155498
2168	Staphylus azteca	Pyrginae	02-SRNP-5688	MHAHH013-06	GU155499
2169	Staphylus azteca	Pyrginae	00-SRNP-20190	MHAHH004-06	GU155513
2170	Staphylus azteca	Pyrginae	03-SRNP-2688	MHAHG929-06	GU151683
2171	Staphylus azteca	Pyrginae	03-SRNP-2728	MHAHG926-06	GU151682
2172	Staphylus azteca	Pyrginae	04-SRNP-20603	MHAHG922-06	GU151675
2173	Staphylus azteca	Pyrginae	02-SRNP-28722	MHAHG908-06	GU151669
2174	Staphylus azteca	Pyrginae	02-SRNP-5769	MHAHG907-06	GU151670
2175	Staphylus azteca	Pyrginae	05-SRNP-55695	MHAHF035-06	GU150878
2176	Staphylus azteca	Pyrginae	05-SRNP-22538	MHAHF015-06	GU150877
2177	Staphylus azteca	Pyrginae	04-SRNP-22976	MHAHD129-05	GU161862
2178	Staphylus azteca	Pyrginae	04-SRNP-23046	MHAHD120-05	GU161861
2179	Staphylus azteca	Pyrginae	98-SRNP-5973	MHAHG916-06	GU151671
2180	Staphylus azteca	Pyrginae	00-SRNP-6536	MHAHI233-06	GU156317
2181	Staphylus azteca	Pyrginae	08-SRNP-55660	MHMXX1079-09	JF778479
2182	Staphylus azteca	Pyrginae	08-SRNP-55779	MHMXX1083-09	JF778482
2183	Staphylus azteca	Pyrginae	08-SRNP-55777	MHMXX1120-09	JF778483

Tree Order	Species	Subfamily	ACG Sampleid	BOLD Processid	Genbank Accession
2184	Staphylus azteca	Pyrginae	00-SRNP-20191	MHAHH005-06	GU155512
2185	Staphylus azteca	Pyrginae	08-SRNP-55729	MHMXX1078-09	JF778478
2186	Staphylus azteca	Pyrginae	07-SRNP-55831	MHAHK295-07	JF761180
2187	Staphylus azteca	Pyrginae	06-SRNP-18820	MHAHK292-07	JF761178
2188	Staphylus azteca	Pyrginae	02-SRNP-5529	MHAHH018-06	GU155504
2189	Staphylus azteca	Pyrginae	00-SRNP-6529	MHAHH015-06	GU155503
2190	Staphylus azteca	Pyrginae	03-SRNP-2686	MHAHG924-06	GU151681
2191	Staphylus azteca	Pyrginae	00-SRNP-6598	MHAHG921-06	GU151677
2192	Staphylus azteca	Pyrginae	00-SRNP-7041	MHAHG920-06	GU151678
2193	Staphylus azteca	Pyrginae	04-SRNP-22251	MHAHG900-06	GU151665
2194	Staphylus azteca	Pyrginae	04-SRNP-22252	MHAHG899-06	GU151666
2195	Staphylus azteca	Pyrginae	05-SRNP-55771	MHAHF027-06	GU150879
2196	Staphylus azteca	Pyrginae	05-SRNP-55417	MHAHF022-06	GU150876
2197	Staphylus azteca	Pyrginae	03-SRNP-2646	MHAHG903-06	GU151668
2198	Staphylus azteca	Pyrginae	02-SRNP-27769	MHAHG927-06	GU151680
2199	Staphylus azteca	Pyrginae	02-SRNP-27768	MHAHH009-06	GU155510
2200	Staphylus azteca	Pyrginae	00-SRNP-7043	MHAHG919-06	GU151674
2201	Staphylus azteca	Pyrginae	03-SRNP-19829	MHAHG917-06	GU151673
2202	Staphylus azteca	Pyrginae	02-SRNP-27766	MHAHH017-06	GU155501
2203	Staphylus azteca	Pyrginae	98-SRNP-13484	MHAHH034-06	GU155507
2204	Staphylus azteca	Pyrginae	00-SRNP-6518	MHAHI232-06	GU156316
2205	Staphylus azteca	Pyrginae	02-SRNP-27389	MHAHG901-06	GU151667
2206	Staphylus azteca	Pyrginae	00-SRNP-6585	MHAHI234-06	GU156314
2207	Staphylus azteca	Pyrginae	00-SRNP-6599	MHAHI235-06	GU156315
2208	Staphylus azteca	Pyrginae	07-SRNP-55820	MHAHK293-07	JF761179
2209	Staphylus azteca	Pyrginae	00-SRNP-20193	MHAHG918-06	GU151676
2210	Staphylus azteca	Pyrginae	02-SRNP-27392	MHAHH011-06	GU155500
2211	Staphylus azteca	Pyrginae	08-SRNP-55943	MHMXX1080-09	JF778480
2212	Staphylus azteca	Pyrginae	98-SRNP-5691	MHAHH032-06	GU155509
2213	Staphylus azteca	Pyrginae	02-SRNP-29445	MHAHH008-06	GU155511
2214	Staphylus azteca	Pyrginae	02-SRNP-27393	MHAHG928-06	GU151679
2215	Staphylus azteca	Pyrginae	00-SRNP-6587	MHAHG911-06	GU151672
2216	Staphylus azteca	Pyrginae	00-SRNP-6533	MHAHI236-06	GU156318
2217	Staphylus azteca	Pyrginae	07-SRNP-55875	MHAHK310-07	JF761181
2218	Staphylus azteca	Pyrginae	08-SRNP-55945	MHMXX1082-09	JF778481
2219	Staphylus azteca	Pyrginae	09-SRNP-68493	MHMYG2487-10	HM885914
2220	Staphylus caribbea	Pyrginae	07-SRNP-65990	MHMXR055-08	JF762937
2221	Staphylus caribbea	Pyrginae	05-SRNP-6745	MHAHF793-06	GU150869
2222	Staphylus caribbea	Pyrginae	08-SRNP-6754	MHMXZ041-09	
2223	Staphylus caribbea	Pyrginae	07-SRNP-22962	MHMXR056-08	JF762936
2224	Staphylus caribbea	Pyrginae	04-SRNP-42021	MHAHD133-05	GU161859
2225	Staphylus caribbea	Pyrginae	07-SRNP-3050	MHAHL161-07	JF762935
2226	Staphylus caribbea	Pyrginae	06-SRNP-2133	MHAHG783-06	GU151663
2227	Staphylus caribbea	Pyrginae	07-SRNP-3052	MHMXP218-08	JF762938
2228	Staphylus caribbea	Pyrginae	06-SRNP-316	MHAHG221-06	GU151664
2229	Staphylus caribbea	Pyrginae	09-SRNP-75511	MHMYG2481-10	HM885908
2230	Staphylus caribbea	Pyrginae	09-SRNP-42728	MHMYG2482-10	HM885909
2231	Staphylus caribbea	Pyrginae	09-SRNP-68492	MHMYG2484-10	HM885911
2232	Staphylus caribbea	Pyrginae	06-SRNP-6883	MHAHJ438-07	JF753160
2233	Staphylus caribbea	Pyrginae	05-SRNP-7211	MHAHF811-06	GU150872
2234	Staphylus caribbea	Pyrginae	05-SRNP-7214	MHAHF806-06	GU150870
2235	Staphylus caribbea	Pyrginae	05-SRNP-6743	MHAHF797-06	GU150867
2236	Staphylus caribbea	Pyrginae	05-SRNP-6797	MHAHF795-06	GU150868
2237	Staphylus caribbea	Pyrginae	05-SRNP-7213	MHAHF794-06	GU150871
2238	Staphylus caribbea	Pyrginae	05-SRNP-6906	MHAHF778-06	GU150873
2239	Staphylus caribbea	Pyrginae	05-SRNP-7752	MHAHF693-06	GU150874

Tree Order	Species	Subfamily	ACG Sampleid	BOLD Processid	Genbank Accession
2240	Staphylus caribbea	Pyrginae	04-SRNP-42026	MHAHD127-05	GU161860
2241	Staphylus caribbea	Pyrginae	04-SRNP-42022	MHAHD125-05	GU161858
2242	Staphylus caribbea	Pyrginae	05-SRNP-7750	MHAHF694-06	GU150875
2243	Staphylus caribbea	Pyrginae	06-SRNP-5510	MHAHI183-06	GU156313
2244	Staphylus caribbea	Pyrginae	06-SRNP-9444	MHAHJ529-07	JF753161
2245	Staphylus caribbea	Pyrginae	06-SRNP-8603	MHAHJ530-07	JF753162
2246	Staphylus caribbea	Pyrginae	06-SRNP-8454	MHAHJ532-07	JF753163
2247	Staphylus caribbea	Pyrginae	06-SRNP-9154	MHAHJ533-07	JF753164
2248	Staphylus caribbea	Pyrginae	06-SRNP-9158	MHAHJ535-07	JF753165
2249	Staphylus caribbea	Pyrginae	06-SRNP-6882	MHAHJ694-07	JF753166
2250	Staphylus caribbea	Pyrginae	07-SRNP-3051	MHMXP217-08	JF762939
2251	Staphylus caribbea	Pyrginae	08-SRNP-6933	MHMYC507-09	GU649776
2252	Staphylus caribbea	Pyrginae	09-SRNP-44334	MHMYE1552-09	HM391112
2253	Staphylus caribbea	Pyrginae	09-SRNP-44524	MHMYE1553-09	HM391113
2254	Staphylus caribbea	Pyrginae	09-SRNP-75315	MHMYG2483-10	HM885910
2255	Staphylus caribbea	Pyrginae	09-SRNP-68494	MHMYG2485-10	HM885912
2256	Staphylus caribbea	Pyrginae	09-SRNP-44999	MHMYG2045-10	HM885447
2257	Spioniades artemides	Pyrginae	07-SRNP-65617	MHMXO946-08	JF762934
2258	Spioniades abbreviata	Pyrginae	05-SRNP-40403	MHAHF319-06	GU150853
2259	Spioniades abbreviata	Pyrginae	05-SRNP-6908	MHAHF779-06	GU150854
2260	Spioniades abbreviata	Pyrginae	03-SRNP-20661	CSCR582-04	DQ293330
2261	Spioniades abbreviata	Pyrginae	03-SRNP-20234	CSCR581-04	DQ293329
2262	Spioniades abbreviata	Pyrginae	04-SRNP-42534	MHAHD879-05	GU161852
2263	Spioniades abbreviata	Pyrginae	08-SRNP-70324	MHMXW538-09	JF754168
2264	Spioniades abbreviata	Pyrginae	09-SRNP-72126	MHMYE1557-09	HM391117
2265	Grais stigmaticus	Pyrginae	95-SRNP-11369	CSCR112-04	DQ292558
2266	Doberes anticus	Pyrginae	00-SRNP-9187	CSRII447-04	DQ292284
2267	Doberes anticus	Pyrginae	00-SRNP-9201	CSCR089-04	DQ292283
2268	Doberes anticus	Pyrginae	00-SRNP-9246	CSRII448-04	DQ292285
2269	Achlyodes pallida	Pyrginae	06-SRNP-55557	MHAHG172-06	GU151042
2270	Achlyodes pallida	Pyrginae	04-SRNP-35303	MHAHC003-05	DQ291768
2271	Achlyodes pallida	Pyrginae	04-SRNP-46891	MHAHC019-05	DQ291770
2272	Achlyodes pallida	Pyrginae	04-SRNP-35345	MHAHC027-05	DQ291771
2273	Achlyodes pallida	Pyrginae	04-SRNP-35699	MHAHC825-05	DQ291776
2274	Achlyodes pallida	Pyrginae	06-SRNP-59832	MHMXH851-07	JF760225
2275	Achlyodes pallida	Pyrginae	05-SRNP-35395	MHAHL106-07	JF761334
2276	Achlyodes pallida	Pyrginae	07-SRNP-1848	MHAHL190-07	JF761338
2277	Achlyodes pallida	Pyrginae	05-SRNP-2176	MHAHL107-07	JF761335
2278	Achlyodes pallida	Pyrginae	05-SRNP-59488	MHAHL105-07	JF761333
2279	Achlyodes pallida	Pyrginae	06-SRNP-60158	MHMXH852-07	JF760224
2280	Achlyodes pallida	Pyrginae	06-SRNP-58206	MHAHJ647-07	JF752347
2281	Achlyodes pallida	Pyrginae	04-SRNP-61445	MHAHC828-05	DQ291778
2282	Achlyodes pallida	Pyrginae	04-SRNP-35305	MHAHC824-05	DQ291775
2283	Achlyodes pallida	Pyrginae	04-SRNP-35977	MHAHC822-05	DQ291774
2284	Achlyodes pallida	Pyrginae	04-SRNP-35301	MHAHC011-05	DQ291769
2285	Achlyodes pallida	Pyrginae	04-SRNP-35304	MHAHC091-05	DQ291773
2286	Achlyodes pallida	Pyrginae	04-SRNP-45082	MHAHC083-05	DQ291772
2287	Achlyodes pallida	Pyrginae	06-SRNP-55370	MHAHG171-06	GU151043
2288	Achlyodes pallida	Pyrginae	03-SRNP-3397	CSCR299-04	DQ291767
2289	Achlyodes pallida	Pyrginae	06-SRNP-60271	MHMXH853-07	JF760223
2290	Achlyodes pallida	Pyrginae	02-SRNP-23406	CSCR005-04	DQ291766
2291	Achlyodes pallida	Pyrginae	04-SRNP-35390	MHAHC827-05	DQ291777
2292	Achlyodes pallida	Pyrginae	07-SRNP-35141	MHAHL188-07	JF761336
2293	Achlyodes pallida	Pyrginae	07-SRNP-35829	MHAHL189-07	JF761337
2294	Achlyodes pallida	Pyrginae	07-SRNP-36029	MHMXP170-08	JF761339
2295	Achlyodes pallida	Pyrginae	09-SRNP-57230	MHMYE1519-09	GU653485

Tree Order	Species	Subfamily	ACG Sampleid	BOLD Processid	Genbank Accession
2296	Eantis thraso	Pyrginae	07-SRNP-1978	MHAHL227-07	JF762153
2297	Eantis thraso	Pyrginae	06-SRNP-22421	MHMXK062-07	JF762156
2298	Eantis thraso	Pyrginae	96-SRNP-1072	CSCR007-04	DQ291780
2299	Eantis thraso	Pyrginae	96-SRNP-262	CSCR006-04	DQ291779
2300	Eantis thraso	Pyrginae	04-SRNP-49038	MHAHE307-05	GU149321
2301	Eantis thraso	Pyrginae	04-SRNP-41151	MHAHE308-05	GU149322
2302	Eantis thraso	Pyrginae	04-SRNP-50037	MHAHE309-05	GU149320
2303	Eantis thraso	Pyrginae	04-SRNP-45216	MHAHE310-05	GU149317
2304	Eantis thraso	Pyrginae	04-SRNP-48345	MHAHE312-05	GU149324
2305	Eantis thraso	Pyrginae	04-SRNP-15358	MHAHE313-05	GU149323
2306	Eantis thraso	Pyrginae	04-SRNP-4114	MHAHE314-05	GU149319
2307	Eantis thraso	Pyrginae	94-SRNP-279	MHMXI577-07	JF760662
2308	Eantis thraso	Pyrginae	07-SRNP-21152	MHAHL228-07	JF762154
2309	Eantis thraso	Pyrginae	07-SRNP-1975	MHAHL235-07	JF762155
2310	Eantis thraso	Pyrginae	04-SRNP-45084	MHAHE311-05	GU149318
2311	Eantis thraso	Pyrginae	08-SRNP-6018	MHMXZ019-09	GU665260
2312	Achlyodes busirus	Pyrginae	03-SRNP-5736	CSCR298-04	DQ291765
2313	Achlyodes busirus	Pyrginae	07-SRNP-1804	MHAHL439-07	JF761326
2314	Achlyodes busirus	Pyrginae	07-SRNP-56770	MHAHL438-07	JF761325
2315	Achlyodes busirus	Pyrginae	07-SRNP-55259	MHMXK076-07	JF761332
2316	Achlyodes busirus	Pyrginae	07-SRNP-2136	MHAHL434-07	JF761324
2317	Achlyodes busirus	Pyrginae	07-SRNP-1984	MHAHL432-07	JF761322
2318	Achlyodes busirus	Pyrginae	07-SRNP-55258	MHMXK078-07	JF761330
2319	Achlyodes busirus	Pyrginae	07-SRNP-55531	MHMXK077-07	JF761331
2320	Achlyodes busirus	Pyrginae	06-SRNP-44397	MHAHK013-07	JF760221
2321	Achlyodes busirus	Pyrginae	06-SRNP-44330	MHAHK011-07	JF760219
2322	Achlyodes busirus	Pyrginae	06-SRNP-9750	MHAHK010-07	JF760218
2323	Achlyodes busirus	Pyrginae	06-SRNP-59206	MHAHK006-07	JF760214
2324	Achlyodes busirus	Pyrginae	06-SRNP-44403	MHAHK005-07	JF760213
2325	Achlyodes busirus	Pyrginae	04-SRNP-47915	MHAHE358-05	GU149314
2326	Achlyodes busirus	Pyrginae	04-SRNP-48202	MHAHE357-05	GU149313
2327	Achlyodes busirus	Pyrginae	04-SRNP-48915	MHAHE356-05	GU149312
2328	Achlyodes busirus	Pyrginae	04-SRNP-48497	MHAHE355-05	GU149311
2329	Achlyodes busirus	Pyrginae	04-SRNP-32916	MHAHE353-05	GU149315
2330	Achlyodes busirus	Pyrginae	04-SRNP-48413	MHAHE352-05	GU149316
2331	Achlyodes busirus	Pyrginae	06-SRNP-44329	MHAHK007-07	JF760215
2332	Achlyodes busirus	Pyrginae	06-SRNP-44395	MHAHK009-07	JF760217
2333	Achlyodes busirus	Pyrginae	03-SRNP-5928	CSCR297-04	DQ291764
2334	Achlyodes busirus	Pyrginae	06-SRNP-58729	MHAHK008-07	JF760216
2335	Achlyodes busirus	Pyrginae	06-SRNP-44631	MHAHK012-07	JF760220
2336	Achlyodes busirus	Pyrginae	06-SRNP-59810	MHAHK014-07	JF760222
2337	Achlyodes busirus	Pyrginae	07-SRNP-1973	MHAHL433-07	JF761323
2338	Achlyodes busirus	Pyrginae	07-SRNP-57186	MHMXP120-08	JF761329
2339	Achlyodes busirus	Pyrginae	07-SRNP-45267	MHMXP121-08	JF761328
2340	Achlyodes busirus	Pyrginae	07-SRNP-45958	MHMXR902-08	JF761327
2341	Achlyodes busirus	Pyrginae	08-SRNP-2576	MHMXX1053-09	JF777534
2342	Achlyodes busirus	Pyrginae	08-SRNP-70349	MHMXX1054-09	JF777535
2343	Achlyodes busirus	Pyrginae	08-SRNP-70359	MHMXX1055-09	JF777536
2344	Achlyodes busirus	Pyrginae	08-SRNP-2332	MHMXX1056-09	JF777537
2345	Achlyodes busirus	Pyrginae	08-SRNP-2138	MHMXX1057-09	GU666352
2346	Achlyodes busirus	Pyrginae	08-SRNP-30931	MHMXX1058-09	JF777538
2347	Achlyodes busirus	Pyrginae	08-SRNP-20760	MHMXX1059-09	JF777539
2348	Achlyodes busirus	Pyrginae	08-SRNP-2135	MHMXX1060-09	JF777540
2349	Achlyodes busirus	Pyrginae	08-SRNP-4869	MHMXX651-09	JF777541
2350	Achlyodes busirus	Pyrginae	08-SRNP-5054	MHMXX652-09	JF777542
2351	Achlyodes busirus	Pyrginae	09-SRNP-69109	MHMYC528-09	GU649813

Tree Order	Species	Subfamily	ACG Sampleid	BOLD Processid	Genbank Accession
2352	Aethilla echina	Pyrginae	05-SRNP-20	MHACG848-05	DQ291781
2353	Aethilla lavochreaDHJ01	Pyrginae	08-SRNP-23841	MHMYB180-09	HM431657
2354	Aethilla lavochreaDHJ02	Pyrginae	07-SRNP-24077	MHMXR901-08	JF761340
2355	Aethilla lavochreaDHJ02	Pyrginae	08-SRNP-55465	MHMXW543-09	JF753640
2356	Aethilla lavochreaDHJ02	Pyrginae	08-SRNP-22923	MHMXY1093-09	HM390682
2357	Aethilla lavochreaDHJ02	Pyrginae	09-SRNP-4333	MHMYG2440-10	HM885864
2358	Aethilla lavochreaDHJ02	Pyrginae	02-SRNP-14545	MHAHD503-05	GU161215
2359	Aethilla lavochreaDHJ02	Pyrginae	00-SRNP-2103	MHAHD513-05	GU161220
2360	Aethilla lavochreaDHJ02	Pyrginae	02-SRNP-4022	MHAHD512-05	GU161212
2361	Aethilla lavochreaDHJ02	Pyrginae	00-SRNP-4557	MHAHD511-05	GU161219
2362	Aethilla lavochreaDHJ02	Pyrginae	00-SRNP-2105	MHAHD509-05	GU161214
2363	Aethilla lavochreaDHJ02	Pyrginae	02-SRNP-4467	MHAHD508-05	GU161217
2364	Aethilla lavochreaDHJ02	Pyrginae	00-SRNP-4261	MHAHD507-05	GU161211
2365	Aethilla lavochreaDHJ02	Pyrginae	00-SRNP-4439	MHAHD506-05	GU161213
2366	Aethilla lavochreaDHJ02	Pyrginae	00-SRNP-4556	MHAHD505-05	GU161216
2367	Aethilla lavochreaDHJ02	Pyrginae	00-SRNP-4260	MHAHD504-05	GU161218
2368	Aethilla lavochreaDHJ02	Pyrginae	04-SRNP-49719	MHAHD900-05	GU161221
2369	Aethilla lavochreaDHJ02	Pyrginae	08-SRNP-23663	MHMXY1094-09	GU666450
2370	Aethilla lavochreaDHJ02	Pyrginae	08-SRNP-23664	MHMYB181-09	GU649663
2371	Aethilla lavochreaDHJ02	Pyrginae	09-SRNP-4334	MHMYG2441-10	HM885866
2372	Ephyriades eugramma	Pyrginae	03-SRNP-3195	CSCR563-04	DQ293126
2373	Ephyriades eugramma	Pyrginae	03-SRNP-3270	CSCR564-04	DQ293127
2374	Ephyriades eugramma	Pyrginae	03-SRNP-22990	CSCR566-04	DQ293129
2375	Ephyriades eugramma	Pyrginae	03-SRNP-22535	CSCR565-04	DQ293128
2376	Ephyriades eugramma	Pyrginae	07-SRNP-35417	MHAHL246-07	JF762233
2377	Ephyriades eugramma	Pyrginae	08-SRNP-31277	MHMXW534-09	JF753902
2378	Ephyriades eugramma	Pyrginae	08-SRNP-35337	MHMXW535-09	JF753903
2379	Ephyriades eugramma	Pyrginae	09-SRNP-35830	MHMYE1544-09	HM391104
2380	Ephyriades eugramma	Pyrginae	09-SRNP-35828	MHMYE1567-09	HM391127
2381	Cycloglypha thrasibulus	Pyrginae	06-SRNP-55047	MHAHG248-06	GU151336
2382	Cycloglypha thrasibulus	Pyrginae	06-SRNP-1733	MHAHG773-06	GU151334
2383	Cycloglypha thrasibulus	Pyrginae	05-SRNP-2186	MHAHF090-06	GU150356
2384	Cycloglypha thrasibulus	Pyrginae	05-SRNP-56292	MHAHF091-06	GU150355
2385	Cycloglypha thrasibulus	Pyrginae	05-SRNP-2205	MHAHF092-06	GU150357
2386	Cycloglypha thrasibulus	Pyrginae	06-SRNP-40014	MHAHG247-06	GU151335
2387	Cycloglypha thrasibulus	Pyrginae	07-SRNP-55252	MHAHK398-07	JF760633
2388	Cycloglypha thrasibulus	Pyrginae	07-SRNP-55550	MHAHK400-07	JF760634
2389	Cycloglypha thrasibulus	Pyrginae	07-SRNP-55357	MHAHK401-07	JF760635
2390	Cycloglypha thrasibulus	Pyrginae	06-SRNP-18966	MHAHK612-07	JF760636
2391	Cycloglypha thrasibulus	Pyrginae	06-SRNP-18825	MHAHK613-07	JF760637
2392	Cycloglypha thrasibulus	Pyrginae	06-SRNP-19741	MHAHK614-07	JF760638
2393	Cycloglypha thrasibulus	Pyrginae	07-SRNP-59528	MHMXP206-08	JF762003
2394	Cycloglypha thrasibulus	Pyrginae	08-SRNP-56046	MHMXX1088-09	JF777821
2395	Cycloglypha thrasibulus	Pyrginae	01-SRNP-18101	MHAHE130-05	GU149570
2396	Cycloglypha thrasibulus	Pyrginae	05-SRNP-40775	MHAHF089-06	GU150358
2397	Cycloglypha thrasibulus	Pyrginae	02-SRNP-31147	MHAHE129-05	GU149567
2398	Cycloglypha thrasibulus	Pyrginae	02-SRNP-7413	MHAHE127-05	GU149573
2399	Cycloglypha thrasibulus	Pyrginae	02-SRNP-4616	MHAHE125-05	GU149569
2400	Cycloglypha thrasibulus	Pyrginae	02-SRNP-3233	MHAHE124-05	GU149568
2401	Cycloglypha thrasibulus	Pyrginae	04-SRNP-3850	MHAHD060-05	GU161392
2402	Cycloglypha thrasibulus	Pyrginae	04-SRNP-14593	MHAHD059-05	GU161391
2403	Cycloglypha thrasibulus	Pyrginae	04-SRNP-55588	MHAHD057-05	GU161393
2404	Cycloglypha thrasibulus	Pyrginae	05-SRNP-55022	MHAHD053-05	GU161388
2405	Cycloglypha thrasibulus	Pyrginae	04-SRNP-15456	MHAHD052-05	GU161387
2406	Cycloglypha thrasibulus	Pyrginae	02-SRNP-32143	MHAHE128-05	GU149571
2407	Cycloglypha thrasibulus	Pyrginae	99-SRNP-18551	CSRII190-04	DQ292227

Tree Order	Species	Subfamily	ACG Sampleid	BOLD Processid	Genbank Accession
2408	Cycloglypha thrasibulus	Pyrginae	02-SRNP-31149	MHAHE126-05	GU149572
2409	Cycloglypha thrasibulus	Pyrginae	01-SRNP-4284	CSRII191-04	DQ292228
2410	Cycloglypha thrasibulus	Pyrginae	04-SRNP-45636	MHAHD054-05	GU161390
2411	Cycloglypha thrasibulus	Pyrginae	07-SRNP-23056	MHMXT191-08	JF762002
2412	Cycloglypha thrasibulus	Pyrginae	04-SRNP-15458	MHAHD055-05	GU161389
2413	Cycloglypha thrasibulus	Pyrginae	06-SRNP-19660	MHMXH864-07	JF760632
2414	Cycloglypha thrasibulus	Pyrginae	08-SRNP-56190	MHMXX1089-09	JF777822
2415	Cycloglypha thrasibulus	Pyrginae	08-SRNP-56183	MHMXX1090-09	JF777823
2416	Cycloglypha thrasibulus	Pyrginae	08-SRNP-21510	MHMXX1091-09	JF777824
2417	Cycloglypha thrasibulus	Pyrginae	08-SRNP-56623	MHMXX592-09	JF777819
2418	Cycloglypha thrasibulus	Pyrginae	08-SRNP-57718	MHMXX593-09	JF777820
2419	Cycloglypha thrasibulus	Pyrginae	08-SRNP-36360	MHMXX594-09	HQ992119
2420	Ebrietas evanidus	Pyrginae	05-SRNP-24244	MHAHF629-06	GU150388
2421	Ebrietas evanidus	Pyrginae	06-SRNP-3928	MHAHI182-06	GU155957
2422	Ebrietas evanidus	Pyrginae	08-SRNP-4776	MHMXX602-09	JF777895
2423	Chiomara mithrax	Pyrginae	08-SRNP-71174	MHMXX1063-09	JF777766
2424	Camptopleura theramenes	Pyrginae	05-SRNP-23809	MHAHF649-06	GU150305
2425	Camptopleura theramenes	Pyrginae	06-SRNP-46916	MHAHJ872-07	JF752496
2426	Camptopleura auxoDHJ01	Pyrginae	05-SRNP-43216	MHAHF676-06	GU150304
2427	Camptopleura auxoDHJ01	Pyrginae	82-SRNP-324	CSRII174-04	DQ291963
2428	Camptopleura auxoDHJ01	Pyrginae	06-SRNP-65116	MHAHJ862-07	JF752495
2429	Camptopleura auxoDHJ01	Pyrginae	07-SRNP-42068	MHMXP203-08	JF761749
2430	Camptopleura auxoDHJ01	Pyrginae	07-SRNP-42282	MHMXP208-08	JF761750
2431	Camptopleura auxoDHJ01	Pyrginae	08-SRNP-71022	MHMXX1092-09	JF777713
2432	Camptopleura auxoDHJ02	Pyrginae	03-SRNP-31276	MHAHI242-06	GU155852
2433	Camptopleura auxoDHJ02	Pyrginae	07-SRNP-41965	MHMXP204-08	JF761753
2434	Camptopleura auxoDHJ02	Pyrginae	07-SRNP-24033	MHMXR047-08	JF761751
2435	Camptopleura auxoDHJ02	Pyrginae	07-SRNP-42067	MHMXP214-08	JF761754
2436	Camptopleura auxoDHJ02	Pyrginae	07-SRNP-42191	MHMXP205-08	JF761752
2437	Camptopleura auxoDHJ02	Pyrginae	06-SRNP-42908	MHAHI593-06	GU155853
2438	Camptopleura auxoDHJ02	Pyrginae	04-SRNP-40680	CSRII173-04	DQ291962
2439	Camptopleura auxoDHJ02	Pyrginae	05-SRNP-43763	MHAHF695-06	GU150303
2440	Camptopleura auxoDHJ02	Pyrginae	07-SRNP-40646	MHAHK399-07	JF760459
2441	Camptopleura auxoDHJ02	Pyrginae	08-SRNP-65495	MHMXX1093-09	GU666353
2442	Camptopleura auxoDHJ02	Pyrginae	08-SRNP-65880	MHMXX588-09	JF777714
2443	Mylon salvia	Pyrginae	06-SRNP-1769	MHAHG723-06	GU151480
2444	Mylon salvia	Pyrginae	02-SRNP-16859	CSCR135-04	DQ292641
2445	Mylon salvia	Pyrginae	02-SRNP-27057	MHAHK474-07	JF760873
2446	Mylon salvia	Pyrginae	02-SRNP-14878	MHAHK476-07	JF760875
2447	Mylon salvia	Pyrginae	05-SRNP-32566	MHAHF718-06	GU150550
2448	Mylon salvia	Pyrginae	02-SRNP-14879	CSCR134-04	DQ292640
2449	Mylon salvia	Pyrginae	02-SRNP-14882	MHAHK473-07	JF760872
2450	Mylon salvia	Pyrginae	02-SRNP-14815	MHAHK472-07	JF760871
2451	Mylon salvia	Pyrginae	06-SRNP-22322	MHAHJ762-07	JF752935
2452	Mylon salvia	Pyrginae	03-SRNP-3274	MHAHK471-07	JF760870
2453	Mylon salvia	Pyrginae	02-SRNP-14880	MHAHK475-07	JF760874
2454	Mylon salvia	Pyrginae	02-SRNP-14883	MHAHK477-07	JF760876
2455	Mylon salvia	Pyrginae	02-SRNP-14884	MHAHK478-07	JF760877
2456	Mylon maimon	Pyrginae	06-SRNP-65117	MHAHJ884-07	JF752934
2457	Mylon maimon	Pyrginae	04-SRNP-15734	MHAHD687-05	GU161669
2458	Mylon maimon	Pyrginae	04-SRNP-15726	MHAHD685-05	GU161666
2459	Mylon maimon	Pyrginae	06-SRNP-33362	MHAHI496-06	GU156002
2460	Mylon maimon	Pyrginae	06-SRNP-55541	MHAHG326-06	GU151478
2461	Mylon maimon	Pyrginae	05-SRNP-33196	MHAHF717-06	GU150548
2462	Mylon maimon	Pyrginae	05-SRNP-45185	MHAHF280-06	GU150547
2463	Mylon maimon	Pyrginae	04-SRNP-15661	MHAHD688-05	GU161668

Tree Order	Species	Subfamily	ACG Sampleid	BOLD Processid	Genbank Accession
2464	Mylon maimon	Pyrginae	04-SRNP-15653	MHAHD686-05	GU161667
2465	Mylon maimon	Pyrginae	02-SRNP-33927	CSCR133-04	DQ292637
2466	Mylon maimon	Pyrginae	04-SRNP-25458	MHAHF287-06	GU150546
2467	Mylon maimon	Pyrginae	05-SRNP-34629	MHAHF675-06	GU150549
2468	Mylon maimon	Pyrginae	96-SRNP-9924	CSCR132-04	DQ292636
2469	Mylon maimon	Pyrginae	05-SRNP-66553	MHAHG327-06	GU151477
2470	Mylon maimon	Pyrginae	07-SRNP-60850	MHMXR005-08	JF762359
2471	Mylon lassia	Pyrginae	04-SRNP-47729	MHAHD699-05	GU161659
2472	Mylon lassia	Pyrginae	04-SRNP-49068	MHAHD703-05	GU161664
2473	Mylon lassia	Pyrginae	05-SRNP-46313	MHAHF269-06	GU150543
2474	Mylon lassia	Pyrginae	07-SRNP-45249	MHMXP219-08	JF762358
2475	Mylon lassia	Pyrginae	09-SRNP-22793	MHMYG2378-10	HM885801
2476	Mylon lassia	Pyrginae	06-SRNP-32968	MHAHI166-06	GU156001
2477	Mylon lassia	Pyrginae	04-SRNP-47995	MHAHD705-05	GU161665
2478	Mylon lassia	Pyrginae	05-SRNP-45388	MHAHF271-06	GU150544
2479	Mylon lassia	Pyrginae	06-SRNP-33008	MHAHI167-06	GU156000
2480	Mylon lassia	Pyrginae	05-SRNP-32431	MHAHF273-06	GU150542
2481	Mylon lassia	Pyrginae	05-SRNP-35383	MHAHF272-06	GU150541
2482	Mylon lassia	Pyrginae	05-SRNP-45964	MHAHF270-06	GU150545
2483	Mylon lassia	Pyrginae	04-SRNP-48829	MHAHD702-05	GU161663
2484	Mylon lassia	Pyrginae	04-SRNP-49039	MHAHD700-05	GU161660
2485	Mylon lassia	Pyrginae	06-SRNP-31605	MHAHH794-06	GU155433
2486	Mylon lassia	Pyrginae	02-SRNP-28954	CSCR131-04	DQ292635
2487	Mylon lassia	Pyrginae	02-SRNP-28773	CSCR130-04	DQ292634
2488	Mylon lassia	Pyrginae	04-SRNP-32066	MHAHD701-05	GU161662
2489	Mylon lassia	Pyrginae	04-SRNP-35482	MHAHD706-05	GU161661
2490	Mylon lassia	Pyrginae	07-SRNP-33635	MHMXT189-08	JF762357
2491	Mylon lassia	Pyrginae	09-SRNP-36601	MHMYE1533-09	HM391093
2492	Mylon lassia	Pyrginae	09-SRNP-22448	MHMYG2379-10	HM885802
2493	Timochares trifasciata	Pyrginae	05-SRNP-13161	MHAHF362-06	GU150931
2494	Timochares trifasciata	Pyrginae	05-SRNP-12097	MHAHF364-06	GU150934
2495	Timochares trifasciata	Pyrginae	03-SRNP-12286	MHAHD517-05	GU161892
2496	Timochares trifasciata	Pyrginae	05-SRNP-12096	MHAHF361-06	GU150930
2497	Timochares trifasciata	Pyrginae	05-SRNP-13158	MHAHF363-06	GU150929
2498	Timochares trifasciata	Pyrginae	05-SRNP-13159	MHAHF366-06	GU150933
2499	Timochares trifasciata	Pyrginae	03-SRNP-13719	MHAHD518-05	GU161893
2500	Timochares trifasciata	Pyrginae	05-SRNP-40802	MHAHF367-06	GU150932
2501	Timochares trifasciata	Pyrginae	05-SRNP-13162	MHAHF360-06	GU150928
2502	Timochares trifasciata	Pyrginae	04-SRNP-14040	MHAHE075-05	GU150124
2503	Timochares trifasciata	Pyrginae	04-SRNP-14041	MHAHE074-05	GU150123
2504	Timochares trifasciata	Pyrginae	03-SRNP-960	MHAHD516-05	GU161895
2505	Timochares trifasciata	Pyrginae	03-SRNP-14113	MHAHD515-05	GU161894
2506	Timochares trifasciata	Pyrginae	05-SRNP-19552	MHAHE375-05	GU150122
2507	Timochares trifasciata	Pyrginae	08-SRNP-72653	MHMXY1078-09	GU666460
2508	Helias cama	Pyrginae	01-SRNP-9588	CSRII213-04	DQ292559
2509	Helias cama	Pyrginae	05-SRNP-30554	MHAHF085-06	GU150494
2510	Helias cama	Pyrginae	05-SRNP-6813	MHAHF819-06	GU150499
2511	Helias cama	Pyrginae	06-SRNP-41760	MHAHI193-06	GU155981
2512	Helias cama	Pyrginae	07-SRNP-20080	MHAHK358-07	JF760781
2513	Helias cama	Pyrginae	08-SRNP-70554	MHMXX711-09	JF778025
2514	Helias cama	Pyrginae	02-SRNP-15049	CSRII214-04	DQ292560
2515	Helias cama	Pyrginae	04-SRNP-31168	MHAHD061-05	GU161536
2516	Helias cama	Pyrginae	05-SRNP-30170	MHAHD062-05	GU161535
2517	Helias cama	Pyrginae	04-SRNP-30824	MHAHD063-05	GU161534
2518	Helias cama	Pyrginae	05-SRNP-31048	MHAHF082-06	GU150497
2519	Helias cama	Pyrginae	05-SRNP-31049	MHAHF083-06	GU150498

Tree Order	Species	Subfamily	ACG Sampleid	BOLD Processid	Genbank Accession
2520	Helias cama	Pyrginae	05-SRNP-31193	MHAHF084-06	GU150495
2521	Helias cama	Pyrginae	05-SRNP-3359	MHAHF086-06	GU150492
2522	Helias cama	Pyrginae	05-SRNP-3199	MHAHF087-06	GU150493
2523	Helias cama	Pyrginae	05-SRNP-2671	MHAHF088-06	GU150496
2524	Helias cama	Pyrginae	06-SRNP-453	MHMXA572-06	JF760778
2525	Helias cama	Pyrginae	06-SRNP-2367	MHAHG771-06	GU151431
2526	Helias cama	Pyrginae	07-SRNP-30806	MHAHK356-07	JF760779
2527	Helias cama	Pyrginae	06-SRNP-22077	MHAHK357-07	JF760780
2528	Helias cama	Pyrginae	07-SRNP-20157	MHAHK359-07	JF760782
2529	Helias cama	Pyrginae	07-SRNP-65129	MHAHL156-07	JF762275
2530	Helias cama	Pyrginae	08-SRNP-2560	MHMXX1098-09	JF778026
2531	Helias cama	Pyrginae	08-SRNP-2431	MHMXX1186-09	JF778027
2532	Helias cama	Pyrginae	08-SRNP-72018	MHMXX595-09	JF778024
2533	Helias cama	Pyrginae	09-SRNP-30202	MHMYC504-09	GU649831
2534	Helias cama	Pyrginae	09-SRNP-30198	MHMYC506-09	GU649833
2535	Ebrietas osyris	Pyrginae	05-SRNP-20008	MHAHD385-05	GU161467
2536	Ebrietas osyris	Pyrginae	05-SRNP-34654	MHAHF633-06	GU150393
2537	Ebrietas osyris	Pyrginae	08-SRNP-70289	MHMXW544-09	JF753875
2538	Ebrietas osyris	Pyrginae	08-SRNP-72341	MHMXX587-09	JF777896
2539	Cycloglypha enega	Pyrginae	08-SRNP-32246	MHMXX589-09	JF777818
2540	Ebrietas anacreonDHJ04	Pyrginae	07-SRNP-61246	MHMXR045-08	JF762160
2541	Ebrietas anacreonDHJ04	Pyrginae	07-SRNP-66037	MHMXR046-08	JF762159
2542	Ebrietas anacreonDHJ02	Pyrginae	07-SRNP-30890	MHMXH856-07	JF760664
2543	Ebrietas anacreonDHJ02	Pyrginae	01-SRNP-17938	XAA737-04	DQ292366
2544	Ebrietas anacreonDHJ02	Pyrginae	93-SRNP-5851	XAA740-04	DQ292369
2545	Ebrietas anacreonDHJ02	Pyrginae	06-SRNP-30797	MHAHG249-06	GU151390
2546	Ebrietas anacreonDHJ02	Pyrginae	04-SRNP-31276	CSRII413-04	DQ292364
2547	Ebrietas anacreonDHJ02	Pyrginae	04-SRNP-30931	CSRII414-04	DQ292365
2548	Ebrietas anacreonDHJ02	Pyrginae	06-SRNP-59644	MHMXH867-07	JF760663
2549	Ebrietas anacreonDHJ02	Pyrginae	07-SRNP-31391	MHAHL165-07	JF762157
2550	Ebrietas anacreonDHJ02	Pyrginae	06-SRNP-30796	MHAHG253-06	GU151391
2551	Ebrietas anacreonDHJ02	Pyrginae	06-SRNP-31143	MHAHG772-06	GU151389
2552	Ebrietas anacreonDHJ02	Pyrginae	06-SRNP-23020	MHAHJ829-07	JF752762
2553	Ebrietas anacreonDHJ02	Pyrginae	07-SRNP-59135	MHMXP207-08	JF762158
2554	Ebrietas anacreonDHJ02	Pyrginae	08-SRNP-23566	MHMXX599-09	JF777893
2555	Ebrietas anacreonDHJ02	Pyrginae	09-SRNP-30594	MHMYC505-09	GU649832
2556	Ebrietas anacreonDHJ03	Pyrginae	93-SRNP-5844	XAA746-04	DQ292375
2557	Ebrietas anacreonDHJ03	Pyrginae	01-SRNP-18080	CSRII370-04	DQ292362
2558	Ebrietas anacreonDHJ03	Pyrginae	94-SRNP-282	XAA745-04	DQ292374
2559	Ebrietas anacreonDHJ03	Pyrginae	05-SRNP-33161	MHAHF781-06	GU150390
2560	Ebrietas anacreonDHJ03	Pyrginae	06-SRNP-30257	MHAHG252-06	GU151396
2561	Ebrietas anacreonDHJ03	Pyrginae	05-SRNP-24345	MHAHF631-06	GU150392
2562	Ebrietas anacreonDHJ03	Pyrginae	04-SRNP-32065	MHAHD798-05	GU161466
2563	Ebrietas anacreonDHJ03	Pyrginae	01-SRNP-18079	XAA752-04	DQ292380
2564	Ebrietas anacreonDHJ03	Pyrginae	93-SRNP-5879	XAA750-04	DQ292378
2565	Ebrietas anacreonDHJ03	Pyrginae	02-SRNP-32566	XAA808-04	DQ292381
2566	Ebrietas anacreonDHJ03	Pyrginae	93-SRNP-5847	XAA743-04	DQ292372
2567	Ebrietas anacreonDHJ03	Pyrginae	93-SRNP-5836	XAA741-04	DQ292370
2568	Ebrietas anacreonDHJ03	Pyrginae	93-SRNP-5928	XAA744-04	DQ292373
2569	Ebrietas anacreonDHJ03	Pyrginae	02-SRNP-28985	CSRII389-04	DQ292363
2570	Ebrietas anacreonDHJ03	Pyrginae	93-SRNP-5876	XAA742-04	DQ292371
2571	Ebrietas anacreonDHJ03	Pyrginae	93-SRNP-5846	XAA739-04	DQ292368
2572	Ebrietas anacreonDHJ03	Pyrginae	93-SRNP-6182	XAA738-04	DQ292367
2573	Ebrietas anacreonDHJ03	Pyrginae	93-SRNP-5839	XAA747-04	DQ292376
2574	Ebrietas anacreonDHJ03	Pyrginae	93-SRNP-5629	XAA749-04	DQ292377
2575	Ebrietas anacreonDHJ03	Pyrginae	93-SRNP-5840	XAA751-04	DQ292379

Tree Order	Species	Subfamily	ACG Sampleid	BOLD Processid	Genbank Accession
2576	Ebrietas anacreonDHJ03	Pyrginae	05-SRNP-31047	MHAHF095-06	GU150389
2577	Ebrietas anacreonDHJ03	Pyrginae	05-SRNP-66020	MHAHF630-06	GU150391
2578	Ebrietas anacreonDHJ03	Pyrginae	06-SRNP-30135	MHAHG250-06	GU151394
2579	Ebrietas anacreonDHJ03	Pyrginae	06-SRNP-30138	MHAHG251-06	GU151393
2580	Ebrietas anacreonDHJ03	Pyrginae	06-SRNP-30137	MHAHG254-06	GU151397
2581	Ebrietas anacreonDHJ03	Pyrginae	06-SRNP-55045	MHAHG255-06	GU151395
2582	Ebrietas anacreonDHJ03	Pyrginae	06-SRNP-30798	MHAHG775-06	GU151392
2583	Ebrietas anacreonDHJ03	Pyrginae	06-SRNP-32332	MHAHI180-06	GU155958
2584	Ebrietas anacreonDHJ03	Pyrginae	07-SRNP-55471	MHAHK319-07	JF760665
2585	Ebrietas anacreonDHJ03	Pyrginae	08-SRNP-23847	MHMXX600-09	JF777894
2586	Ebrietas anacreonDHJ03	Pyrginae	09-SRNP-71739	MHMYE1568-09	HM391128
2587	Ebrietas anacreonDHJ03	Pyrginae	09-SRNP-57295	MHMYG2475-10	HM885902
2588	Ebrietas anacreonDHJ03	Pyrginae	09-SRNP-12023	MHMYG2042-10	HM885444
2589	Potamanaxas Burns01	Pyrginae	91-SRNP-132	CSCR209-04	DQ293099
2590	Potamanaxas Burns01	Pyrginae	07-SRNP-31875	MHMXN368-07	JF762690
2591	Potamanaxas Burns02	Pyrginae	07-SRNP-65900	MHMXR939-08	JF762692
2592	Potamanaxas Burns02	Pyrginae	07-SRNP-65901	MHMXR940-08	JF762691
2593	Potamanaxas Burns02	Pyrginae	09-SRNP-71252	MHMYE1543-09	HM391103
2594	Mylon pelopidas	Pyrginae	00-SRNP-6099	MHAHK467-07	JF760866
2595	Mylon pelopidas	Pyrginae	00-SRNP-6369	MHAHK464-07	JF760863
2596	Mylon pelopidas	Pyrginae	00-SRNP-6128	MHAHK460-07	JF760859
2597	Mylon pelopidas	Pyrginae	06-SRNP-12608	MHAHG800-06	GU151479
2598	Mylon pelopidas	Pyrginae	01-SRNP-17181	MHAHK469-07	JF760868
2599	Mylon pelopidas	Pyrginae	03-SRNP-649	CSCR371-04	DQ292638
2600	Mylon pelopidas	Pyrginae	99-SRNP-6171	MHAHK462-07	JF760861
2601	Mylon pelopidas	Pyrginae	97-SRNP-2965	MHAHK470-07	JF760869
2602	Mylon pelopidas	Pyrginae	97-SRNP-3873	MHAHK463-07	JF760862
2603	Mylon pelopidas	Pyrginae	02-SRNP-10065	MHAHK468-07	JF760867
2604	Mylon pelopidas	Pyrginae	03-SRNP-12052	CSCR372-04	DQ292639
2605	Mylon pelopidas	Pyrginae	93-SRNP-4445	MHAHK465-07	JF760864
2606	Mylon pelopidas	Pyrginae	05-SRNP-55075	MHAHD689-05	GU161670
2607	Mylon pelopidas	Pyrginae	97-SRNP-54	MHAHK459-07	JF760858
2608	Mylon pelopidas	Pyrginae	94-SRNP-9689	MHAHK461-07	JF760860
2609	Mylon pelopidas	Pyrginae	95-SRNP-6458	MHAHK466-07	JF760865
2610	Mylon pelopidas	Pyrginae	07-SRNP-55508	MHAHK715-07	JF760853
2611	Mylon pelopidas	Pyrginae	07-SRNP-55502	MHAHK716-07	JF760854
2612	Mylon pelopidas	Pyrginae	07-SRNP-55540	MHAHK717-07	JF760855
2613	Mylon pelopidas	Pyrginae	07-SRNP-55977	MHAHK718-07	JF760856
2614	Mylon pelopidas	Pyrginae	07-SRNP-55365	MHAHK719-07	JF760857
2615	Mylon pelopidas	Pyrginae	08-SRNP-55556	MHMXX886-09	JF778091
2616	Potamanaxas Burns03	Pyrginae	07-SRNP-32749	MHMXN250-07	JF762694
2617	Potamanaxas Burns03	Pyrginae	07-SRNP-32750	MHMXN367-07	JF762693
2618	Potamanaxas Burns03	Pyrginae	07-SRNP-32792	MHMXO968-08	JF762695
2619	Sostrata pusilla	Pyrginae	05-SRNP-7425	MHAHF790-06	GU150850
2620	Sostrata pusilla	Pyrginae	07-SRNP-30539	MHMXH887-07	JF761171
2621	Sostrata pusilla	Pyrginae	06-SRNP-4305	MHAHI196-06	GU156310
2622	Sostrata pusilla	Pyrginae	06-SRNP-2403	MHAHG799-06	GU151644
2623	Sostrata pusilla	Pyrginae	05-SRNP-1719	MHAHF008-06	GU150849
2624	Sostrata pusilla	Pyrginae	05-SRNP-1859	MHAHF005-06	GU150848
2625	Sostrata pusilla	Pyrginae	05-SRNP-41675	MHAHE935-06	GU150006
2626	Sostrata pusilla	Pyrginae	04-SRNP-3394	MHAHD075-05	GU161849
2627	Sostrata pusilla	Pyrginae	02-SRNP-18306	CSCR249-04	DQ293325
2628	Sostrata pusilla	Pyrginae	05-SRNP-42029	MHAHE934-06	GU150005
2629	Sostrata pusilla	Pyrginae	07-SRNP-1449	MHAHK397-07	JF761172
2630	Sostrata pusilla	Pyrginae	07-SRNP-2998	MHAHL171-07	JF762927
2631	Sostrata pusilla	Pyrginae	08-SRNP-30865	MHMXX1099-09	JF778472

Tree Order	Species	Subfamily	ACG Sampleid	BOLD Processid	Genbank Accession
2632	Sostrata bifasciata nordica	Pyrginae	03-SRNP-1099	CSCR415-04	DQ293323
2633	Sostrata bifasciata nordica	Pyrginae	05-SRNP-2227	MHAHF007-06	GU150840
2634	Sostrata bifasciata nordica	Pyrginae	08-SRNP-844	MHMXW420-09	JF754163
2635	Sostrata bifasciata nordica	Pyrginae	07-SRNP-1567	MHAHK395-07	JF761169
2636	Sostrata bifasciata nordica	Pyrginae	07-SRNP-1932	MHAHL172-07	JF762922
2637	Sostrata bifasciata nordica	Pyrginae	07-SRNP-58638	MHMXP216-08	JF762924
2638	Sostrata bifasciata nordica	Pyrginae	06-SRNP-3708	MHAHG798-06	GU151640
2639	Sostrata bifasciata nordica	Pyrginae	06-SRNP-3696	MHAHH427-06	GU155494
2640	Sostrata bifasciata nordica	Pyrginae	05-SRNP-213	MHAHD072-05	GU161834
2641	Sostrata bifasciata nordica	Pyrginae	05-SRNP-214	MHAHF004-06	GU150838
2642	Sostrata bifasciata nordica	Pyrginae	04-SRNP-22231	MHAHD086-05	GU161846
2643	Sostrata bifasciata nordica	Pyrginae	04-SRNP-61492	MHAHD085-05	GU161845
2644	Sostrata bifasciata nordica	Pyrginae	04-SRNP-61382	MHAHD084-05	GU161842
2645	Sostrata bifasciata nordica	Pyrginae	04-SRNP-3640	MHAHD083-05	GU161844
2646	Sostrata bifasciata nordica	Pyrginae	04-SRNP-1343	MHAHD082-05	GU161843
2647	Sostrata bifasciata nordica	Pyrginae	04-SRNP-22116	MHAHD076-05	GU161840
2648	Sostrata bifasciata nordica	Pyrginae	04-SRNP-2136	MHAHD074-05	GU161839
2649	Sostrata bifasciata nordica	Pyrginae	05-SRNP-211	MHAHD073-05	GU161841
2650	Sostrata bifasciata nordica	Pyrginae	04-SRNP-50041	MHAHD070-05	GU161836
2651	Sostrata bifasciata nordica	Pyrginae	05-SRNP-212	MHAHD067-05	GU161835
2652	Sostrata bifasciata nordica	Pyrginae	05-SRNP-296	MHAHF003-06	GU150837
2653	Sostrata bifasciata nordica	Pyrginae	05-SRNP-2711	MHAHE940-06	GU149999
2654	Sostrata bifasciata nordica	Pyrginae	04-SRNP-47237	MHAHD071-05	GU161838
2655	Sostrata bifasciata nordica	Pyrginae	03-SRNP-5074	CSCR416-04	DQ293324
2656	Sostrata bifasciata nordica	Pyrginae	08-SRNP-24554	MHMYB161-09	GU649080
2657	Sostrata bifasciata nordica	Pyrginae	04-SRNP-23519	MHAHD087-05	GU161837
2658	Sostrata bifasciata nordica	Pyrginae	04-SRNP-24494	MHAHD088-05	GU161847
2659	Sostrata bifasciata nordica	Pyrginae	04-SRNP-22118	MHAHD089-05	GU161848
2660	Sostrata bifasciata nordica	Pyrginae	05-SRNP-47046	MHAHE933-06	GU150000
2661	Sostrata bifasciata nordica	Pyrginae	05-SRNP-4845	MHAHE936-06	GU150004
2662	Sostrata bifasciata nordica	Pyrginae	05-SRNP-32215	MHAHE937-06	GU150001
2663	Sostrata bifasciata nordica	Pyrginae	05-SRNP-46868	MHAHE938-06	GU150002
2664	Sostrata bifasciata nordica	Pyrginae	05-SRNP-3026	MHAHE939-06	GU150003
2665	Sostrata bifasciata nordica	Pyrginae	05-SRNP-45186	MHAHF001-06	GU150836
2666	Sostrata bifasciata nordica	Pyrginae	05-SRNP-2678	MHAHF002-06	GU150835
2667	Sostrata bifasciata nordica	Pyrginae	05-SRNP-31549	MHAHF006-06	GU150839
2668	Sostrata bifasciata nordica	Pyrginae	05-SRNP-22997	MHAHF009-06	GU150842
2669	Sostrata bifasciata nordica	Pyrginae	05-SRNP-40774	MHAHF010-06	GU150841
2670	Sostrata bifasciata nordica	Pyrginae	05-SRNP-707	MHAHF011-06	GU150845
2671	Sostrata bifasciata nordica	Pyrginae	05-SRNP-2270	MHAHF012-06	GU150844
2672	Sostrata bifasciata nordica	Pyrginae	05-SRNP-295	MHAHF013-06	GU150843
2673	Sostrata bifasciata nordica	Pyrginae	05-SRNP-6575	MHAHF789-06	GU150846
2674	Sostrata bifasciata nordica	Pyrginae	05-SRNP-33197	MHAHF816-06	GU150847
2675	Sostrata bifasciata nordica	Pyrginae	06-SRNP-1499	MHAHG239-06	GU151643
2676	Sostrata bifasciata nordica	Pyrginae	06-SRNP-55120	MHAHG240-06	GU151642
2677	Sostrata bifasciata nordica	Pyrginae	06-SRNP-3033	MHAHG797-06	GU151641
2678	Sostrata bifasciata nordica	Pyrginae	06-SRNP-7519	MHAHJ440-07	JF753155
2679	Sostrata bifasciata nordica	Pyrginae	06-SRNP-23019	MHAHJ485-07	JF753156
2680	Sostrata bifasciata nordica	Pyrginae	07-SRNP-637	MHAHK396-07	JF761170
2681	Sostrata bifasciata nordica	Pyrginae	07-SRNP-36575	MHMXO989-08	JF762926
2682	Sostrata bifasciata nordica	Pyrginae	07-SRNP-58637	MHMXP215-08	JF762925
2683	Sostrata bifasciata nordica	Pyrginae	07-SRNP-36790	MHMXR002-08	JF762923
2684	Sostrata bifasciata nordica	Pyrginae	08-SRNP-2265	MHMXW421-09	JF754164
2685	Sostrata bifasciata nordica	Pyrginae	08-SRNP-1977	MHMXW422-09	JF754165
2686	Sostrata bifasciata nordica	Pyrginae	07-SRNP-60822	MHMXW423-09	JF754166
2687	Sostrata bifasciata nordica	Pyrginae	08-SRNP-35027	MHMXW425-09	JF754167

Tree Order	Species	Subfamily	ACG Sampleid	BOLD Processid	Genbank Accession
2688	Sostrata bifasciata nordica	Pyrginae	08-SRNP-36391	MHMXX598-09	JF778471
2689	Sostrata bifasciata nordica	Pyrginae	09-SRNP-30271	MHMYC545-09	GU649798
2690	Sostrata bifasciata nordica	Pyrginae	09-SRNP-35749	MHMYE1547-09	HM391107
2691	Sostrata bifasciata nordica	Pyrginae	09-SRNP-35141	MHMYE1548-09	HM391108
2692	Gesta gesta	Pyrginae	05-SRNP-62098	MHAHF687-06	GU150448
2693	Gesta gesta	Pyrginae	96-SRNP-10372	CSRII197-04	DQ292509
2694	Gesta gesta	Pyrginae	06-SRNP-18550	MHAHK322-07	JF760737
2695	Gesta gesta	Pyrginae	05-SRNP-61955	MHAHF820-06	GU150446
2696	Gesta gesta	Pyrginae	93-SRNP-5391	CSRII196-04	DQ292508
2697	Gesta gesta	Pyrginae	05-SRNP-63396	MHAHF688-06	GU150449
2698	Gesta gesta	Pyrginae	06-SRNP-18548	MHMXH865-07	JF760735
2699	Gesta gesta	Pyrginae	06-SRNP-19618	MHAHI542-06	GU155976
2700	Gesta gesta	Pyrginae	06-SRNP-18549	MHMXH868-07	JF760736
2701	Gesta gesta	Pyrginae	04-SRNP-15977	MHAHF116-06	GU150445
2702	Gesta gesta	Pyrginae	05-SRNP-63399	MHAHF653-06	GU150447
2703	Gesta gesta	Pyrginae	07-SRNP-61370	MHMXX1096-09	JF777976
2704	Gesta gesta	Pyrginae	07-SRNP-61369	MHMXX1097-09	JF777977
2705	Erynnis tristis	Pyrginae	07-SRNP-55295	MHMXK031-07	JF762246
2706	Erynnis tristis	Pyrginae	09-SRNP-55044	MHMYC438-09	GU649893
2707	Erynnis tristis	Pyrginae	07-SRNP-55321	MHMXK032-07	JF762245
2708	Erynnis tristis	Pyrginae	02-SRNP-10325	MHAHI218-06	GU155970
2709	Erynnis tristis	Pyrginae	09-SRNP-55140	MHMYC437-09	HM893840
2710	Erynnis tristis	Pyrginae	06-SRNP-57856	MHAHJ881-07	JF752780
2711	Erynnis tristis	Pyrginae	06-SRNP-57862	MHAHJ880-07	JF752779
2712	Erynnis tristis	Pyrginae	06-SRNP-57467	MHAHJ879-07	JF752778
2713	Erynnis tristis	Pyrginae	06-SRNP-57463	MHAHJ686-07	JF752776
2714	Erynnis tristis	Pyrginae	06-SRNP-57863	MHAHJ685-07	JF752775
2715	Erynnis tristis	Pyrginae	06-SRNP-57940	MHAHJ570-07	JF752774
2716	Erynnis tristis	Pyrginae	01-SRNP-15342	MHAHI221-06	GU155965
2717	Erynnis tristis	Pyrginae	05-SRNP-64103	MHAHG680-06	GU151406
2718	Erynnis tristis	Pyrginae	05-SRNP-64104	MHAHG679-06	GU151407
2719	Erynnis tristis	Pyrginae	04-SRNP-12369	MHAHE068-05	GU149665
2720	Erynnis tristis	Pyrginae	01-SRNP-16043	MHAHI222-06	GU155966
2721	Erynnis tristis	Pyrginae	05-SRNP-13296	MHAHF359-06	GU150442
2722	Erynnis tristis	Pyrginae	06-SRNP-57870	MHAHJ687-07	JF752777
2723	Erynnis tristis	Pyrginae	02-SRNP-10318	MHAHI217-06	GU155968
2724	Erynnis tristis	Pyrginae	08-SRNP-58579	MHMYC439-09	GU649894
2725	Erynnis tristis	Pyrginae	06-SRNP-57465	MHAHJ882-07	JF752781
2726	Erynnis tristis	Pyrginae	05-SRNP-64102	MHAHG681-06	GU151405
2727	Erynnis tristis	Pyrginae	02-SRNP-10323	MHAHI220-06	GU155969
2728	Erynnis tristis	Pyrginae	02-SRNP-10320	MHAHI219-06	GU155967
2729	Erynnis tristis	Pyrginae	08-SRNP-58610	MHMYC440-09	
2730	Erynnis tristis	Pyrginae	08-SRNP-58615	MHMYE887-09	GU653714
2731	Anastrus sempiternus	Pyrginae	01-SRNP-17323	CSCR012-04	DQ291808
2732	Anastrus sempiternus	Pyrginae	08-SRNP-2645	MHMXS077-08	JF761380
2733	Anastrus sempiternus	Pyrginae	07-SRNP-4363	MHMXR769-08	JF761377
2734	Anastrus sempiternus	Pyrginae	07-SRNP-4306	MHMXR768-08	JF761378
2735	Anastrus sempiternus	Pyrginae	07-SRNP-32152	MHAHL249-07	JF761376
2736	Anastrus sempiternus	Pyrginae	07-SRNP-12147	MHMXK431-07	JF761381
2737	Anastrus sempiternus	Pyrginae	07-SRNP-55616	MHAHK329-07	JF760230
2738	Anastrus sempiternus	Pyrginae	06-SRNP-60163	MHMXH860-07	JF760228
2739	Anastrus sempiternus	Pyrginae	06-SRNP-60206	MHMXH854-07	JF760229
2740	Anastrus sempiternus	Pyrginae	06-SRNP-57897	MHAHJ886-07	JF752354
2741	Anastrus sempiternus	Pyrginae	06-SRNP-57895	MHAHJ885-07	JF752353
2742	Anastrus sempiternus	Pyrginae	06-SRNP-57849	MHAHJ819-07	JF752352
2743	Anastrus sempiternus	Pyrginae	06-SRNP-23197	MHAHJ752-07	JF752351

Tree Order	Species	Subfamily	ACG Sampleid	BOLD Processid	Genbank Accession
2744	Anastrus sempiternus	Pyrginae	05-SRNP-64291	MHAHF632-06	GU150171
2745	Anastrus sempiternus	Pyrginae	05-SRNP-64292	MHAHF624-06	GU150170
2746	Anastrus sempiternus	Pyrginae	05-SRNP-1770	MHAHF179-06	GU150169
2747	Anastrus sempiternus	Pyrginae	04-SRNP-4595	MHAHE182-05	GU149355
2748	Anastrus sempiternus	Pyrginae	08-SRNP-66006	MHMXY1050-09	GU666485
2749	Anastrus sempiternus	Pyrginae	08-SRNP-58583	MHMYC510-09	GU649829
2750	Anastrus sempiternus	Pyrginae	07-SRNP-4302	MHMXR767-08	JF761379
2751	Anastrus sempiternus	Pyrginae	01-SRNP-17736	CSCR013-04	DQ291809
2752	Anastrus sempiternus	Pyrginae	09-SRNP-57315	MHMYE1493-09	GU653509
2753	Clito aberrans	Pyrginae	06-SRNP-12726	MHAHG731-06	GU151305
2754	Clito aberrans	Pyrginae	07-SRNP-55359	MHAHK710-07	JF760544
2755	Clito aberrans	Pyrginae	05-SRNP-46256	MHAHF285-06	GU150338
2756	Clito aberrans	Pyrginae	08-SRNP-55841	MHMXW539-09	JF753825
2757	Clito aberrans	Pyrginae	99-SRNP-18483	CSCR070-04	DQ292155
2758	Clito aberrans	Pyrginae	94-SRNP-3152	CSCR069-04	DQ292154
2759	Clito aberrans	Pyrginae	05-SRNP-46254	MHAHF283-06	GU150336
2760	Clito aberrans	Pyrginae	05-SRNP-46249	MHAHF284-06	GU150337
2761	Clito aberrans	Pyrginae	06-SRNP-15483	MHAHH814-06	GU155334
2762	Clito aberrans	Pyrginae	07-SRNP-3664	MHMXO969-08	JF761952
2763	Clito aberrans	Pyrginae	07-SRNP-3662	MHMXO970-08	JF761951
2764	Clito aberrans	Pyrginae	07-SRNP-3660	MHMXO971-08	JF761950
2765	Clito aberrans	Pyrginae	08-SRNP-4314	MHMXX520-09	JF777769
2766	Clito aberrans	Pyrginae	08-SRNP-4317	MHMXX521-09	JF777770
2767	Clito aberrans	Pyrginae	08-SRNP-4315	MHMXX522-09	JF777771
2768	Clito aberrans	Pyrginae	08-SRNP-4313	MHMXX523-09	JF777772
2769	Clito Burns01	Pyrginae	05-SRNP-34516	MHAHG225-06	GU151306
2770	Clito Burns01	Pyrginae	01-SRNP-9229	CSCR560-04	DQ292156
2771	Clito Burns01	Pyrginae	01-SRNP-9304	CSCR561-04	DQ292157
2772	Clito Burns01	Pyrginae	04-SRNP-21031	CSRII189-04	DQ292158
2773	Clito Burns01	Pyrginae	08-SRNP-30288	MHMXT155-08	JF761953
2774	Clito Burns01	Pyrginae	08-SRNP-70430	MHMXX526-09	JF777773
2775	Anastrus neaeris	Pyrginae	05-SRNP-6078	MHAHF719-06	GU150168
2776	Anastrus neaeris	Pyrginae	07-SRNP-65050	MHAHL233-07	JF761372
2777	Anastrus neaeris	Pyrginae	05-SRNP-5537	MHAHF180-06	GU150167
2778	Anastrus neaeris	Pyrginae	04-SRNP-60781	MHAHE186-05	GU149353
2779	Anastrus neaeris	Pyrginae	04-SRNP-60867	MHAHE185-05	GU149352
2780	Anastrus neaeris	Pyrginae	04-SRNP-60780	MHAHE184-05	GU149350
2781	Anastrus neaeris	Pyrginae	02-SRNP-18593	CSCR011-04	DQ291807
2782	Anastrus neaeris	Pyrginae	07-SRNP-65052	MHAHL231-07	JF761370
2783	Anastrus neaeris	Pyrginae	04-SRNP-60339	MHAHE183-05	GU149349
2784	Anastrus neaeris	Pyrginae	06-SRNP-1371	MHAHG129-06	GU151045
2785	Anastrus neaeris	Pyrginae	06-SRNP-1373	MHAHG130-06	GU151047
2786	Anastrus neaeris	Pyrginae	06-SRNP-1372	MHAHG131-06	GU151046
2787	Anastrus neaeris	Pyrginae	07-SRNP-65051	MHAHL232-07	JF761371
2788	Anastrus neaeris	Pyrginae	07-SRNP-35858	MHMXP174-08	JF761375
2789	Anastrus neaeris	Pyrginae	08-SRNP-65477	MHMXS078-08	JF761374
2790	Anastrus neaeris	Pyrginae	08-SRNP-65478	MHMXS079-08	JF761373
2791	Anastrus neaeris	Pyrginae	08-SRNP-40484	MHMXW540-09	JF753651
2792	Anastrus neaeris	Pyrginae	08-SRNP-65476	MHMXW541-09	JF753652
2793	Anastrus neaeris	Pyrginae	08-SRNP-2106	MHMXW542-09	JF753653
2794	Anastrus neaeris	Pyrginae	02-SRNP-16276	CSCR010-04	DQ291806
2795	Anastrus neaeris	Pyrginae	04-SRNP-56411	MHAHE188-05	GU149354
2796	Anastrus neaeris	Pyrginae	04-SRNP-34562	MHAHE187-05	GU149351
2797	Anastrus neaeris	Pyrginae	08-SRNP-36419	MHMXX584-09	JF777579
2798	Anastrus neaeris	Pyrginae	09-SRNP-67369	MHMYE1495-09	GU653507
2799	Eracon cliniasDHJ02	Pyrginae	05-SRNP-1688	MHAHF337-06	GU150438

Tree Order	Species	Subfamily	ACG Sampleid	BOLD Processid	Genbank Accession
2800	Eracon cliniasDHJ02	Pyrginae	09-SRNP-40707	MHMYE1545-09	HM391105
2801	Eracon cliniasDHJ01	Pyrginae	05-SRNP-43583	MHAHF639-06	GU150436
2802	Eracon cliniasDHJ01	Pyrginae	05-SRNP-22364	MHAHF339-06	GU150427
2803	Eracon cliniasDHJ01	Pyrginae	07-SRNP-42619	MHMXR774-08	JF762235
2804	Eracon cliniasDHJ01	Pyrginae	07-SRNP-42093	MHMXO944-08	JF762237
2805	Eracon cliniasDHJ01	Pyrginae	07-SRNP-2980	MHMXO943-08	JF762238
2806	Eracon cliniasDHJ01	Pyrginae	06-SRNP-44151	MHAHJ469-07	JF752773
2807	Eracon cliniasDHJ01	Pyrginae	06-SRNP-40016	MHAHG223-06	GU151403
2808	Eracon cliniasDHJ01	Pyrginae	05-SRNP-7368	MHAHF640-06	GU150437
2809	Eracon cliniasDHJ01	Pyrginae	05-SRNP-22120	MHAHF336-06	GU150434
2810	Eracon cliniasDHJ01	Pyrginae	05-SRNP-22121	MHAHF329-06	GU150430
2811	Eracon cliniasDHJ01	Pyrginae	05-SRNP-22467	MHAHF328-06	GU150429
2812	Eracon cliniasDHJ01	Pyrginae	07-SRNP-42094	MHMXO945-08	JF762236
2813	Eracon cliniasDHJ01	Pyrginae	03-SRNP-37802	CSCR580-04	DQ292501
2814	Eracon cliniasDHJ01	Pyrginae	01-SRNP-5247	CSCR108-04	DQ292499
2815	Eracon cliniasDHJ01	Pyrginae	05-SRNP-1713	MHAHF334-06	GU150428
2816	Eracon cliniasDHJ01	Pyrginae	05-SRNP-22396	MHAHF330-06	GU150433
2817	Eracon cliniasDHJ01	Pyrginae	05-SRNP-3449	MHAHF331-06	GU150432
2818	Eracon cliniasDHJ01	Pyrginae	05-SRNP-1714	MHAHF332-06	GU150431
2819	Eracon cliniasDHJ01	Pyrginae	01-SRNP-5289	CSCR109-04	DQ292500
2820	Eracon cliniasDHJ01	Pyrginae	05-SRNP-3823	MHAHF333-06	GU150435
2821	Eracon cliniasDHJ01	Pyrginae	05-SRNP-4520	MHAHF338-06	GU150426
2822	Eracon cliniasDHJ01	Pyrginae	07-SRNP-42620	MHMXT200-08	JF762234
2823	Eracon cliniasDHJ01	Pyrginae	08-SRNP-65365	MHMXW402-09	JF753904
2824	Eracon cliniasDHJ01	Pyrginae	08-SRNP-21838	MHMXW403-09	JF753905
2825	Eracon cliniasDHJ01	Pyrginae	08-SRNP-41449	MHMXX527-09	JF777920
2826	Eracon cliniasDHJ01	Pyrginae	08-SRNP-41450	MHMXX528-09	JF777921
2827	Eracon cliniasDHJ01	Pyrginae	09-SRNP-1734	MHMYE1546-09	HM391106
2828	Chiomara georgina	Pyrginae	07-SRNP-55431	MHAHK707-07	JF760541
2829	Chiomara georgina	Pyrginae	03-SRNP-12148	CSCR344-04	DQ292136
2830	Chiomara georgina	Pyrginae	07-SRNP-20138	MHAHK704-07	JF760539
2831	Chiomara georgina	Pyrginae	08-SRNP-55568	MHMXX867-09	JF777765
2832	Chiomara georgina	Pyrginae	06-SRNP-12610	MHAHG732-06	GU151303
2833	Chiomara georgina	Pyrginae	04-SRNP-14129	MHAHD675-05	GU161330
2834	Chiomara georgina	Pyrginae	04-SRNP-14128	MHAHD674-05	GU161328
2835	Chiomara georgina	Pyrginae	04-SRNP-14583	MHAHD673-05	GU161329
2836	Chiomara georgina	Pyrginae	03-SRNP-12149	CSCR343-04	DQ292135
2837	Chiomara georgina	Pyrginae	05-SRNP-12171	MHAHF282-06	GU150334
2838	Chiomara georgina	Pyrginae	06-SRNP-22681	MHAHJ708-07	JF752568
2839	Chiomara georgina	Pyrginae	06-SRNP-22680	MHAHJ709-07	JF752569
2840	Chiomara georgina	Pyrginae	07-SRNP-12184	MHAHK706-07	JF760540
2841	Chiomara georgina	Pyrginae	07-SRNP-55287	MHAHK708-07	JF760542
2842	Chiomara georgina	Pyrginae	07-SRNP-20170	MHAHK709-07	JF760543
2843	Chiomara georgina	Pyrginae	07-SRNP-57560	MHMXO965-08	JF761940
2844	Chiomara georgina	Pyrginae	09-SRNP-72879	MHMYG2381-10	HM885805
2845	Gorgythion begga pyralinaDHJ01	Pyrginae	01-SRNP-15890	CSRII737-05	DQ292556
2846	Gorgythion begga pyralinaDHJ01	Pyrginae	02-SRNP-16048	CSRII201-04	DQ292517
2847	Gorgythion begga pyralinaDHJ01	Pyrginae	02-SRNP-32020	CSRII203-04	DQ292519
2848	Gorgythion begga pyralinaDHJ01	Pyrginae	05-SRNP-31852	MHAHF039-06	GU150459
2849	Gorgythion begga pyralinaDHJ01	Pyrginae	07-SRNP-20872	MHAHL178-07	JF762263
2850	Gorgythion begga pyralinaDHJ01	Pyrginae	05-SRNP-358	MHAHF040-06	GU150458
2851	Gorgythion begga pyralinaDHJ01	Pyrginae	05-SRNP-40713	MHAHE929-06	GU149685
2852	Gorgythion begga pyralinaDHJ01	Pyrginae	05-SRNP-1709	MHAHF045-06	GU150457
2853	Gorgythion begga pyralinaDHJ01	Pyrginae	05-SRNP-40714	MHAHF048-06	GU150460
2854	Gorgythion begga pyralinaDHJ01	Pyrginae	05-SRNP-1707	MHAHF049-06	GU150461
2855	Gorgythion begga pyralinaDHJ01	Pyrginae	05-SRNP-1706	MHAHF055-06	GU150455

Tree Order	Species	Subfamily	ACG Sampleid	BOLD Processid	Genbank Accession
2856	Gorgythion begga pyralinaDHJ01	Pyrginae	05-SRNP-1710	MHAHF060-06	GU150464
2857	Gorgythion begga pyralinaDHJ01	Pyrginae	05-SRNP-64097	MHAHF818-06	GU150466
2858	Gorgythion begga pyralinaDHJ01	Pyrginae	06-SRNP-531	MHAHG275-06	GU151421
2859	Gorgythion begga pyralinaDHJ01	Pyrginae	06-SRNP-532	MHAHG281-06	GU151424
2860	Gorgythion begga pyralinaDHJ01	Pyrginae	06-SRNP-41261	MHAHG762-06	GU151416
2861	Gorgythion begga pyralinaDHJ01	Pyrginae	06-SRNP-31441	MHAHG766-06	GU151415
2862	Gorgythion begga pyralinaDHJ01	Pyrginae	06-SRNP-2368	MHAHG768-06	GU151417
2863	Gorgythion begga pyralinaDHJ01	Pyrginae	06-SRNP-20632	MHAHH425-06	GU155413
2864	Gorgythion begga pyralinaDHJ01	Pyrginae	06-SRNP-41943	MHAHI188-06	GU155977
2865	Gorgythion begga pyralinaDHJ01	Pyrginae	97-SRNP-5411	MHAHJ234-07	JF752798
2866	Gorgythion begga pyralinaDHJ01	Pyrginae	00-SRNP-2146	MHAHJ241-07	JF752801
2867	Gorgythion begga pyralinaDHJ01	Pyrginae	00-SRNP-2408	MHAHJ243-07	JF752802
2868	Gorgythion begga pyralinaDHJ01	Pyrginae	99-SRNP-2221	MHAHJ250-07	JF752804
2869	Gorgythion begga pyralinaDHJ01	Pyrginae	99-SRNP-15519	MHAHJ255-07	JF752806
2870	Gorgythion begga pyralinaDHJ01	Pyrginae	00-SRNP-11026	MHAHJ263-07	JF752808
2871	Gorgythion begga pyralinaDHJ01	Pyrginae	99-SRNP-15470	MHAHJ264-07	JF752809
2872	Gorgythion begga pyralinaDHJ01	Pyrginae	00-SRNP-3210	MHAHJ266-07	JF752810
2873	Gorgythion begga pyralinaDHJ01	Pyrginae	99-SRNP-4380	MHAHJ267-07	JF752811
2874	Gorgythion begga pyralinaDHJ01	Pyrginae	98-SRNP-4012	MHAHJ269-07	JF752812
2875	Gorgythion begga pyralinaDHJ01	Pyrginae	04-SRNP-50125	MHMXG186-07	JF760742
2876	Gorgythion begga pyralinaDHJ01	Pyrginae	04-SRNP-14948	MHMXG189-07	JF760746
2877	Gorgythion begga pyralinaDHJ01	Pyrginae	06-SRNP-1869	MHAHG769-06	GU151420
2878	Gorgythion begga pyralinaDHJ01	Pyrginae	06-SRNP-1799	MHAHG770-06	GU151419
2879	Gorgythion begga pyralinaDHJ01	Pyrginae	98-SRNP-4195	MHAHJ271-07	JF752813
2880	Gorgythion begga pyralinaDHJ01	Pyrginae	04-SRNP-45528	MHMXG182-07	JF760745
2881	Gorgythion begga pyralinaDHJ01	Pyrginae	04-SRNP-21113	MHMXG190-07	JF760739
2882	Gorgythion begga pyralinaDHJ01	Pyrginae	04-SRNP-45237	MHMXG192-07	JF760741
2883	Gorgythion begga pyralinaDHJ01	Pyrginae	07-SRNP-55339	MHAHK284-07	JF760747
2884	Gorgythion begga pyralinaDHJ01	Pyrginae	08-SRNP-70487	MHMXX1102-09	JF777981
2885	Gorgythion begga pyralinaDHJ01	Pyrginae	08-SRNP-45022	MHMXX1106-09	JF777982
2886	Gorgythion begga pyralinaDHJ01	Pyrginae	08-SRNP-20764	MHMXX1112-09	JF777983
2887	Gorgythion begga pyralinaDHJ01	Pyrginae	08-SRNP-30986	MHMXX1113-09	JF777984
2888	Gorgythion begga pyralinaDHJ01	Pyrginae	08-SRNP-30987	MHMXX1114-09	JF777985
2889	Gorgythion begga pyralinaDHJ01	Pyrginae	05-SRNP-24241	MHAHF775-06	GU150456
2890	Gorgythion begga pyralinaDHJ01	Pyrginae	05-SRNP-24247	MHAHF776-06	GU150465
2891	Gorgythion begga pyralinaDHJ01	Pyrginae	02-SRNP-32047	CSRII621-05	DQ292549
2892	Gorgythion begga pyralinaDHJ01	Pyrginae	02-SRNP-2287	CSRII620-05	DQ292548
2893	Gorgythion begga pyralinaDHJ01	Pyrginae	02-SRNP-32909	CSRII615-05	DQ292543
2894	Gorgythion begga pyralinaDHJ01	Pyrginae	02-SRNP-5882	CSRII614-05	DQ292542
2895	Gorgythion begga pyralinaDHJ01	Pyrginae	02-SRNP-13764	CSRII612-05	DQ292540
2896	Gorgythion begga pyralinaDHJ01	Pyrginae	02-SRNP-5878	CSRII611-05	DQ292539
2897	Gorgythion begga pyralinaDHJ01	Pyrginae	02-SRNP-14308	CSRII605-05	DQ292533
2898	Gorgythion begga pyralinaDHJ01	Pyrginae	01-SRNP-4872	CSRII735-05	DQ292554
2899	Gorgythion begga pyralinaDHJ01	Pyrginae	02-SRNP-13763	CSRII603-05	DQ292531
2900	Gorgythion begga pyralinaDHJ01	Pyrginae	04-SRNP-15314	MHMXG178-07	JF760740
2901	Gorgythion begga pyralinaDHJ01	Pyrginae	02-SRNP-2285	CSRII198-04	DQ292514
2902	Gorgythion begga pyralinaDHJ01	Pyrginae	06-SRNP-30280	MHAHG277-06	GU151418
2903	Gorgythion begga pyralinaDHJ01	Pyrginae	05-SRNP-206	MHMXG185-07	JF760743
2904	Gorgythion begga pyralinaDHJ01	Pyrginae	06-SRNP-753	MHAHG282-06	GU151422
2905	Gorgythion begga pyralinaDHJ01	Pyrginae	05-SRNP-23371	MHAHF050-06	GU150463
2906	Gorgythion begga pyralinaDHJ01	Pyrginae	06-SRNP-1800	MHAHG767-06	GU151414
2907	Gorgythion begga pyralinaDHJ01	Pyrginae	04-SRNP-27297	MHAHF054-06	GU150462
2908	Gorgythion begga pyralinaDHJ01	Pyrginae	96-SRNP-886	MHAHJ236-07	JF752799
2909	Gorgythion begga pyralinaDHJ01	Pyrginae	06-SRNP-30794	MHAHG279-06	GU151423
2910	Gorgythion begga pyralinaDHJ01	Pyrginae	99-SRNP-322	MHAHJ260-07	JF752807
2911	Gorgythion begga pyralinaDHJ01	Pyrginae	99-SRNP-298	MHAHJ249-07	JF752803

Tree Order	Species	Subfamily	ACG Sampleid	BOLD Processid	Genbank Accession
2912	Gorgythion begga pyralinaDHJ01	Pyrginae	96-SRNP-12038	MHAHJ233-07	JF752797
2913	Gorgythion begga pyralinaDHJ01	Pyrginae	99-SRNP-2514	MHAHJ252-07	JF752805
2914	Gorgythion begga pyralinaDHJ01	Pyrginae	06-SRNP-263	MHMXA565-06	JF760744
2915	Gorgythion begga pyralinaDHJ01	Pyrginae	95-SRNP-6465	MHAHJ237-07	JF752800
2916	Gorgythion begga pyralinaDHJ01	Pyrginae	03-SRNP-28548	CSRII211-04	DQ292527
2917	Gorgythion begga pyralinaDHJ01	Pyrginae	02-SRNP-17248	CSRII202-04	DQ292518
2918	Gorgythion begga pyralinaDHJ01	Pyrginae	92-SRNP-3165	CSCR542-04	DQ292513
2919	Gorgythion begga pyralinaDHJ01	Pyrginae	07-SRNP-20992	MHAHL176-07	JF762262
2920	Gorgythion begga pyralinaDHJ01	Pyrginae	07-SRNP-20948	MHAHL179-07	JF762264
2921	Gorgythion begga pyralinaDHJ01	Pyrginae	08-SRNP-40557	MHMXX1185-09	JF777986
2922	Gorgythion begga pyralinaDHJ01	Pyrginae	08-SRNP-70553	MHMXX710-09	JF777980
2923	Gorgythion begga pyralinaDHJ02	Pyrginae	03-SRNP-1239	CSRII205-04	DQ292521
2924	Gorgythion begga pyralinaDHJ02	Pyrginae	03-SRNP-20279	CSRII206-04	DQ292522
2925	Gorgythion begga pyralinaDHJ02	Pyrginae	03-SRNP-1176	CSRII204-04	DQ292520
2926	Gorgythion begga pyralinaDHJ02	Pyrginae	05-SRNP-33821	MHAHF043-06	GU150470
2927	Gorgythion begga pyralinaDHJ02	Pyrginae	05-SRNP-33754	MHAHF810-06	GU150484
2928	Gorgythion begga pyralinaDHJ02	Pyrginae	05-SRNP-33763	MHAHF822-06	GU150486
2929	Gorgythion begga pyralinaDHJ02	Pyrginae	01-SRNP-17454	CSRII738-05	DQ292557
2930	Gorgythion begga pyralinaDHJ02	Pyrginae	06-SRNP-40222	MHMXA569-06	JF760771
2931	Gorgythion begga pyralinaDHJ02	Pyrginae	07-SRNP-55213	MHAHK287-07	JF760776
2932	Gorgythion begga pyralinaDHJ02	Pyrginae	01-SRNP-1269	CSRII734-05	DQ292553
2933	Gorgythion begga pyralinaDHJ02	Pyrginae	08-SRNP-20679	MHMXX1105-09	JF777991
2934	Gorgythion begga pyralinaDHJ02	Pyrginae	08-SRNP-24442	MHMYB160-09	GU649682
2935	Gorgythion begga pyralinaDHJ02	Pyrginae	07-SRNP-60837	MHMXR052-08	JF762271
2936	Gorgythion begga pyralinaDHJ02	Pyrginae	08-SRNP-2157	MHMXX1104-09	JF777990
2937	Gorgythion begga pyralinaDHJ02	Pyrginae	06-SRNP-19738	MHMXI575-07	JF760767
2938	Gorgythion begga pyralinaDHJ02	Pyrginae	07-SRNP-55196	MHAHK285-07	JF760774
2939	Gorgythion begga pyralinaDHJ02	Pyrginae	99-SRNP-2400	MHAHJ257-07	JF752832
2940	Gorgythion begga pyralinaDHJ02	Pyrginae	98-SRNP-4745	MHAHJ261-07	JF752835
2941	Gorgythion begga pyralinaDHJ02	Pyrginae	00-SRNP-2145	MHAHJ265-07	JF752837
2942	Gorgythion begga pyralinaDHJ02	Pyrginae	98-SRNP-13474	MHAHJ270-07	JF752839
2943	Gorgythion begga pyralinaDHJ02	Pyrginae	06-SRNP-42907	MHAHJ434-07	JF752841
2944	Gorgythion begga pyralinaDHJ02	Pyrginae	06-SRNP-22800	MHAHJ475-07	JF752842
2945	Gorgythion begga pyralinaDHJ02	Pyrginae	04-SRNP-42567	MHMXG177-07	JF760750
2946	Gorgythion begga pyralinaDHJ02	Pyrginae	04-SRNP-41839	MHMXG193-07	JF760757
2947	Gorgythion begga pyralinaDHJ02	Pyrginae	06-SRNP-57189	MHAHI189-06	GU155979
2948	Gorgythion begga pyralinaDHJ02	Pyrginae	06-SRNP-57188	MHAHI191-06	GU155978
2949	Gorgythion begga pyralinaDHJ02	Pyrginae	97-SRNP-127	MHAHJ232-07	JF752817
2950	Gorgythion begga pyralinaDHJ02	Pyrginae	02-SRNP-14120	MHAHJ246-07	JF752825
2951	Gorgythion begga pyralinaDHJ02	Pyrginae	06-SRNP-30444	MHAHG276-06	GU151429
2952	Gorgythion begga pyralinaDHJ02	Pyrginae	06-SRNP-30443	MHAHG278-06	GU151425
2953	Gorgythion begga pyralinaDHJ02	Pyrginae	05-SRNP-65607	MHAHF785-06	GU150483
2954	Gorgythion begga pyralinaDHJ02	Pyrginae	05-SRNP-66197	MHAHF786-06	GU150481
2955	Gorgythion begga pyralinaDHJ02	Pyrginae	05-SRNP-30599	MHAHF052-06	GU150478
2956	Gorgythion begga pyralinaDHJ02	Pyrginae	05-SRNP-2471	MHAHF059-06	GU150476
2957	Gorgythion begga pyralinaDHJ02	Pyrginae	06-SRNP-22074	MHAHJ688-07	JF752843
2958	Gorgythion begga pyralinaDHJ02	Pyrginae	06-SRNP-65353	MHAHJ690-07	JF752845
2959	Gorgythion begga pyralinaDHJ02	Pyrginae	07-SRNP-42322	MHMXP211-08	JF762274
2960	Gorgythion begga pyralinaDHJ02	Pyrginae	07-SRNP-42321	MHMXP212-08	JF762273
2961	Gorgythion begga pyralinaDHJ02	Pyrginae	06-SRNP-30986	MHAHG764-06	GU151428
2962	Gorgythion begga pyralinaDHJ02	Pyrginae	06-SRNP-31171	MHAHG765-06	GU151427
2963	Gorgythion begga pyralinaDHJ02	Pyrginae	05-SRNP-33765	MHAHF821-06	GU150487
2964	Gorgythion begga pyralinaDHJ02	Pyrginae	05-SRNP-33759	MHAHF825-06	GU150489
2965	Gorgythion begga pyralinaDHJ02	Pyrginae	02-SRNP-5740	CSRII619-05	DQ292547
2966	Gorgythion begga pyralinaDHJ02	Pyrginae	02-SRNP-5307	CSRII618-05	DQ292546
2967	Gorgythion begga pyralinaDHJ02	Pyrginae	02-SRNP-5972	CSRII613-05	DQ292541

Tree Order	Species	Subfamily	ACG Sampleid	BOLD Processid	Genbank Accession
2968	Gorgythion begga pyralinaDHJ02	Pyrginae	02-SRNP-4537	CSRII610-05	DQ292538
2969	Gorgythion begga pyralinaDHJ02	Pyrginae	02-SRNP-5739	CSRII609-05	DQ292537
2970	Gorgythion begga pyralinaDHJ02	Pyrginae	02-SRNP-33710	CSRII608-05	DQ292536
2971	Gorgythion begga pyralinaDHJ02	Pyrginae	01-SRNP-9221	CSRII736-05	DQ292555
2972	Gorgythion begga pyralinaDHJ02	Pyrginae	02-SRNP-2286	CSRII606-05	DQ292534
2973	Gorgythion begga pyralinaDHJ02	Pyrginae	01-SRNP-1270	CSRII733-05	DQ292552
2974	Gorgythion begga pyralinaDHJ02	Pyrginae	01-SRNP-17453	CSRII732-05	DQ292551
2975	Gorgythion begga pyralinaDHJ02	Pyrginae	01-SRNP-15739	CSRII731-05	DQ292550
2976	Gorgythion begga pyralinaDHJ02	Pyrginae	02-SRNP-27923	MHAHJ245-07	JF752824
2977	Gorgythion begga pyralinaDHJ02	Pyrginae	04-SRNP-41616	MHMXG194-07	JF760756
2978	Gorgythion begga pyralinaDHJ02	Pyrginae	03-SRNP-21505	CSRII209-04	DQ292525
2979	Gorgythion begga pyralinaDHJ02	Pyrginae	06-SRNP-23305	MHAHJ433-07	JF752840
2980	Gorgythion begga pyralinaDHJ02	Pyrginae	08-SRNP-65479	MHMXX1107-09	JF777992
2981	Gorgythion begga pyralinaDHJ02	Pyrginae	08-SRNP-4702	MHMXX597-09	JF777988
2982	Gorgythion begga pyralinaDHJ02	Pyrginae	05-SRNP-66163	MHAHF682-06	GU150491
2983	Gorgythion begga pyralinaDHJ02	Pyrginae	05-SRNP-40934	MHAHF042-06	GU150475
2984	Gorgythion begga pyralinaDHJ02	Pyrginae	02-SRNP-15032	CSRII604-05	DQ292532
2985	Gorgythion begga pyralinaDHJ02	Pyrginae	07-SRNP-31672	MHAHL183-07	JF762267
2986	Gorgythion begga pyralinaDHJ02	Pyrginae	07-SRNP-65124	MHAHL181-07	JF762266
2987	Gorgythion begga pyralinaDHJ02	Pyrginae	05-SRNP-33764	MHMXI581-07	JF760761
2988	Gorgythion begga pyralinaDHJ02	Pyrginae	03-SRNP-28550	CSRII212-04	DQ292528
2989	Gorgythion begga pyralinaDHJ02	Pyrginae	98-SRNP-4629	MHAHJ247-07	JF752826
2990	Gorgythion begga pyralinaDHJ02	Pyrginae	99-SRNP-2220	MHAHJ268-07	JF752838
2991	Gorgythion begga pyralinaDHJ02	Pyrginae	98-SRNP-4280	MHAHJ262-07	JF752836
2992	Gorgythion begga pyralinaDHJ02	Pyrginae	99-SRNP-2219	MHAHJ259-07	JF752834
2993	Gorgythion begga pyralinaDHJ02	Pyrginae	02-SRNP-14184	MHAHJ244-07	JF752823
2994	Gorgythion begga pyralinaDHJ02	Pyrginae	05-SRNP-41201	MHAHF044-06	GU150473
2995	Gorgythion begga pyralinaDHJ02	Pyrginae	95-SRNP-7044	MHAHJ235-07	JF752818
2996	Gorgythion begga pyralinaDHJ02	Pyrginae	06-SRNP-40632	MHMXA566-06	JF760763
2997	Gorgythion begga pyralinaDHJ02	Pyrginae	95-SRNP-6166	MHAHJ229-07	JF752814
2998	Gorgythion begga pyralinaDHJ02	Pyrginae	06-SRNP-30946	MHAHG763-06	GU151426
2999	Gorgythion begga pyralinaDHJ02	Pyrginae	05-SRNP-40817	MHAHF051-06	GU150480
3000	Gorgythion begga pyralinaDHJ02	Pyrginae	96-SRNP-9689	MHAHJ230-07	JF752815
3001	Gorgythion begga pyralinaDHJ02	Pyrginae	97-SRNP-5666	MHAHJ239-07	JF752820
3002	Gorgythion begga pyralinaDHJ02	Pyrginae	99-SRNP-3525	MHAHJ258-07	JF752833
3003	Gorgythion begga pyralinaDHJ02	Pyrginae	03-SRNP-21608	CSRII210-04	DQ292526
3004	Gorgythion begga pyralinaDHJ02	Pyrginae	02-SRNP-5088	CSRII199-04	DQ292515
3005	Gorgythion begga pyralinaDHJ02	Pyrginae	98-SRNP-4756	MHAHJ248-07	JF752827
3006	Gorgythion begga pyralinaDHJ02	Pyrginae	04-SRNP-43161	MHMXG176-07	JF760752
3007	Gorgythion begga pyralinaDHJ02	Pyrginae	97-SRNP-380	MHAHJ238-07	JF752819
3008	Gorgythion begga pyralinaDHJ02	Pyrginae	05-SRNP-32342	MHAHF036-06	GU150468
3009	Gorgythion begga pyralinaDHJ02	Pyrginae	97-SRNP-11537	MHAHJ231-07	JF752816
3010	Gorgythion begga pyralinaDHJ02	Pyrginae	00-SRNP-2655	MHAHJ253-07	JF752829
3011	Gorgythion begga pyralinaDHJ02	Pyrginae	08-SRNP-40397	MHMXT193-08	JF762270
3012	Gorgythion begga pyralinaDHJ02	Pyrginae	08-SRNP-40417	MHMXT195-08	JF762268
3013	Gorgythion begga pyralinaDHJ02	Pyrginae	93-SRNP-7034	MHMYD751-09	
3014	Gorgythion begga pyralinaDHJ02	Pyrginae	03-SRNP-20828	CSRII207-04	DQ292523
3015	Gorgythion begga pyralinaDHJ02	Pyrginae	05-SRNP-30971	MHAHF041-06	GU150474
3016	Gorgythion begga pyralinaDHJ02	Pyrginae	07-SRNP-23849	MHMXR050-08	JF762272
3017	Gorgythion begga pyralinaDHJ02	Pyrginae	06-SRNP-59585	MHMXH888-07	JF760769
3018	Gorgythion begga pyralinaDHJ02	Pyrginae	06-SRNP-59797	MHMXH884-07	JF760751
3019	Gorgythion begga pyralinaDHJ02	Pyrginae	06-SRNP-59614	MHMXH883-07	JF760755
3020	Gorgythion begga pyralinaDHJ02	Pyrginae	06-SRNP-43424	MHAHJ691-07	JF752846
3021	Gorgythion begga pyralinaDHJ02	Pyrginae	06-SRNP-22111	MHAHJ689-07	JF752844
3022	Gorgythion begga pyralinaDHJ02	Pyrginae	04-SRNP-30434	MHMXG196-07	JF760753
3023	Gorgythion begga pyralinaDHJ02	Pyrginae	04-SRNP-40895	MHMXG191-07	JF760758

Tree Order	Species	Subfamily	ACG Sampleid	BOLD Processid	Genbank Accession
3024	Gorgythion begga pyralinaDHJ02	Pyrginae	04-SRNP-35213	MHMXG187-07	JF760760
3025	Gorgythion begga pyralinaDHJ02	Pyrginae	04-SRNP-15268	MHMXG181-07	JF760766
3026	Gorgythion begga pyralinaDHJ02	Pyrginae	04-SRNP-33308	MHMXG180-07	JF760768
3027	Gorgythion begga pyralinaDHJ02	Pyrginae	00-SRNP-6011	MHAHJ256-07	JF752831
3028	Gorgythion begga pyralinaDHJ02	Pyrginae	00-SRNP-2364	MHAHJ254-07	JF752830
3029	Gorgythion begga pyralinaDHJ02	Pyrginae	97-SRNP-10376	MHAHJ251-07	JF752828
3030	Gorgythion begga pyralinaDHJ02	Pyrginae	00-SRNP-2182	MHAHJ242-07	JF752822
3031	Gorgythion begga pyralinaDHJ02	Pyrginae	00-SRNP-2038	MHAHJ240-07	JF752821
3032	Gorgythion begga pyralinaDHJ02	Pyrginae	06-SRNP-41729	MHAHH424-06	GU155414
3033	Gorgythion begga pyralinaDHJ02	Pyrginae	06-SRNP-30448	MHMXA570-06	JF760770
3034	Gorgythion begga pyralinaDHJ02	Pyrginae	06-SRNP-30751	MHMXA568-06	JF760759
3035	Gorgythion begga pyralinaDHJ02	Pyrginae	06-SRNP-30445	MHMXA567-06	JF760762
3036	Gorgythion begga pyralinaDHJ02	Pyrginae	06-SRNP-30942	MHAHG280-06	GU151430
3037	Gorgythion begga pyralinaDHJ02	Pyrginae	05-SRNP-33753	MHAHF824-06	GU150490
3038	Gorgythion begga pyralinaDHJ02	Pyrginae	05-SRNP-33752	MHAHF823-06	GU150488
3039	Gorgythion begga pyralinaDHJ02	Pyrginae	05-SRNP-33272	MHAHF783-06	GU150482
3040	Gorgythion begga pyralinaDHJ02	Pyrginae	05-SRNP-30970	MHAHF058-06	GU150479
3041	Gorgythion begga pyralinaDHJ02	Pyrginae	05-SRNP-31860	MHAHF038-06	GU150471
3042	Gorgythion begga pyralinaDHJ02	Pyrginae	02-SRNP-21092	CSRII617-05	DQ292545
3043	Gorgythion begga pyralinaDHJ02	Pyrginae	02-SRNP-33944	CSRII616-05	DQ292544
3044	Gorgythion begga pyralinaDHJ02	Pyrginae	02-SRNP-19850	CSRII607-05	DQ292535
3045	Gorgythion begga pyralinaDHJ02	Pyrginae	02-SRNP-5107	CSRII602-05	DQ292530
3046	Gorgythion begga pyralinaDHJ02	Pyrginae	02-SRNP-27777	CSRII601-05	DQ292529
3047	Gorgythion begga pyralinaDHJ02	Pyrginae	07-SRNP-55244	MHAHK283-07	JF760773
3048	Gorgythion begga pyralinaDHJ02	Pyrginae	07-SRNP-55198	MHAHK286-07	JF760775
3049	Gorgythion begga pyralinaDHJ02	Pyrginae	05-SRNP-31861	MHAHF037-06	GU150472
3050	Gorgythion begga pyralinaDHJ02	Pyrginae	04-SRNP-43162	MHMXG184-07	JF760764
3051	Gorgythion begga pyralinaDHJ02	Pyrginae	05-SRNP-30226	MHMXG183-07	JF760765
3052	Gorgythion begga pyralinaDHJ02	Pyrginae	06-SRNP-57350	MHAHI192-06	GU155980
3053	Gorgythion begga pyralinaDHJ02	Pyrginae	05-SRNP-30688	MHAHF053-06	GU150477
3054	Gorgythion begga pyralinaDHJ02	Pyrginae	02-SRNP-14229	CSCR541-04	DQ292512
3055	Gorgythion begga pyralinaDHJ02	Pyrginae	02-SRNP-13839	CSRII200-04	DQ292516
3056	Gorgythion begga pyralinaDHJ02	Pyrginae	04-SRNP-56552	MHMXG188-07	JF760772
3057	Gorgythion begga pyralinaDHJ02	Pyrginae	07-SRNP-41296	MHAHL180-07	JF762265
3058	Gorgythion begga pyralinaDHJ02	Pyrginae	08-SRNP-40399	MHMXT194-08	JF762269
3059	Gorgythion begga pyralinaDHJ02	Pyrginae	08-SRNP-70432	MHMXX1103-09	JF777989
3060	Gorgythion begga pyralinaDHJ02	Pyrginae	08-SRNP-1816	MHMXX1108-09	JF777993
3061	Gorgythion begga pyralinaDHJ02	Pyrginae	08-SRNP-30607	MHMXX1110-09	JF777995
3062	Gorgythion begga pyralinaDHJ02	Pyrginae	08-SRNP-20433	MHMXX1111-09	JF777996
3063	Gorgythion begga pyralinaDHJ02	Pyrginae	08-SRNP-30605	MHMXX1115-09	JF777997
3064	Gorgythion begga pyralinaDHJ02	Pyrginae	08-SRNP-30606	MHMXX1116-09	JF777998
3065	Gorgythion begga pyralinaDHJ02	Pyrginae	08-SRNP-22577	MHMXX596-09	JF777987
3066	Gorgythion begga pyralinaDHJ02	Pyrginae	08-SRNP-24676	MHMYB159-09	GU649681
3067	Gorgythion begga pyralinaDHJ02	Pyrginae	09-SRNP-72858	MHMYE1549-09	HM391109
3068	Gorgythion begga pyralinaDHJ02	Pyrginae	06-SRNP-60373	MHAHK288-07	JF760777
3069	Gorgythion begga pyralinaDHJ02	Pyrginae	04-SRNP-42979	MHMXG195-07	JF760754
3070	Gorgythion begga pyralinaDHJ02	Pyrginae	05-SRNP-66115	MHAHF787-06	GU150485
3071	Gorgythion begga pyralinaDHJ02	Pyrginae	05-SRNP-41039	MHAHF056-06	GU150467
3072	Gorgythion begga pyralinaDHJ02	Pyrginae	03-SRNP-21087	CSRII208-04	DQ292524
3073	Gorgythion begga pyralinaDHJ02	Pyrginae	06-SRNP-30446	MHMXA571-06	JF760748
3074	Gorgythion begga pyralinaDHJ02	Pyrginae	04-SRNP-60168	MHMXG179-07	JF760749
3075	Gorgythion begga pyralinaDHJ02	Pyrginae	05-SRNP-20969	MHAHF057-06	GU150469
3076	Gorgythion begga pyralinaDHJ02	Pyrginae	08-SRNP-65113	MHMXX1109-09	JF777994
3077	Gorgythion begga pyralinaDHJ02	Pyrginae	09-SRNP-66079	MHMYI654-10	HQ963966
3078	Telemiades fides	Pyrginae	07-SRNP-16611	MHMXX722-09	JF778534
3079	Telemiades fides	Pyrginae	05-SRNP-55775	MHAHF939-06	GU150916

Tree Order	Species	Subfamily	ACG Sampleid	BOLD Processid	Genbank Accession
3080	Telemiades fides	Pyrginae	08-SRNP-65257	MHMXW260-09	JF754304
3081	Telemiades fides	Pyrginae	02-SRNP-14055	CSRII681-05	DQ293552
3082	Telemiades fides	Pyrginae	07-SRNP-23507	MHMXR597-08	JF763181
3083	Telemiades fides	Pyrginae	05-SRNP-13191	MHAHF566-06	GU150912
3084	Telemiades fides	Pyrginae	07-SRNP-65513	MHMXR596-08	JF763182
3085	Telemiades fides	Pyrginae	07-SRNP-61381	MHMXW223-09	JF754270
3086	Telemiades fides	Pyrginae	07-SRNP-60109	MHMXR576-08	JF763200
3087	Telemiades fides	Pyrginae	07-SRNP-21983	MHAHL507-07	JF763151
3088	Telemiades fides	Pyrginae	07-SRNP-65137	MHAHL506-07	JF763150
3089	Telemiades fides	Pyrginae	07-SRNP-31688	MHAHL496-07	JF763142
3090	Telemiades fides	Pyrginae	07-SRNP-31687	MHAHL493-07	JF763140
3091	Telemiades fides	Pyrginae	07-SRNP-21628	MHAHL484-07	JF763134
3092	Telemiades fides	Pyrginae	07-SRNP-20486	MHAHK342-07	JF761214
3093	Telemiades fides	Pyrginae	07-SRNP-21257	MHAHK339-07	JF761211
3094	Telemiades fides	Pyrginae	06-SRNP-60265	MHAHK336-07	JF761208
3095	Telemiades fides	Pyrginae	02-SRNP-3677	CSRII680-05	DQ293551
3096	Telemiades fides	Pyrginae	07-SRNP-5104	MHMXS002-08	JF763176
3097	Telemiades fides	Pyrginae	07-SRNP-31870	MHAHL491-07	JF763139
3098	Telemiades fides	Pyrginae	07-SRNP-65687	MHMXR599-08	JF763179
3099	Telemiades fides	Pyrginae	03-SRNP-1234	CSCR427-04	DQ293542
3100	Telemiades fides	Pyrginae	02-SRNP-4033	CSRII694-05	DQ293565
3101	Telemiades fides	Pyrginae	07-SRNP-3685	MHMXO922-08	JF763212
3102	Telemiades fides	Pyrginae	02-SRNP-17931	CSRII676-05	DQ293548
3103	Telemiades fides	Pyrginae	07-SRNP-31873	MHAHL497-07	JF763143
3104	Telemiades fides	Pyrginae	08-SRNP-45092	MHMXW214-09	JF754261
3105	Telemiades fides	Pyrginae	08-SRNP-24174	MHMYB148-09	
3106	Telemiades fides	Pyrginae	05-SRNP-55645	MHAHF935-06	GU150910
3107	Telemiades fides	Pyrginae	07-SRNP-3734	MHMXO913-08	JF763215
3108	Telemiades fides	Pyrginae	07-SRNP-3520	MHMXO920-08	JF763213
3109	Telemiades fides	Pyrginae	05-SRNP-55619	MHAHF938-06	GU150920
3110	Telemiades fides	Pyrginae	07-SRNP-32190	MHAHK338-07	JF761210
3111	Telemiades fides	Pyrginae	02-SRNP-10030	CSRII678-05	DQ293549
3112	Telemiades fides	Pyrginae	07-SRNP-31486	MHAHL490-07	JF763138
3113	Telemiades fides	Pyrginae	05-SRNP-13188	MHAHF565-06	GU150913
3114	Telemiades fides	Pyrginae	07-SRNP-55335	MHAHK343-07	JF761215
3115	Telemiades fides	Pyrginae	07-SRNP-55240	MHAHK344-07	JF761216
3116	Telemiades fides	Pyrginae	07-SRNP-55110	MHAHK346-07	JF761218
3117	Telemiades fides	Pyrginae	07-SRNP-1307	MHAHK347-07	JF761219
3118	Telemiades fides	Pyrginae	07-SRNP-55464	MHAHK349-07	JF761220
3119	Telemiades fides	Pyrginae	07-SRNP-55163	MHAHK350-07	JF761221
3120	Telemiades fides	Pyrginae	07-SRNP-1762	MHAHL477-07	JF763127
3121	Telemiades fides	Pyrginae	07-SRNP-21361	MHAHL478-07	JF763128
3122	Telemiades fides	Pyrginae	07-SRNP-21318	MHAHL479-07	JF763129
3123	Telemiades fides	Pyrginae	07-SRNP-21194	MHAHL480-07	JF763130
3124	Telemiades fides	Pyrginae	07-SRNP-20999	MHAHL481-07	JF763131
3125	Telemiades fides	Pyrginae	07-SRNP-21302	MHAHL482-07	JF763132
3126	Telemiades fides	Pyrginae	07-SRNP-21275	MHAHL483-07	JF763133
3127	Telemiades fides	Pyrginae	07-SRNP-21243	MHAHL485-07	JF763135
3128	Telemiades fides	Pyrginae	07-SRNP-21453	MHAHL486-07	JF763136
3129	Telemiades fides	Pyrginae	07-SRNP-65185	MHAHL488-07	JF763137
3130	Telemiades fides	Pyrginae	07-SRNP-20827	MHAHL498-07	JF763144
3131	Telemiades fides	Pyrginae	07-SRNP-31658	MHAHL499-07	JF763145
3132	Telemiades fides	Pyrginae	07-SRNP-30815	MHAHL500-07	JF763146
3133	Telemiades fides	Pyrginae	07-SRNP-57095	MHAHL502-07	JF763147
3134	Telemiades fides	Pyrginae	07-SRNP-2224	MHAHL504-07	JF763148
3135	Telemiades fides	Pyrginae	07-SRNP-56685	MHAHL505-07	JF763149

Tree Order	Species	Subfamily	ACG Sampleid	BOLD Processid	Genbank Accession
3136	Telemiades fides	Pyrginae	07-SRNP-31298	MHAHL509-07	JF763152
3137	Telemiades fides	Pyrginae	07-SRNP-3731	MHMXO914-08	JF763214
3138	Telemiades fides	Pyrginae	07-SRNP-3500	MHMXO924-08	JF763210
3139	Telemiades fides	Pyrginae	07-SRNP-3567	MHMXO934-08	JF763206
3140	Telemiades fides	Pyrginae	07-SRNP-3838	MHMXR570-08	JF763205
3141	Telemiades fides	Pyrginae	07-SRNP-65761	MHMXR572-08	JF763203
3142	Telemiades fides	Pyrginae	07-SRNP-4407	MHMXR578-08	JF763199
3143	Telemiades fides	Pyrginae	07-SRNP-4674	MHMXR579-08	JF763198
3144	Telemiades fides	Pyrginae	07-SRNP-59719	MHMXR580-08	JF763197
3145	Telemiades fides	Pyrginae	07-SRNP-65688	MHMXR581-08	JF763196
3146	Telemiades fides	Pyrginae	07-SRNP-65514	MHMXR582-08	JF763195
3147	Telemiades fides	Pyrginae	07-SRNP-65511	MHMXR583-08	JF763194
3148	Telemiades fides	Pyrginae	07-SRNP-65572	MHMXR584-08	JF763193
3149	Telemiades fides	Pyrginae	07-SRNP-59610	MHMXR585-08	JF763192
3150	Telemiades fides	Pyrginae	07-SRNP-20495	MHMXR586-08	JF763191
3151	Telemiades fides	Pyrginae	07-SRNP-3892	MHMXR588-08	JF763190
3152	Telemiades fides	Pyrginae	07-SRNP-4701	MHMXR589-08	JF763189
3153	Telemiades fides	Pyrginae	07-SRNP-4102	MHMXR590-08	JF763188
3154	Telemiades fides	Pyrginae	07-SRNP-33424	MHMXR591-08	JF763187
3155	Telemiades fides	Pyrginae	07-SRNP-3891	MHMXR592-08	JF763186
3156	Telemiades fides	Pyrginae	07-SRNP-3839	MHMXR593-08	JF763185
3157	Telemiades fides	Pyrginae	07-SRNP-3983	MHMXR594-08	JF763184
3158	Telemiades fides	Pyrginae	07-SRNP-4100	MHMXR595-08	JF763183
3159	Telemiades fides	Pyrginae	07-SRNP-65718	MHMXR598-08	JF763180
3160	Telemiades fides	Pyrginae	07-SRNP-5003	MHMXS004-08	JF763174
3161	Telemiades fides	Pyrginae	07-SRNP-5103	MHMXS005-08	JF763173
3162	Telemiades fides	Pyrginae	07-SRNP-5105	MHMXS006-08	JF763172
3163	Telemiades fides	Pyrginae	07-SRNP-5174	MHMXS007-08	JF763171
3164	Telemiades fides	Pyrginae	08-SRNP-551	MHMXS008-08	JF763170
3165	Telemiades fides	Pyrginae	07-SRNP-5111	MHMXS010-08	JF763168
3166	Telemiades fides	Pyrginae	07-SRNP-5168	MHMXS011-08	JF763167
3167	Telemiades fides	Pyrginae	07-SRNP-3984	MHMXS013-08	JF763165
3168	Telemiades fides	Pyrginae	07-SRNP-42715	MHMXS014-08	JF763164
3169	Telemiades fides	Pyrginae	07-SRNP-4405	MHMXS015-08	JF763163
3170	Telemiades fides	Pyrginae	07-SRNP-3835	MHMXS016-08	JF763162
3171	Telemiades fides	Pyrginae	07-SRNP-4291	MHMXS017-08	JF763161
3172	Telemiades fides	Pyrginae	07-SRNP-5162	MHMXS018-08	JF763160
3173	Telemiades fides	Pyrginae	07-SRNP-4770	MHMXS019-08	JF763159
3174	Telemiades fides	Pyrginae	08-SRNP-129	MHMXS025-08	JF763154
3175	Telemiades fides	Pyrginae	07-SRNP-5139	MHMXS026-08	JF763153
3176	Telemiades fides	Pyrginae	07-SRNP-46433	MHMXS021-08	JF763158
3177	Telemiades fides	Pyrginae	07-SRNP-4902	MHMXS022-08	JF763157
3178	Telemiades fides	Pyrginae	08-SRNP-1791	MHMXW184-09	JF754233
3179	Telemiades fides	Pyrginae	08-SRNP-20925	MHMXW185-09	JF754234
3180	Telemiades fides	Pyrginae	08-SRNP-20965	MHMXW186-09	JF754235
3181	Telemiades fides	Pyrginae	08-SRNP-21076	MHMXW187-09	JF754236
3182	Telemiades fides	Pyrginae	08-SRNP-21078	MHMXW188-09	JF754237
3183	Telemiades fides	Pyrginae	08-SRNP-20956	MHMXW189-09	JF754238
3184	Telemiades fides	Pyrginae	08-SRNP-21028	MHMXW190-09	JF754239
3185	Telemiades fides	Pyrginae	08-SRNP-21036	MHMXW191-09	JF754240
3186	Telemiades fides	Pyrginae	08-SRNP-21077	MHMXW192-09	JF754241
3187	Telemiades fides	Pyrginae	08-SRNP-21084	MHMXW193-09	JF754242
3188	Telemiades fides	Pyrginae	08-SRNP-20967	MHMXW194-09	JF754243
3189	Telemiades fides	Pyrginae	08-SRNP-21070	MHMXW195-09	JF754244
3190	Telemiades fides	Pyrginae	08-SRNP-20494	MHMXW196-09	JF754245
3191	Telemiades fides	Pyrginae	08-SRNP-65208	MHMXW197-09	JF754246

Tree Order	Species	Subfamily	ACG Sampleid	BOLD Processid	Genbank Accession
3192	Telemiades fides	Pyrginae	08-SRNP-20966	MHMXW198-09	JF754247
3193	Telemiades fides	Pyrginae	08-SRNP-21083	MHMXW199-09	JF754248
3194	Telemiades fides	Pyrginae	08-SRNP-971	MHMXW201-09	JF754249
3195	Telemiades fides	Pyrginae	08-SRNP-2340	MHMXW204-09	JF754251
3196	Telemiades fides	Pyrginae	08-SRNP-65455	MHMXW205-09	JF754252
3197	Telemiades fides	Pyrginae	08-SRNP-45069	MHMXW206-09	JF754253
3198	Telemiades fides	Pyrginae	07-SRNP-24562	MHMXW207-09	JF754254
3199	Telemiades fides	Pyrginae	08-SRNP-45067	MHMXW209-09	JF754256
3200	Telemiades fides	Pyrginae	08-SRNP-55478	MHMXW210-09	JF754257
3201	Telemiades fides	Pyrginae	08-SRNP-45144	MHMXW211-09	JF754258
3202	Telemiades fides	Pyrginae	08-SRNP-2342	MHMXW253-09	JF754297
3203	Telemiades fides	Pyrginae	07-SRNP-61016	MHMXW254-09	JF754298
3204	Telemiades fides	Pyrginae	08-SRNP-1790	MHMXW257-09	JF754301
3205	Telemiades fides	Pyrginae	08-SRNP-65277	MHMXW258-09	JF754302
3206	Telemiades fides	Pyrginae	08-SRNP-45161	MHMXW212-09	JF754259
3207	Telemiades fides	Pyrginae	07-SRNP-61357	MHMXW213-09	JF754260
3208	Telemiades fides	Pyrginae	08-SRNP-21071	MHMXW215-09	JF754262
3209	Telemiades fides	Pyrginae	08-SRNP-45065	MHMXW216-09	JF754263
3210	Telemiades fides	Pyrginae	08-SRNP-65278	MHMXW218-09	JF754265
3211	Telemiades fides	Pyrginae	08-SRNP-65292	MHMXW219-09	JF754266
3212	Telemiades fides	Pyrginae	08-SRNP-65279	MHMXW220-09	JF754267
3213	Telemiades fides	Pyrginae	08-SRNP-45093	MHMXW221-09	JF754268
3214	Telemiades fides	Pyrginae	08-SRNP-45143	MHMXW222-09	JF754269
3215	Telemiades fides	Pyrginae	07-SRNP-61184	MHMXW224-09	JF754271
3216	Telemiades fides	Pyrginae	08-SRNP-21378	MHMXW225-09	JF754272
3217	Telemiades fides	Pyrginae	08-SRNP-2123	MHMXW226-09	JF754273
3218	Telemiades fides	Pyrginae	08-SRNP-40973	MHMXW227-09	JF754274
3219	Telemiades fides	Pyrginae	08-SRNP-55982	MHMXW229-09	JF754275
3220	Telemiades fides	Pyrginae	08-SRNP-2424	MHMXW230-09	JF754276
3221	Telemiades fides	Pyrginae	08-SRNP-1346	MHMXW231-09	JF754277
3222	Telemiades fides	Pyrginae	07-SRNP-61358	MHMXW232-09	JF754278
3223	Telemiades fides	Pyrginae	08-SRNP-2357	MHMXW233-09	JF754279
3224	Telemiades fides	Pyrginae	07-SRNP-60943	MHMXW234-09	JF754280
3225	Telemiades fides	Pyrginae	08-SRNP-55069	MHMXW235-09	JF754281
3226	Telemiades fides	Pyrginae	08-SRNP-55417	MHMXW236-09	JF754282
3227	Telemiades fides	Pyrginae	08-SRNP-65209	MHMXW237-09	JF754283
3228	Telemiades fides	Pyrginae	08-SRNP-65076	MHMXW238-09	JF754284
3229	Telemiades fides	Pyrginae	08-SRNP-2338	MHMXW240-09	JF754285
3230	Telemiades fides	Pyrginae	08-SRNP-2339	MHMXW241-09	JF754286
3231	Telemiades fides	Pyrginae	08-SRNP-65207	MHMXW242-09	JF754287
3232	Telemiades fides	Pyrginae	08-SRNP-700	MHMXW243-09	JF754288
3233	Telemiades fides	Pyrginae	08-SRNP-65456	MHMXW244-09	JF754289
3234	Telemiades fides	Pyrginae	08-SRNP-55442	MHMXW245-09	JF754290
3235	Telemiades fides	Pyrginae	08-SRNP-1136	MHMXW246-09	JF754291
3236	Telemiades fides	Pyrginae	08-SRNP-65115	MHMXW247-09	JF754292
3237	Telemiades fides	Pyrginae	08-SRNP-55513	MHMXW248-09	JF754293
3238	Telemiades fides	Pyrginae	02-SRNP-31483	CSRII691-05	DQ293562
3239	Telemiades fides	Pyrginae	02-SRNP-33670	CSRII688-05	DQ293559
3240	Telemiades fides	Pyrginae	02-SRNP-34173	CSRII711-05	DQ293573
3241	Telemiades fides	Pyrginae	02-SRNP-5432	CSRII698-05	DQ293567
3242	Telemiades fides	Pyrginae	02-SRNP-5434	CSRII695-05	DQ293566
3243	Telemiades fides	Pyrginae	02-SRNP-31828	CSRII703-05	DQ293570
3244	Telemiades fides	Pyrginae	02-SRNP-5700	CSRII679-05	DQ293550
3245	Telemiades fides	Pyrginae	02-SRNP-27163	CSRII687-05	DQ293558
3246	Telemiades fides	Pyrginae	02-SRNP-14146	CSRII674-05	DQ293546
3247	Telemiades fides	Pyrginae	02-SRNP-5436	CSRII671-05	DQ293544

Tree Order	Species	Subfamily	ACG Sampleid	BOLD Processid	Genbank Accession
3248	Telemiades fides	Pyrginae	02-SRNP-19106	CSRII692-05	DQ293563
3249	Telemiades fides	Pyrginae	02-SRNP-18972	CSRII699-05	DQ293568
3250	Telemiades fides	Pyrginae	02-SRNP-2328	CSRII712-05	DQ293574
3251	Telemiades fides	Pyrginae	02-SRNP-2456	CSRII675-05	DQ293547
3252	Telemiades fides	Pyrginae	03-SRNP-5752	CSRII682-05	DQ293553
3253	Telemiades fides	Pyrginae	03-SRNP-5014	CSRII685-05	DQ293556
3254	Telemiades fides	Pyrginae	03-SRNP-31075	CSRII690-05	DQ293561
3255	Telemiades fides	Pyrginae	02-SRNP-20490	CSRII693-05	DQ293564
3256	Telemiades fides	Pyrginae	02-SRNP-18846	CSRII704-05	DQ293571
3257	Telemiades fides	Pyrginae	07-SRNP-4623	MHMXS024-08	JF763155
3258	Telemiades fides	Pyrginae	07-SRNP-4756	MHMXS003-08	JF763175
3259	Telemiades fides	Pyrginae	07-SRNP-429	MHAHK333-07	JF761206
3260	Telemiades fides	Pyrginae	07-SRNP-20828	MHAHL495-07	JF763141
3261	Telemiades fides	Pyrginae	08-SRNP-20412	MHMXW203-09	JF754250
3262	Telemiades fides	Pyrginae	05-SRNP-45213	MHAHF567-06	GU150911
3263	Telemiades fides	Pyrginae	07-SRNP-3837	MHMXR575-08	JF763201
3264	Telemiades fides	Pyrginae	07-SRNP-33828	MHMXR573-08	JF763202
3265	Telemiades fides	Pyrginae	07-SRNP-3730	MHMXO927-08	JF763208
3266	Telemiades fides	Pyrginae	05-SRNP-55621	MHAHF937-06	GU150914
3267	Telemiades fides	Pyrginae	96-SRNP-992	CSRII749-05	DQ293577
3268	Telemiades fides	Pyrginae	00-SRNP-20860	CSRII748-05	DQ293576
3269	Telemiades fides	Pyrginae	92-SRNP-6139	CSRII763-05	DQ293579
3270	Telemiades fides	Pyrginae	02-SRNP-31421	CSCR464-04	DQ293541
3271	Telemiades fides	Pyrginae	92-SRNP-5681	CSRII762-05	DQ293578
3272	Telemiades fides	Pyrginae	03-SRNP-12110	CSRII670-05	DQ293543
3273	Telemiades fides	Pyrginae	06-SRNP-12339	MHAHG735-06	GU151743
3274	Telemiades fides	Pyrginae	07-SRNP-1494	MHAHK341-07	JF761213
3275	Telemiades fides	Pyrginae	07-SRNP-4234	MHMXR600-08	JF763178
3276	Telemiades fides	Pyrginae	07-SRNP-60566	MHMXR571-08	JF763204
3277	Telemiades fides	Pyrginae	07-SRNP-30817	MHAHK345-07	JF761217
3278	Telemiades fides	Pyrginae	08-SRNP-20211	MHMXW208-09	JF754255
3279	Telemiades fides	Pyrginae	07-SRNP-42397	MHMXO933-08	JF763207
3280	Telemiades fides	Pyrginae	07-SRNP-3474	MHMXO923-08	JF763211
3281	Telemiades fides	Pyrginae	07-SRNP-4408	MHMXS012-08	JF763166
3282	Telemiades fides	Pyrginae	08-SRNP-65408	MHMXW217-09	JF754264
3283	Telemiades fides	Pyrginae	07-SRNP-4099	MHMXR602-08	JF763177
3284	Telemiades fides	Pyrginae	07-SRNP-3733	MHMXO926-08	JF763209
3285	Telemiades fides	Pyrginae	07-SRNP-5157	MHMXS009-08	JF763169
3286	Telemiades fides	Pyrginae	07-SRNP-4492	MHMXS023-08	JF763156
3287	Telemiades fides	Pyrginae	07-SRNP-24713	MHMXW250-09	JF754295
3288	Telemiades fides	Pyrginae	08-SRNP-65206	MHMXW249-09	JF754294
3289	Telemiades fides	Pyrginae	08-SRNP-55064	MHMXW251-09	JF754296
3290	Telemiades fides	Pyrginae	08-SRNP-55444	MHMXW255-09	JF754299
3291	Telemiades fides	Pyrginae	07-SRNP-60770	MHMXW256-09	JF754300
3292	Telemiades fides	Pyrginae	08-SRNP-65185	MHMXW259-09	JF754303
3293	Telemiades fides	Pyrginae	08-SRNP-2343	MHMXW261-09	JF754305
3294	Telemiades fides	Pyrginae	07-SRNP-15871	MHMXX1174-09	JF778537
3295	Telemiades fides	Pyrginae	07-SRNP-16610	MHMXX1175-09	JF778538
3296	Telemiades fides	Pyrginae	08-SRNP-20930	MHMXX1181-09	JF778539
3297	Telemiades fides	Pyrginae	07-SRNP-31883	MHMXX148-09	JF778529
3298	Telemiades fides	Pyrginae	07-SRNP-15746	MHMXX720-09	JF778532
3299	Telemiades fides	Pyrginae	07-SRNP-16467	MHMXX721-09	JF778533
3300	Telemiades fides	Pyrginae	08-SRNP-5844	MHMXY974-09	GU666547
3301	Telemiades fides	Pyrginae	08-SRNP-5513	MHMXY975-09	GU666548
3302	Telemiades fides	Pyrginae	07-SRNP-30816	MHMXX153-09	JF778530
3303	Telemiades fides	Pyrginae	07-SRNP-16643	MHMXX719-09	JF778531

Tree Order	Species	Subfamily	ACG Sampleid	BOLD Processid	Genbank Accession
3304	Telemiades fides	Pyrginae	07-SRNP-16048	MHMXX723-09	JF778535
3305	Telemiades fides	Pyrginae	07-SRNP-16680	MHMXX724-09	JF778536
3306	Telemiades fides	Pyrginae	08-SRNP-66070	MHMXY976-09	GU666549
3307	Telemiades fides	Pyrginae	08-SRNP-23199	MHMXY977-09	GU666550
3308	Telemiades fides	Pyrginae	07-SRNP-20485	MHAHK330-07	JF761203
3309	Telemiades fides	Pyrginae	07-SRNP-20483	MHAHK331-07	JF761204
3310	Telemiades fides	Pyrginae	06-SRNP-23268	MHAHK332-07	JF761205
3311	Telemiades fides	Pyrginae	06-SRNP-67814	MHAHK335-07	JF761207
3312	Telemiades fides	Pyrginae	07-SRNP-55749	MHAHK337-07	JF761209
3313	Telemiades fides	Pyrginae	06-SRNP-22405	MHAHK340-07	JF761212
3314	Telemiades fides	Pyrginae	02-SRNP-14139	CSRII683-05	DQ293554
3315	Telemiades fides	Pyrginae	02-SRNP-5368	CSRII673-05	DQ293545
3316	Telemiades fides	Pyrginae	02-SRNP-10004	CSRII686-05	DQ293557
3317	Telemiades fides	Pyrginae	02-SRNP-10052	CSRII702-05	DQ293569
3318	Telemiades fides	Pyrginae	02-SRNP-13773	CSRII710-05	DQ293572
3319	Telemiades fides	Pyrginae	01-SRNP-9333	CSRII717-05	DQ293575
3320	Telemiades fides	Pyrginae	05-SRNP-55777	MHAHF928-06	GU150915
3321	Telemiades fides	Pyrginae	04-SRNP-36226	MHAHF929-06	GU150904
3322	Telemiades fides	Pyrginae	05-SRNP-2772	MHAHF930-06	GU150908
3323	Telemiades fides	Pyrginae	05-SRNP-1858	MHAHF931-06	GU150907
3324	Telemiades fides	Pyrginae	05-SRNP-32225	MHAHF932-06	GU150918
3325	Telemiades fides	Pyrginae	05-SRNP-13190	MHAHF933-06	GU150919
3326	Telemiades fides	Pyrginae	05-SRNP-55784	MHAHF934-06	GU150917
3327	Telemiades fides	Pyrginae	05-SRNP-55783	MHAHF936-06	GU150909
3328	Telemiades fides	Pyrginae	05-SRNP-1265	MHAHF568-06	GU150905
3329	Telemiades fides	Pyrginae	05-SRNP-55774	MHAHF569-06	GU150906
3330	Telemiades fides	Pyrginae	06-SRNP-12677	MHAHG733-06	GU151742
3331	Telemiades fides	Pyrginae	06-SRNP-12708	MHAHG734-06	GU151741
3332	Telemiades fides	Pyrginae	06-SRNP-60264	MHMXH878-07	JF761201
3333	Telemiades fides	Pyrginae	06-SRNP-60266	MHMXH879-07	JF761202
3334	Telemiades fides	Pyrginae	02-SRNP-1254	CSRII689-05	DQ293560
3335	Telemiades fides	Pyrginae	02-SRNP-2194	CSRII684-05	DQ293555
3336	Telemiades fides	Pyrginae	09-SRNP-65293	MHMYE883-09	GU653718
3337	Telemiades fides	Pyrginae	09-SRNP-65344	MHMYE884-09	GU653719
3338	Telemiades fides	Pyrginae	09-SRNP-44485	MHMYE1502-09	GU653502
3339	Telemiades fides	Pyrginae	09-SRNP-66013	MHMYH122-10	HM887274
3340	Telemiades Burns02	Pyrginae	05-SRNP-40200	MHAHD384-05	GU161887
3341	Telemiades Burns02	Pyrginae	05-SRNP-2164	MHAHE359-05	GU150113
3342	Telemiades Burns02	Pyrginae	05-SRNP-33036	MHAHF622-06	GU150903
3343	Telemiades Burns02	Pyrginae	05-SRNP-43775	MHAHF623-06	GU150902
3344	Telemiades Burns02	Pyrginae	07-SRNP-3595	MHMXP190-08	JF763124
3345	Telemiades gallius	Pyrginae	00-SRNP-4456	CSCR262-04	DQ293581
3346	Telemiades gallius	Pyrginae	99-SRNP-13875	CSCR261-04	DQ293580
3347	Telemiades chrysorrhoeaDHJ01	Pyrginae	03-SRNP-3888	XAA823-04	DQ293521
3348	Telemiades chrysorrhoeaDHJ01	Pyrginae	02-SRNP-23372	CSRII160-04	DQ293506
3349	Telemiades chrysorrhoeaDHJ01	Pyrginae	02-SRNP-23231	MHAHC273-05	DQ293534
3350	Telemiades chrysorrhoeaDHJ01	Pyrginae	04-SRNP-35467	MHAHD897-05	GU161888
3351	Telemiades chrysorrhoeaDHJ01	Pyrginae	03-SRNP-4126	XAA829-04	DQ293522
3352	Telemiades chrysorrhoeaDHJ01	Pyrginae	03-SRNP-4445	XAA813-04	DQ293516
3353	Telemiades chrysorrhoeaDHJ01	Pyrginae	03-SRNP-3666	XAA831-04	DQ293523
3354	Telemiades chrysorrhoeaDHJ01	Pyrginae	03-SRNP-4745	XAA815-04	DQ293517
3355	Telemiades chrysorrhoeaDHJ01	Pyrginae	03-SRNP-4261	XAA799-04	DQ293513
3356	Telemiades chrysorrhoeaDHJ01	Pyrginae	02-SRNP-9407	XAA783-04	DQ293512
3357	Telemiades chrysorrhoeaDHJ01	Pyrginae	02-SRNP-9730	XAA767-04	DQ293510
3358	Telemiades chrysorrhoeaDHJ01	Pyrginae	02-SRNP-9405	XAA832-04	DQ293524
3359	Telemiades chrysorrhoeaDHJ01	Pyrginae	02-SRNP-9230	XAA816-04	DQ293518

Tree Order	Species	Subfamily	ACG Sampleid	BOLD Processid	Genbank Accession
3360	Telemiades chrysorrhoeaDHJ01	Pyrginae	07-SRNP-36106	MHMXP126-08	JF763126
3361	Telemiades chrysorrhoeaDHJ01	Pyrginae	00-SRNP-9621	MHAHC368-05	DQ293539
3362	Telemiades chrysorrhoeaDHJ01	Pyrginae	00-SRNP-9804	MHAHC367-05	DQ293538
3363	Telemiades chrysorrhoeaDHJ01	Pyrginae	01-SRNP-6022	MHAHC366-05	DQ293537
3364	Telemiades chrysorrhoeaDHJ01	Pyrginae	02-SRNP-8012	MHAHC269-05	DQ293531
3365	Telemiades chrysorrhoeaDHJ01	Pyrginae	01-SRNP-6829	MHAHC365-05	DQ293536
3366	Telemiades chrysorrhoeaDHJ01	Pyrginae	03-SRNP-4023	MHAHC267-05	DQ293530
3367	Telemiades chrysorrhoeaDHJ01	Pyrginae	03-SRNP-4298	MHAHC266-05	DQ293529
3368	Telemiades chrysorrhoeaDHJ01	Pyrginae	03-SRNP-4631	MHAHC265-05	DQ293528
3369	Telemiades chrysorrhoeaDHJ01	Pyrginae	01-SRNP-6010	MHAHC364-05	DQ293535
3370	Telemiades chrysorrhoeaDHJ01	Pyrginae	03-SRNP-4020	XAA822-04	DQ293520
3371	Telemiades chrysorrhoeaDHJ01	Pyrginae	03-SRNP-4299	MHAHC264-05	DQ293527
3372	Telemiades chrysorrhoeaDHJ01	Pyrginae	02-SRNP-23178	CSRII519-04	DQ293507
3373	Telemiades chrysorrhoeaDHJ01	Pyrginae	02-SRNP-23351	CSRII520-04	DQ293508
3374	Telemiades chrysorrhoeaDHJ01	Pyrginae	02-SRNP-23358	CSRII521-04	DQ293509
3375	Telemiades chrysorrhoeaDHJ01	Pyrginae	02-SRNP-23070	XAA821-04	DQ293519
3376	Telemiades chrysorrhoeaDHJ01	Pyrginae	03-SRNP-4448	XAA807-04	DQ293515
3377	Telemiades chrysorrhoeaDHJ01	Pyrginae	02-SRNP-9520	XAA775-04	DQ293511
3378	Telemiades chrysorrhoeaDHJ01	Pyrginae	03-SRNP-4025	XAA805-04	DQ293514
3379	Telemiades chrysorrhoeaDHJ01	Pyrginae	02-SRNP-9560	XAA841-04	DQ293526
3380	Telemiades chrysorrhoeaDHJ01	Pyrginae	02-SRNP-9227	XAA840-04	DQ293525
3381	Telemiades chrysorrhoeaDHJ01	Pyrginae	02-SRNP-8011	MHAHC270-05	DQ293532
3382	Telemiades chrysorrhoeaDHJ01	Pyrginae	03-SRNP-4743	MHAHC271-05	DQ293533
3383	Telemiades chrysorrhoeaDHJ01	Pyrginae	01-SRNP-6170	MHAHC369-05	DQ293540
3384	Telemiades chrysorrhoeaDHJ01	Pyrginae	04-SRNP-35573	MHAHD898-05	GU161889
3385	Telemiades chrysorrhoeaDHJ01	Pyrginae	08-SRNP-35603	MHMXX490-09	JF778526
3386	Telemiades chrysorrhoeaDHJ01	Pyrginae	08-SRNP-35580	MHMXX492-09	JF778527
3387	Telemiades chrysorrhoeaDHJ01	Pyrginae	08-SRNP-35605	MHMXX497-09	JF778528
3388	Telemiades chrysorrhoeaDHJ02	Pyrginae	03-SRNP-4636	XAA830-04	JF750786
3389	Telemiades chrysorrhoeaDHJ02	Pyrginae	03-SRNP-4892	XAA814-04	JF750785
3390	Telemiades chrysorrhoeaDHJ02	Pyrginae	08-SRNP-35601	MHMXX493-09	GU666362
3391	Telemiades chrysorrhoeaDHJ02	Pyrginae	02-SRNP-8074	CSRII516-04	JF750790
3392	Telemiades chrysorrhoeaDHJ02	Pyrginae	03-SRNP-4837	XAA782-04	JF750782
3393	Telemiades chrysorrhoeaDHJ02	Pyrginae	02-SRNP-23202	MHAHC272-05	JF750794
3394	Telemiades chrysorrhoeaDHJ02	Pyrginae	08-SRNP-35613	MHMXX496-09	GU666361
3395	Telemiades chrysorrhoeaDHJ02	Pyrginae	08-SRNP-35384	MHMXX495-09	GU666360
3396	Telemiades chrysorrhoeaDHJ02	Pyrginae	08-SRNP-35968	MHMXX494-09	GU666363
3397	Telemiades chrysorrhoeaDHJ02	Pyrginae	08-SRNP-35614	MHMXX491-09	GU666365
3398	Telemiades chrysorrhoeaDHJ02	Pyrginae	08-SRNP-35579	MHMXX498-09	GU666358
3399	Telemiades chrysorrhoeaDHJ02	Pyrginae	08-SRNP-35966	MHMXX499-09	GU666359
3400	Telemiades chrysorrhoeaDHJ02	Pyrginae	03-SRNP-4632	XAA846-04	JF750789
3401	Telemiades chrysorrhoeaDHJ02	Pyrginae	03-SRNP-4447	XAA838-04	JF750788
3402	Telemiades chrysorrhoeaDHJ02	Pyrginae	02-SRNP-9408	CSRII517-04	JF750791
3403	Telemiades chrysorrhoeaDHJ02	Pyrginae	02-SRNP-9930	CSRII518-04	JF750792
3404	Telemiades chrysorrhoeaDHJ02	Pyrginae	03-SRNP-4026	XAA765-04	JF750779
3405	Telemiades chrysorrhoeaDHJ02	Pyrginae	03-SRNP-4297	XAA773-04	JF750780
3406	Telemiades chrysorrhoeaDHJ02	Pyrginae	03-SRNP-4742	XAA797-04	JF750784
3407	Telemiades chrysorrhoeaDHJ02	Pyrginae	03-SRNP-3440	CSCR425-04	JF750797
3408	Telemiades chrysorrhoeaDHJ02	Pyrginae	03-SRNP-3441	CSCR424-04	JF750798
3409	Telemiades chrysorrhoeaDHJ02	Pyrginae	02-SRNP-23096	CSRII158-04	JF750793
3410	Telemiades chrysorrhoeaDHJ02	Pyrginae	02-SRNP-23179	CSRII159-04	JF750799
3411	Telemiades chrysorrhoeaDHJ02	Pyrginae	00-SRNP-9561	MHAHC372-05	JF750801
3412	Telemiades chrysorrhoeaDHJ02	Pyrginae	01-SRNP-6918	MHAHC371-05	JF750796
3413	Telemiades chrysorrhoeaDHJ02	Pyrginae	02-SRNP-9664	MHAHC277-05	JF750795
3414	Telemiades chrysorrhoeaDHJ02	Pyrginae	01-SRNP-6864	MHAHC370-05	JF750800
3415	Telemiades chrysorrhoeaDHJ02	Pyrginae	02-SRNP-9445	XAA758-04	JF750778

Tree Order	Species	Subfamily	ACG Sampleid	BOLD Processid	Genbank Accession
3416	Telemiades chrysorrhoeaDHJ02	Pyrginae	02-SRNP-9929	XAA790-04	JF750783
3417	Telemiades chrysorrhoeaDHJ02	Pyrginae	02-SRNP-23273	XAA833-04	JF750787
3418	Telemiades chrysorrhoeaDHJ02	Pyrginae	02-SRNP-23201	XAA774-04	JF750781
3419	Telemiades chrysorrhoeaDHJ02	Pyrginae	08-SRNP-37068	MHMYE893-09	GU653711
3420	Telemiades megallus	Pyrginae	08-SRNP-66068	MHMXY1089-09	GU666454
3421	Telemiades megallus	Pyrginae	08-SRNP-40055	MHMXT088-08	JF763222
3422	Telemiades megallus	Pyrginae	07-SRNP-42824	MHMXT089-08	JF763221
3423	Telemiades megallus	Pyrginae	03-SRNP-31164	CSCR428-04	DQ293583
3424	Telemiades megallus	Pyrginae	07-SRNP-3719	MHMXR624-08	JF763234
3425	Telemiades megallus	Pyrginae	08-SRNP-40056	MHMXT170-08	JF763223
3426	Telemiades megallus	Pyrginae	07-SRNP-42778	MHMXT169-08	JF763224
3427	Telemiades megallus	Pyrginae	07-SRNP-42752	MHMXR770-08	JF763227
3428	Telemiades megallus	Pyrginae	07-SRNP-33307	MHMXP172-08	JF763236
3429	Telemiades megallus	Pyrginae	07-SRNP-24437	MHMXR627-08	JF763231
3430	Telemiades megallus	Pyrginae	07-SRNP-43135	MHMXT090-08	JF763220
3431	Telemiades megallus	Pyrginae	07-SRNP-43082	MHMXX932-09	JF778540
3432	Telemiades megallus	Pyrginae	09-SRNP-69855	MHMYG2445-10	HM885870
3433	Telemiades megallus	Pyrginae	07-SRNP-42971	MHMXT168-08	JF763225
3434	Telemiades megallus	Pyrginae	07-SRNP-42910	MHMXT092-08	JF763218
3435	Telemiades megallus	Pyrginae	07-SRNP-3809	MHMXR630-08	JF763228
3436	Telemiades megallus	Pyrginae	07-SRNP-42969	MHMXR629-08	JF763229
3437	Telemiades megallus	Pyrginae	07-SRNP-33308	MHMXP189-08	JF763235
3438	Telemiades megallus	Pyrginae	07-SRNP-41026	MHAHL248-07	JF763216
3439	Telemiades megallus	Pyrginae	07-SRNP-30422	MHMXK037-07	JF763237
3440	Telemiades megallus	Pyrginae	05-SRNP-24526	MHAHF621-06	GU150921
3441	Telemiades megallus	Pyrginae	04-SRNP-111	CSRII157-04	DQ293585
3442	Telemiades megallus	Pyrginae	03-SRNP-30710	CSCR583-04	DQ293584
3443	Telemiades megallus	Pyrginae	07-SRNP-42440	MHMXR628-08	JF763230
3444	Telemiades megallus	Pyrginae	00-SRNP-21554	CSCR263-04	DQ293582
3445	Telemiades megallus	Pyrginae	07-SRNP-4414	MHMXR625-08	JF763233
3446	Telemiades megallus	Pyrginae	07-SRNP-42987	MHMXT091-08	JF763219
3447	Telemiades megallus	Pyrginae	07-SRNP-5171	MHMXT093-08	JF763217
3448	Telemiades megallus	Pyrginae	07-SRNP-42879	MHMXT167-08	JF763226
3449	Telemiades megallus	Pyrginae	09-SRNP-69856	MHMYG2451-10	HM885877
3450	Telemiades oiclus	Pyrginae	03-SRNP-7387	MHAHJ145-07	JF753191
3451	Telemiades oiclus	Pyrginae	05-SRNP-5462	MHAHF503-06	GU150926
3452	Telemiades oiclus	Pyrginae	03-SRNP-12536.1	MHAHJ143-07	JF753189
3453	Telemiades oiclus	Pyrginae	04-SRNP-30865	MHAHJ140-07	JF753186
3454	Telemiades oiclus	Pyrginae	04-SRNP-55048	MHAHE351-05	GU150115
3455	Telemiades oiclus	Pyrginae	04-SRNP-56957	MHAHE350-05	GU150116
3456	Telemiades oiclus	Pyrginae	04-SRNP-56954	MHAHE349-05	GU150114
3457	Telemiades oiclus	Pyrginae	03-SRNP-7308	CSCR400-04	DQ293124
3458	Telemiades oiclus	Pyrginae	01-SRNP-3727	CSCR214-04	DQ293123
3459	Telemiades oiclus	Pyrginae	03-SRNP-12588.1	MHAHJ139-07	JF753185
3460	Telemiades oiclus	Pyrginae	03-SRNP-20865	MHAHJ141-07	JF753187
3461	Telemiades oiclus	Pyrginae	00-SRNP-22202	MHAHJ142-07	JF753188
3462	Telemiades oiclus	Pyrginae	01-SRNP-2947	MHAHJ146-07	JF753192
3463	Telemiades oiclus	Pyrginae	07-SRNP-2201	MHAHL222-07	JF763243
3464	Telemiades oiclus	Pyrginae	01-SRNP-25089	MHAHJ144-07	JF753190
3465	Telemiades oiclus	Pyrginae	05-SRNP-42621	MHAHF504-06	GU150927
3466	Telemiades oiclus	Pyrginae	07-SRNP-2119	MHAHL223-07	JF763244
3467	Telemiades oiclus	Pyrginae	07-SRNP-1786	MHAHL224-07	JF763245
3468	Telemiades oiclus	Pyrginae	07-SRNP-2582	MHAHL225-07	JF763246
3469	Telemiades oiclus	Pyrginae	07-SRNP-33526	MHMXR897-08	JF763247
3470	Telemiades nicomedes	Pyrginae	07-SRNP-42342	MHMXP209-08	JF763241
3471	Telemiades nicomedes	Pyrginae	05-SRNP-33522	MHAHF620-06	GU150925

Tree Order	Species	Subfamily	ACG Sampleid	BOLD Processid	Genbank Accession
3472	Telemiades nicomedes	Pyrginae	06-SRNP-42935	MHAHI608-06	GU156359
3473	Telemiades nicomedes	Pyrginae	05-SRNP-33746	MHAHF617-06	GU150922
3474	Telemiades nicomedes	Pyrginae	03-SRNP-21802	CSCR584-04	DQ293586
3475	Telemiades nicomedes	Pyrginae	03-SRNP-27835	CSRII161-04	DQ293587
3476	Telemiades nicomedes	Pyrginae	04-SRNP-2174	XAA794-04	DQ293589
3477	Telemiades nicomedes	Pyrginae	05-SRNP-43154	MHAHF619-06	GU150924
3478	Telemiades nicomedes	Pyrginae	06-SRNP-31788	MHAHH466-06	GU155672
3479	Telemiades nicomedes	Pyrginae	06-SRNP-31705	MHAHH467-06	GU155673
3480	Telemiades nicomedes	Pyrginae	06-SRNP-43594	MHAHJ483-07	JF753184
3481	Telemiades nicomedes	Pyrginae	06-SRNP-65735	MHMXK061-07	JF763242
3482	Telemiades nicomedes	Pyrginae	07-SRNP-42490	MHMXR623-08	JF763240
3483	Telemiades nicomedes	Pyrginae	08-SRNP-65358	MHMXS086-08	JF763239
3484	Telemiades nicomedes	Pyrginae	08-SRNP-65381	MHMXW545-09	JF754306
3485	Telemiades nicomedes	Pyrginae	08-SRNP-40801	MHMXX1068-09	JF778541
3486	Telemiades nicomedes	Pyrginae	08-SRNP-22689	MHMXX580-09	JF778542
3487	Telemiades nicomedes	Pyrginae	08-SRNP-22691	MHMXX581-09	JF778543
3488	Telemiades nicomedes	Pyrginae	08-SRNP-2225	MHMXW546-09	JF754307
3489	Telemiades nicomedes	Pyrginae	05-SRNP-33923	MHAHF618-06	GU150923
3490	Telemiades nicomedes	Pyrginae	06-SRNP-44852	MHMXH858-07	JF761222
3491	Telemiades nicomedes	Pyrginae	05-SRNP-34684	MHAHG224-06	GU151744
3492	Telemiades nicomedes	Pyrginae	03-SRNP-38197	CSRII162-04	DQ293588
3493	Telemiades nicomedes	Pyrginae	08-SRNP-40117	MHMXT166-08	JF763238
3494	Telemiades nicomedes	Pyrginae	08-SRNP-66200	MHMXY1076-09	GU666466
3495	Telemiades nicomedes	Pyrginae	08-SRNP-72830	MHMXY1077-09	GU666459
3496	Telemiades Burns01	Pyrginae	98-SRNP-6688	CSRII223-04	DQ293501
3497	Telemiades Burns01	Pyrginae	02-SRNP-19058	CSRII224-04	DQ293502
3498	Telemiades Burns01	Pyrginae	07-SRNP-4410	MHMXR587-08	JF763116
3499	Telemiades Burns01	Pyrginae	07-SRNP-3801	MHMXO919-08	JF763121
3500	Telemiades Burns01	Pyrginae	07-SRNP-3542	MHMXO928-08	JF763119
3501	Telemiades Burns01	Pyrginae	07-SRNP-3804	MHMXO925-08	JF763120
3502	Telemiades Burns01	Pyrginae	07-SRNP-3803	MHMXO917-08	JF763123
3503	Telemiades Burns01	Pyrginae	07-SRNP-2223	MHAHL501-07	JF763111
3504	Telemiades Burns01	Pyrginae	07-SRNP-1335	MHAHK348-07	JF761200
3505	Telemiades Burns01	Pyrginae	02-SRNP-19057	CSRII696-05	DQ293504
3506	Telemiades Burns01	Pyrginae	03-SRNP-13003.1	CSRII700-05	DQ293505
3507	Telemiades Burns01	Pyrginae	07-SRNP-3807	MHMXR574-08	JF763118
3508	Telemiades Burns01	Pyrginae	07-SRNP-4083	MHMXR577-08	JF763117
3509	Telemiades Burns01	Pyrginae	07-SRNP-4699	MHMXS020-08	JF763114
3510	Telemiades Burns01	Pyrginae	08-SRNP-79	MHMXS027-08	JF763113
3511	Telemiades Burns01	Pyrginae	08-SRNP-80	MHMXS028-08	JF763112
3512	Telemiades Burns01	Pyrginae	08-SRNP-941	MHMXW200-09	JF754229
3513	Telemiades Burns01	Pyrginae	08-SRNP-41014	MHMXW228-09	JF754230
3514	Telemiades Burns01	Pyrginae	08-SRNP-2336	MHMXW239-09	JF754231
3515	Telemiades Burns01	Pyrginae	02-SRNP-18967	CSRII672-05	DQ293503
3516	Telemiades Burns01	Pyrginae	07-SRNP-3519	MHMXO918-08	JF763122
3517	Telemiades Burns01	Pyrginae	07-SRNP-4034	MHMXR601-08	JF763115
3518	Telemiades Burns01	Pyrginae	08-SRNP-1107	MHMXW252-09	JF754232
3519	Telemiades Burns08	Pyrginae	08-SRNP-65335	MHMXS076-08	JF763125
3520	Telemiades Burns08	Pyrginae	09-SRNP-65738	MHMYC379-09	GU649933
3521	Telemiades Burns08	Pyrginae	09-SRNP-65754	MHMYE1494-09	GU653510
3522	Telemiades antiopeDHJ04	Pyrginae	03-SRNP-12790.1	CSRII154-04	DQ293471
3523	Telemiades antiopeDHJ04	Pyrginae	08-SRNP-65458	MHMXW286-09	JF754226
3524	Telemiades antiopeDHJ04	Pyrginae	07-SRNP-5113	MHMXT003-08	JF763095
3525	Telemiades antiopeDHJ04	Pyrginae	08-SRNP-40546	MHMXW279-09	JF754224
3526	Telemiades antiopeDHJ04	Pyrginae	08-SRNP-20658	MHMXW278-09	JF754223
3527	Telemiades antiopeDHJ04	Pyrginae	08-SRNP-2356	MHMXW271-09	JF754221

Tree Order	Species	Subfamily	ACG Sampleid	BOLD Processid	Genbank Accession
3528	Telemiades antiopeDHJ04	Pyrginae	07-SRNP-5149	MHMXW263-09	JF754220
3529	Telemiades antiopeDHJ04	Pyrginae	08-SRNP-213	MHMXW262-09	JF754219
3530	Telemiades antiopeDHJ04	Pyrginae	07-SRNP-4662	MHMXS073-08	JF763099
3531	Telemiades antiopeDHJ04	Pyrginae	07-SRNP-42683	MHMXS065-08	JF763100
3532	Telemiades antiopeDHJ04	Pyrginae	08-SRNP-25	MHMXS063-08	JF763101
3533	Telemiades antiopeDHJ04	Pyrginae	07-SRNP-65997	MHMXS059-08	JF763102
3534	Telemiades antiopeDHJ04	Pyrginae	07-SRNP-4830	MHMXS052-08	JF763103
3535	Telemiades antiopeDHJ04	Pyrginae	08-SRNP-90	MHMXS042-08	JF763104
3536	Telemiades antiopeDHJ04	Pyrginae	08-SRNP-40014	MHMXS035-08	JF763106
3537	Telemiades antiopeDHJ04	Pyrginae	08-SRNP-78	MHMXS031-08	JF763097
3538	Telemiades antiopeDHJ04	Pyrginae	07-SRNP-4413	MHMXR068-08	JF763098
3539	Telemiades antiopeDHJ04	Pyrginae	07-SRNP-4181	MHMXR618-08	JF763107
3540	Telemiades antiopeDHJ04	Pyrginae	07-SRNP-66030	MHMXR606-08	JF763108
3541	Telemiades antiopeDHJ04	Pyrginae	02-SRNP-5610	XAA877-04	DQ293494
3542	Telemiades antiopeDHJ04	Pyrginae	07-SRNP-4622	MHMXS036-08	JF763105
3543	Telemiades antiopeDHJ04	Pyrginae	08-SRNP-77	MHMXT004-08	JF763094
3544	Telemiades antiopeDHJ04	Pyrginae	07-SRNP-3260	MHMXO932-08	JF763109
3545	Telemiades antiopeDHJ04	Pyrginae	07-SRNP-5121	MHMXT001-08	JF763096
3546	Telemiades antiopeDHJ04	Pyrginae	02-SRNP-31972	XAA793-04	DQ293479
3547	Telemiades antiopeDHJ04	Pyrginae	08-SRNP-20471	MHMXW275-09	JF754222
3548	Telemiades antiopeDHJ04	Pyrginae	08-SRNP-65366	MHMXW283-09	JF754225
3549	Telemiades antiopeDHJ04	Pyrginae	08-SRNP-20470	MHMXW298-09	JF754227
3550	Telemiades antiopeDHJ04	Pyrginae	08-SRNP-85	MHMXW299-09	JF754228
3551	Telemiades antiopeDHJ04	Pyrginae	08-SRNP-22138	MHMXX611-09	JF778524
3552	Telemiades antiopeDHJ04	Pyrginae	08-SRNP-66178	MHMXY967-09	GU666556
3553	Telemiades antiopeDHJ04	Pyrginae	08-SRNP-66078	MHMXY968-09	GU666557
3554	Telemiades antiopeDHJ04	Pyrginae	08-SRNP-32462	MHMXY971-09	GU666552
3555	Telemiades antiopeDHJ02	Pyrginae	08-SRNP-32857	MHMYC470-09	GU649868
3556	Telemiades antiopeDHJ02	Pyrginae	02-SRNP-19299	XAA785-04	DQ293478
3557	Telemiades antiopeDHJ02	Pyrginae	07-SRNP-32031	MHAHL508-07	JF763059
3558	Telemiades antiopeDHJ02	Pyrginae	07-SRNP-4321	MHMXS055-08	JF763064
3559	Telemiades antiopeDHJ02	Pyrginae	08-SRNP-31060	MHMXW284-09	JF754204
3560	Telemiades antiopeDHJ02	Pyrginae	08-SRNP-66086	MHMXY965-09	GU666562
3561	Telemiades antiopeDHJ02	Pyrginae	08-SRNP-66041	MHMXY969-09	GU666558
3562	Telemiades antiopeDHJ02	Pyrginae	01-SRNP-25374	XAA777-04	DQ293477
3563	Telemiades antiopeDHJ02	Pyrginae	07-SRNP-4412	MHMXS049-08	JF763067
3564	Telemiades antiopeDHJ02	Pyrginae	07-SRNP-4814	MHMXS044-08	JF763069
3565	Telemiades antiopeDHJ02	Pyrginae	07-SRNP-4822	MHMXS037-08	JF763070
3566	Telemiades antiopeDHJ02	Pyrginae	07-SRNP-3864	MHMXR609-08	JF763073
3567	Telemiades antiopeDHJ02	Pyrginae	06-SRNP-9304	MHAHJ452-07	JF753182
3568	Telemiades antiopeDHJ02	Pyrginae	00-SRNP-14600	XAA926-04	DQ293498
3569	Telemiades antiopeDHJ02	Pyrginae	07-SRNP-42387	MHMXO915-08	JF763077
3570	Telemiades antiopeDHJ02	Pyrginae	07-SRNP-45352	MHMXO931-08	JF763075
3571	Telemiades antiopeDHJ02	Pyrginae	07-SRNP-4821	MHMXS060-08	JF763063
3572	Telemiades antiopeDHJ02	Pyrginae	07-SRNP-42887	MHMXS067-08	JF763061
3573	Telemiades antiopeDHJ02	Pyrginae	07-SRNP-3822	MHMXS072-08	JF763060
3574	Telemiades antiopeDHJ02	Pyrginae	08-SRNP-2038	MHMXW264-09	JF754201
3575	Telemiades antiopeDHJ02	Pyrginae	08-SRNP-40378	MHMXW291-09	JF754206
3576	Telemiades antiopeDHJ02	Pyrginae	08-SRNP-31062	MHMXW294-09	JF754209
3577	Telemiades antiopeDHJ02	Pyrginae	08-SRNP-1018	MHMXW295-09	JF754210
3578	Telemiades antiopeDHJ02	Pyrginae	08-SRNP-5514	MHMXY970-09	GU666551
3579	Telemiades antiopeDHJ02	Pyrginae	08-SRNP-72542	MHMXY973-09	GU666554
3580	Telemiades antiopeDHJ02	Pyrginae	09-SRNP-40086	MHMYC469-09	GU649834
3581	Telemiades antiopeDHJ02	Pyrginae	07-SRNP-1844	MHAHL476-07	JF763057
3582	Telemiades antiopeDHJ02	Pyrginae	02-SRNP-1005	XAA825-04	DQ293484
3583	Telemiades antiopeDHJ02	Pyrginae	02-SRNP-1008	XAA761-04	DQ293475

Tree Order	Species	Subfamily	ACG Sampleid	BOLD Processid	Genbank Accession
3584	Telemiades antiopeDHJ02	Pyrginae	02-SRNP-1004	XAA834-04	DQ293486
3585	Telemiades antiopeDHJ02	Pyrginae	02-SRNP-1451	XAA826-04	DQ293485
3586	Telemiades antiopeDHJ02	Pyrginae	08-SRNP-41239	MHMXX612-09	JF778523
3587	Telemiades antiopeDHJ02	Pyrginae	08-SRNP-1347	MHMXW301-09	JF754212
3588	Telemiades antiopeDHJ02	Pyrginae	08-SRNP-40203	MHMXW297-09	JF754211
3589	Telemiades antiopeDHJ02	Pyrginae	08-SRNP-30634	MHMXW293-09	JF754208
3590	Telemiades antiopeDHJ02	Pyrginae	08-SRNP-40136	MHMXW292-09	JF754207
3591	Telemiades antiopeDHJ02	Pyrginae	08-SRNP-520	MHMXW285-09	JF754205
3592	Telemiades antiopeDHJ02	Pyrginae	08-SRNP-2122	MHMXW272-09	JF754203
3593	Telemiades antiopeDHJ02	Pyrginae	08-SRNP-75	MHMXW266-09	JF754202
3594	Telemiades antiopeDHJ02	Pyrginae	07-SRNP-4808	MHMXS066-08	JF763062
3595	Telemiades antiopeDHJ02	Pyrginae	07-SRNP-4177	MHMXS051-08	JF763065
3596	Telemiades antiopeDHJ02	Pyrginae	07-SRNP-4810	MHMXS050-08	JF763066
3597	Telemiades antiopeDHJ02	Pyrginae	07-SRNP-4465	MHMXS045-08	JF763068
3598	Telemiades antiopeDHJ02	Pyrginae	07-SRNP-5159	MHMXS034-08	JF763071
3599	Telemiades antiopeDHJ02	Pyrginae	07-SRNP-4154	MHMXR619-08	JF763072
3600	Telemiades antiopeDHJ02	Pyrginae	02-SRNP-1007	XAA934-04	DQ293500
3601	Telemiades antiopeDHJ02	Pyrginae	02-SRNP-19206	XAA870-04	DQ293493
3602	Telemiades antiopeDHJ02	Pyrginae	02-SRNP-4634	XAA809-04	DQ293481
3603	Telemiades antiopeDHJ02	Pyrginae	07-SRNP-30338	MHAHL489-07	JF763058
3604	Telemiades antiopeDHJ02	Pyrginae	07-SRNP-3292	MHMXO935-08	JF763074
3605	Telemiades antiopeDHJ02	Pyrginae	07-SRNP-42386	MHMXO916-08	JF763076
3606	Telemiades antiopeDHJ02	Pyrginae	07-SRNP-42388	MHMXO911-08	JF763078
3607	Telemiades antiopeDHJ02	Pyrginae	02-SRNP-1638	XAA842-04	DQ293487
3608	Telemiades antiopeDHJ02	Pyrginae	02-SRNP-1003	XAA878-04	DQ293495
3609	Telemiades antiopeDHJ02	Pyrginae	02-SRNP-19728	CSCR462-04	DQ293469
3610	Telemiades antiopeDHJ02	Pyrginae	09-SRNP-65787	MHMYC471-09	GU649863
3611	Telemiades antiopeDHJ02	Pyrginae	09-SRNP-69681	MHMYH121-10	HM887273
3612	Telemiades antiopeDHJ03	Pyrginae	03-SRNP-5635	CSRII155-04	DQ293472
3613	Telemiades antiopeDHJ03	Pyrginae	07-SRNP-3532	MHMXO930-08	JF763092
3614	Telemiades antiopeDHJ03	Pyrginae	07-SRNP-5153	MHMXS062-08	JF763083
3615	Telemiades antiopeDHJ03	Pyrginae	08-SRNP-56	MHMXS061-08	JF763084
3616	Telemiades antiopeDHJ03	Pyrginae	07-SRNP-4621	MHMXS039-08	JF763086
3617	Telemiades antiopeDHJ03	Pyrginae	08-SRNP-88	MHMXS032-08	JF763087
3618	Telemiades antiopeDHJ03	Pyrginae	07-SRNP-65881	MHMXR612-08	JF763089
3619	Telemiades antiopeDHJ03	Pyrginae	07-SRNP-4322	MHMXR607-08	JF763090
3620	Telemiades antiopeDHJ03	Pyrginae	07-SRNP-65754	MHMXR604-08	JF763091
3621	Telemiades antiopeDHJ03	Pyrginae	07-SRNP-2288	MHAHL494-07	JF763080
3622	Telemiades antiopeDHJ03	Pyrginae	05-SRNP-31699	MHAHL020-07	JF763079
3623	Telemiades antiopeDHJ03	Pyrginae	07-SRNP-4816	MHMXS048-08	JF763085
3624	Telemiades antiopeDHJ03	Pyrginae	07-SRNP-3834	MHMXR613-08	JF763088
3625	Telemiades antiopeDHJ03	Pyrginae	07-SRNP-3258	MHMXO910-08	JF763093
3626	Telemiades antiopeDHJ03	Pyrginae	08-SRNP-53	MHMXT005-08	JF763082
3627	Telemiades antiopeDHJ03	Pyrginae	08-SRNP-94	MHMXT007-08	JF763081
3628	Telemiades antiopeDHJ03	Pyrginae	08-SRNP-212	MHMXW267-09	JF754213
3629	Telemiades antiopeDHJ03	Pyrginae	08-SRNP-65345	MHMXW269-09	JF754214
3630	Telemiades antiopeDHJ03	Pyrginae	08-SRNP-2235	MHMXW273-09	JF754215
3631	Telemiades antiopeDHJ03	Pyrginae	08-SRNP-65187	MHMXW281-09	JF754216
3632	Telemiades antiopeDHJ03	Pyrginae	08-SRNP-2052	MHMXW288-09	JF754217
3633	Telemiades antiopeDHJ03	Pyrginae	08-SRNP-1022	MHMXW296-09	JF754218
3634	Telemiades antiopeDHJ03	Pyrginae	08-SRNP-5335	MHMXY966-09	GU666555
3635	Telemiades antiopeDHJ03	Pyrginae	09-SRNP-80154	MHMYH123-10	HM887275
3636	Telemiades antiopeDHJ01	Pyrginae	03-SRNP-5756	CSRII156-04	DQ293473
3637	Telemiades antiopeDHJ01	Pyrginae	03-SRNP-5262	CSCR423-04	DQ293470
3638	Telemiades antiopeDHJ01	Pyrginae	07-SRNP-4829	MHMXT010-08	JF763018
3639	Telemiades antiopeDHJ01	Pyrginae	07-SRNP-42569	MHMXR616-08	JF763047

Tree Order	Species	Subfamily	ACG Sampleid	BOLD Processid	Genbank Accession
3640	Telemiades antiopeDHJ01	Pyrginae	07-SRNP-33538	MHMXR603-08	JF763053
3641	Telemiades antiopeDHJ01	Pyrginae	02-SRNP-882	XAA801-04	DQ293480
3642	Telemiades antiopeDHJ01	Pyrginae	02-SRNP-19458	XAA862-04	DQ293491
3643	Telemiades antiopeDHJ01	Pyrginae	07-SRNP-4743	MHMXS041-08	JF763039
3644	Telemiades antiopeDHJ01	Pyrginae	07-SRNP-5126	MHMXS043-08	JF763038
3645	Telemiades antiopeDHJ01	Pyrginae	07-SRNP-4700	MHMXS056-08	JF763033
3646	Telemiades antiopeDHJ01	Pyrginae	07-SRNP-4317	MHMXS057-08	JF763032
3647	Telemiades antiopeDHJ01	Pyrginae	07-SRNP-4480	MHMXS046-08	JF763037
3648	Telemiades antiopeDHJ01	Pyrginae	07-SRNP-4666	MHMXS047-08	JF763036
3649	Telemiades antiopeDHJ01	Pyrginae	07-SRNP-41772	MHAHL503-07	JF763016
3650	Telemiades antiopeDHJ01	Pyrginae	07-SRNP-41859	MHAHL510-07	JF763017
3651	Telemiades antiopeDHJ01	Pyrginae	07-SRNP-32623	MHAHL492-07	JF763015
3652	Telemiades antiopeDHJ01	Pyrginae	05-SRNP-2943	MHAHL022-07	JF763013
3653	Telemiades antiopeDHJ01	Pyrginae	05-SRNP-31617	MHAHL021-07	JF763012
3654	Telemiades antiopeDHJ01	Pyrginae	05-SRNP-31616	MHAHL019-07	JF763011
3655	Telemiades antiopeDHJ01	Pyrginae	06-SRNP-22168	MHAHK334-07	JF761199
3656	Telemiades antiopeDHJ01	Pyrginae	02-SRNP-19799	XAA869-04	DQ293492
3657	Telemiades antiopeDHJ01	Pyrginae	02-SRNP-31830	XAA861-04	DQ293490
3658	Telemiades antiopeDHJ01	Pyrginae	97-SRNP-6271	XAA853-04	DQ293488
3659	Telemiades antiopeDHJ01	Pyrginae	98-SRNP-4297	XAA927-04	DQ293499
3660	Telemiades antiopeDHJ01	Pyrginae	01-SRNP-9439	XAA918-04	DQ293497
3661	Telemiades antiopeDHJ01	Pyrginae	98-SRNP-4296	XAA894-04	DQ293496
3662	Telemiades antiopeDHJ01	Pyrginae	07-SRNP-65162	MHAHL487-07	JF763014
3663	Telemiades antiopeDHJ01	Pyrginae	07-SRNP-4438	MHMXR610-08	JF763050
3664	Telemiades antiopeDHJ01	Pyrginae	08-SRNP-74	MHMXT006-08	JF763021
3665	Telemiades antiopeDHJ01	Pyrginae	07-SRNP-3358	MHMXO929-08	JF763054
3666	Telemiades antiopeDHJ01	Pyrginae	07-SRNP-5012	MHMXT002-08	JF763022
3667	Telemiades antiopeDHJ01	Pyrginae	07-SRNP-3831	MHMXO921-08	JF763055
3668	Telemiades antiopeDHJ01	Pyrginae	07-SRNP-3359	MHMXO912-08	JF763056
3669	Telemiades antiopeDHJ01	Pyrginae	02-SRNP-31831	XAA769-04	DQ293476
3670	Telemiades antiopeDHJ01	Pyrginae	02-SRNP-19598	XAA854-04	DQ293489
3671	Telemiades antiopeDHJ01	Pyrginae	02-SRNP-19126	XAA753-04	DQ293474
3672	Telemiades antiopeDHJ01	Pyrginae	02-SRNP-6041	XAA817-04	DQ293482
3673	Telemiades antiopeDHJ01	Pyrginae	01-SRNP-23390	XAA818-04	DQ293483
3674	Telemiades antiopeDHJ01	Pyrginae	07-SRNP-4015	MHMXR615-08	JF763048
3675	Telemiades antiopeDHJ01	Pyrginae	07-SRNP-4768	MHMXT008-08	JF763020
3676	Telemiades antiopeDHJ01	Pyrginae	07-SRNP-5014	MHMXT009-08	JF763019
3677	Telemiades antiopeDHJ01	Pyrginae	07-SRNP-4178	MHMXR620-08	JF763045
3678	Telemiades antiopeDHJ01	Pyrginae	07-SRNP-4705	MHMXR621-08	JF763044
3679	Telemiades antiopeDHJ01	Pyrginae	07-SRNP-4179	MHMXR622-08	JF763043
3680	Telemiades antiopeDHJ01	Pyrginae	08-SRNP-72	MHMXS029-08	JF763024
3681	Telemiades antiopeDHJ01	Pyrginae	08-SRNP-84	MHMXS030-08	JF763023
3682	Telemiades antiopeDHJ01	Pyrginae	07-SRNP-4472	MHMXS033-08	JF763042
3683	Telemiades antiopeDHJ01	Pyrginae	07-SRNP-4747	MHMXS038-08	JF763041
3684	Telemiades antiopeDHJ01	Pyrginae	07-SRNP-5047	MHMXS040-08	JF763040
3685	Telemiades antiopeDHJ01	Pyrginae	07-SRNP-4146	MHMXS053-08	JF763035
3686	Telemiades antiopeDHJ01	Pyrginae	07-SRNP-4186	MHMXS054-08	JF763034
3687	Telemiades antiopeDHJ01	Pyrginae	07-SRNP-4665	MHMXS058-08	JF763031
3688	Telemiades antiopeDHJ01	Pyrginae	07-SRNP-65996	MHMXS064-08	JF763030
3689	Telemiades antiopeDHJ01	Pyrginae	07-SRNP-4466	MHMXS068-08	JF763029
3690	Telemiades antiopeDHJ01	Pyrginae	07-SRNP-42746	MHMXS069-08	JF763028
3691	Telemiades antiopeDHJ01	Pyrginae	07-SRNP-4202	MHMXS070-08	JF763027
3692	Telemiades antiopeDHJ01	Pyrginae	08-SRNP-65077	MHMXS071-08	JF763026
3693	Telemiades antiopeDHJ01	Pyrginae	08-SRNP-2032	MHMXW274-09	JF754193
3694	Telemiades antiopeDHJ01	Pyrginae	08-SRNP-20474	MHMXW277-09	JF754194
3695	Telemiades antiopeDHJ01	Pyrginae	08-SRNP-241	MHMXW280-09	JF754195

Tree Order	Species	Subfamily	ACG Sampleid	BOLD Processid	Genbank Accession
3696	Telemiades antiopeDHJ01	Pyrginae	08-SRNP-21085	MHMXW282-09	JF754196
3697	Telemiades antiopeDHJ01	Pyrginae	07-SRNP-23802	MHMXR605-08	JF763052
3698	Telemiades antiopeDHJ01	Pyrginae	07-SRNP-4183	MHMXR608-08	JF763051
3699	Telemiades antiopeDHJ01	Pyrginae	07-SRNP-3832	MHMXR611-08	JF763049
3700	Telemiades antiopeDHJ01	Pyrginae	07-SRNP-42570	MHMXR617-08	JF763046
3701	Telemiades antiopeDHJ01	Pyrginae	08-SRNP-593	MHMXW287-09	JF754197
3702	Telemiades antiopeDHJ01	Pyrginae	08-SRNP-31223	MHMXW289-09	JF754198
3703	Telemiades antiopeDHJ01	Pyrginae	07-SRNP-4831	MHMXS095-08	JF763025
3704	Telemiades antiopeDHJ01	Pyrginae	08-SRNP-1861	MHMXW265-09	JF754190
3705	Telemiades antiopeDHJ01	Pyrginae	08-SRNP-65346	MHMXW268-09	JF754191
3706	Telemiades antiopeDHJ01	Pyrginae	08-SRNP-2236	MHMXW270-09	JF754192
3707	Telemiades antiopeDHJ01	Pyrginae	08-SRNP-40725	MHMXW290-09	JF754199
3708	Telemiades antiopeDHJ01	Pyrginae	08-SRNP-1849	MHMXW300-09	JF754200
3709	Telemiades antiopeDHJ01	Pyrginae	08-SRNP-66184	MHMXY964-09	GU666561
3710	Telemiades antiopeDHJ01	Pyrginae	08-SRNP-22019	MHMXY972-09	GU666553
3711	Telemiades antiopeDHJ01	Pyrginae	09-SRNP-66051	MHMYH120-10	HM887272
3712	Telemiades antiopeDHJ01	Pyrginae	09-SRNP-44901	MHMYG1999-10	JF751944
3713	Telemiades Burns03	Pyrginae	06-SRNP-43497	MHAHJ484-07	JF753183
3714	Cogia eluina	Pyrginae	04-SRNP-50096	MHAHL103-07	JF761979
3715	Cogia eluina	Pyrginae	04-SRNP-50103	MHAHL102-07	JF761978
3716	Cogia eluina	Pyrginae	05-SRNP-64296	MHAHH527-06	GU155339
3717	Cogia eluina	Pyrginae	05-SRNP-64303	MHAHH528-06	GU155340
3718	Cogia eluina	Pyrginae	01-SRNP-16128	CSCR078-04	DQ292216
3719	Cogia eluina	Pyrginae	02-SRNP-32045	CSCR080-04	DQ292218
3720	Cogia eluina	Pyrginae	04-SRNP-15978	MHAHF860-06	GU150345
3721	Cogia eluina	Pyrginae	02-SRNP-17121	CSCR079-04	DQ292217
3722	Cogia eluina	Pyrginae	91-SRNP-1362	CSCR077-04	DQ292215
3723	Cogia eluina	Pyrginae	04-SRNP-16107	MHAHF861-06	GU150344
3724	Cogia eluina	Pyrginae	06-SRNP-21562	MHAHK360-07	JF760588
3725	Cogia eluina	Pyrginae	05-SRNP-56836	MHAHF863-06	GU150348
3726	Cogia eluina	Pyrginae	05-SRNP-56837	MHAHF862-06	GU150347
3727	Cogia eluina	Pyrginae	04-SRNP-16191	MHAHF859-06	GU150343
3728	Cogia eluina	Pyrginae	04-SRNP-16194	MHAHF858-06	GU150346
3729	Cogia eluina	Pyrginae	04-SRNP-14135	MHAHE073-05	GU149557
3730	Cogia eluina	Pyrginae	04-SRNP-14051	MHAHE072-05	GU149556
3731	Cogia eluina	Pyrginae	04-SRNP-13940	MHAHE071-05	GU149558
3732	Cogia eluina	Pyrginae	05-SRNP-12004	MHAHL104-07	JF761980
3733	Cogia eluina	Pyrginae	07-SRNP-22100	MHAHL142-07	JF761981
3734	Telemiades avitus	Pyrginae	05-SRNP-55667	MHAHD388-05	GU161886
3735	Telemiades avitus	Pyrginae	07-SRNP-41266	MHAHL261-07	JF763110
3736	Telemiades avitus	Pyrginae	08-SRNP-65922	MHMXX610-09	JF778525
3737	Heliopetes lavianaDHJ01	Pyrginae	07-SRNP-55300	MHAHK730-07	JF760785
3738	Heliopetes lavianaDHJ01	Pyrginae	09-SRNP-57620	MHMYG2382-10	HM885806
3739	Polygonus savignyDHJ01	Pyrginae	01-SRNP-14590	MHAHK610-07	JF761049
3740	Polygonus savignyDHJ01	Pyrginae	06-SRNP-2530	MHAHG740-06	GU151573
3741	Polygonus savignyDHJ01	Pyrginae	02-SRNP-16605	MHAHJ150-07	JF753071
3742	Polygonus savignyDHJ01	Pyrginae	03-SRNP-15205	MHAHJ151-07	JF753072
3743	Polygonus savignyDHJ01	Pyrginae	91-SRNP-954	MHAHK605-07	JF761046
3744	Polygonus savignyDHJ01	Pyrginae	03-SRNP-16118	MHAHJ152-07	JF753073
3745	Polygonus savignyDHJ01	Pyrginae	04-SRNP-46430	MHAHC159-05	DQ293046
3746	Polygonus savignyDHJ01	Pyrginae	04-SRNP-46324	MHAHC167-05	DQ293047
3747	Polygonus savignyDHJ01	Pyrginae	06-SRNP-45458	MHAHI165-06	GU156083
3748	Polygonus savignyDHJ01	Pyrginae	02-SRNP-1520	MHAHJ153-07	JF753074
3749	Polygonus savignyDHJ01	Pyrginae	01-SRNP-14675	MHAHK606-07	JF761047
3750	Polygonus savignyDHJ01	Pyrginae	02-SRNP-17731	MHAHK607-07	JF761048
3751	Polygonus savignyDHJ01	Pyrginae	01-SRNP-14738	MHAHK611-07	JF761050

Tree Order	Species	Subfamily	ACG Sampleid	BOLD Processid	Genbank Accession
3752	Polygonus savignyDHJ02	Pyrginae	06-SRNP-16949	MHAHK055-07	JF761051
3753	Polygonus savignyDHJ02	Pyrginae	06-SRNP-56369	MHAHH454-06	GU155457
3754	Polygonus savignyDHJ02	Pyrginae	06-SRNP-56370	MHAHI164-06	GU156084
3755	Polygonus savignyDHJ02	Pyrginae	01-SRNP-14737	MHAHJ147-07	JF753075
3756	Polygonus savignyDHJ02	Pyrginae	01-SRNP-14672	MHAHJ148-07	JF753076
3757	Polygonus savignyDHJ02	Pyrginae	01-SRNP-14673	MHAHJ149-07	JF753077
3758	Polygonus savignyDHJ02	Pyrginae	01-SRNP-14671	MHAHJ154-07	JF753078
3759	Polygonus savignyDHJ02	Pyrginae	01-SRNP-14670	MHAHK601-07	JF761052
3760	Polygonus savignyDHJ02	Pyrginae	01-SRNP-14669	MHAHK602-07	JF761053
3761	Polygonus savignyDHJ02	Pyrginae	01-SRNP-14668	MHAHK603-07	JF761054
3762	Polygonus savignyDHJ02	Pyrginae	01-SRNP-14674	MHAHK604-07	JF761055
3763	Polygonus savignyDHJ02	Pyrginae	01-SRNP-14676	MHAHK608-07	JF761056
3764	Polygonus savignyDHJ02	Pyrginae	91-SRNP-431	MHAHK609-07	JF761057
3765	Polygonus savignyDHJ02	Pyrginae	08-SRNP-1689	MHMXX1029-09	JF778405
3766	Polygonus savignyDHJ02	Pyrginae	08-SRNP-1690	MHMXX1030-09	JF778406
3767	Polygonus leo	Pyrginae	05-SRNP-1988	MHAHE463-05	GU149848
3768	Polygonus leo	Pyrginae	05-SRNP-45857	MHAHF543-06	GU150706
3769	Polygonus leo	Pyrginae	04-SRNP-46889	MHAHC103-05	DQ293045
3770	Polygonus leo	Pyrginae	05-SRNP-1445	MHAHE465-05	GU149850
3771	Polygonus leo	Pyrginae	05-SRNP-1699	MHAHF544-06	GU150708
3772	Polygonus leo	Pyrginae	05-SRNP-63797	MHAHF681-06	GU150709
3773	Polygonus leo	Pyrginae	05-SRNP-1444	MHAHE464-05	GU149849
3774	Polygonus leo	Pyrginae	05-SRNP-1448	MHAHE466-05	GU149851
3775	Polygonus leo	Pyrginae	05-SRNP-1987	MHAHF545-06	GU150707
3776	Polygonus leo	Pyrginae	08-SRNP-55973	MHMXX1032-09	JF778403
3777	Polygonus leo	Pyrginae	06-SRNP-15696	MHAHH452-06	GU155455
3778	Polygonus leo	Pyrginae	06-SRNP-15549	MHAHH451-06	GU155456
3779	Polygonus leo	Pyrginae	06-SRNP-15550	MHAHH455-06	GU155454
3780	Polygonus leo	Pyrginae	06-SRNP-15695	MHAHK054-07	JF761045
3781	Polygonus leo	Pyrginae	08-SRNP-55955	MHMXX1033-09	JF778404
3782	Polygonus leo	Pyrginae	04-SRNP-46785	MHAHC095-05	DQ293044
3783	Polygonus leo	Pyrginae	09-SRNP-14024	MHMYG2041-10	HM885443
3784	Astraptes INGCUP	Eudaminae	97-SRNP-6203	EPAF127-03	AY666765
3785	Astraptes INGCUP	Eudaminae	00-SRNP-20677	EPAF228-03	AY666910
3786	Astraptes INGCUP	Eudaminae	07-SRNP-4771	MHMXT033-08	JF761556
3787	Astraptes INGCUP	Eudaminae	08-SRNP-1416	MHMXW154-09	JF753689
3788	Astraptes INGCUP	Eudaminae	08-SRNP-1414	MHMXW151-09	JF753688
3789	Astraptes INGCUP	Eudaminae	08-SRNP-82	MHMXW150-09	JF753687
3790	Astraptes INGCUP	Eudaminae	08-SRNP-130	MHMXW148-09	JF753685
3791	Astraptes INGCUP	Eudaminae	95-SRNP-8692	EPAF087-03	AY666844
3792	Astraptes INGCUP	Eudaminae	01-SRNP-5312	EPAF241-03	AY666665
3793	Astraptes INGCUP	Eudaminae	02-SRNP-29904	EPAF287-03	AY666908
3794	Astraptes INGCUP	Eudaminae	01-SRNP-508	EPAF231-03	AY666726
3795	Astraptes INGCUP	Eudaminae	99-SRNP-4982	EPAF182-03	AY666600
3796	Astraptes INGCUP	Eudaminae	96-SRNP-9864	EPAF099-03	AY666834
3797	Astraptes INGCUP	Eudaminae	98-SRNP-6438	EPAF158-03	AY666692
3798	Astraptes INGCUP	Eudaminae	97-SRNP-6595	EPAF136-03	AY666739
3799	Astraptes INGCUP	Eudaminae	99-SRNP-4173	EPAF175-03	AY666626
3800	Astraptes INGCUP	Eudaminae	02-SRNP-24431	EPAF360-03	AY666953
3801	Astraptes INGCUP	Eudaminae	97-SRNP-6205	EPAF130-03	AY666825
3802	Astraptes INGCUP	Eudaminae	02-SRNP-9735	EPAF283-03	AY666615
3803	Astraptes INGCUP	Eudaminae	02-SRNP-20351	EPAF359-03	AY666962
3804	Astraptes INGCUP	Eudaminae	02-SRNP-19906	EPAF364-03	AY667031
3805	Astraptes INGCUP	Eudaminae	02-SRNP-20353	EPAF372-03	AY666796
3806	Astraptes INGCUP	Eudaminae	02-SRNP-9734	EPAF274-03	AY666848
3807	Astraptes INGCUP	Eudaminae	99-SRNP-5123	EPAF191-03	AY667023

Tree Order	Species	Subfamily	ACG Sampleid	BOLD Processid	Genbank Accession
3808	Astraptes INGCUP	Eudaminae	99-SRNP-4985	EPAF189-03	AY667058
3809	Astraptes INGCUP	Eudaminae	97-SRNP-6231	EPAF124-03	AY666774
3810	Astraptes INGCUP	Eudaminae	99-SRNP-5917	EPAF192-03	AY667033
3811	Astraptes INGCUP	Eudaminae	95-SRNP-4448	EPAF071-03	AY666873
3812	Astraptes INGCUP	Eudaminae	97-SRNP-6024	EPAF122-03	AY666773
3813	Astraptes INGCUP	Eudaminae	01-SRNP-1966	EPAF237-03	AY666682
3814	Astraptes INGCUP	Eudaminae	01-SRNP-1389	EPAF235-03	AY666705
3815	Astraptes INGCUP	Eudaminae	01-SRNP-710	EPAF233-03	AY666824
3816	Astraptes INGCUP	Eudaminae	02-SRNP-19350	EPAF299-03	AY667038
3817	Astraptes INGCUP	Eudaminae	02-SRNP-21515	EPAF371-03	AY666797
3818	Astraptes INGCUP	Eudaminae	97-SRNP-7123	EPAF152-03	AY666713
3819	Astraptes INGCUP	Eudaminae	01-SRNP-947	EPAF234-03	AY666933
3820	Astraptes INGCUP	Eudaminae	97-SRNP-7160	EPAF157-03	AY666696
3821	Astraptes INGCUP	Eudaminae	93-SRNP-2396	EPAF029-03	AY666915
3822	Astraptes INGCUP	Eudaminae	00-SRNP-15880	EPAF218-03	AY666941
3823	Astraptes INGCUP	Eudaminae	93-SRNP-2622	EPAF032-03	AY667027
3824	Astraptes INGCUP	Eudaminae	93-SRNP-2796	EPAF035-03	AY666901
3825	Astraptes INGCUP	Eudaminae	99-SRNP-10568	EPAF197-03	AY666916
3826	Astraptes INGCUP	Eudaminae	02-SRNP-32205	EPAF314-03	AY667017
3827	Astraptes INGCUP	Eudaminae	92-SRNP-3026	EPAF018-03	AY666926
3828	Astraptes INGCUP	Eudaminae	91-SRNP-2219	EPAF009-03	AY666936
3829	Astraptes INGCUP	Eudaminae	00-SRNP-2547	EPAF212-03	AY666960
3830	Astraptes INGCUP	Eudaminae	02-SRNP-32206	EPAF310-03	AY667026
3831	Astraptes INGCUP	Eudaminae	02-SRNP-19576	EPAF320-03	AY667018
3832	Astraptes INGCUP	Eudaminae	02-SRNP-20092	EPAF366-03	AY666809
3833	Astraptes INGCUP	Eudaminae	96-SRNP-934	EPAF093-03	AY666842
3834	Astraptes INGCUP	Eudaminae	02-SRNP-19721	EPAF337-03	AY666991
3835	Astraptes INGCUP	Eudaminae	98-SRNP-6516	EPAF168-03	AY666657
3836	Astraptes INGCUP	Eudaminae	02-SRNP-29906	EPAF291-03	AY666814
3837	Astraptes INGCUP	Eudaminae	98-SRNP-6370	EPAF165-03	AY666674
3838	Astraptes INGCUP	Eudaminae	94-SRNP-4843	EPAF056-03	AY666876
3839	Astraptes INGCUP	Eudaminae	02-SRNP-32204	EPAF477-03	AY666625
3840	Astraptes INGCUP	Eudaminae	92-SRNP-3121	EPAF017-03	AY666928
3841	Astraptes INGCUP	Eudaminae	99-SRNP-10560	EPAF195-03	AY667004
3842	Astraptes INGCUP	Eudaminae	94-SRNP-3011	EPAF055-03	AY666890
3843	Astraptes INGCUP	Eudaminae	00-SRNP-15081	EPAF215-03	AY666966
3844	Astraptes INGCUP	Eudaminae	94-SRNP-4958	EPAF054-03	AY666896
3845	Astraptes INGCUP	Eudaminae	96-SRNP-6841.1	EPAF098-03	AY666820
3846	Astraptes INGCUP	Eudaminae	08-SRNP-5592	MHMXY1115-09	GU666430
3847	Astraptes INGCUP	Eudaminae	08-SRNP-1415	MHMXW182-09	JF753684
3848	Astraptes INGCUP	Eudaminae	07-SRNP-42970	MHMXW179-09	JF753683
3849	Astraptes INGCUP	Eudaminae	08-SRNP-21067	MHMXW172-09	JF753682
3850	Astraptes INGCUP	Eudaminae	08-SRNP-65396	MHMXW168-09	JF753681
3851	Astraptes INGCUP	Eudaminae	08-SRNP-70409	MHMXW167-09	JF753680
3852	Astraptes INGCUP	Eudaminae	08-SRNP-2460	MHMXW166-09	JF753679
3853	Astraptes INGCUP	Eudaminae	08-SRNP-1900	MHMXW165-09	JF753678
3854	Astraptes INGCUP	Eudaminae	07-SRNP-61013	MHMXW164-09	JF753677
3855	Astraptes INGCUP	Eudaminae	08-SRNP-1792	MHMXW163-09	JF753676
3856	Astraptes INGCUP	Eudaminae	08-SRNP-40658	MHMXW162-09	JF753675
3857	Astraptes INGCUP	Eudaminae	08-SRNP-81	MHMXW161-09	JF753674
3858	Astraptes INGCUP	Eudaminae	08-SRNP-280	MHMXW160-09	JF753673
3859	Astraptes INGCUP	Eudaminae	08-SRNP-1417	MHMXW158-09	JF753671
3860	Astraptes INGCUP	Eudaminae	07-SRNP-5172	MHMXW157-09	JF753670
3861	Astraptes INGCUP	Eudaminae	08-SRNP-1901	MHMXW156-09	JF753690
3862	Astraptes INGCUP	Eudaminae	07-SRNP-42840	MHMXT039-08	JF761553
3863	Astraptes INGCUP	Eudaminae	07-SRNP-5170	MHMXT016-08	JF761572

Tree Order	Species	Subfamily	ACG Sampleid	BOLD Processid	Genbank Accession
3864	Astraptes INGCUP	Eudaminae	08-SRNP-59	MHMXT014-08	JF761573
3865	Astraptes INGCUP	Eudaminae	07-SRNP-45415	MHMXR730-08	JF761575
3866	Astraptes INGCUP	Eudaminae	07-SRNP-58527	MHMXR727-08	JF761576
3867	Astraptes INGCUP	Eudaminae	07-SRNP-3511	MHMXO870-08	JF761577
3868	Astraptes INGCUP	Eudaminae	07-SRNP-45414	MHMXO856-08	JF761578
3869	Astraptes INGCUP	Eudaminae	07-SRNP-58050	MHMXO848-08	JF761581
3870	Astraptes INGCUP	Eudaminae	07-SRNP-3512	MHMXO838-08	JF761582
3871	Astraptes INGCUP	Eudaminae	07-SRNP-21910	MHAHL294-07	JF761547
3872	Astraptes INGCUP	Eudaminae	07-SRNP-1753	MHAHL287-07	JF761545
3873	Astraptes INGCUP	Eudaminae	07-SRNP-1790	MHAHL283-07	JF761543
3874	Astraptes INGCUP	Eudaminae	07-SRNP-40141	MHMXK342-07	JF761583
3875	Astraptes INGCUP	Eudaminae	07-SRNP-579	MHMXK340-07	JF761584
3876	Astraptes INGCUP	Eudaminae	07-SRNP-1286	MHMXK335-07	JF761585
3877	Astraptes INGCUP	Eudaminae	07-SRNP-1285	MHMXK334-07	JF761586
3878	Astraptes INGCUP	Eudaminae	07-SRNP-380	MHAHK275-07	JF760357
3879	Astraptes INGCUP	Eudaminae	07-SRNP-381	MHAHK274-07	JF760356
3880	Astraptes INGCUP	Eudaminae	06-SRNP-9898	MHAHK250-07	JF760354
3881	Astraptes INGCUP	Eudaminae	06-SRNP-9899	MHAHK249-07	JF760353
3882	Astraptes INGCUP	Eudaminae	06-SRNP-46318	MHAHJ937-07	JF752404
3883	Astraptes INGCUP	Eudaminae	06-SRNP-34320	MHAHJ814-07	JF752403
3884	Astraptes INGCUP	Eudaminae	06-SRNP-8910	MHAHJ803-07	JF752401
3885	Astraptes INGCUP	Eudaminae	06-SRNP-34321	MHAHJ797-07	JF752400
3886	Astraptes INGCUP	Eudaminae	06-SRNP-46151	MHAHJ669-07	JF752399
3887	Astraptes INGCUP	Eudaminae	06-SRNP-35256	MHAHJ623-07	JF752398
3888	Astraptes INGCUP	Eudaminae	06-SRNP-8752	MHAHJ584-07	JF752395
3889	Astraptes INGCUP	Eudaminae	04-SRNP-46483	MHAHH383-06	GU154997
3890	Astraptes INGCUP	Eudaminae	04-SRNP-40688	MHAHH380-06	GU155001
3891	Astraptes INGCUP	Eudaminae	04-SRNP-46477	MHAHH378-06	GU154998
3892	Astraptes INGCUP	Eudaminae	04-SRNP-60805	MHAHH367-06	GU154996
3893	Astraptes INGCUP	Eudaminae	03-SRNP-5634	MHAHH291-06	GU154992
3894	Astraptes INGCUP	Eudaminae	05-SRNP-48590	MHAHG478-06	GU151125
3895	Astraptes INGCUP	Eudaminae	05-SRNP-58120	MHAHG436-06	GU151123
3896	Astraptes INGCUP	Eudaminae	05-SRNP-40683	MHAHG413-06	GU151122
3897	Astraptes INGCUP	Eudaminae	05-SRNP-2221	MHAHG384-06	GU151120
3898	Astraptes INGCUP	Eudaminae	06-SRNP-55334	MHAHG174-06	GU151118
3899	Astraptes INGCUP	Eudaminae	07-SRNP-4895	MHMXT024-08	JF761565
3900	Astraptes INGCUP	Eudaminae	07-SRNP-4323	MHMXT030-08	JF761559
3901	Astraptes INGCUP	Eudaminae	07-SRNP-4552	MHMXT042-08	JF761550
3902	Astraptes INGCUP	Eudaminae	07-SRNP-4225	MHMXT031-08	JF761558
3903	Astraptes INGCUP	Eudaminae	07-SRNP-42991	MHMXT017-08	JF761571
3904	Astraptes INGCUP	Eudaminae	07-SRNP-4229	MHMXT021-08	JF761568
3905	Astraptes INGCUP	Eudaminae	07-SRNP-46516	MHMXT013-08	JF761574
3906	Astraptes INGCUP	Eudaminae	07-SRNP-5013	MHMXT018-08	JF761570
3907	Astraptes INGCUP	Eudaminae	07-SRNP-4884	MHMXT019-08	JF761569
3908	Astraptes INGCUP	Eudaminae	06-SRNP-31876	MHAHH484-06	GU155003
3909	Astraptes INGCUP	Eudaminae	99-SRNP-4980	EPAF190-03	AY667025
3910	Astraptes INGCUP	Eudaminae	97-SRNP-6144	EPAF126-03	AY666767
3911	Astraptes INGCUP	Eudaminae	02-SRNP-20352	EPAF373-03	AY666805
3912	Astraptes INGCUP	Eudaminae	02-SRNP-19284	EPAF309-03	AY667022
3913	Astraptes INGCUP	Eudaminae	07-SRNP-4462	MHMXT027-08	JF761562
3914	Astraptes INGCUP	Eudaminae	07-SRNP-4324	MHMXT037-08	JF761554
3915	Astraptes INGCUP	Eudaminae	07-SRNP-4806	MHMXT040-08	JF761552
3916	Astraptes INGCUP	Eudaminae	07-SRNP-2374	MHAHL290-07	JF761546
3917	Astraptes INGCUP	Eudaminae	07-SRNP-3068	MHMXO852-08	JF761579
3918	Astraptes INGCUP	Eudaminae	04-SRNP-40472	MHAHH381-06	GU155000
3919	Astraptes INGCUP	Eudaminae	06-SRNP-46319	MHAHJ621-07	JF752397

Tree Order	Species	Subfamily	ACG Sampleid	BOLD Processid	Genbank Accession
3920	Astraptes INGCUP	Eudaminae	03-SRNP-5870	MHAHH312-06	GU154993
3921	Astraptes INGCUP	Eudaminae	04-SRNP-47856	MHAHH248-06	GU154989
3922	Astraptes INGCUP	Eudaminae	99-SRNP-13677	EPAF202-03	AY667010
3923	Astraptes INGCUP	Eudaminae	07-SRNP-31660	MHAHL298-07	JF761548
3924	Astraptes INGCUP	Eudaminae	06-SRNP-46276	MHAHJ938-07	JF752405
3925	Astraptes INGCUP	Eudaminae	04-SRNP-47345	MHAHH394-06	GU155002
3926	Astraptes INGCUP	Eudaminae	04-SRNP-2251	MHAHH250-06	GU154988
3927	Astraptes INGCUP	Eudaminae	05-SRNP-1361	MHAHG443-06	GU151124
3928	Astraptes INGCUP	Eudaminae	07-SRNP-58736	MHMXO849-08	JF761580
3929	Astraptes INGCUP	Eudaminae	04-SRNP-47196	MHAHH253-06	GU154990
3930	Astraptes INGCUP	Eudaminae	06-SRNP-55405	MHAHG178-06	GU151119
3931	Astraptes INGCUP	Eudaminae	07-SRNP-1054	MHAHL272-07	JF761541
3932	Astraptes INGCUP	Eudaminae	07-SRNP-1595	MHAHL278-07	JF761542
3933	Astraptes INGCUP	Eudaminae	04-SRNP-61428	MHAHH276-06	GU154991
3934	Astraptes INGCUP	Eudaminae	05-SRNP-19576	MHAHG481-06	GU151126
3935	Astraptes INGCUP	Eudaminae	05-SRNP-58121	MHAHG440-06	GU151121
3936	Astraptes INGCUP	Eudaminae	07-SRNP-4606	MHMXT025-08	JF761564
3937	Astraptes INGCUP	Eudaminae	97-SRNP-5640	EPAF151-03	AY666699
3938	Astraptes INGCUP	Eudaminae	07-SRNP-4488	MHMXT022-08	JF761567
3939	Astraptes INGCUP	Eudaminae	07-SRNP-4489	MHMXT023-08	JF761566
3940	Astraptes INGCUP	Eudaminae	07-SRNP-4608	MHMXT026-08	JF761563
3941	Astraptes INGCUP	Eudaminae	04-SRNP-47346	MHAHH261-06	GU154987
3942	Astraptes INGCUP	Eudaminae	08-SRNP-1418	MHMXW159-09	JF753672
3943	Astraptes INGCUP	Eudaminae	04-SRNP-40582	MHAHH382-06	GU154999
3944	Astraptes INGCUP	Eudaminae	07-SRNP-4277	MHMXT041-08	JF761551
3945	Astraptes INGCUP	Eudaminae	04-SRNP-35086	CSRII426-04	DQ291880
3946	Astraptes INGCUP	Eudaminae	07-SRNP-4833	MHMXT032-08	JF761557
3947	Astraptes INGCUP	Eudaminae	03-SRNP-15979	MHAHH321-06	GU154995
3948	Astraptes HIHAMP	Eudaminae	04-SRNP-35067	CSRII172-04	AY724412
3949	Astraptes HIHAMP	Eudaminae	04-SRNP-35081	CSRII425-04	AY724411
3950	Astraptes HIHAMP	Eudaminae	06-SRNP-36085	MHAHK276-07	JF760350
3951	Astraptes HIHAMP	Eudaminae	07-SRNP-35469	MHMXK352-07	JF761527
3952	Astraptes HIHAMP	Eudaminae	06-SRNP-36608	MHAHK255-07	JF760348
3953	Astraptes HIHAMP	Eudaminae	99-SRNP-1215	EPAF199-03	AY667056
3954	Astraptes HIHAMP	Eudaminae	04-SRNP-56052	MHAHH283-06	GU154963
3955	Astraptes HIHAMP	Eudaminae	03-SRNP-22543	MHAHH302-06	GU154964
3956	Astraptes HIHAMP	Eudaminae	07-SRNP-45714	MHMXO860-08	JF761523
3957	Astraptes HIHAMP	Eudaminae	05-SRNP-35903	MHAHG431-06	GU151115
3958	Astraptes HIHAMP	Eudaminae	07-SRNP-45437	MHMXR734-08	JF761521
3959	Astraptes HIHAMP	Eudaminae	07-SRNP-36293	MHMXO836-08	JF761524
3960	Astraptes HIHAMP	Eudaminae	07-SRNP-35467	MHMXK355-07	JF761525
3961	Astraptes HIHAMP	Eudaminae	07-SRNP-35468	MHMXK354-07	JF761526
3962	Astraptes HIHAMP	Eudaminae	06-SRNP-36705	MHMXK349-07	JF761528
3963	Astraptes HIHAMP	Eudaminae	06-SRNP-36706	MHMXK331-07	JF761529
3964	Astraptes HIHAMP	Eudaminae	06-SRNP-35831	MHAHK281-07	JF760351
3965	Astraptes HIHAMP	Eudaminae	06-SRNP-36787	MHAHK260-07	JF760349
3966	Astraptes HIHAMP	Eudaminae	06-SRNP-36083	MHAHJ926-07	JF752392
3967	Astraptes HIHAMP	Eudaminae	06-SRNP-35716	MHAHI014-06	GU155807
3968	Astraptes HIHAMP	Eudaminae	03-SRNP-3079	MHAHH322-06	GU154965
3969	Astraptes HIHAMP	Eudaminae	05-SRNP-35763	MHAHG438-06	GU151117
3970	Astraptes HIHAMP	Eudaminae	05-SRNP-35765	MHAHG437-06	GU151116
3971	Astraptes HIHAMP	Eudaminae	06-SRNP-35832	MHAHJ935-07	JF752393
3972	Astraptes HIHAMP	Eudaminae	97-SRNP-1804	EPAF135-03	AY666757
3973	Astraptes HIHAMP	Eudaminae	01-SRNP-7374	EPAF244-03	AY666655
3974	Astraptes HIHAMP	Eudaminae	02-SRNP-23035	EPAF269-03	AY666947
3975	Astraptes HIHAMP	Eudaminae	99-SRNP-1220	EPAF200-03	AY666999

Tree Order	Species	Subfamily	ACG Sampleid	BOLD Processid	Genbank Accession
3976	Astraptes HIHAMP	Eudaminae	00-SRNP-22183	EPAF226-03	AY666868
3977	Astraptes HIHAMP	Eudaminae	01-SRNP-6199	EPAF229-03	AY666938
3978	Astraptes HIHAMP	Eudaminae	99-SRNP-1098	EPAF196-03	AY667013
3979	Astraptes HIHAMP	Eudaminae	95-SRNP-666	EPAF065-03	AY666903
3980	Astraptes HIHAMP	Eudaminae	00-SRNP-10424	EPAF221-03	AY666950
3981	Astraptes HIHAMP	Eudaminae	97-SRNP-1588	EPAF485-03	AY666607
3982	Astraptes HIHAMP	Eudaminae	97-SRNP-1613	EPAF134-03	AY666748
3983	Astraptes INGCUP	Eudaminae	05-SRNP-34301	MHAHI674-06	GU155808
3984	Astraptes HIHAMP	Eudaminae	01-SRNP-7327	EPAF242-03	AY666663
3985	Astraptes HIHAMP	Eudaminae	97-SRNP-1641	EPAF137-03	AY666742
3986	Astraptes HIHAMP	Eudaminae	07-SRNP-45715	MHMXR722-08	JF761522
3987	Astraptes HIHAMP	Eudaminae	07-SRNP-36295	MHMXT020-08	JF761520
3988	Astraptes HIHAMP	Eudaminae	08-SRNP-35798	MHMXX474-09	JF777625
3989	Astraptes HIHAMP	Eudaminae	08-SRNP-6196	MHMXY1114-09	GU666429
3990	Astraptes fulgerator group	Eudaminae	09-SRNP-72096	MHMYE1576-09	HM391136
3991	Astraptes INGCUP	Eudaminae	06-SRNP-23021	MHAHJ807-07	JF752402
3992	Astraptes INGCUP	Eudaminae	07-SRNP-4664	MHMXT029-08	JF761560
3993	Astraptes INGCUP	Eudaminae	07-SRNP-55016	MHAHK254-07	JF760355
3994	Astraptes MYST	Eudaminae	90-SRNP-1632	EPAF008-03	AY666937
3995	Astraptes MYST	Eudaminae	02-SRNP-33369	EPAF344-03	AY666975
3996	Astraptes MYST	Eudaminae	02-SRNP-33451	EPAF363-03	AY667054
3997	Astraptes MYST	Eudaminae	03-SRNP-21723	MHAHH180-06	GU155087
3998	Astraptes MYST	Eudaminae	03-SRNP-20884	MHAHH341-06	GU155088
3999	Astraptes MYST	Eudaminae	03-SRNP-31167	MHAHH350-06	GU155089
4000	Astraptes MYST	Eudaminae	06-SRNP-41913	MHAHI012-06	GU155813
4001	Astraptes YESENN	Eudaminae	07-SRNP-45998	MHMXT012-08	JF761655
4002	Astraptes INGCUP	Eudaminae	03-SRNP-10415	MHAHH318-06	GU154994
4003	Astraptes INGCUP	Eudaminae	06-SRNP-3381	MHAHH487-06	GU155004
4004	Astraptes INGCUP	Eudaminae	07-SRNP-4663	MHMXT028-08	JF761561
4005	Astraptes INGCUP	Eudaminae	02-SRNP-33256	EPAF318-03	AY667007
4006	Astraptes INGCUP	Eudaminae	07-SRNP-1756	MHAHL286-07	JF761544
4007	Astraptes INGCUP	Eudaminae	07-SRNP-4469	MHMXT043-08	JF761549
4008	Astraptes INGCUP	Eudaminae	07-SRNP-33557	MHMXT036-08	JF761555
4009	Astraptes FABOV	Eudaminae	94-SRNP-678	EPAF052-03	AY666886
4010	Astraptes FABOV	Eudaminae	06-SRNP-47519	MHAHK237-07	JF760347
4011	Astraptes FABOV	Eudaminae	98-SRNP-79	EPAF171-03	AY666627
4012	Astraptes FABOV	Eudaminae	03-SRNP-38625	MHAHH190-06	GU154949
4013	Astraptes FABOV	Eudaminae	05-SRNP-34585	MHAHG176-06	GU151105
4014	Astraptes FABOV	Eudaminae	03-SRNP-38030	MHAHH218-06	GU154947
4015	Astraptes FABOV	Eudaminae	04-SRNP-47477	MHAHH225-06	GU154951
4016	Astraptes FABOV	Eudaminae	03-SRNP-18628	MHAHH204-06	GU154950
4017	Astraptes FABOV	Eudaminae	03-SRNP-21672	MHAHH217-06	GU154948
4018	Astraptes FABOV	Eudaminae	03-SRNP-1100	MHAHH323-06	GU154957
4019	Astraptes FABOV	Eudaminae	04-SRNP-47263	MHAHH364-06	GU154959
4020	Astraptes FABOV	Eudaminae	05-SRNP-20134	MHAHH274-06	GU154956
4021	Astraptes FABOV	Eudaminae	04-SRNP-27294	MHAHH278-06	GU154953
4022	Astraptes FABOV	Eudaminae	04-SRNP-47478	MHAHH226-06	GU154952
4023	Astraptes FABOV	Eudaminae	04-SRNP-15696	MHAHH267-06	GU154954
4024	Astraptes FABOV	Eudaminae	07-SRNP-40452	MHMXK350-07	JF761518
4025	Astraptes FABOV	Eudaminae	05-SRNP-30622	MHAHG396-06	GU151108
4026	Astraptes FABOV	Eudaminae	08-SRNP-45170	MHMXW178-09	JF753667
4027	Astraptes FABOV	Eudaminae	08-SRNP-6000	MHMXY1110-09	GU666433
4028	Astraptes FABOV	Eudaminae	07-SRNP-21410	MHAHL301-07	JF761515
4029	Astraptes FABOV	Eudaminae	07-SRNP-58607	MHMXR726-08	JF761516
4030	Astraptes FABOV	Eudaminae	07-SRNP-40453	MHMXK338-07	JF761519
4031	Astraptes FABOV	Eudaminae	07-SRNP-22021	MHAHL293-07	JF761514

Tree Order	Species	Subfamily	ACG Sampleid	BOLD Processid	Genbank Accession
4032	Astraptes FABOV	Eudaminae	04-SRNP-22024	MHAHH395-06	GU154960
4033	Astraptes FABOV	Eudaminae	05-SRNP-25512	MHAHH482-06	GU154962
4034	Astraptes FABOV	Eudaminae	03-SRNP-15973	MHAHH327-06	GU154958
4035	Astraptes FABOV	Eudaminae	03-SRNP-19356	MHAHH159-06	GU154961
4036	Astraptes FABOV	Eudaminae	04-SRNP-50141	MHAHG445-06	GU151113
4037	Astraptes FABOV	Eudaminae	04-SRNP-56195	MHAHG422-06	GU151112
4038	Astraptes FABOV	Eudaminae	04-SRNP-26414	MHAHG406-06	GU151109
4039	Astraptes FABOV	Eudaminae	94-SRNP-5469	EPAF057-03	AY666875
4040	Astraptes FABOV	Eudaminae	80-SRNP-216	EPAF001-03	AY666939
4041	Astraptes FABOV	Eudaminae	97-SRNP-5023	EPAF139-03	AY666746
4042	Astraptes FABOV	Eudaminae	97-SRNP-5143	EPAF138-03	AY666740
4043	Astraptes FABOV	Eudaminae	02-SRNP-13079	EPAF282-03	AY666831
4044	Astraptes FABOV	Eudaminae	95-SRNP-6867	EPAF404-03	AY666745
4045	Astraptes FABOV	Eudaminae	92-SRNP-6023	EPAF026-03	AY666922
4046	Astraptes FABOV	Eudaminae	93-SRNP-6312	EPAF046-03	AY666904
4047	Astraptes FABOV	Eudaminae	95-SRNP-6871	EPAF080-03	AY666866
4048	Astraptes FABOV	Eudaminae	97-SRNP-5060	EPAF141-03	AY666731
4049	Astraptes FABOV	Eudaminae	93-SRNP-7060	EPAF048-03	AY666898
4050	Astraptes FABOV	Eudaminae	02-SRNP-31579	EPAF327-03	AY667002
4051	Astraptes FABOV	Eudaminae	95-SRNP-9320	EPAF480-03	AY666624
4052	Astraptes FABOV	Eudaminae	02-SRNP-31569	EPAF326-03	AY666987
4053	Astraptes FABOV	Eudaminae	02-SRNP-13082	EPAF286-03	AY666867
4054	Astraptes FABOV	Eudaminae	96-SRNP-171	EPAF090-03	AY666837
4055	Astraptes FABOV	Eudaminae	95-SRNP-6103	EPAF076-03	AY666863
4056	Astraptes FABOV	Eudaminae	95-SRNP-511	EPAF064-03	AY666895
4057	Astraptes FABOV	Eudaminae	99-SRNP-8551	EPAF193-03	AY667014
4058	Astraptes FABOV	Eudaminae	02-SRNP-28909	EPAF292-03	AY666823
4059	Astraptes FABOV	Eudaminae	97-SRNP-5809	EPAF154-03	AY666737
4060	Astraptes FABOV	Eudaminae	97-SRNP-5926	EPAF153-03	AY666722
4061	Astraptes FABOV	Eudaminae	92-SRNP-4645	EPAF023-03	AY666921
4062	Astraptes FABOV	Eudaminae	95-SRNP-6866	EPAF079-03	AY666852
4063	Astraptes FABOV	Eudaminae	94-SRNP-745	EPAF053-03	AY666883
4064	Astraptes FABOV	Eudaminae	04-SRNP-15697	MHAHH271-06	GU154955
4065	Astraptes FABOV	Eudaminae	05-SRNP-45420	MHAHG390-06	GU151107
4066	Astraptes FABOV	Eudaminae	80-SRNP-160	EPAF384-03	AY666775
4067	Astraptes FABOV	Eudaminae	97-SRNP-5927	EPAF156-03	AY666813
4068	Astraptes FABOV	Eudaminae	04-SRNP-27295	MHAHG416-06	GU151110
4069	Astraptes FABOV	Eudaminae	07-SRNP-56300	MHMXK353-07	JF761517
4070	Astraptes FABOV	Eudaminae	05-SRNP-20856	MHAHG378-06	GU151106
4071	Astraptes FABOV	Eudaminae	95-SRNP-8306	EPAF085-03	AY666881
4072	Astraptes FABOV	Eudaminae	97-SRNP-5969	EPAF435-03	AY666694
4073	Astraptes FABOV	Eudaminae	05-SRNP-21651	MHAHG420-06	GU151111
4074	Astraptes FABOV	Eudaminae	05-SRNP-60050	MHAHG480-06	GU151114
4075	Astraptes FABOV	Eudaminae	08-SRNP-58528	MHMYC537-09	GU649805
4076	Astraptes enta	Eudaminae	03-SRNP-11423	MHAHH214-06	GU154946
4077	Astraptes enta	Eudaminae	07-SRNP-42567	MHMXR731-08	JF761513
4078	Astraptes enta	Eudaminae	09-SRNP-75531	MHMYG2383-10	HM885807
4079	Astraptes BYTTNER	Eudaminae	95-SRNP-8045	EPAF082-03	AY666854
4080	Astraptes BYTTNER	Eudaminae	95-SRNP-8046	EPAF083-03	AY666853
4081	Astraptes BYTTNER	Eudaminae	95-SRNP-8044	EPAF084-03	AY666872
4082	Astraptes LOHAMP	Eudaminae	99-SRNP-17057	EPAF207-03	AY666997
4083	Astraptes LOHAMP	Eudaminae	06-SRNP-36233	MHAHK242-07	JF760365
4084	Astraptes LOHAMP	Eudaminae	06-SRNP-36395	MHAHK238-07	JF760361
4085	Astraptes LOHAMP	Eudaminae	06-SRNP-31669	MHAHH486-06	GU155081
4086	Astraptes LOHAMP	Eudaminae	06-SRNP-36238	MHAHK243-07	JF760366
4087	Astraptes LOHAMP	Eudaminae	03-SRNP-4251	MHAHH304-06	GU155046

Tree Order	Species	Subfamily	ACG Sampleid	BOLD Processid	Genbank Accession
4088	Astraptes LOHAMP	Eudaminae	06-SRNP-36296	MHAHK240-07	JF760363
4089	Astraptes LOHAMP	Eudaminae	07-SRNP-2042	MHAHL281-07	JF761592
4090	Astraptes LOHAMP	Eudaminae	04-SRNP-35879	MHAHH282-06	GU155036
4091	Astraptes LOHAMP	Eudaminae	06-SRNP-36344	MHAHK239-07	JF760362
4092	Astraptes LOHAMP	Eudaminae	06-SRNP-36346	MHAHK241-07	JF760364
4093	Astraptes LOHAMP	Eudaminae	06-SRNP-36338	MHAHK268-07	JF760376
4094	Astraptes LOHAMP	Eudaminae	06-SRNP-7043	MHAHJ581-07	JF752413
4095	Astraptes LOHAMP	Eudaminae	06-SRNP-7363	MHAHJ578-07	JF752411
4096	Astraptes LOHAMP	Eudaminae	06-SRNP-8179	MHAHJ589-07	JF752416
4097	Astraptes LOHAMP	Eudaminae	04-SRNP-35147	MHAHH388-06	GU155073
4098	Astraptes LOHAMP	Eudaminae	03-SRNP-3139	MHAHH324-06	GU155055
4099	Astraptes LOHAMP	Eudaminae	04-SRNP-61342	MHAHH275-06	GU155029
4100	Astraptes LOHAMP	Eudaminae	04-SRNP-56771	MHAHH270-06	GU155030
4101	Astraptes LOHAMP	Eudaminae	03-SRNP-15889	MHAHH188-06	GU155011
4102	Astraptes LOHAMP	Eudaminae	06-SRNP-36052	MHMXH849-07	JF760384
4103	Astraptes LOHAMP	Eudaminae	06-SRNP-6884	MHAHJ580-07	JF752412
4104	Astraptes LOHAMP	Eudaminae	08-SRNP-35977	MHMXX478-09	JF777634
4105	Astraptes LOHAMP	Eudaminae	08-SRNP-35969	MHMXX476-09	JF777633
4106	Astraptes LOHAMP	Eudaminae	08-SRNP-35978	MHMXX475-09	JF777632
4107	Astraptes LOHAMP	Eudaminae	08-SRNP-72414	MHMXX472-09	JF777631
4108	Astraptes LOHAMP	Eudaminae	08-SRNP-35033	MHMXW181-09	JF753694
4109	Astraptes LOHAMP	Eudaminae	08-SRNP-21411	MHMXW171-09	JF753692
4110	Astraptes LOHAMP	Eudaminae	08-SRNP-35306	MHMXW170-09	JF753691
4111	Astraptes LOHAMP	Eudaminae	07-SRNP-36287	MHMXR719-08	JF761600
4112	Astraptes LOHAMP	Eudaminae	07-SRNP-3020	MHMXO864-08	JF761602
4113	Astraptes LOHAMP	Eudaminae	07-SRNP-42071	MHMXO847-08	JF761604
4114	Astraptes LOHAMP	Eudaminae	07-SRNP-36173	MHMXO844-08	JF761605
4115	Astraptes LOHAMP	Eudaminae	07-SRNP-35819	MHMXO843-08	JF761606
4116	Astraptes LOHAMP	Eudaminae	07-SRNP-35848	MHMXO840-08	JF761607
4117	Astraptes LOHAMP	Eudaminae	07-SRNP-42123	MHMXO839-08	JF761608
4118	Astraptes LOHAMP	Eudaminae	07-SRNP-35826	MHAHL296-07	JF761597
4119	Astraptes LOHAMP	Eudaminae	07-SRNP-35754	MHAHL295-07	JF761596
4120	Astraptes LOHAMP	Eudaminae	07-SRNP-35755	MHAHL291-07	JF761595
4121	Astraptes LOHAMP	Eudaminae	07-SRNP-35709	MHAHL289-07	JF761594
4122	Astraptes LOHAMP	Eudaminae	07-SRNP-697	MHAHL273-07	JF761591
4123	Astraptes LOHAMP	Eudaminae	07-SRNP-1983	MHAHL270-07	JF761589
4124	Astraptes LOHAMP	Eudaminae	07-SRNP-35414	MHMXK356-07	JF761609
4125	Astraptes LOHAMP	Eudaminae	06-SRNP-65614	MHMXK347-07	JF761611
4126	Astraptes LOHAMP	Eudaminae	07-SRNP-35525	MHMXK345-07	JF761612
4127	Astraptes LOHAMP	Eudaminae	07-SRNP-1211	MHMXK341-07	JF761614
4128	Astraptes LOHAMP	Eudaminae	07-SRNP-1467	MHMXK337-07	JF761615
4129	Astraptes LOHAMP	Eudaminae	06-SRNP-36758	MHMXH850-07	JF760383
4130	Astraptes LOHAMP	Eudaminae	06-SRNP-36094	MHMXH848-07	JF760385
4131	Astraptes LOHAMP	Eudaminae	06-SRNP-36941	MHAHK280-07	JF760382
4132	Astraptes LOHAMP	Eudaminae	06-SRNP-36618	MHAHK279-07	JF760381
4133	Astraptes LOHAMP	Eudaminae	06-SRNP-36087	MHAHK278-07	JF760380
4134	Astraptes LOHAMP	Eudaminae	06-SRNP-36707	MHAHK273-07	JF760379
4135	Astraptes LOHAMP	Eudaminae	06-SRNP-47853	MHAHK272-07	JF760378
4136	Astraptes LOHAMP	Eudaminae	06-SRNP-36541	MHAHK267-07	JF760375
4137	Astraptes LOHAMP	Eudaminae	06-SRNP-36616	MHAHK266-07	JF760374
4138	Astraptes LOHAMP	Eudaminae	06-SRNP-36544	MHAHK262-07	JF760370
4139	Astraptes LOHAMP	Eudaminae	06-SRNP-36237	MHAHK261-07	JF760369
4140	Astraptes LOHAMP	Eudaminae	07-SRNP-35045	MHAHK236-07	JF760360
4141	Astraptes LOHAMP	Eudaminae	07-SRNP-35040	MHAHK235-07	JF760359
4142	Astraptes LOHAMP	Eudaminae	06-SRNP-35627	MHAHJ934-07	JF752433
4143	Astraptes LOHAMP	Eudaminae	06-SRNP-35607	MHAHJ931-07	JF752431

Tree Order	Species	Subfamily	ACG Sampleid	BOLD Processid	Genbank Accession
4144	Astraptes LOHAMP	Eudaminae	06-SRNP-35688	MHAHJ930-07	JF752430
4145	Astraptes LOHAMP	Eudaminae	06-SRNP-35628	MHAHJ929-07	JF752429
4146	Astraptes LOHAMP	Eudaminae	06-SRNP-35619	MHAHJ928-07	JF752428
4147	Astraptes LOHAMP	Eudaminae	06-SRNP-35710	MHAHJ924-07	JF752427
4148	Astraptes LOHAMP	Eudaminae	06-SRNP-9530	MHAHJ813-07	JF752426
4149	Astraptes LOHAMP	Eudaminae	06-SRNP-6234	MHAHJ810-07	JF752425
4150	Astraptes LOHAMP	Eudaminae	06-SRNP-21646	MHAHJ809-07	JF752424
4151	Astraptes LOHAMP	Eudaminae	06-SRNP-9420	MHAHJ806-07	JF752423
4152	Astraptes LOHAMP	Eudaminae	06-SRNP-23242	MHAHJ798-07	JF752422
4153	Astraptes LOHAMP	Eudaminae	06-SRNP-6037	MHAHJ732-07	JF752420
4154	Astraptes LOHAMP	Eudaminae	06-SRNP-6038	MHAHJ730-07	JF752419
4155	Astraptes LOHAMP	Eudaminae	06-SRNP-47681	MHAHJ671-07	JF752418
4156	Astraptes LOHAMP	Eudaminae	06-SRNP-7042	MHAHJ664-07	JF752417
4157	Astraptes LOHAMP	Eudaminae	06-SRNP-5587	MHAHJ588-07	JF752415
4158	Astraptes LOHAMP	Eudaminae	06-SRNP-6933	MHAHJ582-07	JF752414
4159	Astraptes LOHAMP	Eudaminae	06-SRNP-8178	MHAHJ577-07	JF752410
4160	Astraptes LOHAMP	Eudaminae	06-SRNP-9171	MHAHJ575-07	JF752409
4161	Astraptes LOHAMP	Eudaminae	06-SRNP-8076	MHAHJ573-07	JF752408
4162	Astraptes LOHAMP	Eudaminae	06-SRNP-35562	MHAHI015-06	GU155809
4163	Astraptes LOHAMP	Eudaminae	06-SRNP-4848	MHAHI010-06	GU155810
4164	Astraptes LOHAMP	Eudaminae	04-SRNP-35126	MHAHH392-06	GU155075
4165	Astraptes LOHAMP	Eudaminae	04-SRNP-35127	MHAHH389-06	GU155071
4166	Astraptes LOHAMP	Eudaminae	04-SRNP-35162	MHAHH386-06	GU155070
4167	Astraptes LOHAMP	Eudaminae	04-SRNP-35149	MHAHH385-06	GU155005
4168	Astraptes LOHAMP	Eudaminae	04-SRNP-46671	MHAHH373-06	GU155067
4169	Astraptes LOHAMP	Eudaminae	04-SRNP-50042	MHAHH370-06	GU155039
4170	Astraptes LOHAMP	Eudaminae	04-SRNP-49891	MHAHH369-06	GU155063
4171	Astraptes LOHAMP	Eudaminae	04-SRNP-60810	MHAHH365-06	GU155066
4172	Astraptes LOHAMP	Eudaminae	04-SRNP-55659	MHAHH362-06	GU155065
4173	Astraptes LOHAMP	Eudaminae	04-SRNP-56335	MHAHH361-06	GU155064
4174	Astraptes LOHAMP	Eudaminae	04-SRNP-61002	MHAHH360-06	GU155041
4175	Astraptes LOHAMP	Eudaminae	04-SRNP-60550	MHAHH359-06	GU155059
4176	Astraptes LOHAMP	Eudaminae	04-SRNP-61012	MHAHH358-06	GU155061
4177	Astraptes LOHAMP	Eudaminae	04-SRNP-60933	MHAHH357-06	GU155058
4178	Astraptes LOHAMP	Eudaminae	04-SRNP-36186	MHAHH356-06	GU155062
4179	Astraptes LOHAMP	Eudaminae	04-SRNP-36170	MHAHH355-06	GU155060
4180	Astraptes LOHAMP	Eudaminae	03-SRNP-22299	MHAHH354-06	GU155052
4181	Astraptes LOHAMP	Eudaminae	03-SRNP-3138	MHAHH326-06	GU155056
4182	Astraptes LOHAMP	Eudaminae	03-SRNP-3509	MHAHH319-06	GU155053
4183	Astraptes LOHAMP	Eudaminae	03-SRNP-3100	MHAHH317-06	GU155042
4184	Astraptes LOHAMP	Eudaminae	03-SRNP-4933	MHAHH316-06	GU155044
4185	Astraptes LOHAMP	Eudaminae	03-SRNP-4776	MHAHH315-06	GU155047
4186	Astraptes LOHAMP	Eudaminae	03-SRNP-4281	MHAHH307-06	GU155048
4187	Astraptes LOHAMP	Eudaminae	03-SRNP-4252	MHAHH306-06	GU155049
4188	Astraptes LOHAMP	Eudaminae	03-SRNP-4410	MHAHH305-06	GU155045
4189	Astraptes LOHAMP	Eudaminae	03-SRNP-6959	MHAHH299-06	GU155040
4190	Astraptes LOHAMP	Eudaminae	03-SRNP-7550	MHAHH298-06	GU155043
4191	Astraptes LOHAMP	Eudaminae	04-SRNP-40265	MHAHH182-06	GU155007
4192	Astraptes LOHAMP	Eudaminae	03-SRNP-4699	MHAHH173-06	GU155008
4193	Astraptes LOHAMP	Eudaminae	03-SRNP-3193	MHAHH166-06	GU155006
4194	Astraptes LOHAMP	Eudaminae	03-SRNP-3258	MHAHH165-06	GU155072
4195	Astraptes LOHAMP	Eudaminae	03-SRNP-5544	MHAHH164-06	GU155076
4196	Astraptes LOHAMP	Eudaminae	03-SRNP-7135	MHAHH163-06	GU155078
4197	Astraptes LOHAMP	Eudaminae	03-SRNP-3112	MHAHH162-06	GU155077
4198	Astraptes LOHAMP	Eudaminae	03-SRNP-3343	MHAHH161-06	GU155079
4199	Astraptes LOHAMP	Eudaminae	03-SRNP-10879	MHAHH160-06	GU155080

Tree Order	Species	Subfamily	ACG Sampleid	BOLD Processid	Genbank Accession
4200	Astraptes LOHAMP	Eudaminae	03-SRNP-6958	MHAHH156-06	GU155069
4201	Astraptes LOHAMP	Eudaminae	06-SRNP-35048	MHAHG454-06	GU151144
4202	Astraptes LOHAMP	Eudaminae	06-SRNP-35127	MHAHG449-06	GU151143
4203	Astraptes LOHAMP	Eudaminae	04-SRNP-36231	MHAHG426-06	GU151141
4204	Astraptes LOHAMP	Eudaminae	05-SRNP-41118	MHAHG414-06	GU151140
4205	Astraptes LOHAMP	Eudaminae	04-SRNP-35980	MHAHG403-06	GU151138
4206	Astraptes LOHAMP	Eudaminae	05-SRNP-350	MHAHG397-06	GU151135
4207	Astraptes LOHAMP	Eudaminae	05-SRNP-35593	MHAHG392-06	GU151128
4208	Astraptes LOHAMP	Eudaminae	04-SRNP-56334	MHAHG382-06	GU151130
4209	Astraptes LOHAMP	Eudaminae	05-SRNP-231	MHAHG379-06	GU151131
4210	Astraptes LOHAMP	Eudaminae	07-SRNP-35931	MHAHL300-07	JF761598
4211	Astraptes LOHAMP	Eudaminae	07-SRNP-40830	MHAHL285-07	JF761593
4212	Astraptes LOHAMP	Eudaminae	06-SRNP-36607	MHAHK263-07	JF760371
4213	Astraptes LOHAMP	Eudaminae	07-SRNP-35002	MHAHK259-07	JF760368
4214	Astraptes LOHAMP	Eudaminae	06-SRNP-42492	MHAHI564-06	GU155811
4215	Astraptes LOHAMP	Eudaminae	06-SRNP-42491	MHAHI563-06	GU155812
4216	Astraptes LOHAMP	Eudaminae	04-SRNP-35218	MHAHH393-06	GU155074
4217	Astraptes LOHAMP	Eudaminae	04-SRNP-35559	MHAHH374-06	GU155068
4218	Astraptes LOHAMP	Eudaminae	03-SRNP-23253	MHAHH338-06	GU155057
4219	Astraptes LOHAMP	Eudaminae	03-SRNP-3354	MHAHH320-06	GU155054
4220	Astraptes LOHAMP	Eudaminae	03-SRNP-4998	MHAHH313-06	GU155050
4221	Astraptes LOHAMP	Eudaminae	03-SRNP-4369	MHAHH311-06	GU155051
4222	Astraptes LOHAMP	Eudaminae	03-SRNP-1331	MHAHH290-06	GU155038
4223	Astraptes LOHAMP	Eudaminae	04-SRNP-35877	MHAHH284-06	GU155033
4224	Astraptes LOHAMP	Eudaminae	04-SRNP-35875	MHAHH281-06	GU155037
4225	Astraptes LOHAMP	Eudaminae	04-SRNP-35694	MHAHH280-06	GU155034
4226	Astraptes LOHAMP	Eudaminae	04-SRNP-3509	MHAHH273-06	GU155031
4227	Astraptes LOHAMP	Eudaminae	04-SRNP-35873	MHAHH272-06	GU155032
4228	Astraptes LOHAMP	Eudaminae	04-SRNP-3852	MHAHH268-06	GU155028
4229	Astraptes LOHAMP	Eudaminae	04-SRNP-35321	MHAHH265-06	GU155026
4230	Astraptes LOHAMP	Eudaminae	04-SRNP-35170	MHAHH264-06	GU155027
4231	Astraptes LOHAMP	Eudaminae	04-SRNP-46672	MHAHH262-06	GU155017
4232	Astraptes LOHAMP	Eudaminae	04-SRNP-35683	MHAHH260-06	GU155024
4233	Astraptes LOHAMP	Eudaminae	04-SRNP-35449	MHAHH254-06	GU155025
4234	Astraptes LOHAMP	Eudaminae	04-SRNP-35500	MHAHH251-06	GU155022
4235	Astraptes LOHAMP	Eudaminae	04-SRNP-35346	MHAHH249-06	GU155023
4236	Astraptes LOHAMP	Eudaminae	04-SRNP-35269	MHAHH244-06	GU155018
4237	Astraptes LOHAMP	Eudaminae	04-SRNP-35395	MHAHH242-06	GU155019
4238	Astraptes LOHAMP	Eudaminae	04-SRNP-35343	MHAHH240-06	GU155020
4239	Astraptes LOHAMP	Eudaminae	04-SRNP-35436	MHAHH237-06	GU155021
4240	Astraptes LOHAMP	Eudaminae	03-SRNP-23619	MHAHH216-06	GU155009
4241	Astraptes LOHAMP	Eudaminae	03-SRNP-4294	MHAHH215-06	GU155013
4242	Astraptes LOHAMP	Eudaminae	03-SRNP-6225	MHAHH212-06	GU155016
4243	Astraptes LOHAMP	Eudaminae	03-SRNP-13031.1	MHAHH201-06	GU155015
4244	Astraptes LOHAMP	Eudaminae	03-SRNP-31151	MHAHH200-06	GU155014
4245	Astraptes LOHAMP	Eudaminae	03-SRNP-23573	MHAHH195-06	GU155010
4246	Astraptes LOHAMP	Eudaminae	03-SRNP-22087	MHAHH191-06	GU155012
4247	Astraptes LOHAMP	Eudaminae	06-SRNP-35022	MHAHG456-06	GU151145
4248	Astraptes LOHAMP	Eudaminae	04-SRNP-36163	MHAHG435-06	GU151142
4249	Astraptes LOHAMP	Eudaminae	05-SRNP-1602	MHAHG410-06	GU151139
4250	Astraptes LOHAMP	Eudaminae	04-SRNP-36230	MHAHG400-06	GU151137
4251	Astraptes LOHAMP	Eudaminae	05-SRNP-35006	MHAHG387-06	GU151134
4252	Astraptes LOHAMP	Eudaminae	04-SRNP-36185	MHAHG386-06	GU151133
4253	Astraptes LOHAMP	Eudaminae	07-SRNP-35003	MHAHK234-07	JF760358
4254	Astraptes LOHAMP	Eudaminae	06-SRNP-7285	MHAHJ733-07	JF752421
4255	Astraptes LOHAMP	Eudaminae	07-SRNP-35593	MHMXK351-07	JF761610

Tree Order	Species	Subfamily	ACG Sampleid	BOLD Processid	Genbank Accession
4256	Astraptes LOHAMP	Eudaminae	06-SRNP-36339	MHAHK265-07	JF760373
4257	Astraptes LOHAMP	Eudaminae	07-SRNP-1982	MHAHL271-07	JF761590
4258	Astraptes LOHAMP	Eudaminae	04-SRNP-4381	MHAHH279-06	GU155035
4259	Astraptes LOHAMP	Eudaminae	00-SRNP-9957	EPAF217-03	AY666996
4260	Astraptes LOHAMP	Eudaminae	01-SRNP-7375	EPAF243-03	AY666671
4261	Astraptes LOHAMP	Eudaminae	02-SRNP-23196	EPAF273-03	AY666836
4262	Astraptes LOHAMP	Eudaminae	02-SRNP-8010	EPAF378-03	AY666783
4263	Astraptes LOHAMP	Eudaminae	01-SRNP-21272	EPAF245-03	AY666648
4264	Astraptes LOHAMP	Eudaminae	02-SRNP-19946	EPAF325-03	AY666998
4265	Astraptes LOHAMP	Eudaminae	02-SRNP-9906	EPAF275-03	AY666857
4266	Astraptes LOHAMP	Eudaminae	02-SRNP-19945	EPAF346-03	AY666955
4267	Astraptes LOHAMP	Eudaminae	02-SRNP-9775	EPAF266-03	AY666638
4268	Astraptes LOHAMP	Eudaminae	02-SRNP-7893	EPAF281-03	AY666819
4269	Astraptes LOHAMP	Eudaminae	02-SRNP-9769	EPAF268-03	AY666815
4270	Astraptes LOHAMP	Eudaminae	02-SRNP-19727	EPAF474-03	AY666623
4271	Astraptes LOHAMP	Eudaminae	02-SRNP-7856	EPAF272-03	AY666838
4272	Astraptes LOHAMP	Eudaminae	02-SRNP-23086	EPAF271-03	AY666849
4273	Astraptes LOHAMP	Eudaminae	02-SRNP-23926	EPAF475-03	AY666621
4274	Astraptes LOHAMP	Eudaminae	02-SRNP-19182	EPAF295-03	AY667048
4275	Astraptes LOHAMP	Eudaminae	01-SRNP-6254	EPAF232-03	AY666758
4276	Astraptes LOHAMP	Eudaminae	98-SRNP-2129	EPAF161-03	AY666675
4277	Astraptes LOHAMP	Eudaminae	97-SRNP-804	EPAF111-03	AY666801
4278	Astraptes LOHAMP	Eudaminae	98-SRNP-2071	EPAF160-03	AY666679
4279	Astraptes LOHAMP	Eudaminae	01-SRNP-21069	EPAF246-03	AY666646
4280	Astraptes LOHAMP	Eudaminae	98-SRNP-2132	EPAF159-03	AY666688
4281	Astraptes LOHAMP	Eudaminae	98-SRNP-6256	EPAF476-03	AY666633
4282	Astraptes LOHAMP	Eudaminae	98-SRNP-6331	EPAF162-03	AY666706
4283	Astraptes LOHAMP	Eudaminae	98-SRNP-2607	EPAF169-03	AY666672
4284	Astraptes LOHAMP	Eudaminae	94-SRNP-10101	EPAF062-03	AY666877
4285	Astraptes LOHAMP	Eudaminae	99-SRNP-17142	EPAF206-03	AY666989
4286	Astraptes LOHAMP	Eudaminae	99-SRNP-17117	EPAF208-03	AY666963
4287	Astraptes LOHAMP	Eudaminae	97-SRNP-795	EPAF110-03	AY666808
4288	Astraptes LOHAMP	Eudaminae	99-SRNP-17030	EPAF204-03	AY666982
4289	Astraptes LOHAMP	Eudaminae	99-SRNP-17039	EPAF205-03	AY666980
4290	Astraptes LOHAMP	Eudaminae	00-SRNP-11876	EPAF219-03	AY666942
4291	Astraptes LOHAMP	Eudaminae	99-SRNP-1893	EPAF203-03	AY666990
4292	Astraptes LOHAMP	Eudaminae	00-SRNP-11725	EPAF216-03	AY666974
4293	Astraptes LOHAMP	Eudaminae	99-SRNP-17192	EPAF210-03	AY666986
4294	Astraptes LOHAMP	Eudaminae	96-SRNP-7037	EPAF097-03	AY666833
4295	Astraptes LOHAMP	Eudaminae	95-SRNP-244	EPAF063-03	AY666888
4296	Astraptes LOHAMP	Eudaminae	02-SRNP-9525	EPAF262-03	AY666601
4297	Astraptes LOHAMP	Eudaminae	05-SRNP-2170	MHAHG383-06	GU151132
4298	Astraptes LOHAMP	Eudaminae	07-SRNP-45889	MHMXT035-08	JF761599
4299	Astraptes LOHAMP	Eudaminae	99-SRNP-1403	EPAF201-03	AY667000
4300	Astraptes LOHAMP	Eudaminae	97-SRNP-1898	EPAF148-03	AY666723
4301	Astraptes LOHAMP	Eudaminae	97-SRNP-6178	EPAF131-03	AY666759
4302	Astraptes LOHAMP	Eudaminae	97-SRNP-6757	EPAF145-03	AY666738
4303	Astraptes LOHAMP	Eudaminae	95-SRNP-365	EPAF114-03	AY666807
4304	Astraptes LOHAMP	Eudaminae	95-SRNP-867	EPAF066-03	AY666871
4305	Astraptes LOHAMP	Eudaminae	06-SRNP-36701	MHAHK264-07	JF760372
4306	Astraptes LOHAMP	Eudaminae	06-SRNP-36231	MHAHK258-07	JF760367
4307	Astraptes LOHAMP	Eudaminae	04-SRNP-36169	MHAHG385-06	GU151129
4308	Astraptes LOHAMP	Eudaminae	05-SRNP-40961	MHAHG402-06	GU151136
4309	Astraptes LOHAMP	Eudaminae	06-SRNP-7520	MHAHJ572-07	JF752407
4310	Astraptes LOHAMP	Eudaminae	06-SRNP-35608	MHAHJ933-07	JF752432
4311	Astraptes LOHAMP	Eudaminae	06-SRNP-36679	MHAHK271-07	JF760377

Tree Order	Species	Subfamily	ACG Sampleid	BOLD Processid	Genbank Accession
4312	Astraptes LOHAMP	Eudaminae	07-SRNP-30949	MHMXK343-07	JF761613
4313	Astraptes LOHAMP	Eudaminae	07-SRNP-2787	MHMXO851-08	JF761603
4314	Astraptes LOHAMP	Eudaminae	07-SRNP-2858	MHMXO867-08	JF761601
4315	Astraptes LOHAMP	Eudaminae	08-SRNP-71001	MHMXW173-09	JF753693
4316	Astraptes LOHAMP	Eudaminae	08-SRNP-35886	MHMXX479-09	JF777635
4317	Astraptes LOHAMP	Eudaminae	08-SRNP-5784	MHMXY1111-09	GU666434
4318	Astraptes LOHAMP	Eudaminae	09-SRNP-69238	MHMYE1402-09	HM424351
4319	Astraptes LOHAMP	Eudaminae	09-SRNP-72074	MHMYE1403-09	HM424352
4320	Astraptes LOHAMP	Eudaminae	09-SRNP-35515	MHMYE1577-09	HM391137
4321	Astraptes LOHAMP	Eudaminae	09-SRNP-41801	MHMYH117-10	HM887269
4322	Astraptes YESENN	Eudaminae	98-SRNP-2713	EPAF450-03	
4323	Astraptes YESENN	Eudaminae	03-SRNP-9129	MHAHH347-06	GU155191
4324	Astraptes YESENN	Eudaminae	05-SRNP-1745	MHAHG411-06	GU151186
4325	Astraptes YESENN	Eudaminae	03-SRNP-22479	MHAHH332-06	GU155182
4326	Astraptes YESENN	Eudaminae	06-SRNP-31612	MHAHH481-06	GU155198
4327	Astraptes YESENN	Eudaminae	07-SRNP-1716	MHAHL274-07	JF761646
4328	Astraptes YESENN	Eudaminae	07-SRNP-1714	MHAHL299-07	JF761651
4329	Astraptes YESENN	Eudaminae	08-SRNP-1634	MHMXW180-09	JF753700
4330	Astraptes YESENN	Eudaminae	08-SRNP-1633	MHMXW183-09	JF753701
4331	Astraptes YESENN	Eudaminae	95-SRNP-512	EPAF442-03	AY666667
4332	Astraptes YESENN	Eudaminae	02-SRNP-9495	EPAF441-03	AY666678
4333	Astraptes YESENN	Eudaminae	95-SRNP-840	EPAF488-03	AY667059
4334	Astraptes YESENN	Eudaminae	07-SRNP-23723	MHMXT015-08	JF761654
4335	Astraptes YESENN	Eudaminae	05-SRNP-2389	MHAHG433-06	GU151188
4336	Astraptes YESENN	Eudaminae	98-SRNP-14616	EPAF406-03	AY666734
4337	Astraptes YESENN	Eudaminae	02-SRNP-21442	EPAF368-03	AY666798
4338	Astraptes YESENN	Eudaminae	03-SRNP-5145	MHAHH325-06	GU155174
4339	Astraptes YESENN	Eudaminae	06-SRNP-34242	MHAHI512-06	GU155815
4340	Astraptes YESENN	Eudaminae	04-SRNP-48269	MHMXY1116-09	GU666423
4341	Astraptes YESENN	Eudaminae	03-SRNP-3194	MHAHH167-06	GU155118
4342	Astraptes YESENN	Eudaminae	02-SRNP-9939	EPAF264-03	AY666606
4343	Astraptes YESENN	Eudaminae	98-SRNP-7982	EPAF410-03	AY666752
4344	Astraptes YESENN	Eudaminae	06-SRNP-45979	MHAHI002-06	GU155825
4345	Astraptes YESENN	Eudaminae	95-SRNP-4551	EPAF070-03	AY666859
4346	Astraptes YESENN	Eudaminae	99-SRNP-302	EPAF179-03	AY666608
4347	Astraptes YESENN	Eudaminae	04-SRNP-46518	MHAHH230-06	GU155151
4348	Astraptes YESENN	Eudaminae	04-SRNP-46751	MHAHH258-06	GU155157
4349	Astraptes YESENN	Eudaminae	95-SRNP-858	EPAF443-03	AY666681
4350	Astraptes YESENN	Eudaminae	03-SRNP-15877	MHAHH329-06	GU155175
4351	Astraptes YESENN	Eudaminae	08-SRNP-36525	MHMXY1112-09	GU666427
4352	Astraptes YESENN	Eudaminae	08-SRNP-36993	MHMXY1117-09	GU666424
4353	Astraptes YESENN	Eudaminae	08-SRNP-2398	MHMXW177-09	JF753699
4354	Astraptes YESENN	Eudaminae	08-SRNP-2397	MHMXX480-09	JF777638
4355	Astraptes YESENN	Eudaminae	07-SRNP-23843	MHMXR724-08	JF761657
4356	Astraptes YESENN	Eudaminae	08-SRNP-65542	MHMXW169-09	JF753697
4357	Astraptes YESENN	Eudaminae	07-SRNP-45395	MHMXO862-08	JF761662
4358	Astraptes YESENN	Eudaminae	07-SRNP-65880	MHMXR721-08	JF761658
4359	Astraptes YESENN	Eudaminae	07-SRNP-45394	MHMXO841-08	JF761664
4360	Astraptes YESENN	Eudaminae	07-SRNP-45298	MHMXO842-08	JF761663
4361	Astraptes YESENN	Eudaminae	07-SRNP-21700	MHAHL275-07	JF761647
4362	Astraptes YESENN	Eudaminae	07-SRNP-36004	MHAHL302-07	JF761652
4363	Astraptes YESENN	Eudaminae	06-SRNP-65728	MHAHJ815-07	JF752461
4364	Astraptes YESENN	Eudaminae	07-SRNP-1339	MHMXK339-07	JF761665
4365	Astraptes INGCUP	Eudaminae	06-SRNP-7673	MHAHJ587-07	JF752396
4366	Astraptes YESENN	Eudaminae	06-SRNP-7197	MHAHJ729-07	JF752450
4367	Astraptes YESENN	Eudaminae	06-SRNP-45902	MHAHI017-06	GU155817

Tree Order	Species	Subfamily	ACG Sampleid	BOLD Processid	Genbank Accession
4368	Astraptes YESENN	Eudaminae	06-SRNP-7523	MHAHJ574-07	JF752443
4369	Astraptes YESENN	Eudaminae	03-SRNP-12813.1	MHAHH352-06	GU155186
4370	Astraptes YESENN	Eudaminae	03-SRNP-20867	MHAHH353-06	GU155184
4371	Astraptes YESENN	Eudaminae	03-SRNP-20900	MHAHH346-06	GU155189
4372	Astraptes YESENN	Eudaminae	03-SRNP-31190	MHAHH348-06	GU155192
4373	Astraptes YESENN	Eudaminae	03-SRNP-9131	MHAHH335-06	GU155179
4374	Astraptes YESENN	Eudaminae	03-SRNP-13054.1	MHAHH342-06	GU155183
4375	Astraptes YESENN	Eudaminae	03-SRNP-19233	MHAHH174-06	GU155123
4376	Astraptes YESENN	Eudaminae	03-SRNP-21719	MHAHH187-06	GU155129
4377	Astraptes YESENN	Eudaminae	03-SRNP-20039	MHAHH303-06	GU155170
4378	Astraptes YESENN	Eudaminae	03-SRNP-12396.1	MHAHH310-06	GU155172
4379	Astraptes YESENN	Eudaminae	03-SRNP-5830	MHAHH292-06	GU155167
4380	Astraptes YESENN	Eudaminae	03-SRNP-18629	MHAHH300-06	GU155169
4381	Astraptes YESENN	Eudaminae	03-SRNP-19981	MHAHH155-06	GU155117
4382	Astraptes YESENN	Eudaminae	03-SRNP-12811.1	MHAHH158-06	GU155196
4383	Astraptes YESENN	Eudaminae	06-SRNP-2596	MHAHG458-06	GU151194
4384	Astraptes YESENN	Eudaminae	03-SRNP-19923	MHAHH154-06	GU155113
4385	Astraptes YESENN	Eudaminae	06-SRNP-35002	MHAHG448-06	GU151189
4386	Astraptes YESENN	Eudaminae	05-SRNP-343	MHAHG399-06	GU151184
4387	Astraptes YESENN	Eudaminae	05-SRNP-46248	MHAHG389-06	GU151181
4388	Astraptes YESENN	Eudaminae	05-SRNP-46247	MHAHG388-06	GU151182
4389	Astraptes YESENN	Eudaminae	05-SRNP-23710	MHAHG467-06	GU151197
4390	Astraptes YESENN	Eudaminae	07-SRNP-1582	MHAHL284-07	JF761649
4391	Astraptes YESENN	Eudaminae	04-SRNP-46657	MHAHH384-06	GU155193
4392	Astraptes YESENN	Eudaminae	04-SRNP-46761	MHAHH372-06	GU155194
4393	Astraptes YESENN	Eudaminae	03-SRNP-29977	MHAHH334-06	GU155180
4394	Astraptes YESENN	Eudaminae	03-SRNP-19925	MHAHH333-06	GU155181
4395	Astraptes YESENN	Eudaminae	04-SRNP-61087	MHAHH266-06	GU155158
4396	Astraptes YESENN	Eudaminae	04-SRNP-42351	MHAHH236-06	GU155155
4397	Astraptes YESENN	Eudaminae	04-SRNP-21144	MHAHH235-06	GU155154
4398	Astraptes YESENN	Eudaminae	03-SRNP-17617	MHAHH222-06	GU155148
4399	Astraptes YESENN	Eudaminae	03-SRNP-19500	MHAHH221-06	GU155149
4400	Astraptes YESENN	Eudaminae	03-SRNP-11292	MHAHH211-06	GU155143
4401	Astraptes YESENN	Eudaminae	03-SRNP-11291	MHAHH210-06	GU155147
4402	Astraptes YESENN	Eudaminae	03-SRNP-13084.1	MHAHH208-06	GU155146
4403	Astraptes YESENN	Eudaminae	03-SRNP-31311	MHAHH207-06	GU155144
4404	Astraptes YESENN	Eudaminae	04-SRNP-56956	MHAHG425-06	GU151187
4405	Astraptes YESENN	Eudaminae	04-SRNP-56955	MHAHG377-06	GU151179
4406	Astraptes YESENN	Eudaminae	03-SRNP-31192	MHAHH343-06	GU155177
4407	Astraptes YESENN	Eudaminae	06-SRNP-46048	MHAHJ936-07	JF752464
4408	Astraptes YESENN	Eudaminae	03-SRNP-8219	MHAHH194-06	GU155134
4409	Astraptes YESENN	Eudaminae	03-SRNP-21599	MHAHH186-06	GU155130
4410	Astraptes YESENN	Eudaminae	05-SRNP-2166	MHAHG381-06	GU151180
4411	Astraptes YESENN	Eudaminae	06-SRNP-35000	MHAHG457-06	GU151193
4412	Astraptes YESENN	Eudaminae	06-SRNP-2689	MHAHG461-06	GU151195
4413	Astraptes YESENN	Eudaminae	07-SRNP-3219	MHMXO866-08	JF761660
4414	Astraptes YESENN	Eudaminae	06-SRNP-35003	MHAHG450-06	GU151190
4415	Astraptes YESENN	Eudaminae	01-SRNP-9652	EPAF401-03	AY666741
4416	Astraptes YESENN	Eudaminae	07-SRNP-31395	MHAHL297-07	JF761650
4417	Astraptes YESENN	Eudaminae	06-SRNP-7000	MHAHJ579-07	JF752445
4418	Astraptes YESENN	Eudaminae	00-SRNP-11880	EPAF484-03	AY666616
4419	Astraptes YESENN	Eudaminae	99-SRNP-3015	EPAF194-03	AY667006
4420	Astraptes YESENN	Eudaminae	02-SRNP-30212	EPAF296-03	AY667046
4421	Astraptes YESENN	Eudaminae	02-SRNP-30211	EPAF298-03	AY667039
4422	Astraptes YESENN	Eudaminae	00-SRNP-11188	EPAF213-03	AY666958
4423	Astraptes YESENN	Eudaminae	94-SRNP-9330	EPAF060-03	AY666880

Tree Order	Species	Subfamily	ACG Sampleid	BOLD Processid	Genbank Accession
4424	Astraptes YESENN	Eudaminae	02-SRNP-19308	EPAF294-03	AY666795
4425	Astraptes YESENN	Eudaminae	98-SRNP-6561	EPAF164-03	AY666662
4426	Astraptes YESENN	Eudaminae	02-SRNP-31858	EPAF361-03	AY666952
4427	Astraptes YESENN	Eudaminae	02-SRNP-29805	EPAF304-03	AY667028
4428	Astraptes YESENN	Eudaminae	02-SRNP-30062	EPAF301-03	AY667042
4429	Astraptes YESENN	Eudaminae	02-SRNP-33820	EPAF369-03	AY666802
4430	Astraptes YESENN	Eudaminae	02-SRNP-30059	EPAF307-03	AY667019
4431	Astraptes YESENN	Eudaminae	02-SRNP-20190	EPAF367-03	AY666806
4432	Astraptes YESENN	Eudaminae	02-SRNP-21384	EPAF354-03	AY666985
4433	Astraptes YESENN	Eudaminae	99-SRNP-4820	EPAF467-03	AY666642
4434	Astraptes YESENN	Eudaminae	99-SRNP-203	EPAF458-03	AY666652
4435	Astraptes YESENN	Eudaminae	99-SRNP-11	EPAF454-03	AY666660
4436	Astraptes YESENN	Eudaminae	99-SRNP-386	EPAF460-03	AY666649
4437	Astraptes YESENN	Eudaminae	99-SRNP-12	EPAF411-03	AY666769
4438	Astraptes YESENN	Eudaminae	99-SRNP-2446	EPAF178-03	AY666618
4439	Astraptes YESENN	Eudaminae	01-SRNP-6896	EPAF481-03	AY666632
4440	Astraptes YESENN	Eudaminae	01-SRNP-1047	EPAF400-03	AY666743
4441	Astraptes YESENN	Eudaminae	00-SRNP-22164	EPAF399-03	AY666770
4442	Astraptes YESENN	Eudaminae	02-SRNP-9496	EPAF403-03	AY666753
4443	Astraptes YESENN	Eudaminae	98-SRNP-14632	EPAF446-03	AY666685
4444	Astraptes YESENN	Eudaminae	03-SRNP-20899	MHAHH199-06	GU155140
4445	Astraptes YESENN	Eudaminae	02-SRNP-23365	EPAF279-03	AY666832
4446	Astraptes YESENN	Eudaminae	98-SRNP-7874	EPAF409-03	AY666744
4447	Astraptes YESENN	Eudaminae	99-SRNP-301	EPAF459-03	AY666644
4448	Astraptes YESENN	Eudaminae	98-SRNP-4773	EPAF451-03	AY666661
4449	Astraptes YESENN	Eudaminae	06-SRNP-41253	MHAHH485-06	GU155199
4450	Astraptes YESENN	Eudaminae	06-SRNP-35006	MHAHG452-06	GU151191
4451	Astraptes YESENN	Eudaminae	06-SRNP-45767	MHAHI011-06	GU155820
4452	Astraptes YESENN	Eudaminae	07-SRNP-42668	MHMXT034-08	JF761653
4453	Astraptes YESENN	Eudaminae	09-SRNP-57375	MHMYE1404-09	HM424353
4454	Astraptes YESENN	Eudaminae	03-SRNP-19491	MHAHH309-06	GU155171
4455	Astraptes YESENN	Eudaminae	03-SRNP-1327	MHAHH285-06	GU155161
4456	Astraptes YESENN	Eudaminae	06-SRNP-45771	MHAHI003-06	GU155824
4457	Astraptes YESENN	Eudaminae	06-SRNP-3660	MHAHI004-06	GU155816
4458	Astraptes YESENN	Eudaminae	03-SRNP-12405.1	MHAHH157-06	GU155197
4459	Astraptes YESENN	Eudaminae	07-SRNP-23600	MHMXT011-08	JF761656
4460	Astraptes YESENN	Eudaminae	08-SRNP-1537	MHMXW155-09	JF753702
4461	Astraptes YESENN	Eudaminae	06-SRNP-5657	MHAHI007-06	GU155822
4462	Astraptes YESENN	Eudaminae	03-SRNP-7325	MHAHH294-06	GU155162
4463	Astraptes YESENN	Eudaminae	03-SRNP-19236	MHAHH328-06	GU155176
4464	Astraptes YESENN	Eudaminae	03-SRNP-16333	MHAHH286-06	GU155163
4465	Astraptes YESENN	Eudaminae	03-SRNP-16844	MHAHH289-06	GU155168
4466	Astraptes YESENN	Eudaminae	03-SRNP-30303	MHAHH336-06	GU155178
4467	Astraptes YESENN	Eudaminae	06-SRNP-43726	MHAHJ585-07	JF752447
4468	Astraptes YESENN	Eudaminae	03-SRNP-12638.1	MHAHH179-06	GU155126
4469	Astraptes YESENN	Eudaminae	03-SRNP-37033	MHAHH183-06	GU155120
4470	Astraptes YESENN	Eudaminae	03-SRNP-19241	MHAHH176-06	GU155125
4471	Astraptes YESENN	Eudaminae	03-SRNP-20422	MHAHH178-06	GU155127
4472	Astraptes YESENN	Eudaminae	03-SRNP-15876	MHAHH171-06	GU155119
4473	Astraptes YESENN	Eudaminae	03-SRNP-4202	MHAHH169-06	GU155114
4474	Astraptes YESENN	Eudaminae	06-SRNP-35033	MHAHG455-06	GU151192
4475	Astraptes YESENN	Eudaminae	04-SRNP-27148	MHAHG404-06	GU151185
4476	Astraptes YESENN	Eudaminae	05-SRNP-41425	MHAHG393-06	GU151183
4477	Astraptes YESENN	Eudaminae	05-SRNP-23696	MHAHG470-06	GU151200
4478	Astraptes YESENN	Eudaminae	05-SRNP-23780	MHAHG466-06	GU151198
4479	Astraptes YESENN	Eudaminae	06-SRNP-2688	MHAHG460-06	GU151196

Tree Order	Species	Subfamily	ACG Sampleid	BOLD Processid	Genbank Accession
4480	Astraptes YESENN	Eudaminae	95-SRNP-4464	EPAF069-03	AY666860
4481	Astraptes YESENN	Eudaminae	02-SRNP-33384	EPAF362-03	AY666948
4482	Astraptes YESENN	Eudaminae	02-SRNP-31970	EPAF352-03	AY666956
4483	Astraptes YESENN	Eudaminae	00-SRNP-20862	EPAF225-03	AY666862
4484	Astraptes YESENN	Eudaminae	01-SRNP-9728	EPAF439-03	AY666686
4485	Astraptes YESENN	Eudaminae	97-SRNP-6181	EPAF445-03	AY666677
4486	Astraptes YESENN	Eudaminae	99-SRNP-4773	EPAF466-03	AY666636
4487	Astraptes YESENN	Eudaminae	98-SRNP-15072	EPAF448-03	AY666673
4488	Astraptes YESENN	Eudaminae	00-SRNP-10415	EPAF222-03	AY666949
4489	Astraptes YESENN	Eudaminae	02-SRNP-17335	EPAF263-03	AY666597
4490	Astraptes YESENN	Eudaminae	03-SRNP-19235	MHAHH177-06	GU155128
4491	Astraptes YESENN	Eudaminae	04-SRNP-30116	MHAHH181-06	GU155124
4492	Astraptes YESENN	Eudaminae	03-SRNP-21722	MHAHH185-06	GU155132
4493	Astraptes YESENN	Eudaminae	03-SRNP-21302	MHAHH198-06	GU155137
4494	Astraptes YESENN	Eudaminae	04-SRNP-47991	MHAHH233-06	GU155152
4495	Astraptes YESENN	Eudaminae	04-SRNP-56475	MHAHH269-06	GU155159
4496	Astraptes YESENN	Eudaminae	03-SRNP-11346	MHAHH293-06	GU155164
4497	Astraptes YESENN	Eudaminae	03-SRNP-2947	MHAHH296-06	GU155160
4498	Astraptes YESENN	Eudaminae	03-SRNP-31288	MHAHH349-06	GU155190
4499	Astraptes YESENN	Eudaminae	04-SRNP-45272	MHAHH379-06	GU155195
4500	Astraptes YESENN	Eudaminae	06-SRNP-22582	MHAHJ796-07	JF752454
4501	Astraptes YESENN	Eudaminae	06-SRNP-7387	MHAHJ586-07	JF752448
4502	Astraptes YESENN	Eudaminae	06-SRNP-23309	MHAHJ799-07	JF752455
4503	Astraptes YESENN	Eudaminae	06-SRNP-23308	MHAHJ812-07	JF752460
4504	Astraptes YESENN	Eudaminae	06-SRNP-46392	MHAHJ941-07	JF752465
4505	Astraptes YESENN	Eudaminae	09-SRNP-21124	MHMYH115-10	HM887267
4506	Astraptes YESENN	Eudaminae	09-SRNP-80729	MHMYH116-10	HM887268
4507	Astraptes YESENN	Eudaminae	03-SRNP-1328	MHAHH287-06	GU155166
4508	Astraptes YESENN	Eudaminae	99-SRNP-17158	EPAF455-03	AY666669
4509	Astraptes YESENN	Eudaminae	04-SRNP-35012	MHAHH192-06	GU155133
4510	Astraptes YESENN	Eudaminae	02-SRNP-29803	EPAF289-03	AY666804
4511	Astraptes YESENN	Eudaminae	02-SRNP-18849	EPAF285-03	AY666856
4512	Astraptes YESENN	Eudaminae	02-SRNP-19921	EPAF345-03	AY666971
4513	Astraptes YESENN	Eudaminae	06-SRNP-33146	MHAHI511-06	GU155821
4514	Astraptes YESENN	Eudaminae	07-SRNP-1583	MHAHL280-07	JF761648
4515	Astraptes YESENN	Eudaminae	06-SRNP-46808	MHAHJ932-07	JF752463
4516	Astraptes YESENN	Eudaminae	06-SRNP-46810	MHAHJ927-07	JF752462
4517	Astraptes YESENN	Eudaminae	06-SRNP-21578	MHAHJ808-07	JF752458
4518	Astraptes YESENN	Eudaminae	06-SRNP-21607	MHAHJ801-07	JF752456
4519	Astraptes YESENN	Eudaminae	06-SRNP-22150	MHAHJ795-07	JF752453
4520	Astraptes YESENN	Eudaminae	06-SRNP-46491	MHAHJ665-07	JF752449
4521	Astraptes YESENN	Eudaminae	06-SRNP-43633	MHAHJ583-07	JF752446
4522	Astraptes YESENN	Eudaminae	06-SRNP-45450	MHAHI018-06	GU155818
4523	Astraptes YESENN	Eudaminae	06-SRNP-2751	MHAHH490-06	GU155200
4524	Astraptes YESENN	Eudaminae	04-SRNP-46943	MHAHH371-06	GU155115
4525	Astraptes YESENN	Eudaminae	03-SRNP-12768.1	MHAHH351-06	GU155188
4526	Astraptes YESENN	Eudaminae	03-SRNP-13044.1	MHAHH344-06	GU155187
4527	Astraptes YESENN	Eudaminae	03-SRNP-31188	MHAHH331-06	GU155173
4528	Astraptes YESENN	Eudaminae	03-SRNP-12300.1	MHAHH297-06	GU155116
4529	Astraptes YESENN	Eudaminae	03-SRNP-28876	MHAHH339-06	GU155185
4530	Astraptes YESENN	Eudaminae	03-SRNP-15893	MHAHH209-06	GU155145
4531	Astraptes YESENN	Eudaminae	03-SRNP-5207	MHAHH288-06	GU155165
4532	Astraptes YESENN	Eudaminae	03-SRNP-28260	MHAHH205-06	GU155139
4533	Astraptes YESENN	Eudaminae	03-SRNP-19922	MHAHH206-06	GU155138
4534	Astraptes YESENN	Eudaminae	03-SRNP-31295	MHAHH197-06	GU155136
4535	Astraptes YESENN	Eudaminae	03-SRNP-13045.1	MHAHH193-06	GU155135

Tree Order	Species	Subfamily	ACG Sampleid	BOLD Processid	Genbank Accession
4536	Astraptes YESENN	Eudaminae	03-SRNP-37165	MHAHH184-06	GU155131
4537	Astraptes YESENN	Eudaminae	03-SRNP-18626	MHAHH175-06	GU155122
4538	Astraptes YESENN	Eudaminae	98-SRNP-6537	EPAF452-03	AY666666
4539	Astraptes YESENN	Eudaminae	04-SRNP-46760	MHAHH231-06	GU155153
4540	Astraptes YESENN	Eudaminae	05-SRNP-23548	MHAHG471-06	GU151199
4541	Astraptes YESENN	Eudaminae	03-SRNP-28257	MHAHH172-06	GU155121
4542	Astraptes YESENN	Eudaminae	03-SRNP-28256	MHAHH203-06	GU155141
4543	Astraptes YESENN	Eudaminae	05-SRNP-65076	MHAHG468-06	GU151201
4544	Astraptes YESENN	Eudaminae	06-SRNP-43453	MHAHI513-06	GU155814
4545	Astraptes YESENN	Eudaminae	89-SRNP-112	EPAF006-03	AY666945
4546	Astraptes YESENN	Eudaminae	99-SRNP-155	EPAF177-03	AY666637
4547	Astraptes YESENN	Eudaminae	99-SRNP-4383	EPAF176-03	AY666622
4548	Astraptes YESENN	Eudaminae	00-SRNP-11805	EPAF220-03	AY666954
4549	Astraptes YESENN	Eudaminae	02-SRNP-19802	EPAF328-03	AY667001
4550	Astraptes YESENN	Eudaminae	02-SRNP-19922	EPAF353-03	AY666965
4551	Astraptes YESENN	Eudaminae	98-SRNP-7848	EPAF453-03	AY666664
4552	Astraptes YESENN	Eudaminae	01-SRNP-6800	EPAF238-03	AY666680
4553	Astraptes YESENN	Eudaminae	99-SRNP-4436	EPAF465-03	AY666701
4554	Astraptes YESENN	Eudaminae	98-SRNP-15015	EPAF447-03	AY666702
4555	Astraptes YESENN	Eudaminae	97-SRNP-6252	EPAF133-03	AY666750
4556	Astraptes YESENN	Eudaminae	98-SRNP-2712	EPAF170-03	AY666683
4557	Astraptes YESENN	Eudaminae	00-SRNP-22003	EPAF227-03	AY666845
4558	Astraptes YESENN	Eudaminae	98-SRNP-6332	EPAF408-03	AY666732
4559	Astraptes YESENN	Eudaminae	99-SRNP-525	EPAF415-03	AY666717
4560	Astraptes YESENN	Eudaminae	95-SRNP-4402	EPAF068-03	AY666874
4561	Astraptes YESENN	Eudaminae	99-SRNP-524	EPAF468-03	AY666631
4562	Astraptes YESENN	Eudaminae	99-SRNP-4418	EPAF414-03	AY666728
4563	Astraptes YESENN	Eudaminae	99-SRNP-2444	EPAF413-03	AY666736
4564	Astraptes YESENN	Eudaminae	06-SRNP-7361	MHAHJ734-07	JF752452
4565	Astraptes YESENN	Eudaminae	06-SRNP-47922	MHAHK253-07	JF760394
4566	Astraptes YESENN	Eudaminae	07-SRNP-1266	MHMXK336-07	JF761666
4567	Astraptes YESENN	Eudaminae	07-SRNP-42069	MHMXO865-08	JF761661
4568	Astraptes YESENN	Eudaminae	07-SRNP-65846	MHMXR720-08	JF761659
4569	Astraptes YESENN	Eudaminae	08-SRNP-2539	MHMXW175-09	JF753698
4570	Astraptes YESENN	Eudaminae	09-SRNP-2732	MHMYH118-10	HM887270
4571	Astraptes YESENN	Eudaminae	06-SRNP-7524	MHAHJ731-07	JF752451
4572	Astraptes YESENN	Eudaminae	06-SRNP-7624	MHAHJ576-07	JF752444
4573	Astraptes YESENN	Eudaminae	06-SRNP-46103	MHAHI016-06	GU155819
4574	Astraptes YESENN	Eudaminae	04-SRNP-47834	MHAHH252-06	GU155156
4575	Astraptes YESENN	Eudaminae	03-SRNP-19933	MHAHH202-06	GU155142
4576	Astraptes YESENN	Eudaminae	06-SRNP-5658	MHAHI008-06	GU155823
4577	Astraptes YESENN	Eudaminae	93-SRNP-3364	EPAF038-03	AY666909
4578	Astraptes YESENN	Eudaminae	03-SRNP-23561	MHAHH220-06	GU155150
4579	Astraptes YESENN	Eudaminae	99-SRNP-402	EPAF461-03	AY666647
4580	Astraptes YESENN	Eudaminae	06-SRNP-5995	MHAHJ804-07	JF752457
4581	Astraptes YESENN	Eudaminae	06-SRNP-22572	MHAHJ811-07	JF752459
4582	Astraptes YESENN	Eudaminae	09-SRNP-20756	MHMYH183-10	HM887330
4583	Astraptes LONCHO	Eudaminae	04-SRNP-35659	MHAHH255-06	GU155085
4584	Astraptes LONCHO	Eudaminae	97-SRNP-721	EPAF118-03	AY666781
4585	Astraptes LONCHO	Eudaminae	04-SRNP-35510	MHAHH229-06	GU155082
4586	Astraptes LONCHO	Eudaminae	02-SRNP-8471	EPAF253-03	AY667047
4587	Astraptes LONCHO	Eudaminae	02-SRNP-9547	EPAF261-03	AY666602
4588	Astraptes LONCHO	Eudaminae	02-SRNP-8068	EPAF249-03	AY666691
4589	Astraptes LONCHO	Eudaminae	02-SRNP-8361	EPAF380-03	AY667051
4590	Astraptes LONCHO	Eudaminae	02-SRNP-8165	EPAF379-03	AY667057
4591	Astraptes LONCHO	Eudaminae	97-SRNP-840	EPAF119-03	AY666784

Tree Order	Species	Subfamily	ACG Sampleid	BOLD Processid	Genbank Accession
4592	Astraptes LONCHO	Eudaminae	04-SRNP-35270	MHAHH238-06	GU155083
4593	Astraptes LONCHO	Eudaminae	04-SRNP-35660	MHAHH246-06	GU155084
4594	Astraptes LONCHO	Eudaminae	03-SRNP-22396	MHAHH314-06	GU155086
4595	Astraptes LONCHO	Eudaminae	06-SRNP-35047	MHAHG451-06	GU151148
4596	Astraptes LONCHO	Eudaminae	00-SRNP-10977	EPAF224-03	AY666861
4597	Astraptes LONCHO	Eudaminae	02-SRNP-9135	EPAF260-03	AY666617
4598	Astraptes LONCHO	Eudaminae	98-SRNP-2703	EPAF166-03	AY666654
4599	Astraptes LONCHO	Eudaminae	00-SRNP-10502	EPAF223-03	AY667052
4600	Astraptes LONCHO	Eudaminae	99-SRNP-17068	EPAF412-03	AY666724
4601	Astraptes LONCHO	Eudaminae	01-SRNP-21505	EPAF247-03	AY666653
4602	Astraptes LONCHO	Eudaminae	99-SRNP-17184	EPAF456-03	AY666658
4603	Astraptes LONCHO	Eudaminae	02-SRNP-9976	EPAF267-03	AY666704
4604	Astraptes LONCHO	Eudaminae	00-SRNP-10501	EPAF438-03	AY666690
4605	Astraptes LONCHO	Eudaminae	00-SRNP-10500	EPAF437-03	AY666676
4606	Astraptes LONCHO	Eudaminae	89-SRNP-736	EPAF381-03	AY666779
4607	Astraptes LONCHO	Eudaminae	97-SRNP-1147	EPAF121-03	AY666790
4608	Astraptes LONCHO	Eudaminae	97-SRNP-842	EPAF120-03	AY666782
4609	Astraptes LONCHO	Eudaminae	02-SRNP-9448	EPAF258-03	AY666610
4610	Astraptes LONCHO	Eudaminae	97-SRNP-1856	EPAF382-03	AY666788
4611	Astraptes LONCHO	Eudaminae	02-SRNP-8281	EPAF256-03	AY666634
4612	Astraptes LONCHO	Eudaminae	97-SRNP-724	EPAF377-03	AY666785
4613	Astraptes LONCHO	Eudaminae	02-SRNP-8140	EPAF254-03	AY666613
4614	Astraptes LONCHO	Eudaminae	97-SRNP-1127	EPAF123-03	AY666776
4615	Astraptes LONCHO	Eudaminae	97-SRNP-755	EPAF116-03	AY666793
4616	Astraptes LONCHO	Eudaminae	02-SRNP-8439	EPAF422-03	AY666719
4617	Astraptes LONCHO	Eudaminae	00-SRNP-9539	EPAF421-03	AY666707
4618	Astraptes LONCHO	Eudaminae	02-SRNP-8360	EPAF255-03	AY666611
4619	Astraptes LONCHO	Eudaminae	97-SRNP-722	EPAF376-03	AY666780
4620	Astraptes LONCHO	Eudaminae	00-SRNP-9040	EPAF211-03	AY666967
4621	Astraptes LONCHO	Eudaminae	02-SRNP-8249	EPAF250-03	AY666628
4622	Astraptes LONCHO	Eudaminae	02-SRNP-8343	EPAF257-03	AY666640
4623	Astraptes LONCHO	Eudaminae	01-SRNP-21520	EPAF423-03	AY666712
4624	Astraptes LONCHO	Eudaminae	97-SRNP-1050	EPAF113-03	AY666799
4625	Astraptes LONCHO	Eudaminae	02-SRNP-8438	EPAF259-03	AY666609
4626	Astraptes LONCHO	Eudaminae	97-SRNP-723	EPAF117-03	AY666791
4627	Astraptes LONCHO	Eudaminae	97-SRNP-720	EPAF115-03	AY666826
4628	Astraptes LONCHO	Eudaminae	02-SRNP-24219	EPAF370-03	AY666800
4629	Astraptes LONCHO	Eudaminae	99-SRNP-17162	EPAF209-03	AY666977
4630	Astraptes LONCHO	Eudaminae	07-SRNP-35892	MHAHL292-07	JF761616
4631	Astraptes LONCHO	Eudaminae	07-SRNP-36055	MHMXO835-08	JF761619
4632	Astraptes LONCHO	Eudaminae	07-SRNP-45518	MHMXO854-08	JF761618
4633	Astraptes LONCHO	Eudaminae	07-SRNP-36122	MHMXO857-08	JF761617
4634	Astraptes LONCHO	Eudaminae	08-SRNP-36242	MHMXX473-09	JF777636
4635	Astraptes LONCHO	Eudaminae	08-SRNP-35999	MHMXX477-09	JF777637
4636	Astraptes LONCHO	Eudaminae	04-SRNP-56583	MHAHG421-06	GU151146
4637	Astraptes LONCHO	Eudaminae	06-SRNP-35016	MHAHG453-06	GU151147
4638	Astraptes LONCHO	Eudaminae	09-SRNP-36882	MHMYG2028-10	HM885429
4639	Astraptes SENNOV	Eudaminae	01-SRNP-2604	EPAF240-03	AY666693
4640	Astraptes SENNOV	Eudaminae	91-SRNP-2454	EPAF013-03	AY666925
4641	Astraptes SENNOV	Eudaminae	95-SRNP-4409	EPAF067-03	AY666882
4642	Astraptes SENNOV	Eudaminae	05-SRNP-45060	MHAHG444-06	GU151162
4643	Astraptes SENNOV	Eudaminae	93-SRNP-3363	EPAF390-03	AY666786
4644	Astraptes SENNOV	Eudaminae	92-SRNP-3975.1	EPAF387-03	AY666768
4645	Astraptes SENNOV	Eudaminae	95-SRNP-6137	EPAF073-03	AY666864
4646	Astraptes SENNOV	Eudaminae	93-SRNP-3377	EPAF429-03	AY666718
4647	Astraptes SENNOV	Eudaminae	90-SRNP-1711	EPAF385-03	AY666771

Tree Order	Species	Subfamily	ACG Sampleid	BOLD Processid	Genbank Accession
4648	Astraptes SENNOV	Eudaminae	92-SRNP-3975	EPAF021-03	AY666917
4649	Astraptes SENNOV	Eudaminae	96-SRNP-10407	EPAF107-03	AY666817
4650	Astraptes SENNOV	Eudaminae	99-SRNP-10683.1	EPAF198-03	AY667020
4651	Astraptes SENNOV	Eudaminae	96-SRNP-10408	EPAF108-03	AY666812
4652	Astraptes SENNOV	Eudaminae	94-SRNP-8089	EPAF059-03	AY666878
4653	Astraptes SENNOV	Eudaminae	96-SRNP-10410	EPAF109-03	AY666810
4654	Astraptes SENNOV	Eudaminae	96-SRNP-10409	EPAF374-03	AY666792
4655	Astraptes SENNOV	Eudaminae	04-SRNP-30803	MHAHH234-06	GU155097
4656	Astraptes SENNOV	Eudaminae	91-SRNP-1741	EPAF478-03	AY666614
4657	Astraptes SENNOV	Eudaminae	06-SRNP-19450	MHAHK277-07	JF760388
4658	Astraptes SENNOV	Eudaminae	99-SRNP-4144	EPAF174-03	AY666619
4659	Astraptes SENNOV	Eudaminae	06-SRNP-31423	MHAHG465-06	GU151165
4660	Astraptes SENNOV	Eudaminae	93-SRNP-954	EPAF027-03	AY666919
4661	Astraptes SENNOV	Eudaminae	93-SRNP-955	EPAF391-03	AY666787
4662	Astraptes SENNOV	Eudaminae	95-SRNP-8555	EPAF375-03	AY666789
4663	Astraptes SENNOV	Eudaminae	07-SRNP-45042	MHMXK346-07	JF761624
4664	Astraptes SENNOV	Eudaminae	07-SRNP-45041	MHMXK348-07	JF761623
4665	Astraptes SENNOV	Eudaminae	07-SRNP-45040	MHMXK332-07	JF761626
4666	Astraptes SENNOV	Eudaminae	07-SRNP-45043	MHMXK333-07	JF761625
4667	Astraptes SENNOV	Eudaminae	04-SRNP-46788	MHAHH391-06	GU155105
4668	Astraptes SENNOV	Eudaminae	06-SRNP-47119	MHAHJ667-07	JF752436
4669	Astraptes SENNOV	Eudaminae	05-SRNP-60388	MHAHG473-06	GU151167
4670	Astraptes SENNOV	Eudaminae	04-SRNP-46986	MHAHH375-06	GU155103
4671	Astraptes SENNOV	Eudaminae	05-SRNP-47264	MHAHG442-06	GU151151
4672	Astraptes SENNOV	Eudaminae	05-SRNP-59407	MHAHG441-06	GU151152
4673	Astraptes SENNOV	Eudaminae	05-SRNP-46328	MHAHG434-06	GU151160
4674	Astraptes SENNOV	Eudaminae	05-SRNP-46845	MHAHG430-06	GU151157
4675	Astraptes SENNOV	Eudaminae	05-SRNP-46843	MHAHG429-06	GU151158
4676	Astraptes SENNOV	Eudaminae	05-SRNP-59544	MHAHG415-06	GU151156
4677	Astraptes SENNOV	Eudaminae	05-SRNP-57580	MHAHG394-06	GU151154
4678	Astraptes SENNOV	Eudaminae	06-SRNP-20364	MHAHG175-06	GU151149
4679	Astraptes SENNOV	Eudaminae	04-SRNP-48059	MHAHH227-06	GU155095
4680	Astraptes SENNOV	Eudaminae	05-SRNP-46846	MHAHG432-06	GU151155
4681	Astraptes SENNOV	Eudaminae	95-SRNP-4807	EPAF075-03	AY666865
4682	Astraptes SENNOV	Eudaminae	99-SRNP-411	EPAF184-03	AY666603
4683	Astraptes SENNOV	Eudaminae	98-SRNP-2714	EPAF167-03	AY666645
4684	Astraptes SENNOV	Eudaminae	99-SRNP-413	EPAF464-03	AY666668
4685	Astraptes SENNOV	Eudaminae	99-SRNP-409	EPAF490-03	AY667043
4686	Astraptes SENNOV	Eudaminae	99-SRNP-412	EPAF463-03	AY666651
4687	Astraptes SENNOV	Eudaminae	95-SRNP-6435	EPAF081-03	AY666855
4688	Astraptes SENNOV	Eudaminae	96-SRNP-10154	EPAF106-03	AY666803
4689	Astraptes SENNOV	Eudaminae	93-SRNP-3603.2	EPAF042-03	AY666891
4690	Astraptes SENNOV	Eudaminae	89-SRNP-23	EPAF005-03	AY666931
4691	Astraptes SENNOV	Eudaminae	93-SRNP-5558.1	EPAF420-03	AY666709
4692	Astraptes SENNOV	Eudaminae	93-SRNP-3701	EPAF039-03	AY666905
4693	Astraptes SENNOV	Eudaminae	95-SRNP-8643	EPAF086-03	AY666843
4694	Astraptes SENNOV	Eudaminae	95-SRNP-9333	EPAF395-03	AY666751
4695	Astraptes SENNOV	Eudaminae	96-SRNP-8770	EPAF102-03	AY666869
4696	Astraptes SENNOV	Eudaminae	96-SRNP-8797	EPAF103-03	AY666911
4697	Astraptes SENNOV	Eudaminae	99-SRNP-207	EPAF181-03	AY666599
4698	Astraptes SENNOV	Eudaminae	95-SRNP-6869	EPAF077-03	AY666885
4699	Astraptes SENNOV	Eudaminae	99-SRNP-403	EPAF462-03	AY666643
4700	Astraptes SENNOV	Eudaminae	93-SRNP-2206	EPAF389-03	AY666778
4701	Astraptes SENNOV	Eudaminae	93-SRNP-2201	EPAF388-03	AY666766
4702	Astraptes SENNOV	Eudaminae	02-SRNP-13088	EPAF277-03	AY666828
4703	Astraptes SENNOV	Eudaminae	95-SRNP-4533	EPAF489-03	AY667045

Tree Order	Species	Subfamily	ACG Sampleid	BOLD Processid	Genbank Accession
4704	Astraptes SENNOV	Eudaminae	93-SRNP-1025	EPAF028-03	AY666920
4705	Astraptes SENNOV	Eudaminae	93-SRNP-2208	EPAF428-03	AY666735
4706	Astraptes SENNOV	Eudaminae	96-SRNP-8773	EPAF104-03	AY667036
4707	Astraptes SENNOV	Eudaminae	99-SRNP-399	EPAF186-03	AY666697
4708	Astraptes SENNOV	Eudaminae	92-SRNP-69	EPAF426-03	AY666711
4709	Astraptes SENNOV	Eudaminae	92-SRNP-70	EPAF427-03	AY666710
4710	Astraptes SENNOV	Eudaminae	96-SRNP-812	EPAF397-03	AY666761
4711	Astraptes SENNOV	Eudaminae	99-SRNP-410	EPAF183-03	AY666598
4712	Astraptes SENNOV	Eudaminae	95-SRNP-6476	EPAF393-03	AY666755
4713	Astraptes SENNOV	Eudaminae	91-SRNP-3005	EPAF386-03	AY666763
4714	Astraptes SENNOV	Eudaminae	96-SRNP-8769	EPAF101-03	AY666846
4715	Astraptes SENNOV	Eudaminae	04-SRNP-47619	MHAHH228-06	GU155096
4716	Astraptes SENNOV	Eudaminae	04-SRNP-47520	MHAHH245-06	GU155094
4717	Astraptes SENNOV	Eudaminae	04-SRNP-47264	MHAHH263-06	GU155090
4718	Astraptes SENNOV	Eudaminae	06-SRNP-46624	MHAHJ939-07	JF752438
4719	Astraptes SENNOV	Eudaminae	07-SRNP-23823	MHMXR728-08	JF761622
4720	Astraptes SENNOV	Eudaminae	99-SRNP-17188	EPAF457-03	AY666656
4721	Astraptes SENNOV	Eudaminae	03-SRNP-22238	MHAHH196-06	GU155092
4722	Astraptes SENNOV	Eudaminae	03-SRNP-22239	MHAHH295-06	GU155099
4723	Astraptes SENNOV	Eudaminae	06-SRNP-2651	MHAHG462-06	GU151164
4724	Astraptes SENNOV	Eudaminae	06-SRNP-36042	MHMXH847-07	JF760389
4725	Astraptes SENNOV	Eudaminae	03-SRNP-3011	MHAHH168-06	GU155091
4726	Astraptes SENNOV	Eudaminae	05-SRNP-55272	MHAHG447-06	GU151161
4727	Astraptes SENNOV	Eudaminae	05-SRNP-45346	MHAHG391-06	GU151153
4728	Astraptes SENNOV	Eudaminae	95-SRNP-10951	EPAF089-03	AY666850
4729	Astraptes SENNOV	Eudaminae	06-SRNP-47118	MHAHJ622-07	JF752434
4730	Astraptes SENNOV	Eudaminae	07-SRNP-58784	MHMXR729-08	JF761621
4731	Astraptes SENNOV	Eudaminae	92-SRNP-5660	EPAF486-03	AY667055
4732	Astraptes SENNOV	Eudaminae	06-SRNP-31380	MHAHG464-06	GU151166
4733	Astraptes SENNOV	Eudaminae	06-SRNP-46575	MHAHJ670-07	JF752437
4734	Astraptes SENNOV	Eudaminae	06-SRNP-46050	MHAHJ624-07	JF752435
4735	Astraptes SENNOV	Eudaminae	04-SRNP-49527	MHAHH368-06	GU155102
4736	Astraptes SENNOV	Eudaminae	04-SRNP-45246	MHAHH366-06	GU155101
4737	Astraptes SENNOV	Eudaminae	03-SRNP-22051	MHAHH301-06	GU155100
4738	Astraptes SENNOV	Eudaminae	05-SRNP-7221	MHAHG477-06	GU151163
4739	Astraptes SENNOV	Eudaminae	05-SRNP-46983	MHAHG428-06	GU151159
4740	Astraptes SENNOV	Eudaminae	04-SRNP-47268	MHAHH247-06	GU155098
4741	Astraptes SENNOV	Eudaminae	03-SRNP-14687	MHAHH213-06	GU155093
4742	Astraptes SENNOV	Eudaminae	06-SRNP-46931	MHAHJ942-07	JF752439
4743	Astraptes SENNOV	Eudaminae	06-SRNP-460	MHAHG179-06	GU151150
4744	Astraptes SENNOV	Eudaminae	91-SRNP-2715	EPAF483-03	AY666604
4745	Astraptes SENNOV	Eudaminae	93-SRNP-3656	EPAF034-03	AY666899
4746	Astraptes SENNOV	Eudaminae	93-SRNP-3655	EPAF036-03	AY666900
4747	Astraptes SENNOV	Eudaminae	96-SRNP-8802	EPAF100-03	AY666841
4748	Astraptes SENNOV	Eudaminae	99-SRNP-2445	EPAF180-03	AY666605
4749	Astraptes SENNOV	Eudaminae	99-SRNP-401	EPAF188-03	AY666811
4750	Astraptes SENNOV	Eudaminae	96-SRNP-6857	EPAF095-03	AY666829
4751	Astraptes SENNOV	Eudaminae	95-SRNP-6898	EPAF078-03	AY666851
4752	Astraptes SENNOV	Eudaminae	92-SRNP-4398	EPAF425-03	AY666714
4753	Astraptes SENNOV	Eudaminae	95-SRNP-6899	EPAF394-03	AY666762
4754	Astraptes SENNOV	Eudaminae	04-SRNP-47266	MHAHH387-06	GU155104
4755	Astraptes SENNOV	Eudaminae	98-SRNP-3453	EPAF436-03	AY666703
4756	Astraptes SENNOV	Eudaminae	91-SRNP-3045	EPAF016-03	AY666929
4757	Astraptes SENNOV	Eudaminae	93-SRNP-3603.1	EPAF040-03	AY666906
4758	Astraptes SENNOV	Eudaminae	06-SRNP-59595	MHAHK251-07	JF760386
4759	Astraptes SENNOV	Eudaminae	06-SRNP-47181	MHAHK270-07	JF760387

Tree Order	Species	Subfamily	ACG Sampleid	BOLD Processid	Genbank Accession
4760	Astraptes SENNOV	Eudaminae	07-SRNP-30435	MHAHL288-07	JF761620
4761	Astraptes SENNOV	Eudaminae	08-SRNP-55549	MHMXW152-09	JF753695
4762	Astraptes INGCUPnumt	Eudaminae	02-SRNP-13284	EPAF270-03	AY667044
4763	Astraptes SENNOVnumt	Eudaminae	06-SRNP-23360	MHMXK344-07	JF761627
4764	Astraptes YESENNnumt	Eudaminae	06-SRNP-46388	MHAHJ625-07	JF752466
4765	Astraptes YESENNnumt	Eudaminae	06-SRNP-33139	MHAHI510-06	GU155826
4766	Astraptes SENNOVnumt	Eudaminae	04-SRNP-47236	MHAHH396-06	GU155107
4767	Astraptes SENNOVnumt	Eudaminae	03-SRNP-3010	MHAHH170-06	GU155106
4768	Astraptes YESENNnumt	Eudaminae	00-SRNP-11386	EPAF214-03	AY666968
4769	Astraptes SENNOVnumt	Eudaminae	94-SRNP-6452	EPAF058-03	AY666889
4770	Astraptes SENNOVnumt	Eudaminae	93-SRNP-2680	EPAF031-03	AY666943
4771	Astraptes YESENNnumt	Eudaminae	05-SRNP-1089	MHAHG398-06	GU151202
4772	Astraptes YESENNnumt	Eudaminae	06-SRNP-46389	MHAHJ940-07	JF752467
4773	Astraptes YESENNnumt	Eudaminae	07-SRNP-24037	MHMXT038-08	JF761667
4774	Astraptes CELT	Eudaminae	02-SRNP-31493	EPAF308-03	AY667032
4775	Astraptes CELT	Eudaminae	02-SRNP-31136	EPAF313-03	AY667034
4776	Astraptes CELT	Eudaminae	06-SRNP-48008	MHAHK231-07	JF760311
4777	Astraptes CELT	Eudaminae	06-SRNP-47989	MHAHK252-07	JF760312
4778	Astraptes CELT	Eudaminae	07-SRNP-45471	MHMXO846-08	JF761486
4779	Astraptes CELT	Eudaminae	07-SRNP-1959	MHAHL269-07	JF761472
4780	Astraptes CELT	Eudaminae	02-SRNP-29799	EPAF305-03	AY667037
4781	Astraptes CELT	Eudaminae	06-SRNP-3010	MHAHH483-06	GU154925
4782	Astraptes CELT	Eudaminae	05-SRNP-20730	MHAHG474-06	GU151100
4783	Astraptes CELT	Eudaminae	05-SRNP-1765	MHAHG401-06	GU151092
4784	Astraptes CELT	Eudaminae	06-SRNP-3231	MHAHG459-06	GU151096
4785	Astraptes CELT	Eudaminae	05-SRNP-20729	MHAHG476-06	GU151097
4786	Astraptes CELT	Eudaminae	06-SRNP-46989	MHAHJ666-07	JF752385
4787	Astraptes CELT	Eudaminae	07-SRNP-23567	MHMXR732-08	JF761478
4788	Astraptes CELT	Eudaminae	07-SRNP-23826	MHMXR735-08	JF761476
4789	Astraptes CELT	Eudaminae	97-SRNP-9604	EPAF150-03	AY666721
4790	Astraptes CELT	Eudaminae	04-SRNP-48372	MHAHH363-06	GU154922
4791	Astraptes CELT	Eudaminae	06-SRNP-45462	MHAHI019-06	GU155775
4792	Astraptes CELT	Eudaminae	06-SRNP-4757	MHAHI005-06	GU155776
4793	Astraptes CELT	Eudaminae	05-SRNP-754	MHAHG417-06	GU151094
4794	Astraptes CELT	Eudaminae	04-SRNP-46734	MHAHH232-06	GU154919
4795	Astraptes CELT	Eudaminae	02-SRNP-31741	EPAF323-03	AY667041
4796	Astraptes CELT	Eudaminae	02-SRNP-29082	EPAF284-03	AY666839
4797	Astraptes CELT	Eudaminae	04-SRNP-46737	MHAHH390-06	GU154924
4798	Astraptes CELT	Eudaminae	04-SRNP-46063	MHAHH259-06	GU154921
4799	Astraptes CELT	Eudaminae	07-SRNP-1969	MHAHL279-07	JF761475
4800	Astraptes CELT	Eudaminae	94-SRNP-9389	EPAF061-03	AY666879
4801	Astraptes CELT	Eudaminae	99-SRNP-5312	EPAF185-03	AY666630
4802	Astraptes CELT	Eudaminae	02-SRNP-33197	EPAF348-03	AY666969
4803	Astraptes CELT	Eudaminae	01-SRNP-1413	EPAF236-03	AY666689
4804	Astraptes CELT	Eudaminae	02-SRNP-31745	EPAF330-03	AY666993
4805	Astraptes CELT	Eudaminae	02-SRNP-33016	EPAF332-03	AY666988
4806	Astraptes CELT	Eudaminae	02-SRNP-29083	EPAF290-03	AY666818
4807	Astraptes CELT	Eudaminae	02-SRNP-31738	EPAF321-03	AY667012
4808	Astraptes CELT	Eudaminae	02-SRNP-29080	EPAF473-03	AY666635
4809	Astraptes CELT	Eudaminae	02-SRNP-29798	EPAF419-03	AY666727
4810	Astraptes CELT	Eudaminae	96-SRNP-227	EPAF091-03	AY666835
4811	Astraptes CELT	Eudaminae	02-SRNP-30241	EPAF300-03	AY667035
4812	Astraptes CELT	Eudaminae	02-SRNP-33002	EPAF356-03	AY667040
4813	Astraptes CELT	Eudaminae	02-SRNP-29054	EPAF288-03	AY667049
4814	Astraptes CELT	Eudaminae	02-SRNP-31739	EPAF324-03	AY667009
4815	Astraptes CELT	Eudaminae	06-SRNP-3011	MHAHH488-06	GU154926

Tree Order	Species	Subfamily	ACG Sampleid	BOLD Processid	Genbank Accession
4816	Astraptes CELT	Eudaminae	04-SRNP-46459	MHAHH257-06	GU154920
4817	Astraptes CELT	Eudaminae	05-SRNP-20279	MHAHG482-06	GU151101
4818	Astraptes CELT	Eudaminae	05-SRNP-20731	MHAHG475-06	GU151099
4819	Astraptes CELT	Eudaminae	05-SRNP-6757	MHAHG472-06	GU151098
4820	Astraptes CELT	Eudaminae	05-SRNP-1608	MHAHG412-06	GU151093
4821	Astraptes CELT	Eudaminae	05-SRNP-45088	MHAHG395-06	GU151091
4822	Astraptes CELT	Eudaminae	02-SRNP-31377	EPAF317-03	AY667003
4823	Astraptes CELT	Eudaminae	05-SRNP-24514	MHAHG479-06	GU151095
4824	Astraptes CELT	Eudaminae	04-SRNP-46431	MHAHH376-06	GU154923
4825	Astraptes CELT	Eudaminae	06-SRNP-4630	MHAHI009-06	GU155777
4826	Astraptes CELT	Eudaminae	06-SRNP-4631	MHAHI013-06	GU155778
4827	Astraptes CELT	Eudaminae	06-SRNP-9528	MHAHJ802-07	JF752386
4828	Astraptes CELT	Eudaminae	06-SRNP-47745	MHAHJ925-07	JF752387
4829	Astraptes CELT	Eudaminae	07-SRNP-1958	MHAHL276-07	JF761473
4830	Astraptes CELT	Eudaminae	07-SRNP-1967	MHAHL277-07	JF761474
4831	Astraptes CELT	Eudaminae	07-SRNP-45467	MHMXO837-08	JF761487
4832	Astraptes CELT	Eudaminae	07-SRNP-3042	MHMXO850-08	JF761485
4833	Astraptes CELT	Eudaminae	07-SRNP-45470	MHMXO853-08	JF761484
4834	Astraptes CELT	Eudaminae	07-SRNP-45603	MHMXO855-08	JF761483
4835	Astraptes CELT	Eudaminae	07-SRNP-45602	MHMXO858-08	JF761482
4836	Astraptes CELT	Eudaminae	07-SRNP-45608	MHMXO859-08	JF761481
4837	Astraptes CELT	Eudaminae	07-SRNP-45607	MHMXO861-08	JF761480
4838	Astraptes CELT	Eudaminae	07-SRNP-45600	MHMXO868-08	JF761479
4839	Astraptes CELT	Eudaminae	07-SRNP-22224	MHMXR733-08	JF761477
4840	Astraptes CELT	Eudaminae	08-SRNP-4065	MHMXW176-09	JF753665
4841	Astraptes CELT	Eudaminae	09-SRNP-65963	MHMYH119-10	HM887271
4842	Astraptes TRIGO	Eudaminae	06-SRNP-55033	MHAHG173-06	GU151168
4843	Astraptes TRIGO	Eudaminae	07-SRNP-45479	MHMXO863-08	JF761638
4844	Astraptes TRIGO	Eudaminae	07-SRNP-45480	MHMXO869-08	JF761637
4845	Astraptes TRIGO	Eudaminae	06-SRNP-59720	MHAHK247-07	JF760392
4846	Astraptes TRIGO	Eudaminae	07-SRNP-45478	MHMXO845-08	JF761639
4847	Astraptes TRIGO	Eudaminae	07-SRNP-45482	MHMXR725-08	JF761635
4848	Astraptes TRIGO	Eudaminae	02-SRNP-31761	EPAF335-03	AY666981
4849	Astraptes TRIGO	Eudaminae	91-SRNP-2346	EPAF416-03	AY666716
4850	Astraptes TRIGO	Eudaminae	06-SRNP-59719	MHAHK232-07	JF760390
4851	Astraptes TRIGO	Eudaminae	97-SRNP-5364	EPAF147-03	AY666730
4852	Astraptes TRIGO	Eudaminae	97-SRNP-5500	EPAF144-03	AY666725
4853	Astraptes TRIGO	Eudaminae	07-SRNP-55072	MHAHK246-07	JF760391
4854	Astraptes TRIGO	Eudaminae	02-SRNP-30290	EPAF302-03	AY667030
4855	Astraptes TRIGO	Eudaminae	02-SRNP-30291	EPAF303-03	AY667029
4856	Astraptes TRIGO	Eudaminae	92-SRNP-4386	EPAF022-03	AY666930
4857	Astraptes TRIGO	Eudaminae	02-SRNP-31535	EPAF357-03	AY666951
4858	Astraptes TRIGO	Eudaminae	03-SRNP-17651	MHAHH308-06	GU155109
4859	Astraptes TRIGO	Eudaminae	05-SRNP-45112	MHAHG424-06	GU151173
4860	Astraptes TRIGO	Eudaminae	05-SRNP-45125	MHAHG423-06	GU151175
4861	Astraptes TRIGO	Eudaminae	05-SRNP-45076	MHAHG418-06	GU151172
4862	Astraptes TRIGO	Eudaminae	05-SRNP-45073	MHAHG409-06	GU151171
4863	Astraptes TRIGO	Eudaminae	01-SRNP-9825	EPAF239-03	AY666687
4864	Astraptes TRIGO	Eudaminae	02-SRNP-29103	EPAF278-03	AY666840
4865	Astraptes TRIGO	Eudaminae	02-SRNP-31684	EPAF315-03	AY667016
4866	Astraptes TRIGO	Eudaminae	02-SRNP-33258	EPAF349-03	AY666957
4867	Astraptes TRIGO	Eudaminae	02-SRNP-31538	EPAF319-03	AY667005
4868	Astraptes TRIGO	Eudaminae	02-SRNP-31536	EPAF306-03	AY667050
4869	Astraptes TRIGO	Eudaminae	02-SRNP-33259	EPAF329-03	AY666995
4870	Astraptes TRIGO	Eudaminae	02-SRNP-31537	EPAF322-03	AY667011
4871	Astraptes TRIGO	Eudaminae	02-SRNP-31762	EPAF341-03	AY666976

Tree Order	Species	Subfamily	ACG Sampleid	BOLD Processid	Genbank Accession
4872	Astraptes TRIGO	Eudaminae	02-SRNP-31756	EPAF339-03	AY666973
4873	Astraptes TRIGO	Eudaminae	02-SRNP-33261	EPAF334-03	AY666983
4874	Astraptes TRIGO	Eudaminae	98-SRNP-5868	EPAF172-03	AY666639
4875	Astraptes TRIGO	Eudaminae	02-SRNP-31748	EPAF340-03	AY666972
4876	Astraptes TRIGO	Eudaminae	02-SRNP-33260	EPAF342-03	AY666964
4877	Astraptes TRIGO	Eudaminae	02-SRNP-31539	EPAF311-03	AY667024
4878	Astraptes TRIGO	Eudaminae	02-SRNP-31755	EPAF350-03	AY666961
4879	Astraptes TRIGO	Eudaminae	97-SRNP-5215	EPAF143-03	AY666756
4880	Astraptes TRIGO	Eudaminae	97-SRNP-5499	EPAF149-03	AY666708
4881	Astraptes TRIGO	Eudaminae	02-SRNP-31620	EPAF336-03	AY666994
4882	Astraptes TRIGO	Eudaminae	92-SRNP-5726	EPAF025-03	AY666907
4883	Astraptes TRIGO	Eudaminae	02-SRNP-31621	EPAF351-03	AY666959
4884	Astraptes TRIGO	Eudaminae	91-SRNP-2576	EPAF014-03	AY666924
4885	Astraptes TRIGO	Eudaminae	02-SRNP-31784	EPAF333-03	AY666979
4886	Astraptes TRIGO	Eudaminae	02-SRNP-31782	EPAF338-03	AY667008
4887	Astraptes TRIGO	Eudaminae	91-SRNP-2694	EPAF469-03	AY666629
4888	Astraptes TRIGO	Eudaminae	02-SRNP-31817	EPAF331-03	AY666992
4889	Astraptes TRIGO	Eudaminae	02-SRNP-33453	EPAF355-03	AY666984
4890	Astraptes TRIGO	Eudaminae	97-SRNP-4485	EPAF129-03	AY666794
4891	Astraptes TRIGO	Eudaminae	91-SRNP-2631	EPAF482-03	AY666650
4892	Astraptes TRIGO	Eudaminae	92-SRNP-4379	EPAF019-03	AY666923
4893	Astraptes TRIGO	Eudaminae	97-SRNP-5939	EPAF155-03	AY666760
4894	Astraptes TRIGO	Eudaminae	93-SRNP-7152	EPAF049-03	AY666897
4895	Astraptes TRIGO	Eudaminae	93-SRNP-8663	EPAF051-03	AY666887
4896	Astraptes TRIGO	Eudaminae	97-SRNP-5453	EPAF146-03	AY666729
4897	Astraptes TRIGO	Eudaminae	02-SRNP-31820	EPAF343-03	AY666978
4898	Astraptes TRIGO	Eudaminae	02-SRNP-31786	EPAF347-03	AY666970
4899	Astraptes TRIGO	Eudaminae	92-SRNP-4372	EPAF020-03	AY666918
4900	Astraptes TRIGO	Eudaminae	97-SRNP-5173	EPAF140-03	AY666733
4901	Astraptes TRIGO	Eudaminae	87-SRNP-1357	EPAF004-03	AY666932
4902	Astraptes TRIGO	Eudaminae	91-SRNP-2522	EPAF011-03	AY666944
4903	Astraptes TRIGO	Eudaminae	01-SRNP-9111	EPAF230-03	AY666720
4904	Astraptes TRIGO	Eudaminae	96-SRNP-9543	EPAF105-03	AY666816
4905	Astraptes TRIGO	Eudaminae	06-SRNP-55035	MHAHG177-06	GU151169
4906	Astraptes TRIGO	Eudaminae	05-SRNP-45110	MHAHG408-06	GU151170
4907	Astraptes TRIGO	Eudaminae	05-SRNP-45111	MHAHG419-06	GU151174
4908	Astraptes TRIGO	Eudaminae	05-SRNP-65453	MHAHG469-06	GU151176
4909	Astraptes TRIGO	Eudaminae	06-SRNP-58075	MHAHJ590-07	JF752440
4910	Astraptes TRIGO	Eudaminae	06-SRNP-58074	MHAHJ591-07	JF752441
4911	Astraptes TRIGO	Eudaminae	06-SRNP-58073	MHAHJ663-07	JF752442
4912	Astraptes TRIGO	Eudaminae	06-SRNP-59785	MHAHK248-07	JF760393
4913	Astraptes TRIGO	Eudaminae	07-SRNP-45559	MHMXR723-08	JF761636
4914	Astraptes TRIGO	Eudaminae	08-SRNP-2317	MHMXW153-09	JF753696
4915	Urbanus evona	Eudaminae	93-SRNP-3406	CSRII323-04	DQ293761
4916	Urbanus evona	Eudaminae	90-SRNP-1654.1	CSRII317-04	DQ293760
4917	Urbanus evona	Eudaminae	04-SRNP-45778	MHAHC045-05	DQ293763
4918	Urbanus evona	Eudaminae	05-SRNP-66411	MHAHG589-06	GU151870
4919	Urbanus evona	Eudaminae	05-SRNP-59072	MHAHF924-06	GU151015
4920	Urbanus evona	Eudaminae	05-SRNP-57365	MHAHF917-06	GU151014
4921	Urbanus evona	Eudaminae	05-SRNP-57364	MHAHF909-06	GU151013
4922	Urbanus evona	Eudaminae	05-SRNP-58937	MHAHF908-06	GU151012
4923	Urbanus evona	Eudaminae	04-SRNP-15286	MHAHC408-05	DQ293768
4924	Urbanus evona	Eudaminae	04-SRNP-14470	MHAHC278-05	DQ293766
4925	Urbanus evona	Eudaminae	02-SRNP-32829	MHAHC248-05	DQ293765
4926	Urbanus evona	Eudaminae	02-SRNP-32830	MHAHC247-05	DQ293764
4927	Urbanus evona	Eudaminae	01-SRNP-14067	MHAHC341-05	DQ293767

Tree Order	Species	Subfamily	ACG Sampleid	BOLD Processid	Genbank Accession
4928	Urbanus evona	Eudaminae	04-SRNP-45787	MHAHC037-05	DQ293762
4929	Urbanus evona	Eudaminae	97-SRNP-4685	MHAHI938-07	GU156473
4930	Urbanus evona	Eudaminae	02-SRNP-32828	MHAHI968-07	GU156474
4931	Urbanus evona	Eudaminae	06-SRNP-57688	MHAHJ632-07	JF753241
4932	Urbanus evona	Eudaminae	07-SRNP-57988	MHMXO834-08	JF750917
4933	Urbanus esta	Eudaminae	93-SRNP-5934	CSRII327-04	DQ293751
4934	Urbanus esta	Eudaminae	97-SRNP-2009	MHAHD451-05	GU161996
4935	Urbanus esta	Eudaminae	97-SRNP-2006	MHAHD450-05	GU161997
4936	Urbanus esta	Eudaminae	04-SRNP-56961	MHAHC413-05	DQ293758
4937	Urbanus esta	Eudaminae	07-SRNP-45289	MHMXO820-08	JF750916
4938	Urbanus esta	Eudaminae	06-SRNP-40068	MHAHG334-06	GU151848
4939	Urbanus esta	Eudaminae	06-SRNP-1725	MHAHG541-06	GU151849
4940	Urbanus esta	Eudaminae	93-SRNP-1169	CSRII322-04	DQ293750
4941	Urbanus esta	Eudaminae	01-SRNP-3184	MHAHD448-05	GU161994
4942	Urbanus esta	Eudaminae	06-SRNP-19547	MHAHK072-07	JF761271
4943	Urbanus esta	Eudaminae	00-SRNP-1522	MHAHD465-05	GU162007
4944	Urbanus esta	Eudaminae	06-SRNP-31215	MHAHG566-06	GU151863
4945	Urbanus esta	Eudaminae	05-SRNP-416	MHAHF913-06	GU151008
4946	Urbanus esta	Eudaminae	07-SRNP-65020	MHAHL461-07	JF763351
4947	Urbanus esta	Eudaminae	06-SRNP-57925	MHAHJ605-07	JF753240
4948	Urbanus esta	Eudaminae	06-SRNP-2685	MHAHH546-06	GU155700
4949	Urbanus esta	Eudaminae	01-SRNP-17208	MHAHD442-05	GU162005
4950	Urbanus esta	Eudaminae	05-SRNP-65398	MHAHG585-06	GU151842
4951	Urbanus esta	Eudaminae	07-SRNP-20182	MHMXK316-07	JF763354
4952	Urbanus esta	Eudaminae	06-SRNP-31006	MHAHG557-06	GU151861
4953	Urbanus esta	Eudaminae	05-SRNP-33820	MHAHG580-06	GU151843
4954	Urbanus esta	Eudaminae	05-SRNP-58910	MHAHF915-06	GU151009
4955	Urbanus esta	Eudaminae	06-SRNP-31058	MHAHG553-06	GU151856
4956	Urbanus esta	Eudaminae	05-SRNP-59305	MHAHF910-06	GU151005
4957	Urbanus esta	Eudaminae	01-SRNP-473	MHAHD468-05	GU162010
4958	Urbanus esta	Eudaminae	02-SRNP-1933	MHAHD461-05	GU161995
4959	Urbanus esta	Eudaminae	02-SRNP-1930	MHAHD460-05	GU162003
4960	Urbanus esta	Eudaminae	00-SRNP-1520	MHAHD459-05	GU162006
4961	Urbanus esta	Eudaminae	00-SRNP-1519	MHAHD458-05	GU162004
4962	Urbanus esta	Eudaminae	01-SRNP-17209	MHAHD441-05	GU161989
4963	Urbanus esta	Eudaminae	04-SRNP-15166	MHAHC400-05	DQ293756
4964	Urbanus esta	Eudaminae	04-SRNP-15168	MHAHC393-05	DQ293755
4965	Urbanus esta	Eudaminae	02-SRNP-1932	MHAHC246-05	DQ293753
4966	Urbanus esta	Eudaminae	02-SRNP-32029	MHAHD397-05	GU161998
4967	Urbanus esta	Eudaminae	06-SRNP-19546	MHAHI463-06	GU156471
4968	Urbanus esta	Eudaminae	07-SRNP-31014	MHAHL445-07	JF763348
4969	Urbanus esta	Eudaminae	06-SRNP-30965	MHAHG551-06	GU151854
4970	Urbanus esta	Eudaminae	06-SRNP-31059	MHAHG552-06	GU151857
4971	Urbanus esta	Eudaminae	06-SRNP-1984	MHAHG554-06	GU151855
4972	Urbanus esta	Eudaminae	06-SRNP-1745	MHAHG556-06	GU151858
4973	Urbanus esta	Eudaminae	06-SRNP-30964	MHAHG559-06	GU151862
4974	Urbanus esta	Eudaminae	06-SRNP-30990	MHAHG560-06	GU151860
4975	Urbanus esta	Eudaminae	06-SRNP-30961	MHAHG563-06	GU151864
4976	Urbanus esta	Eudaminae	06-SRNP-31004	MHAHG564-06	GU151866
4977	Urbanus esta	Eudaminae	06-SRNP-1743	MHAHG565-06	GU151865
4978	Urbanus esta	Eudaminae	06-SRNP-1985	MHAHG567-06	GU151859
4979	Urbanus esta	Eudaminae	05-SRNP-59539	MHAHF923-06	GU151010
4980	Urbanus esta	Eudaminae	05-SRNP-59065	MHAHF926-06	GU151007
4981	Urbanus esta	Eudaminae	06-SRNP-1798	MHAHG568-06	GU151851
4982	Urbanus esta	Eudaminae	06-SRNP-1747	MHAHG570-06	GU151867
4983	Urbanus esta	Eudaminae	06-SRNP-1746	MHAHG571-06	GU151869

Tree Order	Species	Subfamily	ACG Sampleid	BOLD Processid	Genbank Accession
4984	Urbanus esta	Eudaminae	06-SRNP-1982	MHAHG572-06	GU151868
4985	Urbanus esta	Eudaminae	06-SRNP-2690	MHAHG601-06	GU151844
4986	Urbanus esta	Eudaminae	06-SRNP-4071	MHAHI032-06	GU156472
4987	Urbanus esta	Eudaminae	07-SRNP-56430	MHMXK295-07	JF750915
4988	Urbanus esta	Eudaminae	07-SRNP-1190	MHMXK318-07	JF763353
4989	Urbanus esta	Eudaminae	07-SRNP-65022	MHAHL455-07	JF763349
4990	Urbanus esta	Eudaminae	07-SRNP-65019	MHAHL456-07	JF763350
4991	Urbanus esta	Eudaminae	06-SRNP-1532	MHAHG546-06	GU151850
4992	Urbanus esta	Eudaminae	06-SRNP-30959	MHAHG550-06	GU151853
4993	Urbanus esta	Eudaminae	06-SRNP-30040	MHAHG332-06	GU151845
4994	Urbanus esta	Eudaminae	06-SRNP-1983	MHAHG545-06	GU151852
4995	Urbanus esta	Eudaminae	05-SRNP-417	MHAHF912-06	GU151006
4996	Urbanus esta	Eudaminae	05-SRNP-41969	MHAHF921-06	GU151011
4997	Urbanus esta	Eudaminae	02-SRNP-2902	MHAHD469-05	GU162011
4998	Urbanus esta	Eudaminae	01-SRNP-1157	MHAHD470-05	GU162012
4999	Urbanus esta	Eudaminae	00-SRNP-20821	MHAHD462-05	GU162008
5000	Urbanus esta	Eudaminae	02-SRNP-1896	MHAHD453-05	GU162002
5001	Urbanus esta	Eudaminae	02-SRNP-1436	MHAHD452-05	GU162001
5002	Urbanus esta	Eudaminae	02-SRNP-2373	MHAHD447-05	GU161992
5003	Urbanus esta	Eudaminae	02-SRNP-1435	MHAHD446-05	GU161991
5004	Urbanus esta	Eudaminae	02-SRNP-546	MHAHD445-05	GU161990
5005	Urbanus esta	Eudaminae	01-SRNP-16946	MHAHD398-05	GU162000
5006	Urbanus esta	Eudaminae	02-SRNP-32024	MHAHD396-05	GU161999
5007	Urbanus esta	Eudaminae	04-SRNP-55662	MHAHC701-05	DQ293759
5008	Urbanus esta	Eudaminae	05-SRNP-30144	MHAHC412-05	DQ293757
5009	Urbanus esta	Eudaminae	04-SRNP-30928	MHAHC079-05	DQ293752
5010	Urbanus esta	Eudaminae	02-SRNP-2320	MHAHC252-05	DQ293754
5011	Urbanus esta	Eudaminae	02-SRNP-402	MHAHD443-05	GU162009
5012	Urbanus esta	Eudaminae	02-SRNP-401	MHAHD444-05	GU161993
5013	Urbanus esta	Eudaminae	06-SRNP-20646	MHAHG333-06	GU151846
5014	Urbanus esta	Eudaminae	07-SRNP-58873	MHMXR789-08	JF763352
5015	Urbanus esmeraldus	Eudaminae	97-SRNP-9206	MHAHI982-07	GU156461
5016	Urbanus esmeraldus	Eudaminae	97-SRNP-9537	MHAHI981-07	GU156460
5017	Urbanus esmeraldus	Eudaminae	97-SRNP-9643	MHAHI935-07	GU156416
5018	Urbanus esmeraldus	Eudaminae	97-SRNP-9544	MHAHI934-07	GU156415
5019	Urbanus esmeraldus	Eudaminae	92-SRNP-4090	CSRII321-04	DQ293722
5020	Urbanus esmeraldus	Eudaminae	93-SRNP-6647.1	CSRII329-04	DQ293723
5021	Urbanus esmeraldus	Eudaminae	02-SRNP-28832	MHAHC193-05	DQ293728
5022	Urbanus esmeraldus	Eudaminae	02-SRNP-28843	MHAHD455-05	GU161985
5023	Urbanus esmeraldus	Eudaminae	99-SRNP-3218	MHAHI951-07	GU156431
5024	Urbanus esmeraldus	Eudaminae	02-SRNP-28844	MHAHI965-07	GU156445
5025	Urbanus esmeraldus	Eudaminae	06-SRNP-59207	MHAHK067-07	JF761270
5026	Urbanus esmeraldus	Eudaminae	97-SRNP-9775	MHAHI918-07	GU156399
5027	Urbanus esmeraldus	Eudaminae	06-SRNP-56793	MHAHI031-06	GU156397
5028	Urbanus esmeraldus	Eudaminae	97-SRNP-9209	MHAHI933-07	GU156414
5029	Urbanus esmeraldus	Eudaminae	97-SRNP-9213	MHAHI926-07	GU156407
5030	Urbanus esmeraldus	Eudaminae	97-SRNP-9207	MHAHI936-07	GU156417
5031	Urbanus esmeraldus	Eudaminae	97-SRNP-9532	MHAHI939-07	GU156419
5032	Urbanus esmeraldus	Eudaminae	02-SRNP-15771	MHAHI977-07	GU156456
5033	Urbanus esmeraldus	Eudaminae	01-SRNP-17432	MHAHI980-07	GU156459
5034	Urbanus esmeraldus	Eudaminae	01-SRNP-17161	MHAHC289-05	DQ293734
5035	Urbanus esmeraldus	Eudaminae	07-SRNP-20116	MHMXK300-07	JF763347
5036	Urbanus esmeraldus	Eudaminae	05-SRNP-56429	MHAHF901-06	GU151002
5037	Urbanus esmeraldus	Eudaminae	05-SRNP-56430	MHAHF903-06	GU150996
5038	Urbanus esmeraldus	Eudaminae	03-SRNP-1944	MHAHI944-07	GU156424
5039	Urbanus esmeraldus	Eudaminae	02-SRNP-17015	MHAHI963-07	GU156443

Tree Order	Species	Subfamily	ACG Sampleid	BOLD Processid	Genbank Accession
5040	Urbanus esmeraldus	Eudaminae	03-SRNP-20895	MHAHI960-07	GU156440
5041	Urbanus esmeraldus	Eudaminae	01-SRNP-17567	MHAHI969-07	GU156448
5042	Urbanus esmeraldus	Eudaminae	03-SRNP-17407	MHAHI949-07	GU156429
5043	Urbanus esmeraldus	Eudaminae	05-SRNP-32493	MHAHG579-06	GU151832
5044	Urbanus esmeraldus	Eudaminae	06-SRNP-31303	MHAHG561-06	GU151841
5045	Urbanus esmeraldus	Eudaminae	06-SRNP-31301	MHAHG555-06	GU151840
5046	Urbanus esmeraldus	Eudaminae	06-SRNP-31302	MHAHG543-06	GU151839
5047	Urbanus esmeraldus	Eudaminae	06-SRNP-30507	MHAHG331-06	GU151835
5048	Urbanus esmeraldus	Eudaminae	01-SRNP-17046	MHAHI978-07	GU156457
5049	Urbanus esmeraldus	Eudaminae	01-SRNP-17434	MHAHI976-07	GU156455
5050	Urbanus esmeraldus	Eudaminae	01-SRNP-17160	MHAHI975-07	GU156454
5051	Urbanus esmeraldus	Eudaminae	01-SRNP-17141	MHAHI974-07	GU156453
5052	Urbanus esmeraldus	Eudaminae	01-SRNP-17081	MHAHI973-07	GU156452
5053	Urbanus esmeraldus	Eudaminae	99-SRNP-3219	MHAHI967-07	GU156447
5054	Urbanus esmeraldus	Eudaminae	01-SRNP-17561	MHAHI964-07	GU156444
5055	Urbanus esmeraldus	Eudaminae	02-SRNP-32156	MHAHI962-07	GU156442
5056	Urbanus esmeraldus	Eudaminae	99-SRNP-7525	MHAHI961-07	GU156441
5057	Urbanus esmeraldus	Eudaminae	03-SRNP-30344	MHAHI958-07	GU156438
5058	Urbanus esmeraldus	Eudaminae	04-SRNP-22082	MHAHI953-07	GU156433
5059	Urbanus esmeraldus	Eudaminae	04-SRNP-22250	MHAHI952-07	GU156432
5060	Urbanus esmeraldus	Eudaminae	03-SRNP-17392	MHAHI950-07	GU156430
5061	Urbanus esmeraldus	Eudaminae	03-SRNP-17393	MHAHI948-07	GU156428
5062	Urbanus esmeraldus	Eudaminae	03-SRNP-17394	MHAHI947-07	GU156427
5063	Urbanus esmeraldus	Eudaminae	06-SRNP-33147	MHAHI618-06	GU156470
5064	Urbanus esmeraldus	Eudaminae	06-SRNP-45697	MHAHI036-06	GU156396
5065	Urbanus esmeraldus	Eudaminae	05-SRNP-34511	MHAHG592-06	GU151834
5066	Urbanus esmeraldus	Eudaminae	05-SRNP-32807	MHAHG578-06	GU151833
5067	Urbanus esmeraldus	Eudaminae	05-SRNP-32806	MHAHG577-06	GU151831
5068	Urbanus esmeraldus	Eudaminae	05-SRNP-34529	MHAHG576-06	GU151836
5069	Urbanus esmeraldus	Eudaminae	05-SRNP-32491	MHAHG575-06	GU151837
5070	Urbanus esmeraldus	Eudaminae	05-SRNP-59480	MHAHF922-06	GU151003
5071	Urbanus esmeraldus	Eudaminae	05-SRNP-46986	MHAHF911-06	GU151001
5072	Urbanus esmeraldus	Eudaminae	05-SRNP-45421	MHAHF907-06	GU150998
5073	Urbanus esmeraldus	Eudaminae	05-SRNP-46937	MHAHF906-06	GU150999
5074	Urbanus esmeraldus	Eudaminae	05-SRNP-59048	MHAHF905-06	GU150997
5075	Urbanus esmeraldus	Eudaminae	04-SRNP-26751	MHAHF904-06	GU151000
5076	Urbanus esmeraldus	Eudaminae	01-SRNP-17442	MHAHD929-05	GU161988
5077	Urbanus esmeraldus	Eudaminae	02-SRNP-29483	MHAHD454-05	GU161986
5078	Urbanus esmeraldus	Eudaminae	97-SRNP-4935	MHAHD401-05	GU161987
5079	Urbanus esmeraldus	Eudaminae	04-SRNP-15171	MHAHC398-05	DQ293749
5080	Urbanus esmeraldus	Eudaminae	04-SRNP-16070	MHAHC386-05	DQ293748
5081	Urbanus esmeraldus	Eudaminae	04-SRNP-13825	MHAHC305-05	DQ293737
5082	Urbanus esmeraldus	Eudaminae	04-SRNP-14316	MHAHC302-05	DQ293736
5083	Urbanus esmeraldus	Eudaminae	04-SRNP-48164	MHAHC296-05	DQ293735
5084	Urbanus esmeraldus	Eudaminae	04-SRNP-13677	MHAHC203-05	DQ293730
5085	Urbanus esmeraldus	Eudaminae	04-SRNP-13824	MHAHC308-05	DQ293738
5086	Urbanus esmeraldus	Eudaminae	01-SRNP-17077	MHAHC356-05	DQ293747
5087	Urbanus esmeraldus	Eudaminae	01-SRNP-17185	MHAHC355-05	DQ293746
5088	Urbanus esmeraldus	Eudaminae	01-SRNP-17421	MHAHC354-05	DQ293745
5089	Urbanus esmeraldus	Eudaminae	01-SRNP-17080.01	MHAHC353-05	DQ293744
5090	Urbanus esmeraldus	Eudaminae	01-SRNP-17163	MHAHC352-05	DQ293743
5091	Urbanus esmeraldus	Eudaminae	01-SRNP-17433	MHAHC351-05	DQ293742
5092	Urbanus esmeraldus	Eudaminae	01-SRNP-11002	MHAHC327-05	DQ293740
5093	Urbanus esmeraldus	Eudaminae	02-SRNP-29162	MHAHC201-05	DQ293729
5094	Urbanus esmeraldus	Eudaminae	01-SRNP-17443	MHAHC318-05	DQ293724
5095	Urbanus esmeraldus	Eudaminae	02-SRNP-5542	MHAHC221-05	DQ293732

Tree Order	Species	Subfamily	ACG Sampleid	BOLD Processid	Genbank Accession
5096	Urbanus esmeraldus	Eudaminae	01-SRNP-17426	MHAHC317-05	DQ293739
5097	Urbanus esmeraldus	Eudaminae	01-SRNP-17562	MHAHC226-05	DQ293733
5098	Urbanus esmeraldus	Eudaminae	02-SRNP-13383	MHAHC218-05	DQ293731
5099	Urbanus esmeraldus	Eudaminae	04-SRNP-45661	MHAHC053-05	DQ293727
5100	Urbanus esmeraldus	Eudaminae	04-SRNP-45660	MHAHC014-05	DQ293725
5101	Urbanus esmeraldus	Eudaminae	04-SRNP-45662	MHAHC030-05	DQ293726
5102	Urbanus esmeraldus	Eudaminae	05-SRNP-33816	MHAHG574-06	GU151838
5103	Urbanus esmeraldus	Eudaminae	03-SRNP-16910	MHAHI956-07	GU156436
5104	Urbanus esmeraldus	Eudaminae	01-SRNP-17439	MHAHC328-05	DQ293741
5105	Urbanus esmeraldus	Eudaminae	97-SRNP-4639	MHAHI940-07	GU156420
5106	Urbanus esmeraldus	Eudaminae	97-SRNP-4567	MHAHI941-07	GU156421
5107	Urbanus esmeraldus	Eudaminae	97-SRNP-4640	MHAHI942-07	GU156422
5108	Urbanus esmeraldus	Eudaminae	99-SRNP-8598	MHAHI966-07	GU156446
5109	Urbanus esmeraldus	Eudaminae	04-SRNP-45064	MHAHI954-07	GU156434
5110	Urbanus esmeraldus	Eudaminae	01-SRNP-17131	MHAHI988-07	GU156466
5111	Urbanus esmeraldus	Eudaminae	97-SRNP-4936	MHAHI943-07	GU156423
5112	Urbanus esmeraldus	Eudaminae	97-SRNP-9658	MHAHI925-07	GU156406
5113	Urbanus esmeraldus	Eudaminae	01-SRNP-17047	MHAHI987-07	GU156465
5114	Urbanus esmeraldus	Eudaminae	97-SRNP-9211	MHAHI923-07	GU156404
5115	Urbanus esmeraldus	Eudaminae	04-SRNP-21981	MHAHI957-07	GU156437
5116	Urbanus esmeraldus	Eudaminae	04-SRNP-45065	MHAHI955-07	GU156435
5117	Urbanus esmeraldus	Eudaminae	03-SRNP-19022	MHAHI946-07	GU156426
5118	Urbanus esmeraldus	Eudaminae	03-SRNP-17465	MHAHI945-07	GU156425
5119	Urbanus esmeraldus	Eudaminae	97-SRNP-5646	MHAHI937-07	GU156418
5120	Urbanus esmeraldus	Eudaminae	97-SRNP-9554	MHAHI932-07	GU156413
5121	Urbanus esmeraldus	Eudaminae	96-SRNP-11210	MHAHI931-07	GU156412
5122	Urbanus esmeraldus	Eudaminae	97-SRNP-9552	MHAHI930-07	GU156411
5123	Urbanus esmeraldus	Eudaminae	97-SRNP-9531	MHAHI929-07	GU156409
5124	Urbanus esmeraldus	Eudaminae	97-SRNP-9530	MHAHI928-07	GU156410
5125	Urbanus esmeraldus	Eudaminae	97-SRNP-9208	MHAHI927-07	GU156408
5126	Urbanus esmeraldus	Eudaminae	97-SRNP-9647	MHAHI924-07	GU156405
5127	Urbanus esmeraldus	Eudaminae	97-SRNP-9558	MHAHI921-07	GU156402
5128	Urbanus esmeraldus	Eudaminae	97-SRNP-9645	MHAHI919-07	GU156400
5129	Urbanus esmeraldus	Eudaminae	97-SRNP-9843	MHAHI917-07	GU156398
5130	Urbanus esmeraldus	Eudaminae	97-SRNP-9646	MHAHI920-07	GU156401
5131	Urbanus esmeraldus	Eudaminae	97-SRNP-5807	MHAHI922-07	GU156403
5132	Urbanus esmeraldus	Eudaminae	05-SRNP-32029	MHAHF902-06	GU151004
5133	Urbanus esmeraldus	Eudaminae	03-SRNP-29671	MHAHI959-07	GU156439
5134	Urbanus esmeraldus	Eudaminae	02-SRNP-5961	MHAHI970-07	GU156449
5135	Urbanus esmeraldus	Eudaminae	01-SRNP-17079	MHAHI971-07	GU156450
5136	Urbanus esmeraldus	Eudaminae	01-SRNP-17158	MHAHI972-07	GU156451
5137	Urbanus esmeraldus	Eudaminae	01-SRNP-17566	MHAHI979-07	GU156458
5138	Urbanus esmeraldus	Eudaminae	95-SRNP-9803	MHAHI984-07	GU156462
5139	Urbanus esmeraldus	Eudaminae	95-SRNP-9805	MHAHI985-07	GU156463
5140	Urbanus esmeraldus	Eudaminae	95-SRNP-9968	MHAHI986-07	GU156464
5141	Urbanus esmeraldus	Eudaminae	95-SRNP-8976	MHAHI989-07	GU156467
5142	Urbanus esmeraldus	Eudaminae	01-SRNP-17083	MHAHI990-07	GU156468
5143	Urbanus esmeraldus	Eudaminae	03-SRNP-16636	MHAHI991-07	GU156469
5144	Urbanus esmeraldus	Eudaminae	06-SRNP-34518	MHAHJ638-07	JF753238
5145	Urbanus esmeraldus	Eudaminae	06-SRNP-46734	MHAHJ742-07	JF753239
5146	Urbanus esmeraldus	Eudaminae	07-SRNP-56341	MHMXK294-07	JF763343
5147	Urbanus esmeraldus	Eudaminae	07-SRNP-56359	MHMXK307-07	JF763346
5148	Urbanus esmeraldus	Eudaminae	07-SRNP-56490	MHMXK308-07	JF763345
5149	Urbanus esmeraldus	Eudaminae	07-SRNP-20117	MHMXK310-07	JF763344
5150	Urbanus esmeraldus	Eudaminae	07-SRNP-58471	MHMXO803-08	JF750913
5151	Urbanus esmeraldus	Eudaminae	07-SRNP-57861	MHMXO806-08	JF750914

Tree Order	Species	Subfamily	ACG Sampleid	BOLD Processid	Genbank Accession
5152	Urbanus esmeraldus	Eudaminae	07-SRNP-57862	MHMXO811-08	JF763342
5153	Urbanus esmeraldus	Eudaminae	08-SRNP-21596	MHMXW468-09	JF754369
5154	Urbanus esmeraldus	Eudaminae	08-SRNP-23543	MHMXY947-09	GU666571
5155	Urbanus esmeraldus	Eudaminae	08-SRNP-58525	MHMYC526-09	GU649818
5156	Urbanus proteus	Eudaminae	97-SRNP-2922	MHAHI906-07	GU156622
5157	Urbanus proteus	Eudaminae	02-SRNP-17912	MHAHC316-05	DQ293828
5158	Urbanus proteus	Eudaminae	02-SRNP-17915	MHAHC189-05	DQ293794
5159	Urbanus proteus	Eudaminae	02-SRNP-15156	MHAHC235-05	DQ293813
5160	Urbanus proteus	Eudaminae	02-SRNP-28591	MHAHI704-07	GU156542
5161	Urbanus proteus	Eudaminae	03-SRNP-16230	MHAHI797-07	GU156691
5162	Urbanus proteus	Eudaminae	02-SRNP-17914	MHAHC250-05	DQ293819
5163	Urbanus proteus	Eudaminae	97-SRNP-4503	MHAHI874-07	GU156514
5164	Urbanus proteus	Eudaminae	94-SRNP-744	MHAHI877-07	GU156583
5165	Urbanus proteus	Eudaminae	07-SRNP-55548	MHMXK284-07	JF763380
5166	Urbanus proteus	Eudaminae	08-SRNP-71742	MHMXX467-09	JF750918
5167	Urbanus proteus	Eudaminae	03-SRNP-16609	MHAHI813-07	GU156710
5168	Urbanus proteus	Eudaminae	04-SRNP-12473	MHAHC281-05	DQ293824
5169	Urbanus proteus	Eudaminae	04-SRNP-12477	MHAHC022-05	DQ293786
5170	Urbanus proteus	Eudaminae	02-SRNP-1925	MHAHC196-05	DQ293798
5171	Urbanus proteus	Eudaminae	04-SRNP-12475	MHAHC211-05	DQ293806
5172	Urbanus proteus	Eudaminae	01-SRNP-4528	MHAHI692-07	GU156484
5173	Urbanus proteus	Eudaminae	06-SRNP-4935	MHAHI027-06	GU156490
5174	Urbanus proteus	Eudaminae	06-SRNP-20639	MHAHH551-06	GU155716
5175	Urbanus proteus	Eudaminae	03-SRNP-16767	MHAHI755-07	GU156653
5176	Urbanus proteus	Eudaminae	06-SRNP-31010	MHAHG606-06	GU151880
5177	Urbanus proteus	Eudaminae	05-SRNP-31866	MHAHF918-06	GU151016
5178	Urbanus proteus	Eudaminae	04-SRNP-45320	MHAHC023-05	DQ293787
5179	Urbanus proteus	Eudaminae	04-SRNP-45321	MHAHC007-05	DQ293784
5180	Urbanus proteus	Eudaminae	06-SRNP-33300	MHAHI464-06	GU156487
5181	Urbanus proteus	Eudaminae	02-SRNP-15123	MHAHI719-07	GU156549
5182	Urbanus proteus	Eudaminae	03-SRNP-16621	MHAHI733-07	GU156562
5183	Urbanus proteus	Eudaminae	03-SRNP-16637	MHAHI734-07	GU156577
5184	Urbanus proteus	Eudaminae	03-SRNP-12324	MHAHI774-07	GU156673
5185	Urbanus proteus	Eudaminae	03-SRNP-16601	MHAHI828-07	GU156612
5186	Urbanus proteus	Eudaminae	03-SRNP-16778	MHAHI829-07	GU156619
5187	Urbanus proteus	Eudaminae	02-SRNP-29399	MHAHC244-05	DQ293816
5188	Urbanus proteus	Eudaminae	03-SRNP-16782	MHAHI730-07	GU156564
5189	Urbanus proteus	Eudaminae	03-SRNP-16605	MHAHI775-07	GU156674
5190	Urbanus proteus	Eudaminae	98-SRNP-4627	MHAHI864-07	GU156488
5191	Urbanus proteus	Eudaminae	02-SRNP-29473	MHAHI866-07	GU156494
5192	Urbanus proteus	Eudaminae	02-SRNP-17900	MHAHC243-05	DQ293815
5193	Urbanus proteus	Eudaminae	02-SRNP-17897	MHAHC242-05	DQ293814
5194	Urbanus proteus	Eudaminae	02-SRNP-17894	MHMXF863-07	JF761274
5195	Urbanus proteus	Eudaminae	01-SRNP-12172	MHAHC335-05	DQ293834
5196	Urbanus proteus	Eudaminae	02-SRNP-28088	MHAHI717-07	GU156556
5197	Urbanus proteus	Eudaminae	02-SRNP-28087	MHAHI898-07	GU156604
5198	Urbanus proteus	Eudaminae	97-SRNP-2035	MHAHI851-07	GU156511
5199	Urbanus proteus	Eudaminae	95-SRNP-4168	MHAHI860-07	GU156641
5200	Urbanus proteus	Eudaminae	02-SRNP-15336	MHAHC227-05	DQ293809
5201	Urbanus proteus	Eudaminae	97-SRNP-3765	MHAHI875-07	GU156523
5202	Urbanus proteus	Eudaminae	97-SRNP-450	MHAHI867-07	GU156480
5203	Urbanus proteus	Eudaminae	97-SRNP-4059	MHAHI876-07	GU156519
5204	Urbanus proteus	Eudaminae	07-SRNP-65362	MHMXO817-08	JF763367
5205	Urbanus proteus	Eudaminae	07-SRNP-65363	MHMXO813-08	JF763371
5206	Urbanus proteus	Eudaminae	07-SRNP-65361	MHMXO812-08	JF763372
5207	Urbanus proteus	Eudaminae	04-SRNP-22094	MHAHI785-07	GU156682

Tree Order	Species	Subfamily	ACG Sampleid	BOLD Processid	Genbank Accession
5208	Urbanus proteus	Eudaminae	06-SRNP-20989	MHAHH553-06	GU155715
5209	Urbanus proteus	Eudaminae	06-SRNP-20638	MHAHH534-06	GU155702
5210	Urbanus proteus	Eudaminae	05-SRNP-42674	MHAHG588-06	GU151877
5211	Urbanus proteus	Eudaminae	05-SRNP-42675	MHAHG586-06	GU151876
5212	Urbanus proteus	Eudaminae	02-SRNP-28144	MHAHC253-05	DQ293821
5213	Urbanus proteus	Eudaminae	09-SRNP-44384	MHMYE865-09	GU653737
5214	Urbanus proteus	Eudaminae	95-SRNP-4171	MHAHI859-07	GU156601
5215	Urbanus proteus	Eudaminae	08-SRNP-65051	MHMXT187-08	JF763363
5216	Urbanus proteus	Eudaminae	07-SRNP-20070	MHMXK314-07	JF763382
5217	Urbanus proteus	Eudaminae	02-SRNP-1919	MHAHI911-07	GU156626
5218	Urbanus proteus	Eudaminae	03-SRNP-16226	MHAHI794-07	GU156695
5219	Urbanus proteus	Eudaminae	04-SRNP-45331	MHAHI752-07	GU156650
5220	Urbanus proteus	Eudaminae	04-SRNP-22097	MHAHI749-07	GU156643
5221	Urbanus proteus	Eudaminae	03-SRNP-16604	MHAHI745-07	GU156644
5222	Urbanus proteus	Eudaminae	03-SRNP-16643	MHAHI743-07	GU156636
5223	Urbanus proteus	Eudaminae	03-SRNP-2587	MHAHI728-07	GU156563
5224	Urbanus proteus	Eudaminae	03-SRNP-2943	MHAHI724-07	GU156524
5225	Urbanus proteus	Eudaminae	06-SRNP-20992	MHAHH542-06	GU155710
5226	Urbanus proteus	Eudaminae	03-SRNP-16622	MHAHI729-07	GU156561
5227	Urbanus proteus	Eudaminae	97-SRNP-4440	MHAHI865-07	GU156491
5228	Urbanus proteus	Eudaminae	03-SRNP-16638	MHAHI744-07	GU156635
5229	Urbanus proteus	Eudaminae	97-SRNP-5092	MHAHI916-07	GU156497
5230	Urbanus proteus	Eudaminae	97-SRNP-2067	MHAHI912-07	GU156628
5231	Urbanus proteus	Eudaminae	95-SRNP-4170	MHAHI907-07	GU156623
5232	Urbanus proteus	Eudaminae	97-SRNP-3766	MHAHI904-07	GU156620
5233	Urbanus proteus	Eudaminae	02-SRNP-28072	MHAHI900-07	GU156606
5234	Urbanus proteus	Eudaminae	01-SRNP-452	MHAHI888-07	GU156594
5235	Urbanus proteus	Eudaminae	01-SRNP-978	MHAHI886-07	GU156588
5236	Urbanus proteus	Eudaminae	95-SRNP-6395	MHAHI881-07	GU156587
5237	Urbanus proteus	Eudaminae	95-SRNP-9306	MHAHI880-07	GU156586
5238	Urbanus proteus	Eudaminae	97-SRNP-3010	MHAHI879-07	GU156585
5239	Urbanus proteus	Eudaminae	95-SRNP-9354	MHAHI870-07	GU156507
5240	Urbanus proteus	Eudaminae	94-SRNP-3026	MHAHI868-07	GU156503
5241	Urbanus proteus	Eudaminae	97-SRNP-2037	MHAHI873-07	GU156495
5242	Urbanus proteus	Eudaminae	04-SRNP-45179	MHAHC046-05	DQ293788
5243	Urbanus proteus	Eudaminae	02-SRNP-28093	MHAHI899-07	GU156605
5244	Urbanus proteus	Eudaminae	03-SRNP-16771	MHAHI772-07	GU156672
5245	Urbanus proteus	Eudaminae	02-SRNP-15128	MHAHI862-07	GU156482
5246	Urbanus proteus	Eudaminae	95-SRNP-4169	MHAHI869-07	GU156506
5247	Urbanus proteus	Eudaminae	97-SRNP-5093	MHAHI910-07	GU156627
5248	Urbanus proteus	Eudaminae	97-SRNP-4803	MHAHI902-07	GU156607
5249	Urbanus proteus	Eudaminae	07-SRNP-65369	MHMXR788-08	JF763366
5250	Urbanus proteus	Eudaminae	03-SRNP-16611	MHAHI758-07	GU156655
5251	Urbanus proteus	Eudaminae	03-SRNP-16895	MHAHI725-07	GU156518
5252	Urbanus proteus	Eudaminae	02-SRNP-28761	MHAHI706-07	GU156540
5253	Urbanus proteus	Eudaminae	02-SRNP-28758	MHAHI708-07	GU156534
5254	Urbanus proteus	Eudaminae	01-SRNP-443	MHAHI882-07	GU156584
5255	Urbanus proteus	Eudaminae	04-SRNP-22098	MHAHI757-07	GU156656
5256	Urbanus proteus	Eudaminae	04-SRNP-22007	MHAHI741-07	GU156481
5257	Urbanus proteus	Eudaminae	04-SRNP-47421	MHAHC313-05	DQ293825
5258	Urbanus proteus	Eudaminae	04-SRNP-33891	MHAHC708-05	DQ293840
5259	Urbanus proteus	Eudaminae	05-SRNP-32458	MHAHG583-06	GU151873
5260	Urbanus proteus	Eudaminae	05-SRNP-32459	MHAHG584-06	GU151872
5261	Urbanus proteus	Eudaminae	04-SRNP-22087	MHAHI761-07	GU156659
5262	Urbanus proteus	Eudaminae	03-SRNP-16631	MHAHI780-07	GU156678
5263	Urbanus proteus	Eudaminae	03-SRNP-17441	MHAHI848-07	GU156603

Tree Order	Species	Subfamily	ACG Sampleid	BOLD Processid	Genbank Accession
5264	Urbanus proteus	Eudaminae	07-SRNP-65360	MHMXO816-08	JF763368
5265	Urbanus proteus	Eudaminae	08-SRNP-55989	MHMXW454-09	JF754370
5266	Urbanus proteus	Eudaminae	08-SRNP-55990	MHMXW460-09	JF754374
5267	Urbanus proteus	Eudaminae	02-SRNP-28070	MHAHI720-07	GU156547
5268	Urbanus proteus	Eudaminae	04-SRNP-33890	MHAHE157-05	GU150135
5269	Urbanus proteus	Eudaminae	06-SRNP-32779	MHAHI461-06	GU156489
5270	Urbanus proteus	Eudaminae	97-SRNP-4502	MHMXF858-07	JF761279
5271	Urbanus proteus	Eudaminae	04-SRNP-21999	MHAHI751-07	GU156649
5272	Urbanus proteus	Eudaminae	04-SRNP-21804	MHAHI747-07	GU156645
5273	Urbanus proteus	Eudaminae	07-SRNP-22160	MHAHL450-07	JF763358
5274	Urbanus proteus	Eudaminae	02-SRNP-28876	MHAHC315-05	DQ293827
5275	Urbanus proteus	Eudaminae	05-SRNP-21817	MHAHF925-06	GU151022
5276	Urbanus proteus	Eudaminae	02-SRNP-17496	MHAHI700-07	GU156533
5277	Urbanus proteus	Eudaminae	03-SRNP-16618	MHAHI826-07	GU156618
5278	Urbanus proteus	Eudaminae	94-SRNP-2742	MHAHI871-07	GU156508
5279	Urbanus proteus	Eudaminae	94-SRNP-3012	MHAHI872-07	GU156509
5280	Urbanus proteus	Eudaminae	01-SRNP-1156	MHMXF857-07	JF761280
5281	Urbanus proteus	Eudaminae	03-SRNP-6987	MHAHI739-07	GU156579
5282	Urbanus proteus	Eudaminae	01-SRNP-5597	MHMXF855-07	JF761272
5283	Urbanus proteus	Eudaminae	03-SRNP-2029	MHAHI806-07	GU156704
5284	Urbanus proteus	Eudaminae	02-SRNP-28921	MHAHC229-05	DQ293810
5285	Urbanus proteus	Eudaminae	06-SRNP-20652	MHAHH548-06	GU155714
5286	Urbanus proteus	Eudaminae	08-SRNP-65133	MHMXW459-09	JF754373
5287	Urbanus proteus	Eudaminae	02-SRNP-28879	MHAHC222-05	DQ293808
5288	Urbanus proteus	Eudaminae	04-SRNP-45319	MHAHC087-05	DQ293792
5289	Urbanus proteus	Eudaminae	03-SRNP-2585	MHAHI756-07	GU156654
5290	Urbanus proteus	Eudaminae	04-SRNP-45322	MHAHI847-07	GU156572
5291	Urbanus proteus	Eudaminae	04-SRNP-22092	MHAHI849-07	GU156513
5292	Urbanus proteus	Eudaminae	04-SRNP-32060	MHAHI845-07	GU156571
5293	Urbanus proteus	Eudaminae	03-SRNP-20447	MHAHI846-07	GU156575
5294	Urbanus proteus	Eudaminae	06-SRNP-6068	MHAHJ739-07	JF753245
5295	Urbanus proteus	Eudaminae	06-SRNP-6067	MHAHJ741-07	JF753246
5296	Urbanus proteus	Eudaminae	02-SRNP-17493	MHAHC202-05	DQ293801
5297	Urbanus proteus	Eudaminae	02-SRNP-29476	MHAHI703-07	GU156537
5298	Urbanus proteus	Eudaminae	03-SRNP-16629	MHAHI818-07	GU156637
5299	Urbanus proteus	Eudaminae	02-SRNP-28091	MHAHC194-05	DQ293796
5300	Urbanus proteus	Eudaminae	02-SRNP-17905	MHAHC230-05	DQ293811
5301	Urbanus proteus	Eudaminae	02-SRNP-17917	MHAHC245-05	DQ293817
5302	Urbanus proteus	Eudaminae	02-SRNP-17500	MHAHC219-05	DQ293807
5303	Urbanus proteus	Eudaminae	04-SRNP-22439	MHAHC061-05	DQ293789
5304	Urbanus proteus	Eudaminae	04-SRNP-22142	MHAHC006-05	DQ293783
5305	Urbanus proteus	Eudaminae	04-SRNP-21276	MHAHC062-05	DQ293790
5306	Urbanus proteus	Eudaminae	04-SRNP-45255	MHAHC015-05	DQ293785
5307	Urbanus proteus	Eudaminae	04-SRNP-21421	MHAHC071-05	DQ293791
5308	Urbanus proteus	Eudaminae	03-SRNP-16772	MHAHI817-07	GU156639
5309	Urbanus proteus	Eudaminae	02-SRNP-28089	MHAHI909-07	GU156625
5310	Urbanus proteus	Eudaminae	05-SRNP-42099	MHAHF898-06	GU151021
5311	Urbanus proteus	Eudaminae	04-SRNP-45328	MHAHI782-07	GU156680
5312	Urbanus proteus	Eudaminae	04-SRNP-21992	MHAHI742-07	GU156498
5313	Urbanus proteus	Eudaminae	04-SRNP-22095	MHAHI787-07	GU156684
5314	Urbanus proteus	Eudaminae	06-SRNP-20650	MHAHH550-06	GU155712
5315	Urbanus proteus	Eudaminae	02-SRNP-28763	MHAHI699-07	GU156532
5316	Urbanus proteus	Eudaminae	04-SRNP-21997	MHAHI835-07	GU156510
5317	Urbanus proteus	Eudaminae	04-SRNP-21991	MHAHI838-07	GU156516
5318	Urbanus proteus	Eudaminae	04-SRNP-21993	MHAHI842-07	GU156570
5319	Urbanus proteus	Eudaminae	03-SRNP-17430	MHAHI731-07	GU156560

Tree Order	Species	Subfamily	ACG Sampleid	BOLD Processid	Genbank Accession
5320	Urbanus proteus	Eudaminae	05-SRNP-22290	MHAHF920-06	GU151019
5321	Urbanus proteus	Eudaminae	07-SRNP-56187	MHMXK289-07	JF763377
5322	Urbanus proteus	Eudaminae	06-SRNP-22060	MHAHJ606-07	JF753244
5323	Urbanus proteus	Eudaminae	06-SRNP-20672	MHAHH552-06	GU155717
5324	Urbanus proteus	Eudaminae	06-SRNP-32781	MHAHI043-06	GU156502
5325	Urbanus proteus	Eudaminae	02-SRNP-32259	MHAHC195-05	DQ293797
5326	Urbanus proteus	Eudaminae	02-SRNP-1921	MHAHI897-07	GU156602
5327	Urbanus proteus	Eudaminae	03-SRNP-2512	MHAHI768-07	GU156667
5328	Urbanus proteus	Eudaminae	03-SRNP-16896	MHAHI732-07	GU156557
5329	Urbanus proteus	Eudaminae	03-SRNP-16625	MHAHI781-07	GU156681
5330	Urbanus proteus	Eudaminae	03-SRNP-16606	MHAHI823-07	GU156614
5331	Urbanus proteus	Eudaminae	02-SRNP-28094	MHAHC206-05	DQ293804
5332	Urbanus proteus	Eudaminae	07-SRNP-32675	MHAHL449-07	JF763357
5333	Urbanus proteus	Eudaminae	08-SRNP-71996	MHMXX469-09	JF750919
5334	Urbanus proteus	Eudaminae	09-SRNP-70674	MHMYE1571-09	HM391131
5335	Urbanus proteus	Eudaminae	02-SRNP-17909	MHAHC314-05	DQ293826
5336	Urbanus proteus	Eudaminae	02-SRNP-17892	MHAHC234-05	DQ293812
5337	Urbanus proteus	Eudaminae	06-SRNP-32816	MHAHI617-06	GU156640
5338	Urbanus proteus	Eudaminae	02-SRNP-15161	MHMXF854-07	JF761273
5339	Urbanus proteus	Eudaminae	06-SRNP-32782	MHAHI040-06	GU156493
5340	Urbanus proteus	Eudaminae	06-SRNP-32807	MHAHI041-06	GU156504
5341	Urbanus proteus	Eudaminae	06-SRNP-21095	MHAHH545-06	GU155711
5342	Urbanus proteus	Eudaminae	06-SRNP-20993	MHAHH549-06	GU155713
5343	Urbanus proteus	Eudaminae	06-SRNP-21060	MHAHH543-06	GU155707
5344	Urbanus proteus	Eudaminae	06-SRNP-21281	MHAHH544-06	GU155709
5345	Urbanus proteus	Eudaminae	06-SRNP-20649	MHAHH538-06	GU155701
5346	Urbanus proteus	Eudaminae	06-SRNP-21046	MHAHH539-06	GU155706
5347	Urbanus proteus	Eudaminae	05-SRNP-55628	MHAHF916-06	GU151017
5348	Urbanus proteus	Eudaminae	06-SRNP-30958	MHAHG330-06	GU151881
5349	Urbanus proteus	Eudaminae	05-SRNP-31865	MHAHF899-06	GU151023
5350	Urbanus proteus	Eudaminae	05-SRNP-41526	MHAHF914-06	GU151018
5351	Urbanus proteus	Eudaminae	05-SRNP-33815	MHAHF893-06	GU151020
5352	Urbanus proteus	Eudaminae	05-SRNP-46456	MHAHF894-06	GU151024
5353	Urbanus proteus	Eudaminae	02-SRNP-17495	MHAHD420-05	GU162014
5354	Urbanus proteus	Eudaminae	01-SRNP-12344	MHAHD930-05	GU162013
5355	Urbanus proteus	Eudaminae	02-SRNP-29166	MHAHC197-05	DQ293799
5356	Urbanus proteus	Eudaminae	02-SRNP-17910	MHAHC205-05	DQ293803
5357	Urbanus proteus	Eudaminae	02-SRNP-17902	MHAHC190-05	DQ293795
5358	Urbanus proteus	Eudaminae	02-SRNP-28882	MHAHC198-05	DQ293800
5359	Urbanus proteus	Eudaminae	02-SRNP-17499	MHAHC188-05	DQ293793
5360	Urbanus proteus	Eudaminae	02-SRNP-17904	MHAHC204-05	DQ293802
5361	Urbanus proteus	Eudaminae	01-SRNP-17048	MHAHC350-05	DQ293839
5362	Urbanus proteus	Eudaminae	02-SRNP-17891	MHAHC209-05	DQ293805
5363	Urbanus proteus	Eudaminae	01-SRNP-444	MHAHC337-05	DQ293835
5364	Urbanus proteus	Eudaminae	01-SRNP-5650	MHAHC338-05	DQ293836
5365	Urbanus proteus	Eudaminae	01-SRNP-448	MHAHC340-05	DQ293837
5366	Urbanus proteus	Eudaminae	01-SRNP-12349	MHAHC343-05	DQ293838
5367	Urbanus proteus	Eudaminae	01-SRNP-12371	MHAHC331-05	DQ293832
5368	Urbanus proteus	Eudaminae	01-SRNP-449	MHAHC332-05	DQ293833
5369	Urbanus proteus	Eudaminae	01-SRNP-12318	MHAHC326-05	DQ293830
5370	Urbanus proteus	Eudaminae	01-SRNP-12370	MHAHC330-05	DQ293831
5371	Urbanus proteus	Eudaminae	01-SRNP-12346	MHAHC324-05	DQ293829
5372	Urbanus proteus	Eudaminae	01-SRNP-12345	MHAHC325-05	DQ293782
5373	Urbanus proteus	Eudaminae	04-SRNP-45076	MHAHI754-07	GU156652
5374	Urbanus proteus	Eudaminae	03-SRNP-7405	MHAHI759-07	GU156657
5375	Urbanus proteus	Eudaminae	04-SRNP-45299	MHAHI750-07	GU156648

Tree Order	Species	Subfamily	ACG Sampleid	BOLD Processid	Genbank Accession
5376	Urbanus proteus	Eudaminae	04-SRNP-45323	MHAHI753-07	GU156651
5377	Urbanus proteus	Eudaminae	02-SRNP-31168	MHAHI696-07	GU156527
5378	Urbanus proteus	Eudaminae	02-SRNP-28914	MHAHI697-07	GU156525
5379	Urbanus proteus	Eudaminae	02-SRNP-16019	MHAHI694-07	GU156529
5380	Urbanus proteus	Eudaminae	02-SRNP-28092	MHAHI695-07	GU156528
5381	Urbanus proteus	Eudaminae	01-SRNP-446	MHAHI691-07	GU156486
5382	Urbanus proteus	Eudaminae	01-SRNP-917	MHAHI693-07	GU156530
5383	Urbanus proteus	Eudaminae	02-SRNP-17899	MHAHI689-07	GU156630
5384	Urbanus proteus	Eudaminae	02-SRNP-17918	MHAHI690-07	GU156483
5385	Urbanus proteus	Eudaminae	01-SRNP-447	MHMXF862-07	JF761275
5386	Urbanus proteus	Eudaminae	02-SRNP-17896	MHMXF864-07	JF761282
5387	Urbanus proteus	Eudaminae	01-SRNP-453	MHMXF860-07	JF761277
5388	Urbanus proteus	Eudaminae	01-SRNP-4731	MHMXF861-07	JF761276
5389	Urbanus proteus	Eudaminae	02-SRNP-15124	MHMXF856-07	JF761281
5390	Urbanus proteus	Eudaminae	01-SRNP-12352	MHMXF859-07	JF761278
5391	Urbanus proteus	Eudaminae	06-SRNP-33586	MHAHI465-06	GU156492
5392	Urbanus proteus	Eudaminae	06-SRNP-33301	MHAHI616-06	GU156642
5393	Urbanus proteus	Eudaminae	06-SRNP-32780	MHAHI042-06	GU156505
5394	Urbanus proteus	Eudaminae	06-SRNP-32342	MHAHI044-06	GU156499
5395	Urbanus proteus	Eudaminae	06-SRNP-21280	MHAHH540-06	GU155705
5396	Urbanus proteus	Eudaminae	06-SRNP-20994	MHAHH541-06	GU155708
5397	Urbanus proteus	Eudaminae	06-SRNP-31653	MHAHH535-06	GU155703
5398	Urbanus proteus	Eudaminae	06-SRNP-20991	MHAHH536-06	GU155704
5399	Urbanus proteus	Eudaminae	05-SRNP-24255	MHAHG595-06	GU151878
5400	Urbanus proteus	Eudaminae	06-SRNP-45207	MHAHG598-06	GU151879
5401	Urbanus proteus	Eudaminae	05-SRNP-32492	MHAHG581-06	GU151875
5402	Urbanus proteus	Eudaminae	05-SRNP-32595	MHAHG582-06	GU151874
5403	Urbanus proteus	Eudaminae	05-SRNP-42295	MHAHF895-06	GU151026
5404	Urbanus proteus	Eudaminae	05-SRNP-31008	MHAHF896-06	GU151025
5405	Urbanus proteus	Eudaminae	02-SRNP-15154	MHAHC254-05	DQ293822
5406	Urbanus proteus	Eudaminae	04-SRNP-12474	MHAHC280-05	DQ293823
5407	Urbanus proteus	Eudaminae	02-SRNP-17907	MHAHC249-05	DQ293818
5408	Urbanus proteus	Eudaminae	02-SRNP-29478	MHAHC251-05	DQ293820
5409	Urbanus proteus	Eudaminae	04-SRNP-22006	MHAHI790-07	GU156690
5410	Urbanus proteus	Eudaminae	03-SRNP-16908	MHAHI791-07	GU156689
5411	Urbanus proteus	Eudaminae	03-SRNP-16781	MHAHI777-07	GU156675
5412	Urbanus proteus	Eudaminae	03-SRNP-16617	MHAHI778-07	GU156677
5413	Urbanus proteus	Eudaminae	03-SRNP-2586	MHAHI770-07	GU156670
5414	Urbanus proteus	Eudaminae	03-SRNP-16902	MHAHI771-07	GU156669
5415	Urbanus proteus	Eudaminae	03-SRNP-8110	MHAHI767-07	GU156665
5416	Urbanus proteus	Eudaminae	03-SRNP-2945	MHAHI769-07	GU156666
5417	Urbanus proteus	Eudaminae	03-SRNP-16897	MHAHI765-07	GU156663
5418	Urbanus proteus	Eudaminae	03-SRNP-2584	MHAHI766-07	GU156664
5419	Urbanus proteus	Eudaminae	04-SRNP-22103	MHAHI763-07	GU156661
5420	Urbanus proteus	Eudaminae	03-SRNP-2108	MHAHI764-07	GU156660
5421	Urbanus proteus	Eudaminae	04-SRNP-22000	MHAHI760-07	GU156658
5422	Urbanus proteus	Eudaminae	04-SRNP-21996	MHAHI762-07	GU156662
5423	Urbanus proteus	Eudaminae	04-SRNP-22004	MHAHI746-07	GU156646
5424	Urbanus proteus	Eudaminae	04-SRNP-45366	MHAHI748-07	GU156647
5425	Urbanus proteus	Eudaminae	04-SRNP-32097	MHAHI738-07	GU156581
5426	Urbanus proteus	Eudaminae	04-SRNP-22008	MHAHI740-07	GU156582
5427	Urbanus proteus	Eudaminae	03-SRNP-16893	MHAHI736-07	GU156578
5428	Urbanus proteus	Eudaminae	03-SRNP-16899	MHAHI737-07	GU156580
5429	Urbanus proteus	Eudaminae	03-SRNP-16635	MHAHI727-07	GU156559
5430	Urbanus proteus	Eudaminae	03-SRNP-16774	MHAHI735-07	GU156576
5431	Urbanus proteus	Eudaminae	03-SRNP-2535	MHAHI723-07	GU156543

Tree Order	Species	Subfamily	ACG Sampleid	BOLD Processid	Genbank Accession
5432	Urbanus proteus	Eudaminae	03-SRNP-16624	MHAHI726-07	GU156558
5433	Urbanus proteus	Eudaminae	02-SRNP-15147	MHAHI721-07	GU156546
5434	Urbanus proteus	Eudaminae	03-SRNP-2027	MHAHI722-07	GU156544
5435	Urbanus proteus	Eudaminae	02-SRNP-28073	MHAHI716-07	GU156548
5436	Urbanus proteus	Eudaminae	02-SRNP-7152	MHAHI718-07	GU156555
5437	Urbanus proteus	Eudaminae	02-SRNP-17906	MHAHI707-07	GU156536
5438	Urbanus proteus	Eudaminae	01-SRNP-976	MHAHI709-07	GU156531
5439	Urbanus proteus	Eudaminae	02-SRNP-17901	MHAHI702-07	GU156539
5440	Urbanus proteus	Eudaminae	02-SRNP-28917	MHAHI705-07	GU156541
5441	Urbanus proteus	Eudaminae	02-SRNP-28874	MHAHI698-07	GU156478
5442	Urbanus proteus	Eudaminae	02-SRNP-17911	MHAHI701-07	GU156538
5443	Urbanus proteus	Eudaminae	03-SRNP-2940	MHAHI798-07	GU156696
5444	Urbanus proteus	Eudaminae	03-SRNP-16894	MHAHI799-07	GU156697
5445	Urbanus proteus	Eudaminae	03-SRNP-19131	MHAHI795-07	GU156694
5446	Urbanus proteus	Eudaminae	03-SRNP-2367	MHAHI796-07	GU156692
5447	Urbanus proteus	Eudaminae	03-SRNP-2588	MHAHI792-07	GU156686
5448	Urbanus proteus	Eudaminae	03-SRNP-2028	MHAHI793-07	GU156693
5449	Urbanus proteus	Eudaminae	04-SRNP-22090	MHAHI788-07	GU156688
5450	Urbanus proteus	Eudaminae	04-SRNP-22089	MHAHI789-07	GU156687
5451	Urbanus proteus	Eudaminae	03-SRNP-2093	MHAHI784-07	GU156683
5452	Urbanus proteus	Eudaminae	04-SRNP-22002	MHAHI786-07	GU156685
5453	Urbanus proteus	Eudaminae	03-SRNP-20714	MHAHI779-07	GU156676
5454	Urbanus proteus	Eudaminae	03-SRNP-16616	MHAHI783-07	GU156679
5455	Urbanus proteus	Eudaminae	04-SRNP-45332	MHAHI773-07	GU156671
5456	Urbanus proteus	Eudaminae	03-SRNP-18903	MHAHI776-07	GU156668
5457	Urbanus proteus	Eudaminae	02-SRNP-28096	MHAHI714-07	GU156552
5458	Urbanus proteus	Eudaminae	02-SRNP-28071	MHAHI715-07	GU156550
5459	Urbanus proteus	Eudaminae	02-SRNP-17898	MHAHI712-07	GU156553
5460	Urbanus proteus	Eudaminae	02-SRNP-15366	MHAHI713-07	GU156554
5461	Urbanus proteus	Eudaminae	02-SRNP-28090	MHAHI710-07	GU156526
5462	Urbanus proteus	Eudaminae	02-SRNP-1917	MHAHI711-07	GU156551
5463	Urbanus proteus	Eudaminae	03-SRNP-16594	MHAHI822-07	GU156613
5464	Urbanus proteus	Eudaminae	03-SRNP-17445	MHAHI824-07	GU156615
5465	Urbanus proteus	Eudaminae	03-SRNP-16769	MHAHI820-07	GU156609
5466	Urbanus proteus	Eudaminae	03-SRNP-16898	MHAHI821-07	GU156610
5467	Urbanus proteus	Eudaminae	03-SRNP-2030	MHAHI811-07	GU156707
5468	Urbanus proteus	Eudaminae	03-SRNP-2112	MHAHI812-07	GU156711
5469	Urbanus proteus	Eudaminae	03-SRNP-2583	MHAHI809-07	GU156708
5470	Urbanus proteus	Eudaminae	04-SRNP-21995	MHAHI810-07	GU156709
5471	Urbanus proteus	Eudaminae	03-SRNP-2537	MHAHI807-07	GU156705
5472	Urbanus proteus	Eudaminae	03-SRNP-16770	MHAHI808-07	GU156706
5473	Urbanus proteus	Eudaminae	03-SRNP-15468	MHAHI804-07	GU156701
5474	Urbanus proteus	Eudaminae	03-SRNP-2944	MHAHI805-07	GU156703
5475	Urbanus proteus	Eudaminae	03-SRNP-2536	MHAHI802-07	GU156699
5476	Urbanus proteus	Eudaminae	03-SRNP-2582	MHAHI803-07	GU156702
5477	Urbanus proteus	Eudaminae	03-SRNP-16634	MHAHI800-07	GU156698
5478	Urbanus proteus	Eudaminae	03-SRNP-17406	MHAHI801-07	GU156700
5479	Urbanus proteus	Eudaminae	09-SRNP-70673	MHMYE1458-09	GU653540
5480	Urbanus proteus	Eudaminae	09-SRNP-70675	MHMYE1459-09	GU653537
5481	Urbanus proteus	Eudaminae	09-SRNP-44562	MHMYE1456-09	GU653542
5482	Urbanus proteus	Eudaminae	09-SRNP-70361	MHMYE1457-09	GU653539
5483	Urbanus proteus	Eudaminae	09-SRNP-70360	MHMYE889-09	GU653712
5484	Urbanus proteus	Eudaminae	09-SRNP-44561	MHMYE1455-09	GU653541
5485	Urbanus proteus	Eudaminae	09-SRNP-44385	MHMYE866-09	GU653734
5486	Urbanus proteus	Eudaminae	09-SRNP-44317	MHMYE868-09	GU653735
5487	Urbanus proteus	Eudaminae	08-SRNP-72574	MHMXY942-09	GU666582

Tree Order	Species	Subfamily	ACG Sampleid	BOLD Processid	Genbank Accession
5488	Urbanus proteus	Eudaminae	08-SRNP-72588	MHMXY948-09	GU666572
5489	Urbanus proteus	Eudaminae	08-SRNP-71676	MHMXX1182-09	JF778582
5490	Urbanus proteus	Eudaminae	08-SRNP-71995	MHMXX468-09	JF750920
5491	Urbanus proteus	Eudaminae	08-SRNP-45057	MHMXW470-09	JF754383
5492	Urbanus proteus	Eudaminae	08-SRNP-45169	MHMXW472-09	JF754384
5493	Urbanus proteus	Eudaminae	08-SRNP-71277	MHMXW467-09	JF754381
5494	Urbanus proteus	Eudaminae	08-SRNP-1259	MHMXW469-09	JF754382
5495	Urbanus proteus	Eudaminae	08-SRNP-65631	MHMXW465-09	JF754379
5496	Urbanus proteus	Eudaminae	08-SRNP-65632	MHMXW466-09	JF754380
5497	Urbanus proteus	Eudaminae	08-SRNP-65633	MHMXW463-09	JF754377
5498	Urbanus proteus	Eudaminae	08-SRNP-65634	MHMXW464-09	JF754378
5499	Urbanus proteus	Eudaminae	07-SRNP-65419	MHMXR791-08	JF763364
5500	Urbanus proteus	Eudaminae	08-SRNP-65052	MHMXT188-08	JF763362
5501	Urbanus proteus	Eudaminae	07-SRNP-57891	MHMXO810-08	JF763373
5502	Urbanus proteus	Eudaminae	07-SRNP-65273	MHMXO814-08	JF763370
5503	Urbanus proteus	Eudaminae	07-SRNP-1369	MHMXK305-07	JF763385
5504	Urbanus proteus	Eudaminae	07-SRNP-56212	MHMXK306-07	JF763384
5505	Urbanus proteus	Eudaminae	07-SRNP-20079	MHMXK313-07	JF763383
5506	Urbanus proteus	Eudaminae	07-SRNP-20455	MHMXK315-07	JF763381
5507	Urbanus proteus	Eudaminae	02-SRNP-5760	MHAHI914-07	GU156631
5508	Urbanus proteus	Eudaminae	02-SRNP-29402	MHAHI915-07	GU156496
5509	Urbanus proteus	Eudaminae	07-SRNP-55788	MHMXK287-07	JF763379
5510	Urbanus proteus	Eudaminae	07-SRNP-56167	MHMXK288-07	JF763378
5511	Urbanus proteus	Eudaminae	07-SRNP-32038	MHMXK297-07	JF763387
5512	Urbanus proteus	Eudaminae	07-SRNP-20045	MHMXK299-07	JF763386
5513	Urbanus proteus	Eudaminae	02-SRNP-15127	MHAHI894-07	GU156598
5514	Urbanus proteus	Eudaminae	02-SRNP-28881	MHAHI895-07	GU156599
5515	Urbanus proteus	Eudaminae	02-SRNP-28760	MHAHI896-07	GU156600
5516	Urbanus proteus	Eudaminae	97-SRNP-4472	MHAHI901-07	GU156608
5517	Urbanus proteus	Eudaminae	02-SRNP-17913	MHAHI903-07	GU156611
5518	Urbanus proteus	Eudaminae	02-SRNP-2958	MHAHI905-07	GU156621
5519	Urbanus proteus	Eudaminae	01-SRNP-1274	MHAHI908-07	GU156624
5520	Urbanus proteus	Eudaminae	02-SRNP-14208	MHAHI913-07	GU156629
5521	Urbanus proteus	Eudaminae	02-SRNP-15153	MHAHI885-07	GU156591
5522	Urbanus proteus	Eudaminae	01-SRNP-450	MHAHI889-07	GU156593
5523	Urbanus proteus	Eudaminae	02-SRNP-15121	MHAHI890-07	GU156592
5524	Urbanus proteus	Eudaminae	02-SRNP-15567	MHAHI891-07	GU156595
5525	Urbanus proteus	Eudaminae	02-SRNP-15155	MHAHI892-07	GU156597
5526	Urbanus proteus	Eudaminae	02-SRNP-2957	MHAHI893-07	GU156596
5527	Urbanus proteus	Eudaminae	02-SRNP-29165	MHAHI861-07	GU156479
5528	Urbanus proteus	Eudaminae	97-SRNP-9660	MHAHI863-07	GU156485
5529	Urbanus proteus	Eudaminae	01-SRNP-12350	MHAHI883-07	GU156589
5530	Urbanus proteus	Eudaminae	01-SRNP-445	MHAHI884-07	GU156590
5531	Urbanus proteus	Eudaminae	02-SRNP-17908	MHAHI857-07	GU156567
5532	Urbanus proteus	Eudaminae	01-SRNP-977	MHAHI858-07	GU156568
5533	Urbanus proteus	Eudaminae	01-SRNP-451	MHAHI853-07	GU156535
5534	Urbanus proteus	Eudaminae	01-SRNP-981	MHAHI854-07	GU156545
5535	Urbanus proteus	Eudaminae	02-SRNP-28762	MHAHI855-07	GU156565
5536	Urbanus proteus	Eudaminae	02-SRNP-28920	MHAHI856-07	GU156566
5537	Urbanus proteus	Eudaminae	03-SRNP-16599	MHAHI850-07	GU156512
5538	Urbanus proteus	Eudaminae	01-SRNP-1273	MHAHI852-07	GU156515
5539	Urbanus proteus	Eudaminae	04-SRNP-45304	MHAHI843-07	GU156573
5540	Urbanus proteus	Eudaminae	04-SRNP-45579	MHAHI844-07	GU156574
5541	Urbanus proteus	Eudaminae	03-SRNP-17056	MHAHI840-07	GU156634
5542	Urbanus proteus	Eudaminae	03-SRNP-20455	MHAHI841-07	GU156632
5543	Urbanus proteus	Eudaminae	04-SRNP-45408	MHAHI837-07	GU156520

Tree Order	Species	Subfamily	ACG Sampleid	BOLD Processid	Genbank Accession
5544	Urbanus proteus	Eudaminae	03-SRNP-16784	MHAHI839-07	GU156633
5545	Urbanus proteus	Eudaminae	04-SRNP-45364	MHAHI834-07	GU156517
5546	Urbanus proteus	Eudaminae	04-SRNP-1039	MHAHI836-07	GU156522
5547	Urbanus proteus	Eudaminae	04-SRNP-45324	MHAHI832-07	GU156501
5548	Urbanus proteus	Eudaminae	04-SRNP-45365	MHAHI833-07	GU156521
5549	Urbanus proteus	Eudaminae	04-SRNP-22001	MHAHI830-07	GU156569
5550	Urbanus proteus	Eudaminae	04-SRNP-22021	MHAHI831-07	GU156500
5551	Urbanus proteus	Eudaminae	03-SRNP-16641	MHAHI825-07	GU156617
5552	Urbanus proteus	Eudaminae	03-SRNP-16603	MHAHI827-07	GU156616
5553	Urbanus proteus	Eudaminae	03-SRNP-16627	MHAHI816-07	GU156714
5554	Urbanus proteus	Eudaminae	03-SRNP-16620	MHAHI819-07	GU156638
5555	Urbanus proteus	Eudaminae	03-SRNP-16891	MHAHI814-07	GU156712
5556	Urbanus proteus	Eudaminae	03-SRNP-16640	MHAHI815-07	GU156713
5557	Urbanus proteus	Eudaminae	07-SRNP-21374	MHAHL440-07	JF763355
5558	Urbanus proteus	Eudaminae	07-SRNP-22165	MHAHL444-07	JF763356
5559	Urbanus proteus	Eudaminae	07-SRNP-1159	MHAHL454-07	JF763359
5560	Urbanus proteus	Eudaminae	07-SRNP-3075	MHAHL457-07	JF763360
5561	Urbanus proteus	Eudaminae	07-SRNP-21822	MHAHL458-07	JF763361
5562	Urbanus proteus	Eudaminae	07-SRNP-45406	MHMXO804-08	JF763376
5563	Urbanus proteus	Eudaminae	07-SRNP-56883	MHMXO805-08	JF763375
5564	Urbanus proteus	Eudaminae	07-SRNP-57878	MHMXO809-08	JF763374
5565	Urbanus proteus	Eudaminae	07-SRNP-65274	MHMXO815-08	JF763369
5566	Urbanus proteus	Eudaminae	07-SRNP-65275	MHMXR790-08	JF763365
5567	Urbanus proteus	Eudaminae	08-SRNP-65195	MHMXW455-09	JF754371
5568	Urbanus proteus	Eudaminae	08-SRNP-55985	MHMXW456-09	JF754372
5569	Urbanus proteus	Eudaminae	08-SRNP-55979	MHMXW461-09	JF754375
5570	Urbanus proteus	Eudaminae	08-SRNP-65630	MHMXW462-09	JF754376
5571	Urbanus proteus	Eudaminae	09-SRNP-57115	MHMYE1460-09	GU653538
5572	Urbanus proteus	Eudaminae	09-SRNP-75601	MHMYG2417-10	HM885840
5573	Urbanus belliDHJ02	Eudaminae	04-SRNP-45491	MHAHD559-05	GU161969
5574	Urbanus belliDHJ02	Eudaminae	03-SRNP-12624.1	MHAHD551-05	GU161974
5575	Urbanus belliDHJ02	Eudaminae	03-SRNP-12634.1	MHAHD566-05	GU161972
5576	Urbanus belliDHJ02	Eudaminae	06-SRNP-47816	MHAHK061-07	JF761266
5577	Urbanus belliDHJ02	Eudaminae	07-SRNP-45071	MHMXK293-07	JF763317
5578	Urbanus belliDHJ02	Eudaminae	06-SRNP-30895	MHAHG548-06	GU151822
5579	Urbanus belliDHJ02	Eudaminae	95-SRNP-10572	MHAHD438-05	GU161964
5580	Urbanus belliDHJ02	Eudaminae	01-SRNP-2952	MHAHD437-05	GU161966
5581	Urbanus belliDHJ02	Eudaminae	01-SRNP-4475	MHAHD434-05	GU161965
5582	Urbanus belliDHJ02	Eudaminae	01-SRNP-487	MHAHC336-05	DQ293703
5583	Urbanus belliDHJ02	Eudaminae	06-SRNP-3621	MHAHI030-06	GU156387
5584	Urbanus belliDHJ02	Eudaminae	07-SRNP-40856	MHAHL443-07	JF763316
5585	Urbanus belliDHJ02	Eudaminae	06-SRNP-47814	MHAHK071-07	JF761269
5586	Urbanus belliDHJ02	Eudaminae	06-SRNP-30896	MHAHG573-06	GU151820
5587	Urbanus belliDHJ02	Eudaminae	06-SRNP-46174	MHAHJ743-07	JF753231
5588	Urbanus belliDHJ02	Eudaminae	06-SRNP-46171	MHAHJ899-07	JF753233
5589	Urbanus belliDHJ02	Eudaminae	06-SRNP-57638	MHAHI045-06	GU156385
5590	Urbanus belliDHJ02	Eudaminae	02-SRNP-1417	MHAHD432-05	GU161961
5591	Urbanus belliDHJ02	Eudaminae	04-SRNP-45586	MHAHD704-05	GU161968
5592	Urbanus belliDHJ02	Eudaminae	95-SRNP-6826	MHAHD409-05	GU161963
5593	Urbanus belliDHJ02	Eudaminae	02-SRNP-954	MHAHD431-05	GU161962
5594	Urbanus belliDHJ02	Eudaminae	04-SRNP-14877	MHAHC407-05	DQ293706
5595	Urbanus belliDHJ02	Eudaminae	04-SRNP-49377	MHAHC411-05	DQ293707
5596	Urbanus belliDHJ02	Eudaminae	04-SRNP-15474	MHAHC395-05	DQ293704
5597	Urbanus belliDHJ02	Eudaminae	04-SRNP-15145	MHAHC404-05	DQ293705
5598	Urbanus belliDHJ02	Eudaminae	04-SRNP-48357	MHAHC294-05	DQ293702
5599	Urbanus belliDHJ02	Eudaminae	04-SRNP-47954	MHAHC288-05	DQ293701

Tree Order	Species	Subfamily	ACG Sampleid	BOLD Processid	Genbank Accession
5600	Urbanus belliDHJ02	Eudaminae	04-SRNP-14569	MHAHC279-05	DQ293700
5601	Urbanus belliDHJ02	Eudaminae	02-SRNP-32172	MHAHC208-05	DQ293697
5602	Urbanus belliDHJ02	Eudaminae	02-SRNP-392	MHAHC217-05	DQ293698
5603	Urbanus belliDHJ02	Eudaminae	02-SRNP-166	MHAHC239-05	DQ293699
5604	Urbanus belliDHJ02	Eudaminae	04-SRNP-20968	MHAHC039-05	DQ293696
5605	Urbanus belliDHJ02	Eudaminae	04-SRNP-20876	MHAHC016-05	DQ293695
5606	Urbanus belliDHJ02	Eudaminae	05-SRNP-59447	MHAHF877-06	GU150970
5607	Urbanus belliDHJ02	Eudaminae	06-SRNP-47801	MHAHK064-07	JF761267
5608	Urbanus belliDHJ02	Eudaminae	02-SRNP-1026	MHAHD436-05	GU161967
5609	Urbanus belliDHJ02	Eudaminae	03-SRNP-12592	MHAHD567-05	GU161973
5610	Urbanus belliDHJ02	Eudaminae	06-SRNP-46184	MHAHJ898-07	JF753232
5611	Urbanus belliDHJ02	Eudaminae	07-SRNP-55283	MHMXK298-07	JF763318
5612	Urbanus belliDHJ02	Eudaminae	04-SRNP-45494	MHAHD562-05	GU161971
5613	Urbanus belliDHJ02	Eudaminae	05-SRNP-1832	MHAHF876-06	GU150969
5614	Urbanus belliDHJ02	Eudaminae	05-SRNP-47043	MHAHF884-06	GU150973
5615	Urbanus belliDHJ02	Eudaminae	05-SRNP-47231	MHAHF886-06	GU150974
5616	Urbanus belliDHJ02	Eudaminae	06-SRNP-2662	MHAHG569-06	GU151821
5617	Urbanus belliDHJ02	Eudaminae	05-SRNP-19630	MHAHG597-06	GU151818
5618	Urbanus belliDHJ02	Eudaminae	05-SRNP-59448	MHAHF879-06	GU150971
5619	Urbanus belliDHJ02	Eudaminae	05-SRNP-59444	MHAHF880-06	GU150972
5620	Urbanus belliDHJ02	Eudaminae	04-SRNP-45492	MHAHD550-05	GU161975
5621	Urbanus belliDHJ02	Eudaminae	03-SRNP-29774	MHAHD558-05	GU161970
5622	Urbanus belliDHJ02	Eudaminae	06-SRNP-2658	MHAHG599-06	GU151819
5623	Urbanus belliDHJ02	Eudaminae	06-SRNP-46172	MHAHI037-06	GU156386
5624	Urbanus belliDHJ02	Eudaminae	06-SRNP-46486	MHAHJ629-07	JF753227
5625	Urbanus belliDHJ02	Eudaminae	06-SRNP-46572	MHAHJ635-07	JF750898
5626	Urbanus belliDHJ02	Eudaminae	06-SRNP-46179	MHAHJ640-07	JF753228
5627	Urbanus belliDHJ02	Eudaminae	06-SRNP-46315	MHAHJ641-07	JF753229
5628	Urbanus belliDHJ02	Eudaminae	06-SRNP-6511	MHAHJ738-07	JF753230
5629	Urbanus belliDHJ02	Eudaminae	06-SRNP-46639	MHAHJ896-07	JF750897
5630	Urbanus belliDHJ02	Eudaminae	06-SRNP-46460	MHAHJ904-07	JF753234
5631	Urbanus belliDHJ02	Eudaminae	06-SRNP-47817	MHAHK065-07	JF761268
5632	Urbanus belliDHJ02	Eudaminae	07-SRNP-40676	MHMXK281-07	JF750902
5633	Urbanus belliDHJ02	Eudaminae	07-SRNP-30613	MHMXK286-07	JF750903
5634	Urbanus belliDHJ02	Eudaminae	07-SRNP-56829	MHMXK291-07	JF750904
5635	Urbanus belliDHJ02	Eudaminae	07-SRNP-56547	MHMXK296-07	JF750905
5636	Urbanus belliDHJ02	Eudaminae	07-SRNP-30615	MHMXK302-07	JF750899
5637	Urbanus belliDHJ02	Eudaminae	07-SRNP-20318	MHMXK311-07	JF750900
5638	Urbanus belliDHJ02	Eudaminae	06-SRNP-18276	MHMXK317-07	JF750901
5639	Urbanus belliDHJ02	Eudaminae	07-SRNP-57868	MHMXO821-08	JF750906
5640	Urbanus belliDHJ02	Eudaminae	08-SRNP-31826	MHMXY949-09	GU666573
5641	Urbanus viterboana	Eudaminae	03-SRNP-23514	CSRII587-05	DQ293844
5642	Urbanus viterboana	Eudaminae	01-SRNP-21478	MHAHC321-05	DQ293847
5643	Urbanus viterboana	Eudaminae	01-SRNP-21220	MHAHC333-05	DQ293848
5644	Urbanus viterboana	Eudaminae	99-SRNP-363	MHAHD404-05	GU162019
5645	Urbanus viterboana	Eudaminae	03-SRNP-23516	MHAHD563-05	GU162018
5646	Urbanus viterboana	Eudaminae	06-SRNP-35186	MHAHH537-06	GU155719
5647	Urbanus viterboana	Eudaminae	05-SRNP-35999	MHAHG594-06	GU151882
5648	Urbanus viterboana	Eudaminae	01-SRNP-21475	MHAHC334-05	DQ293849
5649	Urbanus viterboana	Eudaminae	01-SRNP-21228	MHAHC320-05	DQ293846
5650	Urbanus viterboana	Eudaminae	01-SRNP-21233	MHAHC319-05	DQ293845
5651	Urbanus viterboana	Eudaminae	01-SRNP-21223	MHAHC339-05	DQ293850
5652	Urbanus viterboana	Eudaminae	99-SRNP-366	MHAHD403-05	GU162020
5653	Urbanus viterboana	Eudaminae	05-SRNP-35553	MHAHF882-06	GU151027
5654	Urbanus viterboana	Eudaminae	06-SRNP-35190	MHAHI039-06	GU156715
5655	Urbanus viterboana	Eudaminae	07-SRNP-35463	MHMXK309-07	JF763396

Tree Order	Species	Subfamily	ACG Sampleid	BOLD Processid	Genbank Accession
5656	Urbanus viterboana	Eudaminae	07-SRNP-35538	MHMXN253-07	JF763395
5657	Urbanus viterboana	Eudaminae	08-SRNP-35218	MHMXW471-09	JF754385
5658	Urbanus belliDHJ03	Eudaminae	03-SRNP-12654.1	MHAHD555-05	GU161977
5659	Urbanus belliDHJ03	Eudaminae	06-SRNP-31264	MHAHG558-06	GU151824
5660	Urbanus belliDHJ03	Eudaminae	07-SRNP-1121	MHMXK279-07	JF763320
5661	Urbanus belliDHJ03	Eudaminae	07-SRNP-1122	MHMXK280-07	JF763319
5662	Urbanus belliDHJ03	Eudaminae	06-SRNP-65065	MHAHJ787-07	JF750907
5663	Urbanus belliDHJ03	Eudaminae	07-SRNP-33182	MHMXR784-08	JF750911
5664	Urbanus belliDHJ03	Eudaminae	08-SRNP-65993	MHMXY953-09	GU666567
5665	Urbanus belliDHJ03	Eudaminae	08-SRNP-66028	MHMXY944-09	GU666576
5666	Urbanus belliDHJ03	Eudaminae	07-SRNP-33183	MHMXR787-08	JF750912
5667	Urbanus belliDHJ03	Eudaminae	07-SRNP-33184	MHMXR782-08	JF750910
5668	Urbanus belliDHJ03	Eudaminae	07-SRNP-287	MHMXK303-07	JF750908
5669	Urbanus belliDHJ03	Eudaminae	07-SRNP-40761	MHMXK282-07	JF750909
5670	Urbanus belliDHJ03	Eudaminae	06-SRNP-46887	MHAHJ902-07	JF753237
5671	Urbanus belliDHJ03	Eudaminae	06-SRNP-7473	MHAHJ740-07	JF753236
5672	Urbanus belliDHJ03	Eudaminae	06-SRNP-46891	MHAHJ633-07	JF753235
5673	Urbanus belliDHJ03	Eudaminae	05-SRNP-7209	MHAHG593-06	GU151823
5674	Urbanus belliDHJ03	Eudaminae	05-SRNP-40545	MHAHF885-06	GU150976
5675	Urbanus belliDHJ03	Eudaminae	05-SRNP-41753	MHAHF878-06	GU150975
5676	Urbanus belliDHJ03	Eudaminae	03-SRNP-12629.1	MHAHD568-05	GU161982
5677	Urbanus belliDHJ03	Eudaminae	03-SRNP-10927	MHAHD565-05	GU161980
5678	Urbanus belliDHJ03	Eudaminae	03-SRNP-12109.1	MHAHD564-05	GU161981
5679	Urbanus belliDHJ03	Eudaminae	03-SRNP-10327	MHAHD554-05	GU161979
5680	Urbanus belliDHJ03	Eudaminae	03-SRNP-12655.1	MHAHD552-05	GU161983
5681	Urbanus belliDHJ03	Eudaminae	99-SRNP-5541	MHAHD423-05	GU161976
5682	Urbanus belliDHJ03	Eudaminae	01-SRNP-23227	MHAHD931-05	GU161978
5683	Urbanus belliDHJ03	Eudaminae	04-SRNP-42794	MHAHC700-05	DQ293712
5684	Urbanus belliDHJ03	Eudaminae	01-SRNP-22184	MHAHC329-05	DQ293711
5685	Urbanus belliDHJ03	Eudaminae	01-SRNP-23228	MHAHC345-05	DQ293708
5686	Urbanus belliDHJ03	Eudaminae	01-SRNP-2104	MHAHC323-05	DQ293710
5687	Urbanus belliDHJ03	Eudaminae	02-SRNP-7163	MHAHC237-05	DQ293709
5688	Urbanus belliDHJ03	Eudaminae	05-SRNP-42394	MHAHF889-06	GU150977
5689	Urbanus belliDHJ03	Eudaminae	06-SRNP-43129	MHAHI467-06	GU156388
5690	Urbanus belliDHJ03	Eudaminae	09-SRNP-57089	MHMYE1572-09	HM391132
5691	Urbanus belliDHJ01	Eudaminae	04-SRNP-49713	MHAHC702-05	DQ293689
5692	Urbanus belliDHJ01	Eudaminae	04-SRNP-49711	MHAHC703-05	DQ293690
5693	Urbanus belliDHJ01	Eudaminae	05-SRNP-49623	MHAHG329-06	GU151811
5694	Urbanus belliDHJ01	Eudaminae	01-SRNP-22185	MHAHD440-05	GU161945
5695	Urbanus belliDHJ01	Eudaminae	05-SRNP-43429	MHAHG590-06	GU151806
5696	Urbanus belliDHJ01	Eudaminae	06-SRNP-31091	MHAHG604-06	GU151810
5697	Urbanus belliDHJ01	Eudaminae	06-SRNP-46459	MHAHJ900-07	JF753224
5698	Urbanus belliDHJ01	Eudaminae	06-SRNP-19690	MHAHK063-07	JF761261
5699	Urbanus belliDHJ01	Eudaminae	07-SRNP-1053	MHMXK277-07	JF763312
5700	Urbanus belliDHJ01	Eudaminae	06-SRNP-47304	MHAHJ631-07	JF753214
5701	Urbanus belliDHJ01	Eudaminae	07-SRNP-23883	MHMXT186-08	JF763303
5702	Urbanus belliDHJ01	Eudaminae	06-SRNP-46173	MHAHI038-06	GU156382
5703	Urbanus belliDHJ01	Eudaminae	06-SRNP-48039	MHAHK068-07	JF761263
5704	Urbanus belliDHJ01	Eudaminae	98-SRNP-4170	MHAHD418-05	GU161937
5705	Urbanus belliDHJ01	Eudaminae	05-SRNP-47230	MHAHF887-06	GU150966
5706	Urbanus belliDHJ01	Eudaminae	05-SRNP-42614	MHAHF888-06	GU150964
5707	Urbanus belliDHJ01	Eudaminae	07-SRNP-45068	MHMXK292-07	JF763307
5708	Urbanus belliDHJ01	Eudaminae	06-SRNP-47295	MHAHJ630-07	JF753213
5709	Urbanus belliDHJ01	Eudaminae	06-SRNP-31265	MHAHH547-06	GU155697
5710	Urbanus belliDHJ01	Eudaminae	05-SRNP-60977	MHAHG596-06	GU151807
5711	Urbanus belliDHJ01	Eudaminae	05-SRNP-42873	MHAHG591-06	GU151803

Tree Order	Species	Subfamily	ACG Sampleid	BOLD Processid	Genbank Accession
5712	Urbanus belliDHJ01	Eudaminae	06-SRNP-40907	MHAHG562-06	GU151817
5713	Urbanus belliDHJ01	Eudaminae	07-SRNP-20462	MHMXR786-08	JF763304
5714	Urbanus belliDHJ01	Eudaminae	96-SRNP-11569	MHAHD414-05	GU161933
5715	Urbanus belliDHJ01	Eudaminae	07-SRNP-41187	MHAHL453-07	JF763302
5716	Urbanus belliDHJ01	Eudaminae	08-SRNP-1439	MHMXW458-09	JF750891
5717	Urbanus belliDHJ01	Eudaminae	08-SRNP-65153	MHMXW457-09	JF750892
5718	Urbanus belliDHJ01	Eudaminae	07-SRNP-65582	MHMXR785-08	JF750894
5719	Urbanus belliDHJ01	Eudaminae	07-SRNP-33232	MHMXR783-08	JF750893
5720	Urbanus belliDHJ01	Eudaminae	07-SRNP-1119	MHMXO819-08	JF750896
5721	Urbanus belliDHJ01	Eudaminae	07-SRNP-42421	MHMXO808-08	JF750895
5722	Urbanus belliDHJ01	Eudaminae	07-SRNP-42423	MHMXO807-08	JF763306
5723	Urbanus belliDHJ01	Eudaminae	07-SRNP-31148	MHAHL452-07	JF763301
5724	Urbanus belliDHJ01	Eudaminae	07-SRNP-31150	MHAHL451-07	JF763300
5725	Urbanus belliDHJ01	Eudaminae	07-SRNP-20874	MHAHL446-07	JF763299
5726	Urbanus belliDHJ01	Eudaminae	07-SRNP-31149	MHAHL442-07	JF763298
5727	Urbanus belliDHJ01	Eudaminae	07-SRNP-20875	MHAHL441-07	JF763297
5728	Urbanus belliDHJ01	Eudaminae	07-SRNP-30807	MHMXK304-07	JF763314
5729	Urbanus belliDHJ01	Eudaminae	07-SRNP-30614	MHMXK301-07	JF763315
5730	Urbanus belliDHJ01	Eudaminae	07-SRNP-45090	MHMXK290-07	JF763308
5731	Urbanus belliDHJ01	Eudaminae	07-SRNP-45088	MHMXK285-07	JF763309
5732	Urbanus belliDHJ01	Eudaminae	07-SRNP-40762	MHMXK283-07	JF763310
5733	Urbanus belliDHJ01	Eudaminae	07-SRNP-30617	MHMXK278-07	JF763311
5734	Urbanus belliDHJ01	Eudaminae	06-SRNP-60091	MHAHK070-07	JF761265
5735	Urbanus belliDHJ01	Eudaminae	06-SRNP-47955	MHAHK069-07	JF761264
5736	Urbanus belliDHJ01	Eudaminae	06-SRNP-59197	MHAHK066-07	JF761262
5737	Urbanus belliDHJ01	Eudaminae	06-SRNP-46570	MHAHJ903-07	JF753226
5738	Urbanus belliDHJ01	Eudaminae	06-SRNP-46180	MHAHJ901-07	JF753225
5739	Urbanus belliDHJ01	Eudaminae	06-SRNP-46176	MHAHJ897-07	JF753223
5740	Urbanus belliDHJ01	Eudaminae	06-SRNP-47233	MHAHJ895-07	JF753222
5741	Urbanus belliDHJ01	Eudaminae	06-SRNP-7474	MHAHJ745-07	JF753221
5742	Urbanus belliDHJ01	Eudaminae	06-SRNP-6164	MHAHJ737-07	JF753220
5743	Urbanus belliDHJ01	Eudaminae	06-SRNP-46703	MHAHJ642-07	JF753219
5744	Urbanus belliDHJ01	Eudaminae	06-SRNP-7378	MHAHJ639-07	JF753218
5745	Urbanus belliDHJ01	Eudaminae	06-SRNP-46183	MHAHJ637-07	JF753217
5746	Urbanus belliDHJ01	Eudaminae	06-SRNP-46487	MHAHJ636-07	JF753216
5747	Urbanus belliDHJ01	Eudaminae	06-SRNP-46333	MHAHJ634-07	JF753215
5748	Urbanus belliDHJ01	Eudaminae	06-SRNP-43281	MHAHI468-06	GU156381
5749	Urbanus belliDHJ01	Eudaminae	06-SRNP-32923	MHAHI466-06	GU156380
5750	Urbanus belliDHJ01	Eudaminae	06-SRNP-46181	MHAHI035-06	GU156383
5751	Urbanus belliDHJ01	Eudaminae	06-SRNP-5512	MHAHI029-06	GU156384
5752	Urbanus belliDHJ01	Eudaminae	06-SRNP-30699	MHAHG605-06	GU151809
5753	Urbanus belliDHJ01	Eudaminae	06-SRNP-3004	MHAHG602-06	GU151804
5754	Urbanus belliDHJ01	Eudaminae	06-SRNP-2656	MHAHG600-06	GU151808
5755	Urbanus belliDHJ01	Eudaminae	05-SRNP-43001	MHAHG587-06	GU151805
5756	Urbanus belliDHJ01	Eudaminae	06-SRNP-40981	MHAHG549-06	GU151816
5757	Urbanus belliDHJ01	Eudaminae	06-SRNP-2074	MHAHG547-06	GU151815
5758	Urbanus belliDHJ01	Eudaminae	06-SRNP-2199	MHAHG544-06	GU151813
5759	Urbanus belliDHJ01	Eudaminae	06-SRNP-30737	MHAHG542-06	GU151814
5760	Urbanus belliDHJ01	Eudaminae	05-SRNP-40888	MHAHF927-06	GU150968
5761	Urbanus belliDHJ01	Eudaminae	05-SRNP-45196	MHAHF919-06	GU150958
5762	Urbanus belliDHJ01	Eudaminae	05-SRNP-42399	MHAHF900-06	GU150967
5763	Urbanus belliDHJ01	Eudaminae	05-SRNP-40898	MHAHF897-06	GU150959
5764	Urbanus belliDHJ01	Eudaminae	05-SRNP-47058	MHAHF892-06	GU150960
5765	Urbanus belliDHJ01	Eudaminae	05-SRNP-42700	MHAHF891-06	GU150965
5766	Urbanus belliDHJ01	Eudaminae	05-SRNP-47229	MHAHF890-06	GU150963
5767	Urbanus belliDHJ01	Eudaminae	05-SRNP-20694	MHAHF883-06	GU150961

Tree Order	Species	Subfamily	ACG Sampleid	BOLD Processid	Genbank Accession
5768	Urbanus belliDHJ01	Eudaminae	05-SRNP-47232	MHAHF881-06	GU150962
5769	Urbanus belliDHJ01	Eudaminae	04-SRNP-4302	MHAHE156-05	GU150133
5770	Urbanus belliDHJ01	Eudaminae	04-SRNP-21913	MHAHD576-05	GU161932
5771	Urbanus belliDHJ01	Eudaminae	03-SRNP-12633.1	MHAHD575-05	GU161958
5772	Urbanus belliDHJ01	Eudaminae	03-SRNP-12657.1	MHAHD574-05	GU161951
5773	Urbanus belliDHJ01	Eudaminae	03-SRNP-12627.1	MHAHD573-05	GU161947
5774	Urbanus belliDHJ01	Eudaminae	03-SRNP-12947.1	MHAHD572-05	GU161957
5775	Urbanus belliDHJ01	Eudaminae	03-SRNP-11566	MHAHD571-05	GU161931
5776	Urbanus belliDHJ01	Eudaminae	03-SRNP-12345.1	MHAHD570-05	GU161956
5777	Urbanus belliDHJ01	Eudaminae	03-SRNP-10529	MHAHD569-05	GU161948
5778	Urbanus belliDHJ01	Eudaminae	03-SRNP-12298.1	MHAHD560-05	GU161954
5779	Urbanus belliDHJ01	Eudaminae	03-SRNP-11211	MHAHD557-05	GU161949
5780	Urbanus belliDHJ01	Eudaminae	04-SRNP-21912	MHAHD556-05	GU161955
5781	Urbanus belliDHJ01	Eudaminae	01-SRNP-23117	MHAHD439-05	GU161946
5782	Urbanus belliDHJ01	Eudaminae	02-SRNP-7107	MHAHD429-05	GU161942
5783	Urbanus belliDHJ01	Eudaminae	01-SRNP-1028	MHAHD428-05	GU161943
5784	Urbanus belliDHJ01	Eudaminae	01-SRNP-22505	MHAHD427-05	GU161944
5785	Urbanus belliDHJ01	Eudaminae	00-SRNP-22025	MHAHD426-05	GU161941
5786	Urbanus belliDHJ01	Eudaminae	99-SRNP-2440	MHAHD425-05	GU161938
5787	Urbanus belliDHJ01	Eudaminae	02-SRNP-590	MHAHD424-05	GU161940
5788	Urbanus belliDHJ01	Eudaminae	02-SRNP-7734	MHAHD422-05	GU161939
5789	Urbanus belliDHJ01	Eudaminae	01-SRNP-5700	MHAHD421-05	GU161953
5790	Urbanus belliDHJ01	Eudaminae	00-SRNP-1534	MHAHD417-05	GU161930
5791	Urbanus belliDHJ01	Eudaminae	00-SRNP-2200	MHAHD416-05	GU161936
5792	Urbanus belliDHJ01	Eudaminae	00-SRNP-22026	MHAHD413-05	GU161934
5793	Urbanus belliDHJ01	Eudaminae	02-SRNP-12992	MHAHD410-05	GU161952
5794	Urbanus belliDHJ01	Eudaminae	04-SRNP-49712	MHAHC707-05	DQ293694
5795	Urbanus belliDHJ01	Eudaminae	04-SRNP-49960	MHAHC706-05	DQ293693
5796	Urbanus belliDHJ01	Eudaminae	04-SRNP-49714	MHAHC705-05	DQ293692
5797	Urbanus belliDHJ01	Eudaminae	04-SRNP-49963	MHAHC704-05	DQ293691
5798	Urbanus belliDHJ01	Eudaminae	04-SRNP-41942	MHAHC699-05	DQ293688
5799	Urbanus belliDHJ01	Eudaminae	04-SRNP-15157	MHAHC410-05	DQ293687
5800	Urbanus belliDHJ01	Eudaminae	04-SRNP-15160	MHAHC409-05	DQ293686
5801	Urbanus belliDHJ01	Eudaminae	04-SRNP-15153	MHAHC406-05	DQ293685
5802	Urbanus belliDHJ01	Eudaminae	04-SRNP-15679	MHAHC405-05	DQ293684
5803	Urbanus belliDHJ01	Eudaminae	04-SRNP-15149	MHAHC403-05	DQ293683
5804	Urbanus belliDHJ01	Eudaminae	04-SRNP-15189	MHAHC402-05	DQ293682
5805	Urbanus belliDHJ01	Eudaminae	04-SRNP-15161	MHAHC401-05	DQ293681
5806	Urbanus belliDHJ01	Eudaminae	04-SRNP-15155	MHAHC399-05	DQ293680
5807	Urbanus belliDHJ01	Eudaminae	04-SRNP-15147	MHAHC397-05	DQ293679
5808	Urbanus belliDHJ01	Eudaminae	04-SRNP-15146	MHAHC396-05	DQ293678
5809	Urbanus belliDHJ01	Eudaminae	04-SRNP-15154	MHAHC394-05	DQ293677
5810	Urbanus belliDHJ01	Eudaminae	04-SRNP-15137	MHAHC392-05	DQ293676
5811	Urbanus belliDHJ01	Eudaminae	04-SRNP-15162	MHAHC391-05	DQ293675
5812	Urbanus belliDHJ01	Eudaminae	04-SRNP-15150	MHAHC390-05	DQ293674
5813	Urbanus belliDHJ01	Eudaminae	04-SRNP-15674	MHAHC389-05	DQ293673
5814	Urbanus belliDHJ01	Eudaminae	04-SRNP-15680	MHAHC388-05	DQ293672
5815	Urbanus belliDHJ01	Eudaminae	04-SRNP-15677	MHAHC387-05	DQ293671
5816	Urbanus belliDHJ01	Eudaminae	04-SRNP-48641	MHAHC210-05	DQ293643
5817	Urbanus belliDHJ01	Eudaminae	04-SRNP-48028	MHAHC306-05	DQ293664
5818	Urbanus belliDHJ01	Eudaminae	04-SRNP-48033	MHAHC298-05	DQ293659
5819	Urbanus belliDHJ01	Eudaminae	04-SRNP-47563	MHAHC290-05	DQ293656
5820	Urbanus belliDHJ01	Eudaminae	04-SRNP-14307	MHAHC286-05	DQ293654
5821	Urbanus belliDHJ01	Eudaminae	04-SRNP-14390	MHAHC284-05	DQ293653
5822	Urbanus belliDHJ01	Eudaminae	04-SRNP-14389	MHAHC310-05	DQ293666
5823	Urbanus belliDHJ01	Eudaminae	04-SRNP-41681	MHAHC307-05	DQ293665

Tree Order	Species	Subfamily	ACG Sampleid	BOLD Processid	Genbank Accession
5824	Urbanus belliDHJ01	Eudaminae	04-SRNP-41680	MHAHC299-05	DQ293660
5825	Urbanus belliDHJ01	Eudaminae	04-SRNP-48639	MHAHC291-05	DQ293657
5826	Urbanus belliDHJ01	Eudaminae	04-SRNP-47956	MHAHC283-05	DQ293652
5827	Urbanus belliDHJ01	Eudaminae	04-SRNP-47824	MHAHC292-05	DQ293658
5828	Urbanus belliDHJ01	Eudaminae	04-SRNP-14108	MHAHC300-05	DQ293661
5829	Urbanus belliDHJ01	Eudaminae	01-SRNP-5310	MHAHC349-05	DQ293670
5830	Urbanus belliDHJ01	Eudaminae	01-SRNP-5311	MHAHC348-05	DQ293669
5831	Urbanus belliDHJ01	Eudaminae	01-SRNP-3745	MHAHC344-05	DQ293668
5832	Urbanus belliDHJ01	Eudaminae	01-SRNP-3404	MHAHC342-05	DQ293632
5833	Urbanus belliDHJ01	Eudaminae	01-SRNP-23060	MHAHC322-05	DQ293667
5834	Urbanus belliDHJ01	Eudaminae	02-SRNP-13670	MHAHC228-05	DQ293646
5835	Urbanus belliDHJ01	Eudaminae	02-SRNP-2246	MHAHC238-05	DQ293648
5836	Urbanus belliDHJ01	Eudaminae	02-SRNP-28991	MHAHC240-05	DQ293649
5837	Urbanus belliDHJ01	Eudaminae	02-SRNP-32169	MHAHC232-05	DQ293647
5838	Urbanus belliDHJ01	Eudaminae	02-SRNP-11533	MHAHC224-05	DQ293645
5839	Urbanus belliDHJ01	Eudaminae	02-SRNP-13111	MHAHC216-05	DQ293644
5840	Urbanus belliDHJ01	Eudaminae	02-SRNP-1028	MHAHC200-05	DQ293642
5841	Urbanus belliDHJ01	Eudaminae	02-SRNP-13671	MHAHC192-05	DQ293641
5842	Urbanus belliDHJ01	Eudaminae	02-SRNP-163	MHAHC241-05	DQ293650
5843	Urbanus belliDHJ01	Eudaminae	04-SRNP-33067	MHAHC078-05	DQ293640
5844	Urbanus belliDHJ01	Eudaminae	04-SRNP-21152	MHAHC070-05	DQ293639
5845	Urbanus belliDHJ01	Eudaminae	04-SRNP-31053	MHAHC054-05	DQ293637
5846	Urbanus belliDHJ01	Eudaminae	04-SRNP-22913	MHAHC063-05	DQ293638
5847	Urbanus belliDHJ01	Eudaminae	04-SRNP-45034	MHAHC047-05	DQ293636
5848	Urbanus belliDHJ01	Eudaminae	04-SRNP-20680	MHAHC031-05	DQ293635
5849	Urbanus belliDHJ01	Eudaminae	04-SRNP-20872	MHAHC024-05	DQ293634
5850	Urbanus belliDHJ01	Eudaminae	04-SRNP-20925	MHAHC008-05	DQ293633
5851	Urbanus belliDHJ01	Eudaminae	06-SRNP-59672	MHAHK062-07	JF761260
5852	Urbanus belliDHJ01	Eudaminae	04-SRNP-47963	MHAHC304-05	DQ293663
5853	Urbanus belliDHJ01	Eudaminae	04-SRNP-47882	MHAHC303-05	DQ293662
5854	Urbanus belliDHJ01	Eudaminae	04-SRNP-47558	MHAHC287-05	DQ293655
5855	Urbanus belliDHJ01	Eudaminae	04-SRNP-14305	MHAHC282-05	DQ293651
5856	Urbanus belliDHJ01	Eudaminae	06-SRNP-40146	MHAHG328-06	GU151812
5857	Urbanus belliDHJ01	Eudaminae	07-SRNP-20167	MHMXK312-07	JF763313
5858	Urbanus belliDHJ01	Eudaminae	01-SRNP-3403	MHAHD932-05	GU161950
5859	Urbanus belliDHJ01	Eudaminae	02-SRNP-21477	MHAHD430-05	GU161935
5860	Urbanus belliDHJ01	Eudaminae	03-SRNP-12581.1	MHAHD553-05	GU161959
5861	Urbanus belliDHJ01	Eudaminae	03-SRNP-12948.1	MHAHD561-05	GU161960
5862	Urbanus belliDHJ01	Eudaminae	07-SRNP-41928	MHMXO818-08	JF763305
5863	Urbanus belliDHJ01	Eudaminae	08-SRNP-4727	MHMXX471-09	JF778578
5864	Urbanus belliDHJ01	Eudaminae	08-SRNP-65935	MHMXY943-09	GU666575
5865	Urbanus belliDHJ01	Eudaminae	08-SRNP-5848	MHMXY945-09	GU666577
5866	Urbanus belliDHJ01	Eudaminae	08-SRNP-23800	MHMXY946-09	GU666578
5867	Urbanus belliDHJ01	Eudaminae	08-SRNP-24278	MHMYB133-09	GU649690
5868	Urbanus belliDHJ01	Eudaminae	09-SRNP-69873	MHMYG2418-10	HM885841
5869	Astraptes tucuti	Eudaminae	98-SRNP-15995	CSCR037-04	DQ291888
5870	Astraptes tucuti	Eudaminae	01-SRNP-21502	CSCR039-04	DQ291889
5871	Astraptes tucuti	Eudaminae	01-SRNP-21504	CSCR040-04	DQ291890
5872	Astraptes tucuti	Eudaminae	04-SRNP-3376	MHAHE028-05	GU149430
5873	Astraptes tucuti	Eudaminae	05-SRNP-4479	MHAHF238-06	GU150254
5874	Astraptes tucuti	Eudaminae	05-SRNP-5094	MHAHF300-06	GU150255
5875	Astraptes tucuti	Eudaminae	07-SRNP-42005	MHMXP152-08	JF761640
5876	Astraptes tucuti	Eudaminae	04-SRNP-35322	MHAHH243-06	GU155110
5877	Astraptes tucuti	Eudaminae	04-SRNP-35430	MHAHH241-06	GU155111
5878	Astraptes tucuti	Eudaminae	04-SRNP-35323	MHAHH239-06	GU155112
5879	Astraptes tucuti	Eudaminae	02-SRNP-3446	CSRII092-04	DQ291891

Tree Order	Species	Subfamily	ACG Sampleid	BOLD Processid	Genbank Accession
5880	Astraptes tucuti	Eudaminae	07-SRNP-1173	MHMXN289-07	JF761644
5881	Astraptes tucuti	Eudaminae	06-SRNP-41042	MHAHG657-06	GU151177
5882	Astraptes tucuti	Eudaminae	06-SRNP-2459	MHAHG656-06	GU151178
5883	Astraptes tucuti	Eudaminae	05-SRNP-4645	MHAHF237-06	GU150253
5884	Astraptes tucuti	Eudaminae	04-SRNP-2718	MHAHE027-05	GU149429
5885	Astraptes tucuti	Eudaminae	04-SRNP-60520	MHAHE026-05	GU149427
5886	Astraptes tucuti	Eudaminae	04-SRNP-60491	MHAHE025-05	GU149428
5887	Astraptes tucuti	Eudaminae	07-SRNP-2625	MHMXN288-07	JF761645
5888	Astraptes tucuti	Eudaminae	07-SRNP-1968	MHMXN292-07	JF761643
5889	Astraptes tucuti	Eudaminae	07-SRNP-35969	MHMXN293-07	JF761642
5890	Astraptes tucuti	Eudaminae	07-SRNP-2349	MHMXN294-07	JF761641
5891	Astraptes tucuti	Eudaminae	09-SRNP-56674	MHMYG2385-10	HM885809
5892	Urbanus pronta	Eudaminae	04-SRNP-46061	MHAHC086-05	DQ293771
5893	Urbanus pronta	Eudaminae	04-SRNP-46058	MHAHC312-05	DQ293779
5894	Urbanus pronta	Eudaminae	04-SRNP-46056	MHAHC055-05	DQ293770
5895	Urbanus pronta	Eudaminae	04-SRNP-46057	MHAHC038-05	DQ293769
5896	Urbanus pronta	Eudaminae	08-SRNP-36361	MHMXX470-09	JF778581
5897	Urbanus pronta	Eudaminae	06-SRNP-9393	MHAHJ788-07	JF753243
5898	Urbanus pronta	Eudaminae	06-SRNP-45648	MHAHI033-06	GU156476
5899	Urbanus pronta	Eudaminae	06-SRNP-45649	MHAHI026-06	GU156477
5900	Urbanus pronta	Eudaminae	06-SRNP-3187	MHAHG603-06	GU151871
5901	Urbanus pronta	Eudaminae	01-SRNP-3205	MHAHC347-05	DQ293781
5902	Urbanus pronta	Eudaminae	01-SRNP-22501	MHAHC346-05	DQ293780
5903	Urbanus pronta	Eudaminae	02-SRNP-17474	MHAHC231-05	DQ293778
5904	Urbanus pronta	Eudaminae	02-SRNP-18620	MHAHC223-05	DQ293777
5905	Urbanus pronta	Eudaminae	02-SRNP-27305	MHAHC215-05	DQ293776
5906	Urbanus pronta	Eudaminae	02-SRNP-27308	MHAHC207-05	DQ293774
5907	Urbanus pronta	Eudaminae	02-SRNP-1605	MHAHC212-05	DQ293775
5908	Urbanus pronta	Eudaminae	02-SRNP-17477	MHAHC191-05	DQ293772
5909	Urbanus pronta	Eudaminae	02-SRNP-17476	MHAHC199-05	DQ293773
5910	Urbanus pronta	Eudaminae	06-SRNP-5958	MHAHI028-06	GU156475
5911	Urbanus pronta	Eudaminae	06-SRNP-23251	MHAHJ744-07	JF753242
5912	Urbanus pronta	Eudaminae	09-SRNP-58058	MHMYG2416-10	HM885839
5913	Urbanus pronta	Eudaminae	09-SRNP-58053	MHMYG2021-10	HM885423
5914	Astraptes apastus	Eudaminae	03-SRNP-20746	CSCR308-04	DQ291856
5915	Astraptes apastus	Eudaminae	07-SRNP-40564	MHMXK102-07	JF761468
5916	Astraptes brevicauda	Eudaminae	03-SRNP-11830	MHAHE108-05	GU149418
5917	Astraptes brevicauda	Eudaminae	08-SRNP-1126	MHMXW554-09	JF753663
5918	Astraptes brevicauda	Eudaminae	05-SRNP-40306	MHAHF243-06	GU150207
5919	Astraptes brevicauda	Eudaminae	01-SRNP-3278	CSCR024-04	DQ291859
5920	Astraptes brevicauda	Eudaminae	01-SRNP-5574	CSCR025-04	DQ291860
5921	Astraptes brevicauda	Eudaminae	05-SRNP-41750	MHAHF299-06	GU150209
5922	Astraptes brevicauda	Eudaminae	04-SRNP-23822	MHAHE042-05	GU149413
5923	Astraptes brevicauda	Eudaminae	04-SRNP-41627	MHAHE043-05	GU149412
5924	Astraptes brevicauda	Eudaminae	04-SRNP-42195	MHAHE044-05	GU149414
5925	Astraptes brevicauda	Eudaminae	04-SRNP-4716	MHAHE045-05	GU149411
5926	Astraptes brevicauda	Eudaminae	03-SRNP-11273	MHAHE107-05	GU149415
5927	Astraptes brevicauda	Eudaminae	03-SRNP-6444	MHAHE109-05	GU149416
5928	Astraptes brevicauda	Eudaminae	03-SRNP-6247	MHAHE110-05	GU149417
5929	Astraptes brevicauda	Eudaminae	05-SRNP-390	MHAHF244-06	GU150206
5930	Astraptes brevicauda	Eudaminae	05-SRNP-41809	MHAHF298-06	GU150208
5931	Astraptes brevicauda	Eudaminae	06-SRNP-31309	MHAHG658-06	GU151090
5932	Astraptes brevicauda	Eudaminae	06-SRNP-3274	MHAHH564-06	GU154918
5933	Astraptes brevicauda	Eudaminae	07-SRNP-1174	MHMXK095-07	JF761471
5934	Astraptes brevicauda	Eudaminae	05-SRNP-7334	MHAHL109-07	JF761469
5935	Astraptes brevicauda	Eudaminae	07-SRNP-66036	MHMXR798-08	JF761470

Tree Order	Species	Subfamily	ACG Sampleid	BOLD Processid	Genbank Accession
5936	Astraptes brevicauda	Eudaminae	08-SRNP-1013	MHMXW555-09	JF753664
5937	Astraptes brevicauda	Eudaminae	08-SRNP-5071	MHMXX485-09	JF777601
5938	Astraptes anaphus annettaDHJ01	Eudaminae	04-SRNP-686	MHAHH759-06	GU154896
5939	Astraptes anaphus annettaDHJ01	Eudaminae	04-SRNP-1067	MHAHE158-05	GU149408
5940	Astraptes anaphus annettaDHJ01	Eudaminae	01-SRNP-16954	MHAHH761-06	GU154897
5941	Astraptes anaphus annettaDHJ01	Eudaminae	02-SRNP-28707	MHAHH817-06	GU154895
5942	Astraptes anaphus annettaDHJ01	Eudaminae	07-SRNP-33566	MHMXT216-08	JF761456
5943	Astraptes anaphus annettaDHJ03	Eudaminae	97-SRNP-417	MHMXG311-07	JF760296
5944	Astraptes anaphus annettaDHJ03	Eudaminae	97-SRNP-451	MHMXG326-07	JF760301
5945	Astraptes anaphus annettaDHJ03	Eudaminae	02-SRNP-28432	MHMXG303-07	JF760304
5946	Astraptes anaphus annettaDHJ03	Eudaminae	00-SRNP-20224	CSCR021-04	DQ291855
5947	Astraptes anaphus annettaDHJ03	Eudaminae	97-SRNP-492	MHMXG284-07	JF760303
5948	Astraptes anaphus annettaDHJ03	Eudaminae	93-SRNP-4847	MHMXG292-07	JF760295
5949	Astraptes anaphus annettaDHJ03	Eudaminae	03-SRNP-26790	MHAHH754-06	GU154906
5950	Astraptes anaphus annettaDHJ03	Eudaminae	97-SRNP-457	MHMXG286-07	JF760299
5951	Astraptes anaphus annettaDHJ03	Eudaminae	93-SRNP-7914	MHMXG302-07	JF760306
5952	Astraptes anaphus annettaDHJ03	Eudaminae	97-SRNP-462	MHAHH772-06	GU154917
5953	Astraptes anaphus annettaDHJ03	Eudaminae	02-SRNP-5751	MHAHH769-06	GU154915
5954	Astraptes anaphus annettaDHJ03	Eudaminae	02-SRNP-5101	MHAHH764-06	GU154911
5955	Astraptes anaphus annettaDHJ03	Eudaminae	02-SRNP-5100	MHAHH763-06	GU154910
5956	Astraptes anaphus annettaDHJ03	Eudaminae	02-SRNP-29489	MHAHH816-06	GU154904
5957	Astraptes anaphus annettaDHJ03	Eudaminae	94-SRNP-7681	MHMXG296-07	JF760292
5958	Astraptes anaphus annettaDHJ03	Eudaminae	06-SRNP-46316	MHAHJ646-07	JF752384
5959	Astraptes anaphus annettaDHJ03	Eudaminae	07-SRNP-20031	MHMXK120-07	JF761467
5960	Astraptes anaphus annettaDHJ03	Eudaminae	07-SRNP-20299	MHMXK123-07	JF761465
5961	Astraptes anaphus annettaDHJ03	Eudaminae	07-SRNP-20217	MHMXK125-07	JF761463
5962	Astraptes anaphus annettaDHJ03	Eudaminae	07-SRNP-23872	MHMXR899-08	JF761461
5963	Astraptes anaphus annettaDHJ03	Eudaminae	94-SRNP-7840	MHMXG317-07	JF760291
5964	Astraptes anaphus annettaDHJ03	Eudaminae	00-SRNP-20296	MHMXG305-07	JF760302
5965	Astraptes anaphus annettaDHJ03	Eudaminae	06-SRNP-3246	MHAHI158-06	GU155770
5966	Astraptes anaphus annettaDHJ03	Eudaminae	00-SRNP-20174	CSCR020-04	DQ291854
5967	Astraptes anaphus annettaDHJ03	Eudaminae	94-SRNP-7838	MHMXG320-07	JF760308
5968	Astraptes anaphus annettaDHJ03	Eudaminae	93-SRNP-6735	MHMXG327-07	JF760298
5969	Astraptes anaphus annettaDHJ03	Eudaminae	92-SRNP-4964	MHMXG323-07	JF760305
5970	Astraptes anaphus annettaDHJ03	Eudaminae	97-SRNP-10083	MHMXG315-07	JF760293
5971	Astraptes anaphus annettaDHJ03	Eudaminae	92-SRNP-4961	MHMXG306-07	JF760300
5972	Astraptes anaphus annettaDHJ03	Eudaminae	07-SRNP-20148	MHMXK121-07	JF761466
5973	Astraptes anaphus annettaDHJ03	Eudaminae	02-SRNP-28615	MHAHI1057-07	GU155771
5974	Astraptes anaphus annettaDHJ03	Eudaminae	05-SRNP-2000	MHAHF519-06	GU150204
5975	Astraptes anaphus annettaDHJ03	Eudaminae	00-SRNP-19298	MHMXG309-07	JF760309
5976	Astraptes anaphus annettaDHJ03	Eudaminae	00-SRNP-20267	MHMXG319-07	JF760289
5977	Astraptes anaphus annettaDHJ03	Eudaminae	97-SRNP-11538	MHMXG333-07	JF760294
5978	Astraptes anaphus annettaDHJ03	Eudaminae	07-SRNP-20264	MHMXK124-07	JF761464
5979	Astraptes anaphus annettaDHJ03	Eudaminae	93-SRNP-8375	MHAHI1061-07	GU155773
5980	Astraptes anaphus annettaDHJ03	Eudaminae	00-SRNP-17721	MHMXG288-07	JF760297
5981	Astraptes anaphus annettaDHJ03	Eudaminae	02-SRNP-28828	MHAHI1058-07	GU155772
5982	Astraptes anaphus annettaDHJ03	Eudaminae	00-SRNP-6012	MHAHI1059-07	GU155774
5983	Astraptes anaphus annettaDHJ03	Eudaminae	01-SRNP-17042	MHAHH819-06	GU154905
5984	Astraptes anaphus annettaDHJ03	Eudaminae	97-SRNP-9242	MHAHH820-06	GU154903
5985	Astraptes anaphus annettaDHJ03	Eudaminae	00-SRNP-20270	MHAHH770-06	GU154916
5986	Astraptes anaphus annettaDHJ03	Eudaminae	02-SRNP-30063	MHAHH768-06	GU154914
5987	Astraptes anaphus annettaDHJ03	Eudaminae	02-SRNP-28709	MHAHH766-06	GU154913
5988	Astraptes anaphus annettaDHJ03	Eudaminae	02-SRNP-28395	MHAHH765-06	GU154912
5989	Astraptes anaphus annettaDHJ03	Eudaminae	97-SRNP-2276	MHAHH762-06	GU154909
5990	Astraptes anaphus annettaDHJ03	Eudaminae	03-SRNP-34428	MHAHH756-06	GU154908
5991	Astraptes anaphus annettaDHJ03	Eudaminae	03-SRNP-7277	MHAHH755-06	GU154907

Tree Order	Species	Subfamily	ACG Sampleid	BOLD Processid	Genbank Accession
5992	Astraptes anaphus annettaDHJ03	Eudaminae	06-SRNP-41210	MHAHG707-06	GU151086
5993	Astraptes anaphus annettaDHJ03	Eudaminae	06-SRNP-41208	MHAHG706-06	GU151087
5994	Astraptes anaphus annettaDHJ03	Eudaminae	04-SRNP-13408	MHAHE159-05	GU149410
5995	Astraptes anaphus annettaDHJ03	Eudaminae	93-SRNP-4846	MHMXG297-07	JF760290
5996	Astraptes anaphus annettaDHJ03	Eudaminae	93-SRNP-7913	MHMXG321-07	JF760307
5997	Astraptes anaphus annettaDHJ03	Eudaminae	07-SRNP-20180	MHMXK126-07	JF761462
5998	Astraptes anaphus annettaDHJ03	Eudaminae	07-SRNP-42788	MHMXT176-08	JF761460
5999	Astraptes anaphus annettaDHJ02	Eudaminae	92-SRNP-4965	MHMXG322-07	JF760283
6000	Astraptes anaphus annettaDHJ02	Eudaminae	97-SRNP-421	MHMXG331-07	JF760274
6001	Astraptes anaphus annettaDHJ02	Eudaminae	00-SRNP-19297	MHMXG330-07	JF760261
6002	Astraptes anaphus annettaDHJ02	Eudaminae	97-SRNP-480	MHMXG314-07	JF760267
6003	Astraptes anaphus annettaDHJ02	Eudaminae	92-SRNP-4960	MHMXG328-07	JF760288
6004	Astraptes anaphus annettaDHJ02	Eudaminae	06-SRNP-30279	MHAHG110-06	GU151083
6005	Astraptes anaphus annettaDHJ02	Eudaminae	97-SRNP-3128	MHMXG304-07	JF760280
6006	Astraptes anaphus annettaDHJ02	Eudaminae	97-SRNP-3009	MHMXG307-07	JF760276
6007	Astraptes anaphus annettaDHJ02	Eudaminae	93-SRNP-8376	MHAHI1062-07	GU155768
6008	Astraptes anaphus annettaDHJ02	Eudaminae	06-SRNP-41207	MHAHH497-06	GU154902
6009	Astraptes anaphus annettaDHJ02	Eudaminae	02-SRNP-28509	MHMXG290-07	JF760259
6010	Astraptes anaphus annettaDHJ02	Eudaminae	93-SRNP-6180	MHMXG293-07	JF760269
6011	Astraptes anaphus annettaDHJ02	Eudaminae	97-SRNP-2560	MHMXG285-07	JF760278
6012	Astraptes anaphus annettaDHJ02	Eudaminae	96-SRNP-4565	MHMXG283-07	JF760282
6013	Astraptes anaphus annettaDHJ02	Eudaminae	93-SRNP-6733	MHAHI1060-07	GU155766
6014	Astraptes anaphus annettaDHJ02	Eudaminae	02-SRNP-32066	MHAHH818-06	GU154898
6015	Astraptes anaphus annettaDHJ02	Eudaminae	97-SRNP-2566	MHAHH771-06	GU154901
6016	Astraptes anaphus annettaDHJ02	Eudaminae	02-SRNP-28069	MHAHH767-06	GU154900
6017	Astraptes anaphus annettaDHJ02	Eudaminae	02-SRNP-2245	MHAHH760-06	GU154899
6018	Astraptes anaphus annettaDHJ02	Eudaminae	06-SRNP-41209	MHAHG705-06	GU151084
6019	Astraptes anaphus annettaDHJ02	Eudaminae	06-SRNP-41039	MHAHG704-06	GU151085
6020	Astraptes anaphus annettaDHJ02	Eudaminae	04-SRNP-61482	MHAHE160-05	GU149409
6021	Astraptes anaphus annettaDHJ02	Eudaminae	94-SRNP-6194	MHMXG329-07	JF760287
6022	Astraptes anaphus annettaDHJ02	Eudaminae	96-SRNP-4564	MHMXG316-07	JF760265
6023	Astraptes anaphus annettaDHJ02	Eudaminae	93-SRNP-6178	MHMXG301-07	JF760284
6024	Astraptes anaphus annettaDHJ02	Eudaminae	94-SRNP-7265	MHMXG300-07	JF760285
6025	Astraptes anaphus annettaDHJ02	Eudaminae	93-SRNP-4708	MHMXG295-07	JF760266
6026	Astraptes anaphus annettaDHJ02	Eudaminae	93-SRNP-8379	MHMXG291-07	JF760273
6027	Astraptes anaphus annettaDHJ02	Eudaminae	93-SRNP-6179	MHMXG289-07	JF760286
6028	Astraptes anaphus annettaDHJ02	Eudaminae	92-SRNP-4963	MHAHI1063-07	GU155767
6029	Astraptes anaphus annettaDHJ02	Eudaminae	02-SRNP-5753	MHMXG298-07	JF760263
6030	Astraptes anaphus annettaDHJ02	Eudaminae	93-SRNP-7917	MHMXG325-07	JF760279
6031	Astraptes anaphus annettaDHJ02	Eudaminae	93-SRNP-5032	MHMXG308-07	JF760275
6032	Astraptes anaphus annettaDHJ02	Eudaminae	94-SRNP-6195	MHMXG324-07	JF760281
6033	Astraptes anaphus annettaDHJ02	Eudaminae	97-SRNP-2556	MHMXG313-07	JF760270
6034	Astraptes anaphus annettaDHJ02	Eudaminae	93-SRNP-8378	MHMXG299-07	JF760262
6035	Astraptes anaphus annettaDHJ02	Eudaminae	97-SRNP-2559	MHMXG287-07	JF760277
6036	Astraptes anaphus annettaDHJ02	Eudaminae	97-SRNP-418	MHMXG332-07	JF760272
6037	Astraptes anaphus annettaDHJ02	Eudaminae	06-SRNP-21977	MHAHJ790-07	JF752383
6038	Astraptes anaphus annettaDHJ02	Eudaminae	07-SRNP-20150	MHMXK119-07	JF761459
6039	Astraptes anaphus annettaDHJ02	Eudaminae	07-SRNP-20439	MHMXK122-07	JF761458
6040	Astraptes anaphus annettaDHJ02	Eudaminae	07-SRNP-20089	MHMXK127-07	JF761457
6041	Astraptes anaphus annettaDHJ02	Eudaminae	02-SRNP-28508	MHMXG294-07	JF760268
6042	Astraptes anaphus annettaDHJ02	Eudaminae	97-SRNP-477	MHMXG310-07	JF760260
6043	Astraptes anaphus annettaDHJ02	Eudaminae	97-SRNP-420	MHMXG312-07	JF760271
6044	Astraptes anaphus annettaDHJ02	Eudaminae	00-SRNP-20266	MHMXG318-07	JF760264
6045	Astraptes anaphus annettaDHJ02	Eudaminae	09-SRNP-44302	MHMYE1518-09	GU653488
6046	Astraptes anaphus annettaDHJ02	Eudaminae	09-SRNP-20617	MHMYG2413-10	HM885836
6047	Astraptes hopfferiDHJ02	Eudaminae	00-SRNP-6038	CSCR031-04	DQ291879

Tree Order	Species	Subfamily	ACG Sampleid	BOLD Processid	Genbank Accession
6048	Astraptes hopfferiDHJ02	Eudaminae	05-SRNP-60351	MHAHF725-06	GU150226
6049	Astraptes hopfferiDHJ02	Eudaminae	05-SRNP-60641	MHAHF723-06	GU150230
6050	Astraptes hopfferiDHJ02	Eudaminae	05-SRNP-24819	MHAHF726-06	GU150236
6051	Astraptes hopfferiDHJ02	Eudaminae	05-SRNP-24755	MHAHF727-06	GU150233
6052	Astraptes hopfferiDHJ02	Eudaminae	05-SRNP-24197	MHAHF732-06	GU150240
6053	Astraptes hopfferiDHJ02	Eudaminae	05-SRNP-19978	MHAHF733-06	GU150239
6054	Astraptes hopfferiDHJ02	Eudaminae	05-SRNP-66135	MHAHF722-06	GU150232
6055	Astraptes hopfferiDHJ02	Eudaminae	05-SRNP-24396	MHAHF731-06	GU150231
6056	Astraptes hopfferiDHJ02	Eudaminae	05-SRNP-60568	MHAHF734-06	GU150237
6057	Astraptes hopfferiDHJ02	Eudaminae	05-SRNP-61464	MHAHF735-06	GU150234
6058	Astraptes hopfferiDHJ02	Eudaminae	05-SRNP-24340	MHAHF737-06	GU150241
6059	Astraptes hopfferiDHJ02	Eudaminae	07-SRNP-57872	MHMXO875-08	JF761533
6060	Astraptes hopfferiDHJ02	Eudaminae	00-SRNP-6035	CSCR030-04	DQ291878
6061	Astraptes hopfferiDHJ02	Eudaminae	06-SRNP-58062	MHAHJ735-07	JF752394
6062	Astraptes hopfferiDHJ02	Eudaminae	07-SRNP-21123	MHMXN285-07	JF761536
6063	Astraptes hopfferiDHJ02	Eudaminae	07-SRNP-20481	MHMXK118-07	JF761539
6064	Astraptes hopfferiDHJ02	Eudaminae	07-SRNP-20482	MHMXK116-07	JF761538
6065	Astraptes hopfferiDHJ02	Eudaminae	02-SRNP-5424	MHAHH885-06	GU154986
6066	Astraptes hopfferiDHJ02	Eudaminae	99-SRNP-2552	MHAHH881-06	GU154982
6067	Astraptes hopfferiDHJ02	Eudaminae	98-SRNP-4521	MHAHH879-06	GU154981
6068	Astraptes hopfferiDHJ02	Eudaminae	01-SRNP-16888	MHAHH875-06	GU154978
6069	Astraptes hopfferiDHJ02	Eudaminae	01-SRNP-16907	MHAHH871-06	GU154977
6070	Astraptes hopfferiDHJ02	Eudaminae	95-SRNP-10163	MHAHH867-06	GU154974
6071	Astraptes hopfferiDHJ02	Eudaminae	05-SRNP-19981	MHAHF265-06	GU150228
6072	Astraptes hopfferiDHJ02	Eudaminae	05-SRNP-19980	MHAHF264-06	GU150229
6073	Astraptes hopfferiDHJ02	Eudaminae	04-SRNP-15947	MHAHE024-05	GU149426
6074	Astraptes hopfferiDHJ02	Eudaminae	05-SRNP-21448	MHAHF231-06	GU150227
6075	Astraptes hopfferiDHJ02	Eudaminae	96-SRNP-9303	MHAHH869-06	GU154975
6076	Astraptes hopfferiDHJ02	Eudaminae	05-SRNP-24589	MHAHF728-06	GU150235
6077	Astraptes hopfferiDHJ02	Eudaminae	05-SRNP-24568	MHAHF729-06	GU150238
6078	Astraptes hopfferiDHJ02	Eudaminae	98-SRNP-4411	MHAHH886-06	GU154985
6079	Astraptes hopfferiDHJ02	Eudaminae	07-SRNP-55079	MHAHK230-07	JF760352
6080	Astraptes hopfferiDHJ02	Eudaminae	07-SRNP-20899	MHMXN284-07	JF761537
6081	Astraptes hopfferiDHJ02	Eudaminae	07-SRNP-24342	MHMXT146-08	JF761530
6082	Astraptes hopfferiDHJ02	Eudaminae	07-SRNP-15266	MHMXX735-09	JF777626
6083	Astraptes hopfferiDHJ02	Eudaminae	07-SRNP-15267	MHMXX736-09	JF777627
6084	Astraptes hopfferiDHJ02	Eudaminae	07-SRNP-15268	MHMXX737-09	JF777628
6085	Astraptes hopfferiDHJ02	Eudaminae	08-SRNP-16500	MHMXY1101-09	GU666441
6086	Astraptes hopfferiDHJ02	Eudaminae	08-SRNP-16501	MHMXY1103-09	GU666435
6087	Astraptes hopfferiDHJ02	Eudaminae	08-SRNP-16509	MHMXY1104-09	GU666436
6088	Astraptes hopfferiDHJ02	Eudaminae	08-SRNP-65151	MHMXS094-08	JF761532
6089	Astraptes hopfferiDHJ02	Eudaminae	07-SRNP-24343	MHMXT145-08	JF761531
6090	Astraptes hopfferiDHJ02	Eudaminae	08-SRNP-16506	MHMXY1105-09	HM390683
6091	Astraptes hopfferiDHJ02	Eudaminae	08-SRNP-14782	MHMXY1106-09	GU666437
6092	Astraptes hopfferiDHJ02	Eudaminae	08-SRNP-16510	MHMXY1107-09	GU666438
6093	Astraptes hopfferiDHJ02	Eudaminae	08-SRNP-16504	MHMXY1108-09	GU666431
6094	Astraptes hopfferiDHJ02	Eudaminae	00-SRNP-18470	MHAHH866-06	GU154973
6095	Astraptes hopfferiDHJ02	Eudaminae	03-SRNP-15756	MHAHH870-06	GU154976
6096	Astraptes hopfferiDHJ02	Eudaminae	02-SRNP-13859	MHAHH876-06	GU154972
6097	Astraptes hopfferiDHJ02	Eudaminae	01-SRNP-16887	MHAHH877-06	GU154979
6098	Astraptes hopfferiDHJ02	Eudaminae	02-SRNP-13861	MHAHH878-06	GU154980
6099	Astraptes hopfferiDHJ02	Eudaminae	02-SRNP-33228	MHAHH882-06	GU154983
6100	Astraptes hopfferiDHJ02	Eudaminae	02-SRNP-33292	MHAHH883-06	GU154984
6101	Astraptes hopfferiDHJ02	Eudaminae	01-SRNP-16885	MHAHH884-06	GU154971
6102	Astraptes hopfferiDHJ02	Eudaminae	07-SRNP-55080	MHMXK117-07	JF761540
6103	Astraptes hopfferiDHJ02	Eudaminae	07-SRNP-57649	MHMXO871-08	JF761535

Tree Order	Species	Subfamily	ACG Sampleid	BOLD Processid	Genbank Accession
6104	Astraptes hopfferiDHJ02	Eudaminae	07-SRNP-57556	MHMXO874-08	JF761534
6105	Astraptes hopfferiDHJ02	Eudaminae	08-SRNP-13809	MHMXW361-09	JF753668
6106	Astraptes hopfferiDHJ02	Eudaminae	08-SRNP-13808	MHMXW362-09	JF753669
6107	Astraptes hopfferiDHJ02	Eudaminae	08-SRNP-16352	MHMXY1102-09	GU666442
6108	Astraptes hopfferiDHJ02	Eudaminae	08-SRNP-16353	MHMXY1109-09	GU666432
6109	Astraptes hopfferiDHJ01	Eudaminae	05-SRNP-60355	MHAHF724-06	GU150223
6110	Astraptes hopfferiDHJ01	Eudaminae	05-SRNP-24692	MHAHF730-06	GU150225
6111	Astraptes hopfferiDHJ01	Eudaminae	05-SRNP-24395	MHAHF736-06	GU150224
6112	Astraptes hopfferiDHJ01	Eudaminae	05-SRNP-21444	MHAHF239-06	GU150222
6113	Astraptes hopfferiDHJ01	Eudaminae	98-SRNP-4412	MHAHH868-06	GU154966
6114	Astraptes hopfferiDHJ01	Eudaminae	98-SRNP-4402	MHAHH872-06	GU154968
6115	Astraptes hopfferiDHJ01	Eudaminae	98-SRNP-4409	MHAHH873-06	GU154969
6116	Astraptes hopfferiDHJ01	Eudaminae	98-SRNP-4399	MHAHH874-06	GU154970
6117	Astraptes hopfferiDHJ01	Eudaminae	98-SRNP-4408	MHAHH880-06	GU154967
6118	Astraptes hopfferiDHJ01	Eudaminae	08-SRNP-24553	MHMYB126-09	GU649699
6119	Astraptes chiriquensis	Eudaminae	05-SRNP-32306	MHAHE426-05	GU149419
6120	Astraptes chiriquensis	Eudaminae	04-SRNP-33647	MHAHD875-05	GU161291
6121	Astraptes chiriquensis	Eudaminae	04-SRNP-2338	CSRII429-04	DQ291861
6122	Astraptes chiriquensis	Eudaminae	06-SRNP-31610	MHAHH496-06	GU154927
6123	Astraptes chiriquensis	Eudaminae	07-SRNP-41188	MHMXN308-07	JF761490
6124	Astraptes chiriquensis	Eudaminae	07-SRNP-40863	MHMXN309-07	JF761489
6125	Astraptes chiriquensis	Eudaminae	07-SRNP-23622	MHMXP167-08	JF761488
6126	Astraptes alardus	Eudaminae	05-SRNP-66269	MHAHG116-06	GU151081
6127	Astraptes alardus	Eudaminae	05-SRNP-57547	MHAHF311-06	GU150201
6128	Astraptes alardus	Eudaminae	07-SRNP-56409	MHMXN314-07	JF761449
6129	Astraptes alardus	Eudaminae	07-SRNP-56408	MHMXN313-07	JF761450
6130	Astraptes alardus	Eudaminae	07-SRNP-35878	MHMXN312-07	JF761451
6131	Astraptes alardus	Eudaminae	07-SRNP-56961	MHMXN310-07	JF761453
6132	Astraptes alardus	Eudaminae	07-SRNP-56397	MHMXK100-07	JF761454
6133	Astraptes alardus	Eudaminae	06-SRNP-58880	MHAHK077-07	JF760258
6134	Astraptes alardus	Eudaminae	04-SRNP-60018	MHAHD719-05	GU161287
6135	Astraptes alardus	Eudaminae	04-SRNP-35460	MHAHD718-05	GU161290
6136	Astraptes alardus	Eudaminae	04-SRNP-45519	MHAHD717-05	GU161289
6137	Astraptes alardus	Eudaminae	04-SRNP-35461	MHAHD716-05	GU161285
6138	Astraptes alardus	Eudaminae	04-SRNP-24624	MHAHD714-05	GU161288
6139	Astraptes alardus	Eudaminae	07-SRNP-35859	MHMXP166-08	JF761444
6140	Astraptes alardus	Eudaminae	07-SRNP-56299	MHMXK099-07	JF761455
6141	Astraptes alardus	Eudaminae	05-SRNP-57546	MHAHF313-06	GU150203
6142	Astraptes alardus	Eudaminae	05-SRNP-66293	MHAHG115-06	GU151082
6143	Astraptes alardus	Eudaminae	04-SRNP-34471	MHAHD715-05	GU161286
6144	Astraptes alardus	Eudaminae	03-SRNP-3693	CSCR306-04	DQ291852
6145	Astraptes alardus	Eudaminae	07-SRNP-56473	MHMXN316-07	JF761447
6146	Astraptes alardus	Eudaminae	06-SRNP-35909	MHAHK075-07	JF760256
6147	Astraptes alardus	Eudaminae	06-SRNP-35908	MHAHK076-07	JF760257
6148	Astraptes alardus	Eudaminae	03-SRNP-3691	CSCR307-04	DQ291853
6149	Astraptes alardus	Eudaminae	05-SRNP-20286	MHAHF312-06	GU150202
6150	Astraptes alardus	Eudaminae	07-SRNP-35953	MHMXN311-07	JF761452
6151	Astraptes alardus	Eudaminae	07-SRNP-56765	MHMXN315-07	JF761448
6152	Astraptes alardus	Eudaminae	07-SRNP-65143	MHMXN317-07	JF761446
6153	Astraptes alardus	Eudaminae	07-SRNP-35914	MHMXP222-08	JF761445
6154	Astraptes alardus	Eudaminae	08-SRNP-45019	MHMXX859-09	JF777600
6155	Astraptes alardus	Eudaminae	08-SRNP-36614	MHMXX484-09	JF777599
6156	Astraptes creteus cranaDHJ01	Eudaminae	02-SRNP-23100	MHAHH835-06	GU154931
6157	Astraptes creteus cranaDHJ01	Eudaminae	97-SRNP-1464	MHAHI276-06	GU155779
6158	Astraptes creteus cranaDHJ01	Eudaminae	02-SRNP-23417	MHAHI274-06	GU155782
6159	Astraptes creteus cranaDHJ01	Eudaminae	99-SRNP-13962	MHAHI285-06	GU155783

Tree Order	Species	Subfamily	ACG Sampleid	BOLD Processid	Genbank Accession
6160	Astraptes creteus cranaDHJ01	Eudaminae	02-SRNP-23933	MHAHI279-06	GU155780
6161	Astraptes creteus cranaDHJ01	Eudaminae	99-SRNP-13961	MHAHI273-06	GU155781
6162	Astraptes creteus cranaDHJ01	Eudaminae	03-SRNP-4189	MHAHH825-06	GU154930
6163	Astraptes creteus cranaDHJ01	Eudaminae	03-SRNP-4188	MHAHH824-06	GU154928
6164	Astraptes creteus cranaDHJ01	Eudaminae	03-SRNP-4333	MHAHH822-06	GU154929
6165	Astraptes creteus cranaDHJ01	Eudaminae	05-SRNP-35360	MHAHF262-06	GU150210
6166	Astraptes creteus cranaDHJ01	Eudaminae	97-SRNP-1467	MHMXG347-07	JF760315
6167	Astraptes creteus cranaDHJ01	Eudaminae	97-SRNP-1465	MHMXG355-07	JF760314
6168	Astraptes creteus cranaDHJ01	Eudaminae	98-SRNP-3078	MHMXG359-07	JF760313
6169	Astraptes creteus cranaDHJ01	Eudaminae	97-SRNP-1253	MHMXG363-07	JF760317
6170	Astraptes creteus cranaDHJ01	Eudaminae	99-SRNP-13963	MHMXG365-07	JF760316
6171	Astraptes creteus cranaDHJ02	Eudaminae	98-SRNP-2979	MHMXG334-07	JF760327
6172	Astraptes creteus cranaDHJ02	Eudaminae	02-SRNP-23727	CSCR026-04	DQ291862
6173	Astraptes creteus cranaDHJ02	Eudaminae	02-SRNP-24378	CSCR027-04	DQ291863
6174	Astraptes creteus cranaDHJ02	Eudaminae	02-SRNP-9497	MHAHH836-06	GU154932
6175	Astraptes creteus cranaDHJ02	Eudaminae	98-SRNP-3853	MHMXG362-07	JF760338
6176	Astraptes creteus cranaDHJ02	Eudaminae	00-SRNP-9497	MHAHI281-06	GU155803
6177	Astraptes creteus cranaDHJ02	Eudaminae	00-SRNP-9363	MHAHI287-06	GU155788
6178	Astraptes creteus cranaDHJ02	Eudaminae	03-SRNP-4452	MHAHI289-06	GU155804
6179	Astraptes creteus cranaDHJ02	Eudaminae	03-SRNP-23876	MHAHH827-06	GU154937
6180	Astraptes creteus cranaDHJ02	Eudaminae	03-SRNP-13379	MHAHH829-06	GU154938
6181	Astraptes creteus cranaDHJ02	Eudaminae	03-SRNP-23795	MHAHH823-06	GU154935
6182	Astraptes creteus cranaDHJ02	Eudaminae	03-SRNP-3042	MHAHH826-06	GU154936
6183	Astraptes creteus cranaDHJ02	Eudaminae	03-SRNP-23880	MHAHH821-06	GU154934
6184	Astraptes creteus cranaDHJ02	Eudaminae	05-SRNP-35359	MHAHF263-06	GU150213
6185	Astraptes creteus cranaDHJ02	Eudaminae	05-SRNP-35230	MHAHF261-06	GU150215
6186	Astraptes creteus cranaDHJ02	Eudaminae	05-SRNP-35582	MHAHF260-06	GU150216
6187	Astraptes creteus cranaDHJ02	Eudaminae	05-SRNP-35228	MHAHF259-06	GU150214
6188	Astraptes creteus cranaDHJ02	Eudaminae	05-SRNP-35253	MHAHF232-06	GU150212
6189	Astraptes creteus cranaDHJ02	Eudaminae	04-SRNP-35324	MHAHE047-05	GU149422
6190	Astraptes creteus cranaDHJ02	Eudaminae	04-SRNP-35590	MHAHE046-05	GU149421
6191	Astraptes creteus cranaDHJ02	Eudaminae	04-SRNP-35599	MHAHE023-05	GU149420
6192	Astraptes creteus cranaDHJ02	Eudaminae	00-SRNP-9654	MHAHI290-06	GU155787
6193	Astraptes creteus cranaDHJ02	Eudaminae	03-SRNP-4174	MHAHH828-06	GU154933
6194	Astraptes creteus cranaDHJ02	Eudaminae	00-SRNP-9951	MHAHH831-06	GU154940
6195	Astraptes creteus cranaDHJ02	Eudaminae	00-SRNP-9783	MHMXG343-07	JF760336
6196	Astraptes creteus cranaDHJ02	Eudaminae	07-SRNP-57106	MHAHL564-07	JF761491
6197	Astraptes creteus cranaDHJ02	Eudaminae	02-SRNP-8259	MHMXG350-07	JF760318
6198	Astraptes creteus cranaDHJ02	Eudaminae	98-SRNP-2019	MHMXG345-07	JF760333
6199	Astraptes creteus cranaDHJ02	Eudaminae	02-SRNP-9883	MHAHI278-06	GU155797
6200	Astraptes creteus cranaDHJ02	Eudaminae	97-SRNP-1362	MHMXG341-07	JF760339
6201	Astraptes creteus cranaDHJ02	Eudaminae	99-SRNP-291	MHMXG339-07	JF760319
6202	Astraptes creteus cranaDHJ02	Eudaminae	00-SRNP-10716	MHMXG351-07	JF760331
6203	Astraptes creteus cranaDHJ02	Eudaminae	02-SRNP-8156	MHAHI286-06	GU155790
6204	Astraptes creteus cranaDHJ02	Eudaminae	02-SRNP-23538	MHAHI275-06	GU155798
6205	Astraptes creteus cranaDHJ02	Eudaminae	97-SRNP-1617	MHMXG338-07	JF760320
6206	Astraptes creteus cranaDHJ02	Eudaminae	98-SRNP-11988	MHMXG352-07	JF760330
6207	Astraptes creteus cranaDHJ02	Eudaminae	98-SRNP-2628	MHMXG337-07	JF760322
6208	Astraptes creteus cranaDHJ02	Eudaminae	98-SRNP-2871	MHMXG336-07	JF760325
6209	Astraptes creteus cranaDHJ02	Eudaminae	98-SRNP-2709	MHMXG335-07	JF760326
6210	Astraptes creteus cranaDHJ02	Eudaminae	00-SRNP-9398	MHAHI288-06	GU155789
6211	Astraptes creteus cranaDHJ02	Eudaminae	01-SRNP-6265	MHAHI283-06	GU155791
6212	Astraptes creteus cranaDHJ02	Eudaminae	01-SRNP-6809	MHAHI282-06	GU155794
6213	Astraptes creteus cranaDHJ02	Eudaminae	95-SRNP-537	MHMXG342-07	JF760337
6214	Astraptes creteus cranaDHJ02	Eudaminae	94-SRNP-5247	MHMXG346-07	JF760332
6215	Astraptes creteus cranaDHJ02	Eudaminae	98-SRNP-2873	MHMXG356-07	JF760324

Tree Order	Species	Subfamily	ACG Sampleid	BOLD Processid	Genbank Accession
6216	Astraptes creteus cranaDHJ02	Eudaminae	07-SRNP-57105	MHMXN283-07	JF761495
6217	Astraptes creteus cranaDHJ02	Eudaminae	02-SRNP-24377	MHAHH830-06	GU154939
6218	Astraptes creteus cranaDHJ02	Eudaminae	01-SRNP-6805	MHAHH832-06	GU154941
6219	Astraptes creteus cranaDHJ02	Eudaminae	01-SRNP-7968	MHAHH833-06	GU154942
6220	Astraptes creteus cranaDHJ02	Eudaminae	02-SRNP-8216	MHAHH837-06	GU154943
6221	Astraptes creteus cranaDHJ02	Eudaminae	01-SRNP-7967	MHAHH838-06	GU154944
6222	Astraptes creteus cranaDHJ02	Eudaminae	02-SRNP-23585	MHAHI266-06	GU155784
6223	Astraptes creteus cranaDHJ02	Eudaminae	02-SRNP-24509	MHAHI267-06	GU155802
6224	Astraptes creteus cranaDHJ02	Eudaminae	02-SRNP-23630	MHAHI268-06	GU155799
6225	Astraptes creteus cranaDHJ02	Eudaminae	01-SRNP-6182	MHAHI269-06	GU155801
6226	Astraptes creteus cranaDHJ02	Eudaminae	01-SRNP-6806	MHAHI271-06	GU155800
6227	Astraptes creteus cranaDHJ02	Eudaminae	02-SRNP-23118	MHAHI277-06	GU155796
6228	Astraptes creteus cranaDHJ02	Eudaminae	02-SRNP-23539	MHAHI284-06	GU155793
6229	Astraptes creteus cranaDHJ02	Eudaminae	98-SRNP-2980	MHMXG340-07	JF760341
6230	Astraptes creteus cranaDHJ02	Eudaminae	97-SRNP-1252	MHMXG344-07	JF760334
6231	Astraptes creteus cranaDHJ02	Eudaminae	97-SRNP-11002	MHMXG348-07	JF760344
6232	Astraptes creteus cranaDHJ02	Eudaminae	02-SRNP-8258	MHMXG349-07	JF760343
6233	Astraptes creteus cranaDHJ02	Eudaminae	00-SRNP-9624	MHMXG353-07	JF760329
6234	Astraptes creteus cranaDHJ02	Eudaminae	98-SRNP-2805	MHMXG354-07	JF760328
6235	Astraptes creteus cranaDHJ02	Eudaminae	00-SRNP-9502	MHMXG357-07	JF760323
6236	Astraptes creteus cranaDHJ02	Eudaminae	98-SRNP-2055	MHMXG358-07	JF760321
6237	Astraptes creteus cranaDHJ02	Eudaminae	98-SRNP-2806	MHMXG360-07	JF760342
6238	Astraptes creteus cranaDHJ02	Eudaminae	98-SRNP-3912	MHMXG361-07	JF760340
6239	Astraptes creteus cranaDHJ02	Eudaminae	97-SRNP-1835	MHMXG364-07	JF760335
6240	Astraptes creteus cranaDHJ02	Eudaminae	06-SRNP-35585	MHAHJ627-07	JF752388
6241	Astraptes creteus cranaDHJ02	Eudaminae	06-SRNP-46102	MHAHJ628-07	JF752389
6242	Astraptes creteus cranaDHJ02	Eudaminae	07-SRNP-36022	MHMXO872-08	JF761494
6243	Astraptes creteus cranaDHJ02	Eudaminae	07-SRNP-57188	MHMXO873-08	JF761493
6244	Astraptes creteus cranaDHJ02	Eudaminae	07-SRNP-36021	MHMXO876-08	JF761492
6245	Astraptes creteus cranaDHJ02	Eudaminae	07-SRNP-36883	MHMXW363-09	JF753666
6246	Astraptes creteus cranaDHJ02	Eudaminae	08-SRNP-35767	MHMXX481-09	JF777602
6247	Astraptes creteus cranaDHJ02	Eudaminae	08-SRNP-35790	MHMXX482-09	JF777603
6248	Astraptes creteus cranaDHJ02	Eudaminae	08-SRNP-35544	MHMXX483-09	JF777604
6249	Astraptes creteus cranaDHJ02	Eudaminae	09-SRNP-56363	MHMYG2402-10	HM885824
6250	Astraptes creteus cranaDHJ02	Eudaminae	09-SRNP-56364	MHMYG2403-10	HM885825
6251	Astraptes creteus cranaDHJ02	Eudaminae	02-SRNP-9064	MHAHI280-06	GU155795
6252	Astraptes creteus cranaDHJ02	Eudaminae	04-SRNP-36066	MHAHF246-06	GU150211
6253	Astraptes creteus cranaDHJ02	Eudaminae	09-SRNP-36873	MHMYG2026-10	HM885428
6254	Astraptes talus	Eudaminae	03-SRNP-1776	CSCR312-04	DQ291887
6255	Astraptes talus	Eudaminae	02-SRNP-28715	CSCR036-04	DQ291886
6256	Astraptes talus	Eudaminae	05-SRNP-22282	MHAHF867-06	GU150249
6257	Astraptes talus	Eudaminae	07-SRNP-42996	MHMXR797-08	JF761632
6258	Astraptes talus	Eudaminae	06-SRNP-21979	MHMXK058-07	JF761634
6259	Astraptes talus	Eudaminae	06-SRNP-56333	MHAHH563-06	GU155108
6260	Astraptes talus	Eudaminae	05-SRNP-22277	MHAHF873-06	GU150250
6261	Astraptes talus	Eudaminae	05-SRNP-22036	MHAHF872-06	GU150252
6262	Astraptes talus	Eudaminae	05-SRNP-22279	MHAHF871-06	GU150251
6263	Astraptes talus	Eudaminae	05-SRNP-22031	MHAHF870-06	GU150246
6264	Astraptes talus	Eudaminae	05-SRNP-22257	MHAHF869-06	GU150247
6265	Astraptes talus	Eudaminae	05-SRNP-22033	MHAHF868-06	GU150248
6266	Astraptes talus	Eudaminae	07-SRNP-1322	MHMXK059-07	JF761633
6267	Astraptes talus	Eudaminae	05-SRNP-25199	MHAHL095-07	JF761628
6268	Astraptes talus	Eudaminae	05-SRNP-25202	MHAHL096-07	JF761629
6269	Astraptes talus	Eudaminae	07-SRNP-43000	MHMXT217-08	JF761631
6270	Astraptes talus	Eudaminae	08-SRNP-40181	MHMXT218-08	JF761630
6271	Astraptes talus	Eudaminae	09-SRNP-41979	MHMYE1407-09	HM424356

Tree Order	Species	Subfamily	ACG Sampleid	BOLD Processid	Genbank Accession
6272	Astraptes talus	Eudaminae	09-SRNP-41975	MHMYE1408-09	HM424357
6273	Astraptes talus	Eudaminae	09-SRNP-41973	MHMYE1409-09	HM424358
6274	Astraptes talus	Eudaminae	09-SRNP-41980	MHMYE1410-09	HM424359
6275	Astraptes talus	Eudaminae	09-SRNP-41977	MHMYE1411-09	HM424360
6276	Astraptes talus	Eudaminae	09-SRNP-41974	MHMYE1412-09	HM424361
6277	Astraptes talus	Eudaminae	09-SRNP-41983	MHMYE1405-09	HM424354
6278	Astraptes talus	Eudaminae	09-SRNP-41978	MHMYE1406-09	HM424355
6279	Astraptes talus	Eudaminae	09-SRNP-41982	MHMYE1413-09	HM424362
6280	Autochton longipennis	Pyrginae	01-SRNP-2960	CSRII105-04	DQ291903
6281	Achalarus albociliatus	Eudaminae	05-SRNP-56408	MHAHF347-06	GU150158
6282	Achalarus albociliatus	Eudaminae	96-SRNP-1083	CSCR001-04	DQ291759
6283	Achalarus albociliatus	Eudaminae	00-SRNP-6810	CSRII522-04	DQ291760
6284	Achalarus albociliatus	Eudaminae	02-SRNP-14451	MHAHE113-05	GU149306
6285	Achalarus albociliatus	Eudaminae	02-SRNP-14113	MHAHE112-05	GU149304
6286	Achalarus albociliatus	Eudaminae	02-SRNP-14234	MHAHE111-05	GU149305
6287	Achalarus albociliatus	Eudaminae	00-SRNP-6827	CSRII523-04	DQ291761
6288	Achalarus albociliatus	Eudaminae	02-SRNP-4786	MHAHE114-05	GU149307
6289	Achalarus albociliatus	Eudaminae	07-SRNP-12984	MHMXS074-08	JF761321
6290	Achalarus toxeus	Eudaminae	96-SRNP-8690	CSCR004-04	DQ291763
6291	Achalarus toxeus	Eudaminae	93-SRNP-7539	CSCR003-04	DQ291762
6292	Achalarus toxeus	Eudaminae	02-SRNP-10179	MHAHE117-05	GU149309
6293	Achalarus toxeus	Eudaminae	02-SRNP-10180	MHAHE118-05	GU149308
6294	Achalarus toxeus	Eudaminae	03-SRNP-237	MHAHE122-05	GU149310
6295	Thessia jalapus	Pyrginae	08-SRNP-22900	MHMXY1080-09	GU666462
6296	Thessia jalapus	Pyrginae	97-SRNP-4702	CSCR265-04	DQ293592
6297	Thessia jalapus	Pyrginae	08-SRNP-22673	MHMXX653-09	JF778550
6298	Thessia jalapus	Pyrginae	08-SRNP-30977	MHMXW453-09	JF754324
6299	Thessia jalapus	Pyrginae	08-SRNP-30974	MHMXW451-09	JF754322
6300	Thessia jalapus	Pyrginae	02-SRNP-13559	MHAHE119-05	GU150120
6301	Thessia jalapus	Pyrginae	08-SRNP-30979	MHMXW449-09	JF754320
6302	Thessia jalapus	Pyrginae	09-SRNP-30937	MHMYC468-09	
6303	Thessia jalapus	Pyrginae	09-SRNP-20372	MHMYG2411-10	HM885834
6304	Thessia jalapus	Pyrginae	08-SRNP-23350	MHMYB132-09	GU649689
6305	Thessia jalapus	Pyrginae	09-SRNP-20138	MHMYG2410-10	HM885832
6306	Thessia jalapus	Pyrginae	08-SRNP-24707	MHMYB131-09	GU649695
6307	Thessia jalapus	Pyrginae	04-SRNP-22865	MHMXY954-09	GU666568
6308	Thessia jalapus	Pyrginae	08-SRNP-55016	MHMXW452-09	JF754323
6309	Thessia jalapus	Pyrginae	08-SRNP-30973	MHMXW448-09	JF754319
6310	Thessia jalapus	Pyrginae	07-SRNP-55386	MHMXK055-07	JF763248
6311	Thessia jalapus	Pyrginae	02-SRNP-13729	MHAHE121-05	GU150121
6312	Thessia jalapus	Pyrginae	02-SRNP-13560	MHAHE120-05	GU150119
6313	Thessia jalapus	Pyrginae	02-SRNP-4830	MHAHE116-05	GU150117
6314	Thessia jalapus	Pyrginae	02-SRNP-14049	MHAHE115-05	GU150118
6315	Thessia jalapus	Pyrginae	04-SRNP-14601	MHAHD876-05	GU161890
6316	Thessia jalapus	Pyrginae	07-SRNP-21140	MHMXK054-07	JF763249
6317	Thessia jalapus	Pyrginae	97-SRNP-4570	CSCR264-04	DQ293591
6318	Thessia jalapus	Pyrginae	08-SRNP-21898	MHMXW450-09	JF754321
6319	Thessia jalapus	Pyrginae	09-SRNP-20140	MHMYG2412-10	HM885835
6320	Astraptes phalaecus	Eudaminae	00-SRNP-17853	CSCR034-04	DQ291885
6321	Astraptes phalaecus	Eudaminae	09-SRNP-33034	MHMYG2414-10	HM885837
6322	Astraptes phalaecus	Eudaminae	09-SRNP-33035	MHMYG2415-10	HM885838
6323	Urbanus albimargo	Eudaminae	99-SRNP-6189	CSRII351-04	DQ293631
6324	Urbanus albimargo	Eudaminae	05-SRNP-55418	MHAHF281-06	GU150957
6325	Urbanus albimargo	Eudaminae	97-SRNP-4266	CSCR273-04	DQ293630
6326	Urbanus albimargo	Eudaminae	06-SRNP-2798	MHAHG663-06	GU151802
6327	Urbanus albimargo	Eudaminae	08-SRNP-4797	MHMXX630-09	JF778576

Tree Order	Species	Subfamily	ACG Sampleid	BOLD Processid	Genbank Accession
6328	Urbanus albimargo	Eudaminae	08-SRNP-4799	MHMXX631-09	HQ992120
6329	Urbanus albimargo	Eudaminae	08-SRNP-4798	MHMXX632-09	JF778577
6330	Urbanus doryssusDHJ01	Eudaminae	09-SRNP-65298	MHMYC463-09	GU649870
6331	Urbanus doryssusDHJ01	Eudaminae	08-SRNP-66077	MHMXY957-09	GU666563
6332	Urbanus doryssusDHJ01	Eudaminae	08-SRNP-65793	MHMXY956-09	GU666570
6333	Urbanus doryssusDHJ01	Eudaminae	08-SRNP-32448	MHMXY955-09	GU666569
6334	Urbanus doryssusDHJ01	Eudaminae	08-SRNP-65792	MHMXX633-09	JF778580
6335	Urbanus doryssusDHJ01	Eudaminae	08-SRNP-30716	MHMXW509-09	JF754359
6336	Urbanus doryssusDHJ01	Eudaminae	08-SRNP-65388	MHMXW504-09	JF754358
6337	Urbanus doryssusDHJ01	Eudaminae	08-SRNP-41168	MHMXW498-09	JF754357
6338	Urbanus doryssusDHJ01	Eudaminae	08-SRNP-30697	MHMXW497-09	JF754356
6339	Urbanus doryssusDHJ01	Eudaminae	08-SRNP-65154	MHMXS075-08	JF763333
6340	Urbanus doryssusDHJ01	Eudaminae	07-SRNP-33176	MHMXR801-08	JF763334
6341	Urbanus doryssusDHJ01	Eudaminae	07-SRNP-65317	MHMXR800-08	JF763335
6342	Urbanus doryssusDHJ01	Eudaminae	07-SRNP-32796	MHMXO829-08	JF763336
6343	Urbanus doryssusDHJ01	Eudaminae	07-SRNP-65529	MHMXO828-08	JF763337
6344	Urbanus doryssusDHJ01	Eudaminae	07-SRNP-41431	MHAHL475-07	JF763332
6345	Urbanus doryssusDHJ01	Eudaminae	07-SRNP-32630	MHAHL474-07	JF763331
6346	Urbanus doryssusDHJ01	Eudaminae	07-SRNP-32039	MHAHL473-07	JF763330
6347	Urbanus doryssusDHJ01	Eudaminae	07-SRNP-30997	MHAHL472-07	JF763329
6348	Urbanus doryssusDHJ01	Eudaminae	07-SRNP-21334	MHAHL468-07	JF763326
6349	Urbanus doryssusDHJ01	Eudaminae	02-SRNP-6305	MHAHI309-06	GU156389
6350	Urbanus doryssusDHJ01	Eudaminae	06-SRNP-31223	MHAHH558-06	GU155699
6351	Urbanus doryssusDHJ01	Eudaminae	07-SRNP-32446	MHAHL470-07	JF763327
6352	Urbanus doryssusDHJ01	Eudaminae	04-SRNP-42344	MHAHI310-06	GU156390
6353	Urbanus doryssusDHJ01	Eudaminae	07-SRNP-41459	MHAHL471-07	JF763328
6354	Urbanus doryssusDHJ01	Eudaminae	05-SRNP-70279	MHAHG149-06	GU151827
6355	Urbanus doryssusDHJ01	Eudaminae	09-SRNP-30058	MHMYC465-09	GU649872
6356	Urbanus doryssusDHJ01	Eudaminae	09-SRNP-33053	MHMYC467-09	GU649867
6357	Urbanus doryssusDHJ01	Eudaminae	09-SRNP-31697	MHMYE1489-09	GU653513
6358	Urbanus doryssusDHJ01	Eudaminae	09-SRNP-31399	MHMYE1491-09	GU653511
6359	Urbanus doryssusDHJ01	Eudaminae	09-SRNP-80602	MHMYG2392-10	HM885816
6360	Urbanus doryssusDHJ01	Eudaminae	09-SRNP-32813	MHMYG2393-10	HM885817
6361	Urbanus doryssusDHJ03	Eudaminae	04-SRNP-46471	MHAHC036-05	DQ293718
6362	Urbanus doryssusDHJ03	Eudaminae	04-SRNP-46473	MHAHC052-05	DQ293720
6363	Urbanus doryssusDHJ02	Eudaminae	05-SRNP-2102	MHAHF512-06	GU150982
6364	Urbanus doryssusDHJ02	Eudaminae	05-SRNP-65996	MHAHF758-06	GU150995
6365	Urbanus doryssusDHJ02	Eudaminae	06-SRNP-1298	MHAHG183-06	GU151829
6366	Urbanus doryssusDHJ02	Eudaminae	07-SRNP-59558	MHMXR799-08	JF763340
6367	Urbanus doryssusDHJ02	Eudaminae	04-SRNP-46474	MHAHC029-05	DQ293717
6368	Urbanus doryssusDHJ02	Eudaminae	06-SRNP-3568	MHAHI153-06	GU156391
6369	Urbanus doryssusDHJ02	Eudaminae	09-SRNP-44478	MHMYE1488-09	GU653516
6370	Urbanus doryssusDHJ02	Eudaminae	09-SRNP-44475	MHMYE1490-09	GU653514
6371	Urbanus doryssusDHJ02	Eudaminae	96-SRNP-11042	CSCR277-04	DQ293715
6372	Urbanus doryssusDHJ02	Eudaminae	05-SRNP-2322	MHAHF516-06	GU150985
6373	Urbanus doryssusDHJ02	Eudaminae	09-SRNP-65637	MHMYC466-09	GU649866
6374	Urbanus doryssusDHJ02	Eudaminae	05-SRNP-20746	MHAHF515-06	GU150989
6375	Urbanus doryssusDHJ02	Eudaminae	08-SRNP-1784	MHMXW507-09	JF754367
6376	Urbanus doryssusDHJ02	Eudaminae	08-SRNP-1779	MHMXW508-09	JF754368
6377	Urbanus doryssusDHJ02	Eudaminae	09-SRNP-20147	MHMXY958-09	GU666564
6378	Urbanus doryssusDHJ02	Eudaminae	09-SRNP-65199	MHMYC461-09	GU649876
6379	Urbanus doryssusDHJ02	Eudaminae	08-SRNP-1324	MHMXW502-09	JF754363
6380	Urbanus doryssusDHJ02	Eudaminae	08-SRNP-1771	MHMXW503-09	JF754364
6381	Urbanus doryssusDHJ02	Eudaminae	08-SRNP-1774	MHMXW500-09	JF754361
6382	Urbanus doryssusDHJ02	Eudaminae	08-SRNP-2350	MHMXW501-09	JF754362
6383	Urbanus doryssusDHJ02	Eudaminae	07-SRNP-1310	MHMXK263-07	JF763341

Tree Order	Species	Subfamily	ACG Sampleid	BOLD Processid	Genbank Accession
6384	Urbanus doryssusDHJ02	Eudaminae	07-SRNP-1447	MHAHL467-07	JF763338
6385	Urbanus doryssusDHJ02	Eudaminae	06-SRNP-3275	MHAHI154-06	GU156393
6386	Urbanus doryssusDHJ02	Eudaminae	06-SRNP-3569	MHAHI151-06	GU156395
6387	Urbanus doryssusDHJ02	Eudaminae	06-SRNP-20390	MHAHG184-06	GU151830
6388	Urbanus doryssusDHJ02	Eudaminae	05-SRNP-2104	MHAHF518-06	GU150990
6389	Urbanus doryssusDHJ02	Eudaminae	05-SRNP-2309	MHAHF517-06	GU150992
6390	Urbanus doryssusDHJ02	Eudaminae	05-SRNP-2103	MHAHF514-06	GU150993
6391	Urbanus doryssusDHJ02	Eudaminae	05-SRNP-2105	MHAHF513-06	GU150994
6392	Urbanus doryssusDHJ02	Eudaminae	05-SRNP-46461	MHAHF511-06	GU150984
6393	Urbanus doryssusDHJ02	Eudaminae	05-SRNP-2108	MHAHF510-06	GU150987
6394	Urbanus doryssusDHJ02	Eudaminae	05-SRNP-2339	MHAHF509-06	GU150991
6395	Urbanus doryssusDHJ02	Eudaminae	05-SRNP-46459	MHAHF507-06	GU150983
6396	Urbanus doryssusDHJ02	Eudaminae	05-SRNP-2106	MHAHF506-06	GU150988
6397	Urbanus doryssusDHJ02	Eudaminae	08-SRNP-2185	MHMXW505-09	JF754365
6398	Urbanus doryssusDHJ02	Eudaminae	06-SRNP-3214	MHAHI150-06	GU156392
6399	Urbanus doryssusDHJ02	Eudaminae	08-SRNP-1781	MHMXW506-09	JF754366
6400	Urbanus doryssusDHJ02	Eudaminae	07-SRNP-1739	MHAHL469-07	JF763339
6401	Urbanus doryssusDHJ02	Eudaminae	06-SRNP-1796	MHAHG662-06	GU151828
6402	Urbanus doryssusDHJ02	Eudaminae	93-SRNP-4324	CSRII324-04	DQ293716
6403	Urbanus doryssusDHJ02	Eudaminae	09-SRNP-65198	MHMYC462-09	GU649869
6404	Urbanus doryssusDHJ02	Eudaminae	09-SRNP-65161	MHMYC464-09	GU649871
6405	Urbanus doryssusDHJ02	Eudaminae	09-SRNP-68103	MHMYG2394-10	HM885818
6406	Urbanus doryssusDHJ02	Eudaminae	05-SRNP-58857	MHAHF508-06	GU150986
6407	Urbanus doryssusDHJ02	Eudaminae	04-SRNP-22318	MHAHC044-05	DQ293719
6408	Urbanus doryssusDHJ02	Eudaminae	04-SRNP-22317	MHAHC060-05	DQ293721
6409	Urbanus doryssusDHJ02	Eudaminae	04-SRNP-48319	MHAHD899-05	GU161984
6410	Urbanus doryssusDHJ02	Eudaminae	06-SRNP-3376	MHAHI152-06	GU156394
6411	Urbanus doryssusDHJ02	Eudaminae	08-SRNP-2186	MHMXW499-09	JF754360
6412	Urbanus doryssusDHJ02	Eudaminae	09-SRNP-23060	MHMYG2022-10	HM885424
6413	Urbanus teleus	Eudaminae	01-SRNP-16521	MHAHD406-05	GU162016
6414	Urbanus teleus	Eudaminae	97-SRNP-3309	CSRII346-04	DQ293843
6415	Urbanus teleus	Eudaminae	00-SRNP-4055	MHAHD407-05	GU162017
6416	Urbanus teleus	Eudaminae	00-SRNP-4054	MHAHD408-05	GU162015
6417	Urbanus teleus	Eudaminae	04-SRNP-30475	MHAHE100-05	GU150139
6418	Urbanus teleus	Eudaminae	07-SRNP-55852	MHMXK327-07	JF763394
6419	Urbanus teleus	Eudaminae	07-SRNP-55853	MHMXK328-07	JF763393
6420	Urbanus teleus	Eudaminae	07-SRNP-58338	MHMXO823-08	JF763392
6421	Autochton zarex	Pyrginae	04-SRNP-42624	MHAHE196-05	GU149437
6422	Autochton zarex	Pyrginae	05-SRNP-57363	MHAHE376-05	GU149436
6423	Autochton zarex	Pyrginae	05-SRNP-43733	MHAHG677-06	GU151211
6424	Autochton zarex	Pyrginae	07-SRNP-58889	MHMXP156-08	JF761682
6425	Autochton zarex	Pyrginae	05-SRNP-59312	MHAHE595-06	GU149438
6426	Autochton zarex	Pyrginae	06-SRNP-23079	MHAHJ554-07	JF752479
6427	Autochton zarex	Pyrginae	03-SRNP-8036	CSCR481-04	DQ291904
6428	Autochton zarex	Pyrginae	95-SRNP-7760	CSRII106-04	DQ291905
6429	Autochton zarex	Pyrginae	97-SRNP-5452	CSRII108-04	DQ291907
6430	Autochton zarex	Pyrginae	97-SRNP-4767	CSRII107-04	DQ291906
6431	Autochton zarex	Pyrginae	06-SRNP-22476	MHAHJ714-07	JF752480
6432	Autochton zarex	Pyrginae	08-SRNP-70876	MHMXW510-09	JF753725
6433	Urbanus dorantes	Eudaminae	05-SRNP-34161	MHAHF739-06	GU150980
6434	Urbanus dorantes	Eudaminae	05-SRNP-6828	MHAHF738-06	GU150981
6435	Urbanus dorantes	Eudaminae	07-SRNP-65123	MHAHL462-07	JF763321
6436	Urbanus dorantes	Eudaminae	02-SRNP-1897	CSCR274-04	DQ293713
6437	Urbanus dorantes	Eudaminae	02-SRNP-15449	CSCR275-04	DQ293714
6438	Urbanus dorantes	Eudaminae	06-SRNP-1986	MHAHG670-06	GU151826
6439	Urbanus dorantes	Eudaminae	06-SRNP-30970	MHAHG669-06	GU151825

Tree Order	Species	Subfamily	ACG Sampleid	BOLD Processid	Genbank Accession
6440	Urbanus dorantes	Eudaminae	05-SRNP-59442	MHAHF346-06	GU150978
6441	Urbanus dorantes	Eudaminae	04-SRNP-16100	MHAHE104-05	GU150134
6442	Urbanus dorantes	Eudaminae	05-SRNP-42411	MHAHF345-06	GU150979
6443	Urbanus dorantes	Eudaminae	06-SRNP-3492	MHAHH555-06	GU155698
6444	Urbanus dorantes	Eudaminae	07-SRNP-32150	MHMXK324-07	JF763325
6445	Urbanus dorantes	Eudaminae	07-SRNP-56431	MHMXK325-07	JF763324
6446	Urbanus dorantes	Eudaminae	07-SRNP-45404	MHMXO825-08	JF763323
6447	Urbanus dorantes	Eudaminae	07-SRNP-45405	MHMXO826-08	JF763322
6448	Urbanus dorantes	Eudaminae	08-SRNP-72130	MHMXX629-09	JF778579
6449	Astraptes egregiusDHJ01	Eudaminae	06-SRNP-41788	MHAHH557-06	GU154945
6450	Astraptes egregiusDHJ01	Eudaminae	05-SRNP-40504	MHAHE440-05	GU149423
6451	Astraptes egregiusDHJ01	Eudaminae	04-SRNP-56511	MHAHE101-05	GU149424
6452	Astraptes egregiusDHJ01	Eudaminae	07-SRNP-21401	MHMXN286-07	JF761496
6453	Astraptes egregiusDHJ02	Eudaminae	02-SRNP-15436	CSCR029-04	DQ291865
6454	Astraptes egregiusDHJ02	Eudaminae	03-SRNP-21843	CSRII239-04	DQ291866
6455	Astraptes egregiusDHJ02	Eudaminae	96-SRNP-11827	CSCR028-04	DQ291864
6456	Astraptes egregiusDHJ02	Eudaminae	05-SRNP-59306	MHAHE594-06	GU149425
6457	Astraptes egregiusDHJ02	Eudaminae	06-SRNP-40597	MHAHG672-06	GU151102
6458	Astraptes egregiusDHJ02	Eudaminae	07-SRNP-40219	MHMXK096-07	JF761497
6459	Astraptes egregiusDHJ02	Eudaminae	08-SRNP-36561	MHMXX635-09	JF777605
6460	Urbanus simplicius	Eudaminae	04-SRNP-45191	MHAHE053-05	GU150137
6461	Urbanus simplicius	Eudaminae	04-SRNP-30838	MHAHE051-05	GU150136
6462	Urbanus simplicius	Eudaminae	02-SRNP-5635	CSCR279-04	DQ293842
6463	Urbanus simplicius	Eudaminae	07-SRNP-20188	MHMXK266-07	JF763389
6464	Urbanus simplicius	Eudaminae	07-SRNP-20175	MHMXK265-07	JF763390
6465	Urbanus simplicius	Eudaminae	07-SRNP-20181	MHMXK264-07	JF763391
6466	Urbanus simplicius	Eudaminae	07-SRNP-20078	MHAHK361-07	JF761284
6467	Urbanus simplicius	Eudaminae	06-SRNP-20658	MHAHH571-06	GU155718
6468	Urbanus simplicius	Eudaminae	06-SRNP-58893	MHAHK074-07	JF761283
6469	Urbanus simplicius	Eudaminae	04-SRNP-45789	MHAHE052-05	GU150138
6470	Urbanus simplicius	Eudaminae	02-SRNP-4745	CSCR278-04	DQ293841
6471	Urbanus simplicius	Eudaminae	07-SRNP-20177	MHMXK267-07	JF763388
6472	Urbanus simplicius	Eudaminae	08-SRNP-58533	MHMYC525-09	GU649775
6473	Urbanus simplicius	Eudaminae	08-SRNP-24796	MHMYG2025-10	HM885427
6474	Astraptes aulus	Eudaminae	97-SRNP-10301	CSCR023-04	DQ291858
6475	Astraptes aulus	Eudaminae	06-SRNP-55030	MHAHG109-06	GU151089
6476	Astraptes aulus	Eudaminae	94-SRNP-7852	CSCR022-04	DQ291857
6477	Astraptes aulus	Eudaminae	05-SRNP-12046	MHAHF286-06	GU150205
6478	Astraptes aulus	Eudaminae	05-SRNP-64313	MHAHG653-06	GU151088
6479	Astraptes aulus	Eudaminae	06-SRNP-60359	MHAHK213-07	JF760310
6480	Astraptes janeiraDHJ01	Eudaminae	06-SRNP-6959	MHAHJ891-07	JF752406
6481	Astraptes janeiraDHJ01	Eudaminae	09-SRNP-65806	MHMYC531-09	GU649815
6482	Astraptes janeiraDHJ02	Eudaminae	08-SRNP-65223	MHMXS092-08	JF761587
6483	Astraptes janeiraDHJ02	Eudaminae	08-SRNP-31029	MHMXX951-09	JF777630
6484	Astraptes janeiraDHJ02	Eudaminae	08-SRNP-40223	MHMXX950-09	JF777629
6485	Astraptes janeiraDHJ02	Eudaminae	08-SRNP-65370	MHMXS091-08	JF761588
6486	Astraptes janeiraDHJ02	Eudaminae	04-SRNP-1932	MHAHC058-05	DQ291884
6487	Astraptes janeiraDHJ02	Eudaminae	03-SRNP-21497	CSCR479-04	DQ291882
6488	Astraptes janeiraDHJ02	Eudaminae	05-SRNP-43094	MHAHF667-06	GU150245
6489	Astraptes janeiraDHJ02	Eudaminae	05-SRNP-21164	MHAHF227-06	GU150243
6490	Astraptes janeiraDHJ02	Eudaminae	05-SRNP-21166	MHAHF226-06	GU150242
6491	Astraptes janeiraDHJ02	Eudaminae	99-SRNP-2448	CSCR032-04	DQ291881
6492	Astraptes janeiraDHJ02	Eudaminae	02-SRNP-5607	XAA802-04	DQ291883
6493	Astraptes janeiraDHJ02	Eudaminae	05-SRNP-32361	MHAHF663-06	GU150244
6494	Astraptes janeiraDHJ02	Eudaminae	06-SRNP-2015	MHAHG655-06	GU151127
6495	Astraptes janeiraDHJ02	Eudaminae	09-SRNP-67362	MHMYE1437-09	GU653557

Tree Order	Species	Subfamily	ACG Sampleid	BOLD Processid	Genbank Accession
6496	Astraptes enotrus	Eudaminae	03-SRNP-3278	MHMXU094-08	JF751160
6497	Astraptes enotrus	Eudaminae	08-SRNP-4337	MHMXX693-09	JF777607
6498	Astraptes enotrus	Eudaminae	08-SRNP-66239	MHMXZ011-09	GU665264
6499	Astraptes enotrus	Eudaminae	08-SRNP-2132	MHMXX945-09	JF777621
6500	Astraptes enotrus	Eudaminae	02-SRNP-29258	MHMXU086-08	JF751152
6501	Astraptes enotrus	Eudaminae	08-SRNP-4382	MHMXX692-09	JF777606
6502	Astraptes enotrus	Eudaminae	07-SRNP-45300	MHMXP135-08	JF761506
6503	Astraptes enotrus	Eudaminae	07-SRNP-57189	MHMXP131-08	JF761507
6504	Astraptes enotrus	Eudaminae	07-SRNP-2586	MHAHL514-07	JF761501
6505	Astraptes enotrus	Eudaminae	03-SRNP-8361	MHMXU070-08	JF751137
6506	Astraptes enotrus	Eudaminae	07-SRNP-45178	MHMXK110-07	JF761509
6507	Astraptes enotrus	Eudaminae	03-SRNP-1513	CSCR309-04	DQ291867
6508	Astraptes enotrus	Eudaminae	04-SRNP-32395	MHAHC002-05	DQ291869
6509	Astraptes enotrus	Eudaminae	05-SRNP-31823	MHAHF235-06	GU150217
6510	Astraptes enotrus	Eudaminae	04-SRNP-30579	MHAHC090-05	DQ291876
6511	Astraptes enotrus	Eudaminae	04-SRNP-33020	MHAHC082-05	DQ291875
6512	Astraptes enotrus	Eudaminae	04-SRNP-30578	MHAHC074-05	DQ291874
6513	Astraptes enotrus	Eudaminae	04-SRNP-31311	MHAHC066-05	DQ291873
6514	Astraptes enotrus	Eudaminae	04-SRNP-32345	MHAHC050-05	DQ291872
6515	Astraptes enotrus	Eudaminae	04-SRNP-22616	MHAHC042-05	DQ291871
6516	Astraptes enotrus	Eudaminae	04-SRNP-1431	MHAHC034-05	DQ291870
6517	Astraptes enotrus	Eudaminae	04-SRNP-4633	MHMXU043-08	JF751111
6518	Astraptes enotrus	Eudaminae	01-SRNP-9736	MHMXU090-08	JF751156
6519	Astraptes enotrus	Eudaminae	06-SRNP-23089	MHAHJ500-07	JF752390
6520	Astraptes enotrus	Eudaminae	04-SRNP-47730	MHMXU084-08	JF751151
6521	Astraptes enotrus	Eudaminae	03-SRNP-20571	MHMXU087-08	JF751153
6522	Astraptes enotrus	Eudaminae	03-SRNP-19578	MHMXU088-08	JF751154
6523	Astraptes enotrus	Eudaminae	08-SRNP-2124	MHMXX938-09	JF777614
6524	Astraptes enotrus	Eudaminae	06-SRNP-36179	MHAHK201-07	JF760345
6525	Astraptes enotrus	Eudaminae	01-SRNP-1057	MHMXU058-08	JF751125
6526	Astraptes enotrus	Eudaminae	03-SRNP-10882	MHMXU089-08	JF751155
6527	Astraptes enotrus	Eudaminae	04-SRNP-4407	MHMXU083-08	JF751150
6528	Astraptes enotrus	Eudaminae	04-SRNP-4531	MHMXU060-08	JF751127
6529	Astraptes enotrus	Eudaminae	04-SRNP-15289	MHMXU059-08	JF751126
6530	Astraptes enotrus	Eudaminae	04-SRNP-23231	MHAHC786-05	DQ291877
6531	Astraptes enotrus	Eudaminae	03-SRNP-5824	CSCR310-04	DQ291868
6532	Astraptes enotrus	Eudaminae	06-SRNP-40208	MHAHG126-06	GU151104
6533	Astraptes enotrus	Eudaminae	07-SRNP-31387	MHAHL516-07	JF761503
6534	Astraptes enotrus	Eudaminae	95-SRNP-7803	MHMXU062-08	JF751129
6535	Astraptes enotrus	Eudaminae	99-SRNP-4986	MHMXU069-08	JF751136
6536	Astraptes enotrus	Eudaminae	00-SRNP-1877	MHMXU077-08	JF751144
6537	Astraptes enotrus	Eudaminae	98-SRNP-4808	MHMXU068-08	JF751135
6538	Astraptes enotrus	Eudaminae	02-SRNP-18995	MHMXU076-08	JF751143
6539	Astraptes enotrus	Eudaminae	07-SRNP-35000	MHAHK233-07	JF760346
6540	Astraptes enotrus	Eudaminae	03-SRNP-20573	MHMXU078-08	JF751145
6541	Astraptes enotrus	Eudaminae	06-SRNP-23433	MHMXK104-07	JF761512
6542	Astraptes enotrus	Eudaminae	03-SRNP-5834	MHMXU091-08	JF751157
6543	Astraptes enotrus	Eudaminae	08-SRNP-1658	MHMXX939-09	JF777615
6544	Astraptes enotrus	Eudaminae	08-SRNP-65001	MHMXT236-08	JF761504
6545	Astraptes enotrus	Eudaminae	04-SRNP-23876	MHMXU040-08	JF751108
6546	Astraptes enotrus	Eudaminae	04-SRNP-48434	MHMXU041-08	JF751109
6547	Astraptes enotrus	Eudaminae	04-SRNP-48802	MHMXU042-08	JF751110
6548	Astraptes enotrus	Eudaminae	04-SRNP-4532	MHMXU044-08	JF751112
6549	Astraptes enotrus	Eudaminae	03-SRNP-37547	MHMXU045-08	JF751113
6550	Astraptes enotrus	Eudaminae	02-SRNP-30318	MHMXU047-08	JF751114
6551	Astraptes enotrus	Eudaminae	03-SRNP-23940	MHMXU048-08	JF751115

Tree Order	Species	Subfamily	ACG Sampleid	BOLD Processid	Genbank Accession
6552	Astraptes enotrus	Eudaminae	07-SRNP-30922	MHMXK108-07	JF761511
6553	Astraptes enotrus	Eudaminae	07-SRNP-895	MHMXK109-07	JF761510
6554	Astraptes enotrus	Eudaminae	06-SRNP-23363	MHMXK111-07	JF761508
6555	Astraptes enotrus	Eudaminae	07-SRNP-32164	MHAHL511-07	JF761498
6556	Astraptes enotrus	Eudaminae	07-SRNP-31091	MHAHL512-07	JF761499
6557	Astraptes enotrus	Eudaminae	07-SRNP-40850	MHAHL513-07	JF761500
6558	Astraptes enotrus	Eudaminae	07-SRNP-2585	MHAHL515-07	JF761502
6559	Astraptes enotrus	Eudaminae	07-SRNP-23362	MHMXR846-08	JF761505
6560	Astraptes enotrus	Eudaminae	03-SRNP-4337	MHMXU049-08	JF751116
6561	Astraptes enotrus	Eudaminae	00-SRNP-3195	MHMXU050-08	JF751117
6562	Astraptes enotrus	Eudaminae	03-SRNP-20168	MHMXU051-08	JF751118
6563	Astraptes enotrus	Eudaminae	03-SRNP-5007	MHMXU052-08	JF751119
6564	Astraptes enotrus	Eudaminae	03-SRNP-38023	MHMXU053-08	JF751120
6565	Astraptes enotrus	Eudaminae	01-SRNP-1374	MHMXU054-08	JF751121
6566	Astraptes enotrus	Eudaminae	01-SRNP-578	MHMXU055-08	JF751122
6567	Astraptes enotrus	Eudaminae	04-SRNP-4535	MHMXU056-08	JF751123
6568	Astraptes enotrus	Eudaminae	04-SRNP-15007	MHMXU057-08	JF751124
6569	Astraptes enotrus	Eudaminae	04-SRNP-61500	MHMXU061-08	JF751128
6570	Astraptes enotrus	Eudaminae	04-SRNP-55371	MHMXU063-08	JF751130
6571	Astraptes enotrus	Eudaminae	02-SRNP-29261	MHMXU064-08	JF751131
6572	Astraptes enotrus	Eudaminae	02-SRNP-4701	MHMXU065-08	JF751132
6573	Astraptes enotrus	Eudaminae	02-SRNP-29260	MHMXU066-08	JF751133
6574	Astraptes enotrus	Eudaminae	02-SRNP-18992	MHMXU067-08	JF751134
6575	Astraptes enotrus	Eudaminae	04-SRNP-14410	MHMXU071-08	JF751138
6576	Astraptes enotrus	Eudaminae	02-SRNP-19031	MHMXU072-08	JF751139
6577	Astraptes enotrus	Eudaminae	02-SRNP-4310	MHMXU073-08	JF751140
6578	Astraptes enotrus	Eudaminae	00-SRNP-12241	MHMXU074-08	JF751141
6579	Astraptes enotrus	Eudaminae	02-SRNP-29259	MHMXU075-08	JF751142
6580	Astraptes enotrus	Eudaminae	03-SRNP-21678	MHMXU079-08	JF751146
6581	Astraptes enotrus	Eudaminae	03-SRNP-27838	MHMXU080-08	JF751147
6582	Astraptes enotrus	Eudaminae	01-SRNP-569	MHMXU081-08	JF751148
6583	Astraptes enotrus	Eudaminae	04-SRNP-16060	MHMXU082-08	JF751149
6584	Astraptes enotrus	Eudaminae	03-SRNP-7340	MHMXU092-08	JF751158
6585	Astraptes enotrus	Eudaminae	03-SRNP-1596	MHMXU093-08	JF751159
6586	Astraptes enotrus	Eudaminae	07-SRNP-60787	MHMXX933-09	JF777609
6587	Astraptes enotrus	Eudaminae	08-SRNP-65002	MHMXX934-09	JF777610
6588	Astraptes enotrus	Eudaminae	08-SRNP-30981	MHMXX935-09	JF777611
6589	Astraptes enotrus	Eudaminae	08-SRNP-40360	MHMXX936-09	JF777612
6590	Astraptes enotrus	Eudaminae	08-SRNP-2125	MHMXX937-09	JF777613
6591	Astraptes enotrus	Eudaminae	08-SRNP-2321	MHMXX940-09	JF777616
6592	Astraptes enotrus	Eudaminae	08-SRNP-65460	MHMXX941-09	JF777617
6593	Astraptes enotrus	Eudaminae	08-SRNP-65411	MHMXX942-09	JF777618
6594	Astraptes enotrus	Eudaminae	08-SRNP-20582	MHMXX943-09	JF777619
6595	Astraptes enotrus	Eudaminae	08-SRNP-20893	MHMXX944-09	JF777620
6596	Astraptes enotrus	Eudaminae	08-SRNP-1656	MHMXX946-09	JF777622
6597	Astraptes enotrus	Eudaminae	08-SRNP-40359	MHMXX947-09	JF777623
6598	Astraptes enotrus	Eudaminae	08-SRNP-1657	MHMXX948-09	JF777624
6599	Astraptes enotrus	Eudaminae	08-SRNP-56038	MHMXX694-09	JF777608
6600	Astraptes enotrus	Eudaminae	05-SRNP-30647	MHAHF248-06	GU150220
6601	Astraptes enotrus	Eudaminae	05-SRNP-30648	MHAHF249-06	GU150218
6602	Astraptes enotrus	Eudaminae	05-SRNP-791	MHAHF252-06	GU150219
6603	Astraptes enotrus	Eudaminae	05-SRNP-30598	MHAHF254-06	GU150221
6604	Astraptes enotrus	Eudaminae	09-SRNP-32555	MHMYE1467-09	GU653533
6605	Astraptes enotrus	Eudaminae	09-SRNP-20260	MHMYG2471-10	HM885898
6606	Astraptes enotrus	Eudaminae	06-SRNP-2122	MHAHG654-06	GU151103
6607	Astraptes enotrus	Eudaminae	06-SRNP-32266	MHAHI107-06	GU155806

Tree Order	Species	Subfamily	ACG Sampleid	BOLD Processid	Genbank Accession
6608	Astraptes enotrus	Eudaminae	06-SRNP-35215	MHAHI111-06	GU155805
6609	Astraptes enotrus	Eudaminae	06-SRNP-22782	MHAHJ652-07	JF752391
6610	Astraptes enotrus	Eudaminae	09-SRNP-22799	MHMYG2472-10	HM885899
6611	Astraptes enotrus	Eudaminae	09-SRNP-20286	MHMYG2506-10	HM885935
6612	Chioides catillus	Eudaminae	07-SRNP-57955	MHMXO831-08	JF761930
6613	Chioides catillus	Eudaminae	07-SRNP-20324	MHMXK320-07	JF761935
6614	Chioides catillus	Eudaminae	06-SRNP-21086	MHAHH554-06	GU155330
6615	Chioides catillus	Eudaminae	07-SRNP-938	MHMXO833-08	JF761928
6616	Chioides catillus	Eudaminae	07-SRNP-58548	MHMXR792-08	JF761927
6617	Chioides catillus	Eudaminae	07-SRNP-32811	MHMXO830-08	JF761931
6618	Chioides catillus	Eudaminae	07-SRNP-939	MHMXO832-08	JF761929
6619	Chioides catillus	Eudaminae	07-SRNP-20124	MHMXK323-07	JF761932
6620	Chioides catillus	Eudaminae	07-SRNP-22429	MHAHL465-07	JF761925
6621	Chioides catillus	Eudaminae	07-SRNP-20115	MHMXK322-07	JF761933
6622	Chioides catillus	Eudaminae	07-SRNP-20187	MHMXK321-07	JF761934
6623	Chioides catillus	Eudaminae	07-SRNP-20128	MHMXK319-07	JF761936
6624	Chioides catillus	Eudaminae	07-SRNP-45146	MHAHK073-07	JF760538
6625	Chioides catillus	Eudaminae	05-SRNP-45682	MHAHF343-06	GU150333
6626	Chioides catillus	Eudaminae	04-SRNP-34914	MHAHE050-05	GU149531
6627	Chioides catillus	Eudaminae	04-SRNP-56106	MHAHE049-05	GU149533
6628	Chioides catillus	Eudaminae	04-SRNP-23839	MHAHC295-05	DQ292124
6629	Chioides catillus	Eudaminae	04-SRNP-34756	MHAHC285-05	DQ292122
6630	Chioides catillus	Eudaminae	04-SRNP-23173	MHAHC293-05	DQ292123
6631	Chioides catillus	Eudaminae	04-SRNP-24064	MHAHC297-05	DQ292125
6632	Chioides catillus	Eudaminae	03-SRNP-1704	CSCR342-04	DQ292121
6633	Chioides catillus	Eudaminae	04-SRNP-23511	MHAHE048-05	GU149532
6634	Chioides catillus	Eudaminae	03-SRNP-1498	CSCR341-04	DQ292120
6635	Chioides catillus	Eudaminae	05-SRNP-31764	MHAHF344-06	GU150332
6636	Chioides catillus	Eudaminae	07-SRNP-58581	MHMXR793-08	JF761926
6637	Chioides zilpa	Eudaminae	02-SRNP-10079	CSCR068-04	DQ292127
6638	Chioides zilpa	Eudaminae	07-SRNP-45681	MHMXR795-08	JF761938
6639	Chioides zilpa	Eudaminae	04-SRNP-45690	MHAHE103-05	GU149534
6640	Chioides zilpa	Eudaminae	04-SRNP-45597	MHAHC021-05	DQ292130
6641	Chioides zilpa	Eudaminae	04-SRNP-45616	MHAHC013-05	DQ292129
6642	Chioides zilpa	Eudaminae	04-SRNP-45596	MHAHC005-05	DQ292128
6643	Chioides zilpa	Eudaminae	04-SRNP-45594	MHAHC093-05	DQ292134
6644	Chioides zilpa	Eudaminae	04-SRNP-45618	MHAHC085-05	DQ292133
6645	Chioides zilpa	Eudaminae	04-SRNP-45595	MHAHC077-05	DQ292132
6646	Chioides zilpa	Eudaminae	04-SRNP-45749	MHAHC069-05	DQ292131
6647	Chioides zilpa	Eudaminae	02-SRNP-10078	CSCR067-04	DQ292126
6648	Chioides zilpa	Eudaminae	07-SRNP-45680	MHMXR796-08	JF761937
6649	Chioides zilpa	Eudaminae	07-SRNP-45864	MHMXO827-08	JF761939
6650	Chioides zilpa	Eudaminae	09-SRNP-57613	MHMYE1442-09	GU653553
6651	Epargyreus Burns04	Pyrginae	02-SRNP-29831	CSCR535-04	DQ292483
6652	Epargyreus Burns04	Pyrginae	02-SRNP-29832	CSCR536-04	DQ292484
6653	Epargyreus Burns04	Pyrginae	01-SRNP-14765	CSCR526-04	DQ292480
6654	Epargyreus Burns04	Pyrginae	03-SRNP-30086	MHAHD488-05	GU161506
6655	Epargyreus Burns04	Pyrginae	03-SRNP-29910	MHAHD489-05	GU161505
6656	Epargyreus Burns04	Pyrginae	03-SRNP-29907	MHAHD490-05	GU161504
6657	Epargyreus Burns04	Pyrginae	01-SRNP-17907	MHAHD492-05	GU161501
6658	Epargyreus Burns05	Pyrginae	02-SRNP-4752	MHAHD474-05	GU161509
6659	Epargyreus Burns04	Pyrginae	03-SRNP-29911	MHAHD487-05	GU161503
6660	Epargyreus Burns04	Pyrginae	07-SRNP-45120	MHMXK133-07	JF762209
6661	Epargyreus Burns04	Pyrginae	02-SRNP-16314	CSCR534-04	DQ292482
6662	Epargyreus Burns04	Pyrginae	04-SRNP-24319	MHAHC771-05	DQ292485
6663	Epargyreus Burns04	Pyrginae	96-SRNP-9218	CSCR527-04	DQ292481

Tree Order	Species	Subfamily	ACG Sampleid	BOLD Processid	Genbank Accession
6664	Epargyreus Burns04	Pyrginae	01-SRNP-16938	MHAHD491-05	GU161502
6665	Epargyreus Burns04	Pyrginae	07-SRNP-20185	MHMXK135-07	JF762208
6666	Epargyreus Burns04	Pyrginae	07-SRNP-20050	MHMXK139-07	JF762207
6667	Epargyreus Burns04	Pyrginae	07-SRNP-20051	MHMXN324-07	JF762206
6668	Epargyreus Burns07	Pyrginae	07-SRNP-21582	MHMXN331-07	JF762218
6669	Epargyreus Burns07	Pyrginae	07-SRNP-21586	MHMXN332-07	JF762217
6670	Epargyreus Burns07	Pyrginae	04-SRNP-55660	MHAHC770-05	DQ292497
6671	Epargyreus Burns07	Pyrginae	05-SRNP-30037	MHAHL074-07	JF762214
6672	Epargyreus Burns07	Pyrginae	05-SRNP-21988	MHAHL078-07	JF762215
6673	Epargyreus Burns07	Pyrginae	04-SRNP-15059	MHAHC784-05	DQ292498
6674	Epargyreus Burns07	Pyrginae	04-SRNP-1184	MHAHC757-05	DQ292496
6675	Epargyreus Burns07	Pyrginae	02-SRNP-16668	CSCR533-04	DQ292495
6676	Epargyreus Burns07	Pyrginae	02-SRNP-16077	CSCR532-04	DQ292494
6677	Epargyreus Burns07	Pyrginae	06-SRNP-32932	MHAHI508-06	GU155963
6678	Epargyreus Burns07	Pyrginae	07-SRNP-21670	MHMXN333-07	JF762216
6679	Epargyreus Burns07	Pyrginae	08-SRNP-2670	MHMXX974-09	JF777919
6680	Epargyreus Burns07	Pyrginae	08-SRNP-31981	MHMXY1118-09	GU666425
6681	Epargyreus Burns05	Pyrginae	06-SRNP-46832	MHAHJ620-07	JF752772
6682	Epargyreus Burns05	Pyrginae	06-SRNP-46827	MHAHJ619-07	JF752771
6683	Epargyreus Burns05	Pyrginae	02-SRNP-29177	MHAHD478-05	GU161510
6684	Epargyreus Burns05	Pyrginae	02-SRNP-29170	MHAHD475-05	GU161507
6685	Epargyreus Burns05	Pyrginae	02-SRNP-29171	MHAHD473-05	GU161514
6686	Epargyreus Burns05	Pyrginae	02-SRNP-4753	MHAHD471-05	GU161511
6687	Epargyreus Burns05	Pyrginae	02-SRNP-5328	CSCR529-04	DQ292488
6688	Epargyreus Burns05	Pyrginae	02-SRNP-17323	CSCR460-04	DQ292486
6689	Epargyreus Burns05	Pyrginae	02-SRNP-4118	MHAHD472-05	GU161513
6690	Epargyreus Burns05	Pyrginae	02-SRNP-15163	MHAHD476-05	GU161512
6691	Epargyreus Burns05	Pyrginae	07-SRNP-56896	MHMXN325-07	JF762211
6692	Epargyreus Burns05	Pyrginae	07-SRNP-46910	MHMXX973-09	JF777917
6693	Epargyreus Burns05	Pyrginae	05-SRNP-65949	MHAHL066-07	JF762210
6694	Epargyreus Burns05	Pyrginae	02-SRNP-28000	CSCR528-04	DQ292487
6695	Epargyreus Burns05	Pyrginae	02-SRNP-4513	CSCR538-04	DQ292489
6696	Epargyreus Burns05	Pyrginae	04-SRNP-14225	MHAHC763-05	DQ292490
6697	Epargyreus Burns05	Pyrginae	02-SRNP-29175	MHAHD477-05	GU161508
6698	Epargyreus Burns05	Pyrginae	08-SRNP-15158	MHMXY1119-09	GU666426
6699	Epargyreus Burns06	Pyrginae	97-SRNP-1659	CSCR531-04	DQ292492
6700	Epargyreus Burns06	Pyrginae	02-SRNP-8179	CSCR530-04	DQ292491
6701	Epargyreus Burns06	Pyrginae	03-SRNP-20073	CSRII397-04	DQ292493
6702	Epargyreus Burns06	Pyrginae	07-SRNP-1276	MHMXK134-07	JF762213
6703	Epargyreus Burns06	Pyrginae	07-SRNP-427	MHMXK141-07	JF762212
6704	Epargyreus Burns06	Pyrginae	08-SRNP-1110	MHMXX1179-09	JF777918
6705	Epargyreus Burns11	Pyrginae	01-SRNP-16427	CSRII364-04	DQ292439
6706	Epargyreus Burns11	Pyrginae	07-SRNP-20344	MHMXK140-07	JF762227
6707	Epargyreus Burns11	Pyrginae	07-SRNP-20063	MHMXK138-07	JF762228
6708	Epargyreus Burns11	Pyrginae	04-SRNP-14219	MHAHC762-05	DQ292442
6709	Epargyreus Burns11	Pyrginae	07-SRNP-20341	MHMXR804-08	JF762222
6710	Epargyreus Burns11	Pyrginae	07-SRNP-23521	MHMXR803-08	JF762223
6711	Epargyreus Burns11	Pyrginae	05-SRNP-45151	MHAHL075-07	JF762219
6712	Epargyreus Burns11	Pyrginae	07-SRNP-20334	MHMXK136-07	JF762229
6713	Epargyreus Burns11	Pyrginae	05-SRNP-45152	MHAHL081-07	JF762220
6714	Epargyreus Burns11	Pyrginae	07-SRNP-20333	MHMXN327-07	JF762225
6715	Epargyreus Burns11	Pyrginae	06-SRNP-67531	MHAHK224-07	JF760675
6716	Epargyreus Burns11	Pyrginae	04-SRNP-21554	MHAHC754-05	DQ292441
6717	Epargyreus Burns11	Pyrginae	01-SRNP-16541	CSRII365-04	DQ292440
6718	Epargyreus Burns11	Pyrginae	06-SRNP-58925	MHAHK225-07	JF760676
6719	Epargyreus Burns11	Pyrginae	07-SRNP-20296	MHMXN326-07	JF762226

Tree Order	Species	Subfamily	ACG Sampleid	BOLD Processid	Genbank Accession
6720	Epargyreus Burns11	Pyrginae	07-SRNP-20364	MHMXN328-07	JF762224
6721	Epargyreus Burns11	Pyrginae	07-SRNP-20329	MHMXT220-08	JF762221
6722	Epargyreus Burns11	Pyrginae	09-SRNP-55106	MHMYE895-09	GU653709
6723	Epargyreus Burns12	Pyrginae	08-SRNP-24800	MHMYB153-09	GU649684
6724	Epargyreus Burns12	Pyrginae	07-SRNP-46132	MHMXT219-08	JF762230
6725	Epargyreus Burns12	Pyrginae	07-SRNP-21329	MHMXN329-07	JF762232
6726	Epargyreus Burns12	Pyrginae	07-SRNP-65538	MHMXR802-08	JF762231
6727	Epargyreus Burns12	Pyrginae	08-SRNP-24801	MHMYB154-09	GU649685
6728	Epargyreus Burns03	Pyrginae	08-SRNP-45027	MHMXX968-09	JF777916
6729	Epargyreus Burns03	Pyrginae	07-SRNP-57869	MHMXP140-08	JF762204
6730	Epargyreus Burns03	Pyrginae	94-SRNP-8731	CSRII331-04	DQ292464
6731	Epargyreus Burns03	Pyrginae	04-SRNP-45025	MHAHC761-05	DQ292471
6732	Epargyreus Burns03	Pyrginae	04-SRNP-47759	MHAHC752-05	DQ292468
6733	Epargyreus Burns03	Pyrginae	07-SRNP-57031	MHMXN330-07	JF762205
6734	Epargyreus Burns03	Pyrginae	05-SRNP-45698	MHAHL064-07	JF762203
6735	Epargyreus Burns03	Pyrginae	06-SRNP-47189	MHAHJ626-07	JF752770
6736	Epargyreus Burns03	Pyrginae	04-SRNP-46436	MHAHC782-05	DQ292479
6737	Epargyreus Burns03	Pyrginae	04-SRNP-46435	MHAHC778-05	DQ292478
6738	Epargyreus Burns03	Pyrginae	04-SRNP-46800	MHAHC777-05	DQ292477
6739	Epargyreus Burns03	Pyrginae	04-SRNP-46437	MHAHC776-05	DQ292476
6740	Epargyreus Burns03	Pyrginae	04-SRNP-46819	MHAHC775-05	DQ292475
6741	Epargyreus Burns03	Pyrginae	04-SRNP-48332	MHAHC773-05	DQ292474
6742	Epargyreus Burns03	Pyrginae	04-SRNP-47032	MHAHC767-05	DQ292473
6743	Epargyreus Burns03	Pyrginae	04-SRNP-47031	MHAHC766-05	DQ292472
6744	Epargyreus Burns03	Pyrginae	04-SRNP-46799	MHAHC756-05	DQ292470
6745	Epargyreus Burns03	Pyrginae	04-SRNP-46820	MHAHC755-05	DQ292469
6746	Epargyreus Burns03	Pyrginae	94-SRNP-8741	CSRII332-04	DQ292465
6747	Epargyreus Burns03	Pyrginae	96-SRNP-10052	CSRII344-04	DQ292466
6748	Epargyreus Burns03	Pyrginae	04-SRNP-48150	MHAHC751-05	DQ292467
6749	Epargyreus Burns03	Pyrginae	08-SRNP-58522	MHMYC530-09	GU649814
6750	Epargyreus Burns02	Pyrginae	01-SRNP-3183	CSCR516-04	DQ292445
6751	Epargyreus Burns02	Pyrginae	02-SRNP-29202	CSCR519-04	DQ292448
6752	Epargyreus Burns02	Pyrginae	05-SRNP-30057	MHAHL069-07	JF762197
6753	Epargyreus Burns02	Pyrginae	04-SRNP-27300	MHAHL080-07	JF762200
6754	Epargyreus Burns02	Pyrginae	07-SRNP-61270	MHMXX969-09	JF777915
6755	Epargyreus Burns02	Pyrginae	08-SRNP-4758	MHMXX655-09	JF777912
6756	Epargyreus Burns02	Pyrginae	09-SRNP-71932	MHMYE1443-09	GU653554
6757	Epargyreus Burns02	Pyrginae	01-SRNP-18804	CSCR515-04	DQ292444
6758	Epargyreus Burns02	Pyrginae	01-SRNP-18806	CSCR459-04	DQ292443
6759	Epargyreus Burns02	Pyrginae	02-SRNP-15408	CSCR517-04	DQ292446
6760	Epargyreus Burns02	Pyrginae	08-SRNP-72029	MHMXX656-09	JF777913
6761	Epargyreus Burns02	Pyrginae	04-SRNP-15015	MHAHC783-05	DQ292462
6762	Epargyreus Burns02	Pyrginae	07-SRNP-57672	MHMXP141-08	JF762202
6763	Epargyreus Burns02	Pyrginae	04-SRNP-15177	MHAHC769-05	DQ292457
6764	Epargyreus Burns02	Pyrginae	05-SRNP-33218	MHAHL062-07	JF762193
6765	Epargyreus Burns02	Pyrginae	07-SRNP-42506	MHMXR805-08	JF762201
6766	Epargyreus Burns02	Pyrginae	02-SRNP-524	CSCR521-04	DQ292450
6767	Epargyreus Burns02	Pyrginae	02-SRNP-525	CSCR522-04	DQ292451
6768	Epargyreus Burns02	Pyrginae	04-SRNP-24265	MHAHC780-05	DQ292460
6769	Epargyreus Burns02	Pyrginae	92-SRNP-2367	CSCR523-04	DQ292452
6770	Epargyreus Burns02	Pyrginae	05-SRNP-2701	MHAHL077-07	JF762199
6771	Epargyreus Burns02	Pyrginae	04-SRNP-13417	MHAHC759-05	DQ292454
6772	Epargyreus Burns02	Pyrginae	04-SRNP-24405	MHAHC758-05	DQ292453
6773	Epargyreus Burns02	Pyrginae	02-SRNP-29200	CSCR518-04	DQ292447
6774	Epargyreus Burns02	Pyrginae	02-SRNP-32945	CSCR520-04	DQ292449
6775	Epargyreus Burns02	Pyrginae	04-SRNP-14174	MHAHC764-05	DQ292455

Tree Order	Species	Subfamily	ACG Sampleid	BOLD Processid	Genbank Accession
6776	Epargyreus Burns02	Pyrginae	04-SRNP-15077	MHAHC768-05	DQ292456
6777	Epargyreus Burns02	Pyrginae	04-SRNP-15090	MHAHC772-05	DQ292458
6778	Epargyreus Burns02	Pyrginae	04-SRNP-15384	MHAHC779-05	DQ292459
6779	Epargyreus Burns02	Pyrginae	04-SRNP-4655	MHAHC781-05	DQ292461
6780	Epargyreus Burns02	Pyrginae	04-SRNP-15720	MHAHC785-05	DQ292463
6781	Epargyreus Burns02	Pyrginae	06-SRNP-43439	MHAHI506-06	GU155961
6782	Epargyreus Burns02	Pyrginae	06-SRNP-43442	MHAHI507-06	GU155962
6783	Epargyreus Burns02	Pyrginae	06-SRNP-43440	MHAHJ680-07	JF752769
6784	Epargyreus Burns02	Pyrginae	05-SRNP-32694	MHAHL063-07	JF762194
6785	Epargyreus Burns02	Pyrginae	05-SRNP-1416	MHAHL067-07	JF762195
6786	Epargyreus Burns02	Pyrginae	04-SRNP-27299	MHAHL068-07	JF762196
6787	Epargyreus Burns02	Pyrginae	05-SRNP-92	MHAHL076-07	JF762198
6788	Epargyreus Burns02	Pyrginae	08-SRNP-4814	MHMXX654-09	JF777911
6789	Epargyreus Burns02	Pyrginae	08-SRNP-4777	MHMXX657-09	JF777914
6790	Epargyreus Burns02	Pyrginae	09-SRNP-72516	MHMYE1444-09	GU653551
6791	Epargyreus Burns02	Pyrginae	09-SRNP-73319	MHMYG2465-10	HM885892
6792	Epargyreus Burns02	Pyrginae	09-SRNP-76379	MHMYG2466-10	HM885893
6793	Epargyreus Burns02	Pyrginae	09-SRNP-75608	MHMYG2468-10	HM885894
6794	Codatractus imalena	Pyrginae	00-SRNP-10752	XAA724-04	N/A
6795	Codatractus imalena	Pyrginae	97-SRNP-1645	XAA735-04	
6796	Codatractus imalena	Pyrginae	02-SRNP-24526	CSCR074-04	DQ292186
6797	Codatractus imalena	Pyrginae	01-SRNP-7390	XAA719-04	DQ292191
6798	Codatractus imalena	Pyrginae	00-SRNP-10734	XAA720-04	DQ292192
6799	Codatractus imalena	Pyrginae	04-SRNP-35578	MHAHD711-05	GU161359
6800	Codatractus imalena	Pyrginae	01-SRNP-7384	XAA718-04	DQ292190
6801	Codatractus imalena	Pyrginae	98-SRNP-3451	XAA734-04	DQ292202
6802	Codatractus imalena	Pyrginae	99-SRNP-5496	XAA716-04	DQ292188
6803	Codatractus melon	Pyrginae	92-SRNP-3686	CSRII443-04	GU161362
6804	Codatractus imalena	Pyrginae	00-SRNP-9613	XAA733-04	DQ292201
6805	Codatractus imalena	Pyrginae	00-SRNP-10005	XAA722-04	DQ292194
6806	Codatractus imalena	Pyrginae	03-SRNP-4322	XAA715-04	DQ292187
6807	Codatractus imalena	Pyrginae	00-SRNP-10020	XAA732-04	DQ292200
6808	Codatractus imalena	Pyrginae	01-SRNP-21206	XAA728-04	DQ292198
6809	Codatractus imalena	Pyrginae	00-SRNP-9747	XAA731-04	DQ292199
6810	Codatractus imalena	Pyrginae	01-SRNP-21186	XAA717-04	DQ292189
6811	Codatractus imalena	Pyrginae	01-SRNP-7119	XAA727-04	DQ292197
6812	Codatractus imalena	Pyrginae	01-SRNP-7392	XAA721-04	DQ292193
6813	Codatractus imalena	Pyrginae	04-SRNP-46325	MHAHC067-05	DQ292205
6814	Codatractus imalena	Pyrginae	04-SRNP-46730	MHAHC075-05	DQ292206
6815	Codatractus imalena	Pyrginae	04-SRNP-35580	MHAHD710-05	GU161360
6816	Codatractus imalena	Pyrginae	04-SRNP-35579	MHAHD712-05	GU161361
6817	Codatractus imalena	Pyrginae	04-SRNP-46729	MHAHC043-05	DQ292203
6818	Codatractus imalena	Pyrginae	04-SRNP-47215	MHAHC059-05	DQ292204
6819	Codatractus imalena	Pyrginae	08-SRNP-35884	MHMXX976-09	JF777791
6820	Codatractus imalena	Pyrginae	01-SRNP-21138	CSRII442-04	GU161358
6821	Codatractus melon	Pyrginae	93-SRNP-5542	CSRII756-05	DQ292209
6822	Codatractus melon	Pyrginae	93-SRNP-5545	CSRII755-05	DQ292208
6823	Codatractus melon	Pyrginae	92-SRNP-3678	CSRII758-05	DQ292210
6824	Codatractus melon	Pyrginae	90-SRNP-1878	CSRII759-05	DQ292211
6825	Codatractus melon	Pyrginae	92-SRNP-3626	CSRII760-05	DQ292212
6826	Codatractus melon	Pyrginae	93-SRNP-3061	CSRII444-04	DQ292207
6827	Codatractus melon	Pyrginae	93-SRNP-5544	CSRII761-05	DQ292213
6828	Codatractus alcaeus	Pyrginae	01-SRNP-16125	CSRII440-04	GU161355
6829	Codatractus alcaeus	Pyrginae	99-SRNP-2489	CSRII439-04	GU161356
6830	Codatractus carlos	Pyrginae	06-SRNP-2517	MHAHG673-06	GU151317
6831	Codatractus imalena	Pyrginae	00-SRNP-10506	CSRII441-04	GU161357

Tree Order	Species	Subfamily	ACG Sampleid	BOLD Processid	Genbank Accession
6832	Codatractus alcaeus	Pyrginae	04-SRNP-45206	MHAHC051-05	DQ292185
6833	Codatractus imalena	Pyrginae	09-SRNP-12121	MHMYG2038-10	HM885440
6834	Codatractus imalena	Pyrginae	09-SRNP-12120	MHMYG2039-10	HM885441
6835	Ridens mephitisDHJ02	Pyrginae	04-SRNP-35805	MHAHF245-06	GU150758
6836	Ridens mephitisDHJ02	Pyrginae	04-SRNP-36233	MHAHD390-05	GU161831
6837	Ridens mephitisDHJ02	Pyrginae	02-SRNP-8370	XAA933-04	DQ293224
6838	Ridens mephitisDHJ02	Pyrginae	03-SRNP-4203	CSRII471-04	DQ293220
6839	Ridens mephitisDHJ02	Pyrginae	00-SRNP-9345	XAA901-04	DQ293222
6840	Ridens mephitisDHJ02	Pyrginae	03-SRNP-3199	CSCR410-04	DQ293219
6841	Ridens mephitisDHJ02	Pyrginae	02-SRNP-23638	XAA925-04	DQ293223
6842	Ridens mephitisDHJ02	Pyrginae	99-SRNP-1768	XAA892-04	DQ293221
6843	Ridens mephitisDHJ02	Pyrginae	05-SRNP-36028	MHAHG124-06	GU151607
6844	Ridens mephitisDHJ02	Pyrginae	05-SRNP-36029	MHAHG125-06	GU151608
6845	Ridens mephitisDHJ04	Pyrginae	06-SRNP-60255	MHMXK105-07	JF762800
6846	Ridens mephitisDHJ04	Pyrginae	06-SRNP-60252	MHAHK207-07	JF761118
6847	Ridens mephitisDHJ04	Pyrginae	93-SRNP-3068	XAA909-04	DQ293234
6848	Ridens mephitisDHJ04	Pyrginae	92-SRNP-5754	XAA917-04	DQ293235
6849	Ridens mephitisDHJ04	Pyrginae	06-SRNP-59545	MHAHK205-07	JF761117
6850	Ridens mephitisDHJ04	Pyrginae	08-SRNP-57638	MHMXX703-09	JF778436
6851	Ridens mephitisDHJ04	Pyrginae	08-SRNP-57623	MHMXX704-09	JF778437
6852	Ridens mephitisDHJ04	Pyrginae	08-SRNP-57620	MHMXX705-09	JF778438
6853	Ridens mephitisDHJ04	Pyrginae	08-SRNP-57621	MHMXX706-09	JF778439
6854	Ridens mephitisDHJ03	Pyrginae	07-SRNP-56610	MHMXP130-08	JF762796
6855	Ridens mephitisDHJ03	Pyrginae	99-SRNP-10376	XAA876-04	DQ293229
6856	Ridens mephitisDHJ03	Pyrginae	08-SRNP-57249	MHMXX700-09	JF778432
6857	Ridens mephitisDHJ03	Pyrginae	06-SRNP-57708	MHAHK203-07	JF761111
6858	Ridens mephitisDHJ03	Pyrginae	06-SRNP-60058	MHAHK202-07	JF761110
6859	Ridens mephitisDHJ03	Pyrginae	06-SRNP-59544	MHAHK199-07	JF761108
6860	Ridens mephitisDHJ03	Pyrginae	06-SRNP-46964	MHAHJ653-07	JF753135
6861	Ridens mephitisDHJ03	Pyrginae	99-SRNP-11670	XAA900-04	DQ293233
6862	Ridens mephitisDHJ03	Pyrginae	02-SRNP-32153	XAA884-04	DQ293230
6863	Ridens mephitisDHJ03	Pyrginae	02-SRNP-32151	XAA868-04	DQ293228
6864	Ridens mephitisDHJ03	Pyrginae	98-SRNP-2715	XAA935-04	DQ293236
6865	Ridens mephitisDHJ03	Pyrginae	99-SRNP-11297	XAA893-04	DQ293232
6866	Ridens mephitisDHJ03	Pyrginae	99-SRNP-10355	XAA885-04	DQ293231
6867	Ridens mephitisDHJ03	Pyrginae	06-SRNP-59546	MHAHK200-07	JF761109
6868	Ridens mephitisDHJ03	Pyrginae	08-SRNP-56874	MHMXX707-09	JF778435
6869	Ridens mephitisDHJ03	Pyrginae	05-SRNP-65555	MHAHF662-06	GU150759
6870	Ridens mephitisDHJ03	Pyrginae	06-SRNP-36844	MHAHK208-07	JF761114
6871	Ridens mephitisDHJ03	Pyrginae	07-SRNP-57507	MHMXP129-08	JF762797
6872	Ridens mephitisDHJ03	Pyrginae	07-SRNP-57088	MHMXP127-08	JF762799
6873	Ridens mephitisDHJ03	Pyrginae	07-SRNP-57579	MHMXP128-08	JF762798
6874	Ridens mephitisDHJ03	Pyrginae	08-SRNP-57406	MHMXX699-09	JF778431
6875	Ridens mephitisDHJ03	Pyrginae	06-SRNP-56397	MHAHI131-06	GU156301
6876	Ridens mephitisDHJ03	Pyrginae	97-SRNP-1073.01	XAA860-04	DQ293227
6877	Ridens mephitisDHJ03	Pyrginae	99-SRNP-11678	XAA852-04	DQ293226
6878	Ridens mephitisDHJ03	Pyrginae	99-SRNP-11679	CSRII470-04	DQ293225
6879	Ridens mephitisDHJ03	Pyrginae	06-SRNP-58702	MHAHK204-07	JF761112
6880	Ridens mephitisDHJ03	Pyrginae	06-SRNP-60253	MHAHK206-07	JF761113
6881	Ridens mephitisDHJ03	Pyrginae	06-SRNP-59547	MHAHK209-07	JF761115
6882	Ridens mephitisDHJ03	Pyrginae	06-SRNP-60254	MHAHK210-07	JF761116
6883	Ridens mephitisDHJ03	Pyrginae	08-SRNP-57289	MHMXX701-09	JF778433
6884	Ridens mephitisDHJ03	Pyrginae	08-SRNP-57560	MHMXX702-09	JF778434
6885	Ridens mephitisDHJ03	Pyrginae	08-SRNP-57622	MHMXZ009-09	GU665263
6886	Ridens pancheDHJ01	Pyrginae	06-SRNP-31875	MHAHI114-06	GU156303
6887	Ridens pancheDHJ01	Pyrginae	06-SRNP-31873	MHAHH491-06	GU155490

Tree Order	Species	Subfamily	ACG Sampleid	BOLD Processid	Genbank Accession
6888	Ridens pancheDHJ01	Pyrginae	06-SRNP-32333	MHAHI565-06	GU156302
6889	Ridens pancheDHJ01	Pyrginae	00-SRNP-9482	MHAHK555-07	JF761119
6890	Ridens pancheDHJ02	Pyrginae	07-SRNP-35883	MHMXP133-08	JF762803
6891	Ridens pancheDHJ02	Pyrginae	01-SRNP-7463	MHAHK552-07	JF761146
6892	Ridens pancheDHJ02	Pyrginae	07-SRNP-35884	MHMXP134-08	JF762802
6893	Ridens pancheDHJ02	Pyrginae	02-SRNP-9387	CSCR231-04	DQ293239
6894	Ridens pancheDHJ02	Pyrginae	01-SRNP-6746	MHAHK509-07	JF761127
6895	Ridens pancheDHJ02	Pyrginae	02-SRNP-9385	MHAHK557-07	JF761150
6896	Ridens pancheDHJ02	Pyrginae	03-SRNP-4091	MHAHK516-07	JF761134
6897	Ridens pancheDHJ02	Pyrginae	02-SRNP-8372	MHAHK505-07	JF761123
6898	Ridens pancheDHJ02	Pyrginae	01-SRNP-6929	MHAHK507-07	JF761125
6899	Ridens pancheDHJ02	Pyrginae	02-SRNP-8538	MHAHK506-07	JF761124
6900	Ridens pancheDHJ02	Pyrginae	06-SRNP-3632	MHAHK195-07	JF761120
6901	Ridens pancheDHJ02	Pyrginae	06-SRNP-35129	MHAHI112-06	GU156304
6902	Ridens pancheDHJ02	Pyrginae	05-SRNP-35185	MHAHF233-06	GU150762
6903	Ridens pancheDHJ02	Pyrginae	05-SRNP-35180	MHAHF228-06	GU150760
6904	Ridens pancheDHJ02	Pyrginae	04-SRNP-35425	MHAHF225-06	GU150763
6905	Ridens pancheDHJ02	Pyrginae	05-SRNP-31719	MHAHF223-06	GU150761
6906	Ridens pancheDHJ02	Pyrginae	04-SRNP-35446	MHAHD726-05	GU161832
6907	Ridens pancheDHJ02	Pyrginae	04-SRNP-35428	MHAHD725-05	GU161833
6908	Ridens pancheDHJ02	Pyrginae	03-SRNP-6073	CSCR412-04	DQ293240
6909	Ridens pancheDHJ02	Pyrginae	06-SRNP-3638	MHAHK197-07	JF761121
6910	Ridens pancheDHJ02	Pyrginae	06-SRNP-3482	MHAHK198-07	JF761122
6911	Ridens pancheDHJ02	Pyrginae	03-SRNP-3775	MHAHK514-07	JF761132
6912	Ridens pancheDHJ02	Pyrginae	01-SRNP-6839	MHAHK510-07	JF761128
6913	Ridens pancheDHJ02	Pyrginae	02-SRNP-9450	MHAHK508-07	JF761126
6914	Ridens pancheDHJ02	Pyrginae	02-SRNP-8537	MHAHK512-07	JF761130
6915	Ridens pancheDHJ02	Pyrginae	03-SRNP-3740	MHAHK517-07	JF761135
6916	Ridens pancheDHJ02	Pyrginae	03-SRNP-4014	MHAHK519-07	JF761137
6917	Ridens pancheDHJ02	Pyrginae	01-SRNP-9377	MHAHK551-07	JF761145
6918	Ridens pancheDHJ02	Pyrginae	01-SRNP-6552	MHAHK553-07	JF761147
6919	Ridens pancheDHJ02	Pyrginae	00-SRNP-10808	MHAHK511-07	JF761129
6920	Ridens pancheDHJ02	Pyrginae	00-SRNP-10799	MHAHK513-07	JF761131
6921	Ridens pancheDHJ02	Pyrginae	03-SRNP-3772	MHAHK515-07	JF761133
6922	Ridens pancheDHJ02	Pyrginae	03-SRNP-3769	MHAHK518-07	JF761136
6923	Ridens pancheDHJ02	Pyrginae	98-SRNP-2005	MHAHK546-07	JF761140
6924	Ridens pancheDHJ02	Pyrginae	03-SRNP-4067	MHAHK547-07	JF761141
6925	Ridens pancheDHJ02	Pyrginae	03-SRNP-3596	MHAHK548-07	JF761142
6926	Ridens pancheDHJ02	Pyrginae	03-SRNP-4312	MHAHK549-07	JF761143
6927	Ridens pancheDHJ02	Pyrginae	03-SRNP-3808	MHAHK550-07	JF761144
6928	Ridens pancheDHJ02	Pyrginae	02-SRNP-8369	MHAHK554-07	JF761148
6929	Ridens pancheDHJ02	Pyrginae	02-SRNP-8368	MHAHK556-07	JF761149
6930	Ridens pancheDHJ02	Pyrginae	03-SRNP-3774	MHAHK558-07	JF761151
6931	Ridens pancheDHJ02	Pyrginae	03-SRNP-3634	MHAHK559-07	JF761152
6932	Ridens pancheDHJ02	Pyrginae	99-SRNP-453	MHAHK560-07	JF761153
6933	Ridens pancheDHJ02	Pyrginae	06-SRNP-3634	MHMXK106-07	JF762804
6934	Ridens pancheDHJ02	Pyrginae	07-SRNP-35521	MHMXR845-08	JF762801
6935	Ridens pancheDHJ02	Pyrginae	08-SRNP-35179	MHMXX949-09	JF778442
6936	Ridens pancheDHJ02	Pyrginae	08-SRNP-35569	MHMXX953-09	JF778443
6937	Ridens pancheDHJ02	Pyrginae	03-SRNP-23955	MHAHK544-07	JF761138
6938	Ridens pancheDHJ02	Pyrginae	03-SRNP-3637	MHAHK545-07	JF761139
6939	Ridens pancheDHJ02	Pyrginae	08-SRNP-35487	MHMXX695-09	JF778440
6940	Ridens pancheDHJ02	Pyrginae	08-SRNP-35331	MHMXX696-09	JF778441
6941	Ridens Burns01	Pyrginae	06-SRNP-31900	MHAHJ818-07	JF753134
6942	Ridens Burns01	Pyrginae	05-SRNP-30993	MHAHF236-06	GU150755
6943	Ridens Burns01	Pyrginae	05-SRNP-30994	MHAHF242-06	GU150754

Tree Order	Species	Subfamily	ACG Sampleid	BOLD Processid	Genbank Accession
6944	Ridens Burns01	Pyrginae	05-SRNP-30995	MHAHF241-06	GU150757
6945	Ridens Burns01	Pyrginae	02-SRNP-28461	CSCR226-04	DQ293217
6946	Ridens Burns01	Pyrginae	05-SRNP-31102	MHAHF240-06	GU150756
6947	Ridens Burns01	Pyrginae	02-SRNP-28462	CSCR227-04	DQ293218
6948	Ridens Burns01	Pyrginae	05-SRNP-31099	MHAHF234-06	GU150753
6949	Ridens Burns01	Pyrginae	06-SRNP-31896	MHAHI493-06	GU156300
6950	Ridens Burns01	Pyrginae	06-SRNP-7320	MHAHJ736-07	JF753133
6951	Ridens Burns01	Pyrginae	06-SRNP-31898	MHAHI492-06	GU156299
6952	Ridens Burns01	Pyrginae	06-SRNP-31899	MHAHJ651-07	JF753132
6953	Ridens Burns01	Pyrginae	06-SRNP-31901	MHMXK103-07	JF762795
6954	Ridens biolleyi	Pyrginae	09-SRNP-35558	MHMYE1434-09	GU653562
6955	Ridens biolleyi	Pyrginae	02-SRNP-8021	CSCR225-04	DQ293206
6956	Ridens biolleyi	Pyrginae	01-SRNP-6396	CSRII718-05	DQ293209
6957	Ridens biolleyi	Pyrginae	98-SRNP-2547	CSRII723-05	DQ293214
6958	Ridens biolleyi	Pyrginae	00-SRNP-9333	CSRII721-05	DQ293212
6959	Ridens biolleyi	Pyrginae	00-SRNP-9370	CSRII720-05	DQ293211
6960	Ridens biolleyi	Pyrginae	00-SRNP-9238	CSRII724-05	DQ293215
6961	Ridens biolleyi	Pyrginae	00-SRNP-9259	CSRII719-05	DQ293210
6962	Ridens biolleyi	Pyrginae	02-SRNP-8016	CSRII469-04	DQ293208
6963	Ridens biolleyi	Pyrginae	00-SRNP-9229	CSRII468-04	DQ293207
6964	Ridens biolleyi	Pyrginae	01-SRNP-6395	CSRII722-05	DQ293213
6965	Ridens biolleyi	Pyrginae	99-SRNP-17191	CSRII725-05	DQ293216
6966	Ridens biolleyi	Pyrginae	08-SRNP-31106	MHMXX659-09	JF778430
6967	Ridens biolleyi	Pyrginae	09-SRNP-36067	MHMYE1435-09	GU653559
6968	Ridens biolleyi	Pyrginae	09-SRNP-36267	MHMYE1436-09	GU653560
6969	Ridens cachinnans	Pyrginae	08-SRNP-37306	MHMYC382-09	HM893835
6970	Ridens cachinnans	Pyrginae	02-SRNP-23213	CSCR229-04	DQ293238
6971	Ridens cachinnans	Pyrginae	02-SRNP-23212	CSCR228-04	DQ293237
6972	Ridens cachinnans	Pyrginae	08-SRNP-35997	MHMYC383-09	GU649918
6973	Ridens cachinnans	Pyrginae	08-SRNP-35556	MHMYC384-09	GU649921
6974	Ridens cachinnans	Pyrginae	09-SRNP-36708	MHMYG2020-10	HM885421
6975	Venada nevada	Pyrginae	05-SRNP-35056	MHAHL056-07	JF763475
6976	Venada nevada	Pyrginae	07-SRNP-35995	MHMXR829-08	JF763476
6977	Venada nevada	Pyrginae	06-SRNP-36696	MHMXH846-07	JF761315
6978	Venada nevada	Pyrginae	03-SRNP-3106	CSRII229-04	DQ293874
6979	Venada nevada	Pyrginae	03-SRNP-4419	CSRII233-04	DQ293878
6980	Venada nevada	Pyrginae	03-SRNP-4070	CSRII232-04	DQ293877
6981	Venada nevada	Pyrginae	03-SRNP-3148	CSCR440-04	DQ293872
6982	Venada nevada	Pyrginae	03-SRNP-3972	CSRII231-04	DQ293876
6983	Venada nevada	Pyrginae	00-SRNP-9986	CSCR281-04	DQ293870
6984	Venada nevada	Pyrginae	05-SRNP-227	MHAHL046-07	JF763470
6985	Venada nevada	Pyrginae	05-SRNP-30877	MHAHL045-07	JF763469
6986	Venada nevada	Pyrginae	05-SRNP-226	MHAHL044-07	JF763468
6987	Venada nevada	Pyrginae	05-SRNP-577	MHAHL042-07	JF763466
6988	Venada nevada	Pyrginae	05-SRNP-35565	MHAHL041-07	JF763465
6989	Venada nevada	Pyrginae	05-SRNP-35356	MHAHL040-07	JF763464
6990	Venada nevada	Pyrginae	04-SRNP-35614	MHAHE016-05	GU150148
6991	Venada nevada	Pyrginae	04-SRNP-35681	MHAHE015-05	GU150147
6992	Venada nevada	Pyrginae	04-SRNP-56616	MHAHE014-05	GU150149
6993	Venada nevada	Pyrginae	04-SRNP-56617	MHAHE013-05	GU150146
6994	Venada nevada	Pyrginae	04-SRNP-61462	MHAHL043-07	JF763467
6995	Venada nevada	Pyrginae	06-SRNP-36333	MHMXH843-07	JF761317
6996	Venada nevada	Pyrginae	06-SRNP-36619	MHMXH844-07	JF761316
6997	Venada nevada	Pyrginae	98-SRNP-2758	CSCR280-04	DQ293869
6998	Venada nevada	Pyrginae	03-SRNP-3990	CSCR439-04	DQ293871
6999	Venada nevada	Pyrginae	03-SRNP-4768	CSCR441-04	DQ293873

Tree Order	Species	Subfamily	ACG Sampleid	BOLD Processid	Genbank Accession
7000	Venada nevada	Pyrginae	03-SRNP-3346	CSRII230-04	DQ293875
7001	Venada nevada	Pyrginae	05-SRNP-35244	MHAHL054-07	JF763473
7002	Venada nevada	Pyrginae	05-SRNP-30878	MHAHL050-07	JF763471
7003	Venada nevada	Pyrginae	05-SRNP-30663	MHAHL053-07	JF763472
7004	Venada nevada	Pyrginae	05-SRNP-30665	MHAHL055-07	JF763474
7005	Venada nevada	Pyrginae	07-SRNP-36157	MHMXO877-08	JF763481
7006	Venada nevada	Pyrginae	07-SRNP-36499	MHMXO878-08	JF763480
7007	Venada nevada	Pyrginae	07-SRNP-35918	MHMXO880-08	JF763479
7008	Venada nevada	Pyrginae	07-SRNP-35840	MHMXO881-08	JF763478
7009	Venada nevada	Pyrginae	07-SRNP-35841	MHMXO882-08	JF763477
7010	Venada nevada	Pyrginae	08-SRNP-35509	MHMXX614-09	JF778605
7011	Venada nevada	Pyrginae	08-SRNP-35511	MHMXX615-09	JF778606
7012	Venada nevada	Pyrginae	08-SRNP-35510	MHMXX616-09	JF778607
7013	Venada nevada	Pyrginae	08-SRNP-35612	MHMXX617-09	JF778608
7014	Venada daneva	Pyrginae	06-SRNP-4962	MHAHI124-06	GU156725
7015	Venada daneva	Pyrginae	03-SRNP-5810	CSRII237-04	DQ293865
7016	Venada daneva	Pyrginae	02-SRNP-20143	CSRII235-04	DQ293863
7017	Venada daneva	Pyrginae	03-SRNP-6535	CSCR448-04	DQ293858
7018	Venada daneva	Pyrginae	99-SRNP-18830	CSCR453-04	DQ293861
7019	Venada daneva	Pyrginae	03-SRNP-5862	CSCR449-04	DQ293859
7020	Venada daneva	Pyrginae	97-SRNP-11560	CSCR452-04	DQ293860
7021	Venada daneva	Pyrginae	03-SRNP-5863	CSRII238-04	DQ293866
7022	Venada daneva	Pyrginae	03-SRNP-5651	CSRII236-04	DQ293864
7023	Venada daneva	Pyrginae	04-SRNP-32605	MHAHE008-05	GU150142
7024	Venada daneva	Pyrginae	04-SRNP-2360	MHAHE009-05	GU150143
7025	Venada daneva	Pyrginae	04-SRNP-2359	MHAHE010-05	GU150141
7026	Venada daneva	Pyrginae	04-SRNP-2191	MHAHE011-05	GU150144
7027	Venada daneva	Pyrginae	04-SRNP-2688	MHAHE012-05	GU150145
7028	Venada daneva	Pyrginae	05-SRNP-2577	MHAHL052-07	JF763457
7029	Venada daneva	Pyrginae	07-SRNP-2501	MHMXN303-07	JF763460
7030	Venada daneva	Pyrginae	07-SRNP-2502	MHMXN306-07	JF763459
7031	Venada daneva	Pyrginae	07-SRNP-2371	MHMXO879-08	JF763458
7032	Venada daneva	Pyrginae	08-SRNP-2594	MHMXW568-09	JF754386
7033	Venada daneva	Pyrginae	08-SRNP-2680	MHMXW569-09	JF754387
7034	Venada daneva	Pyrginae	08-SRNP-2705	MHMXW570-09	JF754388
7035	Venada daneva	Pyrginae	08-SRNP-31224	MHMXX856-09	JF778604
7036	Venada daneva	Pyrginae	09-SRNP-32294	MHMYE1420-09	HM424369
7037	Venada naranja	Pyrginae	00-SRNP-9226	MHMXI685-07	JF761312
7038	Venada naranja	Pyrginae	01-SRNP-6992	MHMXI682-07	JF761304
7039	Venada naranja	Pyrginae	02-SRNP-8086	MHMXI681-07	JF761305
7040	Venada naranja	Pyrginae	03-SRNP-22199	MHMXI680-07	JF761306
7041	Venada naranja	Pyrginae	00-SRNP-9327	MHMXI678-07	JF761307
7042	Venada naranja	Pyrginae	00-SRNP-9252	MHMXI677-07	JF761308
7043	Venada naranja	Pyrginae	00-SRNP-9183	MHMXI676-07	JF761309
7044	Venada naranja	Pyrginae	00-SRNP-9324	MHMXI675-07	JF761311
7045	Venada naranja	Pyrginae	99-SRNP-1069	MHMXI674-07	JF761313
7046	Venada naranja	Pyrginae	00-SRNP-9050	MHMXI673-07	JF761302
7047	Venada naranja	Pyrginae	06-SRNP-35940	MHMXH845-07	JF761303
7048	Venada naranja	Pyrginae	03-SRNP-4740	CSCR444-04	DQ293868
7049	Venada naranja	Pyrginae	02-SRNP-24573	CSCR442-04	DQ293867
7050	Venada naranja	Pyrginae	03-SRNP-3901	MHMXI679-07	JF761301
7051	Venada naranja	Pyrginae	04-SRNP-35682	MHAHL039-07	JF763461
7052	Venada naranja	Pyrginae	00-SRNP-9329	MHMXI686-07	JF761310
7053	Venada naranja	Pyrginae	04-SRNP-36174	MHAHL051-07	JF763462
7054	Venada naranja	Pyrginae	05-SRNP-35059	MHAHL057-07	JF763463
7055	Venada naranja	Pyrginae	09-SRNP-35765	MHMYE1417-09	HM424366

Tree Order	Species	Subfamily	ACG Sampleid	BOLD Processid	Genbank Accession
7056	Venada Janzen01	Pyrginae	09-SRNP-36371	MHMYE1418-09	HM424367
7057	Venada Janzen01	Pyrginae	09-SRNP-36370	MHMYE1419-09	HM424368
7058	Venada cacao	Pyrginae	02-SRNP-23324	MHMXI672-07	JF761298
7059	Venada cacao	Pyrginae	02-SRNP-23004	CSCR283-04	DQ293856
7060	Venada cacao	Pyrginae	02-SRNP-23362	MHMXI671-07	JF761299
7061	Venada cacao	Pyrginae	02-SRNP-23364	CSCR284-04	DQ293857
7062	Venada cacao	Pyrginae	01-SRNP-6880	MHMXI670-07	JF761300
7063	Venada cacaoDHJ02	Pyrginae	09-SRNP-36883	MHMYH1524-10	JF751917
7064	Polythrix kanshul	Pyrginae	04-SRNP-60560	MHAHC721-05	DQ293078
7065	Polythrix kanshul	Pyrginae	05-SRNP-5452	MHAHF746-06	GU150724
7066	Polythrix kanshul	Pyrginae	09-SRNP-65100	MHMYE894-09	GU653708
7067	Polythrix kanshul	Pyrginae	05-SRNP-2332	MHAHF355-06	GU150725
7068	Polythrix kanshul	Pyrginae	00-SRNP-11396	CSRII147-04	DQ293076
7069	Polythrix kanshul	Pyrginae	00-SRNP-373	CSRII148-04	DQ293077
7070	Polythrix kanshul	Pyrginae	04-SRNP-60051	MHAHE102-05	GU149864
7071	Polythrix kanshul	Pyrginae	06-SRNP-8907	MHAHJ499-07	JF753083
7072	Polythrix kanshul	Pyrginae	06-SRNP-9915	MHAHJ643-07	JF753084
7073	Polythrix kanshul	Pyrginae	07-SRNP-738	MHMXK268-07	JF762670
7074	Polythrix kanshul	Pyrginae	07-SRNP-65895	MHMXR794-08	JF762669
7075	Polythrix kanshul	Pyrginae	09-SRNP-42534	MHMYG2429-10	HM885852
7076	Porphyrogenes sula	Pyrginae	06-SRNP-42916	MHAHI503-06	GU156281
7077	Ocyba calathana	Pyrginae	98-SRNP-6146	CSCR167-04	DQ292782
7078	Ocyba calathana	Pyrginae	06-SRNP-55791	MHAHH499-06	GU155439
7079	Ocyba calathana	Pyrginae	06-SRNP-55767	MHAHH498-06	GU155440
7080	Ocyba calathana	Pyrginae	01-SRNP-12165	CSRII536-04	DQ292784
7081	Ocyba calathana	Pyrginae	00-SRNP-2597	CSRII535-04	DQ292783
7082	Ocyba calathana	Pyrginae	06-SRNP-12192	MHAHG709-06	GU151504
7083	Ocyba calathana	Pyrginae	05-SRNP-64295	MHAHG710-06	GU151505
7084	Ocyba calathana	Pyrginae	07-SRNP-65680	MHMXR898-08	JF762473
7085	Ocyba calathana	Pyrginae	08-SRNP-12162	MHMXX713-09	JF778157
7086	Porphyrogenes peterwegei	Pyrginae	06-SRNP-41790	MHAHI024-06	GU156273
7087	Porphyrogenes peterwegei	Pyrginae	06-SRNP-59687	MHAHK192-07	JF761061
7088	Porphyrogenes peterwegei	Pyrginae	08-SRNP-65472	MHMXW033-09	JF754107
7089	Porphyrogenes peterwegei	Pyrginae	07-SRNP-45285	MHMXP165-08	JF762681
7090	Porphyrogenes peterwegei	Pyrginae	05-SRNP-2308	MHMXN282-07	JF762683
7091	Porphyrogenes peterwegei	Pyrginae	05-SRNP-4096	MHAHI677-06	GU156280
7092	Porphyrogenes peterwegei	Pyrginae	02-SRNP-14654	CSRII291-04	DQ293094
7093	Porphyrogenes peterwegei	Pyrginae	07-SRNP-65894	MHMXR765-08	JF762678
7094	Porphyrogenes peterwegei	Pyrginae	06-SRNP-7825	MHAHJ773-07	JF753092
7095	Porphyrogenes peterwegei	Pyrginae	05-SRNP-25294	MHAHH502-06	GU155462
7096	Porphyrogenes peterwegei	Pyrginae	06-SRNP-31563	MHAHH501-06	GU155460
7097	Porphyrogenes peterwegei	Pyrginae	06-SRNP-32892	MHAHI505-06	GU156277
7098	Porphyrogenes peterwegei	Pyrginae	03-SRNP-6133	CSCR397-04	DQ293088
7099	Porphyrogenes peterwegei	Pyrginae	07-SRNP-23407	MHMXR763-08	JF762680
7100	Porphyrogenes peterwegei	Pyrginae	06-SRNP-65685	MHAHL351-07	JF762677
7101	Porphyrogenes peterwegei	Pyrginae	07-SRNP-31916	MHAHL349-07	JF762675
7102	Porphyrogenes peterwegei	Pyrginae	07-SRNP-30450	MHAHL348-07	JF762674
7103	Porphyrogenes peterwegei	Pyrginae	05-SRNP-1922	MHAHL003-07	JF762673
7104	Porphyrogenes peterwegei	Pyrginae	05-SRNP-41224	MHMXN281-07	JF762684
7105	Porphyrogenes peterwegei	Pyrginae	05-SRNP-2099	MHMXN280-07	JF762685
7106	Porphyrogenes peterwegei	Pyrginae	05-SRNP-22389	MHMXN278-07	JF762687
7107	Porphyrogenes peterwegei	Pyrginae	07-SRNP-40389	MHMXK160-07	JF762688
7108	Porphyrogenes peterwegei	Pyrginae	06-SRNP-22070	MHAHJ775-07	JF753094
7109	Porphyrogenes peterwegei	Pyrginae	06-SRNP-22071	MHAHJ774-07	JF753093
7110	Porphyrogenes peterwegei	Pyrginae	06-SRNP-22084	MHAHJ772-07	JF753091
7111	Porphyrogenes peterwegei	Pyrginae	06-SRNP-33708	MHAHJ771-07	JF753090

Tree Order	Species	Subfamily	ACG Sampleid	BOLD Processid	Genbank Accession
7112	Porphyrogenes peterwegei	Pyrginae	06-SRNP-43467	MHAHJ681-07	JF753087
7113	Porphyrogenes peterwegei	Pyrginae	05-SRNP-1925	MHAHI675-06	GU156278
7114	Porphyrogenes peterwegei	Pyrginae	06-SRNP-45298	MHAHI022-06	GU156275
7115	Porphyrogenes peterwegei	Pyrginae	06-SRNP-45299	MHAHI021-06	GU156276
7116	Porphyrogenes peterwegei	Pyrginae	06-SRNP-31354	MHAHH500-06	GU155461
7117	Porphyrogenes peterwegei	Pyrginae	05-SRNP-43764	MHAHG713-06	GU151584
7118	Porphyrogenes peterwegei	Pyrginae	04-SRNP-4234	MHAHD860-05	GU161796
7119	Porphyrogenes peterwegei	Pyrginae	04-SRNP-24187	MHAHC805-05	DQ293098
7120	Porphyrogenes peterwegei	Pyrginae	04-SRNP-4400	MHAHC804-05	DQ293097
7121	Porphyrogenes peterwegei	Pyrginae	04-SRNP-45221	MHAHC127-05	DQ293095
7122	Porphyrogenes peterwegei	Pyrginae	05-SRNP-43653	MHAHG712-06	GU151585
7123	Porphyrogenes peterwegei	Pyrginae	06-SRNP-22153	MHAHJ747-07	JF753089
7124	Porphyrogenes peterwegei	Pyrginae	03-SRNP-21521	CSRII289-04	DQ293092
7125	Porphyrogenes peterwegei	Pyrginae	05-SRNP-45183	MHAHL002-07	JF762672
7126	Porphyrogenes peterwegei	Pyrginae	07-SRNP-41033	MHAHL350-07	JF762676
7127	Porphyrogenes peterwegei	Pyrginae	07-SRNP-2767	MHMXP164-08	JF762682
7128	Porphyrogenes peterwegei	Pyrginae	04-SRNP-22615	MHAHC803-05	DQ293096
7129	Porphyrogenes peterwegei	Pyrginae	07-SRNP-42518	MHMXR764-08	JF762679
7130	Porphyrogenes peterwegei	Pyrginae	08-SRNP-65165	MHMXW027-09	JF754101
7131	Porphyrogenes peterwegei	Pyrginae	08-SRNP-55407	MHMXW028-09	JF754102
7132	Porphyrogenes peterwegei	Pyrginae	08-SRNP-2369	MHMXW029-09	JF754103
7133	Porphyrogenes peterwegei	Pyrginae	08-SRNP-65274	MHMXW030-09	JF754104
7134	Porphyrogenes peterwegei	Pyrginae	08-SRNP-2549	MHMXW031-09	JF754105
7135	Porphyrogenes peterwegei	Pyrginae	08-SRNP-2551	MHMXW032-09	JF754106
7136	Porphyrogenes peterwegei	Pyrginae	08-SRNP-2281	MHMXW034-09	JF754108
7137	Porphyrogenes peterwegei	Pyrginae	08-SRNP-2554	MHMXW035-09	JF754109
7138	Porphyrogenes peterwegei	Pyrginae	08-SRNP-2550	MHMXW036-09	JF754110
7139	Porphyrogenes peterwegei	Pyrginae	08-SRNP-65453	MHMXW038-09	JF754111
7140	Porphyrogenes peterwegei	Pyrginae	08-SRNP-65190	MHMXW039-09	JF754112
7141	Porphyrogenes peterwegei	Pyrginae	08-SRNP-5053	MHMXX624-09	JF778412
7142	Porphyrogenes peterwegei	Pyrginae	08-SRNP-4015	MHMXX625-09	JF778413
7143	Porphyrogenes peterwegei	Pyrginae	08-SRNP-65797	MHMXY1081-09	HM390681
7144	Porphyrogenes peterwegei	Pyrginae	08-SRNP-5051	MHMYC547-09	GU649800
7145	Porphyrogenes peterwegei	Pyrginae	09-SRNP-70382	MHMYE1517-09	GU653487
7146	Porphyrogenes peterwegei	Pyrginae	09-SRNP-21376	MHMYG2473-10	HM885900
7147	Porphyrogenes peterwegei	Pyrginae	03-SRNP-20026	CSRII288-04	DQ293091
7148	Porphyrogenes peterwegei	Pyrginae	03-SRNP-9403	CSRII286-04	DQ293089
7149	Porphyrogenes peterwegei	Pyrginae	02-SRNP-6580	CSRII290-04	DQ293093
7150	Porphyrogenes peterwegei	Pyrginae	05-SRNP-42535	MHAHI676-06	GU156279
7151	Porphyrogenes peterwegei	Pyrginae	06-SRNP-32698	MHAHI020-06	GU156274
7152	Porphyrogenes peterwegei	Pyrginae	06-SRNP-32697	MHAHI023-06	GU156272
7153	Porphyrogenes peterwegei	Pyrginae	06-SRNP-22072	MHAHJ746-07	JF753088
7154	Porphyrogenes peterwegei	Pyrginae	03-SRNP-9405	CSRII287-04	DQ293090
7155	Porphyrogenes peterwegei	Pyrginae	02-SRNP-21541	CSCR208-04	DQ293087
7156	Porphyrogenes peterwegei	Pyrginae	06-SRNP-22118	MHAHJ501-07	JF753086
7157	Porphyrogenes peterwegei	Pyrginae	07-SRNP-904	MHMXK159-07	JF762689
7158	Porphyrogenes peterwegei	Pyrginae	05-SRNP-46602	MHMXN279-07	JF762686
7159	Porphyrogenes peterwegei	Pyrginae	09-SRNP-2624	MHMYG2474-10	HM885901
7160	Calliades zeutus	Pyrginae	05-SRNP-58434	MHAHF349-06	GU150300
7161	Calliades zeutus	Pyrginae	06-SRNP-19561	MHMXK042-07	JF761737
7162	Calliades zeutus	Pyrginae	07-SRNP-20366	MHMXK041-07	JF761738
7163	Calliades zeutus	Pyrginae	06-SRNP-58046	MHAHJ785-07	JF752494
7164	Calliades zeutus	Pyrginae	04-SRNP-49938	MHAHD908-05	GU161302
7165	Calliades zeutus	Pyrginae	04-SRNP-14586	MHAHD907-05	GU161304
7166	Calliades zeutus	Pyrginae	04-SRNP-14067	MHAHD906-05	GU161300
7167	Calliades zeutus	Pyrginae	04-SRNP-49935	MHAHD905-05	GU161303

Tree Order	Species	Subfamily	ACG Sampleid	BOLD Processid	Genbank Accession
7168	Calliades zeutus	Pyrginae	04-SRNP-49393	MHAHD904-05	GU161301
7169	Calliades zeutus	Pyrginae	05-SRNP-61209	MHAHF744-06	GU150302
7170	Calliades zeutus	Pyrginae	06-SRNP-19539	MHMXI085-07	JF760458
7171	Calliades zeutus	Pyrginae	05-SRNP-55236	MHAHF350-06	GU150301
7172	Calliades zeutus	Pyrginae	02-SRNP-4294	CSRII110-04	DQ291955
7173	Calliades zeutus	Pyrginae	02-SRNP-4104	CSRII109-04	DQ291954
7174	Calliades zeutus	Pyrginae	07-SRNP-20861	MHMXN295-07	JF761736
7175	Calliades zeutus	Pyrginae	07-SRNP-21845	MHMXN297-07	JF761735
7176	Calliades zeutus	Pyrginae	07-SRNP-21844	MHMXN299-07	JF761734
7177	Proteides mercurius	Pyrginae	95-SRNP-6422	MHAHK561-07	JF761062
7178	Proteides mercurius	Pyrginae	05-SRNP-59505	MHAHF847-06	GU150727
7179	Proteides mercurius	Pyrginae	95-SRNP-4261	MHAHK564-07	JF761065
7180	Proteides mercurius	Pyrginae	95-SRNP-7319	MHAHK569-07	JF761069
7181	Proteides mercurius	Pyrginae	02-SRNP-4516	MHAHK571-07	JF761071
7182	Proteides mercurius	Pyrginae	95-SRNP-74	MHAHK568-07	JF761068
7183	Proteides mercurius	Pyrginae	00-SRNP-2023	MHAHK570-07	JF761070
7184	Proteides mercurius	Pyrginae	99-SRNP-6062	MHAHK572-07	JF761072
7185	Proteides mercurius	Pyrginae	95-SRNP-6413	MHAHK562-07	JF761063
7186	Proteides mercurius	Pyrginae	95-SRNP-7833	MHAHK567-07	JF761067
7187	Proteides mercurius	Pyrginae	04-SRNP-2133	MHAHC717-05	DQ293121
7188	Proteides mercurius	Pyrginae	05-SRNP-19609	MHAHF846-06	GU150726
7189	Proteides mercurius	Pyrginae	04-SRNP-14109	MHAHC718-05	DQ293122
7190	Proteides mercurius	Pyrginae	95-SRNP-6352	MHAHK566-07	JF761066
7191	Proteides mercurius	Pyrginae	95-SRNP-4269	MHAHK563-07	JF761064
7192	Proteides mercurius	Pyrginae	06-SRNP-55587	MHAHG157-06	GU151587
7193	Proteides mercurius	Pyrginae	95-SRNP-6265	CSCR212-04	DQ293120
7194	Proteides mercurius	Pyrginae	07-SRNP-40797	MHMXN334-07	JF762697
7195	Spathilepia clonius	Pyrginae	95-SRNP-9249	CSCR250-04	DQ293326
7196	Spathilepia clonius	Pyrginae	02-SRNP-5756	CSRII477-04	DQ293328
7197	Spathilepia clonius	Pyrginae	07-SRNP-20091	MHMXK026-07	JF762932
7198	Spathilepia clonius	Pyrginae	05-SRNP-20626	MHAHD713-05	GU161850
7199	Spathilepia clonius	Pyrginae	07-SRNP-20254	MHMXK029-07	JF762929
7200	Spathilepia clonius	Pyrginae	04-SRNP-47760	MHAHD871-05	GU161851
7201	Spathilepia clonius	Pyrginae	04-SRNP-27278	MHAHF288-06	GU150852
7202	Spathilepia clonius	Pyrginae	05-SRNP-46985	MHAHF289-06	GU150851
7203	Spathilepia clonius	Pyrginae	07-SRNP-20183	MHMXK027-07	JF762931
7204	Spathilepia clonius	Pyrginae	01-SRNP-15478	CSRII476-04	DQ293327
7205	Spathilepia clonius	Pyrginae	07-SRNP-20074	MHMXK025-07	JF762933
7206	Spathilepia clonius	Pyrginae	07-SRNP-20201	MHMXK028-07	JF762930
7207	Spathilepia clonius	Pyrginae	07-SRNP-20075	MHMXK030-07	JF762928
7208	Spathilepia clonius	Pyrginae	09-SRNP-76098	MHMYH135-10	HM887283
7209	Narcosius samson	Pyrginae	05-SRNP-32509	MHAHF691-06	GU150569
7210	Narcosius samson	Pyrginae	02-SRNP-3561	CSCR142-04	DQ292674
7211	Narcosius samson	Pyrginae	02-SRNP-18532	CSCR461-04	DQ292671
7212	Narcosius samson	Pyrginae	07-SRNP-45154	MHMXK107-07	JF762400
7213	Narcosius samson	Pyrginae	05-SRNP-21384	MHAHF229-06	GU150567
7214	Narcosius samson	Pyrginae	04-SRNP-23182	MHAHD709-05	GU161685
7215	Narcosius samson	Pyrginae	04-SRNP-46044	MHAHD708-05	GU161684
7216	Narcosius samson	Pyrginae	07-SRNP-2014	MHAHL519-07	JF762386
7217	Narcosius samson	Pyrginae	07-SRNP-33701	MHMXT237-08	JF762398
7218	Narcosius samson	Pyrginae	08-SRNP-30598	MHMXX952-09	JF778110
7219	Narcosius samson	Pyrginae	05-SRNP-22628	MHAHF690-06	GU150570
7220	Narcosius samson	Pyrginae	06-SRNP-6237	MHAHI108-06	GU156006
7221	Narcosius samson	Pyrginae	02-SRNP-373	CSCR465-04	DQ292672
7222	Narcosius samson	Pyrginae	07-SRNP-65943	MHMXT244-08	JF762391
7223	Narcosius samson	Pyrginae	07-SRNP-65845	MHMXT243-08	JF762392

Tree Order	Species	Subfamily	ACG Sampleid	BOLD Processid	Genbank Accession
7224	Narcosius samson	Pyrginae	08-SRNP-40054	MHMXT242-08	JF762393
7225	Narcosius samson	Pyrginae	07-SRNP-4271	MHMXT241-08	JF762394
7226	Narcosius samson	Pyrginae	07-SRNP-4275	MHMXT240-08	JF762395
7227	Narcosius samson	Pyrginae	07-SRNP-4276	MHMXT239-08	JF762396
7228	Narcosius samson	Pyrginae	08-SRNP-65210	MHMXS093-08	JF762399
7229	Narcosius samson	Pyrginae	07-SRNP-41022	MHAHL522-07	JF762389
7230	Narcosius samson	Pyrginae	07-SRNP-1456	MHAHL521-07	JF762388
7231	Narcosius samson	Pyrginae	07-SRNP-1341	MHAHL520-07	JF762387
7232	Narcosius samson	Pyrginae	06-SRNP-33348	MHAHI113-06	GU156005
7233	Narcosius samson	Pyrginae	04-SRNP-60936	MHAHF219-06	GU150568
7234	Narcosius samson	Pyrginae	07-SRNP-33230	MHMXT238-08	JF762397
7235	Narcosius samson	Pyrginae	02-SRNP-1251	CSCR141-04	DQ292673
7236	Narcosius samson	Pyrginae	07-SRNP-4272	MHMXT245-08	JF762390
7237	Narcosius samson	Pyrginae	08-SRNP-1747	MHMXX955-09	JF778111
7238	Narcosius colossus	Pyrginae	07-SRNP-1788	MHAHL518-07	JF762377
7239	Narcosius colossus	Pyrginae	05-SRNP-3183	MHAHF221-06	GU150562
7240	Narcosius colossus	Pyrginae	05-SRNP-35254	MHAHF230-06	GU150561
7241	Narcosius colossus	Pyrginae	04-SRNP-34370	MHAHD707-05	GU161678
7242	Narcosius colossus	Pyrginae	05-SRNP-35044	MHAHF218-06	GU150563
7243	Narcosius colossus	Pyrginae	07-SRNP-60189	MHMXR843-08	JF762379
7244	Narcosius colossus	Pyrginae	07-SRNP-58794	MHMXR844-08	JF762378
7245	Narcosius colossus	Pyrginae	07-SRNP-1843	MHAHL517-07	JF762376
7246	Narcosius colossus	Pyrginae	03-SRNP-79	CSCR381-04	DQ292669
7247	Narcosius colossus	Pyrginae	06-SRNP-4365	MHAHI110-06	GU156003
7248	Narcosius colossus	Pyrginae	06-SRNP-35026	MHAHG703-06	GU151483
7249	Narcosius colossus	Pyrginae	06-SRNP-2399	MHAHG702-06	GU151482
7250	Narcosius colossus	Pyrginae	06-SRNP-12012	MHAHG701-06	GU151481
7251	Narcosius colossus	Pyrginae	04-SRNP-61447	MHAHF220-06	GU150564
7252	Narcosius colossus	Pyrginae	04-SRNP-35290	MHAHD724-05	GU161679
7253	Narcosius colossus	Pyrginae	04-SRNP-35050	MHAHD723-05	GU161681
7254	Narcosius colossus	Pyrginae	04-SRNP-35268	MHAHD721-05	GU161682
7255	Narcosius colossus	Pyrginae	04-SRNP-35266	MHAHD720-05	GU161683
7256	Narcosius colossus	Pyrginae	05-SRNP-35355	MHAHF224-06	GU150560
7257	Narcosius colossus	Pyrginae	06-SRNP-31983	MHAHH492-06	GU155434
7258	Narcosius colossus	Pyrginae	02-SRNP-9121	CSCR471-04	DQ292668
7259	Narcosius colossus	Pyrginae	06-SRNP-36131	MHAHK196-07	JF760884
7260	Narcosius colossus	Pyrginae	07-SRNP-1842	MHMXN252-07	JF762384
7261	Narcosius colossus	Pyrginae	07-SRNP-60168	MHMXR842-08	JF762380
7262	Narcosius colossus	Pyrginae	08-SRNP-1953	MHMXX954-09	JF778107
7263	Narcosius colossus	Pyrginae	08-SRNP-2685	MHMXX956-09	JF778108
7264	Narcosius colossus	Pyrginae	08-SRNP-2684	MHMXX957-09	JF778109
7265	Narcosius colossus	Pyrginae	07-SRNP-57343	MHMXP136-08	JF762383
7266	Narcosius colossus	Pyrginae	07-SRNP-23367	MHMXR841-08	JF762381
7267	Narcosius colossus	Pyrginae	04-SRNP-35101	MHAHD722-05	GU161680
7268	Narcosius colossus	Pyrginae	05-SRNP-2080	MHAHF222-06	GU150565
7269	Narcosius colossus	Pyrginae	07-SRNP-23315	MHMXR840-08	JF762382
7270	Narcosius colossus	Pyrginae	08-SRNP-4789	MHMXX698-09	JF778106
7271	Narcosius helen	Pyrginae	96-SRNP-7367	CSCR139-04	DQ292670
7272	Narcosius helen	Pyrginae	06-SRNP-59517	MHAHK194-07	JF760886
7273	Narcosius helen	Pyrginae	06-SRNP-22184	MHAHJ789-07	JF752937
7274	Narcosius helen	Pyrginae	06-SRNP-3211	MHAHI109-06	GU156004
7275	Narcosius helen	Pyrginae	05-SRNP-65551	MHAHF689-06	GU150566
7276	Narcosius helen	Pyrginae	06-SRNP-46972	MHAHJ758-07	JF752936
7277	Narcosius helen	Pyrginae	06-SRNP-60063	MHAHK193-07	JF760885
7278	Narcosius helen	Pyrginae	07-SRNP-57650	MHMXP132-08	JF762385
7279	Narcosius nazaraeus	Pyrginae	09-SRNP-20099	MHMYB188-09	HM431658

Tree Order	Species	Subfamily	ACG Sampleid	BOLD Processid	Genbank Accession
7280	Aguna claxon	Eudaminae	05-SRNP-41660	MHAHE433-05	GU149333
7281	Aguna claxon	Eudaminae	05-SRNP-41659	MHAHE436-05	GU149332
7282	Aguna claxon	Eudaminae	05-SRNP-41925	MHAHE614-06	GU149334
7283	Aguna claxon	Eudaminae	06-SRNP-42825	MHAHJ894-07	JF752350
7284	Aguna claxon	Eudaminae	07-SRNP-41625	MHAHL403-07	JF761356
7285	Aguna claxon	Eudaminae	09-SRNP-44598	MHMYE1463-09	GU653536
7286	Aguna panama	Eudaminae	07-SRNP-55426	MHMXK262-07	JF761359
7287	Aguna panama	Eudaminae	00-SRNP-6020	CSRII354-04	DQ291798
7288	Aguna panama	Eudaminae	07-SRNP-61073	MHMXW478-09	JF753647
7289	Aguna panama	Eudaminae	07-SRNP-56460	MHAHL402-07	JF761358
7290	Aguna panama	Eudaminae	06-SRNP-58291	MHMXH840-07	JF760226
7291	Aguna panama	Eudaminae	05-SRNP-57368	MHAHE435-05	GU149338
7292	Aguna panama	Eudaminae	00-SRNP-6052	CSRII355-04	DQ291799
7293	Aguna panama	Eudaminae	08-SRNP-55747	MHMXW479-09	JF753648
7294	Aguna panama	Eudaminae	07-SRNP-61072	MHMXW480-09	JF753649
7295	Aguna panama	Eudaminae	08-SRNP-55705	MHMXW525-09	JF753646
7296	Aguna panama	Eudaminae	09-SRNP-57341	MHMYE1462-09	GU653535
7297	Aguna metophis	Eudaminae	05-SRNP-41056	MHAHD391-05	GU161222
7298	Aguna asander	Eudaminae	05-SRNP-56838	MHAHE437-05	GU149327
7299	Aguna asander	Eudaminae	06-SRNP-56534	MHAHI116-06	GU155733
7300	Aguna asander	Eudaminae	07-SRNP-56019	MHMXK115-07	JF761352
7301	Aguna asander	Eudaminae	07-SRNP-56139	MHMXK113-07	JF761354
7302	Aguna asander	Eudaminae	05-SRNP-56840	MHAHE439-05	GU149325
7303	Aguna asander	Eudaminae	05-SRNP-56839	MHAHE438-05	GU149326
7304	Aguna asander	Eudaminae	06-SRNP-56535	MHAHI120-06	GU155731
7305	Aguna asander	Eudaminae	07-SRNP-56140	MHMXK112-07	JF761355
7306	Aguna asander	Eudaminae	07-SRNP-56354	MHMXN318-07	JF761351
7307	Aguna asander	Eudaminae	07-SRNP-57812	MHMXP137-08	JF761345
7308	Aguna asander	Eudaminae	07-SRNP-56518	MHMXN320-07	JF761349
7309	Aguna asander	Eudaminae	07-SRNP-56519	MHMXK114-07	JF761353
7310	Aguna asander	Eudaminae	07-SRNP-56833	MHMXN322-07	JF761347
7311	Aguna asander	Eudaminae	98-SRNP-3363	CSCR009-04	DQ291783
7312	Aguna asander	Eudaminae	06-SRNP-56500	MHAHI118-06	GU155732
7313	Aguna asander	Eudaminae	06-SRNP-56093	MHAHI119-06	GU155734
7314	Aguna asander	Eudaminae	97-SRNP-2360	CSCR008-04	DQ291782
7315	Aguna asander	Eudaminae	07-SRNP-56789	MHMXN319-07	JF761350
7316	Aguna asander	Eudaminae	06-SRNP-56319	MHAHI117-06	GU155735
7317	Aguna asander	Eudaminae	07-SRNP-56524	MHMXN321-07	JF761348
7318	Aguna asander	Eudaminae	07-SRNP-56433	MHMXN323-07	JF761346
7319	Aguna asander	Eudaminae	07-SRNP-57226	MHMXP138-08	JF761344
7320	Aguna asander	Eudaminae	07-SRNP-56791	MHMXP139-08	JF761343
7321	Aguna arunce hypozonius	Eudaminae	05-SRNP-57391	MHAHE434-05	GU149336
7322	Aguna arunce hypozonius	Eudaminae	05-SRNP-57392	MHAHE432-05	GU149337
7323	Aguna arunce hypozonius	Eudaminae	05-SRNP-57393	MHAHE431-05	GU149335
7324	Aguna arunce hypozonius	Eudaminae	07-SRNP-56141	MHMXK007-07	JF761342
7325	Aguna arunce hypozonius	Eudaminae	07-SRNP-58253	MHMXN251-07	JF761341
7326	Aguna Burns01	Eudaminae	04-SRNP-42390	MHAHE098-05	GU149329
7327	Aguna Burns01	Eudaminae	03-SRNP-21307	CSCR475-04	DQ291784
7328	Aguna Burns01	Eudaminae	03-SRNP-21306	CSRII398-04	DQ291786
7329	Aguna Burns01	Eudaminae	03-SRNP-37032	CSRII091-04	DQ291785
7330	Aguna Burns01	Eudaminae	05-SRNP-41612	MHAHE430-05	GU149328
7331	Aguna Burns01	Eudaminae	05-SRNP-70024	MHAHF598-06	GU150159
7332	Aguna Burns01	Eudaminae	03-SRNP-21373	CSRII399-04	DQ291787
7333	Aguna Burns01	Eudaminae	03-SRNP-21386	CSRII400-04	DQ291788
7334	Aguna Burns01	Eudaminae	08-SRNP-71779	MHMXW482-09	JF753643
7335	Aguna Burns01	Eudaminae	08-SRNP-71112	MHMXW477-09	JF753641

Tree Order	Species	Subfamily	ACG Sampleid	BOLD Processid	Genbank Accession
7336	Aguna Burns01	Eudaminae	08-SRNP-71115	MHMXW481-09	JF753642
7337	Aguna Burns01	Eudaminae	08-SRNP-71111	MHMXW483-09	JF753644
7338	Aguna coeloides	Eudaminae	07-SRNP-31814	MHAHL404-07	JF761357
7339	Aguna coeloides	Eudaminae	02-SRNP-6676	CSRII381-04	DQ291795
7340	Aguna coeloides	Eudaminae	03-SRNP-20032	CSCR474-04	DQ291794
7341	Aguna coeloides	Eudaminae	03-SRNP-20033	CSRII396-04	DQ291796
7342	Aguna coeloides	Eudaminae	02-SRNP-6344	CSRII428-04	DQ291797
7343	Aguna coeloides	Eudaminae	08-SRNP-71144	MHMXW484-09	JF753645
7344	Aguna coeloides	Eudaminae	08-SRNP-32489	MHMXY1012-09	GU666517
7345	Aguna Burns02	Eudaminae	03-SRNP-10346	CSCR300-04	DQ291789
7346	Aguna Burns02	Eudaminae	05-SRNP-43291	MHAHF599-06	GU150160
7347	Aguna Burns02	Eudaminae	05-SRNP-42263	MHAHE613-06	GU149331
7348	Aguna Burns02	Eudaminae	05-SRNP-40266	MHAHE429-05	GU149330
7349	Aguna Burns02	Eudaminae	03-SRNP-10765	CSRII395-04	DQ291793
7350	Aguna Burns02	Eudaminae	03-SRNP-10241	CSCR476-04	DQ291791
7351	Aguna Burns02	Eudaminae	03-SRNP-10064	CSCR301-04	DQ291790
7352	Aguna Burns02	Eudaminae	03-SRNP-31756	CSRII090-04	DQ291792
7353	Aguna Burns02	Eudaminae	06-SRNP-42285	MHAHI128-06	GU155737
7354	Aguna Burns02	Eudaminae	06-SRNP-42575	MHAHI497-06	GU155736
7355	Aguna Burns02	Eudaminae	06-SRNP-42313	MHAHI604-06	GU155738
7356	Aguna Burns02	Eudaminae	06-SRNP-44328	MHAHJ607-07	JF752348
7357	Aguna Burns02	Eudaminae	06-SRNP-44327	MHAHJ893-07	JF752349
7358	Aguna Burns02	Eudaminae	09-SRNP-69431	MHMYH151-10	HM887299
7359	Aguna Burns02	Eudaminae	09-SRNP-69465	MHMYH153-10	HM887301
7360	Autochton bipunctatus	Pyrginae	08-SRNP-30643	MHMXW323-09	JF753703
7361	Autochton Burns01DHJ02	Pyrginae	04-SRNP-45713	MHAHD313-05	GU161297
7362	Autochton Burns01DHJ02	Pyrginae	08-SRNP-1103	MHMXW322-09	JF753715
7363	Autochton Burns01DHJ02	Pyrginae	08-SRNP-1650	MHMXW317-09	JF753713
7364	Autochton Burns01DHJ02	Pyrginae	08-SRNP-1753	MHMXW315-09	JF753712
7365	Autochton Burns01DHJ02	Pyrginae	02-SRNP-2645	MHAHK437-07	JF760401
7366	Autochton Burns01DHJ02	Pyrginae	03-SRNP-19145	MHAHK422-07	JF760400
7367	Autochton Burns01DHJ02	Pyrginae	06-SRNP-59183	MHAHK032-07	JF760398
7368	Autochton Burns01DHJ02	Pyrginae	02-SRNP-2824	MHAHI305-06	GU155842
7369	Autochton Burns01DHJ02	Pyrginae	02-SRNP-2646	MHAHI302-06	GU155841
7370	Autochton Burns01DHJ02	Pyrginae	02-SRNP-2647	MHAHI300-06	GU155831
7371	Autochton Burns01DHJ02	Pyrginae	02-SRNP-2644	MHAHI299-06	GU155834
7372	Autochton Burns01DHJ02	Pyrginae	02-SRNP-2825	MHAHI298-06	GU155836
7373	Autochton Burns01DHJ02	Pyrginae	02-SRNP-2822	MHAHI297-06	GU155835
7374	Autochton Burns01DHJ02	Pyrginae	02-SRNP-2821	MHAHI296-06	GU155838
7375	Autochton Burns01DHJ02	Pyrginae	02-SRNP-6791	MHAHI294-06	GU155840
7376	Autochton Burns01DHJ02	Pyrginae	02-SRNP-2643	MHAHI292-06	GU155833
7377	Autochton Burns01DHJ02	Pyrginae	06-SRNP-2611	MHAHG630-06	GU151209
7378	Autochton Burns01DHJ02	Pyrginae	06-SRNP-1336	MHAHG629-06	GU151210
7379	Autochton Burns01DHJ02	Pyrginae	04-SRNP-45765	MHAHD312-05	GU161296
7380	Autochton Burns01DHJ02	Pyrginae	00-SRNP-3690	MHAHI295-06	GU155837
7381	Autochton Burns01DHJ02	Pyrginae	05-SRNP-46948	MHAHE596-06	GU149432
7382	Autochton Burns01DHJ02	Pyrginae	01-SRNP-1372	MHAHI293-06	GU155839
7383	Autochton Burns01DHJ02	Pyrginae	02-SRNP-2823	MHAHI301-06	GU155832
7384	Autochton Burns01DHJ02	Pyrginae	05-SRNP-59071	MHAHE598-06	GU149433
7385	Autochton Burns01DHJ02	Pyrginae	06-SRNP-9152	MHAHJ453-07	JF752470
7386	Autochton Burns01DHJ02	Pyrginae	06-SRNP-9303	MHAHK033-07	JF760399
7387	Autochton Burns01DHJ02	Pyrginae	08-SRNP-1961	MHMXW320-09	JF753714
7388	Autochton Burns01DHJ02	Pyrginae	08-SRNP-1113	MHMXW324-09	JF753716
7389	Autochton Burns01DHJ02	Pyrginae	08-SRNP-1014	MHMXW325-09	JF753717
7390	Autochton Burns01DHJ02	Pyrginae	08-SRNP-1534	MHMXW326-09	JF753718
7391	Autochton Burns01DHJ02	Pyrginae	08-SRNP-1533	MHMXW327-09	JF753704

Tree Order	Species	Subfamily	ACG Sampleid	BOLD Processid	Genbank Accession
7392	Autochton Burns01DHJ02	Pyrginae	08-SRNP-40551	MHMXW328-09	JF753705
7393	Autochton Burns01DHJ02	Pyrginae	08-SRNP-749	MHMXW332-09	JF753706
7394	Autochton Burns01DHJ02	Pyrginae	08-SRNP-718	MHMXW334-09	JF753707
7395	Autochton Burns01DHJ02	Pyrginae	08-SRNP-1752	MHMXW337-09	JF753708
7396	Autochton Burns01DHJ02	Pyrginae	08-SRNP-1710	MHMXW338-09	JF753709
7397	Autochton Burns01DHJ02	Pyrginae	08-SRNP-1649	MHMXW339-09	JF753710
7398	Autochton Burns01DHJ02	Pyrginae	08-SRNP-1532	MHMXW340-09	JF753711
7399	Autochton Burns01DHJ03	Pyrginae	03-SRNP-21795	MHAHK434-07	JF760414
7400	Autochton Burns01DHJ03	Pyrginae	05-SRNP-5444	MHAHE597-06	GU149435
7401	Autochton Burns01DHJ04	Pyrginae	01-SRNP-2361	MHAHK417-07	JF760431
7402	Autochton Burns01DHJ03	Pyrginae	03-SRNP-8841	MHAHK419-07	JF760408
7403	Autochton Burns01DHJ03	Pyrginae	02-SRNP-7452	MHAHK453-07	JF760421
7404	Autochton Burns01DHJ04	Pyrginae	05-SRNP-42409	MHAHE599-06	GU149434
7405	Autochton Burns01DHJ03	Pyrginae	02-SRNP-7369	MHAHK454-07	JF760422
7406	Autochton Burns01DHJ03	Pyrginae	02-SRNP-19075	MHAHK455-07	JF760423
7407	Autochton Burns01DHJ03	Pyrginae	07-SRNP-1204	MHMXK052-07	JF761675
7408	Autochton Burns01DHJ03	Pyrginae	00-SRNP-11807	MHAHK411-07	JF760407
7409	Autochton Burns01DHJ03	Pyrginae	01-SRNP-2359	MHAHK407-07	JF760404
7410	Autochton Burns01DHJ03	Pyrginae	02-SRNP-6764	MHAHK448-07	JF760420
7411	Autochton Burns01DHJ03	Pyrginae	01-SRNP-2360	MHAHK410-07	JF760406
7412	Autochton Burns01DHJ03	Pyrginae	01-SRNP-25357	MHAHK451-07	GU162122
7413	Autochton Burns01DHJ03	Pyrginae	07-SRNP-524	MHMXK049-07	JF761677
7414	Autochton Burns01DHJ03	Pyrginae	07-SRNP-466	MHMXK048-07	JF761678
7415	Autochton Burns01DHJ03	Pyrginae	06-SRNP-34485	MHAHJ888-07	JF752473
7416	Autochton Burns01DHJ03	Pyrginae	03-SRNP-9633	MHAHK421-07	JF760409
7417	Autochton Burns01DHJ03	Pyrginae	06-SRNP-3321	MHAHH508-06	GU155206
7418	Autochton Burns01DHJ03	Pyrginae	06-SRNP-3415	MHAHH507-06	GU155205
7419	Autochton Burns01DHJ03	Pyrginae	07-SRNP-40843	MHMXN301-07	JF761674
7420	Autochton Burns01DHJ03	Pyrginae	03-SRNP-9631	MHAHK445-07	JF760419
7421	Autochton Burns01DHJ03	Pyrginae	00-SRNP-1531	MHAHK408-07	JF760405
7422	Autochton Burns01DHJ03	Pyrginae	03-SRNP-21535	MHAHK429-07	JF760413
7423	Autochton Burns01DHJ03	Pyrginae	02-SRNP-15018	MHAHK456-07	JF760424
7424	Autochton Burns01DHJ03	Pyrginae	06-SRNP-43554	MHAHJ556-07	JF752472
7425	Autochton Burns01DHJ03	Pyrginae	02-SRNP-15017	MHAHK440-07	JF760416
7426	Autochton Burns01DHJ03	Pyrginae	03-SRNP-8817	MHAHK444-07	JF760418
7427	Autochton Burns01DHJ03	Pyrginae	01-SRNP-3734	MHAHK406-07	JF760403
7428	Autochton Burns01DHJ03	Pyrginae	03-SRNP-9632	MHAHK428-07	JF760412
7429	Autochton Burns01DHJ04	Pyrginae	03-SRNP-6742	MHAHK442-07	JF760440
7430	Autochton Burns01DHJ04	Pyrginae	02-SRNP-17340	MHAHK446-07	JF760441
7431	Autochton Burns01DHJ04	Pyrginae	07-SRNP-76	MHMXK046-07	JF761680
7432	Autochton Burns01DHJ03	Pyrginae	07-SRNP-834	MHMXK051-07	JF761676
7433	Autochton Burns01DHJ03	Pyrginae	03-SRNP-21652	MHAHK441-07	JF760417
7434	Autochton Burns01DHJ03	Pyrginae	03-SRNP-9634	MHAHK436-07	JF760415
7435	Autochton Burns01DHJ03	Pyrginae	03-SRNP-6163	MHAHK427-07	JF760411
7436	Autochton Burns01DHJ03	Pyrginae	03-SRNP-21660	MHAHK424-07	JF760410
7437	Autochton Burns01DHJ03	Pyrginae	06-SRNP-43772	MHAHJ555-07	JF752471
7438	Autochton Burns01DHJ03	Pyrginae	07-SRNP-3126	MHMXP154-08	JF761673
7439	Autochton Burns01DHJ03	Pyrginae	08-SRNP-1863	MHMXW316-09	JF753721
7440	Autochton Burns01DHJ03	Pyrginae	08-SRNP-724	MHMXW330-09	JF753719
7441	Autochton Burns01DHJ03	Pyrginae	01-SRNP-1271	MHAHK404-07	JF760402
7442	Autochton Burns01DHJ03	Pyrginae	08-SRNP-723	MHMXW335-09	JF753720
7443	Autochton Burns01DHJ04	Pyrginae	03-SRNP-5976	MHAHK418-07	JF760432
7444	Autochton Burns01DHJ04	Pyrginae	06-SRNP-40471	MHAHG103-06	GU151208
7445	Autochton Burns01DHJ04	Pyrginae	02-SRNP-5075	MHAHK457-07	JF760446
7446	Autochton Burns01DHJ05	Pyrginae	07-SRNP-2033	MHMXN296-07	JF761681
7447	Autochton Burns01DHJ04	Pyrginae	02-SRNP-17338	MHAHI307-06	GU155829

Tree Order	Species	Subfamily	ACG Sampleid	BOLD Processid	Genbank Accession
7448	Autochton Burns01DHJ04	Pyrginae	08-SRNP-1535	MHMXW321-09	JF753723
7449	Autochton Burns01DHJ04	Pyrginae	08-SRNP-1648	MHMXW331-09	JF753722
7450	Autochton Burns01DHJ04	Pyrginae	05-SRNP-4301	MHAHF303-06	GU150262
7451	Autochton Burns01DHJ04	Pyrginae	05-SRNP-32030	MHAHF301-06	GU150260
7452	Autochton Burns01DHJ04	Pyrginae	01-SRNP-2650	MHAHK409-07	JF760426
7453	Autochton Burns01DHJ04	Pyrginae	01-SRNP-2651	CSRII097-04	DQ291896
7454	Autochton Burns01DHJ04	Pyrginae	01-SRNP-2652	CSRII098-04	DQ291897
7455	Autochton Burns01DHJ04	Pyrginae	02-SRNP-17339	MHAHK447-07	JF760442
7456	Autochton Burns01DHJ04	Pyrginae	04-SRNP-60519	MHAHE099-05	GU149431
7457	Autochton Burns01DHJ04	Pyrginae	01-SRNP-1371	MHAHK414-07	JF760428
7458	Autochton Burns01DHJ04	Pyrginae	05-SRNP-43435	MHAHF692-06	GU150263
7459	Autochton Burns01DHJ04	Pyrginae	03-SRNP-8819	MHAHK423-07	JF760433
7460	Autochton Burns01DHJ04	Pyrginae	03-SRNP-21425	MHAHK425-07	JF760434
7461	Autochton Burns01DHJ04	Pyrginae	08-SRNP-41353	MHMXX486-09	GU666366
7462	Autochton Burns01DHJ04	Pyrginae	06-SRNP-43553	MHAHJ557-07	JF752474
7463	Autochton Burns01DHJ04	Pyrginae	03-SRNP-9628	MHAHK432-07	JF760436
7464	Autochton Burns01DHJ04	Pyrginae	07-SRNP-1157	MHMXK050-07	JF761679
7465	Autochton Burns01DHJ04	Pyrginae	02-SRNP-30483	MHAHK452-07	JF760445
7466	Autochton Burns01DHJ04	Pyrginae	02-SRNP-1953	MHAHK450-07	JF760444
7467	Autochton Burns01DHJ04	Pyrginae	02-SRNP-3878	MHAHK449-07	JF760443
7468	Autochton Burns01DHJ04	Pyrginae	02-SRNP-28933	MHAHK439-07	JF760439
7469	Autochton Burns01DHJ04	Pyrginae	03-SRNP-9630	MHAHK431-07	JF760435
7470	Autochton Burns01DHJ04	Pyrginae	00-SRNP-11621	MHAHK415-07	JF760429
7471	Autochton Burns01DHJ04	Pyrginae	00-SRNP-12409	MHAHK412-07	JF760427
7472	Autochton Burns01DHJ04	Pyrginae	01-SRNP-3899	MHAHK402-07	JF760425
7473	Autochton Burns01DHJ04	Pyrginae	06-SRNP-65526	MHAHJ684-07	JF752475
7474	Autochton Burns01DHJ04	Pyrginae	01-SRNP-25365	MHAHI304-06	GU155828
7475	Autochton Burns01DHJ04	Pyrginae	02-SRNP-19076	MHAHI303-06	GU155827
7476	Autochton Burns01DHJ04	Pyrginae	04-SRNP-3794	MHAHD909-05	GU161295
7477	Autochton Burns01DHJ04	Pyrginae	02-SRNP-2648	MHAHK438-07	JF760438
7478	Autochton Burns01DHJ04	Pyrginae	01-SRNP-3735	MHAHK416-07	JF760430
7479	Autochton Burns01DHJ04	Pyrginae	04-SRNP-60826	MHAHD912-05	GU161294
7480	Autochton Burns01DHJ04	Pyrginae	06-SRNP-65122	MHAHJ887-07	JF752476
7481	Autochton Burns01DHJ04	Pyrginae	06-SRNP-34487	MHAHJ889-07	JF752477
7482	Autochton Burns01DHJ04	Pyrginae	05-SRNP-21742	MHAHF304-06	GU150261
7483	Autochton Burns01DHJ04	Pyrginae	03-SRNP-21668	CSRII101-04	DQ291899
7484	Autochton Burns01DHJ04	Pyrginae	03-SRNP-9627	CSRII103-04	DQ291901
7485	Autochton Burns01DHJ04	Pyrginae	03-SRNP-9626	MHAHK433-07	JF760437
7486	Autochton Burns01DHJ04	Pyrginae	02-SRNP-19077	MHAHK458-07	JF760447
7487	Autochton Burns01DHJ04	Pyrginae	08-SRNP-5469	MHMXY1083-09	GU666456
7488	Autochton Burns01DHJ05	Pyrginae	00-SRNP-11187	CSRII096-04	DQ291895
7489	Autochton Burns01DHJ05	Pyrginae	06-SRNP-2060	MHAHG631-06	GU151207
7490	Autochton Burns01DHJ05	Pyrginae	01-SRNP-3733	MHAHK403-07	JF760448
7491	Autochton Burns01DHJ05	Pyrginae	03-SRNP-9837	CSRII104-04	DQ291902
7492	Autochton Burns01DHJ05	Pyrginae	03-SRNP-21655	MHAHK435-07	JF760452
7493	Autochton Burns01DHJ05	Pyrginae	03-SRNP-31321	CSRII102-04	DQ291900
7494	Autochton Burns01DHJ05	Pyrginae	06-SRNP-9700	MHAHJ784-07	JF752478
7495	Autochton Burns01DHJ05	Pyrginae	03-SRNP-13047.1	MHAHK420-07	JF760451
7496	Autochton Burns01DHJ05	Pyrginae	03-SRNP-10151	CSRII099-04	DQ291898
7497	Autochton Burns01DHJ05	Pyrginae	04-SRNP-22708	MHAHD910-05	GU161292
7498	Autochton Burns01DHJ05	Pyrginae	04-SRNP-22710	MHAHD911-05	GU161293
7499	Autochton Burns01DHJ05	Pyrginae	02-SRNP-14078	MHAHI308-06	GU155830
7500	Autochton Burns01DHJ05	Pyrginae	01-SRNP-3736	MHAHK405-07	JF760449
7501	Autochton Burns01DHJ05	Pyrginae	01-SRNP-1893	MHAHK413-07	JF760450
7502	Autochton Burns01DHJ05	Pyrginae	08-SRNP-1358	MHMXW319-09	JF753724
7503	Autochton Burns01DHJ05	Pyrginae	08-SRNP-6305	MHMXY1084-09	GU666457

Tree Order	Species	Subfamily	ACG Sampleid	BOLD Processid	Genbank Accession
7504	Cabares potrillo	Pyrginae	09-SRNP-57001	MHMYH186-10	HM887333
7505	Cabares potrillo	Pyrginae	06-SRNP-58154	MHAHJ833-07	JF752493
7506	Cabares potrillo	Pyrginae	95-SRNP-6015	CSCR049-04	DQ291952
7507	Cabares potrillo	Pyrginae	09-SRNP-57000	MHMYH184-10	HM887331
7508	Cabares potrillo	Pyrginae	09-SRNP-57002	MHMYH185-10	HM887332
7509	Cabares potrillo	Pyrginae	09-SRNP-57090	MHMYG2497-10	HM885925
7510	Mysoria ambigua	Pyrginae	03-SRNP-1431	CSCR380-04	DQ292659
7511	Mysoria ambigua	Pyrginae	08-SRNP-24392	MHMYB120-09	GU649703
7512	Mysoria ambigua	Pyrginae	08-SRNP-55431	MHMXW083-09	JF753985
7513	Mysoria ambigua	Pyrginae	05-SRNP-57606	MHAHL028-07	JF762374
7514	Mysoria ambigua	Pyrginae	05-SRNP-55647	MHAHL027-07	JF762373
7515	Mysoria ambigua	Pyrginae	04-SRNP-23872	MHAHD833-05	GU161677
7516	Mysoria ambigua	Pyrginae	04-SRNP-14802	MHAHD832-05	GU161676
7517	Mysoria ambigua	Pyrginae	04-SRNP-14797	MHAHD831-05	GU161674
7518	Mysoria ambigua	Pyrginae	04-SRNP-14796	MHAHD830-05	GU161672
7519	Mysoria ambigua	Pyrginae	04-SRNP-14795	MHAHD829-05	GU161675
7520	Mysoria ambigua	Pyrginae	04-SRNP-14800	MHAHC716-05	DQ292665
7521	Mysoria ambigua	Pyrginae	04-SRNP-14801	MHAHC715-05	DQ292664
7522	Mysoria ambigua	Pyrginae	05-SRNP-55178	MHAHC713-05	DQ292662
7523	Mysoria ambigua	Pyrginae	04-SRNP-14558	MHAHC711-05	DQ292660
7524	Mysoria ambigua	Pyrginae	04-SRNP-14099	MHAHC712-05	DQ292661
7525	Mysoria ambigua	Pyrginae	05-SRNP-55193	MHAHC714-05	DQ292663
7526	Mysoria ambigua	Pyrginae	05-SRNP-55175	MHAHL029-07	JF762375
7527	Mysoria ambigua	Pyrginae	08-SRNP-55530	MHMXW084-09	JF753986
7528	Mysoria ambigua	Pyrginae	03-SRNP-1293	CSCR379-04	DQ292658
7529	Mysoria ambigua	Pyrginae	04-SRNP-14584	MHAHD828-05	GU161673
7530	Mysoria ambigua	Pyrginae	08-SRNP-24269	MHMYB122-09	GU649697
7531	Jemadia Burns01	Pyrginae	01-SRNP-9029	CSCR118-04	DQ292569
7532	Jemadia Burns01	Pyrginae	04-SRNP-30754	MHAHC145-05	DQ292573
7533	Jemadia Burns01	Pyrginae	03-SRNP-21823	MHAHC153-05	DQ292574
7534	Jemadia Burns01	Pyrginae	03-SRNP-21528	MHAHC161-05	DQ292575
7535	Jemadia Burns01	Pyrginae	04-SRNP-32358	MHAHD741-05	GU161555
7536	Jemadia Burns01	Pyrginae	04-SRNP-34396	MHAHD742-05	GU161554
7537	Jemadia Burns01	Pyrginae	05-SRNP-31969	MHAHG484-06	GU151442
7538	Jemadia Burns01	Pyrginae	00-SRNP-4482	CSRII253-04	DQ292571
7539	Jemadia Burns01	Pyrginae	04-SRNP-56811	MHAHF455-06	GU150503
7540	Jemadia Burns01	Pyrginae	05-SRNP-31086	MHAHF454-06	GU150502
7541	Jemadia Burns01	Pyrginae	96-SRNP-12846	CSRII254-04	DQ292572
7542	Jemadia Burns01	Pyrginae	02-SRNP-13059	CSCR119-04	DQ292570
7543	Jemadia Burns01	Pyrginae	09-SRNP-30034	MHMYC450-09	GU649882
7544	Elbella scylla	Pyrginae	04-SRNP-13405	MHAHD819-05	GU161469
7545	Elbella scylla	Pyrginae	07-SRNP-32122	MHMXO770-08	JF762170
7546	Elbella scylla	Pyrginae	07-SRNP-65187	MHMXR813-08	JF762167
7547	Elbella scylla	Pyrginae	08-SRNP-70068	MHMXW075-09	JF753883
7548	Elbella scylla	Pyrginae	08-SRNP-70229	MHMXW076-09	JF753884
7549	Elbella scylla	Pyrginae	05-SRNP-31078	MHAHF135-06	GU150398
7550	Elbella scylla	Pyrginae	05-SRNP-12075	MHAHF452-06	GU150396
7551	Elbella scylla	Pyrginae	05-SRNP-31568	MHAHF453-06	GU150397
7552	Elbella scylla	Pyrginae	08-SRNP-72172	MHMXX667-09	JF777898
7553	Elbella scylla	Pyrginae	08-SRNP-70307	MHMXW072-09	JF753881
7554	Elbella scylla	Pyrginae	08-SRNP-55460	MHMXW071-09	JF753880
7555	Elbella scylla	Pyrginae	08-SRNP-55401	MHMXW070-09	JF753879
7556	Elbella scylla	Pyrginae	08-SRNP-55397	MHMXW069-09	JF753878
7557	Elbella scylla	Pyrginae	08-SRNP-70314	MHMXW068-09	JF753877
7558	Elbella scylla	Pyrginae	06-SRNP-67774	MHMXH895-07	JF760666
7559	Elbella scylla	Pyrginae	05-SRNP-31077	MHAHF451-06	GU150395

Tree Order	Species	Subfamily	ACG Sampleid	BOLD Processid	Genbank Accession
7560	Elbella scylla	Pyrginae	05-SRNP-55736	MHAHF136-06	GU150400
7561	Elbella scylla	Pyrginae	05-SRNP-13170	MHAHF134-06	GU150399
7562	Elbella scylla	Pyrginae	04-SRNP-13706	MHAHD822-05	GU161472
7563	Elbella scylla	Pyrginae	04-SRNP-13406	MHAHD821-05	GU161468
7564	Elbella scylla	Pyrginae	04-SRNP-13082	MHAHD820-05	GU161470
7565	Elbella scylla	Pyrginae	04-SRNP-13081	MHAHD818-05	GU161471
7566	Elbella scylla	Pyrginae	04-SRNP-21136	MHAHC137-05	DQ292385
7567	Elbella scylla	Pyrginae	07-SRNP-21861	MHMXR811-08	JF762168
7568	Elbella scylla	Pyrginae	07-SRNP-57964	MHMXR810-08	JF762169
7569	Elbella scylla	Pyrginae	03-SRNP-232	CSCR361-04	DQ292383
7570	Elbella scylla	Pyrginae	07-SRNP-65934	MHMXT083-08	JF762166
7571	Elbella scylla	Pyrginae	08-SRNP-70185	MHMXW073-09	JF753882
7572	Elbella scylla	Pyrginae	03-SRNP-635	CSCR362-04	DQ292384
7573	Elbella scylla	Pyrginae	08-SRNP-23853	MHMYB121-09	GU649696
7574	Elbella patrobas	Pyrginae	07-SRNP-31402	MHAHL418-07	JF762162
7575	Elbella patrobas	Pyrginae	06-SRNP-33024	MHAHJ791-07	JF752763
7576	Elbella patrobas	Pyrginae	03-SRNP-6393	CSCR508-04	DQ292382
7577	Elbella patrobas	Pyrginae	07-SRNP-2792	MHMXO756-08	JF762164
7578	Elbella patrobas	Pyrginae	07-SRNP-4287	MHMXR633-08	JF762163
7579	Elbella patrobas	Pyrginae	08-SRNP-2588	MHMXW007-09	JF753876
7580	Elbella patrobasDHJ05	Pyrginae	07-SRNP-3722	MHMXO760-08	JF762165
7581	Elbella merops	Pyrginae	05-SRNP-32782	MHAHG483-06	GU151398
7582	Elbella merops	Pyrginae	08-SRNP-70940	MHMXX662-09	JF777897
7583	Elbella merops	Pyrginae	05-SRNP-41519	MHAHF450-06	GU150394
7584	Elbella merops	Pyrginae	09-SRNP-30538	MHMYC451-09	GU649883
7585	Elbella merops	Pyrginae	09-SRNP-71893	MHMYE1438-09	GU653558
7586	Jemadia pseudognetus	Pyrginae	01-SRNP-1773	CSCR116-04	DQ292576
7587	Jemadia pseudognetus	Pyrginae	02-SRNP-3211	CSRII250-04	DQ292579
7588	Jemadia pseudognetus	Pyrginae	08-SRNP-4381	MHMXW010-09	JF753963
7589	Jemadia pseudognetus	Pyrginae	08-SRNP-639	MHMXT065-08	JF762297
7590	Jemadia pseudognetus	Pyrginae	07-SRNP-65061	MHMXR634-08	JF762299
7591	Jemadia pseudognetus	Pyrginae	07-SRNP-45220	MHMXO753-08	JF762300
7592	Jemadia pseudognetus	Pyrginae	07-SRNP-2561	MHMXO751-08	JF762302
7593	Jemadia pseudognetus	Pyrginae	07-SRNP-1947	MHMXO750-08	JF762303
7594	Jemadia pseudognetus	Pyrginae	07-SRNP-2837	MHMXO749-08	JF762304
7595	Jemadia pseudognetus	Pyrginae	07-SRNP-3517	MHMXO742-08	JF762305
7596	Jemadia pseudognetus	Pyrginae	05-SRNP-7825	MHAHG682-06	GU151443
7597	Jemadia pseudognetus	Pyrginae	05-SRNP-4394	MHAHF463-06	GU150509
7598	Jemadia pseudognetus	Pyrginae	05-SRNP-2922	MHAHF462-06	GU150508
7599	Jemadia pseudognetus	Pyrginae	05-SRNP-3192	MHAHF461-06	GU150504
7600	Jemadia pseudognetus	Pyrginae	05-SRNP-4069	MHAHF460-06	GU150505
7601	Jemadia pseudognetus	Pyrginae	04-SRNP-4113	MHAHD737-05	GU161556
7602	Jemadia pseudognetus	Pyrginae	06-SRNP-1088	MHAHG337-06	GU151444
7603	Jemadia pseudognetus	Pyrginae	05-SRNP-4395	MHAHF458-06	GU150506
7604	Jemadia pseudognetus	Pyrginae	02-SRNP-18659	CSRII251-04	DQ292580
7605	Jemadia pseudognetus	Pyrginae	02-SRNP-18802	CSRII252-04	DQ292581
7606	Jemadia pseudognetus	Pyrginae	04-SRNP-26478	MHAHF459-06	GU150507
7607	Jemadia pseudognetus	Pyrginae	06-SRNP-67850	MHMXO1035-08	JF762306
7608	Jemadia pseudognetus	Pyrginae	07-SRNP-2602	MHMXO752-08	JF762301
7609	Jemadia pseudognetus	Pyrginae	08-SRNP-289	MHMXT064-08	JF762298
7610	Jemadia pseudognetus	Pyrginae	01-SRNP-2420	CSCR117-04	DQ292577
7611	Jemadia pseudognetus	Pyrginae	02-SRNP-1552	CSRII249-04	DQ292578
7612	Jemadia pseudognetus	Pyrginae	08-SRNP-65423	MHMXY1126-09	GU666418
7613	Parelbella macleannani	Pyrginae	06-SRNP-8158	MHAHJ910-07	JF752970
7614	Parelbella macleannani	Pyrginae	04-SRNP-24065	MHAHD743-05	GU161761
7615	Parelbella macleannani	Pyrginae	04-SRNP-23689	MHAHD748-05	GU161764

Tree Order	Species	Subfamily	ACG Sampleid	BOLD Processid	Genbank Accession
7616	Parelbella macleannani	Pyrginae	04-SRNP-21620	MHAHD745-05	GU161760
7617	Parelbella macleannani	Pyrginae	04-SRNP-23317	MHAHD744-05	GU161762
7618	Parelbella macleannani	Pyrginae	04-SRNP-23795	MHAHD747-05	GU161765
7619	Parelbella macleannani	Pyrginae	04-SRNP-23300	MHAHD746-05	GU161763
7620	Parelbella macleannani	Pyrginae	01-SRNP-163	CSRII546-04	DQ292831
7621	Parelbella macleannani	Pyrginae	00-SRNP-571	CSRII545-04	DQ292830
7622	Parelbella macleannani	Pyrginae	07-SRNP-19	MHMXK147-07	JF762521
7623	Parelbella macleannani	Pyrginae	07-SRNP-425	MHMXK155-07	JF762520
7624	Parelbella macleannani	Pyrginae	07-SRNP-20933	MHAHL416-07	JF762513
7625	Parelbella macleannani	Pyrginae	06-SRNP-23440	MHAHL417-07	JF762514
7626	Parelbella macleannani	Pyrginae	07-SRNP-2509	MHMXO744-08	JF762519
7627	Parelbella macleannani	Pyrginae	07-SRNP-3362	MHMXO745-08	JF762518
7628	Parelbella macleannani	Pyrginae	07-SRNP-2510	MHMXO746-08	JF762517
7629	Parelbella macleannani	Pyrginae	07-SRNP-23463	MHMXR636-08	JF762516
7630	Parelbella macleannani	Pyrginae	07-SRNP-22879	MHMXR637-08	JF762515
7631	Parelbella macleannani	Pyrginae	09-SRNP-20914	MHMYH111-10	HM887263
7632	Yanguna cosyra	Pyrginae	03-SRNP-5253	CSCR457-04	DQ293932
7633	Yanguna cosyra	Pyrginae	03-SRNP-3068	CSCR458-04	DQ293933
7634	Yanguna cosyra	Pyrginae	04-SRNP-35097	MHAHC106-05	DQ293934
7635	Yanguna cosyra	Pyrginae	04-SRNP-35058	MHAHC114-05	DQ293935
7636	Yanguna cosyra	Pyrginae	04-SRNP-35285	MHAHC122-05	DQ293936
7637	Yanguna cosyra	Pyrginae	04-SRNP-35120	MHAHC130-05	DQ293937
7638	Yanguna cosyra	Pyrginae	04-SRNP-35115	MHAHC138-05	DQ293938
7639	Yanguna cosyra	Pyrginae	04-SRNP-35110	MHAHC146-05	DQ293939
7640	Yanguna cosyra	Pyrginae	04-SRNP-35116	MHAHC154-05	DQ293940
7641	Yanguna cosyra	Pyrginae	04-SRNP-35122	MHAHC162-05	DQ293941
7642	Yanguna cosyra	Pyrginae	05-SRNP-35027	MHAHL008-07	JF763512
7643	Yanguna cosyra	Pyrginae	05-SRNP-35042	MHAHL009-07	JF763513
7644	Yanguna cosyra	Pyrginae	05-SRNP-35054	MHAHL010-07	JF763514
7645	Yanguna cosyra	Pyrginae	06-SRNP-65100	MHAHL431-07	JF763515
7646	Yanguna cosyra	Pyrginae	07-SRNP-41792	MHMXO761-08	JF763522
7647	Yanguna cosyra	Pyrginae	07-SRNP-2192	MHMXO762-08	JF763521
7648	Yanguna cosyra	Pyrginae	07-SRNP-2272	MHMXO763-08	JF763520
7649	Yanguna cosyra	Pyrginae	07-SRNP-41479	MHMXO764-08	JF763519
7650	Yanguna cosyra	Pyrginae	07-SRNP-41979	MHMXO765-08	JF763518
7651	Yanguna cosyra	Pyrginae	07-SRNP-36506	MHMXR806-08	JF763517
7652	Yanguna cosyra	Pyrginae	07-SRNP-35955	MHMXR807-08	JF763516
7653	Yanguna cosyra	Pyrginae	08-SRNP-35914	MHMXX668-09	JF778646
7654	Yanguna cosyra	Pyrginae	08-SRNP-31712	MHMXY1120-09	GU666420
7655	Jonaspyge aesculapus	Pyrginae	03-SRNP-3336	MHAHE105-05	GU149691
7656	Jonaspyge aesculapus	Pyrginae	04-SRNP-35065	MHAHC152-05	DQ292603
7657	Jonaspyge aesculapus	Pyrginae	98-SRNP-2286	MHAHK573-07	JF760830
7658	Jonaspyge aesculapus	Pyrginae	98-SRNP-2048.1	MHAHK575-07	JF760832
7659	Jonaspyge aesculapus	Pyrginae	97-SRNP-514	CSCR120-04	DQ292599
7660	Jonaspyge aesculapus	Pyrginae	04-SRNP-35064	CSRII215-04	DQ292601
7661	Jonaspyge aesculapus	Pyrginae	04-SRNP-35130	MHAHC144-05	DQ292602
7662	Jonaspyge aesculapus	Pyrginae	05-SRNP-35055	MHAHF151-06	GU150510
7663	Jonaspyge aesculapus	Pyrginae	02-SRNP-23111	CSCR121-04	DQ292600
7664	Jonaspyge aesculapus	Pyrginae	04-SRNP-35066	MHAHC160-05	DQ292604
7665	Jonaspyge aesculapus	Pyrginae	97-SRNP-2105	MHAHK574-07	JF760831
7666	Jonaspyge aesculapus	Pyrginae	00-SRNP-9360	MHAHK576-07	JF760833
7667	Creonpyge creon	Pyrginae	06-SRNP-36175	MHMXK158-07	JF762001
7668	Creonpyge creon	Pyrginae	02-SRNP-8028	MHAHK583-07	JF760631
7669	Creonpyge creon	Pyrginae	01-SRNP-7147	MHAHK580-07	JF760629
7670	Creonpyge creon	Pyrginae	01-SRNP-7399	MHAHK579-07	JF760628
7671	Creonpyge creon	Pyrginae	02-SRNP-9441	MHAHK577-07	JF760626

Tree Order	Species	Subfamily	ACG Sampleid	BOLD Processid	Genbank Accession
7672	Creonpyge creon	Pyrginae	03-SRNP-3109	MHAHE106-05	GU149566
7673	Creonpyge creon	Pyrginae	98-SRNP-2317	MHAHK578-07	JF760627
7674	Creonpyge creon	Pyrginae	03-SRNP-3108	CSCR355-04	DQ292226
7675	Creonpyge creon	Pyrginae	02-SRNP-8026	MHAHK582-07	JF760630
7676	Creonpyge creon	Pyrginae	06-SRNP-36176	MHAHL430-07	JF762000
7677	Melanopyge erythrosticta	Pyrginae	03-SRNP-5457	CSCR370-04	DQ292612
7678	Melanopyge erythrosticta	Pyrginae	00-SRNP-15831	CSCR123-04	DQ292611
7679	Melanopyge erythrosticta	Pyrginae	04-SRNP-34289	MHAHD728-05	GU161575
7680	Melanopyge erythrosticta	Pyrginae	05-SRNP-894	MHAHF149-06	GU150524
7681	Melanopyge Burns01	Pyrginae	07-SRNP-992	MHMXK142-07	JF762329
7682	Melanopyge Burns01	Pyrginae	09-SRNP-722	MHMYC453-09	GU649885
7683	Melanopyge Burns01	Pyrginae	05-SRNP-34170	MHAHG189-06	GU151453
7684	Melanopyge Burns01	Pyrginae	07-SRNP-2732	MHMXO767-08	JF762327
7685	Melanopyge Burns01	Pyrginae	05-SRNP-3132	MHAHF148-06	GU150523
7686	Melanopyge Burns01	Pyrginae	05-SRNP-309	MHAHF147-06	GU150522
7687	Melanopyge Burns01	Pyrginae	04-SRNP-3561	MHAHD731-05	GU161574
7688	Melanopyge Burns01	Pyrginae	04-SRNP-3562	MHAHD730-05	GU161572
7689	Melanopyge Burns01	Pyrginae	04-SRNP-4008	MHAHD729-05	GU161573
7690	Melanopyge Burns01	Pyrginae	04-SRNP-3996	MHAHD727-05	GU161571
7691	Melanopyge Burns01	Pyrginae	04-SRNP-3563	MHAHC720-05	DQ292610
7692	Melanopyge Burns01	Pyrginae	07-SRNP-65400	MHMXO766-08	JF762328
7693	Melanopyge Burns01	Pyrginae	02-SRNP-14905	CSCR122-04	DQ292609
7694	Melanopyge Burns01	Pyrginae	05-SRNP-34206	MHAHG188-06	GU151454
7695	Melanopyge Burns01	Pyrginae	08-SRNP-71628	MHMXX959-09	JF778067
7696	Melanopyge Burns01	Pyrginae	09-SRNP-65504	MHMYC454-09	GU649878
7697	Pyrrhopyge zenodorusDHJ01	Pyrginae	04-SRNP-41678	MHAHD823-05	GU161799
7698	Pyrrhopyge zenodorusDHJ01	Pyrginae	00-SRNP-1645	MHAHH091-06	GU155464
7699	Pyrrhopyge zenodorusDHJ01	Pyrginae	01-SRNP-9155	MHAHH098-06	GU155466
7700	Pyrrhopyge zenodorusDHJ01	Pyrginae	06-SRNP-1697	MHAHH431-06	GU155467
7701	Pyrrhopyge zenodorusDHJ01	Pyrginae	06-SRNP-42909	MHAHI515-06	GU156286
7702	Pyrrhopyge zenodorusDHJ01	Pyrginae	06-SRNP-4369	MHAHJ649-07	JF753096
7703	Pyrrhopyge zenodorusDHJ03	Pyrginae	05-SRNP-59692	MHAHF168-06	GU150736
7704	Pyrrhopyge zenodorusDHJ03	Pyrginae	06-SRNP-60372	MHMXK146-07	JF762731
7705	Pyrrhopyge zenodorusDHJ03	Pyrginae	05-SRNP-66260	MHAHG180-06	GU151590
7706	Pyrrhopyge zenodorusDHJ03	Pyrginae	05-SRNP-66171	MHAHG181-06	GU151589
7707	Pyrrhopyge zenodorusDHJ03	Pyrginae	99-SRNP-5161	MHAHH105-06	GU155465
7708	Pyrrhopyge zenodorusDHJ03	Pyrginae	07-SRNP-3197	MHMXO772-08	JF762730
7709	Pyrrhopyge zenodorusDHJ03	Pyrginae	08-SRNP-70278	MHMXW086-09	JF754120
7710	Pyrrhopyge zenodorusDHJ03	Pyrginae	08-SRNP-70280	MHMXW088-09	JF754121
7711	Pyrrhopyge zenodorusDHJ02	Pyrginae	08-SRNP-72317	MHMXY1121-09	HM893816
7712	Pyrrhopyge zenodorusDHJ02	Pyrginae	00-SRNP-20566	MHAHH095-06	GU155468
7713	Pyrrhopyge zenodorusDHJ02	Pyrginae	07-SRNP-65619	MHMXR812-08	JF762725
7714	Pyrrhopyge zenodorusDHJ02	Pyrginae	07-SRNP-45223	MHMXO771-08	JF762727
7715	Pyrrhopyge zenodorusDHJ02	Pyrginae	06-SRNP-9502	MHMXK144-07	JF762729
7716	Pyrrhopyge zenodorusDHJ02	Pyrginae	06-SRNP-46215	MHAHJ650-07	JF753099
7717	Pyrrhopyge zenodorusDHJ02	Pyrginae	06-SRNP-6822	MHAHJ648-07	JF753098
7718	Pyrrhopyge zenodorusDHJ02	Pyrginae	06-SRNP-7823	MHAHJ515-07	JF753097
7719	Pyrrhopyge zenodorusDHJ02	Pyrginae	06-SRNP-20133	MHAHH432-06	GU155484
7720	Pyrrhopyge zenodorusDHJ02	Pyrginae	99-SRNP-12050	MHAHH104-06	GU155479
7721	Pyrrhopyge zenodorusDHJ02	Pyrginae	99-SRNP-5532	MHAHH103-06	GU155482
7722	Pyrrhopyge zenodorusDHJ02	Pyrginae	02-SRNP-14950	MHAHH102-06	GU155483
7723	Pyrrhopyge zenodorusDHJ02	Pyrginae	01-SRNP-1501	MHAHH101-06	GU155477
7724	Pyrrhopyge zenodorusDHJ02	Pyrginae	00-SRNP-14177	MHAHH100-06	GU155478
7725	Pyrrhopyge zenodorusDHJ02	Pyrginae	00-SRNP-2007	MHAHH097-06	GU155481
7726	Pyrrhopyge zenodorusDHJ02	Pyrginae	00-SRNP-22185	MHAHH094-06	GU155469
7727	Pyrrhopyge zenodorusDHJ02	Pyrginae	00-SRNP-14143	MHAHH093-06	GU155470

Tree Order	Species	Subfamily	ACG Sampleid	BOLD Processid	Genbank Accession
7728	Pyrrhopyge zenodorusDHJ02	Pyrginae	00-SRNP-11309	MHAHH092-06	GU155471
7729	Pyrrhopyge zenodorusDHJ02	Pyrginae	00-SRNP-2010	MHAHH090-06	GU155475
7730	Pyrrhopyge zenodorusDHJ02	Pyrginae	02-SRNP-23595	MHAHH089-06	GU155472
7731	Pyrrhopyge zenodorusDHJ02	Pyrginae	02-SRNP-14179	MHAHH088-06	GU155474
7732	Pyrrhopyge zenodorusDHJ02	Pyrginae	01-SRNP-4744	MHAHH087-06	GU155473
7733	Pyrrhopyge zenodorusDHJ02	Pyrginae	03-SRNP-1421	MHAHH086-06	GU155485
7734	Pyrrhopyge zenodorusDHJ02	Pyrginae	05-SRNP-49652	MHAHG698-06	GU151592
7735	Pyrrhopyge zenodorusDHJ02	Pyrginae	06-SRNP-116	MHAHG697-06	GU151591
7736	Pyrrhopyge zenodorusDHJ02	Pyrginae	05-SRNP-59691	MHAHF169-06	GU150737
7737	Pyrrhopyge zenodorusDHJ02	Pyrginae	04-SRNP-47942	MHAHD827-05	GU161800
7738	Pyrrhopyge zenodorusDHJ02	Pyrginae	04-SRNP-47938	MHAHD826-05	GU161803
7739	Pyrrhopyge zenodorusDHJ02	Pyrginae	04-SRNP-48199	MHAHD825-05	GU161802
7740	Pyrrhopyge zenodorusDHJ02	Pyrginae	04-SRNP-47940	MHAHD824-05	GU161801
7741	Pyrrhopyge zenodorusDHJ02	Pyrginae	02-SRNP-15035	MHAHH099-06	GU155476
7742	Pyrrhopyge zenodorusDHJ02	Pyrginae	07-SRNP-45520	MHMXO768-08	JF762728
7743	Pyrrhopyge zenodorusDHJ02	Pyrginae	07-SRNP-45519	MHMXO773-08	JF762726
7744	Pyrrhopyge zenodorusDHJ02	Pyrginae	06-SRNP-65140	MHAHJ816-07	JF753100
7745	Pyrrhopyge zenodorusDHJ02	Pyrginae	03-SRNP-1418	CSCR402-04	DQ293154
7746	Pyrrhopyge zenodorusDHJ02	Pyrginae	03-SRNP-5466	CSCR403-04	DQ293155
7747	Pyrrhopyge zenodorusDHJ02	Pyrginae	00-SRNP-14681	MHAHH096-06	GU155480
7748	Pyrrhopyge zenodorusDHJ02	Pyrginae	06-SRNP-44426	MHAHK212-07	JF761080
7749	Pyrrhopyge zenodorusDHJ02	Pyrginae	08-SRNP-65047	MHMXT084-08	JF762724
7750	Pyrrhopyge zenodorusDHJ02	Pyrginae	08-SRNP-70328	MHMXW074-09	JF754118
7751	Pyrrhopyge zenodorusDHJ02	Pyrginae	08-SRNP-2416	MHMXW087-09	JF754119
7752	Pyrrhopyge zenodorusDHJ02	Pyrginae	09-SRNP-69426	MHMYE1439-09	HQ992246
7753	Pyrrhopyge zenodorusDHJ02	Pyrginae	09-SRNP-71854	MHMYE1440-09	GU653555
7754	Pyrrhopyge zenodorusDHJ02	Pyrginae	09-SRNP-71853	MHMYE1441-09	GU653556
7755	Pyrrhopyge zenodorusDHJ02	Pyrginae	09-SRNP-41493	MHMYH112-10	HM887264
7756	Pyrrhopyge crida	Pyrginae	07-SRNP-65395	MHMXR817-08	JF762720
7757	Pyrrhopyge crida	Pyrginae	07-SRNP-65251	MHMXR822-08	JF762715
7758	Pyrrhopyge crida	Pyrginae	07-SRNP-65439	MHMXR820-08	JF762717
7759	Pyrrhopyge crida	Pyrginae	07-SRNP-65440	MHMXR824-08	JF762713
7760	Pyrrhopyge crida	Pyrginae	07-SRNP-65431	MHMXR823-08	JF762714
7761	Pyrrhopyge crida	Pyrginae	07-SRNP-65427	MHMXR821-08	JF762716
7762	Pyrrhopyge crida	Pyrginae	07-SRNP-65438	MHMXR819-08	JF762718
7763	Pyrrhopyge crida	Pyrginae	07-SRNP-65517	MHMXR818-08	JF762719
7764	Pyrrhopyge crida	Pyrginae	07-SRNP-65248	MHMXR815-08	JF762722
7765	Pyrrhopyge crida	Pyrginae	07-SRNP-65186	MHMXO774-08	JF762723
7766	Pyrrhopyge crida	Pyrginae	07-SRNP-21159	MHAHL429-07	JF762708
7767	Pyrrhopyge crida	Pyrginae	07-SRNP-65432	MHAHL428-07	JF762707
7768	Pyrrhopyge crida	Pyrginae	05-SRNP-23087	MHAHL108-07	JF762706
7769	Pyrrhopyge crida	Pyrginae	05-SRNP-20774	MHAHL006-07	JF762704
7770	Pyrrhopyge crida	Pyrginae	05-SRNP-20610	MHAHL005-07	JF762703
7771	Pyrrhopyge crida	Pyrginae	05-SRNP-20173	MHAHL004-07	JF762702
7772	Pyrrhopyge crida	Pyrginae	04-SRNP-32575	MHACG850-05	DQ293142
7773	Pyrrhopyge crida	Pyrginae	04-SRNP-34740	MHAHC467-05	DQ293152
7774	Pyrrhopyge crida	Pyrginae	04-SRNP-34738	MHAHC466-05	DQ293151
7775	Pyrrhopyge crida	Pyrginae	04-SRNP-34984	MHAHC465-05	DQ293150
7776	Pyrrhopyge crida	Pyrginae	04-SRNP-33852	MHAHC464-05	DQ293149
7777	Pyrrhopyge crida	Pyrginae	04-SRNP-34744	MHAHC463-05	DQ293148
7778	Pyrrhopyge crida	Pyrginae	04-SRNP-34924	MHAHC462-05	DQ293147
7779	Pyrrhopyge crida	Pyrginae	05-SRNP-20692	MHAHC461-05	DQ293146
7780	Pyrrhopyge crida	Pyrginae	05-SRNP-20180	MHAHC460-05	DQ293145
7781	Pyrrhopyge crida	Pyrginae	05-SRNP-20171	MHAHC459-05	DQ293144
7782	Pyrrhopyge crida	Pyrginae	05-SRNP-20775	MHAHC458-05	DQ293143
7783	Pyrrhopyge crida	Pyrginae	04-SRNP-34275	MHAHC301-05	DQ293139

Tree Order	Species	Subfamily	ACG Sampleid	BOLD Processid	Genbank Accession
7784	Pyrrhopyge crida	Pyrginae	04-SRNP-32812	MHAHC309-05	DQ293140
7785	Pyrrhopyge crida	Pyrginae	04-SRNP-32576	MHAHC311-05	DQ293141
7786	Pyrrhopyge crida	Pyrginae	03-SRNP-21852	MHAHC136-05	DQ293137
7787	Pyrrhopyge crida	Pyrginae	03-SRNP-21862	MHAHC120-05	DQ293135
7788	Pyrrhopyge crida	Pyrginae	04-SRNP-30780	MHAHC104-05	DQ293133
7789	Pyrrhopyge crida	Pyrginae	03-SRNP-21855	MHAHC096-05	DQ293132
7790	Pyrrhopyge crida	Pyrginae	03-SRNP-21853	MHAHC185-05	DQ293138
7791	Pyrrhopyge crida	Pyrginae	05-SRNP-20952	MHAHL007-07	JF762705
7792	Pyrrhopyge crida	Pyrginae	07-SRNP-65585	MHMXR826-08	JF762711
7793	Pyrrhopyge crida	Pyrginae	07-SRNP-65426	MHMXR825-08	JF762712
7794	Pyrrhopyge crida	Pyrginae	03-SRNP-1149	CSCR401-04	DQ293131
7795	Pyrrhopyge crida	Pyrginae	01-SRNP-9130	CSCR215-04	DQ293130
7796	Pyrrhopyge crida	Pyrginae	07-SRNP-65584	MHMXR816-08	JF762721
7797	Pyrrhopyge crida	Pyrginae	07-SRNP-66213	MHMXT081-08	JF762710
7798	Pyrrhopyge crida	Pyrginae	03-SRNP-21536	MHAHC128-05	DQ293136
7799	Pyrrhopyge crida	Pyrginae	03-SRNP-21486	MHAHC112-05	DQ293134
7800	Pyrrhopyge crida	Pyrginae	05-SRNP-20964	MHAHC468-05	DQ293153
7801	Pyrrhopyge crida	Pyrginae	08-SRNP-65031	MHMXT082-08	JF762709
7802	Pyrrhopyge crida	Pyrginae	07-SRNP-65757	MHMXW089-09	JF754116
7803	Pyrrhopyge crida	Pyrginae	07-SRNP-65984	MHMXW090-09	JF754117
7804	Pyrrhopyge crida	Pyrginae	09-SRNP-20790	MHMYH114-10	HM887266
7805	Myscelus amystis	Pyrginae	96-SRNP-365	CSCR136-04	DQ292642
7806	Myscelus amystis	Pyrginae	05-SRNP-20512	MHAHF140-06	GU150555
7807	Myscelus amystis	Pyrginae	04-SRNP-26567	MHAHF144-06	GU150557
7808	Myscelus amystis	Pyrginae	05-SRNP-20288	MHAHF142-06	GU150558
7809	Myscelus amystis	Pyrginae	05-SRNP-20428	MHAHF141-06	GU150554
7810	Myscelus amystis	Pyrginae	05-SRNP-20589	MHAHF139-06	GU150553
7811	Myscelus amystis	Pyrginae	05-SRNP-20514	MHAHF138-06	GU150552
7812	Myscelus amystis	Pyrginae	04-SRNP-24102	MHAHD883-05	GU161671
7813	Myscelus amystis	Pyrginae	04-SRNP-21290	MHAHC121-05	DQ292645
7814	Myscelus amystis	Pyrginae	00-SRNP-6023	CSRII526-04	DQ292643
7815	Myscelus amystis	Pyrginae	00-SRNP-20049	CSRII527-04	DQ292644
7816	Myscelus amystis	Pyrginae	05-SRNP-20426	MHAHF143-06	GU150556
7817	Myscelus amystis	Pyrginae	05-SRNP-20706	MHAHF145-06	GU150551
7818	Myscelus amystis	Pyrginae	04-SRNP-26566	MHAHL033-07	JF762360
7819	Myscelus amystis	Pyrginae	05-SRNP-20582	MHAHL034-07	JF762361
7820	Passova gellias	Pyrginae	06-SRNP-47761	MHAHK211-07	JF760986
7821	Passova gellias	Pyrginae	08-SRNP-5050	MHMXX663-09	JF778305
7822	Passova gellias	Pyrginae	98-SRNP-2698	CSCR175-04	DQ292835
7823	Passova gellias	Pyrginae	02-SRNP-33556	MHAHK591-07	JF760994
7824	Passova gellias	Pyrginae	03-SRNP-4222	MHAHK589-07	JF760992
7825	Passova gellias	Pyrginae	01-SRNP-3818	MHAHK585-07	JF760988
7826	Passova gellias	Pyrginae	08-SRNP-5586	MHMXX664-09	JF778306
7827	Passova gellias	Pyrginae	08-SRNP-5267	MHMXX665-09	JF778307
7828	Passova gellias	Pyrginae	07-SRNP-23493	MHMXR809-08	JF762541
7829	Passova gellias	Pyrginae	07-SRNP-65612	MHMXR814-08	JF762540
7830	Passova gellias	Pyrginae	07-SRNP-2695	MHMXO769-08	JF762543
7831	Passova gellias	Pyrginae	07-SRNP-65778	MHMXR808-08	JF762542
7832	Passova gellias	Pyrginae	02-SRNP-33544	MHAHK594-07	JF760997
7833	Passova gellias	Pyrginae	02-SRNP-18208	MHAHK593-07	JF760996
7834	Passova gellias	Pyrginae	03-SRNP-25876	MHAHK590-07	JF760993
7835	Passova gellias	Pyrginae	03-SRNP-34742	MHAHK588-07	JF760991
7836	Passova gellias	Pyrginae	02-SRNP-18690	MHAHK587-07	JF760990
7837	Passova gellias	Pyrginae	02-SRNP-18842	MHAHK586-07	JF760989
7838	Passova gellias	Pyrginae	04-SRNP-35968	MHAHF150-06	GU150671
7839	Passova gellias	Pyrginae	04-SRNP-33962	MHAHD817-05	GU161778

Tree Order	Species	Subfamily	ACG Sampleid	BOLD Processid	Genbank Accession
7840	Passova gellias	Pyrginae	04-SRNP-46731	MHAHC177-05	DQ292838
7841	Passova gellias	Pyrginae	04-SRNP-2750	MHAHC169-05	DQ292837
7842	Passova gellias	Pyrginae	02-SRNP-18968	MHAHK592-07	JF760995
7843	Passova gellias	Pyrginae	07-SRNP-501	MHMXK145-07	JF762544
7844	Passova gellias	Pyrginae	02-SRNP-903	MHAHK584-07	JF760987
7845	Passova gellias	Pyrginae	04-SRNP-4530	MHAHD816-05	GU161777
7846	Passova gellias	Pyrginae	02-SRNP-18863	CSCR176-04	DQ292836
7847	Passova gellias	Pyrginae	08-SRNP-348	MHMXT085-08	JF762539
7848	Passova gellias	Pyrginae	09-SRNP-881	MHMYC456-09	GU649880
7849	Passova gellias	Pyrginae	08-SRNP-5268	MHMXX666-09	JF778308
7850	Passova gellias	Pyrginae	09-SRNP-57397	MHMYH113-10	HM887265
7851	Myscelus assaricus michaeli	Pyrginae	04-SRNP-1946	MHAHC097-05	DQ292648
7852	Myscelus assaricus michaeli	Pyrginae	02-SRNP-33561	MHAHK598-07	JF760881
7853	Myscelus assaricus michaeli	Pyrginae	02-SRNP-33559	MHAHK597-07	JF760880
7854	Myscelus assaricus michaeli	Pyrginae	02-SRNP-33574	MHAHK595-07	JF760878
7855	Myscelus assaricus michaeli	Pyrginae	04-SRNP-2100	MHAHC113-05	DQ292650
7856	Myscelus assaricus michaeli	Pyrginae	04-SRNP-2099	MHAHC105-05	DQ292649
7857	Myscelus assaricus michaeli	Pyrginae	03-SRNP-6544	CSCR375-04	DQ292647
7858	Myscelus assaricus michaeli	Pyrginae	03-SRNP-6120	CSCR374-04	DQ292646
7859	Myscelus assaricus michaeli	Pyrginae	02-SRNP-33572	MHAHK596-07	JF760879
7860	Myscelus assaricus michaeli	Pyrginae	02-SRNP-33568	MHAHK599-07	JF760882
7861	Myscelus assaricus michaeli	Pyrginae	03-SRNP-5954	MHAHK600-07	JF760883
7862	Myscelus assaricus michaeli	Pyrginae	08-SRNP-1740	MHMXS085-08	JF762362
7863	Myscelus assaricus michaeli	Pyrginae	08-SRNP-65643	MHMXX660-09	JF778092
7864	Myscelus perissodora	Pyrginae	03-SRNP-6256	CSCR378-04	DQ292657
7865	Myscelus perissodora	Pyrginae	99-SRNP-2587	CSCR138-04	DQ292656
7866	Myscelus belti	Pyrginae	07-SRNP-66198	MHMXT175-08	JF762365
7867	Myscelus belti	Pyrginae	07-SRNP-1252	MHAHL118-07	JF762364
7868	Myscelus belti	Pyrginae	07-SRNP-3251	MHMXP148-08	JF762372
7869	Myscelus belti	Pyrginae	08-SRNP-1651	MHMXX960-09	JF778093
7870	Myscelus belti	Pyrginae	08-SRNP-72474	MHMXY1134-09	GU666405
7871	Myscelus belti	Pyrginae	07-SRNP-3252	MHMXP149-08	JF762371
7872	Myscelus belti	Pyrginae	07-SRNP-36505	MHMXR752-08	JF762369
7873	Myscelus belti	Pyrginae	09-SRNP-20103	MHMXY1135-09	GU666409
7874	Myscelus belti	Pyrginae	09-SRNP-20720	MHMYG2460-10	HM885886
7875	Myscelus belti	Pyrginae	09-SRNP-4463	MHMYG2461-10	HM885888
7876	Myscelus belti	Pyrginae	09-SRNP-4464	MHMYG2462-10	HM885889
7877	Myscelus belti	Pyrginae	07-SRNP-32809	MHAHL117-07	JF762363
7878	Myscelus belti	Pyrginae	05-SRNP-2091	MHAHF153-06	GU150559
7879	Myscelus belti	Pyrginae	04-SRNP-34960	MHAHE346-05	GU149747
7880	Myscelus belti	Pyrginae	04-SRNP-3418	MHAHE345-05	GU149748
7881	Myscelus belti	Pyrginae	04-SRNP-23919	MHAHE344-05	GU149749
7882	Myscelus belti	Pyrginae	04-SRNP-23918	MHAHE343-05	GU149750
7883	Myscelus belti	Pyrginae	04-SRNP-31804	MHAHE342-05	GU149745
7884	Myscelus belti	Pyrginae	04-SRNP-33998	MHAHE339-05	GU149746
7885	Myscelus belti	Pyrginae	04-SRNP-35333	MHAHC186-05	DQ292655
7886	Myscelus belti	Pyrginae	04-SRNP-35100	MHAHC178-05	DQ292654
7887	Myscelus belti	Pyrginae	04-SRNP-35393	MHAHC170-05	DQ292653
7888	Myscelus belti	Pyrginae	07-SRNP-65976	MHMXT172-08	JF762368
7889	Myscelus belti	Pyrginae	07-SRNP-3391	MHMXP150-08	JF762370
7890	Myscelus belti	Pyrginae	03-SRNP-6307	CSCR376-04	DQ292651
7891	Myscelus belti	Pyrginae	07-SRNP-65974	MHMXT173-08	JF762367
7892	Myscelus belti	Pyrginae	03-SRNP-3151	CSCR377-04	DQ292652
7893	Myscelus belti	Pyrginae	07-SRNP-66043	MHMXT174-08	JF762366
7894	Myscelus belti	Pyrginae	09-SRNP-69008	MHMYC455-09	GU649879
7895	Myscelus belti	Pyrginae	09-SRNP-20102	MHMYG2500-10	HM885928

Tree Order	Species	Subfamily	ACG Sampleid	BOLD Processid	Genbank Accession
7896	Oxynetra hopfferi	Pyrginae	02-SRNP-23286	CSCR169-04	DQ292811
7897	Oxynetra hopfferi	Pyrginae	02-SRNP-23284	CSCR168-04	DQ292810
7898	Oxynetra hopfferi	Pyrginae	01-SRNP-6995	CSRII747-05	DQ292818
7899	Oxynetra hopfferi	Pyrginae	02-SRNP-23283	CSRII714-05	DQ292815
7900	Oxynetra hopfferi	Pyrginae	02-SRNP-23540	CSRII715-05	DQ292816
7901	Oxynetra hopfferi	Pyrginae	02-SRNP-24529	CSRII716-05	DQ292817
7902	Oxynetra hopfferi	Pyrginae	02-SRNP-23285	CSRII697-05	DQ292812
7903	Oxynetra hopfferi	Pyrginae	02-SRNP-23109	CSRII705-05	DQ292813
7904	Oxynetra hopfferi	Pyrginae	02-SRNP-23110	CSRII713-05	DQ292814
7905	Oxynetra hopfferi	Pyrginae	03-SRNP-3638	MHAHK543-07	JF760954
7906	Bungalotis erythus	Pyrginae	08-SRNP-65506	MHMXW127-09	JF753731
7907	Bungalotis erythus	Pyrginae	08-SRNP-70954	MHMXW125-09	JF753729
7908	Bungalotis erythus	Pyrginae	08-SRNP-21006	MHMXW119-09	JF753728
7909	Bungalotis erythus	Pyrginae	08-SRNP-20672	MHMXW118-09	JF753727
7910	Bungalotis erythus	Pyrginae	07-SRNP-46538	MHMXT225-08	JF761704
7911	Bungalotis erythus	Pyrginae	07-SRNP-65250	MHMXR756-08	JF761705
7912	Bungalotis erythus	Pyrginae	07-SRNP-41269	MHAHL532-07	JF761703
7913	Bungalotis erythus	Pyrginae	07-SRNP-21991	MHAHL531-07	JF761702
7914	Bungalotis erythus	Pyrginae	07-SRNP-940	MHAHL530-07	JF761701
7915	Bungalotis erythus	Pyrginae	05-SRNP-33392	MHAHI684-06	GU155846
7916	Bungalotis erythus	Pyrginae	06-SRNP-20583	MHAHH437-06	GU155207
7917	Bungalotis erythus	Pyrginae	05-SRNP-45216	MHAHF562-06	GU150269
7918	Bungalotis erythus	Pyrginae	05-SRNP-47613	MHAHF561-06	GU150268
7919	Bungalotis erythus	Pyrginae	04-SRNP-55857	MHAHF137-06	GU150270
7920	Bungalotis erythus	Pyrginae	04-SRNP-22883	MHAHE306-05	GU149448
7921	Bungalotis erythus	Pyrginae	04-SRNP-61431	MHAHE305-05	GU149446
7922	Bungalotis erythus	Pyrginae	04-SRNP-3609	MHAHE304-05	GU149444
7923	Bungalotis erythus	Pyrginae	05-SRNP-20556	MHAHE303-05	GU149445
7924	Bungalotis erythus	Pyrginae	04-SRNP-22815	MHAHE302-05	GU149449
7925	Bungalotis erythus	Pyrginae	04-SRNP-4963	MHAHE301-05	GU149447
7926	Bungalotis erythus	Pyrginae	07-SRNP-20903	MHAHL528-07	JF761699
7927	Bungalotis erythus	Pyrginae	01-SRNP-24401	CSCR046-04	DQ291913
7928	Bungalotis erythus	Pyrginae	07-SRNP-941	MHAHL529-07	JF761700
7929	Bungalotis erythus	Pyrginae	97-SRNP-6142	CSCR045-04	DQ291912
7930	Bungalotis erythus	Pyrginae	08-SRNP-70955	MHMXW126-09	JF753730
7931	Bungalotis erythus	Pyrginae	08-SRNP-65224	MHMXW135-09	JF753732
7932	Bungalotis erythus	Pyrginae	07-SRNP-36879	MHMXW136-09	JF753733
7933	Bungalotis erythus	Pyrginae	08-SRNP-200	MHMXW138-09	JF753734
7934	Bungalotis erythus	Pyrginae	08-SRNP-65769	MHMXY1139-09	GU666407
7935	Bungalotis quadratumDHJ02	Pyrginae	05-SRNP-2515	MHAHF369-06	GU150278
7936	Bungalotis quadratumDHJ02	Pyrginae	07-SRNP-66034	MHMXT234-08	JF761728
7937	Bungalotis quadratumDHJ02	Pyrginae	05-SRNP-23625	MHAHI686-06	GU155850
7938	Bungalotis quadratumDHJ02	Pyrginae	05-SRNP-3116	MHAHF396-06	GU150288
7939	Bungalotis quadratumDHJ02	Pyrginae	07-SRNP-4096	MHMXT132-08	JF761730
7940	Bungalotis quadratumDHJ02	Pyrginae	07-SRNP-66035	MHMXT131-08	JF761731
7941	Bungalotis quadratumDHJ02	Pyrginae	06-SRNP-31687	MHAHH531-06	GU155211
7942	Bungalotis quadratumDHJ02	Pyrginae	03-SRNP-1484	CSCR321-04	DQ291921
7943	Bungalotis quadratumDHJ02	Pyrginae	02-SRNP-33238	XAA804-04	DQ291940
7944	Bungalotis quadratumDHJ02	Pyrginae	02-SRNP-14657	XAA756-04	DQ291924
7945	Bungalotis quadratumDHJ02	Pyrginae	02-SRNP-20465	XAA771-04	DQ291929
7946	Bungalotis quadratumDHJ02	Pyrginae	05-SRNP-21979	MHAHF389-06	GU150291
7947	Bungalotis quadratumDHJ02	Pyrginae	06-SRNP-517	MHAHG691-06	GU151221
7948	Bungalotis quadratumDHJ02	Pyrginae	08-SRNP-65212	MHMXW050-09	JF753772
7949	Bungalotis quadratumDHJ02	Pyrginae	08-SRNP-65420	MHMXW098-09	JF753774
7950	Bungalotis quadratumDHJ02	Pyrginae	05-SRNP-41328	MHAHF385-06	GU150285
7951	Bungalotis quadratumDHJ02	Pyrginae	05-SRNP-3115	MHAHF393-06	GU150292

Tree Order	Species	Subfamily	ACG Sampleid	BOLD Processid	Genbank Accession
7952	Bungalotis quadratumDHJ02	Pyrginae	04-SRNP-10345	MHAHE331-05	GU149456
7953	Bungalotis quadratumDHJ02	Pyrginae	05-SRNP-21480	MHAHF378-06	GU150279
7954	Bungalotis quadratumDHJ02	Pyrginae	05-SRNP-41367	MHAHF387-06	GU150289
7955	Bungalotis quadratumDHJ02	Pyrginae	05-SRNP-40975	MHAHF391-06	GU150295
7956	Bungalotis quadratumDHJ02	Pyrginae	06-SRNP-3854	MHAHI139-06	GU155847
7957	Bungalotis quadratumDHJ02	Pyrginae	08-SRNP-65367	MHMXW045-09	JF753770
7958	Bungalotis quadratumDHJ02	Pyrginae	08-SRNP-65296	MHMXW058-09	JF753773
7959	Bungalotis quadratumDHJ02	Pyrginae	08-SRNP-2181	MHMXW105-09	JF753775
7960	Bungalotis quadratumDHJ02	Pyrginae	05-SRNP-21366	MHAHF394-06	GU150275
7961	Bungalotis quadratumDHJ02	Pyrginae	07-SRNP-42298	MHMXO896-08	JF761733
7962	Bungalotis quadratumDHJ02	Pyrginae	07-SRNP-2736	MHAHL543-07	JF761727
7963	Bungalotis quadratumDHJ02	Pyrginae	05-SRNP-1726	MHAHF395-06	GU150276
7964	Bungalotis quadratumDHJ02	Pyrginae	05-SRNP-12052	MHAHF392-06	GU150294
7965	Bungalotis quadratumDHJ02	Pyrginae	04-SRNP-21541	MHAHE333-05	GU149455
7966	Bungalotis quadratumDHJ02	Pyrginae	05-SRNP-8122	MHAHG689-06	GU151215
7967	Bungalotis quadratumDHJ02	Pyrginae	04-SRNP-20799	MHAHE328-05	GU149457
7968	Bungalotis quadratumDHJ02	Pyrginae	05-SRNP-64394	MHAHG690-06	GU151220
7969	Bungalotis quadratumDHJ02	Pyrginae	07-SRNP-2182	MHMXO898-08	JF761732
7970	Bungalotis quadratumDHJ02	Pyrginae	07-SRNP-65520	MHMXT133-08	JF761729
7971	Bungalotis quadratumDHJ02	Pyrginae	08-SRNP-65306	MHMXW044-09	JF753769
7972	Bungalotis quadratumDHJ02	Pyrginae	08-SRNP-318	MHMXW047-09	JF753771
7973	Bungalotis quadratumDHJ02	Pyrginae	08-SRNP-21949	MHMXY1137-09	GU666411
7974	Bungalotis quadratumDHJ02	Pyrginae	08-SRNP-21946	MHMXY1138-09	GU666406
7975	Bungalotis quadratumDHJ01	Pyrginae	07-SRNP-20456	MHMXR750-08	JF761723
7976	Bungalotis quadratumDHJ01	Pyrginae	07-SRNP-65502	MHMXR751-08	JF761722
7977	Bungalotis quadratumDHJ01	Pyrginae	08-SRNP-1902	MHMXW040-09	JF753735
7978	Bungalotis quadratumDHJ01	Pyrginae	08-SRNP-55655	MHMXW041-09	JF753736
7979	Bungalotis quadratumDHJ01	Pyrginae	08-SRNP-65339	MHMXW042-09	JF753737
7980	Bungalotis quadratumDHJ01	Pyrginae	08-SRNP-20633	MHMXW051-09	JF753742
7981	Bungalotis quadratumDHJ01	Pyrginae	08-SRNP-20846	MHMXW052-09	JF753743
7982	Bungalotis quadratumDHJ01	Pyrginae	08-SRNP-65211	MHMXW094-09	JF753752
7983	Bungalotis quadratumDHJ01	Pyrginae	07-SRNP-42880	MHMXW095-09	JF753753
7984	Bungalotis quadratumDHJ01	Pyrginae	08-SRNP-65342	MHMXW101-09	JF753758
7985	Bungalotis quadratumDHJ01	Pyrginae	08-SRNP-2564	MHMXW103-09	JF753760
7986	Bungalotis quadratumDHJ01	Pyrginae	07-SRNP-1559	MHAHL544-07	JF761713
7987	Bungalotis quadratumDHJ01	Pyrginae	07-SRNP-1560	MHAHL541-07	JF761711
7988	Bungalotis quadratumDHJ01	Pyrginae	07-SRNP-1561	MHAHL526-07	JF761708
7989	Bungalotis quadratumDHJ01	Pyrginae	06-SRNP-5401	MHAHI138-06	GU155848
7990	Bungalotis quadratumDHJ01	Pyrginae	05-SRNP-2294	MHAHF388-06	GU150287
7991	Bungalotis quadratumDHJ01	Pyrginae	05-SRNP-2447	MHAHF386-06	GU150274
7992	Bungalotis quadratumDHJ01	Pyrginae	05-SRNP-21145	MHAHF384-06	GU150293
7993	Bungalotis quadratumDHJ01	Pyrginae	05-SRNP-12053	MHAHF381-06	GU150286
7994	Bungalotis quadratumDHJ01	Pyrginae	05-SRNP-2293	MHAHF380-06	GU150272
7995	Bungalotis quadratumDHJ01	Pyrginae	05-SRNP-21739	MHAHF375-06	GU150280
7996	Bungalotis quadratumDHJ01	Pyrginae	04-SRNP-32299	MHAHE334-05	GU149452
7997	Bungalotis quadratumDHJ01	Pyrginae	04-SRNP-20807	MHAHE329-05	GU149458
7998	Bungalotis quadratumDHJ01	Pyrginae	04-SRNP-21611	MHAHE327-05	GU149451
7999	Bungalotis quadratumDHJ01	Pyrginae	07-SRNP-1876	MHAHL540-07	JF761710
8000	Bungalotis quadratumDHJ01	Pyrginae	03-SRNP-1652	CSCR320-04	DQ291920
8001	Bungalotis quadratumDHJ01	Pyrginae	07-SRNP-21698	MHMXT135-08	JF761720
8002	Bungalotis quadratumDHJ01	Pyrginae	05-SRNP-3021	MHAHF376-06	GU150281
8003	Bungalotis quadratumDHJ01	Pyrginae	02-SRNP-1284	XAA803-04	DQ291939
8004	Bungalotis quadratumDHJ01	Pyrginae	02-SRNP-20466	XAA763-04	DQ291926
8005	Bungalotis quadratumDHJ01	Pyrginae	02-SRNP-31454	XAA820-04	DQ291944
8006	Bungalotis quadratumDHJ01	Pyrginae	03-SRNP-1253	XAA796-04	DQ291938
8007	Bungalotis quadratumDHJ01	Pyrginae	02-SRNP-14135	XAA780-04	DQ291933

Tree Order	Species	Subfamily	ACG Sampleid	BOLD Processid	Genbank Accession
8008	Bungalotis quadratumDHJ01	Pyrginae	02-SRNP-4554	XAA819-04	DQ291943
8009	Bungalotis quadratumDHJ01	Pyrginae	02-SRNP-2667	XAA837-04	DQ291949
8010	Bungalotis quadratumDHJ01	Pyrginae	02-SRNP-1283	XAA755-04	DQ291923
8011	Bungalotis quadratumDHJ01	Pyrginae	02-SRNP-33827	XAA779-04	DQ291932
8012	Bungalotis quadratumDHJ01	Pyrginae	02-SRNP-2075	XAA787-04	DQ291935
8013	Bungalotis quadratumDHJ01	Pyrginae	02-SRNP-4552	XAA835-04	DQ291947
8014	Bungalotis quadratumDHJ01	Pyrginae	07-SRNP-46004	MHMXT231-08	JF761716
8015	Bungalotis quadratumDHJ01	Pyrginae	08-SRNP-65328	MHMXW108-09	JF753764
8016	Bungalotis quadratumDHJ01	Pyrginae	08-SRNP-40971	MHMXX681-09	JF777712
8017	Bungalotis quadratumDHJ01	Pyrginae	08-SRNP-65688	MHMXY1136-09	GU666410
8018	Bungalotis quadratumDHJ01	Pyrginae	09-SRNP-20206	MHMYG2454-10	HM885880
8019	Bungalotis quadratumDHJ01	Pyrginae	05-SRNP-12177	MHAHF390-06	GU150296
8020	Bungalotis quadratumDHJ01	Pyrginae	05-SRNP-3054	MHAHF371-06	GU150297
8021	Bungalotis quadratumDHJ01	Pyrginae	05-SRNP-30602	MHAHF372-06	GU150282
8022	Bungalotis quadratumDHJ01	Pyrginae	05-SRNP-3055	MHAHF370-06	GU150298
8023	Bungalotis quadratumDHJ01	Pyrginae	07-SRNP-2743	MHMXO900-08	JF761724
8024	Bungalotis quadratumDHJ01	Pyrginae	02-SRNP-6982	XAA827-04	DQ291945
8025	Bungalotis quadratumDHJ01	Pyrginae	05-SRNP-3484	MHAHF377-06	GU150277
8026	Bungalotis quadratumDHJ01	Pyrginae	07-SRNP-5038	MHMXT136-08	JF761719
8027	Bungalotis quadratumDHJ01	Pyrginae	05-SRNP-3088	MHAHF368-06	GU150299
8028	Bungalotis quadratumDHJ01	Pyrginae	08-SRNP-40281	MHMXW111-09	JF753767
8029	Bungalotis quadratumDHJ01	Pyrginae	08-SRNP-40180	MHMXW110-09	JF753766
8030	Bungalotis quadratumDHJ01	Pyrginae	08-SRNP-586	MHMXW109-09	JF753765
8031	Bungalotis quadratumDHJ01	Pyrginae	08-SRNP-65341	MHMXW107-09	JF753763
8032	Bungalotis quadratumDHJ01	Pyrginae	08-SRNP-65340	MHMXW106-09	JF753762
8033	Bungalotis quadratumDHJ01	Pyrginae	07-SRNP-12438	MHMXW104-09	JF753761
8034	Bungalotis quadratumDHJ01	Pyrginae	08-SRNP-65454	MHMXW102-09	JF753759
8035	Bungalotis quadratumDHJ01	Pyrginae	08-SRNP-65515	MHMXW100-09	JF753757
8036	Bungalotis quadratumDHJ01	Pyrginae	08-SRNP-65368	MHMXW099-09	JF753756
8037	Bungalotis quadratumDHJ01	Pyrginae	08-SRNP-65459	MHMXW097-09	JF753755
8038	Bungalotis quadratumDHJ01	Pyrginae	08-SRNP-65164	MHMXW093-09	JF753751
8039	Bungalotis quadratumDHJ01	Pyrginae	08-SRNP-267	MHMXW092-09	JF753750
8040	Bungalotis quadratumDHJ01	Pyrginae	07-SRNP-4896	MHMXW091-09	JF753749
8041	Bungalotis quadratumDHJ01	Pyrginae	08-SRNP-65276	MHMXW057-09	JF753748
8042	Bungalotis quadratumDHJ01	Pyrginae	08-SRNP-585	MHMXW056-09	JF753747
8043	Bungalotis quadratumDHJ01	Pyrginae	08-SRNP-65305	MHMXW055-09	JF753746
8044	Bungalotis quadratumDHJ01	Pyrginae	08-SRNP-21065	MHMXW054-09	JF753745
8045	Bungalotis quadratumDHJ01	Pyrginae	08-SRNP-1750	MHMXW053-09	JF753744
8046	Bungalotis quadratumDHJ01	Pyrginae	08-SRNP-45152	MHMXW049-09	JF753741
8047	Bungalotis quadratumDHJ01	Pyrginae	08-SRNP-296	MHMXW048-09	JF753740
8048	Bungalotis quadratumDHJ01	Pyrginae	08-SRNP-2182	MHMXW046-09	JF753739
8049	Bungalotis quadratumDHJ01	Pyrginae	08-SRNP-65327	MHMXW043-09	JF753738
8050	Bungalotis quadratumDHJ01	Pyrginae	08-SRNP-164	MHMXT233-08	JF761714
8051	Bungalotis quadratumDHJ01	Pyrginae	07-SRNP-3504	MHMXT232-08	JF761715
8052	Bungalotis quadratumDHJ01	Pyrginae	08-SRNP-40213	MHMXT134-08	JF761721
8053	Bungalotis quadratumDHJ01	Pyrginae	07-SRNP-21331	MHMXO895-08	JF761725
8054	Bungalotis quadratumDHJ01	Pyrginae	07-SRNP-3488	MHMXO894-08	JF761726
8055	Bungalotis quadratumDHJ01	Pyrginae	07-SRNP-566	MHAHL542-07	JF761712
8056	Bungalotis quadratumDHJ01	Pyrginae	07-SRNP-21349	MHAHL525-07	JF761707
8057	Bungalotis quadratumDHJ01	Pyrginae	05-SRNP-22370	MHAHI679-06	GU155849
8058	Bungalotis quadratumDHJ01	Pyrginae	06-SRNP-41272	MHAHH438-06	GU155209
8059	Bungalotis quadratumDHJ01	Pyrginae	06-SRNP-20160	MHAHH436-06	GU155210
8060	Bungalotis quadratumDHJ01	Pyrginae	06-SRNP-1694	MHAHG692-06	GU151222
8061	Bungalotis quadratumDHJ01	Pyrginae	06-SRNP-2564	MHAHG688-06	GU151218
8062	Bungalotis quadratumDHJ01	Pyrginae	06-SRNP-1945	MHAHG687-06	GU151217
8063	Bungalotis quadratumDHJ01	Pyrginae	06-SRNP-1686	MHAHG686-06	GU151219

Tree Order	Species	Subfamily	ACG Sampleid	BOLD Processid	Genbank Accession
8064	Bungalotis quadratumDHJ01	Pyrginae	06-SRNP-260	MHAHG685-06	GU151216
8065	Bungalotis quadratumDHJ01	Pyrginae	05-SRNP-4567	MHAHF383-06	GU150290
8066	Bungalotis quadratumDHJ01	Pyrginae	05-SRNP-999	MHAHF382-06	GU150273
8067	Bungalotis quadratumDHJ01	Pyrginae	05-SRNP-3053	MHAHF379-06	GU150284
8068	Bungalotis quadratumDHJ01	Pyrginae	04-SRNP-48823	MHAHE332-05	GU149454
8069	Bungalotis quadratumDHJ01	Pyrginae	05-SRNP-55167	MHAHE330-05	GU149453
8070	Bungalotis quadratumDHJ01	Pyrginae	07-SRNP-4148	MHMXT137-08	JF761718
8071	Bungalotis quadratumDHJ01	Pyrginae	05-SRNP-20357	MHAHF373-06	GU150283
8072	Bungalotis quadratumDHJ01	Pyrginae	07-SRNP-4097	MHMXT230-08	JF761717
8073	Bungalotis quadratumDHJ01	Pyrginae	07-SRNP-1859	MHAHL539-07	JF761709
8074	Bungalotis quadratumDHJ01	Pyrginae	02-SRNP-3261	XAA836-04	DQ291948
8075	Bungalotis quadratumDHJ01	Pyrginae	02-SRNP-14043	XAA811-04	DQ291941
8076	Bungalotis quadratumDHJ01	Pyrginae	02-SRNP-3643	XAA795-04	DQ291937
8077	Bungalotis quadratumDHJ01	Pyrginae	02-SRNP-2979	XAA844-04	DQ291950
8078	Bungalotis quadratumDHJ01	Pyrginae	02-SRNP-3867	XAA828-04	DQ291946
8079	Bungalotis quadratumDHJ01	Pyrginae	02-SRNP-18838	XAA812-04	DQ291942
8080	Bungalotis quadratumDHJ01	Pyrginae	02-SRNP-2994	XAA772-04	DQ291930
8081	Bungalotis quadratumDHJ01	Pyrginae	02-SRNP-14137	XAA764-04	DQ291927
8082	Bungalotis quadratumDHJ01	Pyrginae	02-SRNP-14083	XAA845-04	DQ291951
8083	Bungalotis quadratumDHJ01	Pyrginae	01-SRNP-628	XAA762-04	DQ291925
8084	Bungalotis quadratumDHJ01	Pyrginae	02-SRNP-5698	XAA788-04	DQ291936
8085	Bungalotis quadratumDHJ01	Pyrginae	02-SRNP-2310	XAA754-04	DQ291922
8086	Bungalotis quadratumDHJ01	Pyrginae	02-SRNP-14094	XAA770-04	DQ291928
8087	Bungalotis quadratumDHJ01	Pyrginae	02-SRNP-14599	XAA778-04	DQ291931
8088	Bungalotis quadratumDHJ01	Pyrginae	02-SRNP-5850	XAA786-04	DQ291934
8089	Bungalotis quadratumDHJ01	Pyrginae	08-SRNP-959	MHMXW096-09	JF753754
8090	Bungalotis quadratumDHJ01	Pyrginae	08-SRNP-40212	MHMXW139-09	JF753768
8091	Bungalotis quadratumDHJ01	Pyrginae	09-SRNP-20809	MHMYG2455-10	HM885881
8092	Bungalotis astylos	Pyrginae	04-SRNP-23054	MHAHD882-05	GU161299
8093	Bungalotis astylos	Pyrginae	05-SRNP-3123	MHAHF563-06	GU150265
8094	Bungalotis astylos	Pyrginae	07-SRNP-32268	MHMXK072-07	JF761692
8095	Bungalotis astylos	Pyrginae	06-SRNP-55070	MHAHG140-06	GU151213
8096	Bungalotis astylos	Pyrginae	04-SRNP-48647	MHAHD881-05	GU161298
8097	Bungalotis astylos	Pyrginae	07-SRNP-31525	MHAHL523-07	JF761684
8098	Bungalotis astylos	Pyrginae	07-SRNP-40972	MHAHL524-07	JF761685
8099	Bungalotis astylos	Pyrginae	07-SRNP-2056	MHAHL538-07	JF761686
8100	Bungalotis astylos	Pyrginae	07-SRNP-42318	MHMXO897-08	JF761691
8101	Bungalotis astylos	Pyrginae	07-SRNP-33568	MHMXT235-08	JF761687
8102	Bungalotis astylos	Pyrginae	06-SRNP-57444	MHAHJ907-07	JF752490
8103	Bungalotis astylos	Pyrginae	06-SRNP-59176	MHAHK191-07	JF760457
8104	Bungalotis astylos	Pyrginae	06-SRNP-43639	MHAHJ908-07	JF752491
8105	Bungalotis astylos	Pyrginae	06-SRNP-33017	MHAHI509-06	GU155843
8106	Bungalotis astylos	Pyrginae	07-SRNP-57567	MHMXO902-08	JF761688
8107	Bungalotis astylos	Pyrginae	07-SRNP-57566	MHMXO901-08	JF761689
8108	Bungalotis astylos	Pyrginae	05-SRNP-3124	MHAHF541-06	GU150264
8109	Bungalotis astylos	Pyrginae	06-SRNP-58700	MHAHK189-07	JF760455
8110	Bungalotis astylos	Pyrginae	01-SRNP-1961	CSCR044-04	DQ291909
8111	Bungalotis astylos	Pyrginae	07-SRNP-57565	MHMXO899-08	JF761690
8112	Bungalotis astylos	Pyrginae	09-SRNP-31141	MHMYC536-09	GU649804
8113	Bungalotis astylos	Pyrginae	05-SRNP-66563	MHAHG139-06	GU151212
8114	Bungalotis astylos	Pyrginae	06-SRNP-59177	MHAHK190-07	JF760456
8115	Bungalotis astylos	Pyrginae	96-SRNP-11379	CSCR043-04	DQ291908
8116	Bungalotis astylos	Pyrginae	09-SRNP-1890	MHMYE1445-09	GU653552
8117	Bungalotis midas	Pyrginae	03-SRNP-12881.1	CSCR317-04	DQ291916
8118	Bungalotis midas	Pyrginae	06-SRNP-43436	MHAHJ521-07	JF752492
8119	Bungalotis midas	Pyrginae	03-SRNP-9076	CSCR318-04	DQ291917

Tree Order	Species	Subfamily	ACG Sampleid	BOLD Processid	Genbank Accession
8120	Bungalotis midas	Pyrginae	05-SRNP-4908	MHAHF564-06	GU150271
8121	Bungalotis midas	Pyrginae	03-SRNP-15139	CSCR316-04	DQ291915
8122	Bungalotis midas	Pyrginae	94-SRNP-308	CSCR047-04	DQ291914
8123	Bungalotis midas	Pyrginae	03-SRNP-9194	CSCR319-04	DQ291918
8124	Bungalotis midas	Pyrginae	03-SRNP-34185	CSCR482-04	DQ291919
8125	Bungalotis midas	Pyrginae	05-SRNP-4469	MHAHE585-06	GU149450
8126	Bungalotis midas	Pyrginae	06-SRNP-21109	MHAHH435-06	GU155208
8127	Bungalotis midas	Pyrginae	07-SRNP-40390	MHMXK071-07	JF761706
8128	Bungalotis midas	Pyrginae	09-SRNP-44040	MHMYE1446-09	GU653549
8129	Bungalotis midas	Pyrginae	09-SRNP-20274	MHMYG2456-10	HM885882
8130	Bungalotis diophorus	Pyrginae	06-SRNP-1016	MHAHG141-06	GU151214
8131	Bungalotis diophorus	Pyrginae	05-SRNP-24718	MHAHI678-06	GU155844
8132	Bungalotis diophorus	Pyrginae	07-SRNP-3318	MHMXO908-08	JF761697
8133	Bungalotis diophorus	Pyrginae	07-SRNP-3315	MHMXO909-08	JF761696
8134	Bungalotis diophorus	Pyrginae	08-SRNP-70990	MHMXX677-09	JF777708
8135	Bungalotis diophorus	Pyrginae	04-SRNP-34119	MHAHE335-05	GU149443
8136	Bungalotis diophorus	Pyrginae	04-SRNP-56177	MHAHF539-06	GU150266
8137	Bungalotis diophorus	Pyrginae	08-SRNP-70423	MHMXX679-09	JF777710
8138	Bungalotis diophorus	Pyrginae	08-SRNP-65707	MHMXZ002-09	GU665265
8139	Bungalotis diophorus	Pyrginae	03-SRNP-1854	CSCR314-04	DQ291910
8140	Bungalotis diophorus	Pyrginae	03-SRNP-10878	CSCR315-04	DQ291911
8141	Bungalotis diophorus	Pyrginae	04-SRNP-34165	MHAHE319-05	GU149440
8142	Bungalotis diophorus	Pyrginae	04-SRNP-34027	MHAHE336-05	GU149441
8143	Bungalotis diophorus	Pyrginae	04-SRNP-33359	MHAHE337-05	GU149439
8144	Bungalotis diophorus	Pyrginae	04-SRNP-33198	MHAHE338-05	GU149442
8145	Bungalotis diophorus	Pyrginae	05-SRNP-21655	MHAHF538-06	GU150267
8146	Bungalotis diophorus	Pyrginae	05-SRNP-34150	MHAHI687-06	GU155845
8147	Bungalotis diophorus	Pyrginae	07-SRNP-65195	MHAHL545-07	JF761693
8148	Bungalotis diophorus	Pyrginae	07-SRNP-3311	MHMXO890-08	JF761698
8149	Bungalotis diophorus	Pyrginae	07-SRNP-23801	MHMXR748-08	JF761695
8150	Bungalotis diophorus	Pyrginae	07-SRNP-23522	MHMXT226-08	JF761694
8151	Bungalotis diophorus	Pyrginae	08-SRNP-70422	MHMXW124-09	JF753726
8152	Bungalotis diophorus	Pyrginae	08-SRNP-70986	MHMXX678-09	JF777709
8153	Bungalotis diophorus	Pyrginae	08-SRNP-71152	MHMXX680-09	JF777711
8154	Bungalotis diophorus	Pyrginae	09-SRNP-22402	MHMYG2457-10	HM885883
8155	Nascus paulliniae	Pyrginae	08-SRNP-5198	MHMXX673-09	JF778116
8156	Nascus paulliniae	Pyrginae	08-SRNP-5133	MHMXX672-09	JF778115
8157	Nascus paulliniae	Pyrginae	99-SRNP-15218	CSCR145-04	DQ292702
8158	Nascus paulliniae	Pyrginae	04-SRNP-55249	MHAHC629-05	DQ292708
8159	Nascus paulliniae	Pyrginae	06-SRNP-7877	MHAHJ676-07	JF752939
8160	Nascus paulliniae	Pyrginae	05-SRNP-42441	MHAHF205-06	GU150583
8161	Nascus paulliniae	Pyrginae	05-SRNP-5420	MHAHF204-06	GU150584
8162	Nascus paulliniae	Pyrginae	05-SRNP-888	MHAHF190-06	GU150586
8163	Nascus paulliniae	Pyrginae	04-SRNP-14706	MHAHC645-05	DQ292712
8164	Nascus paulliniae	Pyrginae	04-SRNP-34021	MHAHC642-05	DQ292711
8165	Nascus paulliniae	Pyrginae	04-SRNP-34682	MHAHC635-05	DQ292710
8166	Nascus paulliniae	Pyrginae	04-SRNP-14655	MHAHC634-05	DQ292709
8167	Nascus paulliniae	Pyrginae	04-SRNP-55654	MHAHC625-05	DQ292707
8168	Nascus paulliniae	Pyrginae	04-SRNP-55794	MHAHC615-05	DQ292706
8169	Nascus paulliniae	Pyrginae	04-SRNP-55367	MHAHC614-05	DQ292705
8170	Nascus paulliniae	Pyrginae	04-SRNP-55964	MHAHC613-05	DQ292704
8171	Nascus paulliniae	Pyrginae	02-SRNP-6701	CSRII529-04	DQ292703
8172	Nascus paulliniae	Pyrginae	95-SRNP-9964	CSCR144-04	DQ292701
8173	Nascus paulliniae	Pyrginae	05-SRNP-45154	MHAHF189-06	GU150585
8174	Nascus paulliniae	Pyrginae	08-SRNP-481	MHMXX964-09	JF778117
8175	Nascus paulliniae	Pyrginae	04-SRNP-14559	MHMXY1079-09	GU666461

Tree Order	Species	Subfamily	ACG Sampleid	BOLD Processid	Genbank Accession
8176	Nicephellus nicephorus	Eudaminae	02-SRNP-27688	CSCR091-04	DQ292303
8177	Nicephellus nicephorus	Eudaminae	02-SRNP-27689	CSCR092-04	DQ292304
8178	Salatis canalis	Pyrginae	02-SRNP-18982	CSCR233-04	DQ293242
8179	Salatis canalis	Pyrginae	05-SRNP-3157	MHAHF522-06	GU150766
8180	Salatis canalis	Pyrginae	08-SRNP-70373	MHMXW128-09	JF754148
8181	Salatis canalis	Pyrginae	08-SRNP-70839	MHMXW122-09	JF754147
8182	Salatis canalis	Pyrginae	08-SRNP-20448	MHMXW117-09	JF754146
8183	Salatis canalis	Pyrginae	07-SRNP-65776	MHMXT229-08	JF762805
8184	Salatis canalis	Pyrginae	08-SRNP-287	MHMXT221-08	JF762806
8185	Salatis canalis	Pyrginae	06-SRNP-9549	MHMXH842-07	JF761154
8186	Salatis canalis	Pyrginae	06-SRNP-44103	MHAHJ519-07	JF753136
8187	Salatis canalis	Pyrginae	05-SRNP-1100	MHAHF520-06	GU150765
8188	Salatis canalis	Pyrginae	04-SRNP-60727	MHAHE321-05	GU149919
8189	Salatis canalis	Pyrginae	04-SRNP-1838	MHAHE320-05	GU149920
8190	Salatis canalis	Pyrginae	03-SRNP-1802	CSRII220-04	DQ293245
8191	Salatis canalis	Pyrginae	03-SRNP-35001	CSRII222-04	DQ293247
8192	Salatis canalis	Pyrginae	02-SRNP-18867	CSCR232-04	DQ293241
8193	Salatis canalis	Pyrginae	02-SRNP-19694	CSRII219-04	DQ293244
8194	Salatis canalis	Pyrginae	05-SRNP-1017	MHAHF521-06	GU150764
8195	Salatis canalis	Pyrginae	02-SRNP-3899	CSRII218-04	DQ293243
8196	Salatis canalis	Pyrginae	03-SRNP-5164	CSRII221-04	DQ293246
8197	Salatis canalis	Pyrginae	07-SRNP-2774	MHMXO907-08	JF762807
8198	Salatis canalis	Pyrginae	08-SRNP-20447	MHMXW146-09	JF754149
8199	Salatis canalis	Pyrginae	08-SRNP-71642	MHMXX676-09	JF778452
8200	Salatis canalis	Pyrginae	08-SRNP-4733	MHMXY1140-09	GU666408
8201	Salatis canalis	Pyrginae	09-SRNP-22934	MHMYG2036-10	HM885438
8202	Cephise aelius	Pyrginae	06-SRNP-20598	MHAHH560-06	GU155325
8203	Cephise aelius	Pyrginae	06-SRNP-2857	MHAHH570-06	GU155326
8204	Cephise aelius	Pyrginae	06-SRNP-30466	MHAHH559-06	GU155327
8205	Cephise aelius	Pyrginae	06-SRNP-12607	MHAHG665-06	GU151300
8206	Cephise aelius	Pyrginae	06-SRNP-12587	MHAHG664-06	GU151301
8207	Cephise aelius	Pyrginae	06-SRNP-12591	MHAHG661-06	GU151299
8208	Cephise aelius	Pyrginae	05-SRNP-55123	MHAHE239-05	GU149529
8209	Cephise aelius	Pyrginae	06-SRNP-3213	MHAHH569-06	GU155328
8210	Cephise aelius	Pyrginae	03-SRNP-234	CSCR328-04	DQ292087
8211	Cephise aelius	Pyrginae	03-SRNP-652	CSCR327-04	DQ292086
8212	Cephise aelius	Pyrginae	02-SRNP-5365	CSCR467-04	DQ292085
8213	Cephise aelius	Pyrginae	02-SRNP-5356	CSCR466-04	DQ292084
8214	Cephise aelius	Pyrginae	07-SRNP-55696	MHMXK326-07	JF761899
8215	Cephise aelius	Pyrginae	08-SRNP-295	MHMXW473-09	JF753808
8216	Cephise aelius	Pyrginae	07-SRNP-61378	MHMXW474-09	JF753809
8217	Cephise aelius	Pyrginae	08-SRNP-55427	MHMXW557-09	JF753807
8218	Cephise aelius	Pyrginae	09-SRNP-22247	MHMYG2422-10	HM885845
8219	Cephise nuspesezDHJ01	Pyrginae	05-SRNP-30053	MHAHL086-07	JF761911
8220	Cephise nuspesezDHJ01	Pyrginae	07-SRNP-30952	MHMXN300-07	JF761912
8221	Cephise nuspesezDHJ01	Pyrginae	08-SRNP-20453	MHMXW560-09	JF753812
8222	Cephise nuspesezDHJ01	Pyrginae	08-SRNP-20452	MHMXW567-09	JF753813
8223	Cephise nuspesezDHJ01	Pyrginae	09-SRNP-68309	MHMYG2425-10	HM885848
8224	Cephise nuspesezDHJ03	Pyrginae	02-SRNP-663	CSCR470-04	DQ292106
8225	Cephise nuspesezDHJ02	Pyrginae	06-SRNP-59594	MHAHK214-07	JF760537
8226	Cephise nuspesezDHJ02	Pyrginae	05-SRNP-40841	MHAHL088-07	JF761914
8227	Cephise nuspesezDHJ02	Pyrginae	07-SRNP-58078	MHMXO883-08	JF761921
8228	Cephise nuspesezDHJ02	Pyrginae	08-SRNP-2349	MHMXX613-09	JF777763
8229	Cephise nuspesezDHJ02	Pyrginae	04-SRNP-21120	MHAHD923-05	GU161324
8230	Cephise nuspesezDHJ02	Pyrginae	04-SRNP-41085	MHAHD916-05	GU161317
8231	Cephise nuspesezDHJ02	Pyrginae	04-SRNP-56573	MHAHD918-05	GU161323

Tree Order	Species	Subfamily	ACG Sampleid	BOLD Processid	Genbank Accession
8232	Cephise nuspesezDHJ02	Pyrginae	04-SRNP-24552	MHAHD921-05	GU161314
8233	Cephise nuspesezDHJ02	Pyrginae	04-SRNP-42550	MHAHD920-05	GU161325
8234	Cephise nuspesezDHJ02	Pyrginae	04-SRNP-56917	MHAHD917-05	GU161316
8235	Cephise nuspesezDHJ02	Pyrginae	04-SRNP-1731	MHAHD915-05	GU161319
8236	Cephise nuspesezDHJ02	Pyrginae	04-SRNP-56576	MHAHD914-05	GU161313
8237	Cephise nuspesezDHJ02	Pyrginae	04-SRNP-56570	MHAHD903-05	GU161320
8238	Cephise nuspesezDHJ02	Pyrginae	04-SRNP-47334	MHAHD902-05	GU161321
8239	Cephise nuspesezDHJ02	Pyrginae	04-SRNP-61251	MHAHD922-05	GU161326
8240	Cephise nuspesezDHJ02	Pyrginae	04-SRNP-35344	MHAHD924-05	GU161327
8241	Cephise nuspesezDHJ02	Pyrginae	04-SRNP-45451	MHAHD901-05	GU161322
8242	Cephise nuspesezDHJ02	Pyrginae	05-SRNP-40050	MHAHD919-05	GU161315
8243	Cephise nuspesezDHJ02	Pyrginae	06-SRNP-23457	MHMXK043-07	JF761924
8244	Cephise nuspesezDHJ02	Pyrginae	07-SRNP-31118	MHMXN307-07	JF761922
8245	Cephise nuspesezDHJ02	Pyrginae	04-SRNP-1075	CSRII187-04	DQ292119
8246	Cephise nuspesezDHJ02	Pyrginae	04-SRNP-40599	CSRII186-04	DQ292118
8247	Cephise nuspesezDHJ02	Pyrginae	03-SRNP-21962	CSRII185-04	DQ292117
8248	Cephise nuspesezDHJ02	Pyrginae	03-SRNP-21961	CSRII184-04	DQ292116
8249	Cephise nuspesezDHJ02	Pyrginae	03-SRNP-5407	CSCR336-04	DQ292110
8250	Cephise nuspesezDHJ02	Pyrginae	03-SRNP-3473	CSCR333-04	DQ292108
8251	Cephise nuspesezDHJ02	Pyrginae	09-SRNP-20005	MHMYG2389-10	HM885813
8252	Cephise nuspesezDHJ02	Pyrginae	09-SRNP-387	MHMYC553-09	GU649797
8253	Cephise nuspesezDHJ02	Pyrginae	08-SRNP-23259	MHMYB128-09	GU649694
8254	Cephise nuspesezDHJ02	Pyrginae	08-SRNP-23248	MHMXY1082-09	GU666455
8255	Cephise nuspesezDHJ02	Pyrginae	08-SRNP-40706	MHMXW564-09	JF753818
8256	Cephise nuspesezDHJ02	Pyrginae	08-SRNP-35369	MHMXW561-09	JF753817
8257	Cephise nuspesezDHJ02	Pyrginae	08-SRNP-20435	MHMXW559-09	JF753816
8258	Cephise nuspesezDHJ02	Pyrginae	08-SRNP-945	MHMXW558-09	JF753815
8259	Cephise nuspesezDHJ02	Pyrginae	08-SRNP-748	MHMXW556-09	JF753814
8260	Cephise nuspesezDHJ02	Pyrginae	08-SRNP-40861	MHMXW329-09	JF753819
8261	Cephise nuspesezDHJ02	Pyrginae	07-SRNP-58267	MHMXP155-08	JF761920
8262	Cephise nuspesezDHJ02	Pyrginae	04-SRNP-61487	MHAHL090-07	JF761915
8263	Cephise nuspesezDHJ02	Pyrginae	04-SRNP-48306	MHAHD913-05	GU161318
8264	Cephise nuspesezDHJ02	Pyrginae	03-SRNP-5930	CSCR338-04	DQ292112
8265	Cephise nuspesezDHJ02	Pyrginae	08-SRNP-655	MHMXT138-08	JF761919
8266	Cephise nuspesezDHJ02	Pyrginae	02-SRNP-33859	CSCR066-04	DQ292107
8267	Cephise nuspesezDHJ02	Pyrginae	03-SRNP-10602	CSCR340-04	DQ292114
8268	Cephise nuspesezDHJ02	Pyrginae	05-SRNP-43495	MHAHL093-07	JF761916
8269	Cephise nuspesezDHJ02	Pyrginae	07-SRNP-31639	MHMXN304-07	JF761923
8270	Cephise nuspesezDHJ02	Pyrginae	00-SRNP-110	CSRII183-04	DQ292115
8271	Cephise nuspesezDHJ02	Pyrginae	03-SRNP-5879	CSCR335-04	DQ292109
8272	Cephise nuspesezDHJ02	Pyrginae	04-SRNP-61513	MHAHL087-07	JF761913
8273	Cephise nuspesezDHJ02	Pyrginae	03-SRNP-5406	CSCR339-04	DQ292113
8274	Cephise nuspesezDHJ02	Pyrginae	02-SRNP-5636	CSCR469-04	DQ292105
8275	Cephise nuspesezDHJ02	Pyrginae	03-SRNP-5475	CSCR337-04	DQ292111
8276	Cephise nuspesezDHJ02	Pyrginae	07-SRNP-66199	MHMXT181-08	JF761917
8277	Cephise nuspesezDHJ02	Pyrginae	08-SRNP-531	MHMXT139-08	JF761918
8278	Cephise nuspesezDHJ02	Pyrginae	09-SRNP-20293	MHMYG2426-10	HM885849
8279	Cephise nuspesezDHJ02	Pyrginae	09-SRNP-20294	MHMYG2427-10	HM885850
8280	Cephise Burns01	Pyrginae	03-SRNP-10046	CSCR332-04	DQ292091
8281	Cephise Burns01	Pyrginae	06-SRNP-41883	MHAHI157-06	GU155895
8282	Cephise Burns01	Pyrginae	08-SRNP-31038	MHMXW562-09	JF753810
8283	Cephise Burns01	Pyrginae	09-SRNP-69829	MHMYG2388-10	HM885812
8284	Cephise Burns01	Pyrginae	03-SRNP-10720	CSCR330-04	DQ292089
8285	Cephise Burns01	Pyrginae	03-SRNP-10166	CSCR331-04	DQ292090
8286	Cephise Burns01	Pyrginae	04-SRNP-34910	MHAHC585-05	DQ292101
8287	Cephise Burns01	Pyrginae	04-SRNP-34943	MHAHC586-05	DQ292102

Tree Order	Species	Subfamily	ACG Sampleid	BOLD Processid	Genbank Accession
8288	Cephise Burns01	Pyrginae	08-SRNP-42404	MHMYC523-09	HM390765
8289	Cephise Burns01	Pyrginae	03-SRNP-21880	CSRII181-04	DQ292094
8290	Cephise Burns01	Pyrginae	03-SRNP-10462	CSCR329-04	DQ292088
8291	Cephise Burns01	Pyrginae	08-SRNP-24687	MHMYB127-09	GU649693
8292	Cephise Burns01	Pyrginae	00-SRNP-11741	CSCR495-04	DQ292092
8293	Cephise Burns01	Pyrginae	05-SRNP-24566	MHAHL094-07	JF761903
8294	Cephise Burns01	Pyrginae	04-SRNP-32954	MHAHC583-05	DQ292099
8295	Cephise Burns01	Pyrginae	04-SRNP-41887	MHAHE004-05	GU149530
8296	Cephise Burns01	Pyrginae	06-SRNP-41095	MHAHG678-06	GU151302
8297	Cephise Burns01	Pyrginae	06-SRNP-23379	MHMXK044-07	JF761910
8298	Cephise Burns01	Pyrginae	05-SRNP-33964	MHAHL091-07	JF761901
8299	Cephise Burns01	Pyrginae	09-SRNP-80522	MHMYG2424-10	HM885847
8300	Cephise Burns01	Pyrginae	07-SRNP-41982	MHMXP157-08	JF761904
8301	Cephise Burns01	Pyrginae	03-SRNP-21881	CSRII182-04	DQ292095
8302	Cephise Burns01	Pyrginae	05-SRNP-41674	MHAHL085-07	JF761900
8303	Cephise Burns01	Pyrginae	05-SRNP-23773	MHAHL092-07	JF761902
8304	Cephise Burns01	Pyrginae	06-SRNP-65516	MHMXK045-07	JF761909
8305	Cephise Burns01	Pyrginae	08-SRNP-40693	MHMXW563-09	JF753811
8306	Cephise Burns01	Pyrginae	09-SRNP-40285	MHMYC519-09	GU649821
8307	Cephise Burns01	Pyrginae	09-SRNP-30023	MHMYC522-09	GU649817
8308	Cephise Burns01	Pyrginae	09-SRNP-40454	MHMYE1416-09	HM424365
8309	Cephise Burns01	Pyrginae	09-SRNP-67702	MHMYG2386-10	HM885810
8310	Cephise Burns01	Pyrginae	09-SRNP-67936	MHMYG2387-10	HM885811
8311	Cephise Burns01	Pyrginae	08-SRNP-72778	MHMYC520-09	GU649822
8312	Cephise Burns01	Pyrginae	08-SRNP-42162	MHMYC521-09	GU649816
8313	Cephise Burns01	Pyrginae	09-SRNP-69828	MHMYG2423-10	HM885846
8314	Cephise Burns01	Pyrginae	07-SRNP-32126	MHMXN305-07	JF761905
8315	Cephise Burns01	Pyrginae	07-SRNP-32127	MHMXN302-07	JF761906
8316	Cephise Burns01	Pyrginae	07-SRNP-31871	MHMXK053-07	JF761908
8317	Cephise Burns01	Pyrginae	06-SRNP-41266	MHAHH533-06	GU155329
8318	Cephise Burns01	Pyrginae	04-SRNP-42479	MHAHC588-05	DQ292104
8319	Cephise Burns01	Pyrginae	04-SRNP-32936	MHAHC587-05	DQ292103
8320	Cephise Burns01	Pyrginae	04-SRNP-55099	MHAHC584-05	DQ292100
8321	Cephise Burns01	Pyrginae	04-SRNP-56368	MHAHC582-05	DQ292098
8322	Cephise Burns01	Pyrginae	04-SRNP-32391	MHAHC581-05	DQ292097
8323	Cephise Burns01	Pyrginae	04-SRNP-42353	MHAHC580-05	DQ292096
8324	Cephise Burns01	Pyrginae	06-SRNP-34228	MHAHJ817-07	JF752567
8325	Cephise Burns01	Pyrginae	07-SRNP-32173	MHMXN298-07	JF761907
8326	Cephise Burns01	Pyrginae	01-SRNP-4401	CSRII180-04	DQ292093
8327	Cephise Burns01	Pyrginae	08-SRNP-24688	MHMYB129-09	
8328	Cephise Burns01	Pyrginae	09-SRNP-68422	MHMYI595-10	HQ963910
8329	Dyscophellus phraxanorDHJ01	Pyrginae	00-SRNP-14033	MHAHJ118-07	JF752647
8330	Dyscophellus phraxanorDHJ01	Pyrginae	07-SRNP-32221	MHAHL537-07	JF762121
8331	Dyscophellus phraxanorDHJ01	Pyrginae	02-SRNP-3482	MHAHJ136-07	JF752651
8332	Dyscophellus phraxanorDHJ01	Pyrginae	01-SRNP-5699	MHAHJ127-07	JF752650
8333	Dyscophellus phraxanorDHJ01	Pyrginae	01-SRNP-22949	MHAHJ125-07	JF752649
8334	Dyscophellus phraxanorDHJ01	Pyrginae	02-SRNP-6877	MHAHJ124-07	JF752648
8335	Dyscophellus phraxanorDHJ01	Pyrginae	99-SRNP-13675	MHAHJ114-07	JF752646
8336	Dyscophellus phraxanorDHJ01	Pyrginae	99-SRNP-12738	MHAHJ107-07	JF752644
8337	Dyscophellus phraxanorDHJ01	Pyrginae	99-SRNP-12756	MHAHJ103-07	JF752643
8338	Dyscophellus phraxanorDHJ01	Pyrginae	99-SRNP-12686	MHAHJ101-07	JF752642
8339	Dyscophellus phraxanorDHJ01	Pyrginae	99-SRNP-12371	MHAHJ096-07	JF752641
8340	Dyscophellus phraxanorDHJ01	Pyrginae	99-SRNP-12368	MHAHJ094-07	JF752640
8341	Dyscophellus phraxanorDHJ01	Pyrginae	01-SRNP-25007	MHAHJ088-07	JF752639
8342	Dyscophellus phraxanorDHJ01	Pyrginae	01-SRNP-175	MHAHJ084-07	JF752638
8343	Dyscophellus phraxanorDHJ01	Pyrginae	00-SRNP-14611	MHAHJ079-07	JF752636

Tree Order	Species	Subfamily	ACG Sampleid	BOLD Processid	Genbank Accession
8344	Dyscophellus phraxanorDHJ01	Pyrginae	00-SRNP-14606	MHAHJ078-07	JF752635
8345	Dyscophellus phraxanorDHJ01	Pyrginae	00-SRNP-12745	MHAHJ077-07	JF752634
8346	Dyscophellus phraxanorDHJ01	Pyrginae	99-SRNP-5588	MHAHJ067-07	JF752633
8347	Dyscophellus phraxanorDHJ01	Pyrginae	01-SRNP-64	MHAHJ063-07	JF752632
8348	Dyscophellus phraxanorDHJ01	Pyrginae	99-SRNP-13192	MHAHJ052-07	JF752631
8349	Dyscophellus phraxanorDHJ01	Pyrginae	99-SRNP-13329	MHAHJ046-07	JF752629
8350	Dyscophellus phraxanorDHJ01	Pyrginae	98-SRNP-7943	MHAHJ023-07	JF752626
8351	Dyscophellus phraxanorDHJ01	Pyrginae	02-SRNP-2032	MHAHC525-05	DQ292352
8352	Dyscophellus phraxanorDHJ01	Pyrginae	02-SRNP-982	MHAHC524-05	DQ292351
8353	Dyscophellus phraxanorDHJ01	Pyrginae	02-SRNP-3712	MHAHC519-05	DQ292346
8354	Dyscophellus phraxanorDHJ01	Pyrginae	01-SRNP-3949	MHAHC504-05	DQ292331
8355	Dyscophellus phraxanorDHJ01	Pyrginae	99-SRNP-13337	MHAHC501-05	DQ292328
8356	Dyscophellus phraxanorDHJ01	Pyrginae	00-SRNP-4533	MHAHC495-05	DQ292322
8357	Dyscophellus phraxanorDHJ01	Pyrginae	01-SRNP-9094	MHAHC493-05	DQ292320
8358	Dyscophellus phraxanorDHJ01	Pyrginae	00-SRNP-14920	MHAHJ082-07	JF752637
8359	Dyscophellus phraxanorDHJ01	Pyrginae	99-SRNP-4770	MHAHJ028-07	JF752627
8360	Dyscophellus phraxanorDHJ01	Pyrginae	00-SRNP-22143	CSCR095-04	DQ292307
8361	Dyscophellus phraxanorDHJ01	Pyrginae	98-SRNP-7950	MHAHJ018-07	JF752625
8362	Dyscophellus phraxanorDHJ01	Pyrginae	99-SRNP-12369	MHAHJ034-07	JF752628
8363	Dyscophellus phraxanorDHJ01	Pyrginae	99-SRNP-13331	MHAHJ047-07	JF752630
8364	Dyscophellus phraxanorDHJ01	Pyrginae	99-SRNP-12797	MHAHJ108-07	JF752645
8365	Dyscophellus phraxanorDHJ01	Pyrginae	07-SRNP-41526	MHAHL536-07	JF762120
8366	Dyscophellus phraxanorDHJ01	Pyrginae	07-SRNP-41240	MHAHL548-07	JF762122
8367	Dyscophellus phraxanorDHJ01	Pyrginae	07-SRNP-65772	MHMXT224-08	JF762123
8368	Dyscophellus phraxanorDHJ01	Pyrginae	08-SRNP-65547	MHMXX857-09	JF777879
8369	Dyscophellus phraxanorDHJ01	Pyrginae	09-SRNP-68521	MHMYI626-10	HQ963940
8370	Dyscophellus phraxanorDHJ01	Pyrginae	09-SRNP-68520	MHMYI627-10	HQ963941
8371	Dyscophellus phraxanorDHJ04	Pyrginae	99-SRNP-12766	MHAHJ119-07	JF752760
8372	Dyscophellus phraxanorDHJ04	Pyrginae	00-SRNP-4233	MHAHJ049-07	JF752759
8373	Dyscophellus phraxanorDHJ04	Pyrginae	07-SRNP-65144	MHAHL533-07	JF762146
8374	Dyscophellus phraxanorDHJ04	Pyrginae	08-SRNP-70094	MHMXX858-09	JF777881
8375	Dyscophellus phraxanorDHJ04	Pyrginae	99-SRNP-12825	MHAHJ120-07	JF752761
8376	Dyscophellus phraxanorDHJ04	Pyrginae	09-SRNP-68523	MHMYI628-10	HQ963942
8377	Dyscophellus phraxanorDHJ02	Pyrginae	08-SRNP-66081	MHMXZ005-09	GU665267
8378	Dyscophellus phraxanorDHJ02	Pyrginae	07-SRNP-65679	MHMXT223-08	JF762125
8379	Dyscophellus phraxanorDHJ02	Pyrginae	00-SRNP-12285	MHAHJ076-07	JF752710
8380	Dyscophellus phraxanorDHJ02	Pyrginae	00-SRNP-14612	MHAHC499-05	DQ292326
8381	Dyscophellus phraxanorDHJ02	Pyrginae	99-SRNP-12737	MHAHJ106-07	JF752724
8382	Dyscophellus phraxanorDHJ02	Pyrginae	99-SRNP-12807	MHAHJ109-07	JF752725
8383	Dyscophellus phraxanorDHJ02	Pyrginae	02-SRNP-6962	MHAHJ129-07	JF752736
8384	Dyscophellus phraxanorDHJ02	Pyrginae	06-SRNP-65714	MHMXK075-07	JF762138
8385	Dyscophellus phraxanorDHJ02	Pyrginae	09-SRNP-67283	MHMYE1452-09	GU653546
8386	Dyscophellus phraxanorDHJ03	Pyrginae	07-SRNP-65163	MHMXO886-08	JF762145
8387	Dyscophellus phraxanorDHJ03	Pyrginae	01-SRNP-4859	CSRII123-04	DQ292311
8388	Dyscophellus phraxanorDHJ03	Pyrginae	08-SRNP-7035	MHMYC538-09	GU649806
8389	Dyscophellus phraxanorDHJ03	Pyrginae	01-SRNP-23461	MHAHJ135-07	JF752758
8390	Dyscophellus phraxanorDHJ03	Pyrginae	01-SRNP-22998	MHAHJ134-07	JF752757
8391	Dyscophellus phraxanorDHJ03	Pyrginae	01-SRNP-23558	MHAHC522-05	DQ292349
8392	Dyscophellus phraxanorDHJ03	Pyrginae	01-SRNP-22434	CSRII120-04	DQ292308
8393	Dyscophellus phraxanorDHJ03	Pyrginae	97-SRNP-6236	MHAHJ059-07	JF752746
8394	Dyscophellus phraxanorDHJ03	Pyrginae	00-SRNP-14728	MHAHJ081-07	JF752748
8395	Dyscophellus phraxanorDHJ03	Pyrginae	07-SRNP-42162	MHMXO888-08	JF762143
8396	Dyscophellus phraxanorDHJ03	Pyrginae	07-SRNP-65384	MHMXR743-08	JF762142
8397	Dyscophellus phraxanorDHJ03	Pyrginae	02-SRNP-2879	MHAHJ133-07	JF752756
8398	Dyscophellus phraxanorDHJ03	Pyrginae	07-SRNP-65189	MHAHL534-07	JF762140
8399	Dyscophellus phraxanorDHJ03	Pyrginae	98-SRNP-15000	MHAHJ066-07	JF752747

Tree Order	Species	Subfamily	ACG Sampleid	BOLD Processid	Genbank Accession
8400	Dyscophellus phraxanorDHJ03	Pyrginae	99-SRNP-12565	MHAHJ041-07	JF752745
8401	Dyscophellus phraxanorDHJ03	Pyrginae	99-SRNP-5787	MHAHJ029-07	JF752744
8402	Dyscophellus phraxanorDHJ03	Pyrginae	04-SRNP-27330	MHAHI682-06	GU155956
8403	Dyscophellus phraxanorDHJ03	Pyrginae	04-SRNP-27328	MHAHF146-06	GU150387
8404	Dyscophellus phraxanorDHJ03	Pyrginae	04-SRNP-34002	MHAHE298-05	GU149652
8405	Dyscophellus phraxanorDHJ03	Pyrginae	99-SRNP-13498	MHAHC513-05	DQ292340
8406	Dyscophellus phraxanorDHJ02	Pyrginae	99-SRNP-5485	MHAHJ011-07	JF752662
8407	Dyscophellus phraxanorDHJ02	Pyrginae	98-SRNP-6815	MHAHJ019-07	JF752668
8408	Dyscophellus phraxanorDHJ02	Pyrginae	99-SRNP-12566	MHAHJ043-07	JF752686
8409	Dyscophellus phraxanorDHJ02	Pyrginae	01-SRNP-22109	CSRII450-04	DQ292316
8410	Dyscophellus phraxanorDHJ02	Pyrginae	97-SRNP-6367	MHAHJ003-07	JF752654
8411	Dyscophellus phraxanorDHJ02	Pyrginae	01-SRNP-3190	CSRII449-04	DQ292315
8412	Dyscophellus phraxanorDHJ02	Pyrginae	99-SRNP-12255	MHAHJ057-07	JF752696
8413	Dyscophellus phraxanorDHJ02	Pyrginae	97-SRNP-6218	MHAHJ062-07	JF752699
8414	Dyscophellus phraxanorDHJ02	Pyrginae	99-SRNP-5786	MHAHJ069-07	JF752703
8415	Dyscophellus phraxanorDHJ03	Pyrginae	01-SRNP-3948	MHAHJ089-07	JF752750
8416	Dyscophellus phraxanorDHJ03	Pyrginae	99-SRNP-12568	MHAHJ100-07	JF752753
8417	Dyscophellus phraxanorDHJ03	Pyrginae	99-SRNP-13505	MHAHJ113-07	JF752754
8418	Dyscophellus phraxanorDHJ03	Pyrginae	99-SRNP-15163	MHAHJ116-07	JF752755
8419	Dyscophellus phraxanorDHJ03	Pyrginae	07-SRNP-45755	MHMXR754-08	JF762141
8420	Dyscophellus phraxanorDHJ03	Pyrginae	08-SRNP-31603	MHMXW123-09	JF753873
8421	Dyscophellus phraxanorDHJ03	Pyrginae	07-SRNP-45264	MHMXO887-08	JF762144
8422	Dyscophellus phraxanorDHJ03	Pyrginae	99-SRNP-12512	MHAHJ099-07	JF752752
8423	Dyscophellus phraxanorDHJ03	Pyrginae	01-SRNP-23096	MHAHJ092-07	JF752751
8424	Dyscophellus phraxanorDHJ03	Pyrginae	99-SRNP-12186	MHAHJ014-07	JF752742
8425	Dyscophellus phraxanorDHJ03	Pyrginae	01-SRNP-22953	CSRII121-04	DQ292309
8426	Dyscophellus phraxanorDHJ03	Pyrginae	02-SRNP-18265	MHAHC520-05	DQ292347
8427	Dyscophellus phraxanorDHJ03	Pyrginae	98-SRNP-14893	MHAHJ027-07	JF752743
8428	Dyscophellus phraxanorDHJ03	Pyrginae	05-SRNP-20557	MHAHI681-06	GU155955
8429	Dyscophellus phraxanorDHJ03	Pyrginae	01-SRNP-9032	MHAHJ085-07	JF752749
8430	Dyscophellus phraxanorDHJ03	Pyrginae	08-SRNP-32859	MHMYC540-09	GU649777
8431	Dyscophellus phraxanorDHJ02	Pyrginae	08-SRNP-6169	MHMXZ007-09	
8432	Dyscophellus phraxanorDHJ02	Pyrginae	00-SRNP-14734	MHAHC506-05	DQ292333
8433	Dyscophellus phraxanorDHJ02	Pyrginae	99-SRNP-13462	MHAHJ121-07	JF752731
8434	Dyscophellus phraxanorDHJ02	Pyrginae	01-SRNP-9165	MHAHC510-05	DQ292337
8435	Dyscophellus phraxanorDHJ02	Pyrginae	99-SRNP-5874	MHAHJ012-07	JF752663
8436	Dyscophellus phraxanorDHJ02	Pyrginae	02-SRNP-3484	MHAHC523-05	DQ292350
8437	Dyscophellus phraxanorDHJ02	Pyrginae	00-SRNP-11848	MHAHC508-05	DQ292335
8438	Dyscophellus phraxanorDHJ02	Pyrginae	00-SRNP-11388	MHAHC500-05	DQ292327
8439	Dyscophellus phraxanorDHJ02	Pyrginae	99-SRNP-5875	MHAHJ021-07	JF752670
8440	Dyscophellus phraxanorDHJ02	Pyrginae	00-SRNP-4333	MHAHJ071-07	JF752705
8441	Dyscophellus phraxanorDHJ02	Pyrginae	99-SRNP-12489	MHAHJ098-07	JF752720
8442	Dyscophellus phraxanorDHJ02	Pyrginae	00-SRNP-14680	MHAHC496-05	DQ292323
8443	Dyscophellus phraxanorDHJ02	Pyrginae	01-SRNP-3501	MHAHJ126-07	JF752734
8444	Dyscophellus phraxanorDHJ02	Pyrginae	07-SRNP-32987	MHMXR742-08	JF762132
8445	Dyscophellus phraxanorDHJ02	Pyrginae	04-SRNP-34005	MHAHE299-05	GU149648
8446	Dyscophellus phraxanorDHJ02	Pyrginae	04-SRNP-34935	MHAHE300-05	GU149651
8447	Dyscophellus phraxanorDHJ02	Pyrginae	07-SRNP-32986	MHMXR745-08	JF762130
8448	Dyscophellus phraxanorDHJ02	Pyrginae	98-SRNP-6943	MHAHJ024-07	JF752672
8449	Dyscophellus phraxanorDHJ02	Pyrginae	02-SRNP-3485	MHAHC529-05	DQ292356
8450	Dyscophellus phraxanorDHJ02	Pyrginae	02-SRNP-3481	MHAHC528-05	DQ292355
8451	Dyscophellus phraxanorDHJ02	Pyrginae	99-SRNP-13135	MHAHC517-05	DQ292344
8452	Dyscophellus phraxanorDHJ02	Pyrginae	00-SRNP-21015	MHAHC507-05	DQ292334
8453	Dyscophellus phraxanorDHJ02	Pyrginae	00-SRNP-4194	MHAHC505-05	DQ292332
8454	Dyscophellus phraxanorDHJ02	Pyrginae	01-SRNP-3303	MHAHC498-05	DQ292325
8455	Dyscophellus phraxanorDHJ02	Pyrginae	01-SRNP-1559	MHAHC494-05	DQ292321

Tree Order	Species	Subfamily	ACG Sampleid	BOLD Processid	Genbank Accession
8456	Dyscophellus phraxanorDHJ02	Pyrginae	06-SRNP-44849	MHMXK074-07	JF762139
8457	Dyscophellus phraxanorDHJ02	Pyrginae	02-SRNP-6149	CSRII127-04	DQ292314
8458	Dyscophellus phraxanorDHJ02	Pyrginae	04-SRNP-23209	MHAHE297-05	GU149649
8459	Dyscophellus phraxanorDHJ02	Pyrginae	01-SRNP-3381	MHAHC521-05	DQ292348
8460	Dyscophellus phraxanorDHJ02	Pyrginae	01-SRNP-9118	MHAHC503-05	DQ292330
8461	Dyscophellus phraxanorDHJ02	Pyrginae	01-SRNP-3519	MHAHC491-05	DQ292318
8462	Dyscophellus phraxanorDHJ02	Pyrginae	97-SRNP-6945	MHAHJ009-07	JF752660
8463	Dyscophellus phraxanorDHJ02	Pyrginae	01-SRNP-25126	CSRII122-04	DQ292310
8464	Dyscophellus phraxanorDHJ02	Pyrginae	01-SRNP-22999	MHAHC515-05	DQ292342
8465	Dyscophellus phraxanorDHJ02	Pyrginae	01-SRNP-3785	MHAHJ055-07	JF752694
8466	Dyscophellus phraxanorDHJ02	Pyrginae	02-SRNP-18329	CSRII125-04	DQ292312
8467	Dyscophellus phraxanorDHJ02	Pyrginae	97-SRNP-6959	MHAHJ005-07	JF752656
8468	Dyscophellus phraxanorDHJ02	Pyrginae	00-SRNP-4255	MHAHJ070-07	JF752704
8469	Dyscophellus phraxanorDHJ02	Pyrginae	97-SRNP-6601	MHAHJ061-07	JF752698
8470	Dyscophellus phraxanorDHJ02	Pyrginae	99-SRNP-12220	MHAHJ058-07	JF752697
8471	Dyscophellus phraxanorDHJ02	Pyrginae	99-SRNP-12767	MHAHJ044-07	JF752687
8472	Dyscophellus phraxanorDHJ02	Pyrginae	99-SRNP-12263	MHAHJ032-07	JF752677
8473	Dyscophellus phraxanorDHJ02	Pyrginae	99-SRNP-13041	MHAHJ111-07	JF752727
8474	Dyscophellus phraxanorDHJ02	Pyrginae	99-SRNP-12798	MHAHJ050-07	JF752690
8475	Dyscophellus phraxanorDHJ02	Pyrginae	00-SRNP-11209	MHAHJ073-07	JF752707
8476	Dyscophellus phraxanorDHJ02	Pyrginae	99-SRNP-12200	MHAHJ031-07	JF752676
8477	Dyscophellus phraxanorDHJ02	Pyrginae	99-SRNP-13130	MHAHJ045-07	JF752688
8478	Dyscophellus phraxanorDHJ02	Pyrginae	01-SRNP-25001	MHAHJ128-07	JF752735
8479	Dyscophellus phraxanorDHJ02	Pyrginae	01-SRNP-22878	CSRII451-04	DQ292317
8480	Dyscophellus phraxanorDHJ02	Pyrginae	98-SRNP-6998	MHAHJ015-07	JF752665
8481	Dyscophellus phraxanorDHJ02	Pyrginae	97-SRNP-6566	MHAHJ004-07	JF752655
8482	Dyscophellus phraxanorDHJ02	Pyrginae	97-SRNP-6747	MHAHJ008-07	JF752659
8483	Dyscophellus phraxanorDHJ02	Pyrginae	00-SRNP-4551	MHAHJ072-07	JF752706
8484	Dyscophellus phraxanorDHJ02	Pyrginae	02-SRNP-3885	MHAHJ138-07	JF752741
8485	Dyscophellus phraxanorDHJ02	Pyrginae	08-SRNP-65057	MHMXT140-08	JF762127
8486	Dyscophellus phraxanorDHJ02	Pyrginae	08-SRNP-2144	MHMXW147-09	JF753872
8487	Dyscophellus phraxanorDHJ02	Pyrginae	01-SRNP-3380	MHAHJ123-07	JF752733
8488	Dyscophellus phraxanorDHJ02	Pyrginae	01-SRNP-3900	MHAHJ131-07	JF752738
8489	Dyscophellus phraxanorDHJ02	Pyrginae	01-SRNP-3947	MHAHJ132-07	JF752739
8490	Dyscophellus phraxanorDHJ02	Pyrginae	07-SRNP-30937	MHAHL535-07	JF762124
8491	Dyscophellus phraxanorDHJ02	Pyrginae	07-SRNP-45419	MHMXO884-08	JF762137
8492	Dyscophellus phraxanorDHJ02	Pyrginae	07-SRNP-65225	MHMXO906-08	JF762133
8493	Dyscophellus phraxanorDHJ02	Pyrginae	99-SRNP-13193	MHAHJ112-07	JF752728
8494	Dyscophellus phraxanorDHJ02	Pyrginae	99-SRNP-15209	MHAHJ122-07	JF752732
8495	Dyscophellus phraxanorDHJ02	Pyrginae	07-SRNP-65246	MHMXR744-08	JF762131
8496	Dyscophellus phraxanorDHJ02	Pyrginae	07-SRNP-33141	MHMXR746-08	JF762129
8497	Dyscophellus phraxanorDHJ02	Pyrginae	07-SRNP-65247	MHMXR747-08	JF762128
8498	Dyscophellus phraxanorDHJ02	Pyrginae	08-SRNP-4730	MHMXX675-09	JF777880
8499	Dyscophellus phraxanorDHJ02	Pyrginae	08-SRNP-65758	MHMXZ004-09	GU665266
8500	Dyscophellus phraxanorDHJ02	Pyrginae	08-SRNP-65873	MHMXZ006-09	GU665262
8501	Dyscophellus phraxanorDHJ02	Pyrginae	99-SRNP-12518	MHAHJ040-07	JF752684
8502	Dyscophellus phraxanorDHJ02	Pyrginae	99-SRNP-12486	MHAHJ042-07	JF752685
8503	Dyscophellus phraxanorDHJ02	Pyrginae	98-SRNP-15081	MHAHJ065-07	JF752701
8504	Dyscophellus phraxanorDHJ02	Pyrginae	00-SRNP-12297	MHAHJ075-07	JF752709
8505	Dyscophellus phraxanorDHJ02	Pyrginae	01-SRNP-3326	MHAHJ090-07	JF752715
8506	Dyscophellus phraxanorDHJ02	Pyrginae	99-SRNP-12366	MHAHJ095-07	JF752718
8507	Dyscophellus phraxanorDHJ02	Pyrginae	99-SRNP-12485	MHAHJ097-07	JF752719
8508	Dyscophellus phraxanorDHJ02	Pyrginae	99-SRNP-12722	MHAHJ102-07	JF752721
8509	Dyscophellus phraxanorDHJ02	Pyrginae	99-SRNP-12705	MHAHJ105-07	JF752723
8510	Dyscophellus phraxanorDHJ02	Pyrginae	99-SRNP-12881	MHAHJ110-07	JF752726
8511	Dyscophellus phraxanorDHJ02	Pyrginae	99-SRNP-12370	MHAHJ035-07	JF752679

Tree Order	Species	Subfamily	ACG Sampleid	BOLD Processid	Genbank Accession
8512	Dyscophellus phraxanorDHJ02	Pyrginae	99-SRNP-12381	MHAHJ036-07	JF752680
8513	Dyscophellus phraxanorDHJ02	Pyrginae	99-SRNP-12380	MHAHJ037-07	JF752681
8514	Dyscophellus phraxanorDHJ02	Pyrginae	99-SRNP-12383	MHAHJ038-07	JF752682
8515	Dyscophellus phraxanorDHJ02	Pyrginae	08-SRNP-66201	MHMYC539-09	GU649807
8516	Dyscophellus phraxanorDHJ02	Pyrginae	09-SRNP-56856	MHMYE1448-09	GU653547
8517	Dyscophellus phraxanorDHJ02	Pyrginae	09-SRNP-57092	MHMYE1451-09	GU653545
8518	Dyscophellus phraxanorDHJ02	Pyrginae	00-SRNP-22220	MHAHJ064-07	JF752700
8519	Dyscophellus phraxanorDHJ02	Pyrginae	99-SRNP-12282	MHAHJ056-07	JF752695
8520	Dyscophellus phraxanorDHJ02	Pyrginae	99-SRNP-12435	MHAHJ039-07	JF752683
8521	Dyscophellus phraxanorDHJ02	Pyrginae	99-SRNP-4100	MHAHJ025-07	JF752673
8522	Dyscophellus phraxanorDHJ02	Pyrginae	99-SRNP-4009	MHAHJ022-07	JF752671
8523	Dyscophellus phraxanorDHJ02	Pyrginae	01-SRNP-22948	MHAHC527-05	DQ292354
8524	Dyscophellus phraxanorDHJ02	Pyrginae	02-SRNP-18890	MHAHC526-05	DQ292353
8525	Dyscophellus phraxanorDHJ02	Pyrginae	99-SRNP-13335	MHAHC514-05	DQ292341
8526	Dyscophellus phraxanorDHJ02	Pyrginae	99-SRNP-13173	MHAHC502-05	DQ292329
8527	Dyscophellus phraxanorDHJ02	Pyrginae	01-SRNP-1865	MHAHC492-05	DQ292319
8528	Dyscophellus phraxanorDHJ02	Pyrginae	99-SRNP-4099	CSCR094-04	DQ292306
8529	Dyscophellus phraxanorDHJ02	Pyrginae	00-SRNP-12748	MHAHC509-05	DQ292336
8530	Dyscophellus phraxanorDHJ02	Pyrginae	02-SRNP-3831	CSRII126-04	DQ292313
8531	Dyscophellus phraxanorDHJ02	Pyrginae	99-SRNP-12291	MHAHJ033-07	JF752678
8532	Dyscophellus phraxanorDHJ02	Pyrginae	98-SRNP-15001	CSCR093-04	DQ292305
8533	Dyscophellus phraxanorDHJ02	Pyrginae	99-SRNP-4098	MHAHJ010-07	JF752661
8534	Dyscophellus phraxanorDHJ02	Pyrginae	98-SRNP-6939	MHAHJ016-07	JF752666
8535	Dyscophellus phraxanorDHJ02	Pyrginae	97-SRNP-6580	MHAHJ007-07	JF752658
8536	Dyscophellus phraxanorDHJ02	Pyrginae	97-SRNP-6790	MHAHJ006-07	JF752657
8537	Dyscophellus phraxanorDHJ02	Pyrginae	99-SRNP-5790	MHAHJ020-07	JF752669
8538	Dyscophellus phraxanorDHJ02	Pyrginae	98-SRNP-6941	MHAHJ017-07	JF752667
8539	Dyscophellus phraxanorDHJ02	Pyrginae	97-SRNP-6220	MHAHJ001-07	JF752652
8540	Dyscophellus phraxanorDHJ02	Pyrginae	99-SRNP-12194	MHAHJ068-07	JF752702
8541	Dyscophellus phraxanorDHJ02	Pyrginae	00-SRNP-14791	MHAHJ080-07	JF752711
8542	Dyscophellus phraxanorDHJ02	Pyrginae	01-SRNP-497	MHAHJ086-07	JF752713
8543	Dyscophellus phraxanorDHJ02	Pyrginae	01-SRNP-9119	MHAHJ087-07	JF752714
8544	Dyscophellus phraxanorDHJ02	Pyrginae	00-SRNP-21016	MHAHJ091-07	JF752716
8545	Dyscophellus phraxanorDHJ02	Pyrginae	99-SRNP-12367	MHAHJ093-07	JF752717
8546	Dyscophellus phraxanorDHJ02	Pyrginae	99-SRNP-12688	MHAHJ104-07	JF752722
8547	Dyscophellus phraxanorDHJ02	Pyrginae	07-SRNP-45265	MHMXO885-08	JF762136
8548	Dyscophellus phraxanorDHJ02	Pyrginae	07-SRNP-45263	MHMXO889-08	JF762135
8549	Dyscophellus phraxanorDHJ02	Pyrginae	09-SRNP-72343	MHMYG2452-10	HM885878
8550	Dyscophellus phraxanorDHJ02	Pyrginae	97-SRNP-6512	MHAHJ002-07	JF752653
8551	Dyscophellus phraxanorDHJ02	Pyrginae	09-SRNP-65857	MHMYE1447-09	GU653550
8552	Dyscophellus phraxanorDHJ02	Pyrginae	09-SRNP-32343	MHMYE1449-09	GU653548
8553	Dyscophellus phraxanorDHJ02	Pyrginae	09-SRNP-67110	MHMYE1450-09	HQ992247
8554	Dyscophellus phraxanorDHJ02	Pyrginae	09-SRNP-72763	MHMYG2453-10	HM885879
8555	Dyscophellus phraxanorDHJ02	Pyrginae	07-SRNP-45964	MHMXO905-08	JF762134
8556	Dyscophellus phraxanorDHJ02	Pyrginae	07-SRNP-45952	MHMXT141-08	JF762126
8557	Dyscophellus phraxanorDHJ02	Pyrginae	01-SRNP-2916	MHAHJ130-07	JF752737
8558	Dyscophellus phraxanorDHJ02	Pyrginae	02-SRNP-2635	MHAHJ137-07	JF752740
8559	Dyscophellus phraxanorDHJ02	Pyrginae	00-SRNP-1057	MHAHJ115-07	JF752729
8560	Dyscophellus phraxanorDHJ02	Pyrginae	01-SRNP-1058	MHAHJ083-07	JF752712
8561	Dyscophellus phraxanorDHJ02	Pyrginae	00-SRNP-12293	MHAHJ074-07	JF752708
8562	Dyscophellus phraxanorDHJ02	Pyrginae	99-SRNP-13496	MHAHJ054-07	JF752693
8563	Dyscophellus phraxanorDHJ02	Pyrginae	99-SRNP-13336	MHAHJ053-07	JF752692
8564	Dyscophellus phraxanorDHJ02	Pyrginae	99-SRNP-13063	MHAHJ051-07	JF752691
8565	Dyscophellus phraxanorDHJ02	Pyrginae	98-SRNP-15079	MHAHJ026-07	JF752674
8566	Dyscophellus phraxanorDHJ02	Pyrginae	99-SRNP-12290	MHAHJ013-07	JF752664
8567	Dyscophellus phraxanorDHJ02	Pyrginae	04-SRNP-34379	MHAHE296-05	GU149650

Tree Order	Species	Subfamily	ACG Sampleid	BOLD Processid	Genbank Accession
8568	Dyscophellus phraxanorDHJ02	Pyrginae	02-SRNP-481	MHAHC518-05	DQ292345
8569	Dyscophellus phraxanorDHJ02	Pyrginae	01-SRNP-22947	MHAHC516-05	DQ292343
8570	Dyscophellus phraxanorDHJ02	Pyrginae	01-SRNP-23370	MHAHC512-05	DQ292339
8571	Dyscophellus phraxanorDHJ02	Pyrginae	01-SRNP-3488	MHAHC511-05	DQ292338
8572	Dyscophellus phraxanorDHJ02	Pyrginae	00-SRNP-4520	MHAHC497-05	DQ292324
8573	Dyscophellus phraxanorDHJ02	Pyrginae	99-SRNP-12028	MHAHJ030-07	JF752675
8574	Dyscophellus phraxanorDHJ02	Pyrginae	99-SRNP-13339	MHAHJ048-07	JF752689
8575	Dyscophellus phraxanorDHJ02	Pyrginae	00-SRNP-4195	MHAHJ117-07	JF752730
8576	Dyscophellus phraxanorDHJ02	Pyrginae	09-SRNP-68551	MHMYI629-10	HQ963943
8577	Dyscophellus Burns02	Pyrginae	05-SRNP-33035	MHAHI680-06	GU155954
8578	Dyscophellus Burns02	Pyrginae	04-SRNP-61423	MHAHF542-06	GU150385
8579	Dyscophellus Burns02	Pyrginae	00-SRNP-21086	MHAHH149-06	GU155365
8580	Dyscophellus Burns02	Pyrginae	98-SRNP-6071	MHAHH151-06	GU155359
8581	Dyscophellus Burns02	Pyrginae	08-SRNP-70869	MHMXX688-09	JF777877
8582	Dyscophellus Burns02	Pyrginae	06-SRNP-4389	MHAHJ610-07	JF752624
8583	Dyscophellus Burns02	Pyrginae	02-SRNP-6754	MHAHH147-06	GU155367
8584	Dyscophellus Burns02	Pyrginae	03-SRNP-12963.1	MHAHH148-06	GU155366
8585	Dyscophellus Burns02	Pyrginae	04-SRNP-32586	MHAHE316-05	GU149647
8586	Dyscophellus Burns02	Pyrginae	06-SRNP-812	MHAHG715-06	GU151386
8587	Dyscophellus Burns02	Pyrginae	02-SRNP-7471	CSCR100-04	DQ292302
8588	Dyscophellus Burns02	Pyrginae	02-SRNP-6761	CSCR099-04	DQ292301
8589	Dyscophellus Burns02	Pyrginae	04-SRNP-41726	MHAHE315-05	GU149645
8590	Dyscophellus Burns02	Pyrginae	04-SRNP-3811	MHAHE317-05	GU149646
8591	Dyscophellus Burns02	Pyrginae	04-SRNP-41073	MHAHE341-05	GU149644
8592	Dyscophellus Burns02	Pyrginae	05-SRNP-40223	MHAHF152-06	GU150386
8593	Dyscophellus Burns02	Pyrginae	02-SRNP-7460	MHAHH145-06	GU155362
8594	Dyscophellus Burns02	Pyrginae	01-SRNP-468	MHAHH146-06	GU155360
8595	Dyscophellus Burns02	Pyrginae	03-SRNP-11903	MHAHH150-06	GU155363
8596	Dyscophellus Burns02	Pyrginae	02-SRNP-7467	MHAHH152-06	GU155358
8597	Dyscophellus Burns02	Pyrginae	01-SRNP-5675	MHAHH153-06	GU155361
8598	Dyscophellus Burns02	Pyrginae	06-SRNP-40602	MHAHH433-06	GU155364
8599	Dyscophellus Burns02	Pyrginae	08-SRNP-70835	MHMXW120-09	JF753871
8600	Dyscophellus Burns02	Pyrginae	08-SRNP-65702	MHMXX689-09	JF777878
8601	Dyscophellus porcius	Pyrginae	01-SRNP-5603	CSCR096-04	DQ292357
8602	Dyscophellus porcius	Pyrginae	07-SRNP-42003	MHMXO893-08	JF762147
8603	Dyscophellus Burns01	Pyrginae	08-SRNP-4491	MHMXX685-09	JF777873
8604	Dyscophellus Burns01	Pyrginae	08-SRNP-914	MHMXW143-09	JF753868
8605	Dyscophellus Burns01	Pyrginae	08-SRNP-32137	MHMXX682-09	JF777870
8606	Dyscophellus Burns01	Pyrginae	08-SRNP-70995	MHMXX683-09	JF777871
8607	Dyscophellus Burns01	Pyrginae	08-SRNP-65309	MHMXW133-09	JF753863
8608	Dyscophellus Burns01	Pyrginae	08-SRNP-790	MHMXW137-09	JF753865
8609	Dyscophellus Burns01	Pyrginae	08-SRNP-21088	MHMXW129-09	JF753859
8610	Dyscophellus Burns01	Pyrginae	08-SRNP-21089	MHMXW130-09	JF753860
8611	Dyscophellus Burns01	Pyrginae	07-SRNP-45367	MHMXR757-08	JF762111
8612	Dyscophellus Burns01	Pyrginae	07-SRNP-33600	MHMXT228-08	JF762108
8613	Dyscophellus Burns01	Pyrginae	07-SRNP-2605	MHMXO903-08	JF762116
8614	Dyscophellus Burns01	Pyrginae	07-SRNP-45366	MHMXR741-08	JF762112
8615	Dyscophellus Burns01	Pyrginae	07-SRNP-2606	MHAHL551-07	JF762106
8616	Dyscophellus Burns01	Pyrginae	07-SRNP-1820	MHAHL550-07	JF762105
8617	Dyscophellus Burns01	Pyrginae	07-SRNP-31532	MHAHL547-07	JF762104
8618	Dyscophellus Burns01	Pyrginae	07-SRNP-1821	MHAHL546-07	JF762103
8619	Dyscophellus Burns01	Pyrginae	06-SRNP-4434	MHAHI133-06	GU155948
8620	Dyscophellus Burns01	Pyrginae	06-SRNP-45197	MHAHG718-06	GU151385
8621	Dyscophellus Burns01	Pyrginae	06-SRNP-2566	MHAHG717-06	GU151384
8622	Dyscophellus Burns01	Pyrginae	04-SRNP-1382	MHAHE347-05	GU149643
8623	Dyscophellus Burns01	Pyrginae	04-SRNP-3019	MHAHD753-05	GU161465

Tree Order	Species	Subfamily	ACG Sampleid	BOLD Processid	Genbank Accession
8624	Dyscophellus Burns01	Pyrginae	04-SRNP-1472	MHAHD752-05	GU161464
8625	Dyscophellus Burns01	Pyrginae	04-SRNP-1145	MHAHD750-05	GU161462
8626	Dyscophellus Burns01	Pyrginae	04-SRNP-41583	MHAHD749-05	GU161463
8627	Dyscophellus Burns01	Pyrginae	05-SRNP-34746	MHAHG118-06	GU151382
8628	Dyscophellus Burns01	Pyrginae	02-SRNP-17977	CSRII118-04	DQ292300
8629	Dyscophellus Burns01	Pyrginae	03-SRNP-5975	CSCR357-04	DQ292296
8630	Dyscophellus Burns01	Pyrginae	01-SRNP-1161	CSRII116-04	DQ292298
8631	Dyscophellus Burns01	Pyrginae	01-SRNP-22233	CSRII117-04	DQ292299
8632	Dyscophellus Burns01	Pyrginae	07-SRNP-20844	MHAHL552-07	JF762107
8633	Dyscophellus Burns01	Pyrginae	08-SRNP-70860	MHMXX734-09	JF777876
8634	Dyscophellus Burns01	Pyrginae	08-SRNP-21379	MHMXW131-09	JF753861
8635	Dyscophellus Burns01	Pyrginae	08-SRNP-21611	MHMXW132-09	JF753862
8636	Dyscophellus Burns01	Pyrginae	08-SRNP-915	MHMXW112-09	JF753853
8637	Dyscophellus Burns01	Pyrginae	08-SRNP-1071	MHMXW115-09	JF753856
8638	Dyscophellus Burns01	Pyrginae	08-SRNP-32136	MHMXX684-09	JF777872
8639	Dyscophellus Burns01	Pyrginae	09-SRNP-57048	MHMYE1453-09	GU653543
8640	Dyscophellus Burns01	Pyrginae	07-SRNP-42747	MHMXT222-08	JF762110
8641	Dyscophellus Burns01	Pyrginae	09-SRNP-67513	MHMYE1454-09	GU653544
8642	Dyscophellus Burns01	Pyrginae	06-SRNP-5185	MHAHI134-06	GU155949
8643	Dyscophellus Burns01	Pyrginae	08-SRNP-2351	MHMXW141-09	JF753866
8644	Dyscophellus Burns01	Pyrginae	08-SRNP-70059	MHMXW134-09	JF753864
8645	Dyscophellus Burns01	Pyrginae	08-SRNP-1139	MHMXW114-09	JF753855
8646	Dyscophellus Burns01	Pyrginae	08-SRNP-1140	MHMXW113-09	JF753854
8647	Dyscophellus Burns01	Pyrginae	07-SRNP-46539	MHMXR739-08	JF762114
8648	Dyscophellus Burns01	Pyrginae	07-SRNP-65613	MHMXO892-08	JF762117
8649	Dyscophellus Burns01	Pyrginae	06-SRNP-5990	MHAHI132-06	GU155953
8650	Dyscophellus Burns01	Pyrginae	06-SRNP-31676	MHAHH434-06	GU155357
8651	Dyscophellus Burns01	Pyrginae	06-SRNP-2371	MHAHG716-06	GU151383
8652	Dyscophellus Burns01	Pyrginae	07-SRNP-45716	MHMXR740-08	JF762113
8653	Dyscophellus Burns01	Pyrginae	03-SRNP-1735	CSCR358-04	DQ292297
8654	Dyscophellus Burns01	Pyrginae	06-SRNP-5186	MHAHI137-06	GU155951
8655	Dyscophellus Burns01	Pyrginae	08-SRNP-1423	MHMXW144-09	JF753869
8656	Dyscophellus Burns01	Pyrginae	08-SRNP-1424	MHMXW145-09	JF753870
8657	Dyscophellus Burns01	Pyrginae	08-SRNP-70994	MHMXX687-09	JF777875
8658	Dyscophellus Burns01	Pyrginae	07-SRNP-42565	MHMXT227-08	JF762109
8659	Dyscophellus Burns01	Pyrginae	08-SRNP-40847	MHMXW142-09	JF753867
8660	Dyscophellus Burns01	Pyrginae	07-SRNP-3025	MHMXO891-08	JF762118
8661	Dyscophellus Burns01	Pyrginae	07-SRNP-833	MHMXK070-07	JF762119
8662	Dyscophellus Burns01	Pyrginae	06-SRNP-4432	MHAHI136-06	GU155950
8663	Dyscophellus Burns01	Pyrginae	06-SRNP-4193	MHAHI135-06	GU155952
8664	Dyscophellus Burns01	Pyrginae	04-SRNP-34190	MHAHD751-05	GU161461
8665	Dyscophellus Burns01	Pyrginae	07-SRNP-45717	MHMXO904-08	JF762115
8666	Dyscophellus Burns01	Pyrginae	08-SRNP-1274	MHMXW116-09	JF753857
8667	Dyscophellus Burns01	Pyrginae	08-SRNP-70502	MHMXW121-09	JF753858
8668	Dyscophellus Burns01	Pyrginae	08-SRNP-35933	MHMXX686-09	JF777874
8669	Dyscophellus Burns01	Pyrginae	09-SRNP-68136	MHMYG2458-10	HM885884
8670	Dyscophellus ramon	Pyrginae	01-SRNP-3569	MHAHH125-06	GU155384
8671	Dyscophellus ramon	Pyrginae	09-SRNP-40055	MHMYC541-09	GU649785
8672	Dyscophellus ramon	Pyrginae	03-SRNP-5396	CSCR359-04	DQ292360
8673	Dyscophellus ramon	Pyrginae	01-SRNP-25139	MHAHH114-06	GU155372
8674	Dyscophellus ramon	Pyrginae	01-SRNP-1770	MHAHH131-06	GU155393
8675	Dyscophellus ramon	Pyrginae	01-SRNP-22279	MHAHH138-06	GU155396
8676	Dyscophellus ramon	Pyrginae	01-SRNP-5763	MHAHH115-06	GU155371
8677	Dyscophellus ramon	Pyrginae	00-SRNP-12919	MHAHH129-06	GU155369
8678	Dyscophellus ramon	Pyrginae	07-SRNP-60549	MHMXR755-08	JF762150
8679	Dyscophellus ramon	Pyrginae	03-SRNP-5318	CSCR360-04	DQ292361

Tree Order	Species	Subfamily	ACG Sampleid	BOLD Processid	Genbank Accession
8680	Dyscophellus ramon	Pyrginae	01-SRNP-24190	CSCR098-04	DQ292359
8681	Dyscophellus ramon	Pyrginae	08-SRNP-1584	MHMXW140-09	JF753874
8682	Dyscophellus ramon	Pyrginae	08-SRNP-71274	MHMYC543-09	GU649803
8683	Dyscophellus ramon	Pyrginae	03-SRNP-30935	MHAHH123-06	GU155381
8684	Dyscophellus ramon	Pyrginae	02-SRNP-169	MHAHH126-06	GU155382
8685	Dyscophellus ramon	Pyrginae	08-SRNP-24064	MHMYB182-09	GU649664
8686	Dyscophellus ramon	Pyrginae	06-SRNP-370	MHAHG117-06	GU151387
8687	Dyscophellus ramon	Pyrginae	08-SRNP-24061	MHMYB183-09	GU649076
8688	Dyscophellus ramon	Pyrginae	08-SRNP-24063	MHMYB184-09	GU649659
8689	Dyscophellus ramon	Pyrginae	03-SRNP-30164	MHAHH122-06	GU155383
8690	Dyscophellus ramon	Pyrginae	00-SRNP-21755	MHAHH124-06	GU155385
8691	Dyscophellus ramon	Pyrginae	02-SRNP-18736	MHAHH135-06	GU155388
8692	Dyscophellus ramon	Pyrginae	02-SRNP-1636	MHAHH136-06	GU155394
8693	Dyscophellus ramon	Pyrginae	02-SRNP-4089	MHAHH142-06	GU155399
8694	Dyscophellus ramon	Pyrginae	07-SRNP-20011	MHMXK073-07	JF762152
8695	Dyscophellus ramon	Pyrginae	05-SRNP-42006	MHAHL038-07	JF762148
8696	Dyscophellus ramon	Pyrginae	07-SRNP-41237	MHAHL549-07	JF762149
8697	Dyscophellus ramon	Pyrginae	07-SRNP-45229	MHMXR749-08	JF762151
8698	Dyscophellus ramon	Pyrginae	08-SRNP-4118	MHMXX690-09	JF777882
8699	Dyscophellus ramon	Pyrginae	02-SRNP-33929	MHAHH140-06	GU155389
8700	Dyscophellus ramon	Pyrginae	01-SRNP-22278	MHAHH141-06	GU155397
8701	Dyscophellus ramon	Pyrginae	02-SRNP-20433	MHAHH137-06	GU155387
8702	Dyscophellus ramon	Pyrginae	00-SRNP-22195	MHAHH139-06	GU155395
8703	Dyscophellus ramon	Pyrginae	02-SRNP-7461	MHAHH132-06	GU155392
8704	Dyscophellus ramon	Pyrginae	02-SRNP-7470	MHAHH133-06	GU155391
8705	Dyscophellus ramon	Pyrginae	01-SRNP-22392	MHAHH127-06	GU155380
8706	Dyscophellus ramon	Pyrginae	02-SRNP-3214	MHAHH128-06	GU155375
8707	Dyscophellus ramon	Pyrginae	01-SRNP-3012	MHAHH121-06	GU155376
8708	Dyscophellus ramon	Pyrginae	01-SRNP-25138	MHAHH120-06	GU155379
8709	Dyscophellus ramon	Pyrginae	02-SRNP-7463	MHAHH119-06	GU155377
8710	Dyscophellus ramon	Pyrginae	02-SRNP-7117	MHAHH118-06	GU155378
8711	Dyscophellus ramon	Pyrginae	00-SRNP-11389	MHAHH117-06	GU155368
8712	Dyscophellus ramon	Pyrginae	02-SRNP-20419	MHAHH116-06	GU155370
8713	Dyscophellus ramon	Pyrginae	02-SRNP-19579	MHAHH113-06	GU155373
8714	Dyscophellus ramon	Pyrginae	01-SRNP-22456	MHAHH112-06	GU155374
8715	Dyscophellus ramon	Pyrginae	06-SRNP-77	MHAHG714-06	GU151388
8716	Dyscophellus ramon	Pyrginae	04-SRNP-60594	MHAHE340-05	GU149653
8717	Dyscophellus ramon	Pyrginae	04-SRNP-3520	MHAHE318-05	GU149654
8718	Dyscophellus ramon	Pyrginae	03-SRNP-5316	MHAHH130-06	GU155386
8719	Dyscophellus ramon	Pyrginae	02-SRNP-1606	MHAHH134-06	GU155390
8720	Dyscophellus ramon	Pyrginae	97-SRNP-6149	MHAHH143-06	GU155398
8721	Dyscophellus ramon	Pyrginae	01-SRNP-11993	CSCR097-04	DQ292358
8722	Dyscophellus ramon	Pyrginae	08-SRNP-4781	MHMXX691-09	JF777883
8723	Dyscophellus ramon	Pyrginae	08-SRNP-23241	MHMYB185-09	GU649660
8724	Dyscophellus ramon	Pyrginae	08-SRNP-58183	MHMYC542-09	GU649802
8725	Dyscophellus ramon	Pyrginae	09-SRNP-67984	MHMYG2459-10	HM885885
8726	Phareas coeleste	Pyrginae	02-SRNP-35232	CSCR191-04	DQ292970
8727	Phareas coeleste	Pyrginae	02-SRNP-35231	CSCR190-04	DQ292969
8728	Phareas coeleste	Pyrginae	04-SRNP-56226	MHAHD859-05	GU161783
8729	Phareas coeleste	Pyrginae	05-SRNP-30644	MHAHE447-05	GU149831
8730	Phareas coeleste	Pyrginae	05-SRNP-30576	MHAHE448-05	GU149829
8731	Phareas coeleste	Pyrginae	05-SRNP-30642	MHAHE449-05	GU149832
8732	Phareas coeleste	Pyrginae	05-SRNP-30469	MHAHE450-05	GU149833
8733	Phareas coeleste	Pyrginae	05-SRNP-30643	MHAHE451-05	GU149834
8734	Phareas coeleste	Pyrginae	05-SRNP-30577	MHAHE452-05	GU149830
8735	Phocides belus	Pyrginae	06-SRNP-55175	MHAHG339-06	GU151551

Tree Order	Species	Subfamily	ACG Sampleid	BOLD Processid	Genbank Accession
8736	Phocides belus	Pyrginae	06-SRNP-55419	MHAHG346-06	GU151556
8737	Phocides belus	Pyrginae	02-SRNP-10072	CSCR193-04	DQ292972
8738	Phocides belus	Pyrginae	06-SRNP-55185	MHAHG345-06	GU151557
8739	Phocides belus	Pyrginae	96-SRNP-51	MHMXI722-07	JF761008
8740	Phocides belus	Pyrginae	01-SRNP-18748	MHMXI717-07	JF761015
8741	Phocides belus	Pyrginae	96-SRNP-12439	MHMXI719-07	JF761001
8742	Phocides belus	Pyrginae	96-SRNP-12538	MHMXI720-07	JF761012
8743	Phocides belus	Pyrginae	96-SRNP-12436	MHMXI721-07	JF761010
8744	Phocides belus	Pyrginae	07-SRNP-55324	MHMXK151-07	JF762597
8745	Phocides belus	Pyrginae	07-SRNP-55469	MHMXK156-07	JF762594
8746	Phocides belus	Pyrginae	06-SRNP-55343	MHAHG342-06	GU151553
8747	Phocides belus	Pyrginae	01-SRNP-17031	MHMXI710-07	JF761011
8748	Phocides belus	Pyrginae	07-SRNP-56444	MHMXK157-07	JF762593
8749	Phocides belus	Pyrginae	96-SRNP-12539	CSRII131-04	DQ292974
8750	Phocides Warren01	Pyrginae	00-SRNP-15104	CSRII134-04	DQ292996
8751	Phocides Warren01	Pyrginae	00-SRNP-15731	CSRII137-04	DQ292999
8752	Phocides Warren01	Pyrginae	01-SRNP-17035	CSCR197-04	DQ292995
8753	Phocides Warren01	Pyrginae	00-SRNP-8936	CSCR196-04	DQ292994
8754	Phocides Warren01	Pyrginae	00-SRNP-15124	CSRII135-04	DQ292997
8755	Phocides Warren01	Pyrginae	00-SRNP-8940	MHMXI700-07	JF761029
8756	Phocides Warren01	Pyrginae	00-SRNP-15109	MHMXI701-07	JF761027
8757	Phocides Warren01	Pyrginae	00-SRNP-15096	MHMXI702-07	JF761024
8758	Phocides Warren01	Pyrginae	00-SRNP-15097	MHMXI703-07	JF761023
8759	Phocides Warren01	Pyrginae	00-SRNP-15177	MHMXI705-07	JF761037
8760	Phocides Warren01	Pyrginae	00-SRNP-15103	MHMXI706-07	JF761035
8761	Phocides Warren01	Pyrginae	01-SRNP-17848	MHMXI708-07	JF761030
8762	Phocides Warren01	Pyrginae	00-SRNP-15187	MHMXI699-07	JF761021
8763	Phocides Warren01	Pyrginae	00-SRNP-15115	MHMXI698-07	JF761041
8764	Phocides Warren01	Pyrginae	01-SRNP-16953	MHMXI697-07	JF761032
8765	Phocides Warren01	Pyrginae	00-SRNP-15106	MHMXI696-07	JF761034
8766	Phocides Warren01	Pyrginae	00-SRNP-15221	MHMXI695-07	JF761036
8767	Phocides Warren01	Pyrginae	00-SRNP-15114	MHMXI693-07	JF761022
8768	Phocides Warren01	Pyrginae	00-SRNP-15894	MHMXI691-07	JF761026
8769	Phocides Warren01	Pyrginae	00-SRNP-15110	MHMXI690-07	JF761028
8770	Phocides Warren01	Pyrginae	01-SRNP-16950	MHMXI688-07	JF761040
8771	Phocides Warren01	Pyrginae	00-SRNP-15186	MHMXI687-07	JF761031
8772	Phocides pigmalionDHJ01	Pyrginae	95-SRNP-7824	CSRII139-04	DQ292984
8773	Phocides pigmalionDHJ01	Pyrginae	00-SRNP-4542	CSRII138-04	DQ292983
8774	Phocides Warren01	Pyrginae	00-SRNP-15238	CSRII136-04	DQ292998
8775	Phocides pigmalionDHJ01	Pyrginae	02-SRNP-14336	CSCR201-04	DQ292982
8776	Phocides pigmalionDHJ01	Pyrginae	02-SRNP-14447	MHAHC255-05	DQ292986
8777	Phocides pigmalionDHJ01	Pyrginae	00-SRNP-16167	MHAHC357-05	DQ292987
8778	Phocides pigmalionDHJ01	Pyrginae	96-SRNP-9016	MHAHC358-05	DQ292988
8779	Phocides pigmalionDHJ01	Pyrginae	00-SRNP-4322	MHAHC361-05	DQ292991
8780	Phocides pigmalionDHJ01	Pyrginae	96-SRNP-9017	MHAHC362-05	DQ292992
8781	Phocides pigmalionDHJ01	Pyrginae	96-SRNP-9015	MHAHC363-05	DQ292993
8782	Phocides pigmalionDHJ01	Pyrginae	04-SRNP-14106	MHAHD738-05	GU161784
8783	Phocides pigmalionDHJ01	Pyrginae	05-SRNP-61368	MHAHF591-06	GU150691
8784	Phocides pigmalionDHJ01	Pyrginae	07-SRNP-42344	MHMXO748-08	JF762616
8785	Phocides pigmalionDHJ01	Pyrginae	07-SRNP-42389	MHMXO754-08	JF762615
8786	Phocides pigmalionDHJ01	Pyrginae	07-SRNP-42343	MHMXO755-08	JF762614
8787	Phocides pigmalionDHJ01	Pyrginae	07-SRNP-42390	MHMXO758-08	JF762613
8788	Phocides pigmalionDHJ01	Pyrginae	08-SRNP-55462	MHMXW004-09	JF754075
8789	Phocides pigmalionDHJ01	Pyrginae	08-SRNP-5779	MHMXY1125-09	GU666417
8790	Phocides belus	Pyrginae	05-SRNP-64113	MHAHF592-06	GU150682
8791	Phocides belus	Pyrginae	06-SRNP-60076	MHAHK227-07	JF761003

Tree Order	Species	Subfamily	ACG Sampleid	BOLD Processid	Genbank Accession
8792	Phocides pigmalionDHJ01	Pyrginae	05-SRNP-6300	MHAHF588-06	GU150692
8793	Phocides pigmalionDHJ01	Pyrginae	00-SRNP-4541	CSCR200-04	DQ292981
8794	Phocides pigmalionDHJ01	Pyrginae	99-SRNP-16188	CSRII140-04	DQ292985
8795	Phocides pigmalionDHJ01	Pyrginae	06-SRNP-60042	MHAHK229-07	JF761019
8796	Phocides Warren01	Pyrginae	00-SRNP-15726	MHMXI704-07	JF761039
8797	Phocides belus	Pyrginae	06-SRNP-59670	MHAHK228-07	JF761004
8798	Phocides belus	Pyrginae	05-SRNP-66416	MHAHG341-06	GU151554
8799	Phocides belus	Pyrginae	05-SRNP-66414	MHAHG338-06	GU151552
8800	Phocides belus	Pyrginae	06-SRNP-55154	MHAHG348-06	GU151560
8801	Phocides belus	Pyrginae	96-SRNP-12435.1	MHMXI709-07	JF761013
8802	Phocides belus	Pyrginae	99-SRNP-8611	MHMXI718-07	JF761014
8803	Phocides belus	Pyrginae	06-SRNP-60276	MHAHK226-07	JF761002
8804	Phocides belus	Pyrginae	01-SRNP-18749	CSCR192-04	DQ292971
8805	Phocides belus	Pyrginae	94-SRNP-7489	MHMXI712-07	JF761007
8806	Phocides belus	Pyrginae	01-SRNP-17030	CSRII133-04	DQ292975
8807	Phocides belus	Pyrginae	05-SRNP-55231	MHAHD739-05	GU161786
8808	Phocides belus	Pyrginae	07-SRNP-55795	MHMXK149-07	JF762599
8809	Phocides belus	Pyrginae	07-SRNP-55340	MHMXK150-07	JF762598
8810	Phocides belus	Pyrginae	07-SRNP-55373	MHMXK152-07	JF762596
8811	Phocides belus	Pyrginae	00-SRNP-20234	CSRII130-04	DQ292973
8812	Phocides belus	Pyrginae	00-SRNP-20231	MHMXI716-07	JF761016
8813	Phocides belus	Pyrginae	96-SRNP-12432	MHMXI723-07	JF761005
8814	Phocides belus	Pyrginae	07-SRNP-55377	MHMXK153-07	JF762595
8815	Phocides belus	Pyrginae	96-SRNP-9221	MHMXI715-07	JF761017
8816	Phocides belus	Pyrginae	97-SRNP-248	MHMXI714-07	JF761018
8817	Phocides belus	Pyrginae	00-SRNP-3582	MHMXI713-07	JF761006
8818	Phocides belus	Pyrginae	93-SRNP-6630	MHMXI711-07	JF761009
8819	Phocides Warren01	Pyrginae	00-SRNP-15102	MHMXI707-07	JF761033
8820	Phocides Warren01	Pyrginae	00-SRNP-15185	MHMXI694-07	JF761038
8821	Phocides Warren01	Pyrginae	00-SRNP-15194	MHMXI692-07	JF761025
8822	Phocides Warren01	Pyrginae	00-SRNP-15236	MHMXI689-07	JF761020
8823	Phocides belus	Pyrginae	06-SRNP-55176	MHAHG347-06	GU151561
8824	Phocides belus	Pyrginae	06-SRNP-55418	MHAHG344-06	GU151559
8825	Phocides belus	Pyrginae	05-SRNP-55233	MHAHD740-05	GU161787
8826	Phocides belus	Pyrginae	05-SRNP-55235	MHAHD389-05	GU161785
8827	Phocides belus	Pyrginae	06-SRNP-55173	MHAHG343-06	GU151558
8828	Phocides belus	Pyrginae	05-SRNP-66417	MHAHG340-06	GU151555
8829	Phocides belus	Pyrginae	08-SRNP-55675	MHMXW003-09	JF754063
8830	Phocides belus	Pyrginae	09-SRNP-55421	MHMYC452-09	GU649884
8831	Phocides pigmalionDHJ02	Pyrginae	06-SRNP-34234	MHAHI517-06	GU156079
8832	Phocides pigmalionDHJ02	Pyrginae	06-SRNP-44090	MHAHJ618-07	JF753066
8833	Phocides pigmalionDHJ02	Pyrginae	08-SRNP-41365	MHMXW008-09	JF754076
8834	Phocides pigmalionDHJ02	Pyrginae	08-SRNP-41366	MHMXW009-09	JF754077
8835	Phocides pigmalionDHJ02	Pyrginae	07-SRNP-42096	MHMXO747-08	JF762619
8836	Phocides pigmalionDHJ02	Pyrginae	07-SRNP-42095	MHMXO759-08	JF762620
8837	Phocides pigmalionDHJ02	Pyrginae	07-SRNP-943	MHAHL419-07	JF762617
8838	Phocides pigmalionDHJ02	Pyrginae	07-SRNP-1304	MHAHL420-07	JF762618
8839	Phocides pigmalionDHJ02	Pyrginae	07-SRNP-1303	MHMXK148-07	JF762621
8840	Phocides pigmalionDHJ02	Pyrginae	06-SRNP-34235	MHAHJ911-07	JF753067
8841	Phocides pigmalionDHJ02	Pyrginae	06-SRNP-43725	MHAHJ614-07	JF753065
8842	Phocides pigmalionDHJ02	Pyrginae	06-SRNP-43751	MHAHJ518-07	JF753064
8843	Phocides pigmalionDHJ02	Pyrginae	06-SRNP-34232	MHAHI520-06	GU156080
8844	Phocides pigmalionDHJ02	Pyrginae	06-SRNP-34233	MHAHI519-06	GU156081
8845	Phocides pigmalionDHJ02	Pyrginae	06-SRNP-41789	MHAHI123-06	GU156082
8846	Phocides pigmalionDHJ02	Pyrginae	00-SRNP-11147	MHAHC360-05	DQ292990
8847	Phocides pigmalionDHJ02	Pyrginae	00-SRNP-12628	MHAHC359-05	DQ292989

Tree Order	Species	Subfamily	ACG Sampleid	BOLD Processid	Genbank Accession
8848	Phocides pigmalionDHJ02	Pyrginae	08-SRNP-66135	MHMXY1124-09	GU666416
8849	Phocides pigmalionDHJ02	Pyrginae	09-SRNP-21125	MHMYH110-10	HM887262
8850	Phocides Burns01	Pyrginae	05-SRNP-41960	MHAHF590-06	GU150683
8851	Phocides Burns01	Pyrginae	05-SRNP-2576	MHAHE365-05	GU149835
8852	Phocides Burns01	Pyrginae	04-SRNP-40653	CSRII467-04	DQ292976
8853	Phocides Burns01	Pyrginae	06-SRNP-42438	MHAHI518-06	GU156077
8854	Phocides Burns01	Pyrginae	06-SRNP-42881	MHAHJ616-07	JF753051
8855	Phocides lilea	Pyrginae	02-SRNP-4215	CSCR194-04	DQ292977
8856	Phocides lilea	Pyrginae	05-SRNP-36020	MHAHG150-06	GU151568
8857	Phocides lilea	Pyrginae	02-SRNP-4582	CSCR195-04	DQ292978
8858	Phocides lilea	Pyrginae	07-SRNP-45148	MHMXK143-07	JF762603
8859	Phocides lilea	Pyrginae	06-SRNP-55337	MHAHG153-06	GU151569
8860	Phocides lilea	Pyrginae	08-SRNP-45148	MHMXW080-09	JF754067
8861	Phocides lilea	Pyrginae	08-SRNP-55012	MHMXW081-09	JF754068
8862	Phocides lilea	Pyrginae	06-SRNP-36875	MHAHL426-07	JF762600
8863	Phocides lilea	Pyrginae	07-SRNP-1991	MHAHL427-07	JF762601
8864	Phocides lilea	Pyrginae	05-SRNP-45014	MHAHF853-06	GU150689
8865	Phocides lilea	Pyrginae	05-SRNP-45013	MHAHF852-06	GU150686
8866	Phocides lilea	Pyrginae	05-SRNP-45012	MHAHF851-06	GU150688
8867	Phocides lilea	Pyrginae	05-SRNP-55954	MHAHF850-06	GU150684
8868	Phocides lilea	Pyrginae	05-SRNP-31874	MHAHF849-06	GU150685
8869	Phocides lilea	Pyrginae	05-SRNP-42094	MHAHF848-06	GU150687
8870	Phocides lilea	Pyrginae	04-SRNP-48827	MHAHD815-05	GU161788
8871	Phocides lilea	Pyrginae	04-SRNP-22905	MHAHD814-05	GU161790
8872	Phocides lilea	Pyrginae	04-SRNP-49744	MHAHD813-05	GU161789
8873	Phocides lilea	Pyrginae	06-SRNP-55641	MHAHG152-06	GU151570
8874	Phocides lilea	Pyrginae	05-SRNP-64067	MHAHG693-06	GU151565
8875	Phocides lilea	Pyrginae	06-SRNP-12035	MHAHG694-06	GU151564
8876	Phocides lilea	Pyrginae	05-SRNP-64081	MHAHG695-06	GU151563
8877	Phocides lilea	Pyrginae	05-SRNP-64071	MHAHG696-06	GU151562
8878	Phocides lilea	Pyrginae	06-SRNP-12616	MHAHG699-06	GU151566
8879	Phocides lilea	Pyrginae	05-SRNP-64077	MHAHG700-06	GU151567
8880	Phocides lilea	Pyrginae	07-SRNP-56871	MHMXO775-08	JF762602
8881	Phocides lilea	Pyrginae	08-SRNP-1660	MHMXW077-09	JF754064
8882	Phocides lilea	Pyrginae	08-SRNP-70870	MHMXW078-09	JF754065
8883	Phocides lilea	Pyrginae	07-SRNP-43071	MHMXW079-09	JF754066
8884	Phocides lilea	Pyrginae	08-SRNP-45036	MHMXW082-09	JF754069
8885	Phocides lilea	Pyrginae	08-SRNP-72107	MHMXX669-09	JF778385
8886	Phocides lilea	Pyrginae	08-SRNP-71270	MHMXX670-09	JF778386
8887	Phocides lilea	Pyrginae	08-SRNP-71658	MHMXX671-09	JF778387
8888	Phocides lilea	Pyrginae	08-SRNP-72658	MHMXY1122-09	GU666421
8889	Phocides lilea	Pyrginae	08-SRNP-37041	MHMXY1123-09	GU666422
8890	Phocides nigrescens	Pyrginae	02-SRNP-24513	CSCR198-04	DQ292979
8891	Phocides nigrescens	Pyrginae	03-SRNP-1248	CSCR394-04	DQ292980
8892	Phocides nigrescens	Pyrginae	09-SRNP-20813	MHMYH108-10	HM887260
8893	Phocides nigrescens	Pyrginae	06-SRNP-6937	MHAHJ611-07	JF753054
8894	Phocides nigrescens	Pyrginae	06-SRNP-9777	MHMXP223-08	JF762611
8895	Phocides nigrescens	Pyrginae	07-SRNP-60225	MHMXR635-08	JF762608
8896	Phocides nigrescens	Pyrginae	08-SRNP-30726	MHMXW005-09	JF754070
8897	Phocides nigrescens	Pyrginae	08-SRNP-30757	MHMXW006-09	JF754071
8898	Phocides nigrescens	Pyrginae	07-SRNP-1654	MHAHL414-07	JF762604
8899	Phocides nigrescens	Pyrginae	07-SRNP-1584	MHAHL415-07	JF762605
8900	Phocides nigrescens	Pyrginae	07-SRNP-1585	MHAHL421-07	JF762606
8901	Phocides nigrescens	Pyrginae	07-SRNP-1599	MHAHL422-07	JF762607
8902	Phocides nigrescens	Pyrginae	07-SRNP-3394	MHMXO743-08	JF762610
8903	Phocides nigrescens	Pyrginae	07-SRNP-45870	MHMXO757-08	JF762609

Tree Order	Species	Subfamily	ACG Sampleid	BOLD Processid	Genbank Accession
8904	Phocides nigrescens	Pyrginae	08-SRNP-1566	MHMXW011-09	JF754072
8905	Phocides nigrescens	Pyrginae	08-SRNP-30756	MHMXW012-09	JF754073
8906	Phocides nigrescens	Pyrginae	08-SRNP-2469	MHMXX661-09	JF778388
8907	Phocides nigrescens	Pyrginae	08-SRNP-23739	MHMYB186-09	GU649661
8908	Phocides nigrescens	Pyrginae	09-SRNP-20870	MHMYH106-10	HM887258
8909	Phocides nigrescens	Pyrginae	09-SRNP-80647	MHMYH107-10	HM887259
8910	Phocides nigrescens	Pyrginae	08-SRNP-33055	MHMYC448-09	GU649888
8911	Phocides nigrescens	Pyrginae	08-SRNP-6886	MHMYC449-09	GU649889
8912	Phocides nigrescens	Pyrginae	06-SRNP-4789	MHAHJ912-07	JF753063
8913	Phocides nigrescens	Pyrginae	07-SRNP-1656	MHMXK154-07	JF762612
8914	Phocides nigrescens	Pyrginae	06-SRNP-4244	MHAHJ793-07	JF753060
8915	Phocides nigrescens	Pyrginae	06-SRNP-6938	MHAHJ909-07	JF753062
8916	Phocides nigrescens	Pyrginae	06-SRNP-7174	MHAHJ615-07	JF753057
8917	Phocides nigrescens	Pyrginae	06-SRNP-6010	MHAHJ792-07	JF753059
8918	Phocides nigrescens	Pyrginae	06-SRNP-6017	MHAHJ612-07	JF753055
8919	Phocides nigrescens	Pyrginae	06-SRNP-6172	MHAHJ613-07	JF753056
8920	Phocides nigrescens	Pyrginae	06-SRNP-6612	MHAHJ516-07	JF753052
8921	Phocides nigrescens	Pyrginae	06-SRNP-23407	MHAHJ517-07	JF753053
8922	Phocides nigrescens	Pyrginae	06-SRNP-4060	MHAHI122-06	GU156078
8923	Phocides nigrescens	Pyrginae	05-SRNP-7497	MHAHG684-06	GU151571
8924	Phocides nigrescens	Pyrginae	06-SRNP-2402	MHAHG683-06	GU151572
8925	Phocides nigrescens	Pyrginae	05-SRNP-7669	MHAHF589-06	GU150690
8926	Phocides nigrescens	Pyrginae	05-SRNP-31898	MHAHE364-05	GU149839
8927	Phocides nigrescens	Pyrginae	05-SRNP-21431	MHAHE363-05	GU149838
8928	Phocides nigrescens	Pyrginae	05-SRNP-3478	MHAHE362-05	GU149836
8929	Phocides nigrescens	Pyrginae	05-SRNP-2553	MHAHE361-05	GU149837
8930	Phocides nigrescens	Pyrginae	04-SRNP-35496	MHAHD736-05	GU161795
8931	Phocides nigrescens	Pyrginae	04-SRNP-3410	MHAHD735-05	GU161791
8932	Phocides nigrescens	Pyrginae	04-SRNP-23796	MHAHD734-05	GU161792
8933	Phocides nigrescens	Pyrginae	04-SRNP-4780	MHAHD733-05	GU161793
8934	Phocides nigrescens	Pyrginae	04-SRNP-35586	MHAHD732-05	GU161794
8935	Phocides nigrescens	Pyrginae	06-SRNP-6936	MHAHJ617-07	JF753058
8936	Phocides nigrescens	Pyrginae	06-SRNP-9333	MHAHJ794-07	JF753061
8937	Phocides nigrescens	Pyrginae	09-SRNP-21126	MHMYH109-10	HM887261
8938	Nascus phintias	Pyrginae	05-SRNP-2410	MHAHF556-06	GU150591
8939	Nascus phintias	Pyrginae	07-SRNP-2575	MHMXP145-08	JF762417
8940	Nascus phintias	Pyrginae	02-SRNP-3373	CSCR147-04	DQ292713
8941	Nascus phintias	Pyrginae	05-SRNP-2034	MHAHF207-06	GU150592
8942	Nascus phintias	Pyrginae	05-SRNP-43646	MHAHL112-07	JF762415
8943	Nascus phintias	Pyrginae	07-SRNP-35296	MHMXK069-07	JF762418
8944	Nascus phintias	Pyrginae	05-SRNP-47456	MHAHF560-06	GU150587
8945	Nascus phintias	Pyrginae	05-SRNP-3879	MHAHF559-06	GU150588
8946	Nascus phintias	Pyrginae	05-SRNP-40848	MHAHF558-06	GU150589
8947	Nascus phintias	Pyrginae	05-SRNP-2532	MHAHF557-06	GU150590
8948	Nascus phintias	Pyrginae	05-SRNP-2535	MHAHF203-06	GU150593
8949	Nascus phintias	Pyrginae	04-SRNP-2156	MHAHC646-05	DQ292715
8950	Nascus phintias	Pyrginae	02-SRNP-3469	CSRII530-04	DQ292714
8951	Nascus phintias	Pyrginae	07-SRNP-36387	MHMXR753-08	JF762416
8952	Nascus phintias	Pyrginae	08-SRNP-31018	MHMXX961-09	JF778118
8953	Nascus Burns01	Pyrginae	04-SRNP-60540	MHAHC612-05	DQ292683
8954	Nascus Burns01	Pyrginae	02-SRNP-135	CSCR151-04	DQ292679
8955	Nascus Burns01	Pyrginae	00-SRNP-1575	CSCR150-04	DQ292678
8956	Nascus Burns01	Pyrginae	04-SRNP-14201	MHAHC643-05	DQ292684
8957	Nascus Burns01	Pyrginae	08-SRNP-14631	MHMXY1127-09	HM390684
8958	Nascus Burns01	Pyrginae	05-SRNP-47006	MHAHF212-06	GU150581
8959	Nascus Burns01	Pyrginae	05-SRNP-47054	MHAHF197-06	GU150582

Tree Order	Species	Subfamily	ACG Sampleid	BOLD Processid	Genbank Accession
8960	Nascus Burns01	Pyrginae	05-SRNP-47007	MHAHF193-06	GU150580
8961	Nascus Burns01	Pyrginae	98-SRNP-15119	CSRII460-04	DQ292681
8962	Nascus Burns01	Pyrginae	07-SRNP-23590	MHMXT144-08	JF762412
8963	Nascus Burns01	Pyrginae	04-SRNP-2064	MHAHC647-05	DQ292685
8964	Nascus Burns01	Pyrginae	96-SRNP-12541	CSRII459-04	DQ292680
8965	Nascus Burns01	Pyrginae	98-SRNP-15121	CSRII461-04	DQ292682
8966	Nascus Burns01	Pyrginae	06-SRNP-47760	MHAHK219-07	JF760887
8967	Nascus Burns01	Pyrginae	08-SRNP-6958	MHMYC534-09	GU649810
8968	Nascus Burns01	Pyrginae	08-SRNP-6946	MHMYC535-09	GU649811
8969	Nascus Burns02	Pyrginae	07-SRNP-45185	MHMXK068-07	JF762414
8970	Nascus Burns02	Pyrginae	02-SRNP-5385	CSRII457-04	DQ292688
8971	Nascus Burns02	Pyrginae	96-SRNP-7368	CSRII455-04	DQ292686
8972	Nascus Burns02	Pyrginae	02-SRNP-2085	CSRII456-04	DQ292687
8973	Nascus Burns02	Pyrginae	02-SRNP-5744	CSRII458-04	DQ292689
8974	Nascus Burns02	Pyrginae	04-SRNP-45037	MHAHC620-05	DQ292690
8975	Nascus Burns02	Pyrginae	04-SRNP-48523	MHAHC626-05	DQ292691
8976	Nascus Burns02	Pyrginae	04-SRNP-23315	MHAHC627-05	DQ292692
8977	Nascus Burns02	Pyrginae	04-SRNP-21643	MHAHC630-05	DQ292693
8978	Nascus Burns02	Pyrginae	04-SRNP-21927	MHAHC633-05	DQ292694
8979	Nascus Burns02	Pyrginae	04-SRNP-47932	MHAHC636-05	DQ292695
8980	Nascus Burns02	Pyrginae	04-SRNP-47541	MHAHC637-05	DQ292696
8981	Nascus Burns02	Pyrginae	04-SRNP-48314	MHAHC638-05	DQ292697
8982	Nascus Burns02	Pyrginae	04-SRNP-48044	MHAHC639-05	DQ292698
8983	Nascus Burns02	Pyrginae	04-SRNP-48045	MHAHC641-05	DQ292699
8984	Nascus Burns02	Pyrginae	04-SRNP-47542	MHAHC644-05	DQ292700
8985	Nascus Burns02	Pyrginae	04-SRNP-11980	MHAHD884-05	GU161687
8986	Nascus Burns02	Pyrginae	06-SRNP-21092	MHAHH530-06	GU155435
8987	Nascus Burns02	Pyrginae	06-SRNP-3613	MHAHI121-06	GU156007
8988	Nascus Burns02	Pyrginae	06-SRNP-58211	MHAHJ520-07	JF752938
8989	Nascus Burns02	Pyrginae	06-SRNP-59675	MHAHK215-07	JF760888
8990	Nascus Burns02	Pyrginae	06-SRNP-47830	MHAHK216-07	JF760889
8991	Nascus Burns02	Pyrginae	06-SRNP-59883	MHAHK218-07	JF760890
8992	Nascus Burns02	Pyrginae	06-SRNP-59658	MHAHK220-07	JF760891
8993	Nascus Burns02	Pyrginae	07-SRNP-60000	MHMXP146-08	JF762413
8994	Nascus Burns02	Pyrginae	08-SRNP-1817	MHMXX962-09	JF778114
8995	Nascus Burns02	Pyrginae	07-SRNP-65044	MHMXX141-09	JF778113
8996	Nascus Burns02	Pyrginae	08-SRNP-22926	MHMXY1128-09	GU666419
8997	Nascus Burns02	Pyrginae	09-SRNP-44171	MHMYE1520-09	GU653486
8998	Nascus broteas	Pyrginae	07-SRNP-58744	MHMXP144-08	JF762408
8999	Nascus broteas	Pyrginae	07-SRNP-65708	MHMXT179-08	JF762404
9000	Nascus broteas	Pyrginae	07-SRNP-45315	MHMXP143-08	JF762409
9001	Nascus broteas	Pyrginae	07-SRNP-45363	MHMXP142-08	JF762410
9002	Nascus broteas	Pyrginae	07-SRNP-65709	MHMXT143-08	JF762405
9003	Nascus broteas	Pyrginae	07-SRNP-20402	MHAHL115-07	JF762402
9004	Nascus broteas	Pyrginae	07-SRNP-65689	MHMXT142-08	JF762406
9005	Nascus broteas	Pyrginae	05-SRNP-47047	MHAHF195-06	GU150578
9006	Nascus broteas	Pyrginae	08-SRNP-66188	MHMYC533-09	GU649809
9007	Nascus broteas	Pyrginae	09-SRNP-57232	MHMYE1522-09	HM391082
9008	Nascus broteas	Pyrginae	08-SRNP-66059	MHMXY1132-09	GU666414
9009	Nascus broteas	Pyrginae	08-SRNP-66039	MHMXY1133-09	GU666415
9010	Nascus broteas	Pyrginae	08-SRNP-22159	MHMXX674-09	JF778112
9011	Nascus broteas	Pyrginae	08-SRNP-32159	MHMXY1129-09	HM390685
9012	Nascus broteas	Pyrginae	07-SRNP-58175	MHMXP147-08	JF762407
9013	Nascus broteas	Pyrginae	07-SRNP-35984	MHMXP224-08	JF762411
9014	Nascus broteas	Pyrginae	09-SRNP-20230	MHMYB187-09	GU649662
9015	Nascus broteas	Pyrginae	08-SRNP-6161	MHMYC532-09	GU649808

Tree Order	Species	Subfamily	ACG Sampleid	BOLD Processid	Genbank Accession
9016	Nascus broteas	Pyrginae	08-SRNP-66029	MHMXY1130-09	GU666412
9017	Nascus broteas	Pyrginae	08-SRNP-66038	MHMXY1131-09	GU666413
9018	Nascus broteas	Pyrginae	07-SRNP-32448	MHAHL113-07	JF762401
9019	Nascus broteas	Pyrginae	07-SRNP-36061	MHAHL116-07	JF762403
9020	Nascus broteas	Pyrginae	05-SRNP-2095	MHAHF215-06	GU150574
9021	Nascus broteas	Pyrginae	05-SRNP-5955	MHAHF214-06	GU150571
9022	Nascus broteas	Pyrginae	05-SRNP-2780	MHAHF210-06	GU150577
9023	Nascus broteas	Pyrginae	05-SRNP-3276	MHAHF209-06	GU150575
9024	Nascus broteas	Pyrginae	05-SRNP-887	MHAHF206-06	GU150573
9025	Nascus broteas	Pyrginae	05-SRNP-47048	MHAHF196-06	GU150576
9026	Nascus broteas	Pyrginae	04-SRNP-46661	MHAHD886-05	GU161686
9027	Nascus broteas	Pyrginae	03-SRNP-37108	CSRII528-04	DQ292677
9028	Nascus broteas	Pyrginae	01-SRNP-7365	CSCR143-04	DQ292675
9029	Nascus broteas	Pyrginae	02-SRNP-9577	CSCR383-04	DQ292676
9030	Nascus broteas	Pyrginae	05-SRNP-47061	MHAHF213-06	GU150579
9031	Nascus broteas	Pyrginae	05-SRNP-30597	MHAHF217-06	GU150572
9032	Nascus broteas	Pyrginae	06-SRNP-1723	MHAHG708-06	GU151484
9033	Nascus broteas	Pyrginae	09-SRNP-80142	MHMYG2463-10	HM885890
9034	Nascus solon	Pyrginae	94-SRNP-4830	CSCR152-04	DQ292716
9035	Nascus solon	Pyrginae	05-SRNP-55806	MHAHF188-06	GU150600
9036	Nascus solon	Pyrginae	04-SRNP-50064	MHAHF216-06	GU150596
9037	Nascus solon	Pyrginae	05-SRNP-46699	MHAHF211-06	GU150595
9038	Nascus solon	Pyrginae	05-SRNP-705	MHAHF208-06	GU150597
9039	Nascus solon	Pyrginae	05-SRNP-58399	MHAHF202-06	GU150598
9040	Nascus solon	Pyrginae	05-SRNP-2536	MHAHF201-06	GU150594
9041	Nascus solon	Pyrginae	05-SRNP-46952	MHAHF199-06	GU150601
9042	Nascus solon	Pyrginae	05-SRNP-30650	MHAHF187-06	GU150599
9043	Nascus solon	Pyrginae	04-SRNP-49682	MHAHC640-05	DQ292729
9044	Nascus solon	Pyrginae	04-SRNP-45029	MHAHC632-05	DQ292728
9045	Nascus solon	Pyrginae	04-SRNP-35293	MHAHC631-05	DQ292727
9046	Nascus solon	Pyrginae	04-SRNP-49716	MHAHC628-05	DQ292726
9047	Nascus solon	Pyrginae	04-SRNP-49717	MHAHC624-05	DQ292725
9048	Nascus solon	Pyrginae	04-SRNP-49681	MHAHC623-05	DQ292724
9049	Nascus solon	Pyrginae	04-SRNP-49777	MHAHC621-05	DQ292723
9050	Nascus solon	Pyrginae	04-SRNP-49768	MHAHC618-05	DQ292721
9051	Nascus solon	Pyrginae	04-SRNP-49335	MHAHC617-05	DQ292720
9052	Nascus solon	Pyrginae	04-SRNP-49680	MHAHC616-05	DQ292719
9053	Nascus solon	Pyrginae	02-SRNP-168	CSRII531-04	DQ292718
9054	Nascus solon	Pyrginae	01-SRNP-597	CSCR153-04	DQ292717
9055	Nascus solon	Pyrginae	04-SRNP-49336	MHAHD887-05	GU161688
9056	Nascus solon	Pyrginae	04-SRNP-49775	MHAHC619-05	DQ292722
9057	Nascus solon	Pyrginae	06-SRNP-59999	MHAHK217-07	JF760892
9058	Nascus solon	Pyrginae	05-SRNP-25375	MHAHL110-07	JF762419
9059	Nascus solon	Pyrginae	05-SRNP-47450	MHAHL111-07	JF762420
9060	Nascus solon	Pyrginae	07-SRNP-21820	MHAHL114-07	JF762421
9061	Nascus solon	Pyrginae	07-SRNP-45627	MHMXR830-08	JF762422
9062	Nascus solon	Pyrginae	08-SRNP-55518	MHMXX963-09	JF778119
9063	Nascus solon	Pyrginae	08-SRNP-21263	MHMXX965-09	JF778120
9064	Nascus solon	Pyrginae	07-SRNP-61336	MHMXX966-09	JF778121
9065	Nascus solon	Pyrginae	08-SRNP-21932	MHMXX967-09	JF778122
9066	Nascus solon	Pyrginae	09-SRNP-57395	MHMYG2464-10	HM885891
9067	Drephalys alcmon	Pyrginae	01-SRNP-9223	MHAHJ276-07	JF752617
9068	Drephalys alcmon	Pyrginae	99-SRNP-2617	CSRII113-04	DQ292287
9069	Drephalys alcmon	Pyrginae	97-SRNP-4047	MHAHJ273-07	JF752614
9070	Drephalys alcmon	Pyrginae	99-SRNP-2188	MHAHJ272-07	JF752613
9071	Drephalys alcmon	Pyrginae	01-SRNP-9331	CSRII112-04	DQ292286

Tree Order	Species	Subfamily	ACG Sampleid	BOLD Processid	Genbank Accession
9072	Drephalys alcmon	Pyrginae	97-SRNP-4150	MHAHJ274-07	JF752615
9073	Drephalys alcmon	Pyrginae	02-SRNP-29274	MHAHJ277-07	JF752618
9074	Drephalys alcmon	Pyrginae	02-SRNP-4202	MHAHJ278-07	JF752619
9075	Drephalys alcmon	Pyrginae	99-SRNP-2646	MHAHJ275-07	JF752616
9076	Drephalys alcmon	Pyrginae	98-SRNP-4184	MHAHJ279-07	JF752620
9077	Drephalys alcmon	Pyrginae	00-SRNP-2692	MHAHJ281-07	JF752622
9078	Drephalys Burns01	Pyrginae	05-SRNP-70289	MHAHG144-06	GU151380
9079	Drephalys Burns01	Pyrginae	06-SRNP-5699	MHAHJ820-07	JF752623
9080	Drephalys Burns01	Pyrginae	07-SRNP-42336	MHMXP163-08	JF762089
9081	Drephalys Burns01	Pyrginae	06-SRNP-42502	MHAHI601-06	GU155931
9082	Drephalys Burns01	Pyrginae	06-SRNP-42807	MHAHI566-06	GU155930
9083	Drephalys Burns01	Pyrginae	05-SRNP-31284	MHAHF595-06	GU150383
9084	Drephalys Burns01	Pyrginae	05-SRNP-43718	MHAHF594-06	GU150382
9085	Drephalys Burns01	Pyrginae	05-SRNP-34501	MHAHF593-06	GU150384
9086	Drephalys Burns01	Pyrginae	05-SRNP-41556	MHAHE612-06	GU149635
9087	Drephalys Burns01	Pyrginae	05-SRNP-41680	MHAHE611-06	GU149636
9088	Drephalys Burns01	Pyrginae	05-SRNP-41447	MHAHE360-05	GU149637
9089	Drephalys Burns01	Pyrginae	05-SRNP-41557	MHAHE610-06	GU149638
9090	Drephalys Burns01	Pyrginae	03-SRNP-21317	CSRII194-04	DQ292288
9091	Drephalys Burns01	Pyrginae	06-SRNP-30793	MHAHG145-06	GU151381
9092	Drephalys Burns01	Pyrginae	06-SRNP-42501	MHAHI602-06	GU155932
9093	Drephalys Burns01	Pyrginae	07-SRNP-41814	MHAHL191-07	JF762087
9094	Drephalys Burns01	Pyrginae	08-SRNP-345	MHMXT148-08	JF762088
9095	Drephalys Burns01	Pyrginae	08-SRNP-41486	MHMXX465-09	JF777861
9096	Drephalys Burns01	Pyrginae	08-SRNP-71839	MHMXX466-09	JF777862
9097	Drephalys kidonoi	Pyrginae	06-SRNP-60166	MHAHK223-07	JF760661
9098	Drephalys kidonoi	Pyrginae	02-SRNP-12965	MHAHI349-06	GU155942
9099	Drephalys kidonoi	Pyrginae	02-SRNP-12979	MHAHI342-06	GU155935
9100	Drephalys kidonoi	Pyrginae	02-SRNP-13368	MHAHI341-06	GU155934
9101	Drephalys kidonoi	Pyrginae	02-SRNP-10261	MHAHI345-06	GU155938
9102	Drephalys kidonoi	Pyrginae	02-SRNP-13429	MHAHI350-06	GU155943
9103	Drephalys kidonoi	Pyrginae	02-SRNP-12978	MHAHI348-06	GU155941
9104	Drephalys kidonoi	Pyrginae	02-SRNP-10329	MHAHI347-06	GU155940
9105	Drephalys kidonoi	Pyrginae	02-SRNP-10250	MHAHI346-06	GU155939
9106	Drephalys kidonoi	Pyrginae	02-SRNP-13369	MHAHI344-06	GU155937
9107	Drephalys kidonoi	Pyrginae	02-SRNP-10252	MHAHI343-06	GU155936
9108	Drephalys kidonoi	Pyrginae	02-SRNP-13103	MHAHI340-06	GU155933
9109	Drephalys kidonoi	Pyrginae	01-SRNP-12341	CSRII114-04	DQ292289
9110	Drephalys kidonoi	Pyrginae	02-SRNP-13370	CSRII115-04	DQ292290
9111	Drephalys kidonoi	Pyrginae	06-SRNP-60079	MHAHK221-07	JF760660
9112	Drephalys kidonoi	Pyrginae	07-SRNP-55278	MHMXK132-07	JF762090
9113	Drephalys kidonoi	Pyrginae	09-SRNP-55224	MHMYE896-09	GU653706
9114	Drephalys kidonoi	Pyrginae	09-SRNP-55518	MHMYE1468-09	GU653534
9115	Cogia hiska	Pyrginae	03-SRNP-250	CSCR353-04	DQ292219
9116	Cogia hiska	Pyrginae	03-SRNP-101	CSCR354-04	DQ292220
9117	Cogia hiska	Pyrginae	06-SRNP-12193	MHAHG671-06	GU151318
9118	Cogia hiska	Pyrginae	03-SRNP-18	MHAHK520-07	JF760589
9119	Cogia hiska	Pyrginae	03-SRNP-14096	MHAHK521-07	JF760590
9120	Cogia hiska	Pyrginae	03-SRNP-13714	MHAHK522-07	JF760591
9121	Cogia hiska	Pyrginae	02-SRNP-12905	MHAHK523-07	JF760592
9122	Cogia hiska	Pyrginae	02-SRNP-13343	MHAHK524-07	JF760593
9123	Cogia hiska	Pyrginae	02-SRNP-13341	MHAHK525-07	JF760594
9124	Cogia hiska	Pyrginae	02-SRNP-13338	MHAHK526-07	JF760595
9125	Cogia hiska	Pyrginae	02-SRNP-13244	MHAHK527-07	JF760596
9126	Cogia hiska	Pyrginae	02-SRNP-12906	MHAHK528-07	JF760597
9127	Cogia hiska	Pyrginae	02-SRNP-13604	MHAHK529-07	JF760598

Tree Order	Species	Subfamily	ACG Sampleid	BOLD Processid	Genbank Accession
9128	Cogia hiska	Pyrginae	02-SRNP-13580	MHAHK530-07	JF760599
9129	Cogia hiska	Pyrginae	02-SRNP-13357	MHAHK531-07	JF760600
9130	Cogia hiska	Pyrginae	02-SRNP-13348	MHAHK532-07	JF760601
9131	Cogia hiska	Pyrginae	02-SRNP-13346	MHAHK533-07	JF760602
9132	Typhedanus undulatus	Pyrginae	05-SRNP-5907	MHAHF352-06	GU150942
9133	Typhedanus undulatus	Pyrginae	05-SRNP-7507	MHAHF596-06	GU150941
9134	Typhedanus undulatus	Pyrginae	05-SRNP-5906	MHAHF597-06	GU150940
9135	Typhedanus undulatus	Pyrginae	99-SRNP-12788	CSCR271-04	DQ293623
9136	Typhedanus undulatus	Pyrginae	98-SRNP-6246	CSCR270-04	DQ293622
9137	Typhedanus undulatus	Pyrginae	04-SRNP-24403	MHAHD874-05	GU161914
9138	Typhedanus undulatus	Pyrginae	06-SRNP-21153	MHAHH556-06	GU155677
9139	Typhedanus undulatus	Pyrginae	07-SRNP-41835	MHAHL464-07	JF763279
9140	Typhedanus undulatus	Pyrginae	07-SRNP-3071	MHMXO824-08	JF763281
9141	Typhedanus undulatus	Pyrginae	07-SRNP-23877	MHMXR828-08	JF763280
9142	Cogia calchasDHJ01	Pyrginae	05-SRNP-12001	MHAHE078-05	GU149554
9143	Cogia calchasDHJ01	Pyrginae	04-SRNP-49737	MHAHL101-07	JF761975
9144	Cogia calchasDHJ01	Pyrginae	07-SRNP-55314	MHMXK056-07	JF761977
9145	Cogia calchasDHJ01	Pyrginae	98-SRNP-6237	MHAHK536-07	JF760580
9146	Cogia calchasDHJ01	Pyrginae	93-SRNP-6683	MHAHK535-07	JF760579
9147	Cogia calchasDHJ01	Pyrginae	06-SRNP-13361	MHAHH471-06	GU155337
9148	Cogia calchasDHJ01	Pyrginae	04-SRNP-48664	MHAHE077-05	GU149553
9149	Cogia calchasDHJ01	Pyrginae	97-SRNP-379	CSCR076-04	DQ292214
9150	Cogia calchasDHJ01	Pyrginae	07-SRNP-45272	MHMXP125-08	JF761976
9151	Cogia calchasDHJ01	Pyrginae	03-SRNP-31305	MHAHK539-07	JF760583
9152	Cogia calchasDHJ01	Pyrginae	98-SRNP-6173	MHAHK538-07	JF760582
9153	Cogia calchasDHJ01	Pyrginae	98-SRNP-6178	MHAHK537-07	JF760581
9154	Cogia calchasDHJ01	Pyrginae	06-SRNP-13417	MHAHH472-06	GU155338
9155	Cogia calchasDHJ01	Pyrginae	05-SRNP-31856	MHAHF864-06	GU150340
9156	Cogia calchasDHJ01	Pyrginae	03-SRNP-6772	MHAHK540-07	JF760584
9157	Cogia calchasDHJ01	Pyrginae	97-SRNP-296	MHAHK541-07	JF760585
9158	Cogia calchasDHJ01	Pyrginae	98-SRNP-6163	MHAHK542-07	JF760586
9159	Cogia calchasDHJ01	Pyrginae	08-SRNP-2425	MHMXX978-09	JF777792
9160	Cogia calchasDHJ01	Pyrginae	08-SRNP-2467	MHMXX979-09	JF777793
9161	Cogia calchasDHJ01	Pyrginae	09-SRNP-80678	MHMYG1990-10	JF751926
9162	Cogia calchasDHJ02	Pyrginae	05-SRNP-32463	MHAHF865-06	GU150341
9163	Cogia calchasDHJ02	Pyrginae	04-SRNP-55362	MHAHE076-05	GU149555
9164	Cogia calchasDHJ02	Pyrginae	05-SRNP-32462	MHAHF866-06	GU150342
9165	Cogia calchasDHJ02	Pyrginae	00-SRNP-21991	MHAHK534-07	JF760587
9166	Cogia calchasDHJ02	Pyrginae	09-SRNP-73335	MHMYG2447-10	HM885872
9167	Cogia calchasDHJ02	Pyrginae	09-SRNP-73332	MHMYG2448-10	HM885873
9168	Cogia calchasDHJ02	Pyrginae	09-SRNP-80699	MHMYG1991-10	JF751927
9169	Udranomia orcinus	Pyrginae	04-SRNP-41230	MHAHD339-05	GU161929
9170	Udranomia orcinus	Pyrginae	05-SRNP-2555	MHAHF318-06	GU150956
9171	Udranomia orcinus	Pyrginae	04-SRNP-30933	MHAHD340-05	GU161927
9172	Udranomia orcinus	Pyrginae	04-SRNP-31229	MHAHD341-05	GU161926
9173	Udranomia orcinus	Pyrginae	03-SRNP-5582	CSCR438-04	DQ293629
9174	Udranomia orcinus	Pyrginae	05-SRNP-1457	MHAHF317-06	GU150952
9175	Udranomia orcinus	Pyrginae	07-SRNP-40320	MHAHK376-07	JF761259
9176	Udranomia orcinus	Pyrginae	08-SRNP-1673	MHMXW405-09	JF754347
9177	Udranomia orcinus	Pyrginae	05-SRNP-21131	MHAHF314-06	GU150953
9178	Udranomia orcinus	Pyrginae	08-SRNP-22893	MHMXX541-09	JF778575
9179	Udranomia orcinus	Pyrginae	08-SRNP-41217	MHMXX536-09	JF778574
9180	Udranomia orcinus	Pyrginae	07-SRNP-20955	MHMXX150-09	GU666370
9181	Udranomia orcinus	Pyrginae	08-SRNP-31332	MHMXW419-09	JF754353
9182	Udranomia orcinus	Pyrginae	08-SRNP-40514	MHMXW413-09	JF754351
9183	Udranomia orcinus	Pyrginae	08-SRNP-1672	MHMXW410-09	JF754350

Tree Order	Species	Subfamily	ACG Sampleid	BOLD Processid	Genbank Accession
9184	Udranomia orcinus	Pyrginae	08-SRNP-20817	MHMXW409-09	JF754349
9185	Udranomia orcinus	Pyrginae	08-SRNP-20850	MHMXW408-09	JF754348
9186	Udranomia orcinus	Pyrginae	08-SRNP-818	MHMXW404-09	JF754346
9187	Udranomia orcinus	Pyrginae	08-SRNP-30988	MHMXW401-09	JF754345
9188	Udranomia orcinus	Pyrginae	07-SRNP-65382	MHMXR933-08	JF763295
9189	Udranomia orcinus	Pyrginae	07-SRNP-40260	MHAHK369-07	JF761258
9190	Udranomia orcinus	Pyrginae	07-SRNP-1287	MHAHK368-07	JF761257
9191	Udranomia orcinus	Pyrginae	06-SRNP-23065	MHAHJ459-07	JF753212
9192	Udranomia orcinus	Pyrginae	04-SRNP-31004	MHAHD342-05	GU161925
9193	Udranomia orcinus	Pyrginae	04-SRNP-42858	MHAHD323-05	GU161928
9194	Udranomia orcinus	Pyrginae	07-SRNP-40862	MHMXN429-07	JF763296
9195	Udranomia orcinus	Pyrginae	06-SRNP-41751	MHAHH797-06	GU155696
9196	Udranomia orcinus	Pyrginae	05-SRNP-31979	MHAHF316-06	GU150954
9197	Udranomia orcinus	Pyrginae	03-SRNP-10433	CSCR437-04	DQ293628
9198	Udranomia orcinus	Pyrginae	05-SRNP-8128	MHAHI606-06	GU156379
9199	Udranomia orcinus	Pyrginae	05-SRNP-41043	MHAHF315-06	GU150955
9200	Udranomia orcinus	Pyrginae	05-SRNP-8135	MHAHG349-06	GU151801
9201	Udranomia orcinus	Pyrginae	07-SRNP-24023	MHMXR934-08	JF763294
9202	Udranomia orcinus	Pyrginae	08-SRNP-70812	MHMXW418-09	JF754352
9203	Udranomia orcinus	Pyrginae	08-SRNP-66180	MHMXY982-09	GU666539
9204	Udranomia orcinus	Pyrginae	09-SRNP-67117	MHMYC509-09	GU649828
9205	Udranomia orcinus	Pyrginae	09-SRNP-69354	MHMYE1564-09	HM391124
9206	Udranomia orcinus	Pyrginae	09-SRNP-80703	MHMYH125-10	HM887277
9207	Udranomia eurus	Pyrginae	03-SRNP-11250	CSCR433-04	DQ293624
9208	Udranomia kikkawaiDHJ03	Pyrginae	08-SRNP-21971	MHMXX538-09	JF778572
9209	Udranomia kikkawaiDHJ03	Pyrginae	08-SRNP-21969	MHMXX540-09	JF778573
9210	Udranomia kikkawaiDHJ03	Pyrginae	96-SRNP-1042	MHAHG858-06	GU151800
9211	Udranomia kikkawaiDHJ03	Pyrginae	06-SRNP-55723	MHAHH795-06	GU155695
9212	Udranomia kikkawaiDHJ03	Pyrginae	07-SRNP-55337	MHAHK379-07	JF761253
9213	Udranomia kikkawaiDHJ03	Pyrginae	07-SRNP-55336	MHAHK377-07	JF761252
9214	Udranomia kikkawaiDHJ03	Pyrginae	08-SRNP-21281	MHMXW396-09	JF754341
9215	Udranomia kikkawaiDHJ03	Pyrginae	07-SRNP-56346	MHAHK394-07	JF761256
9216	Udranomia kikkawaiDHJ03	Pyrginae	07-SRNP-56440	MHAHK392-07	JF761255
9217	Udranomia kikkawaiDHJ03	Pyrginae	07-SRNP-56118	MHAHK372-07	JF761250
9218	Udranomia kikkawaiDHJ03	Pyrginae	07-SRNP-55138	MHMXH873-07	JF761249
9219	Udranomia kikkawaiDHJ03	Pyrginae	01-SRNP-9758	MHAHG847-06	GU151798
9220	Udranomia kikkawaiDHJ03	Pyrginae	01-SRNP-24435	MHAHG845-06	GU151796
9221	Udranomia kikkawaiDHJ03	Pyrginae	02-SRNP-11244	MHAHG841-06	GU151797
9222	Udranomia kikkawaiDHJ03	Pyrginae	02-SRNP-11245	MHAHG839-06	GU151795
9223	Udranomia kikkawaiDHJ03	Pyrginae	00-SRNP-2831	MHAHG848-06	GU151799
9224	Udranomia kikkawaiDHJ03	Pyrginae	01-SRNP-17596	MHAHG831-06	GU151793
9225	Udranomia kikkawaiDHJ03	Pyrginae	07-SRNP-55917	MHAHK386-07	JF761254
9226	Udranomia kikkawaiDHJ03	Pyrginae	02-SRNP-32867	MHAHG837-06	GU151794
9227	Udranomia kikkawaiDHJ03	Pyrginae	07-SRNP-56117	MHAHK374-07	JF761251
9228	Udranomia kikkawaiDHJ03	Pyrginae	07-SRNP-20950	MHMXN424-07	JF763293
9229	Udranomia kikkawaiDHJ03	Pyrginae	07-SRNP-23337	MHMXR937-08	JF763292
9230	Udranomia kikkawaiDHJ03	Pyrginae	07-SRNP-23338	MHMXR938-08	JF763291
9231	Udranomia kikkawaiDHJ03	Pyrginae	08-SRNP-56040	MHMXW392-09	JF754340
9232	Udranomia kikkawaiDHJ03	Pyrginae	08-SRNP-20600	MHMXW400-09	JF754342
9233	Udranomia kikkawaiDHJ03	Pyrginae	08-SRNP-20931	MHMXW406-09	JF754343
9234	Udranomia kikkawaiDHJ03	Pyrginae	08-SRNP-21219	MHMXW417-09	JF754344
9235	Udranomia kikkawaiDHJ03	Pyrginae	08-SRNP-21967	MHMXX534-09	JF778570
9236	Udranomia kikkawaiDHJ03	Pyrginae	08-SRNP-21965	MHMXX535-09	JF778571
9237	Udranomia kikkawaiDHJ03	Pyrginae	08-SRNP-23929	MHMXY981-09	GU666546
9238	Udranomia kikkawaiDHJ03	Pyrginae	08-SRNP-16988	MHMXY986-09	GU666535
9239	Udranomia kikkawaiDHJ01	Pyrginae	03-SRNP-12017	MHAHG827-06	GU151756

Tree Order	Species	Subfamily	ACG Sampleid	BOLD Processid	Genbank Accession
9240	Udranomia kikkawaiDHJ01	Pyrginae	96-SRNP-807	MHAHG853-06	GU151769
9241	Udranomia kikkawaiDHJ01	Pyrginae	96-SRNP-1053	MHAHG851-06	GU151772
9242	Udranomia kikkawaiDHJ01	Pyrginae	96-SRNP-1007	MHAHG857-06	GU151774
9243	Udranomia kikkawaiDHJ01	Pyrginae	00-SRNP-6094	MHAHG852-06	GU151770
9244	Udranomia kikkawaiDHJ01	Pyrginae	01-SRNP-17593	MHAHG834-06	GU151750
9245	Udranomia kikkawaiDHJ01	Pyrginae	02-SRNP-4194	MHAHG836-06	GU151761
9246	Udranomia kikkawaiDHJ01	Pyrginae	02-SRNP-10083	MHAHG838-06	GU151758
9247	Udranomia kikkawaiDHJ01	Pyrginae	07-SRNP-55350	MHAHK390-07	JF761244
9248	Udranomia kikkawaiDHJ01	Pyrginae	08-SRNP-21920	MHMXX726-09	JF778564
9249	Udranomia kikkawaiDHJ01	Pyrginae	07-SRNP-55803	MHAHK375-07	JF761233
9250	Udranomia kikkawaiDHJ01	Pyrginae	07-SRNP-55804	MHAHK371-07	JF761232
9251	Udranomia kikkawaiDHJ01	Pyrginae	07-SRNP-55142	MHMXH872-07	JF761229
9252	Udranomia kikkawaiDHJ01	Pyrginae	06-SRNP-56437	MHAHI203-06	GU156376
9253	Udranomia kikkawaiDHJ01	Pyrginae	01-SRNP-24380	MHAHG844-06	GU151765
9254	Udranomia kikkawaiDHJ01	Pyrginae	03-SRNP-966	MHAHG842-06	GU151767
9255	Udranomia kikkawaiDHJ01	Pyrginae	01-SRNP-17592	MHAHG829-06	GU151753
9256	Udranomia kikkawaiDHJ01	Pyrginae	06-SRNP-13261	MHAHH796-06	GU155679
9257	Udranomia kikkawaiDHJ01	Pyrginae	97-SRNP-4074	MHAHG826-06	GU151757
9258	Udranomia kikkawaiDHJ01	Pyrginae	06-SRNP-13260	MHAHH812-06	GU155678
9259	Udranomia kikkawaiDHJ01	Pyrginae	96-SRNP-930	MHAHG860-06	GU151775
9260	Udranomia kikkawaiDHJ01	Pyrginae	07-SRNP-20867	MHAHK384-07	JF761239
9261	Udranomia kikkawaiDHJ01	Pyrginae	07-SRNP-20972	MHMXN423-07	JF763287
9262	Udranomia kikkawaiDHJ01	Pyrginae	07-SRNP-23327	MHMXR935-08	JF763283
9263	Udranomia kikkawaiDHJ01	Pyrginae	08-SRNP-20601	MHMXW411-09	JF754331
9264	Udranomia kikkawaiDHJ01	Pyrginae	08-SRNP-20596	MHMXW412-09	JF754332
9265	Udranomia kikkawaiDHJ01	Pyrginae	08-SRNP-21165	MHMXW416-09	JF754335
9266	Udranomia kikkawaiDHJ01	Pyrginae	08-SRNP-12275	MHMXX728-09	JF778566
9267	Udranomia kikkawaiDHJ01	Pyrginae	08-SRNP-12197	MHMXX729-09	JF778567
9268	Udranomia kikkawaiDHJ01	Pyrginae	07-SRNP-55686	MHAHK388-07	JF761242
9269	Udranomia kikkawaiDHJ01	Pyrginae	07-SRNP-56119	MHAHK387-07	JF761241
9270	Udranomia kikkawaiDHJ01	Pyrginae	07-SRNP-12122	MHAHK385-07	JF761240
9271	Udranomia kikkawaiDHJ01	Pyrginae	02-SRNP-14029	MHAHG832-06	GU151752
9272	Udranomia kikkawaiDHJ01	Pyrginae	08-SRNP-12195	MHMXX725-09	JF778563
9273	Udranomia kikkawaiDHJ01	Pyrginae	97-SRNP-4069	MHAHG855-06	GU151762
9274	Udranomia kikkawaiDHJ01	Pyrginae	08-SRNP-16999	MHMXY987-09	HM390688
9275	Udranomia kikkawaiDHJ01	Pyrginae	08-SRNP-75002	MHMXY985-09	GU666542
9276	Udranomia kikkawaiDHJ01	Pyrginae	08-SRNP-16997	MHMXY984-09	GU666541
9277	Udranomia kikkawaiDHJ01	Pyrginae	08-SRNP-12158	MHMXX727-09	JF778565
9278	Udranomia kikkawaiDHJ01	Pyrginae	08-SRNP-21923	MHMXX539-09	JF778562
9279	Udranomia kikkawaiDHJ01	Pyrginae	08-SRNP-21924	MHMXX533-09	JF778561
9280	Udranomia kikkawaiDHJ01	Pyrginae	08-SRNP-21968	MHMXX532-09	JF778560
9281	Udranomia kikkawaiDHJ01	Pyrginae	08-SRNP-21926	MHMXX531-09	JF778559
9282	Udranomia kikkawaiDHJ01	Pyrginae	07-SRNP-20946	MHMXX149-09	GU666369
9283	Udranomia kikkawaiDHJ01	Pyrginae	08-SRNP-55525	MHMXX1189-09	JF778568
9284	Udranomia kikkawaiDHJ01	Pyrginae	08-SRNP-20481	MHMXW415-09	JF754334
9285	Udranomia kikkawaiDHJ01	Pyrginae	08-SRNP-20636	MHMXW414-09	JF754333
9286	Udranomia kikkawaiDHJ01	Pyrginae	08-SRNP-20882	MHMXW407-09	JF754330
9287	Udranomia kikkawaiDHJ01	Pyrginae	08-SRNP-20635	MHMXW399-09	JF754329
9288	Udranomia kikkawaiDHJ01	Pyrginae	08-SRNP-45146	MHMXW394-09	JF754328
9289	Udranomia kikkawaiDHJ01	Pyrginae	07-SRNP-23341	MHMXR936-08	JF763282
9290	Udranomia kikkawaiDHJ01	Pyrginae	07-SRNP-20956	MHMXN427-07	JF763284
9291	Udranomia kikkawaiDHJ01	Pyrginae	07-SRNP-20953	MHMXN425-07	JF763286
9292	Udranomia kikkawaiDHJ01	Pyrginae	07-SRNP-56274	MHAHK391-07	JF761245
9293	Udranomia kikkawaiDHJ01	Pyrginae	07-SRNP-12359	MHAHK389-07	JF761243
9294	Udranomia kikkawaiDHJ01	Pyrginae	07-SRNP-12102	MHAHK383-07	JF761238
9295	Udranomia kikkawaiDHJ01	Pyrginae	07-SRNP-55349	MHAHK382-07	JF761237

Tree Order	Species	Subfamily	ACG Sampleid	BOLD Processid	Genbank Accession
9296	Udranomia kikkawaiDHJ01	Pyrginae	07-SRNP-55351	MHAHK381-07	JF761236
9297	Udranomia kikkawaiDHJ01	Pyrginae	07-SRNP-12180	MHAHK378-07	JF761234
9298	Udranomia kikkawaiDHJ01	Pyrginae	07-SRNP-12127	MHMXH900-07	JF761225
9299	Udranomia kikkawaiDHJ01	Pyrginae	07-SRNP-12034	MHMXH899-07	JF761226
9300	Udranomia kikkawaiDHJ01	Pyrginae	07-SRNP-12098	MHMXH897-07	JF761231
9301	Udranomia kikkawaiDHJ01	Pyrginae	07-SRNP-55263	MHMXH874-07	JF761228
9302	Udranomia kikkawaiDHJ01	Pyrginae	06-SRNP-13329	MHAHH813-06	GU155681
9303	Udranomia kikkawaiDHJ01	Pyrginae	06-SRNP-56079	MHAHH798-06	GU155680
9304	Udranomia kikkawaiDHJ01	Pyrginae	96-SRNP-1155	MHAHG856-06	GU151759
9305	Udranomia kikkawaiDHJ01	Pyrginae	96-SRNP-832	MHAHG854-06	GU151768
9306	Udranomia kikkawaiDHJ01	Pyrginae	99-SRNP-2706	MHAHG849-06	GU151771
9307	Udranomia kikkawaiDHJ01	Pyrginae	01-SRNP-24490	MHAHG846-06	GU151763
9308	Udranomia kikkawaiDHJ01	Pyrginae	01-SRNP-24434	MHAHG843-06	GU151766
9309	Udranomia kikkawaiDHJ01	Pyrginae	02-SRNP-5956	MHAHG840-06	GU151764
9310	Udranomia kikkawaiDHJ01	Pyrginae	01-SRNP-17953	MHAHG835-06	GU151760
9311	Udranomia kikkawaiDHJ01	Pyrginae	02-SRNP-4127	MHAHG833-06	GU151751
9312	Udranomia kikkawaiDHJ01	Pyrginae	01-SRNP-17591	MHAHG830-06	GU151755
9313	Udranomia kikkawaiDHJ01	Pyrginae	03-SRNP-12056	MHAHG828-06	GU151754
9314	Udranomia kikkawaiDHJ01	Pyrginae	05-SRNP-45233	MHAHF531-06	GU150944
9315	Udranomia kikkawaiDHJ01	Pyrginae	05-SRNP-45219	MHAHF530-06	GU150943
9316	Udranomia kikkawaiDHJ01	Pyrginae	04-SRNP-45504	MHAHD334-05	GU161917
9317	Udranomia kikkawaiDHJ01	Pyrginae	04-SRNP-16075	MHAHD333-05	GU161915
9318	Udranomia kikkawaiDHJ01	Pyrginae	04-SRNP-45505	MHAHD332-05	GU161919
9319	Udranomia kikkawaiDHJ01	Pyrginae	07-SRNP-20954	MHMXN426-07	JF763285
9320	Udranomia kikkawaiDHJ01	Pyrginae	05-SRNP-12029	MHAHD330-05	GU161916
9321	Udranomia kikkawaiDHJ01	Pyrginae	04-SRNP-45599	MHAHD328-05	GU161918
9322	Udranomia kikkawaiDHJ01	Pyrginae	07-SRNP-12099	MHMXH898-07	JF761227
9323	Udranomia kikkawaiDHJ01	Pyrginae	07-SRNP-12101	MHMXI576-07	JF761230
9324	Udranomia kikkawaiDHJ01	Pyrginae	07-SRNP-56275	MHAHK393-07	JF761246
9325	Udranomia kikkawaiDHJ01	Pyrginae	07-SRNP-55265	MHAHK380-07	JF761235
9326	Udranomia kikkawaiDHJ01	Pyrginae	07-SRNP-20952	MHMXN422-07	JF763288
9327	Udranomia kikkawaiDHJ01	Pyrginae	08-SRNP-75004	MHMXY983-09	GU666540
9328	Udranomia kikkawaiDHJ01	Pyrginae	09-SRNP-12113	MHMXY988-09	GU666536
9329	Udranomia kikkawaiDHJ01	Pyrginae	08-SRNP-16994	MHMXY989-09	GU666537
9330	Udranomia kikkawaiDHJ01	Pyrginae	09-SRNP-20996	MHMYH127-10	HM887278
9331	Udranomia kikkawaiDHJ02	Pyrginae	96-SRNP-1054	MHAHG859-06	GU151781
9332	Udranomia kikkawaiDHJ02	Pyrginae	05-SRNP-41045	MHAHF526-06	GU150948
9333	Udranomia kikkawaiDHJ02	Pyrginae	04-SRNP-30985	MHAHD329-05	GU161921
9334	Udranomia kikkawaiDHJ02	Pyrginae	05-SRNP-21026	MHAHF528-06	GU150946
9335	Udranomia kikkawaiDHJ02	Pyrginae	01-SRNP-22738	MHAHG864-06	GU151782
9336	Udranomia kikkawaiDHJ02	Pyrginae	03-SRNP-10461	MHAHG870-06	GU151788
9337	Udranomia kikkawaiDHJ02	Pyrginae	05-SRNP-40465	MHAHF532-06	GU150945
9338	Udranomia kikkawaiDHJ02	Pyrginae	06-SRNP-3255	MHAHG744-06	GU151778
9339	Udranomia kikkawaiDHJ02	Pyrginae	03-SRNP-10577	CSCR435-04	DQ293626
9340	Udranomia kikkawaiDHJ02	Pyrginae	04-SRNP-40828	MHAHD322-05	GU161922
9341	Udranomia kikkawaiDHJ02	Pyrginae	08-SRNP-31128	MHMXW398-09	JF754339
9342	Udranomia kikkawaiDHJ02	Pyrginae	08-SRNP-21090	MHMXW397-09	JF754338
9343	Udranomia kikkawaiDHJ02	Pyrginae	08-SRNP-70104	MHMXW395-09	JF754337
9344	Udranomia kikkawaiDHJ02	Pyrginae	07-SRNP-40724	MHAHK370-07	JF761247
9345	Udranomia kikkawaiDHJ02	Pyrginae	06-SRNP-23003	MHAHJ460-07	JF753211
9346	Udranomia kikkawaiDHJ02	Pyrginae	06-SRNP-41739	MHAHH803-06	GU155688
9347	Udranomia kikkawaiDHJ02	Pyrginae	06-SRNP-41738	MHAHH800-06	GU155684
9348	Udranomia kikkawaiDHJ02	Pyrginae	06-SRNP-41442	MHAHH799-06	GU155685
9349	Udranomia kikkawaiDHJ02	Pyrginae	03-SRNP-10604	MHAHG872-06	GU151791
9350	Udranomia kikkawaiDHJ02	Pyrginae	03-SRNP-10392	MHAHG871-06	GU151792
9351	Udranomia kikkawaiDHJ02	Pyrginae	03-SRNP-10390	MHAHG869-06	GU151789

Tree Order	Species	Subfamily	ACG Sampleid	BOLD Processid	Genbank Accession
9352	Udranomia kikkawaiDHJ02	Pyrginae	03-SRNP-11383	MHAHG868-06	GU151790
9353	Udranomia kikkawaiDHJ02	Pyrginae	02-SRNP-21390	MHAHG867-06	GU151786
9354	Udranomia kikkawaiDHJ02	Pyrginae	03-SRNP-10391	MHAHG865-06	GU151780
9355	Udranomia kikkawaiDHJ02	Pyrginae	02-SRNP-2617	MHAHG863-06	GU151783
9356	Udranomia kikkawaiDHJ02	Pyrginae	02-SRNP-6916	MHAHG862-06	GU151784
9357	Udranomia kikkawaiDHJ02	Pyrginae	06-SRNP-3420	MHAHG747-06	GU151779
9358	Udranomia kikkawaiDHJ02	Pyrginae	06-SRNP-3422	MHAHG746-06	GU151776
9359	Udranomia kikkawaiDHJ02	Pyrginae	05-SRNP-31041	MHAHF525-06	GU150947
9360	Udranomia kikkawaiDHJ02	Pyrginae	05-SRNP-21024	MHAHF524-06	GU150949
9361	Udranomia kikkawaiDHJ02	Pyrginae	04-SRNP-31720	MHAHD335-05	GU161920
9362	Udranomia kikkawaiDHJ02	Pyrginae	04-SRNP-41255	MHAHD331-05	GU161924
9363	Udranomia kikkawaiDHJ02	Pyrginae	04-SRNP-2413	MHAHD327-05	GU161923
9364	Udranomia kikkawaiDHJ02	Pyrginae	06-SRNP-41737	MHAHH810-06	GU155693
9365	Udranomia kikkawaiDHJ02	Pyrginae	06-SRNP-41741	MHAHH806-06	GU155690
9366	Udranomia kikkawaiDHJ02	Pyrginae	06-SRNP-3253	MHAHG745-06	GU151777
9367	Udranomia kikkawaiDHJ02	Pyrginae	06-SRNP-32415	MHAHI202-06	GU156378
9368	Udranomia kikkawaiDHJ02	Pyrginae	06-SRNP-34647	MHAHI580-06	GU156377
9369	Udranomia kikkawaiDHJ02	Pyrginae	07-SRNP-40376	MHAHK373-07	JF761248
9370	Udranomia kikkawaiDHJ02	Pyrginae	08-SRNP-40430	MHMXT123-08	JF763290
9371	Udranomia kikkawaiDHJ02	Pyrginae	08-SRNP-65801	MHMXY990-09	GU666538
9372	Udranomia kikkawaiDHJ02	Pyrginae	08-SRNP-41215	MHMXX537-09	JF778569
9373	Udranomia kikkawaiDHJ02	Pyrginae	08-SRNP-32449	MHMXY980-09	GU666545
9374	Udranomia kikkawaiDHJ02	Pyrginae	03-SRNP-10011	CSCR436-04	DQ293627
9375	Udranomia kikkawaiDHJ02	Pyrginae	06-SRNP-41523	MHAHH801-06	GU155686
9376	Udranomia kikkawaiDHJ02	Pyrginae	09-SRNP-69425	MHMYE1562-09	HM391122
9377	Udranomia kikkawaiDHJ02	Pyrginae	06-SRNP-3714	MHAHH805-06	GU155689
9378	Udranomia kikkawaiDHJ02	Pyrginae	06-SRNP-3715	MHAHH807-06	GU155691
9379	Udranomia kikkawaiDHJ02	Pyrginae	06-SRNP-32537	MHAHH809-06	GU155692
9380	Udranomia kikkawaiDHJ02	Pyrginae	03-SRNP-10389	CSCR434-04	DQ293625
9381	Udranomia kikkawaiDHJ02	Pyrginae	05-SRNP-31092	MHAHF523-06	GU150951
9382	Udranomia kikkawaiDHJ02	Pyrginae	06-SRNP-31634	MHAHH804-06	GU155682
9383	Udranomia kikkawaiDHJ02	Pyrginae	08-SRNP-40400	MHMXT124-08	JF763289
9384	Udranomia kikkawaiDHJ02	Pyrginae	08-SRNP-71686	MHMXW393-09	JF754336
9385	Udranomia kikkawaiDHJ02	Pyrginae	09-SRNP-69506	MHMYE1560-09	HM391120
9386	Udranomia kikkawaiDHJ02	Pyrginae	09-SRNP-69421	MHMYE1561-09	HM391121
9387	Udranomia kikkawaiDHJ02	Pyrginae	09-SRNP-69505	MHMYE1563-09	HM391123
9388	Udranomia kikkawaiDHJ02	Pyrginae	09-SRNP-41711	MHMYH128-10	HM887279
9389	Udranomia kikkawaiDHJ02	Pyrginae	02-SRNP-19637	MHAHG861-06	GU151785
9390	Udranomia kikkawaiDHJ02	Pyrginae	06-SRNP-41526	MHAHH802-06	GU155687
9391	Udranomia kikkawaiDHJ02	Pyrginae	05-SRNP-31080	MHAHF529-06	GU150950
9392	Udranomia kikkawaiDHJ02	Pyrginae	03-SRNP-5785	MHAHG866-06	GU151787
9393	Udranomia kikkawaiDHJ02	Pyrginae	06-SRNP-41524	MHAHH811-06	GU155694
9394	Udranomia kikkawaiDHJ02	Pyrginae	09-SRNP-42041	MHMYH129-10	HM887280
